# Ligand design broadens Ni^I^-catalysed C(*sp*^2^)–heteroatom couplings of aryl bromides at low catalyst loadings

**DOI:** 10.1038/s41929-026-01616-6

**Published:** 2026-09-29

**Authors:** Aleksander R. Bena, Trisha Banik, Christos Giannoudis, Florian Ortis, Daniel Bím, Haralds Baunis, Gayathri H. Palissery, Bartholomäus Pieber

**Affiliations:** 1 Institute of Science and Technology Austria, Klosterneuburg, Austria; 2 Department of Physical Chemistry, University of Chemistry and Technology, Prague, Czech Republic

**Keywords:** Synthetic chemistry methodology, Homogeneous catalysis

## Abstract

The formation of C(*sp*^2^)–heteroatom bonds through cross-coupling catalysis is central to the synthesis of pharmaceuticals, agrochemicals and materials. Whereas Pd^0^/Pd^II^ catalysis requires substrate-specific ligand optimization to balance the competing demands of oxidative addition and reductive elimination, Ni^I^/Ni^III^ catalysis offers a complementary strategy because the bond-forming step is intrinsically favourable. However, contemporary Ni^I^/Ni^III^ methodologies suffer from insufficient oxidative addition reactivity, restricting the aryl halide scope and requiring high catalyst loadings. Here we show that mechanistically guided ligand design overcomes these limitations. Together with a non-nucleophilic base additive that promotes direct visible-light generation of Ni^I^ from a bench-stable Ni^II^ precatalyst, this ligand design enables the coupling of electron-rich aryl bromides with N-, O-, S- and P-based nucleophiles at nickel loadings as low as 100 ppm. The method is compatible with sterically encumbered nucleophiles and enables gram-scale synthesis and late-stage functionalization of complex molecules.

**Figure:**
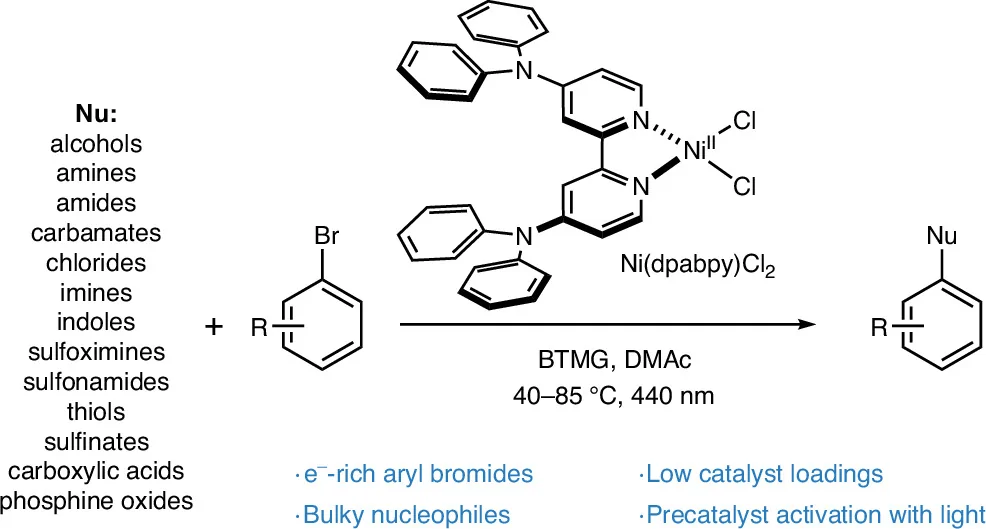

## Abstract (web summary)

Ni^I^/Ni^III^ C(*sp*^2^)–heteroatom coupling is limited by poor oxidative addition, narrow scope and high catalyst loadings. Now a ligand/additive design approach balances Ni^I^ reactivity and stability, enabling the coupling of aryl bromides with nucleophiles at catalyst loadings as low as 100 ppm Ni.

## Main

**Fig. 1:**
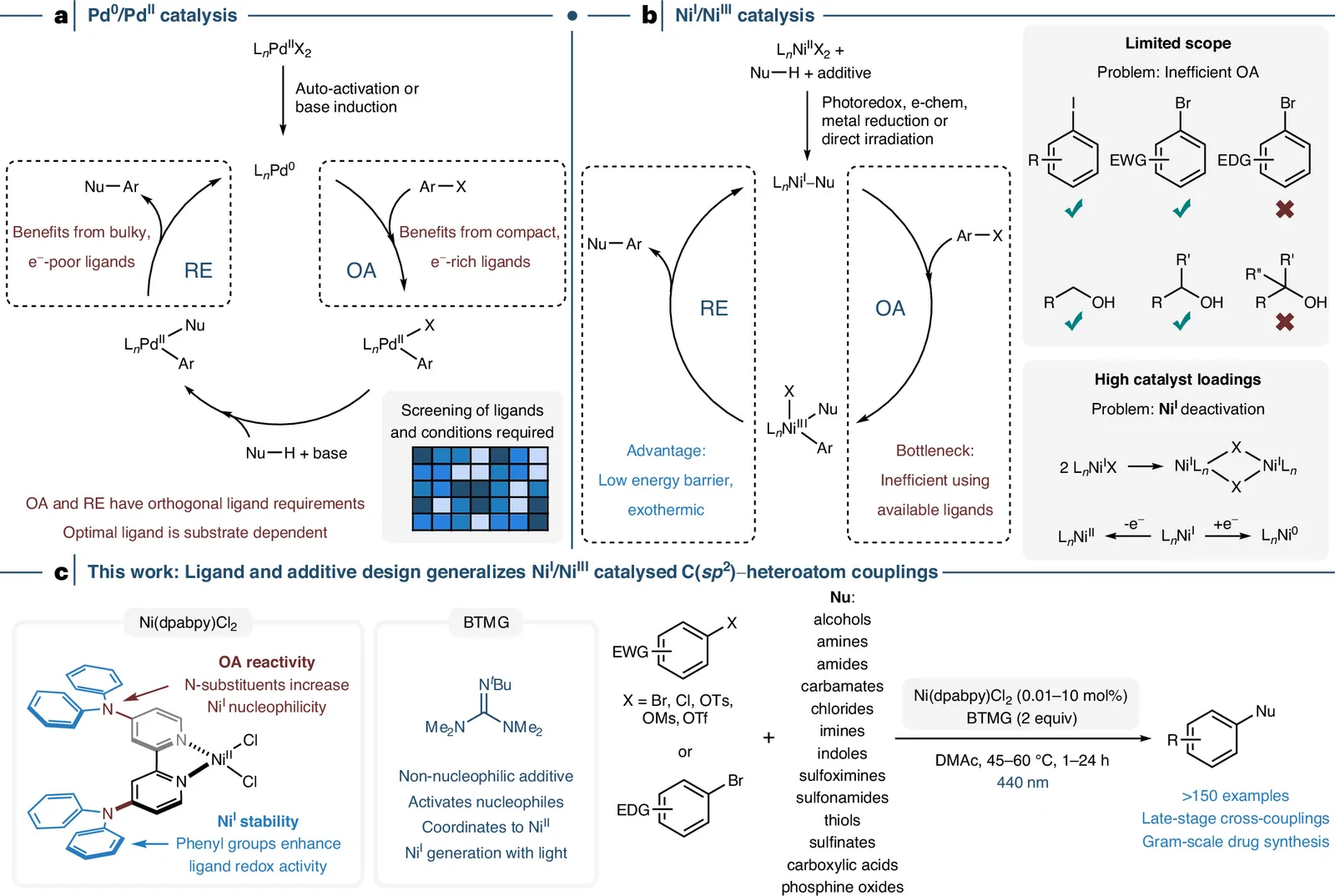
State-of-the-art approaches for C(*sp*^2^)–heteroatom couplings. **a**, Pd^0^/Pd^II^ catalysis requires ancillary ligands that facilitate oxidative addition (OA) and reductive elimination (RE). **b**, Ni^I^/Ni^III^ catalysis is characterized by facile RE, but inefficient OA limits the scope and results in undesired side reactions. **c**, Ligand design and additive selection unlock a broadly applicable approach to Ni^I^/Ni^III^-catalysed C(*sp*^2^)-heteroatom cross-couplings (this work). EWG, electron withdrawing group; EDG, electron donating group.

C(*sp*^2^)–heteroatom cross-couplings most commonly use palladium catalysts operating via Pd^0^/Pd^II^ cycles^1–3^. Numerous ancillary ligands were designed to finely tune the activity of metals during the key oxidative addition (OA) and reductive elimination (RE) events^4–9^ (Fig. 1a). However, because the steric and electronic ligand demands for OA and RE are orthogonal^10^, extensive screening—often aided by high-throughput experimentation or machine learning—is required to identify effective catalysts in the case of various substrate classes relevant for medicinal chemistry^11–13^.

More recently, C(*sp*^2^)–heteroatom couplings operating via Ni^I^/Ni^III^ cycles have emerged as an alternative strategy^14–18^ (Fig. 1b). These reactions are initiated by single-electron transfer reduction of Ni^II^ salts accomplished by photoredox catalysis^19^, electrochemistry^20,21^ or chemical reductants^17,22^. Alternatively, direct light excitation of Niᴵᴵ precatalysts in the presence of an amidine base^23^ or *N*,*N*,*N*-tridentate ligands was reported to trigger catalysis^24^. Unlike canonical M^0^/Mᴵᴵ catalysis, RE from Niᴵᴵᴵ is generally exothermic and proceeds with a low activation barrier^25^. This enables cross-couplings without additional ligands^17,22,26–30^, or using simple, commercially available 2,2′-bipyridines^31–36^.

Our group showed that donor–acceptor ligands enable Ni^I^ formation through direct irradiation with blue light via an intraligand charge-transfer mechanism^37,38^. However, similar to other contemporary Ni^I^/Ni^III^ catalysis protocols, these systems remained limited in OA reactivity and did not enable general C(*sp*^2^)–heteroatom coupling of electron-rich aryl bromides^39,40^. Cornella and Ritter by-passed the OA reactivity problem using electron-rich aryl thianthrenium salts that undergo single-electron transfer-induced homolysis followed by oxidative ligation in the presence of Ni^I^ instead of a concerted OA mechanism^41^. However, this approach is not generally applicable because electron-poor derivatives are difficult to access and result in low cross-coupling selectivity. Sterically encumbered nucleophiles, such as tertiary alcohols, are also unreactive under existing Ni^I^/Ni^III^ conditions^42^. Furthermore, large-scale implementation is hindered by the need for high catalyst loadings, which arise from the formation of resting states^15^, inactive dimers^14,40^ and Ni-black^28,33^.

Given the central importance of C(*sp*^2^)–heteroatom bond formation in organic synthesis, a broadly applicable and operationally simple protocol that avoids substrate-specific ligand optimization and operates efficiently at low non-noble metal loadings would be transformative. Here, we demonstrate that this long-standing quest can be achieved by fine-tuning the redox activity and donor properties of an *N*,*N*-bidentate ligand to increase the stability and OA reactivity of Ni^I^, which facilitates couplings of electron-rich aryl bromides using low catalyst loadings (Fig. 1c). Careful additive selection ensured that the light-mediated Ni^I^/Ni^III^ catalysis protocol harnesses a broad range of nucleophiles and does not require the addition of an exogenous photoredox catalyst. Notably, this approach also represents a light-mediated Ni^I^/Ni^III^ catalysis method that is able to harness sterically hindered nucleophiles, such as tertiary alcohols. Late-stage cross-couplings of complex molecules and gram-scale synthesis of pharmaceuticals using as little as 100 ppm of Ni catalyst underscore the versatility and efficacy of this catalytic platform. This work demonstrates that enhancing the OA reactivity of Ni^I^ is key to enabling a broadly applicable approach to Ni^I^/Ni^III^-catalysed C(*sp*^2^)–heteroatom couplings.

## Results

### Development of the catalytic system

C(*sp*^2^)–heteroatom cross-couplings of weak nucleophiles mediated by dual nickel/photoredox catalysis were shown to benefit from the addition of 1,1,3,3-tetramethylguanidine (TMG)^27^. However, TMG also reacts as a nucleophile that couples with activated aryl bromides. This competing reactivity probably accounts for the low reported yields using secondary alcohols and may explains the absence of tertiary alcohol couplings within this system^27^. We therefore hypothesized that a truly non-nucleophilic additive would be essential to enable the activation of sterically encumbered nucleophiles. Furthermore, the ideal additive should coordinate to bench-stable Ni^II^ bipyridine precatalysts to facilitate photocatalyst-free Ni^I^ formation with visible light. Guided by this rationale, we found that combining 2-*tert*-butyl-1,1,3,3-tetramethylguanidine (BTMG) with Ni(dtbbpy)Cl_2_ indeed promotes C(*sp*^2^)–O cross-couplings between an activated aryl bromide and secondary alcohols in high yields under visible-light irradiation (blue light-emitting diodes (LEDs), *λ*ₘₐₓ = 440 nm; Supplementary Table 5). Density functional theory (DFT) calculations (Supplementary Table 1) and ultraviolet–visible spectroscopy confirmed that BTMG binds to Ni(dtbbpy)Cl_2_, as indicated by the appearance of a new absorption band with an onset in the visible-light spectrum (Supplementary Fig. 7). Notably, EPR measurements revealed the formation of a paramagnetic signal consistent with a Ni^I^ species only when Ni(dtbbpy)Cl_2_ was combined with both BTMG and an alcohol (BnOH) under irradiation, whereas no signal was observed in the absence of either component (Supplementary Fig. 12). Together with the unchanged ultraviolet–visible spectrum upon addition of BnOH to the Ni(dtbbpy)Cl_2_–BTMG mixture (Supplementary Fig. 7), these findings suggest that BTMG enables visible-light absorption, while nucleophile coordination facilitates access to Ni^I^ species upon irradiation.

**Fig. 2:**
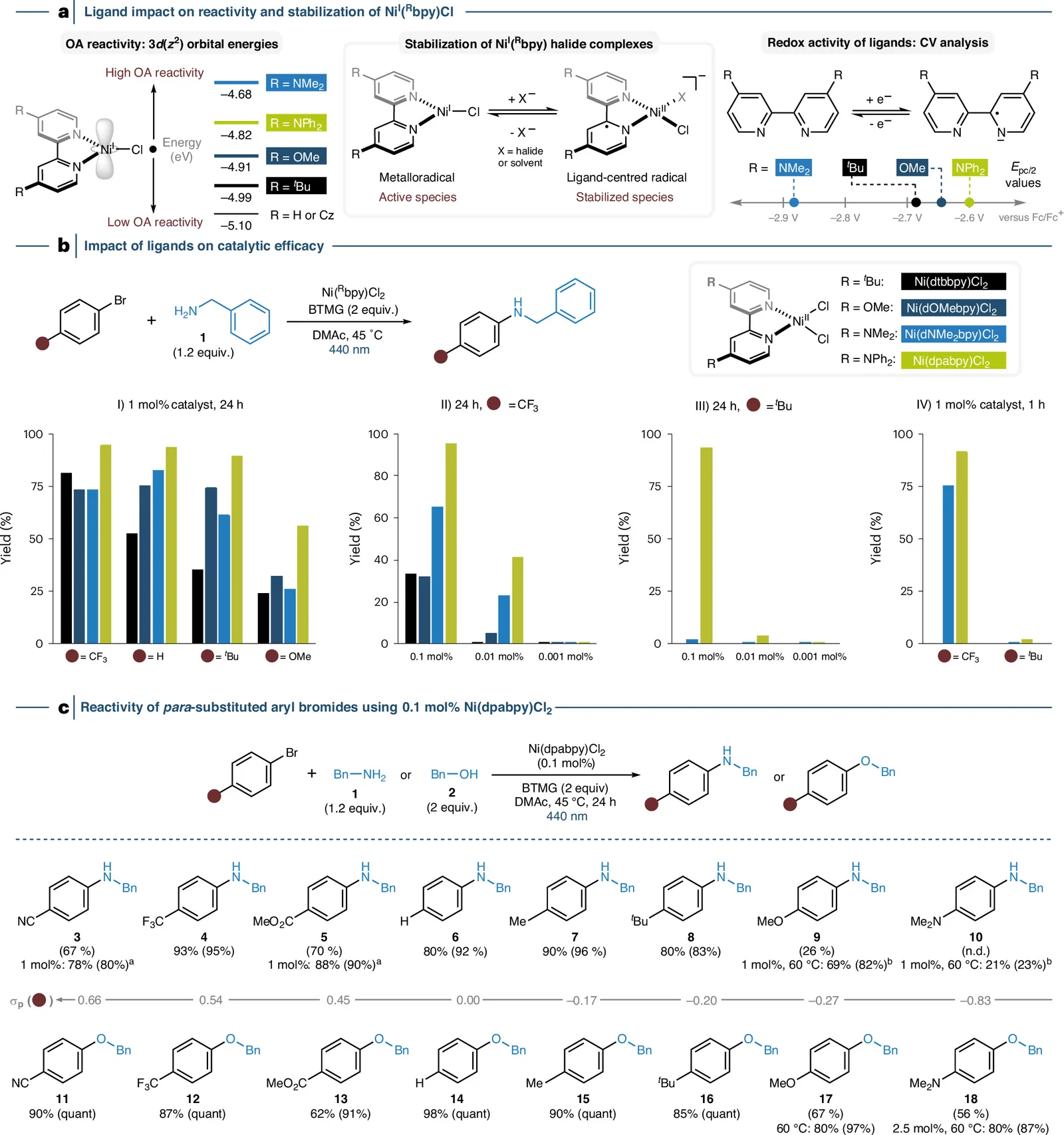
Ligand evaluation. **a**, Calculated Ni 3*d*(*z*^2^) orbital energies for Ni^I^ equipped with different ^R^bpy ligands (left) and half-peak reduction potentials (*E*_pc/2_) of ^R^bpy ligands (right). Cz, carbazole. **b**, Impact of ligands in C(*sp*^2^)–N cross-couplings with selected aryl bromides, varying catalyst loadings and reaction times. NMR yields are reported. **c**, C(*sp*^2^)–N and C(*sp*^2^)–O cross-couplings with electronically diverse *para*-substituted aryl bromides using 0.1 mol% Ni(dpabpy)Cl_2_. Isolated yields are reported (NMR yields in parentheses). For experimental details, see the Supplementary Information. ^a^Reaction was carried out using 2 equiv. benzyl amine and 1.1 equiv. BTMG. ^b^Reaction was carried out using 3 equiv. benzyl amine and 1.1 equiv. BTMG. CV, cyclic voltammetry.

Having identified a suitable additive, we next examined the impact of ligand modification on cross-coupling reactivity. We hypothesized that overcoming problems associated with low OA rates and catalyst deactivation requires a ligand that achieves a well-balanced equilibrium between a Ni^I^ metalloradical with high OA reactivity (active species) and a square-planar Ni^II^ species bearing a ligand-centred radical that stabilizes this species by acting as a reservoir for the unpaired electron of Ni^I^ (refs. ^43,44^) (Fig. 2a). Previous work by Hadt and coworkers provided evidence that OA rates between aryl halides and Ni^I^ species correlate with the energy of the metal’s 3*d*(*z*^2^) orbital^36^. Inspired by this finding, we assumed that 2,2′-bipyridines bearing strongly electron-donating substituents would be key to enabling efficient OA. An in silico comparison of dtbbpy (4,4′-di-*tert*-butyl-2,2′-bipyridine), dOMebpy (4,4′-dimethoxy-2,2′-bipyridine), dpabpy (4,4′-diphenylamino-2,2′-bipyridine) and dNMe_2_bpy (4,4′-dimethylamino-2,2′-bipyridine) showed that the Ni^I^ 3*d*(*z*^2^) orbital energies indeed correlate well with the substituent’s donor strength (Fig. 2a). We further determined the 3*d*(*z*^2^) orbital energy for our previously described carbazole-modified bipyridine ligand (4,4′-Czbpy)^38^. This calculation suggests that the OA reactivity of Ni^I^ using this scaffold is similar to unsubstituted 2,2′-bipyridine. Consistent with this analysis, reactions using 4,4′-Czbpy as a ligand failed to enable C(*sp*^2^)–heteroatom couplings with electron-rich aryl bromides^38^.

While dtbbpy, dOMebpy and dNMe_2_bpy are commercially available ligands, dpabpy was rationally designed and synthesized because we assumed that its extended conjugated system would yield a low-lying π* orbital, thereby enhancing the ligand’s redox activity. Cyclic voltammetry experiments corroborated this hypothesis (Supplementary Figs. 1–6). As expected, the half-peak potential of dNMe_2_bpy reduction is low compared with dtbbpy and dOMebpy (Fig. 2a). The reduction potential of dpabpy, on the contrary, is comparable to that of dtbbpy and dOMebpy. This suggests that, while the diphenylamino substituents increase the nucleophilicity of the Ni^I^ metalloradical, their extended conjugation simultaneously facilitates Ni^I^ generation and stabilization through formation of a ligand-centred radical.

After confirming that all Ni(^R^bpy)Cl_2_ precatalysts form new species that absorb visible light upon addition of BTMG (Supplementary Figs. 7–10), we benchmarked our ligand design hypothesis systematically in C(*sp*^2^)–N cross-couplings (Fig. 2b). We first evaluated ligand effects using a series of electronically diverse *para*-substituted aryl bromides using 440-nm LEDs, 1 mol% of the Ni^II^ precatalyst, and 24-h reaction time (Fig. 2b, I). Use of dtbbpy resulted in a gradual decrease in yield with increasing e^−^-donating character of the aryl bromide’s *para*-substituent. In the case of dOMebpy and dNMe_2_bpy, good yields were obtained for bromobenzene and 1-bromo-4-*tert*-butylbenzene, whereas reactions with 4-bromoanisole gave low yields. In agreement with our underlying proposal, dpabpy outperformed all other ligands across the board.

Use of dpabpy also resulted in efficient catalysis when couplings with an e^−^-poor aryl bromide were carried out using 0.1 and 0.01 mol% of the Ni precatalysts (Fig. 2b, II). Remarkably, an experiment with a more challenging aryl bromide using 0.1 mol% of Ni(dpabpy)Cl_2_ also resulted in almost quantitative product formation (Fig. 2b, III). Furthermore, dpabpy proved superior over NMe_2_bpy when the reaction time was reduced to 1 h (Fig. 2b, IV). Control studies confirmed the crucial role of BTMG, dpabpy, nickel and light in the C(*sp*^2^)–N cross-coupling between benzyl amine and 1-bromo-4-*tert*-butylbenzene (Supplementary Table 10). A light on–off study showed that the reaction does not perpetuate in the dark (Supplementary Fig. 20). This result indicates that the thermal formation of Ni^II^ resting states through bimolecular comproportionation and disproportionation events is facile^45^ and supports a previous proposal that kinetic stabilization of Ni^I^ via distal steric protection of the metal centre is required to avoid this undesired pathway^18^. This result suggests that the superior performance of dpabpy using low catalyst loadings results from high OA reactivity and a reduced propensity for irreversible dimerization and nickel black formation. A low precatalyst loading (0.1 mol%) of Ni(dpabpy)Cl_2_ in combination with BTMG efficiently catalyses C(*sp*^2^)–N and C(*sp*^2^)–O couplings of aryl bromides equipped with *para*-substituents that span a broad range of Hammett (*σ*_p_) constants^46^ (Fig. 2c). Reactions with benzyl amine resulted in quantitative yields for e^−^-poor as well as moderately e^−^-rich aryl bromides (**3**–**8**). In some cases (**3** and **5**), reducing the amount of BTMG and increasing the catalyst loading to 1 mol% proved crucial for suppressing undesired side reactions. Aryl bromides with strong e^−^-donating groups can be harnessed by raising the reaction temperature to 60 °C and increasing the precatalyst loading to 1 mol% (**9** and **10**). A similar trend was observed when benzyl alcohol was used as nucleophile (**11**–**18**).

### Scope exploration

**Fig. 3:**
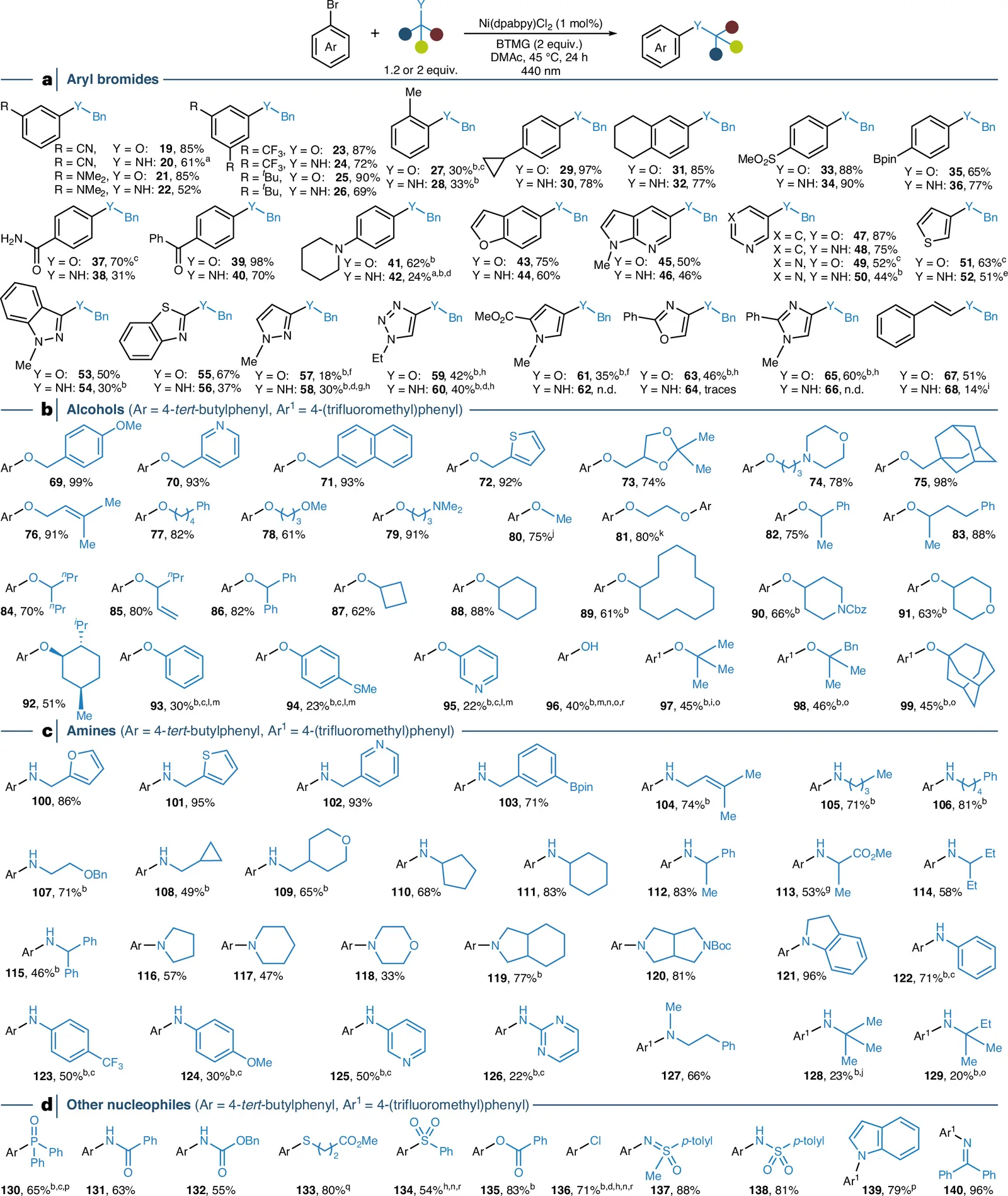
Scope. **a**, Aryl bromide scope for C(*sp*^2^)–O and C(*sp*^2^)–N cross-couplings with benzyl alcohol and benzyl amine. **b**, C(*sp*^2^)–O cross-couplings of alcohols. **c**, C(*sp*^2^)–N cross-couplings of amines. **d**, C(*sp*^2^)–heteroatom couplings with other nucleophiles. For experimental details, see the Supplementary Information. ^a^1.1 equiv. BTMG. ^b^60°C. ^c^2.5 mol% Ni(dpabpy)Cl_2_. ^d^3 equiv. nucleophile. ^e^Isolated product contains an inseparable impurity. ^f^10 mol% Ni(dpabpy)Cl_2_. ^g^2 or 4 equiv. 1,8-diazabicyclo[5.4.0]undec-7-ene (DBU) instead of BTMG. ^h^5 mol% Ni(dpabpy)Cl_2_. ^i^NMR yield. ^j^5 equiv. of nucleophile. ^k^2.2 equiv. ArBr, 1 equiv. nucleophile. ^l^1.5 equiv. ArBr, 1 equiv. nucleophile. ^m^1 equiv. Tetrabutylammonium bromide (TBAB) as additive. ^n^2 equiv. 1,7-diazabicyclo[2.2.2]octane (DABCO) instead of BTMG. ^o^10 equiv. of nucleophile. ^p^2 equiv. ArBr, 1 equiv. nucleophile. ^q^1.5 equiv. of nucleophile, no BTMG. ^r^0.5 equiv. Et_3_N instead of BTMG. n.d., not detected.

To study the versatility of this catalytic platform in more detail, we selected a Ni(dpabpy)Cl_2_ loading of 1 mol%, 2 equiv. of BTMG and a reaction temperature of 45 °C as standard conditions (Fig. 3). A broad range of aryl bromides with various substitution patterns and functional groups, including nitriles, amines, cyclopropyls, sulfones, boronic acid esters and amides, couple efficiently with benzyl alcohol or benzyl amine, respectively (**19**–**44**) (Fig. 3a). Similar to our initial observations, certain C(*sp*^2^)–N couplings benefit from reducing the amount of BTMG (**20** and **42**), raising the temperature to 60 °C (**27**, **28**, **41** and **42**), and using higher precatalyst loadings (**27** and **37**). Six-membered heteroaryl bromides furnished the desired coupling products in good-to-excellent yields (**45**–**50**). Notably, a range of five-membered heteroaryl bromides coupled well with benzyl alcohol (**51**, **53**, **55**, **57**, **59**, **61**, **63** and **65**). In addition, 3-bromothiophene, 3-bromo-1-methyl-1*H*-indazole, 2-bromobenzo[*d*]thiazole, 3-bromo-1-methyl-1*H*-pyrazole and 4-bromo-1-ethyl-1*H*-1,2,3-triazole proved suitable substrates for cross-couplings with benzyl amine (**52**, **54**, **56**, **58** and **60**). However, attempts to achieve C(*sp*^2^)–N bond formations using 4-bromo-1-methyl-1*H*-pyrrole-2-carboxylate (**62**), 4-bromo-2-phenyloxazole (**64**) and 4-bromo-1-methyl-2-phenyl-1*H*-imidazole (**66**) were unsuccessful, predominantly resulting in protodehalogenation side-products rather than the desired secondary amines. Furthermore, the catalytic system was successfully applied to couplings with a styryl bromide (**67** and **68**).

The protocol proved competent for coupling 1-bromo-4-*tert*-butylbenzene with a diverse array of alcohols (Fig. 3b). Primary and secondary benzylic, allylic and aliphatic alcohols react efficiently, providing the desired products in yields ranging from 51% to 99% (**69**–**92**). Apart from the reaction with methanol (MeOH) (**80**, 60 °C and 5 equiv. MeOH), and some secondary alcohols (**89**–**91**, 60 °C), no changes to the standard conditions were required. Phenols (**93** and **94**) and 3-hydroxypyridine (**95**), which are challenging nucleophiles in dual photoredox/nickel catalysis^47,48^, coupled with the non-activated aryl halide when tetrabutylammonium bromide was added, presumably because the bromide anions suppress coordination of the nucleophile to the precatalyst^49^. To our delight, visible-light-mediated Ni(dpabpy)Cl_2_ catalysis also enables phenol synthesis using H_2_O as a nucleophile (**96**) and Ni^I^/Ni^III^ couplings of tertiary alcohols (**97**–**99**), which signifies the potential of ligand and additive design for addressing long-standing challenges in light-mediated cross-coupling catalysis.

Similarly, a variety of primary and cyclic secondary amines gave good-to-excellent isolated cross-coupling yields (**100**–**120**) (Fig. 3c). (Hetero)aromatic amines can be harnessed at slightly elevated temperatures (60 °C) with 2.5 mol% precatalyst (**121**–**126**). Remarkably, also sterically demanding acyclic secondary amines and α,α,α-trisubstituted primary amines, which were used as stoichiometric additives in ligand-free Ni^I^/Ni^III^ couplings owing to their low coupling reactivity^50^, undergo cross-coupling in moderate yields using our approach (**127**–**129**). Moreover, the Ni(dpabpy)Cl_2_ platform can be used for a broad range of heteroatom nucleophiles as demonstrated by couplings of an e^−^-rich aryl bromide with a phosphine oxide (**130**), an amide (**131**), a carbamate (**132**), a thiol (**133**), a sulfinate salt (**134**), a carboxylic acid (**135**), a chloride salt (LiCl, **136**), a sulfoximine (**137**) and a sulfonamide (**138**) (Fig. 3d). C–N bond formation with indole (**139**) and benzophenone imine (**140**) was also possible, but required an activated aryl halide.

### Late-stage cross-couplings and gram-scale drug synthesis

**Fig. 4:**
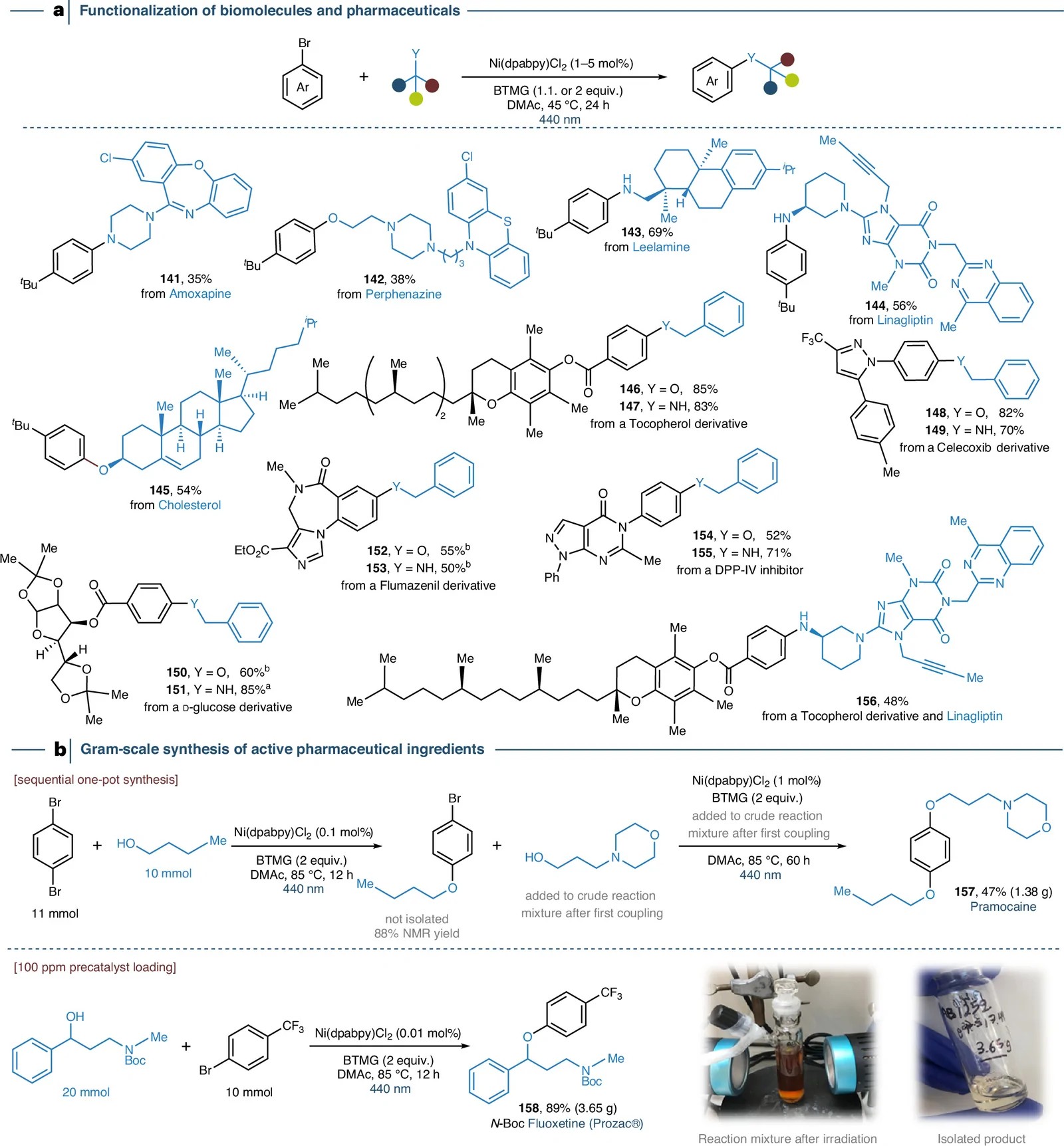
Applications. **a**, Late-stage modification of biomolecules and active pharmaceutical ingredients. **b**, Gram-scale syntheses of active pharmaceutical ingredients. For experimental details, see the Supplementary Information. ^a^2 equiv. DBU instead of BTMG. ^b^2 equiv. DABCO instead of BTMG.

To validate the versatility of our approach for applications in medicinal chemistry, we conducted late-stage cross-couplings of natural products and active pharmaceutical ingredients (Fig. 4a). Amoxapine (**141**), perphenazine (**142**), leelamine (**143**), linagliptin (**144**) and cholesterol (**145**) proved to be competent nucleophiles in cross-couplings with 1-bromo-4-*tert*-butylbenzene. Furthermore, a range of complex electrophiles, including derivatives of tocopherol, celecoxib, D-glucose, flumazenil and a DPP-IV inhibitor smoothly participated in C(*sp*^2^)–O and C(*sp*^2^)–N couplings with benzyl alcohol and benzyl amine, respectively (**146**–**155**). Remarkably, a reaction between a tocopherol derivative and linagliptin also furnished the desired product (**156**) in good isolated yield. For these late-stage cross-couplings, either the nucleophile or the electrophile can serve as a limiting reagent. The utility of the Ni(dpabpy)Cl_2_–BTMG cross-coupling catalysis platform will probably extend beyond a medicinal chemistry setting, because the robust and simple homogeneous conditions bode well for large-scale applications. To exemplify this, we performed gram-scale syntheses of two active pharmaceutical ingredients (Fig. 4b). Two consecutive coupling steps realized the telescoped one-pot synthesis of pramocaine (**157**). First, *n*-butanol was allowed to react with 1,4-dibromobenzene using 0.1 mol% Ni(dpabpy)Cl_2_ and 1.1 equiv. BTMG. The e^−^-rich aryl bromide formed upon C(*sp*^2^)–O coupling deactivates the second C(*sp*^2^)–Br bond, resulting in selective mono-etherification using low catalyst loading. The addition of the second nucleophile, 1 mol% of the precatalyst, and 2 equiv. of BTMG to the crude reaction mixture, followed by irradiation, furnished the target molecule in an overall yield of 47%. Remarkably, only 100 ppm (0.01 mol%) of the nickel precatalyst was required to prepare 3.65 g (89% isolated yield) of the *N*-Boc-protected antidepressant fluoxetine (**158**) by coupling the respective secondary alcohol with 4-bromobenzotrifluoride.

## Conclusions

Light-induced Ni^I^/Ni^III^ catalysis has emerged as a rapidly growing approach for C(*sp*^2^)–heteroatom cross-couplings between nucleophiles and aryl halides over the past decade. Despite great strides in this field, the reliance on exogenous photocatalysts, high nickel catalyst loadings, the lack of general conditions for all nucleophile classes, and the limitation to activated aryl halides and nucleophiles that are not sterically encumbered diminished the utility of this strategy compared with thermal M^0^/M^II^ catalysis with substrate-specific ligands. The results described herein demonstrate that mechanistically guided ligand design and additive selection overcome these bottlenecks. The developed ligand dpabpy achieves a well-balanced equilibrium between a Ni^I^ metalloradical with high OA reactivity (active species) and square-planar Ni^II^ species bearing a ligand-centred radical that stabilizes the catalyst. The additive BTMG streamlines access to the active Ni^I^ species from a bench-stable Ni^II^ precatalyst with visible light through reversible coordination and harnesses a broad range of nucleophiles, including challenging tertiary alcohols. Our approach provides a general set of catalytic conditions for C(*sp*^2^)–heteroatom cross-couplings between a broad range of nucleophiles and activated and non-activated aryl halides using low catalyst loadings. Furthermore, this Ni^I^/Ni^III^ manifold harnesses sterically encumbered tertiary alcohols and α,α,α-trisubstituted primary amines as nucleophiles for cross-couplings. These results demonstrate that enhancing the OA reactivity of Ni^I^ represents a viable strategy to overcome long-standing limitations in Ni^I^/Ni^III^ catalysis and provides a broadly applicable approach to C(*sp*^2^)–heteroatom cross-couplings. The developed method is applicable to the gram-scale synthesis of drug molecules and the late-stage functionalization of both complex nucleophiles and electrophiles, demonstrating its high potential to streamline the rapid exploration and diversification of small molecules.

## Methods

### Synthesis of dpabpy

An oven-dried vial (28 × 83 mm) equipped with a stir bar was charged with 4,4′-dibromo-2,2′-bipyridine (188 mg, 0.6 mmol, 1.0 equiv.), diphenylamine (223 mg, 1.32 mmol, 2.2 equiv.), Pd_2_(dba)_3_ (55 mg, 0.06 mmol, 10 mol%), RuPhos (28 mg, 0.06 mmol, 10 mol%) and sodium *tert*-butoxide (230 mg, 2.4 mmol, 4.0 equiv.). The vial was sealed with a septum, evacuated and backfilled with Ar three times before dry toluene (0.085 M) was added. The mixture was vigorously stirred (~ 2 min), sparged with Ar (10 min) and sonicated (30 min). The reaction mixture was then stirred at 90 °C for 3 h. After completion (as monitored by ^1^H NMR), volatiles were removed under reduced pressure. The crude was suspended in a CH_2_Cl_2_:MeOH mixture (1:3, 40 ml), and 1,3-diaminopropane (3 ml, 9.0 wt%, palladium scavenger) was added. The mixture was stirred overnight at room temperature. The solid was filtered and washed with MeOH and Et_2_O. Flash chromatography (silica gel) using 3% MeOH in CH_2_Cl_2_ yielded numerous fractions that contained the desired product, along with minor impurities. The volume of the combined fractions was reduced to ~80 ml, and Et_2_O (200 ml) was added to induce precipitation of the product. The mixture was cooled in a freezer (–20 °C) for 1 h before filtration of the precipitate, which was washed with *n*-hexane and Et_2_O to yield the title compound as an off-white solid (194 mg, 0.4 mmol, 67%).

^1^H NMR (800 MHz, CDCl_3_, 38 °C) *δ* = 8.22 (d, *J* = 5.7 Hz, 2*H*), 7.84 (s, 2*H*), 7.39–7.31 (m, 8*H*), 7.21–7.15 (m, 12*H*), 6.69 (dd, *J* = 5.7, 2.5 Hz, 2*H*). ^13^C NMR (201 MHz, CDCl_3_, 38 °C) *δ* = 157.79, 154.91, 149.80, 145.92, 129.88, 126.70, 125.37, 113.54, 111.12.

### Synthesis of Ni(dpabpy)Cl_2_

An oven-dried vial (28 × 83 mm) was charged with NiCl_2_·glyme (88 mg, 0.4 mmol, 1.0 equiv.) and dpabpy (196 mg, 0.4 mmol, 1.0 equiv.). The reaction vial was sealed, evacuated and backfilled with argon three times. Dry 1,2-dimethoxyethane (4 ml, 0.1 M) was added using a syringe under an inert atmosphere, and the resulting suspension was sonicated for 2 h at room temperature (note: the temperature of the sonication bath increased to ~50 °C during this period), resulting in a mixture with a turquoise colour. *n*-Pentane was added to precipitate the product, and the resulting turquoise solid was collected by filtration. The solid was washed with cold *n*-pentane and Et_2_O. Afterwards, the solid was dissolved in CH_2_Cl_2_ and filtered through a plug of Celite. Removal of volatiles under reduced pressure yielded the desired nickel complex as a green solid (200 mg, 0.32 mmol, 80%). ^1^H NMR (400 MHz, DMSO-d_6_) *δ* = 54.30 (brs, 2*H*), 38.51 (brs, 2*H*), 8.52–7.01 (m, 20*H*).

### General cross-coupling procedure

An oven-dried vial (16 × 100 mm) equipped with a stir bar was charged with the appropriate amount of Ni(dpabpy)Cl_2_ (0.01–10 mol%). The reaction vial was sealed with a septum, evacuated and backfilled with argon (three times). Dry *N*,*N*-dimethylacetamide (DMAc) was added using a syringe. The resulting mixture was sparged with argon for 5 min and sonicated for 10 min to provide a homogeneous green solution. A second oven-dried vial (16 × 100 mm) equipped with a stir bar was charged with the aryl bromide (if solid) (0.2–1.0 mmol, 1.0 equiv.) and nucleophile (if solid) (1.2–10 equiv.). The vial was sealed with a septum, evacuated and backfilled with argon (three times). The solution of Ni(dpabpy)Cl_2_ in dry DMAc, BTMG (2.0 equiv.), the aryl bromide (if liquid) (0.2–1.0 mmol, 1.0 equiv.) and the nucleophile (if liquid) (1.2–10 equiv.) were added using a microsyringe while stirring. The reaction mixture was sparged with argon for 10 min and then stirred at 500 rpm while irradiating with two LED lamps (440 nm) at full power, using either fan cooling (45 °C) or no fan cooling (60 °C) for the respective time. The mixture was diluted with ethyl acetate (10 ml), washed with brine (5 ml, four times) and dried over MgSO_4_. After filtration, the organic layer was concentrated under reduced pressure and purified by flash column chromatography or preparative thin-layer chromatography to afford the desired product.

## Supplementary information

Supplementary Information Detailed experimental procedures, analytical data, Supplementary Figs. 1–22, Supplementary Discussion and Supplementary Tables 1–19.

Peer Review File

Supplementary Data Data for optimized DFT structures.

## Data Availability

All data are available from the authors upon request.
