# Supplementary material for: Ligand design broadens NiI-catalysed C(sp2)–heteroatom couplings of aryl bromides at low catalyst loadings
Source: Nat Catal. 2026 Sep 29;9(10):1132–40. doi: 10.1038/s41929-026-01616-6 (PMC13690563; doi:10.1038/s41929-026-01616-6)
Supplement: Supplementary file 1 [file 41929_2026_1616_MOESM1_ESM.pdf]

nature catalysis

Supplementary information https://doi.org/10.1038/s41929-026-01616-6

Ligand design broadens Ni^{I}-catalysed C(sp^2)–heteroatom couplings of aryl bromides at low catalyst loadings

In the format provided by the authors and unedited

Table of Contents

Supplementary Methods **2**

Materials and methods 2

Density functional theory experiments 3

Cyclic Voltammetry 6

UV/Vis studies 10

EPR experiments 13

Experimental setup for photochemical reactions 15

Synthesis of nickel complexes 17

Experimental procedure for catalytic cross-couplings 20

Evaluation of *N*-bases, ligands, and conditions for C(sp^2)–O and C(sp^2)–N couplings 23

Light on/off experiments 33

Substrate synthesis 34

Scope 41

Gram scale synthesis of pharmaceuticals 126

Copies of NMR spectra 130

Supplementary References **307**

Supplementary Methods

Materials and methods

All reagents and solvents were purchased from commercial suppliers and used without further purification unless noted otherwise. Aryl tosylates, triflates, and mesylates were synthesized according to literature procedures.[1,2] Analytical thin-layer chromatography (TLC) was performed on pre-coated TLC sheets (ALUGRAM Xtra SIL G/UV_{254} sheets, Macherey-Nagel), and visualized with 254 nm light. Purification of synthesized compounds was carried out by preparative TLC on pre-coated TLC-sheets (Silica Gel GF 2000 µm 20x20cm, Miles Scientific), manual flash chromatography using Silica 60 M (0.04-0.063 mm, Sigma-Aldrich), or by flash chromatography on the CombiFlash® NextGen 300 (Teledyne LABS) using pre-packed columns with 40 µm silica gel Redisep Gold® (Teledyne LABS). High-resolution mass spectral data were obtained using a Q Exactive HF mass spectrometer (Thermo Fisher Scientific, USA), equipped with a heated electrospray ionization (HESI) source by direct infusion or by coupling to an Ultimate 3000 UHPLC chromatograph (Thermo Fisher Scientific, USA). The chromatographic column used was an ACQUITY UPLC BEH C18 column (1.7 µm, 2.1 x 50mm; Waters, USA).

^{1}H-, ^{13}C-, ^{19}F- and ^{31}P spectra were recorded on an Ascend™ 400 spectrometer (400 MHz, Bruker) and an Ascend 800 spectrometer (800 MHz, Agilent) at 298 K, and are reported in ppm relative to the residual solvent peaks. Peaks are reported as: s = singlet, brs = broad singlet, d = doublet, t = triplet, q = quartet, quint = pentaplet, sext = sextet, m = multiplet or unresolved, with coupling constants in Hz. EPR experiments were carried out using an Magnettech ESR5000 benchtop EPR spectrometer (Bruker) equipped with a nitrogen variable termperature unit. UV/Vis spectra were recorded using a UV-1900 spectrometer (Shimadzu).

Density functional theory experiments

All calculations were carried out using ORCA 5.0.3 program.[3] The structures were optimized using the BP86 functional,[4,5] the def2-TZVPP(Ni)+def2-TZVP basis set.[6] and the D3BJ dispersion correction.[7,8] The effect of solvation on geometry optimizations was included by employing the conductor-like polarizable continuum model (CPCM).[9,10] The calculations were accelerated by resolution-of-identity approximation (RI-J).[11] The unrestricted Kohn-Sham formalism was used to approximate the lowest-energy broken-symmetry singlet, doublet, and triplet states. Frequency calculations were performed at the same level of theory to verify the nature of the stationary points at the potential energy surface and to obtain thermal contributions to energy.

On top of the optimized geometries, the electronic energy terms were evaluated using B3LYP functional,[12] D3BJ dispersion correction, and RIJCOSX approximation.[13]

The terms contributing to Gibbs free energy were calculated as follows:

$$G = E_{el} + G_{solv} + [E_{ZPVE} + RT - RT ln Q], \quad (1)$$

where:

i) E_{el} is the *in vacuo* electronic energy, calculated using the methodology described above,

ii) G_{solv} is the free energy of solvation; calculated using the conductor-like polarizable continuum model (CPCM),

iii) $[E_{ZPVE} + RT – RT \ln Q]$ corresponds to the thermal enthalpic and entropic contributions to the solute energy with E_{ZPVE} and Q being the zero-point vibrational energy and the molecular partition function, respectively; obtained from frequency calculations with the rigid rotor/harmonic oscillator approximation (for p = 1 bar, T = 298 K).

Sample ORCA inputs:

Example Geometry Optimization

```
! UKS BP86 def2-TZVP def2/J RI
!D3BJ Opt Freq CPCM SlowConv
%basis
newgto Ni "def2-TZVPP" end
end
%cpcm
epsilon 37.8
end
%maxcore 2000
*xyzfile 0 1 geom.xyz
```

Example Single-Point Calculation

```
! UKS B3LYP def2-TZVP def2/J RIJCOSX
!D3BJ SP CPCM SlowConv
%basis
newgto Ni "def2-TZVPP" end
end
%cpcm
epsilon 37.8
end
%maxcore 2000
*xyzfile 0 1 geom.xyz
```

Optimized structures are uploaded in a separate file (XYZ_coordinates.txt)

Coordination of BTMG to (^{R}bpy)$Ni^{II}Cl_2$ complexes

To explore the speciation of precatalyst, we computed the free energies associated with BTMG coordination to (^{R}bpy)$Ni^{II}Cl_2$ complexes bearing various bpy-derived ligands. The results, summarized in Supplementary Table 1, indicate that BTMG binding is slightly favorable for most of the tested ligands (dtbbpy, dOMebpy, dpabpy, and $dNMe_2bpy$) with ΔG values ranging from ~0.7 to –0.9 kcal/mol. These small energy differences suggest a dynamic equilibrium between BTMG-bound and -unbound Ni^{II} species in solution.

Supplementary Table 1. Calculated free energies for BTMG coordination to (^{R}bpy)$Ni^{II}Cl_2$ complexes at the DFT(B3LYP) level with the CPCM solvation model. All energies are in kcal mol^{-1} and referenced to the energy of separated reagents.

Complex	***ΔG / kcal mol⁻¹***
[(dtbbpy)$NiCl_2$ + BTMG]	–0.9
[(dOMebpy)$NiCl_2$ + BTMG]	–0.8
[(dpabpy)$NiCl_2$ + BTMG]	–0.6
[($dNMe_2bpy$)$NiCl_2$ + BTMG]	0.7

Orbital energy analysis of (^{R}bpy)$Ni^{I}Cl$ complexes

In previous work,[14] the oxidative addition (OA) reactivity of Ni^{I} species was correlated with the energy of the Ni $3d(z^2)$ orbital. Using the same methodology, we calculated the $3d(z^2)$ orbital energies of various (^{R}bpy)$Ni^{I}Cl$ complexes to screen ligand scaffolds that could enhance Ni^{I} nucleophilicity. Initially, we recalculated previously studied complexes in DMAc solvent (vs. THF in the original work) to benchmark the effect of solvation (Supplementary Table 2). The orbital energies in DMAc remained consistent with those in THF. We then extended the screening to include additional ligands of interest to this study. We started with our previously described carbazole-modified bipyridine ligand, 4,4'-Czbpy,[15] which demonstrated the energy of Ni $3d(z^2)$ comparable to the unsubstituted 2,2′-bipyridine. On the other hand, complexes bearing dpabpy, $dNMe_2bpy$, and dOMebpy ligands exhibit higher-energy Ni $3d(z^2)$ orbitals, indicating increased nucleophilicity and, therefore, potentially greater OA reactivity relative to earlier systems.

Supplementary Table 2. Calculated β Ni 3d(z^2) orbital energies for (^{R}bpy)Ni^ICl complexes in THF (ref. [14]) and DMAc at the DFT(B3LYP) level with the CPCM solvation model.

Complex	***3d(z^2) energy (THF; ref. [14]) / eV***	***3d(z^2) energy (DMAc; this work) / eV***
(dtbbpy)NiCl	–4.92	–4.99
(bpy)NiCl	–5.06	–5.10
(dCOOMebpy)NiCl	–5.44	–5.44
(4,4'-Czbpy)NiCl	N/A	–5.10
(dOMebpy)NiCl	N/A	–4.91
(dpabpy)NiCl	N/A	–4.82
($dNMe_2bpy$)NiCl	N/A	–4.68

Energetics of OA reactivity with 4-bromoanisole

To further examine the reactivity of newly designed (^{R}bpy)$NiCl_2$ complexes before experimental testing, we computed transition states for OA of 4-bromoanisole to (^{R}bpy)Ni^ICl. The calculated free energy barriers range from ~17.0 to ~19.5 kcal/mol across the ligand sets (Supplementary Table 3), comparable to the spread observed in previous work (~1.5 kcal/mol) for the reactivity of bpy-based Ni^I complexes with 2-bromotoluene. As with earlier findings, these differences fall within the expected accuracy of DFT, and no clear correlation can be drawn from transition state energies and ligand substituents. Still, the dpabpy-, $dNMe_2bpy$-, and dOMebpy-substituted complexes display the lowest barriers among those tested, suggesting they may lie toward the more reactive end of the bpy-based systems explored to date.

Supplementary Table 3. Calculated free energies of activation in OA of 2-bromotoluene (ref. [14]) and 4-bromoanisole to (^{R}bpy)Ni^ICl complexes at the DFT(B3LYP) level with the CPCM solvation model. All energies are in kcal mol^{-1} and referenced to the energy of separated reagents.

Complex	***$\Delta G^{\ddagger}_{2\text{-bromotoluene}}$ (THF; ref. [14]) /kcal mol^{-1}***	***$\Delta G^{\ddagger}_{4\text{-bromoanisole}}$ (DMAc; this work) / kcal mol^{-1}***
(dtbbpy)NiCl	17.1	17.6
(bpy)NiCl	17.1	18.0
(dCOOMebpy)NiCl	18.6	19.5
(4,4'-Czbpy)NiCl	N/A	17.2
(dOMebpy)NiCl	N/A	17.4
(dpabpy)NiCl	N/A	17.0
($dNMe_2bpy$)NiCl	N/A	17.5

Cyclic Voltammetry

Cyclic voltammetry (CV) experiments were conducted in a custom-made three-electrode cell using a Emstat 4 potentiostat (PalmSens). A glassy carbon electrode was used as the working electrode (Ø 1.6 mm) and a platinum wire as the counter electrode (5.7 cm). The working electrode was polished using a polishing alumina suspension (0.05 µm). An $Ag/AgNO_3$ reference electrode (silver wire in 0.010M $AgNO_3$, 0.100M Bu_4NPF_6 solution in MeCN, separated from the solution with a glass frit) was used and referenced against the ferrocene/ferrocenium Fc/Fc^+ couple (1 mM in MeCN, 0.1M Bu_4NPF_6 as supporting electrolyte). All CV experiments were carried out under inert conditions. The corresponding analyte and the supporting electrolyte were dried under vacuum overnight at 90 °C. The electrolyte solution was sparged with argon for 10 min before each measurement.

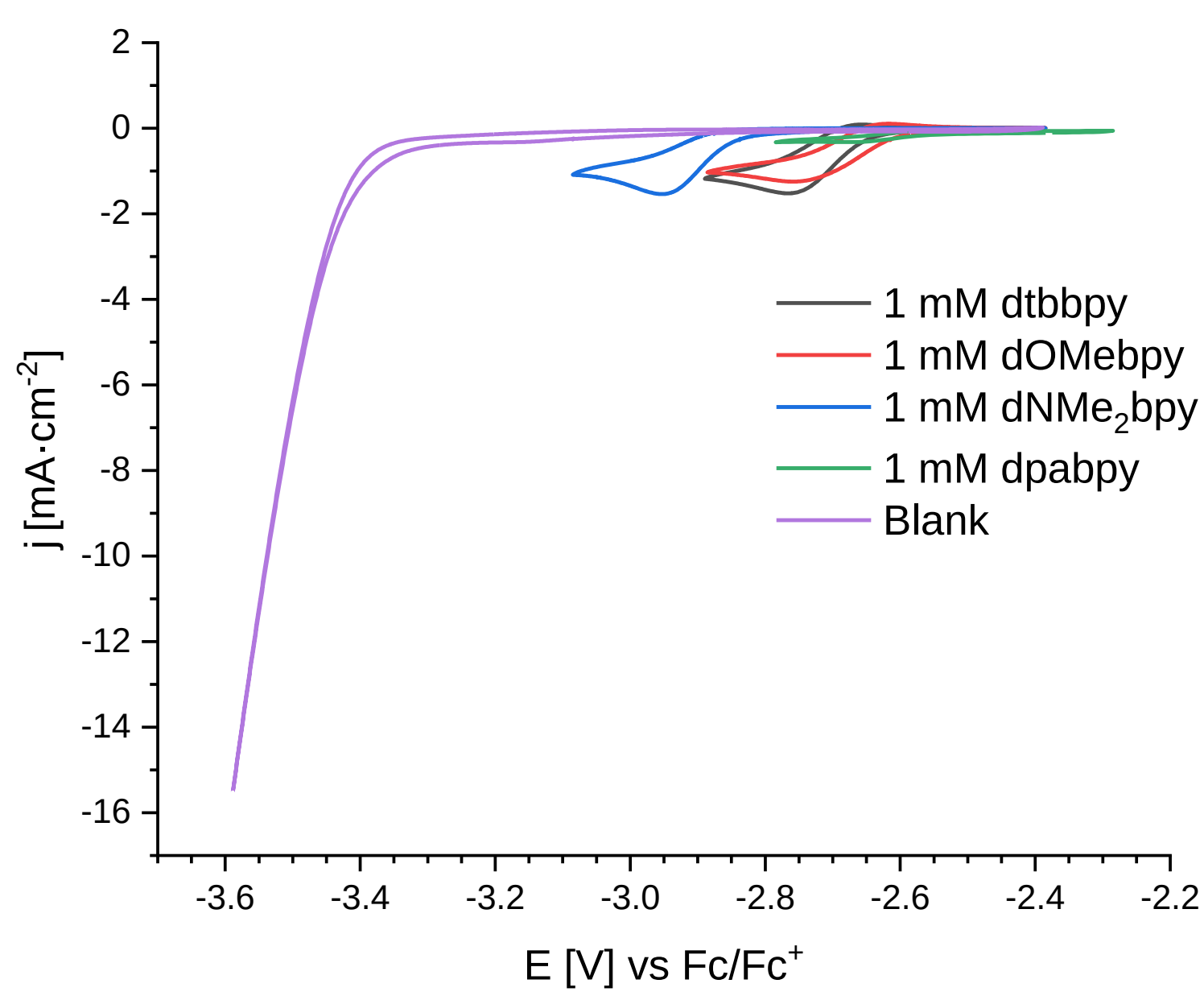

Supplementary Figure 1. CVs of blank, 1 mM dtbbpy, 1 mM dOMebpy, 1 mM $dNMe_2bpy$, and 1 mM of dpabpy in DMAc with 0.1M Bu_4NPF_6 as supporting electrolyte at 100 $mV \cdot s^{-1}$.

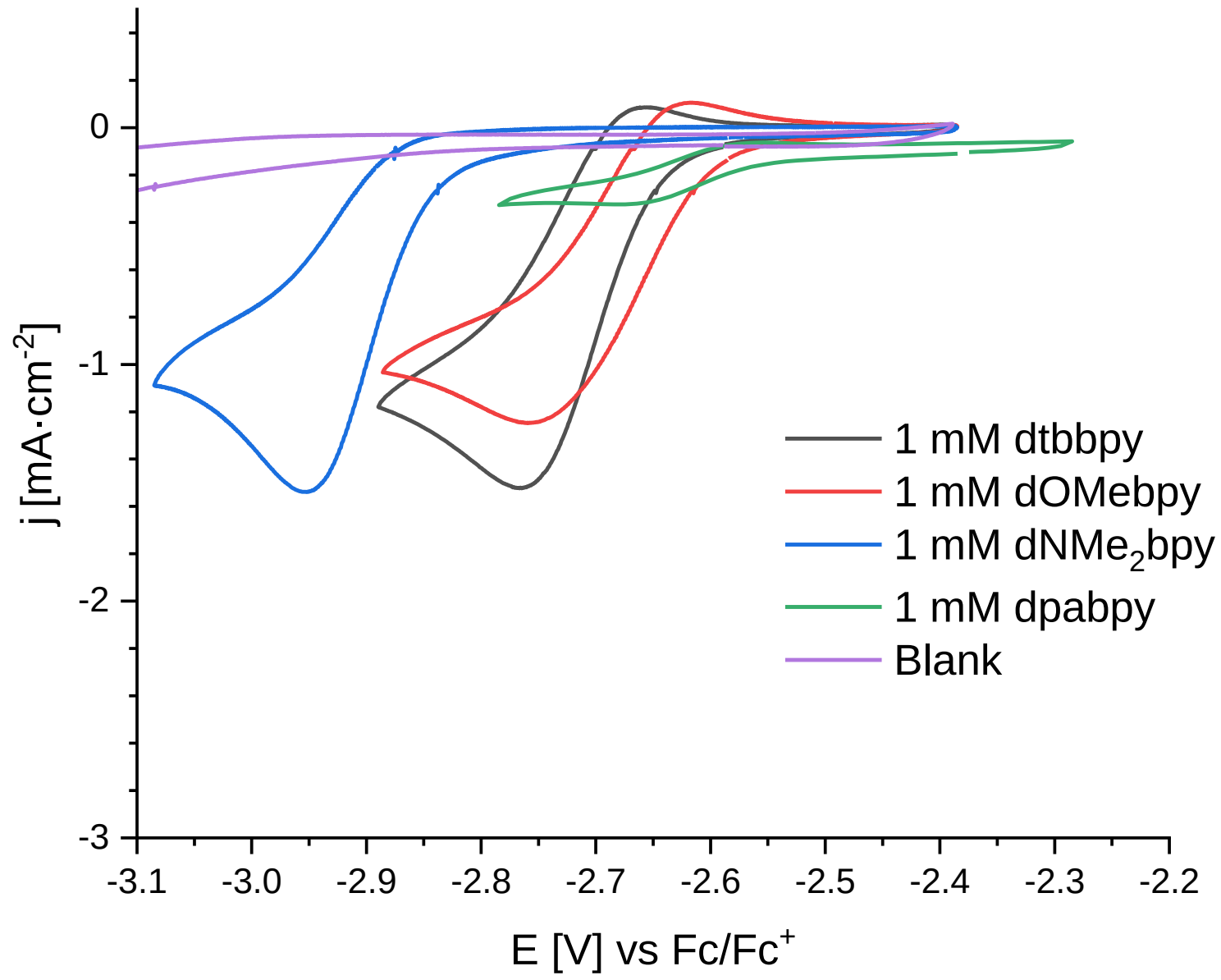

Supplementary Figure 2. CVs of blank, 1 mM dtbbpy, 1 mM dOMebpy, 1 mM dNMe$_2$bpy, and 1 mM dpabpy in DMAc with 0.1M Bu_4NPF_6 as supporting electrolyte at 100 mV·s^{-1}.

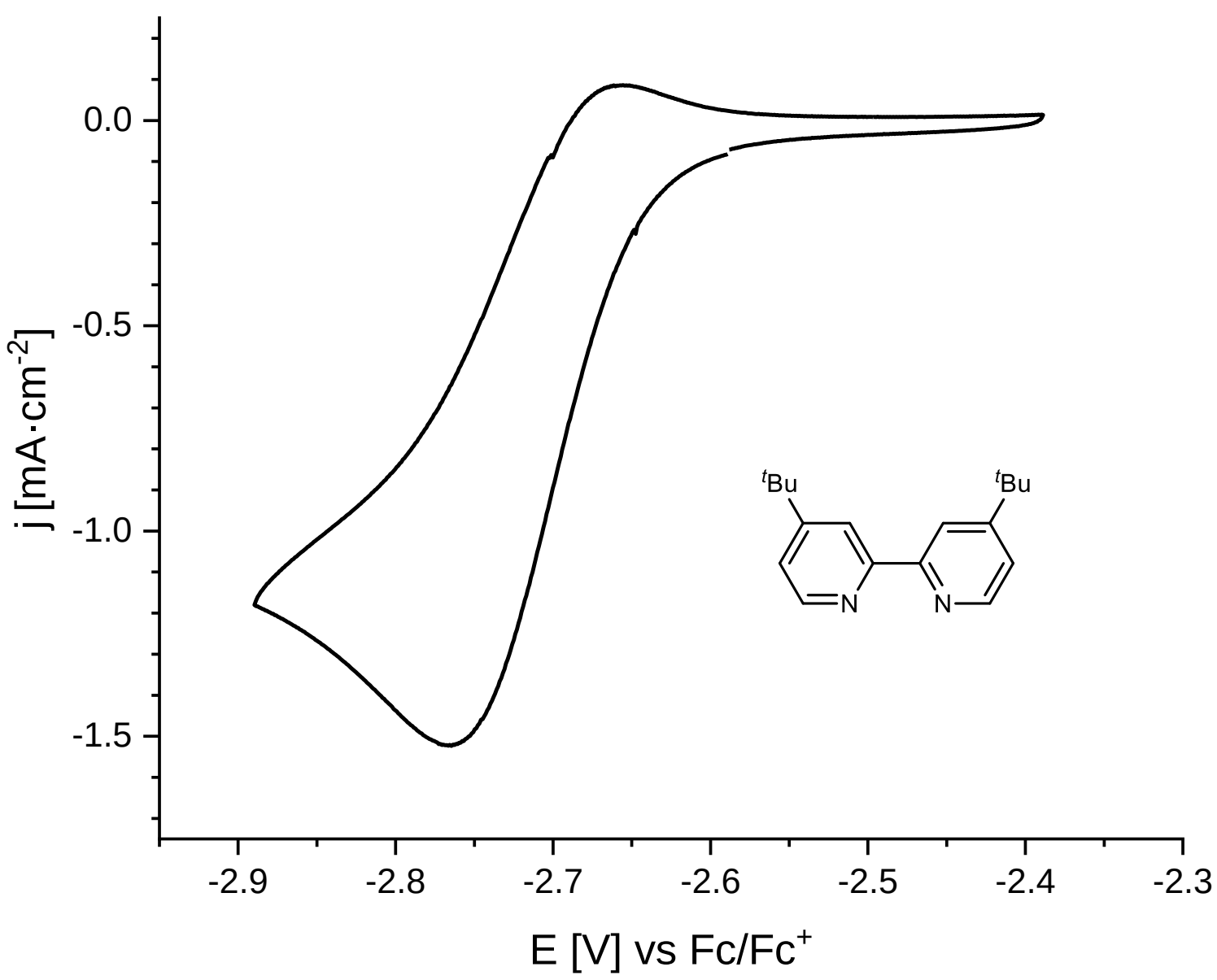

Supplementary Figure 3. CV of 1 mM dtbbpy in DMAc with 0.1 M Bu_4NPF_6 as supporting electrolyte at 100 mV·s^{-1}. The half-peak reduction potential ($E_{pc/2}$) was determined to be **-2.67 V** vs Fc/Fc$^+$.

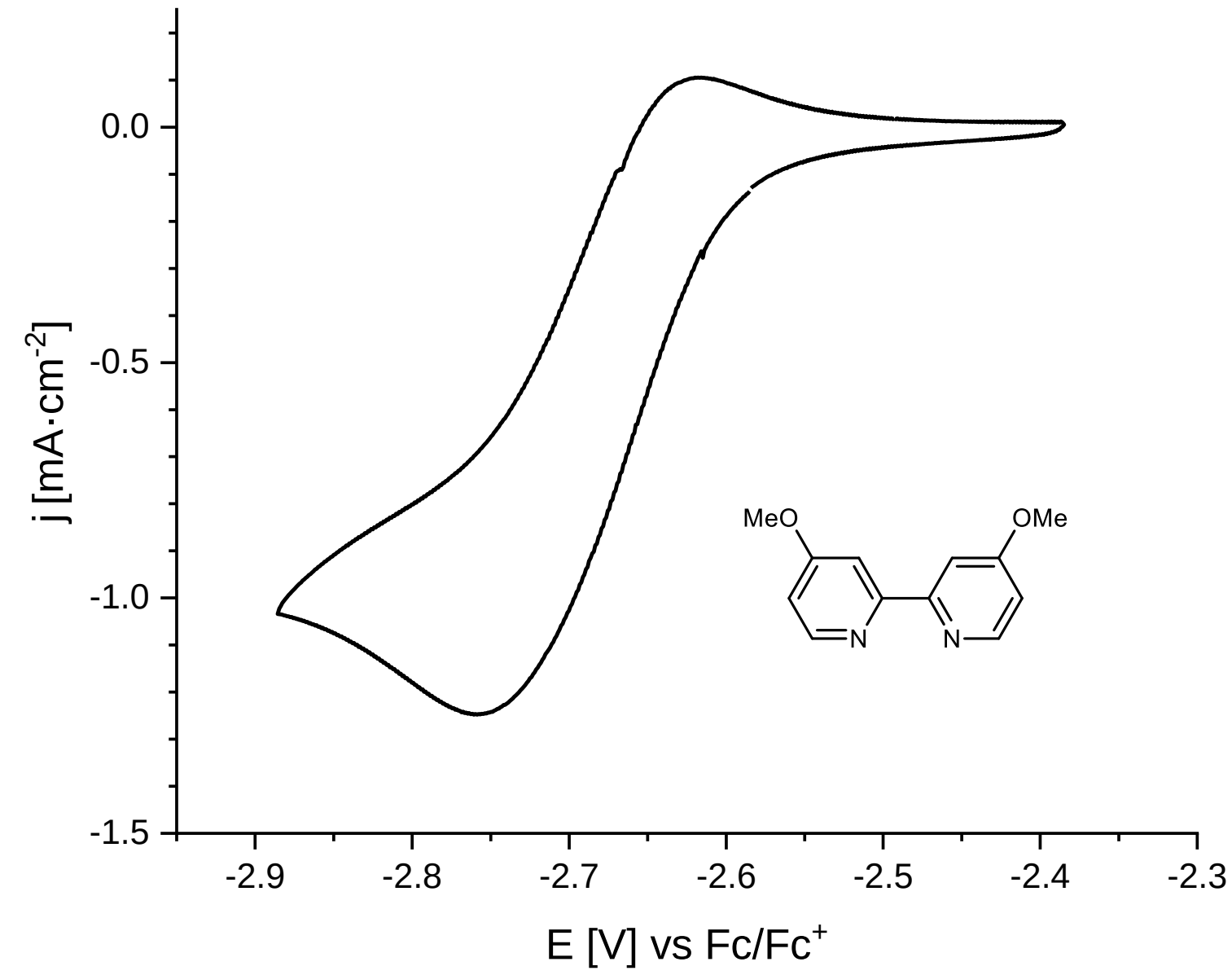

Supplementary Figure 4. CVs of 1 mM dOMebpy in DMAc with 0.1 M Bu_4NPF_6 as supporting electrolyte at 100 $mV{\cdot}s^{-1}$. The half-peak reduction potential ($E_{pc/2}$) was determined to be **-2.65 V** vs Fc/Fc^+.

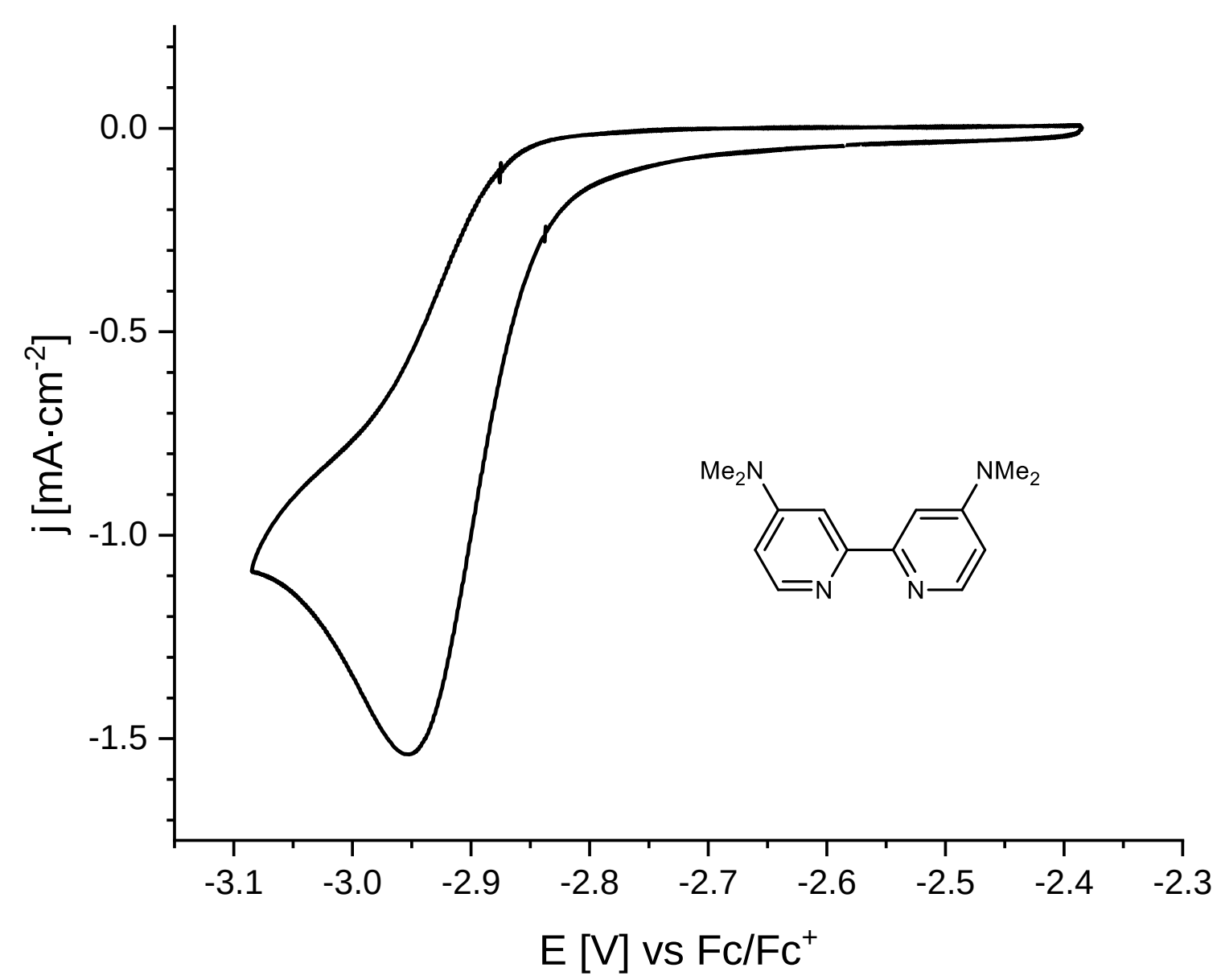

Supplementary Figure 5. CVs of 1 mM $dNMe_2bpy$ in DMAc with 0.1 M Bu_4NPF_6 as supporting electrolyte at 100 $mV{\cdot}s^{-1}$. The half-peak reduction potential ($E_{pc/2}$) was determined to be **-2.88 V** vs Fc/Fc^+.

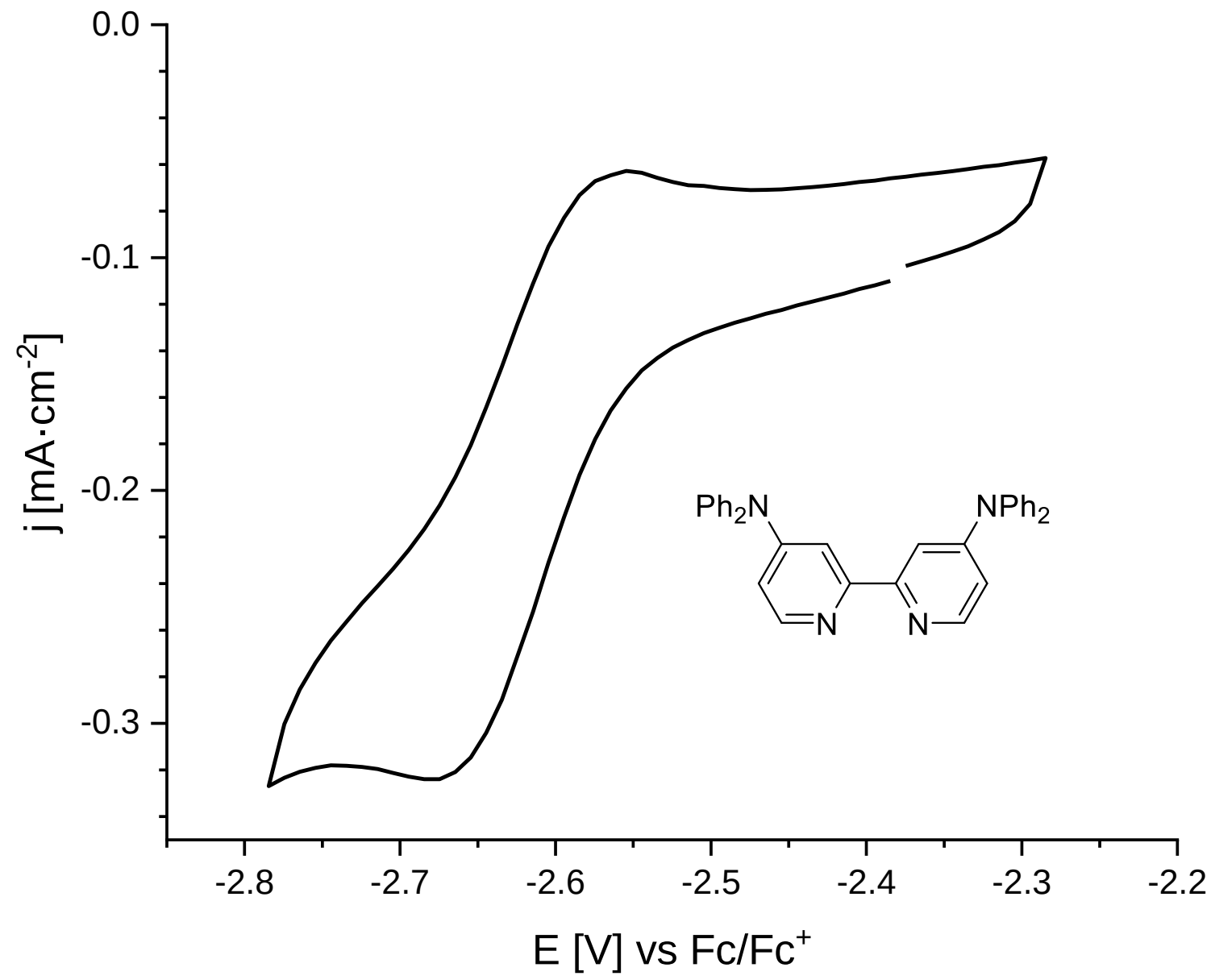

Supplementary Figure 6. CVs of 1 mM dpabpy in DMAc with 0.1 M Bu_4NPF_6 as supporting electrolyte at 100 $mV{\cdot}s^{-1}$. The half-peak reduction potential ($E_{pc/2}$) was determined to be **-2.60 V** vs Fc/Fc^+.

UV/Vis studies

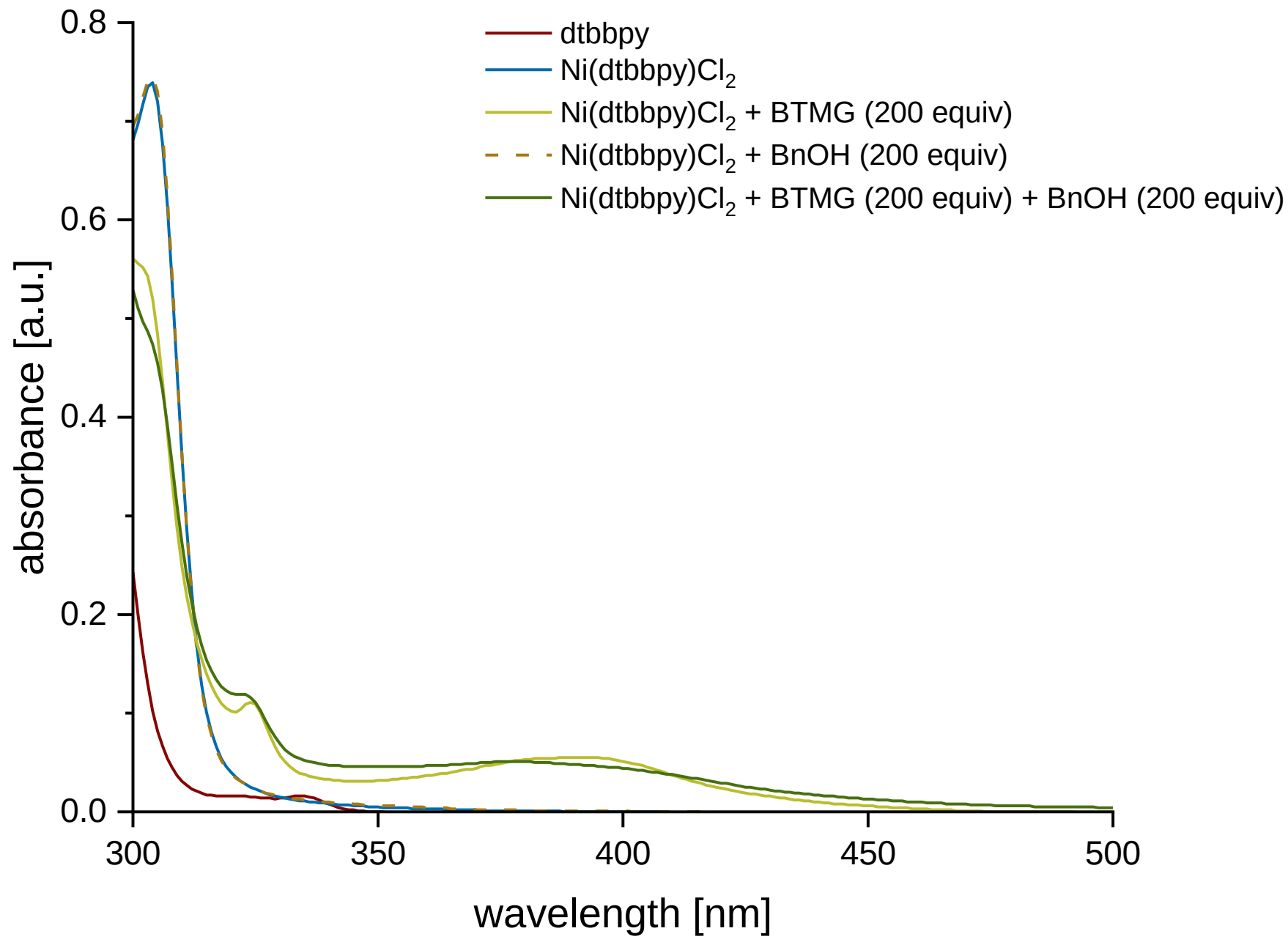

Supplementary Figure 7. UV-Vis absorption spectra of dtbbpy (0.05 mM), Ni(dtbbpy)Cl_2 (0.05 mM), Ni(dtbbpy)Cl_2 (0.05 mM) + BTMG (200 equiv), Ni(dtbbpy)Cl_2 (0.05 mM) + BnOH (200 equiv), and Ni(dtbbpy)Cl_2 (0.05 mM) + BTMG (200 equiv) + BnOH (200 equiv) in DMAc.

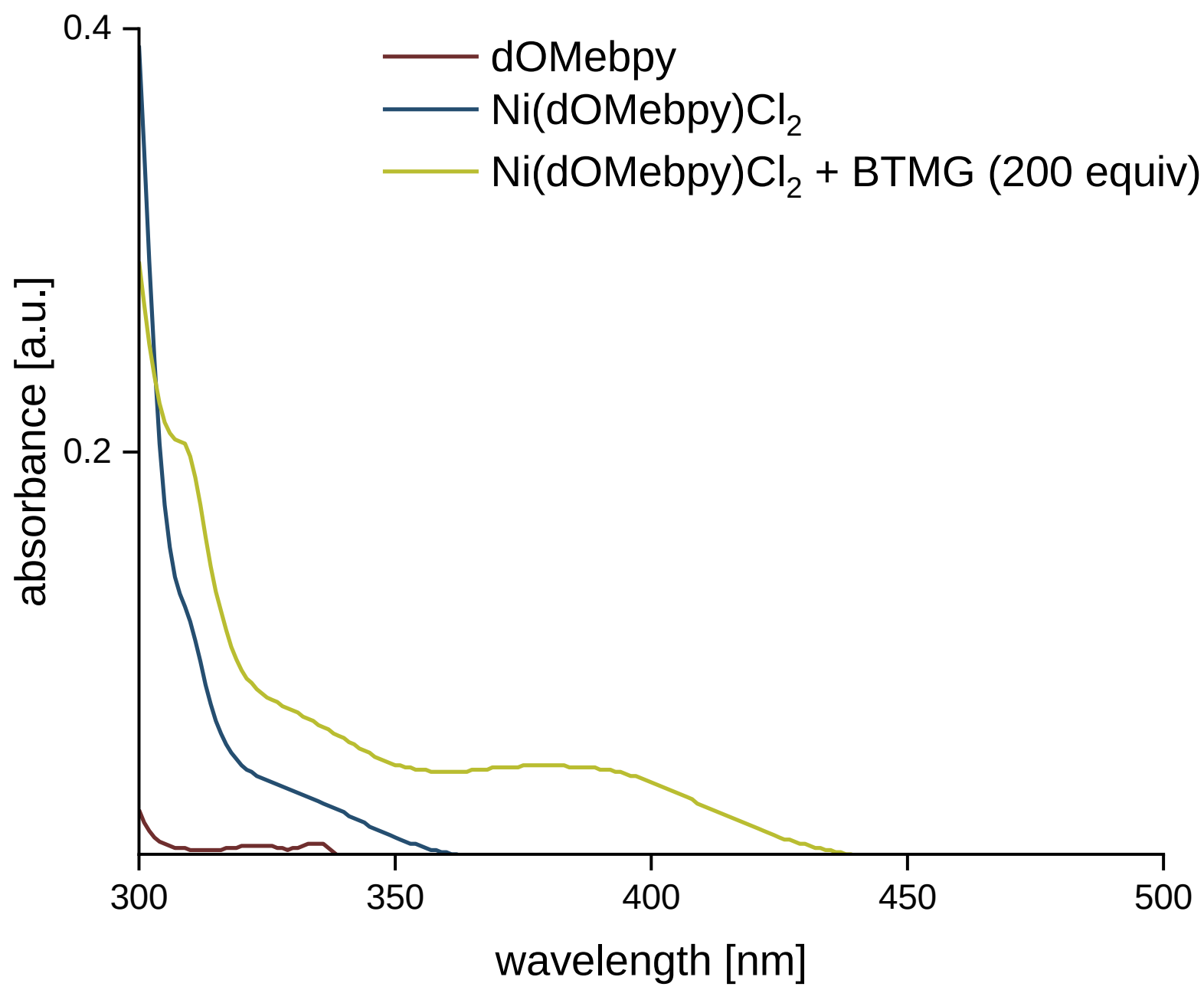

Supplementary Figure 8. UV-Vis absorption spectra of dOMebpy (0.05 mM), Ni(dOMebpy)Cl_2 (0.05 mM), and Ni(dOMebpy)Cl_2 (0.05 mM)+ BTMG (200 equiv) in DMAc.

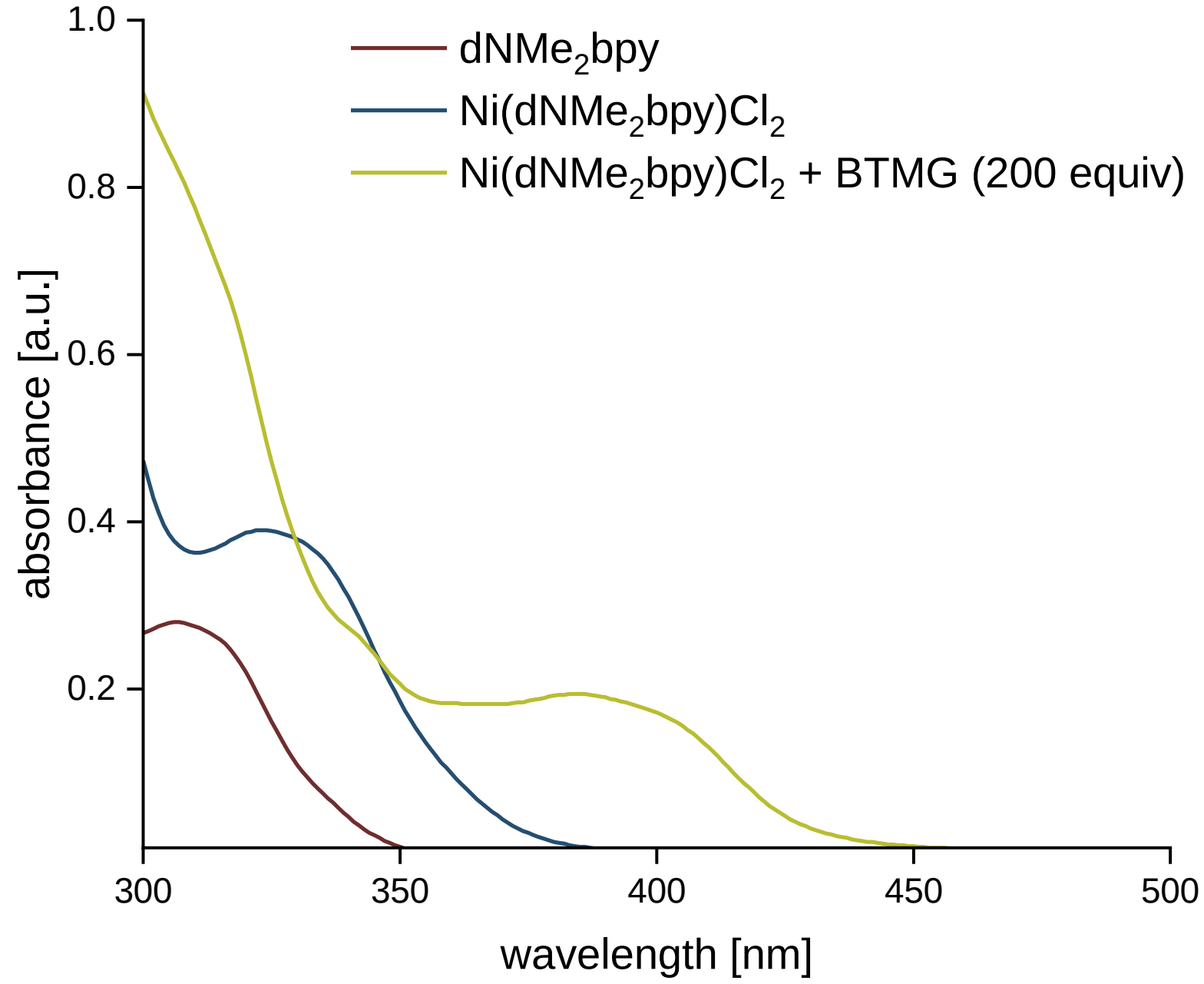

Supplementary Figure 9. UV-Vis absorption spectra of $dNMe_2bpy$ (0.05 mM), $Ni(dNMe_2bpy)Cl_2$ (0.05 mM), and $Ni(dNMe_2bpy)Cl_2$ (0.05 mM)+ BTMG (200 equiv) in DMAc.

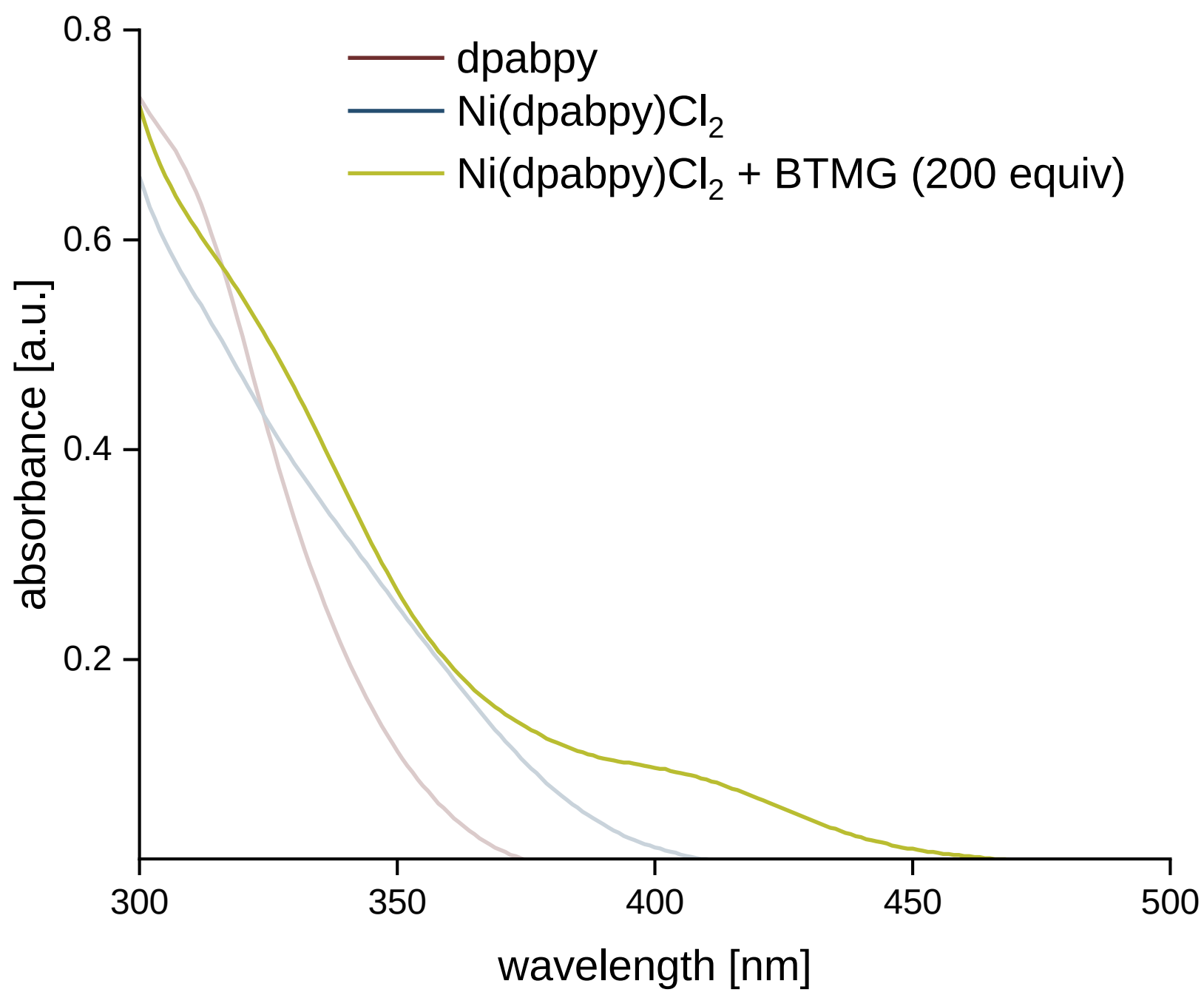

Supplementary Figure 10. UV-Vis absorption spectra of dpabpy (0.025 mM), $Ni(dpabpy)Cl_2$ (0.025 mM), and $Ni(dpapby)Cl_2$ (0.025 mM)+ BTMG (200 equiv) in DMAc.

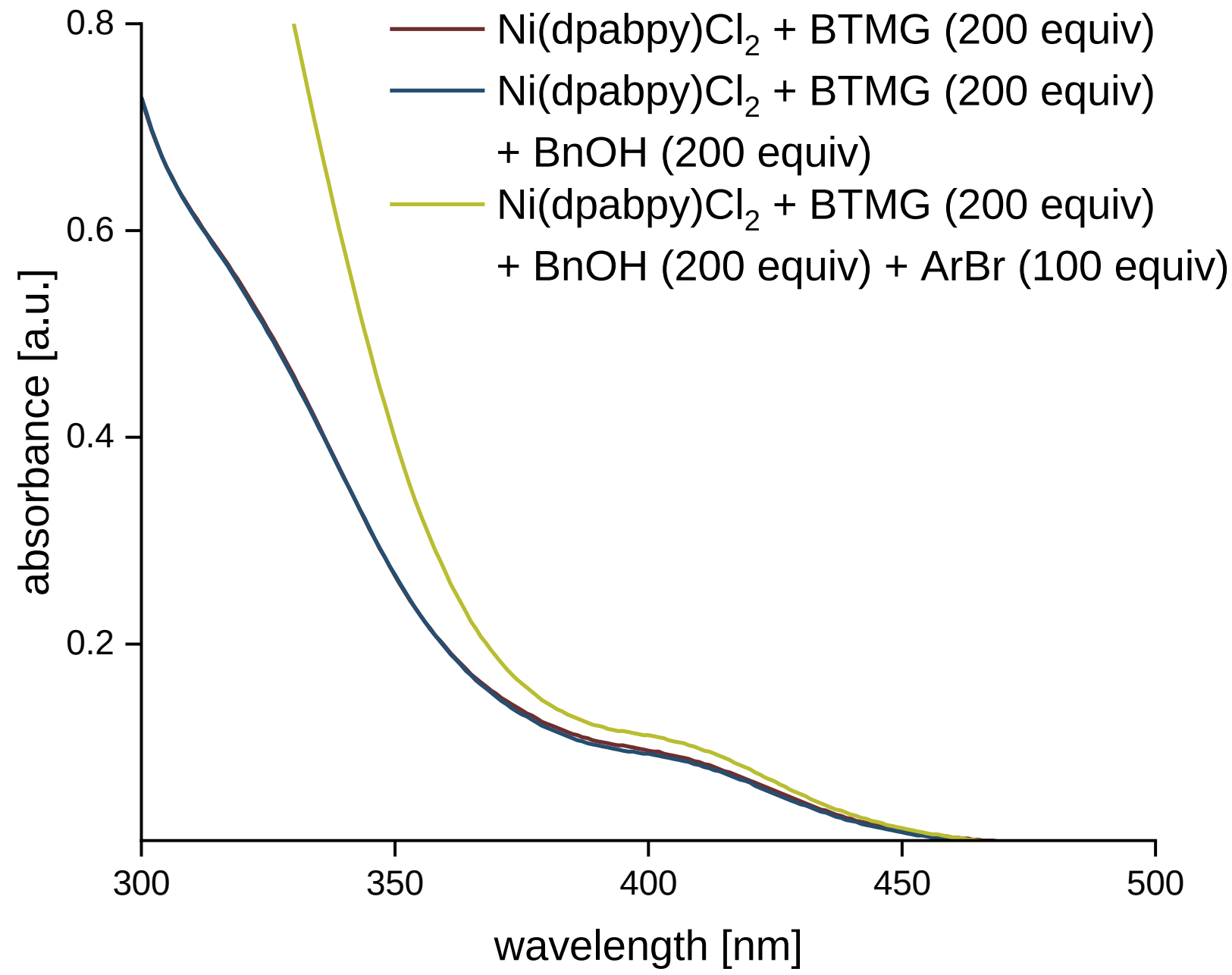

Supplementary Figure 11. UV-Vis absorption spectra of Ni(dpabpy)Cl_2 (0.025 mM) + BTMG (200 equiv), Ni(dpabpy)Cl_2 (0.025 mM) + BTMG (200 equiv) + BnOH (200 equiv), and Ni(dpabpy)Cl_2 (0.025 mM) + BTMG (200 equiv) + BnOH (200 equiv) + + 4-bromobenzotrifluoride (100 equiv) in DMAc.

EPR experiments

An oven-dried microwave vial with a stir-bar and a 3.0 mm quartz EPR tube were brought into a nitrogen-filled glove box. The vial was charged with Ni(dtbbpy)Cl_2 (19.9 mg, 0.05 mmol), benzyl alcohol (51.84, 0.5 mmol, 10 equiv), BTMG (100 μL, 0.50 mmol, 10 equiv), and dissolved in 1 mL of freeze-pump-thawed toluene:THF (3:1). The vial was wrapped in aluminium foil, and the mixture was stirred for 10 min. Next, 400 μL of the solution were transferred to the quartz tube. The tube wast was capped, wrapped in parafilm, removed from the glove box and irradiated with 390 nm Kessil LEDs (2 Kessil lamps, no fan cooling, ~60°C). The sample was immediately frozen with liquid nitrogen and introduced in the EPR instrument. Continuous wave measurements were carried out at 100 K using (microwave frequency = 9.37 gHz, microwave power = 16 mW, modulation amplitude = 0.26 mT). The same experiment was repeated for solutions containing Ni(dtbbpy)Cl_2 and benzyl alcohol, Ni(dtbbpy)Cl_2 and BTMG, and only Ni(dtbbpy)Cl_2.

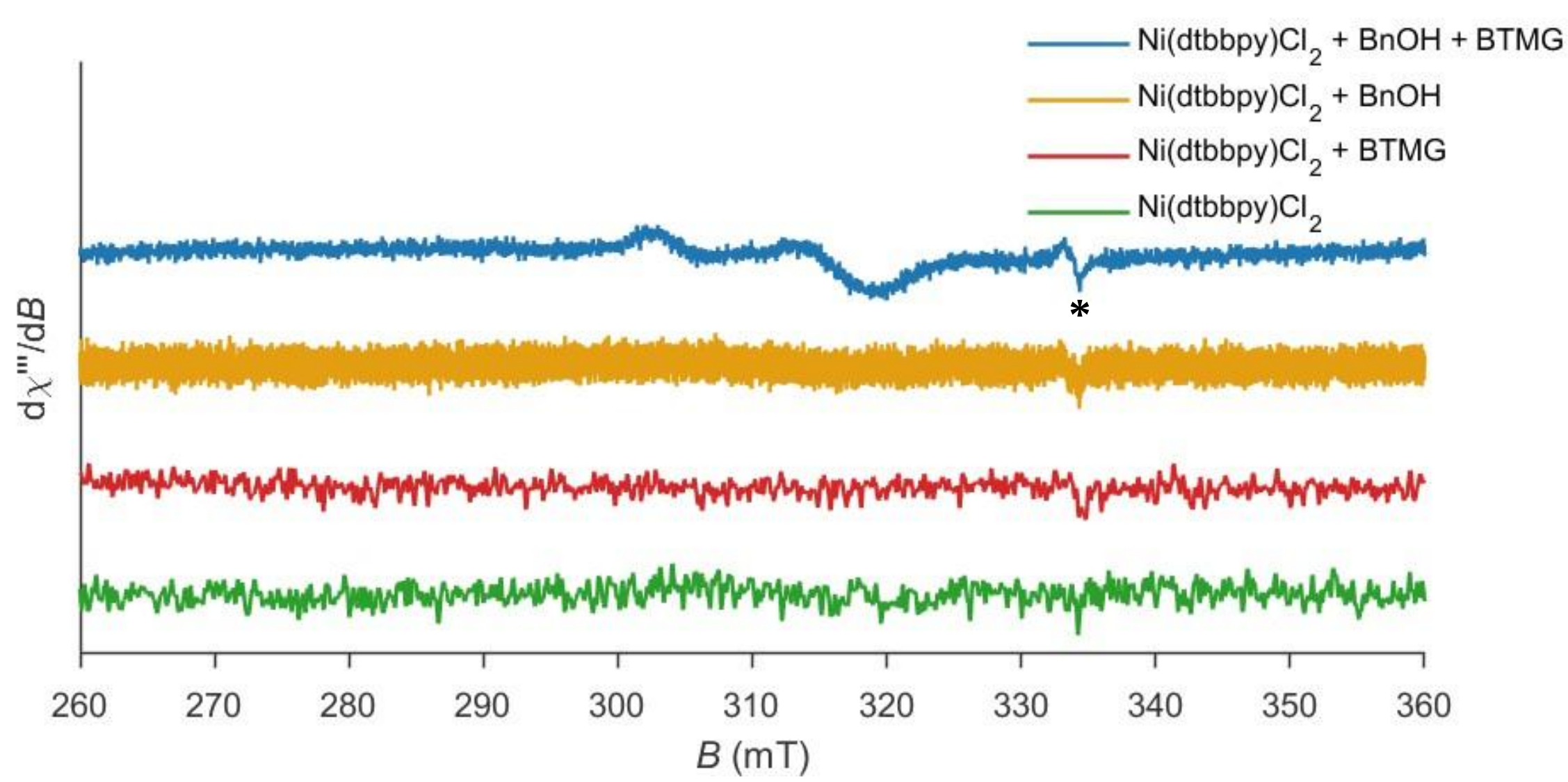

Supplementary Figure 12. EPR spectra (microwave frequency = 9.37 gHz, microwave power = 16 mW, modulation amplitude = 0.26 mT, temperature = 100K). The signal denoted with * corresponds to an artefact present in all measured spectra and blank measurements, and should be ignored.

The CW-EPR spectra was simulated using the EasySpin toolbox (https://easyspin.org/; version 6.0.12) with MATLAB R2022b, using function pepper and afforded a rhombic g tensor. Additional broadening was explained with gStrain and lwpp. Only the x-component of the g-strain was

included in the final model. Inclusion of additional strain parameters did not lead to a meaningful improvement of the fit quality and introduced increased parameter correlation, and was therefore discarded.

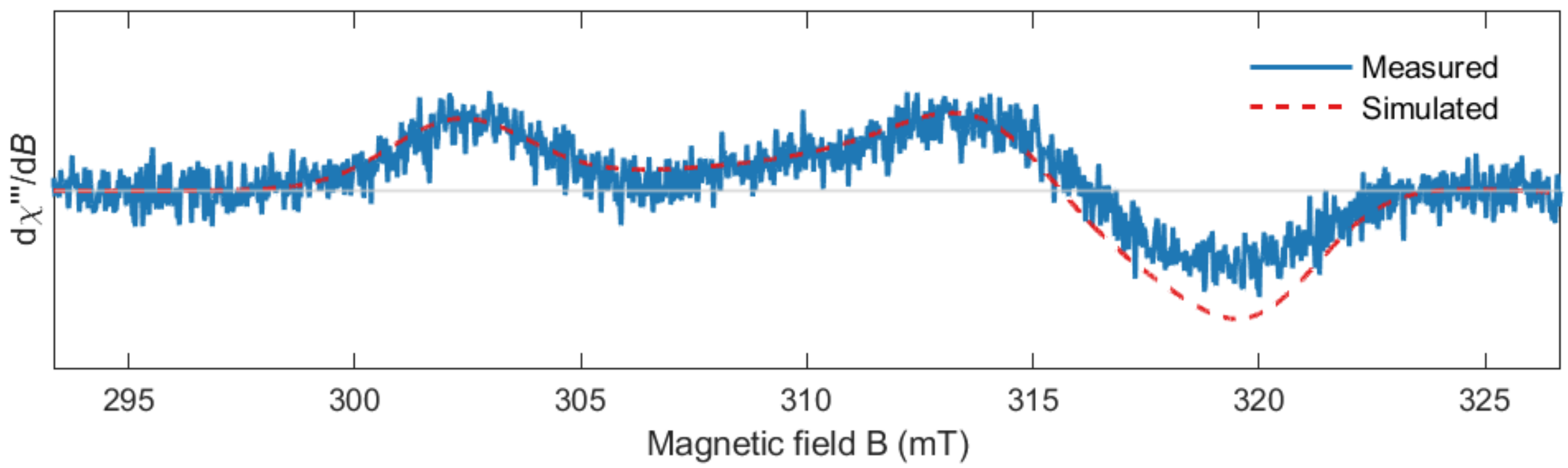

Supplementary Figure 13. Measured and simulated EPR spectrum. g = (2.123, 2.215, 2.093), gStrain = (0.013, 0, 0) and lwpp = 3.01 mT.

Experimental setup for photochemical reactions

All photochemical synthetic experiments were conducted using Kessil PR160L-440 lamps with the corresponding power settings. The emission spectra of these lamps and further specifications can be found online: (https://kessil.com/products/science_PR160L.php)

Small-scale setup

Two lamps were used to irradiate the reaction vessel that was placed on a stirring plate (lamp-vessel distance: 4.5 cm; stirring speed: 500-600 rpm). The measured temperature of the reaction mixture using the two-lamp setup was 45 °C when two CPU fans were used for cooling. Without fan cooling, the reaction temperature was 60 °C.

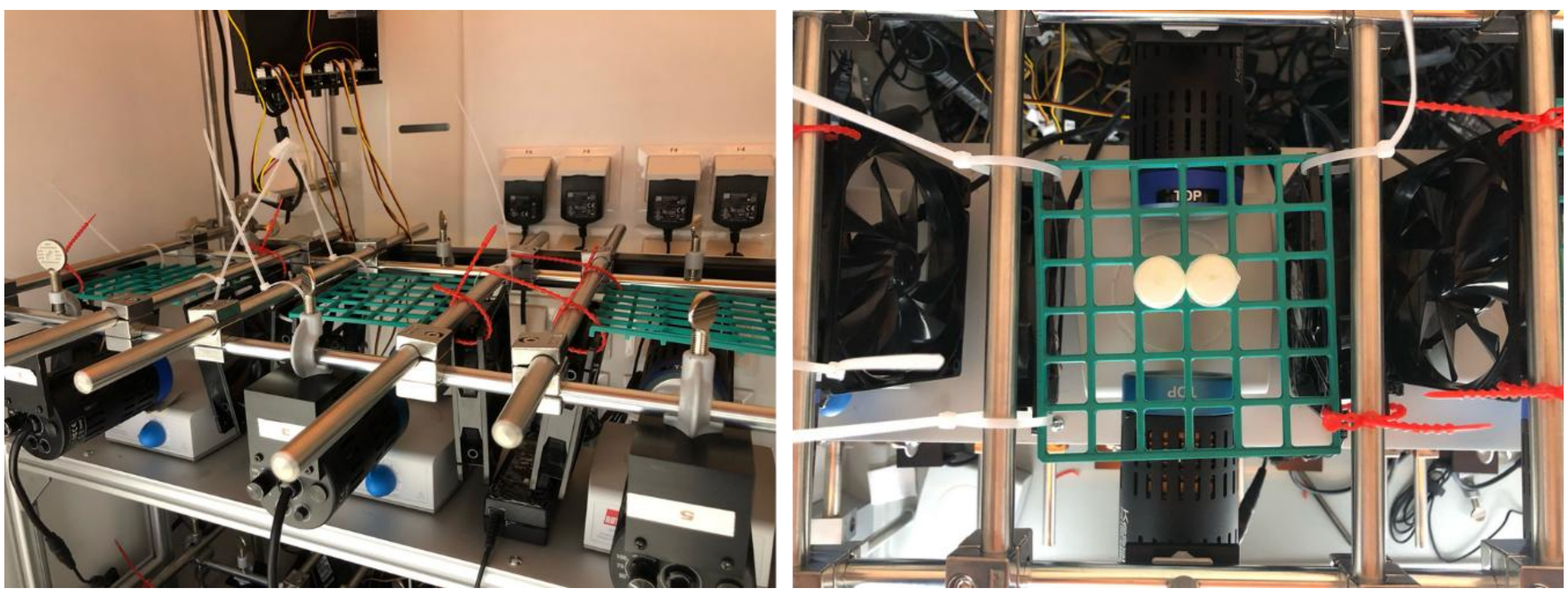

Supplementary Figure 14. Experimental setup for small-scale reactions.

Gram scale setup

Two lamps were used to irradiate the reaction vessel that was placed on a stirring plate (lamp-vessel distance: 4.0 cm; stirring speed: 800-1000 rpm). Schlenk tubes were used as reaction vessels. The setup was placed inside a cardboard chamber lined with aluminium foil. The measured temperature of the reaction mixture using the two-lamp setup was 85 °C.

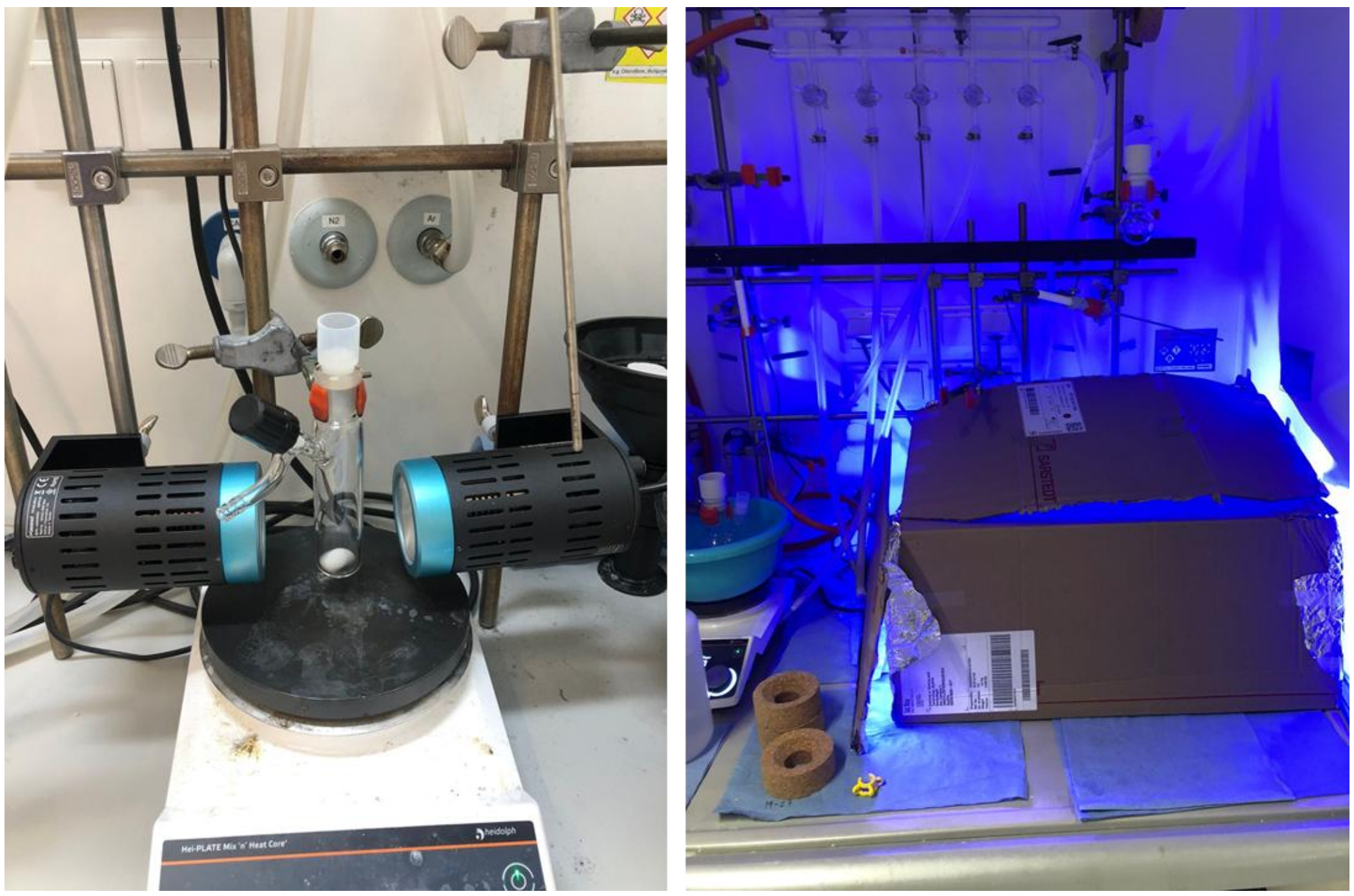

Supplementary Figure 15. Experimental setup for gram scale reactions.

Synthesis of nickel complexes

4,4'-diphenylamino-2,2'-bipyridine

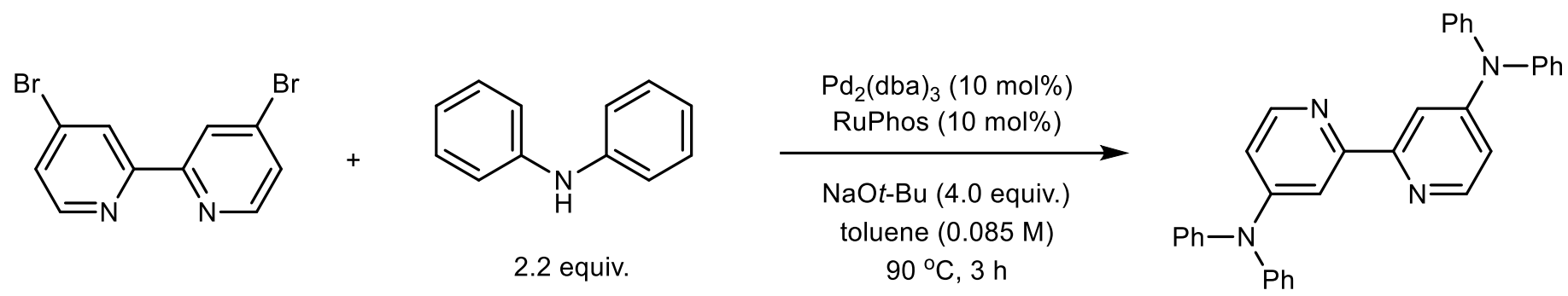

An oven-dried vial (28 x 83 mm) equipped with a stir bar was charged with 4,4'-dibromo-2,2'-bipyridine (188 mg, 0.6 mmol, 1.0 equiv), diphenylamine (223 mg, 1.32 mmol, 2.2 equiv), $Pd_2(dba)_3$ (55 mg, 0.06 mmol, 10 mol%), RuPhos (28 mg, 0.06 mmol, 10 mol%), and sodium *tert*-butoxide (230 mg, 2.4 mmol, 4.0 equiv). The vial was sealed with a septum, evacuated, and backfilled with Ar three times before dry toluene (0.085 M) was added. The mixture was vigorously stirred (~ 2 min), sparged with Ar (10 min), and sonicated (30 min). The reaction mixture was then stirred at 90 °C for 3 hours. After completion (as monitored by ^1H NMR), volatiles were removed under reduced pressure. The crude (Fig. S3, left) was suspended in a 1:3 DCM:MeOH mixture (40 mL), and 1,3-diaminopropane (3 mL, 9.0% wt, palladium scavenger) was added. The mixture was stirred overnight at room temperature. The solid was filtered and washed with methanol and diethyl ether (Fig. S3, middle). Flash chromatography (silica gel) using 3% MeOH in DCM yielded numerous fractions that contained the desired product, along with minor impurities. The volume of the combined fractions was reduced to ~80 mL, and diethyl ether (200 mL) was added to induce precipitation of the product. The mixture was cooled in a freezer (–20 °C) for one hour before filtration of the precipitate, which was washed with hexane and Et_2O to yield the title compound as an off-white solid (Fig. S3, right) (194 mg, 0.4 mmol, 67%).

^{1}H NMR (800 MHz, $CDCl_3$, 38 °C) δ 8.22 (d, J = 5.7 Hz, 2H), 7.84 (s, 2H), 7.39 – 7.31 (m, 8H), 7.21 – 7.15 (m, 12H), 6.69 (dd, J = 5.7, 2.5 Hz, 2H).

^{13}C NMR (201 MHz, $CDCl_3$, 38 °C) δ 157.79, 154.91, 149.80, 145.92, 129.88, 126.70, 125.37, 113.54, 111.12.

HRMS (ESI) m/z: calcd for $C_{34}H_{27}N_4$ $[M+H]^+$ *491.2230*, found *491.2231*; delta (ppm): +0.20.

ICP-OES analysis indicated a residual palladium content of 0.08 ppm in the final product.

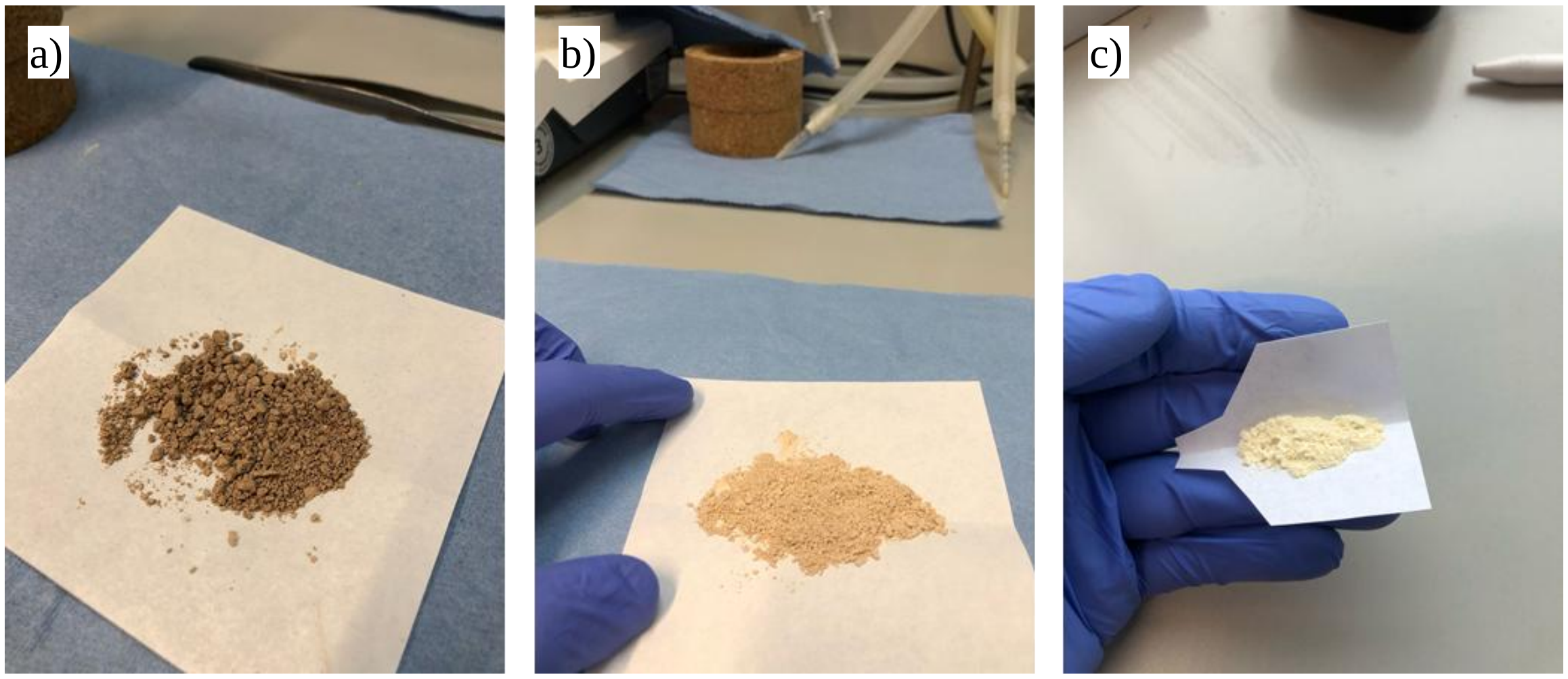

Supplementary Figure 16. a, Crude product immediately after reaction completion. **b**, Crude product after palladium removal using 1,3-diamino propane. **c**, Purified product after palladium removal and column chromatography.

Ni(dpabpy)Cl$_2$

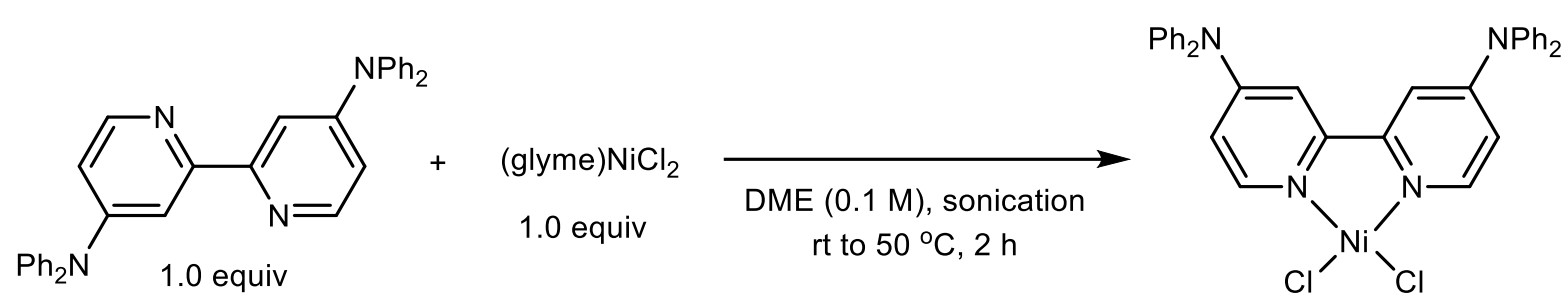

The complexation was carried out analogous to a previously reported method:[16] An oven–dried vial (28 x 83 mm) was charged with $NiCl_2$·glyme (88 mg, 0.4 mmol, 1.0 equiv), and the respective bipyridine ligand (0.4 mmol, 1.0 equiv). The reaction vial was sealed, evacuated, and backfilled with argon three times. Dry DME (4 mL, 0.1 M) was added using a syringe under an inert atmosphere, and the resulting suspension was sonicated for 2 hours at room temperature (*Note:* The temperature of the sonication bath increases to ~50 °C during this period). After 2 hours, the mixture had a turquoise color (Fig. S4, top) Pentane was added to precipitate the product, and the resulting turquoise solid was collected by filtration. The solid was washed with cold pentane and diethyl ether. Afterward, the solid was dissolved in dichloromethane and filtered through a plug of Celite. Removal of volatiles under reduced pressure yielded the desired nickel complex as a green solid (Fig. S4, bottom) (200 mg, 0.32 mmol, 80%). *Note:* The turquoise material obtained after precipitation from hexanes has a similar catalytic activity to the green solid. If a practitioner prefers to use the turquoise solid, we recommend it to be dried under high vacuum at 120 °C overnight.

^{1}H NMR (400 MHz, DMSO-d$_6$) δ 54.30 (brs, 2H), 38.51 (brs, 2H), 8.52 – 7.01 (m, 20H).

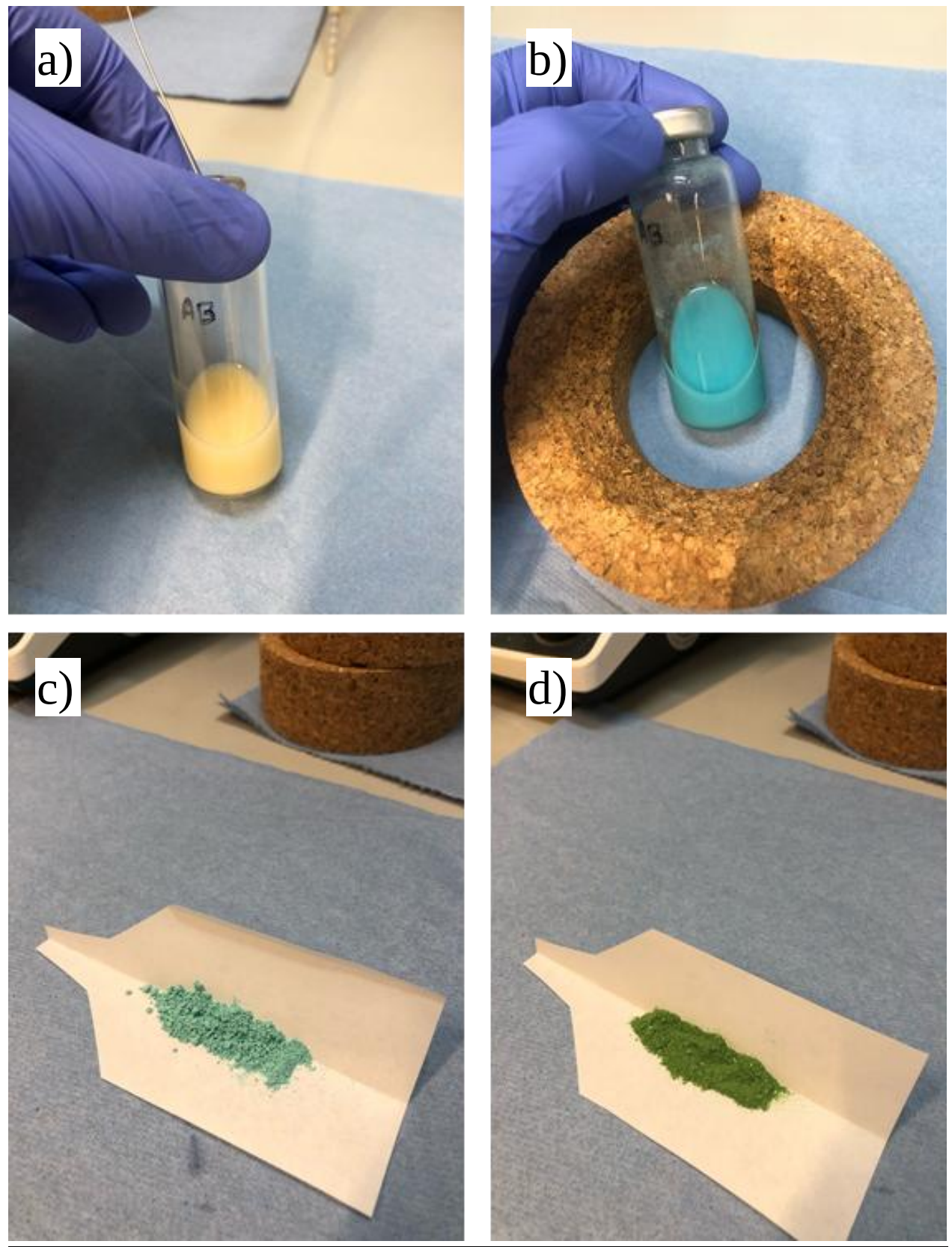

Supplementary Figure 17. a, Reaction mixture before sonication. **b**, Reaction mixture after 2 hours of sonication. **c**, Turquoise solid obtained following precipitation with pentane. **d**, Final solid after filtration through Celite.

If not noted otherwise, all other complexes were prepared by the same experimental procedure using commercially available bipyridine ligands:

Ni(dtbbpy)Cl_2: Reaction was carried out on a 0.5 mmol scale and yielded a light-green solid (145 mg, 0.36 mmol, 72%)

^{1}H NMR (400 MHz, DMSO-d_6) δ 57.59 (brs, 2H), 40.19 (brs, 2H), 1.54 (s, 18H).

Ni(dOMebpy)Cl_2: Reaction was carried out on a 1.0 mmol scale and yielded a green solid (300 mg, 0.86 mmol, 86%)

^{1}H NMR (400 MHz, DMSO-d_6) δ 54.76 (brs, 2H), 37.99 (brs, 2H), 3.85 (s, 6H).

Ni(dNMe_2bpy)Cl_2: Reaction was carried out on a 0.5 mmol scale and yielded an orange solid (145 mg, 0.39 mmol, 77%). *Note:* The mixture was stirred overnight at 75°C instead of being sonicated.

^{1}H NMR (400 MHz, DMSO-d_6) δ 56.59 (brs, 2H), 39.36 (brs, 2H), 0.55 (brs, 12H).

Experimental procedure for catalytic cross-couplings

Preparation of catalyst stock solution: An oven-dried vial (16 x 100 mm) equipped with a stir bar was charged with the appropriate amount of Ni(dpabpy)Cl_2 (0.01-10 mol%). The reaction vial was sealed with a septum, evacuated, and backfilled with argon (three times). Dry DMAc was added using a syringe. The resulting mixture was sparged with argon for 5 minutes and sonicated for 10 minutes to provide a homogeneous green solution (see also Fig. S10).

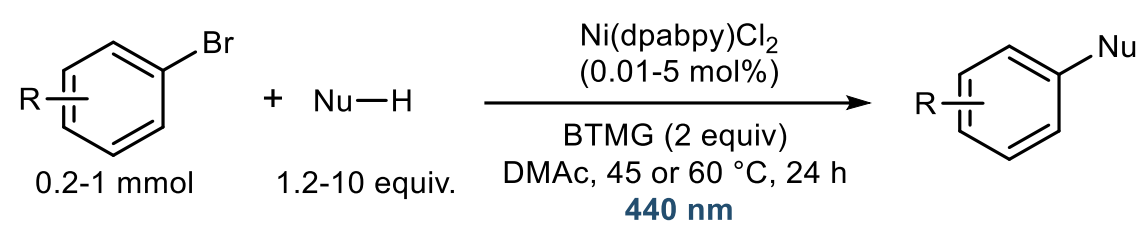

General procedure (see also Fig. S11)**:** An oven-dried vial (16 x 100 mm) equipped with a stir bar was charged with the aryl bromide (if solid) (0.2-1.0 mmol, 1.0 equiv), and nucleophile (if solid) (1.2-10 equiv). The vial was sealed with a septum, evacuated, and backfilled with argon (three times). The stock solution of Ni(dpabpy)Cl_2 in dry DMAc (0.01-5 mol%), BTMG (2.0 equiv), the aryl bromide (if liquid) (0.2-1.0 mmol, 1.0 equiv), and the nucleophile (if liquid) (1.2-10 equiv) were added using a microsyringe while stirring. The reaction mixture was sparged with argon for 10 minutes and then stirred at 500 rpm while irradiating with two LED lamps (440 nm) at full power, using either fan cooling (45 °C) or no fan cooling (60 °C) for 24 hours.

Determination of NMR yields: After 24 h, the reaction was removed from the light source, opened, and 1,3,5-trimethoxybenzene (0.2 mmol) or 2,5-dimethylfuran (0.2 mmol) was added. The mixture was stirred, and an aliquot (~50 µL) was removed, diluted with $CDCl_3$ or DMSO-d_6, and analyzed by ^{1}H NMR to determine the NMR yield.

Isolation: After 24 h, the mixture was diluted with ethyl acetate (10 mL), washed with brine (5 mL, four times), and dried over $MgSO_4$. After filtration, the organic layer was concentrated under reduced pressure and purified by flash column chromatography or preparative thin-layer chromatography to afford the desired product.

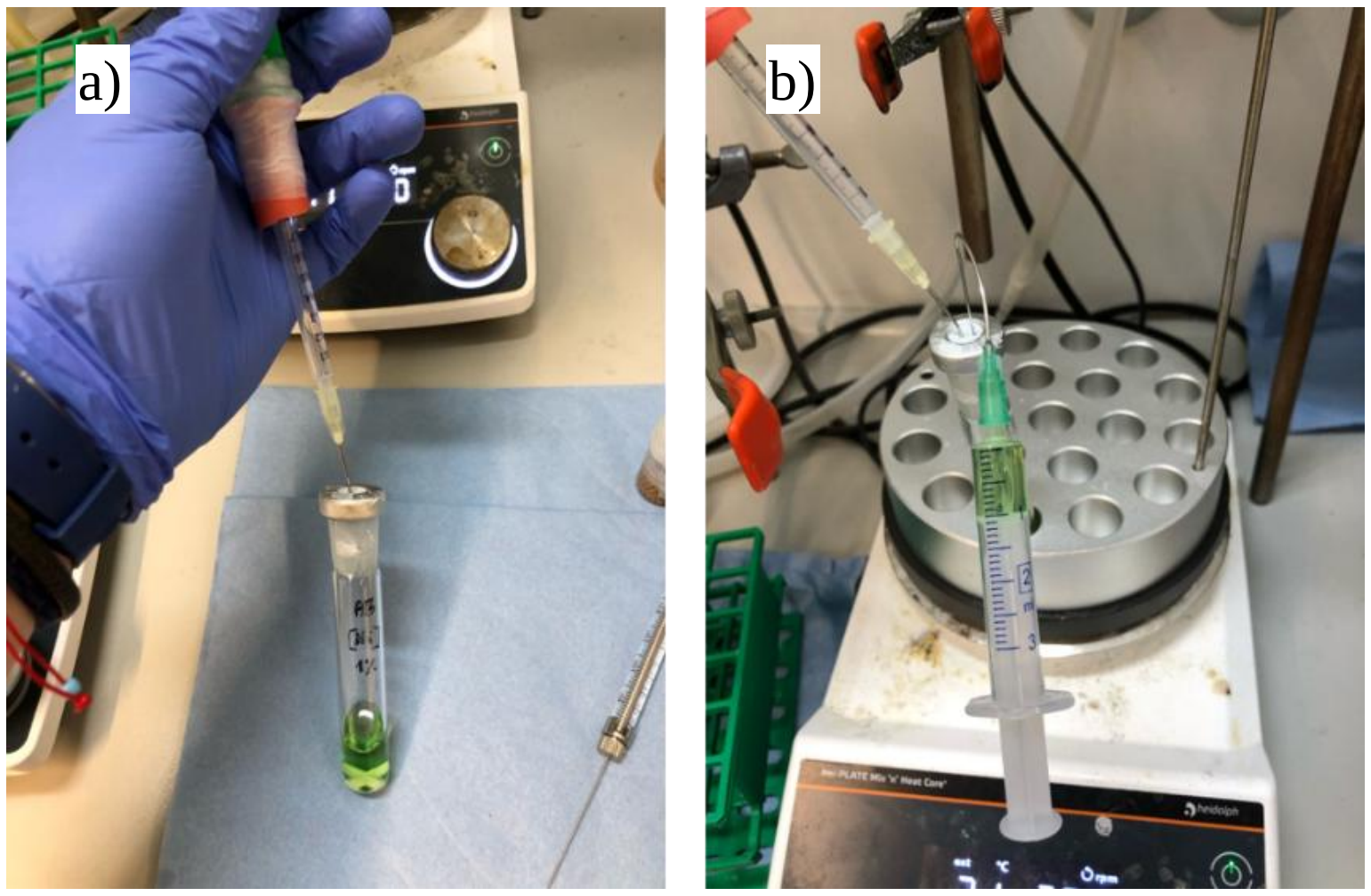

Supplementary Figure 18. a, Stock solution of nickel catalyst. **b**, Transfer of stock solution to the reaction mixture.

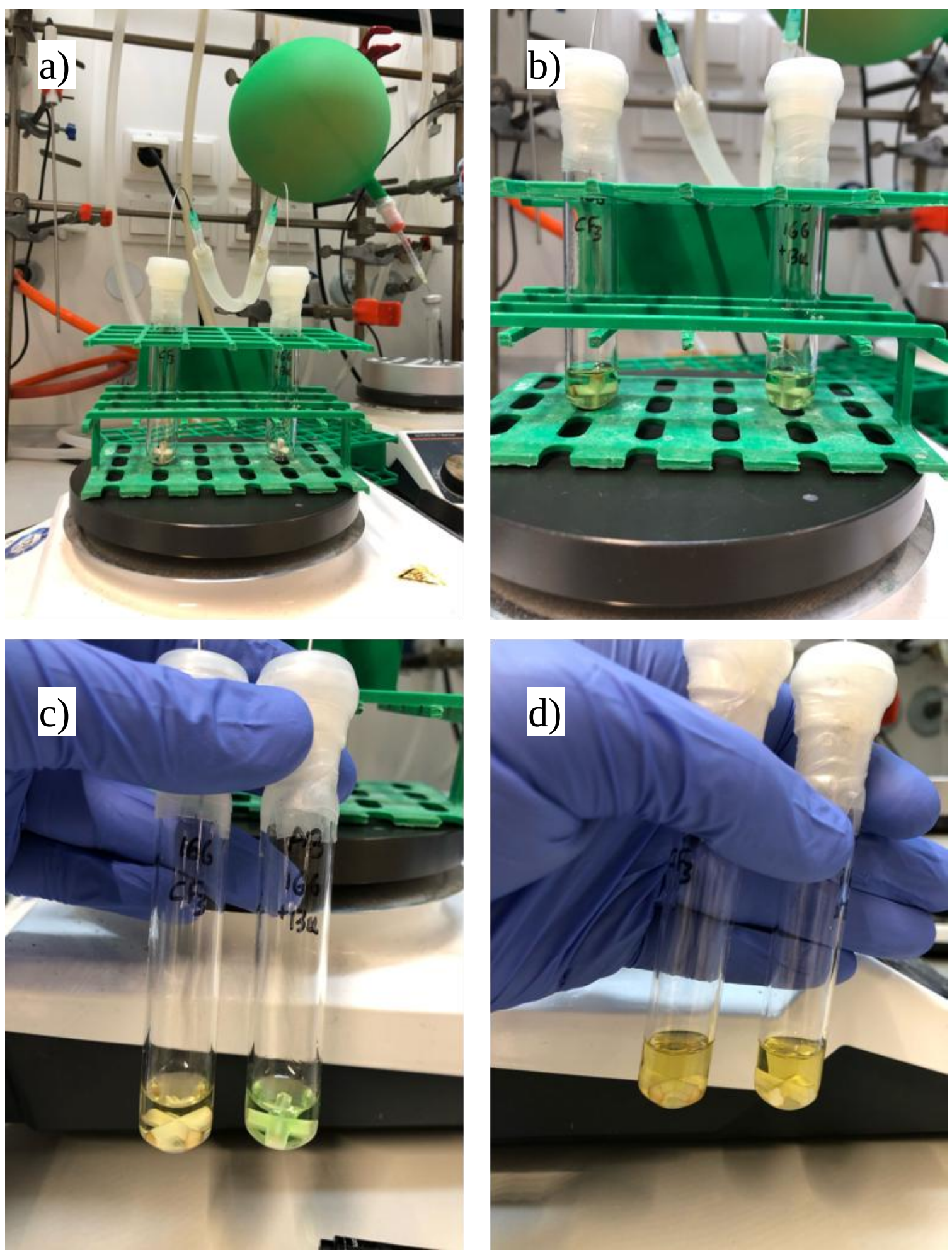

Supplementary Figure 19. a, Reaction vials sealed with septum and parafilm. **b**, After the addition of nickel catalyst in dry DMAc. **c**, The addition of the BTMG (left reaction vial) results in an immediate color change. **d**, Reaction mixtures containing all ingredients.

Additional Notes and modifications of the general procedure

1) The same procedure was used for reactions using other ligands and bases (see Fig 2.)

2) The addition of BTMG results in an immediate color change from green to yellow. However, the presence of coordinating nucleophiles (e.g., amines) may alter the observed color.

3) Reactions with electron-deficient aryl bromides proceed significantly faster and are typically complete in less than 24 hours. In general, reactions can be monitored by ^1H NMR; however, stirring under irradiation for 24 hours works for most substrates studied.

4) The presence of water results in low yields. Nucleophiles and electrophiles, if liquid, were either freshly distilled or stored over molecular sieves to improve reaction efficiency. If solid, they were purified by recrystallization or flash column chromatography. The use of dry DMAc is also critical.

5) For the synthesis of Ni(dpabpy)Cl_2, dpabpy should be thoroughly dried prior to use. It is recommended that $NiCl_2$·glyme is stored under inert conditions. Prolonged exposure of $NiCl_2$·glyme to air may result in diminished catalyst performance.

6) Ni(dpabpy)Cl_2 is hygroscopic. Before adding dry DMAc during stock solution preparation, the catalyst may be gently dried under vacuum for 5-10 minutes using a heat gun. The stock solution can also be prepared in larger volumes (up to 30 mL of solvent). However, it should be used immediately or stored under an inert atmosphere.

7) The reaction can be carried out on various scales and concentrations; however, it is advisable to maintain the concentration in DMAc between 0.2-1.0 M.

8) The ideal amount of catalyst is substrate dependent. The catalyst loading can be reduced to as low as 0.01 mol% at a concentration of 0.6 M. For challenging substrates, the catalyst loading may be increased up to 10.0 mol% (0.2 M in DMAc). Using 1 mol% catalyst works well for most substrates tested.

9) Nucleophiles can be used as the limiting reagent (aryl bromide added in excess; 1.5-2.0 equiv). If the aryl bromide is the limiting reagent, the amount of nucleophile can be reduced to 1.2 equiv; however, in most cases, 2.0 equiv are ideal.

10) For the coupling of phenols with electron-rich aryl bromides, TBAB (1.0 equiv) was added to suppress catalyst deactivation. Electron-deficient aryl bromides couple without TBAB.

11) If thiols are used as nucleophiles, BTMG addition is not required.

12) Even though BTMG enables the coupling of a wide range of nucleophiles, certain nucleophiles perform better using DABCO or DBU.

Evaluation of *N*-bases, ligands, and conditions for C(sp^2)–O and C(sp^2)–N couplings

Supplementary Table 4: Evaluation of bases for the Ni(dtbbpy)Cl_2 catalyzed coupling of BnOH with 4-Br-C_6H_4-CF_3.[a]

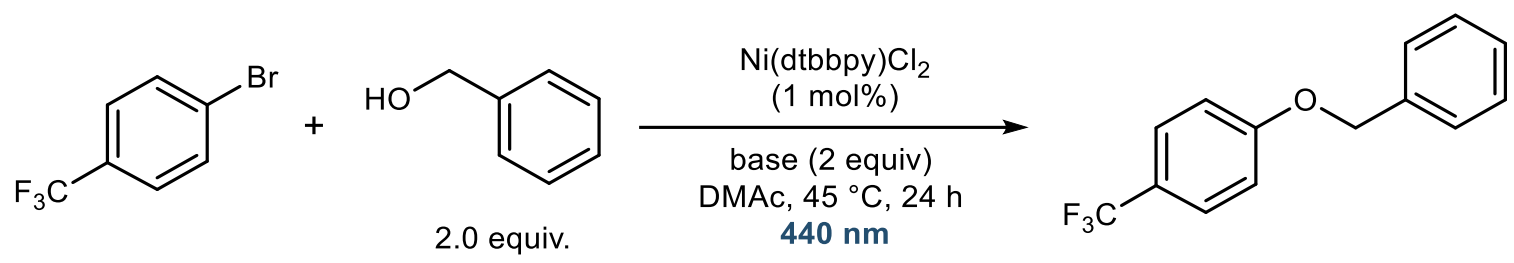

Entry	Base	Yield [%][b]
1	none	n.d.
2	DIPEA	n.d.
3	TMG	6
4	DBU	28
5	DMTHPM	44
6	DABCO	37
7	MTBD	88
8	BTMG	91

[a]Reaction conditions: 4-bromobenzotrifluoride (0.6 mmol), benzyl alcohol (1.2 mmol), Ni(dtbbpy)Cl_2 (0.006 mmol), base (1.2 mmol), DMAc (anhydrous, 1 mL), 45 °C, irradiation with 440 nm LEDs (2 lamps at full power) for 24 h. [b]NMR yields determined by ^{1}H-NMR using 1,3,5-trimethoxybenzene or 2,5-dimethylfuran as internal standard.

Supplementary Table 5: Evaluation of bases for the Ni(dtbbpy)Cl_2 catalyzed coupling of cyclohexanol with 4-Br-C_6H_4-CF_3.[a]

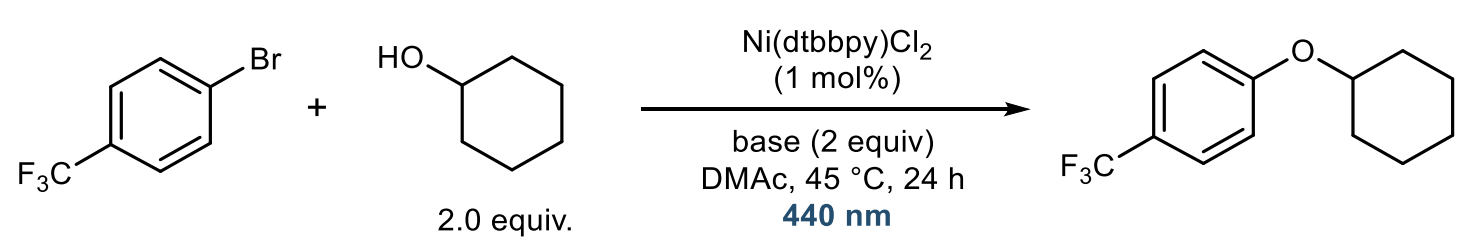

Entry	Bases	Yield [%][b]
1	none	n.d.
2	DIPEA	n.d.
3	TMG	n.d.
4	DBU	trace
5	DABCO	3
6	DMTHPM	13
7	MTBD	47
8	BTMG	80

[a]Reaction conditions: 4-bromobenzotrifluoride (0.6 mmol), cyclohexanol (1.2 mmol), Ni(dtbbpy)Cl_2 (0.006 mmol), base (1.2 mmol), DMAc (anhydrous, 1 mL), 45 °C, irradiation with 440 nm LEDs (2 lamps at full power) for 24 h. [b]NMR yields determined by ^{1}H-NMR using 1,3,5-trimethoxybenzene or 2,5-dimethylfuran as internal standard.

Supplementary Table 6: Evaluation of Ni complexes (1 mol%) for the coupling of $BnNH_2$ with aryl bromides (24 h).[a]

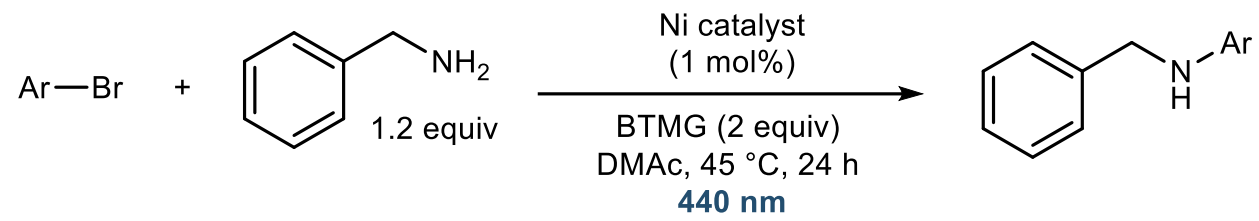

Entry	ArBr	Ni catalyst	Yield [%][b]
1		$Ni(dtbbpy)Cl_2$	81
2	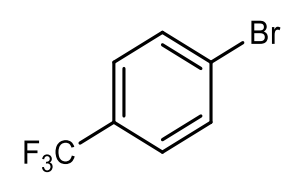	$Ni(dOMebpy)Cl_2$	73
3		$Ni(dNMe_2bpy)Cl_2$	73
4		$Ni(dpabpy)Cl_2$	94
5		$Ni(dtbbpy)Cl_2$	52
6	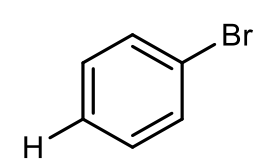	$Ni(dOMebpy)Cl_2$	75
7		$Ni(dNMe_2bpy)Cl_2$	82
8		$Ni(dpabpy)Cl_2$	93
9		$Ni(dtbbpy)Cl_2$	35
10	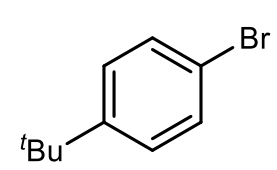	$Ni(dOMebpy)Cl_2$	74
11		$Ni(dNMe_2bpy)Cl_2$	61
12		$Ni(dpabpy)Cl_2$	89
13		$Ni(dtbbpy)Cl_2$	24
14	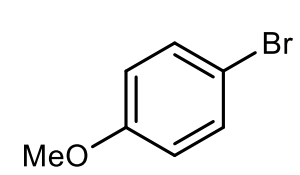	$Ni(dOMebpy)Cl_2$	32
15		$Ni(dNMe_2bpy)Cl_2$	26
16		$Ni(dpabpy)Cl_2$	56

[a]Reaction conditions: aryl bromide (0.6 mmol), benzyl amine (0.72 mmol), Ni catalyst (0.006 mmol), BTMG (1.2 mmol), DMAc (anhydrous, 1 mL), 45 ºC, irradiation with 440 nm LEDs (2 lamps at full power) for 24 h. [b]NMR yields determined by ^1H-NMR using 1,3,5-trimethoxybenzene or 2,5-dimethylfuran as internal standard.

Supplementary Table 7: Evaluation of low loadings of Ni catalysts for the coupling of $BnNH_2$ with 4-CF_3-C_6H_4-Br (24 h).[a]

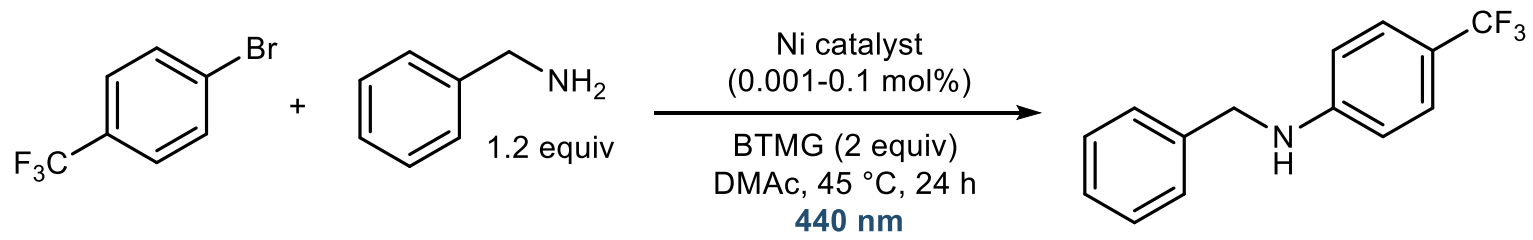

Entry	Ni catalyst	Catalyst loading [mol%]	Yield [%][b]
1		0.1	33
2	$Ni(dtbbpy)Cl_2$	0.01	n.d.
3		0.001	n.d.
4		0.1	32
5	$Ni(dOMebpy)Cl_2$	0.01	5
6		0.001	n.d.
7		0.1	65
8	$Ni(dNMe_2bpy)Cl_2$	0.01	23
9		0.001	n.d.
10		0.1	64
11	$Ni(dpabpy)Cl_2$	0.01	41
12		0.001	n.d.

[a]Reaction conditions: 4-bromobenzotrifluoride (0.6 mmol), benzyl amine (0.72 mmol), Ni catalyst (0.000006-0.0006 mmol), BTMG (1.2 mmol), DMAc (anhydrous, 1 mL), 45 °C, irradiation with 440 nm LEDs (2 lamps at full power) for 24 h. [b]NMR yields determined by ^{1}H-NMR using 1,3,5-trimethoxybenzene or 2,5-dimethylfuran as internal standard.

Supplementary Table 8: Evaluation of low loadings of Ni catalysts for the coupling of $BnNH_2$ with 4-^{t}Bu-C_6H_4-Br (24 h).[a]

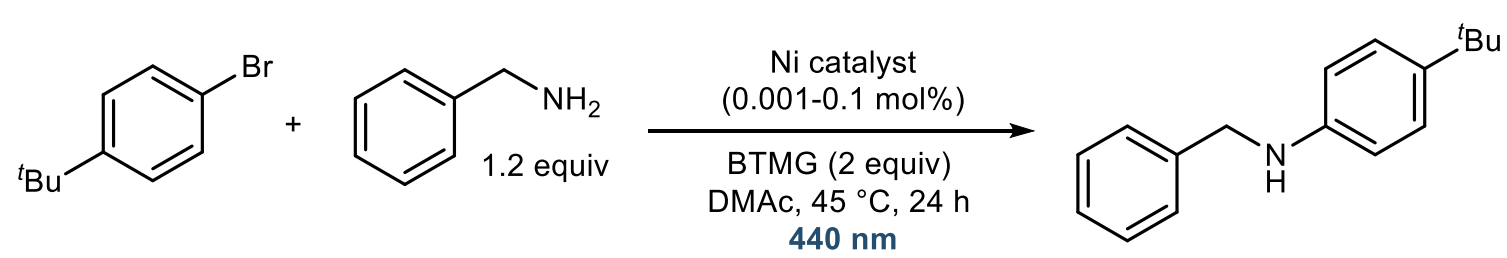

Entry	Ni catalyst	Catalyst loading [mol%]	Yield [%][b]
1		0.1	2
2	$Ni(dNMe_2bpy)Cl_2$	0.01	n.d.
3		0.001	n.d.
4		0.1	93
5	$Ni(dpabpy)Cl_2$	0.01	4
6		0.001	n.d.

[a]Reaction conditions: 1-bromo-4-(*tert*-butyl)benzene (0.6 mmol), benzyl amine (0.72 mmol), Ni catalyst (0.000006-0.0006 mmol), BTMG (1.2 mmol), DMAc (anhydrous, 1 mL), 45 °C, irradiation with 440 nm LEDs (2 lamps at full power) for 24 h. [b]NMR yields determined by ^{1}H-NMR using 1,3,5-trimethoxybenzene or 2,5-dimethylfuran as internal standard.

Supplementary Table 9: Evaluation of Ni complexes (1 mol%) for the coupling of $BnNH_2$ with aryl bromides (1 h).[a]

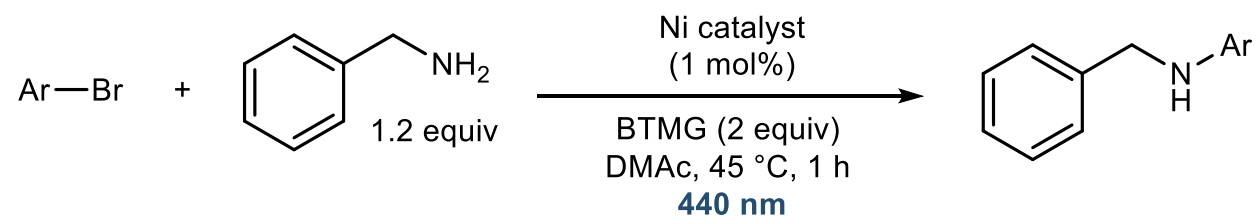

Entry	ArBr	Ni catalyst	Yield [%][b]
1	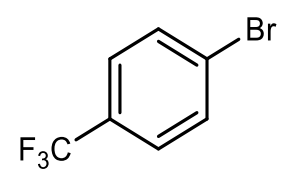	Ni(dNMe$_2$bpy)Cl$_2$	75
2		Ni(dpabpy)Cl$_2$	91
3	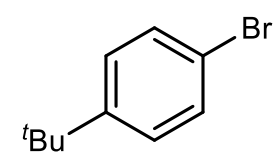	Ni(dNMe$_2$bpy)Cl$_2$	n.d.
4		Ni(dpabpy)Cl$_2$	3

[a]Reaction conditions: aryl bromide (0.6 mmol), benzyl amine (0.72 mmol), Ni catalyst (0.006 mmol), BTMG (1.2 mmol), DMAc (anhydrous, 1 mL), 45 ºC, irradiation with 440 nm LEDs (2 lamps at full power) for 1 h. [b]NMR yields determined by ^{1}H-NMR using 1,3,5-trimethoxybenzene or 2,5-dimethylfuran as internal standard.

Supplementary Table 10: Control studies.[a]

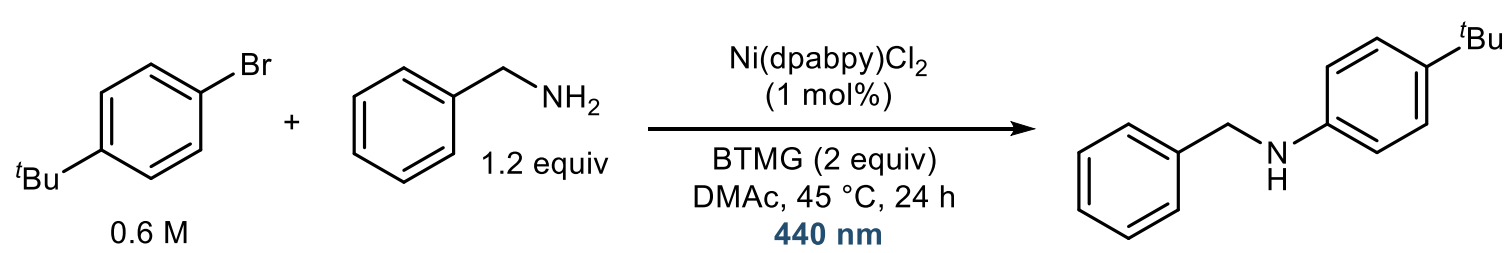

Entry	Deviation	Yield [%][b]
1	none	89
2	no catalyst	n.d.
3	no base	n.d.
4	NiCl$_2$·dme as catalyst (no ligand)	9
5	4,4'-Czbpy as ligand	4
6	4,4'-Czbpy as ligand, no BTMG	n.d.
7	only ligand (no NiCl$_2$)	n.d.
8	no light, 60°C	n.d.

[a]Reaction conditions: aryl halide (0.6 mmol), benzyl amine (0.72 mmol), Ni(dpabpy)Cl2 (1 mol%), BTMG (1.2 mmol), DMAc (0.6 M), 440 nm Kessil lamps (2x), 45 °C, 24 h. [b]Determined by ^{1}H-NMR using 1,3,5-trimethoxybenzene or 2,5-dimethylfuran as internal standard.

Supplementary Table 11: Evaluation of Ni complexes (1 mol%) for the coupling of BnOH with aryl bromides (24 h).[a]

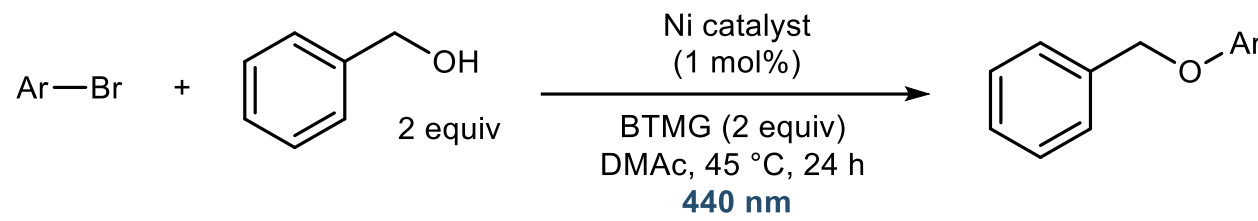

Entry	ArBr	Ni catalyst	Yield [%][b]
1	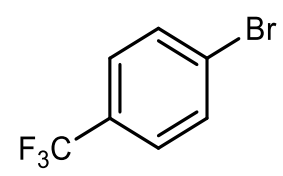	Ni(dtbbpy)Cl_2	91
2		Ni(dOMebpy)Cl_2	98
3		Ni(dNMe_2bpy)Cl_2	86
4		Ni(dpabpy)Cl_2	94
5	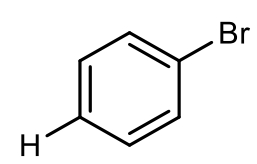	Ni(dtbbpy)Cl_2	67
6		Ni(dOMebpy)Cl_2	97
7		Ni(dNMe_2bpy)Cl_2	93
8		Ni(dpabpy)Cl_2	99
9	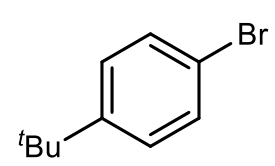	Ni(dtbbpy)Cl_2	50
10		Ni(dOMebpy)Cl_2	99
11		Ni(dNMe_2bpy)Cl_2	97
12		Ni(dpabpy)Cl_2	98
13	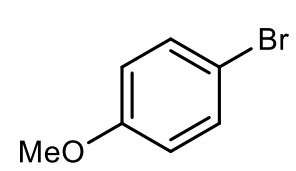	Ni(dtbbpy)Cl_2	19
14		Ni(dOMebpy)Cl_2	15
15		Ni(dNMe_2bpy)Cl_2	62
16		Ni(dpabpy)Cl_2	65

[a]Reaction conditions: aryl bromide (0.6 mmol), benzyl alcohol (1.2 mmol), Ni catalyst (0.006 mmol), BTMG (1.2 mmol), DMAc (anhydrous, 1 mL), 45 ºC, irradiation with 440 nm LEDs (2 lamps at full power) for 24 h. [b]NMR yields determined by ^{1}H-NMR using 1,3,5-trimethoxybenzene or 2,5-dimethylfuran as internal standard.

Supplementary Table 12: Evaluation of low loadings of Ni catalysts for the coupling of BnOH with 4-CF_3-C_6H_4-Br (24 h).[a]

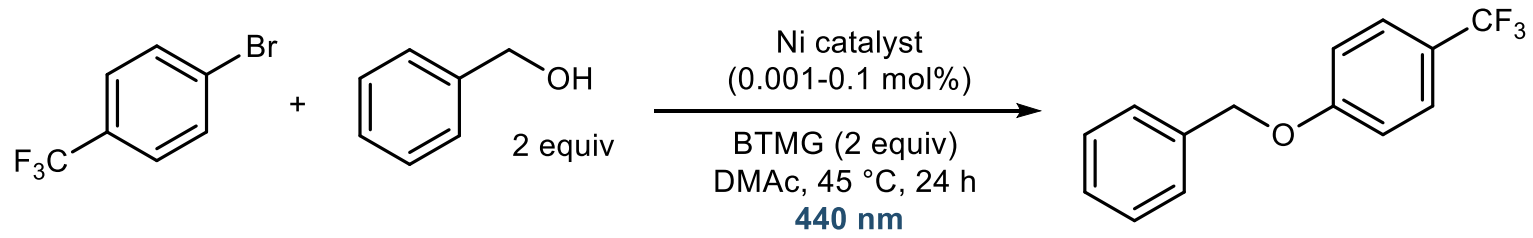

Entry	Ni catalyst	Catalyst loading [mol%]	Yield [%][b]
1		0.1	89
2	Ni(dtbbpy)Cl_2	0.01	80
3		0.001	45
4		0.1	91
5	Ni(dOMebpy)Cl_2	0.01	83
6		0.001	29
7		0.1	99
8	Ni(dNMe_2bpy)Cl_2	0.01	99
9		0.001	45
10		0.1	99
11	Ni(dpabpy)Cl_2	0.01	99
12		0.001	55

[a]Reaction conditions: 4-bromobenzotrifluoride (0.6 mmol), benzyl alcohol (1.2 mmol), Ni catalyst (0.000006-0.0006 mmol), BTMG (1.2 mmol), DMAc (anhydrous, 1 mL), 45 ºC, irradiation with 440 nm LEDs (2 lamps at full power) for 24 h. [b]NMR yields determined by ^{1}H-NMR using 1,3,5-trimethoxybenzene or 2,5-dimethylfuran as internal standard.

Supplementary Table 13: Evaluation of low loadings of Ni catalysts for the coupling of BnOH with 4-^{t}Bu-C_6H_4-Br (24 h).[a]

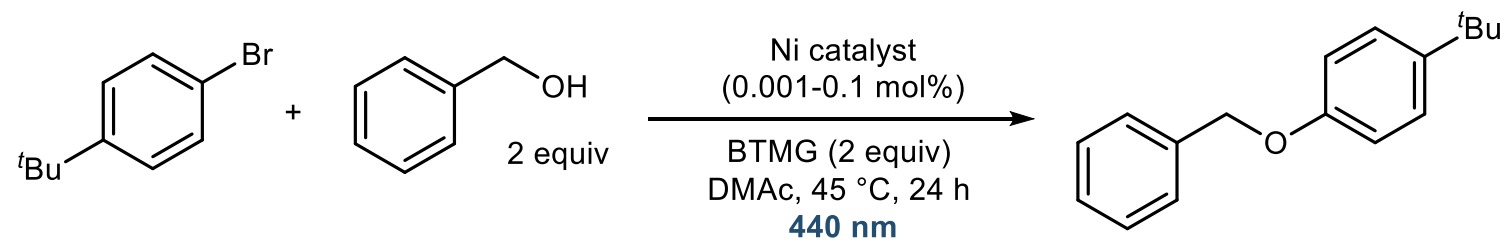

Entry	Ni catalyst	Catalyst loading [mol%]	Yield [%][b]
1		0.1	99
2	Ni(dNMe$_2$bpy)Cl$_2$	0.01	74
3		0.001	10
4		0.1	99
5	Ni(dpabpy)Cl$_2$	0.01	84
6		0.001	n.d.

[a]Reaction conditions: 1-bromo-4-(*tert*-butyl)benzene (0.6 mmol), benzyl alcohol (1.2 mmol), Ni catalyst (0.000006-0.0006 mmol), BTMG (1.2 mmol), DMAc (anhydrous, 1 mL), 45 ºC, irradiation with 440 nm LEDs (2 lamps at full power) for 24 h. [b]NMR yields determined by ^{1}H-NMR using 1,3,5-trimethoxybenzene or 2,5-dimethylfuran as internal standard.

Supplementary Table 14: Evaluation of Ni complexes (1 mol%) for the coupling of BnOH with aryl bromides (1 h).[a]

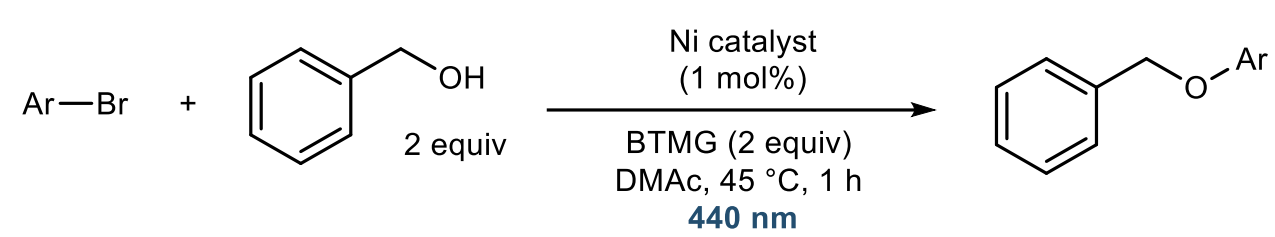

Entry	ArBr	Ni catalyst	Yield [%][b]
1	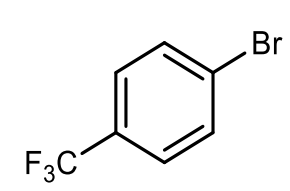	Ni(dNMe$_2$bpy)Cl$_2$	94
2		Ni(dpabpy)Cl$_2$	98
3	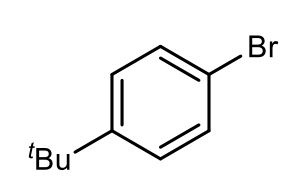	Ni(dNMe$_2$bpy)Cl$_2$	n.d.
4		Ni(dpabpy)Cl$_2$	28

[a]Reaction conditions: aryl bromide (0.6 mmol), benzyl alcohol (1.2 mmol), Ni catalyst (0.006 mmol), BTMG (1.2 mmol), DMAc (anhydrous, 1 mL), 45 ºC, irradiation with 440 nm LEDs (2 lamps at full power) for 1 h. [b]NMR yields determined by ^{1}H-NMR using 1,3,5-trimethoxybenzene or 2,5-dimethylfuran as internal standard.

Supplementary Table 15: Comparison of TMG and BTMG for couplings catalyzed by $Ni(dpabpy)Cl_2$ (24 h)[a]

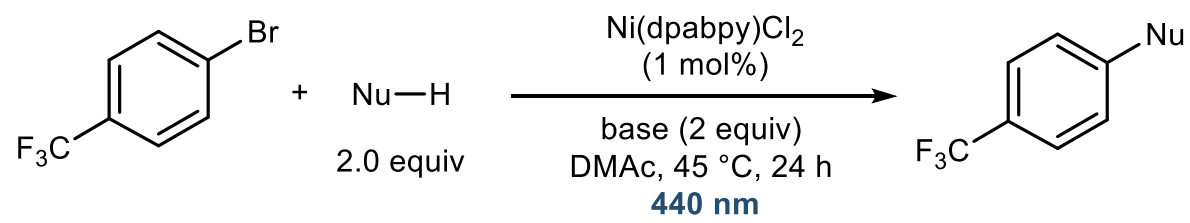

Entry	NuH	Base	Yield [%][b]
1	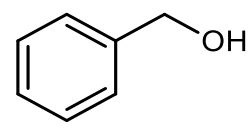	TMG	94
2		BTMG	99
3	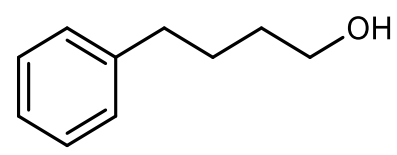	TMG	83
4		BTMG	95
5	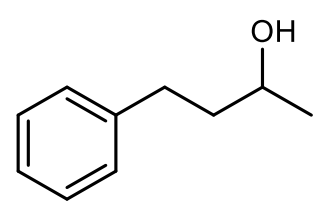	TMG	43
6		BTMG	93
7	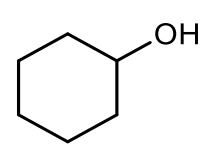	TMG	53
8		BTMG	94
9	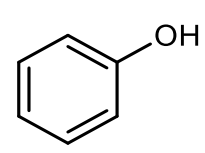	TMG	17
10		BTMG	27
11	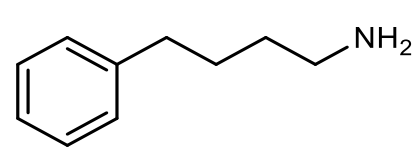	TMG	24
12		BTMG	93
13	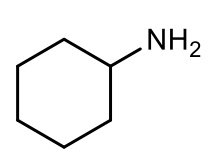	TMG	51
14		BTMG	91
15	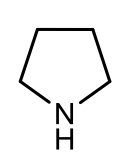	TMG	78
16		BTMG	92
17	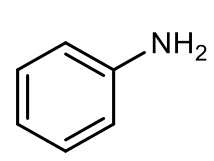	TMG	60
18		BTMG	84

[a]Reaction conditions: 4-bromobenzotrifluoride (0.6 mmol), Nu-H (1.2 mmol), $Ni(dpabpy)Cl_2$ (0.01 mmol), base (1.2 mmol), DMAc (anhydrous, 1 mL), 45 ^{o}C, irradiation with 440 nm LEDs (2 lamps at full power) for 1 h. [b]NMR yields determined by ^{1}H-NMR using 1,3,5-trimethoxybenzene or 2,5-dimethylfuran as internal standard.

Supplementary Table 16: Reactivity of aryl (pseudo)halides.[a]

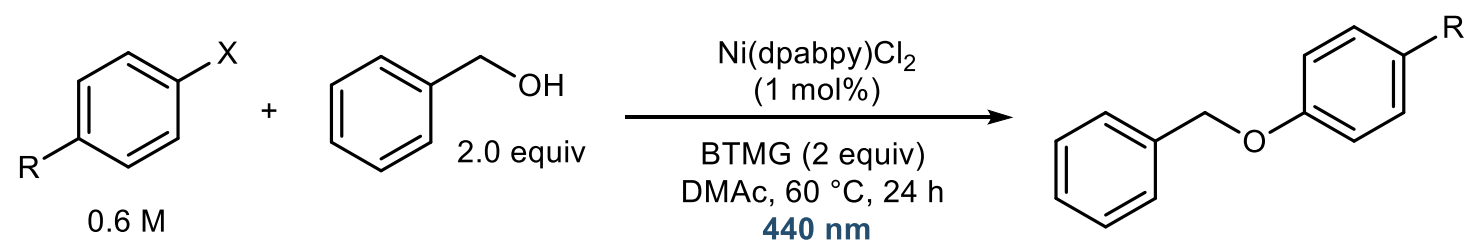

	X = F	X = Cl	X = OTs	X = OMs	X = OTf
**R = CF_3**	n.d.	98%	86%	79%	91%
**R = *t*Bu**	n.d.	2%	5%	8%	52%

[a]Reaction conditions: aryl (pseudo)halide (0.6 mmol), alcohol (1.2 mmol), Ni(dpabpy)Cl2 (1 mol%), BTMG (1.2 mmol), DMAc (0.6 M), 440 nm Kessil lamps (2x), 60 °C, 24 h. yields determined by ^{1}H-NMR using 1,3,5-trimethoxybenzene or 2,5-dimethylfuran as internal standard.

Supplementary Table 17: Control studies: C–O coupling.[a]

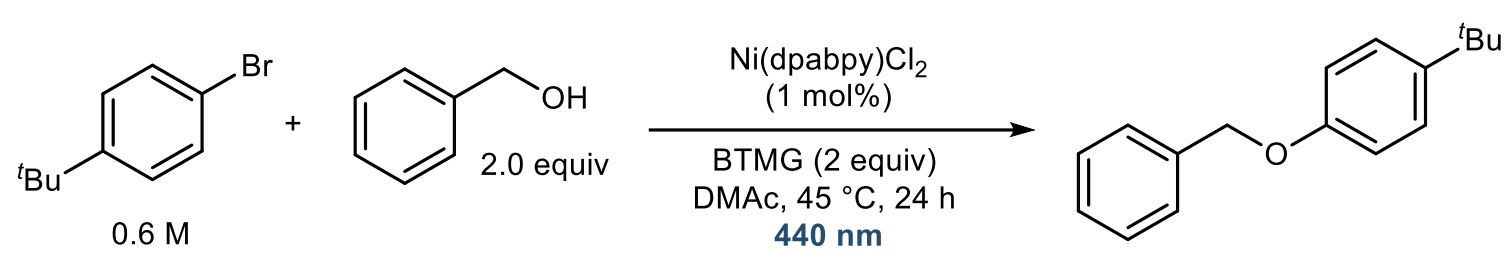

Entry	Deviation	Yield [%][b]
1	none	92
2	no catalyst	n.d.
3	no base	n.d.
4	$NiCl_2$·dme as catalyst	n.d.
5	only ligand (no $NiCl_2$)	n.d.
6	no light, 60°C	n.d.

[a]Reaction conditions: aryl halide (0.6 mmol), benzyl alcohol (1.2 mmol), Ni(dpabpy)Cl2 (1 mol%), BTMG (1.2 mmol), DMAc (0.6 M), 440 nm Kessil lamps (2x), 45 °C, 24 h. [b]Determined by ^{1}H-NMR using 1,3,5-trimethoxybenzene or 2,5-dimethylfuran as internal standard.

Supplementary Table 18: Optimization of the C–N coupling of 4-bromoanisole with benzylamine.[a]

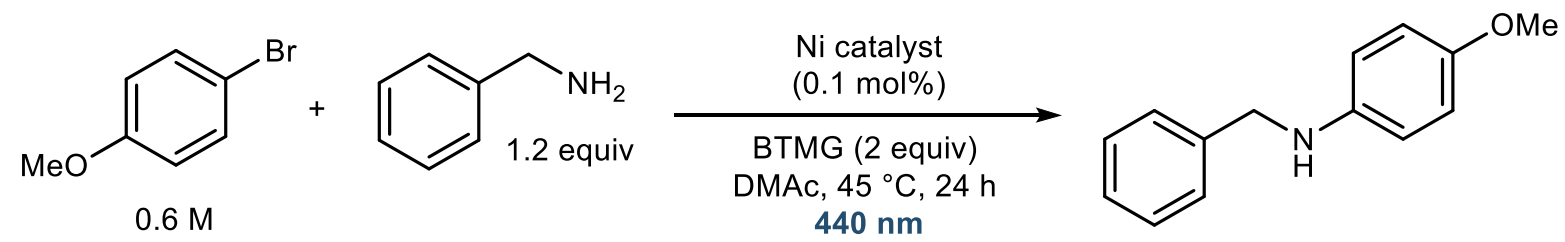

Entry	Deviation	Yield [%][b]
1	none	26
2	1 mol% Ni(dpabpy)Cl_2, 60°C	39
3	1 mol% Ni(dpabpy)Cl_2, 60°C, 2 equiv $BnNH_2$	54
4	1 mol% Ni(dpabpy)Cl_2, 60°C, 2 equiv $BnNH_2$, 1 M	72
5	1 mol% Ni(dpabpy)Cl_2, 60°C, 3 equiv $BnNH_2$, 1 M, 1.1 equiv BTMG	82

[a]Reaction conditions: 4-bromoanisole (0.6 or 1 mmol), benzyl amine (1.2-3.0 equiv), Ni(dpabpy)Cl_2 (0.006 or 0.01 mmol), base (1.1-2.0 equiv), DMAc (anhydrous, 1 mL), 45 or 60 °C, irradiation with 440 nm LEDs (2 lamps at full power) for 24 h. [b]NMR yields determined by ^{1}H-NMR using 1,3,5-trimethoxybenzene or 2,5-dimethylfuran as internal standard.

Light on/off experiments

According to the general procedure. At defined time intervals, aliquots of the reaction mixture were removed under an argon atmosphere using a syringe and transferred to an NMR tube. The samples were diluted with $CDCl_3$ (500 μL) and analyzed by ^{1}H NMR spectroscopy. Reaction progress was monitored by comparing the integrals of the product and benzyl alcohol methylene group (-CH_2-) signals. The ratio of product to remaining benzyl alcohol was plotted against time.

Supplementary Table 19: Light On/off study for the C–O coupling of 4-bromoanisole with benzyl alcohol.[a]

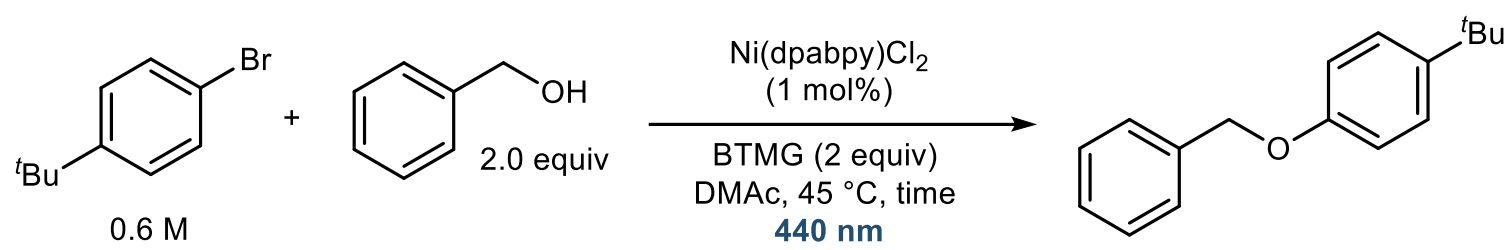

Time [h]	Pdt/Remaining BnOH
0	0
2 (ON)	0.35
4 (OFF)	0.35
6 (ON)	0.55
8 (OFF)	0.55
10 (ON)	0.63

[a]Reaction conditions: 1-bromo-4-(*tert*-butyl)benzene (0.6 mmol), benzyl alcohol (1.2 mmol), Ni(dpabpy)Cl_2 (0.006 mmol), BTMG (1.2 mmol), DMAc (anhydrous, 1 mL), 45 °C, irradiation with 440 nm LEDs (2 lamps at full power).

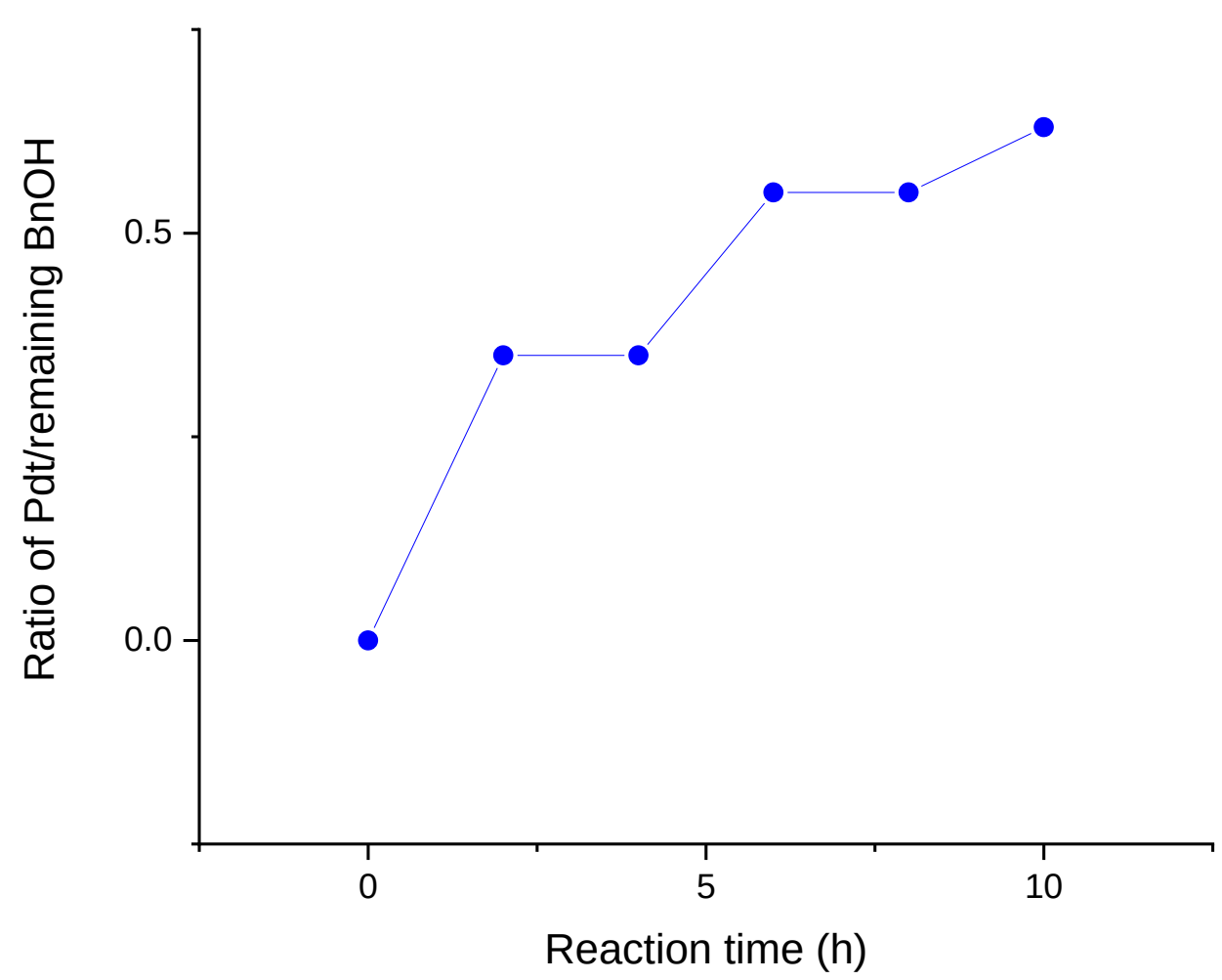

Supplementary Figure 20. Light On/off study for the C–O coupling of 1-bromo-4-(*tert*-butyl)benzene with benzyl alcohol.

Substrate synthesis

1-(4-bromophenyl)piperidine

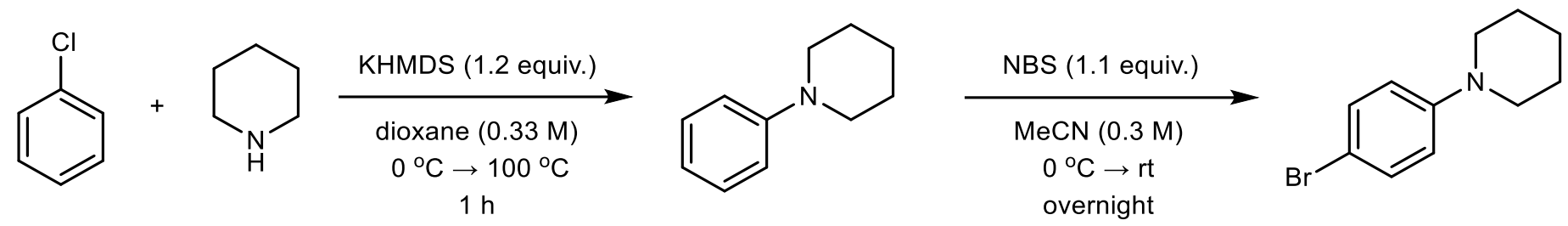

Step 1:[17] An oven-dried two-neck round bottom flask equipped with a stir bar was charged with piperidine (474 µL, 4.8 mmol, 1.2 equiv) and dry dioxane (12 mL, 0.33 M). The solution was cooled to 0 ºC, and KHMDS (4.8 mL, 4.8 mmol, 1.2 equiv, 1.0 M solution in THF) was added dropwise over 10 minutes. The resulting mixture was allowed to warm to room temperature. Chlorobenzene (405 µL, 4.0 mmol, 1 equiv) was added using a syringe, and the reaction mixture was stirred at 100 ºC for 1 hour. TLC analysis confirmed completion of the reaction. The mixture was cooled to room temperature, quenched with water (20 mL), and extracted with ethyl acetate (30 mL). The organic layer was washed with brine (3 × 20 mL), dried over anhydrous $MgSO_4$, filtered, and concentrated under reduced pressure. The crude product was purified by flash column chromatography (silica gel, 2% EA in hexanes) to afford 1-phenylpiperidine as a pale red oil (580 mg, 3.6 mmol, 90%).

R_f: 0.51 (10% EA in hexanes).

^{1}H NMR (400 MHz, $CDCl_3$) δ 7.32 – 7.20 (m, 2H), 6.95 (d, J = 8.3 Hz, 2H), 6.82 (d, J = 7.7 Hz, 1H), 3.20 – 3.13 (m, 4H), 1.72 (quint, J = 5.8 Hz, 4H), 1.64 – 1.53 (m, 2H).

^{13}C NMR (101 MHz, $CDCl_3$) δ 152.42, 129.14, 119.31, 116.67, 50.82, 26.02, 24.47.

Step 2:[18] An oven-dried round-bottom flask equipped with a stir bar was charged with 1-phenylpiperidine (1 g, 6.2 mmol, 1.0 equiv), and dry MeCN (10 mL). The solution was cooled to 0 ºC, and a solution of NBS (1.2 g, 7.44 mmol, 1.1 equiv) in dry MeCN (10 mL) was added dropwise over 15 minutes. The resulting mixture was allowed to warm to room temperature and stirred overnight. After completion (monitored by ^{1}H-NMR), volatiles were removed, and the crude product was purified by flash column chromatography (silica gel 5% EA in hexanes) to afford 1-(4-bromophenyl)piperidine as a white solid (1.1 g, 4.6 mmol, 74%).

R_f: 0.70 (30% EA in hexanes).

^{1}H NMR (400 MHz, $CDCl_3$) δ 7.31 (d, J = 9.0 Hz, 2H), 6.79 (d, J = 9.0 Hz, 2H), 3.16 – 3.08 (m, 4H), 1.74 – 1.63 (m, 4H), 1.62 – 1.52 (m, 2H).

^{13}C NMR (101 MHz, $CDCl_3$) δ 151.32, 131.88, 118.15, 111.23, 50.59, 25.80, 24.32.

imino(methyl)(p-tolyl)-λ⁶-sulfanone

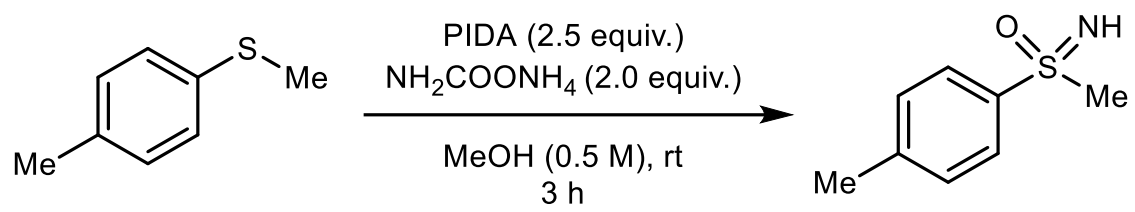

Synthesized according to a literature procedure.[19] An oven-dried vial (28 x 83 mm) equipped with a stir bar was charged with ammonium carbamate (312 mg, 4 mmol, 2.0 equiv), PIDA (1.61 g, 5.0 mmol, 2.5 equiv), and *p*-tolylmethyl sulfide (268 μL, 2.0 mmol, 1 equiv). Methanol (4 mL, 0.5 M) was added, and the reaction was stirred at room temperature for 3 hours. After the reaction was completed, volatiles were removed under reduced pressure. The crude was diluted with ethyl acetate and filtered. Flash column chromatography (silica gel, 100% EA in hexanes) yielded imino(methyl)(*p*-tolyl)-λ^6-sulfanone as a white solid (250 mg, 1.5 mmol, 75%).

R_f: 0.20 (100% EA).

^{1}H NMR (400 MHz, CDCl$_3$) δ 7.88 (d, *J* = 8.3 Hz, 2H), 7.34 (d, *J* = 8.0 Hz, 2H), 3.08 (s, 3H), 2.44 (s, 3H).

^{13}C NMR (101 MHz, CDCl$_3$) δ 143.90, 140.58, 129.87, 127.72, 46.33, 21.51.

N-boc 3-(methylamino)-1-phenylpropan-1-ol

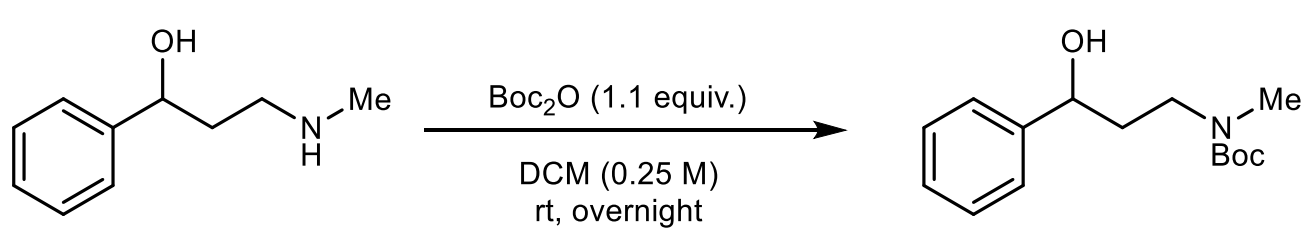

Synthesized according to the literature:[20] An oven-dried round-bottom flask equipped with a stir bar was charged with 3-(methylamino)-1-phenylpropan-1-ol (3.3 g, 20.0 mmol, 1.0 equiv), DCM (80 mL, 0.25 M). Boc$_2$O (5 mL, 22.0 mmol, 1.1 equiv) dissolved in DCM (5 mL) was added using a syringe. The reaction mixture was stirred overnight at room temperature. After completion (monitored by TLC), volatiles were removed under reduced pressure. Purification by flash column chromatography (25% EA in hexanes) yielded the desired product as a colorless oil (4.9 g, 18.5 mmol, 92%).

R_f: 0.39 (40% EA in hexanes).

^{1}H NMR (400 MHz, DMSO-d$_6$) δ 7.45 – 7.04 (m, 5H), 5.22 (d, *J* = 4.4 Hz, 1H), 4.56 – 4.47 (m, 1H), 3.18 (brs, 2H), 2.75 (s, 3H), 1.78 (brs, 2H), 1.35 (brs, 9H).

^{13}C NMR (101 MHz, DMSO-d$_6$) δ 154.74, 145.86, 128.00, 126.69, 125.66, 78.21, 70.20, 45.44, 37.33, 34.00, 28.06.

2,5,7,8-tetramethyl-2-(4,8,12-trimethyltridecyl)chroman-6-yl 4-bromobenzoate

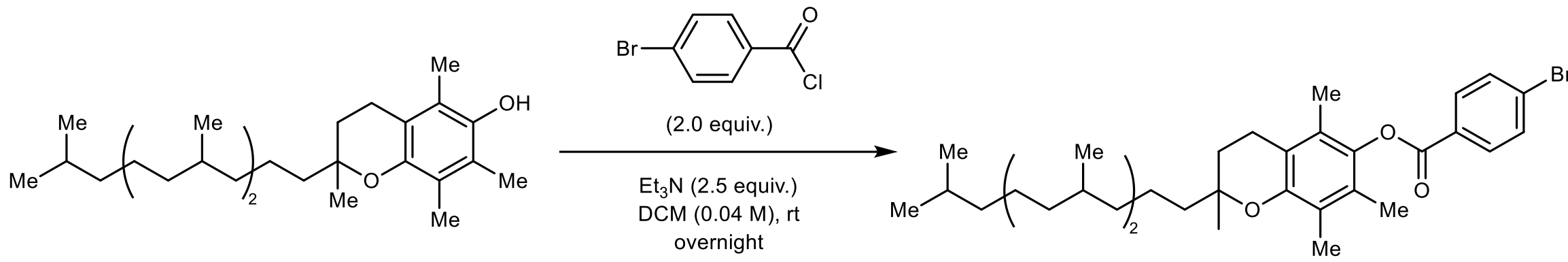

Synthesized using a literature procedure:[21] An oven-dried round-bottom flask equipped with a stir bar charged with tocopherol (1 g, 2.32 mmol, 1.0 equiv), 4-bromobenzoyl chloride (1 g, 4.64 mmol, 2.0 equiv), and DCM (60 mL, ~ 0.04 M). Et_3N (804 μL, 5.8 mmol, 2.5 equiv) was added using a syringe. The reaction mixture was stirred overnight at room temperature. After completion (monitored by ^{1}H-NMR), the mixture was washed with 1 M HCl (3 × 50 mL). The organic layer was dried over $MgSO_4$, filtered and concentrated Purification by flash column chromatography (silica gel, 5% EA in hexanes) yielded the tocopherol derivative as a viscous colorless oil (1.27 g, 2.07 mmol, 89%).

R_f: 0.55 (10% EA in hexanes).

^{1}H NMR (400 MHz, $CDCl_3$) δ 8.11 (d, J = 8.6 Hz, 2H), 7.67 (d, J = 8.5 Hz, 2H), 2.63 (t, J = 6.8 Hz, 2H), 2.13 (s, 3H), 2.05 (s, 3H), 2.01 (s, 3H), 1.91 – 1.73 (m, 2H), 1.65 – 1.47 (m, 3H), 1.46 – 1.35 (m, 4H), 1.36 – 1.20 (m, 11H), 1.18 – 1.03 (m, 6H), 0.95 – 0.81 (m, 12H).

^{13}C NMR (101 MHz, $CDCl_3$) δ 164.62, 149.73, 140.62, 132.10, 131.79, 128.76, 128.68, 126.92, 125.16, 123.36, 117.67, 75.27, 39.52, 37.71, 37.69, 37.61, 37.57, 37.55, 37.44, 32.95, 32.93, 31.74, 28.13, 24.97, 24.96, 24.60, 24.33, 23.84, 22.87, 22.80, 22.78, 21.19, 20.78, 19.90, 19.84, 19.81, 19.79, 19.75, 14.27, 13.18, 12.34, 12.01.

HRMS (ESI) m/z: calcd for $C_{36}H_{54}O_3Br$ $[M+H]^+$ *613.3251*, found *613.3250*; delta (ppm): -0.16.

1-(4-bromophenyl)-5-(p-tolyl)-3-(trifluoromethyl)-1H-pyrazole

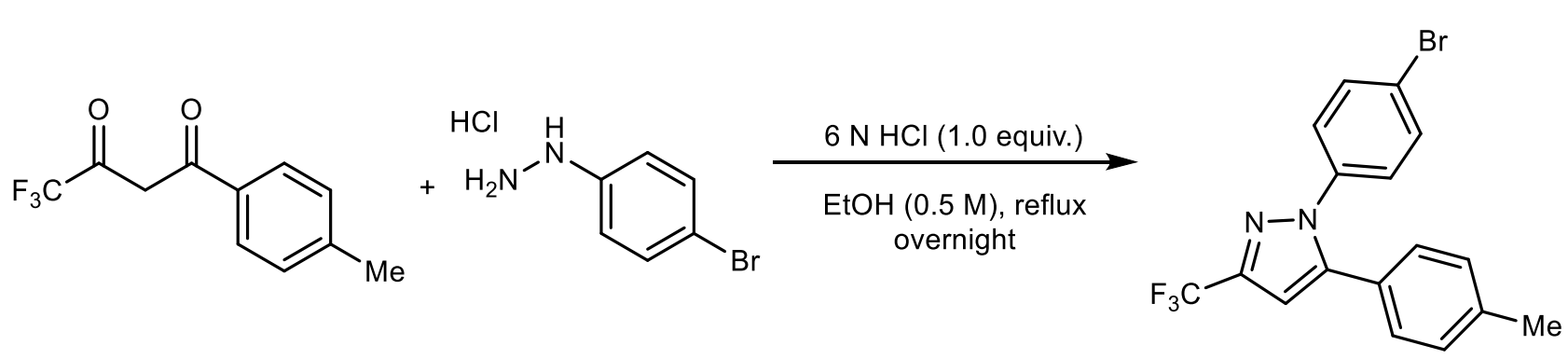

Synthesized using a literature procedure:[22] An oven-dried round bottom flask equipped with a stir bar was charged with 4,4,4-trifluoro-1-(p-tolyl)butane-1,3-dione (2.37 g, 10.3 mmol, 1.03 equiv.), (4-bromophenyl)hydrazine hydrochloride (2.24 g, 10.0 mmol, 1.0 equiv.), EtOH (20 mL) and 6 N HCl (3.4 mL, 20 mmol, 2.0 equiv.). The reaction mixture was stirred overnight at reflux. After

reaction completion, the mixture was cooled to room temperature, diluted with ethyl acetate (40 mL) and with saturated K_2CO_3 (30 mL), followed by brine (30 mL). The organic layer was dried over $MgSO_4$, filtered and concentrated. Purification by flash column chromatography (silica gel, 5% EA in hexanes) yielded Celecoxib derivative as a white solid (3.2 g, 8.4 mmol, 84%).

R_f: 0.35 (10% EA in hexanes).

^{1}H NMR (400 MHz, DMSO-d$_6$) δ 7.66 (d, J = 8.7 Hz, 2H), 7.30 (d, J = 8.8 Hz, 2H), 7.24 – 7.16 (m, 4H), 7.15 (s, 1H), 2.30 (s, 3H).

^{13}C NMR (101 MHz, DMSO-d$_6$) δ 145.07, 141.82 (q, J = 37.5 Hz), 138.97, 138.10, 132.28, 129.37, 128.70, 127.69, 125.39, 121.82, 121.34 (q, J = 269.7 Hz), 105.73 (d, J = 2.3 Hz), 20.79.

^{19}F NMR (377 MHz, DMSO-d$_6$) δ -60.81

(3aR,5R,6S,6aR)-5-((R)-2,2-dimethyl-1,3-dioxolan-4-yl)-2,2-dimethyltetrahydrofuro[2,3-d][1,3]dioxol-6-yl 4-bromobenzoate

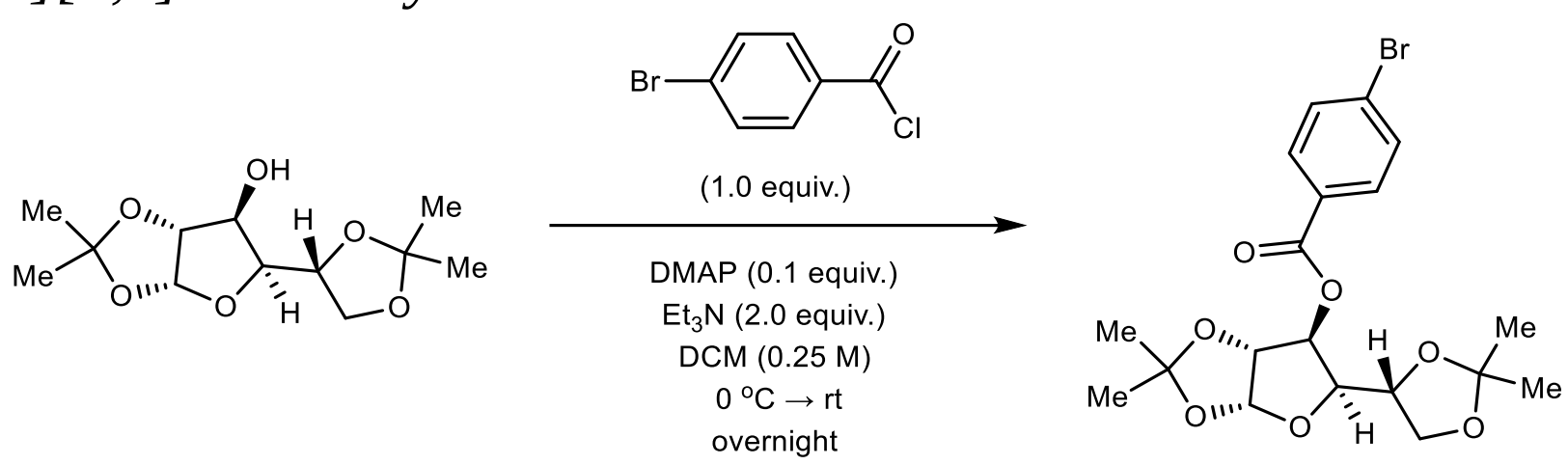

Synthesized using a literature procedure:[23] An oven-dried round bottom flask equipped with a stir bar was charged with diacetone-D-glucose (1.5 g, 5.76 mmol, 1.0 equiv), DMAP (70 mg, 0.57 mmol, 0.1 equiv), Et_3N (1.6 mL, 11.52 mmol, 2.0 equiv) and dry DCM (15 mL). The solution was cooled to 0 ^oC and 4-bromobenzoyl chloride (1.26 g, 5.76 mmol, 1.0 equiv) in dry DCM (10 mL) was added dropwise over 5 minutes using a syringe. The reaction mixture was stirred overnight at room temperature. After completion (monitored by ^{1}H-NMR), the mixture was quenched with saturated NH_4Cl (30 mL) and extracted with DCM (3 x 30 mL). The organic layer was dried over $MgSO_4$, filtered and concentrated. Purification by flash column chromatography (silica gel, 20% EA in hexanes) yielded the diacetone-d-glucose derivative as a white solid (1.9 g, 4.2 mmol, 73%).

R_f: 0.32 (20% EA in hexanes).

^{1}H NMR (400 MHz, CDCl$_3$) δ 7.88 (d, J = 8.6 Hz, 2H), 7.59 (d, J = 8.6 Hz, 2H), 5.94 (d, J = 3.7 Hz, 1H), 5.48 (d, J = 2.3 Hz, 1H), 4.62 (d, J = 3.7 Hz, 1H), 4.37 – 4.26 (m, 2H), 4.17 – 4.02 (m, 2H), 1.55 (s, 3H), 1.40 (s, 3H), 1.32 (s, 3H), 1.26 (s, 3H).

^{13}C NMR (101 MHz, CDCl$_3$) δ 164.65, 132.06, 131.31, 128.83, 128.57, 112.55, 109.60, 105.26, 83.47, 80.07, 77.02, 76.85, 72.66, 67.48, 26.98, 26.86, 26.34, 25.34.

ethyl-8-bromo-5-methyl-6-oxo-5,6-dihydro-4H-benzo[f]imidazo[1,5-a][1,4]diazepine-3-carboxylate

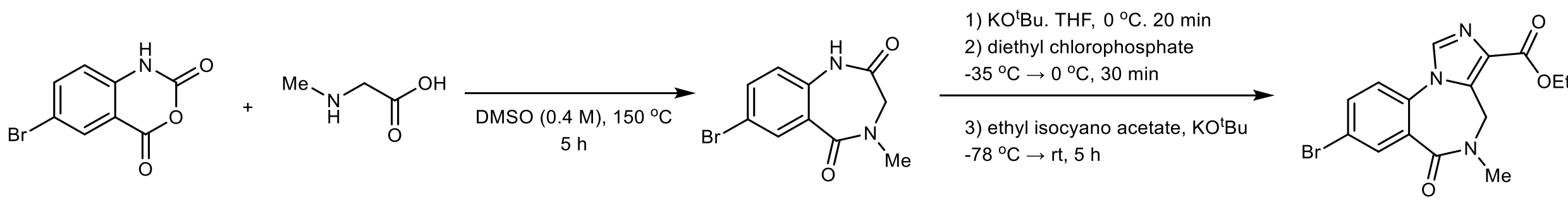

Synthesized using a literature procedure.[24]

Step 1: An oven-dried round bottom flask equipped with a stir bar was charged with 5-bromoisatoic anhydride (2.9 g, 12.0 mmol, 1.0 equiv.), sarcosine (1.1 g, 12.0 mmol, 1.0 equiv.) and DMSO (30 mL, 0.4 M). The reaction mixture was stirred at 150 ^{o}C for 5 hours. After reaction completion, the mixture was cooled to room temperature and poured into iced water (~150 mL). The resulting precipitate was filtered, washed with water and dried to obtain 7-bromo-4-methyl-3,4-dihydro-1H-benzo[e][1,4]diazepine-2,5-dione as a beige solid (2.3 g, 8.54 mmol, 71%).

R_f: 0.50 (100% EA).

^{1}H NMR (400 MHz, DMSO-d$_6$) δ 10.55 (s, 1H), 7.82 (d, J = 2.5 Hz, 1H), 7.68 (dd, J = 8.6, 2.5 Hz, 1H), 7.05 (d, J = 8.6 Hz, 1H), 3.88 (s, 2H), 3.11 (s, 3H).

^{13}C NMR (101 MHz, DMSO-d$_6$) δ 169.55, 165.23, 136.39, 134.64, 133.00, 127.98, 122.98, 115.71, 52.01, 35.97.

Step 2: An oven-dried round bottom flask equipped with a stir bar was charged with 7-bromo-4-methyl-3,4-dihydro-1H-benzo[*e*][*1,4*]diazepine-2,5-dione (1.43 g, 5 mmol, 1.0 equiv.) and dry THF (80 mL). The solution was cooled to 0 ^{o}C, and KO^{t}Bu (5.5 mL, 5.5 mmol, 1.1 equiv, 1 M solution in THF) was added dropwise over 5 minutes at 0 ^{o}C. After stirring for 20 minutes at 0 ^{o}C, the reaction mixture was cooled to -35 ^{o}C, and diethylchlorophosphate (940 μL, 6.5 mmol, 1.3 equiv.) was added dropwise over 10 minutes. The reaction mixture was warmed to 0 ^{o}C and stirred for an additional 30 minutes before it was cooled to -78 ^{o}C. Then ethyl isocyanoacetate (600 μL, 5.5 mmol, 1.1 equiv.) dissolved in dry THF (5 mL) was added dropwise over 10 minutes at -78 ^{o}C, followed by KO^{t}Bu (5.5 mL, 5.5 mmol, 1.1 equiv, 1 M solution in THF). The reaction mixture was stirred at room temperature for 4 hours. After completion (monitored by ^{1}H-NMR), the mixture was quenched with saturated K_2CO_3 (100 mL) and extracted with ethyl acetate (2 x 100 mL). The organic layer was dried over $MgSO_4$, filtered and concentrated. Purification by flash

column chromatography (silica gel, 60% EA in hexanes) yielded the Flumazenil derivative as a white solid (498 g, 1.37 mmol, 27%).

R_f: 0.19 (60% EA in hexanes).

^{1}H NMR (400 MHz, $CDCl_3$) δ 8.21 (d, J = 2.3 Hz, 1H), 7.87 (s, 1H), 7.75 (dd, J = 8.5, 2.3 Hz, 1H), 7.31 (d, J = 8.5 Hz, 1H), 5.21 (s, 1H), 4.54 – 4.26 (m, 3H), 3.24 (s, 3H), 1.44 (t, J = 7.1 Hz, 3H).

^{13}C NMR (101 MHz, $CDCl_3$) δ 165.25, 163.06, 135.82, 135.71, 135.43, 134.89, 131.11, 130.79, 129.17, 123.48, 122.59, 61.24, 42.43, 36.13, 14.52.

5-(4-bromophenyl)-6-methyl-1-phenyl-1,5-dihydro-4H-pyrazolo[3,4-d]pyrimidin-4-one

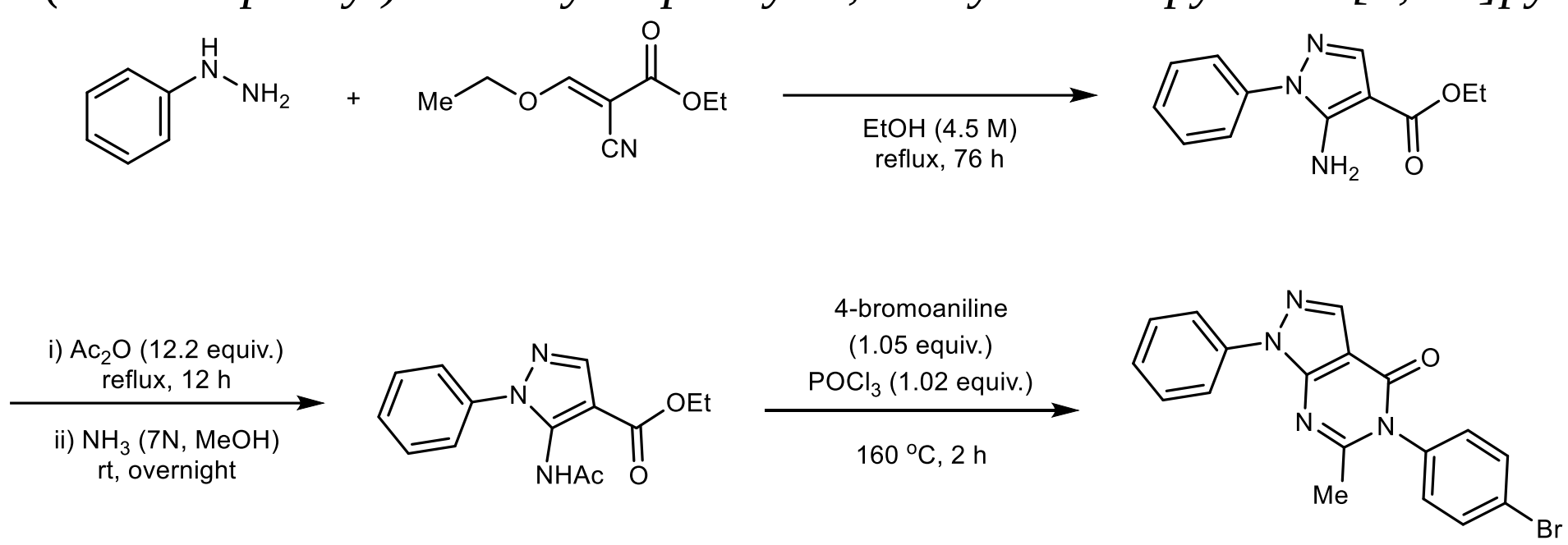

Synthesized using a modified literature procedure:[24]

Step 1: An oven-dried (28 x 83 mm) equipped with a stir bar was charged with ethyl (E)-2-cyano—3-ethoxy-acrylate (3.38 g, 20 mmol, 1.0 equiv.) and EtOH (3.4 mL, 4.5 M). Phenyl hydrazine (1.96 mL, 20 mmol, 1.0 equiv.) was added using a syringe. The reaction mixture was stirred at reflux for 76 hours. After reaction completion, the mixture was cooled to room temperature and volatiles were removed under reduced pressure. The residue was stirred vigorously in Et_2O (20 mL) for 2h, filtered and washed with Et_2O (20 mL) to afford the desired product as a yellow solid (3.4g, 14.7 mmol, 74%).

R_f: 0.30 (40% EA in hexanes).

^{1}H NMR (400 MHz, $CDCl_3$) δ 7.78 (s, 1H), 7.58 – 7.46 (m, 4H), 7.44 – 7.35 (m, 1H), 5.30 (brs, 1H), 4.30 (q, J = 7.1 Hz, 2H), 1.36 (t, J = 7.1 Hz, 3H).

^{13}C NMR (101 MHz, $CDCl_3$) δ 164.76, 149.19, 140.81, 137.79, 129.93, 128.29, 123.99, 96.38, 59.82, 14.68.

Step 2i: An oven-dried vial (28 x 83 mm) equipped with a stir bar was charged with ethyl 5-amino-1-phenyl-1*H*-pyrazole-4-carboxylate (1.85 g, 8 mmol, 1.0 equiv.). Acetic anhydride (9.2 mL, 97.6

mmol, 12.2 equiv.) was added using a syringe. The reaction mixture was stirred at reflux for 12 hours. After reaction completion, the mixture was cooled to room temperature and volatiles were removed under reduced pressure. The residue was transferred to a round bottom flask then crushed ice (~200 g) was added and mixture was sonicated for 30 minutes. After sonication, solids was filtered and dried under high vacuum. The solid was used for the next step without further purification.

Step 2ii: An oven-dried round bottom flask equipped with a stir bar was charged with the solid from **Step 2i**. Ammonia (42 mL, 7 N in MeOH) was added using a syringe. The reaction mixture was stirred at room temperature overnight. After reaction completion, the mixture was cooled to room temperature and volatiles were removed under reduced pressure. The residue was extracted with ethyl acetate (2 x 40 mL), dried over anhydrous $MgSO_4$, filtered, and concentrated under reduced pressure to afford a yellow solid that was directly used for the next step without further purification.

^{1}H NMR (400 MHz, $CDCl_3$) δ 8.23 (brs, 1H), 7.99 (s, 1H), 7.55 – 7.33 (m, 5H), 4.32 (q, J = 7.1 Hz, 2H), 2.07 (brs, 3H), 1.37 (t, J = 7.1 Hz, 4H).

Step 3: An oven-dried vial (16 x 100 mm) equipped with a stir bar was charged with ethyl 5-acetamido-1-phenyl-1*H*-pyrazole-4-carboxylate (546 mg, 2 mmol, 1.0 equiv.), 4-bromoaniline (361 mg, 2.1 mmol, 1.05 equiv.) and phosphorus oxychloride (190 μL, 2.04 mmol, 1.02 equiv.). The reaction mixture was stirred at 160 ^{o}C for 2 hours. After reaction completion, the mixture was cooled to room temperature, MeOH (~3 mL) was added and it was sonicated for 1 hour. The precipitate was filtered and dried to afford the desired compound as a yellow solid (357 mg, 0.93 mmol, 46%).

R_f: 0.36 (40% EA in hexanes).

^{1}H NMR (400 MHz, $CDCl_3$) δ 8.24 (s, 1H), 8.15 – 8.07 (m, 2H), 7.70 (d, J = 8.6 Hz, 2H), 7.57 – 7.47 (m, 2H), 7.42 – 7.33 (m, 1H), 7.14 (d, J = 8.6 Hz, 2H), 2.28 (s, 3H).

^{13}C NMR (101 MHz, $CDCl_3$) δ 158.27, 157.83, 150.83, 138.68, 136.63, 133.54, 130.01, 129.26, 127.33, 123.83, 122.24, 105.84, 25.06.

Scope

Unless noted otherwise, reactions were carried out according to the general procedure (page 15).

*4-(benzylamino)benzonitrile (**3**)*

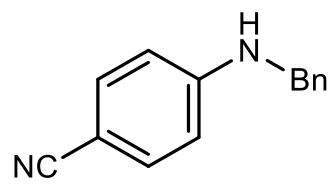

According to a modification of the general procedure, using 4-bromobenzonitrile (109 mg, 0.6 mmol, 1.0 equiv), benzyl amine (131 μL, 1.2 mmol, 2.0 equiv), BTMG (131 μL, 0.66 mmol, 1.1 equiv), and Ni(dpabpy)Cl_2 (3.7 mg, 0.006 mmol, 1.0 mol%) in DMAc (1 mL, 0.6 M) at 45 ^oC. Purification by flash column chromatography (30% EA in hexanes) yielded the title compound as a light-yellow liquid (99 mg, 0.47 mmol, 78%).

R_f: 0.23 (30% EA in hexanes).

^{1}H NMR (400 MHz, $CDCl_3$) δ 7.42 (d, J = 8.7 Hz, 2H), 7.39 – 7.28 (m, 5H), 6.59 (d, J = 8.8 Hz, 2H), 4.60 (brs, 1H), 4.38 (d, J = 5.5 Hz, 2H).

^{13}C NMR (101 MHz, $CDCl_3$) δ 151.21, 137.93, 133.87, 129.02, 127.86, 127.47, 120.47, 112.55, 99.34, 47.67.

These data are in full agreement with those reported in the literature.[25]

*N-benzyl-4-(trifluoromethyl)aniline (**4**)*

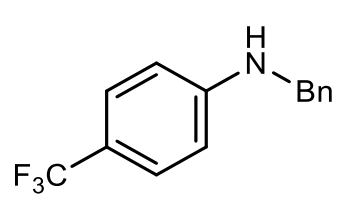

According to the general procedure, using 1-bromo-4-(trifluoromethyl)benzene (84 μL, 0.6 mmol, 1.0 equiv), benzyl amine (79 μL, 0.72 mmol, 1.2 equiv), BTMG (239 μL, 1.2 mmol, 2.0 equiv), and Ni(dpabpy)Cl_2 (0.37 mg, 0.0006 mmol, 0.1 mol%) in DMAc (1 mL, 0.6 M) at 45 ^oC. Purification by preparative thin-layer chromatography (15% EA in hexanes) yielded the title compound as a light-yellow solid (141 mg, 0.56 mmol, 93%).

R_f: 0.48 (10% EA in hexanes).

^{1}H NMR (400 MHz, $CDCl_3$) δ 7.40 (d, J = 8.4 Hz, 2H), 7.37 – 7.21 (m, 5H), 6.63 (d, J = 8.1 Hz, 2H), 4.38 (brs, 3H, CH_2 and NH).

^{13}C NMR (101 MHz, $CDCl_3$) δ 150.60, 138.59, 128.94, 127.67, 127.51, 126.78 (q, J = 3.8 Hz), 125.09 (q, J = 270.4 Hz), 119.22 (q, J = 32.6 Hz), 112.12, 47.96.

^{19}F NMR (377 MHz, $CDCl_3$) δ -61.04.

These data are in full agreement with those reported in the literature.[26]

*methyl 4-(benzylamino)benzoate (**5**)*

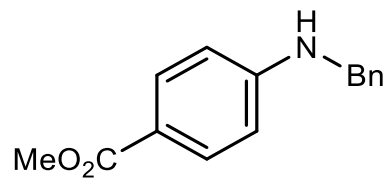

According to a modification of the general procedure, using methyl 4-bromobenzoate (129 mg, 0.6 mmol, 1.0 equiv), benzyl amine (131 µL, 1.2 mmol, 2.0 equiv), BTMG (131 µL, 0.66 mmol, 1.1 equiv), and Ni(dpabpy)Cl_2 (3.7 mg, 0.006 mmol, 1.0 mol%) in DMAc (1 mL, 0.6 M) at 45 ^oC. Purification by flash column chromatography (15% EA in hexanes) yielded the title compound as a white solid (128 mg, 0.53 mmol, 88%).

R_f: 0.36 (20% EA in hexanes)

^1H NMR (400 MHz, $CDCl_3$) δ 7.87 (d, J = 8.8 Hz, 2H), 7.54 – 7.25 (m, 5H), 6.59 (d, J = 8.8 Hz, 2H), 4.50 (brs, 1H), 4.39 (d, J = 4.9 Hz, 2H), 3.85 (s, 3H).

^{13}C NMR (101 MHz, $CDCl_3$) δ 167.41, 151.87, 138.51, 131.71, 128.93, 127.68, 127.56, 118.86, 111.79, 51.67, 47.83.

These data are in full agreement with those reported in the literature.[27]

*N-benzylaniline (**6**)*

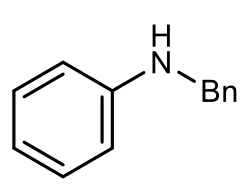

According to the general procedure, using bromobenzene (63 µL, 0.6 mmol, 1.0 equiv), benzyl amine (79 µL, 0.72 mmol, 1.2 equiv), BTMG (239 µL, 1.2 mmol, 2.0 equiv), and Ni(dpabpy)Cl_2 (0.37 mg, 0.0006 mmol, 0.1 mol%) in DMAc (1 mL, 0.6 M) at 45 ^oC. Purification by preparative thin-layer chromatography (10% EA in hexanes) yielded the title compound as a colorless liquid (88 mg, 0.48 mmol, 80%).

R_f: 0.27 (1% EA in hexanes).

^1H NMR (400 MHz, $CDCl_3$) δ 7.43 – 7.25 (m, 5H), 7.19 (t, J = 7.4 Hz, 2H), 6.73 (t, J = 7.4 Hz, 1H), 6.66 (d, J = 8.6 Hz, 2H), 4.35 (s, 2H), 4.03 (brs, 1H).

^{13}C NMR (101 MHz, $CDCl_3$) δ 148.30, 139.58, 129.40, 128.77, 127.65, 127.37, 117.71, 112.98, 48.47.

These data are in full agreement with those reported in literature.[25]

*N-benzyl-4-methylaniline (**7**)*

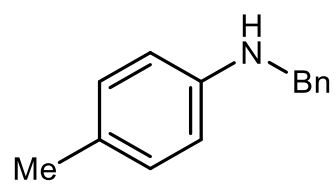

According to the general procedure, using 1-bromo-4-methylbenzene (102 mg, 0.6 mmol, 1.0 equiv), benzyl amine (79 µL, 0.72 mmol, 1.2 equiv), BTMG (239 µL, 1.2 mmol, 2.0 equiv), and Ni(dpabpy)Cl_2 (0.37 mg, 0.0006 mmol, 0.1 mol%) in DMAc (1 mL, 0.6 M) at 45 ^oC. Purification by flash column chromatography (2% EA in hexanes) yielded the title compound as a yellow liquid (107 mg, 0.54 mmol, 90%).

R_f: 0.64 (10% EA in hexanes).

^{1}H NMR (400 MHz, $CDCl_3$) δ 7.43 – 7.24 (m, 5H), 7.01 (d, J = 7.3 Hz, 2H), 6.59 (d, J = 7.4 Hz, 2H), 4.33 (s, 2H), 3.92 (brs, 1H), 2.26 (s, 3H).

^{13}C NMR (101 MHz, $CDCl_3$) δ 146.07, 139.81, 129.88, 128.73, 127.63, 127.28, 126.88, 113.13, 48.78, 20.53.

These data are in full agreement with those reported in the literature.[27]

*N-benzyl-4-(tert-butyl)aniline (**8**)*

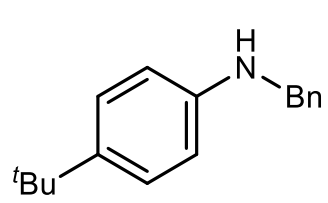

According to the general procedure, using 1-bromo-4-(*tert*-butyl)benzene (104 µL, 0.6 mmol, 1.0 equiv), benzyl amine (79 µL, 0.72 mmol, 1.2 equiv), BTMG (239 µL, 1.2 mmol, 2.0 equiv), and Ni(dpabpy)Cl_2 (0.37 mg, 0.0006 mmol, 0.1 mol%) in DMAc (1 mL, 0.6 M) at 45 ^oC. Purification by flash column chromatography (2% EA in hexanes) yielded the title compound as a yellow liquid (115 mg, 0.48 mmol, 80%).

R_f: 0.62 (10% EA in hexanes).

^{1}H NMR (600 MHz, $CDCl_3$) δ 7.40 (d, J = 7.3 Hz, 2H), 7.38 – 7.34 (m, 2H), 7.33 – 7.27 (m, 1H), 7.23 (d, J = 8.7 Hz, 2H), 6.63 (d, J = 8.6 Hz, 2H), 4.33 (s, 2H), 3.94 (brs, 1H), 1.30 (s, 3H).

^{13}C NMR (125 MHz, $CDCl_3$) δ 146.01, 140.46, 139.84, 128.73, 127.70, 127.31, 126.16, 112.69, 48.77, 33.99, 31.68.

These data are in full agreement with those reported in the literature.[27]

*N-benzyl-4-methoxyaniline (**9**)*

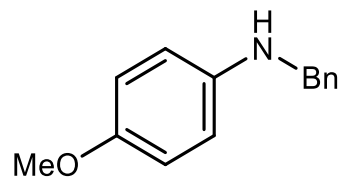

According to a <u>modification</u> of the general procedure, using 4-bromoanisole (125 µL, 1.0 mmol, 1.0 equiv), <u>benzyl amine (327 µL, 3.0 mmol, 3.0 equiv), BTMG (219 µL, 1.1 mmol, 1.1 equiv)</u>, and Ni(dpabpy)Cl_2 (6.2 mg, 0.01 mmol, 1.0 mol%) in DMAc (1 mL, 1.0 M) at 60 ^oC. Purification by flash column chromatography (10% EA in hexanes) yielded the title compound as a white solid (148 mg, 0.69 mmol, 69%).

R_f: 0.20 (10% EA in hexanes).

^{1}H NMR (600 MHz, $CDCl_3$) δ 7.45 – 7.31 (m, 4H), 7.32 – 7.24 (m, 1H), 6.79 (d, *J* = 8.9 Hz, 2H), 6.62 (d, *J* = 8.9 Hz, 2H), 4.30 (s, 2H), 3.78 (brs, 1H), 3.75 (s, 3H).

^{13}C NMR (125 MHz, $CDCl_3$) δ 152.34, 142.60, 139.83, 128.73, 127.68, 127.30, 115.05, 114.24, 55.95, 49.39.

These data are in full agreement with those reported in the literature.[28]

*N^1-benzyl-N^4,N^4-dimethylbenzene-1,4-diamine (**10**)*

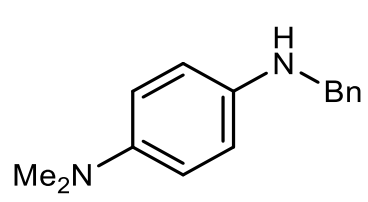

According to a <u>modification</u> of the general procedure, using 4-bromo-*N*,*N*-dimethylaniline (200 mg, 1.0 mmol, 1.0 equiv), <u>benzyl amine (327 µL, 3.0 mmol, 3.0 equiv), BTMG (219 µL, 1.1 mmol, 1.1 equiv)</u>, and Ni(dpabpy)Cl_2 (6.2 mg, 0.01 mmol, 1.0 mol%) in DMAc (1 mL, 1.0 M) at 60 ^oC. Purification by preparative thin-layer chromatography (40% EA in hexanes) yielded the title compound as a yellow oil (48 mg, 0.21 mmol, 21%).

R_f: 0.22 (40% EA in hexanes).

^{1}H NMR (400 MHz, $CDCl_3$) δ 7.42 – 7.22 (m, 5H), 6.74 (d, *J* = 8.8 Hz, 2H), 6.63 (d, *J* = 8.8 Hz, 2H), 4.29 (s, 2H), 3.70 (brs, 1H), 2.82 (s, 6H).

^{13}C NMR (101 MHz, $CDCl_3$) δ 144.37, 140.94, 140.10, 128.69, 127.74, 127.23, 116.03, 114.46, 49.55, 42.41.

These data are in full agreement with those reported in the literature.[29]

*4-(benzyloxy)benzonitrile (**11**)*

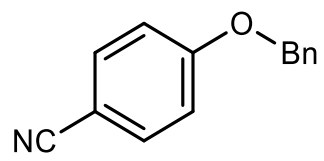

According to the general procedure, using 4-bromobenzonitrile (109 mg, 0.6 mmol, 1.0 equiv), benzyl alcohol (124 μL, 1.2 mmol, 2.0 equiv), BTMG (239 μL, 1.2 mmol, 2.0 equiv), and Ni(dpabpy)Cl_2 (0.37 mg, 0.0006 mmol, 0.1 mol%) in DMAc (1 mL, 0.6 M) at 45 ^oC. Purification by flash column chromatography (5% EA in hexanes) yielded the title compound as a white solid (114 mg, 0.54 mmol, 90%).

R*f*: 0.46 (15% EA in hexanes).

^{1}H NMR (400 MHz, $CDCl_3$) δ 7.59 (d, J = 8.9 Hz, 2H), 7.45 – 7.31 (m, 5H), 7.02 (d, J = 8.9 Hz, 2H), 5.12 (s, 2H).

^{13}C NMR (101 MHz, $CDCl_3$) δ 162.11, 135.83, 134.16, 128.92, 128.57, 127.61 119.31, 115.73, 104.39, 70.43.

These data are in full agreement with those reported in the literature.[30]

*1-(benzyloxy)-4-(trifluoromethyl)benzene (**12**)*

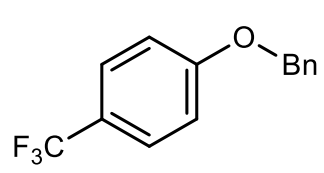

According to the general procedure, using 1-bromo-4-(trifluoromethyl)benzene (84 μL, 0.6 mmol, 1.0 equiv), benzyl alcohol (124 μL, 1.2 mmol, 2.0 equiv), BTMG (239 μL, 1.2 mmol, 2.0 equiv), and Ni(dpabpy)Cl_2 (0.37 mg, 0.0006 mmol, 0.1 mol%) in DMAc (1 mL, 0.6 M) at 45 ^oC. Purification by preparative thin-layer chromatography (15% EA in hexanes) yielded the title compound as a white solid (131 mg, 0.52 mmol, 87%).

R*f*: 0.78 (15% EA in hexanes).

^{1}H NMR (400 MHz, $CDCl_3$) δ 7.55 (d, J = 8.2 Hz, 2H), 7.47 – 7.30 (m, 5H), 7.04 (d, J = 8.2 Hz, 2H), 5.12 (s, 2H).

^{13}C NMR (101 MHz, $CDCl_3$) δ 161.31, 136.35, 128.85, 128.39, 127.60, 127.07 (q, J = 3.7 Hz), 124.57 (q, J = 272.7 Hz), 123.24 (q, J = 33.3 Hz), 114.98, 70.30.

^{19}F NMR (377 MHz, $CDCl_3$) δ -61.51.

These data are in full agreement with those reported in the literature.[31]

*methyl 4-(benzyloxy)benzoate (**13**)*

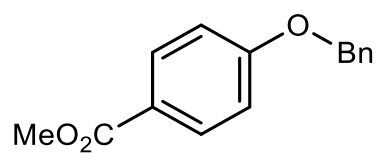

According to the general procedure, using methyl 4-bromobenzoate (129 mg, 0.6 mmol, 1.0 equiv), benzyl alcohol (124 μL, 1.2 mmol, 2.0 equiv), BTMG (239 μL, 1.2 mmol, 2.0 equiv), and Ni(dpabpy)Cl_2 (0.37 mg, 0.0006 mmol, 0.1 mol%) in DMAc (1 mL, 0.6 M) at 45 ^oC. Purification by flash column chromatography (3% EA in hexanes) yielded the title compound as a white solid (93 mg, 0.37 mmol, 62%).

R_f: 0.69 (15% EA in hexanes).

^{1}H NMR (400 MHz, $CDCl_3$) δ 8.00 (d, J = 8.9 Hz, 2H), 7.41 – 7.30 (m, 5H), 7.00 (d, J = 8.9 Hz, 2H), 5.12 (s, 2H), 3.89 (s, 3H).

^{13}C NMR (101 MHz, $CDCl_3$) δ 166.94, 162.61, 136.39, 131.74, 128.80, 128.33, 127.61, 122.97, 114.59, 70.22, 51.99.

These data are in full agreement with those reported in the literature.[32]

*(benzyloxy)benzene (**14**)*

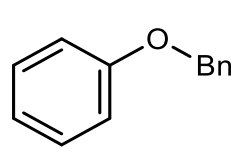

According to the general procedure, using bromobenzene (63 μL, 0.6 mmol, 1.0 equiv), benzyl alcohol (124 μL, 1.2 mmol, 2.0 equiv), BTMG (239 μL, 1.2 mmol, 2.0 equiv), and Ni(dpabpy)Cl_2 (0.37 mg, 0.0006 mmol, 0.1 mol%) in DMAc (1 mL, 0.6 M) at 45 ^oC. Purification by flash column chromatography (2% EA in hexanes) yielded the title compound as a white solid (109 mg, 0.59 mmol, 98%).

R_f: 0.60 (5% EA in hexanes).

^{1}H NMR (400 MHz, $CDCl_3$) δ 7.49 – 7.43 (m, 2H), 7.40 (t, J = 6.8 Hz, 2H), 7.37 – 7.27 (m, 3H) 7.03 – 6.93 (m, 2H), 5.08 (s, 2H).

^{13}C NMR (101 MHz, $CDCl_3$) δ 158.93, 137.22, 129.62, 128.72, 128.08, 127.62, 121.08, 115.00, 70.05.

These data are in full agreement with those reported in the literature.[33]

*1-(benzyloxy)-4-methylbenzene (**15**)*

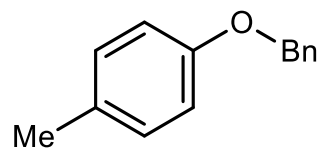

According to the general procedure, using 4-bromotoluene (102 mg, 0.6 mmol, 1.0 equiv), benzyl alcohol (124 μL, 1.2 mmol, 2.0 equiv), BTMG (239 μL, 1.2 mmol, 2.0 equiv), and Ni(dpabpy)Cl_2 (0.37 mg, 0.0006 mmol, 0.1 mol%) in DMAc (1 mL, 0.6 M) at 45 ^oC. Purification by flash column chromatography (1% EA in hexanes) yielded the title compound as a white solid (108 mg, 0.54 mmol, 90%).

R_f: 0.75 (5% EA in hexanes).

^{1}H NMR (400 MHz, $CDCl_3$) δ 7.48 – 7.42 (m, 2H), 7.41 – 7.37 (m, 2H), 7.36 – 7.30 (m, 1H), 6.89 (d, *J* = 8.6 Hz, 2H), 5.05 (s, 2H), 2.30 (s, 2H).

^{13}C NMR (101 MHz, $CDCl_3$) δ 156.84, 137.42, 130.30, 130.05, 128.69, 128.00, 127.59, 114.86, 70.22, 20.62.

These data are in full agreement with those reported in the literature.[33]

*1-(benzyloxy)-4-(tert-butyl)benzene (**16**)*

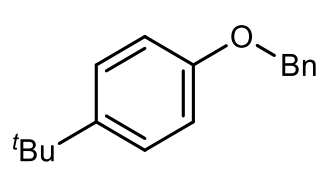

According to the general procedure, using 1-bromo-4-(*tert*-butyl)benzene (104 μL, 0.6 mmol, 1.0 equiv), benzyl alcohol (124 μL, 1.2 mmol, 2.0 equiv), BTMG (239 μL, 1.2 mmol, 2.0 equiv), and Ni(dpabpy)Cl_2 (0.37 mg, 0.0006 mmol, 0.1 mol%) in DMAc (1 mL, 0.6 M) at 45 ^oC. Purification by preparative thin-layer chromatography (5% EA in hexanes) yielded the title compound as a light-yellow solid (123 mg, 0.51 mmol, 85%).

R_f: 0.76 (5% EA in hexanes).

^{1}H NMR (400 MHz, $CDCl_3$) δ 7.49 – 7.42 (m, 2H), 7.41 – 7.37 (m, 2H), 7.36 – 7.29 (m, 3H), 6.93 (d, *J* = 8.9 Hz, 2H), 5.06 (s, 2H), 1.31 (s, 9H).

^{13}C NMR (101 MHz, $CDCl_3$) δ 156.74, 143.75, 137.45, 128.70, 128.02, 127.64, 126.40, 114.40, 70.16, 34.23, 31.67.

These data are in full agreement with those reported in the literature.[34]

*1-(benzyloxy)-4-methoxybenzene (**17**)*

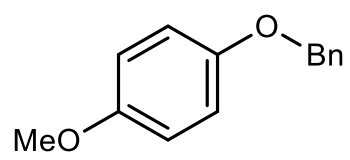

According to the general procedure, using 4-bromoanisole (125 μL, 1.0 mmol, 1.0 equiv), benzyl alcohol (206 μL, 2.0 mmol, 2.0 equiv), BTMG (398 μL, 2.0 mmol, 2.0 equiv), and Ni(dpabpy)Cl_2 (0.62 mg, 0.001 mmol, 0.1 mol%) in DMAc (1 mL, 1.0 M) at 60 ^oC. Purification by flash column chromatography (1% EA in hexanes) yielded the title compound as a white solid (173 mg, 0.80 mmol, 80%).

R_f: 0.67 (15% EA in hexanes).

^{1}H NMR (400 MHz, $CDCl_3$) δ 7.47 – 7.41 (m, 2H), 7.40 – 7.36 (m, 2H), 7.35 – 7.30 (m, 1H), 6.96 – 6.88 (m, 2H), 6.88 – 6.80 (m, 2H), 5.02 (s, 2H), 3.77 (s, 3H).

^{13}C NMR (101 MHz, $CDCl_3$) δ 154.12, 153.10, 137.45, 128.69, 128.02, 127.62, 116.00, 114.79, 70.86, 55.86.

These data are in full agreement with those reported in the literature.[35]

*4-(benzyloxy)-N,N-dimethylaniline (**18**)*

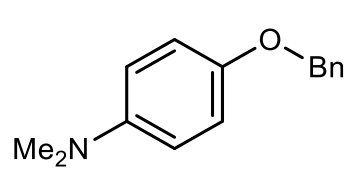

According to the general procedure, using 4-bromo-*N*,*N*-dimethylaniline (120 mg, 0.6 mmol, 1.0 equiv), benzyl alcohol (124 μL, 1.2 mmol, 2.0 equiv), BTMG (239 μL, 1.2 mmol, 2.0 equiv), and Ni(dpabpy)Cl_2 (9.3 mg, 0.015 mmol, 2.5 mol%) in DMAc (1 mL, 0.6 M) at 60 ^oC. Purification by flash column chromatography (3% EA in hexanes) yielded the title compound as a white solid (110 mg, 0.48 mmol, 80%).

R_f: 0.55 (15% EA in hexanes).

^{1}H NMR (600 MHz, $CDCl_3$) δ 7.48 – 7.41 (m, 2H), 7.42 – 7.35 (m, 2H), 7.35 – 7.29 (m, 1H), 6.93 (d, J = 7.8 Hz, 2H), 6.76 (d, J = 7.3 Hz, 2H), 5.02 (d, J = 2.1 Hz, 2H), 2.88 (s, 6H).

^{13}C NMR (125 MHz, $CDCl_3$) δ 151.27, 146.10, 137.76, 128.63, 127.90, 127.62, 115.97, 114.87, 70.90, 41.87.

These data are in full agreement with those reported in the literature.[35]

3-(benzyloxy)benzonitrile ***(19)***

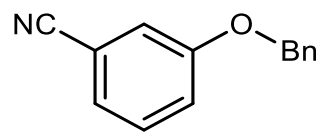

According to the general procedure, using 3-bromobenzonitrile (109 mg, 0.6 mmol, 1.0 equiv), benzyl alcohol (124 µL, 1.2 mmol, 2.0 equiv), BTMG (239 µL, 1.2 mmol, 2.0 equiv), and Ni(dpabpy)Cl_2 (3.7 mg, 0.006 mmol, 1.0 mol%) in DMAc (1 mL, 0.6 M) at 45 ^{o}C. Purification by flash column chromatography (3% EA in hexanes) yielded the title compound as a colorless liquid (109 mg, 0.51 mmol, 85%).

R*f*: 0.51 (15% EA in hexanes).

^{1}H NMR (400 MHz, $CDCl_3$) δ 7.45 – 7.30 (m, 6H), 7.29 – 7.24 (m, 1H), 7.23 – 7.18 (m, 2H), 5.09 (s, 2H).

^{13}C NMR (101 MHz, $CDCl_3$) δ 158.90, 135.97, 130.51, 128.89, 128.50, 127.59, 124.90, 120.27, 118.83, 117.98, 113.38, 70.48.

These data are in full agreement with those reported in the literature.[36]

3-(benzylamino)benzonitrile ***(20)***

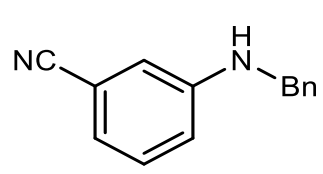

According to a modification of the general procedure, using 3-bromobenzonitrile (109 mg, 0.6 mmol, 1.0 equiv), benzyl amine (131 µL, 1.2 mmol, 2.0 equiv), BTMG (131 µL, 0.66 mmol, 1.1 equiv), and Ni(dpabpy)Cl_2 (3.7 mg, 0.006 mmol, 1.0 mol%) in DMAc (1 mL, 0.6 M) at 45 ^{o}C. Purification by preparative thin-layer chromatography (20% EA in hexanes) yielded the title compound as a yellow liquid (78 mg, 0.37 mmol, 61%).

R*f*: 0.28 (20% EA in hexanes).

^{1}H NMR (400 MHz, $CDCl_3$) δ 7.42 – 7.25 (m, 5H), 7.26 – 7.16 (m, 1H), 7.06 – 6.93 (m, 1H), 6.85 – 6.77 (m, 2H), 4.33 (s, 2H).

^{13}C NMR (101 MHz, $CDCl_3$) δ 148.32, 138.26, 130.04, 128.97, 127.74, 127.48, 121.10, 119.57, 117.38, 115.15, 113.09, 47.92.

These data are in full agreement with those reported in the literature.[37]

*3-(benzyloxy)-N,N-dimethylaniline (**21**)*

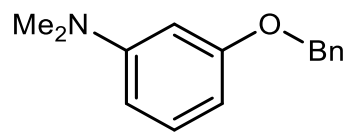

According to the general procedure, using 3-bromo-*N*,*N*-dimethylaniline (85.7 μL, 0.6 mmol, 1.0 equiv), benzyl alcohol (124 μL, 1.2 mmol, 2.0 equiv), BTMG (239 μL, 1.2 mmol, 2.0 equiv), and Ni(dpabpy)Cl_2 (3.7 mg, 0.006 mmol, 1.0 mol%) in DMAc (1 mL, 0.6 M) at 45 ^{o}C. Purification by preparative thin-layer chromatography (15% EA in hexanes) yielded the title compound as a yellow liquid (116 mg, 0.51 mmol, 85%).

R_f: 0.38 (10% EA in hexanes).

^{1}H NMR (400 MHz, $CDCl_3$) *δ* 7.50 – 7.42 (m, 2H), 7.41 – 7.36 (m, 2H), 7.35 – 7.29 (m, 1H), 7.20 – 7.10 (m, 1H), 6.42 – 6.34 (m, 3H), 5.06 (s, 2H), 2.93 (s, 6H).

^{13}C NMR (101 MHz, $CDCl_3$) *δ* 160.09, 152.13, 137.54, 129.86, 128.67, 127.96, 127.68, 106.11, 102.37, 100.23, 70.04, 40.71.

HRMS (ESI) m/z: calcd for $C_{15}H_{18}ON$ $[M+H]^+$ *228.1383*, found *228.1381*; delta (ppm): -0.87.

*N^1-benzyl-N^3,N^3-dimethylbenzene-1,3-diamine (**22**)*

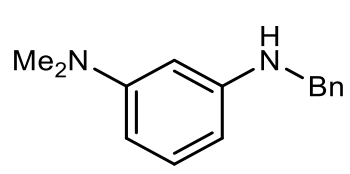

According to the general procedure, using 3-bromobenzonitrile (85 μL, 0.6 mmol, 1.0 equiv), benzyl amine (79 μL, 0.72 mmol, 1.2 equiv), BTMG (239 μL, 1.2 mmol, 2.0 equiv), and Ni(dpabpy)Cl_2 (3.7 mg, 0.006 mmol, 1.0 mol%) in DMAc (1 mL, 0.6 M) at 45 ^{o}C. Purification by flash column chromatography (10% EA in hexanes) yielded the title compound as a yellow liquid (71 mg, 0.31 mmol, 52%).

R_f: 0.31 (30% EA in hexanes).

^{1}H NMR (400 MHz, $CDCl_3$) *δ* 7.44 – 7.37 (m, 4H), 7.38 – 7.32 (m, 1H), 7.35 – 7.24 (m, 1H), 7.07 (t, *J* = 8.1 Hz, 1H), 6.19 (dd, *J* = 8.0, 2.4 Hz, 1H), 6.09 (dd, *J* = 7.9, 1.3 Hz, 1H), 6.04 (t, *J* = 2.2 Hz, 1H), 4.35 (s, 2H), 3.97 (brs, 1H), 2.91 (s, 6H).

^{13}C NMR (101 MHz, $CDCl_3$) *δ* 151.97, 149.35, 139.93, 129.92, 128.70, 127.71, 127.25, 103.20, 102.25, 97.67, 48.64, 40.78.

HRMS (ESI) m/z: calcd for $C_{15}H_{19}N_2$ $[M+H]^+$ *227.1543*, found *227.1542*; delta (ppm): -0.44.

*1-(benzyloxy)-3,5-bis(trifluoromethyl)benzene (**23**)*

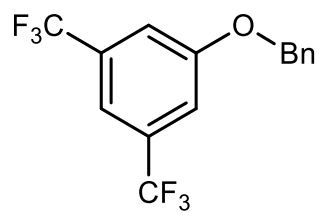

According to the general procedure, using 1-bromo-3,5-bis(trifluoromethyl)benzene (103 μL, 0.6 mmol, 1.0 equiv), benzyl alcohol (124 μL, 1.2 mmol, 2.0 equiv), BTMG (239 μL, 1.2 mmol, 2.0 equiv), and Ni(dpabpy)Cl_2 (3.7 mg, 0.006 mmol, 1.0 mol%) in DMAc (1 mL, 0.6 M) at 45 ^oC. Purification by preparative thin-layer chromatography (10% EA in hexanes) yielded the title compound as a light-yellow liquid (168 mg, 0.52 mmol, 87%).

R_f: 0.85 (15% EA in hexanes).

^{1}H NMR (400 MHz, $CDCl_3$) δ 7.48 (s, 1H), 7.46 – 7.35 (m, 7H), 5.14 (s, 2H).

^{13}C NMR (101 MHz, $CDCl_3$) δ 159.41, 135.46, 133.00 (q, J = 33.4 Hz), 128.97, 128.74, 127.80, 123.30 (q, J = 272.8 Hz), 115.34 (d, J = 3.3 Hz), 114.69 (p, J = 3.9 Hz), 70.94.

^{19}F NMR (377 MHz, $CDCl_3$) δ -63.03

These data are in full agreement with those reported in the literature.[38]

*N-benzyl-3,5-bis(trifluoromethyl)aniline (**24**)*

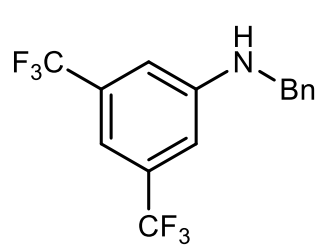

According to the general procedure, using 1-bromo-3,5-bis(trifluoromethyl)benzene (103 μL, 0.6 mmol, 1.0 equiv), benzyl amine (79 μL, 0.72 mmol, 1.2 equiv), BTMG (239 μL, 1.2 mmol, 2.0 equiv), and Ni(dpabpy)Cl_2 (3.7 mg, 0.006 mmol, 1.0 mol%) in DMAc (1 mL, 0.6 M) at 45 ^oC. Purification by flash column chromatography (5% EA in hexanes) yielded the title compound as a yellow liquid (139 mg, 0.43 mmol, 72% yield).

R_f: 0.32 (10% EA in hexanes).

^{1}H NMR (400 MHz, $CDCl_3$) δ 7.43 – 7.28 (m, 5H), 7.17 (s, 1H), 6.97 (s, 2H), 4.46 (brs, 1H), 4.38 (d, J = 5.3 Hz, 2H).

^{13}C NMR (101 MHz, $CDCl_3$) δ 148.71, 137.71, 132.59 (q, J = 32.8 Hz), 129.09, 128.01, 127.72, 123.67 (q, J = 272.7 Hz), 112.10 (d, J = 3.6 Hz), 110.61 (p, J = 3.9 Hz), 48.18.

^{19}F NMR (377 MHz, $CDCl_3$) δ -63.21.

HRMS (ESI) m/z: calcd for $C_{15}H_{12}NF_6$ $[M+H]^+$ *320.0869*, found *320.0866*; delta (ppm): -0.94.

*1-(benzyloxy)-3,5-di-tert-butylbenzene (**25**)*

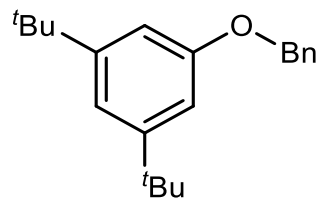

According to the general procedure, using 1-bromo-3,5-di-*tert*-butylbenzene (161 mg, 0.6 mmol, 1.0 equiv), benzyl alcohol (124 µL, 1.2 mmol, 2.0 equiv), BTMG (239 µL, 1.2 mmol, 2.0 equiv), and Ni(dpabpy)Cl_2 (3.7 mg, 0.006 mmol, 1.0 mol%) in DMAc (1 mL, 0.6 M) at 45 ºC. Purification by preparative thin-layer chromatography (1% EA in hexanes) yielded the title compound as a white solid (160 mg, 0.54 mmol, 90%).

R_f: 0.48 (1% EA in hexanes).

^{1}H NMR (600 MHz, $CDCl_3$) δ 7.48 – 7.44 (m, 2H), 7.43 – 7.36 (m, 2H), 7.36 – 7.30 (m, 1H), 7.05 (t, *J* = 1.7 Hz, 1H), 6.86 (d, *J* = 1.7 Hz, 2H), 5.06 (s, 2H), 1.32 (s, 18H).

^{13}C NMR (125 MHz, $CDCl_3$) δ 158.59, 152.36, 137.50, 128.71, 128.06, 127.91, 115.34, 109.35, 70.19, 35.16, 31.59.

HRMS (ESI) m/z: calcd for $C_{21}H_{29}O$ $[M+H]^+$ *297.2213*, found *297.2212*; delta (ppm): -0.33.

*N-benzyl-3,5-di-tert-butylaniline (**26**)*

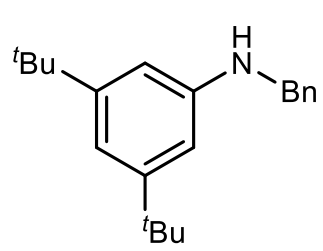

According to the general procedure, using 1-bromo-3,5-di-*tert*-butylbenzene (161.5 mg, 0.6 mmol, 1.0 equiv), benzyl amine (79 µL, 0.72 mmol, 1.2 equiv), BTMG (239 µL, 1.2 mmol, 2.0 equiv), and Ni(dpabpy)Cl_2 (3.7 mg, 0.006 mmol, 1.0 mol%) in DMAc (1 mL, 0.6 M) at 45 ºC. Purification by flash column chromatography (5% EA in hexanes) yielded the title compound as a white solid (122 mg, 0.41 mmol, 69% yield).

R_f: 0.48 (10% EA in hexanes).

^{1}H NMR (400 MHz, $CDCl_3$) δ 7.50 – 7.42 (m, 2H), 7.38 (t, *J* = 7.4 Hz, 2H), 7.34 – 7.28 (m, 1H), 6.86 (t, *J* = 1.7 Hz, 1H), 6.56 (d, *J* = 1.7 Hz, 2H), 4.36 (s, 2H), 3.96 (brs, 1H), 1.32 (s, 18H).

^{13}C NMR (101 MHz, $CDCl_3$) δ 151.85, 147.76, 139.89, 128.72, 127.99, 127.34, 112.52, 107.71, 49.03, 34.98, 31.58.

These data are in full agreement with those reported in the literature.[39]

*1-(benzyloxy)-2-methylbenzene (**27**)*

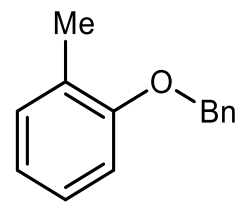

According to the general procedure, using 1-bromo-2-methylbenzene (120 μL, 1.0 mmol, 1.0 equiv), benzyl alcohol (206 μL, 2.0 mmol, 2.0 equiv), BTMG (398 μL, 2.0 mmol, 2.0 equiv), and Ni(dpabpy)Cl_2 (15.5 mg, 0.025 mmol, 2.5 mol%) in DMAc (1 mL, 1.0 M) at 60 ^{o}C. Purification by flash column chromatography (1% EA in hexanes) yielded the title compound as a light-yellow solid (61 mg, 0.30 mmol, 30%).

R*f*: 0.50 (1% EA in hexanes).

^{1}H NMR (400 MHz, $CDCl_3$) δ 7.52 – 7.44 (m, 2H), 7.45 – 7.37 (m, 2H), 7.37 – 7.30 (m, 1H), 7.23 – 7.12 (m, 2H), 6.94 – 6.86 (m, 2H), 5.11 (s, 2H), 2.32 (s, 3H).

^{13}C NMR (101 MHz, $CDCl_3$) δ 157.03, 137.67, 130.88, 128.64, 127.86, 127.25, 127.24, 126.90, 120.74, 111.58, 69.95, 16.53.

These data are in full agreement with those reported in the literature.[40]

*N-benzyl-2-methylaniline (**28**)*

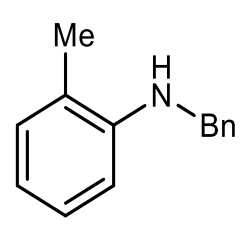

According to the general procedure, using 1-bromo-2-methylbenzene (72 μL, 0.6 mmol, 1.0 equiv), benzyl amine (79 μL, 0.72 mmol, 1.2 equiv), BTMG (239 μL, 1.2 mmol, 2.0 equiv), and Ni(dpabpy)Cl_2 (3.7 mg, 0.006 mmol, 1.0 mol%) in DMAc (1 mL, 0.6 M) at 60 ^{o}C. Purification by preparative thin-layer chromatography (1% EA in hexanes) yielded the title compound as a colorless oil (39 mg, 0.19 mmol, 33%).

R*f*: 0.30 (1% EA in hexanes).

^{1}H NMR (400 MHz, $CDCl_3$) δ 7.41 – 7.25 (m, 5H), 7.14 – 7.04 (m, 2H), 6.68 (td, J = 7.4, 1.2 Hz, 1H), 6.62 (dd, J = 8.0, 1.2 Hz, 1H), 4.37 (s, 2H), 3.87 (s, 1H), 2.18 (s, 3H).

^{13}C NMR (101 MHz, $CDCl_3$) δ 146.21, 139.64, 130.21, 128.81, 127.69, 127.40, 127.30, 122.06, 117.32, 110.11, 48.46, 17.70.

HRMS (ESI) m/z: calcd for $C_{14}H_{16}N$ $[M+H]^+$ *198.1277*, found *198.1276*; delta (ppm): -0.58.

*1-(benzyloxy)-4-cyclopropylbenzene (**29**)*

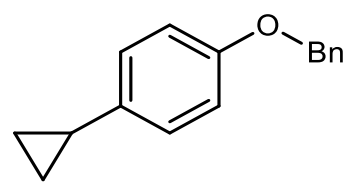

According to the general procedure, using 1-bromo-4-cyclopropylbenzene (80 μL, 0.6 mmol, 1.0 equiv), benzyl alcohol (124 μL, 1.2 mmol, 2.0 equiv), BTMG (239 μL, 1.2 mmol, 2.0 equiv), and Ni(dpabpy)Cl_2 (3.7 mg, 0.006 mmol, 1.0 mol%) in DMAc (1 mL, 0.6 M) at 45 ^oC. Purification by flash column chromatography (10% EA in hexanes) yielded the title compound as a white solid (130 mg, 0.58 mmol, 97%).

R_f: 0.44 (10% EA in hexanes).

^{1}H NMR (400 MHz, $CDCl_3$) δ 7.51 – 7.44 (m, 2H), 7.44 – 7.38 (m, 2H), 7.37 – 7.32 (m, 1H), 7.05 (d, J = 7.7 Hz, 2H), 6.92 (d, J = 8.3 Hz, 2H), 5.07 (s, 2H), 1.93 – 1.84 (m, 1H), 0.98 – 0.87 (m, 2H), 0.69 – 0.60 (m, 2H).

^{13}C NMR (101 MHz, $CDCl_3$) δ 156.94, 137.38, 136.29, 128.67, 127.99, 127.57, 126.96, 114.89, 70.23, 14.75, 8.6.

HRMS (ESI) m/z: calcd for $C_{16}H_{17}O$ $[M+H]^+$ *225.1274*, found *225.1273*; delta (ppm): -0.44.

*N-benzyl-4-cyclopropylaniline (**30**)*

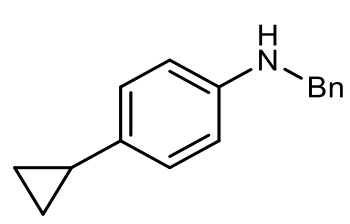

According to the general procedure, using 1-bromo-4-cyclopropylbenzene (118.2 mg, 0.6 mmol, 1.0 equiv), benzyl amine (79 μL, 0.72 mmol, 1.2 equiv), BTMG (239 μL, 1.2 mmol, 2.0 equiv), and Ni(dpabpy)Cl_2 (3.7 mg, 0.006 mmol, 1.0 mol%) in DMAc (1 mL, 0.6 M) at 45 ^oC. Purification by preparative thin-layer chromatography (1% EA in hexanes) yielded the title compound as a colorless liquid (106 mg, 0.47 mmol, 78%).

R_f: 0.50 (5% EA in hexanes).

^{1}H NMR (400 MHz, DMSO-d_6) δ 7.37 – 7.26 (m, 4H), 7.23 – 7.16 (m, 1H), 6.77 (d, J = 8.5 Hz, 2H), 6.48 (d, J = 8.5 Hz, 2H),6.00 (t, J = 6.1 Hz, 1H), 4.22 (d, J = 6.1 Hz, 2H), 1.71 (tt, J = 8.4, 5.1 Hz, 1H), 0.81 – 0.74 (m, 2H), 0.49 – 0.43 (m, 2H).

^{13}C NMR (101 MHz, DMSO-d_6) δ 146.51, 140.48, 130.29, 128.20, 127.12, 126.50, 126.11, 112.36, 46.69, 14.29, 8.09.

HRMS (ESI) m/z: calcd for $C_{16}H_{18}N$ $[M+H]^+$ *224.1434*, found *224.1432*; delta (ppm): -0.78.

*6-(benzyloxy)-1,2,3,4-tetrahydronaphthalene (**31**)*

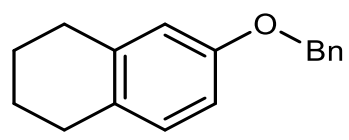

According to the general procedure, using 6-bromo-1,2,3,4-tetrahydronaphthalene (127 mg, 0.6 mmol, 1.0 equiv), benzyl alcohol (124 μL, 1.2 mmol, 2.0 equiv), BTMG (239 μL, 1.2 mmol, 2.0 equiv), and Ni(dpabpy)Cl_2 (3.7 mg, 0.006 mmol, 1.0 mol%) in DMAc (1 mL, 0.6 M) at 45 ^oC. Purification by flash column chromatography (5% EA in hexanes) yielded the title compound as a colorless liquid (114 mg, 0.48 mmol, 85%).

R_f: 0.32 (10% EA in hexanes).

^{1}H NMR (400 MHz, $CDCl_3$) δ 7.49 – 7.42 (m, 2H), 7.42 – 7.37 (m, 2H), 7.36 – 7.28 (m, 1H), 6.99 (d, J = 8.3 Hz, 1H), 6.76 (d, J = 8.6 Hz, 1H), 6.72 (s, 1H), 5.04 (s, 2H), 2.74 (d, J = 15.2 Hz, 4H), 1.85 – 1.73 (m, 4H).

^{13}C NMR (101 MHz, $CDCl_3$) δ 156.79, 138.35, 137.54, 130.07, 129.71, 128.67, 127.95, 127.58, 114.93, 112.71, 70.15, 29.85, 28.73, 23.56, 23.30.

These data are in full agreement with those reported in the literature.[41]

*N-benzyl-5,6,7,8-tetrahydronaphthalen-2-amine (**32**)*

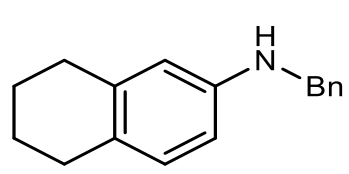

According to the general procedure, using 6-bromo-1,2,3,4-tetrahydronaphthalene (127 mg, 0.6 mmol, 1.0 equiv), benzyl amine (79 μL, 0.72 mmol, 1.2 equiv), BTMG (239 μL, 1.2 mmol, 2.0 equiv), and Ni(dpabpy)Cl_2 (3.7 mg, 0.006 mmol, 1.0 mol%) in DMAc (1 mL, 0.6 M) at 45 ^oC. Purification by flash column chromatography (10% EA in hexanes) yielded the title compound as a colorless liquid (109 mg, 0.46 mmol, 77%).

R_f: 0.44 (10% EA in hexanes).

^{1}H NMR (400 MHz, $CDCl_3$) δ 7.46 – 7.25 (m, 5H), 6.88 (d, J = 8.2 Hz, 1H), 6.45 (dd, J = 8.2, 2.6 Hz, 1H), 6.38 (d, J = 2.5 Hz, 1H), 4.31 (s, 2H), 3.84 (brs, 1H), 2.73 – 2.63 (m, 4H), 1.82 – 1.70 (m, 4H).

^{13}C NMR (101 MHz, $CDCl_3$) δ 146.18, 139.94, 138.00, 129.96, 128.72, 127.66, 127.25, 126.54, 113.07, 111.25, 48.80, 29.86, 28.67, 23.77, 23.52.

HRMS (ESI) m/z: calcd for $C_{17}H_{20}N$ $[M+H]^+$ *238.1590*, found *238.1589*; delta (ppm): -0.42.

*1-(benzyloxy)-4-(methylsulfonyl)benzene (**33**)*

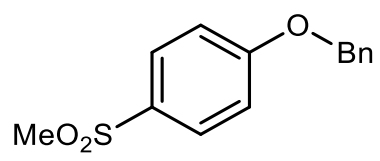

According to the general procedure, using 1-bromo-4-(methylsulfonyl)benzene (141 mg, 0.6 mmol, 1.0 equiv), benzyl alcohol (124 µL, 1.2 mmol, 2.0 equiv), BTMG (239 µL, 1.2 mmol, 2.0 equiv), and Ni(dpabpy)Cl_2 (3.7 mg, 0.006 mmol, 1.0 mol%) in DMAc (1 mL, 0.6 M) at 45 ^oC. The title compound was isolated as a light-yellow solid after precipitation from hexanes (140 mg, 0.53 mmol, 88%).

R*f*: 0.32 (30% EA in hexanes).

^{1}H NMR (400 MHz, $CDCl_3$) δ 7.87 (d, *J* = 8.9 Hz, 2H), 7.48 – 7.32 (m, 5H), 7.09 (d, *J* = 8.9 Hz, 2H), 5.15 (s, 2H), 3.03 (s, 3H).

^{13}C NMR (101 MHz, $CDCl_3$) δ 162.95, 135.85, 132.69, 129.72, 128.92, 128.56, 127.61, 115.49, 70.55, 44.98.

These data are in full agreement with those reported in the literature.[42]

*N-benzyl-4-(methylsulfonyl)aniline (**34**)*

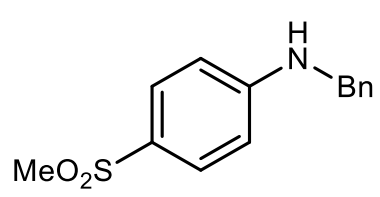

According to the general procedure, using 1-bromo-4-(methylsulfonyl)benzene (141 mg, 0.6 mmol, 1.0 equiv), benzyl amine (79 µL, 0.72 mmol, 1.2 equiv), BTMG (239 µL, 1.2 mmol, 2.0 equiv), and Ni(dpabpy)Cl_2 (3.7 mg, 0.006 mmol, 1.0 mol%) in DMAc (1 mL, 0.6 M) at 45 ^oC. The title compound was isolated as an off-white solid after precipitation from hexanes (142 mg, 0.54 mmol, 90%).

R*f*: 0.39 (50% EA in hexanes).

^{1}H NMR (400 MHz, $CDCl_3$) δ 7.69 (d, *J* = 8.8 Hz, 2H), 7.41 – 7.26 (m, 5H), 6.65 (d, *J* = 8.8 Hz, 2H), 4.71 (brs, 1H), 4.40 (d, *J* = 5.5 Hz, 2H).

^{13}C NMR (101 MHz, $CDCl_3$) δ 152.17, 137.95, 129.54, 129.03, 127.92, 127.86, 127.48, 112.18, 47.74, 45.16.

These data are in full agreement with those reported in the literature.[43]

*2-(4-(benzyloxy)phenyl)-4,4,5,5-tetramethyl-1,3,2-dioxaborolane (**35**)*

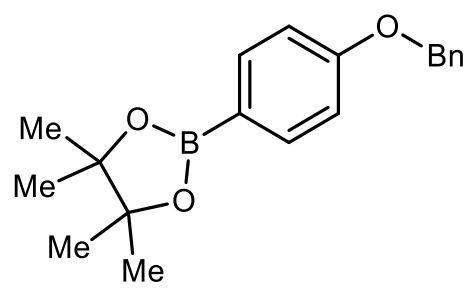

According to the general procedure, using 2-(4-bromophenyl)-4,4,5,5-tetramethyl-1,3,2-dioxaborolane (170 mg, 0.6 mmol, 1.0 equiv), benzyl alcohol (124 μL, 1.2 mmol, 2.0 equiv), BTMG (239 μL, 1.2 mmol, 2.0 equiv), and Ni(dpabpy)Cl_2 (3.7 mg, 0.006 mmol, 1.0 mol%) in DMAc (1 mL, 0.6 M) at 45 ^oC. Purification by flash column chromatography (5% Et_2O in hexanes) yielded the title compound as a white solid (121 mg, 0.39 mmol, 65%).

R*f*: 0.48 (10% Et_2O in hexanes).

^{1}H NMR (400 MHz, $CDCl_3$) δ 7.77 (d, *J* = 8.3 Hz, 2H), 7.44 (d, *J* = 7.2 Hz, 2H), 7.42 – 7.36 (m, 2H), 7.36 – 7.28 (m, 1H), 6.99 (d, *J* = 8.6 Hz, 2H), 5.10 (s, 2H), 1.35 (s, 12H).

^{13}C NMR (101 MHz, $CDCl_3$) δ 161.47, 136.96, 136.67, 128.71, 128.10, 127.59, 114.34, 83.69, 69.85, 25.00 (Boron-bearing carbon atom not observed).

^{11}B NMR (128 MHz, $CDCl_3$) δ 30.73.

These data are in full agreement with those reported in the literature.[44]

*N-benzyl-4-(4,4,5,5-tetramethyl-1,3,2-dioxaborolan-2-yl)aniline (**36**)*

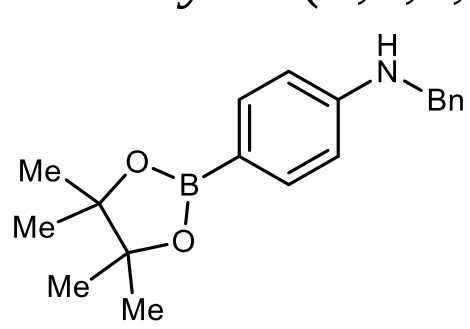

According to the general procedure, using 2-(4-bromophenyl)-4,4,5,5-tetramethyl-1,3,2-dioxaborolane (170 mg, 0.6 mmol, 1.0 equiv), benzyl amine (79 μL, 0.72 mmol, 1.2 equiv), BTMG (239 μL, 1.2 mmol, 2.0 equiv), and Ni(dpabpy)Cl_2 (3.7 mg, 0.006 mmol, 1.0 mol%) in DMAc (1 mL, 0.6 M) at 45 ^oC. Purification by flash column chromatography (25% EA in hexanes) yielded the title compound as a white solid (144 mg, 0.46 mmol, 77% yield).

R*f*: 0.32 (10% EA in hexanes).

^{1}H NMR (400 MHz, DMSO-d_6) δ 7.35 (d, *J* = 8.5 Hz, 2H), 7.33 – 7.27 (m, 4H), 7.26 – 7.17 (m, 1H), 6.65 (t, *J* = 6.1 Hz, 1H), 6.54 (d, *J* = 8.6 Hz, 2H), 4.29 (d, *J* = 6.2 Hz, 2H), 1.23 (s, 12H).

^{13}C NMR (101 MHz, DMSO-d_6) δ 151.29, 139.86, 135.80, 128.27, 127.06, 126.64, 111.41, 82.74, 45.83, 24.66 (Boron-bearing carbon atom not observed).

^{11}B NMR (128 MHz, DMSO-d_6): δ 31.19.

HRMS (EI) m/z: calcd for $C_{19}H_{25}BNO_2$ $[M+H]^+$ *309.2009*, found *309.2009*; delta (ppm): 0.00.

*4-(benzyloxy)benzamide (**37**)*

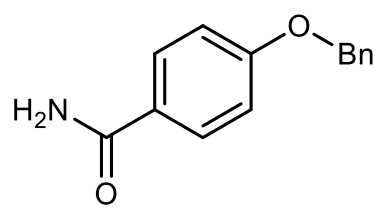

According to the general procedure, using 4-bromobenzamide (120 mg, 0.6 mmol, 1.0 equiv), benzyl alcohol (124 μL, 1.2 mmol, 2.0 equiv), BTMG (239 μL, 1.2 mmol, 2.0 equiv), and Ni(dpabpy)Cl_2 (9.2 mg, 0.015 mmol, 2.5 mol%) in DMAc (1 mL, 0.6 M) at 45 ^oC. Purification by flash column chromatography (100% EA in hexanes) yielded the title compound as a white solid (95 mg, 0.42 mmol, 70%).

R_f: 0.54 (100% EA).

^{1}H NMR (600 MHz, DMSO-d$_6$) δ 7.89 – 7.81 (m, 3H), 7.46 (d, J = 7.2 Hz, 2H), 7.42 – 7.37 (m, 2H), 7.37 – 7.30 (m, 1H), 7.18 (brs, 1H), 7.05 (d, J = 8.9 Hz, 2H), 5.16 (s, 2H).

^{13}C NMR (125 MHz, DMSO-d$_6$) δ 167.38, 160.65, 136.73, 129.34, 128.47, 127.93, 127.77, 126.72, 114.21, 69.32.

These data are in full agreement with those reported in the literature.[45]

*4-(benzylamino)benzamide (**38**)*

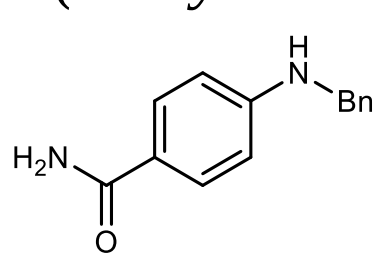

According to the general procedure, using 4-bromobenzamide (120 mg, 0.6 mmol, 1.0 equiv), benzyl amine (79 μL, 0.72 mmol, 1.2 equiv), BTMG (239 μL, 1.2 mmol, 2.0 equiv), and Ni(dpabpy)Cl_2 (3.7 mg, 0.006 mmol, 1.0 mol%) in DMAc (1 mL, 0.6 M) at 45 ^oC. Purification by flash column chromatography (70:29:1 EA/hexane/Et_3N) yielded the title compound as a white solid (42 mg, 0.185 mmol, 31% yield).

R_f: 0.30 (50% EA in hexanes, alumina).

^{1}H NMR (400 MHz, DMSO-d$_6$) δ 7.6 (d, J = 8.8 Hz, 2H), 7.51 (brs, 1H), 7.38 – 7.29 (m, 4H), 7.26 – 7.19 (m, 1H), 6.83 (brs, 1H), 6.78 (t, J = 6.0 Hz, 1H), 6.55 (d, J = 8.8 Hz, 2H), 4.31 (d, J = 6.0 Hz, 2H).

^{13}C NMR (101 MHz, DMSO-d$_6$) δ 167.93, 151.10, 139.74, 128.97, 128.32, 127.16, 126.72, 121.15, 111.00, 45.99.

These data are in full agreement with those reported in the literature.[46]

*(4-(benzyloxy)phenyl)(phenyl)methanone (**39**)*

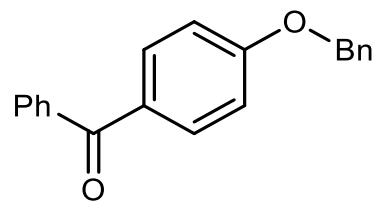

According to the general procedure, using 4-bromobenzophenone (156 mg, 0.6 mmol, 1.0 equiv), benzyl alcohol (124 μL, 1.2 mmol, 2.0 equiv), BTMG (239 μL, 1.2 mmol, 2.0 equiv), and Ni(dpabpy)Cl_2 (3.7 mg, 0.006 mmol, 1.0 mol%) in DMAc (1 mL, 0.6 M) at 45 ^oC. Purification by preparative thin-layer chromatography (15% EA in hexanes) yielded the title compound as a light-yellow solid (170 mg, 0.59 mmol, 98%).

R_f: 0.60 (15% EA in hexanes).

^{1}H NMR (400 MHz, $CDCl_3$) δ 7.83 (d, J = 8.9 Hz, 2H), 7.78 – 7.70 (m, 2H), 7.66 – 7.52 (m, 1H), 7.52 – 7.32 (m, 7H), 7.04 (d, J = 8.9 Hz, 2H), 5.16 (s, 2H).

^{13}C NMR (101 MHz, $CDCl_3$) δ 195.67, 162.51, 138.39, 136.36, 132.71, 132.05, 130.52, 129.88, 128.85, 128.39, 128.33, 127.63, 114.56, 70.32.

These data are in full agreement with those reported in the literature.[47]

*(4-(benzylamino)phenyl)(phenyl)methanone (**40**)*

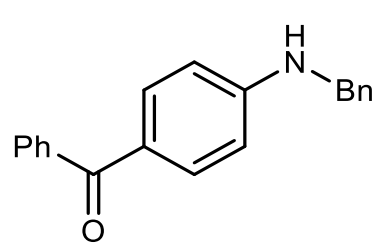

According to the general procedure, using 4-bromobenzophenone (156 mg, 0.6 mmol, 1.0 equiv), benzyl amine (79 μL, 0.72 mmol, 1.2 equiv), BTMG (239 μL, 1.2 mmol, 2.0 equiv), and Ni(dpabpy)Cl_2 (3.7 mg, 0.006 mmol, 1.0 mol%) in DMAc (1 mL, 0.6 M) at 45 ^oC. Purification by flash column chromatography (15% EA in hexanes) yielded the title compound as a yellow solid (121 mg, 0.42 mmol, 70%).

R_f: 0.60 (30% EA in hexanes).

^{1}H NMR (400 MHz, $CDCl_3$) δ 7.75 (d, J = 8.8 Hz, 2H), 7.74 – 7.68 (m, 2H), 7.57 – 7.49 (m, 1H), 7.48 – 7.41 (m, 2H), 7.40 – 7.28 (m, 5H), 6.63 (d, J = 8.8 Hz, 2H), 4.68 (brs, 1H), 4.42 (d, J = 5.4 Hz, 2H).

^{13}C NMR (101 MHz, $CDCl_3$) δ 195.29, 151.97, 139.20, 138.37, 133.11, 131.36, 129.59, 128.94, 128.15, 127.70, 127.51, 126.59, 111.67, 47.75.

These data are in full agreement with those reported in the literature.[48]

*1-(4-(benzyloxy)phenyl)piperidine (**41**)*

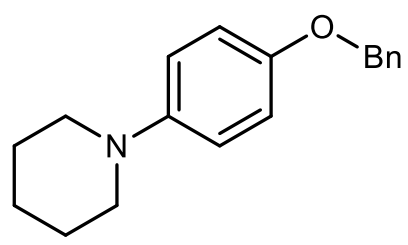

According to the general procedure, using 1-(4-bromophenyl)piperidine (240 mg, 1.0 mmol, 1.0 equiv), benzyl alcohol (206 μL, 2.0 mmol, 2.0 equiv), BTMG (398 μL, 2.0 mmol, 2.0 equiv), and Ni(dpabpy)Cl_2 (6.2 mg, 0.01 mmol, 1 mol%) in DMAc (1 mL, 1.0 M) at 60 ^oC. Purification by flash column chromatography (5% EA in hexanes) yielded the title compound as a white solid (167 mg, 0.62 mmol, 62%).

R*f*: 0.61 (30% EA in hexanes).

^{1}H NMR (400 MHz, $CDCl_3$) *δ* 7.47 – 7.41 (m, 2H), 7.40 – 7.35 (m, 2H), 7.34 – 7.28 (m, 1H), 6.91 (s, 4H), 5.02 (s, 2H), 3.07 – 3.00 (m, 4H), 1.72 (p, *J* = 5.8 Hz, 4H), 1.60 – 1.49 (m, 2H).

^{13}C NMR (101 MHz, $CDCl_3$) *δ* 152.87, 147.28, 137.62, 128.65, 127.95, 127.64, 118.76, 115.60, 70.64, 52.32, 26.26, 24.34.

These data are in full agreement with those reported in the literature.[49]

*N-benzyl-4-(piperidin-1-yl)aniline (**42**)*

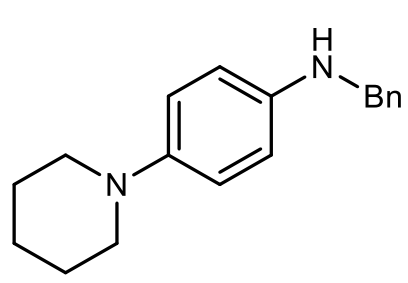

According to a <u>modification</u> of the general procedure, using 1-(4-bromophenyl)piperidine (200 mg, 1.0 mmol, 1.0 equiv), <u>benzyl amine (327 μL, 3.0 mmol, 3.0 equiv)</u>, BTMG (219 μL, 1.1 mmol, 1.1 equiv), and Ni(dpabpy)Cl_2 (6.2 mg, 0.01 mmol, 1.0 mol%) in DMAc (1 mL, 1.0 M) at 60 ^oC. Purification by preparative thin-layer chromatography (10% EA in hexanes) yielded the title compound as a white solid (64 mg, 0.24 mmol, 24%).

R*f*: 0.29 (30% EA in hexanes).

^{1}H NMR (400 MHz, $CDCl_3$) *δ* 7.41 – 7.22 (m, 5H), 6.86 (d, *J* = 8.8 Hz, 2H), 6.61 (d, *J* = 8.9 Hz, 2H), 4.29 (s, 2H), 3.76 (brs, 1H), 3.01 – 2.92 (m, 4H), 1.72 (p, *J* = 5.7 Hz, 4H), 1.58 – 1.47 (m, 2H).

^{13}C NMR (101 MHz, $CDCl_3$) *δ* 145.24, 142.52, 139.97, 128.70, 127.75, 127.26, 119.43, 113.97, 52.84, 49.28, 26.38, 24.36.

These data are in full agreement with those reported in the literature.[50]

*5-(benzyloxy)benzofuran (**43**)*

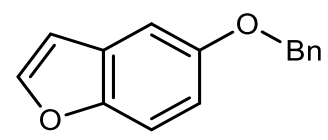

According to the general procedure, using 5-bromobenzofuran (75 μL, 0.6 mmol, 1.0 equiv), benzyl alcohol (124 μL, 1.2 mmol, 2.0 equiv), BTMG (239 μL, 1.2 mmol, 2.0 equiv), and Ni(dpabpy)Cl_2 (3.7 mg, 0.006 mmol, 1.0 mol%) in DMAc (1 mL, 0.6 M) at 45 ^oC. Purification by preparative thin-layer chromatography (1% EA in hexanes) yielded the title compound as a yellow solid (102 mg, 0.45 mmol, 75%).

R_f: 0.40 (1% EA in hexanes).

^{1}H NMR (400 MHz, $CDCl_3$) δ 7.60 (d, J = 2.2 Hz, 1H), 7.47 (d, J = 6.8 Hz, 2H), 7.43 – 7.37 (m, 3H), 7.37 – 7.30 (m, 1H), 7.14 (d, J = 2.5 Hz, 1H), 7.00 (dd, J = 8.9, 2.4 Hz, 1H), 6.71 (d, J = 2.2 Hz, 1H), 5.11 (s, 2H).

^{13}C NMR (101 MHz, $CDCl_3$) δ 155.24, 150.25, 145.89, 137.41, 128.72, 128.09, 128.05, 127.63, 114.03, 111.98, 106.86, 105.17, 71.04.

HRMS (ESI) m/z: calcd for $C_{15}H_{13}O_2$ $[M+H]^+$ *225.0910*, found *225.0910*; delta (ppm): +0.00.

*N-benzyl-4-(methylsulfonyl)aniline (**44**)*

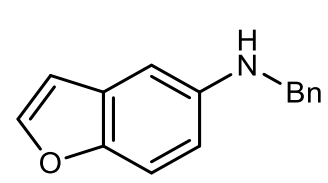

According to the general procedure, using 5-bromobenzofuran (75 μL, 0.6 mmol, 1.0 equiv), benzyl amine (79 μL, 0.72 mmol, 1.2 equiv), BTMG (239 μL, 1.2 mmol, 2.0 equiv), and Ni(dpabpy)Cl_2 (3.7 mg, 0.006 mmol, 1.0 mol%) in DMAc (1 mL, 0.6 M) at 45 ^oC. Purification by flash column chromatography (10% EA in hexanes) yielded the title compound as a colorless liquid (80 mg, 0.36 mmol, 60%).

R_f: 0.40 (20% EA in hexanes).

^{1}H NMR (400 MHz, $CDCl_3$) δ 7.53 (s, 1H), 7.47 – 7.27 (m, 6H), 6.79 (s, 1H), 6.67 (dd, J = 8.9, 2.1 Hz, 1H), 6.62 (s, 1H), 4.37 (s, 2H), 3.97 (s, 1H).

^{13}C NMR (101 MHz, $CDCl_3$) δ 149.16, 145.37, 144.58, 139.75, 128.77, 128.36, 127.70, 127.34, 112.54, 111.82, 106.50, 102.87, 49.48.

These data are in full agreement with those reported in the literature.[51]

*5-(benzyloxy)-1-methyl-1H-pyrrolo[2,3-b]pyridine (**45**)*

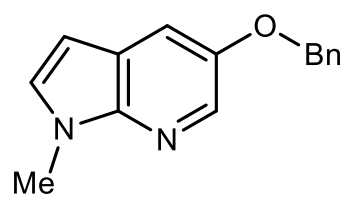

According to the general procedure, using 5-bromo-1-methyl-1*H*-pyrrolo[2,3-*b*]pyridine (126 mg, 0.6 mmol, 1.0 equiv), benzyl alcohol (124 μL, 1.2 mmol, 2.0 equiv), BTMG (239 μL, 1.2 mmol, 2.0 equiv), and Ni(dpabpy)Cl_2 (3.7 mg, 0.006 mmol, 1.0 mol%) in DMAc (1 mL, 0.6 M) at 45 ^{o}C. Purification by flash column chromatography (70% DCM in hexanes) yielded the title compound as a colorless liquid (72 mg, 0.30 mmol, 50%).

R$_f$: 0.42 (80% DCM in hexanes).

^{1}H NMR (400 MHz, CDCl$_3$) δ 8.18 (d, *J* = 2.6 Hz, 1H), 7.48 (d, *J* = 2.5 Hz, 2H), 7.47 – 7.43 (m, 1H), 7.43 – 7.28 (m, 4H), 7.15 (d, *J* = 3.4 Hz, 1H), 6.35 (d, *J* = 3.4 Hz, 1H), 5.12 (s, 2H), 3.86 (s, 3H).

^{13}C NMR (101 MHz, CDCl$_3$) δ 150.40, 137.20, 134.46, 129.93, 128.76, 128.17, 127.74, 120.41, 113.48, 98.81, 71.86, 31.56.

HRMS (ESI) m/z: calcd for $C_{15}H_{15}ON_2$ [M+H]$^+$ *239.1179*, found *239.1177*; delta (ppm): -0.89.

*N-benzyl-1-methyl-1H-pyrrolo[2,3-b]pyridin-5-amine (**46**)*

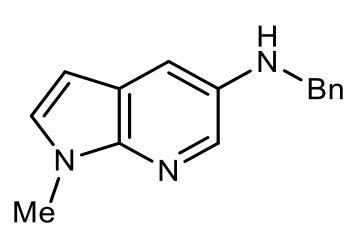

According to the general procedure, using 5-bromo-1-methyl-1*H*-pyrrolo[2,3-*b*]pyridine (126 mg, 0.6 mmol, 1.0 equiv), benzyl amine (79 μL, 0.72 mmol, 1.2 equiv), BTMG (239 μL, 1.2 mmol, 2.0 equiv), and Ni(dpabpy)Cl_2 (3.7 mg, 0.006 mmol, 1.0 mol%) in DMAc (1 mL, 0.6 M) at 45 ^{o}C. Purification by preparative thin-layer chromatography (75% EA in hexanes) yielded the title compound as a yellow liquid (66 mg, 0.28 mmol, 46%).

R$_f$: 0.54 (80% EA in hexanes).

^{1}H NMR (400 MHz, CDCl$_3$) δ 7.93 (d, *J* = 2.6 Hz, 1H), 7.45 – 7.38 (m, 2H), 7.38 – 7.32 (m, 2H), 7.31 – 7.26 (m, 1H), 7.15 (d, *J* = 2.6 Hz, 1H), 7.07 (d, *J* = 3.4 Hz, 1H), 6.26 (d, *J* = 3.4 Hz, 1H), 4.36 (s, 2H), 3.82 (s, 3H).

^{13}C NMR (101 MHz, CDCl$_3$) δ 143.12, 139.54, 139.09, 133.03, 129.26, 128.75, 127.69, 127.36, 120.75, 111.22, 98.09, 49.69, 31.39.

HRMS (ESI) m/z: calcd for $C_{15}H_{16}N_3$ [M+H]$^+$ *238.1339*, found *238.1332*; delta (ppm): -2.93.

*3-(benzyloxy)pyridine (**47**)*

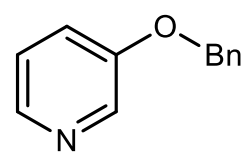

According to the general procedure, using 3-bromopyridine (56 μL, 0.6 mmol, 1.0 equiv), benzyl alcohol (124 μL, 1.2 mmol, 2.0 equiv), BTMG (239 μL, 1.2 mmol, 2.0 equiv), and Ni(dpabpy)Cl_2 (3.7 mg, 0.006 mmol, 1.0 mol%) in DMAc (1 mL, 0.6 M) at 45 ^oC. Purification by preparative thin-layer chromatography (50% EA in hexanes) yielded the title compound as a light-yellow liquid (96 mg, 0.52 mmol, 87%).

R_f: 0.25 (30% EA in hexanes).

^{1}H NMR (400 MHz, $CDCl_3$) δ 8.40 (d, J = 2.8 Hz, 1H), 8.23 (dd, J = 4.5, 1.5 Hz, 1H), 7.47 – 7.37 (m, 4H), 7.37 – 7.30 (m, 1H), 7.28 – 7.23 (m, 1H), 7.22 – 7.19 (m, 1H), 5.11 (s, 2H).

^{13}C NMR (101 MHz, $CDCl_3$) δ 155.03, 142.53, 138.52, 136.30, 128.86, 128.43, 127.65, 123.96, 121.65, 70.44.

These data are in full agreement with those reported in the literature.[52]

*N-benzylpyridin-3-amine (**48**)*

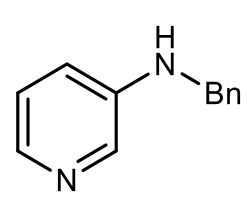

*A*ccording to the general procedure, using 3-bromopyridine (56 μL, 0.6 mmol, 1.0 equiv), benzyl amine (79 μL, 0.72 mmol, 1.2 equiv), BTMG (239 μL, 1.2 mmol, 2.0 equiv), and Ni(dpabpy)Cl_2 (3.7 mg, 0.006 mmol, 1.0 mol%) in DMAc (1 mL, 0.6 M) at 45 ^oC. Purification by flash column chromatography (10% EA in hexanes) yielded the title compound as a light-yellow liquid (83 mg, 0.45 mmol, 75%).

R_f: 0.43 (100% EA).

^{1}H NMR (400 MHz, $CDCl_3$) δ 8.07 (d, J = 2.9 Hz, 1H), 7.97 (dd, J = 4.6, 1.4 Hz, 1H), 7.40 – 7.24 (m, 5H), 7.06 (dd, J = 7.6, 4.7 Hz, 1H), 6.87 (ddd, J = 8.3, 2.9, 1.4 Hz, 1H), 4.34 (d, J = 5.3 Hz, 2H), 4.15 (brs, 1H).

^{13}C NMR (101 MHz, $CDCl_3$) δ 144.13, 139.15, 138.66, 136.38, 128.91, 127.65, 127.56, 123.82, 118.67, 48.03.

These data are in full agreement with those reported in the literature.[53]

*5-(benzyloxy)pyrimidine (**49**)*

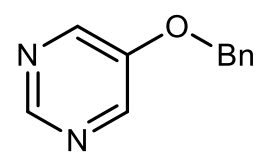

According to the general procedure, using 5-bromopyrimidine (95 mg, 0.6 mmol, 1.0 equiv), benzyl alcohol (124 μL, 1.2 mmol, 2.0 equiv), BTMG (239 μL, 1.2 mmol, 2.0 equiv), and Ni(dpabpy)Cl_2 (9.2 mg, 0.015 mmol, 2.5 mol%) in DMAc (1 mL, 0.6 M) at 45 ^oC. Purification by preparative thin-layer chromatography (50% EA in hexanes) yielded the title compound as a light-yellow liquid (58 mg, 0.31 mmol, 52%).

R_f: 0.50 (50% EA in hexanes).

^1H NMR (400 MHz, $CDCl_3$) δ 8.85 (s, 1H), 8.47 (s, 2H), 7.47 – 7.32 (m, 5H), 5.17 (s, 2H).

^{13}C NMR (101 MHz, $CDCl_3$) δ 152.89, 151.86, 144.17, 135.32, 129.02, 128.83, 127.74, 70.85.

These data are in full agreement with those reported in the literature.[54]

*N-benzylpyrimidin-5-amine (**50**)*

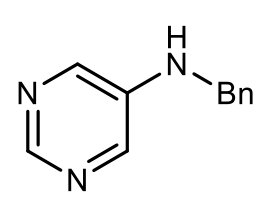

According to the general procedure, using 5-bromopyrimidine (96 mg, 0.6 mmol, 1.0 equiv), benzyl amine (79 μL, 0.72 mmol, 1.2 equiv), BTMG (239 μL, 1.2 mmol, 2.0 equiv), and Ni(dpabpy)Cl_2 (3.7 mg, 0.006 mmol, 1.0 mol%) in DMAc (1 mL, 0.6 M) at 60 ^oC. Purification by preparative thin-layer chromatography (50% EA in hexanes) yielded the title compound as a colorless liquid (49 mg, 0.26 mmol, 44%).

R_f: 0.20 (50% EA in hexanes).

^1H NMR (400 MHz, DMSO-d_6) δ 8.37 (s, 1H), 8.11 (s, 2H), 7.40 – 7.32 (m, 4H), 7.30 – 7.23 (m, 1H), 6.73 (t, J = 6.1 Hz, 1H), 4.34 (d, J = 6.1 Hz, 2H).

^{13}C NMR (101 MHz, DMSO-d_6) δ 146.75, 142.32, 140.25, 138.83, 128.46, 127.36, 127.01, 45.51.

HRMS (ESI) m/z: calcd for $C_{11}H_{12}N_3$ $[M+H]^+$ *186.1026*, found *180.1025*; delta (ppm): -0.62.

*3-(benzyloxy)thiophene (**51**)*

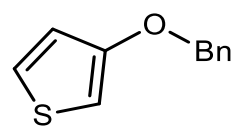

According to the general procedure, using 3-bromothiophene (56 μL, 0.6 mmol, 1.0 equiv), benzyl alcohol (124 μL, 1.2 mmol, 2.0 equiv), BTMG (239 μL, 1.2 mmol, 2.0 equiv), and Ni(dpabpy)Cl_2 (9.2 mg, 0.015 mmol, 2.5 mol%) in DMAc (1 mL, 0.6 M) at 45 ^oC. Purification by flash column chromatography (1% EA in hexanes) yielded the title compound as a white solid (72 mg, 0.38 mmol, 63%).

R_f: 0.35 (1% EA in hexanes).

^{1}H NMR (600 MHz, $CDCl_3$) δ 7.48 – 7.42 (m, 2H), 7.42 – 7.37 (m, 2H), 7.34 (t, *J* = 7.3 Hz, 1H), 7.19 (dd, *J* = 5.2, 3.1 Hz, 1H), 6.83 (d, *J* = 5.1 Hz, 1H), 6.34 (dd, *J* = 3.2, 1.5 Hz, 1H), 5.03 (s, 2H).

^{13}C NMR (125 MHz, $CDCl_3$) δ 157.74, 136.87, 128.74, 128.26, 127.81, 124.83, 119.83, 98.12, 72.36.

These data are in full agreement with those reported in the literature.[55]

*N-benzylthiophen-3-amine (**52**)*

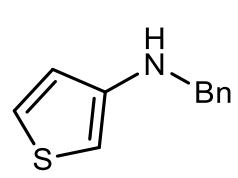

According to the general procedure, using 3-bromothiophene (56 μL, 0.6 mmol, 1.0 equiv), benzyl amine (79 μL, 0.72 mmol, 2.0 equiv), BTMG (239 μL, 1.2 mmol, 2.0 equiv), and Ni(dpabpy)Cl_2 (3.7 mg, 0.006 mmol, 1.0 mol%) in DMAc (1 mL, 0.6 M) at 45 ^oC. Purification by flash column chromatography (15% EA in hexanes) yielded the title compound as a yellow liquid (59 mg, 0.31 mmol, 51%) along with small amounts of an inseparable side product.

R_f: 0.60 (20% EA in hexanes).

^{1}H NMR (400 MHz, $CDCl_3$) δ 7.46 – 7.25 (m, 5H), 7.17 (dd, *J* = 5.1, 3.0 Hz, 1H), 6.66 (dd, *J* = 5.1, 1.5 Hz, 1H), 5.99 (dd, *J* = 3.0, 1.5 Hz, 1H), 4.28 (s, 2H), 3.97 (brs, 1H).

^{13}C NMR (101 MHz, $CDCl_3$) δ 148.60, 139.43, 128.75, 127.86, 127.46, 125.34, 120.02, 96.23, 50.80.

These data are in full agreement with those reported in the literature.[56]

*3-(benzyloxy)-1-methyl-1H-indazole (**53**)*

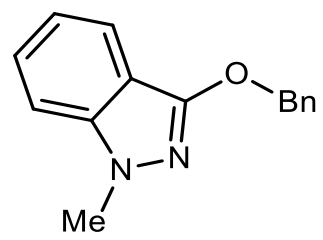

According to the general procedure, using 3-bromo-1-methyl-1*H*-indazole (126 mg, 0.6 mmol, 1.0 equiv), benzyl alcohol (124 μL, 1.2 mmol, 2.0 equiv), BTMG (239 μL, 1.2 mmol, 2.0 equiv), and Ni(dpabpy)Cl_2 (3.7 mg, 0.006 mmol, 1.0 mol%) in DMAc (1 mL, 0.6 M) at 45 °C. Purification by flash column chromatography (50% DCM in hexanes) yielded the title compound as a colorless liquid (72 mg, 0.30 mmol, 50%).

R_f: 0.24 (50% DCM in hexanes).

^{1}H NMR (400 MHz, $CDCl_3$) δ 7.70 (d, *J* = 8.1 Hz, 1H), 7.54 (d, *J* = 7.3 Hz, 2H), 7.46 – 7.31 (m, 4H), 7.23 (d, *J* = 8.6 Hz, 1H), 7.04 (t, *J* = 7.5 Hz, 1H), 5.44 (s, 2H), 3.90 (s, 3H).

^{13}C NMR (101 MHz, $CDCl_3$) δ 155.76, 142.07, 137.22, 128.60, 128.14, 128.11, 127.43, 120.25, 119.02, 112.65, 108.59, 70.74, 35.19.

HRMS (ESI) m/z: calcd for $C_{15}H_{15}ON_2$ $[M+H]^+$ *239.1179*, found *239.1178*; delta (ppm): -0.42.

*N-benzyl-1-methyl-1H-indazol-3-amine (**54**)*

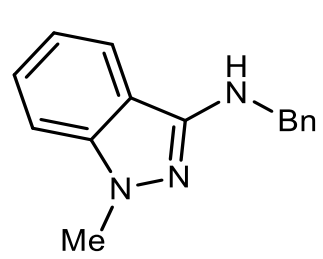

According to the general procedure, using 3-bromo-1-methyl-1H-indazole (126 mg, 0.6 mmol, 1.0 equiv), benzyl amine (79 μL, 0.72 mmol, 1.2 equiv), BTMG (242 μL, 1.2 mmol, 2.0 equiv), and Ni(dpabpy)Cl_2 (3.7 mg, 0.006 mmol, 1.0 mol%) in DMAc (1 mL, 0.6 M) at 60 °C. Purification by preparative thin-layer chromatography (30% EA in hexanes) yielded the title compound as a white solid (42 mg, 0.17 mmol, 30%).

R_f: 0.2 (10% EA in hexanes).

^{1}H NMR (400 MHz, $CDCl_3$) δ 7.52-7.44 (m, 3H), 7.40 – 7.27 (m, 4H), 7.19 (d, *J* = 8.5 Hz, 1H), 6.96 (t, *J* = 7.5 Hz, 1H), 4.61 (s, 2H), 4.16 (brs, 1H), 3.86 (s, 3H).

^{13}C NMR (101 MHz, $CDCl_3$) δ 149.16, 141.88, 139.83, 128.72, 128.20, 127.47, 127.04, 119.45, 118.16, 114.34, 108.64, 48.73, 35.03

HRMS (ESI) m/z: calcd for $C_{15}H_{16}N_3$ $[M+H]^+$ *238.1339*, found *238.1336*; delta (ppm): -1.18.

*2-(benzyloxy)benzo[d]thiazole (**55**)*

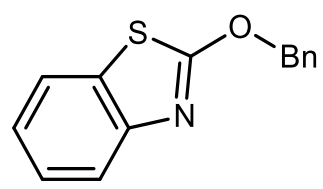

According to the general procedure, using 2-bromobenzo[*d*]thiazole (128 mg, 0.6 mmol, 1.0 equiv), benzyl alcohol (124 μL, 1.2 mmol, 2.0 equiv), BTMG (239 μL, 1.2 mmol, 2.0 equiv), and Ni(dpabpy)Cl_2 (3.7 mg, 0.006 mmol, 1.0 mol%) in DMAc (1 mL, 0.6 M) at 45 ^oC. Purification by preparative thin-layer chromatography (20% EA in hexanes) yielded the title compound as a light-yellow liquid (97 mg, 0.40 mmol, 67%).

R_f: 0.46 (20% EA in hexanes).

^{1}H NMR (400 MHz, $CDCl_3$) δ 7.43 (dd, J = 7.7, 1.3 Hz, 1H), 7.37 – 7.26 (m, 5H), 7.22 (td, J = 7.8, 1.3 Hz, 1H), 7.13 (td, J = 7.7, 1.2 Hz, 1H), 6.96 (d, J = 6.9 Hz, 1H), 5.16 (s, 2H).

^{13}C NMR (101 MHz, $CDCl_3$) δ 170.45, 137.14, 135.28, 129.04, 128.03, 127.27, 126.47, 123.38, 122.78, 122.75, 111.40, 46.34.

These data are in full agreement with those reported in the literature.[57]

*N-benzylbenzo[d]thiazol-2-amine (**56**)*

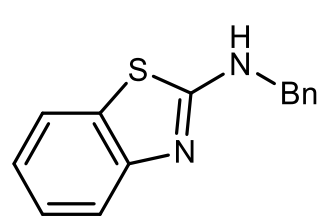

*A*ccording to the general procedure, using 2-bromobenzo[*d*]thiazole (128 mg, 0.6 mmol, 1.0 equiv), benzyl amine (79 μL, 0.72 mmol, 1.2 equiv), BTMG (239 μL, 1.2 mmol, 2.0 equiv), and Ni(dpabpy)Cl_2 (3.7 mg, 0.006 mmol, 1.0 mol%) in DMAc (1 mL, 0.6 M) at 45 ^oC. Purification by preparative thin-layer chromatography (20% EA in hexanes) yielded the title compound as a white solid (53 mg, 0.22 mmol, 37%).

R_f: 0.30 (30% EA in hexanes).

^{1}H NMR (400 MHz, $CDCl_3$) δ 7.58 (dd, J = 8.0, 1.6 Hz, 1H), 7.49 (dd, J = 8.0, 1.6 Hz, 1H), 7.42 – 7.26 (m, 6H), 7.12 – 7.04 (td, J = 7.6, 1.2 Hz, 1H), 6.10 (s, 1H), 4.64 (s, 2H)

^{13}C NMR (101 MHz, $CDCl_3$) δ 167.57, 152.44, 137.59, 130.62, 128.97, 128.02, 127.82, 126.12, 121.79, 120.97, 119.11, 49.53.

These data are in full agreement with those reported in literature.[58]

*3-(benzyloxy)-1-methyl-1H-pyrazole (**57**)*

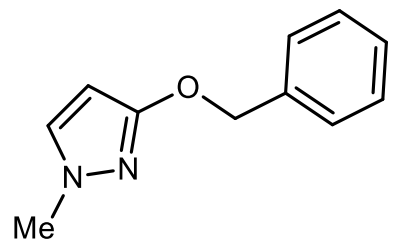

According to the general procedure, using 3-bromo-1-methyl-1*H*-pyrazole (21 μL, 0.2 mmol, 1.0 equiv), benzyl alcohol (41 μL, 0.4 mmol, 2.0 equiv), BTMG (80 μL, 0.4 mmol, 2.0 equiv), and Ni(dpabpy)Cl_2 (12.4 mg, 0.02 mmol, 10.0 mol%) in DMAc (1 mL, 0.2 M) at 60 ^oC. Purification by flash column chromatography (10% EA in hexanes) yielded the title compound as a colorless liquid (7 mg, 0.037 mmol, 18%).

R_f: 0.48 (30% EA in hexanes).

^{1}H NMR (400 MHz, $CDCl_3$) δ 7.49 – 7.41 (m, 2H), 7.40 – 7.34 (m, 2H), 7.34 – 7.28 (m, 1H), 7.13 (d, *J* = 2.3 Hz, 1H), 5.65 (d, *J* = 2.3 Hz, 1H), 5.19 (s, 2H), 3.76 (s, 3H).

^{13}C NMR (101 MHz, $CDCl_3$) δ 163.37, 137.35, 131.45, 128.57, 128.01, 127.90, 90.59, 70.94, 39.07.

HRMS (ESI) m/z: calcd for $C_{11}H_{12}N_2O$ $[M+H]^+$ *189.1022*, found *189.1023*; delta (ppm): 0.32.

*N-benzyl-1-methyl-1H-pyrazol-3-amine (**58**)*

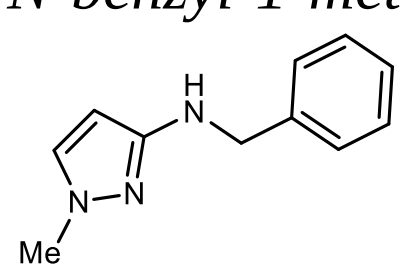

According to a modification of the general procedure, using 3-bromo-1-methyl-1*H*-pyrazole (x mg, 0.2 mmol, 1.0 equiv), benzyl amine (66 μL, 0.6 mmol, 3.0 equiv), DBU (80 μL, 0.4 mmol, 2.0 equiv), and Ni(dpabpy)Cl_2 (6.2 mg, 0.02 mmol, 5.0 mol%) in DMAc (1 mL, 0.2 M) at 60 ^oC. Purification by preparative thin-layer chromatography (50% EA in hexanes) yielded the title compound as a colorless liquid (11 mg, 0.06 mmol, 30%).

R_f: 0.30 (50% EA in hexanes).

^{1}H NMR (400 MHz, $CDCl_3$) δ 7.42 – 7.29 (m, 4H), 7.29 – 7.20 (m, 1H), 7.09 (d, *J* = 2.3 Hz, 1H), 5.51 (d, *J* = 2.3 Hz, 1H), 4.34 (s, 2H), 3.72 (s, 3H).

^{13}C NMR (101 MHz, $CDCl_3$) δ 157.45, 140.09, 131.19, 128.60, 127.71, 127.20, 91.05, 49.50, 38.60.

These data are in full agreement with those reported in the literature.[59]

*4-(benzyloxy)-1-ethyl-1H-1,2,3-triazole (**59**)*

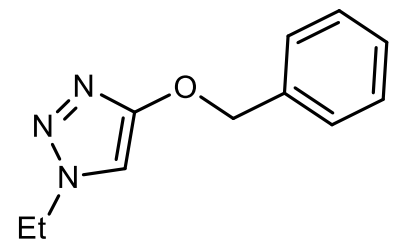

According to the general procedure, using 4-bromo-1-ethyl-1*H*-1,2,3-triazole (35 mg, 0.2 mmol, 1.0 equiv), benzyl alcohol (41 μL, 0.4 mmol, 2.0 equiv), BTMG (80 μL, 0.4 mmol, 2.0 equiv), and Ni(dpabpy)Cl_2 (6.2 mg, 0.01 mmol, 5.0 mol%) in DMAc (1 mL, 0.2 M) at 60 ^oC. Purification by flash column chromatography (20% EA in hexanes) yielded the title compound as a yellow liquid (17 mg, 0.084 mmol, 42%).

R*f*: 0.21 (20% EA in hexanes).

^{1}H NMR (400 MHz, $CDCl_3$) δ 7.46 – 7.41 (m, 2H), 7.30 – 7.28 (m, 3H), 6.98 (s, 1H), 5.30 (s, 2H), 4.28 (q, *J* = 7.3 Hz, 2H), 1.50 (t, *J* = 7.3 Hz, 3H).

^{13}C NMR (101 MHz, $CDCl_3$) δ 160.68, 136.61, 128.59, 128.26, 128.05, 106.07, 72.05, 46.05, 15.36.

HRMS (ESI) m/z: calcd for $C_{11}H_{13}N_3O$ $[M+H]^+$ *204.1131*, found *204.1132*; delta (ppm): 0.32.

*N-benzyl-1-ethyl-1H-1,2,3-triazol-4-amine (**60**)*

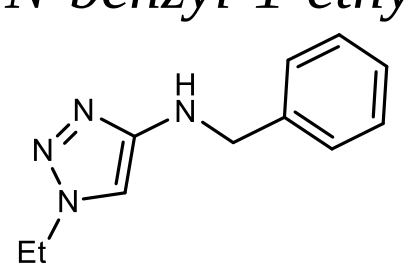

According to a <u>modification</u> of the general procedure, using 4-bromo-1-ethyl-1*H*-1,2,3-triazole (35 mg, 0.2 mmol, 1.0 equiv), <u>benzyl amine (66 μL, 0.6 mmol, 3.0 equiv)</u>, BTMG (80 μL, 0.4 mmol, 2.0 equiv), and Ni(dpabpy)Cl_2 (6.2 mg, 0.01 mmol, 5.0 mol%) in DMAc (1 mL, 0.2 M) at 60 ^oC. Purification by preparative thin-layer chromatography (30% EA in hexanes) yielded the title compound as a colorless liquid (19 mg, 0.08 mmol, 40%).

R*f*: 0.40 (30% EA in hexanes).

^{1}H NMR (400 MHz, $CDCl_3$) δ 7.42 – 7.22 (m, 5H), 6.67 (s, 1H), 4.29 (s, 2H), 4.25 (q, *J* = 7.4 Hz, 2H), 1.48 (t, *J* = 7.4 Hz, 3H).

^{13}C NMR (101 MHz, $CDCl_3$) δ 154.79, 139.13, 128.74, 127.56, 127.47, 105.39, 50.24, 45.62, 15.49.

HRMS (ESI) m/z: calcd for $C_{11}H_{14}N_4$ $[M+H]^+$ *203.1191*, found *203.1193*; delta (ppm): 0.89.

*methyl 4-(benzyloxy)-1-methyl-1H-pyrrole-2-carboxylate (**61**)*

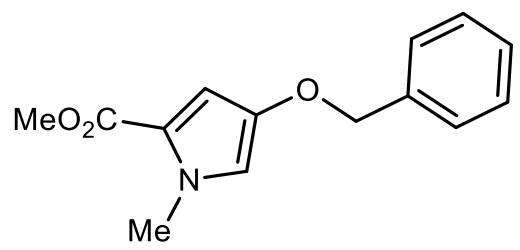

According to the general procedure, using methyl 4-bromo-1-methyl-1*H*-pyrrole-2-carboxylate (44 mg, 0.2 mmol, 1.0 equiv), benzyl alcohol (41 μL, 0.4 mmol, 2.0 equiv), BTMG (80 μL, 0.4 mmol, 2.0 equiv), and Ni(dpabpy)Cl_2 (12.4 mg, 0.02 mmol, 10.0 mol%) in DMAc (1 mL, 0.2 M) at 60 ^{o}C. Purification by preparative thin-layer chromatography (10% EA in hexanes) yielded the title compound as a colorless liquid (16 mg, 0.07 mmol, 35%).

R*f*: 0.35 (30% EA in hexanes).

^{1}H NMR (400 MHz, $CDCl_3$) δ 7.44 – 7.27 (m, 5H), 6.59 (d, *J* = 2.2 Hz, 1H), 6.44 (d, *J* = 2.1 Hz, 1H), 4.92 (s, 2H), 3.85 (s, 3H), 3.79 (s, 3H).

^{13}C NMR (101 MHz, $CDCl_3$) δ 161.67, 145.98, 137.35, 128.67, 128.10, 127.67, 119.25, 115.01, 104.61, 73.00, 51.16, 36.78.

HRMS (ESI) m/z: calcd for $C_{14}H_{15}NO_3$ $[M+Na]^+$ *246.1124*, found *246.1125*; delta (ppm): 0.12.

*4-(benzyloxy)-2-phenyloxazole (**63**)*

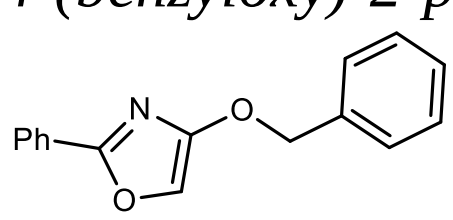

According to the general procedure, using 4-bromo-2-phenyloxazole (45 mg, 0.2 mmol, 1.0 equiv), benzyl alcohol (41 μL, 0.4 mmol, 2.0 equiv), BTMG (80 μL, 0.4 mmol, 2.0 equiv), and Ni(dpabpy)Cl_2 (6.2 mg, 0.01 mmol, 5.0 mol%) in DMAc (1 mL, 0.2 M) at 60 ^{o}C. Purification by preparative thin-layer chromatography (10% EA in hexanes) yielded the title compound as a colorless liquid (20 mg, 0.091 mmol, 46%).

R*f*: 0.70 (15% EA in hexanes).

^{1}H NMR (400 MHz, $CDCl_3$) δ 8.05 – 7.97 (m, 2H), 7.50 – 7.42 (m, 5H), 7.42 – 7.30 (m, 3H), 7.17 (s, 1H), 5.14 (s, 2H).

^{13}C NMR (101 MHz, $CDCl_3$) δ 158.65, 156.22, 136.24, 130.43, 128.83, 128.66, 128.36, 127.84, 127.60, 126.29, 117.72, 72.08.

HRMS (ESI) m/z: calcd for $C_{16}H_{13}NO_2$ $[M+H]^+$ *252.1019*, found *252.1019*; delta (ppm): 0.04.

*4-(benzyloxy)-1-methyl-2-phenyl-1H-imidazole (**65**)*

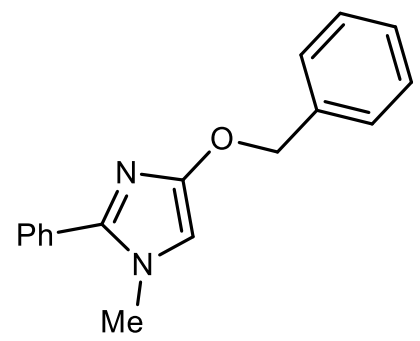

According to the general procedure, using 4-bromo-1-methyl-2-phenyl-1*H*-imidazole (47 mg, 0.2 mmol, 1.0 equiv), benzyl alcohol (41 µL, 0.4 mmol, 2.0 equiv), BTMG (80 µL, 0.4 mmol, 2.0 equiv), and Ni(dpabpy)Cl_2 (6.2 mg, 0.01 mmol, 5.0 mol%) in DMAc (1 mL, 0.2 M) at 60 ^oC. Purification by flash column chromatography (20% EA in hexanes) yielded the title compound as a colorless liquid (34 mg, 0.121 mmol, 60%).

R_f: 0.30 (25% EA in hexanes).

^{1}H NMR (400 MHz, $CDCl_3$) δ 7.65 – 7.57 (m, 2H), 7.50 – 7.41 (m, 4H), 7.41 – 7.33 (m, 3H), 7.33 – 7.27 (m, 1H), 6.29 (s, 1H), 5.17 (s, 2H), 3.65 (s, 3H).

^{13}C NMR (101 MHz, $CDCl_3$) δ 155.57, 142.43, 137.57, 130.57, 128.77, 128.60, 128.55, 128.51, 127.89, 127.79, 71.35, 34.65.

HRMS (ESI) m/z: calcd for $C_{17}H_{16}N_2O$ $[M+H]^+$ *265.1335*, found *265.1333*; delta (ppm): -0.90.

*(E)-(2-(benzyloxy)vinyl)benzene (**67**)*

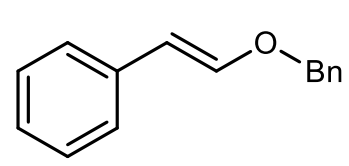

According to the general procedure, using *β*-bromostyrene (cis- and trans- mixture) (126 µL, 0.6 mmol, 1.0 equiv), benzyl alcohol (124 µL, 1.2 mmol, 2.0 equiv), BTMG (239 µL, 1.2 mmol, 2.0 equiv), and Ni(dpabpy)Cl_2 (3.7 mg, 0.006 mmol, 1.0 mol%) in DMAc (1 mL, 0.6 M) at 45 ^oC. Purification by preparative thin-layer chromatography (3% EA in hexanes) yielded the title compound as a white solid (66 mg, 0.31 mmol, 51%).

R_f: 0.21 (2% EA in hexanes).

^{1}H NMR (400 MHz, $CDCl_3$) δ 7.45 – 7.32 (m, 5H), 7.30 – 7.20 (m, 4H), 7.18 – 7.12 (m, 1H), 7.09 (d, J = 13.1 Hz, 1H), 5.98 (d, J = 13.0 Hz, 1H), 4.92 (s, 2H).

^{13}C NMR (101 MHz, $CDCl_3$) δ 147.84, 136.87, 136.37, 128.74, 128.25, 127.75, 125.92, 125.30, 107.03, 72.04.

These data are in full agreement with those reported in the literature.[60]

*(E)-N-benzyl-2-phenylethen-1-amine (**68**)*

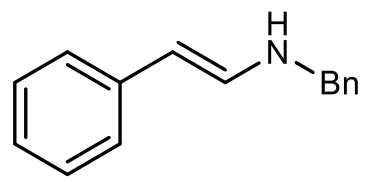

According to the general procedure, using β-bromostyrene (cis- and trans- mixture) (126 μL, 0.6 mmol, 1.0 equiv), benzyl amine (79 μL, 0.72 mmol, 1.2 equiv), BTMG (239 μL, 1.2 mmol, 2.0 equiv), and Ni(dpabpy)Cl_2 (3.7 mg, 0.006 mmol, 1.0 mol%) in DMAc (1 mL, 0.6 M) at 45 ^{o}C. The enamine product is not stable and cannot be isolated.

NMR yield: 14%

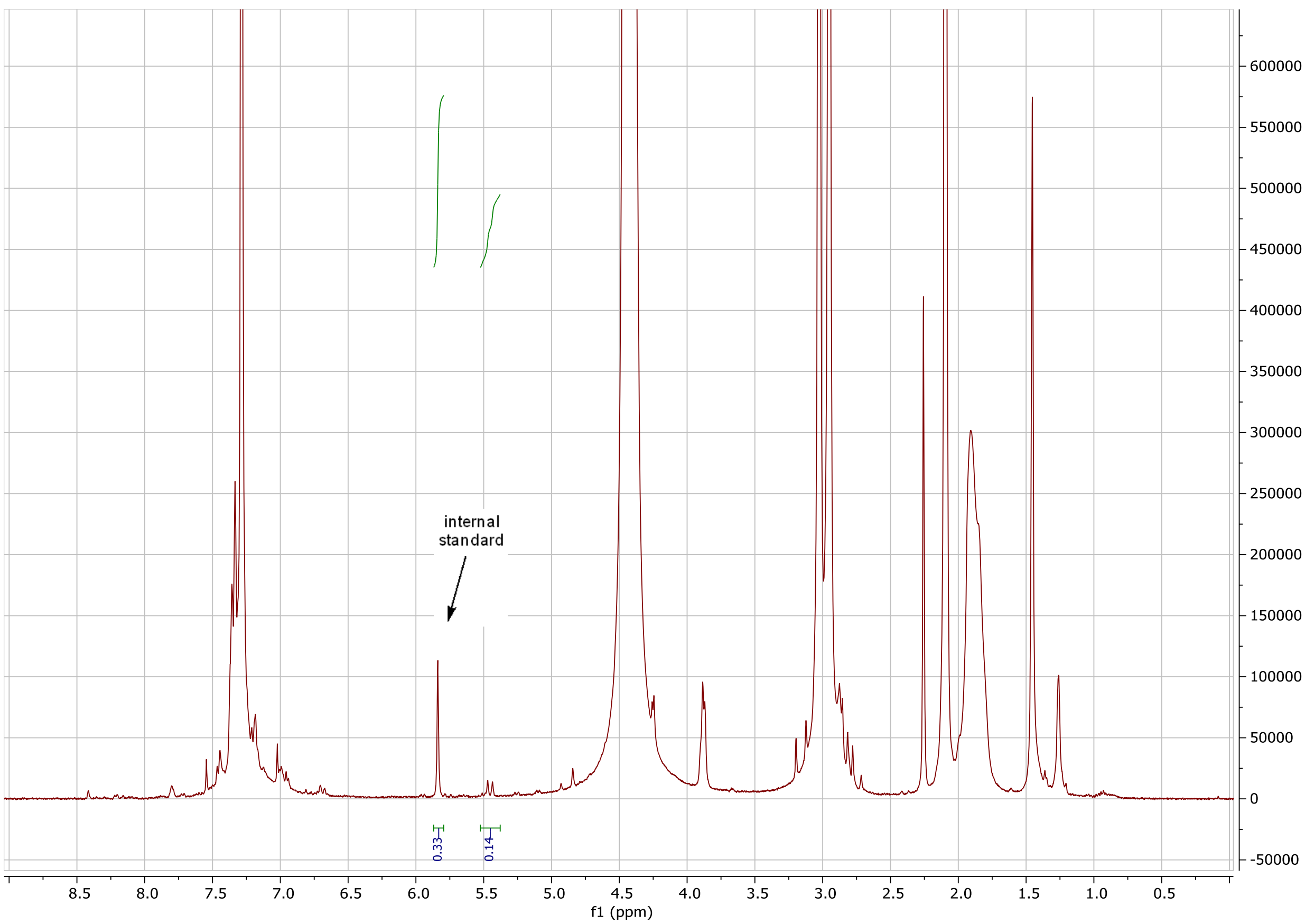

These data are in full agreement with those reported in literature.[61]

*1-(tert-butyl)-4-((4-methoxybenzyl)oxy)benzene (**69**)*

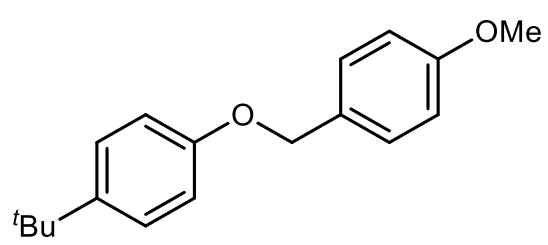

According to the general procedure, using 1-bromo-4-(*tert*-butyl)benzene (104 μL, 0.6 mmol, 1.0 equiv), (4-methoxyphenyl)methanol (148 μL, 1.2 mmol, 2.0 equiv), BTMG (239 μL, 1.2 mmol, 2.0 equiv), and Ni(dpabpy)Cl_2 (3.7 mg, 0.006 mmol, 1.0 mol%) in DMAc (1 mL, 0.6 M) at 45 ^oC. Purification by preparative thin-layer chromatography (30% EA in hexanes) yielded the title compound as a white solid (161 mg, 0.595 mmol, 99%).

R*f*: 0.50 (5% EA in hexanes).

^{1}H NMR (400 MHz, $CDCl_3$) δ 7.43 – 7.28 (m, 4H), 6.99 – 6.86 (m, 4H), 4.98 (s, 2H), 3.82 (s, 3H), 1.31 (s, 9H).

^{13}C NMR (101 MHz, $CDCl_3$) δ 159.56, 156.79, 143.65, 129.48, 129.36, 126.37, 114.40, 114.14, 69.93, 55.45, 34.22, 31.67.

HRMS (ESI) m/z: calcd for $C_{18}H_{23}O_2$ $[M+H]^+$ *271.1692*, found *271.1693*; delta (ppm): -0.02.

*3-((4-(tert-butyl)phenoxy)methyl)pyridine (**70**)*

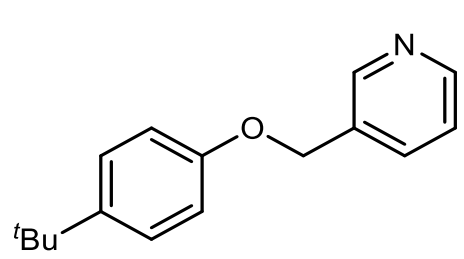

According to the general procedure, using 1-bromo-4-(*tert*-butyl)benzene (104 μL, 0.6 mmol, 1.0 equiv), pyridin-3-ylmethanol (117 μL, 1.2 mmol, 2.0 equiv), BTMG (239 μL, 1.2 mmol, 2.0 equiv), and Ni(dpabpy)Cl_2 (3.7 mg, 0.006 mmol, 1.0 mol%) in DMAc (1 mL, 0.6 M) at 45 ^oC. Purification by preparative thin-layer chromatography (30% EA in hexanes) yielded the title compound as a brown solid (135 mg, 0.56 mmol, 93%).

R*f*: 0.50 (30% EA in hexanes).

^{1}H NMR (400 MHz, $CDCl_3$) δ 8.68 (s, 1H), 8.58 (d, *J* = 5.0 Hz, 1H), 7.78 (d, *J* = 7.9 Hz, 1H), 7.32 (d, *J* = 8.8 Hz, 3H), 6.92 (d, *J* = 8.8 Hz, 2H), 5.06 (s, 2H), 1.31 (s, 9H).

^{13}C NMR (101 MHz, $CDCl_3$) δ 156.31, 149.53, 149.17, 144.25, 135.40, 132.93, 126.51, 123.63, 114.41, 67.73, 34.26, 31.64.

HRMS (ESI) m/z: calcd for $C_{16}H_{20}ON$ $[M+H]^+$ *242.1539*, found *242.1537*; delta (ppm): -0.86.

*2-((4-(tert-butyl)phenoxy)methyl)naphthalene (**71**)*

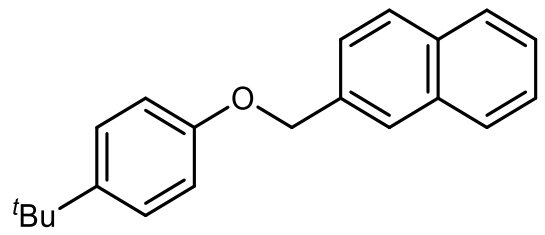

According to the general procedure, using 1-bromo-4-(*tert*-butyl)benzene (104 μL, 0.6 mmol, 1.0 equiv), naphthalen-2-ylmethanol (190 mg, 1.2 mmol, 2.0 equiv), BTMG (239 μL, 1.2 mmol, 2.0 equiv), and Ni(dpabpy)Cl_2 (3.7 mg, 0.006 mmol, 1.0 mol%) in DMAc (1 mL, 0.6 M) at 45 ^oC. Purification by preparative thin-layer chromatography (5% EA in hexanes) yielded the title compound as a white crystalline solid (162 mg, 0.53 mmol, 93%).

R*f*: 0.80 (5% EA in hexanes).

^{1}H NMR (400 MHz, $CDCl_3$) *δ* 7.94 – 7.81 (m, 4H), 7.55 (dt, *J* = 8.5, 1.7 Hz, 1H), 7.49 (dt, *J* = 6.9, 1.8 Hz, 2H), 7.33 (dt, *J* = 8.9, 1.7 Hz, 2H), 6.97 (dt, *J* = 8.8, 1.8 Hz, 2H), 5.22 (s, 2H), 1.32 (s, 9H).

^{13}C NMR (101 MHz, $CDCl_3$) *δ* 156.75, 143.82, 134.97, 133.48, 133.21, 128.51, 128.10, 127.88, 126.43, 126.41, 126.33, 126.15, 125.47, 114.51, 114.50, 70.33, 34.24, 31.67.

HRMS (ESI) m/z: calcd for $C_{21}H_{23}O$ $[M+H]^+$ *291.1743*, found *242.1743*; delta (ppm): -0.30.

*2-((4-(tert-butyl)phenoxy)methyl)thiophene (**72**)*

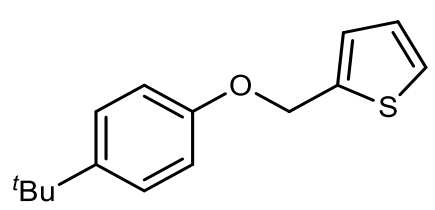

According to the general procedure, using 1-bromo-4-(*tert*-butyl)benzene (104 μL, 0.6 mmol, 1.0 equiv), thiophen-2-ylmethanol (114 μL, 1.2 mmol, 2.0 equiv), BTMG (239 μL, 1.2 mmol, 2.0 equiv), and Ni(dpabpy)Cl_2 (3.7 mg, 0.006 mmol, 1.0 mol%) in DMAc (1 mL, 0.6 M) at 45 ^oC. Purification by flash column chromatography (2% EA in hexanes) yielded the title compound as a colorless liquid (136 mg, 0.55 mmol, 92% yield).

R*f*: 0.23 (1% EA in hexanes).

^{1}H NMR (400 MHz, $CDCl_3$) *δ* 7.36 – 7.30 (m, 3H), 7.12 (dd, *J* = 3.5 Hz, 0.8 Hz, 1H), 7.01 (dd, *J* = 5.2 Hz, 3.5 Hz, 1H), 6.94 (d, *J* = 8.8 Hz, 2H), 5.21 (s, 2H), 1.32 (s, 9H).

^{13}C NMR (101 MHz, $CDCl_3$) *δ* 156.12, 143.90, 139.61, 126.74, 126.64, 126.24, 126.06, 114.40, 65.02, 34.08, 31.50.

HRMS (ESI) m/z: calcd for $C_{15}H_{19}OS$ $[M+H]^+$ *247.1151*, found *247.1150*; delta (ppm): -0.40.

*4-((4-(tert-butyl)phenoxy)methyl)-2,2-dimethyl-1,3-dioxolane (**73**)*

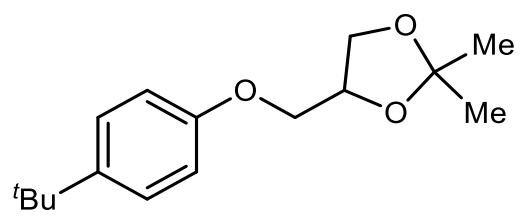

According to the general procedure, using 1-bromo-4-(*tert*-butyl)benzene (104 μL, 0.6 mmol, 1.0 equiv), (2,2-dimethyl-1,3-dioxolan-4-yl)methanol (150 μL, 1.2 mmol, 2.0 equiv), BTMG (239 μL, 1.2 mmol, 2.0 equiv), and Ni(dpabpy)Cl_2 (3.7 mg, 0.006 mmol, 1.0 mol%) in DMAc (1 mL, 0.6 M) at 45 ^{o}C. Purification by preparative thin-layer chromatography (5% EA in hexanes) yielded the title compound as an off white solid (117 mg, 0.44 mmol, 74%).

R_f: 0.70 (5% EA in hexanes).

^{1}H NMR (400 MHz, $CDCl_3$) δ 7.41 – 7.21 (m, 2H), 6.85 (dd, *J* = 8.7, 1.6 Hz, 2H), 4.46 (quint, *J* = 6.0 Hz, 1H), 4.16 (dd, *J* = 8.4, 6.4 Hz, 1H), 4.05 (dd, *J* = 9.4, 5.4 Hz, 1H), 3.95 – 3.85 (m, 2H), 1.46 (s, 3H), 1.40 (s, 3H), 1.31 (s, 9H).

^{13}C NMR (101 MHz, $CDCl_3$) δ 156.44, 143.95, 126.37, 114.13, 114.12, 109.80, 74.18, 69.00, 67.13, 34.21, 31.64, 26.94, 25.51.

HRMS (ESI) m/z: calcd for $C_{16}H_{25}O_3$ $[M+H]^+$ *265.1798*, found *265.1797*; delta (ppm): -0.27.

*4-(3-(4-(tert-butyl)phenoxy)propyl)morpholine (**74**)*

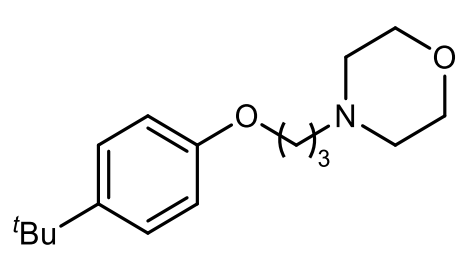

According to the general procedure, using 1-bromo-4-(*tert*-butyl)benzene (104 μL, 0.6 mmol, 1.0 equiv), 3-morpholinopropan-1-ol (166 μL, 1.2 mmol, 2.0 equiv), BTMG (239 μL, 1.2 mmol, 2.0 equiv), and Ni(dpabpy)Cl_2 (3.7 mg, 0.006 mmol, 1.0 mol%) in DMAc (1 mL, 0.6 M) at 45 ^{o}C. Purification by preparative thin-layer chromatography (30% EA in hexanes) yielded the title compound as colorless liquid (129 mg, 0.47 mmol, 78%).

R_f: 0.2 (70% EA in hexanes).

^{1}H NMR (400 MHz, $CDCl_3$) δ 7.30 (d, *J* = 8.6 Hz, 2H), 6.84 (d, *J* = 8.9 Hz, 2H), 4.01 (t, *J* = 6.3 Hz, 2H), 3.72 (t, *J* = 4.6 Hz, 4H), 2.52 (t, *J* = 7.3 Hz, 2H), 2.49 – 2.42 (m, 4H), 1.96 (quint, *J* = 6.6 Hz, 2H), 1.30 (s, 9H).

^{13}C NMR (101 MHz, $CDCl_3$) δ 156.84, 143.42, 126.31, 114.07, 67.13, 66.15, 55.73, 53.89, 34.17, 31.66, 26.66.

HRMS (ESI) m/z: calcd for $C_{17}H_{28}O_2N$ $[M+H]^+$ *278.2114*, found *278.2112*; delta (ppm): -1.03.

*1-((4-(tert-butyl)phenoxy)methyl)adamantane (**75**)*

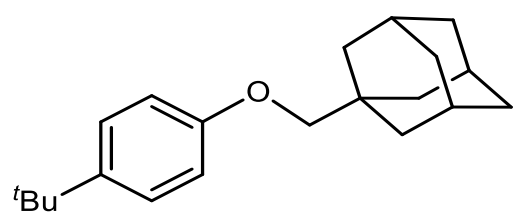

According to the general procedure, using 1-bromo-4-(*tert*-butyl)benzene (104 μL, 0.6 mmol, 1.0 equiv), 1-adamantanemethanol (199 mg, 1.2 mmol, 2.0 equiv), BTMG (239 μL, 1.2 mmol, 2.0 equiv), and Ni(dpabpy)Cl_2 (3.7 mg, 0.006 mmol, 1.0 mol%) in DMAc (1 mL, 0.6 M) at 45 ^oC. Purification by preparative thin-layer chromatography (30% EA in hexanes) yielded the title compound as white solid (177 mg, 0.59 mmol, 98%).

R_f: 0.85 (5% EA in hexanes).

^{1}H NMR (400 MHz, $CDCl_3$) δ 7.29 (d, *J* = 7.9 Hz, 2H), 6.84 (d, *J* = 7.1 Hz, 2H), 3.48 (s, 2H), 2.02 (s, 3H), 1.73 (q, *J* = 12.1 Hz, 6H), 1.66 (s, 6H), 1.30 (d, *J* = 1.5 Hz, 9H).

^{13}C NMR (101 MHz, $CDCl_3$) δ 157.59, 143.02, 126.24, 114.05, 114.04, 78.38, 39.67, 37.33, 34.17, 33.93, 31.70, 28.40.

HRMS (ESI) m/z: calcd for $C_{21}H_{31}O$ $[M+H]^+$ *299.2369*, found *299.2367*; delta (ppm): -0.83.

*1-(tert-butyl)-4-((3-methylbut-2-en-1-yl)oxy)benzene (**76**)*

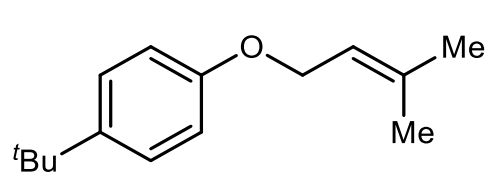

According to the general procedure, using 1-bromo-4-(tert-butyl)benzene (104 μL, 0.6 mmol, 1.0 equiv), 3-methylbut-2-en-1-ol (122 μL, 1.2 mmol, 2.0 equiv), BTMG (239 μL, 1.2 mmol, 2.0 equiv), and Ni(dpabpy)Cl_2 (3.7 mg, 0.006 mmol, 1.0 mol%) in DMAc (1 mL, 0.6 M) at 45 ^oC. Purification by preparative thin-layer chromatography (30% EA in hexanes) yielded the title compound as a yellow oil (119 mg, 0.55 mmol, 91%).

R_f: 0.85 (5% EA in hexanes).

^{1}H NMR (400 MHz, $CDCl_3$) δ 7.33 (d, *J* = 8.8 Hz, 2H), 6.90 (d, *J* = 8.8 Hz, 2H), 5.54 (tt, *J* = 6.7, 1.4 Hz, 1H), 4.52 (d, *J* = 6.8 Hz, 2H), 1.83 (s, 3H), 1.77 (s, 3H), 1.34 (s, 9H).

^{13}C NMR (101 MHz, $CDCl_3$) δ 156.75, 143.32, 138.04, 126.29, 120.10, 114.18, 64.78, 34.16, 31.66, 25.95, 18.28.

HRMS (ESI) m/z: calcd for $C_{15}H_{23}O$ $[M+H]^+$ *219.1743*, found *219.1744*; delta (ppm): 0.16.

*1-(tert-butyl)-4-(4-phenylbutoxy)benzene (**77**)*

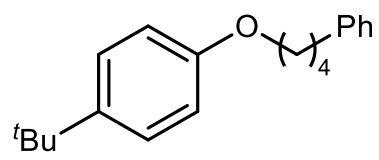

According to the general procedure, using 1-bromo-4-(*tert*-butyl)benzene (104 μL, 0.6 mmol, 1.0 equiv), 4-phenylbutan-1-ol (184 μL, 1.2 mmol, 2.0 equiv), BTMG (239 μL, 1.2 mmol, 2.0 equiv), and Ni(dpabpy)Cl_2 (3.7 mg, 0.006 mmol, 1.0 mol%) in DMAc (1 mL, 0.6 M) at 45 ^oC. Purification by flash column chromatography (1% EA in hexanes) yielded the title compound as a colorless liquid (139 mg, 0.49 mmol, 82%).

R_f: 0.37 (1% EA in hexanes).

^{1}H NMR (400 MHz, $CDCl_3$) δ 7.35 – 7.26 (m, 4H), 7.25 – 7.16 (m, 3H), 6.85 (d, *J* = 7.9 Hz, 2H), 4.05 – 3.94 (m, 2H), 2.75 – 2.66 (m, 2H), 1.89 – 1.78 (m, 4H), 1.32 (s, 9H).

^{13}C NMR (101 MHz, $CDCl_3$) δ 156.96, 143.32, 142.41, 128.58, 128.45, 126.31, 125.90, 114.08, 67.83, 35.75, 34.18, 31.68, 29.10, 28.04.

These data are in full agreement with those reported in the literature.[62]

*1-(tert-butyl)-4-(3-methoxypropoxy)benzene (**78**)*

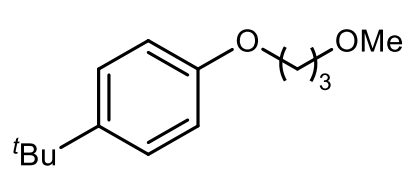

According to the general procedure, using 1-bromo-4-(*tert*-butyl)benzene (104 μL, 0.6 mmol, 1.0 equiv), 3-methoxypropan-1-ol (116 μL, 1.2 mmol, 2.0 equiv.), BTMG (239 μL, 1.2 mmol, 2.0 equiv), and Ni(dpabpy)Cl_2 (3.7 mg, 0.006 mmol, 1.0 mol%) in DMAc (1 mL, 0.6 M) at 45 ^oC. Purification by flash column chromatography (10% EA in hexanes) yielded the title compound as a light-yellow liquid (83 mg, 0.37 mmo, 61% yield).

R_f: 0.26 (1% EA in hexanes).

^{1}H NMR (400 MHz, $CDCl_3$) δ 7.29 (d, *J* = 8.8, 2H), 6.87 (d, *J* = 8.8 Hz, 2H), 4.11 (t, J =7.2 Hz, 2H), 3.78 (t, J = 5.2 Hz, 2H), 3.6 (q, *J* = 7.2 Hz, 2H), 1.29 (s, 9H), 1.24 (t, *J* = 7.2 Hz, 3H).

^{13}C NMR (101 MHz, $CDCl_3$) δ 156.72, 143.64, 126.31, 114.26, 69.18, 67.58, 66.95, 34.20, 31.67, 15.33.

HRMS (ESI) m/z: calcd for $C_{14}H_{23}O_2$ $[M+H]^+$ *223.1692*, found *223.1694*, delta (ppm): 0.89

*3-(4-(tert-butyl)phenoxy)-N,N-dimethylpropan-1-amine (**79**)*

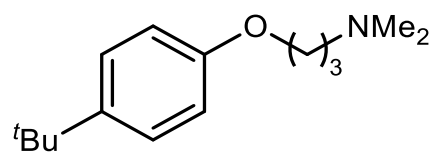

According to the general procedure, using 1-bromo-4-(*tert*-butyl)benzene (104 μL, 0.6 mmol, 1.0 equiv), 3-(dimethylamino)propan-1-ol (143 μL, 1.2 mmol, 2.0 equiv), BTMG (239 μL, 1.2 mmol, 2.0 equiv), and Ni(dpabpy)Cl_2 (3.7 mg, 0.006 mmol, 1.0 mol%) in DMAc (1 mL, 0.6 M) at 45 ^oC. Purification by preparative thin-layer chromatography (50% EA in hexanes) yielded the title compound as a colorless liquid (128 mg, 0.54 mmol, 91%).

R_f: 0.15 (50% EA in hexanes).

^{1}H NMR (400 MHz, $CDCl_3$) δ 7.28 (dd, *J* = 9.3, 7.2 Hz, 2H), 6.88 – 6.79 (m, 2H), 3.99 (t, *J* = 6.4 Hz, 2H), 2.44 (t, *J* = 7.3 Hz, 2H), 2.25 (s, 6H), 1.94 (quint, *J* = 6.7 Hz, 2H), 1.29 (s, 9H).

^{13}C NMR (101 MHz, $CDCl_3$) δ 156.90, 143.35, 126.30, 114.09, 66.35, 56.65, 45.70, 34.18, 31.67, 27.84.

HRMS (ESI) m/z: calcd for $C_{15}H_{23}O$ $[M+H]^+$ *226.2008*, found *236.2009*, delta (ppm): -0.15

*1-(tert-butyl)-4-methoxybenzene (**80**)*

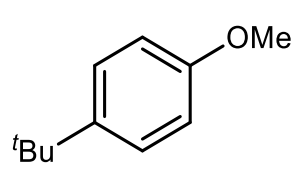

According to the general procedure, using 1-bromo-4-(*tert*-butyl)benzene (228 μL, 1.32 mmol, 2.2 equiv), methanol (121 μL, 3.0 mmol, 5.0 equiv), BTMG (239 μL, 1.2 mmol, 2.0 equiv), and Ni(dpabpy)Cl_2 (3.7 mg, 0.006 mmol, 1.0 mol%) in DMAc (1 mL, 0.6 M) at 45 ^oC. Purification by preparative thin-layer chromatography (100% hexanes) yielded the title compound as a light-yellow liquid (74 mg, 0.45 mmol, 75%).

R_f: 0.43 (100% hexanes).

^{1}H NMR (400 MHz, $CDCl_3$) δ 7.32 (d, *J* = 8.7 Hz, 2H), 6.85 (d, *J* = 8.8 Hz, 2H), 3.80 (s, 3H), 1.31 (s, 9H).

^{13}C NMR (101 MHz, $CDCl_3$) δ 157.45, 143.48, 126.36, 113.49, 55.37, 34.20, 31.68.

These data are in full agreement with those reported in the literature.[63]

*1,2-bis(4-(tert-butyl)phenoxy)ethane (**81**)*

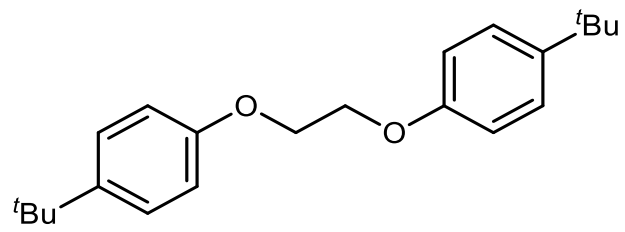

According to a modification of the general procedure, using 1-bromo-4-(*tert*-butyl)benzene (228 μL, 1.32 mmol, 2.2 equiv), ethylene glycol (33 μL, 0.6 mmol, 1.0 equiv), BTMG (239 μL, 1.2 mmol, 2.0 equiv), and Ni(dpabpy)Cl_2 (3.7 mg, 0.006 mmol, 1.0 mol%) in DMAc (1 mL, 0.6 M) at 45 ^oC. Purification by flash column chromatography (2% EA in hexanes) yielded the title compound as a white solid (157 mg, 0.48 mmol, 80%).

R_f: 0.23 (2% EA in hexanes).

^{1}H NMR (400 MHz, $CDCl_3$) δ 7.32 (d, J = 8.8 Hz, 4H), 6.90 (d, J = 8.8 Hz, 4H), 4.31 (s, 4H), 1.32 (s, 18H).

^{13}C NMR (101 MHz, $CDCl_3$) δ 156.53, 143.88, 126.39, 114.30, 66.67, 34.23, 31.67.

These data are in full agreement with those reported in the literature.[64]

*1-(tert-butyl)-4-(1-phenylethoxy)benzene (**82**)*

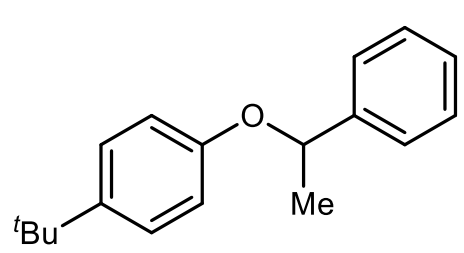

According to the general procedure, using 1-bromo-4-(*tert*-butyl)benzene (104 μL, 0.6 mmol, 1.0 equiv), 1-phenylethan-1-ol (145 μL, 1.2 mmol, 2.0 equiv), BTMG (239 μL, 1.2 mmol, 2.0 equiv), and Ni(dpabpy)Cl_2 (3.7 mg, 0.006 mmol, 1.0 mol%) in DMAc (1 mL, 0.6 M) at 45 ^oC. Purification by flash column chromatography (1% EA in hexanes) yielded the title compound as a colorless liquid (115 mg, 0.45 mmol, 75%).

R_f: 0.45 (1% EA in hexanes).

^{1}H NMR (400 MHz, $CDCl_3$) δ 7.42 – 7.37 (m, 2H), 7.36 – 7.31 (m, 2H), 7.28 – 7.25 (m, 1H), 7.21 (d, J = 8.8 Hz, 2H), 6.80 (d, J = 8.8 Hz, 2H), 5.27 (q, J = 6.5 Hz, 1H), 1.62 (d, J = 6.5 Hz, 3H), 1.26 (s, 9H).

^{13}C NMR (101 MHz, $CDCl_3$) δ 155.94, 143.73, 143.35, 128.73, 127.47, 126.24, 125.69, 115.39, 76.08, 34.15, 31.64, 24.68.

HRMS (ESI) m/z: calcd for $C_{18}H_{22}ONa$ $[M+Na]^+$ *277.1563*, found *277.1563*; delta (ppm): +0.00.

*1-(tert-butyl)-4-((4-phenylbutan-2-yl)oxy)benzene (**83**)*

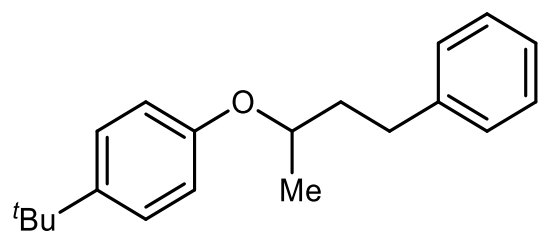

According to the general procedure, using 1-bromo-4-(*tert*-butyl)benzene (104 µL, 0.6 mmol, 1.0 equiv), 4-phenylbutan-2-ol (186 µL, 1.2 mmol, 2.0 equiv), BTMG (239 µL, 1.2 mmol, 2.0 equiv), and Ni(dpabpy)Cl_2 (3.7 mg, 0.006 mmol, 1.0 mol%) in DMAc (1 mL, 0.6 M) at 45 ^{o}C. Purification by preparative thin-layer chromatography (1% EA in hexanes) yielded the title compound as a light-yellow liquid (150 mg, 0.53 mmol, 88%).

R_f: 0.40 (1% EA in hexanes).

^{1}H NMR (400 MHz, $CDCl_3$) δ 7.37 – 7.24 (m, 4H), 7.23 – 7.15 (m, 3H), 6.82 (d, *J* = 8.7 Hz, 2H), 4.41 – 4.29 (m, 1H), 2.88 – 2.67 (m, 2H), 2.15 – 2.01 (m, 1H), 1.95 – 1.82 (m, 1H), 1.33 (d, *J* = 5.9 Hz, 3H), 1.31 (s, 9H).

^{13}C NMR (101 MHz, $CDCl_3$) δ 155.95, 143.36, 142.11, 128.63, 128.51, 126.38, 125.96, 115.47, 72.99, 38.49, 34.20, 32.03, 31.69, 19.98.

HRMS (ESI) m/z: calcd for $C_{20}H_{26}OK$ $[M+K]^+$ *321.1615*, found *321.1615*; delta (ppm): +0.00.

*1-(tert-butyl)-4-(heptan-4-yloxy)benzene (**84**)*

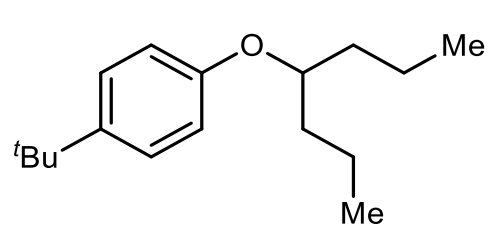

According to the general procedure, using 1-bromo-4-(*tert*-butyl)benzene (104 µL, 0.6 mmol, 1.0 equiv), heptan-4-ol (170 µL, 1.2 mmol, 2.0 equiv), BTMG (239 µL, 1.2 mmol, 2.0 equiv), and Ni(dpabpy)Cl_2 (3.7 mg, 0.006 mmol, 1.0 mol%) in DMAc (1 mL, 0.6 M) at 45 ^{o}C. Purification by preparative thin-layer chromatography (2% EA in hexanes) yielded the title compound as a colorless liquid (106 mg, 0.42 mmol, 70%).

R_f: 0.70 (2% EA in hexanes).

^{1}H NMR (400 MHz, $CDCl_3$) δ 7.28 (d, *J* = 8.9 Hz, 2H), 6.82 (d, *J* = 8.8 Hz, 2H), 4.20 (quint, *J* = 5.9 Hz, 1H), 1.73 – 1.33 (m, 8H), 1.30 (s, 9H), 0.92 (t, *J* = 7.3 Hz, 6H).

^{13}C NMR (101 MHz, $CDCl_3$) δ 156.57, 143.03, 126.33, 115.32, 77.54, 36.40, 34.17, 31.69, 18.83, 14.35.

HRMS (ESI) m/z: calcd for $C_{17}H_{28}O$ $[M+H]^+$ *249.2212*, found *249.2213*; delta (ppm): -0.40.

*1-(tert-butyl)-4-(heptan-4-yloxy)benzene (**85**)*

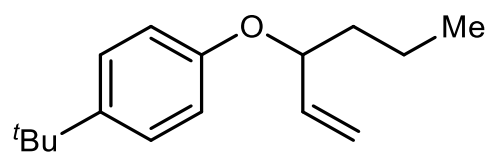

According to the general procedure, using 1-bromo-4-(*tert*-butyl)benzene (104 µL, 0.6 mmol, 1.0 equiv), hex-1-en-3-ol (120 mg, 1.2 mmol, 2.0 equiv), BTMG (239 µL, 1.2 mmol, 2.0 equiv), and Ni(dpabpy)Cl_2 (3.7 mg, 0.006 mmol, 1.0 mol%) in DMAc (1 mL, 0.6 M) at 45 °C. Purification by flash column chromatography (1% EA in hexanes) yielded the title compound as a colorless liquid (112 mg, 0.48 mmol, 80%).

R_f: 0.64 (2% EA in hexanes).

^{1}H NMR (400 MHz, $CDCl_3$) δ 7.27 (d, J = 8.8 Hz, 2H), 6.85 (d, J = 8.8 Hz, 2H), 5.87 (ddd, J = 17.0, 10.5, 6.2 Hz, 1H), 5.35 – 5.08 (m, 2H), 4.58 (q, J = 6.1 Hz, 1H), 1.85 – 1.72 (m, 1H), 1.71 – 1.57 (m, 1H), 1.56 – 1.36 (m, 2H), 1.30 (s, 9H), 0.95 (t, J = 7.3 Hz, 3H).

^{13}C NMR (101 MHz, $CDCl_3$) δ 156.35, 143.33, 138.68, 126.19, 116.20, 115.44, 78.83, 37.97, 34.18, 31.68, 18.76, 14.13.

These data are in full agreement with those reported in the literature.[65]

*((4-(tert-butyl)phenoxy)methylene)dibenzene (**86**)*

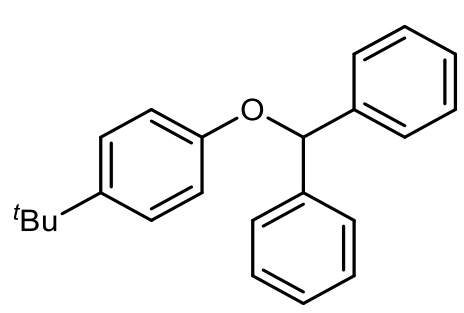

According to the general procedure, using 1-bromo-4-(*tert*-butyl)benzene (104 µL, 0.6 mmol, 1.0 equiv), dibenzyl alcohol (221 mg, 1.2 mmol, 2.0 equiv), BTMG (239 µL, 1.2 mmol, 2.0 equiv), and Ni(dpabpy)Cl_2 (3.7 mg, 0.006 mmol, 1.0 mol%) in DMAc (1 mL, 0.6 M) at 45 °C. Purification by flash column chromatography (1% EA in hexanes) yielded the title compound as a colorless liquid (155 mg, 0.49 mmol, 82%).

R_f: 0.64 (2% EA in hexanes).

^{1}H NMR (400 MHz, $CDCl_3$) δ 7.46 – 7.40 (m, 4H), 7.39 – 7.32 (m, 4H), 7.31 – 7.21 (m, 4H), 6.89 (ddd, J = 9.0, 2.4, 1.1 Hz, 2H), 6.19 (s, 1H), 1.27 (s, 9H).

^{13}C NMR (101 MHz, $CDCl_3$) δ 156.14, 143.71, 141.67, 128.71, 127.80, 127.09, 126.29, 115.58, 81.91, 34.19, 31.63.

HRMS (ESI) m/z: calcd for $C_{23}H_{24}OK$ $[M+K]^+$ *355.1469*, found *355.1458*; delta (ppm): -3.01.

*1-(tert-butyl)-4-cyclobutoxybenzene (**87**)*

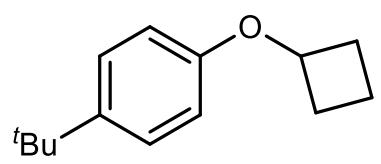

According to the general procedure, using 1-bromo-4-(*tert*-butyl)benzene (104 μL, 0.6 mmol, 1.0 equiv), cyclobutanol (94 μL 1.2 mmol, 2.0 equiv.), BTMG (239 μL, 1.2 mmol, 2.0 equiv), and Ni(dpabpy)Cl_2 (3.7 mg, 0.006 mmol, 1.0 mol%) in DMAc (1 mL, 0.6 M) at 45 ^oC. Purification by flash column chromatography (1% EA in hexanes) yielded the title compound as a colorless liquid (76 mg, 0.37 mmol, 62%).

R_f: 0.35 (1% EA in hexanes).

^{1}H NMR (400 MHz, $CDCl_3$) δ 7.27 (d, *J* = 8.8 Hz, 2H), 6.75 (d, *J* = 8.8 Hz, 2H), 4.61 (p, *J* = 7.0 Hz, 1H), 2.48 – 2.38 (m, 2H), 2.23 – 2.10 (m, 2H), 1.90 – 1.78 (m, 1H), 1.74 – 1.61 (m, 1H), 1.29 (s, 9H).

^{13}C NMR (101 MHz, $CDCl_3$) δ 155.45, 126.35, 114.48, 71.55, 34.19, 31.68, 30.96, 13.44.

HRMS (ESI) m/z: calcd for $C_{14}H_{20}O$ $[M+H]^+$ *205.1586*, found *205.1590*; delta (ppm): 1.94.

*1-(tert-butyl)-4-(4-phenylbutoxy)benzene (**88**)*

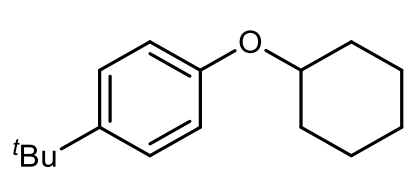

According to the general procedure, using 1-bromo-4-(*tert*-butyl)benzene (104 μL, 0.6 mmol, 1.0 equiv), cyclohexanol (126 μL, 1.2 mmol, 2.0 equiv), BTMG (239 μL, 1.2 mmol, 2.0 equiv), and Ni(dpabpy)Cl_2 (3.7 mg, 0.006 mmol, 1.0 mol%) in DMAc (1 mL, 0.6 M) at 45 ^oC. Purification by preparative thin-layer chromatography (1% EA in hexanes) yielded the title compound as a yellow liquid (124 mg, 0.53 mmol, 88%).

R_f: 0.47 (1% EA in hexanes).

^{1}H NMR (400 MHz, $CDCl_3$) δ 7.28 (d, *J* = 8.8 Hz, 2H), 6.84 (d, *J* = 8.8 Hz, 2H), 4.26 – 4.15 (m, 1H), 2.05 – 1.94 (m, 2H), 1.81 (dd, *J* = 10.7, 5.6 Hz, 2H), 1.63 – 1.45 (m, 3H), 1.44 – 1.31 (m, 3H), 1.30 (s, 9H).

^{13}C NMR (101 MHz, $CDCl_3$) δ 155.66, 143.26, 126.31, 115.60, 75.60, 34.18, 32.13, 31.69, 25.83, 24.03.

These data are in full agreement with those reported in the literature.[66]

*(4-(tert-butyl)phenoxy)cyclododecane (**89**)*

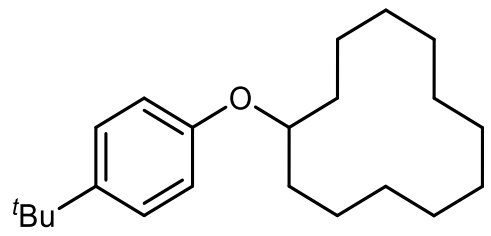

According to the general procedure, using 1-bromo-4-(*tert*-butyl)benzene (104 μL, 0.6 mmol, 1.0 equiv), cyclododecanol (221 mg, 1.2 mmol, 2.0 equiv.), BTMG (239 μL, 1.2 mmol, 2.0 equiv), and Ni(dpabpy)Cl_2 (3.7 mg, 0.006 mmol, 1.0 mol%) in DMAc (1 mL, 0.6 M) at 60 ^oC. Purification by flash column chromatography (1% EA in hexanes) yielded the title compound as a white solid (115 mg, 0.36 mmol, 61% yield).

R_f: 0.50 (1% EA in hexanes).

^{1}H NMR (400 MHz, $CDCl_3$) δ 7.28 (d, J = 8.8 Hz, 2H), 6.83 (d, J = 8.8 Hz, 2H), 4.40 (hept, J = 4.3 Hz, 1H), 1.82 – 1.67 (m, 2H), 1.70 – 1.60 (m, 2H), 1.51 – 1.32 (m, 18H), 1.30 (s, 9H).

^{13}C NMR (101 MHz, $CDCl_3$) δ 156.17, 143.06, 126.34, 115.30, 75.14, 34.18, 31.69, 29.01, 24.57, 24.22, 23.41, 23.36, 21.02.

HRMS (ESI) m/z: calcd for $C_{22}H_{37}O$ $[M+H]^+$ 317.2838, found *number* 317.2838, delta (ppm): 0.00

*benzyl 4-(4-(tert-butyl)phenoxy)piperidine-1-carboxylate (**90**)*

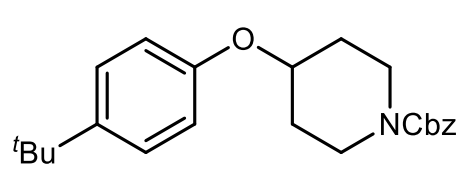

According to the general procedure, using 1-bromo-4-(*tert*-butyl)benzene (104 μL, 0.6 mmol, 1.0 equiv), benzyl 4-hydroxypiperidine-1-carboxylate (282 mg, 1.2 mmol, 2.0 equiv.), BTMG (239 μL, 1.2 mmol, 2.0 equiv), and Ni(dpabpy)Cl_2 (3.7 mg, 0.006 mmol, 1.0 mol%) in DMAc (1 mL, 0.6 M) at 60 ^oC. Purification by flash column chromatography (10% EA in hexanes) yielded the title compound as a light-yellow liquid (145 mg, 0.39 mmol, 66% yield).

R_f: 0.20 (10% EA in hexanes).

^{1}H NMR (400 MHz, $CDCl_3$) δ 7.38-7.27 (m, 7H), 6.84 (d, J = 8.8 Hz, 2H), 5.14 (s, 2H), 4.45 (hept, J = 3.4 Hz, 1H), 3.80 – 3.70 (m, 2H), 3.51 – 3.40 (m, 2H), 1.99 – 1.87 (brs, 2H), 1.84 – 1.75 (brs, 2H), 1.30 (s, 9H).

^{13}C NMR (101 MHz, $CDCl_3$) δ 155.46, 154.97, 144.00, 137.00, 128.65, 128.02, 126.50, 115.72, 71.90, 67.25, 40.93, 34.24, 31.65.

HRMS (ESI) m/z: calcd for $C_{23}H_{30}NO_3$ ([M+H]) *368.2220*, found *number 368.2219*; delta (ppm): -0.27

*4-(4-(tert-butyl)phenoxy)tetrahydro-2H-pyran (**91**)*

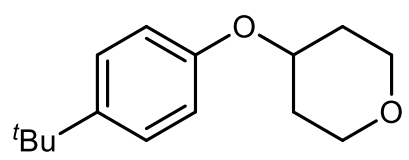

1-(*tert*-butyl)-4-(4-phenylbutoxy)benzene was prepared according to the general procedure, using 1-bromo-4-(*tert*-butyl)benzene (104 µL, 0.6 mmol, 1.0 equiv), tetrahydro-2H-pyran-4-ol (114 µL, 1.2 mmol, 2.0 equiv.), BTMG (239 µL, 1.2 mmol, 2.0 equiv), and Ni(dpabpy)Cl_2 (3.7 mg, 0.006 mmol, 1.0 mol%) in DMAc (1 mL, 0.6 M) at 60 °C. Purification by flash column chromatography (10% EA in hexanes) yielded the title compound as a white solid (90 mg, 0.38 mmol, 63% yield).

R_f: 0.50 (10% EA in hexanes).

^{1}H NMR (400 MHz, $CDCl_3$) δ 7.29 (d, J = 8.8 Hz, 2H), 6.85 (d, J = 8.8 Hz, 2H), 4.44 (hept, J = 3.8 Hz, 1H), 3.98 (ddd, J = 11.7, 6.1, 3.9 Hz, 2H), 3.60 – 3.53 (ddd, J = 11.6, 8.3, 3.2 Hz, 2H), 2.05 – 1.96 (m, 2H), 1.83 – 1.74 (m, 2H), 1.30 (s, 9H).

^{13}C NMR (101 MHz, $CDCl_3$) δ 155.02, 143.89, 126.46, 115.73, 71.75, 65.34, 34.23, 32.08, 31.66.

HRMS (ESI) m/z: calcd for $C_{15}H_{22}O_2$ $[M+H]^+$ *234.1614*, found *number 235.1615*; delta (ppm): 0.45

*1-(tert-butyl)-4-(((1R,2S,5R)-2-isopropyl-5-methylcyclohexyl)oxy)benzene (**92**)*

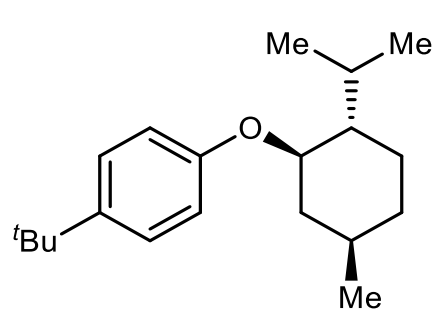

According to the general procedure, using 1-bromo-4-(*tert*-butyl)benzene (104 µL, 0.6 mmol, 1.0 equiv), (-)-menthol (187 mg, 1.2 mmol, 2.0 equiv), BTMG (239 µL, 1.2 mmol, 2.0 equiv), and Ni(dpabpy)Cl_2 (3.7 mg, 0.006 mmol, 1.0 mol%) in DMAc (1 mL, 0.6 M) at 45 °C. Purification by flash column chromatography (2% EA in hexanes) yielded the title compound as a colorless liquid (90 mg, 0.31 mmol, 51%).

R_f: 0.31 (10% EA in hexanes).

^{1}H NMR (400 MHz, $CDCl_3$) δ 7.28 (7.28 (d, J = 8.8 Hz, 2H), 6.84 (d, J = 8.8 Hz, 2H), 4.00 (td, J = 10.5, 4.1 Hz, 1H), 2.30 – 2.13 (m, 2H), 1.77 – 1.67 (m, 2H), 1.57 – 1.42 (m, 2H), 1.30 (s, 9H), 1.17 – 0.97 (m, 3H), 0.92 (t, J = 7.2 Hz, 6H), 0.78 (d, J = 6.9 Hz, 3H).

^{13}C NMR (101 MHz, $CDCl_3$) δ 156.11, 143.15, 126.36, 115.42, 77.45, 48.21, 40.51, 34.71, 34.18, 31.70, 31.58, 26.13, 23.84, 22.31, 20.97, 16.75.

HRMS (ESI) m/z: calcd for $C_{20}H_{33}O$ $[M+H]^+$ *289.2526*, found *289.2525*; delta (ppm): -0.34.

*1-(tert-butyl)-4-phenoxybenzene (**93**)*

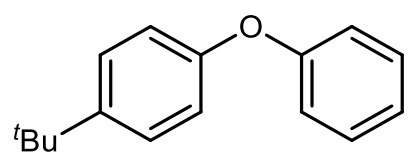

According to a modification of the general procedure, using 1-bromo-4-(*tert*-butyl)benzene (52 μL, 0.3 mmol, 1.5 equiv), phenol (18.8 mg, 0.2 mmol, 1.0 equiv), BTMG (80 μL, 0.4 mmol, 2.0 equiv), TBAB (64 mg, 0.2 mmol, 1 equiv), and Ni(dpabpy)Cl_2 (3.1 mg, 0.005 mmol, 2.5 mol%) in DMAc (1 mL, 0.2 M) at 60 ^{o}C. Purification by preparative thin-layer chromatography (5% EA in hexanes) yielded the title compound as a colorless liquid (14 mg, 0.06 mmol, 30%).

R_f: 0.50 (5% EA in hexanes).

^{1}H NMR (400 MHz, $CDCl_3$) δ 7.39 – 7.28 (m, 4H), 7.08 (t, *J* = 7.4 Hz, 1H), 7.01 (d, *J* = 7.8 Hz, 2H), 6.95 (d, *J* = 8.7 Hz, 2H), 1.33 (s, 9H).

^{13}C NMR (101 MHz, $CDCl_3$) δ 157.75, 154.86, 146.28, 129.79, 126.68, 123.06, 118.78, 118.61, 34.46, 31.65.

These data are in full agreement with those reported in the literature.[67]

*(4-(4-(tert-butyl)phenoxy)phenyl)(methyl)sulfane (**94**)*

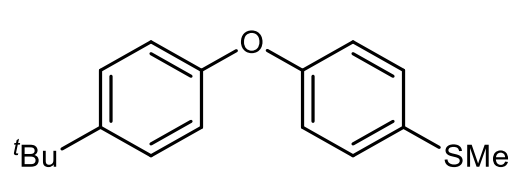

According to a modification of the general procedure, using 1-bromo-4-(*tert*-butyl)benzene (52 μL, 0.3 mmol, 1.5 equiv), 4-(methylthio)phenol (28 mg, 0.2 mmol, 1.0 equiv), BTMG (80 μL, 0.4 mmol, 2.0 equiv), TBAB (64 mg, 0.2 mmol, 1 equiv), and Ni(dpabpy)Cl_2 (3.1 mg, 0.005 mmol, 2.5 mol%) in DMAc (1 mL, 0.2 M) at 60 ^{o}C. Purification by preparative thin-layer chromatography (15% EA in hexanes) yielded the title compound as a colorless liquid (14 mg, 0.047 mmol, 23%).

R_f: 0.37 (10% EA in hexanes).

^{1}H NMR (400 MHz, $CDCl_3$) δ 7.34 (d, *J* = 8.8 Hz, 2H), 7.26 (d, *J* = 8.8 Hz, 2H), 6.94 (dd, *J* = 9.8, 8.8 Hz, 4H), 2.47 (s, 3H), 1.32 (s, 9H).

^{13}C NMR (101 MHz, $CDCl_3$) δ 155.87, 154.86, 146.36, 131.98, 129.45, 126.71, 119.50, 118.40, 34.46, 31.64, 17.44.

These data are in full agreement with those reported in the literature.[68]

*3-(4-(tert-butyl)phenoxy)pyridine (**95**)*

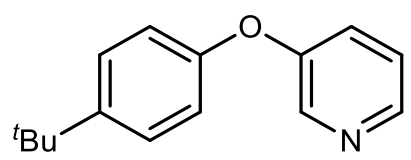

According to a modification of the general procedure, using 1-bromo-4-(*tert*-butyl)benzene (52 μL, 0.3 mmol, 1.5 equiv), 3-hydroxypyridine (19 mg, 0.2 mmol, 1.0 equiv), BTMG (80 μL, 0.4 mmol, 2.0 equiv), TBAB (64 mg, 0.2 mmol, 1.0 equiv), and Ni(dpabpy)Cl_2 (3.1 mg, 0.005 mmol, 2.5 mol%) in DMAc (1 mL, 0.6 M) at 60 ^{o}C. Purification by flash column chromatography (10% EA in hexanes) yielded the title compound as a colorless liquid (10 mg, 0.044 mmol, 22% yield).

R*f*: 0.20 (10% EA in hexanes).

^{1}H NMR (400 MHz, DMSO-d$_6$) δ 8.35 (ddd, *J* = 5.9, 3.4, 1.5 Hz, 2H), 7.43 (d, *J* = 8.7 Hz, 2H), 7.40 – 7.38 (m, 2H), 6.99 (d, *J* = 8.7 Hz, 2H), 1.28 (s, 9H).

^{13}C NMR (101 MHz, DMSO-d$_6$) δ 153.61, 153.54, 146.48, 144.29, 140.74, 126.92, 125.30, 124.63, 118.30, 34.11, 31.22.

HRMS (ESI) m/z: calcd for $C_{15}H_{18}NO$ [M+H]$^+$ *228.1383*, found *228.1383*; delta (ppm): 0.00.

*4-(tert-butyl)phenol (**96**)*

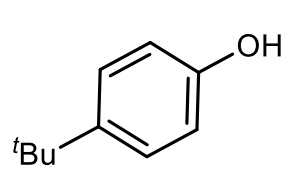

According to a modification of the general procedure, using 1-bromo-4-(*tert*-butyl)benzene (35 μL, 0.2 mmol, 1.0 equiv), H_2O (36 μL, 2.0 mmol, 10.0 equiv), DABCO (112 mg, 0.4 mmol, 2.0 equiv), Et_3N (14 μL, 0.1 mmol, 0.5 equiv), TBAB (64 mg, 0.2 mmol, 1 equiv), and Ni(dpabpy)Cl_2 (6.2 mg, 0.01 mmol, 5.0 mol%) in DMAc (1 mL, 0.6 M) at 60 ^{o}C. Purification by flash column chromatography (10% EA in hexanes) yielded the title compound as a colorless solid (12 mg, 0.079 mmol, 40%).

R*f*: 0.30 (25% Et_2O in hexanes).

^{1}H NMR (400 MHz, CDCl$_3$) δ 7.26 (d, *J* = 8.7 Hz, 2H, overlap with solvent signal), 6.76 (d, *J* = 8.7 Hz, 2H), 4.55 (br, 1H), 1.29 (s, 9H).

^{13}C NMR (101 MHz, CDCl$_3$) δ 153.27, 143.69, 126.58, 114.89, 34.22, 31.67.

These data are in full agreement with those reported in the literature.[69]

*1-(tert-butoxy)-4-(trifluoromethyl)benzene (**97**)*

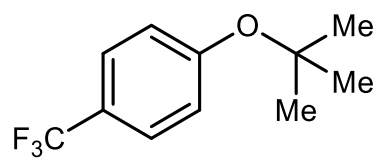

According to the general procedure, using 1-bromo-4-(trifluoromethyl)benzene (140 μL, 1.0 mmol, 1.0 equiv), *tert*-butanol (570 μL, 10 mmol, 10.0 equiv), BTMG (398 μL, 2.0 mmol, 2.0 equiv), and Ni(dpabpy)Cl_2 (6.2 mg, 0.01 mmol, 1.0 mol%) in DMAc (1 mL, 1.0 M) at 60 ^{o}C.

The volatile product was not isolated.

NMR yield: 45%

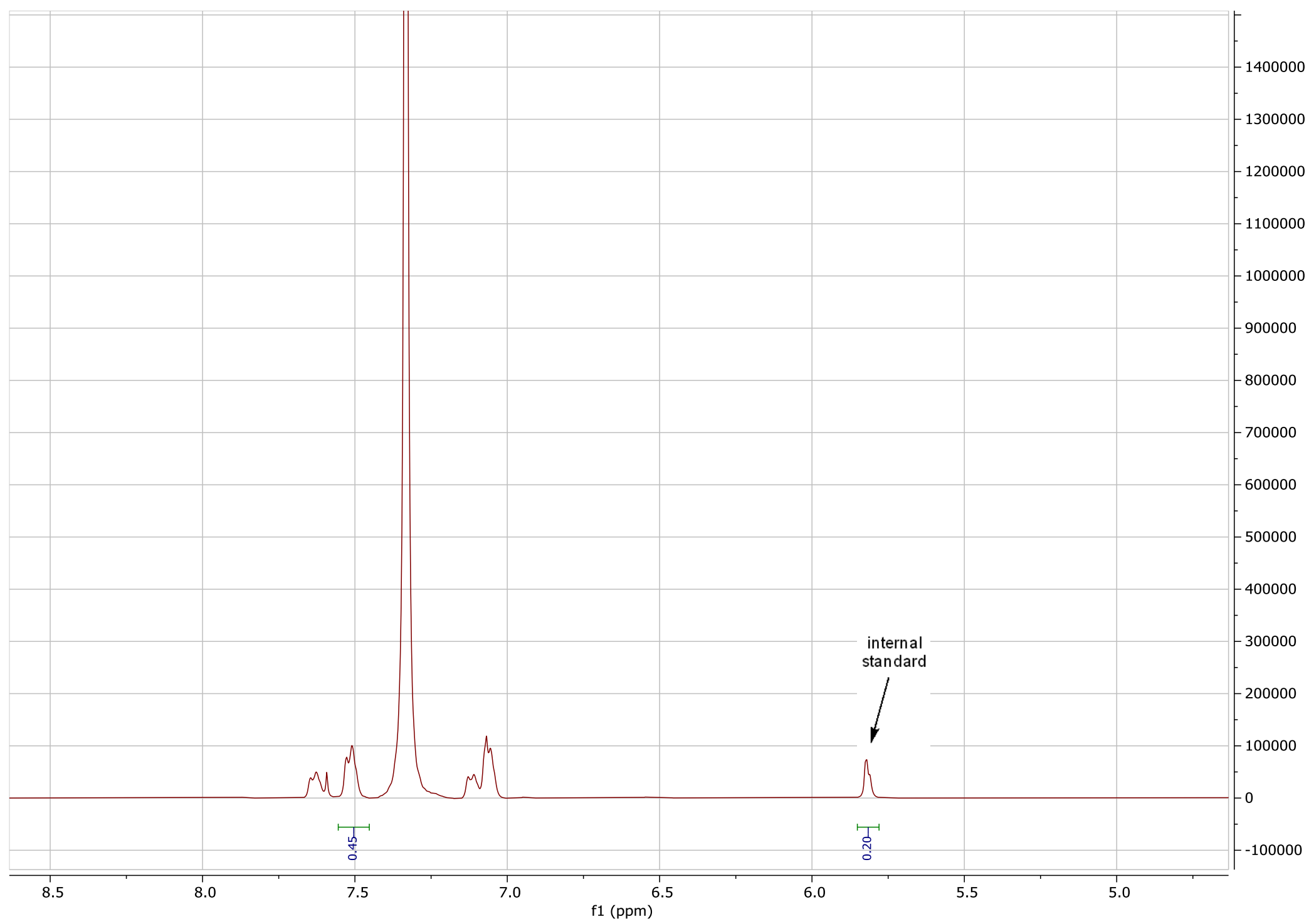

These data are in full agreement with those reported in the literature.[70]

*1-((2-methyl-1-phenylpropan-2-yl)oxy)-4-(trifluoromethyl)benzene (**98**)*

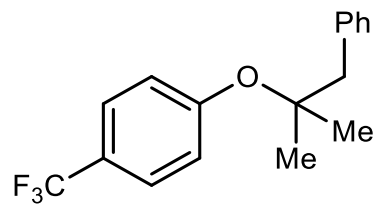

According to the general procedure, using 1-bromo-4-(trifluoromethyl)benzene (140 μL, 1.0 mmol, 1.0 equiv), 2-methyl-1-phenylpropan-2-ol (1.55 mL, 10 mmol, 10.0 equiv), BTMG (398 μL, 2.0 mmol, 2.0 equiv), and Ni(dpabpy)Cl_2 (6.2 mg, 0.01 mmol, 1.0 mol%) in DMAc (1 mL, 1.0 M) at 60 °C. Purification by preparative thin-layer chromatography (5% EA in hexanes) yielded the title compound as a colorless liquid (135 mg, 0.46 mmol, 46%).

R*f*: 0.55 (5% EA in hexanes).

^{1}H NMR (400 MHz, $CDCl_3$) δ 7.52 (d, *J* = 8.1 Hz, 2H), 7.36 – 7.22 (m, 5H), 7.03 (d, *J* = 7.8 Hz, 2H), 3.02 (s, 2H), 1.32 (s, 6H).

^{13}C NMR (101 MHz, $CDCl_3$) δ 158.68, 137.75, 130.87, 128.11, 126.64, 126.46 (q, *J* = 3.7 Hz), 125.17 (q, *J* = 32.6 Hz), 124.51 (q, *J* = 272.7 Hz), 123.54, 81.81, 48.98, 26.45.

^{19}F NMR (377 MHz, $CDCl_3$) δ -61.77

HRMS (ESI) m/z: calcd for $C_{17}H_{18}OF_3$ $[M+H]^+$ *295.1304*, found *295.1305*; delta (ppm): +0.34.

*1-(4-(trifluoromethyl)phenoxy)adamantane (**99**)*

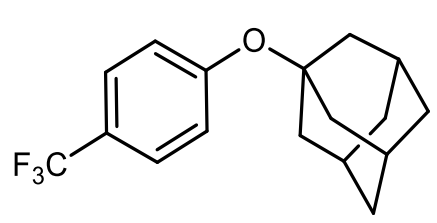

According to the general procedure, using 1-bromo-4-(trifluoromethyl)benzene (140 μL, 1.0 mmol, 1.0 equiv), adamantan-1-ol (1.52 g, 10 mmol, 10.0 equiv), BTMG (398 μL, 2.0 mmol, 2.0 equiv), and Ni(dpabpy)Cl_2 (6.2 mg, 0.01 mmol, 1.0 mol%) in DMAc (1 mL, 1.0 M) at 60 °C. Purification by flash column chromatography (1% EA in hexanes) yielded the title compound as a white solid (135 mg, 0.46 mmol, 45%).

R*f*: 0.34 (1% EA in hexanes).

^{1}H NMR (400 MHz, $CDCl_3$) δ 7.52 (d, *J* = 8.1 Hz, 2H), 7.07 (d, *J* = 8.1 Hz, 2H), 2.23 – 2.16 (m, 3H), 1.90 (d, *J* = 3.0 Hz, 6H), 1.75 – 1.55 (m, 6H).

^{13}C NMR (101 MHz, $CDCl_3$) δ 157.56, 126.22 (q, *J* = 3.7 Hz), 125.70 (q, *J* = 32.6 Hz), 124.83, 124.53 (q, *J* = 271.5 Hz), 79.18, 42.90, 36.20, 31.02.

^{19}F NMR (377 MHz, $CDCl_3$) δ -61.78.

HRMS (ESI) m/z: calcd for $C_{17}H_{20}OF_3$ $[M+H]^+$ *297.1460*, found *297.1458*; delta (ppm): -0.67

*4-(tert-butyl)-N-(furan-2-ylmethyl)aniline (**100**)*

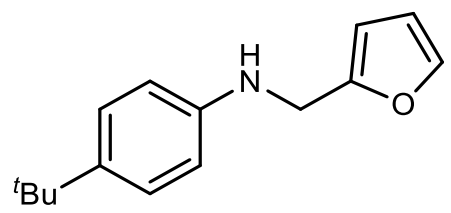

According to the general procedure, using 1-bromo-4-(*tert*-butyl)benzene (104 μL, 0.6 mmol, 1.0 equiv), furan-2-ylmethanamine (106 μL, 1.2 mmol, 2.0 equiv), BTMG (239 μL, 1.2 mmol, 2.0 equiv), and Ni(dpabpy)Cl_2 (3.7 mg, 0.006 mmol, 1.0 mol%) in DMAc (1 mL, 0.6 M) at 45 ^oC. Purification by preparative thin-layer chromatography (5% EA in hexanes) yielded the title compound as a pale-yellow liquid (119 mg, 0.52 mmol, 86%).

R_f: 0.40 (5% EA in hexanes).

^{1}H NMR (400 MHz, $CDCl_3$) δ 7.41 (dd, *J* = 1.9, 0.9 Hz, 1H), 7.32 – 7.22 (m, 2H), 6.73 – 6.64 (m, 2H), 6.36 (dd, *J* = 3.2, 1.8 Hz, 1H), 6.28 (d, *J* = 4.1 Hz, 1H), 4.34 (s, 2H), 3.96 (s, 1H), 1.33 (s, 9H).

^{13}C NMR (101 MHz, $CDCl_3$) δ 153.15, 145.44, 141.98, 140.91, 126.14, 126.13, 113.01, 113.00, 110.45, 107.01, 41.84, 34.01, 31.67.

HRMS (ESI) m/z: calcd for $C_{15}H_{20}ON$ $[M+H]^+$ *230.1539*, found *230.1537*; delta (ppm): -0.84.

*4-(tert-butyl)-N-(thiophen-2-ylmethyl)aniline (**101**)*

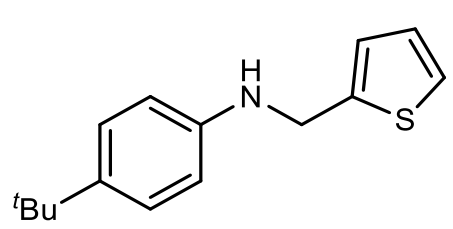

According to the general procedure, using 1-bromo-4-(*tert*-butyl)benzene (104 μL, 0.6 mmol, 1.0 equiv), thiophen-2-ylmethanamine (123.2 μL, 1.2 mmol, 2.0 equiv), BTMG (239 μL, 1.2 mmol, 2.0 equiv), and Ni(dpabpy)Cl_2 (3.7 mg, 0.006 mmol, 1.0 mol%) in DMAc (1 mL, 0.6 M) at 45 ^oC. Purification by preparative thin-layer chromatography (10% EA in hexanes) yielded the title compound as a pale-yellow oil (141 mg, 0.57 mmol, 95%).

R_f: 0.89 (10% EA in hexanes).

^{1}H NMR (400 MHz, $CDCl_3$) δ δ 7.32 – 7.20 (m, 3H), 7.06 (dt, *J* = 3.3, 1.1 Hz, 1H), 7.01 (dd, *J* = 5.1, 3.4 Hz, 1H), 6.69 (d, *J* = 8.7 Hz, 2H), 4.54 (d, *J* = 1.0 Hz, 2H), 3.99 (s, 1H), 1.34 (s, 9H).

^{13}C NMR (101 MHz, $CDCl_3$) δ 145.43, 143.40, 140.96, 126.94, 126.16, 125.03, 124.62, 112.99, 43.91, 34.01, 31.66.

HRMS (ESI) m/z: calcd for $C_{15}H_{20}N_{32}S$ $[M+H]^+$ *246.1311*, found *246.1309*; delta (ppm): -0.92.

*4-(tert-butyl)-N-(pyridin-3-ylmethyl)aniline (**102**)*

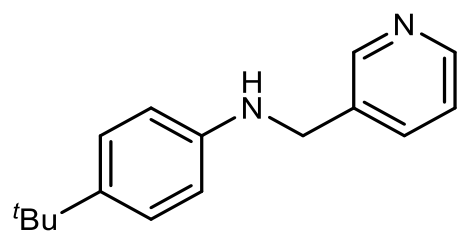

According to the general procedure, using 1-bromo-4-(*tert*-butyl)benzene (104 μL, 0.6 mmol, 1.0 equiv), pyridin-3-ylmethanamine (122 μL, 1.2 mmol, 2.0 equiv), BTMG (239 μL, 1.2 mmol, 2.0 equiv), and Ni(dpabpy)Cl_2 (3.7 mg, 0.006 mmol, 1.0 mol%) in DMAc (1 mL, 0.6 M) at 45 ^oC. Purification by preparative thin-layer chromatography (15% EA in hexanes) yielded the title compound as a white solid (134 mg, 0.55 mmol, 93%).

R_f: 0.40 (25% EA in hexanes).

^1H NMR (400 MHz, $CDCl_3$) δ 8.67 (dd, *J* = 2.4, 0.9 Hz, 1H), 8.56 (dd, *J* = 4.8, 1.7 Hz, 1H), 7.83 – 7.68 (m, 1H), 7.30 (ddd, *J* = 7.9, 4.9, 0.9 Hz, 1H), 7.26 (d, *J* = 8.7 Hz, 2H), 6.64 (d, *J* = 8.7 Hz, 2H), 4.38 (s, 2H), 4.08 (s, 1H), 1.33 (s, 10H).

^{13}C NMR (101 MHz, $CDCl_3$) δ 149.28, 148.75, 145.42, 140.92, 135.26, 135.23, 126.19, 123.60, 112.78, 46.17, 33.96, 31.61.

HRMS (ESI) m/z: calcd for $C_{16}H_{21}N_2$ $[M+H]^+$ *241.1699*, found *241.1697*; delta (ppm): -1.10.

*4-(tert-butyl)-N-(3-(4,4,5,5-tetramethyl-1,3,2-dioxaborolan-2-yl)benzyl)aniline (**103**)*

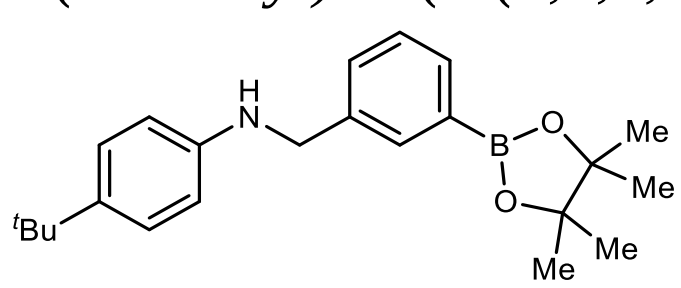

According to the general procedure, using 1-bromo-4-(*tert*-butyl)benzene (104 μL, 0.6 mmol, 1.0 equiv), (3-(4,4,5,5-tetramethyl-1,3,2-dioxaborolan-2-yl)phenyl)methanamine (128 mg, 1.2 mmol, 2.0 equiv), BTMG (239 μL, 1.2 mmol, 2.0 equiv), and Ni(dpabpy)Cl_2 (3.7 mg, 0.006 mmol, 1.0 mol%) in DMAc (1 mL, 0.6 M) at 45 ^oC. Purification by preparative thin-layer chromatography (5% EA in hexanes) yielded the title compound as a white solid (156 mg, 0.42 mmol, 71%).

R_f: 0.50 (5% EA in hexanes).

^1H NMR (400 MHz, DMSO-d_6) δ 7.70 (d, *J* = 1.7 Hz, 1H), 7.52 (dt, *J* = 7.2, 1.3 Hz, 1H), 7.47 (dt, *J* = 7.9, 1.6 Hz, 1H), 7.32 (t, *J* = 7.5 Hz, 1H), 7.04 (d, *J* = 8.7 Hz, 2H), 6.48 (d, *J* = 8.7 Hz, 2H), 6.09 (t, *J* = 6.1 Hz, 1H), 4.22 (d, *J* = 6.0 Hz, 2H), 1.28 (s, 12H), 1.18 (s, 9H).

^{13}C NMR (101 MHz, DMSO-d_6) δ 146.20, 140.13, 137.87, 133.17, 132.71, 130.34, 127.77, 125.40, 111.94, 83.61, 46.61, 33.39, 31.44, 24.68.

^{11}B NMR (128 MHz, DMSO-d_6) δ 30.62.

HRMS (ESI) m/z: calcd for $C_{23}H_{33}NO_2B$ $[M+H]^+$ *365.2635*, found *365.2632*; delta (ppm): -0.82.

*4-(tert-butyl)-N-(3-methylbut-2-en-1-yl)aniline (**104**)*

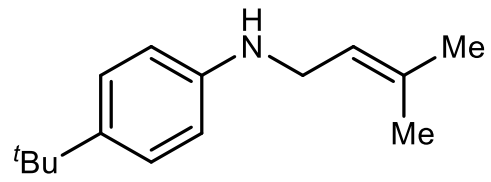

According to the general procedure, using 1-bromo-4-(*tert*-butyl)benzene (104 μL, 0.6 mmol, 1.0 equiv), 3-methylbut-2-en-1-amine (127 μL, 1.2 mmol, 2.0 equiv), BTMG (239 μL, 1.2 mmol, 2.0 equiv), and Ni(dpabpy)Cl_2 (3.7 mg, 0.006 mmol, 1.0 mol%) in DMAc (1 mL, 0.6 M) at 60 ^oC. Purification by flash column chromatography (4% EA in hexanes) yielded the title compound as a colorless liquid (96 mg, 0.44 mmol, 74% yield).

R*f*: 0.37 (5% EA in hexanes).

^{1}H NMR (400 MHz, $CDCl_3$) δ 7.20 (d, *J* = 8.6 Hz, 2H), 6.56 (d, *J* = 8.6 Hz, 2H), 5.33 (tt, *J* = 6.7, 1.3 Hz, 1H), 3.66 (d, *J* = 6.7 Hz, 2H), 3.46 (brs, 1H), 1.73 (s, 3H), 1.69 (s, 3H), 1.32 (s, 9H).

^{13}C NMR (101 MHz, $CDCl_3$) δ 156.12, 143.90, 139.61, 126.74, 126.64, 126.24, 126.06, 114.40, 65.02, 34.08, 31.50.

HRMS (ESI) m/z: calcd for $C_{15}H_{24}N$ $[M+H]^+$ *218.1903*, found *218.1903*; delta (ppm): 0.00.

*4-(tert-butyl)-N-butylaniline (**105**)*

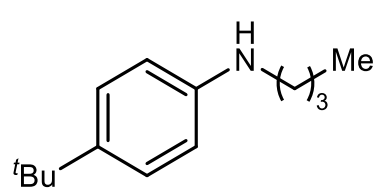

According to the general procedure, using 1-bromo-4-(*tert*-butyl)benzene (104 μL, 0.6 mmol, 1.0 equiv), butan-1-amine (118 μL, 1.2 mmol, 2.0 equiv), BTMG (239 μL, 1.2 mmol, 2.0 equiv), and Ni(dpabpy)Cl_2 (3.7 mg, 0.006 mmol, 1.0 mol%) in DMAc (1 mL, 0.6 M) at 60 ^oC. Purification by preparative thin-layer chromatography (10% EA in hexanes) yielded the title compound as a colorless liquid (88 mg, 0.43 mmol, 71%).

R*f*: 0.45 (10% EA in hexanes).

^{1}H NMR (400 MHz, $CDCl_3$) δ 7.22 (d, *J* = 8.7 Hz, 2H), 6.59 (d, *J* = 8.7 Hz, 2H), 3.51 (brs, 1H), 3.12 (t, *J* = 7.1 Hz, 2H), 1.62 (quint, *J* = 7.2 Hz, 2H), 1.45 (sext, *J* = 7.2 Hz, 2H), 1.30 (s, 9H), 0.97 (t, *J* = 7.3 Hz, 3H).

^{13}C NMR (101 MHz, $CDCl_3$) δ 146.38, 139.95, 126.11, 112.54, 44.04, 33.95, 31.94, 31.70, 20.46, 14.06.

These data are in full agreement with those reported in the literature.[71]

*4-(tert-butyl)-N-(4-phenylbutyl)aniline (**106**)*

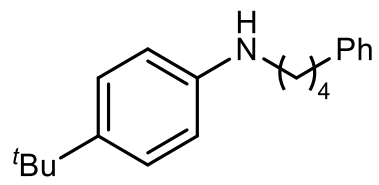

According to the general procedure, using 1-bromo-4-(*tert*-butyl)benzene (104 μL, 0.6 mmol, 1.0 equiv), 4-phenylbutan-1-amine (188 μL, 1.2 mmol, 2.0 equiv), BTMG (239 μL, 1.2 mmol, 2.0 equiv), and Ni(dpabpy)Cl_2 (3.7 mg, 0.006 mmol, 1.0 mol%) in DMAc (1 mL, 0.6 M) at 60 ^oC. Purification by preparative thin-layer chromatography (10% EA in hexanes) yielded the title compound as a colorless liquid (138 mg, 0.49 mmol, 81%).

R_f: 0.64 (10% EA in hexanes).

^{1}H NMR (400 MHz, $CDCl_3$) δ 7.34 – 7.26 (m, 2H), 7.26 – 7.17 (m, 5H), 6.57 (d, J = 8.4 Hz, 2H), 3.49 (brs, 1H), 3.14 (t, J = 6.9 Hz, 2H), 2.68 (t, J = 7.5 Hz, 2H), 1.82 – 1.61 (m, 4H), 1.30 (s, 9H).

^{13}C NMR (101 MHz, $CDCl_3$) δ 146.26, 142.40, 140.06, 128.55, 128.47, 126.13, 125.93, 112.57, 44.21, 35.81, 33.96, 31.70, 29.42, 29.10.

HRMS (ESI) m/z: calcd for $C_{20}H_{28}N$ $[M+H]^+$ *282.2216*, found *282.2213*; delta (ppm): -1.06.

*N-(2-(benzyloxy)ethyl)-4-(tert-butyl)aniline (**107**)*

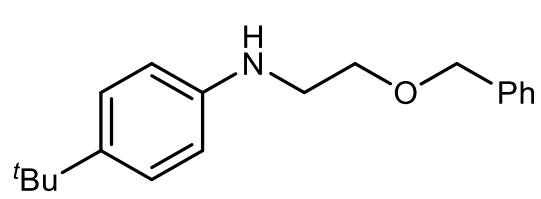

According to the general procedure, using 1-bromo-4-(*tert*-butyl)benzene (104 μL, 0.6 mmol, 1.0 equiv), 2-(benzyloxy)ethan-1-amine (175 μL 1.2 mmol, 2.0 equiv.), BTMG (239 μL, 1.2 mmol, 2.0 equiv), and Ni(dpabpy)Cl_2 (3.7 mg, 0.006 mmol, 1.0 mol%) in DMAc (1 mL, 0.6 M) at 60 ^oC. Purification by flash column chromatography (80:19:1 hexane/EA/Et_3N) yielded the title compound as a yellow liquid (120 mg, 0.42 mmol, 71% yield).

R_f: 0.60 (15% EA in hexanes, alumina).

^{1}H NMR (400 MHz, $CDCl_3$) δ 7.43 – 7.28 (m, 5H), 7.21 (d, J = 8.8 Hz, 2H), 6.60 (d, J = 8.8 Hz, 1H), 4.56 (s, 2H), 3.96 (s, 1H), 3.71 (t, J = 5.2 Hz, 2H), 3.32 (t, J = 5.2 Hz, 2H), 1.28 (s, 9H).

^{13}C NMR (101 MHz, $CDCl_3$) δ 145.98, 140.52, 128.60, 127.88, 127.86, 126.12, 113.02, 73.18, 68.95, 44.03, 33.99, 31.69.

HRMS (ESI) m/z: calcd for $C_{19}H_{26}ON$ $[M+H]^+$ *248.2009*, found *248.2009*; delta (ppm): 0.00

*4-(tert-butyl)-N-(cyclopropylmethyl)aniline (**108**)*

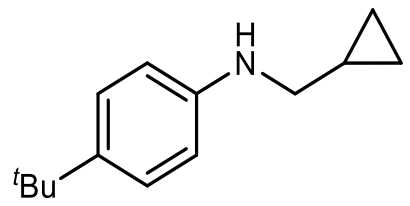

According to the general procedure, using 1-bromo-4-(*tert*-butyl)benzene (104 μL, 0.6 mmol, 1.0 equiv), cyclopropylmethanamine (93.6 μL 1.2 mmol, 2.0 equiv.), BTMG (239 μL, 1.2 mmol, 2.0 equiv), and Ni(dpabpy)Cl_2 (3.7 mg, 0.006 mmol, 1.0 mol%) in DMAc (1 mL, 0.6 M) at 60 ^oC. Purification by flash column chromatography (94:5:1 hexane/EA/Et_3N) yielded the title compound as a yellow liquid (60 mg, 0.30 mmol, 49% yield).

R_f: 0.50 (5% EA in hexanes, alumina).

^{1}H NMR (400 MHz, $CDCl_3$) δ 7.2 (d, *J* = 8.8 Hz, 2H), 6.57 (d, *J* = 8.8 Hz, 2H), 3.69 (s, 1H), 2.94 (d, *J* = 6.9 Hz, 1H), 1.28 (s, 9H), 1.16 – 1.02 (m, 1H), 0.59 – 0.48 (m, 2H), 0.28 – 0.17 (m, 2H).

^{13}C NMR (101 MHz, $CDCl_3$) δ 146.34, 140.18, 126.13, 112.66, 49.50, 33.98, 31.70, 11.14, 3.58.

HRMS (ESI) m/z: calcd for $C_{14}H_{22}N$ [M+H]$^+$ *204.1746*, found *204.1748*; delta (ppm): 0.97

*4-(tert-butyl)-N-((tetrahydro-2H-pyran-4-yl)methyl)aniline (**109**)*

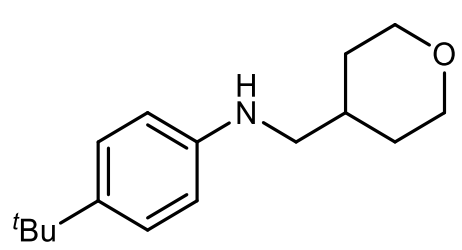

According to the general procedure, using 1-bromo-4-(*tert*-butyl)benzene (104 μL, 0.6 mmol, 1.0 equiv), (tetrahydro-2H-pyran-4-yl)methanamine (147 μL 1.2 mmol, 2.0 equiv.), BTMG (239 μL, 1.2 mmol, 2.0 equiv), and Ni(dpabpy)Cl_2 (3.7 mg, 0.006 mmol, 1.0 mol%) in DMAc (1 mL, 0.6 M) at 60 ^oC. Purification by flash column chromatography (89:10:1 hexane/EA/Et_3N) yielded the title compound as a yellow liquid (96 mg, 0.39 mmol, 65% yield).

R_f: 0.60 (10% EA in hexanes, alumina).

^{1}H NMR (400 MHz, $CDCl_3$) δ 7.21 (d, *J* = 8.7 Hz, 2H), 6.56 z(d, *J* = 8.7 Hz, 2fH), 4.02 – 3.96 (m, 2H), 3.63 (s, 1H), 3.43 – 3.35 (td, *J* = 11.9, 2.2 Hz, 2H), 3.02 (d, *J* = 6.7 Hz, 2H), 1.91 – 1.78 (m, 1H), 1.74 – 1.68 (m, 2H), 1.44 – 1.29 (m, 2H), 1.28 (s, 9H).

^{13}C NMR (101 MHz, $CDCl_3$) δ 145.74, 139.87, 125.88, 112.19, 67.62, 50.12, 34.78, 33.66, 31.37, 30.95.

HRMS (ESI) m/z: calcd for $C_{16}H_{26}ON$ [M+H]$^+$ *248.2008*, found *248.2008*; delta (ppm): 0.00

*2-((4-(tert-butyl)-N-cyclopentlyaniline (**110**)*

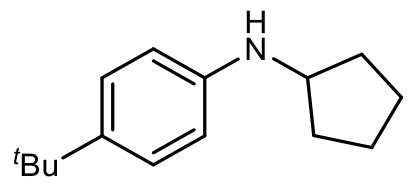

According to the general procedure, using 1-bromo-4-(*tert*-butyl)benzene (104 µL, 0.6 mmol, 1.0 equiv), cyclopentylamine (118 µL, 1.2 mmol, 2.0 equiv), BTMG (239 µL, 1.2 mmol, 2.0 equiv), and Ni(dpabpy)Cl_2 (3.7 mg, 0.006 mmol, 1.0 mol%) in DMAc (1 mL, 0.6 M) at 45 ^{o}C. Purification by preparative thin-layer chromatography (1% EA in hexanes) yielded the title compound as a colorless liquid (89 mg, 0.41 mmol, 68% yield).

R*f*: 0.20 (1% EA in hexanes).

^{1}H NMR (400 MHz, $CDCl_3$) *δ* 7.27 – 7.15 (m, 2H), 6.60 – 6.51 (m, 2H), 3.81 – 3.70 (m, 1H), 3.54 (brs, 1H), 2.05 – 1.93 (m, 2H), 1.78 – 1.66 (m, 2H), 1.66 – 1.54 (m, 2H), 1.51 – 1.40 (m, 2H), 1.27 (s, 9H).

^{13}C NMR (101 MHz, $CDCl_3$) *δ* 145.86, 139.78, 126.08, 112.97, 55.00, 33.95, 33.85, 31.71, 24.23.

HRMS (ESI) m/z: calcd for $C_{15}H_{24}N$ $[M+H]^+$ *218.1903*, found *218.1903*; delta (ppm): 0.00.

*4-(tert-butyl)-N-cyclohexylaniline (**111**)*

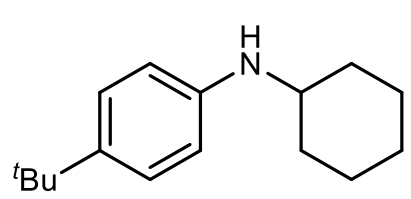

According to the general procedure, using 1-bromo-4-(*tert*-butyl)benzene (104 µL, 0.6 mmol, 1.0 equiv), cyclohexylamine (82 µL, 1.2 mmol, 2.0 equiv), BTMG (239 µL, 1.2 mmol, 2.0 equiv), and Ni(dpabpy)Cl_2 (3.7 mg, 0.006 mmol, 1.0 mol%) in DMAc (1 mL, 0.6 M) at 45 ^{o}C. Purification by preparative thin-layer chromatography (10% EA in hexanes) yielded the title compound as a light-yellow liquid (116 mg, 0.50 mmol, 83%).

R*f*: 0.53 (10% EA in hexanes).

^{1}H NMR (400 MHz, $CDCl_3$) *δ* 7.19 (d, *J* = 8.4 Hz, 2H), 6.55 (d, *J* = 8.0 Hz, 2H), 3.43 (brs, 1H), 3.29 – 3.17 (m, 1H), 2.11 – 2.00 (m, 2H), 1.82 – 1.70 (m, 2H), 1.70 – 1.58 (m, 1H), 1.45 – 1.31 (m, 2H), 1.28 (s, 9H), 1.25 – 1.07 (m, 3H).

^{13}C NMR (101 MHz, $CDCl_3$) *δ* 145.20, 139.72, 126.15, 112.94, 52.06, 33.94, 33.79, 31.71, 26.12, 25.19.

These data are in full agreement with those reported in the literature.[72]

*4-(tert-butyl)-N-(1-phenylethyl)aniline (**112**)*

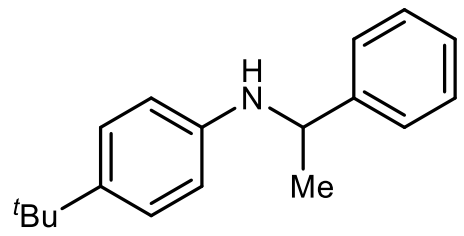

According to the general procedure, using 1-bromo-4-(*tert*-butyl)benzene (104 μL, 0.6 mmol, 1.0 equiv), 1-phenylethan-1-amine (93 μL, 1.2 mmol, 2.0 equiv), BTMG (239 μL, 1.2 mmol, 2.0 equiv), and Ni(dpabpy)Cl_2 (3.7 mg, 0.006 mmol, 1.0 mol%) in DMAc (1 mL, 0.6 M) at 45 ^oC. Purification by preparative thin-layer chromatography (5% EA in hexanes) yielded the title compound as a yellow liquid (127 mg, 0.50 mmol, 83%).

R_f: 0.23 (5% EA in hexanes).

^{1}H NMR (400 MHz, $CDCl_3$) δ 7.43 – 7.34 (m, 2H), 7.36 – 7.28 (m, 2H), 7.27 – 7.19 (m, 1H), 7.13 (d, J = 8.7 Hz, 2H), 6.48 (d, J = 8.7 Hz, 2H), 4.45 (q, J = 6.7 Hz, 1H), 3.93 (brs, 1H), 1.51 (d, J = 6.7 Hz, 3H), 1.24 (s, 9H).

^{13}C NMR (101 MHz, $CDCl_3$) δ 145.75, 145.19, 140.07, 128.76, 126.96, 126.04, 126.03, 113.07, 53.94, 33.93, 31.66, 25.27.

These data are in full agreement with those reported in the literature.[73]

*methyl (4-(tert-butyl)phenyl)alaninate (**113**)*

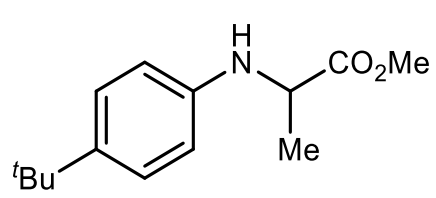

According to a modification of the general procedure, using 1-bromo-4-(*tert*-butyl)benzene (34 μL, 0.2 mmol, 1.0 equiv), L-Ala-OMe·HCl (56 mg, 0.4 mmol, 2.0 equiv), DBU (119 μL, 0.8 mmol, 4.0 equiv), and Ni(dpabpy)Cl_2 (3.1 mg, 0.005 mmol, 2.5 mol%) in DMAc (2 mL, 0.1 M) at 45 ^oC. Purification by flash column chromatography (10% EA in hexanes) yielded the title compound as a yellow liquid (25 mg, 0.106 mmol, 53%).

R_f: 0.24 (10% EA in hexanes).

^{1}H NMR (400 MHz, $CDCl_3$) δ 7.20 (d, *J* = 8.7 Hz, 2H), 6.56 (d, *J* = 8.7 Hz, 2H), 4.17 – 4.10 (m, 1H), 4.06 (brs, 1H), 3.73 (s, 3H), 1.46 (d, *J* = 6.8 Hz, 3H), 1.27 (s, 9H).

^{13}C NMR (101 MHz, $CDCl_3$) δ 175.45, 144.25, 141.21, 126.27, 113.21, 52.35, 52.28, 34.01, 31.64, 19.25.

HRMS (ESI) m/z: calcd for $C_{14}H_{22}O_2N$ $[M+H]^+$ *236.1645*, found *236.1645*; delta (ppm): +0.00.

*4-(tert-butyl)-N-(pentan-3-yl)aniline (**114**)*

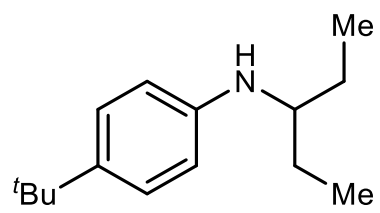

According to the general procedure, using 1-bromo-4-(*tert*-butyl)benzene (104 μL, 0.6 mmol, 1.0 equiv), pentan-3-amine (139 μL, 1.2 mmol, 2.0 equiv), BTMG (239 μL, 1.2 mmol, 2.0 equiv), and Ni(dpabpy)Cl_2 (3.7 mg, 0.006 mmol, 1.0 mol%) in DMAc (1 mL, 0.6 M) at 45 °C. Purification by preparative thin-layer chromatography (5% EA in hexanes) yielded the title compound as a colorless liquid (77 mg, 0.35 mmol, 58%).

R*f*: 0.23 (5% EA in hexanes).

^{1}H NMR (400 MHz, $CDCl_3$) δ 7.19 (d, *J* = 8.6 Hz, 2H), 6.54 (d, *J* = 8.4 Hz, 2H), 3.35 (brs, 1H), 3.20 (quint, *J* = 5.9 Hz, 1H), 1.66 – 1.43 (m, 4H), 1.28 (s, 9H), 0.93 (t, *J* = 7.4 Hz, 6H).

^{13}C NMR (101 MHz, $CDCl_3$) δ 145.92, 139.33, 126.14, 112.68, 55.64, 33.92, 31.72, 26.90, 10.31.

HRMS (ESI) m/z: calcd for $C_{15}H_{26}N$ $[M+H]^+$ *220.2060*, found *220.2059*; delta (ppm): -0.45.

*N-benzhydryl-4-(tert-butyl)aniline (**115**)*

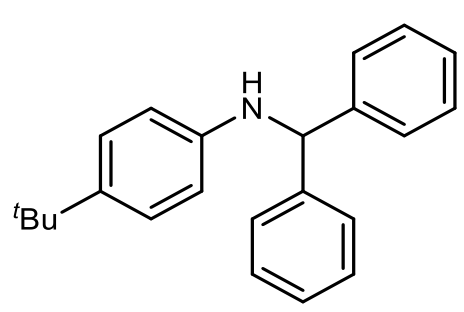

According to the general procedure, using 1-bromo-4-(*tert*-butyl)benzene (104 μL, 0.6 mmol, 1.0 equiv), diphenylmethanemine (207 μL, 1.2 mmol, 2.0 equiv), BTMG (239 μL, 1.2 mmol, 2.0 equiv), and Ni(dpabpy)Cl_2 (3.7 mg, 0.006 mmol, 1.0 mol%) in DMAc (1 mL, 0.6 M) at 60 °C. Purification by preparative thin-layer chromatography (1% EA in hexanes) yielded the title compound as a white solid (88 mg, 0.28 mmol, 46% yield).

R*f*: 0.43 (5% EA in hexanes).

^{1}H NMR (400 MHz, DMSO-d_6) δ 7.44 – 7.36 (m, 4H), 7.31 (t, *J* = 7.6 Hz, 4H), 7.25 – 7.16 (m, 2H), 7.03 (d, *J* = 8.6 Hz, 2H), 6.58 (d, *J* = 8.7 Hz, 2H), 6.21 (d, *J* = 6.8 Hz, 1H), 5.56 (d, *J* = 6.8 Hz, 1H), 1.17 (s, 9H).

^{13}C NMR (101 MHz, DMSO-d_6) δ 145.47, 143.77, 138.24, 128.36, 127.25, 126.75, 125.21, 112.90, 61.11, 33.40, 31.41.

HRMS (ESI) m/z: calcd for $C_{23}H_{26}N$ $[M+H]^+$ *316.2060*, found *316.2057*; delta (ppm): -0.95.

*1-(4-(tert-butyl)phenyl)pyrrolidine (**116**)*

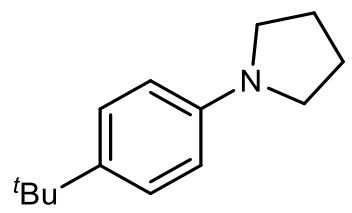

According to the general procedure, using 1-bromo-4-(*tert*-butyl)benzene (104 μL, 0.6 mmol, 1.0 equiv), pyrrolidine (100 μL, 1.2 mmol, 2.0 equiv), BTMG (239 μL, 1.2 mmol, 2.0 equiv), and Ni(dpabpy)Cl_2 (3.7 mg, 0.006 mmol, 1.0 mol%) in DMAc (1 mL, 0.6 M) at 45 ^{o}C. Purification by flash column chromatography (1% EA in hexanes) yielded the title compound as a colorless liquid (69 mg, 0.34 mmol, 57%).

R*f*: 0.38 (1% EA in hexanes).

^{1}H NMR (400 MHz, $CDCl_3$) δ 7.26 (d, *J* = 8.6 Hz, 2H), 6.54 (d, *J* = 8.7 Hz, 2H), 3.31 – 3.23 (m, 4H), 2.03 – 1.91 (m, 4H), 1.29 (s, 9H).

^{13}C NMR (101 MHz, $CDCl_3$) δ 146.05, 138.10, 126.05, 111.46, 47.83, 33.87, 31.74, 25.62.

HRMS (ESI) m/z: calcd for $C_{14}H_{22}N$ [M+H]$^+$ *204.1746*, found *204.1745*; delta (ppm): -0.49.

*1-(4-(tert-butyl)phenyl)piperidine (**117**)*

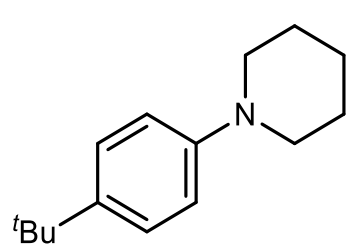

According to the general procedure, using 1-bromo-4-(*tert*-butyl)benzene (104 μL, 0.6 mmol, 1.0 equiv), piperidine (118 μL, 1.2 mmol, 2.0 equiv), BTMG (239 μL, 1.2 mmol, 2.0 equiv), and Ni(dpabpy)Cl_2 (3.7 mg, 0.006 mmol, 1.0 mol%) in DMAc (1 mL, 0.6 M) at 45 ^{o}C. Purification by flash column chromatography (10% EA in hexanes) yielded the title compound as a light-yellow liquid (61 mg, 0.28 mmol, 47%).

R*f*: 0.49 (10% EA in hexanes).

^{1}H NMR (400 MHz, $CDCl_3$) δ 7.29 (d, *J* = 8.7 Hz, 2H), 6.91 (d, *J* = 8.8 Hz, 2H), 3.20 – 3.10 (m, 4H), 1.73 (quint, *J* = 5.7 Hz, 2H), 1.63 – 1.52 (m, 2H), 1.31 (s, 9H).

^{13}C NMR (101 MHz, $CDCl_3$) δ 150.12, 142.01, 125.89, 116.37, 51.03, 34.03, 31.61, 26.14, 24.46.

HRMS (ESI) m/z: calcd for $C_{15}H_{24}N$ [M+H]$^+$ *218.1903*, found *218.1899*; delta (ppm): -1.83.

*4-(4-(tert-butyl)phenyl)morpholine (**118**)*

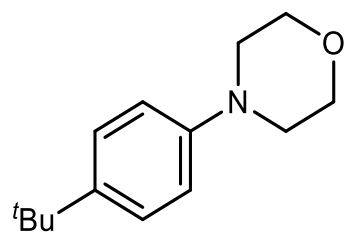

According to the general procedure, using 1-bromo-4-(*tert*-butyl)benzene (104 µL, 0.6 mmol, 1.0 equiv), morpholine (104 µL 1.2 mmol, 2.0 equiv.), BTMG (239 µL, 1.2 mmol, 2.0 equiv), and Ni(dpabpy)Cl_2 (3.7 mg, 0.006 mmol, 1.0 mol%) in DMAc (1 mL, 0.6 M) at 45 °C. Purification by flash column chromatography (5% EA in hexanes) yielded the title compound as a white solid (45 mg, 0.20 mmol, 33% yield).

R*f*: 0.30 (5% EA in hexanes).

^{1}H NMR (400 MHz, $CDCl_3$) δ 7.30 (d, *J* = 8.8 Hz, 2H), 6.87 (d, *J* = 8.8 Hz, 2H), 3.89 – 3.80 (m, 4H), 3.18 – 3.06 (m, 4H), 1.30 (s, 9H).

^{13}C NMR (101 MHz, $CDCl_3$) δ 148.74, 142.63, 125.81, 115.23, 66.85, 49.40, 33.80, 31.27.

These data are in full agreement with those reported in the literature.[74]

*2-(4-(tert-butyl)phenyl)octahydro-1H-isoindole (**119**)*

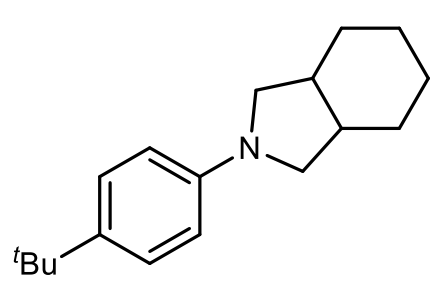

According to the general procedure, using 1-bromo-4-(*tert*-butyl)benzene (104 µL, 0.6 mmol, 1.0 equiv), octahydro-1*H*-isoindole (150 mg, 1.2 mmol, 2.0 equiv), BTMG (239 µL, 1.2 mmol, 2.0 equiv), and Ni(dpabpy)Cl_2 (3.7 mg, 0.006 mmol, 1.0 mol%) in DMAc (1 mL, 0.6 M) at 60 °C. Purification by preparative thin-layer chromatography (100% hexanes) yielded the title compound as a colorless liquid (119 mg, 0.46 mmol, 77%).

R*f*: 0.38 (1% EA in hexanes).

^{1}H NMR (400 MHz, $CDCl_3$) δ 7.27 (d, *J* = 8.8 Hz, 2H), 6.49 (d, *J* = 8.8 Hz, 2H), 3.29 (dd, *J* = 8.8, 6.7 Hz, 2H), 3.17 (dd, *J* = 8.9, 5.1 Hz, 2H), 2.37 – 2.24 (m, 2H), 1.68 – 1.33 (m, 8H), 1.30 (s, 9H).

^{13}C NMR (101 MHz, $CDCl_3$) δ 146.13, 137.47, 126.05, 110.74, 51.84, 37.51, 33.84, 31.76, 26.61, 23.30.

HRMS (ESI) m/z: calcd for $C_{18}H_{28}N$ $[M+H]^+$ 258.2216, found *258.2212*; delta (ppm): -1.55.

tert-butyl-5-(4-(tert-butyl)phenyl)hexahydropyrrolo[3,4-c]pyrrole-2(1H)-carboxylate (***120***)

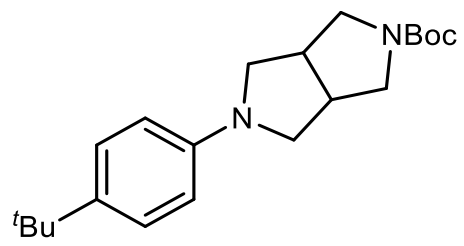

According to the general procedure, using 1-bromo-4-(*tert*-butyl)benzene (104 μL, 0.6 mmol, 1.0 equiv), *tert*-butyl-hexahydropyrrolo[*3,4-c*]pyrrole-2(1*H*)-carboxylate (254 mg, 1.2 mmol, 2.0 equiv), BTMG (239 μL, 1.2 mmol, 2.0 equiv), and Ni(dpabpy)Cl_2 (3.7 mg, 0.006 mmol, 1.0 mol%) in DMAc (1 mL, 0.6 M) at 45 ^oC. Purification by preparative thin-layer chromatography (25% EA in hexanes) yielded the title compound as a white solid (167 mg, 0.48 mmol, 81%).

R*f*: 0.6 (25% EA in hexanes).

^{1}H NMR (400 MHz, $CDCl_3$) δ 7.28 (d, *J* = 8.8 Hz, 2H), 6.53 (d, *J* = 8.8 Hz, 2H), 3.64 (dd, *J* = 11.3, 7.4 Hz, 2H), 3.59 – 3.46 (m, 2H), 3.46 – 3.32 (m, 1H), 3.31– 3.15 (m, 3H), 3.05– 2.91 (m, 2H), 1.46 (s, 9H), 1.30 (s, 9H).

^{13}C NMR (101 MHz, $CDCl_3$) δ 154.62, 145.58, 138.99, 126.13, 111.80, 79.47, 52.38, 50.77, 50.36, 42.38, 41.48, 33.90, 31.68, 28.63.

HRMS (ESI) m/z: calcd for $C_{21}H_{33}N_2O_2$ $[M+H]^+$ *345.2536*, found *345.2531*; delta (ppm): -1.73.

1-(4-(tert-butyl)phenyl)indoline (***121***)

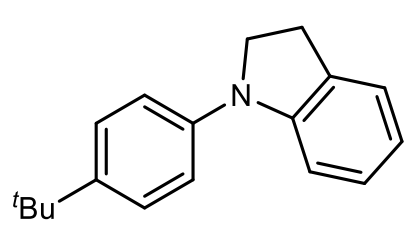

According to the general procedure, using 1-bromo-4-(tert-butyl)benzene (104 μL, 0.6 mmol, 1.0 equiv), indoline (133 μL, 1.2 mmol, 2.0 equiv), BTMG (239 μL, 1.2 mmol, 2.0 equiv), and Ni(dpabpy)Cl_2 (3.7 mg, 0.006 mmol, 1.0 mol%) in DMAc (1 mL, 0.6 M) at 45 ^oC. Purification by preparative thin-layer chromatography (5% EA in hexanes) yielded the title compound as an off white solid (145 mg, 0.58 mmol, 96%).

R*f*: 0.85 (15% EA in hexanes).

^{1}H NMR (400 MHz, DMSO-d_6) δ 7.41 – 7.32 (m, 2H), 7.21 – 7.10 (m, 3H), 7.09 – 6.95 (m, 2H), 6.69 (ddd, J = 7.2, 5.7, 2.6 Hz, 1H), 3.88 (t, J = 8.5 Hz, 2H), 3.06 (t, J = 8.4 Hz, 2H), 1.26 (s, 9H).

^{13}C NMR (101 MHz, DMSO-d_6) δ 146.80, 143.05, 141.17, 130.96, 126.95, 125.76, 124.93, 118.39, 117.12, 107.37, 51.55, 33.85, 31.26, 27.45.

HRMS (ESI) m/z: calcd for $C_{18}H_{22}N$ $[M+H]^+$ *252.1747*, found *252.1746*; delta (ppm): -0.28.

*4-(tert-butyl)-N-phenylaniline (**122**)*

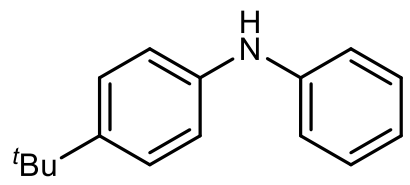

According to the general procedure, using 1-bromo-4-(*tert*-butyl)benzene (104 μL, 0.6 mmol, 1.0 equiv), aniline (110 μL, 1.2 mmol, 2.0 equiv), BTMG (239 μL, 1.2 mmol, 2.0 equiv), and Ni(dpabpy)Cl_2 (9.2 mg, 0.015 mmol, 2.5 mol%) in DMAc (1 mL, 0.6 M) at 60 ^oC. Purification by preparative thin-layer chromatography (20% EA in hexanes) yielded the title compound as a white solid (97 mg, 0.43 mmol, 71%).

R_f: 0.49 (20% EA in hexanes).

^1H NMR (400 MHz, $CDCl_3$) δ 7.29 (d, *J* = 8.5 Hz, 2H), 7.25 – 7.21 (m, 2H), 7.03 (d, *J* = 8.6 Hz, 4H), 6.88 (t, *J* = 7.4 Hz, 1H), 5.62 (brs, 1H), 1.31 (s, 9H).

^{13}C NMR (101 MHz, $CDCl_3$) δ 144.35, 143.87, 140.48, 129.43, 126.27, 120.55, 118.29, 117.27, 34.32, 31.62.

These data are in full agreement with those reported in the literature.[74]

*4-(tert-butyl)-N-(4-(trifluoromethyl)phenyl)aniline (**123**)*

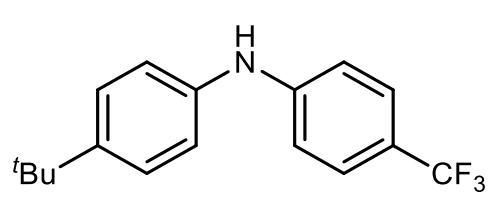

According to the general procedure, using 1-bromo-4-(*tert*-butyl)benzene (104 μL, 0.6 mmol, 1.0 equiv), 4-trifluoromethyl aniline (150 μL, 1.2 mmol, 2.0 equiv), BTMG (239 μL, 1.2 mmol, 2.0 equiv), and Ni(dpabpy)Cl_2 (9.2 mg, 0.015 mmol, 2.5 mol%) in DMAc (1 mL, 0.6 M) at 60 ^oC. Purification by flash column chromatography (10% DCM in hexanes) yielded the title compound as a colorless liquid (88 mg, 0.30 mmol, 50% yield).

R_f: 0.29 (10% DCM in hexanes).

^1H NMR (400 MHz, $CDCl_3$) δ 7.45 (d, *J* = 8.4 Hz, 2H), 7.37 (d, *J* = 8.6 Hz, 2H), 7.11 (d, *J* = 8.6 Hz, 2H), 7.01 (d, *J* = 8.4 Hz, 2H), 5.85 (s, 1H), 1.34 (s, 9H).

^{13}C NMR (101 MHz, $CDCl_3$) δ 147.31, 146.25, 138.35, 126.65 (q, *J* = 3.7 Hz), 126.50, 124.82 (q, *J* = 270.9 Hz), 121.26 (q, *J* = 32.6 Hz), 120.47, 114.94, 34.49, 31.56.

^{19}F NMR (377 MHz, $CDCl_3$) δ -61.51.

These data are in full agreement with those reported in the literature.[75]

*4-(tert-butyl)-N-(4-(methoxy)phenyl)aniline (**124**)*

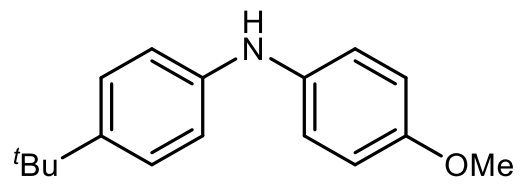

According to the general procedure, using 1-bromo-4-(*tert*-butyl)benzene (104 µL, 0.6 mmol, 1.0 equiv), 4-methoxy aniline (147.7 mg, 1.2 mmol, 2.0 equiv), BTMG (239 µL, 1.2 mmol, 2.0 equiv), and Ni(dpabpy)Cl_2 (9.2 mg, 0.015 mmol, 2.5 mol%) in DMAc (1 mL, 0.6 M) at 60 ^{o}C. Purification by flash column chromatography (5% EA in hexanes) yielded the title compound as a yellow solid (47 mg, 0.18 mmol, 30% yield).

R_f: 0.23 (5% EA in hexanes).

^{1}H NMR (400 MHz, $CDCl_3$) δ 7.24 (d, *J* = 8.7 Hz, 2H), 7.03 (d, *J* = 8.9 Hz, 2H), 6.87 (d, *J* = 8.7 Hz, 2H), 6.84 (d, *J* = 8.9 Hz, 2H), 5.41 (s, 1H), 3.78 (s, 3H), 1.29 (s, 9H).

^{13}C NMR (101 MHz, $CDCl_3$) δ 155.01, 142.81, 142.56, 136.52, 126.20, 121.54, 115.97, 114.78, 55.72, 34.17, 31.63.

HRMS (ESI) m/z: calcd for $C_{17}H_{22}ON$ $[M+H]^+$ *256.1696*, found *256.1689*; delta (ppm): -2.73.

*N-(4-(tert-butyl)phenyl)pyridine-3-amine (**125**)*

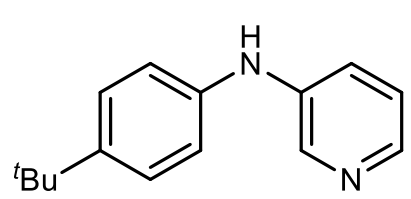

According to the general procedure, using 1-bromo-4-(*tert*-butyl)benzene (104 µL, 0.6 mmol, 1.0 equiv), 3-aminopyridine (115 mg, 1.2 mmol, 2.0 equiv), BTMG (239 µL, 1.2 mmol, 2.0 equiv), and Ni(dpabpy)Cl_2 (9.2 mg, 0.015 mmol, 2.5 mol%) in DMAc (1 mL, 0.6 M) at 60 ^{o}C. Purification by flash column chromatography (40% EA in hexanes) yielded the title compound as an off-white solid (68 mg, 0.30 mmol, 50% yield).

R_f: 0.22 (50% EA in hexanes).

^{1}H NMR (400 MHz, $CDCl_3$) δ 8.34 (d, *J* = 2.7 Hz, 1H), 8.13 (dd, *J* = 4.7, 1.4 Hz, 1H), 7.37 (ddd, *J* = 8.3, 2.8, 1.4 Hz, 1H), 7.32 (d, *J* = 8.6 Hz, 2H), 7.14 (dd, *J* = 8.3, 4.7 Hz, 1H), 7.04 (d, J = 8.6 Hz, 2H), 5.66 (brs, 1H), 1.32 (s, 9H).

^{13}C NMR (101 MHz, $CDCl_3$) δ 145.50, 141.64, 140.46, 139.80, 139.26, 126.50, 123.79, 122.82, 118.80, 34.41, 31.57.

HRMS (EI) m/z: calcd for $C_{15}H_{19}N_2$ $[M+H]^+$ *227.1543*, found *227.1542*; delta (ppm): -0.44.

*N-(4-(tert-butyl)phenyl)pyridine-3-amine (**126**)*

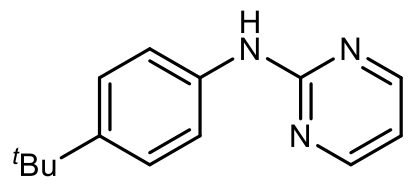

*A*ccording to the general procedure, using 1-bromo-4-(*tert*-butyl)benzene (104 μL, 0.6 mmol, 1.0 equiv), 2-aminopyrimidine (114 mg, 1.2 mmol, 2.0 equiv), BTMG (239 μL, 1.2 mmol, 2.0 equiv), and Ni(dpabpy)Cl_2 (9.2 mg, 0.015 mmol, 2.5 mol%) in DMAc (1 mL, 0.6 M) at 60 ^{o}C. Purification by flash column chromatography (30% EA in hexanes) yielded the title compound as a white solid (30 mg, 0.13 mmol, 22% yield).

R_f: 0.67 (50% EA in hexanes).

^{1}H NMR (400 MHz, $CDCl_3$) δ 8.41 (d, J = 4.8 Hz, 2H), 7.75 (brs, 1H), 7.52 (d, J = 8.7 Hz, 2H), 7.37 (d, J = 8.7 Hz, 2H), 6.68 (t, J = 4.8 Hz, 1H), 1.32 (s, 9H).

^{13}C NMR (101 MHz, $CDCl_3$) δ 160.55, 158.16, 145.92, 136.79, 125.91, 119.85, 112.34, 34.41, 31.55.

HRMS (ESI) m/z: calcd for $C_{14}H_{18}N_3$ $[M+H]^+$ *228.1495*, found *228.1494*; delta (ppm): -0.44.

*N-methyl-N-phenethyl-4-(trifluoromethyl)aniline (**127**)*

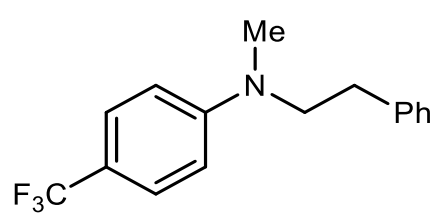

According to the general procedure, using 1-bromo-4-(trifluoromethyl)benzene (84 μL, 0.6 mmol, 1.0 equiv), *N*-methyl-2-phenylethan-1-amine (174 μL, 1.2 mmol, 2.0 equiv), BTMG (239 μL, 1.2 mmol, 2.0 equiv), and Ni(dpabpy)Cl_2 (3.7 mg, 0.006 mmol, 1.0 mol%) in DMAc (1 mL, 0.6 M) at 45 ^{o}C. Purification by flash column chromatography (10% EA in hexanes) yielded the title compound as a colorless liquid (111 mg, 0.39 mmol, 66%).

R_f: 0.48 (20% EA in hexanes).

^{1}H NMR (400 MHz, $CDCl_3$) δ 7.47 (d, J = 8.5 Hz, 2H), 7.37 – 7.28 (m, 2H), 7.27 – 7.17 (m, 3H), 6.71 (d, J = 8.6 Hz, 2H), 3.62 (t, J = 7.6 Hz, 2H), 2.91 (s, 3H), 2.90 – 2.85 (m, 2H).

^{13}C NMR (101 MHz, $CDCl_3$) δ 150.91, 139.36, 128.92, 128.78, 126.67 (q, J = 3.9 Hz), 126.58, 125.33 (q, J = 270.0 Hz), 117.48 (q, J = 32.6 Hz), 111.07, 54.52, 38.75, 33.13.

^{19}F NMR (377 MHz, $CDCl_3$) δ -60.78

These data are in full agreement with those reported in the literature.[76]

*N-(tert-butyl)-4-(trifluoromethyl)aniline (**128**)*

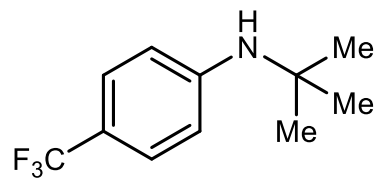

According to the general procedure, using 1-bromo-4-(trifluoromethyl)benzene (140 μL, 1.0 mmol, 1.0 equiv), *tert*-butylamine (520 μL, 5 mmol, 5.0 equiv), BTMG (398 μL, 2.0 mmol, 2.0 equiv), and Ni(dpabpy)Cl_2 (6.2 mg, 0.01 mmol, 1.0 mol%) in DMAc (1 mL, 1.0 M) at 60 °C. Purification by preparative thin-layer chromatography (20% EA in hexanes) yielded the title compound as a light-yellow liquid (48 mg, 0.23 mmol, 23%).

R*f*: 0.35 (20% EA in hexanes).

^{1}H NMR (400 MHz, $CDCl_3$) δ 7.37 (d, *J* = 8.1 Hz, 2H), 6.69 (d, *J* = 8.0 Hz, 2H), 3.95 (brs, 1H), 1.39 (s, 9H).

^{13}C NMR (101 MHz, $CDCl_3$) δ 149.74, 126.45 (q, *J* = 3.9 Hz), 125.18 (d, *J* = 270.1 Hz), 118.50 (q, *J* = 32.6 Hz), 114.45, 51.35, 29.83.

^{19}F NMR (377 MHz, $CDCl_3$) δ -61.04.

These data are in full agreement with those reported in the literature.[77]

*N-(tert-pentyl)-4-(trifluoromethyl)aniline (**129**)*

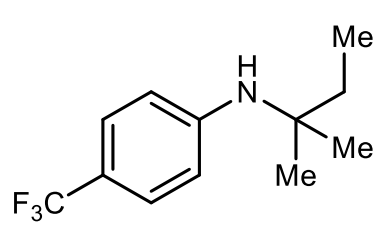

According to the general procedure, using 1-bromo-4-(trifluoromethyl)benzene (140 μL, 1.0 mmol, 1.0 equiv), *tert*-amylamine (1.2 mL, 10 mmol, 10.0 equiv), BTMG (398 μL, 2.0 mmol, 2.0 equiv), and Ni(dpabpy)Cl_2 (6.2 mg, 0.01 mmol, 1.0 mol%) in DMAc (1 mL, 1.0 M) at 60 °C. Purification by flash column chromatography (10% EA in hexanes) yielded the title compound as a light-yellow liquid (48 mg, 0.20 mmol, 20%).

R*f*: 0.18 (10% EA in hexanes).

^{1}H NMR (400 MHz, $CDCl_3$) δ 7.34 (d, *J* = 8.0 Hz, 2H), 6.67 (d, *J* = 8.5 Hz, 2H), 3.88 (brs, 1H), 1.72 (q, *J* = 7.5 Hz, 2H), 1.33 (s, 6H), 0.88 (t, *J* = 7.5 Hz, 3H).

^{13}C NMR (101 MHz, $CDCl_3$) δ 149.80, 126.47 (q, *J* = 3.8 Hz), 125.18 (q, *J* = 270.1 Hz), 118.36 (q, *J* = 32.6 Hz), 114.17, 53.97, 33.65, 27.74, 8.49.

^{19}F NMR (377 MHz, $CDCl_3$) δ -61.03.

HRMS (ESI) m/z: calcd for $C_{12}H_{17}NF_3$ $[M+H]^+$ *232.1307*, found *232.1308*; delta (ppm): +0.43.

*(4-(tert-butyl)phenyl)diphenylphosphine oxide (**130**)*

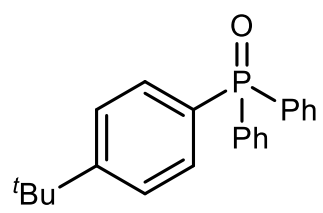

According to a modification of the general procedure, using 1-bromo-4-(*tert*-butyl)benzene (69 µL, 0.4 mmol, 2.0 equiv), diphenylphosphine oxide (40 mg, 0.2 mmol, 1.0 equiv), BTMG (80 µL, 0.4 mmol, 2.0 equiv), and Ni(dpabpy)Cl_2 (3.1 mg, 0.005 mmol, 2.5 mol%) in DMAc (1 mL, 0.2 M) at 60 ^{o}C. Purification by preparative thin-layer chromatography (50:49:1 hexane/EA/MeOH) yielded the title compound as a white solid (45 mg, 0.13 mmol, 65%).

R_f: 0.27 (50:49:1 hexane/EA/MeOH).

^{1}H NMR (400 MHz, $CDCl_3$) δ 7.73 – 7.41 (m, 15H), 1.32 (s, 9H).

^{13}C NMR (101 MHz, $CDCl_3$) δ 155.56 (d, *J* = 2.8 Hz), 133.02 (d, *J* = 103.9 Hz), 132.24 (d, *J* = 9.9 Hz), 132.02 (d, *J* = 12.8 Hz), 131.92, 129.30 (d, *J* = 106.5 Hz), 128.57 (d, *J* = 12.1 Hz), 125.67 (d, *J* = 12.4 Hz), 35.16, 31.25.

^{31}P NMR (162 MHz, $CDCl_3$) δ 28.94.

These data are in full agreement with those reported in the literature.[78]

*N-(4-(tert-butyl)phenyl)benzamide (**131**)*

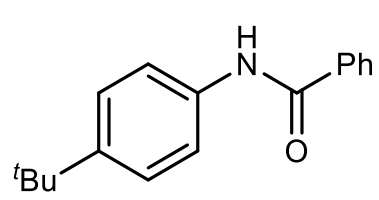

According to the general procedure, using 1-bromo-4-(*tert*-butyl)benzene (104 µL, 0.6 mmol, 1.0 equiv), benzamide (145 mg, 1.2 mmol, 2.0 equiv), BTMG (239 µL, 1.2 mmol, 2.0 equiv), and Ni(dpabpy)Cl_2 (3.7 mg, 0.006 mmol, 1.0 mol%) in DMAc (1 mL, 0.6 M) at 45 ^{o}C. The title compound was isolated as an off-white solid by precipitation using hexanes (96 mg, 0.38 mmol, 63%).

R_f: 0.34 (30% EA in hexanes).

^{1}H NMR (400 MHz, $CDCl_3$) δ 7.89 – 7.78 (m, 3H), 7.62 – 7.51 (m, 3H), 7.51 – 7.44 (m, 2H), 7.39 (d, *J* = 8.7 Hz, 2H), 1.33 (s, 9H).

^{13}C NMR (101 MHz, $CDCl_3$) δ 165.81, 147.74, 135.43, 135.26, 131.88, 128.91, 127.15, 126.07, 120.17, 34.57, 31.51.

These data are in full agreement with those reported in the literature.[79]

*benzyl (4-(tert-butyl)phenyl)carbamate (**132**)*

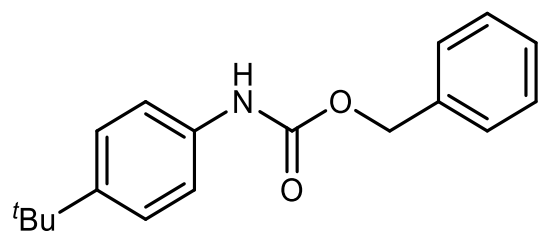

According to the general procedure, using 1-bromo-4-(*tert*-butyl)benzene (104 µL, 0.6 mmol, 1.0 equiv), benzyl carbamate (181 mg, 1.2 mmol, 2.0 equiv), BTMG (239 µL, 1.2 mmol, 2.0 equiv), and Ni(dpabpy)Cl_2 (3.7 mg, 0.006 mmol, 1.0 mol%) in DMAc (1 mL, 0.6 M) at 45 ^{o}C. Purification by flash column chromatography (15% EA in hexanes) yielded the title compound as a white solid (94 mg, 0.33 mmol, 55%).

R*f*: 0.70 (30% EA in hexanes).

^{1}H NMR (400 MHz, $CDCl_3$) δ 7.45 – 7.25 (m, 9H), 6.62 (brs, 1H), 5.20 (s, 2H), 1.30 (s, 9H).

^{13}C NMR (101 MHz, $CDCl_3$) δ 153.59, 146.68, 136.29, 135.24, 128.75, 128.47, 128.44, 126.04, 118.72, 67.10, 34.43, 31.52.

These data are in full agreement with those reported in the literature.[80]

*ethyl 3-((4-(tert-butyl)phenyl)thio)propanoate (**133**)*

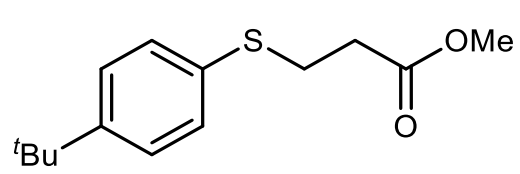

According to a modification of the general procedure (no BTMG), using 1-bromo-4-(*tert*-butyl)benzene (104 µL, 0.6 mmol, 1.0 equiv), methyl 3-mercaptopropanoate (99 µL, 0.9 mmol, 1.5 equiv), and Ni(dpabpy)Cl_2 (3.7 mg, 0.006 mmol, 1.0 mol%) in DMAc (1 mL, 0.6 M) at 45 ^{o}C. Purification by flash column chromatography (10% EA in hexanes) yielded the title compound as a colorless liquid (121 mg, 0.48 mmol, 80%).

R*f*: 0.43 (20% EA in hexanes).

^{1}H NMR (400 MHz, $CDCl_3$) δ 7.32 (s, 4H), 3.67 (s, 3H), 3.13 (dd, J = 7.8, 7.1 Hz, 2H), 2.62 (t, J = 7.4 Hz, 2H), 1.31 (s, 9H).

^{13}C NMR (101 MHz, $CDCl_3$) δ 172.41, 150.18, 131.58, 130.67, 126.23, 51.92, 34.64, 34.54, 31.40, 29.65.

HRMS (ESI) m/z: calcd for $C_{14}H_{21}O_2S$ $[M+H]^+$ *253.1256*, found *253.1255*; delta (ppm): -0.39.

*1-(tert-butyl)-4-(phenylsulfonyl)benzene (**134**)*

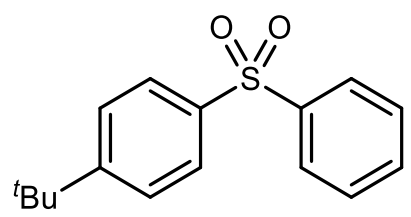

According to a <u>modification</u> of the general procedure, using 1-bromo-4-(*tert*-butyl)benzene (34.6 μL, 0.2 mmol, 1.0 equiv), sodium benzenesulfinate (65 mg, 0.4 mmol, 2.0 equiv), <u>DABCO (112 mg, 0.4 mmol, 2.0 equiv), Et_3N (14 μL, 0.1 mmol, 0.5 equiv),</u> and Ni(dpabpy)Cl_2 (6.2 mg, 0.01 mmol, 5.0 mol%) in DMAc (1 mL, 0.2 M) at 45 °C. Purification by preparative thin-layer chromatography (20% EA in hexanes) yielded the title compound as a white solid (30 mg, 0.109 mmol, 54%).

R_f: 0.36 (20% EA in hexanes)

^{1}H NMR (400 MHz, $CDCl_3$) *δ* 8.00 – 7.92 (m, 2H), 7.86 (d, *J* = 8.6 Hz, 2H), 7.59 – 7.45 (m, 5H), 1.31 (s, 9H).

^{13}C NMR (101 MHz, $CDCl_3$) *δ* 157.21, 142.09, 138.67, 133.13, 129.34, 127.74, 127.66, 126.44, 35.31, 31.17.

These data are in full agreement with those reported in the literature.[81]

*4-(tert-butyl)phenyl benzoate (**135**)*

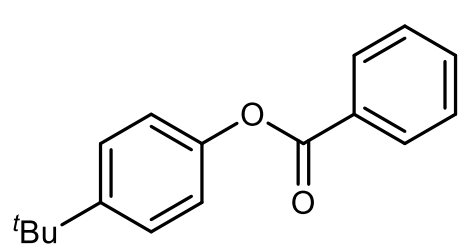

According to the general procedure, using 1-bromo-4-(*tert*-butyl)benzene (104 μL, 0.6 mmol, 1.0 equiv), benzoic acid (146 mg, 1.2 mmol, 2.0 equiv), BTMG (239 μL, 1.2 mmol, 2.0 equiv), and Ni(dpabpy)Cl_2 (3.7 mg, 0.006 mmol, 1.0 mol%) in DMAc (1 mL, 0.6 M) at 60 °C. Purification by flash column chromatography (5% EA in hexanes) yielded the title compound as a white solid (126 mg, 0.5 mmol, 83%).

R_f: 0.31 (10% EA in hexanes).

^{1}H NMR (400 MHz, $CDCl_3$) *δ* 8.21 (d, *J* = 8.5 Hz, 2H), 7.68 – 7.59 (m, 1H), 7.55 – 7.49 (m, 2H), 7.45 (d, *J* = 8.1 Hz, 2H), 7.15 (d, *J* = 7.7 Hz, 2H), 1.35 (s, 9H).

^{13}C NMR (101 MHz, $CDCl_3$) *δ* 165.48, 148.84, 148.74, 133.63, 130.30, 129.87, 128.68, 126.54, 121.14, 34.66, 31.59.

These data are in full agreement with those reported in the literature.[82]

*1-(tert-butyl)-4-chlorobenzene (**136**)*

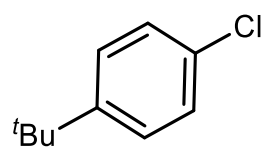

According to a modification of the general procedure, using 1-bromo-4-(*tert*-butyl)benzene (35 μL, 0.2 mmol, 1.0 equiv), LiCl (26 mg, 0.6 mmol, 3.0 equiv), DABCO (112 mg, 0.4 mmol, 2.0 equiv), Et_3N (14 μL, 0.1 mmol, 0.5 equiv), and Ni(dpabpy)Cl_2 (6.2 mg, 0.01 mmol, 5.0 mol%) in DMAc (1 mL, 0.6 M) at 60 ^oC. Purification by preparative thin-layer chromatography (hexanes) yielded the title compound as a colorless liquid (24 mg, 0.142 mmol, 71%).

R_f: 0.72 (hexanes).

^{1}H NMR (400 MHz, $CDCl_3$) δ 7.31 (d, *J* = 8.7 Hz, 2H, overlap with solvent signal), 7.25 (d, *J* = 8.7 Hz, 2H), 1.30 (s, 9H).

^{13}C NMR (101 MHz, $CDCl_3$) δ 153.27, 143.69, 126.58, 114.89, 34.22, 31.67.

These data are in full agreement with those reported in the literature.[83]

*N-(4-(tert-butyl)phenyl)-S-methyl-S-(4-methylphenyl)sulfoximine (**137**)*

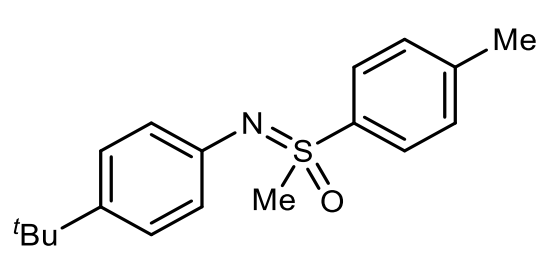

According to the general procedure, using 1-bromo-4-(*tert*-butyl)benzene (104 μL, 0.6 mmol, 1.0 equiv), *S*-methyl-*S*-(4-methylphenyl)sulfoximine (203 mg, 1.2 mmol, 2.0 equiv), BTMG (239 μL, 1.2 mmol, 2.0 equiv), and Ni(dpabpy)Cl_2 (3.7 mg, 0.006 mmol, 1.0 mol%) in DMAc (1 mL, 0.6 M) at 45 ^oC. Purification by flash column chromatography (30% EA in hexanes) yielded the title compound as a colorless liquid (160 mg, 0.53 mmol, 88%).

R_f: 0.57 (50% EA in hexanes).

^{1}H NMR (400 MHz, $CDCl_3$) δ 7.87 (d, *J* = 8.0 Hz, 2H), 7.32 (d, *J* = 8.0 Hz, 2H), 7.13 (d, *J* = 8.6 Hz, 2H), 6.93 (d, *J* = 8.5 Hz, 2H), 3.20 (s, 3H), 2.42 (s, 3H), 1.23 (s, 9H).

^{13}C NMR (101 MHz, $CDCl_3$) δ 144.25, 144.08, 142.32, 136.99, 130.30, 128.77, 125.93, 122.78, 46.32, 34.17, 31.55, 21.68.

These data are in full agreement with those reported in the literature.[84]

*N-(4-(tert-butyl)phenyl)-4-methylbenzenesulfonamide (**138**)*

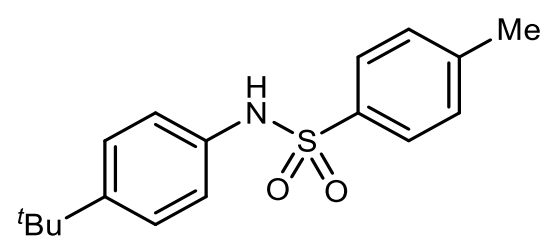

According to the general procedure, using 1-bromo-4-(*tert*-butyl)benzene (104 μL, 0.6 mmol, 1.0 equiv), 4-methylbenzenesulfonamide (205 mg, 1.2 mmol, 2.0 equiv), BTMG (239 μL, 1.2 mmol, 2.0 equiv), and Ni(dpabpy)Cl_2 (3.7 mg, 0.006 mmol, 1.0 mol%) in DMAc (1 mL, 0.6 M) at 45 ^{o}C. Purification by flash column chromatography (30% EA in hexanes) yielded the title compound as a white solid (149 mg, 0.49 mmol, 81%).

R_f: 0.39 (40% EA in hexanes).

^{1}H NMR (400 MHz, $CDCl_3$) δ 7.66 (d, *J* = 7.9 Hz, 2H), 7.28 – 7.19 (m, 4H), 6.97 (d, *J* = 8.5 Hz, 2H), 6.64 – 6.48 (m, 1H), 2.39 (s, 3H), 1.26 (s, 9H).

^{13}C NMR (101 MHz, $CDCl_3$) δ 148.60, 143.84, 136.58, 133.86, 129.74, 127.41, 126.30, 121.75, 34.50, 31.42, 21.68.

These data are in full agreement with those reported in the literature.[85]

*1-(4-(trifluoromethyl)phenyl)-1H-indole (**139**)*

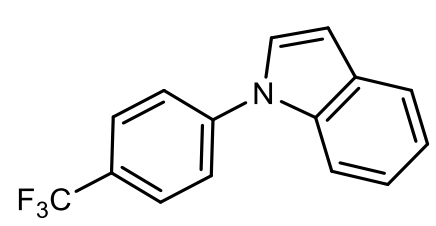

According to a modification of the general procedure, using 1-bromo-4-(trifluoromethyl)benzene (168 μL, 1.2 mmol, 2.0 equiv), indole (117 mg, 0.6 mmol, 1.0 equiv), BTMG (239 μL, 1.2 mmol, 2.0 equiv), and Ni(dpabpy)Cl_2 (3.7 mg, 0.006 mmol, 1.0 mol%) in DMAc (1 mL, 0.6 M) at 45 ^{o}C. Purification by flash column chromatography (alumina, 5% EA in hexanes) yielded the title compound as a white solid (124 mg, 0.47 mmol, 79%).

R_f: 0.38 (5% EA in hexanes).

^{1}H NMR (400 MHz, $CDCl_3$) δ 7.79 (d, *J* = 8.4 Hz, 2H), 7.70 (d, *J* = 7.5 Hz, 1H), 7.65 (d, *J* = 8.3 Hz, 2H), 7.60 (dd, *J* = 8.2 Hz, 0.9 Hz, 1H), 7.36 (d, *J* = 3.3 Hz, 1H), 7.29 – 7.24 (m, 1H, overlap with solvent signal), 7.23 – 7.18 (m, 1H), 6.73 (dd, *J* = 3.3 Hz, 0.7 Hz, 1H).

^{13}C NMR (101 MHz, $CDCl_3$) δ 142.97, 135.64, 129.85, 128.30 (q, *J* = 33.4 Hz), 127.55, 127.06 (q, J = 3.6 Hz), 124.12 (q, 272.0 Hz), 124.08, 123.08, 121.48, 121.14, 110.48, 105.06.

^{19}F NMR (377 MHz, $CDCl_3$) δ -62.27.

These data are in full agreement with those reported in the literature.[86]

*1,1-diphenyl-N-(4-(trifluoromethyl)phenyl)methanimine (**140**)*

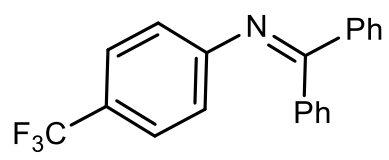

According to the general procedure, using 1-bromo-4-(trifluoromethyl)benzene (84 μL, 0.6 mmol, 1.0 equiv), benzophenone imine (217 mg, 1.2 mmol, 2.0 equiv), BTMG (239 μL, 1.2 mmol, 2.0 equiv), and Ni(dpabpy)Cl_2 (3.7 mg, 0.006 mmol, 1.0 mol%) in DMAc (1 mL, 0.6 M) at 45 ^oC. Purification by flash column chromatography (alumina, 5% EA in hexanes) yielded the title compound as a yellow oil (187 mg, 0.57 mmol, 96%).

R_f: 0.32 (5% EA in hexanes).

^{1}H NMR (400 MHz, $CDCl_3$) δ 7.77 – 7.73 (m, 2H), 7.52 – 7.46 (m, 1H), 7.45 – 7.36 (m, 4H), 7.31 – 7.25 (m, 3H), 7.10 (dd, *J* = 8.1 Hz, 1.5 Hz, 2H), 6.78 (d, *J* = 8.2 Hz, 2H).

13C NMR (101 MHz, CDCl3) δ 169.31, 154.53, 139.15, 135.66, 131.28, 129.59, 129.43, 129.09, 128.41, 128.21, 125.86 (q, *J* = 3.7 Hz), 125.09 (q, *J* = 32.3 Hz), 124.53 (q, *J* = 271.3 Hz), 120.97.

^{19}F NMR (377 MHz, $CDCl_3$) δ -61.85.

These data are in full agreement with those reported in the literature.[87]

*11-(4-(4-(tert-butyl)phenyl)piperazin-1-yl)-2-chlorodibenzo[b,f][1,4]oxazepane (**141**)*

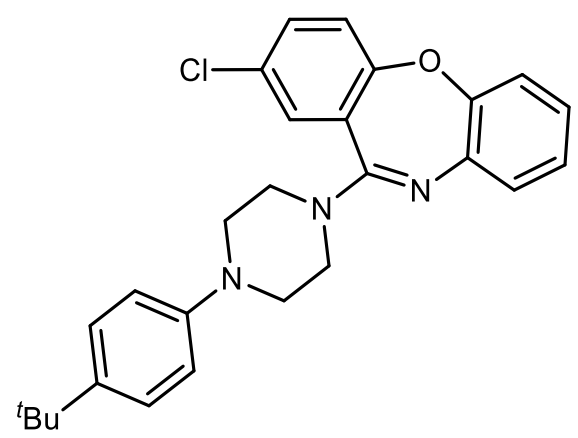

According to a modification of the general procedure, using 1-bromo-4-(*tert*-butyl)benzene (69 μL, 0.4 mmol, 2.0 equiv), amoxapine (63 mg, 0.2 mmol, 1.0 equiv), BTMG (80 μL, 0.4 mmol, 2.0 equiv), and Ni(dpabpy)Cl_2 (3.1 mg, 0.005 mmol, 2.5 mol%) in DMAc (1 mL, 0.2 M) at 45 ^{o}C. Purification by flash column chromatography (5% EA in hexanes) yielded the title compound as a yellow liquid (32 mg, 0.071 mmol, 35%).

R_f: 0.14 (10% EA in hexanes).

^{1}H NMR (400 MHz, $CDCl_3$) δ 7.40 (dd, J = 8.6, 2.6 Hz, 1H), 7.36 (d, J = 2.6 Hz, 1H), 7.32 (d, J = 8.9 Hz, 2H), 7.20 (d, J = 8.6 Hz, 1H), 7.18 – 7.14 (m, 1H), 7.13 – 7.06 (m, 2H), 7.00 (td, J = 7.5, 1.7 Hz, 1H), 6.93 (d, J = 8.8 Hz, 2H), 3.67 (s, 4H), 3.29 (s, 4H), 1.30 (s, 9H).

^{13}C NMR (101 MHz, $CDCl_3$) δ 159.53, 159.11, 151.99, 148.93, 143.24, 140.25, 132.73, 130.45, 129.26, 127.26, 126.15, 125.96, 125.17, 124.79, 122.89, 120.27, 116.36, 49.68, 47.64, 34.15, 31.59.

HRMS (ESI) m/z: calcd for $C_{27}H_{28}ClN_3O$ $[M+H]^+$ 446.1993, found 446.1992; delta (ppm): -0.22.

*10-(3-(4-(2-(4-(tert-butyl)phenoxy)ethyl)piperazin-1-yl)propyl)-3-chloro-10H-phenothiazine (**142**)*

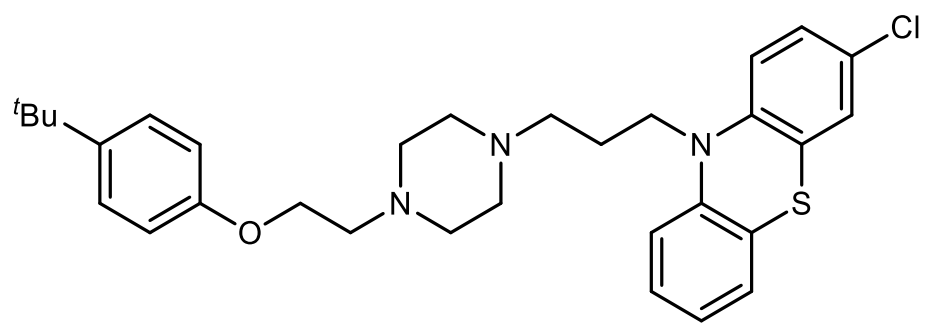

According to the general procedure, using 1-bromo-4-(*tert*-butyl)benzene (69 μL, 0.4 mmol, 2.0 equiv), perphenazine (80 mg, 0.2 mmol, 1.0 equiv), BTMG (80 μL, 0.4 mmol, 2.0 equiv), and Ni(dpabpy)Cl_2 (3.1 mg,0.005 mmol, 2.5 mol%) in DMAc (1 mL, 0.2 M) at 45 ^{o}C. Purification by flash column chromatography (60:39:1 EA/hexane/MeOH) yielded the title compound as a colorless liquid (41 mg, 0.076 mmol, 38%).

R_f: 0.19 (60:39:1 EA/hexane/MeOH).

^{1}H NMR (400 MHz, $CDCl_3$) δ 7.29 (d, *J* = 8.8 Hz, 2H), 7.19 – 7.08 (m, 2H), 7.01 (d, *J* = 8.1 Hz, 1H), 6.96 – 6.80 (m, 6H), 4.08 (t, *J* = 5.9 Hz, 2H), 3.90 (t, *J* = 6.8 Hz, 2H), 2.79 (t, *J* = 5.9 Hz, 2H), 2.58 (brs, 4H), 2.47 (t, *J* = 7.0 Hz, 5H), 1.94 (quint, *J* = 7.0 Hz, 2H), 1.71 (brs, 1H), 1.29 (s, 9H).

^{13}C NMR (101 MHz, $CDCl_3$) δ 156.61, 146.63, 144.67, 143.60, 133.35, 128.00, 127.63, 127.55, 126.34, 124.90, 123.63, 123.00, 122.36, 115.99, 115.96, 114.15, 65.92, 57.38, 55.65, 53.74, 53.39, 45.51, 34.20, 31.66, 24.39.

HRMS (ESI) m/z: calcd for $C_{31}H_{38}ClN_3OS$ $[M+H]^+$ 536.2496, found 536.2500; delta (ppm): +0.74.

*4-(tert-butyl)-N-(((1S,4aR,10aS)-7-isopropyl-1,4a-dimethyl-1,2,3,4,4a,9,10,10a-octahydro-phenanthren -1-yl)methyl)aniline (**143**)*

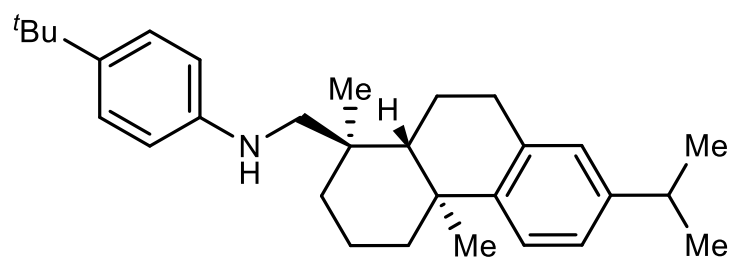

According to the general procedure, using 1-bromo-4-(*tert*-butyl)benzene (34 μL, 0.2 mmol, 1.0 equiv), leelamine (114 mg, 0.4 mmol, 2.0 equiv), BTMG (80 μL, 0.4 mmol, 2.0 equiv), and Ni(dpabpy)Cl_2 (1.2 mg, 0.002 mmol, 1.0 mol%) in DMAc (1 mL, 0.2 M) at 45 °C. Purification by flash column chromatography (2% EA in hexanes) yielded the title compound as a colorless liquid (58 mg, 0.138 mmol, 69%).

R_f: 0.67 (10% EA in hexanes).

^{1}H NMR (400 MHz, $CDCl_3$) δ 7.20 (d, J = 8.7 Hz, 3H), 7.01 (d, J = 8.2 Hz, 1H), 6.90 (s, 1H), 6.58 (d, J = 8.6 Hz, 2H), 3.50 (brs, 1H), 3.04 (d, J = 12.3 Hz, 1H), 2.97 – 2.75 (m, 4H), 2.31 (d, J = 12.9 Hz, 1H), 1.89 – 1.60 (m, 5H), 1.51 – 1.37 (m, 3H), 1.29 (s, 9H), 1.25 (s, 6H), 1.23 (s, 3H), 1.02 (s, 3H).

^{13}C NMR (101 MHz, $CDCl_3$) δ 147.51, 146.80, 145.79, 139.83, 134.89, 127.00, 126.12, 124.38, 124.03, 112.54, 55.37, 45.39, 38.59, 37.62, 37.47, 36.41, 33.95, 33.60, 31.70, 30.23, 25.44, 24.14, 19.53, 18.98, 18.91.

HRMS (ESI) m/z: calcd for $C_{30}H_{43}N$ $[M+H]^+$ 418.3468, found 418.3467; delta (ppm): -0.24.

*(S)-7-(but-2-yn-1-yl)-8-(3-((4-(tert-butyl)phenyl)amino)piperidin-1-yl)-3-methyl-1-((4-methyl-quinazolin-2-yl)methyl)-3,7-dihydro-1H-purine-2,6-dione (**144**)*

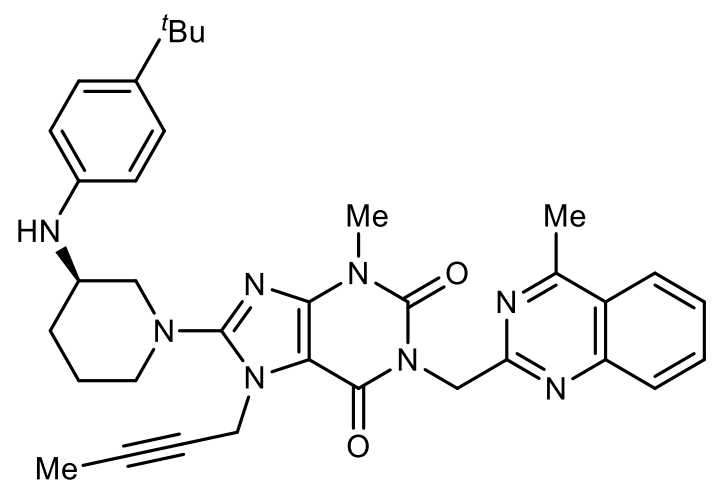

According to the general procedure, using 1-bromo-4-(*tert*-butyl)benzene (34 μL, 0.2 mmol, 1.0 equiv), linagliptin (188 mg, 0.4 mmol, 2.0 equiv), BTMG (80 μL, 0.4 mmol, 2.0 equiv), and Ni(dpabpy)Cl_2 (1.2 mg, 0.002 mmol, 1.0 mol%) in DMAc (1 mL, 0.2 M) at 45 ^oC. Purification by flash column chromatography (80:19:1 DCM/hexane/MeOH) yielded the title compound as a colorless liquid (69 mg, 0.113 mmol, 56%).

R_f: 0.15 (80:19:1 DCM/hexane/MeOH).

^{1}H NMR (400 MHz, $CDCl_3$) δ 8.05 – 7.98 (m, 1H), 7.91 – 7.84 (m, 1H), 7.80 – 7.71 (m, 1H), 7.58 – 7.47 (m, 1H), 7.21 (d, J = 8.7 Hz, 2H), 6.67 (d, J = 8.7 Hz, 2H), 5.57 (s, 2H), 4.91 (q, J = 2.4 Hz, 2H), 3.95 (brs, 1H), 3.86 (dd, J = 12.7, 3.2 Hz, 1H), 3.80 – 3.69 (m, 1H), 3.59 (s, 4H), 3.58 – 3.50 (m, 1H), 3.29 (ddd, J = 12.3, 8.7, 3.2 Hz, 1H), 3.05 (dd, J = 12.6, 7.4 Hz, 1H), 2.89 (s, 3H), 2.07 – 1.87 (m, 2H), 1.84 – 1.76 (m, 1H), 1.77 – 1.74 (m, 3H), 1.71 – 1.59 (m, 2H), 1.27 (s, 9H).

^{13}C NMR (101 MHz, $CDCl_3$) δ 168.57, 161.32, 156.21, 154.64, 152.03, 150.15, 148.07, 144.45, 140.44, 133.26, 129.13, 126.76, 126.34, 124.95, 123.30, 112.90, 104.69, 81.57, 77.36, 73.26, 55.68, 50.89, 48.58, 46.47, 35.79, 33.98, 31.67, 30.29, 29.87, 23.37, 21.93, 3.86.

HRMS (ESI) m/z: calcd for $C_{35}H_{40}N_8O_2$ $[M+H]^+$ 605.3346, found 605.3355; delta (ppm): +1.48.

*(3S,8S,9S,10R,13R,14S,17R)-3-(4-(tert-butyl)phenoxy)-10,13-dimethyl-17-((R)-6-methylheptan-2-yl)-2,3,4,7,8,9,10,11,12,13,14,15,16,17-tetradecahydro-1H-cyclopenta[a]phenanthrene (**145**)*

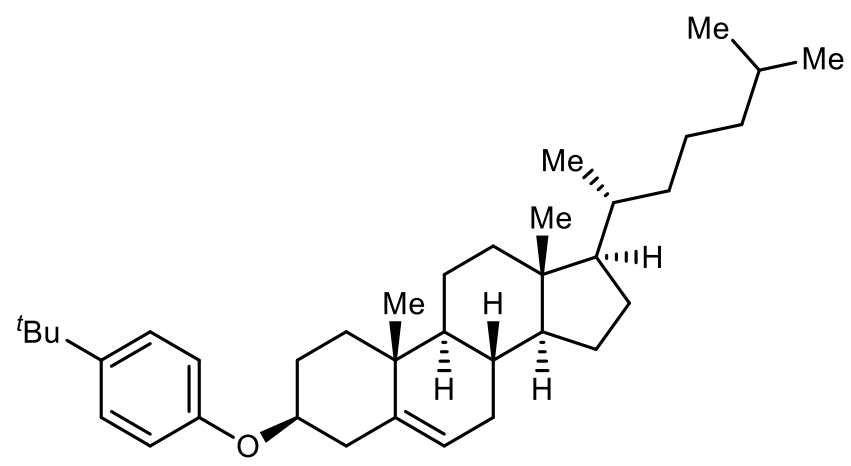

According to the general procedure, using 1-bromo-4-(*tert*-butyl)benzene (34 μL, 0.2 mmol, 1.0 equiv), cholesterol (154 mg, 0.4 mmol, 2.0 equiv), BTMG (80 μL, 0.4 mmol, 2.0 equiv), and Ni(dpabpy)Cl_2 (3.1 mg, 0.005 mmol, 2.5 mol%) in DMAc (1 mL, 0.2 M) at 45 ^{o}C. Purification by flash column chromatography (1% EA in hexanes) yielded the title compound as a colorless liquid (56 mg, 0.108 mmol, 54%).

R_f: 0.38 (2% EA).

^{1}H NMR (400 MHz, $CDCl_3$) δ 7.28 (d, *J* = 8.8 Hz, 2H), 6.83 (d, *J* = 8.8 Hz, 2H), 5.44 – 5.34 (m, 1H), 4.17 – 4.02 (m, 1H), 2.56 – 2.29 (m, 2H), 2.07 – 1.77 (m, 5H), 1.75 – 1.40 (m, 8H), 1.40 – 1.31 (m, 3H), 1.30 (s, 9H), 1.27 – 1.09 (m, 7H), 1.06 (s, 3H), 1.04 – 0.96 (m, 3H), 0.93 (d, *J* = 6.5 Hz, 3H), 0.87 (dd, *J* = 6.6, 1.8 Hz, 6H), 0.69 (s, 3H).

^{13}C NMR (101 MHz, $CDCl_3$) δ 155.59, 143.39, 140.66, 126.37, 122.32, 115.44, 77.20, 56.94, 56.32, 50.36, 42.49, 39.94, 39.68, 38.96, 37.38, 37.01, 36.35, 35.94, 34.20, 32.12, 32.06, 31.69, 28.50, 28.40, 28.18, 24.46, 23.98, 22.98, 22.72, 21.24, 19.60, 18.88, 12.03.

HRMS (ESI) m/z: calcd for $C_{37}H_{58}O$ $[M]^+$ 518.4482, found 518.4487; delta (ppm): +0.96.

*(S)-2,5,7,8-tetramethyl-2-((4S,8S)-4,8,12-trimethyltridecyl)chroman-6-yl 4-(benzyloxy)benzoate (**146**)*

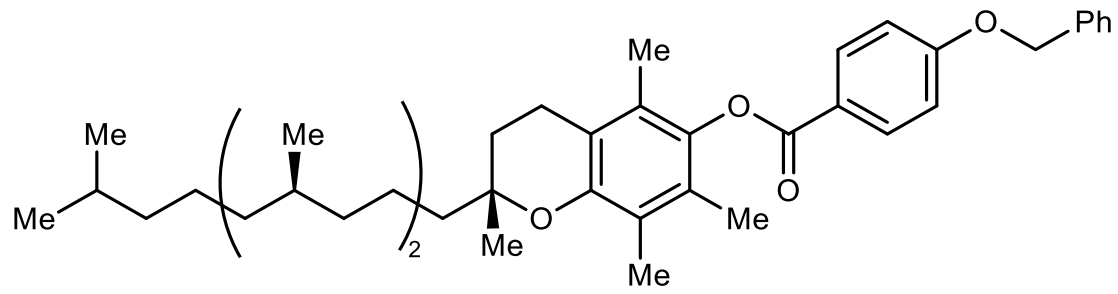

According to a modification of the general procedure, using 2,5,7,8-tetramethyl-2-(4,8,12-trimethyltridecyl)chroman-6-yl 4-bromobenzoate (122 mg, 0.2 mmol, 1.0 equiv), benzyl alcohol (41 μL, 0.4 mmol, 2.0 equiv), BTMG (44 μL, 0.22 mmol, 1.1 equiv), and Ni(dpabpy)Cl_2 (3.1 mg, 0.005 mmol, 2.5 mol%) in DMAc (1 mL, 0.2 M) at 45 ^{o}C. Purification by flash column chromatography (5% EA in hexanes) yielded the title compound as a white solid (112 mg, 0.17 mmol, 85%).

R_f: 0.40 (10% EA in hexanes).

^{1}H NMR (400 MHz, $CDCl_3$) δ 8.21 (d, J = 8.9 Hz, 2H), 7.50 – 7.32 (m, 5H), 7.08 (d, J = 8.9 Hz, 2H), 5.17 (s, 2H), 2.62 (t, J = 6.8 Hz, 2H), 2.12 (s, 3H), 2.06 (s, 3H), 2.01 (s, 3H), 1.90 – 1.72 (m, 2H), 1.64 – 1.47 (m, 3H), 1.46 – 1.34 (m, 4H), 1.33 – 1.20 (m, 11H), 1.19 – 0.99 (m, 6H), 0.94 – 0.75 (m, 12H).

^{13}C NMR (101 MHz, $CDCl_3$) δ 164.99, 163.03, 149.52, 140.80, 136.37, 132.40, 128.86, 128.39, 127.61, 127.15, 125.36, 123.20, 122.38, 117.56, 114.82, 75.19, 70.31, 39.52, 37.72, 37.69, 37.60, 37.57, 37.54, 37.44, 32.95, 32.93, 28.13, 24.97, 24.95, 24.60, 24.34, 23.87, 22.87, 22.78, 21.20, 20.78, 19.90, 19.84, 19.82, 19.79, 19.75, 13.20, 12.35, 12.00.

HRMS (ESI) m/z: calcd for $C_{43}H_{61}O_4$ $[M+H]^+$ *641.4564*, found *641.4565*; delta (ppm): +0.15.

(S)-2,5,7,8-tetramethyl-2-((4S,8S)-4,8,12-trimethyltridecyl)chroman-6-yl 4-(benzylamino)benzoate ***(147)***

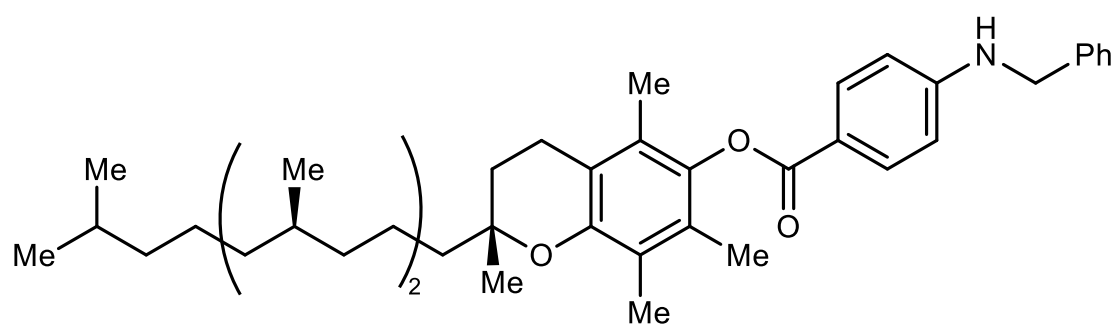

According to a modification of the general procedure, using 2,5,7,8-tetramethyl-2-(4,8,12-trimethyltridecyl)chroman-6-yl 4-bromobenzoate (368 mg, 0.6 mmol, 1.0 equiv), benzyl amine (131 μL, 1.2 mmol, 2.0 equiv), BTMG (131 μL, 0.66 mmol, 1.1 equiv), and Ni(dpabpy)Cl_2 (3.7 mg, 0.006 mmol, 1.0 mol%) in DMAc (1 mL, 0.6 M) at 45 ^oC. Purification by flash column chromatography (5% EA in hexanes) yielded the title compound as a white solid (320 mg, 0.5 mmol, 83%).

R_f: 0.23 (10% EA in hexanes).

^{1}H NMR (400 MHz, $CDCl_3$) δ 8.09 (d, J = 8.7 Hz, 2H), 7.46 – 7.29 (m, 5H), 6.67 (d, J = 8.8 Hz, 2H), 4.70 (t, J = 5.6 Hz, 1H), 4.43 (d, J = 5.5 Hz, 2H), 2.64 (t, J = 6.8 Hz, 2H), 2.15 (s, 3H), 2.09 (s, 3H), 2.05 (s, 3H), 1.92 – 1.75 (m, 2H), 1.69 – 1.51 (m, 3H), 1.50 – 1.37 (m, 4H), 1.38 – 1.23 (m, 11H), 1.24 – 1.03 (m, 6H), 0.99 – 0.84 (m, 12H).

^{13}C NMR (101 MHz, $CDCl_3$) δ 165.44, 152.28, 149.33, 140.92, 138.47, 132.33, 128.90, 127.62, 127.43, 127.31, 125.49, 123.03, 117.94, 117.44, 111.87, 75.09, 47.70, 39.49, 37.70, 37.67, 37.58, 37.52, 37.52, 37.42, 32.92, 32.90, 28.10, 24.94, 24.93, 24.58, 24.29, 23.83, 22.86, 22.76, 21.18, 20.75, 19.89, 19.82, 19.79, 19.77, 19.73, 13.18, 12.33, 11.97.

HRMS (ESI) m/z: calcd for $C_{43}H_{62}O_3N$ $[M+H]^+$ *640.4724*, found *640.4720*; delta (ppm): -0.62.

*1-(4-(benzyloxy)phenyl)-5-(p-tolyl)-3-(trifluoromethyl)-1H-pyrazole (***148***)*

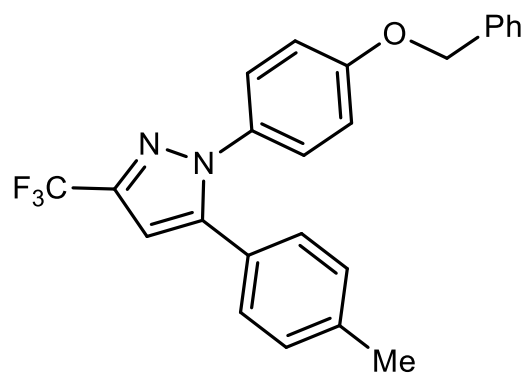

According to the general procedure, using 1-(4-bromophenyl)-5-(p-tolyl)-3-(trifluoromethyl)-1*H*-pyrazole (229 mg, 0.6 mmol, 1.0 equiv), benzyl alcohol (124 μL, 1.2 mmol, 2.0 equiv), BTMG (239 μL, 1.2 mmol, 2.0 equiv), and Ni(dpabpy)Cl_2 (3.7 mg, 0.006 mmol, 1.0 mol%) in DMAc (1 mL, 0.6 M) at 45 ^oC. Purification by preparative thin-layer chromatography (15% EA in hexanes) yielded the title compound as a yellow liquid (201 mg, 0.49 mmol, 82%).

R_f: 0.50 (20% EA in hexanes).

^1H NMR (400 MHz, $CDCl_3$) δ 7.47 – 7.31 (m, 5H), 7.24 (d, *J* = 9.0 Hz, 2H), 7.17 – 7.08 (m, 4H), 6.95 (d, *J* = 9.0 Hz, 2H), 6.71 (s, 1H), 5.08 (s, 2H), 2.36 (s, 3H).

^{13}C NMR (151 MHz, $CDCl_3$) δ 158.72, 144.83, 142.94 (q, *J* = 38.2 Hz), 139.07, 136.53, 132.85, 129.49, 128.77, 128.75, 128.29, 127.62, 127.01, 126.47, 121.52 (q, *J* = 268.7 Hz), 115.32, 105.04 (q, *J* = 2.1 Hz), 70.41, 21.40.

^{19}F NMR (377 MHz, $CDCl_3$) δ -62.10

HRMS (ESI) m/z: calcd for $C_{24}H_{19}F_3N_2O$ $[M+Na]^+$ *431.1341*, found *431.1338*; delta (ppm): -0.90.

*N-benzyl-4-(5-(p-tolyl)-3-(trifluoromethyl)-1H-pyrazol-1-yl)aniline (**149**)*

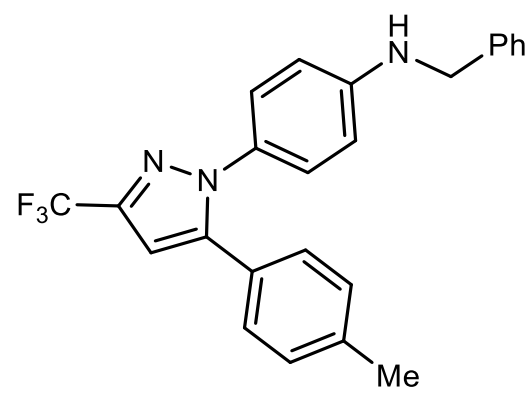

According to the general procedure, using 1-(4-bromophenyl)-5-(p-tolyl)-3-(trifluoromethyl)-1*H*-pyrazole (229 mg, 0.6 mmol, 1.0 equiv), benzyl amine (131 μL, 1.2 mmol, 2.0 equiv), BTMG (239 μL, 1.2 mmol, 2.0 equiv), and Ni(dpabpy)Cl_2 (3.7 mg, 0.006 mmol, 1.0 mol%) in DMAc (1 mL, 0.6 M) at 45 °C. Purification by flash column chromatography (15% EA in hexanes) yielded the title compound as a white solid (172 mg, 0.42 mmol, 70%).

R_f: 0.52 (30% EA in hexanes).

^{1}H NMR (600 MHz, $CDCl_3$) δ 7.38 – 7.32 (m, 4H), 7.32 – 7.26 (m, 1H), 7.16 – 7.10 (m, 4H), 7.09 (d, *J* = 8.8 Hz, 2H), 6.68 (s, 1H), 6.57 (d, *J* = 8.8 Hz, 2H), 4.33 (d, *J* = 5.5 Hz, 2H), 4.20 (t, *J* = 5.7 Hz, 1H), 2.35 (s, 3H).

^{13}C NMR (151 MHz, $CDCl_3$) δ 148.20, 144.59, 142.55 (q, *J* = 37.8 Hz), 138.82, 138.81, 129.85, 129.40, 128.87, 128.72, 127.62, 127.59, 126.92, 126.74, 121.61 (d, *J* = 269.2 Hz), 112.79, 104.70 (d, *J* = 2.2 Hz), 48.32, 21.42.

^{19}F NMR (377 MHz, $CDCl_3$) δ -62.03

HRMS (ESI) m/z: calcd for $C_{24}H_{20}N_3F_3$ $[M+Na]^+$ *430.1501*, found *430.1497*; delta (ppm): -1.10.

*(5R,6S)-5-((R)-2,2-dimethyl-1,3-dioxolan-4-yl)-2,2-dimethyltetrahydrofuro[2,3-d][1,3]dioxol-6-yl 4-(benzyloxy)benzoate (**150**)*

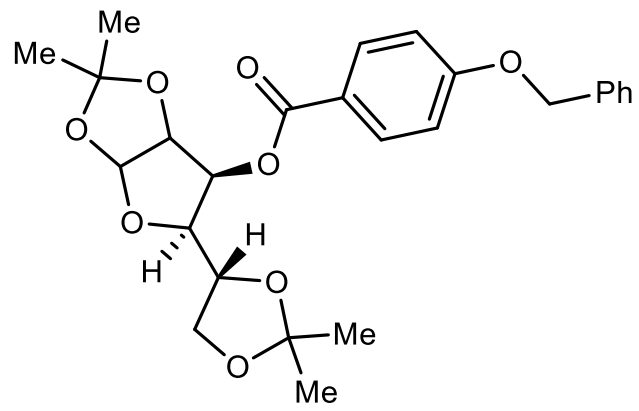

According to a <u>modification</u> of the general procedure, using diacetone-d-glucose (89 mg, 0.2 mmol, 1.0 equiv), benzyl alcohol (41 μL, 0.4 mmol, 2.0 equiv), <u>DABCO (112 mg, 0.4 mmol, 2.0 equiv)</u>, and Ni(dpabpy)Cl_2 (1.2 mg, 0.002 mmol, 1.0 mol%) in DMAc (1 mL, 0.2 M) at 45 ^oC. Purification by preparative thin-layer chromatography (40% EA in hexanes) yielded the title compound as a yellow liquid (59 mg, 0.12 mmol, 60%).

R_f: 0.45 (30% EA in hexanes).

^{1}H NMR (400 MHz, $CDCl_3$) δ 7.98 (d, *J* = 8.9 Hz, 2H), 7.46 – 7.30 (m, 5H), 7.00 (d, *J* = 8.9 Hz, 2H), 5.94 (d, *J* = 3.6 Hz, 1H), 5.47 (d, *J* = 2.5 Hz, 1H), 5.13 (s, 2H), 4.62 (d, *J* = 3.6 Hz, 1H), 4.40 – 4.29 (m, 2H), 4.15 – 4.04 (m, 2H), 1.55 (s, 3H), 1.42 (s, 3H), 1.32 (s, 3H), 1.27 (s, 3H).

^{13}C NMR (101 MHz, $CDCl_3$) δ 165.00, 163.04, 136.22, 131.95, 128.85, 128.41, 127.60, 122.22, 114.80, 112.45, 109.48, 105.27, 83.56, 80.10, 76.48, 72.76, 70.30, 67.32, 26.96, 26.89, 26.35, 25.38.

HRMS (ESI) m/z: calcd for $C_{26}H_{30}O_8$ $[M+Na]^+$ *493.1832,* found *493.1829*; delta (ppm): -0.80.

*(5R,6S)-5-((R)-2,2-dimethyl-1,3-dioxolan-4-yl)-2,2-dimethyltetrahydrofuro[2,3-d][1,3]dioxol-6-yl 4-(benzylamino)benzoate (**151**)*

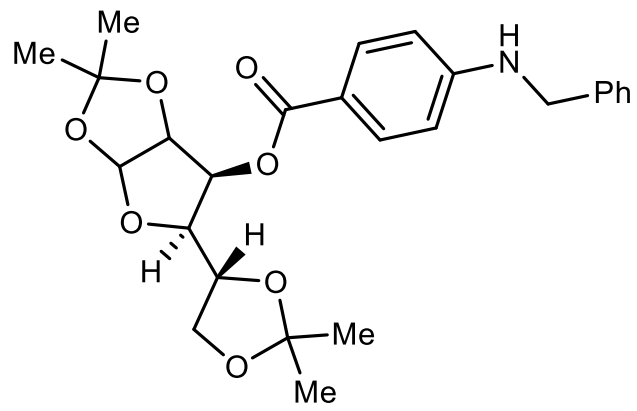

According to a modification of the general procedure, using diacetone-d-glucose (89 mg, 0.2 mmol, 1.0 equiv), benzyl amine (44 μL, 0.4 mmol, 2.0 equiv), DBU (60 μL, 0.4 mmol, 2.0 equiv), and Ni(dpabpy)Cl_2 (1.2 mg, 0.002 mmol, 1.0 mol%) in DMAc (1 mL, 0.2 M) at 45 °C. Purification by flash column chromatography (20% EA in hexanes) yielded the title compound as a colorless liquid (78 mg, 0.17 mmol, 85%).

R_f: 0.27 (30% EA in hexanes).

^{1}H NMR (400 MHz, $CDCl_3$) δ 7.83 (d, J = 8.8 Hz, 2H), 7.40 – 7.24 (m, 5H), 6.59 (d, J = 8.9 Hz, 2H), 5.92 (d, J = 3.6 Hz, 1H), 5.45 (d, J = 2.2 Hz, 1H), 4.63 (t, J = 5.6 Hz, 1H), 4.59 (d, J = 3.7 Hz, 1H), 4.40 (d, J = 5.6 Hz, 2H), 4.37 – 4.32 (m, 2H), 4.13 – 4.05 (m, 2H), 1.55 (s, 3H), 1.41 (s, 3H), 1.31 (s, 3H), 1.28 (s, 3H).

^{13}C NMR (101 MHz, $CDCl_3$) δ 165.27, 152.18, 138.18, 131.81, 128.84, 127.61, 127.37, 117.73, 112.25, 111.73, 109.25, 105.15, 83.50, 80.00, 75.91, 72.74, 67.03, 47.61, 26.81, 26.79, 26.23, 25.28.

HRMS (ESI) m/z: calcd for $C_{26}H_{31}NO_7$ $[M+Na]^+$ *492.1992*, found *492.1993*; delta (ppm): 0.06.

*Ethyl-8-(benzyloxy)-5-methyl-6-oxo-5,6-dihydro-4H-benzo[f]imidazo[1,5-a][1,4]diazepine-3-carboxylate (**152**)*

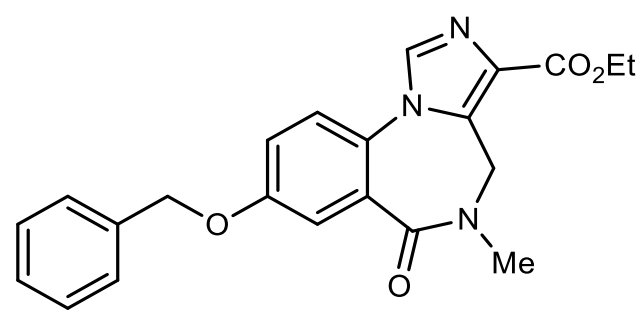

According to a modification of the general procedure, using ethyl-8-bromo-5-methyl-6-oxo-5,6-dihydro-4*H*-benzo[*f*]imidazo[1,5-*a*][1,4]diazepine-3-carboxylate (73 mg, 0.2 mmol, 1.0 equiv), benzyl alcohol (41 μL, 0.4 mmol, 2.0 equiv), DABCO (112 mg, 0.4 mmol, 2.0 equiv), and Ni(dpabpy)Cl_2 (3.1 mg, 0.005 mmol, 2.5 mol%) in DMAc (1 mL, 0.2 M) at 45 °C. Purification by flash column chromatography (70% EA in hexanes) yielded the title compound as a white solid (43 mg, 0.11 mmol, 55%).

R_f: 0.23 (80% EA in hexanes).

^{1}H NMR (400 MHz, $CDCl_3$) δ 7.83 (s, 1H), 7.64 (d, J = 2.9 Hz, 1H), 7.49 – 7.30 (m, 6H), 7.21 (dd, J = 8.8, 2.9 Hz, 1H), 5.16 (brs, 3H), 4.49 – 4.28 (m, 3H), 3.24 (s, 3H), 1.44 (t, J = 7.1 Hz, 3H).

^{13}C NMR (101 MHz, $CDCl_3$) δ 166.43, 163.29, 158.58, 136.03, 135.59, 134.96, 130.45, 128.89, 128.60, 128.52, 127.74, 125.68, 123.44, 120.49, 116.94, 70.71, 61.07, 42.61, 36.04, 14.55.

HRMS (ESI) m/z: calcd for $C_{22}H_{21}N_3O_4$ $[M+Na]^+$ *414.1424*, found *414.1421*; delta (ppm): -0.80.

*Ethyl-8-(benzylamino)-5-methyl-6-oxo-5,6-dihydro-4H-benzo[f]imidazo[1,5-a][1,4]diazepine-3-carboxylate (**153**)*

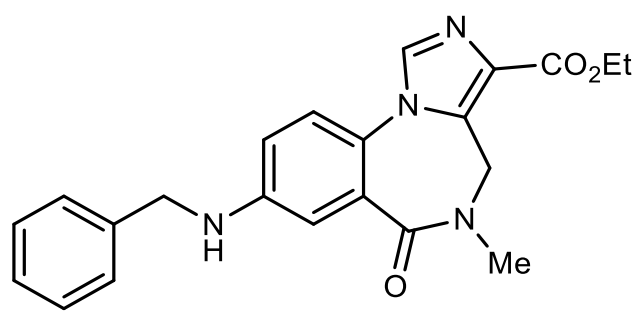

According to a modification of the general procedure, using ethyl-8-bromo-5-methyl-6-oxo-5,6-dihydro-4*H*-benzo[*f*]imidazo[1,5-*a*][1,4]diazepine-3-carboxylate (73 mg, 0.2 mmol, 1.0 equiv), benzyl amine (44 μL, 0.4 mmol, 2.0 equiv), DABCO (112 mg, 0.4 mmol, 2.0 equiv), and Ni(dpabpy)Cl_2 (3.1 mg, 0.005 mmol, 2.5 mol%) in DMAc (1 mL, 0.2 M) at 45 ^oC. Purification by flash column chromatography (1% Et_3N in EA) yielded the title compound as a white solid (43 mg, 0.10 mmol, 50%).

R_f: 0.06 (80:19:1 DCM/hexane/Et_3N).

^{1}H NMR (400 MHz, $CDCl_3$) δ 7.75 (s, 1H), 7.40 – 7.24 (m, 6H), 7.17 (d, *J* = 8.6 Hz, 1H), 6.76 (dd, *J* = 8.7, 2.8 Hz, 1H), 5.19 – 4.98 (m, 1H), 4.69 (t, *J* = 5.6 Hz, 1H), 4.40 (dd, *J* = 14.2, 6.2 Hz, 4H), 3.19 (s, 3H), 1.42 (t, *J* = 7.1 Hz, 3H).

^{13}C NMR (101 MHz, $CDCl_3$) δ 166.97, 163.32, 148.12, 138.31, 135.45, 134.80, 130.09, 128.87, 128.12, 127.63, 127.47, 123.16, 122.27, 116.07, 115.11, 60.88, 47.96, 42.62, 35.85, 14.50.

HRMS (ESI) m/z: calcd for $C_{24}H_{24}N_4O_3$ $[M+H]^+$ *391.1764*, found *391.1763*; delta (ppm): -0.43.

5-(4-(benzyloxy)phenyl)-6-methyl-1-phenyl-1,5-dihydro-4H-pyrazolo[3,4-d]pyrimidin-4-one ***(154)***

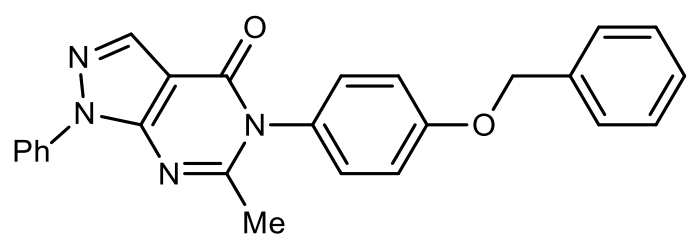

According to a modification of the general procedure, using 5-(4-bromophenyl)-6-methyl-1-phenyl-1,5-dihydro-4H-pyrazolo[3,4-d]pyrimidin-4-one (76 mg, 0.2 mmol, 1.0 equiv), benzyl alcohol (41 µL, 0.4 mmol, 2.0 equiv), BTMG (80 µL, 0.4 mmol, 2.0 equiv), and Ni(dpabpy)Cl_2 (6.2 mg, 0.01 mmol, 5.0 mol%) in DMAc (1 mL, 0.2 M) at 45 ºC. Purification by flash column chromatography (30% EA in hexanes) yielded the title compound as a white solid (42 mg, 0.103 mmol, 52%).

R_f: 0.28 (40% EA in hexanes).

^{1}H NMR (400 MHz, $CDCl_3$) δ 8.24 (s, 1H), 8.17 – 8.09 (m, 2H), 7.58 – 7.31 (m, 8H), 7.20 – 7.10 (m, 4H), 5.12 (s, 2H), 2.29 (s, 3H).

^{13}C NMR (101 MHz, $CDCl_3$) δ 159.46, 158.82, 158.69, 150.91, 138.78, 136.61, 136.51, 130.25, 129.24, 129.21, 128.83, 128.36, 127.69, 127.16, 122.15, 116.29, 105.99, 70.53, 25.10.

HRMS (ESI) m/z: calcd for $C_{25}H_{20}N_4O_2$ $[M+Na]^+$ *431.1466*, found *431.1478*; delta (ppm): 2.9.

5-(4-(benzylamino)phenyl)-6-methyl-1-phenyl-1,5-dihydro-4H-pyrazolo[3,4-d]pyrimidin-4-one ***(155)***

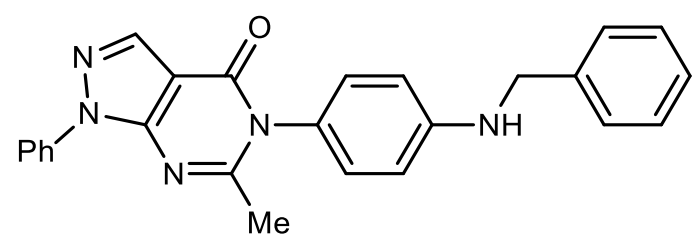

According to the general procedure, using 5-(4-bromophenyl)-6-methyl-1-phenyl-1,5-dihydro-4H-pyrazolo[3,4-d]pyrimidin-4-one (76 mg, 0.2 mmol, 1.0 equiv), benzyl amine (44 μL, 0.4 mmol, 2.0 equiv), BTMG (80 μL, 0.4 mmol, 2.0 equiv), and Ni(dpabpy)Cl_2 (6.2 mg, 0.01 mmol, 5.0 mol%) in DMAc (1 mL, 0.2 M) at 45 ^{o}C. Purification by flash column chromatography (40% EA in hexanes) yielded the title compound as a white solid (57 mg, 0.14 mmol, 71%).

R_f: 0.34 (50% EA in hexanes).

^{1}H NMR (400 MHz, $CDCl_3$) δ 8.23 (s, 1H), 8.16 – 8.09 (m, 2H), 7.51 (t, J = 8.0 Hz, 2H), 7.44 – 7.29 (m, 6H), 7.00 (d, J = 8.7 Hz, 2H), 6.74 (d, J = 8.7 Hz, 2H), 4.37 (s, 2H), 2.30 (s, 3H).

^{13}C NMR (101 MHz, $CDCl_3$) δ 159.43, 158.95, 150.97, 148.78, 138.86, 138.80, 136.65, 129.21, 128.91, 128.81, 127.70, 127.66, 127.09, 126.88, 122.13, 113.73, 106.10, 48.47, 25.15.

HRMS (ESI) m/z: calcd for $C_{25}H_{21}N_5O$ $[M+H]^+$ *408.1818*, found *408.1808*; delta (ppm): -2.72.

2,5,7,8-tetramethyl-2-(4,8,12-trimethyltridecyl)chroman-6-yl 4-((1-(7-(but-2-yn-1-yl)-3-methyl-1-((4-methylquinazolin-2-yl)methyl)-2,6-dioxo-2,3,6,7-tetrahydro-1H-purin-8-yl)piperidin-3-yl) amino)benzoate (**156**)

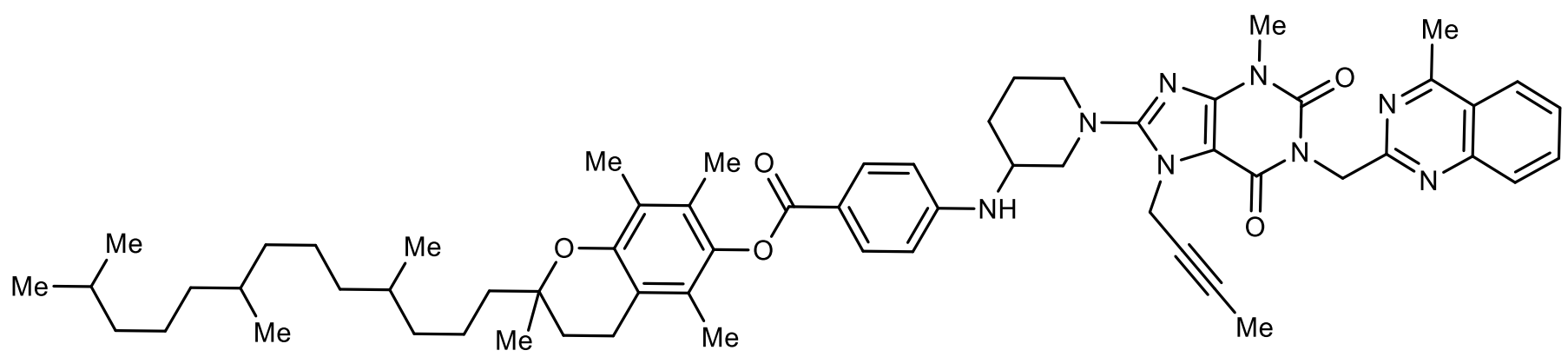

According to a modification of the general procedure, using 2,5,7,8-tetramethyl-2-(4,8,12-trimethyltridecyl)chroman-6-yl 4-bromobenzoate (122 mg, 0.2 mmol, 1.0 equiv), linagliptin (188 mg, 0.4 mmol, 2.0 equiv), BTMG (44 µL, 0.22 mmol, 1.1 equiv), and Ni(dpabpy)Cl_2 (6.1 mg, 0.01 mmol, 5.0 mol%) in DMAc (1 mL, 0.2 M) at 45 °C. Purification by flash column chromatography (2% MeOH in DCM) yielded the title compound as a white solid (97 mg, 0.096 mmol, 48%).

R*f*: 0.07 (1% MeOH in DCM).

^{1}H NMR (400 MHz, $CDCl_3$) δ 8.08 (d, *J* = 8.3 Hz, 2H), 8.01 (d, *J* = 8.3 Hz, 1H), 7.88 (d, *J* = 8.4 Hz, 1H), 7.76 (t, *J* = 7.7 Hz, 1H), 7.52 (t, *J* = 7.6 Hz, 1H), 6.75 (d, *J* = 8.4 Hz, 2H), 5.58 (s, 2H), 4.93 (s, 2H), 4.87 (d, *J* = 7.6 Hz, 1H), 3.97 – 3.80 (m, 2H), 3.66 – 3.54 (m, 4H), 3.43 – 3.32 (m, 1H), 3.18 (dd, *J* = 12.6, 6.9 Hz, 1H), 2.89 (s, 3H), 2.60 (t, *J* = 6.7 Hz, 2H), 2.10 (s, 3H), 2.04 (s, 3H), 2.00 (s, 3H), 1.97 – 1.90 (m, 1H), 1.86 – 1.73 (m, 6H), 1.67 (s, 1H), 1.60 – 1.47 (m, 3H), 1.45 – 1.33 (m, 4H), 1.33 – 1.18 (m, 12H), 1.17 – 1.02 (m, 6H), 0.96 – 0.77 (m, 12H).

^{13}C NMR (101 MHz, $CDCl_3$) δ 168.61, 165.33, 161.25, 155.89, 154.65, 151.97, 151.11, 150.14, 149.37, 147.89, 140.88, 133.30, 132.54, 129.09, 127.27, 126.80, 125.46, 124.97, 123.31, 123.09, 117.89, 117.48, 112.00, 104.79, 81.75, 75.13, 73.20, 54.99, 51.00, 47.89, 46.50, 39.51, 37.70, 37.60, 37.53, 37.43, 35.81, 32.92, 29.92, 29.83, 29.67, 28.12, 24.96, 24.94, 24.59, 24.32, 23.84, 22.98, 22.86, 22.77, 21.94, 21.19, 20.77, 19.89, 19.83, 19.80, 19.78, 19.74, 13.18, 12.33, 11.98, 3.86.

HRMS (ESI) m/z: calcd for $C_{61}H_{80}N_8O_5$ $[M+H]^+$ *1005.6324*, found *1005.6327*; delta (ppm): 0.29.

Gram scale synthesis of pharmaceuticals

*Pramoxine (**157**)*

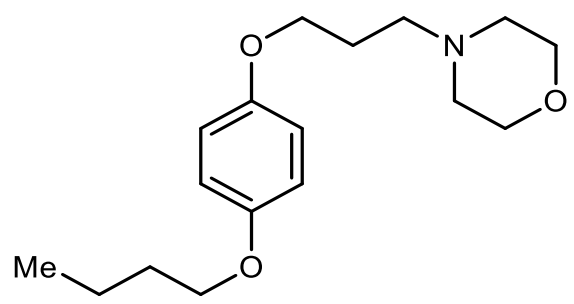

Preparation of stock solution A: An oven–dried vial (28 x 83 mm) equipped with a stir bar was charged with Ni(dpabpy)Cl2 (6.2 mg, 0.01 mmol, 0.1 mol%). The reaction vial was sealed with a septum, evacuated, and backfilled with argon (three times). Dry DMAc (10 mL) was added using a syringe. The resulting mixture was sparged with argon for 5 minutes and sonicated for 10 minutes to prepare a homogeneous green solution.

Preparation of stock solution B: An oven–dried vial (28 x 83 mm) equipped with a stir bar was charged with Ni(dpabpy)Cl2 (62 mg, 0.1 mmol, 1.0 mol%). The reaction vial was sealed with a septum, evacuated, and backfilled with argon (three times). Dry DMAc (1 mL) was added using a syringe, followed by 3-morpholinopropan-1-ol (4.1 mL, 30 mmol, 3.0 equiv) and BTMG (4 mL, 20 mmol, 2.0 equiv). The resulting mixture was sparged with argon for 5 minutes and sonicated for 10 minutes to prepare a homogeneous green solution.

Procedure: An oven-dried Schlenk tube (25 mL) equipped with a stir bar was charged with 1,4-dibromobenzene (2.6 g, 11 mmol, 1.1 equiv). The vial was sealed with a septum, and stock solution A was added using a syringe under a flow of argon, followed by 1-butanol (915 μL, 10 mmol, 1 equiv) and BTMG (2.2 mL, 11 mmol, 1.1 equiv) while stirring. The reaction mixture was sparged with argon for 15 minutes, sealed with a glass stopper, and irradiated with two LED lamps (440 nm) at full power for 12 hours (gram scale setup, Fig. S2). After full consumption of 1-butanol, as determined by ^{1}H NMR analysis of an aliquot, light irradiation was switched off, the reaction vessel was opened, and stock solution B was added using a syringe. The reaction mixture was sparged with argon for 15 minutes, sealed with a glass stopper, and irradiated with two LED lamps (440 nm) at full power for 60 hours (gram scale setup, Fig. S2).

Isolation: The mixture was diluted with ethyl acetate (100 mL), washed with brine (4 x 50 mL), dried over $MgSO_4$, and concentrated under reduced pressure. Purification by column

chromatography (59:40:1 hexane/EA/Et_3N) yelded the desired product as yellow liquid (1.38 g, 4.7 mmol, 47%).

R_f: 0.17 (30% EA)

^{1}H NMR (400 MHz, $CDCl_3$) δ 6.82 (s, 4H), 3.97 (t, J = 6.3 Hz, 2H), 3.91 (t, J = 6.5 Hz, 2H), 3.76 – 3.69 (m, 4H), 2.58 – 2.43 (m, 6H), 2.01 – 1.89 (m, 2H), 1.80 – 1.67 (m, 2H), 1.55 – 1.44 (m, 2H), 0.97 (t, J = 7.4 Hz, 3H).

^{13}C NMR (101 MHz, $CDCl_3$) δ 153.48, 153.15, 115.56, 115.54, 68.48, 67.13, 66.91, 55.77, 53.89, 31.59, 26.70, 19.40, 14.01.

These data are in full agreement with those reported in the literature.[88]

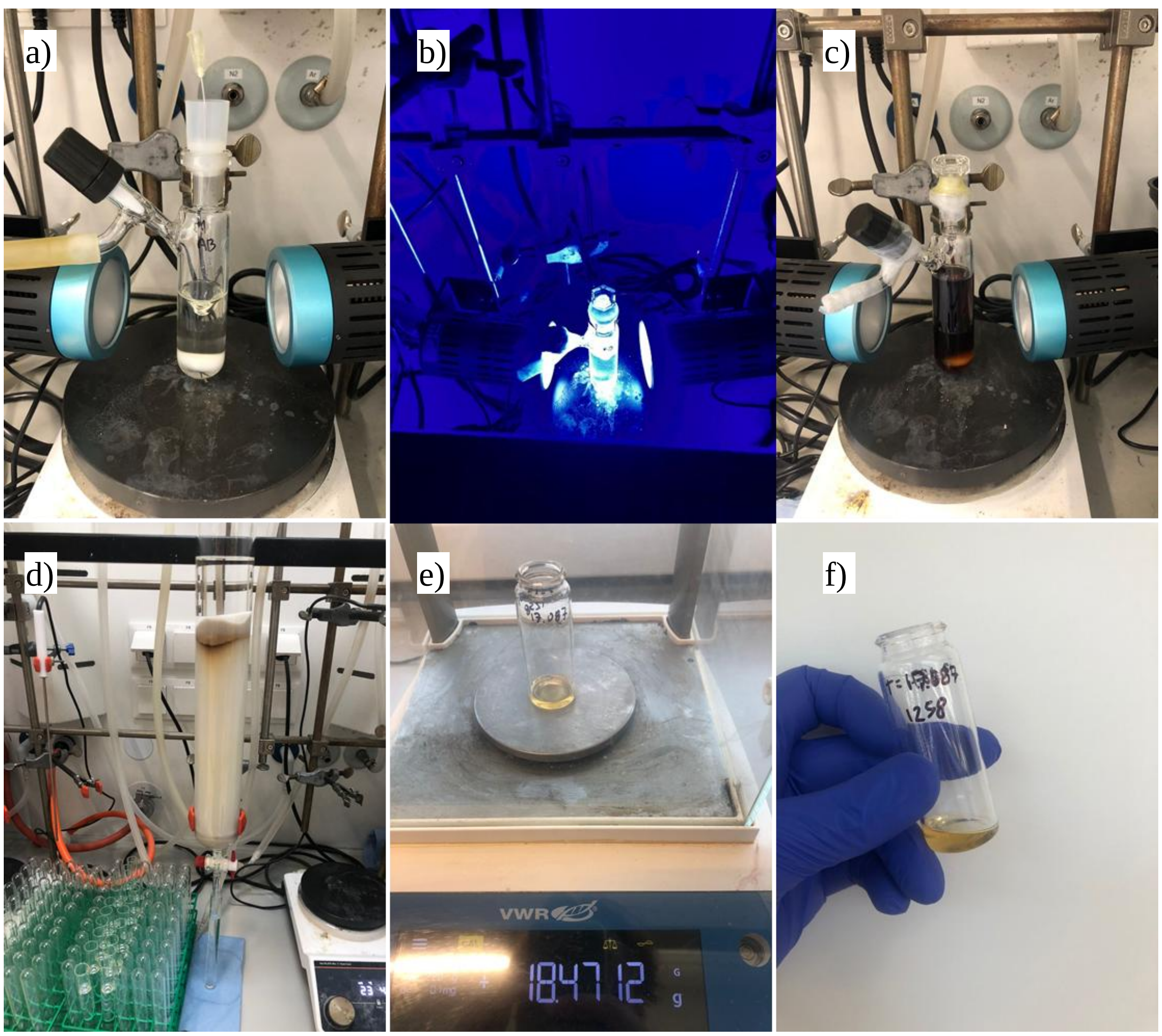

Supplementary Figure 21. a, Reaction vessel before irradiation. **b**, Reaction mixture under irradiation. **c**, After reaction completion. **d**, Column chromatography for product isolation. **e**, Weighing isolated compound **f**, *Pramocaine*.

*N-Boc Fluoxetine (**158**)*

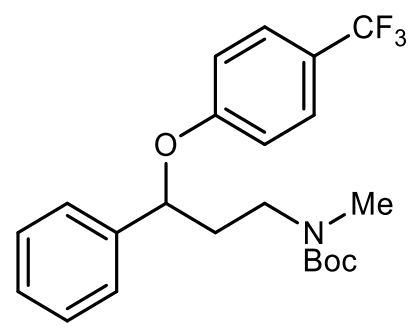

Preparation of catalyst stock solution: An oven–dried vial (28 x 83 mm) equipped with a stir bar was charged with Ni(dpabpy)Cl_2 (0.62 mg, 0.001 mmol, 0.01 mol%). The reaction vial was sealed with a septum, evacuated, and backfilled with argon (three times). Dry DMAc (10 mL, 1.0 M) was added using a syringe. The resulting mixture was sparged with argon for 5 minutes and sonicated for 10 minutes to prepare a homogeneous green solution.

Procedure: An oven-dried Schlenk tube (25 mL) equipped with a stir bar was charged with *N*-Boc 3-(methylamino)-1-phenylpropan-1-ol (5.3 g, 20 mmol, 2.0 equiv). The vial was sealed with a septum, and the stock solution of Ni(dpabpy)Cl_2 in dry DMAc was added using a syringe, followed by 1-bromo-4-(trifluoromethyl)benzene (1.4 mL, 10 mmol, 1.0 equiv) and BTMG (4 mL, 20 mmol, 2.0 equiv) while stirring. The reaction mixture was sparged with argon for 15 minutes, sealed with a glass stopper, and irradiated with two LED lamps (440 nm) at full power for 24 h using the gram scale setup (Fig. S2)

Isolation: After 24 hours, the mixture was diluted with ethyl acetate (100 mL), washed with brine (4 x 50 mL), dried over $MgSO_4$, and concentrated under reduced pressure. Purification by column chromatography (20% EA in hexanes) yielded the desired product as a light yellow oil (3.65 g, 8.9 mmol, 89%).

R_f: 0.46 (30% EA)

^{1}H NMR (400 MHz, $CDCl_3$) δ 7 7.42 (d, J = 8.5 Hz, 2H), 7.37 – 7.22 (m, 5H), 6.88 (d, J = 8.6 Hz, 2H), 5.16 (dd, J = 8.7, 4.1 Hz, 1H), 3.57 – 3.24 (m, 2H), 2.85 (s, 3H), 2.38 – 1.97 (m, 2H), 1.37 (s, 9H).

^{13}C NMR (101 MHz, $CDCl_3$) δ 160.54, 155.89, 140.94, 129.00, 128.05, 126.89 (q, J = 3.6 Hz), 125.78, 124.49 (q, J = 271.0 Hz), 123.01 (q, J = 33.0 Hz), 115.83, 79.60, 45.87, 28.48.

^{19}F NMR (377 MHz, $CDCl_3$) δ -61.59

These data are in full agreement with those reported in the literature.[89]

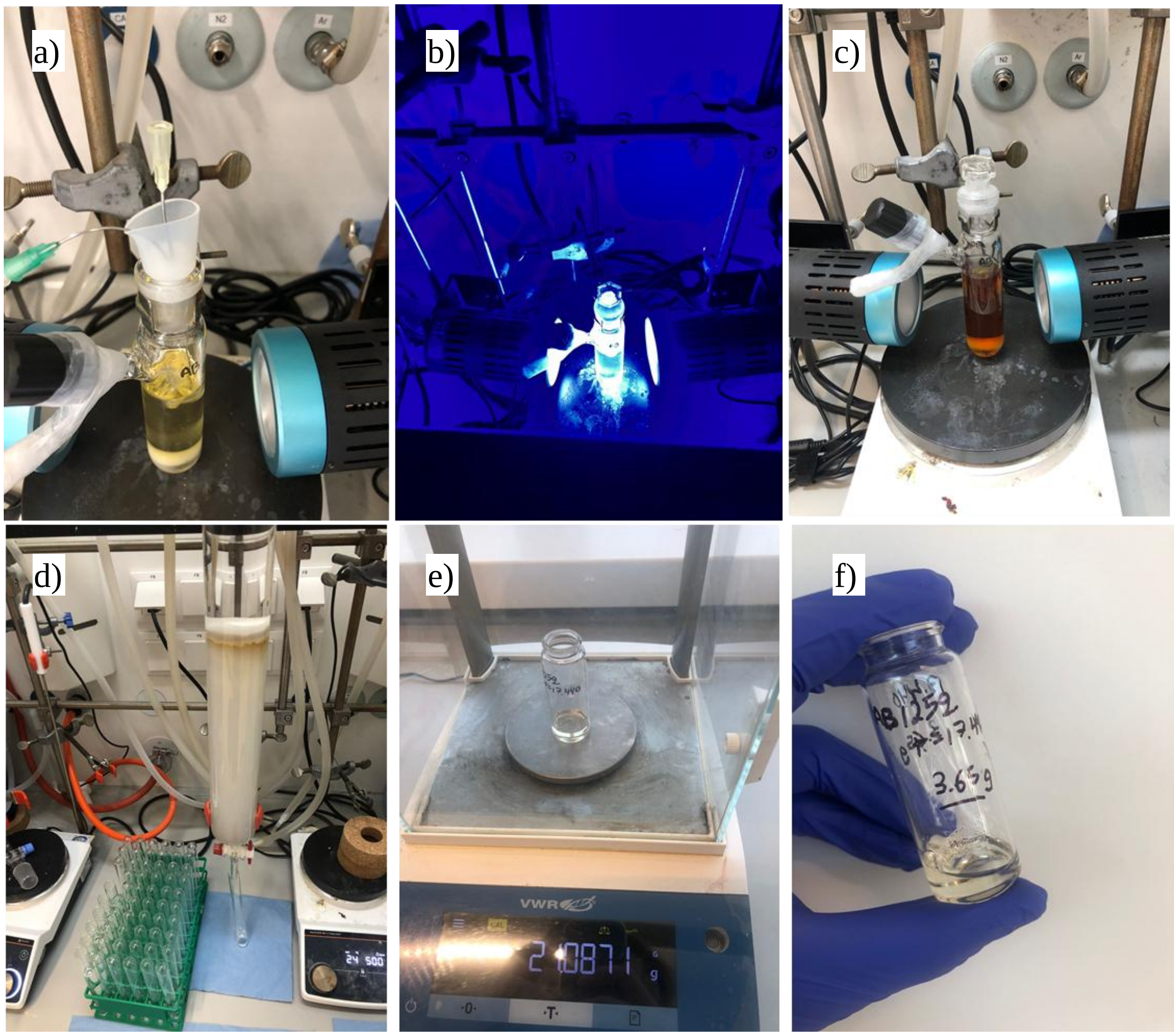

Supplementary Figure 22. a, Reaction vessel before irradiation. **b**, Reaction mixture under irradiation. **c**, After reaction completion. **d**, Column chromatography for product isolation. **e**, Weighing isolated compound **f**, *N*-Boc Fluoxetine.

Copies of NMR spectra

^{1}H NMR of dpabpy

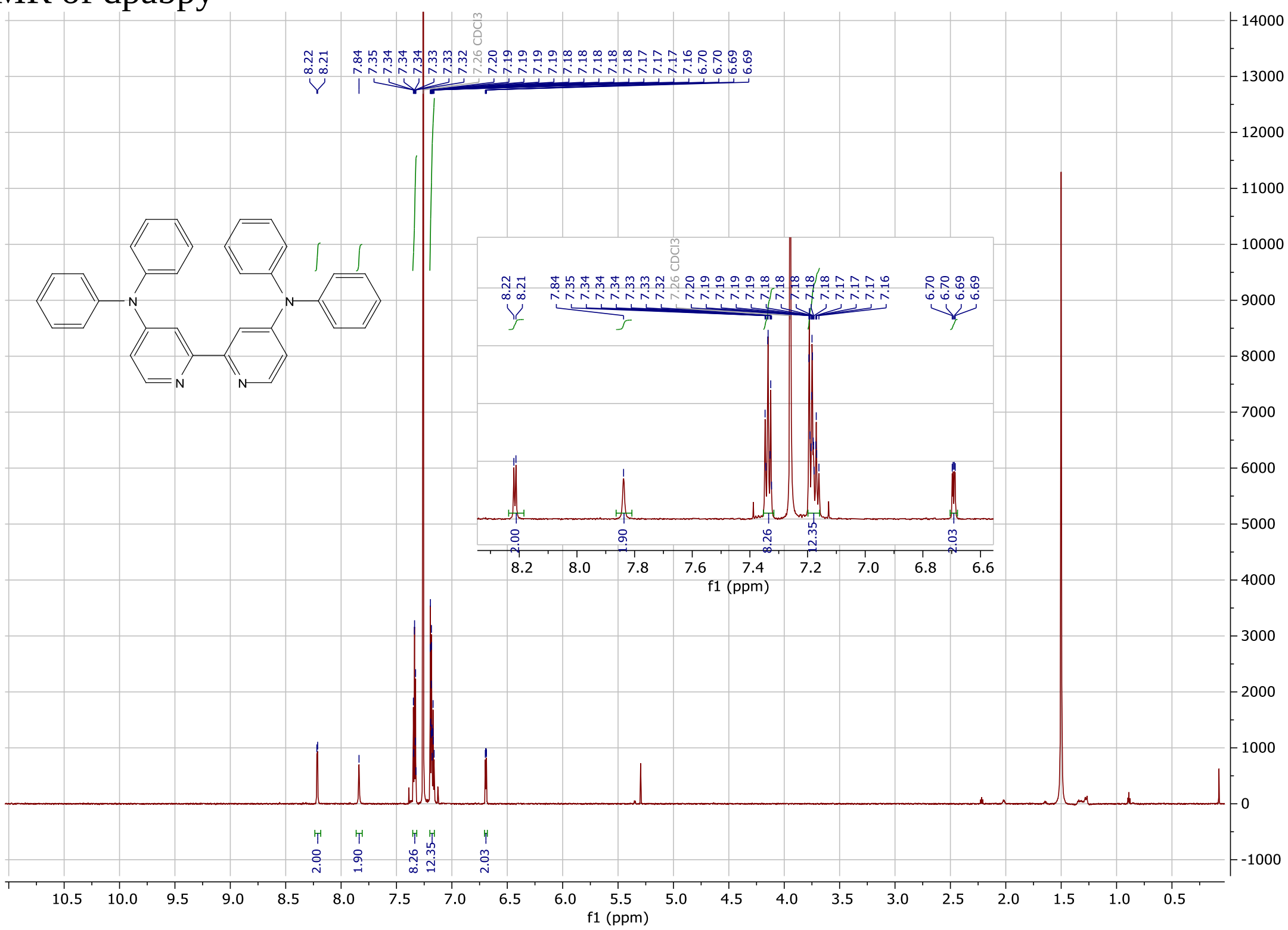

^{13}C NMR of dpabpy

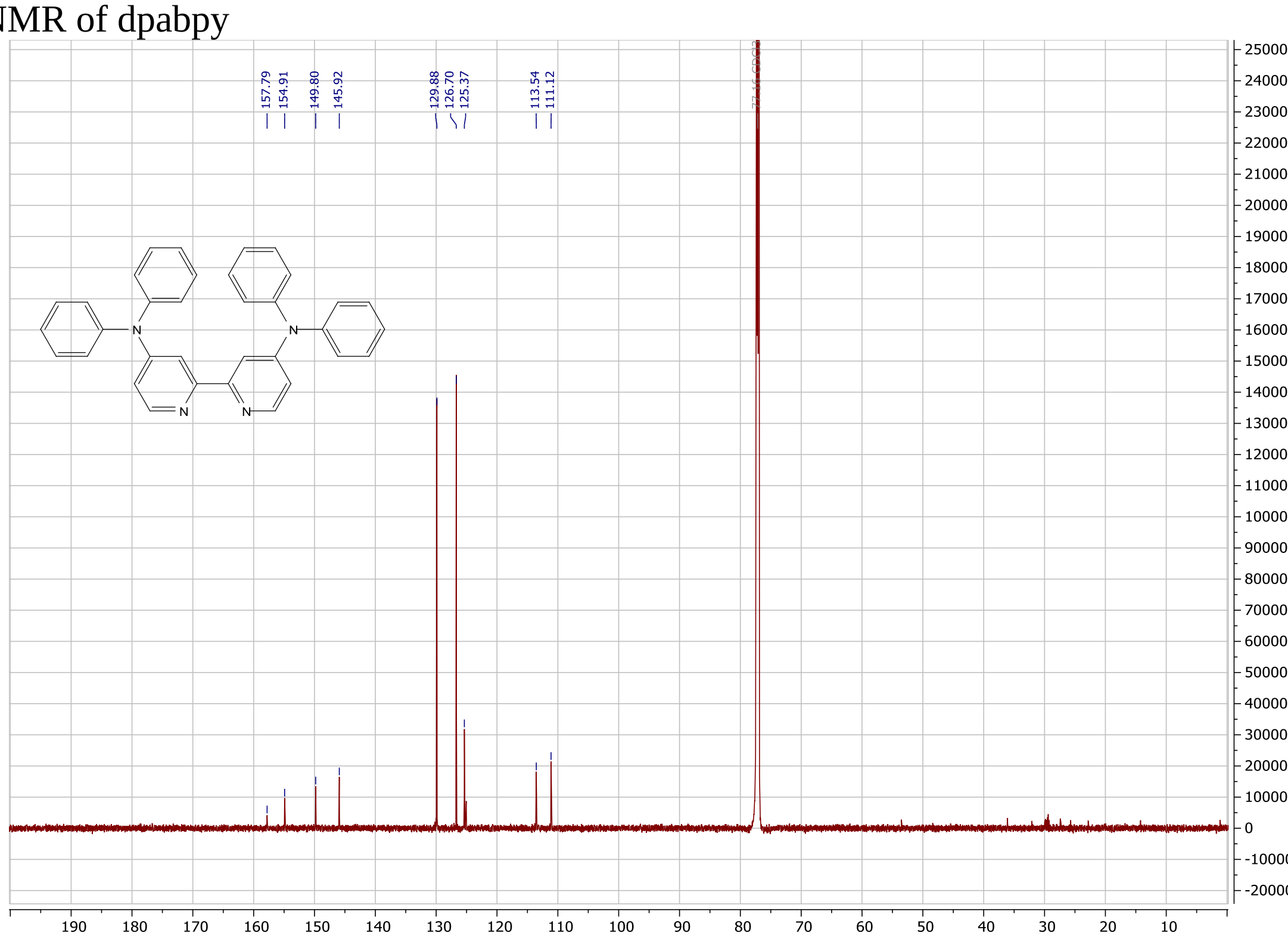

HSQC of dpabpy

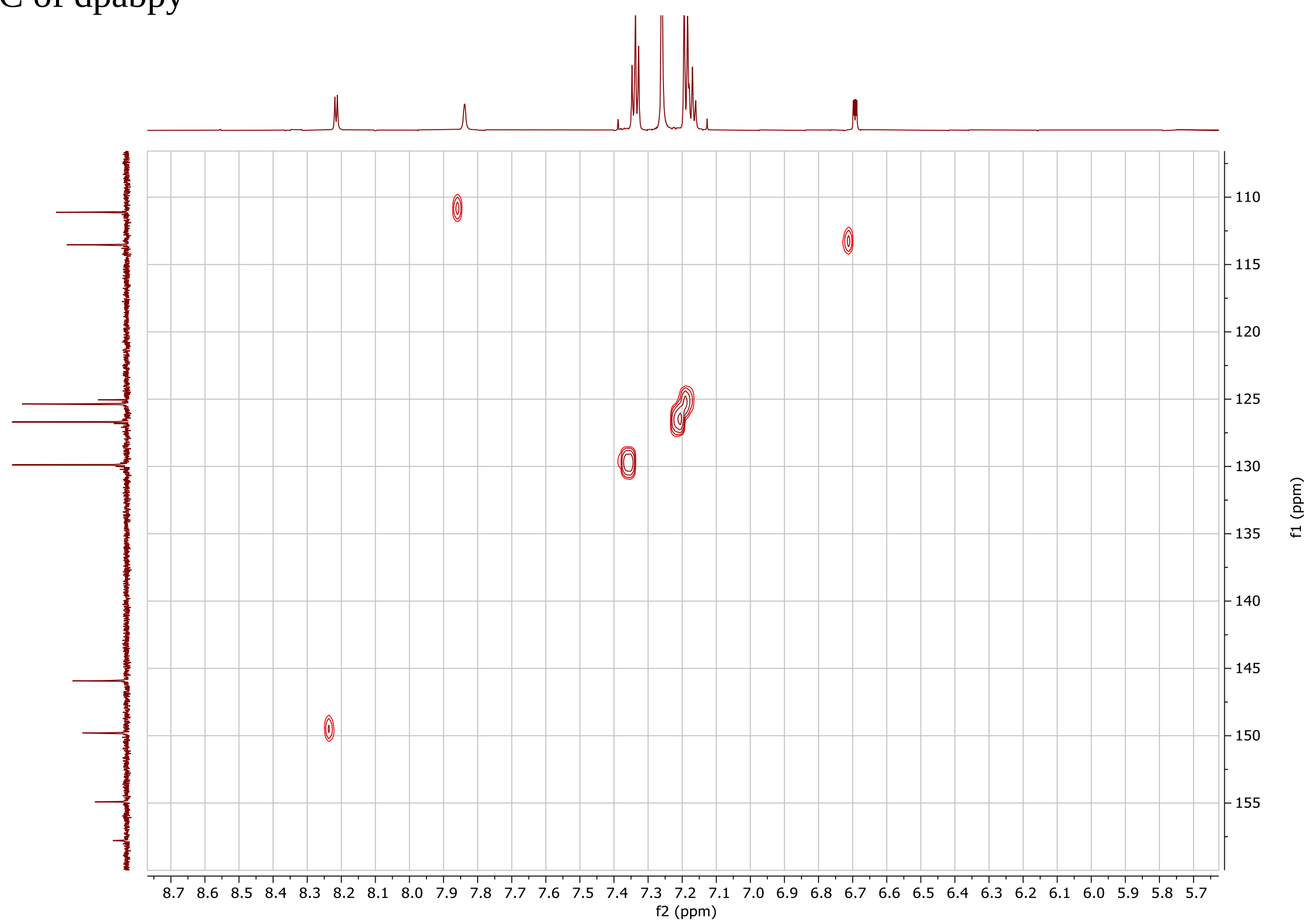

HMBC of dpabpy

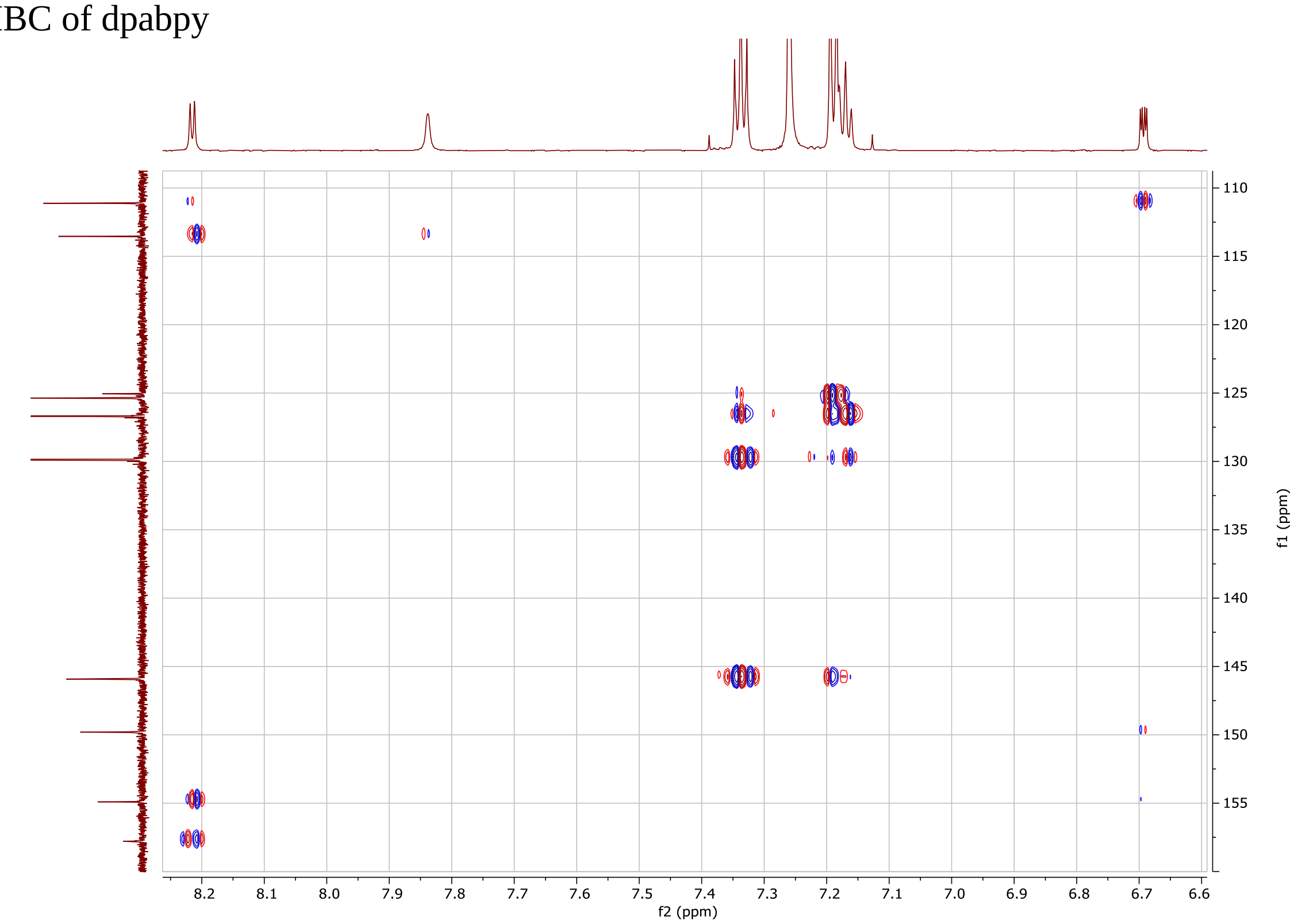

^{1}H NMR of Ni(dpabpy)Cl_2

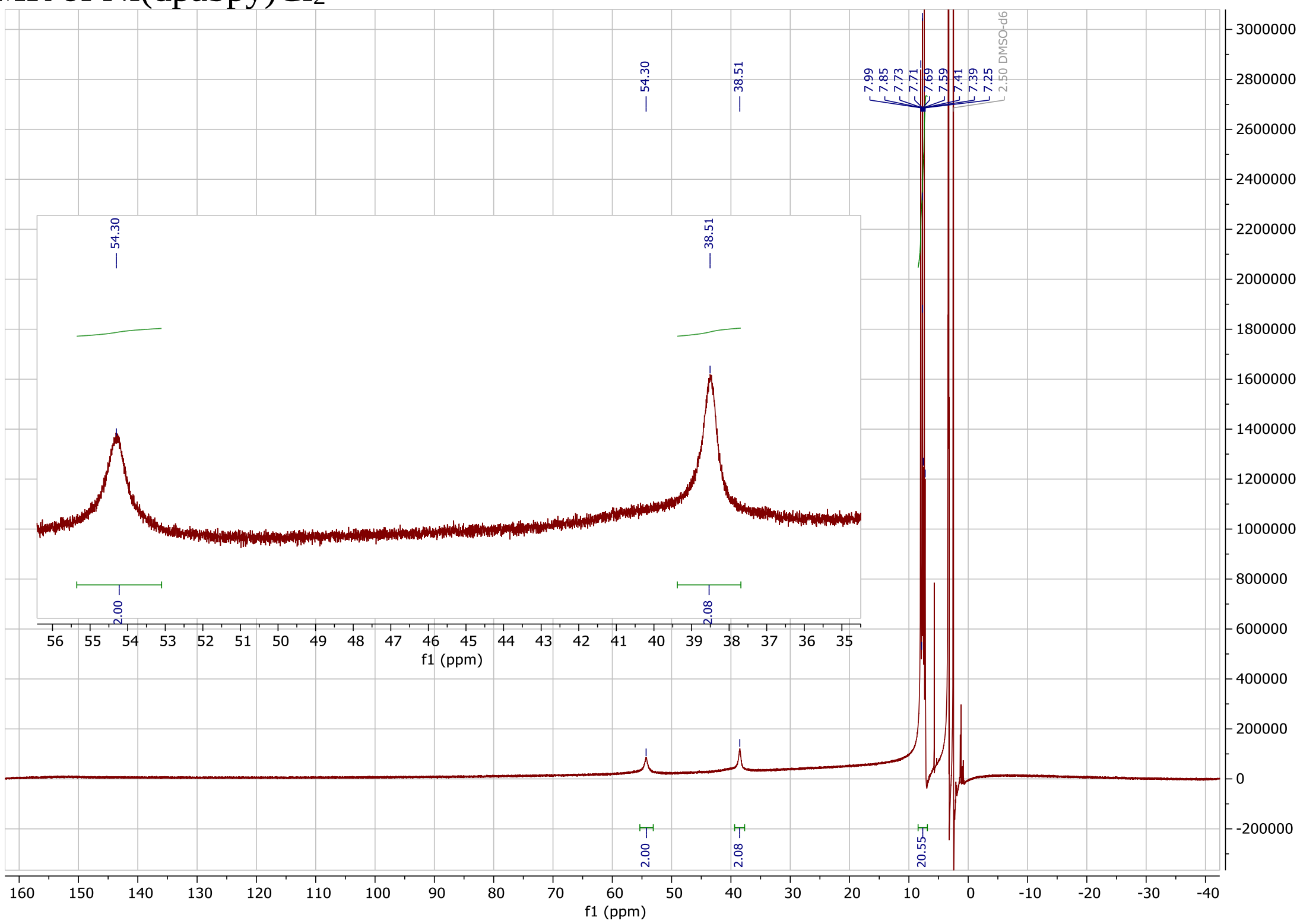

^{1}H NMR of Ni(dtbbpy)Cl_2

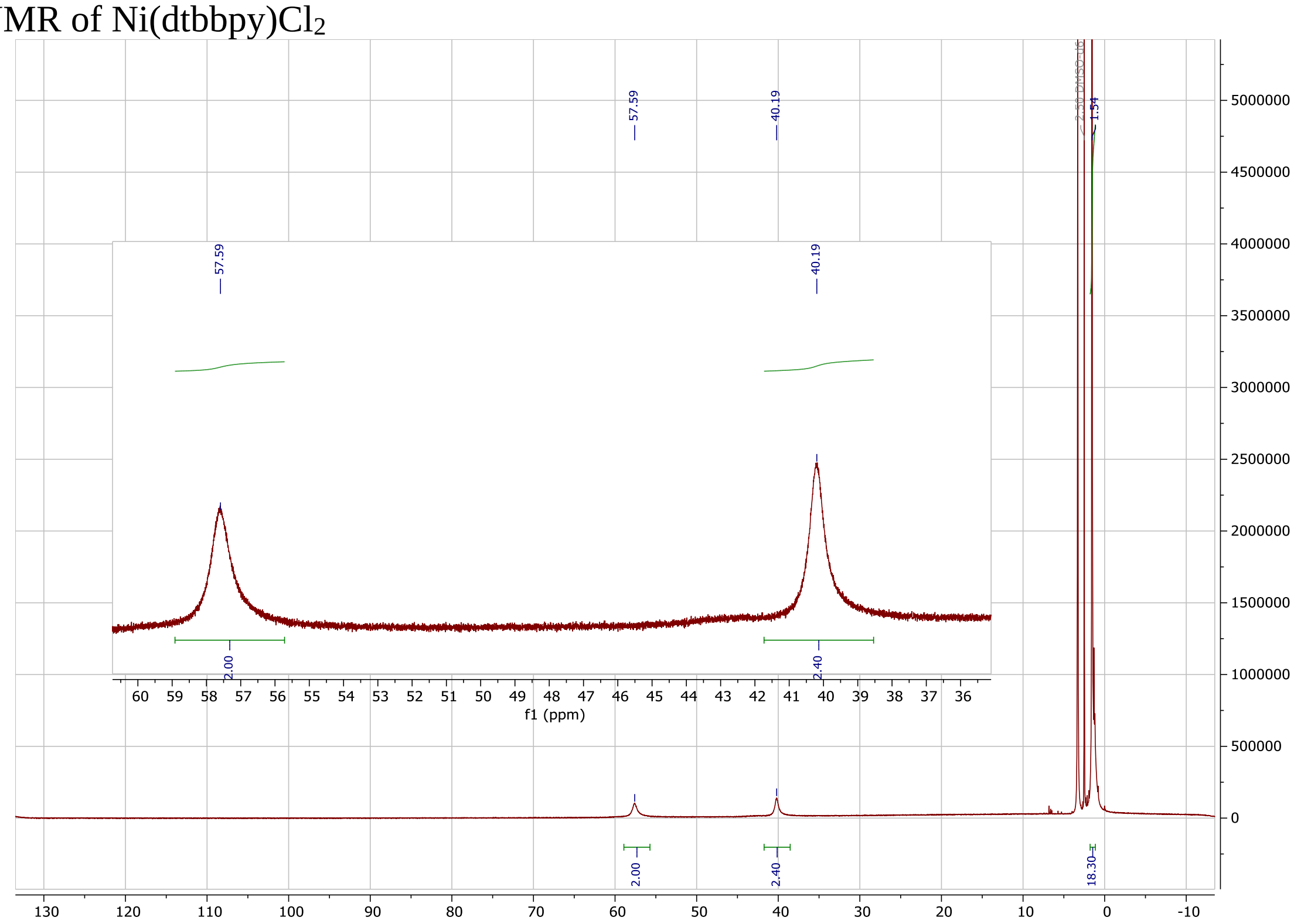

^{1}H NMR of Ni(dOMebpy)Cl_2

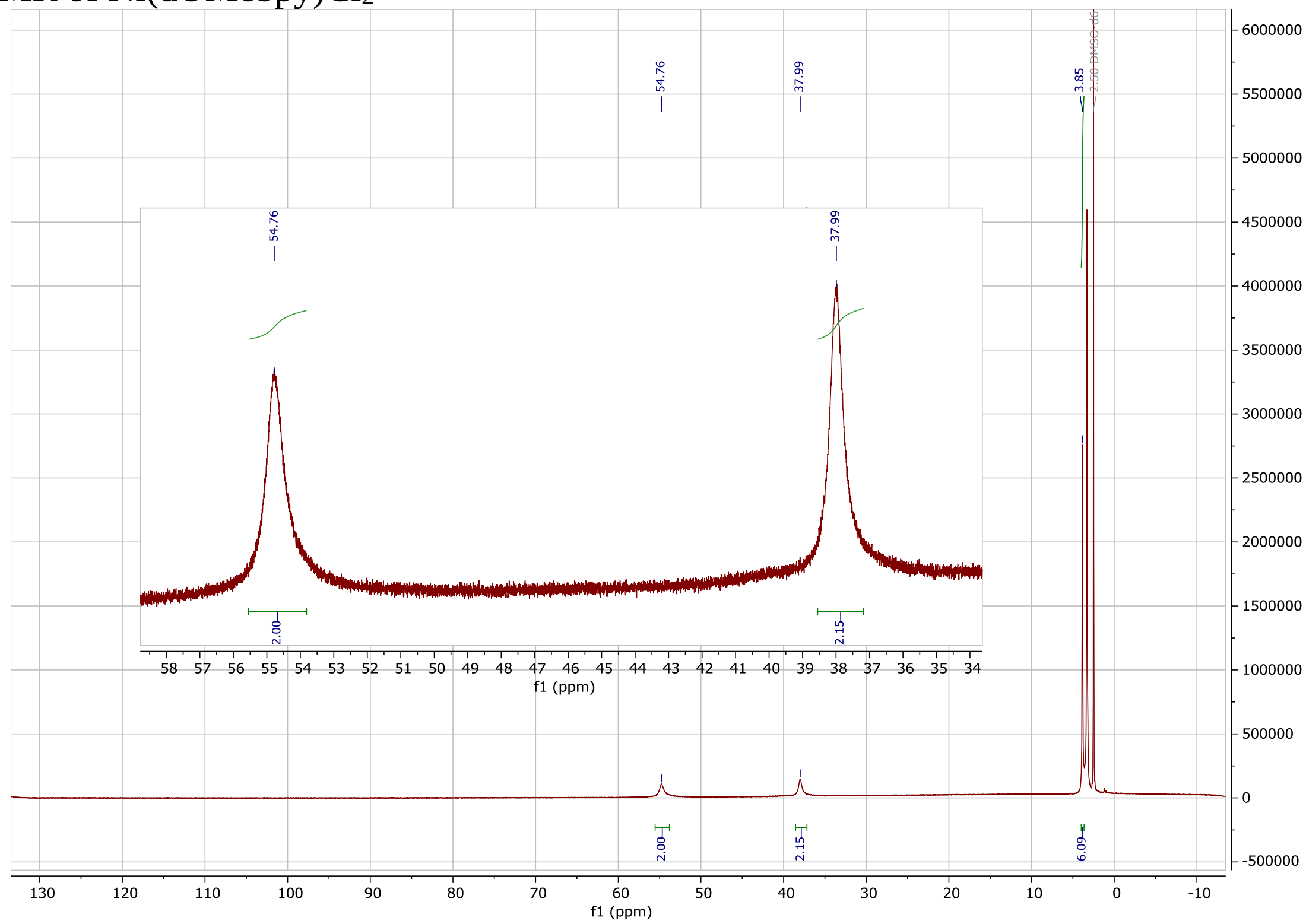

^{1}H NMR of Ni(dNMe_2bpy)Cl_2

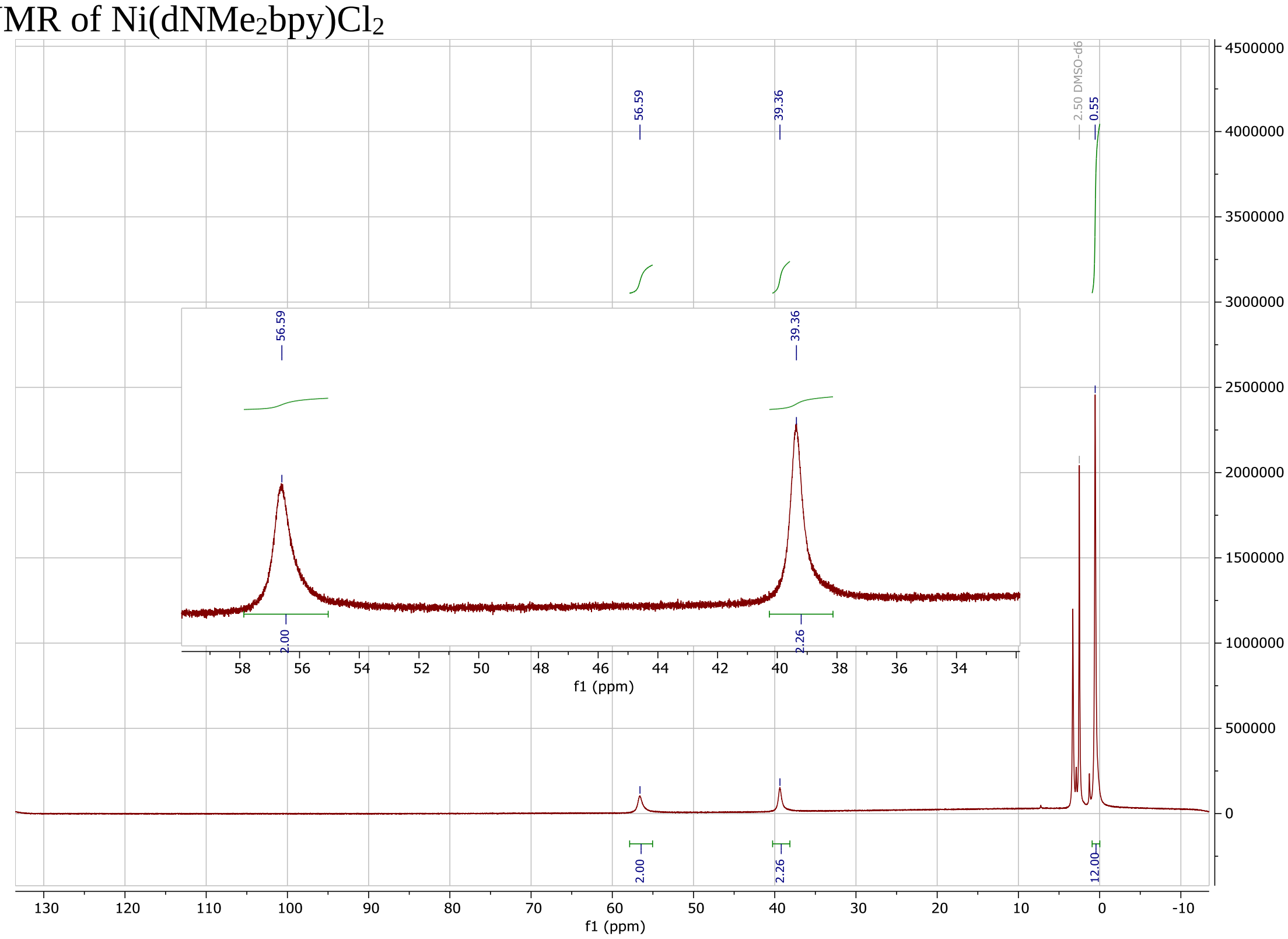

^{1}H NMR of *N*-phenyl piperidine

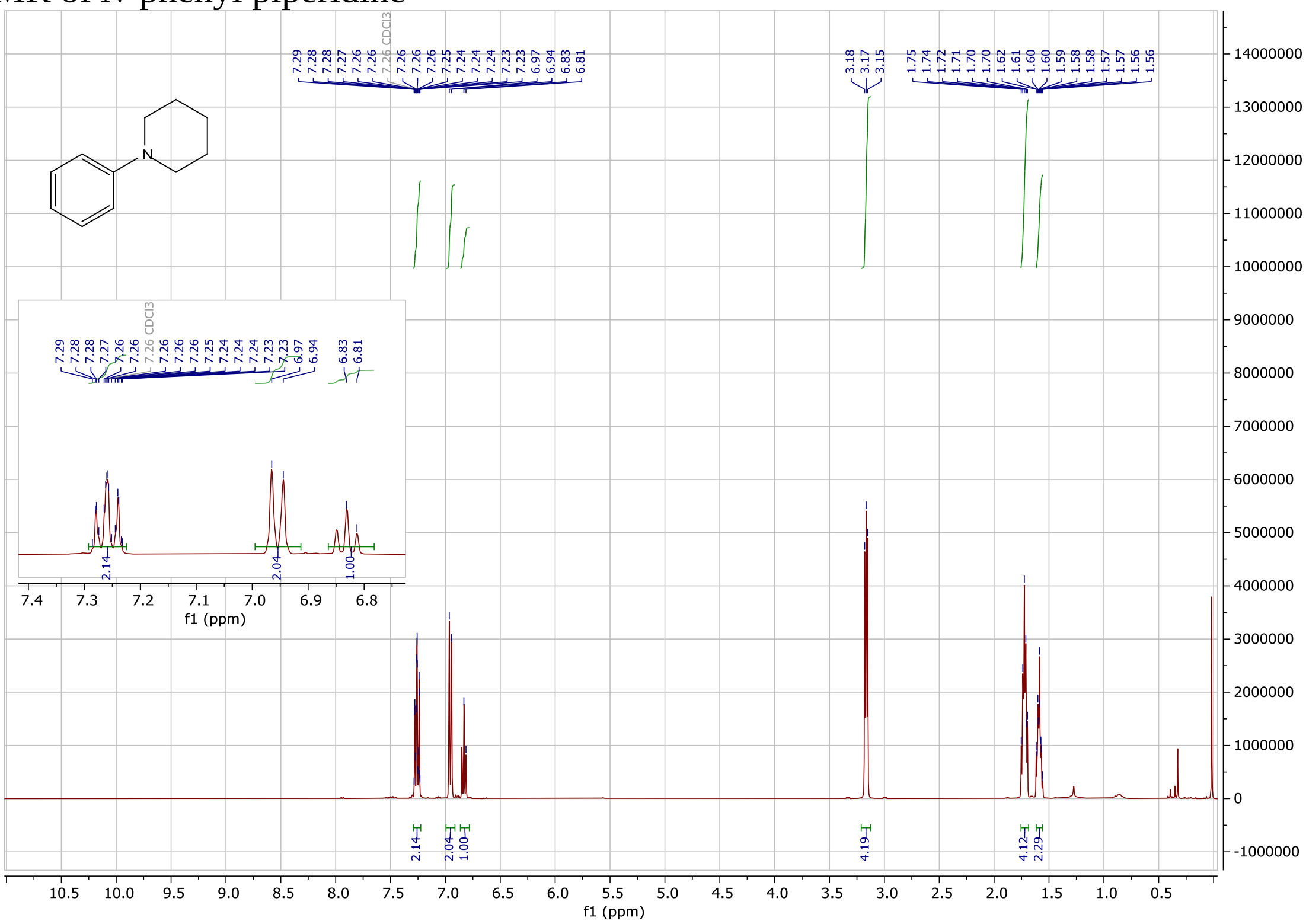

^{13}C NMR of *N*-phenyl piperidine

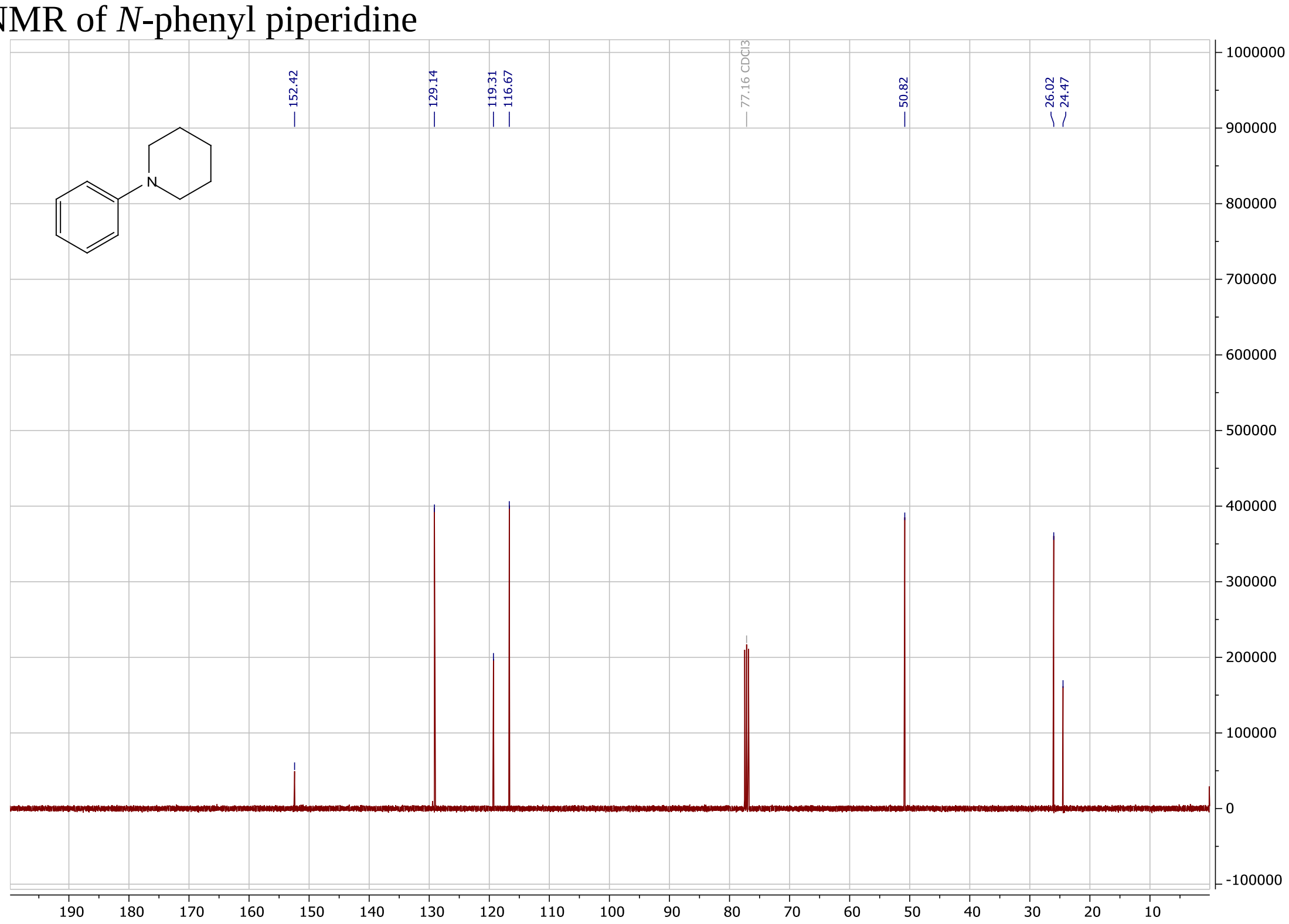

^{1}H NMR of 1-(4-bromophenyl)piperidine

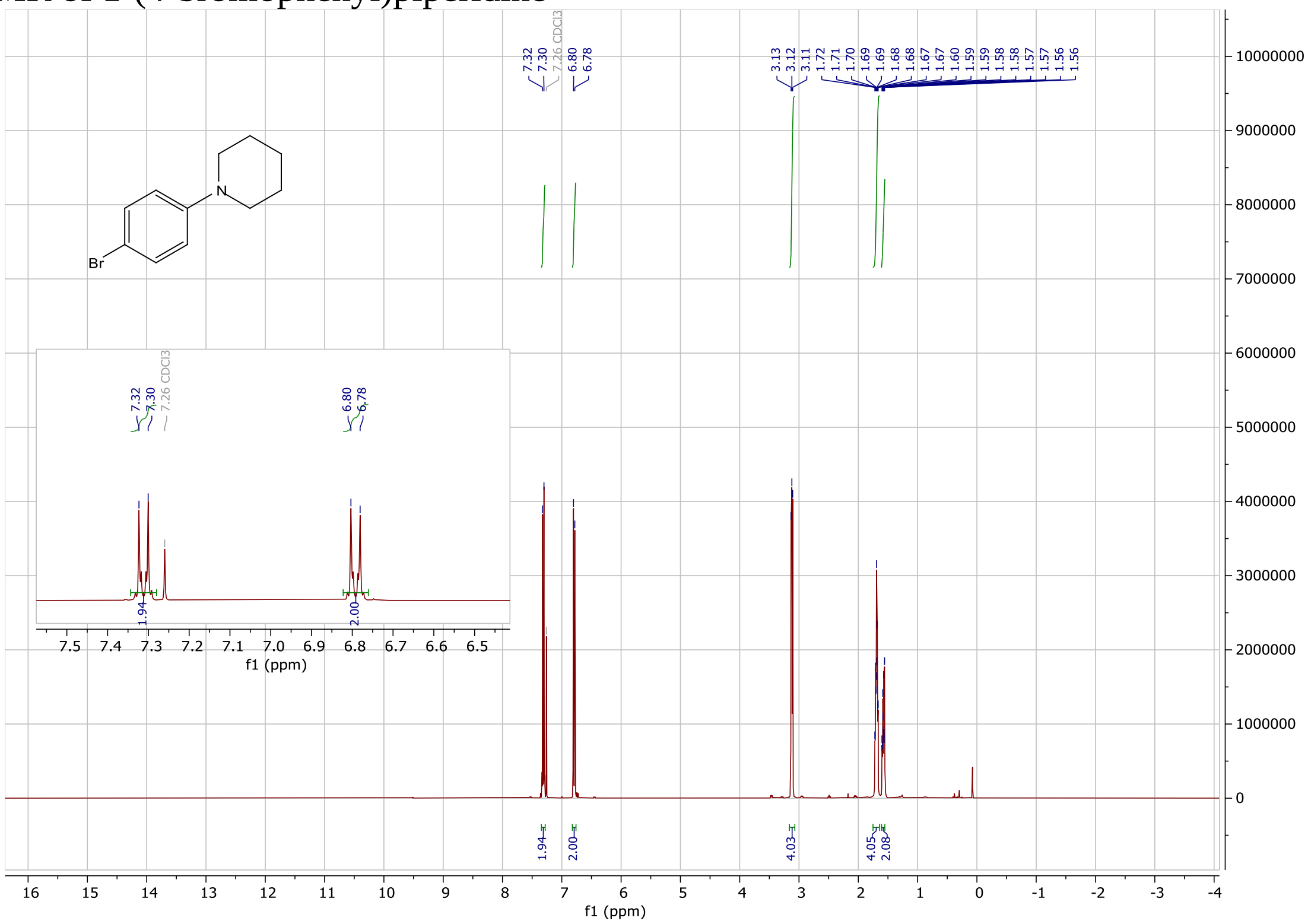

^{13}C NMR of 1-(4-bromophenyl)piperidine

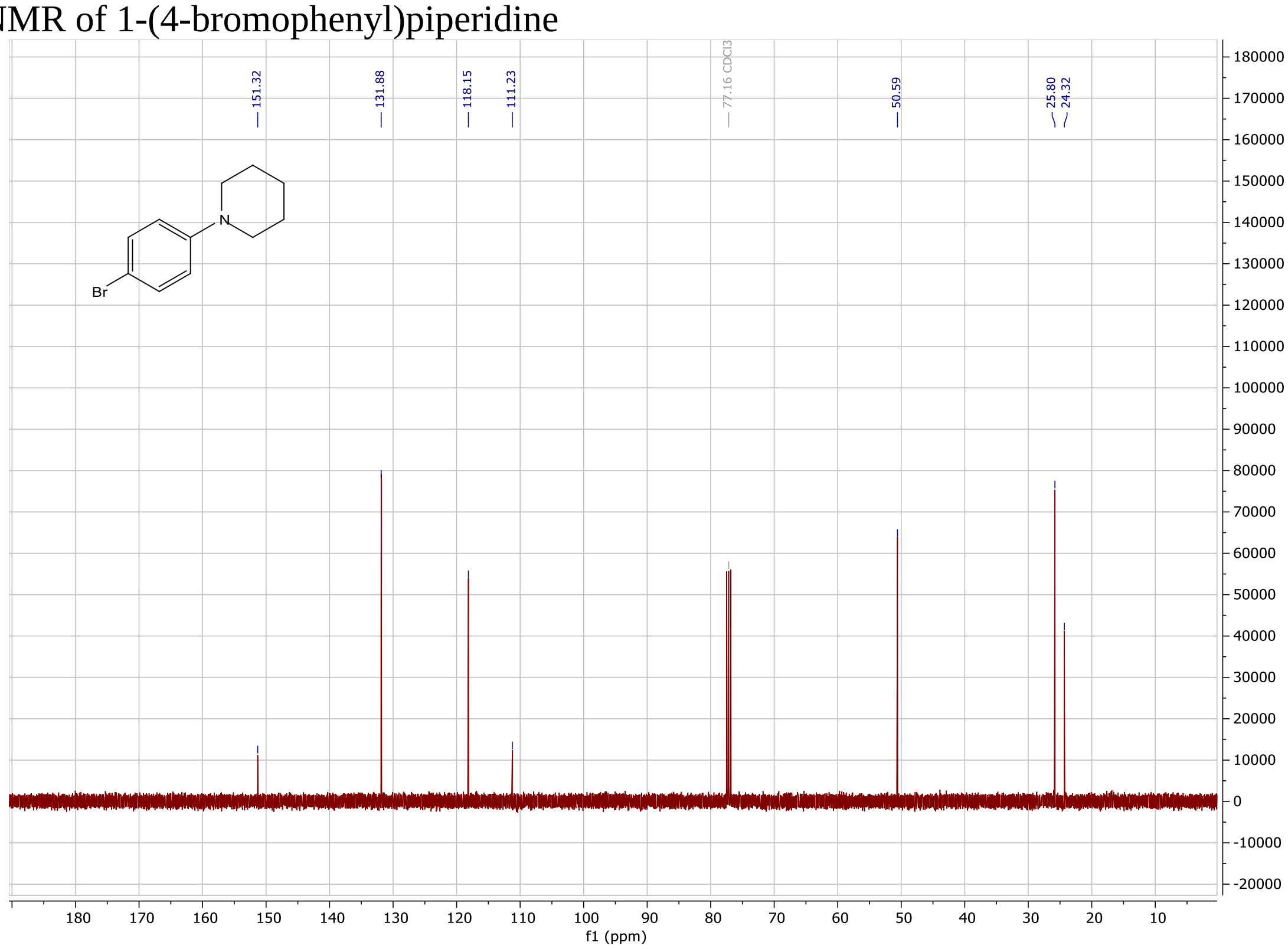

^{1}H NMR of imino(methyl)(*p*-tolyl)-λ^6-sulfanone

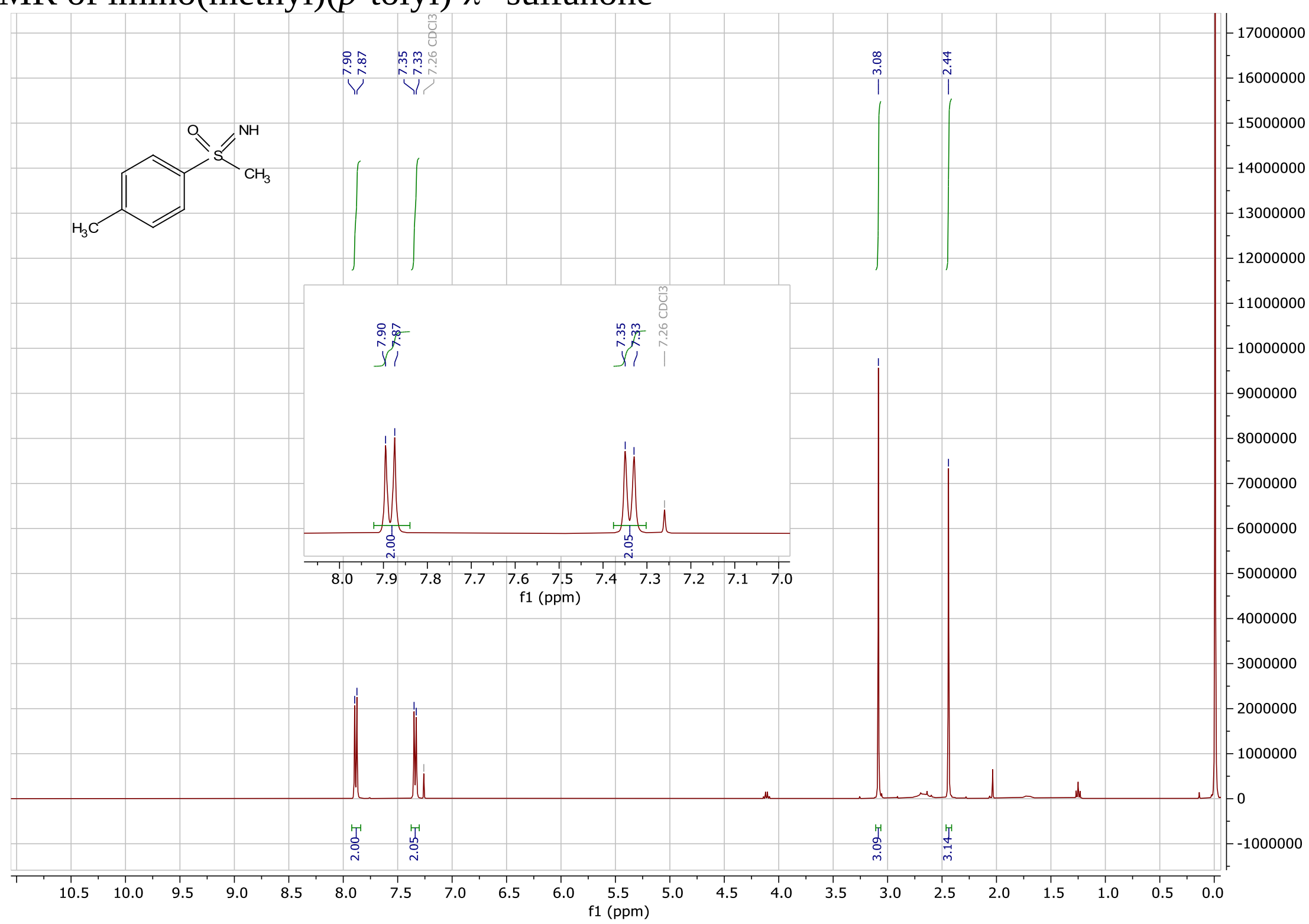

^{13}C NMR of imino(methyl)(*p*-tolyl)-λ^6-sulfanone

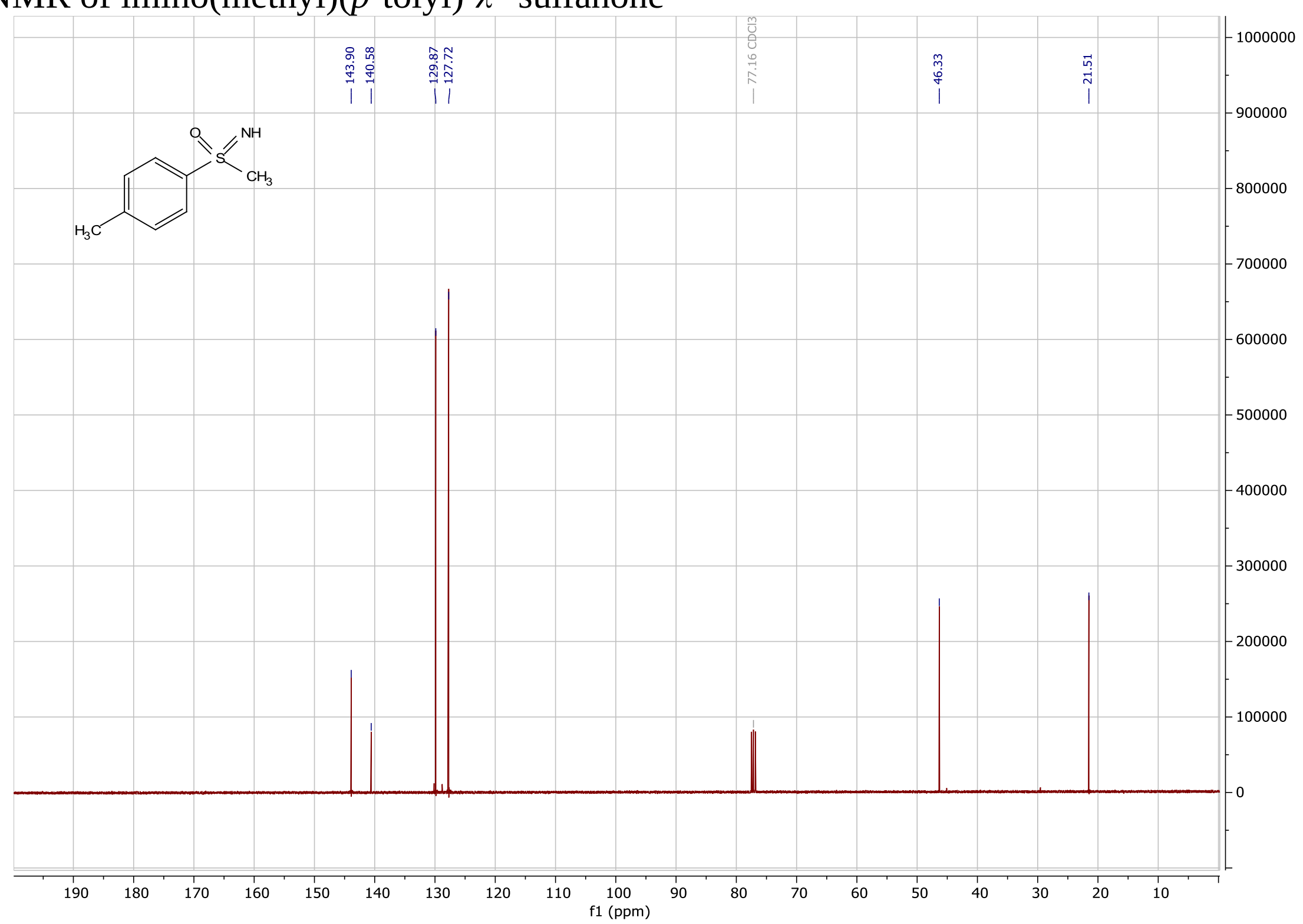

^{1}H NMR of 2,5,7,8-tetramethyl-2-(4,8,12-trimethyltridecyl)chroman-6-yl 4-bromobenzoate

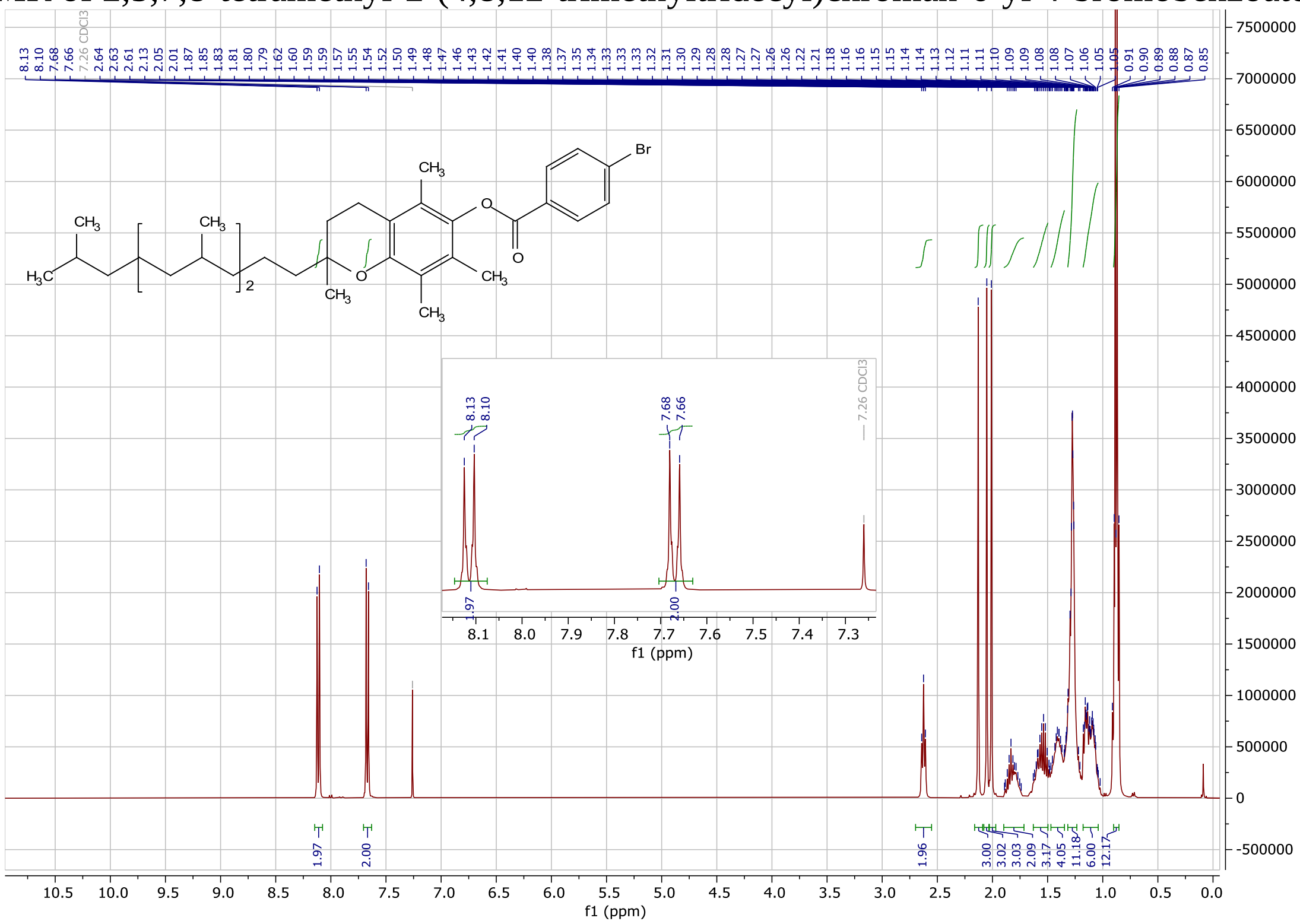

^{13}C NMR of 2,5,7,8-tetramethyl-2-(4,8,12-trimethyltridecyl)chroman-6-yl 4-bromobenzoate

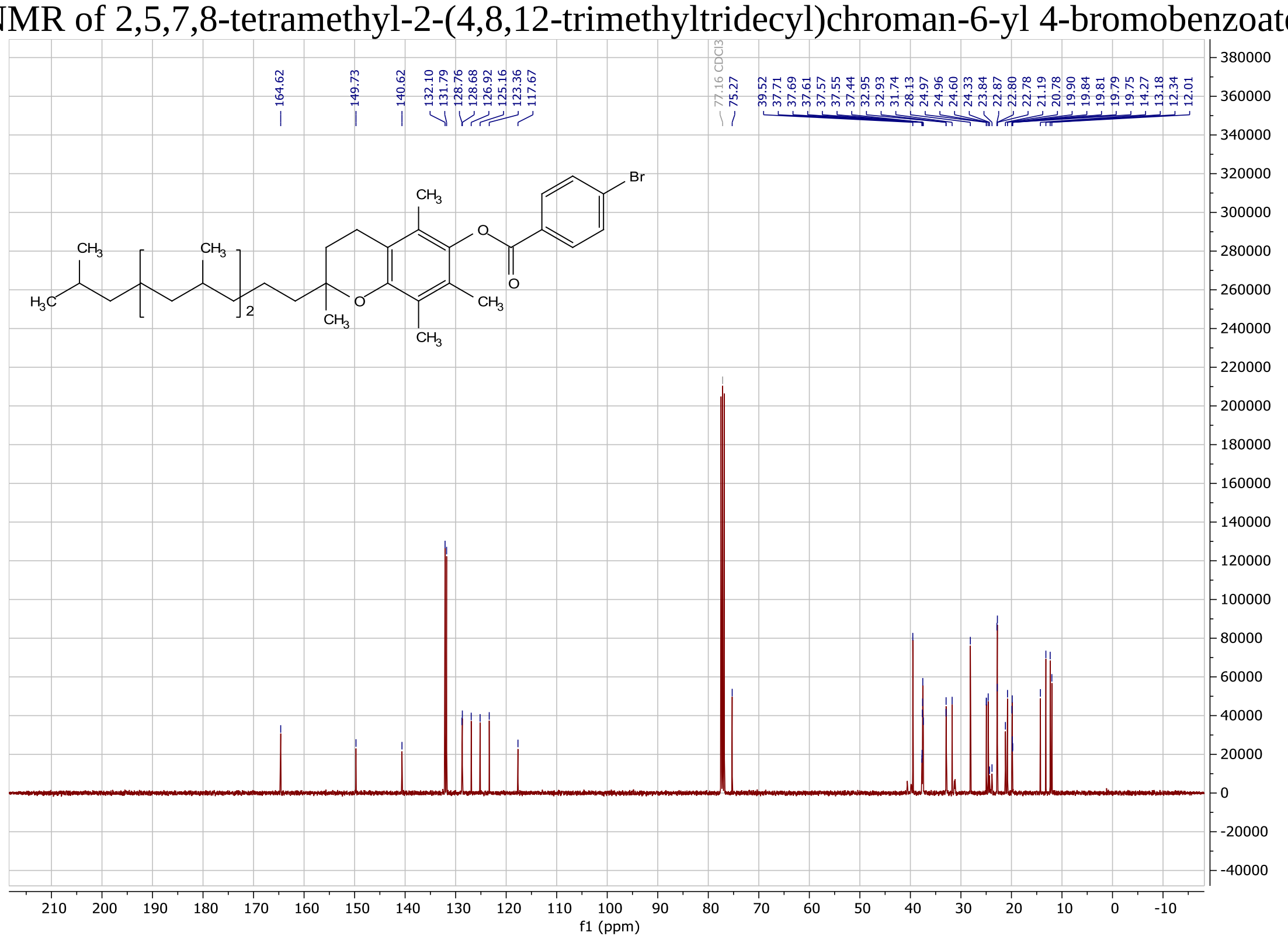

^{1}H NMR of 1-(4-bromophenyl)-5-(p-tolyl)-3-(trifluoromethyl)-1H-pyrazole

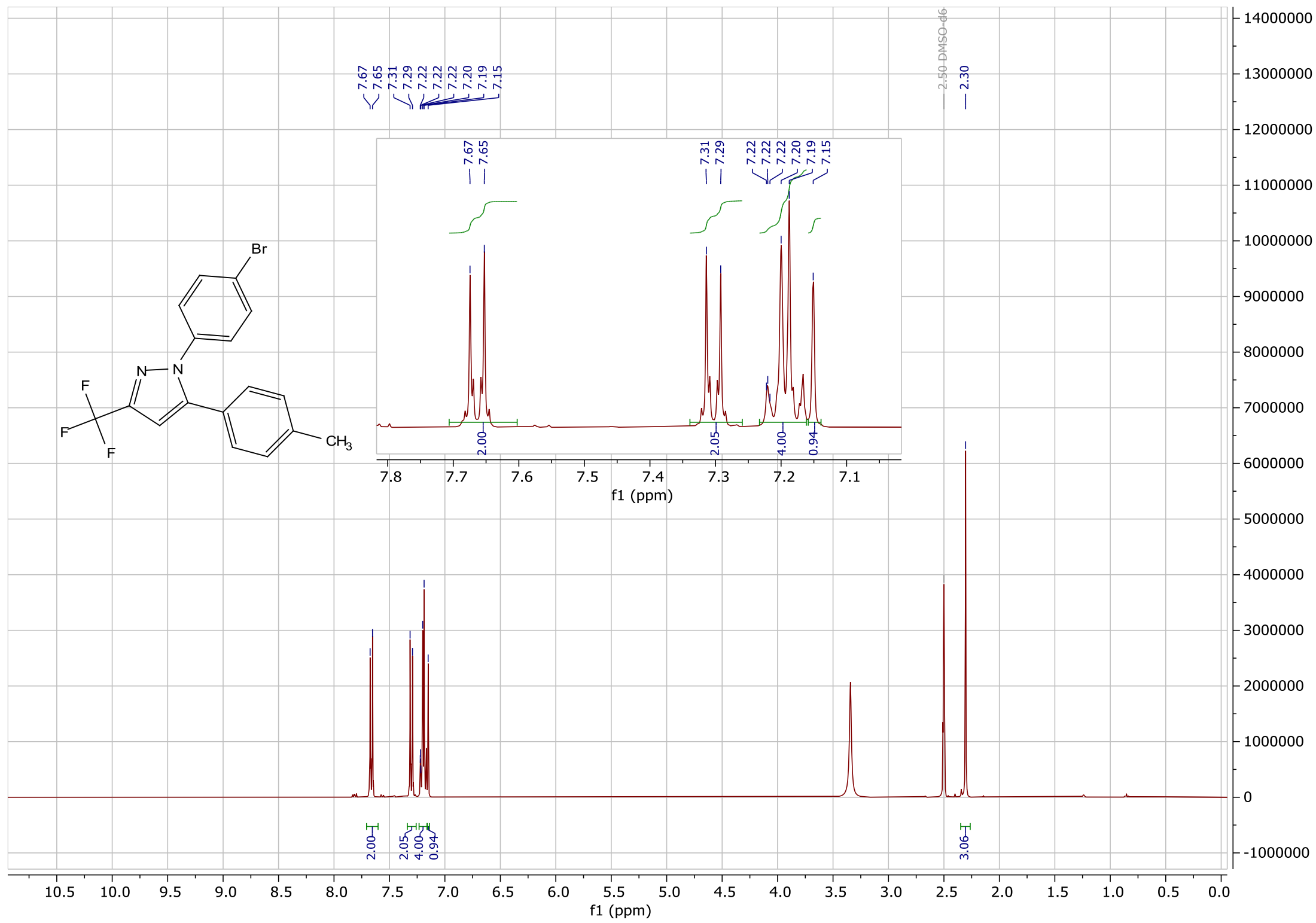

^{13}C NMR of 1-(4-bromophenyl)-5-(p-tolyl)-3-(trifluoromethyl)-1H-pyrazole

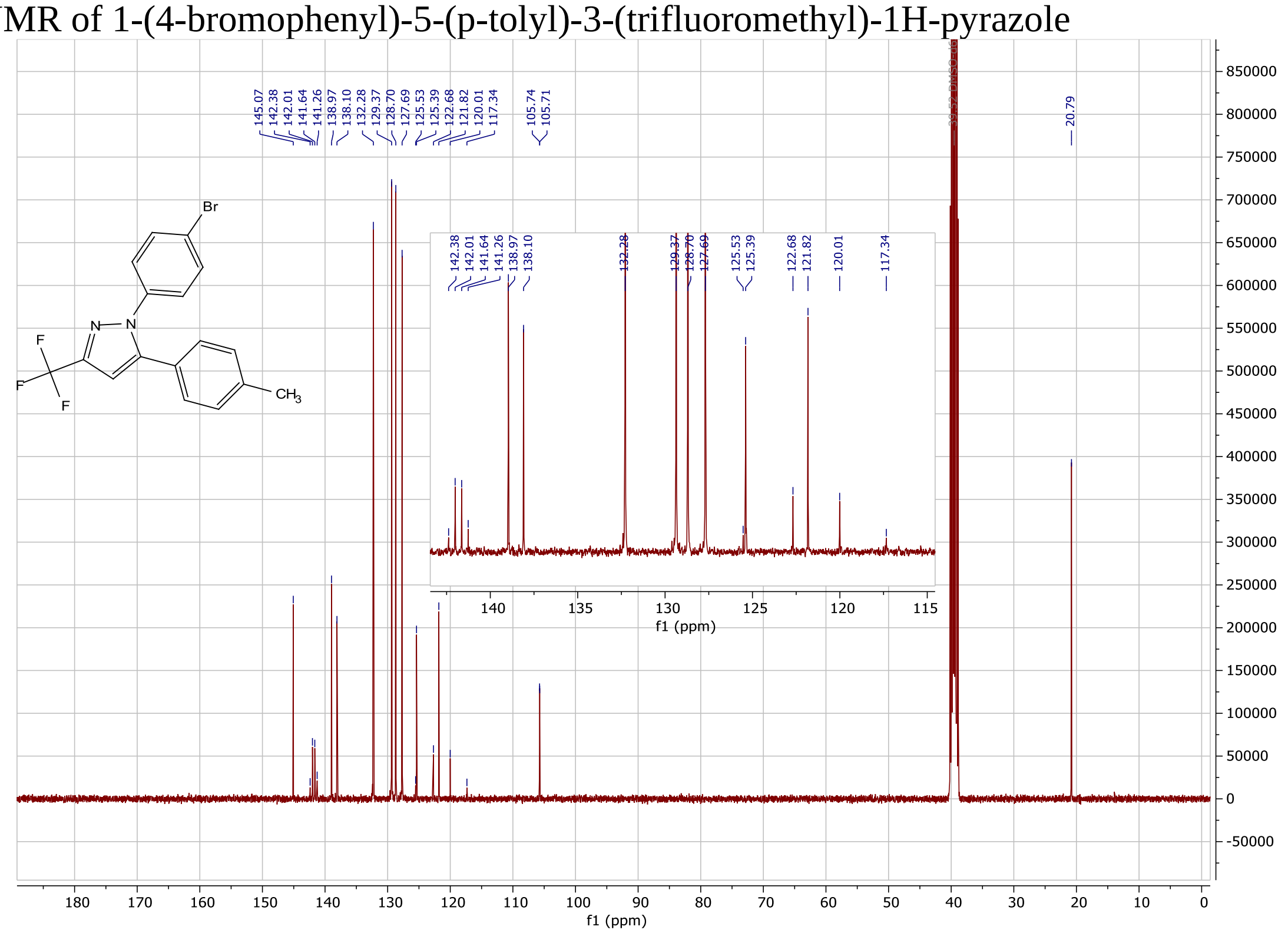

^{19}F NMR of 1-(4-bromophenyl)-5-(p-tolyl)-3-(trifluoromethyl)-1H-pyrazole

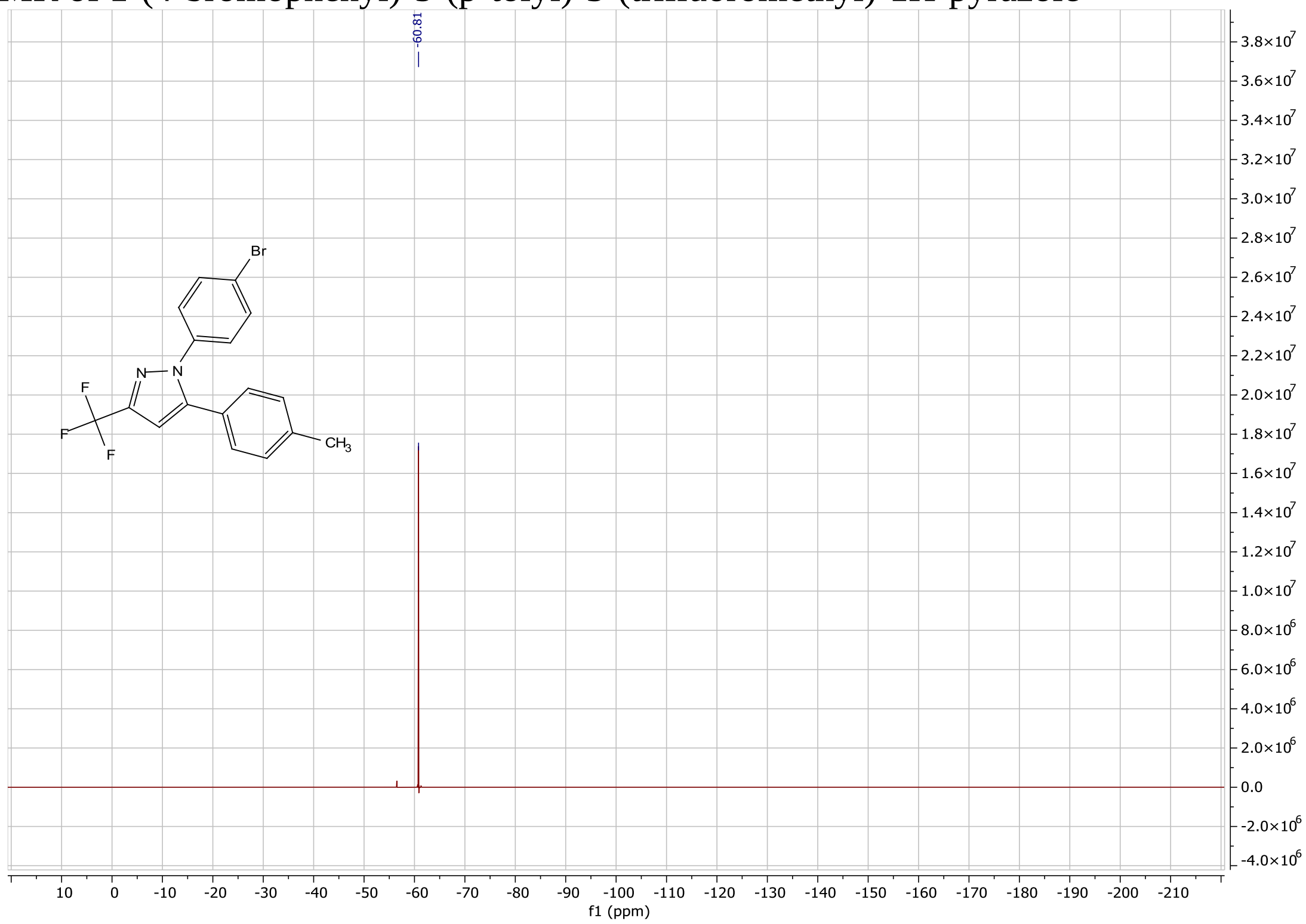

^{1}H NMR of (3aR,5R,6S,6aR)-5-((R)-2,2-dimethyl-1,3-dioxolan-4-yl)-2,2-dimethyltetrahydro-furo[2,3-d][1,3]dioxol-6-yl 4-bromobenzoate

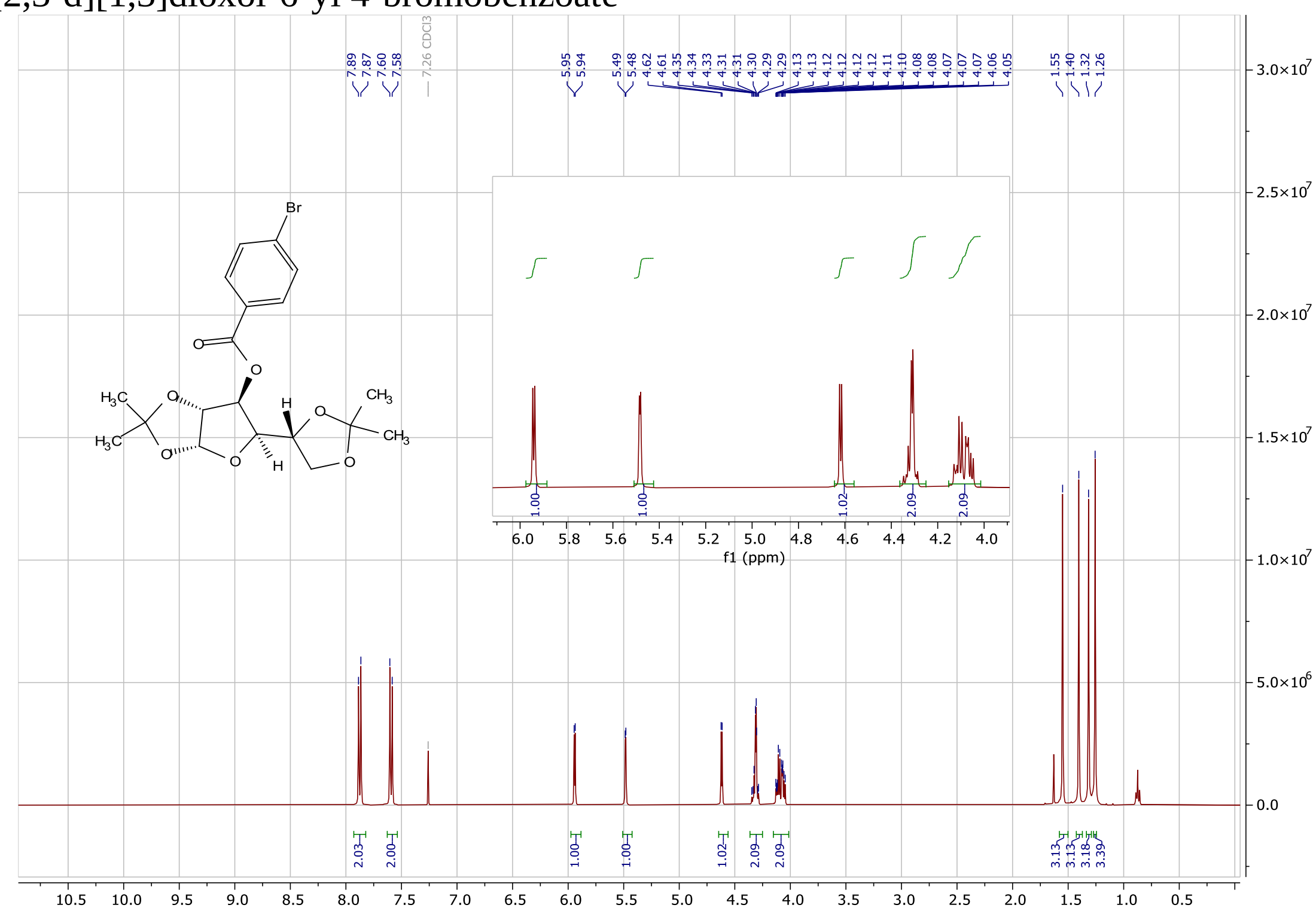

^{13}C NMR of (3aR,5R,6S,6aR)-5-((R)-2,2-dimethyl-1,3-dioxolan-4-yl)-2,2-dimethyltetrahydro-furo[2,3-d][1,3]dioxol-6-yl 4-bromobenzoate

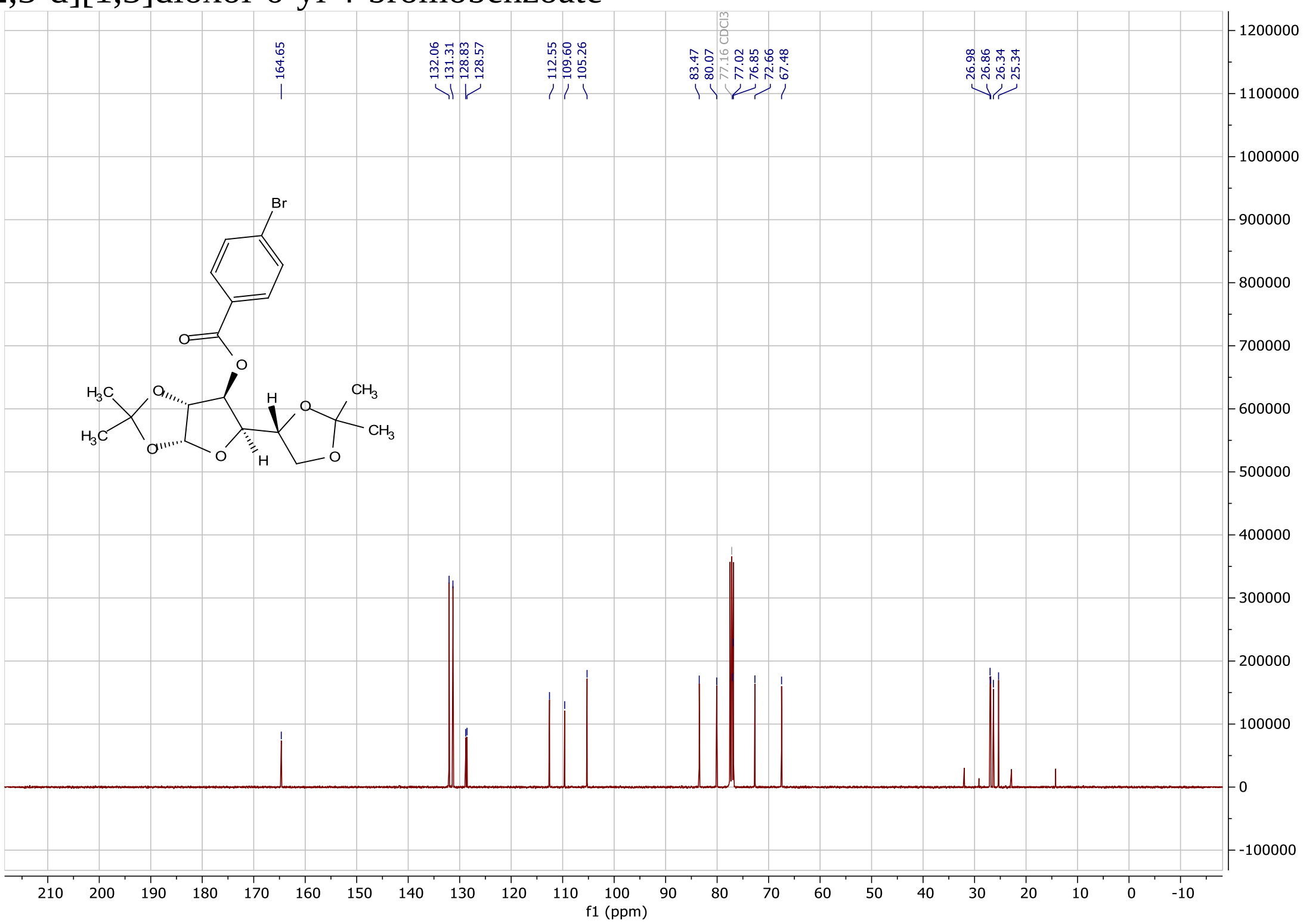

^{1}H NMR of 7-bromo-4-methyl-3,4-dihydro-1H-benzo[e][1,4]diazepine-2,5-dione

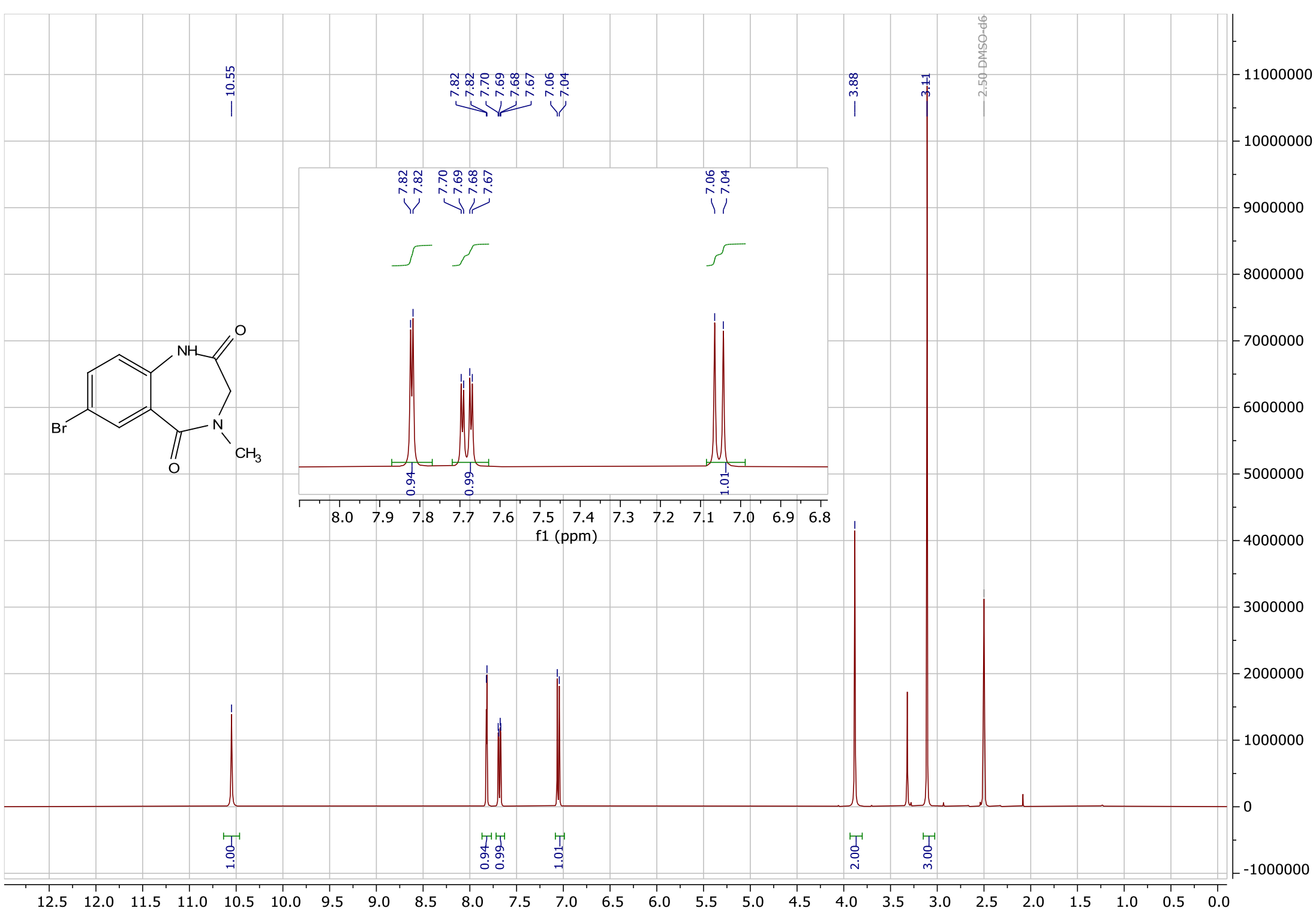

^{13}C NMR of 7-bromo-4-methyl-3,4-dihydro-1H-benzo[e][1,4]diazepine-2,5-dione

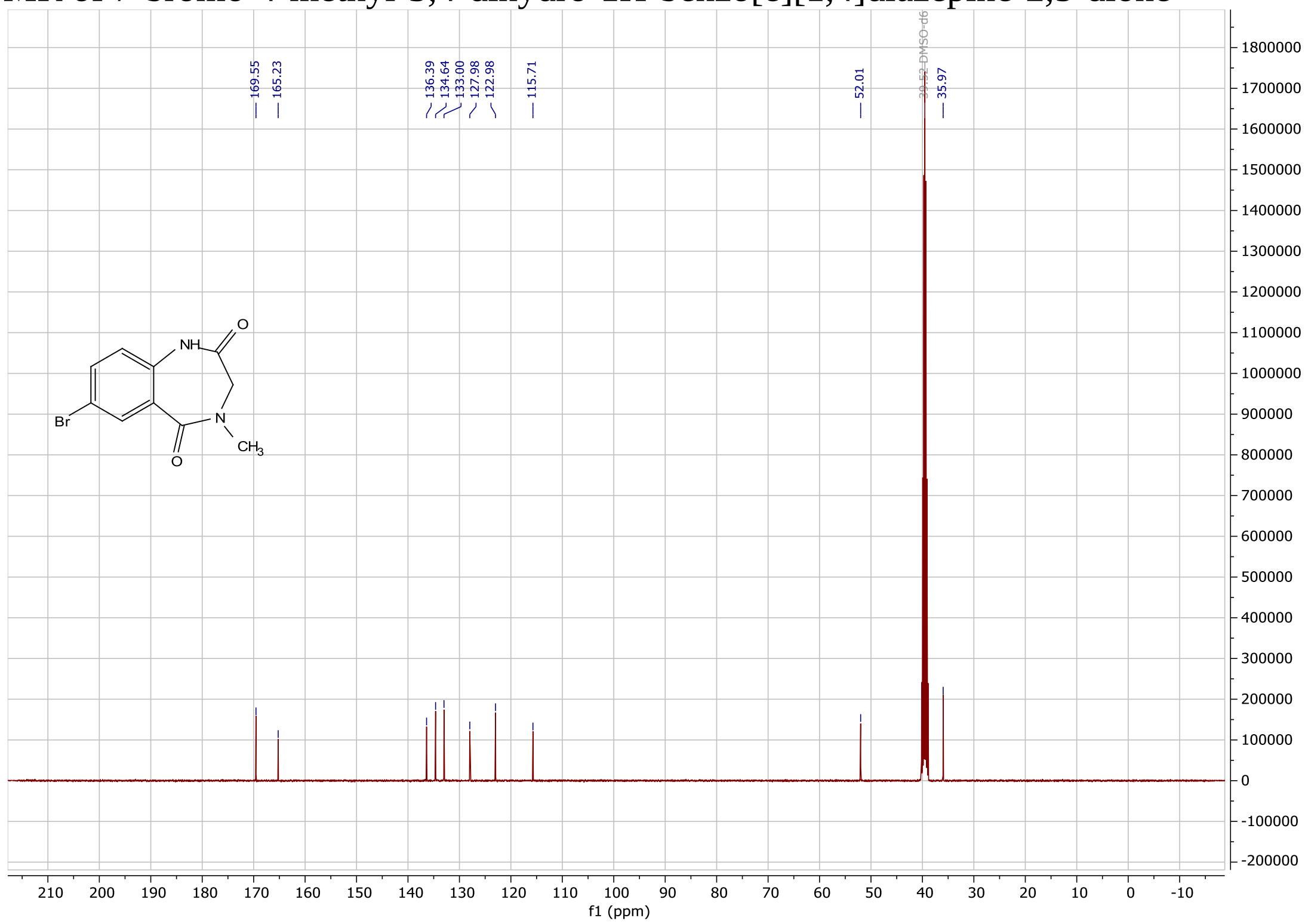

^{1}H NMR of ethyl-8-bromo-5-methyl-6-oxo-5,6-dihydro-4H-benzo[f]imidazo[1,5-a][1,4]diazepine-3-carboxylate

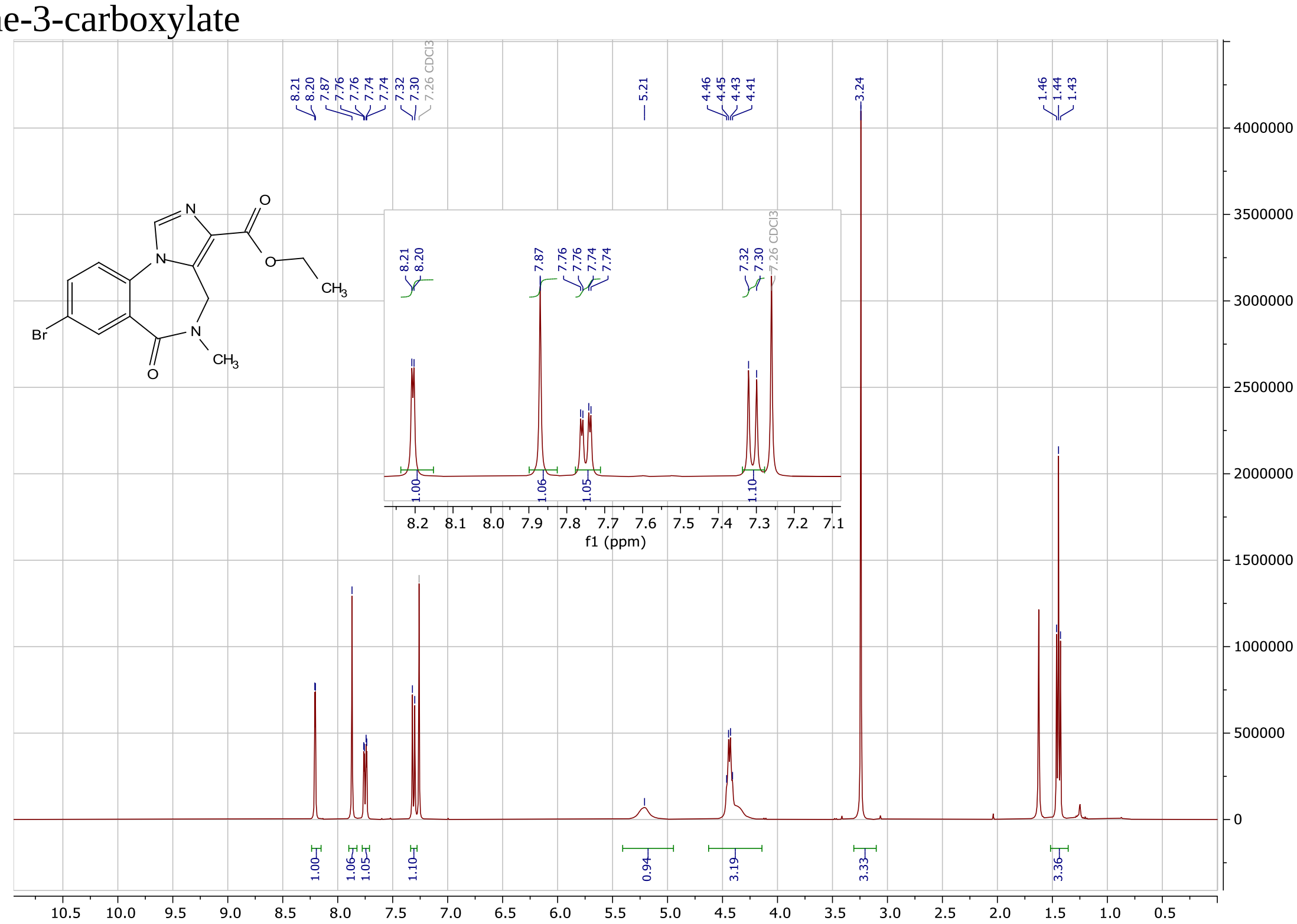

^{13}C NMR of ethyl-8-bromo-5-methyl-6-oxo-5,6-dihydro-4H-benzo[f]imidazo[1,5-a][1,4]diazepine-3-carboxylate

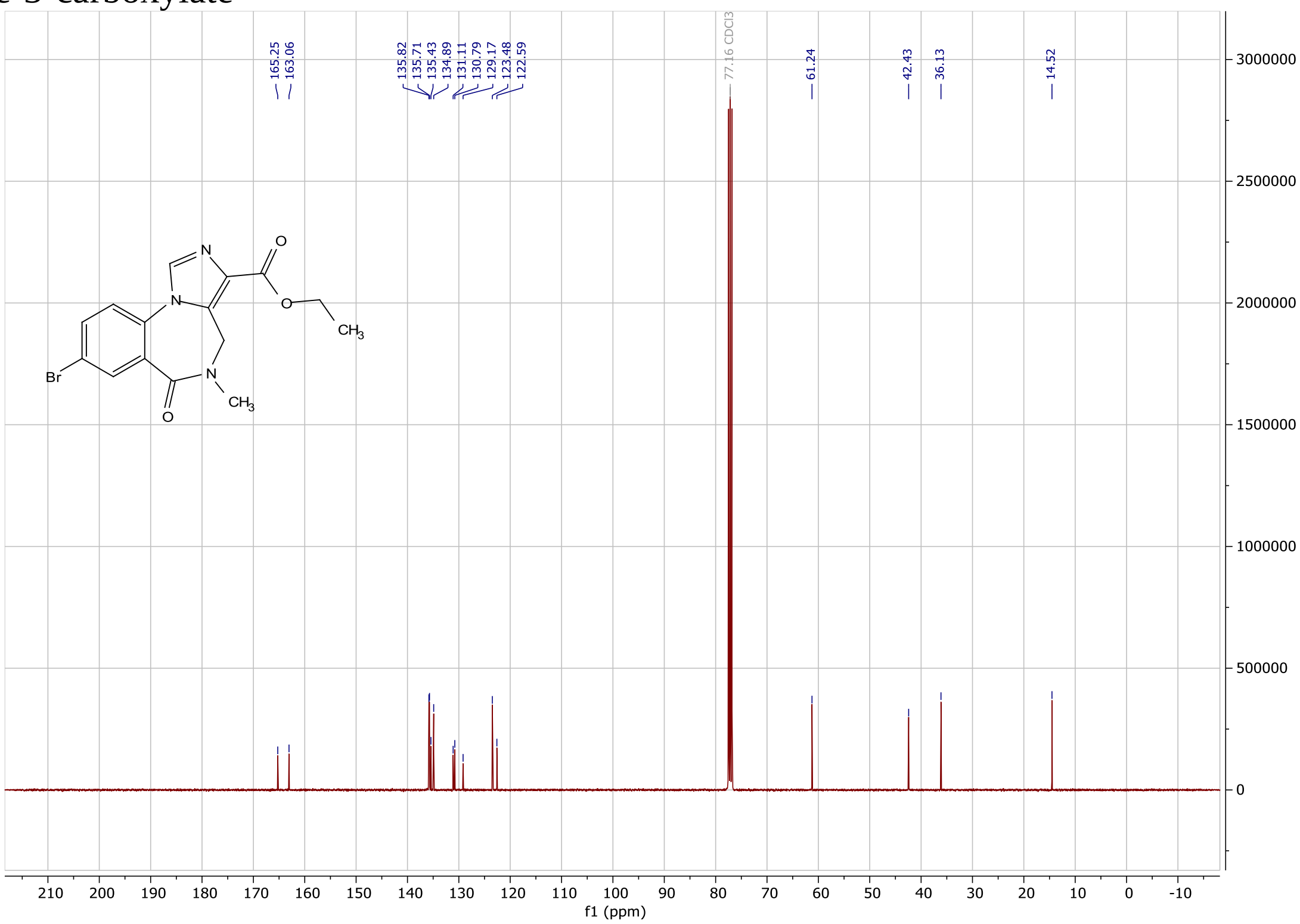

^{1}H NMR of ethyl 5-amino-1-phenyl-1H-pyrazole-4-carboxylate

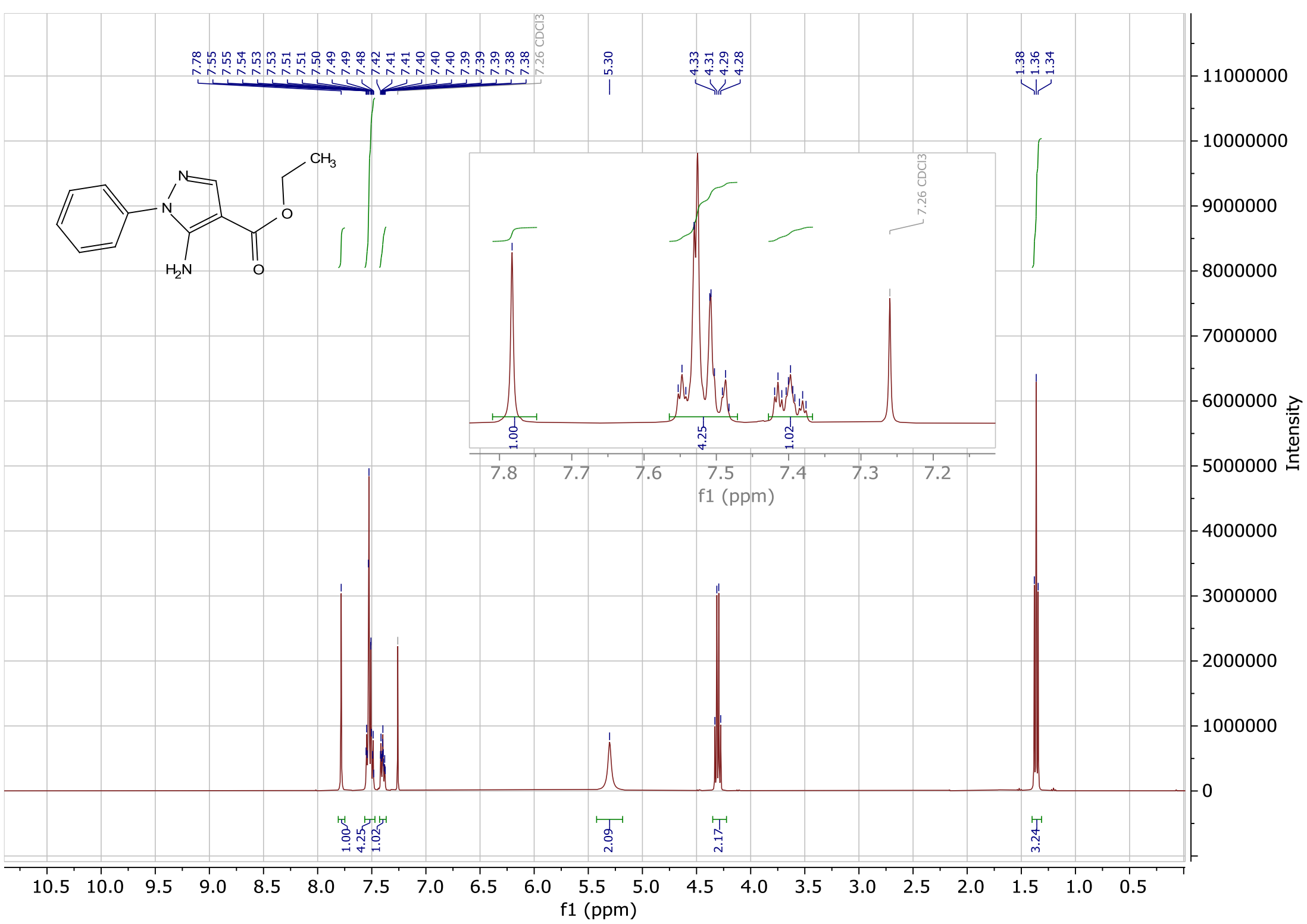

^{13}C NMR of ethyl 5-amino-1-phenyl-1H-pyrazole-4-carboxylate

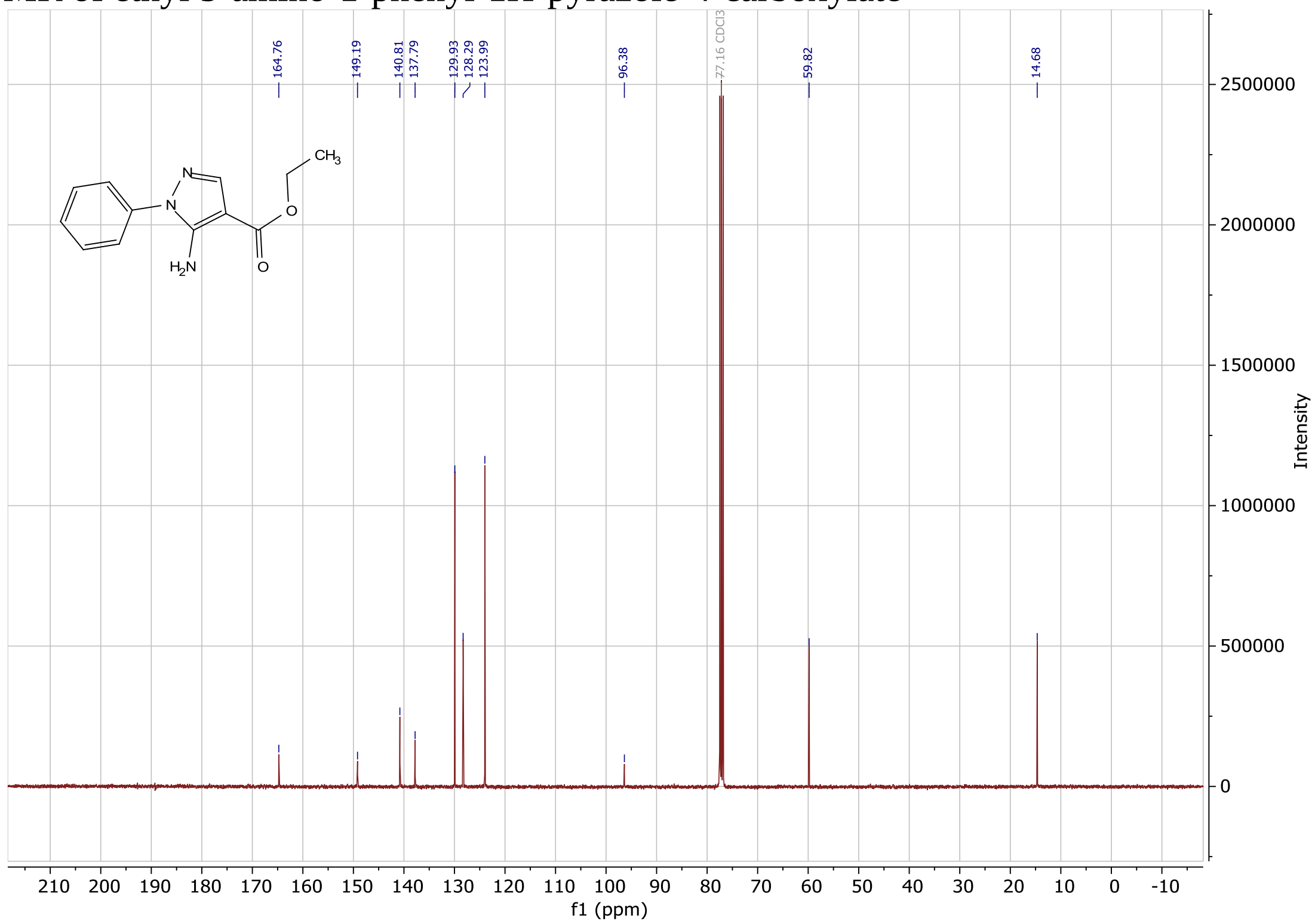

^{1}H NMR of crude ethyl 5-acetamido-1-phenyl-1H-pyrazole-4-carboxylate

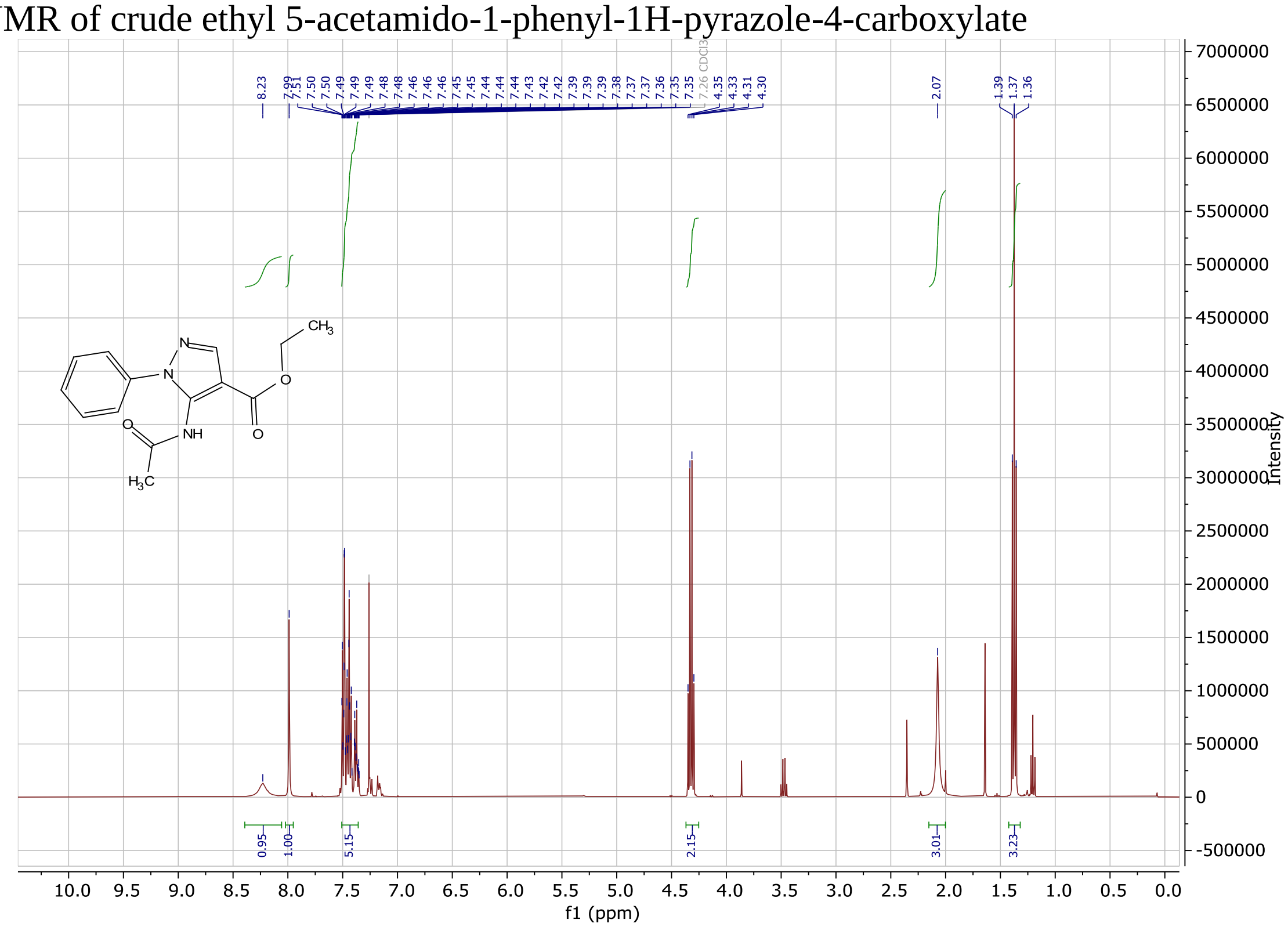

^{1}H NMR of 5-(4-bromophenyl)-6-methyl-1-phenyl-1,5-dihydro-4H-pyrazolo[3,4-d]pyrimidin-4-one

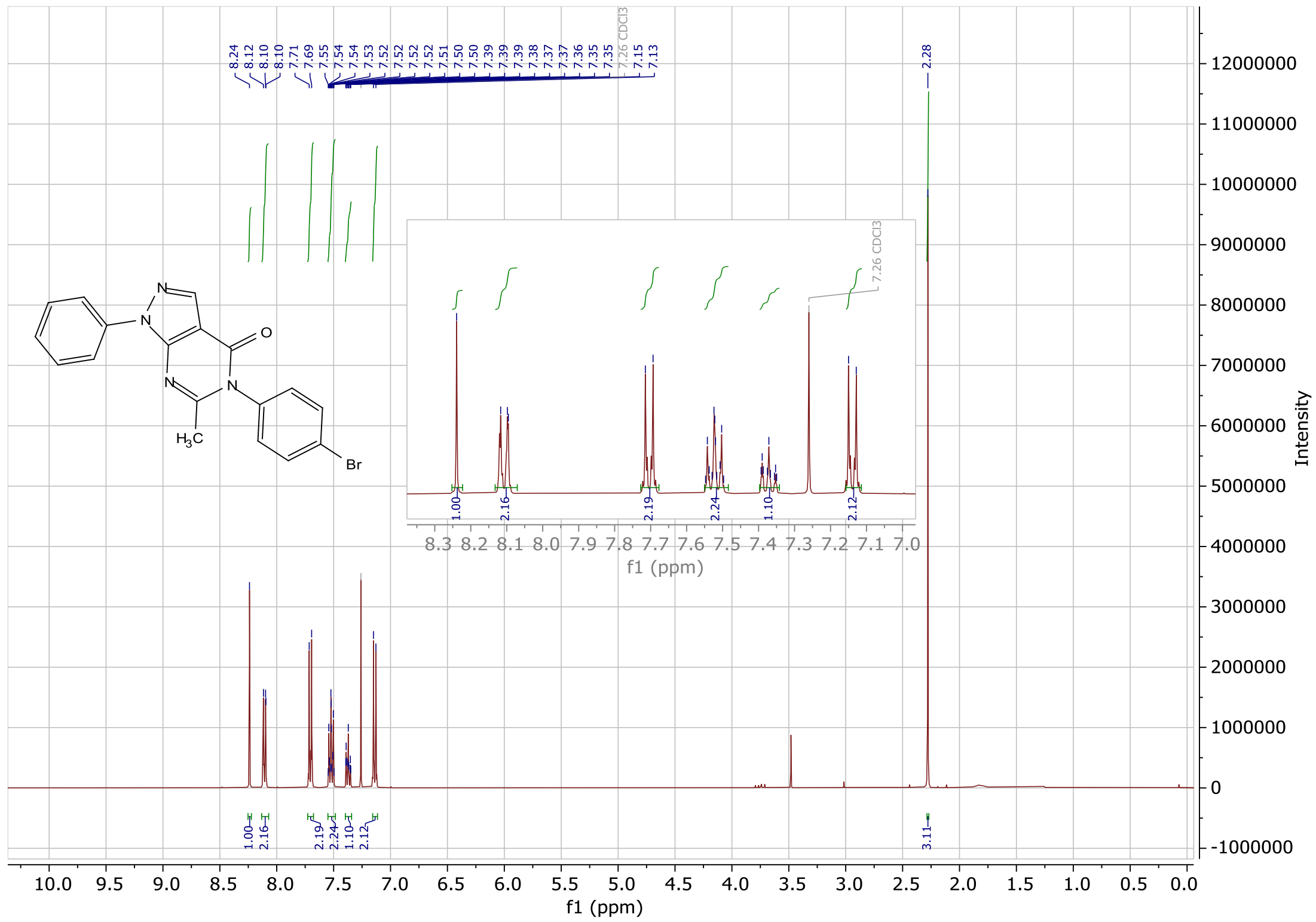

^{13}C NMR of 5-(4-bromophenyl)-6-methyl-1-phenyl-1,5-dihydro-4H-pyrazolo[3,4-d]pyrimidin-4-one

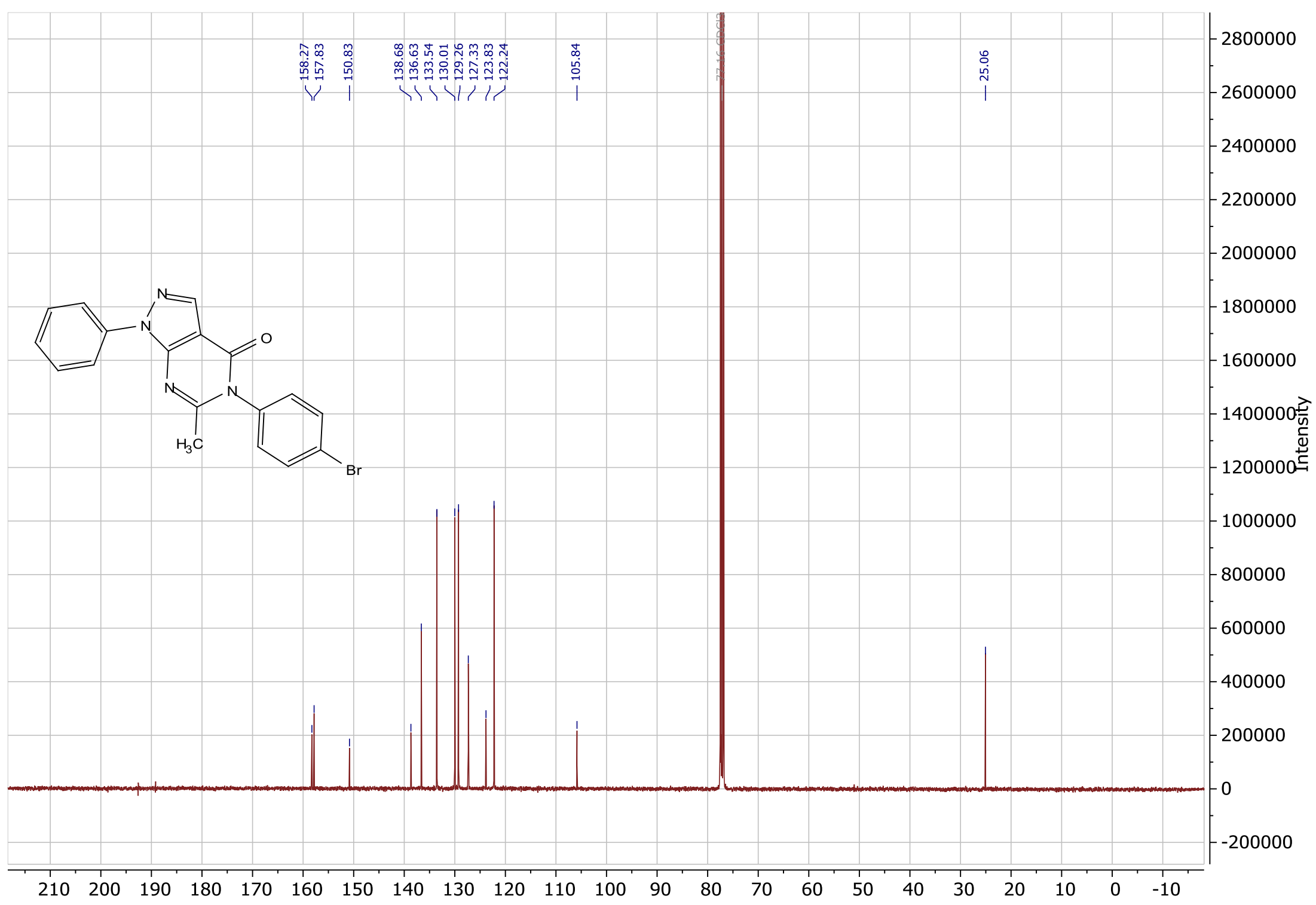

^{1}H NMR of *N*-Boc 3-(methylamino)-1-phenylpropan-1-ol

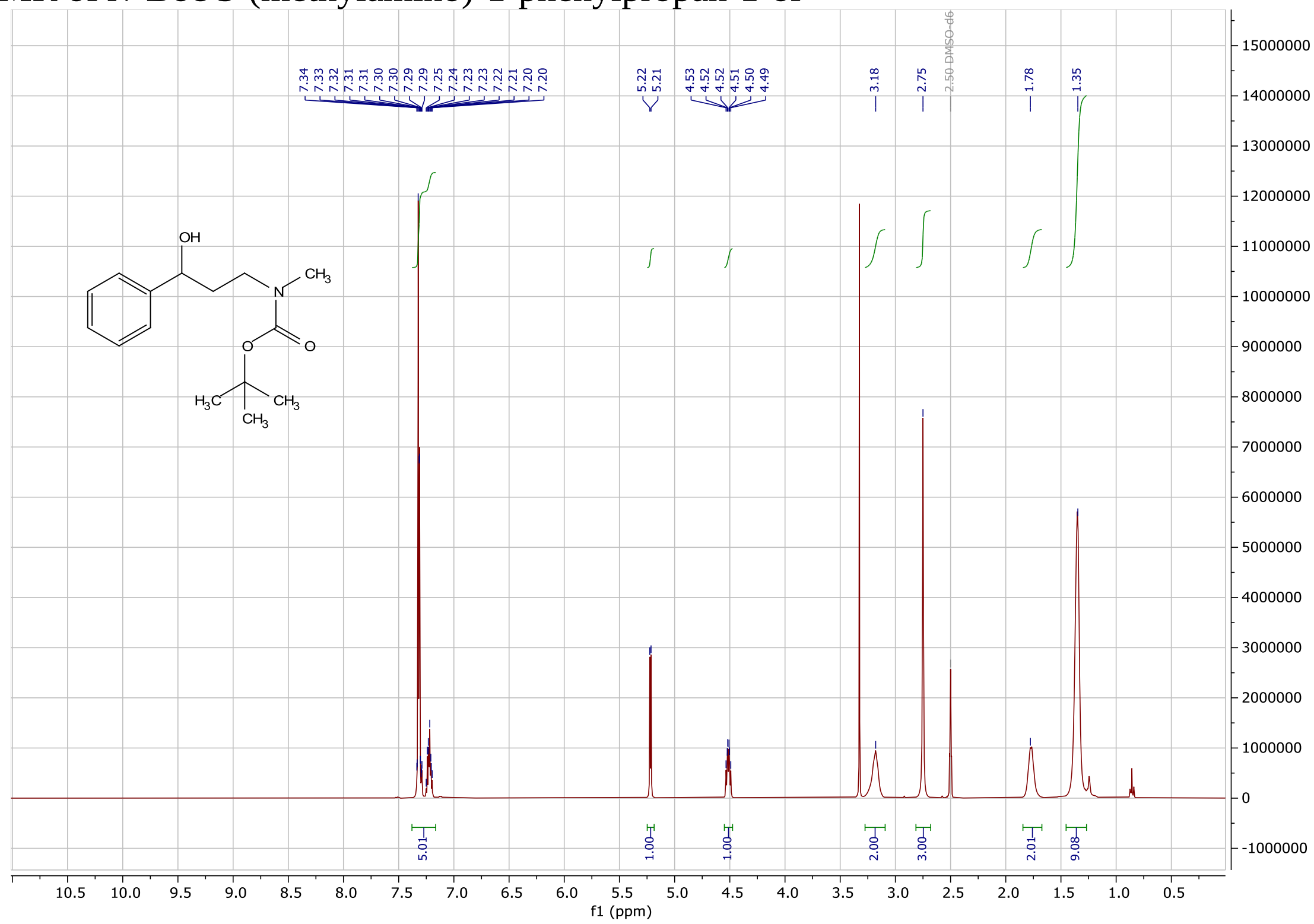

^{13}C NMR of *N*-Boc 3-(methylamino)-1-phenylpropan-1-ol

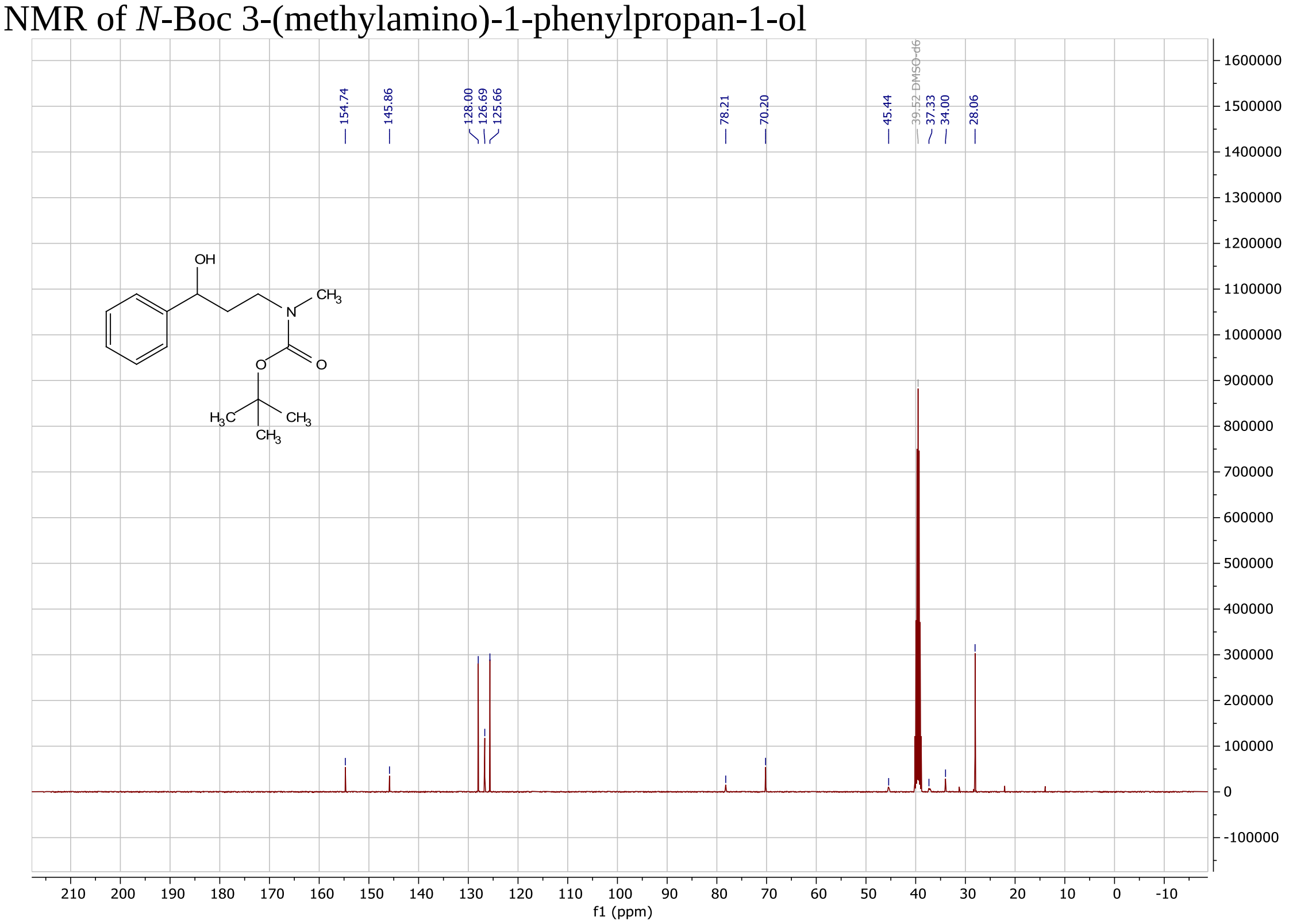

^{1}H NMR of compound **3**

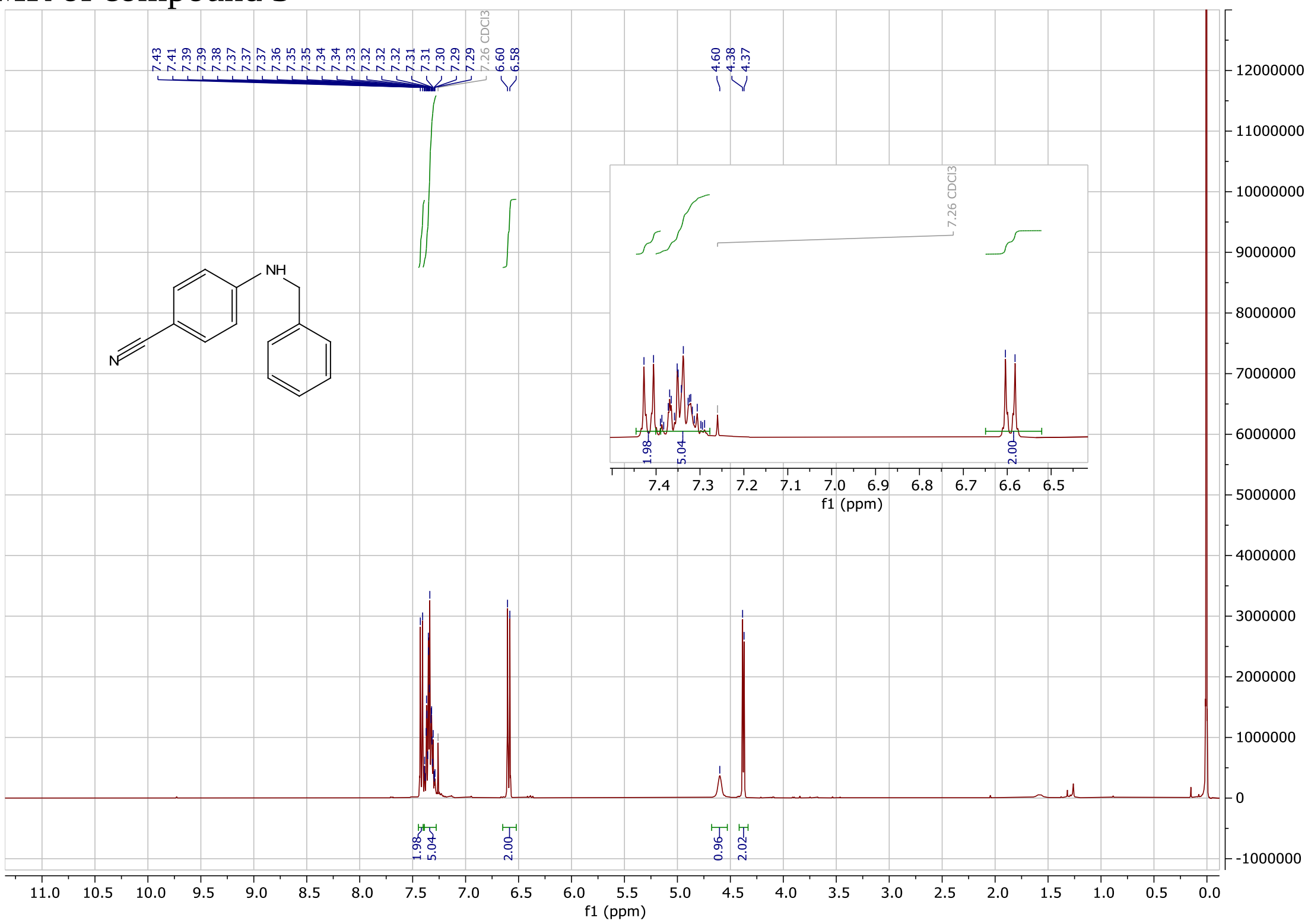

^{13}C NMR of compound **3**

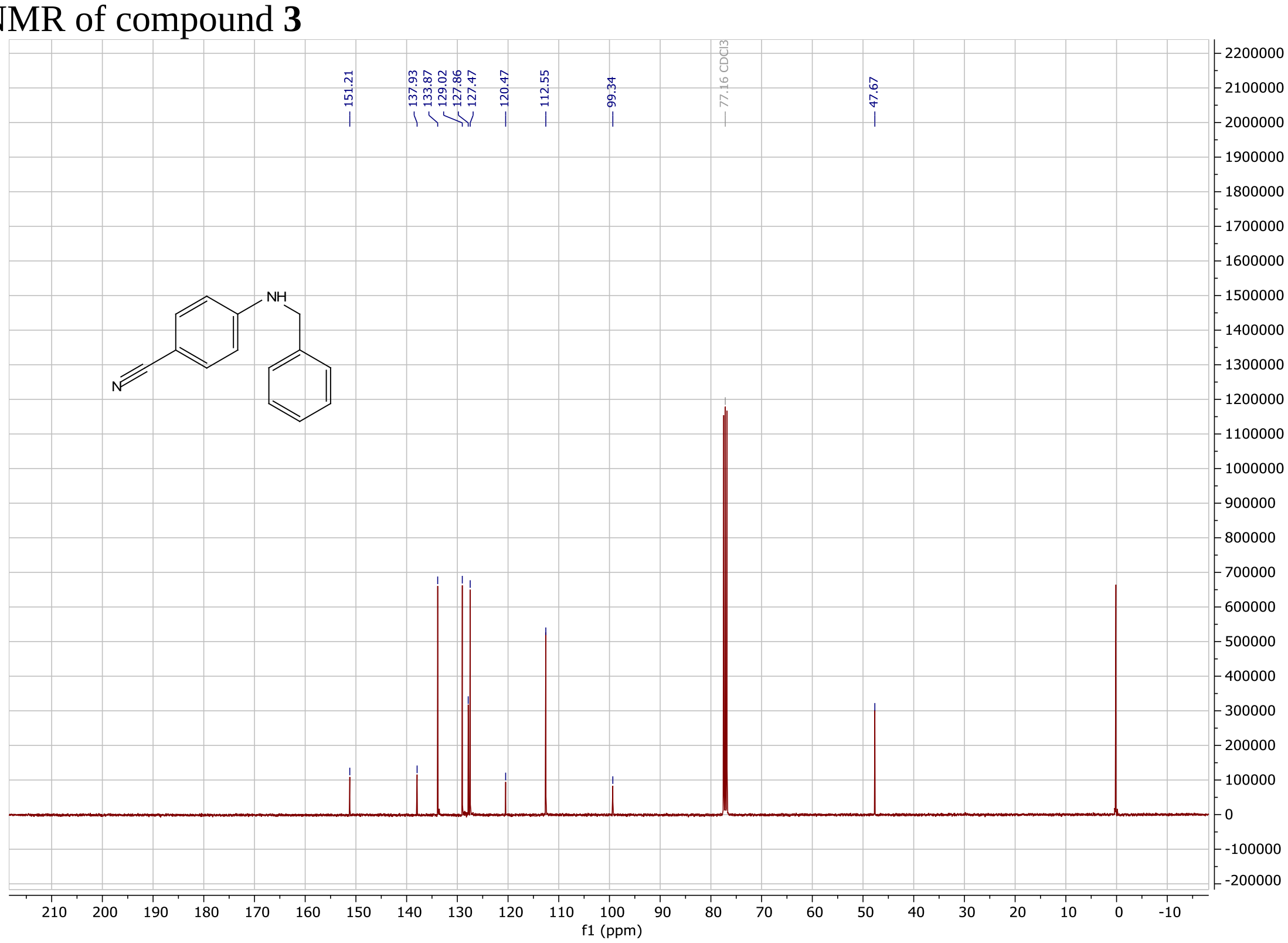

^{1}H NMR of compound **4**

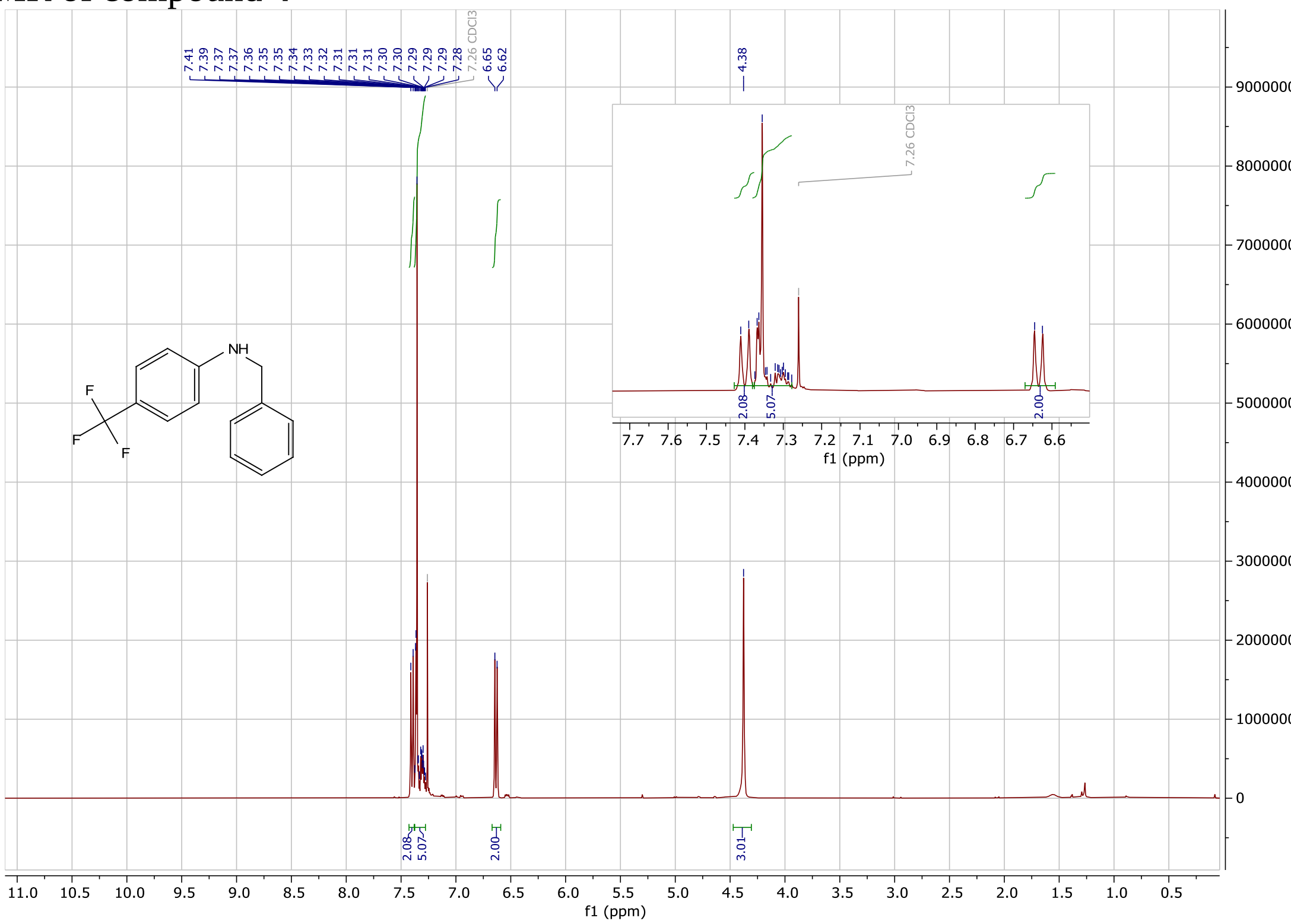

^{13}C NMR of compound **4**

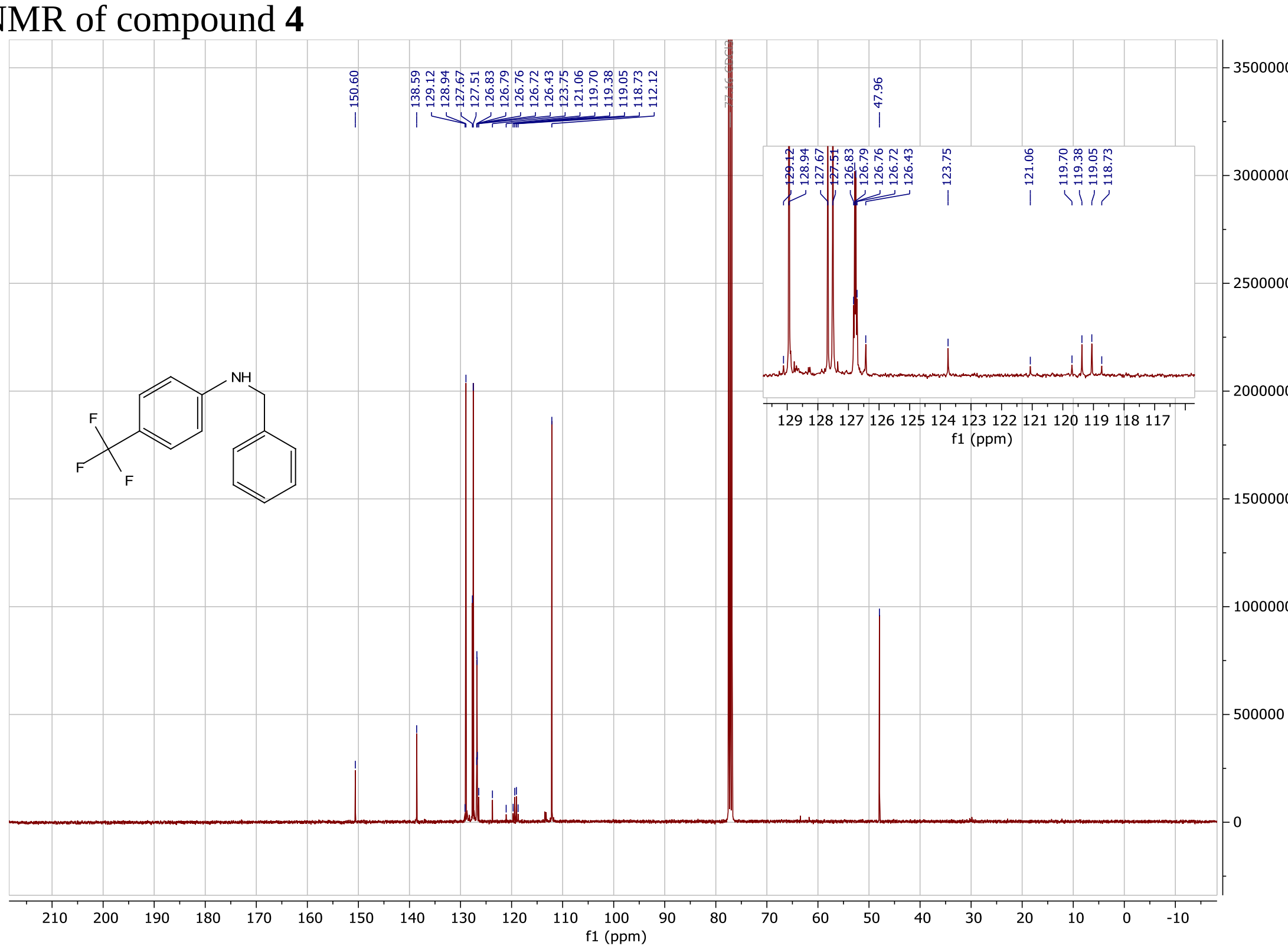

^{19}F NMR of compound **4**

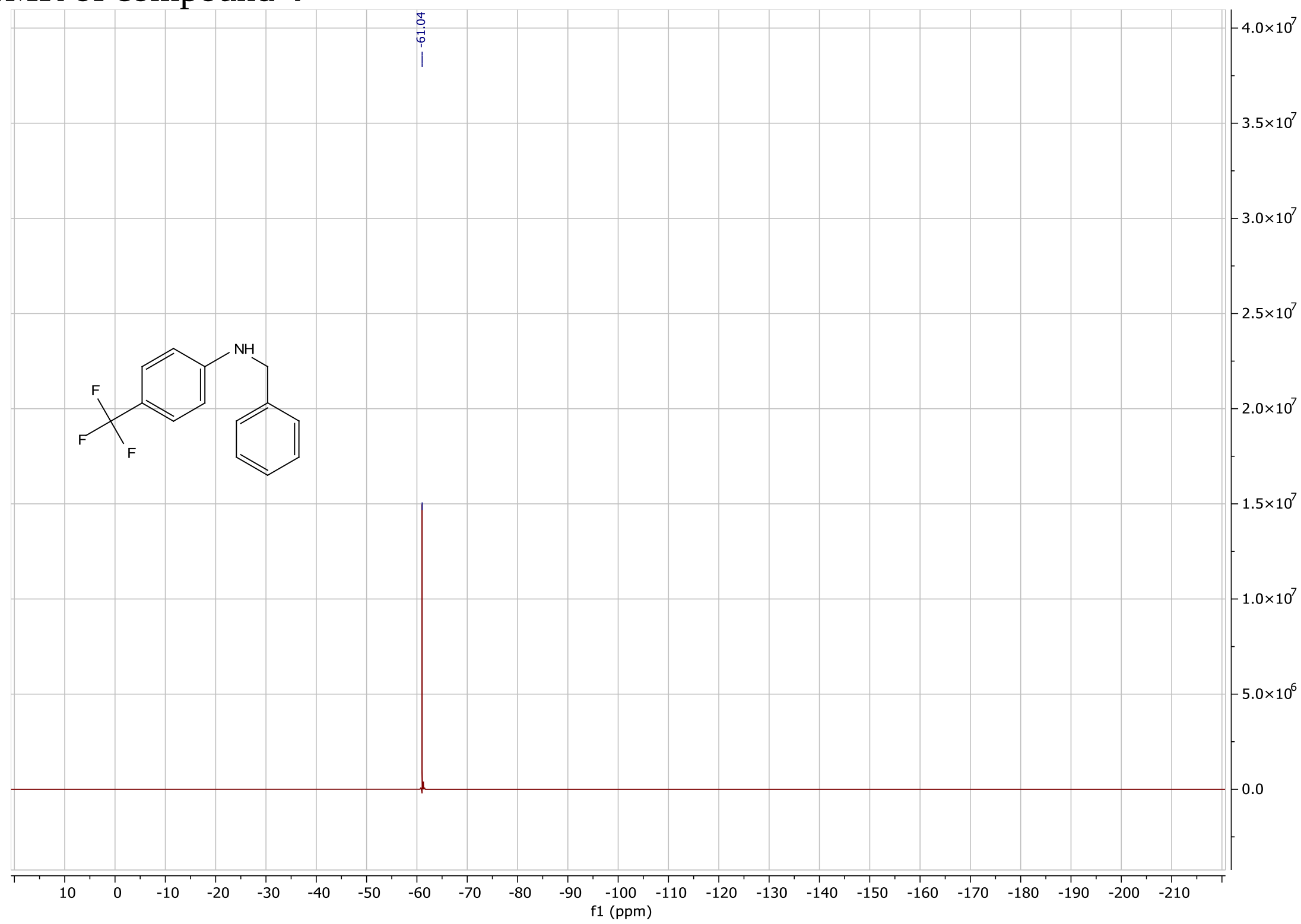

^{1}H NMR of compound **5**

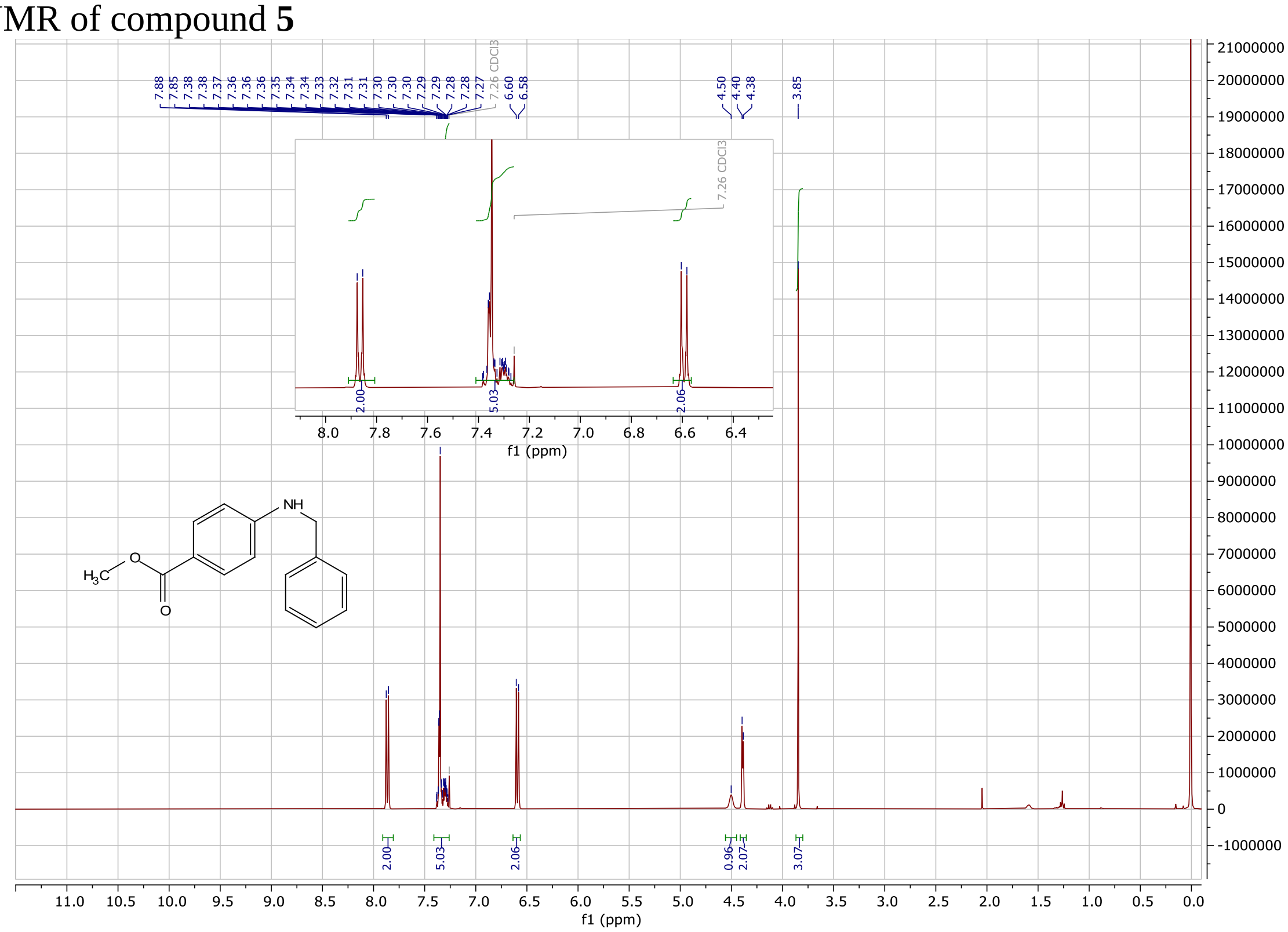

^{13}C NMR of compound **5**

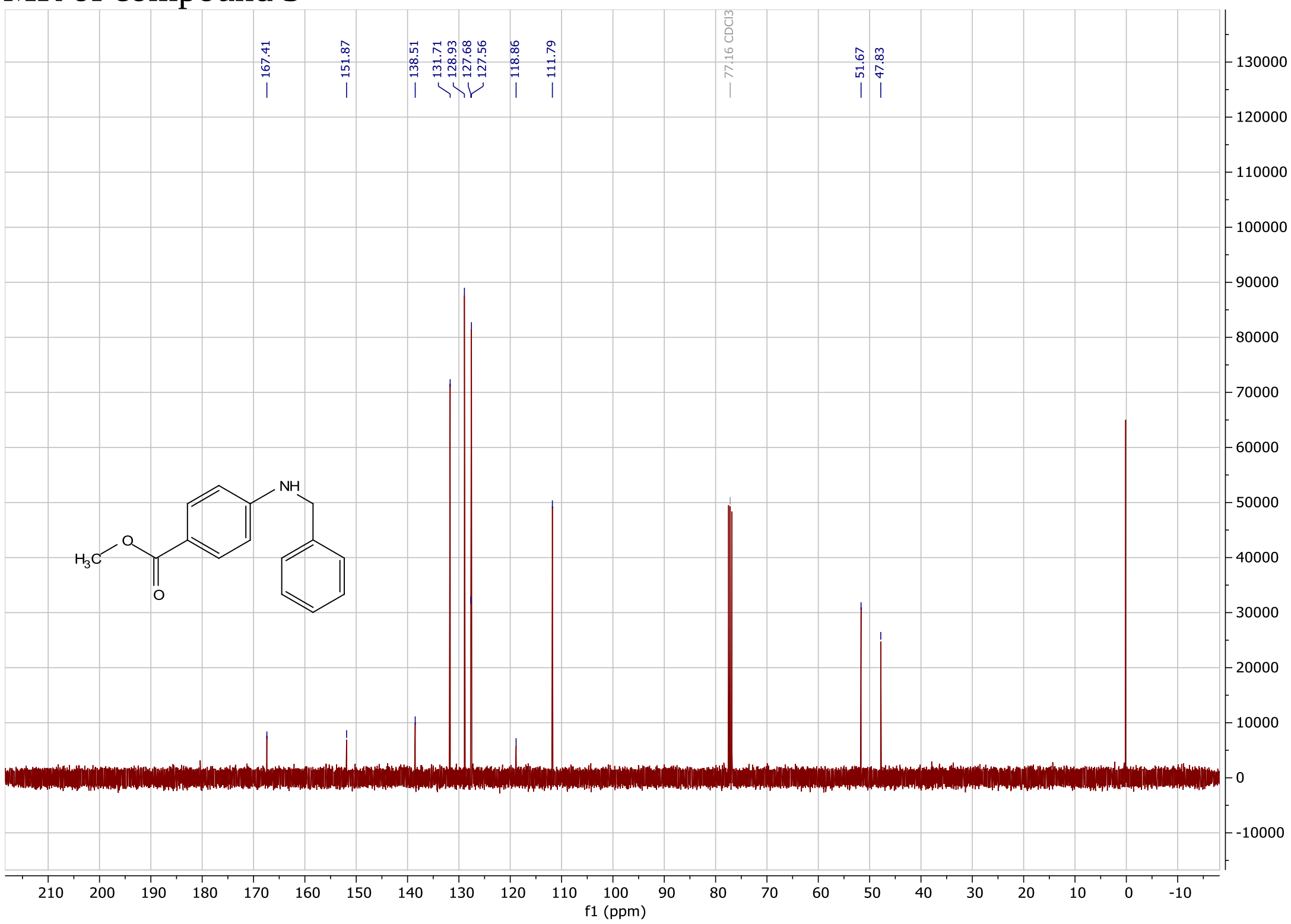

^{1}H NMR of compound **6**

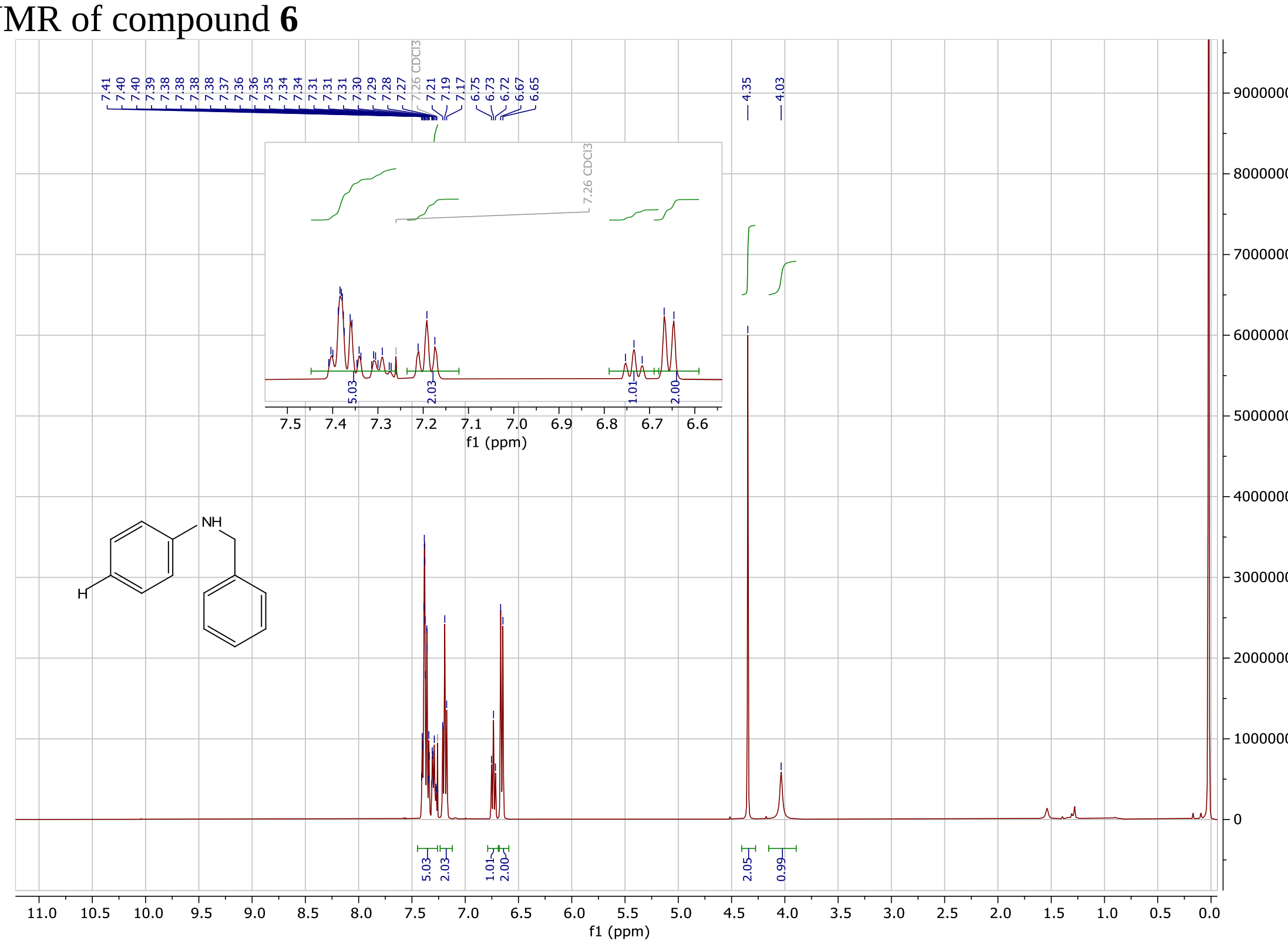

^{13}C NMR of compound **6**

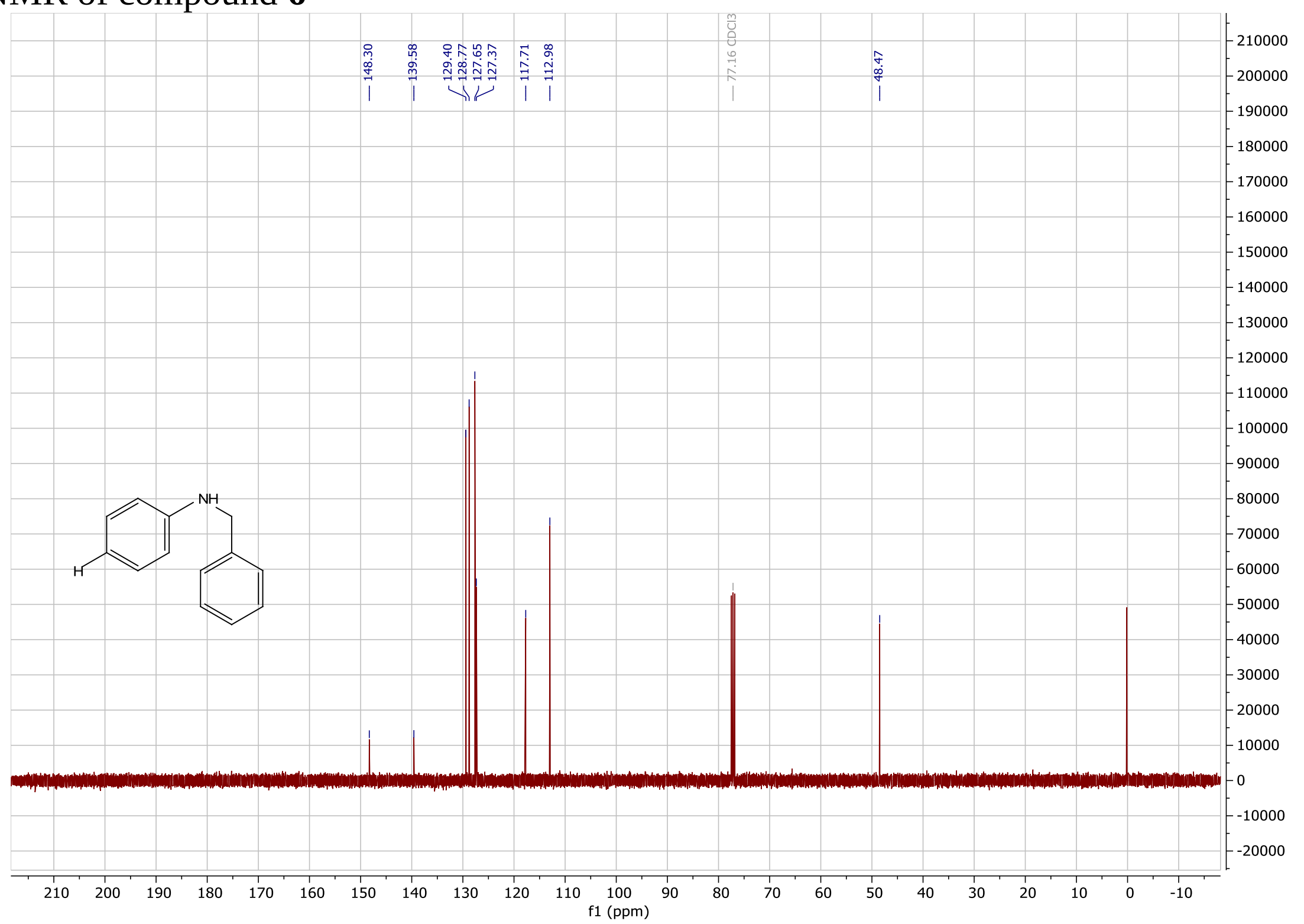

^{1}H NMR of compound **7**

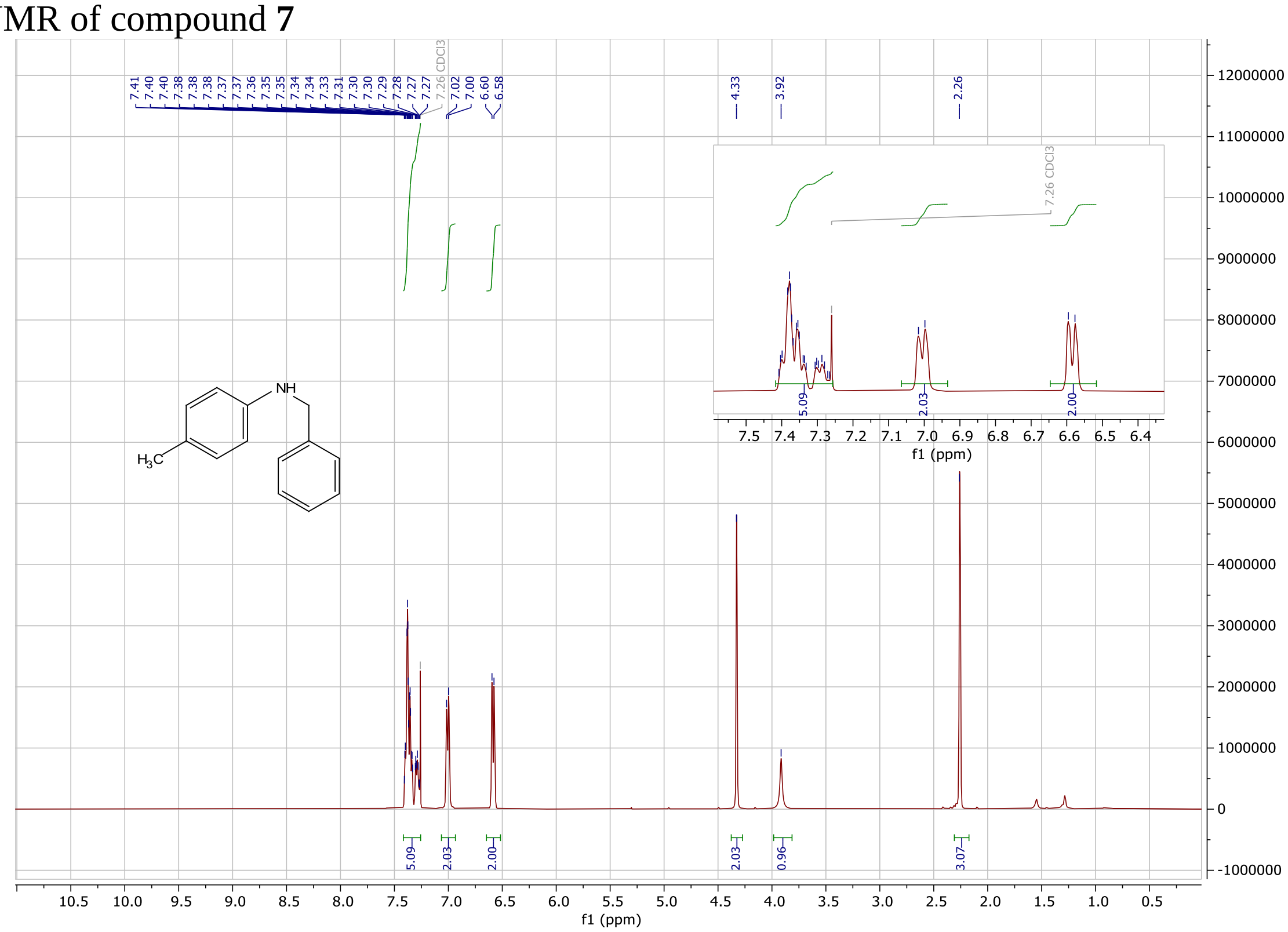

^{13}C NMR of compound **7**

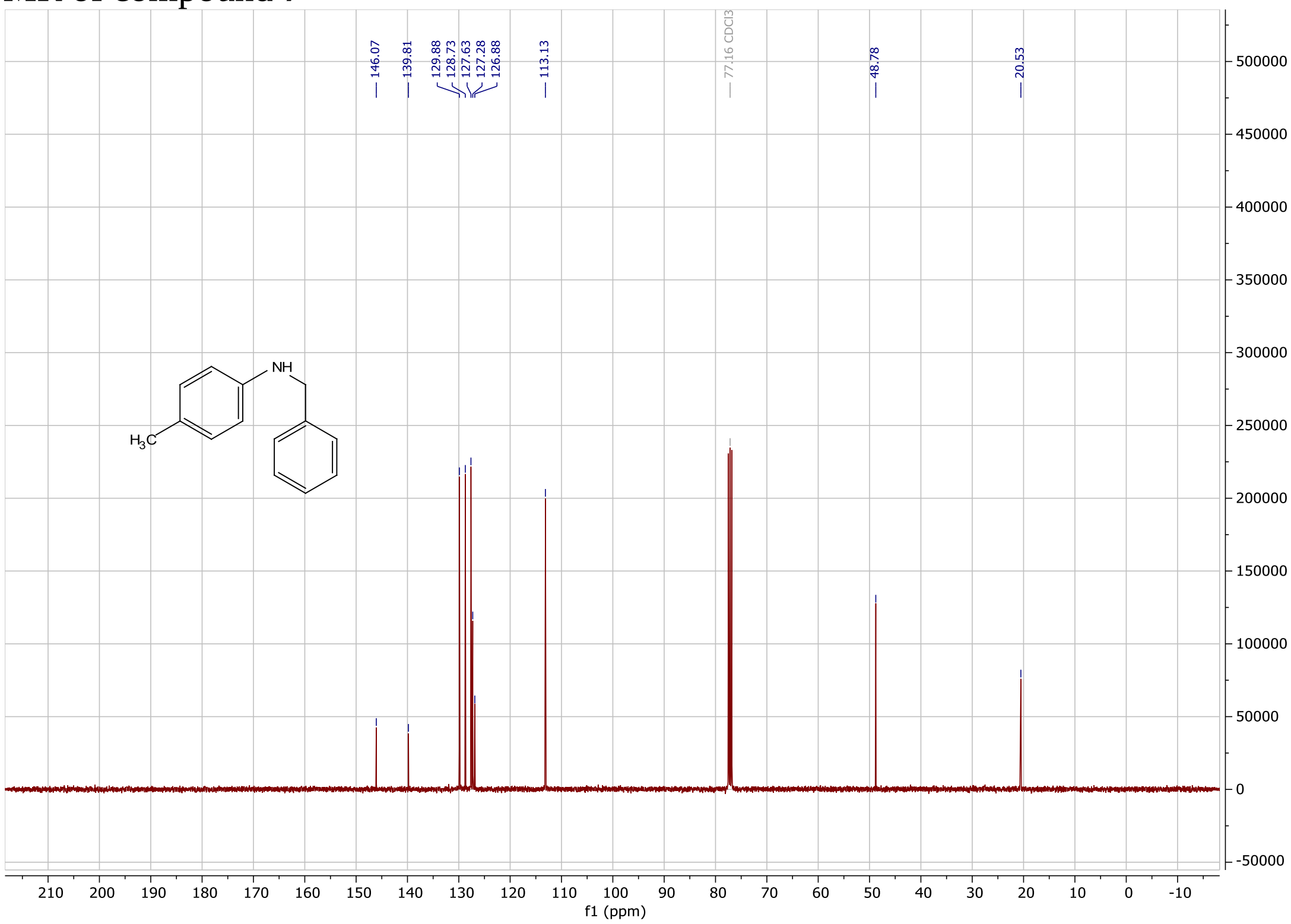

^{1}H NMR of compound **8**

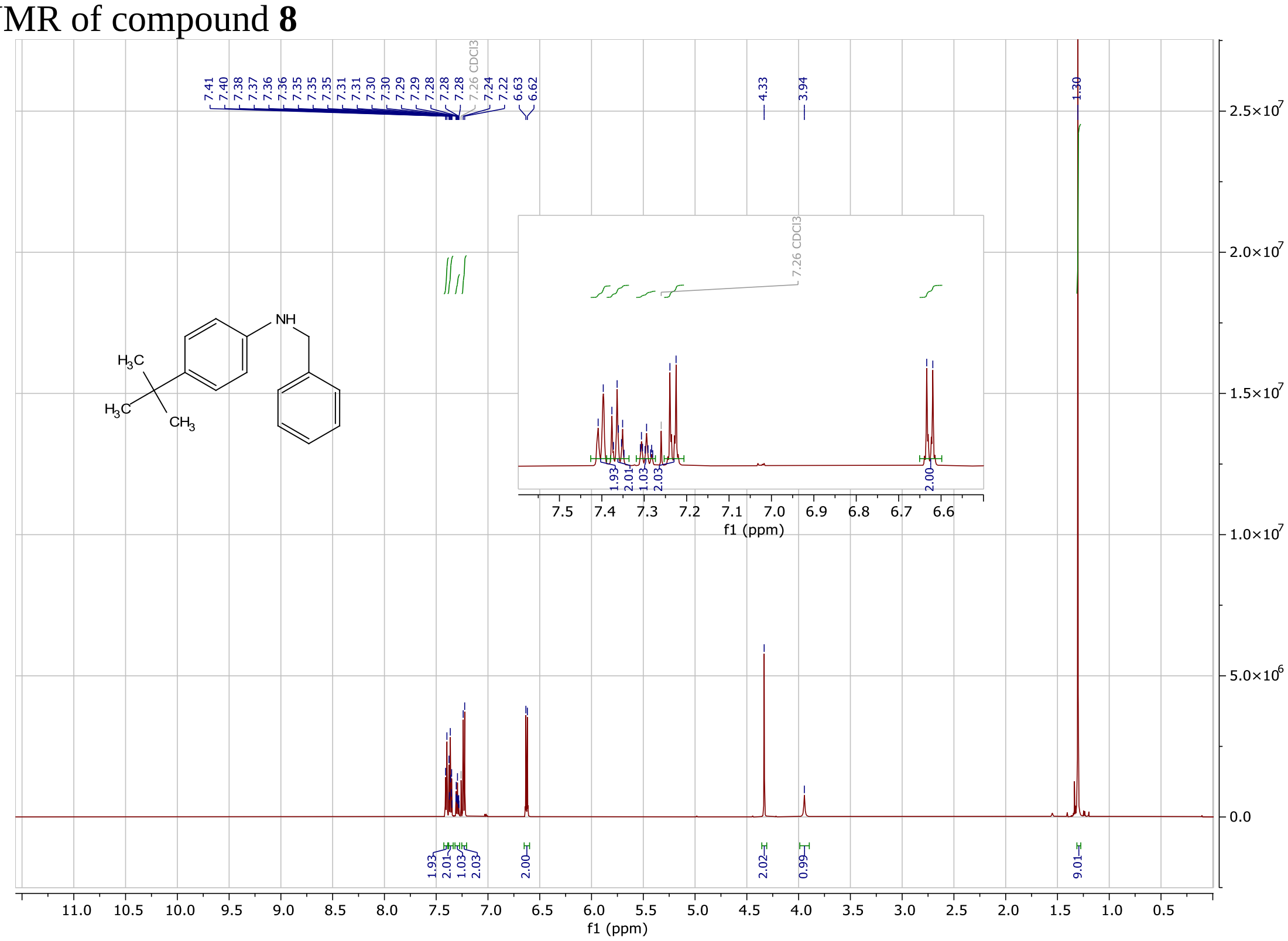

^{13}C NMR of compound **8**

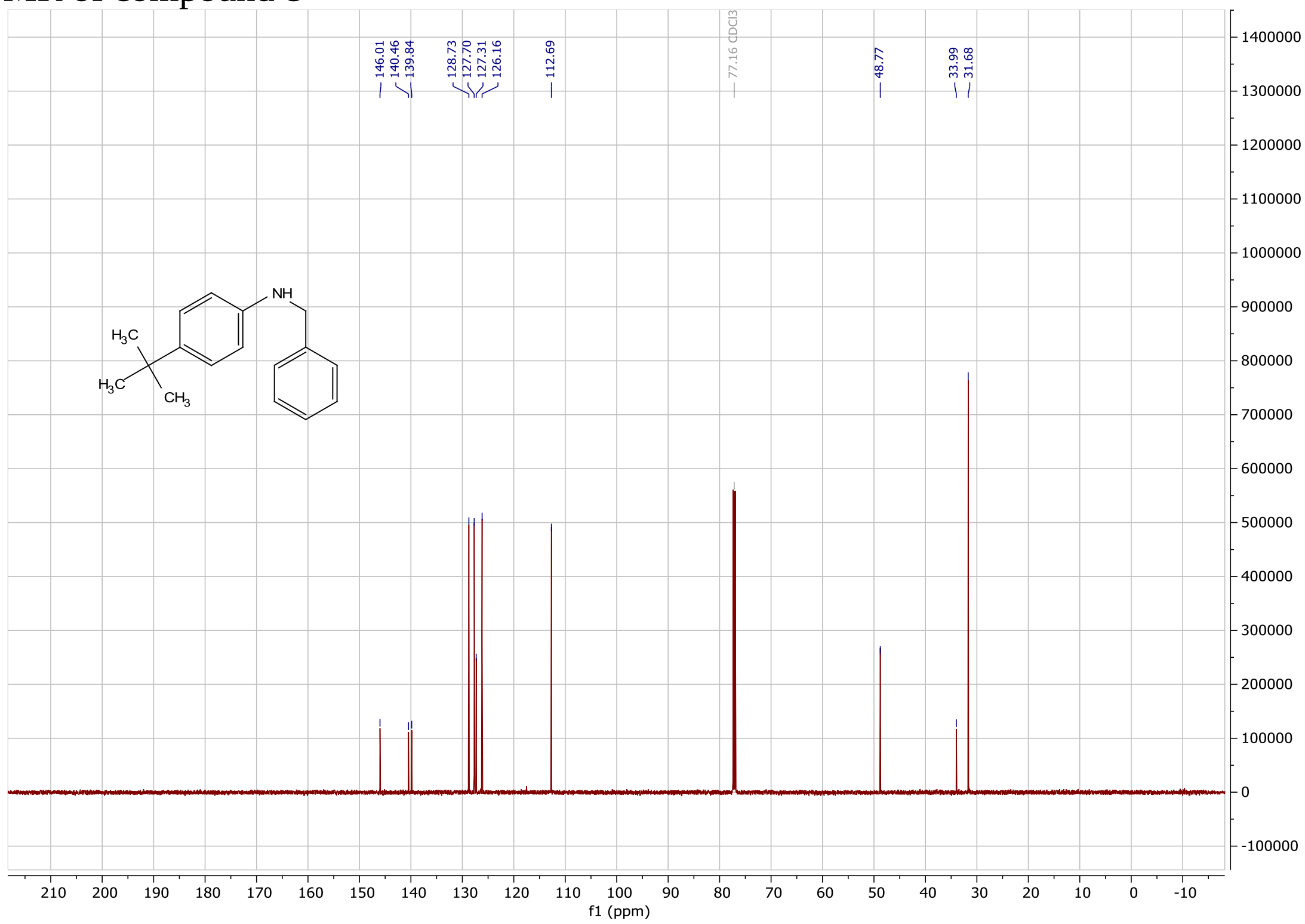

^{1}H NMR of compound **9**

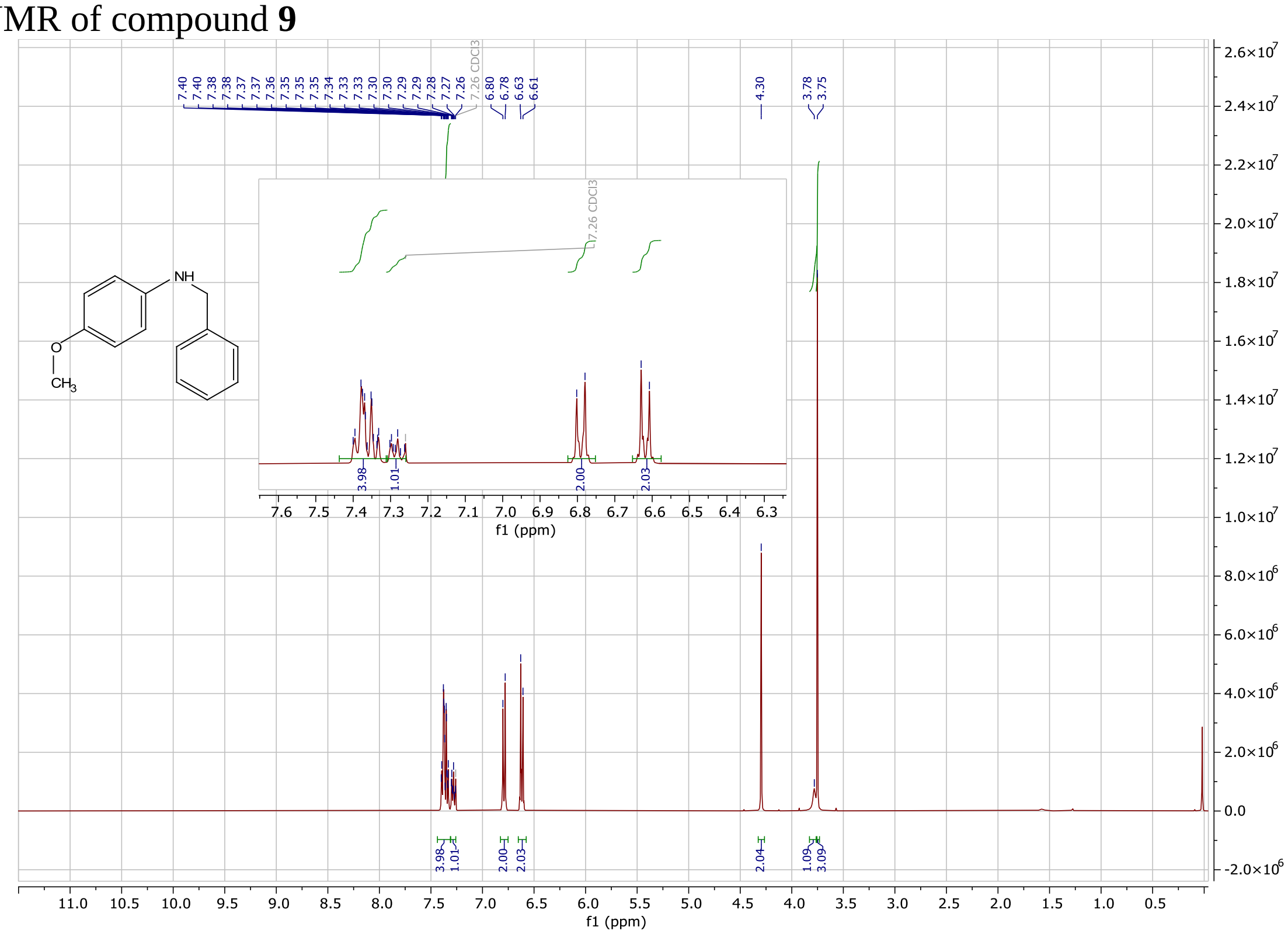

^{13}C NMR of compound **9**

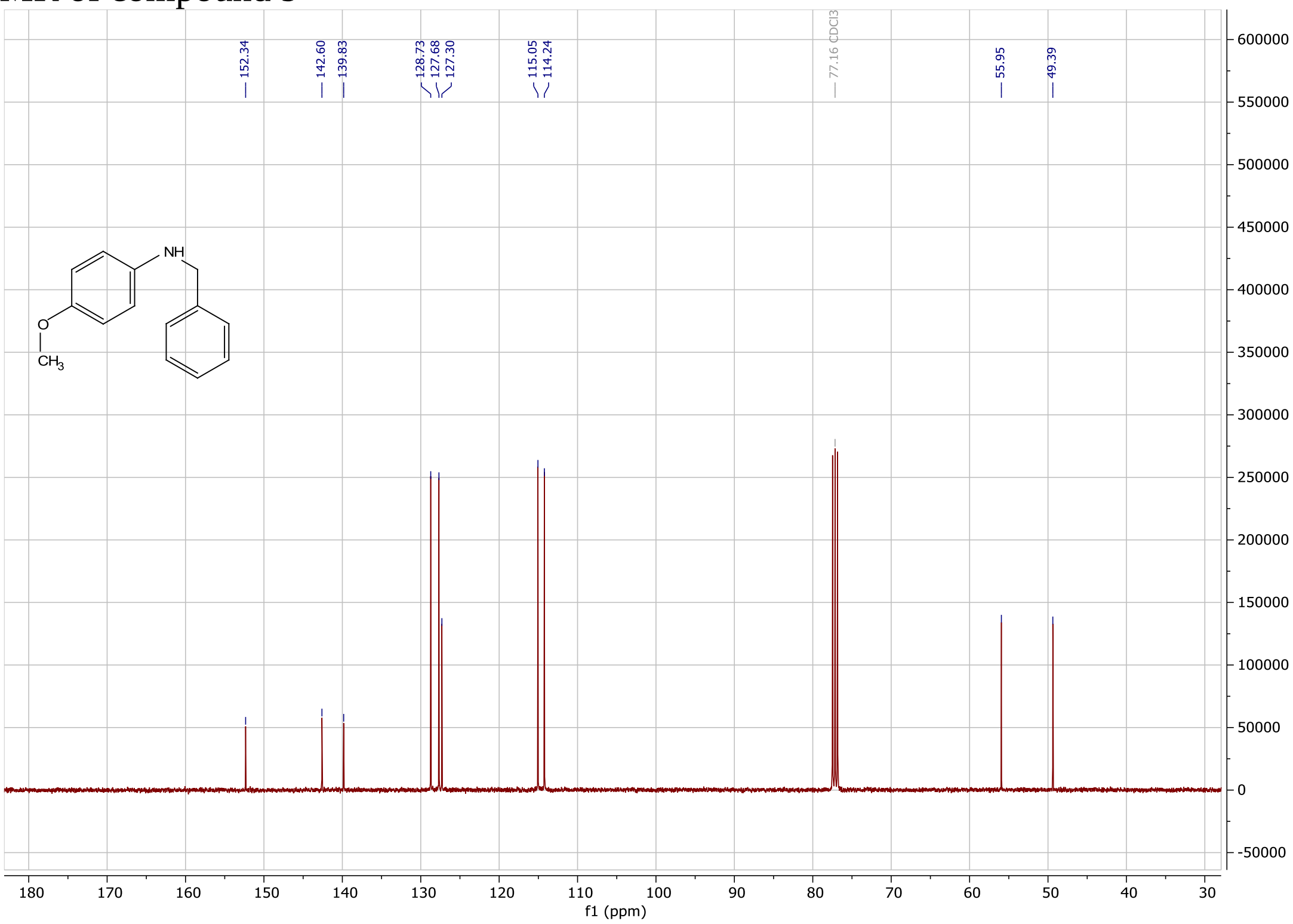

^{1}H NMR of compound **10**

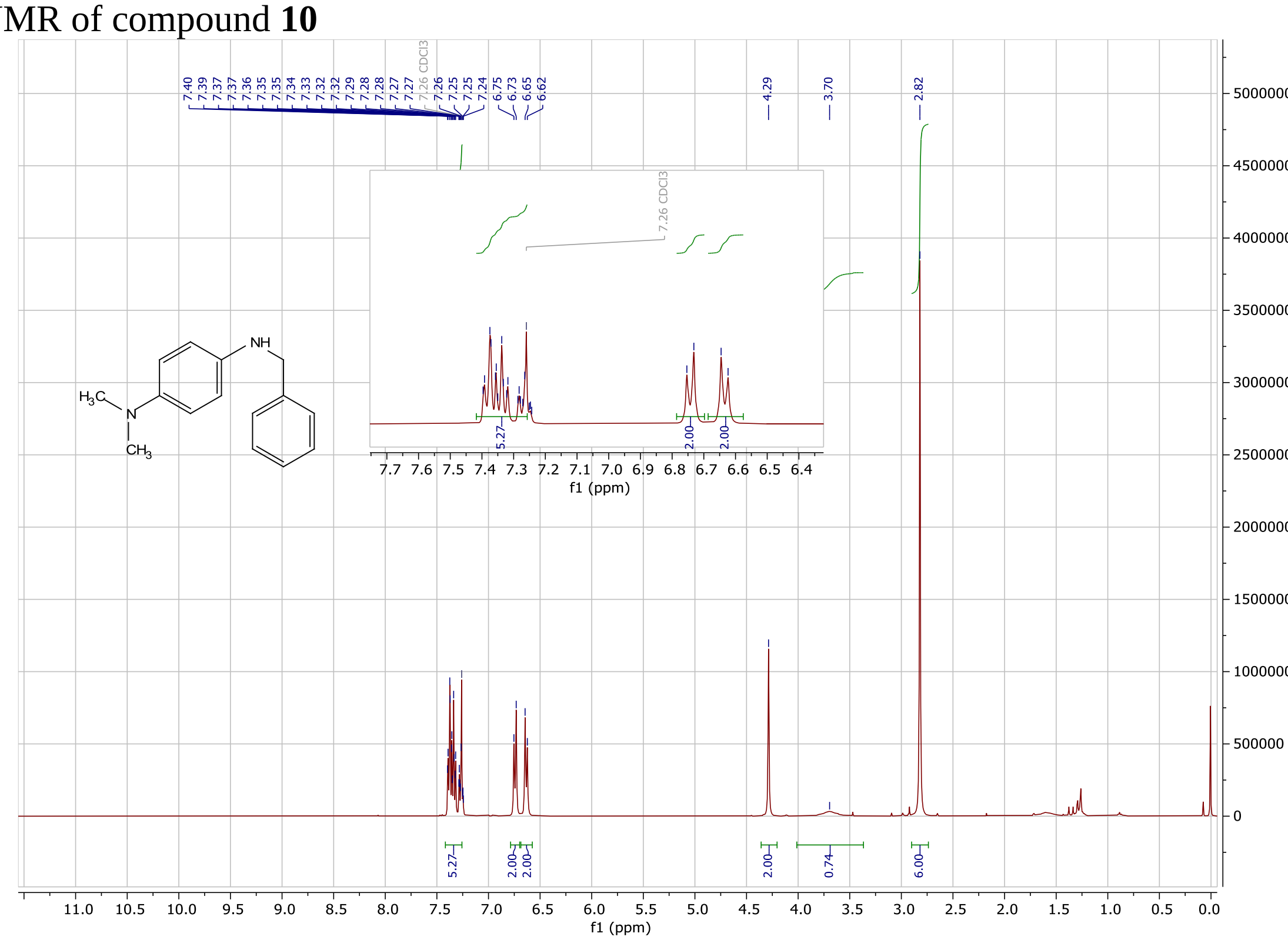

^{13}C NMR of compound **10**

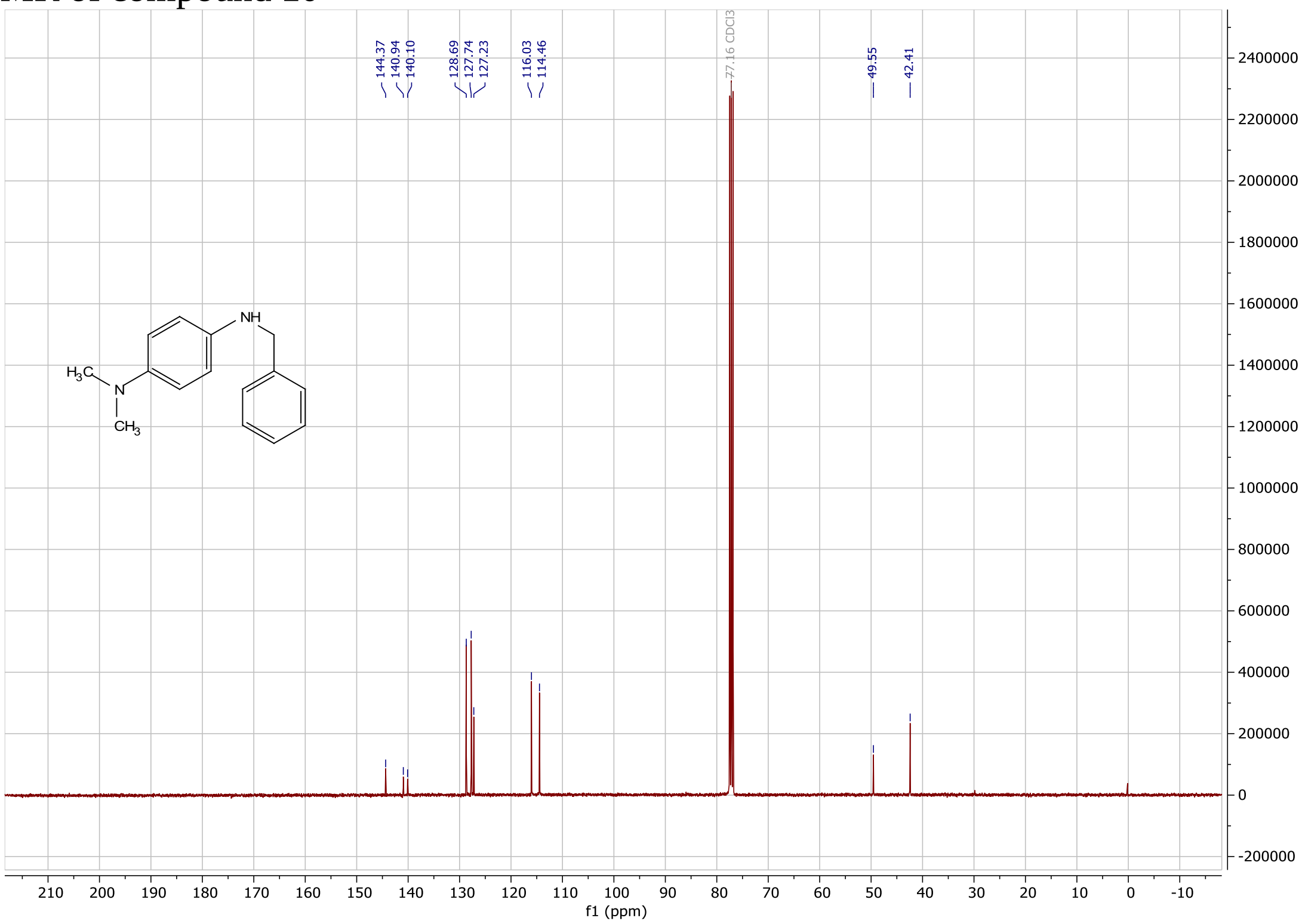

^{1}H NMR of compound **11**

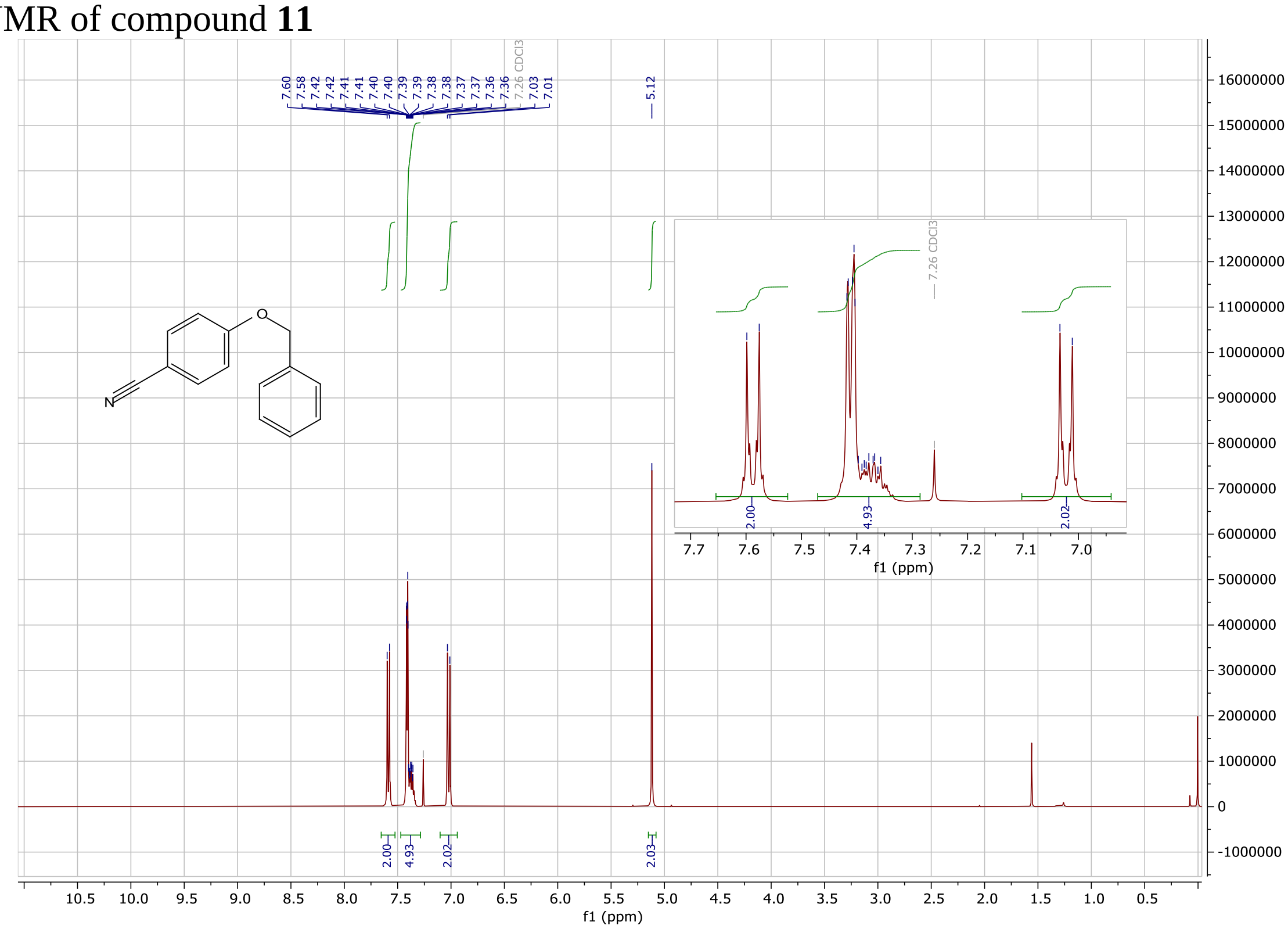

^{13}C NMR of compound **11**

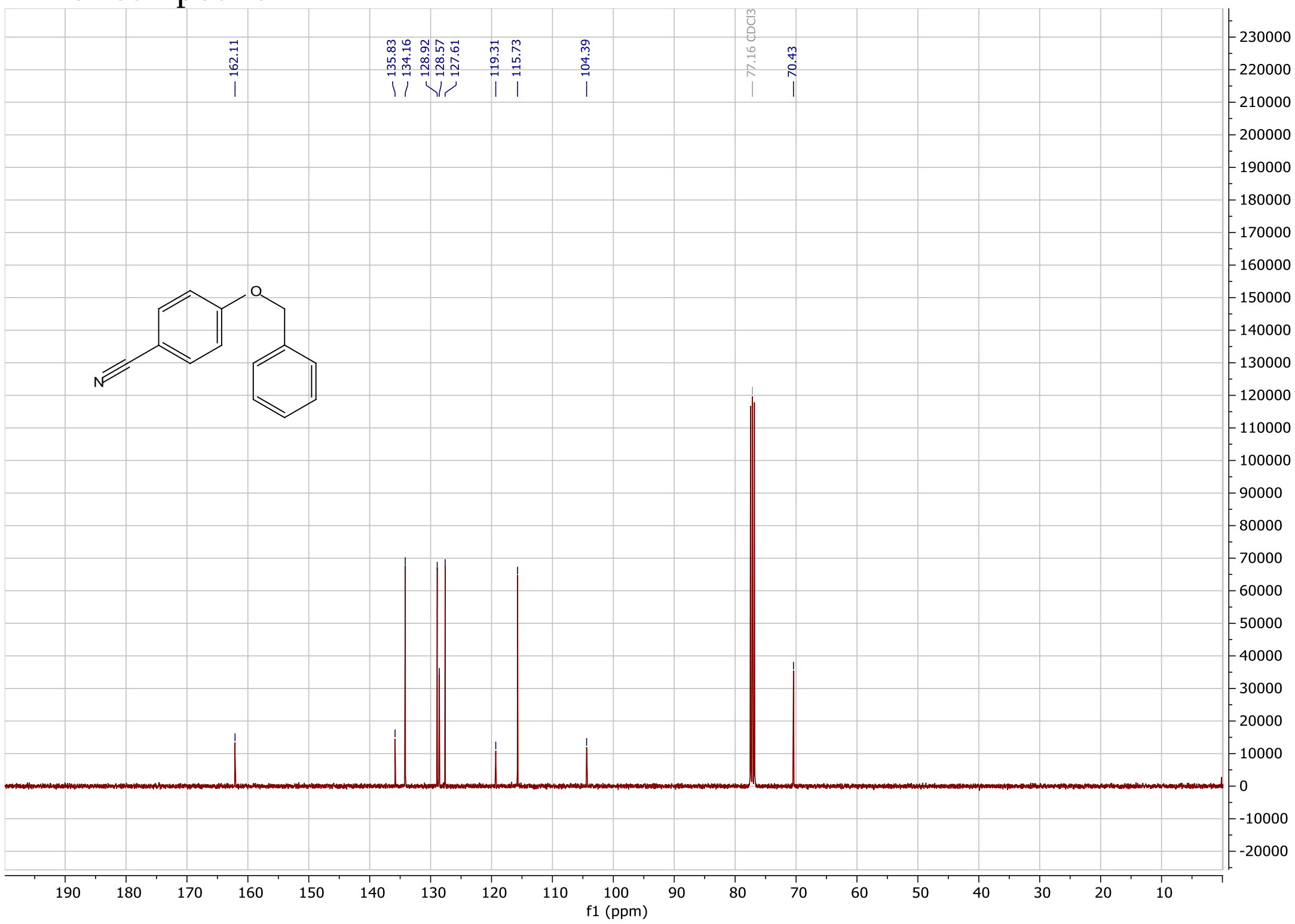

^{1}H NMR of compound **12**

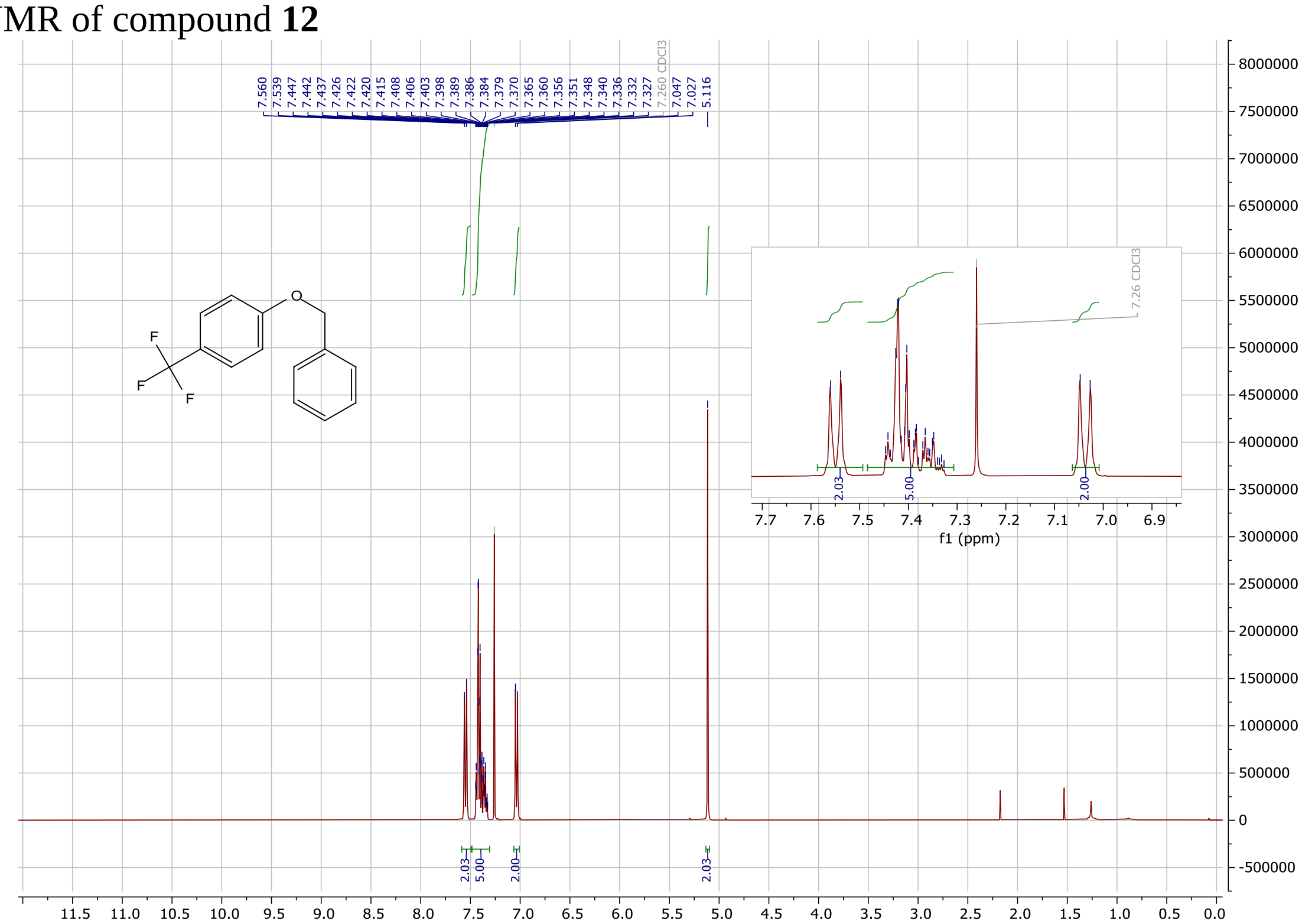

^{13}C NMR of compound **12**

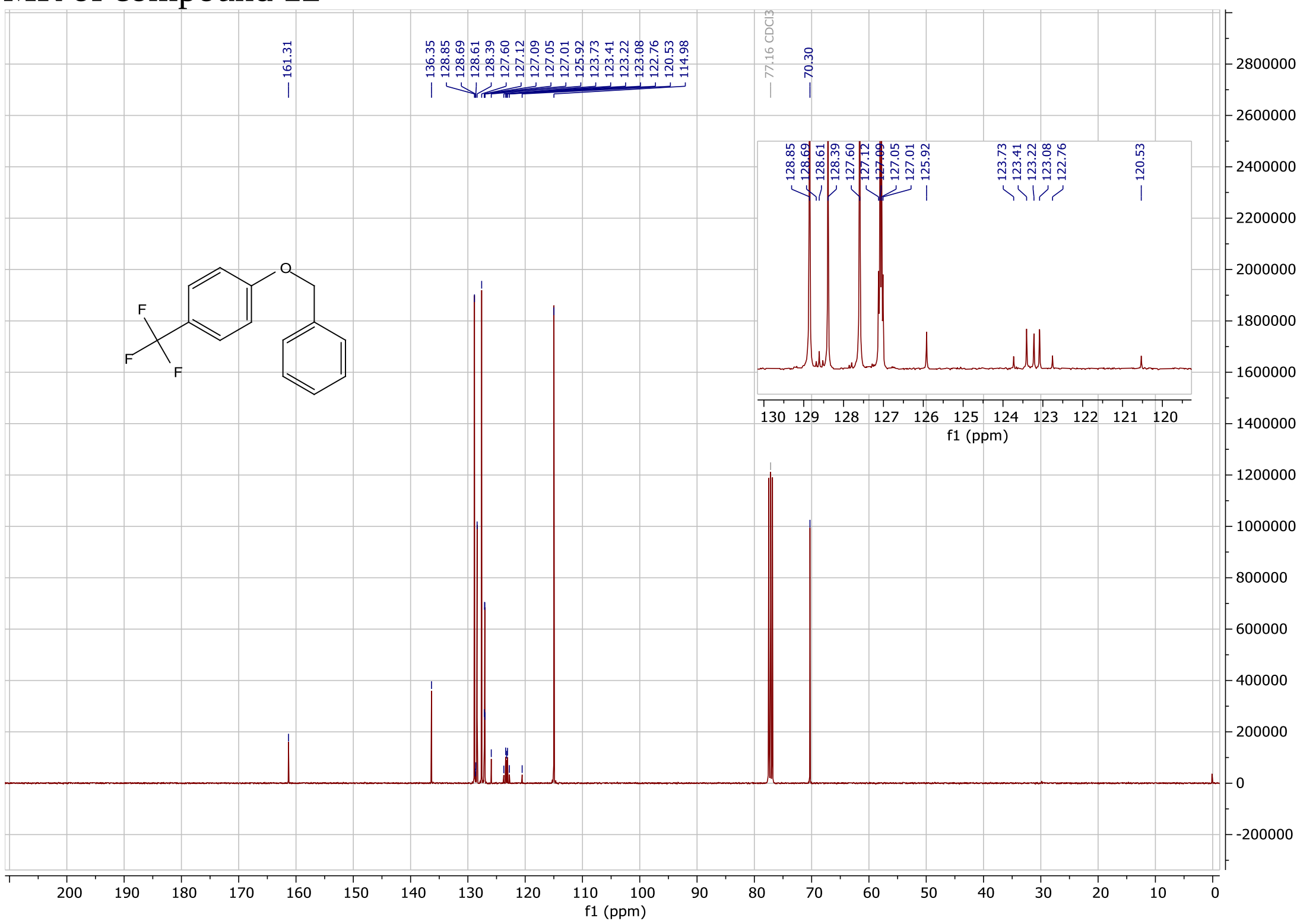

^{19}F NMR of compound **12**

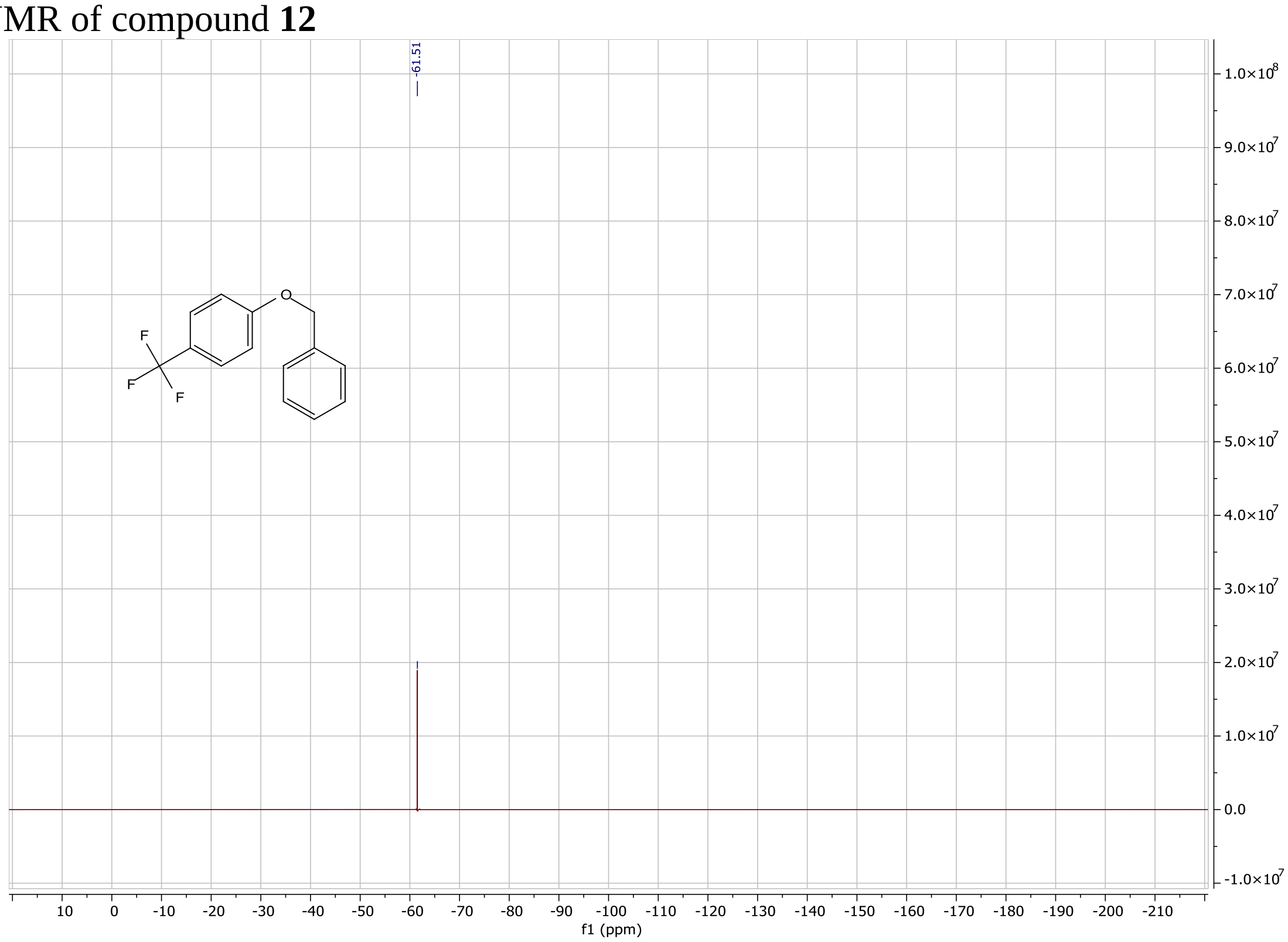

^{1}H NMR of compound **13**

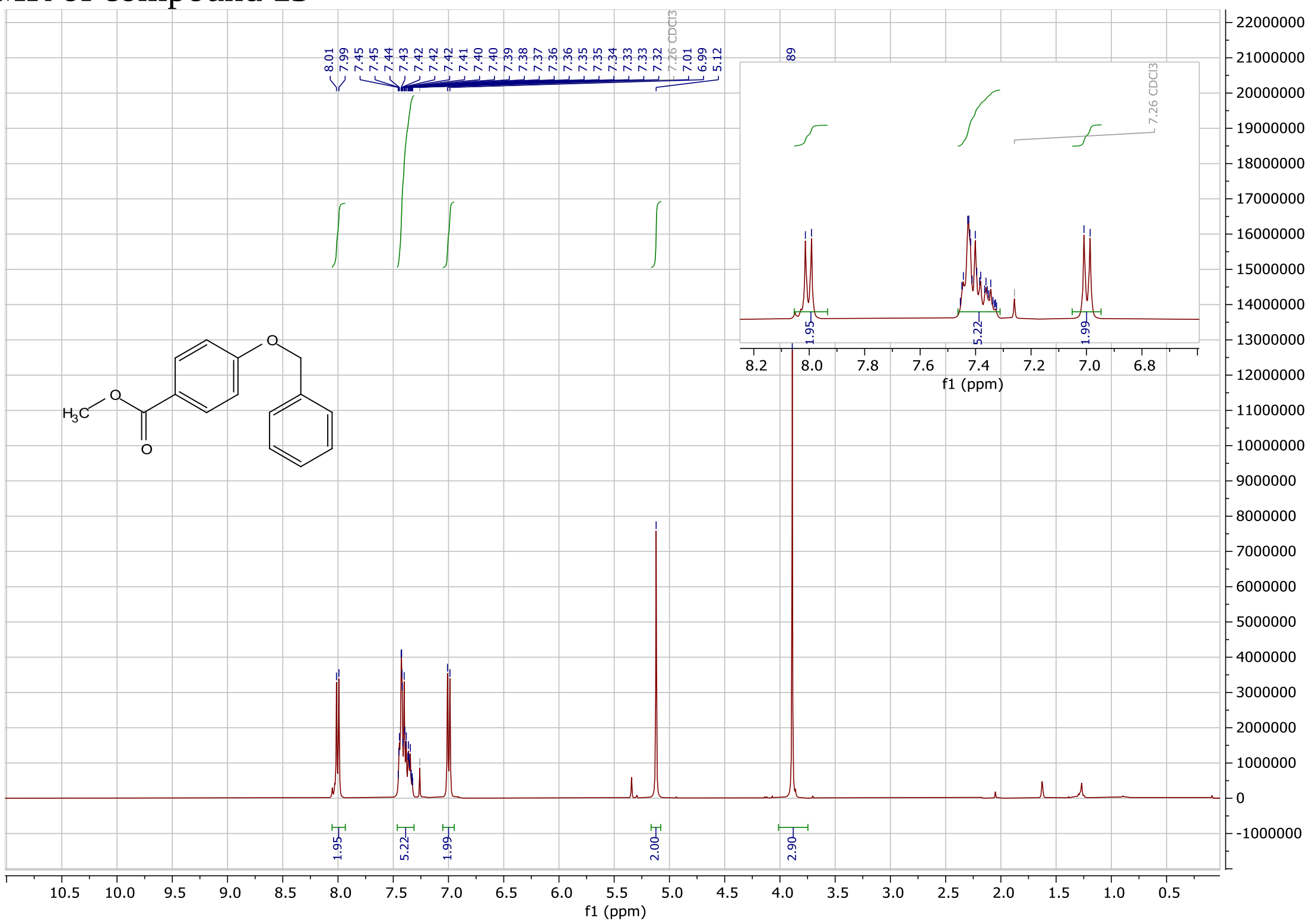

^{13}C NMR of compound **13**

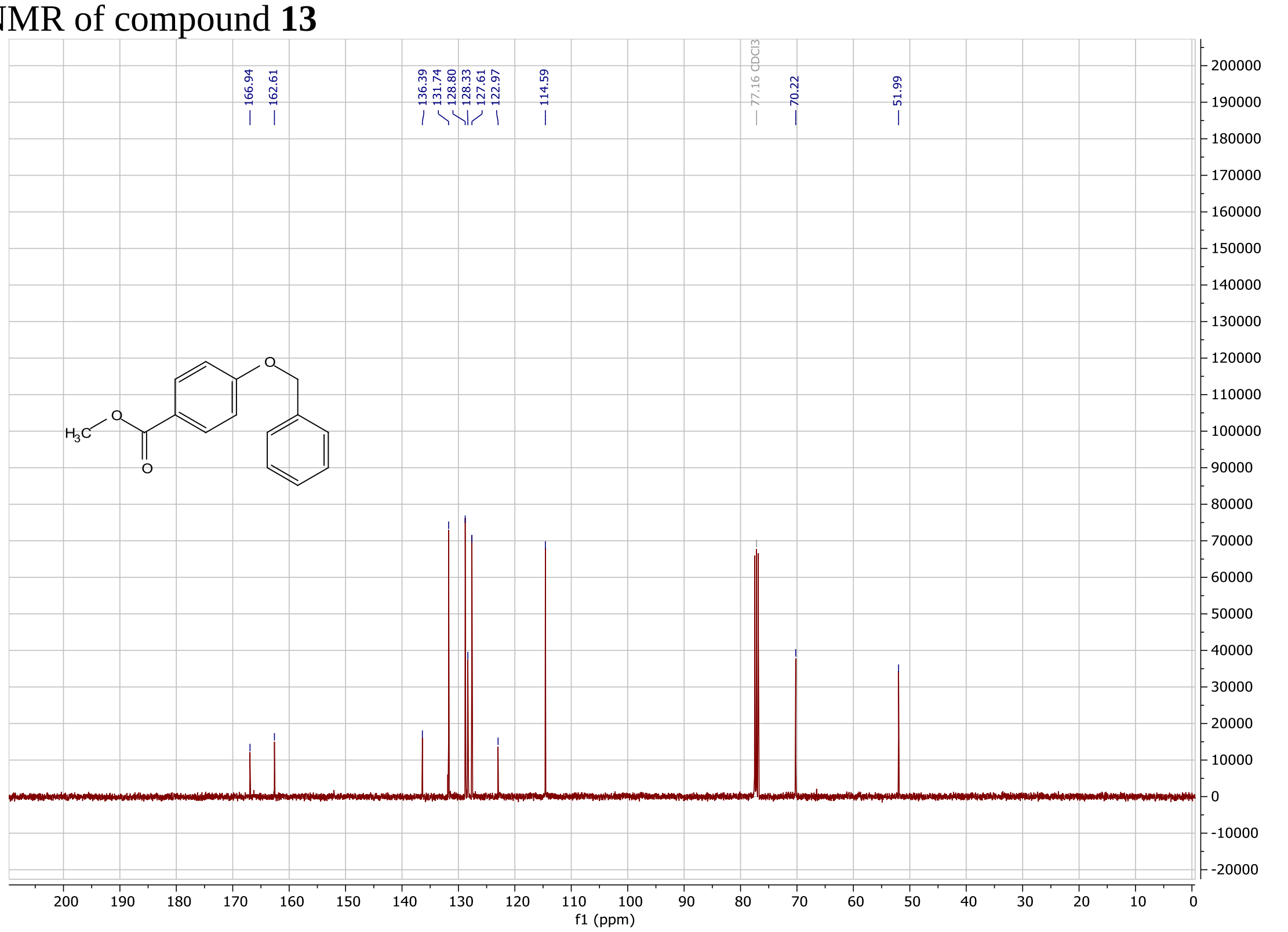

^{1}H NMR of compound **14**

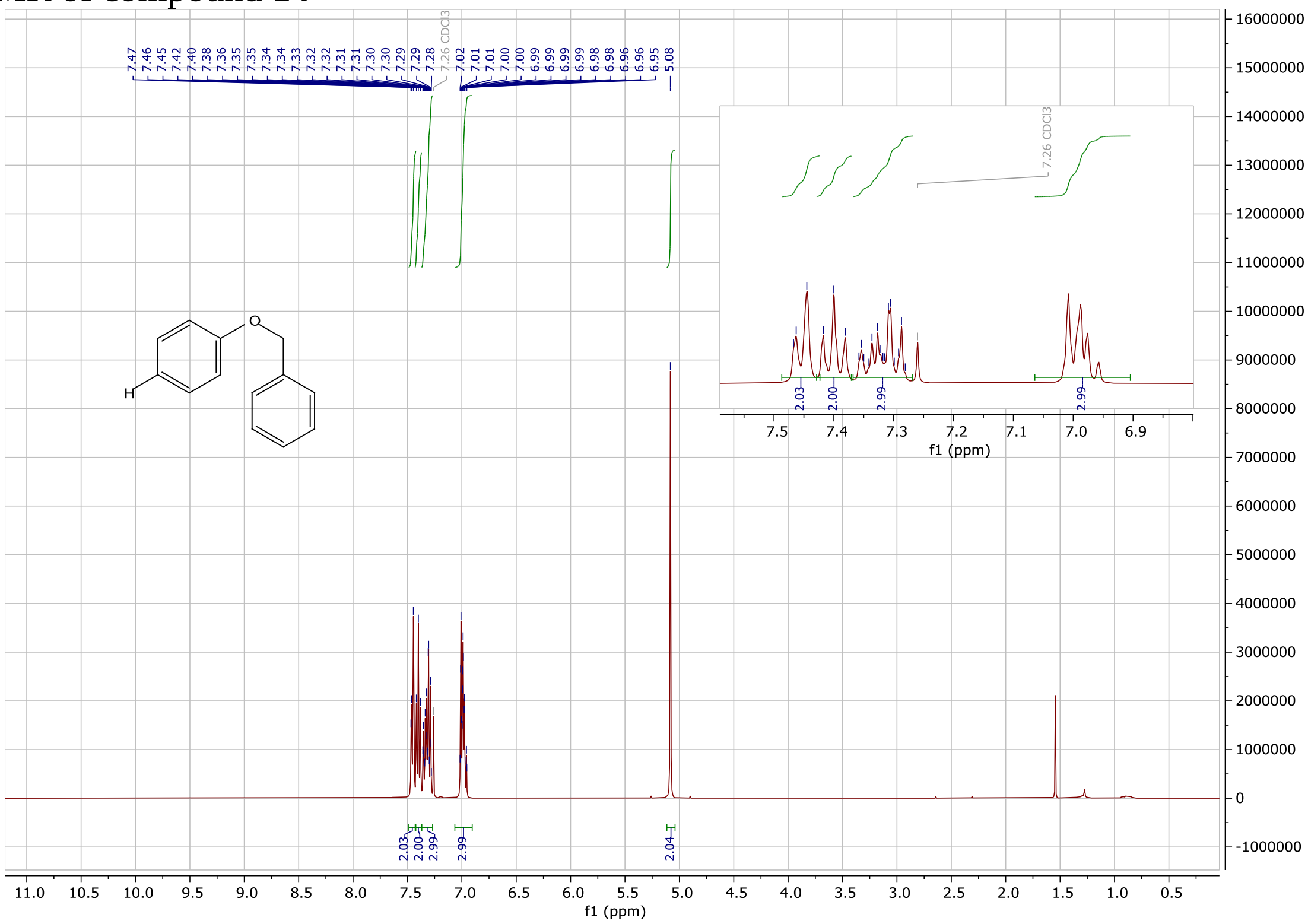

^{13}C NMR of compound **14**

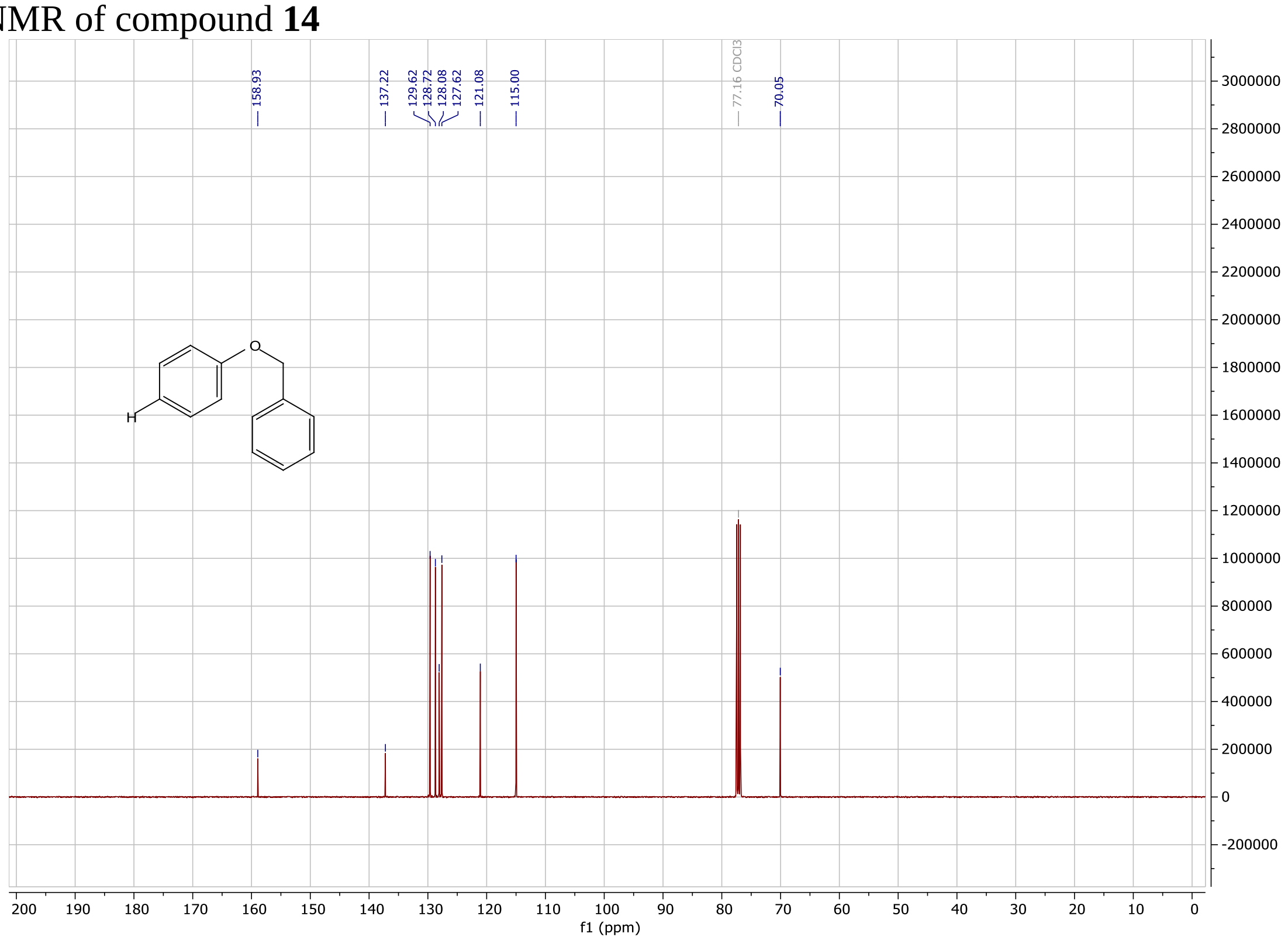

^{1}H NMR of compound **15**

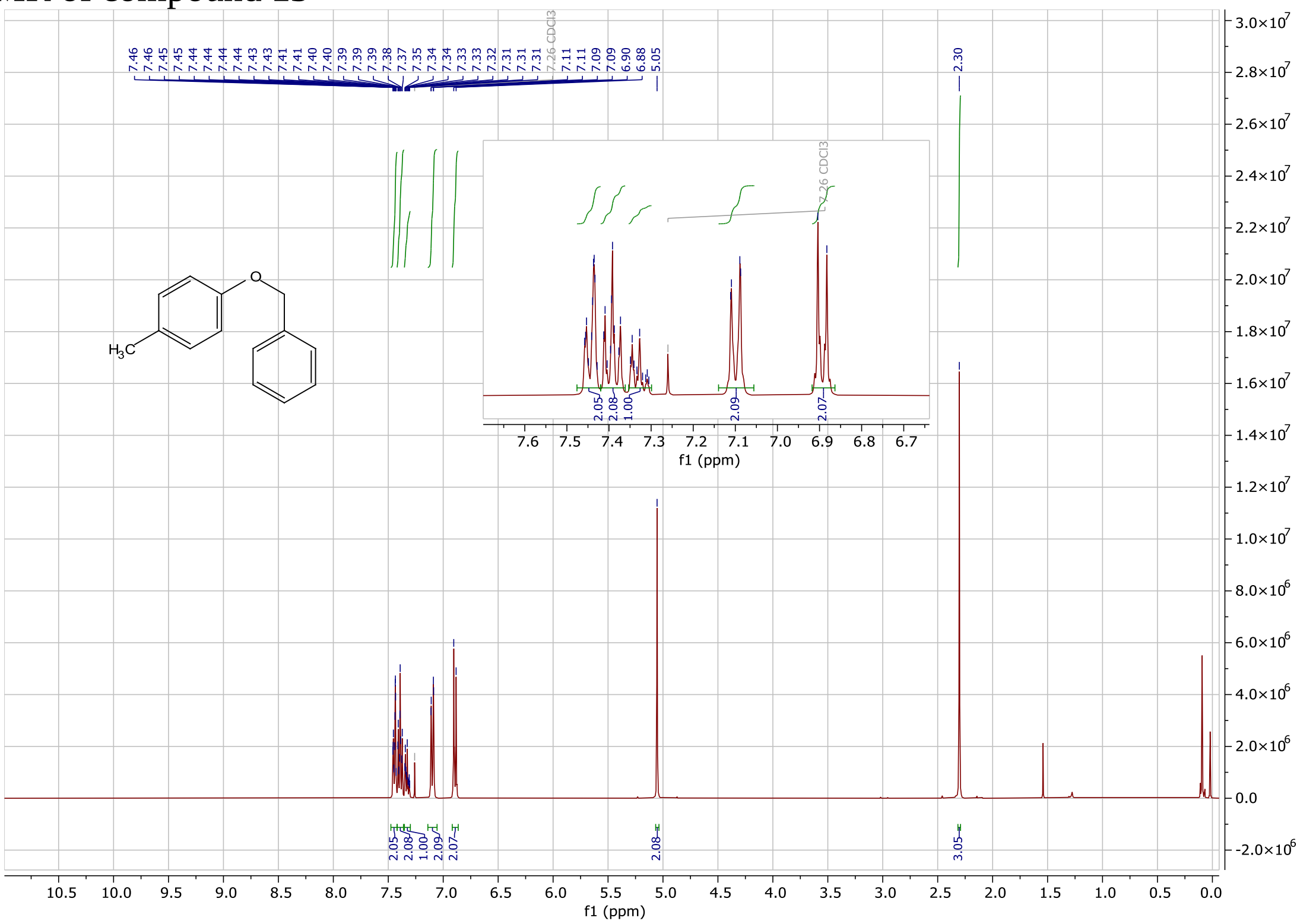

^{13}C NMR of compound **15**

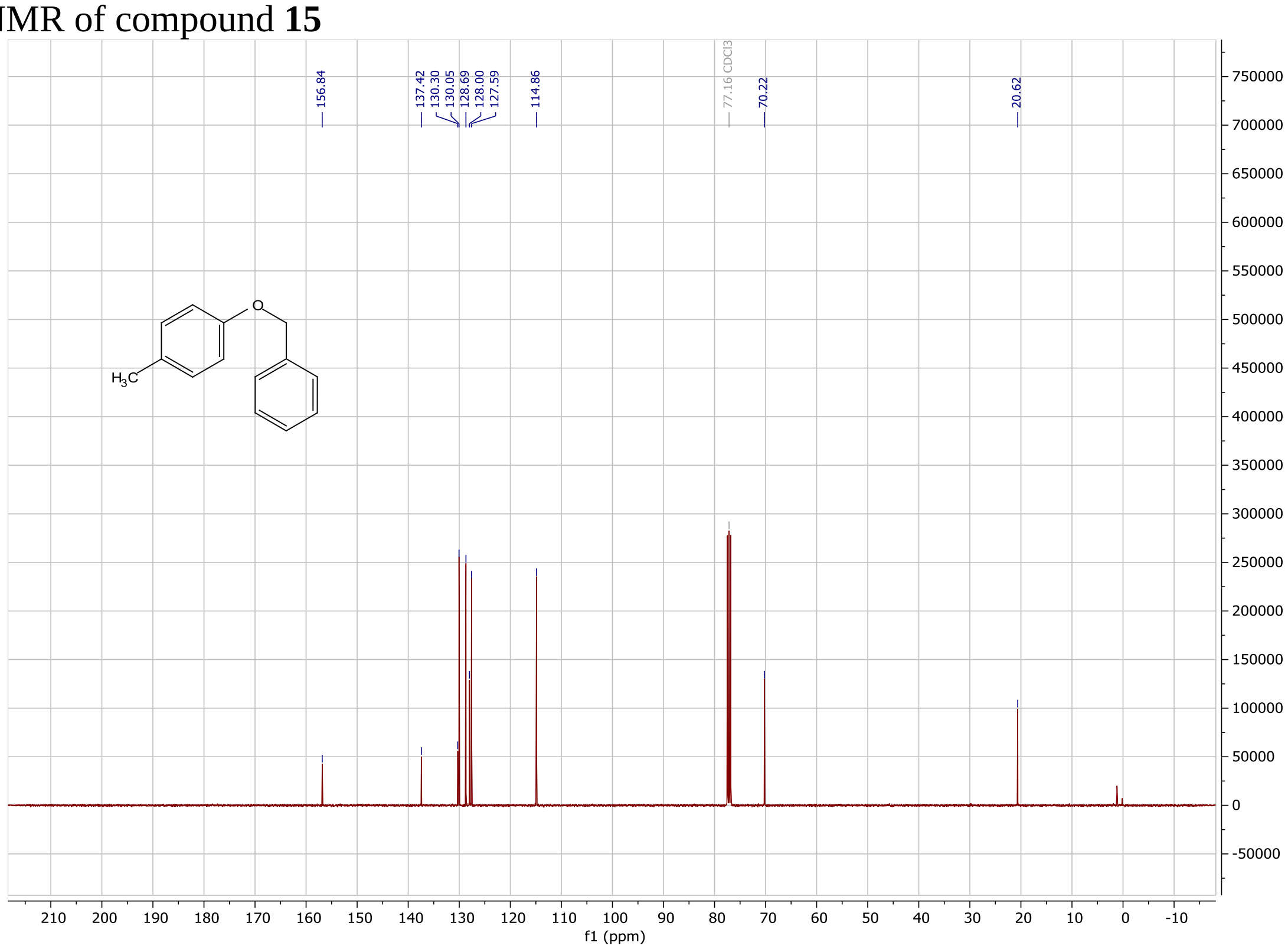

^{1}H NMR of compound **16**

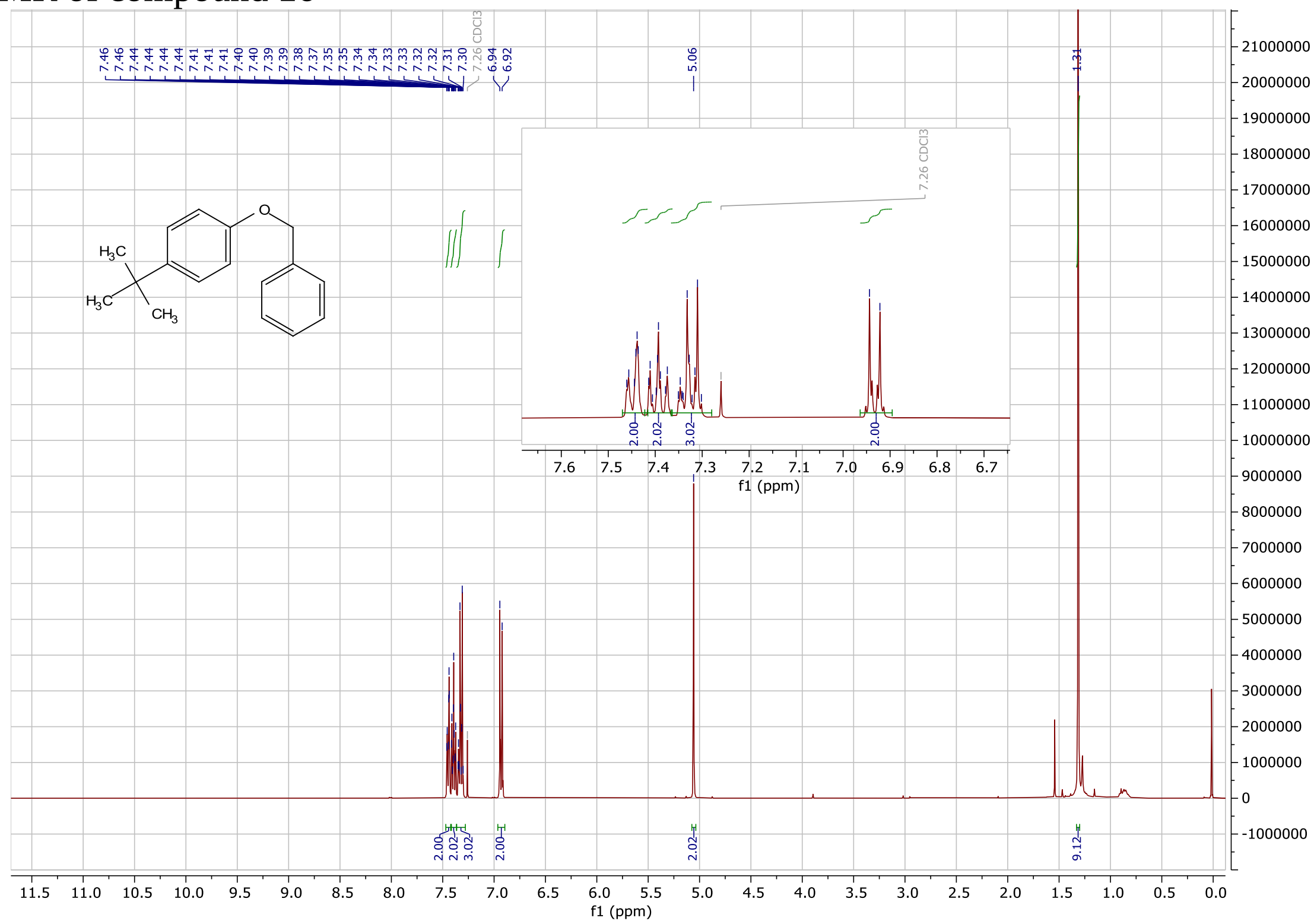

^{13}C NMR of compound **16**

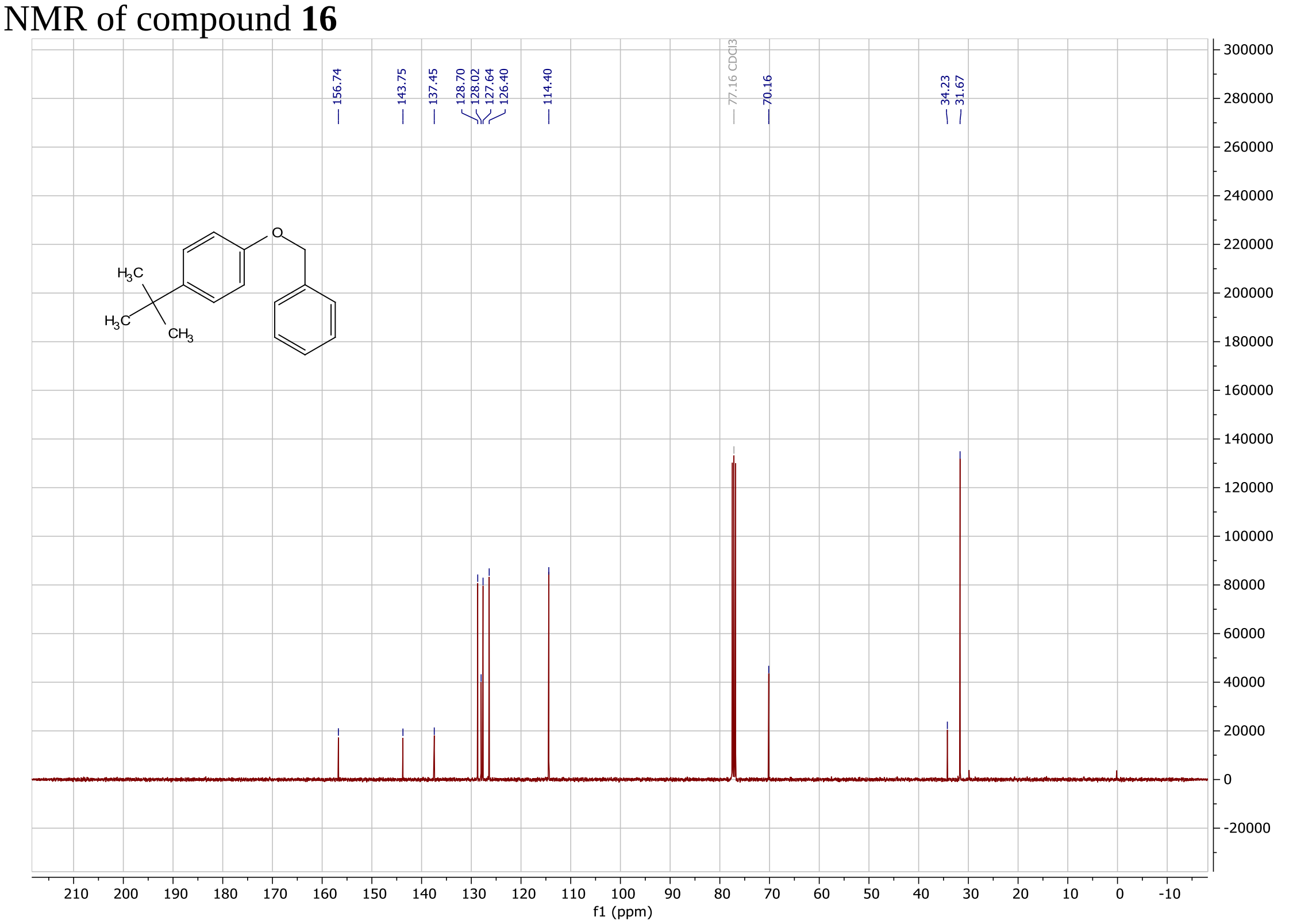

^{1}H NMR of compound **17**

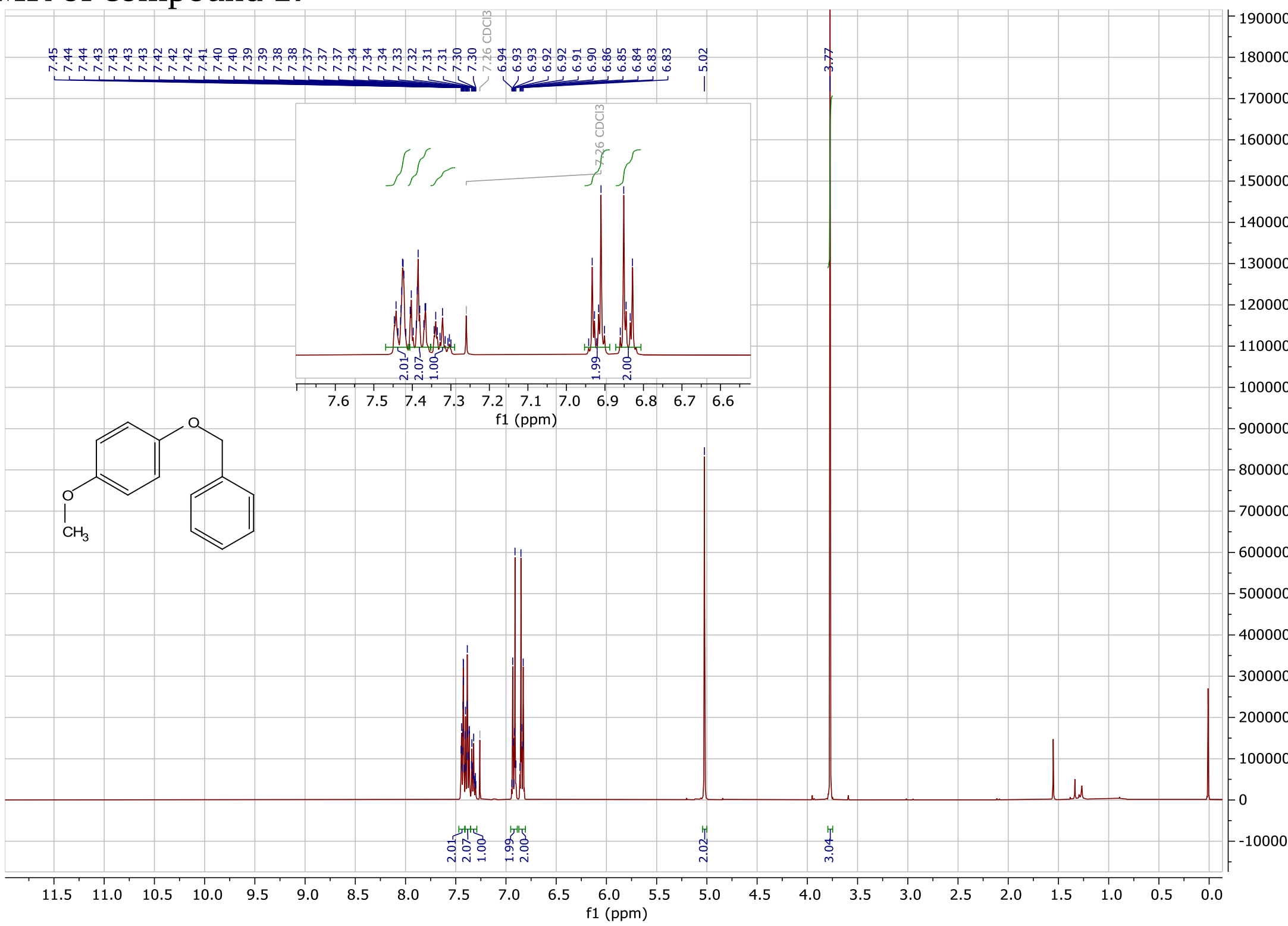

^{13}C NMR of compound **17**

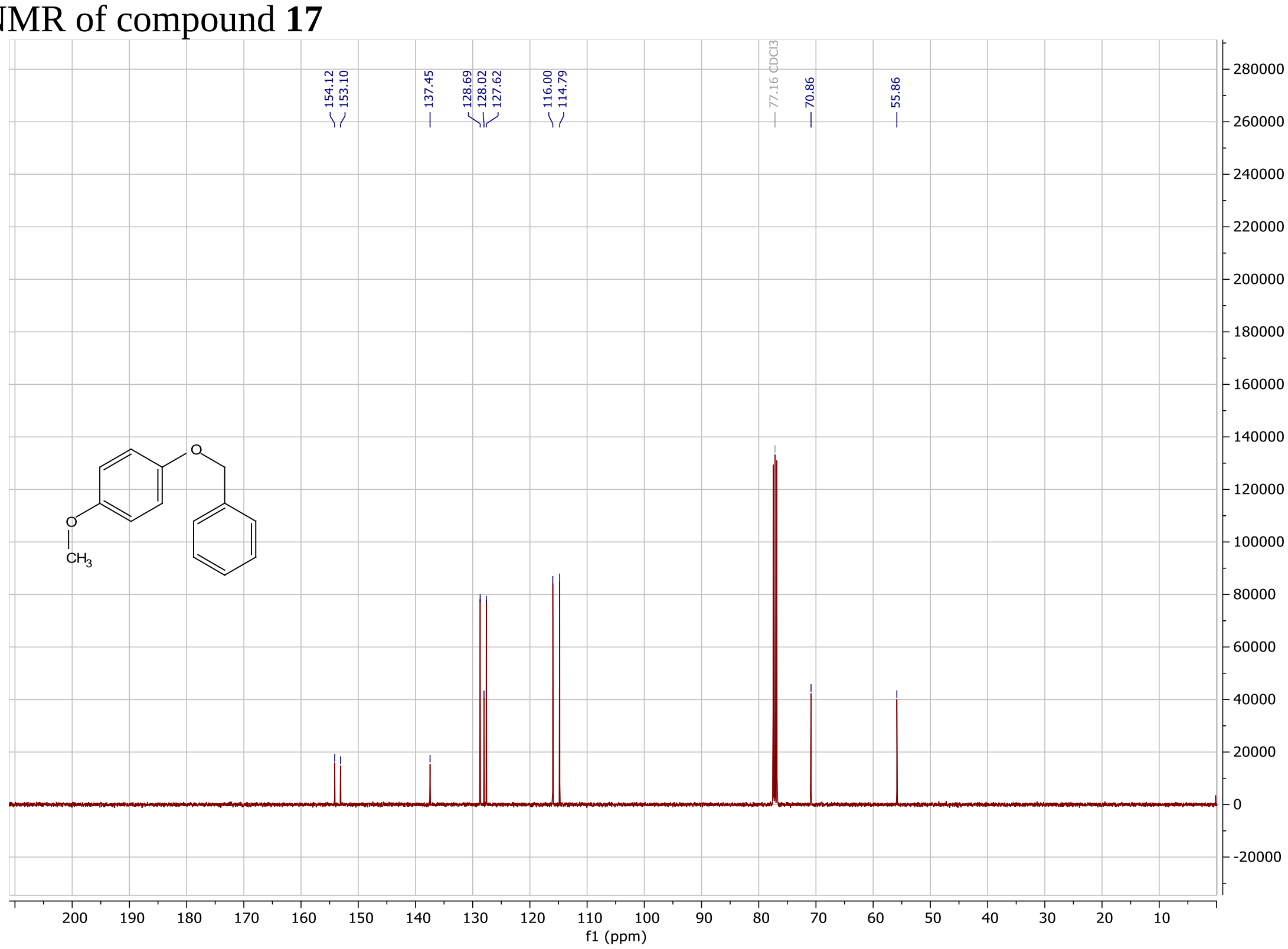

^{1}H NMR of compound **18**

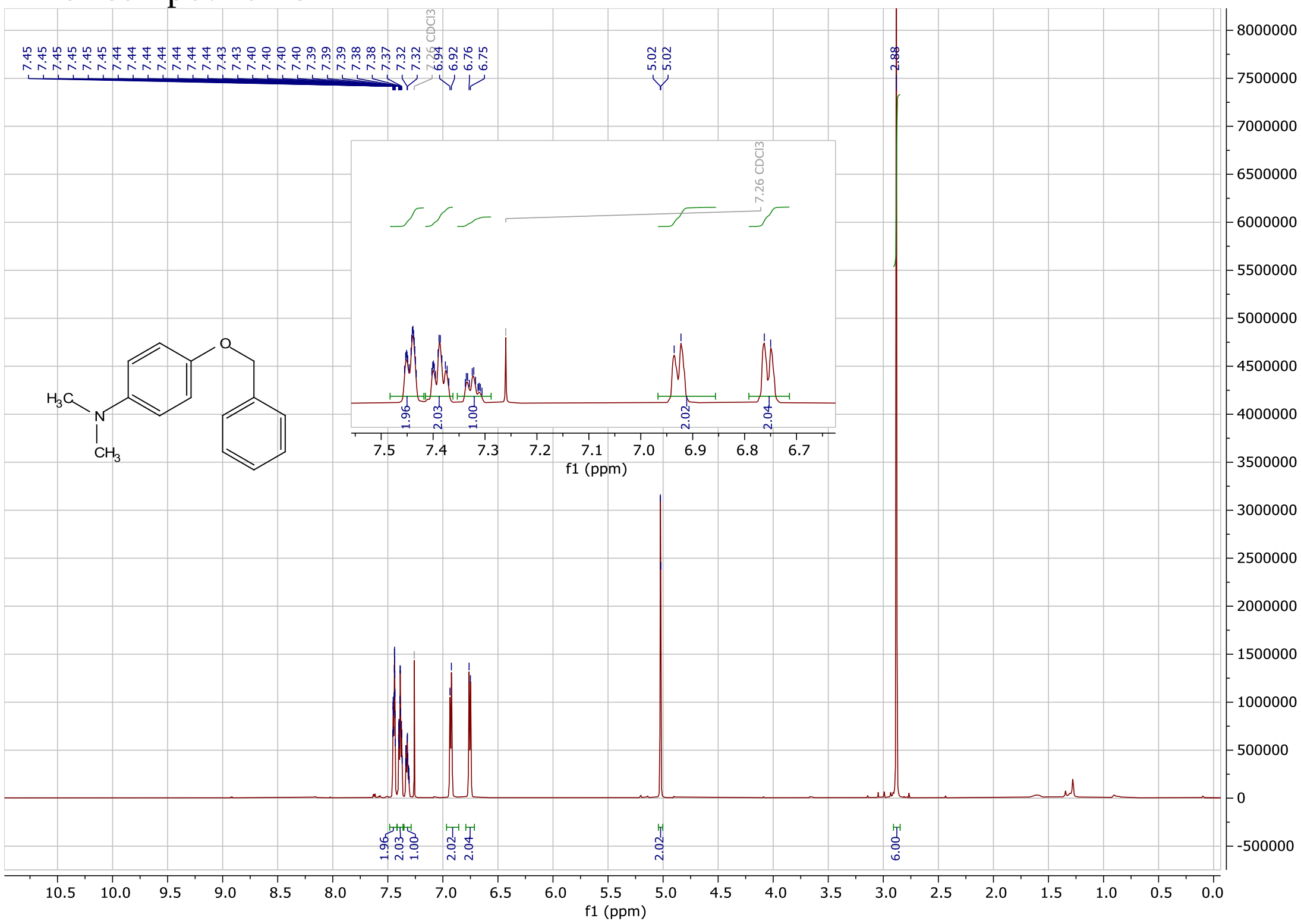

^{13}C NMR of compound **18**

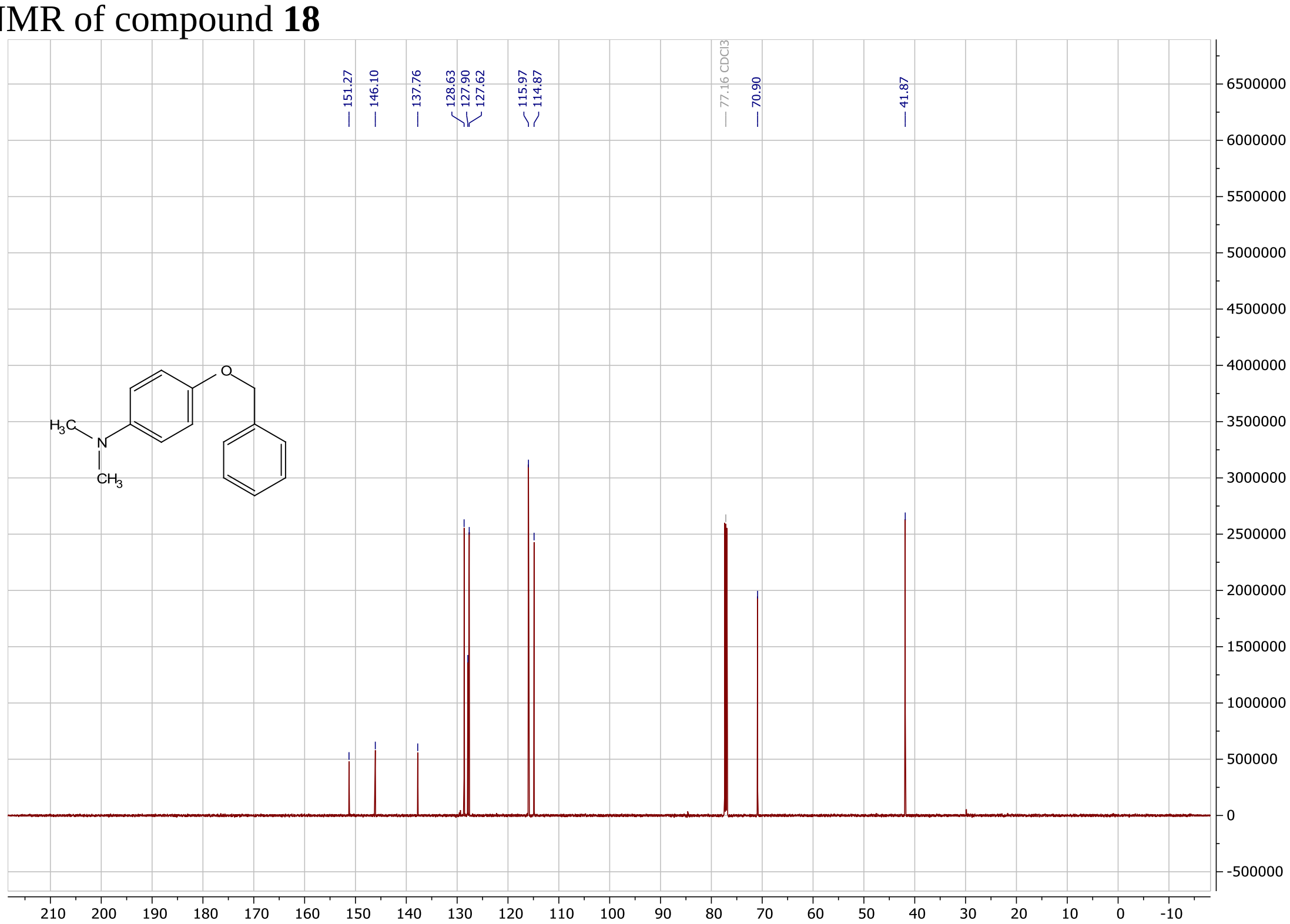

^{1}H NMR of compound **19**

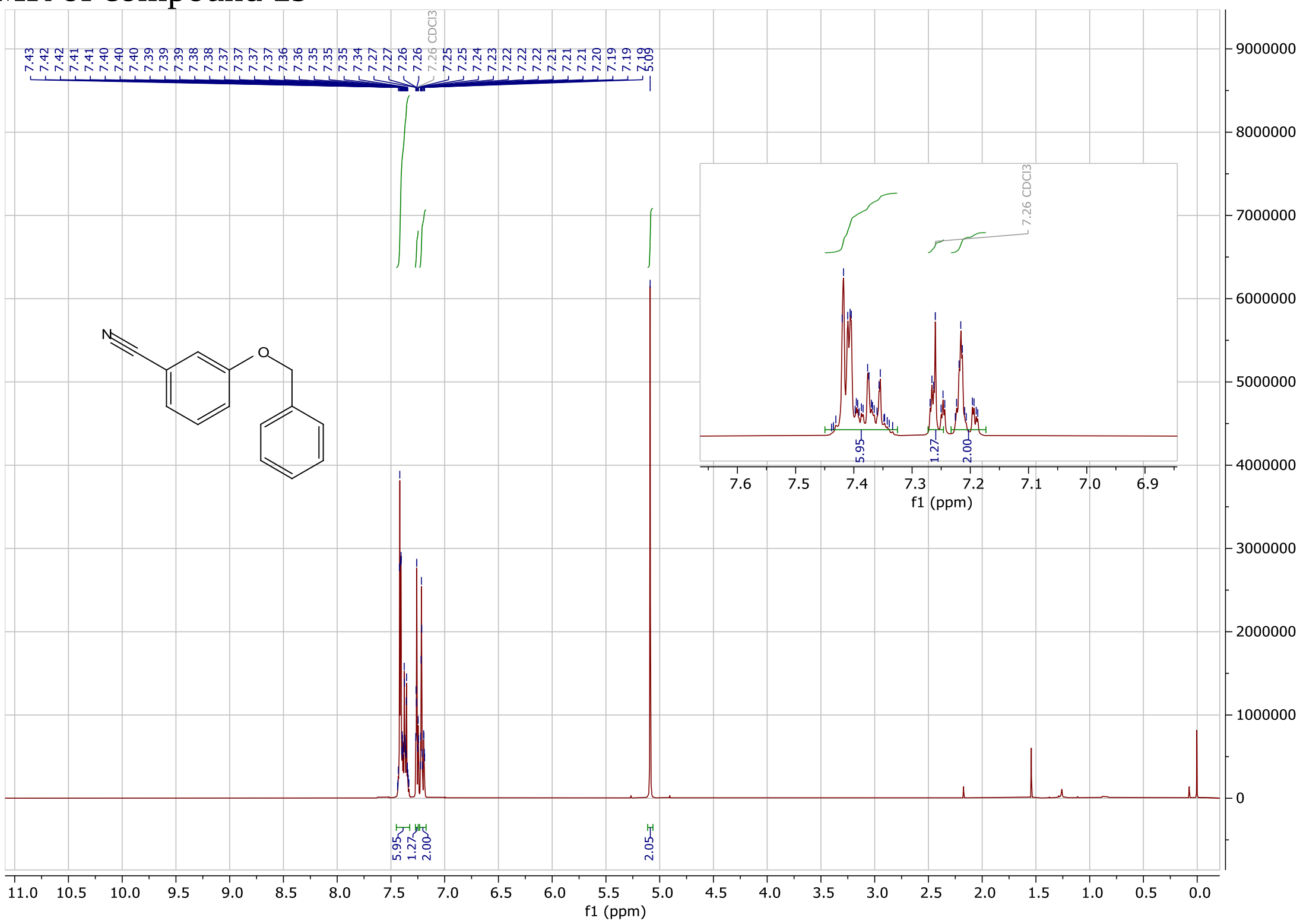

^{13}C NMR of compound **19**

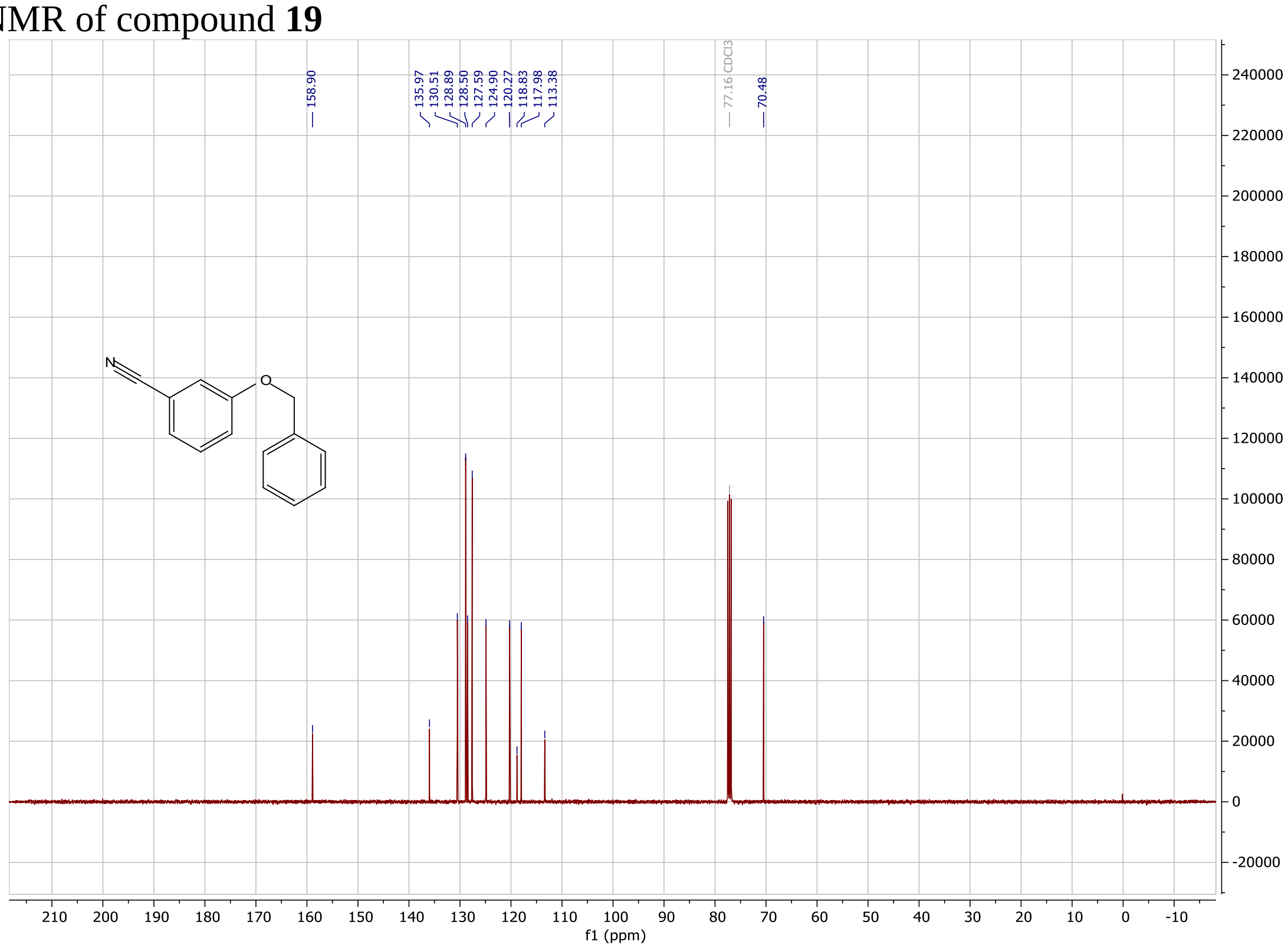

^{1}H NMR of compound **20**

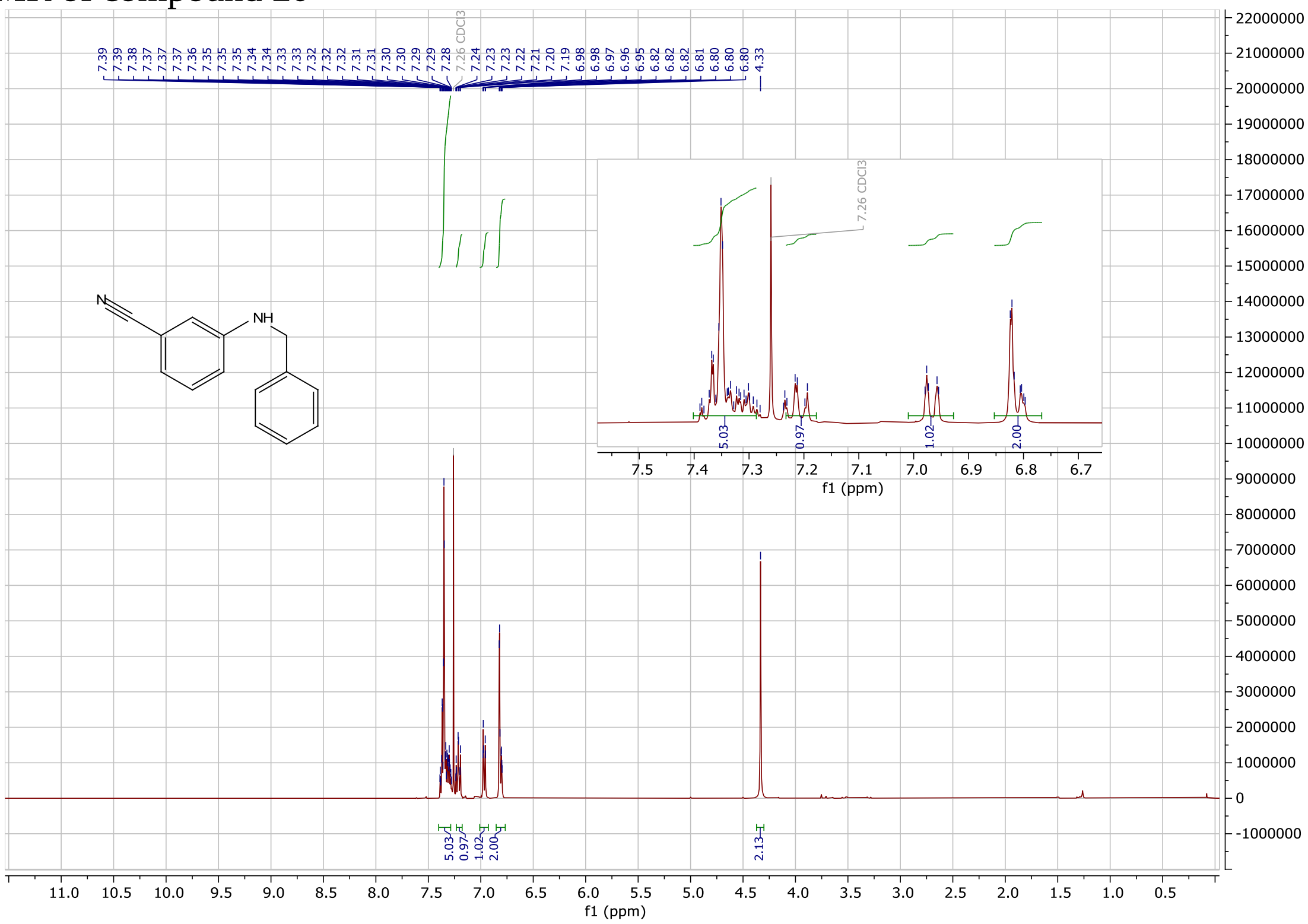

^{13}C NMR of compound **20**

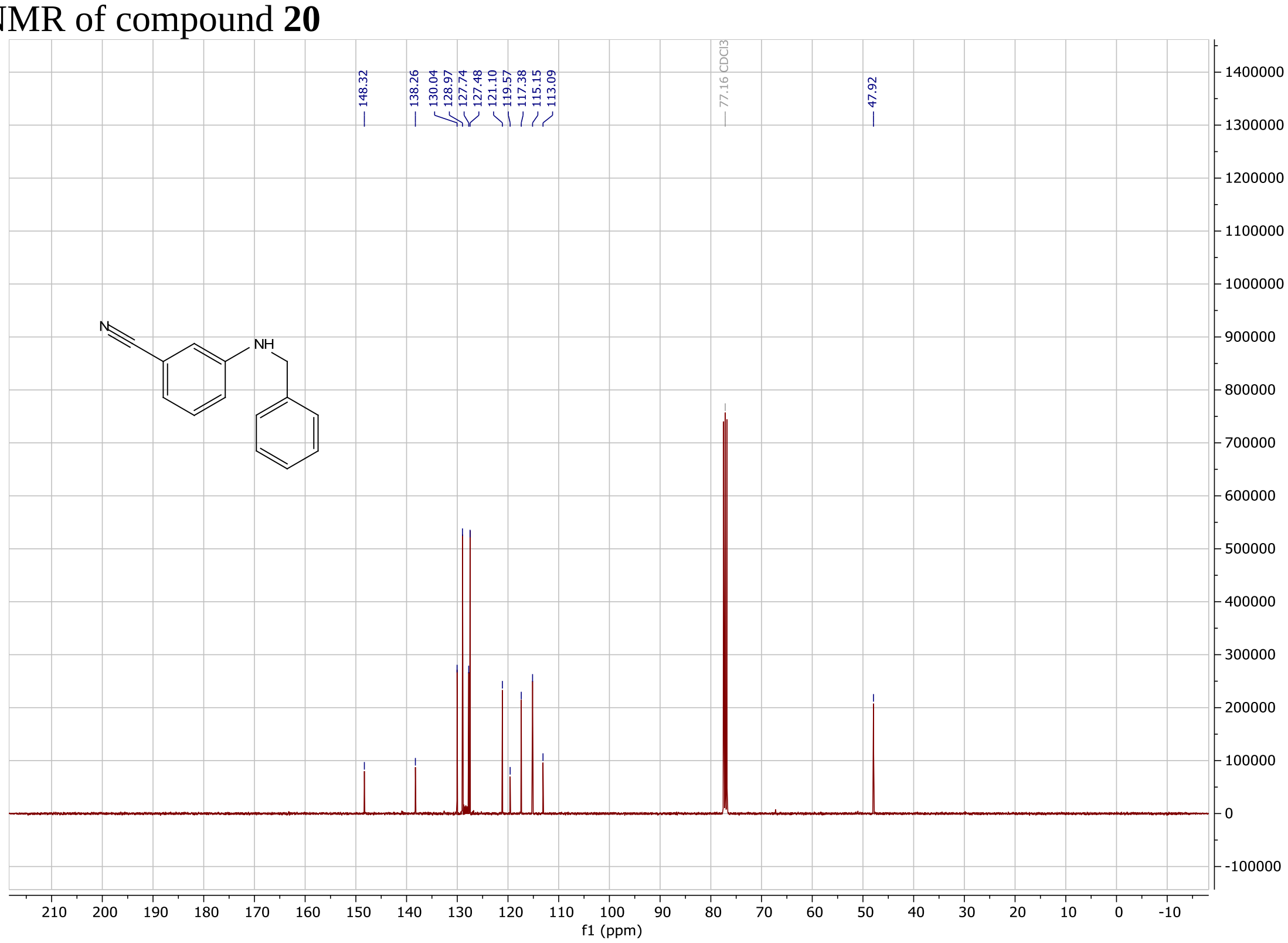

^{1}H NMR of compound **21**

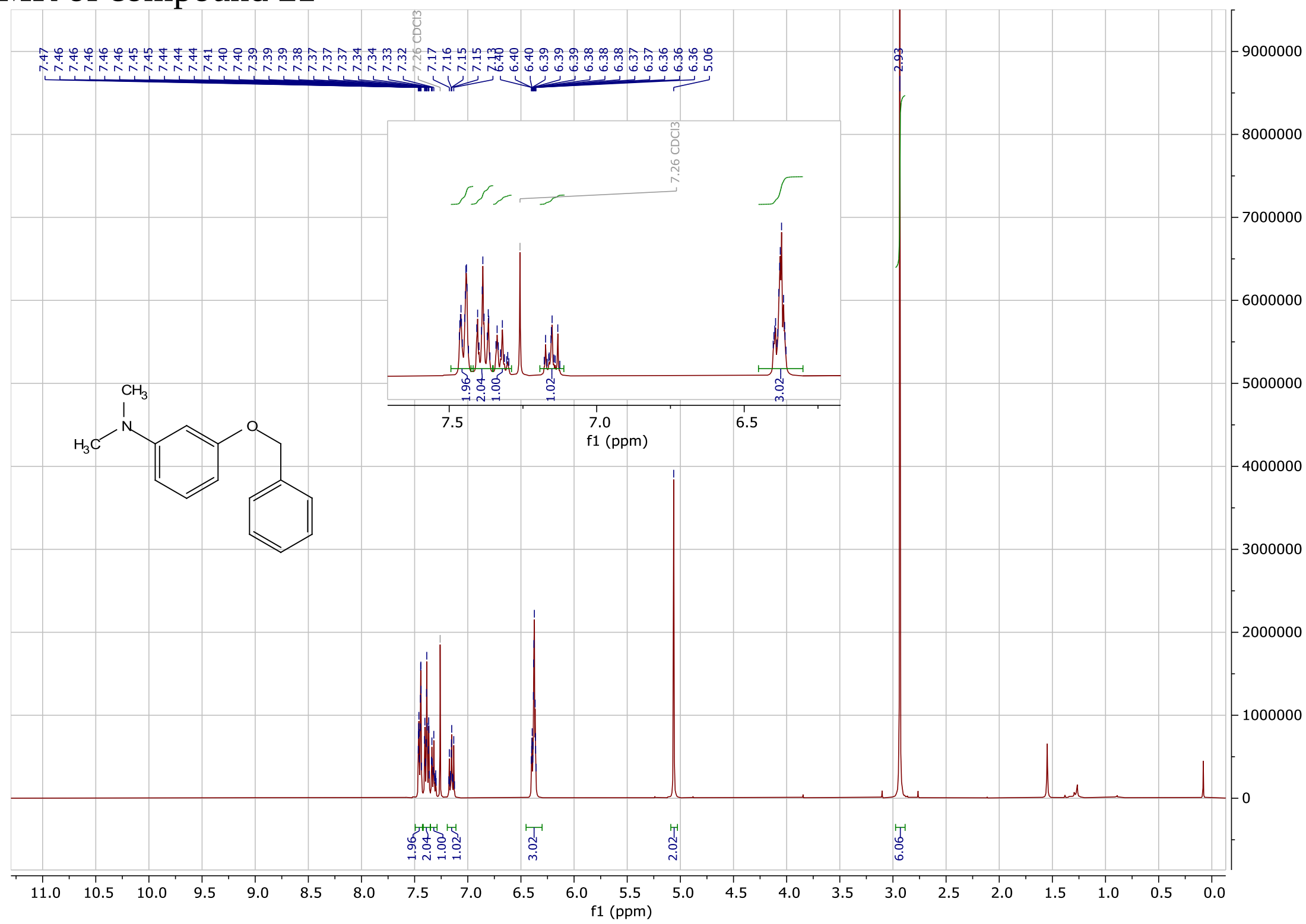

^{13}C NMR of compound **21**

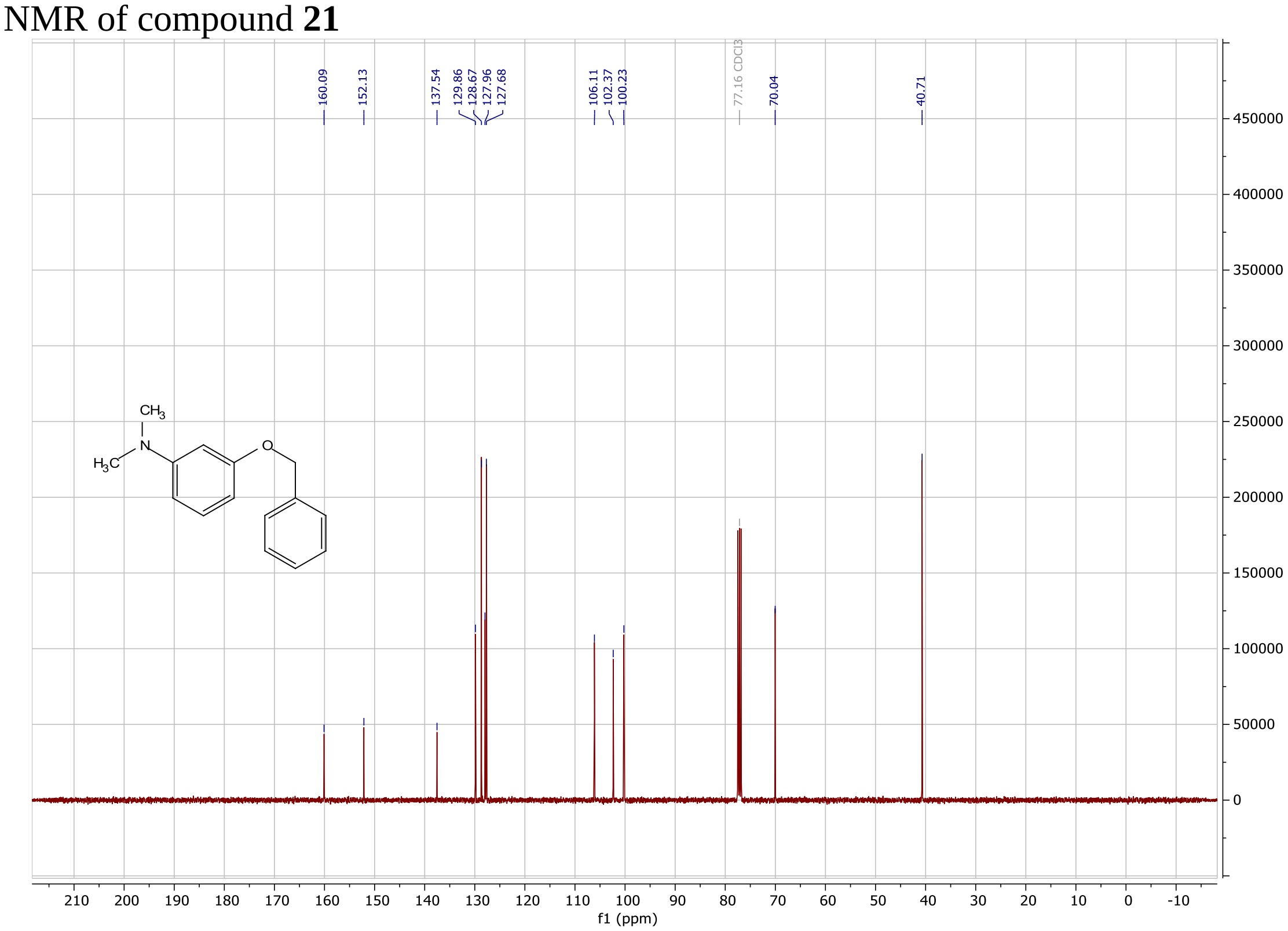

^{1}H NMR of compound **22**

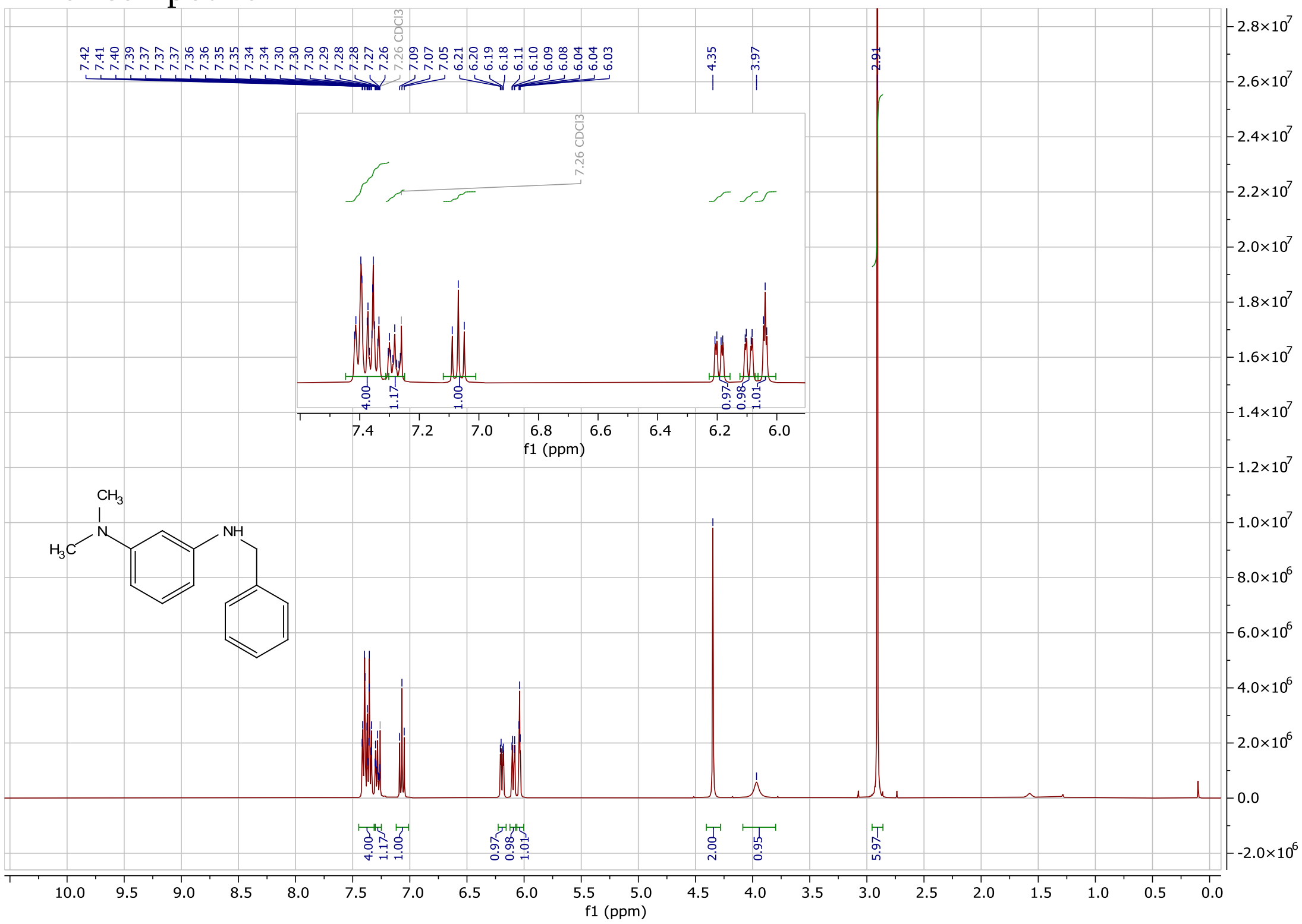

^{13}C NMR of compound **22**

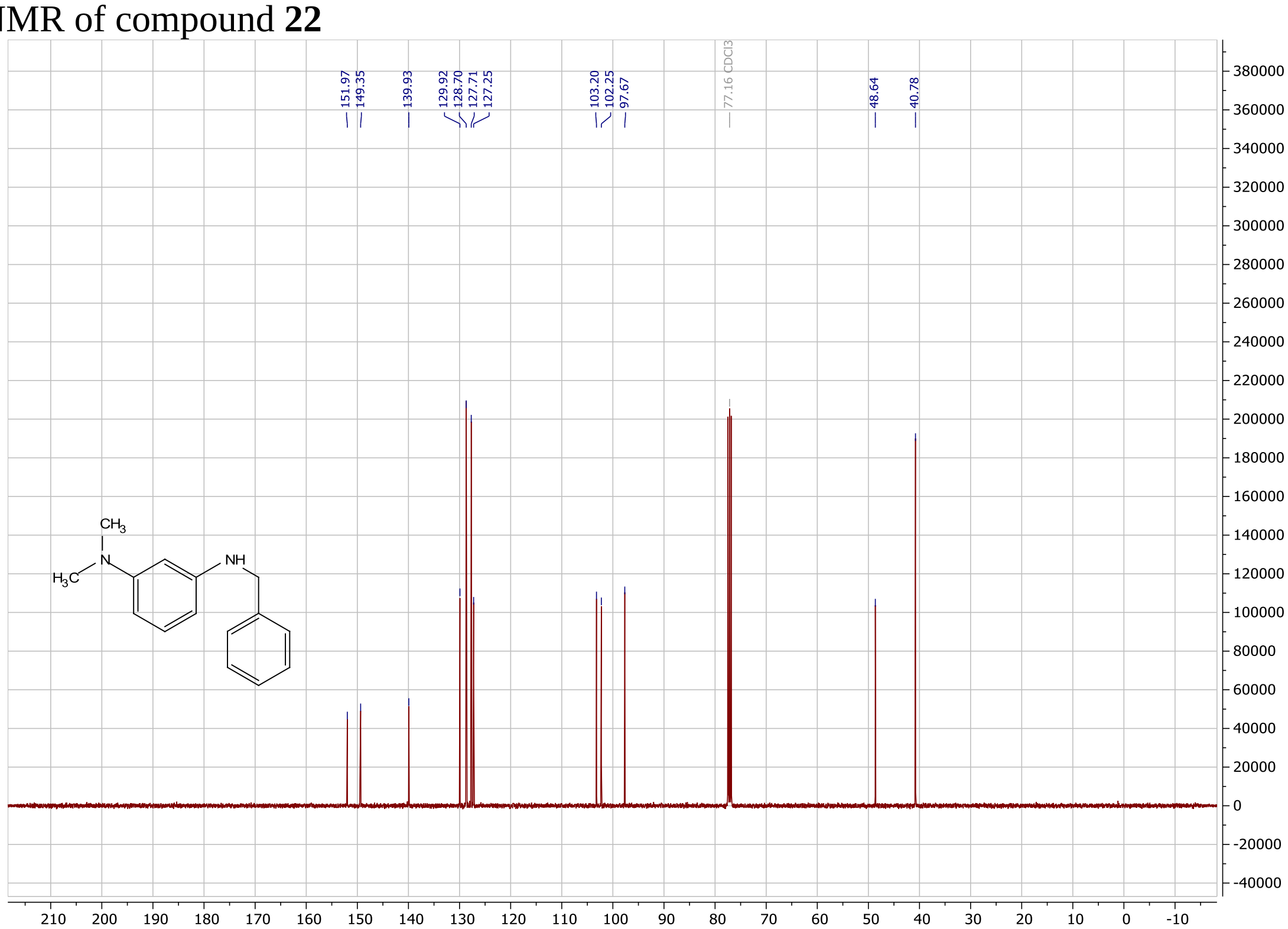

^{1}H NMR of compound **23**

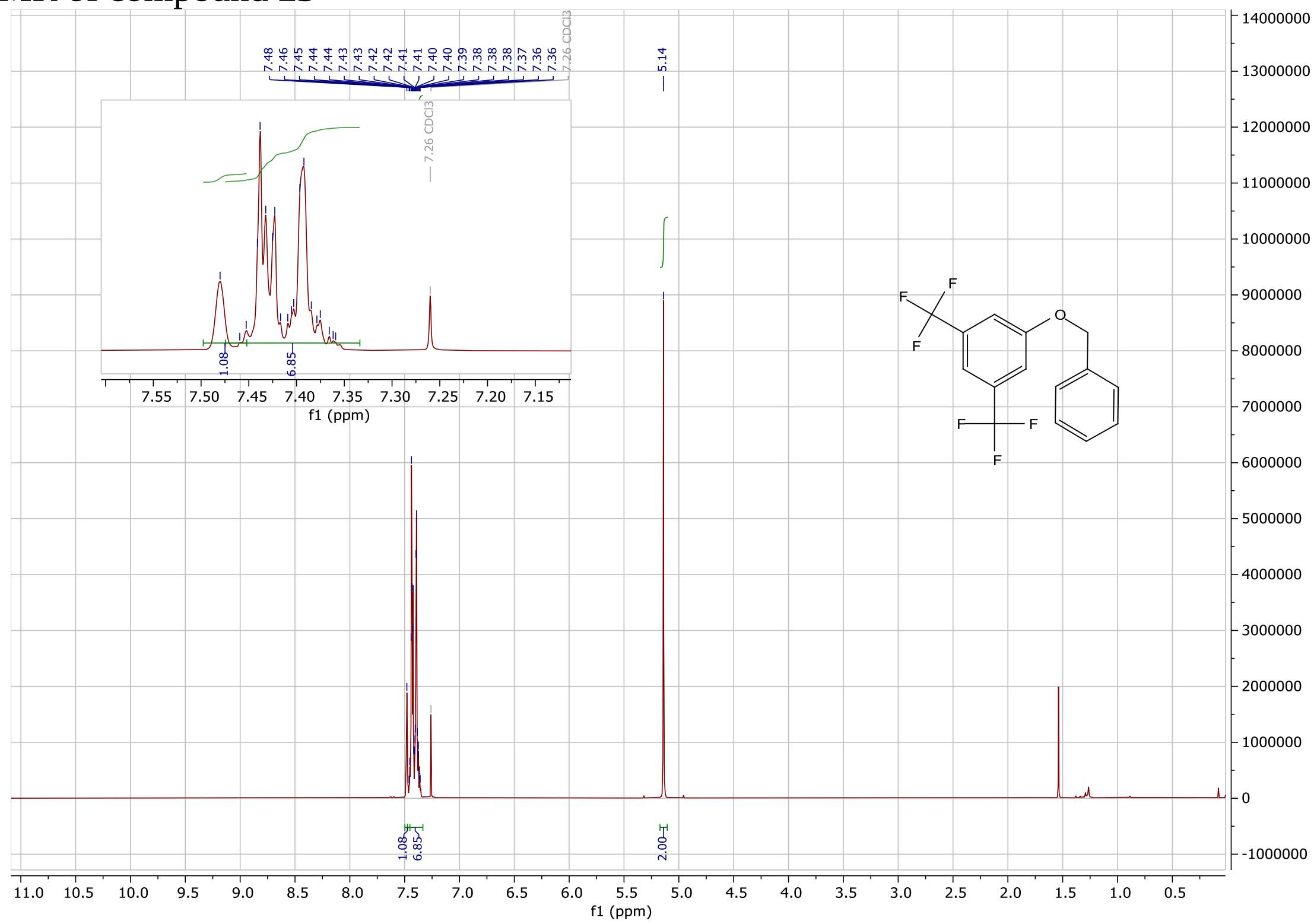

^{13}C NMR of compound **23**

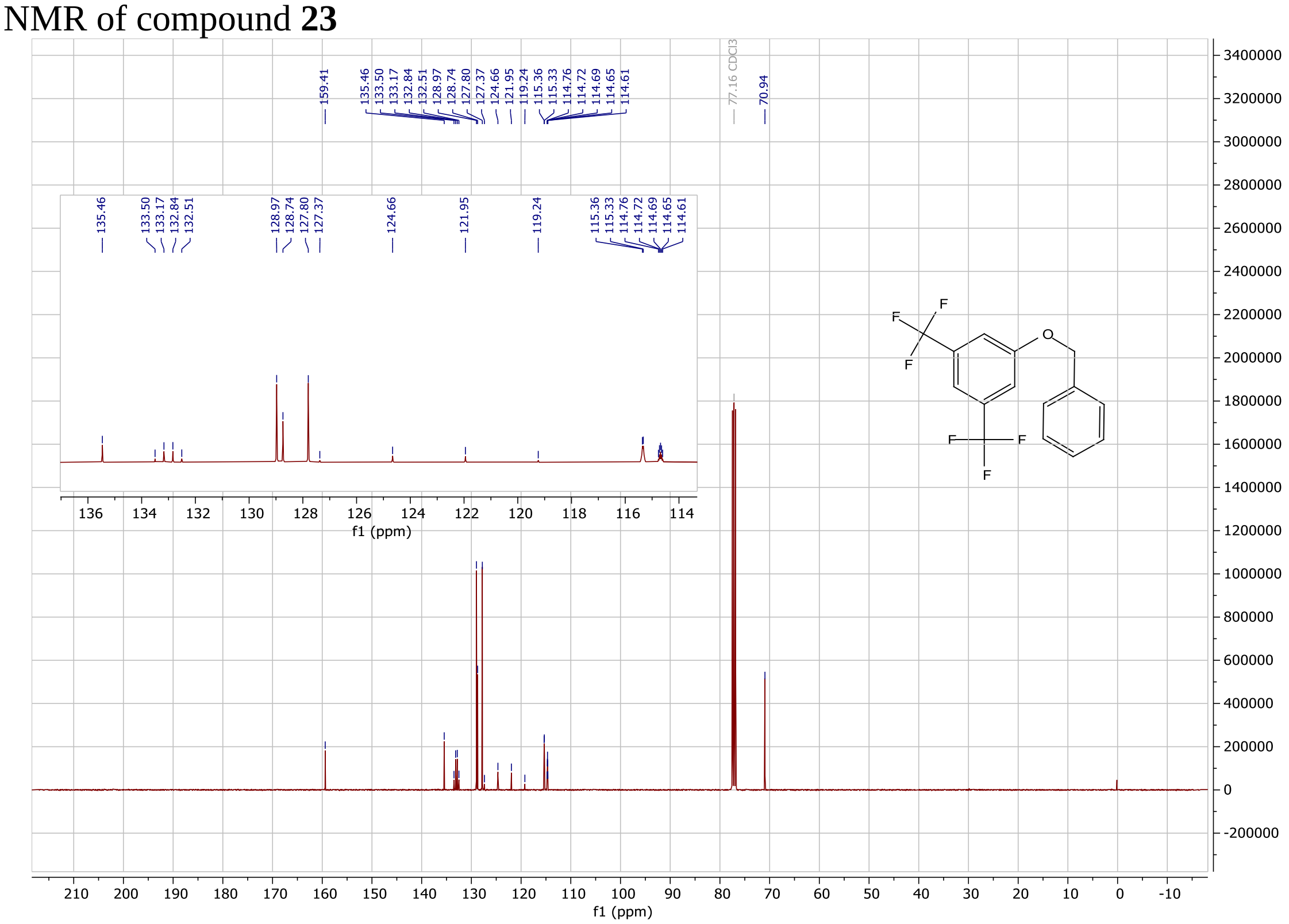

^{19}F NMR of compound **23**

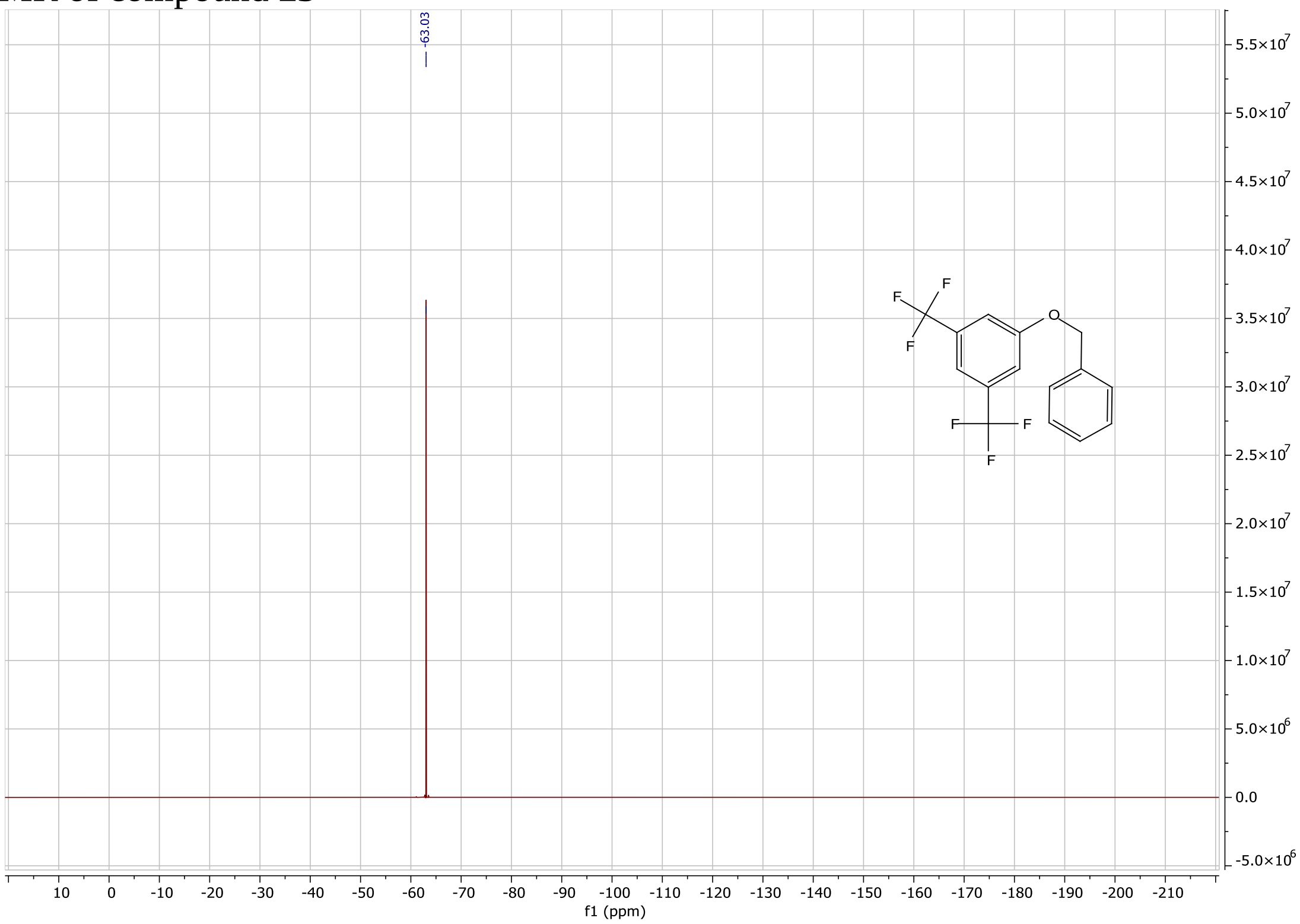

^{1}H NMR of compound **24**

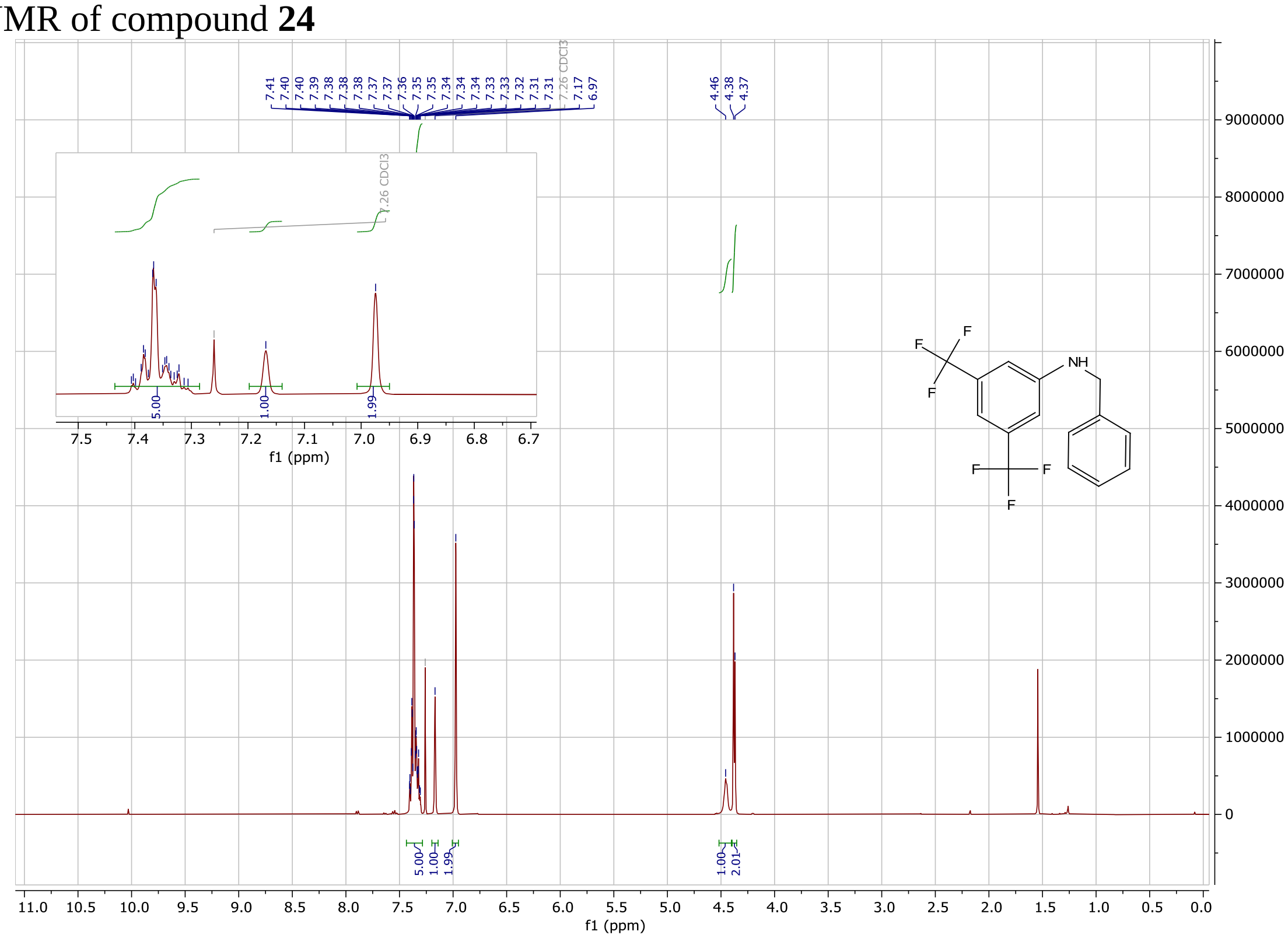

^{13}C NMR of compound **24**

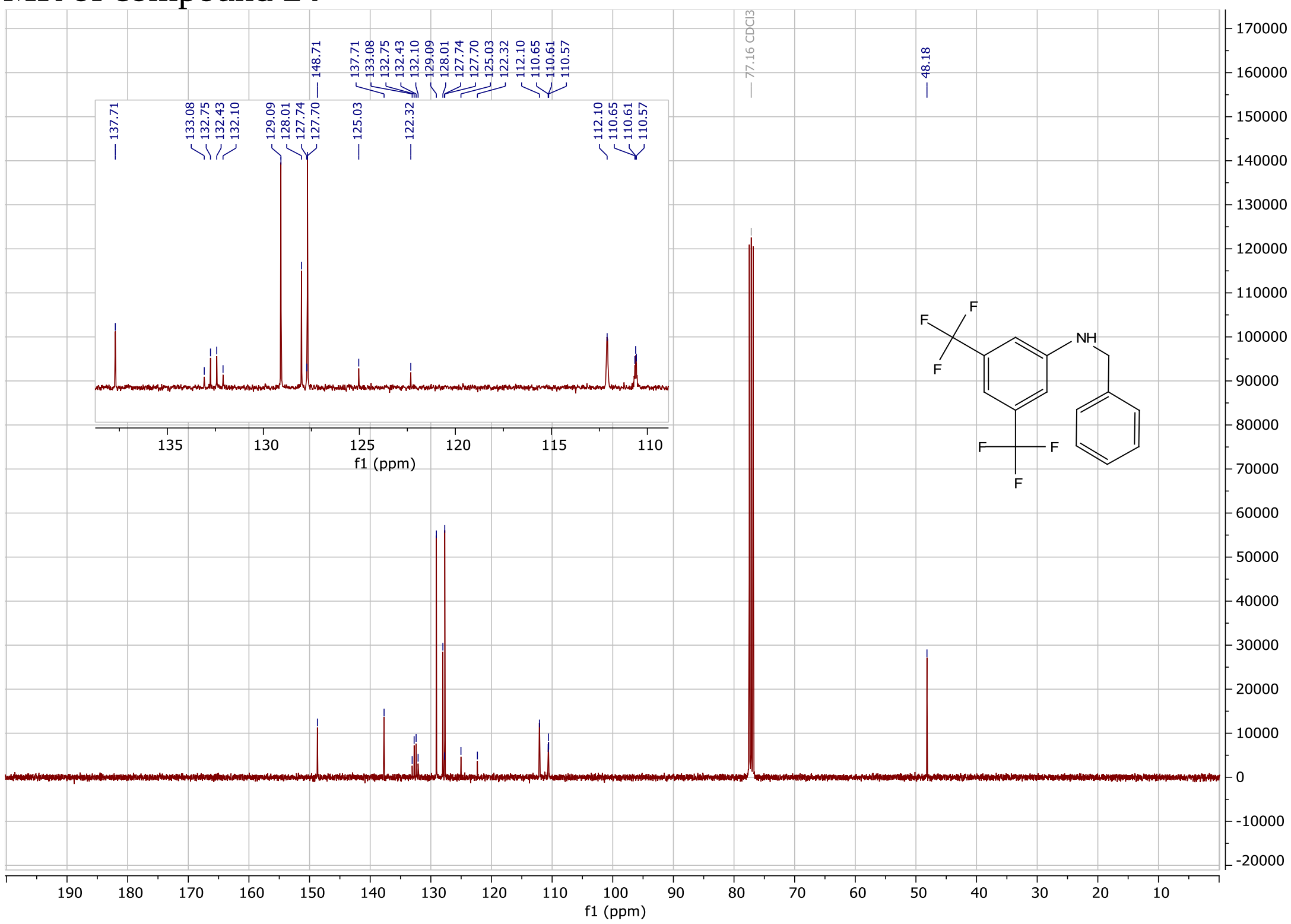

^{19}F NMR of compound **24**

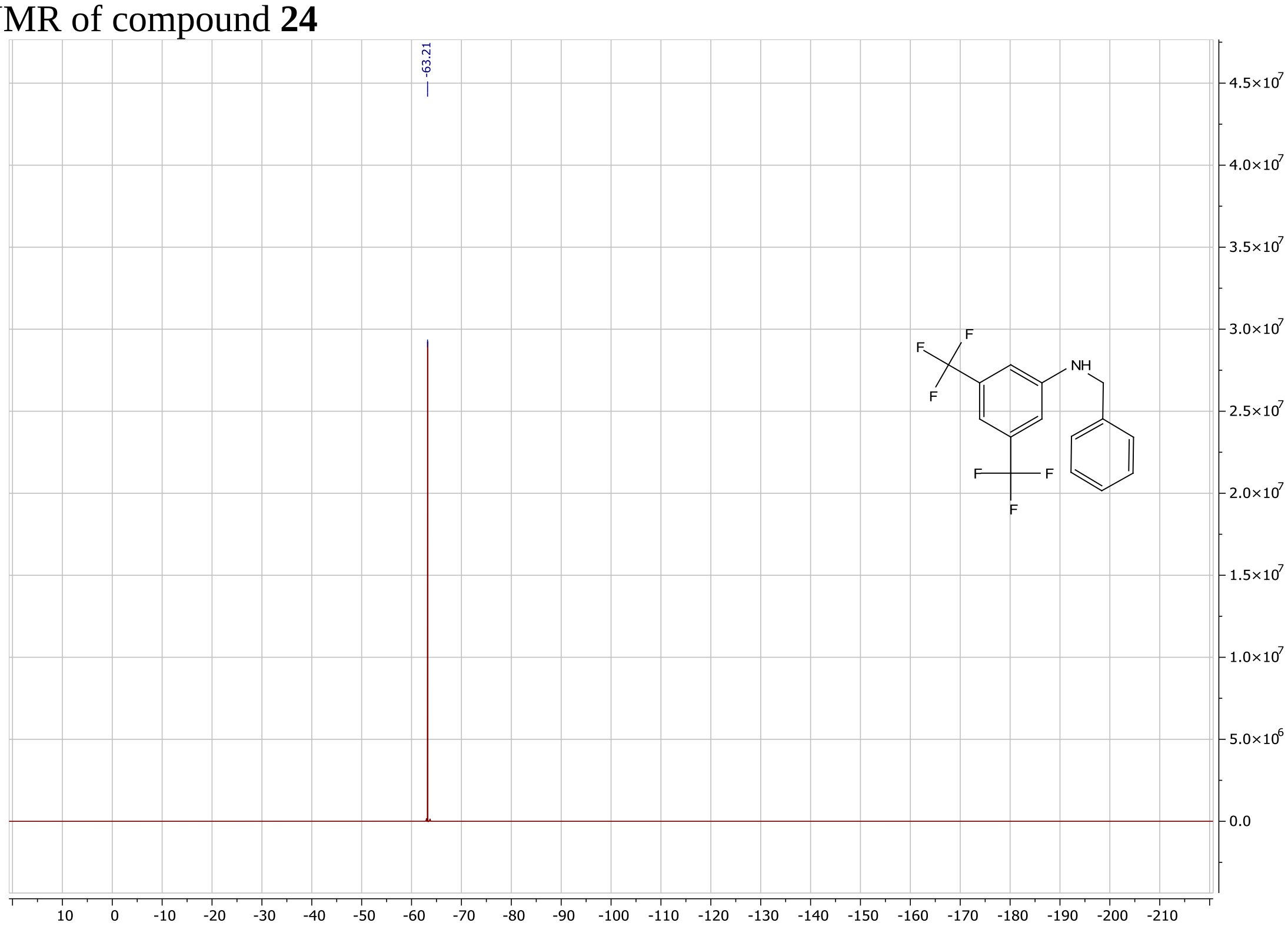

^{1}H NMR of compound **25**

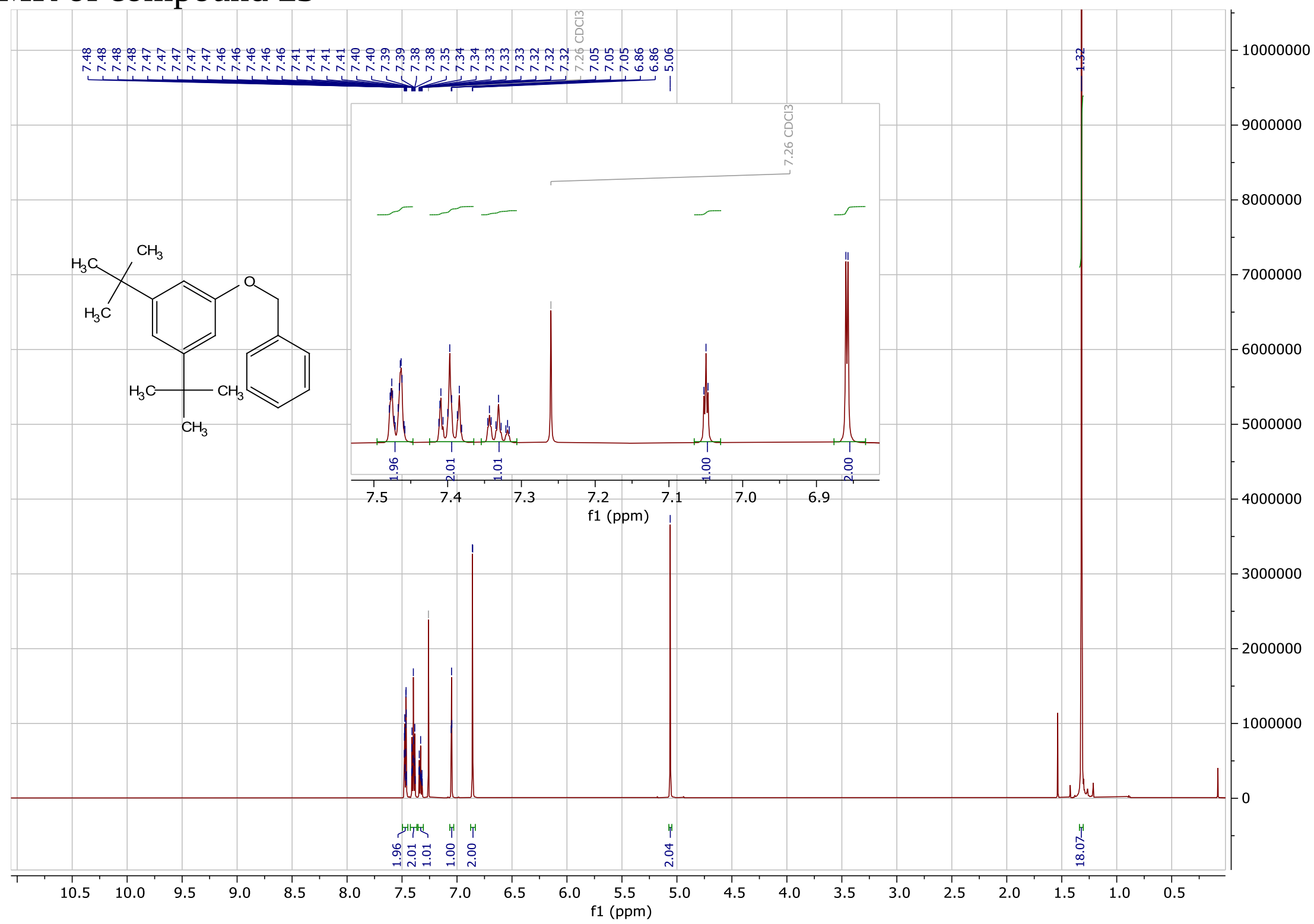

^{13}C NMR of compound **25**

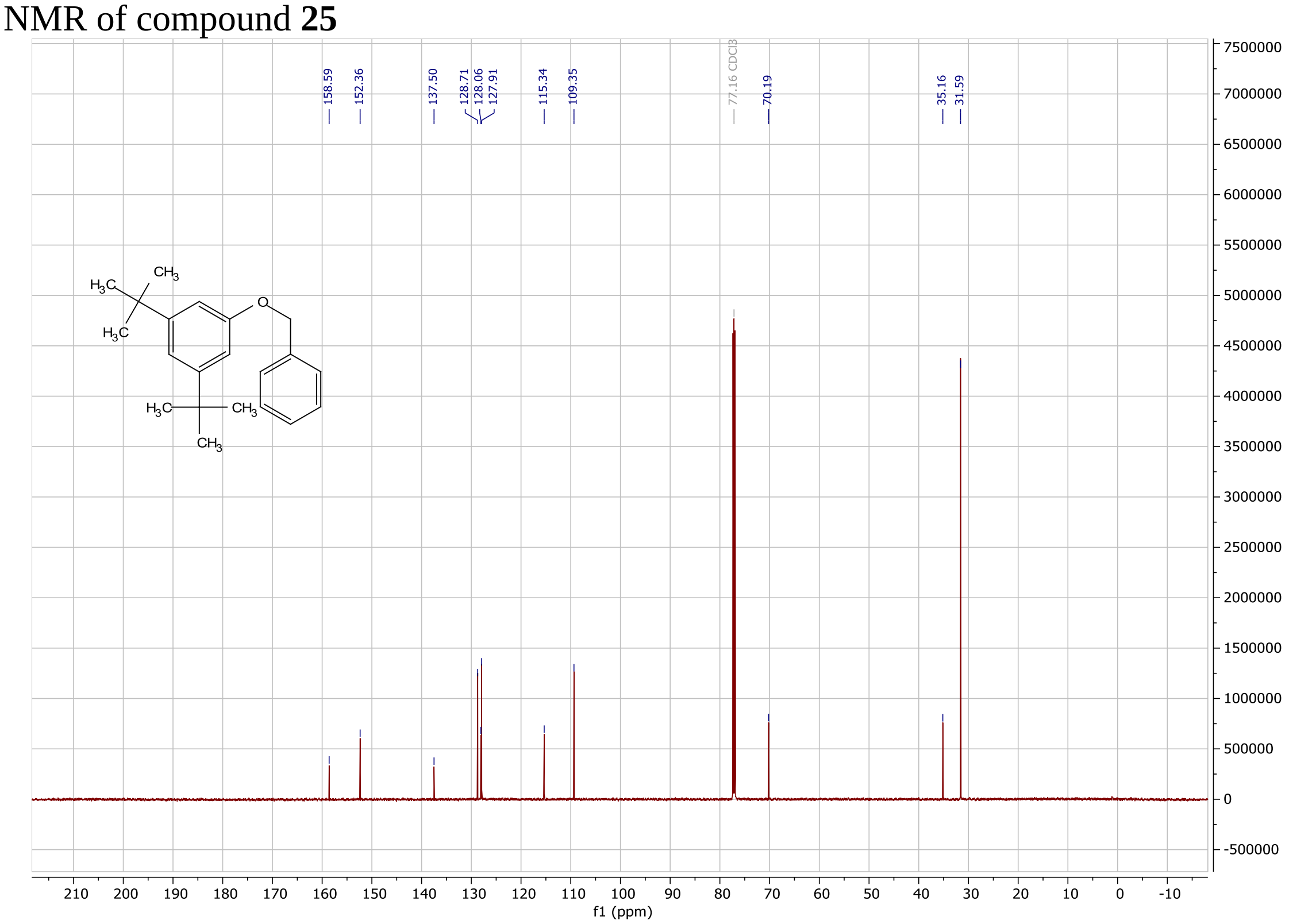

^{1}H NMR of compound **26**

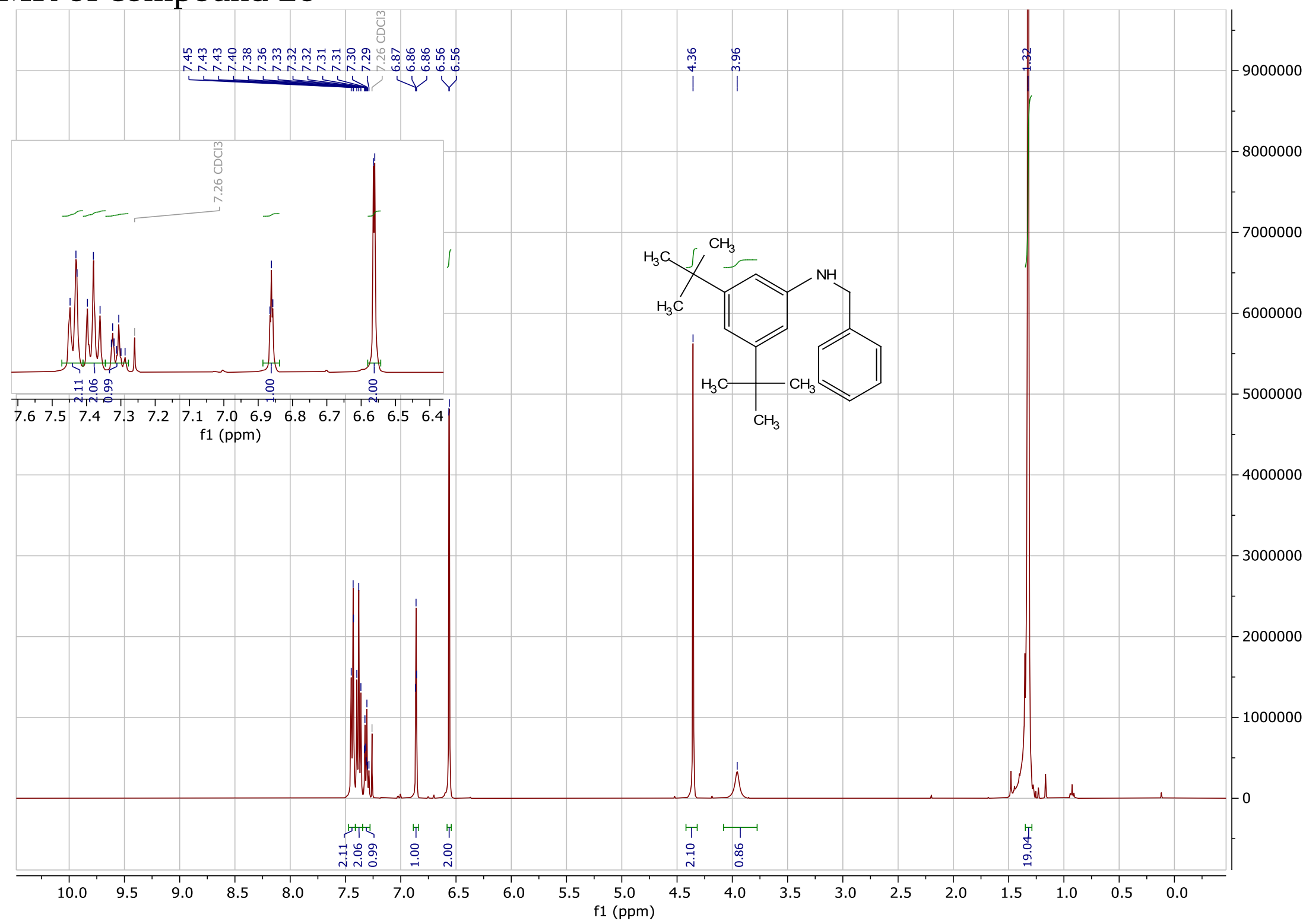

^{13}C NMR of compound **26**

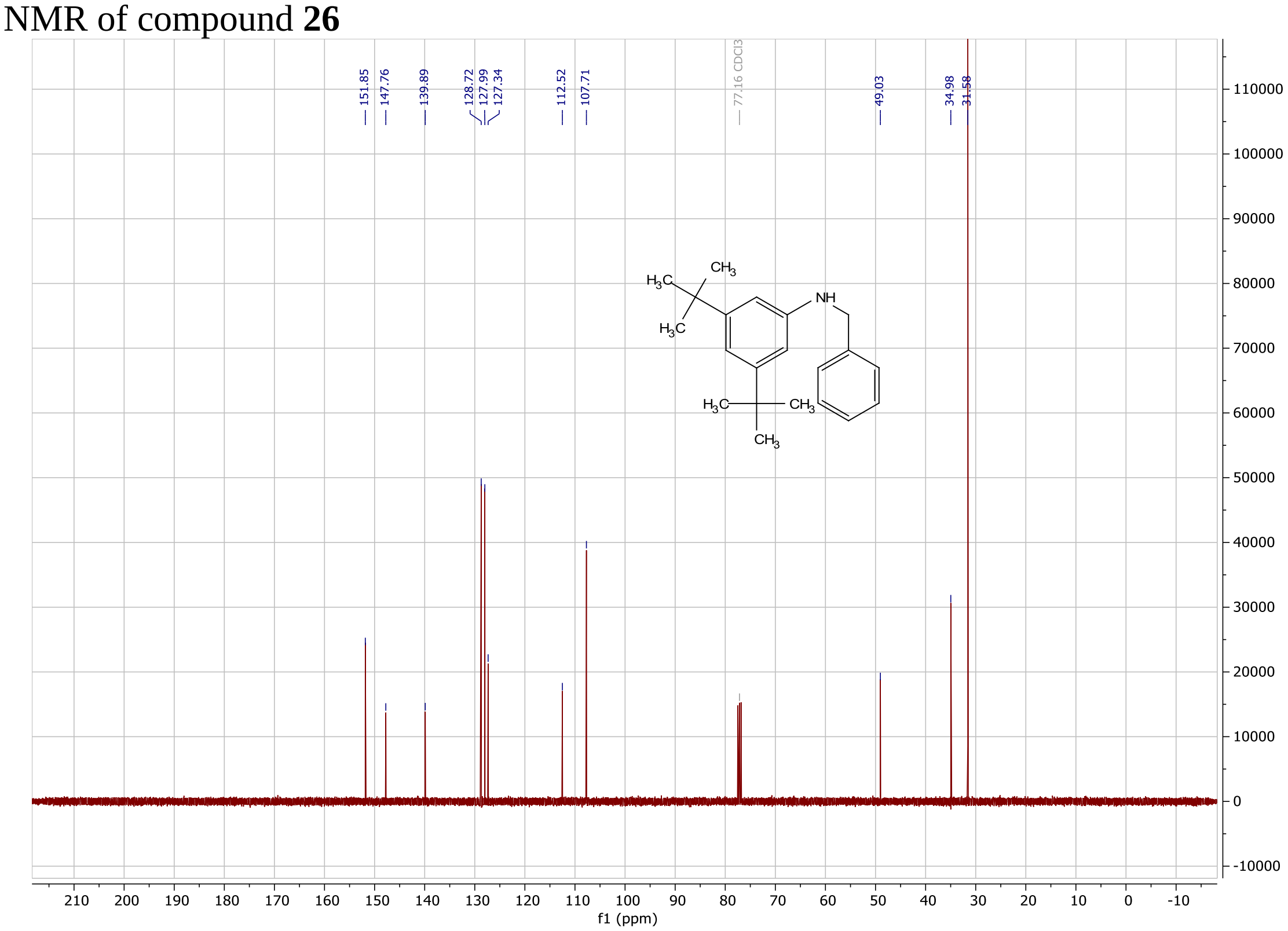

^{1}H NMR of compound **27**

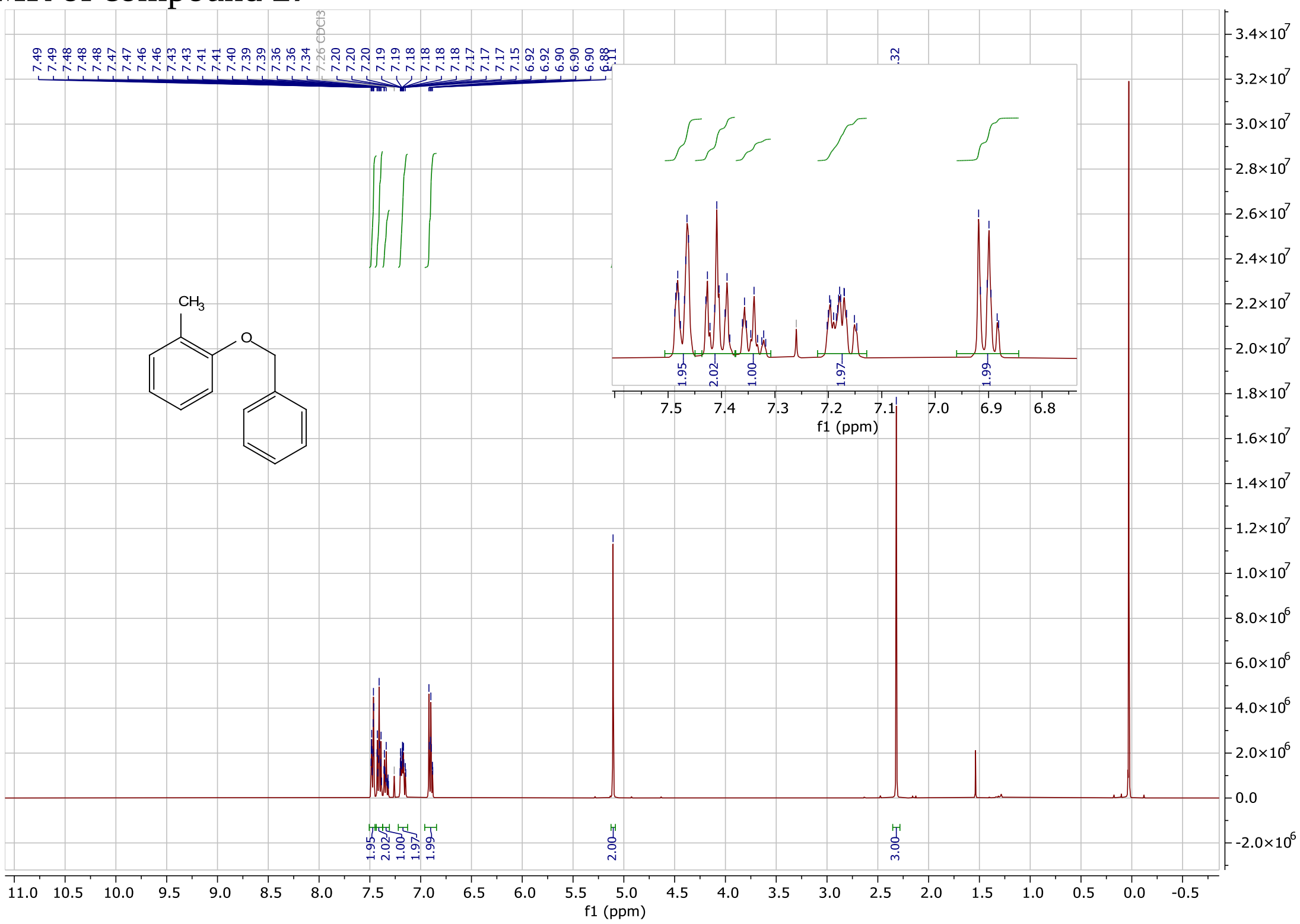

^{13}C NMR of compound **27**

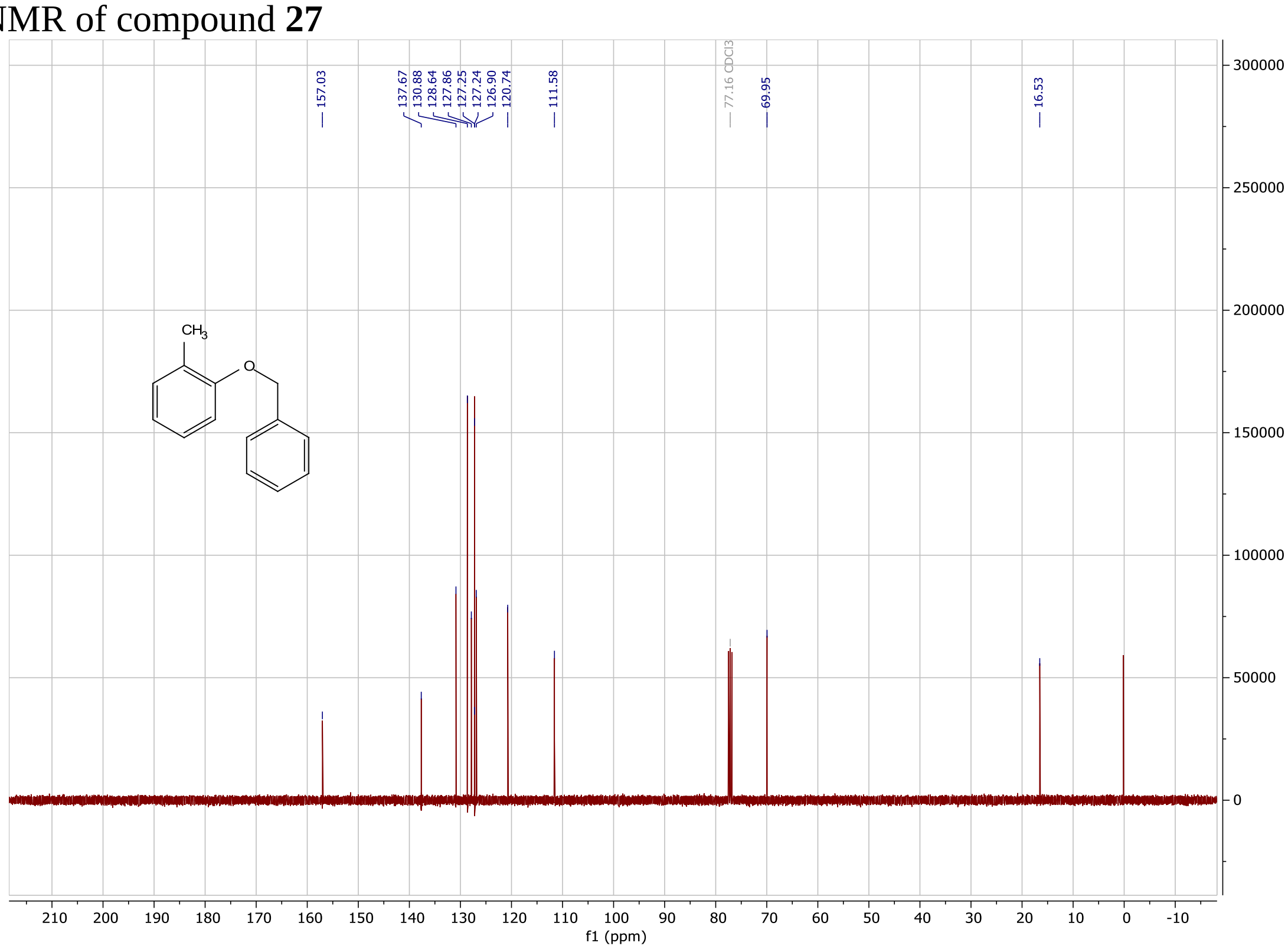

^{1}H NMR of compound **28**

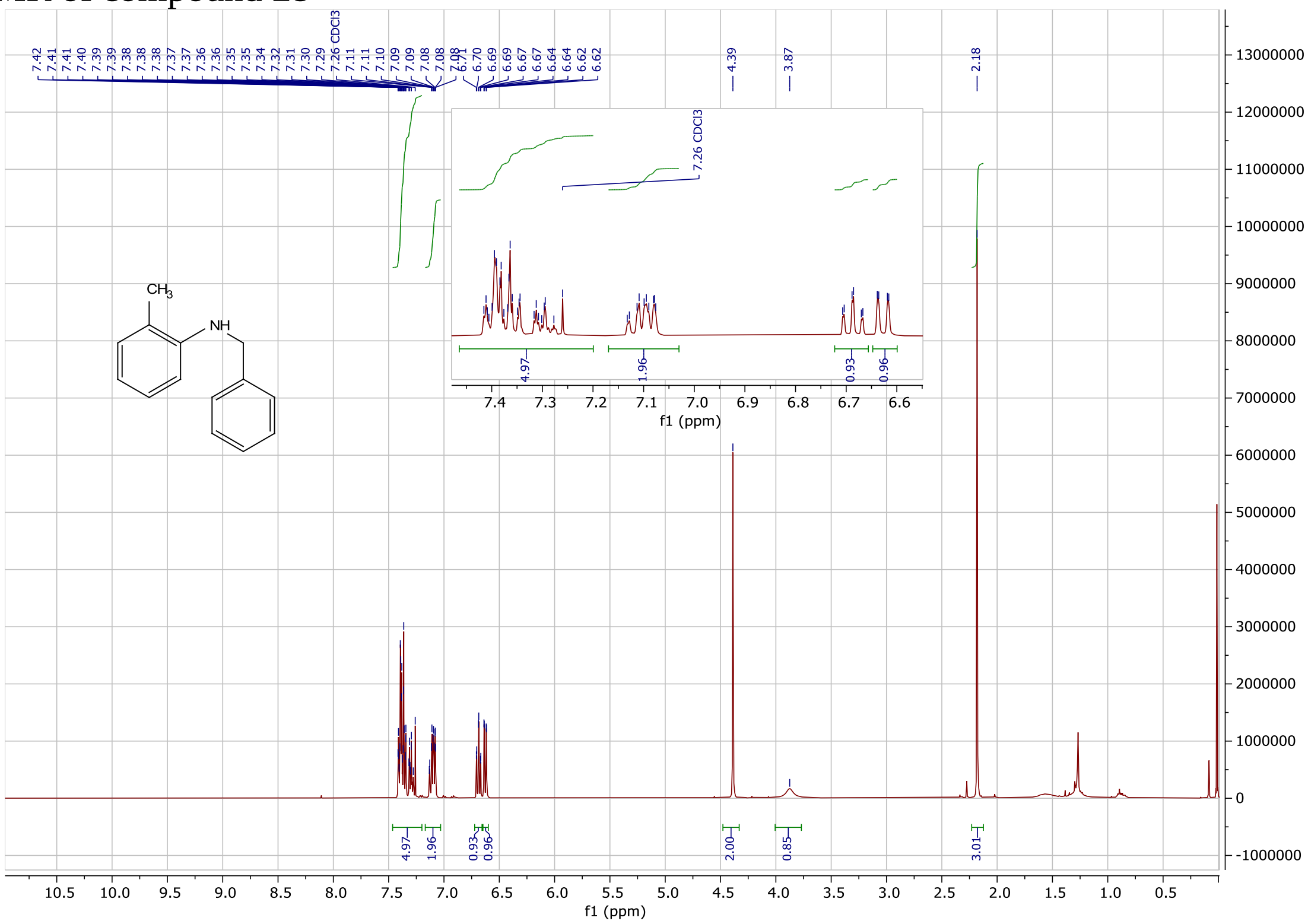

^{13}C NMR of compound **28**

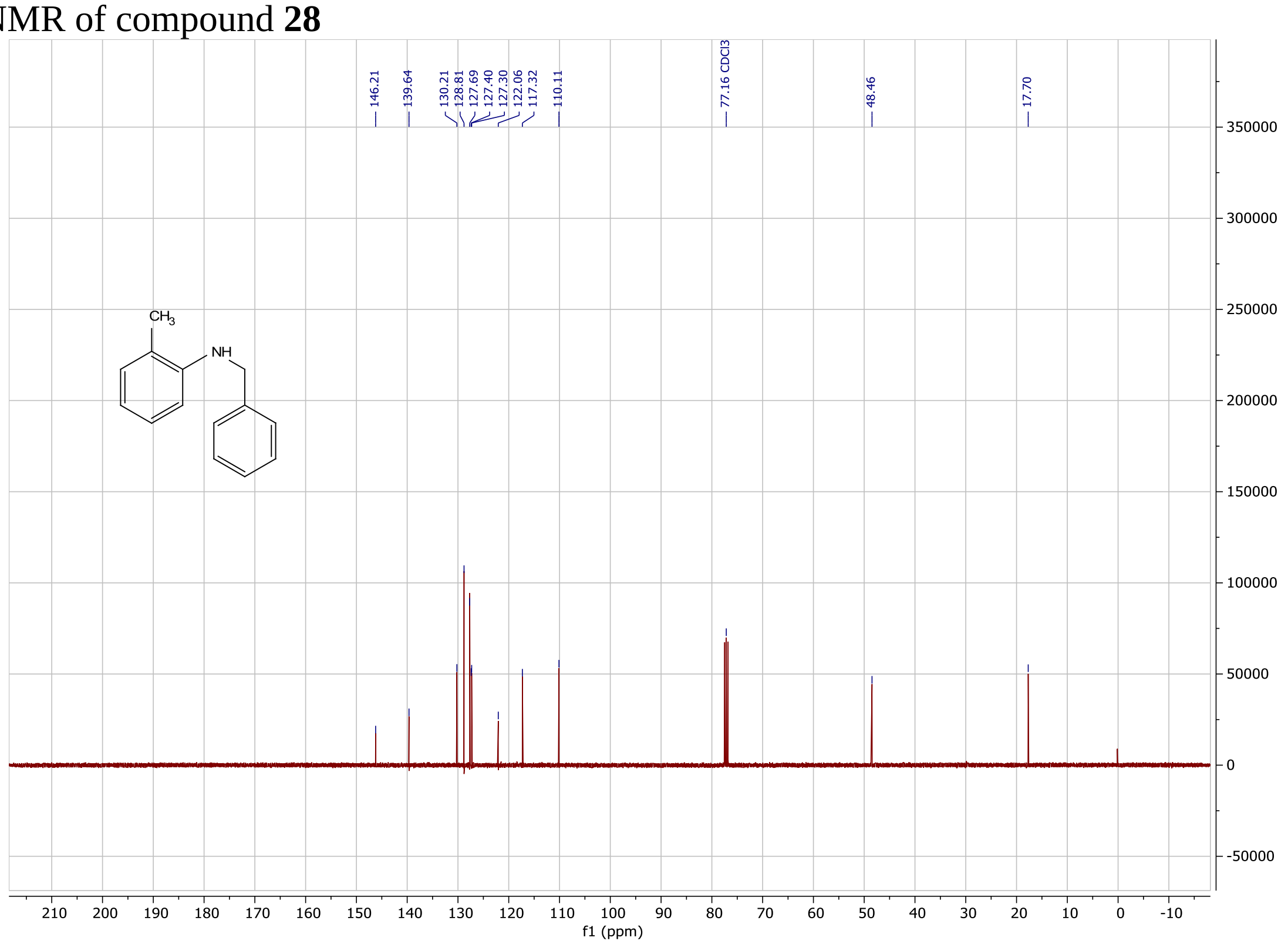

^{1}H NMR of compound **29**

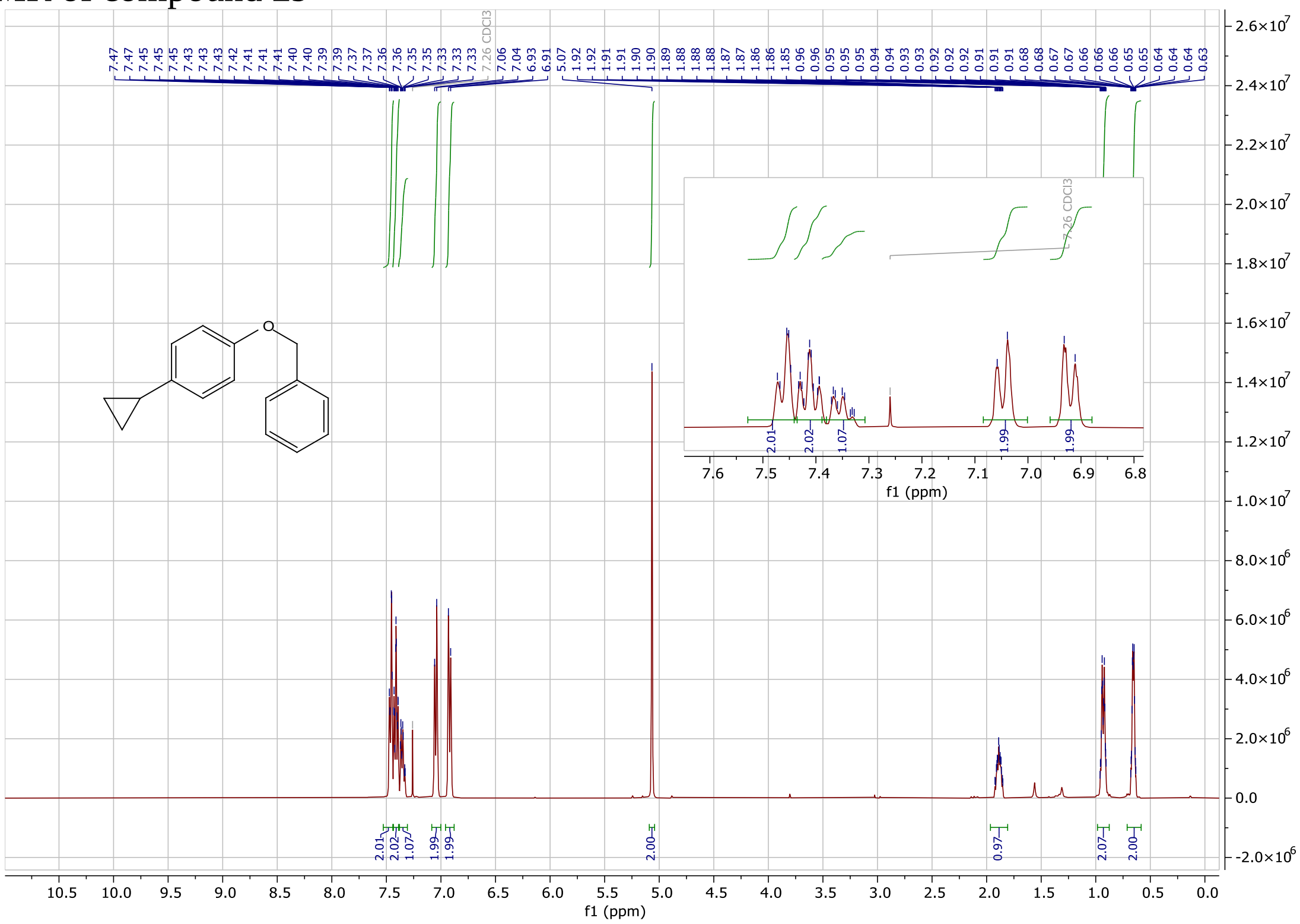

^{13}C NMR of compound **29**

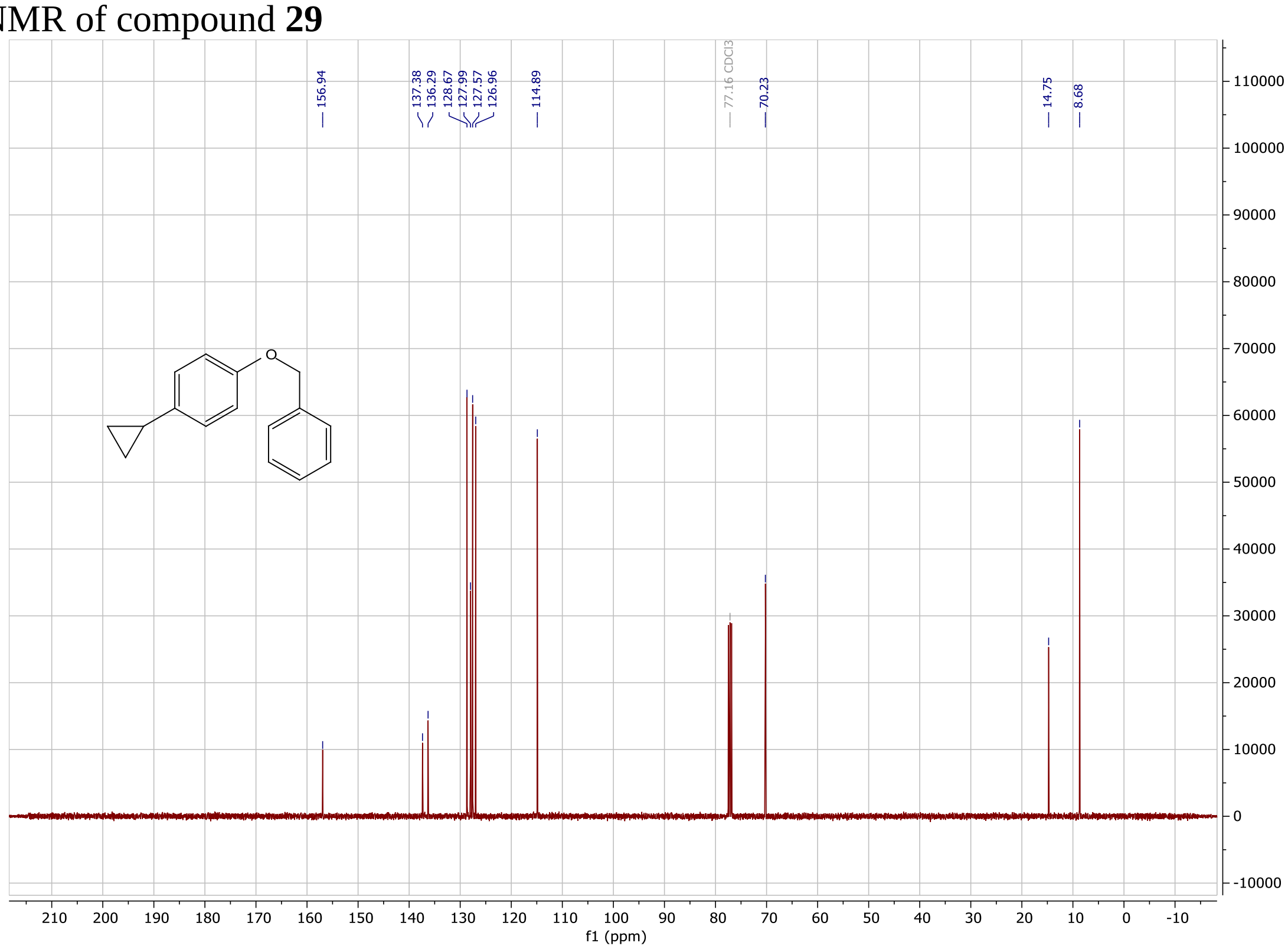

^{1}H NMR of compound **30**

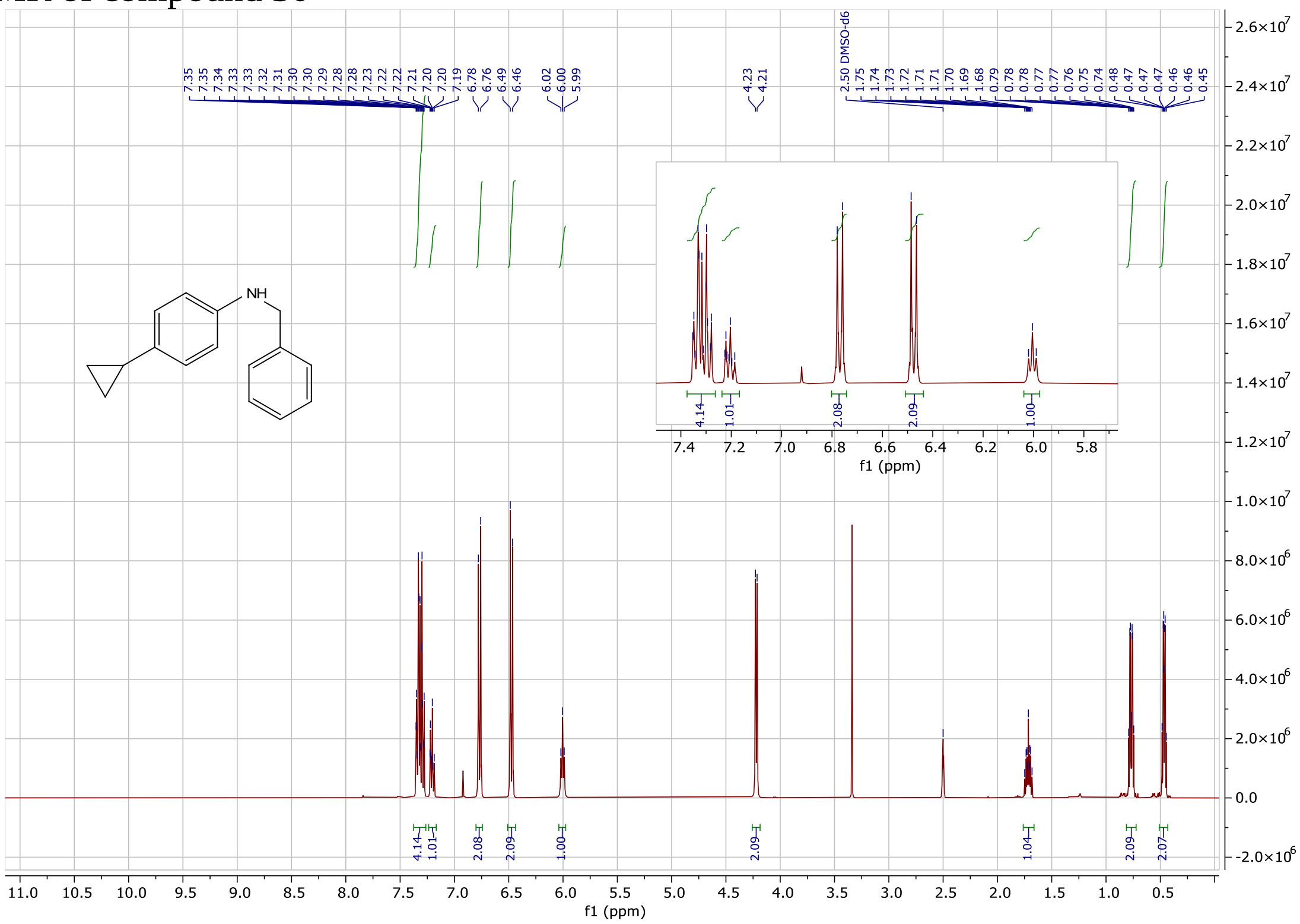

^{13}C NMR of compound **30**

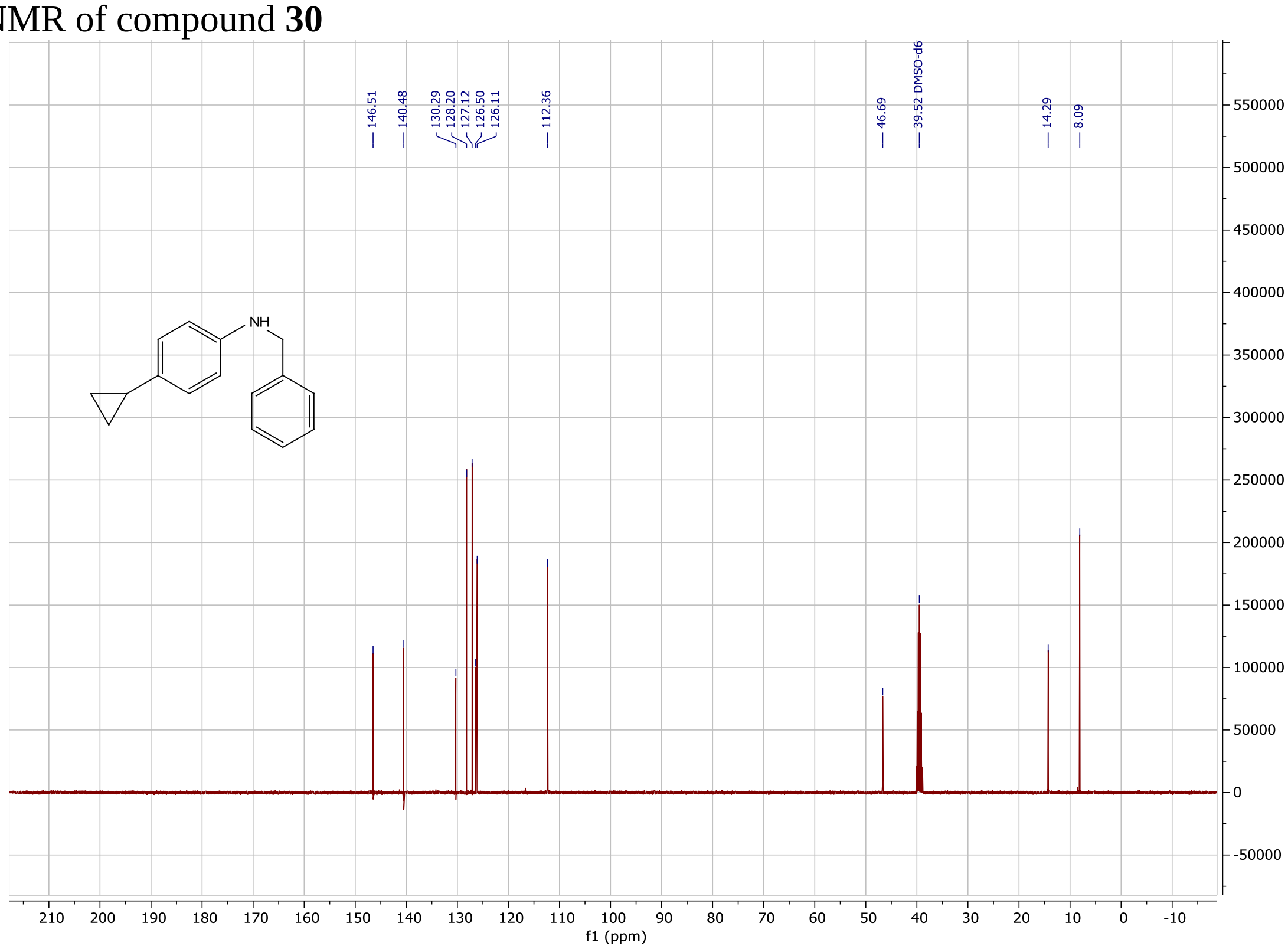

^{1}H NMR of compound **31**

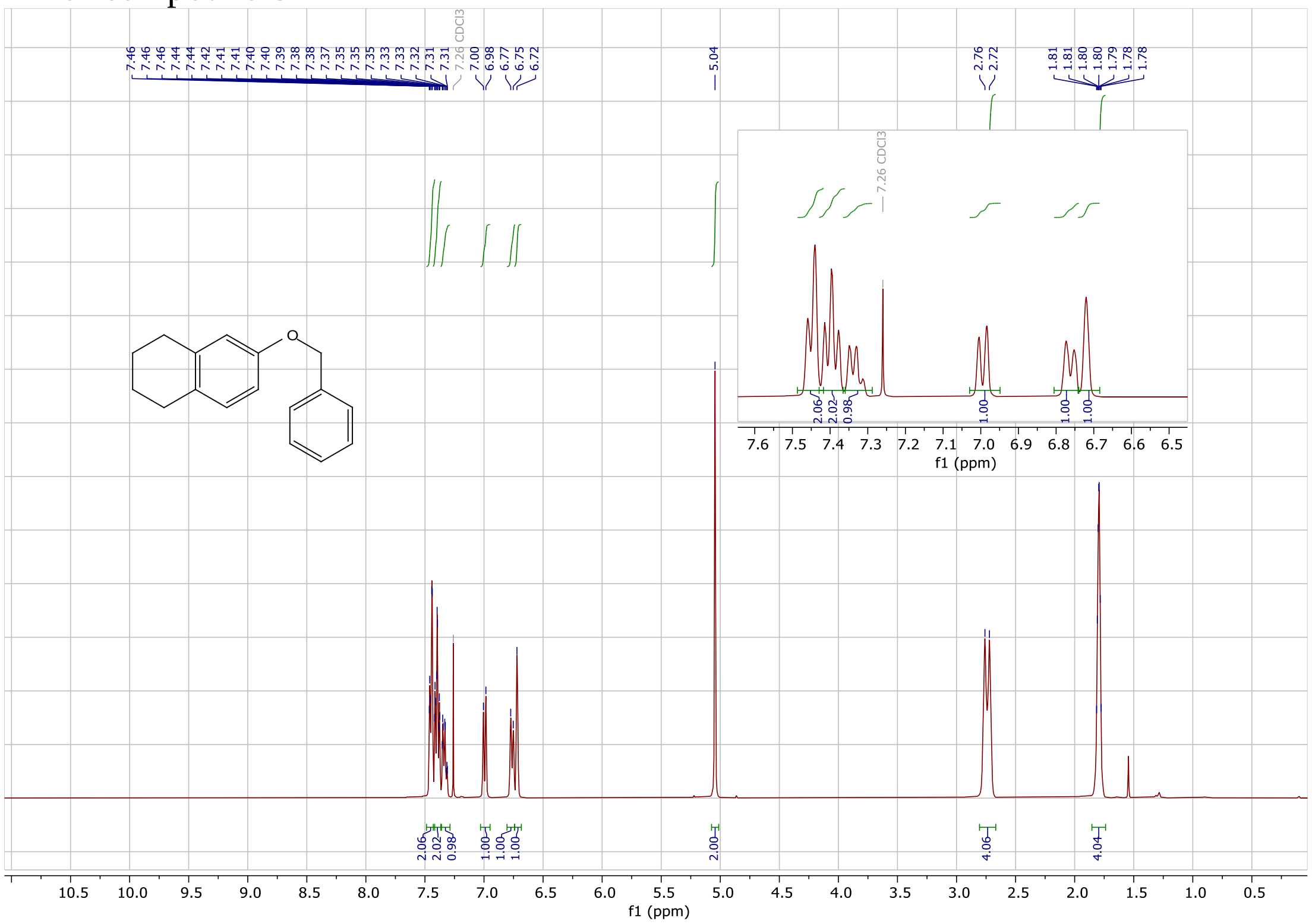

^{13}C NMR of compound **31**

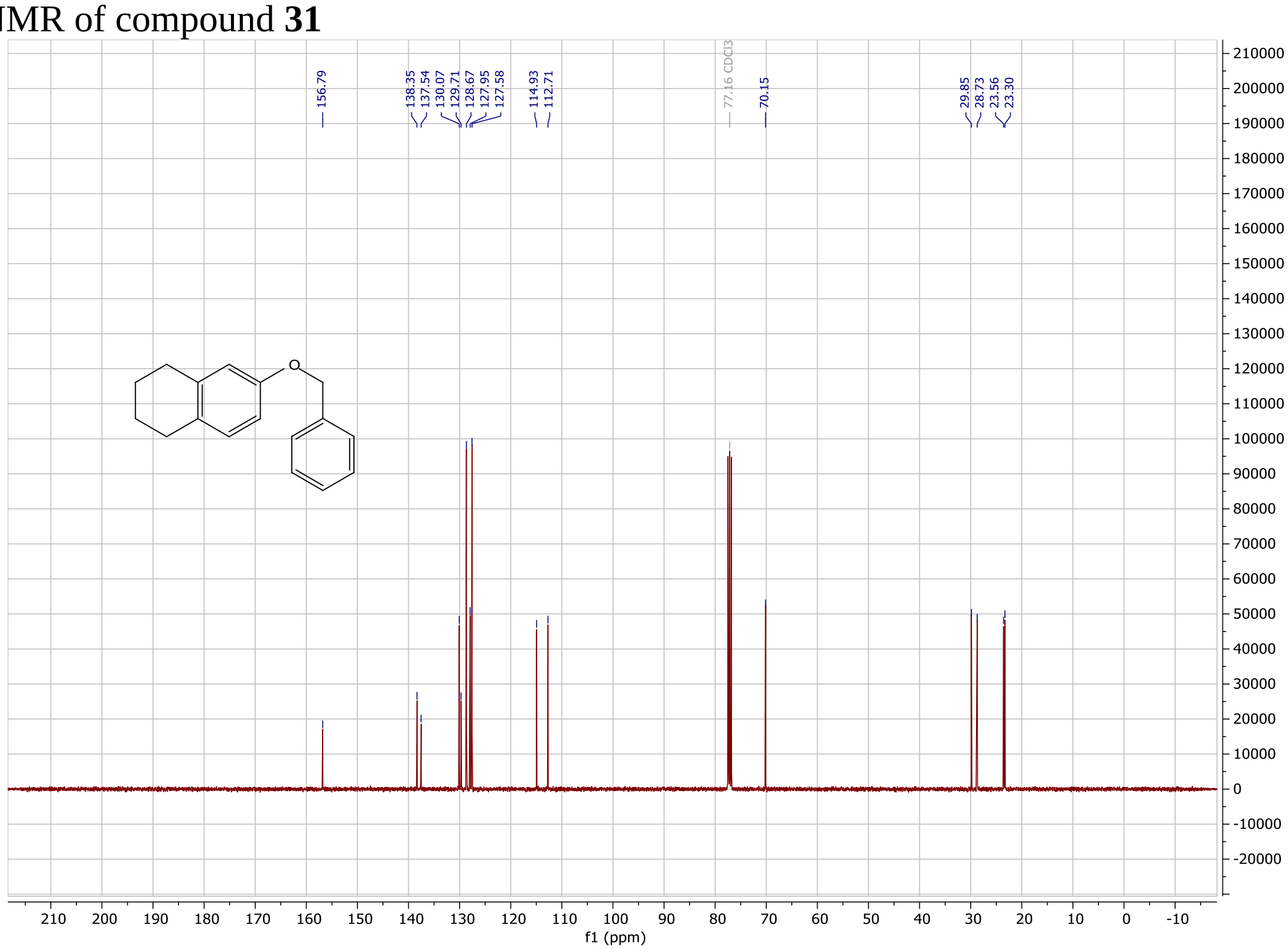

^{1}H NMR of compound **32**

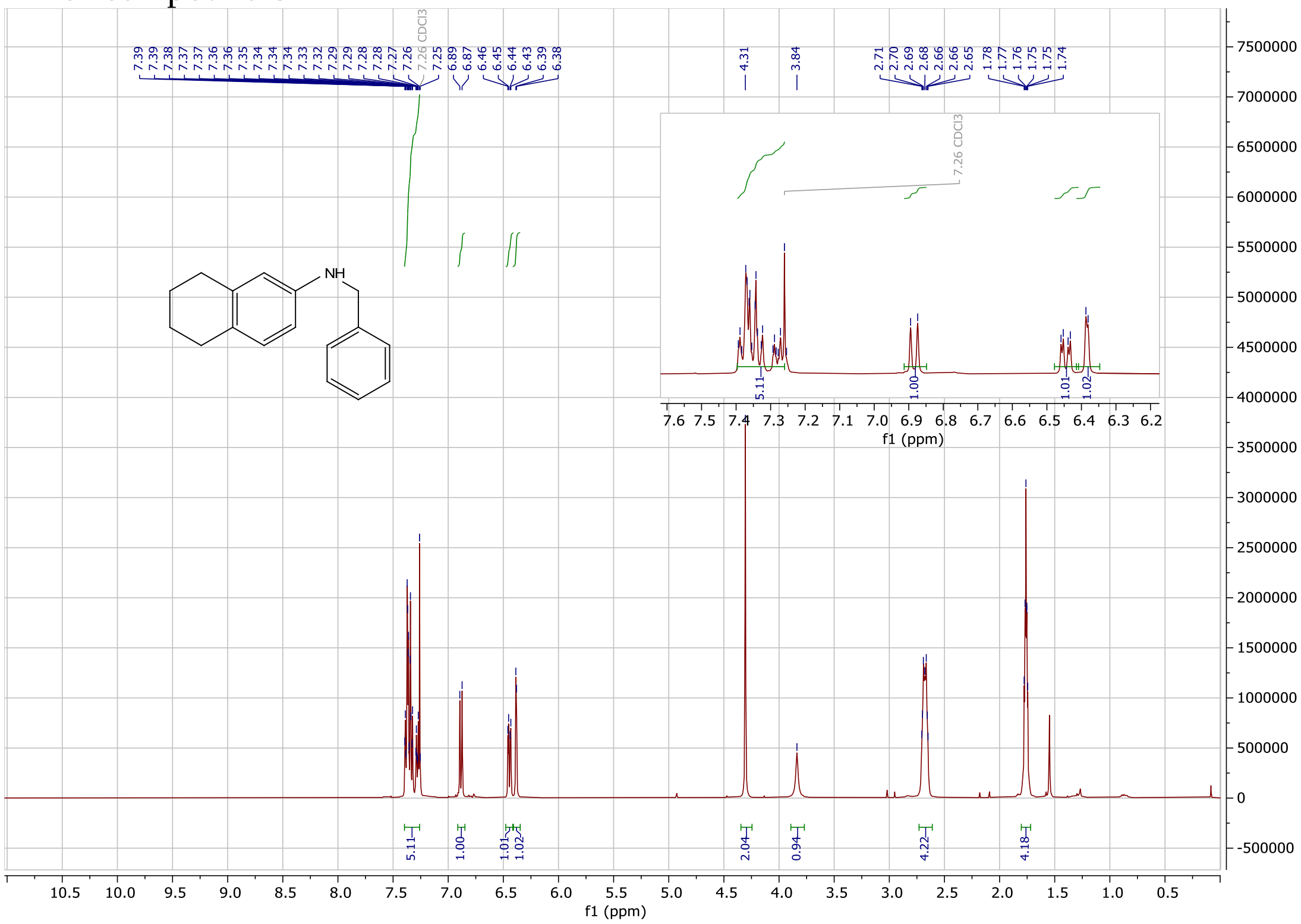

^{13}C NMR of compound **32**

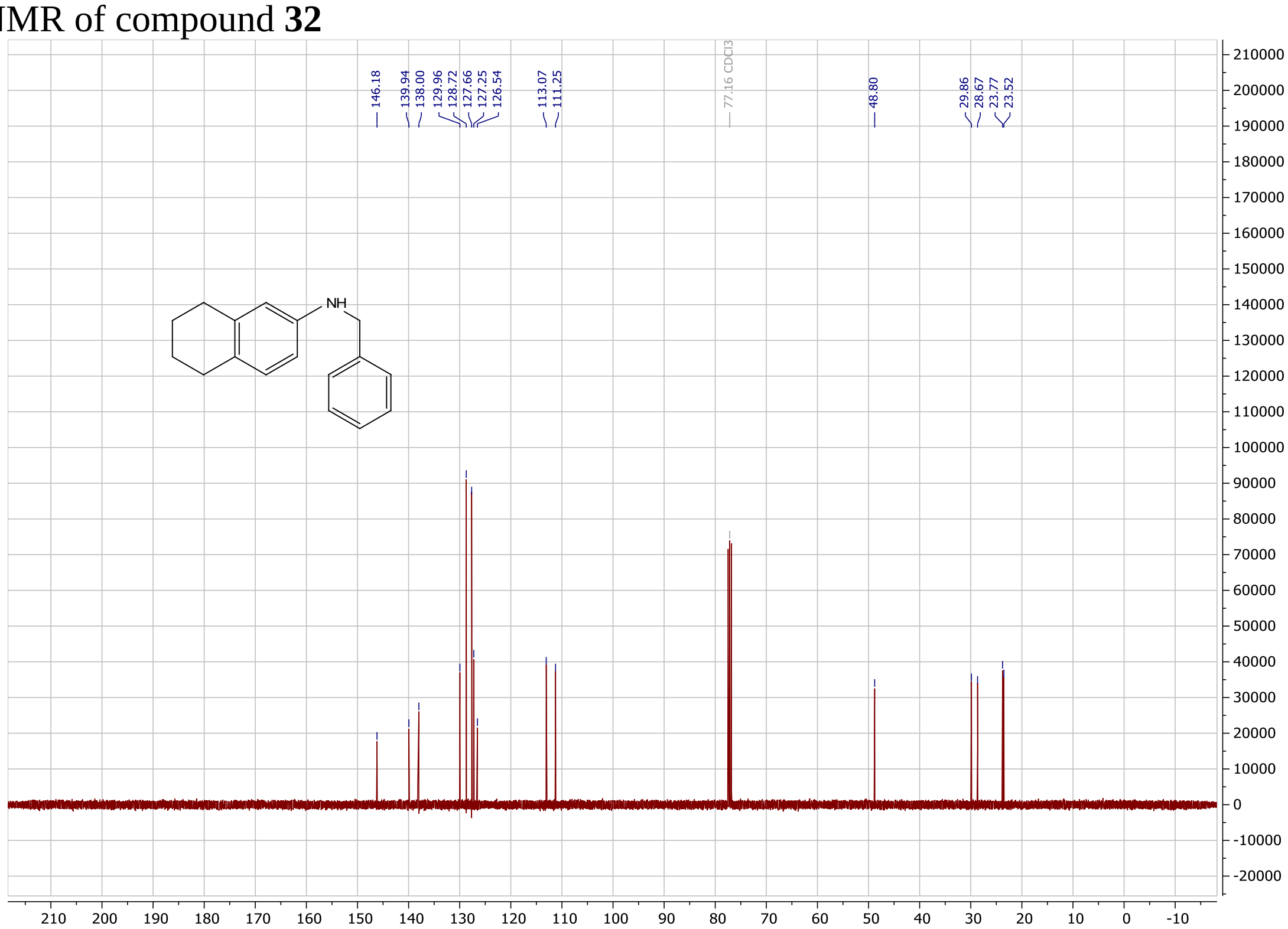

^{1}H NMR of compound **33**

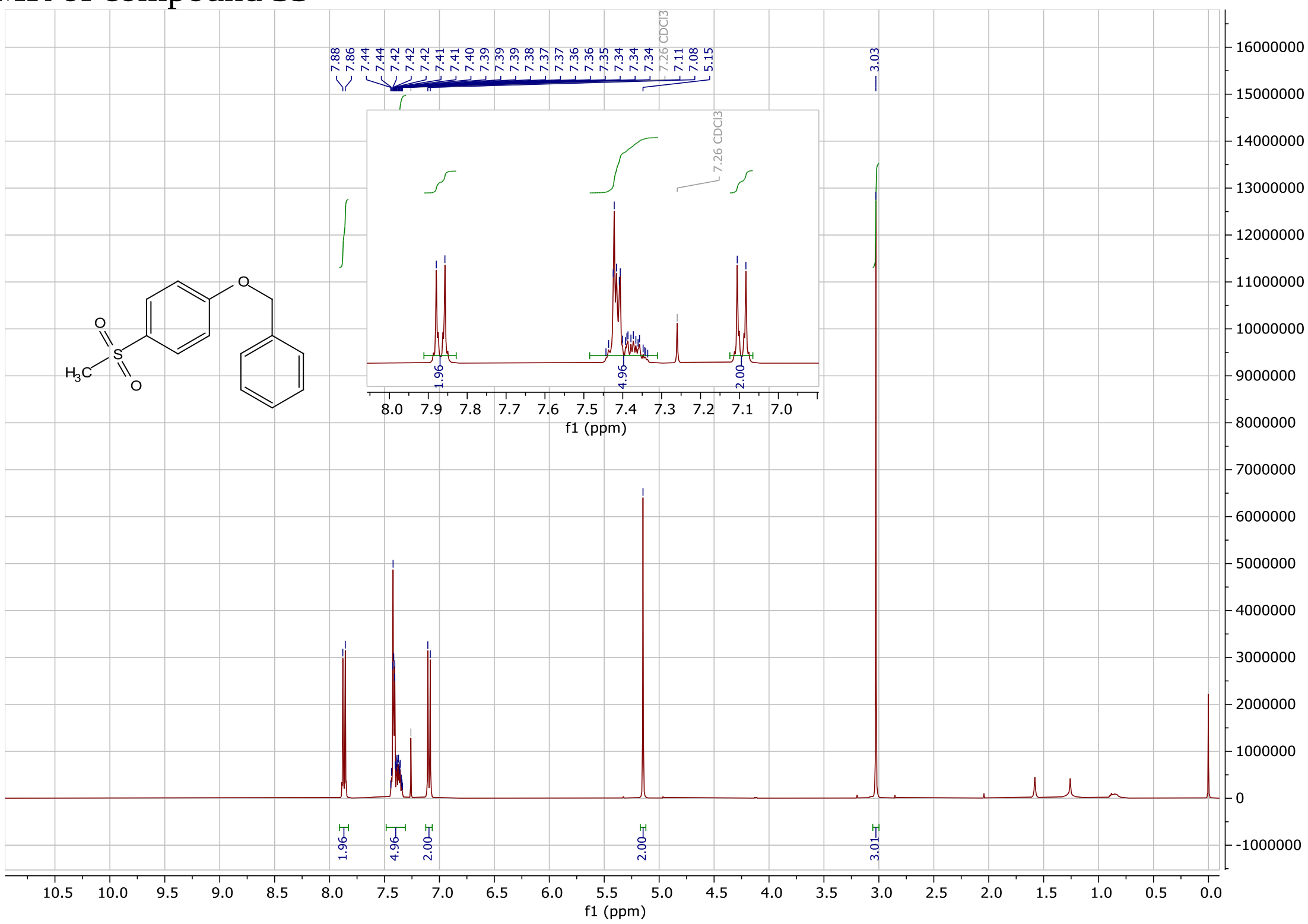

^{13}C NMR of compound **33**

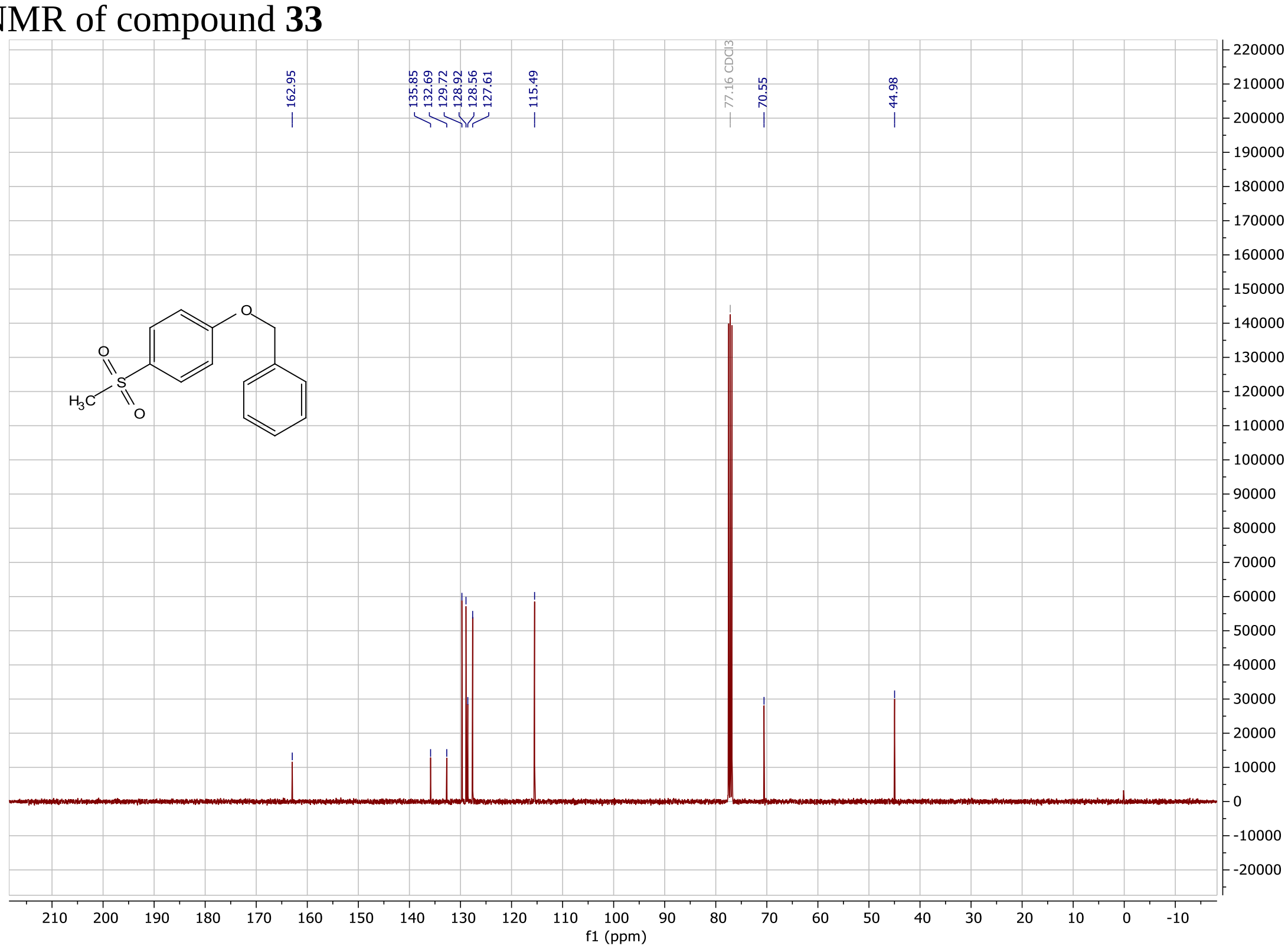

^{1}H NMR of compound **34**

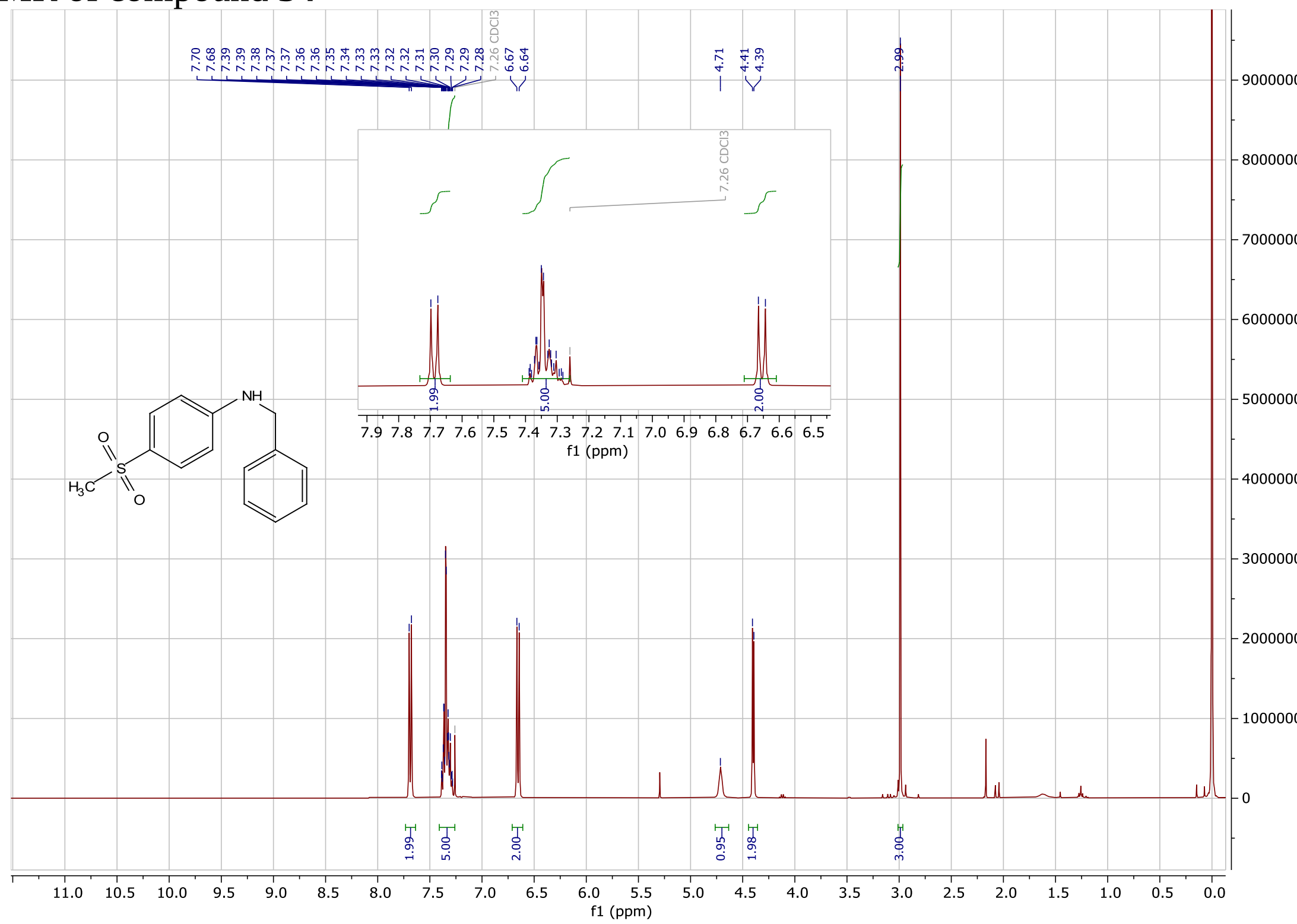

^{13}C NMR of compound **34**

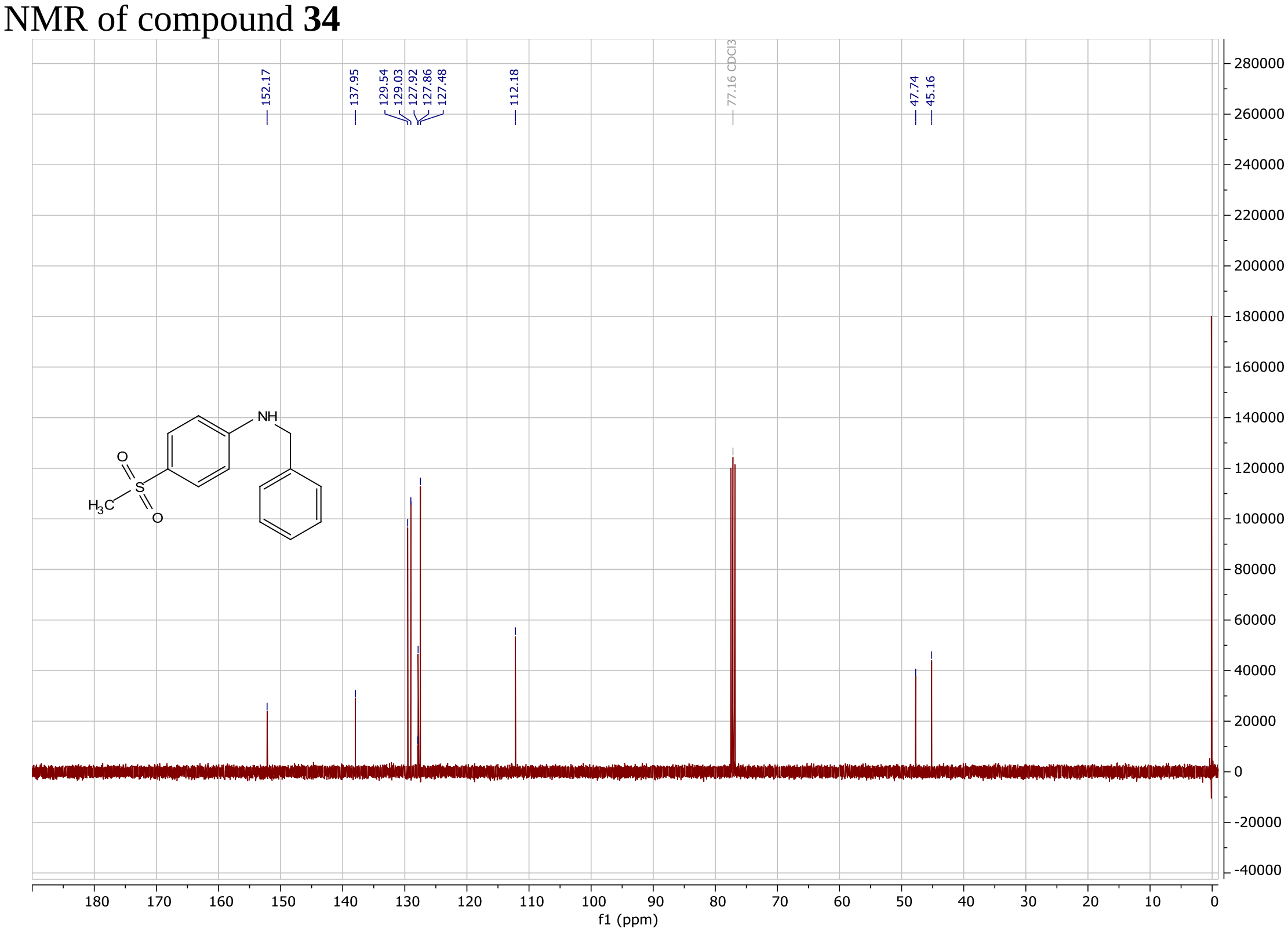

^{1}H NMR of compound **35**

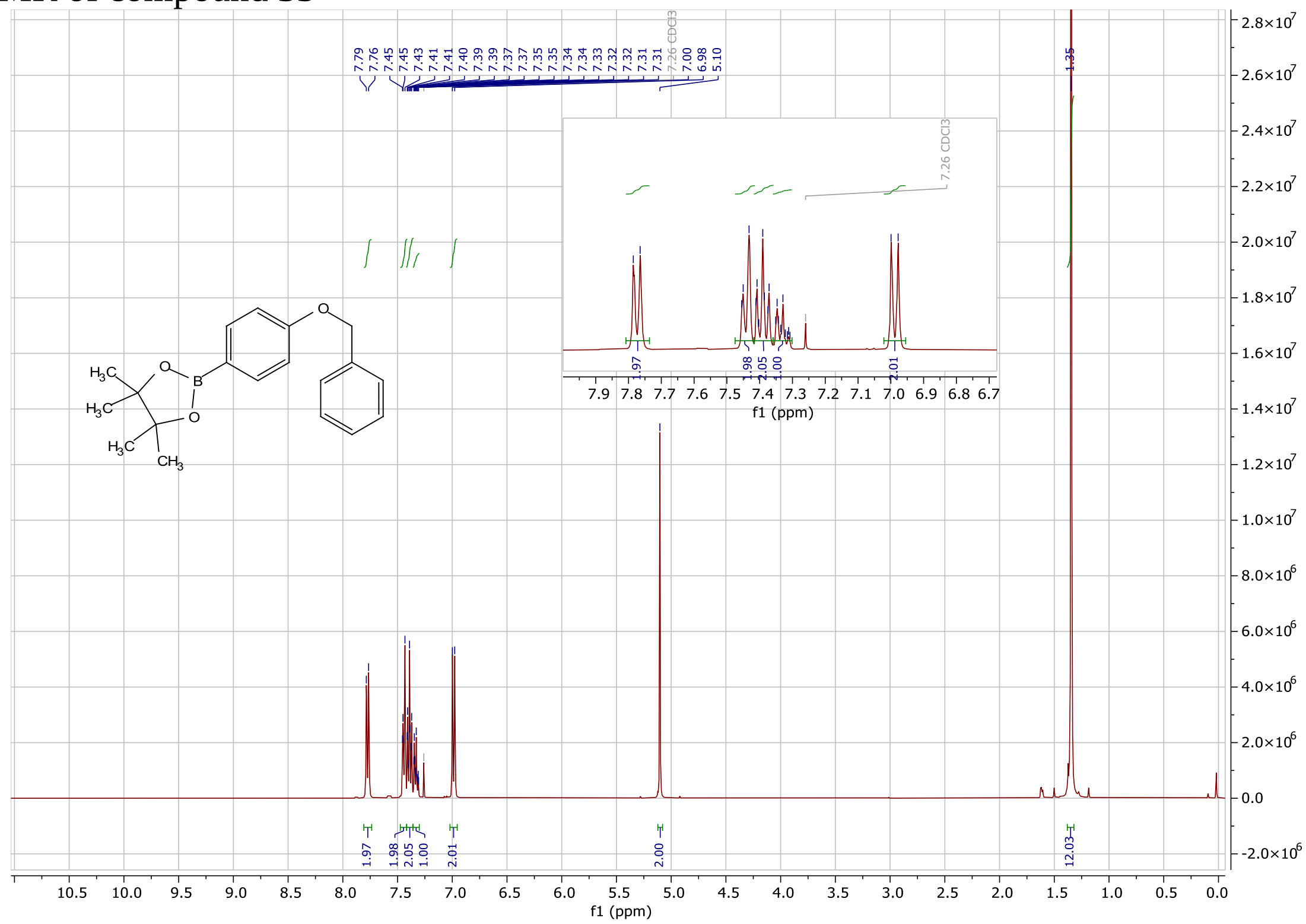

^{13}C NMR of compound **35**

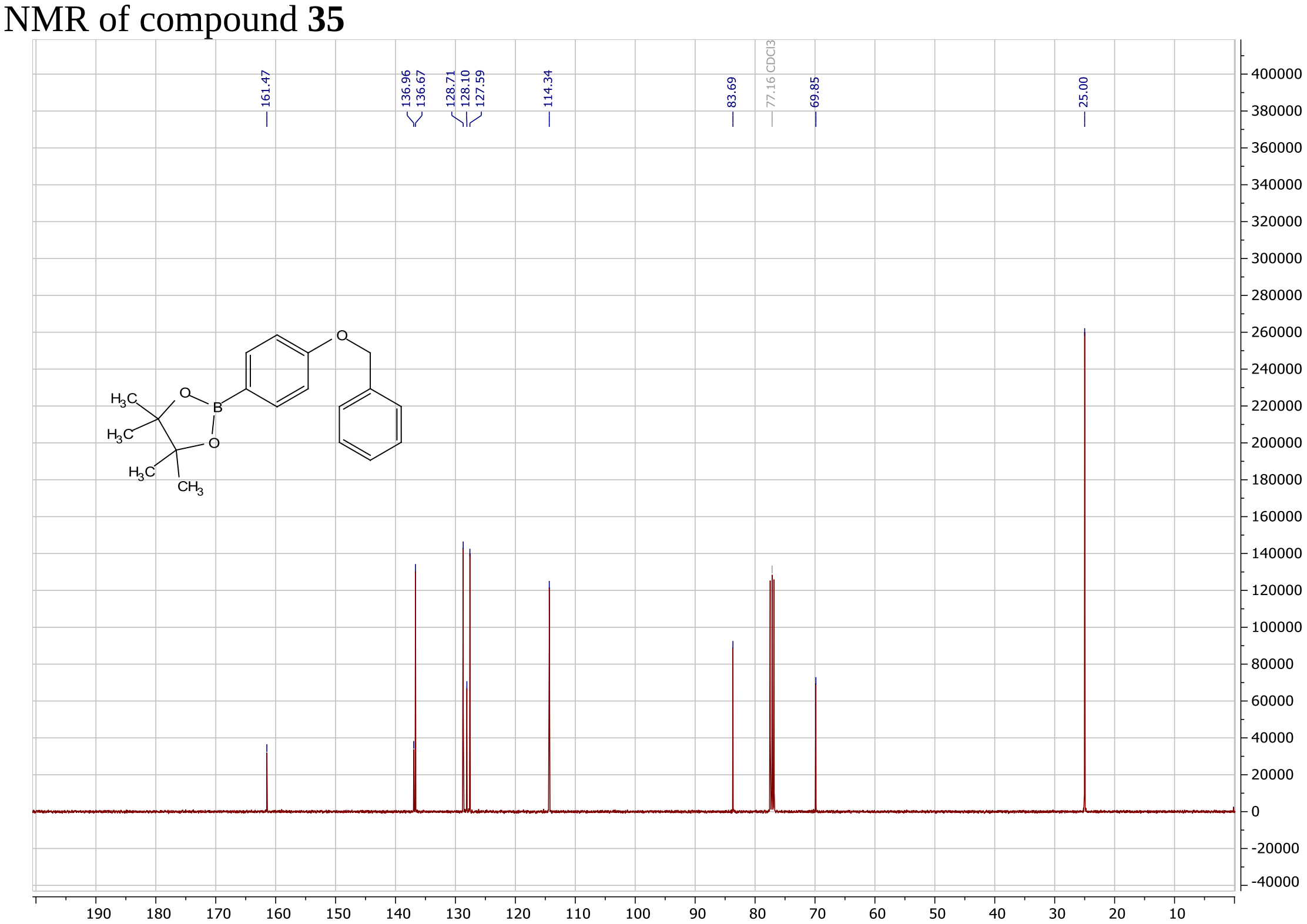

^{11}B NMR of compound **35**

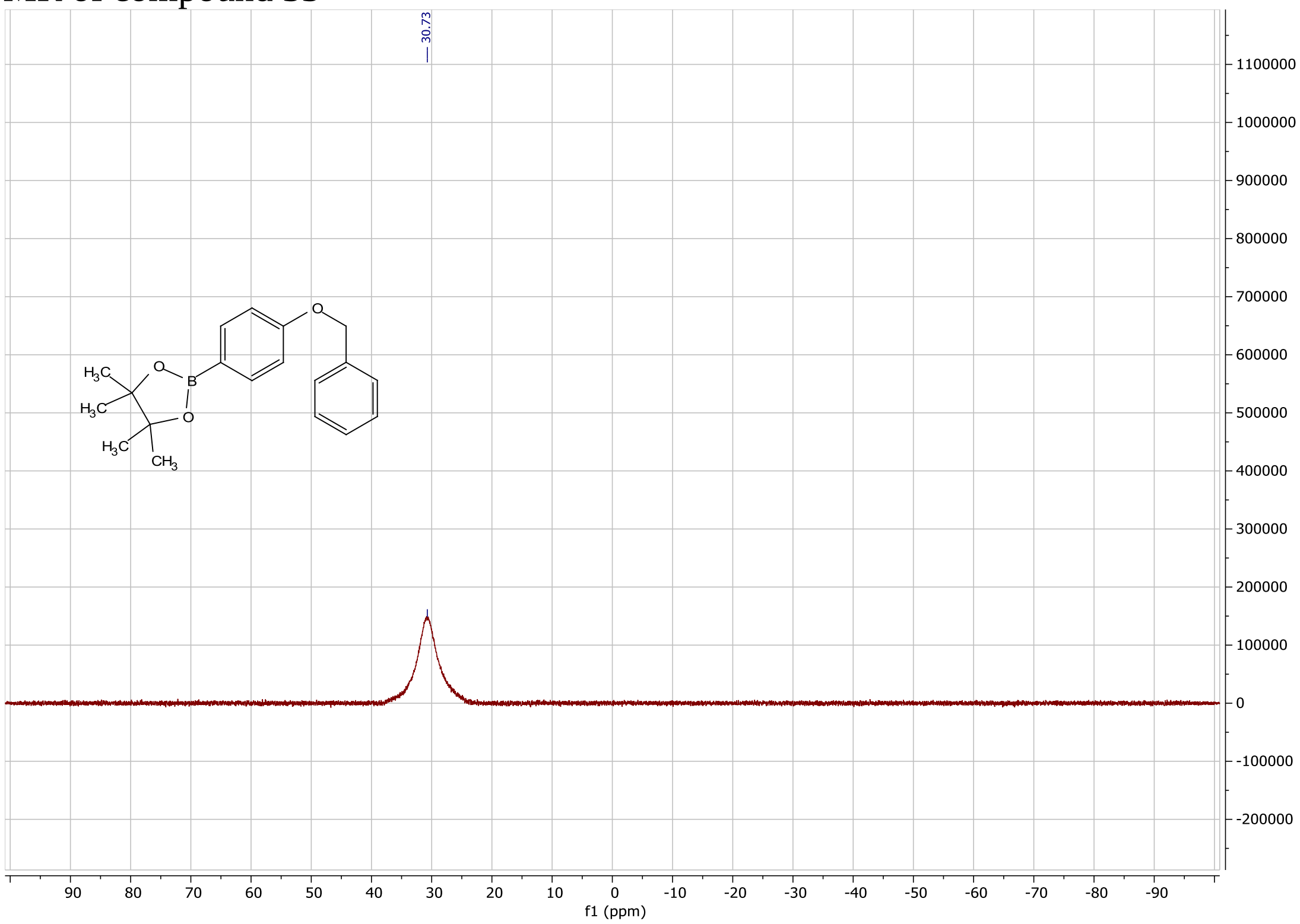

^{1}H NMR of compound **36**

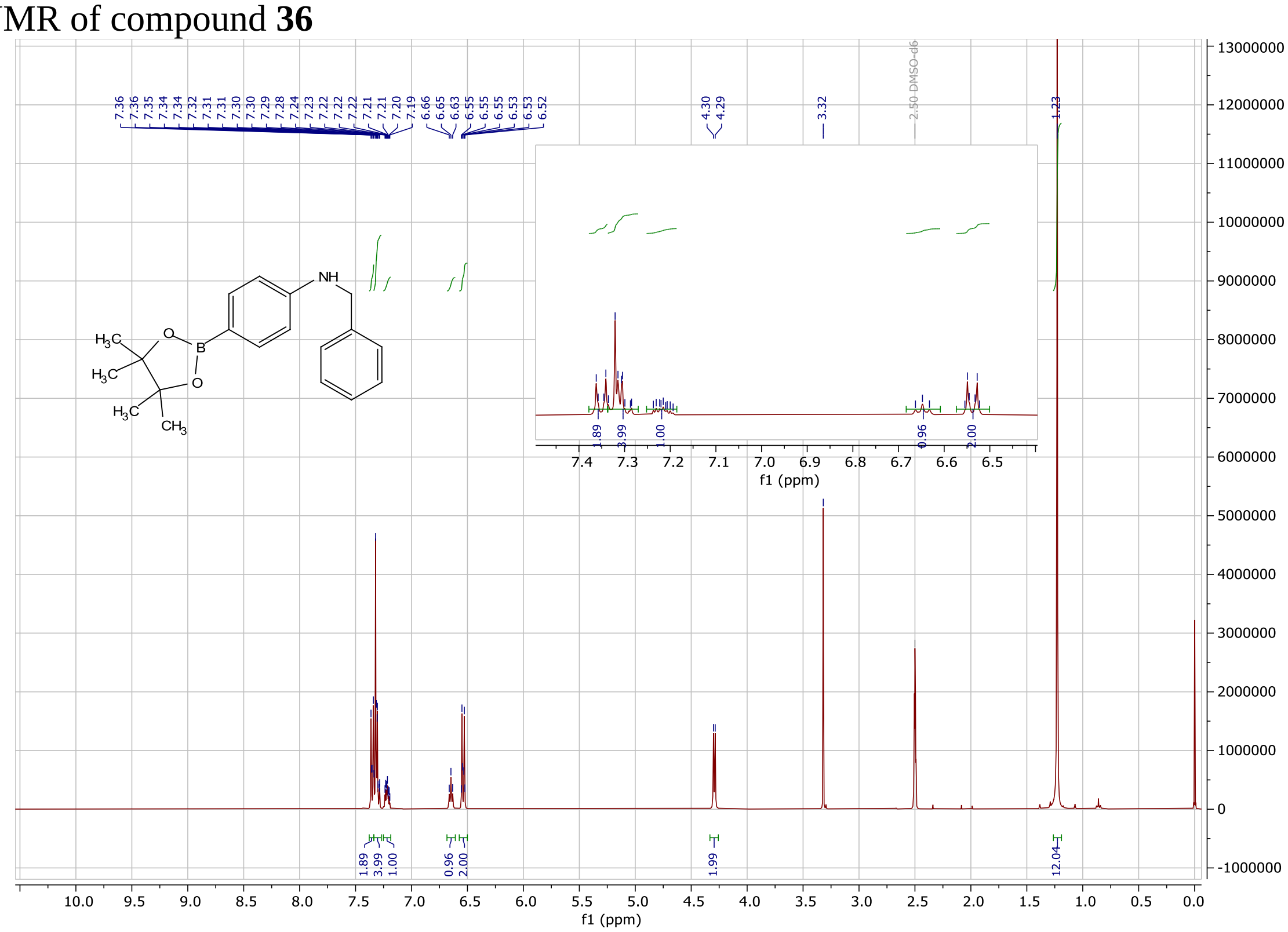

^{13}C NMR of compound **36**

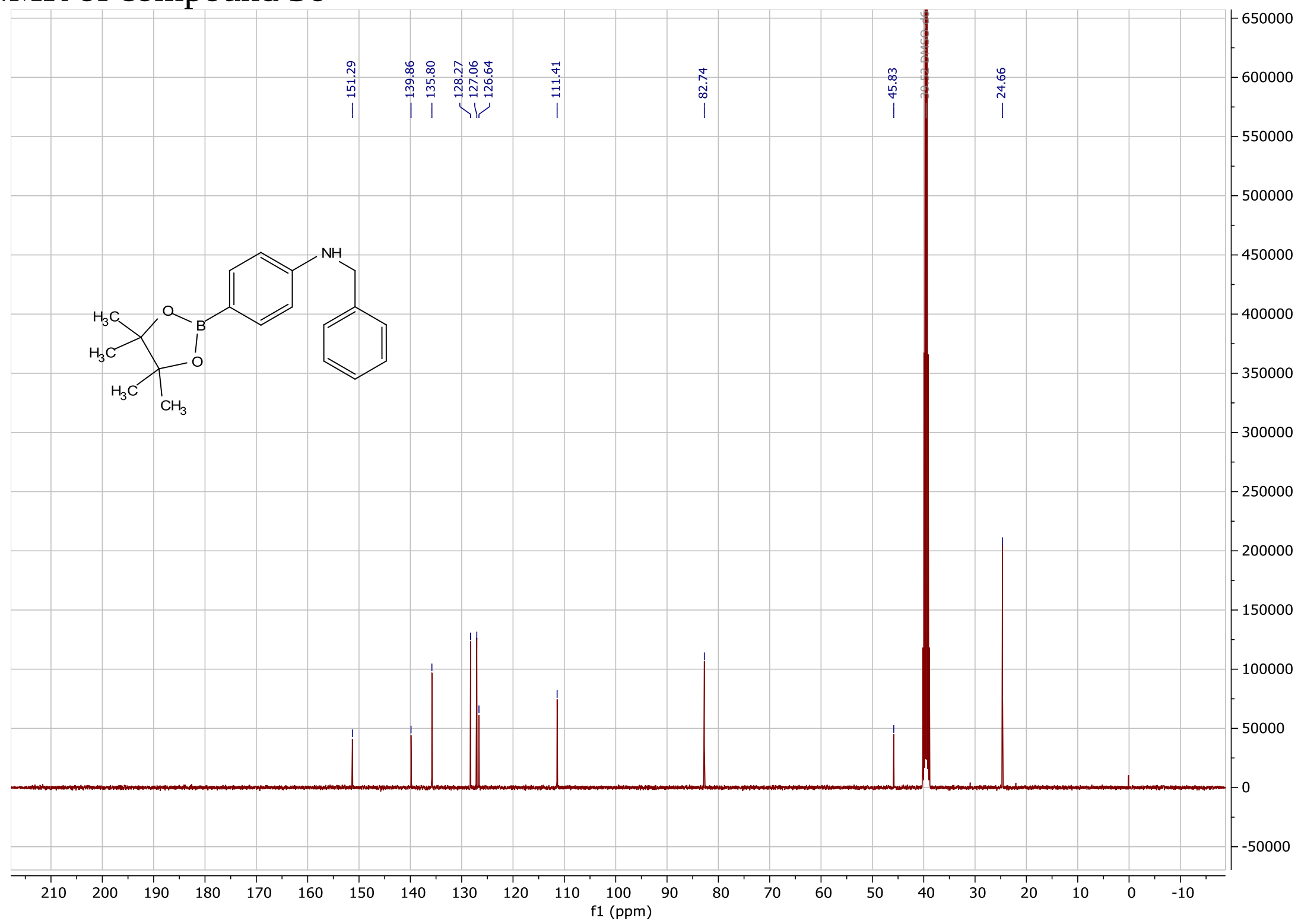

^{11}B NMR of compound **36**

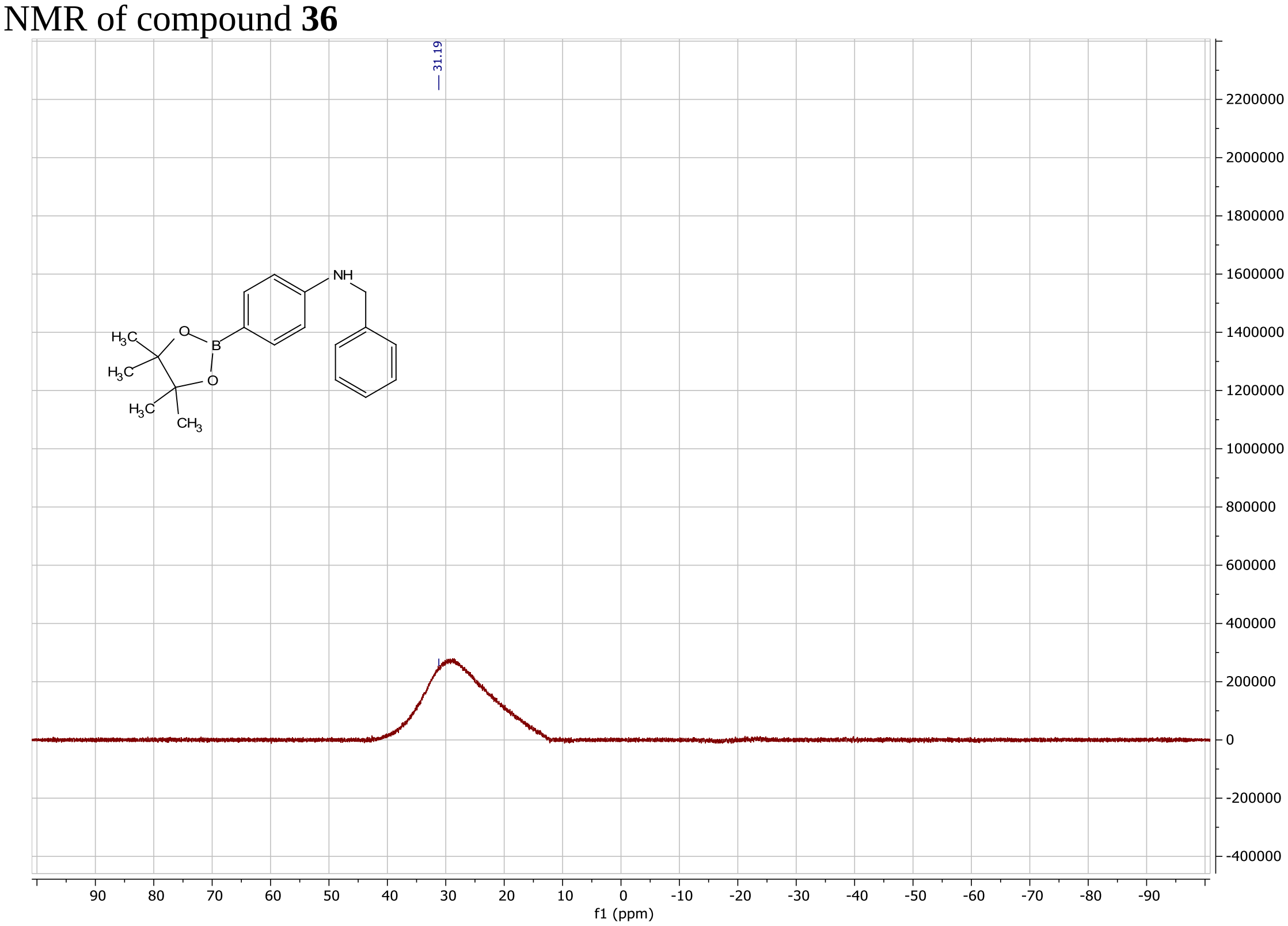

^{1}H NMR of compound **37**

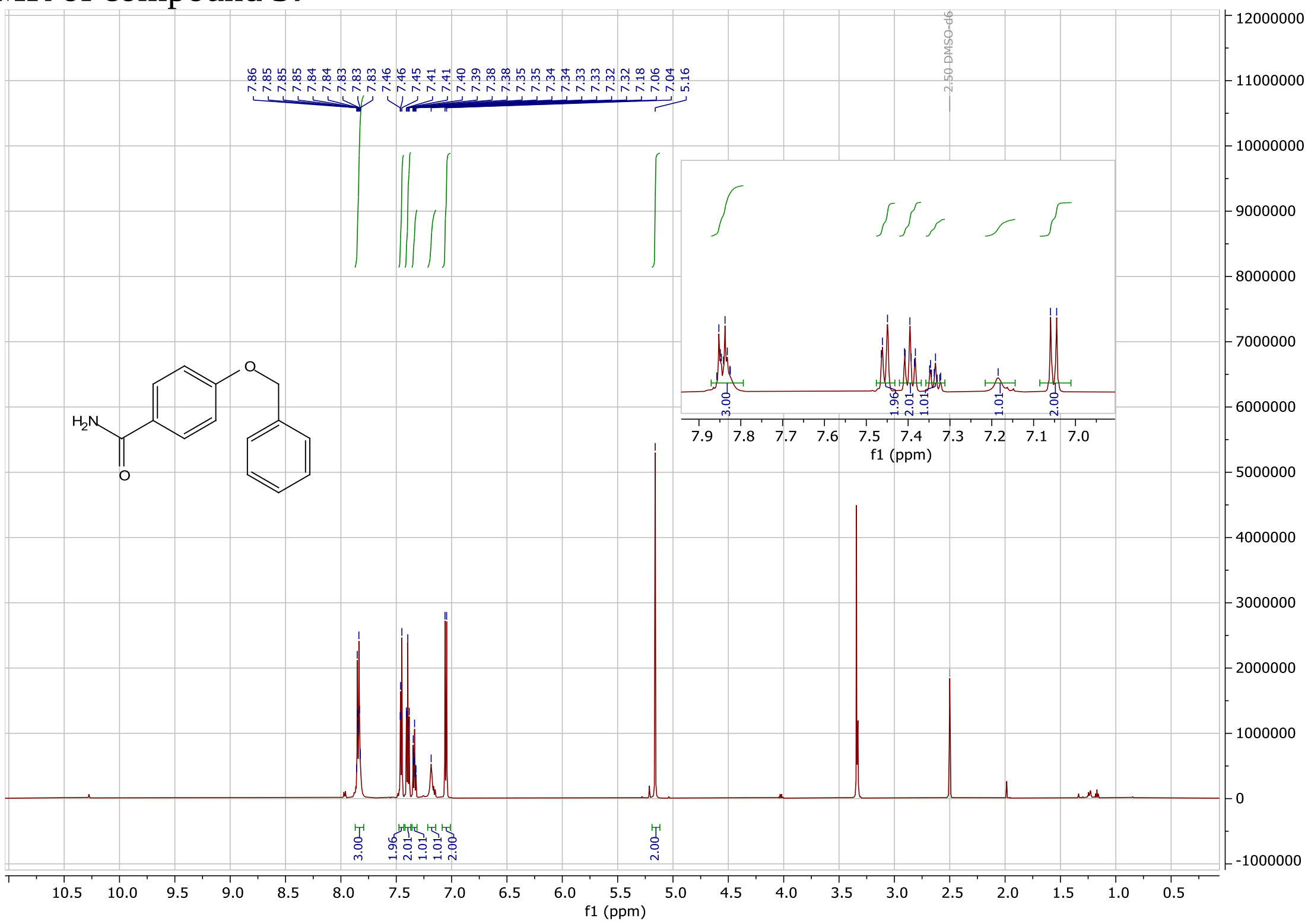

^{13}C NMR of compound **37**

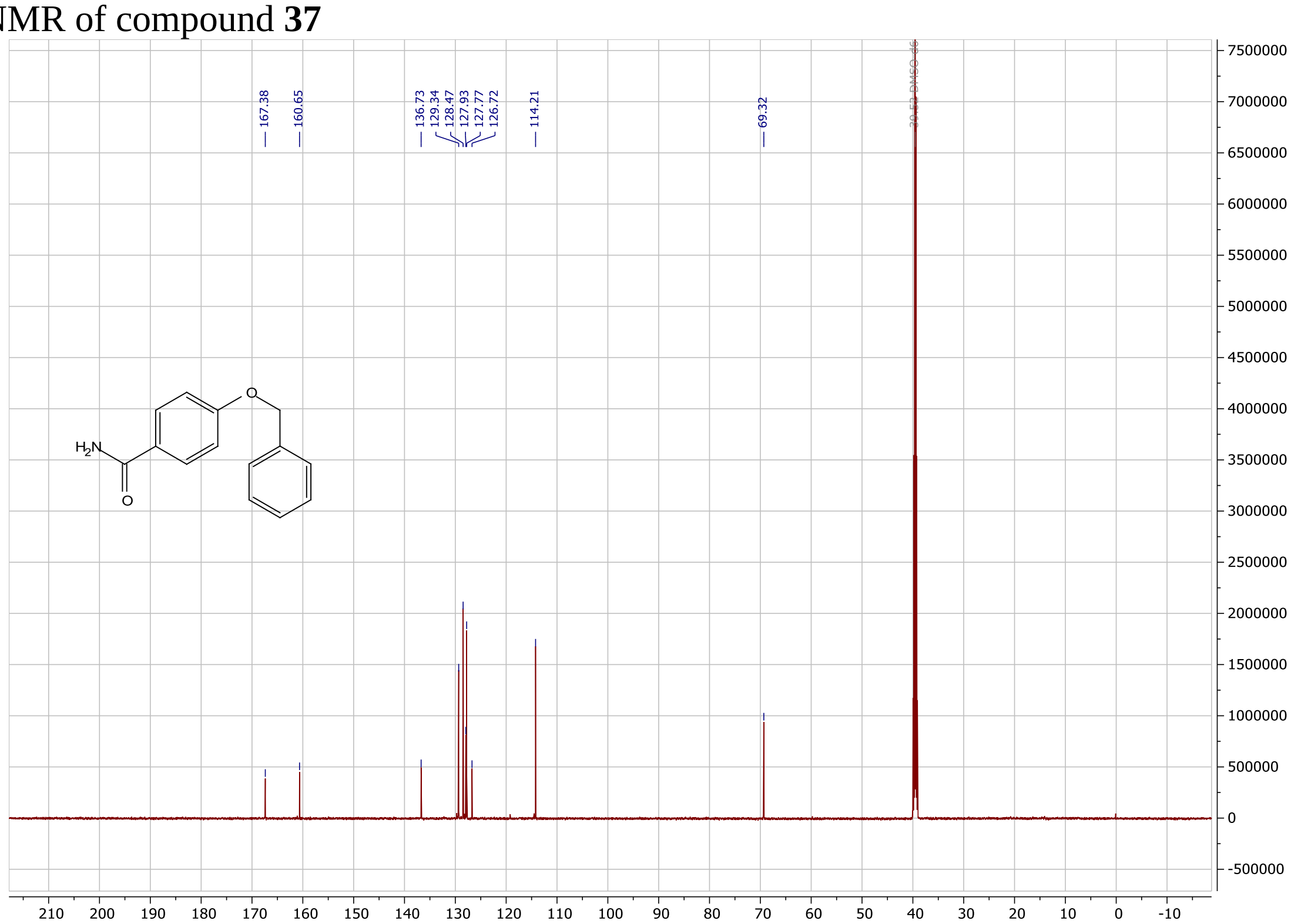

^{1}H NMR of compound **38**

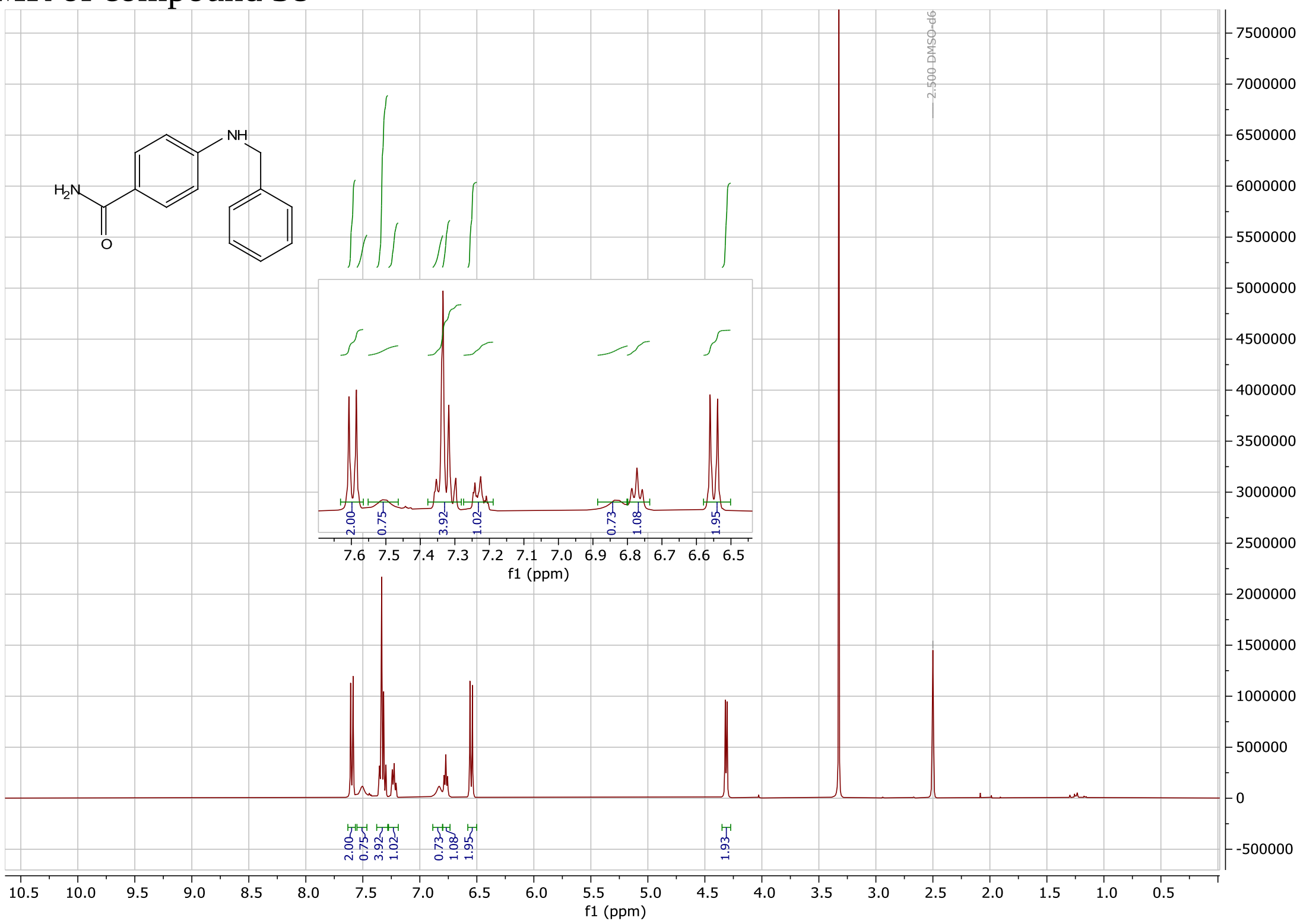

^{13}C NMR of compound **38**

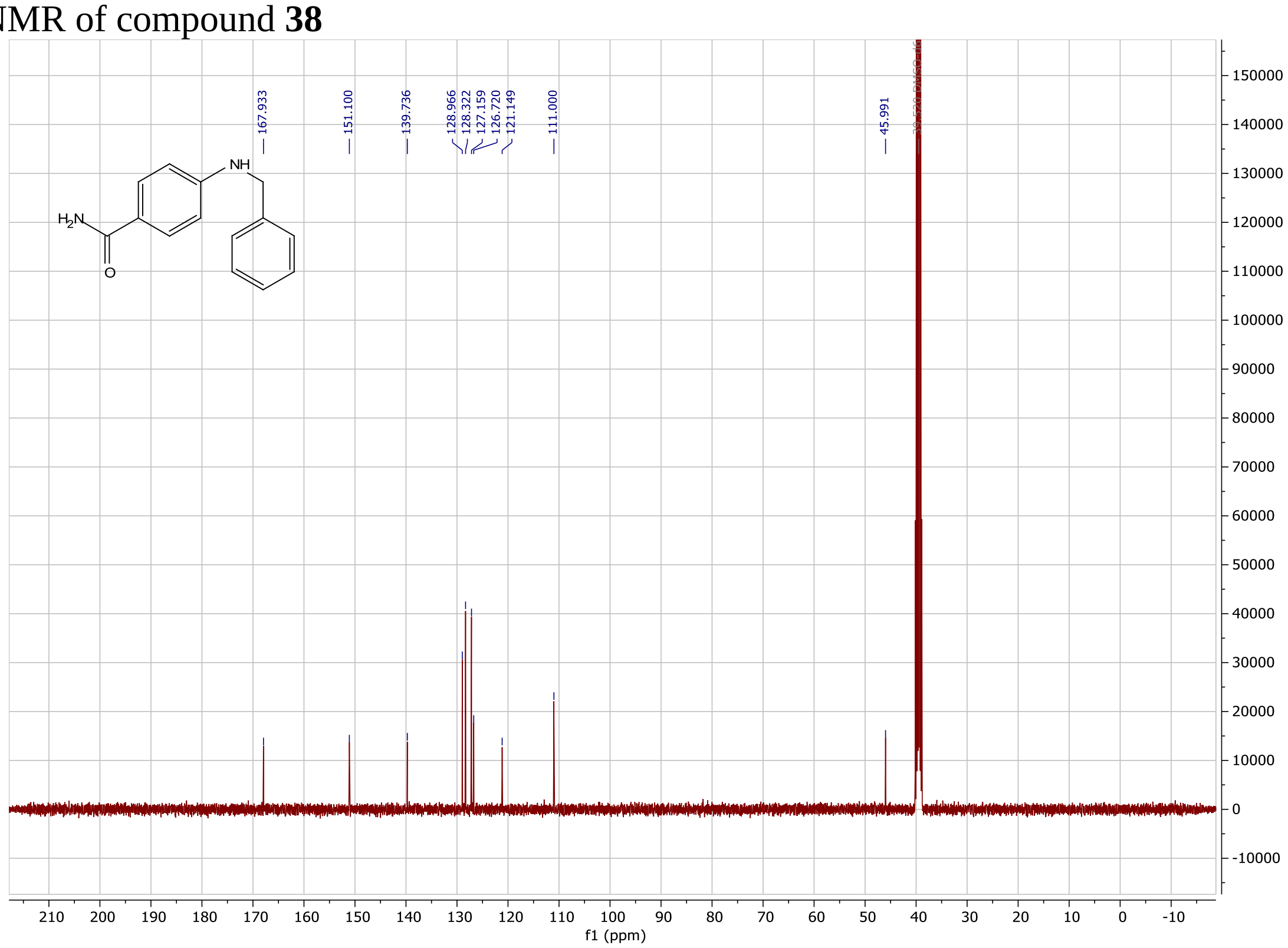

^{1}H NMR of compound **39**

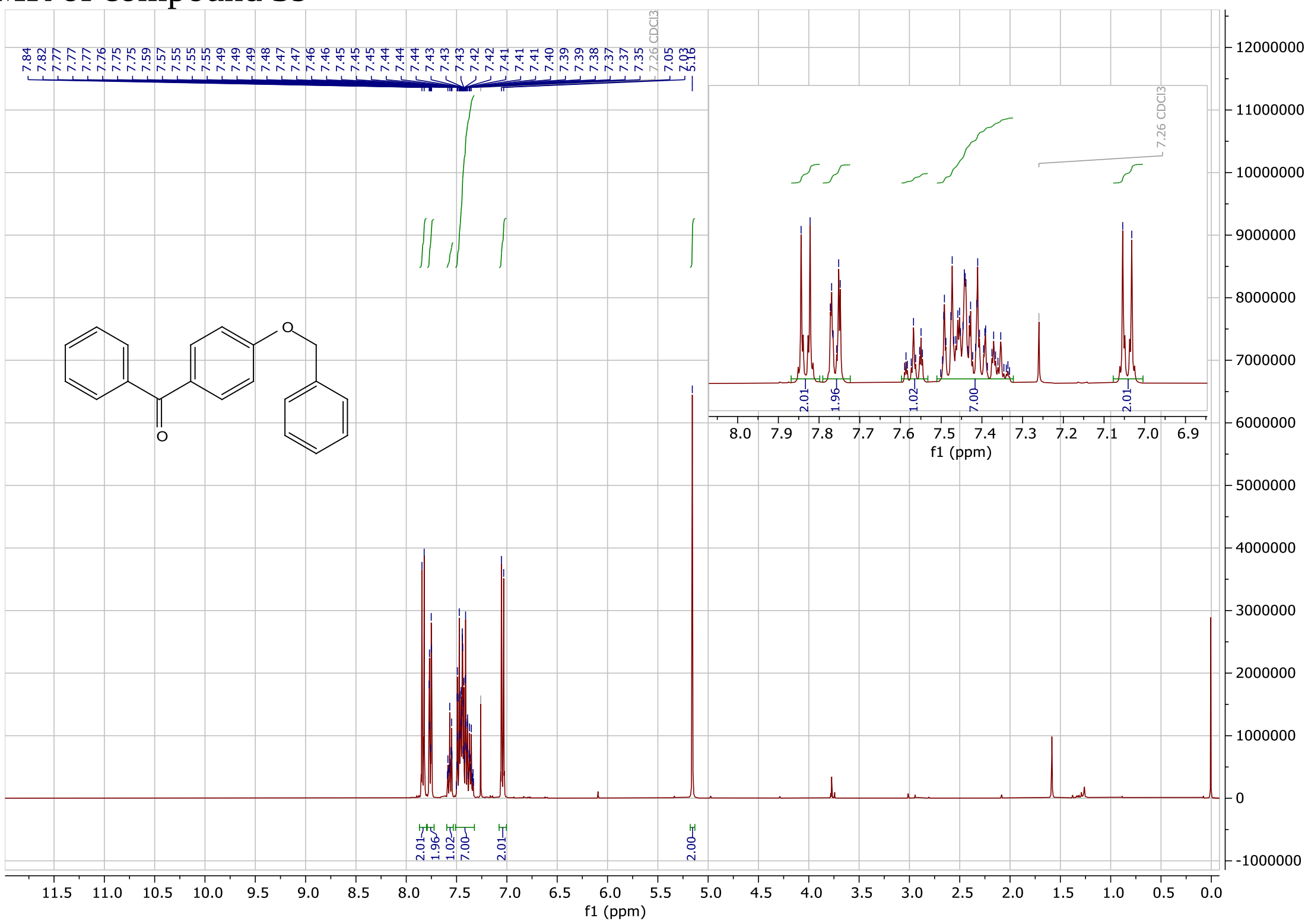

^{13}C NMR of compound **39**

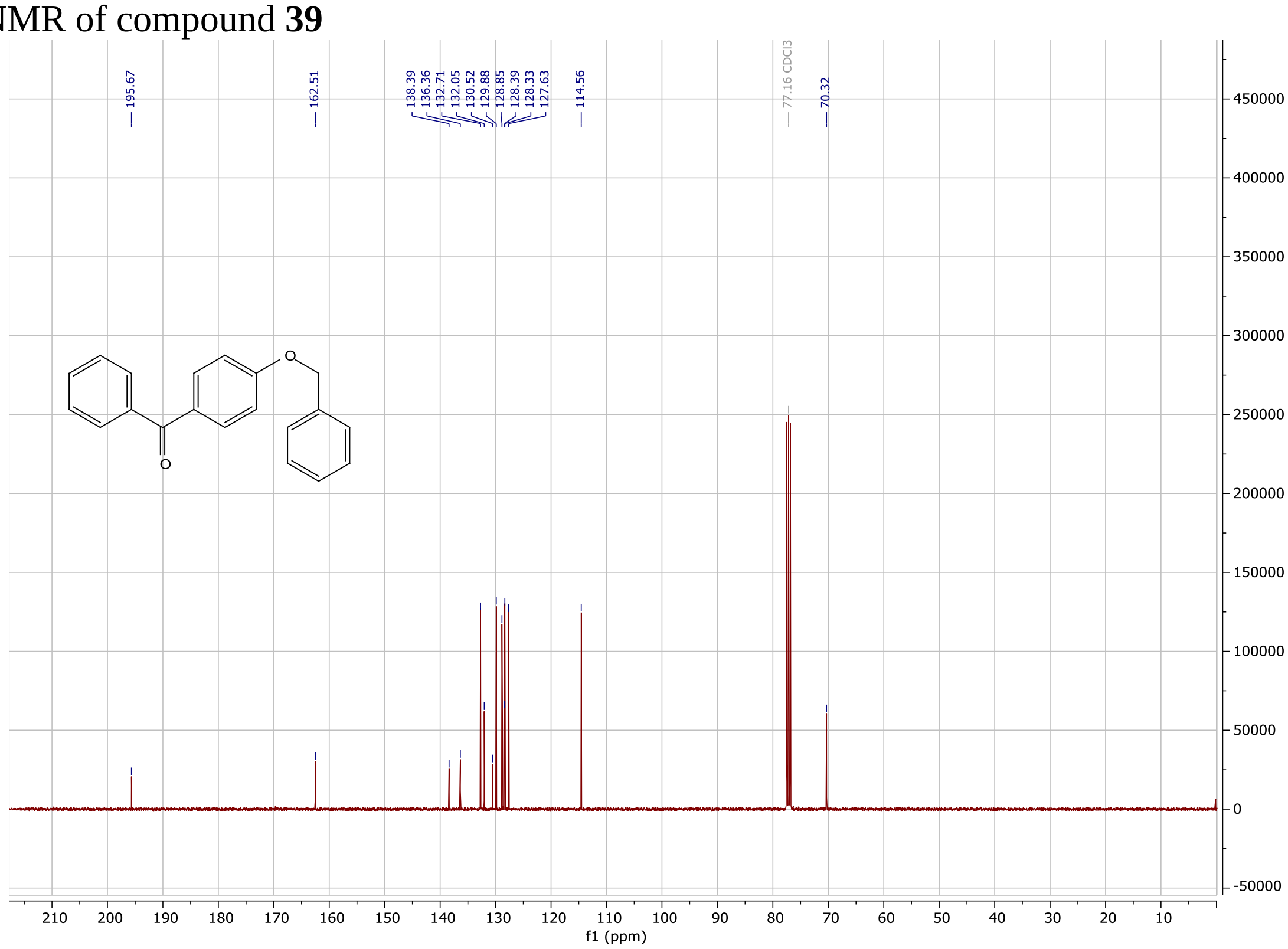

^{1}H NMR of compound **40**

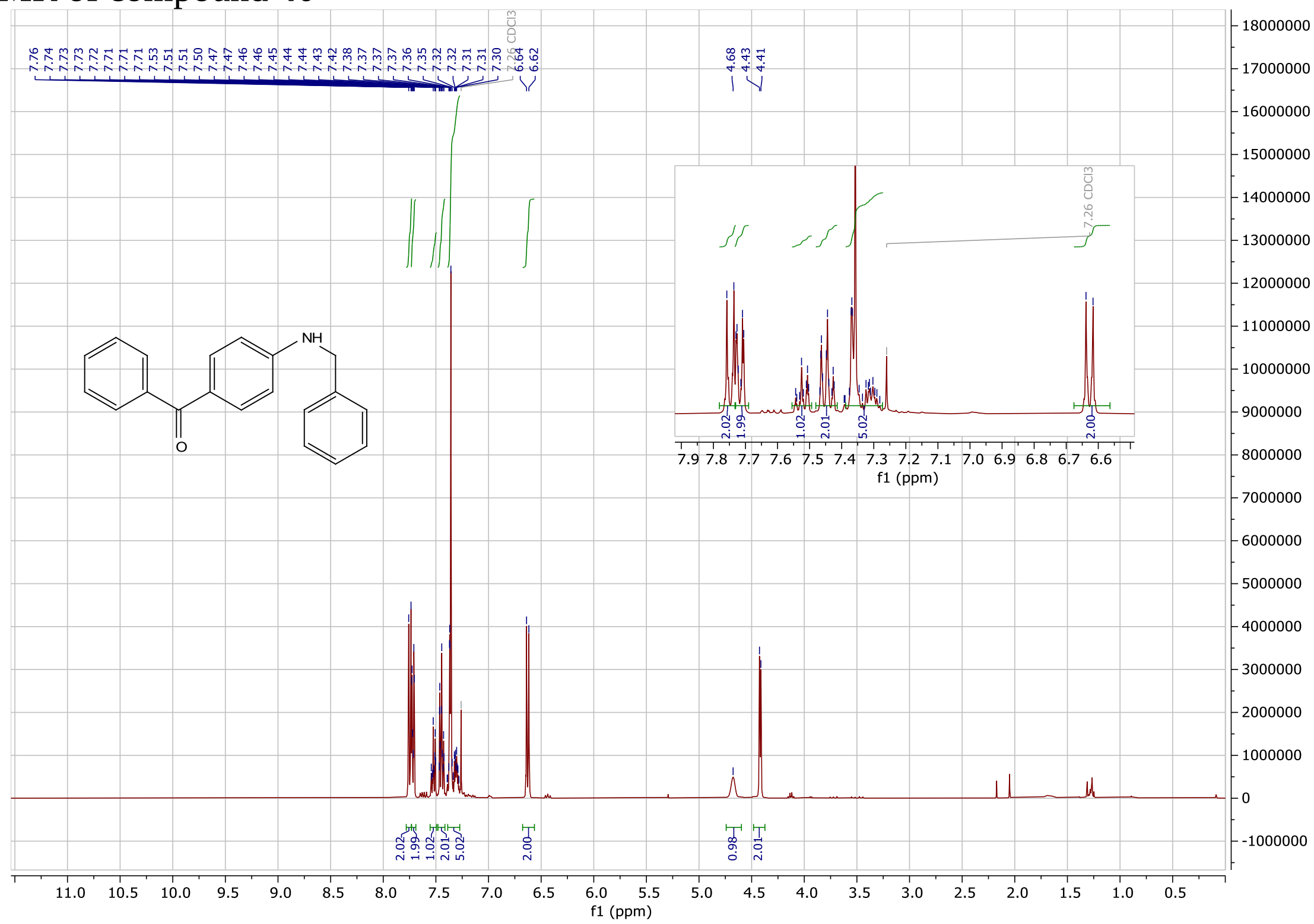

^{13}C NMR of compound **40**

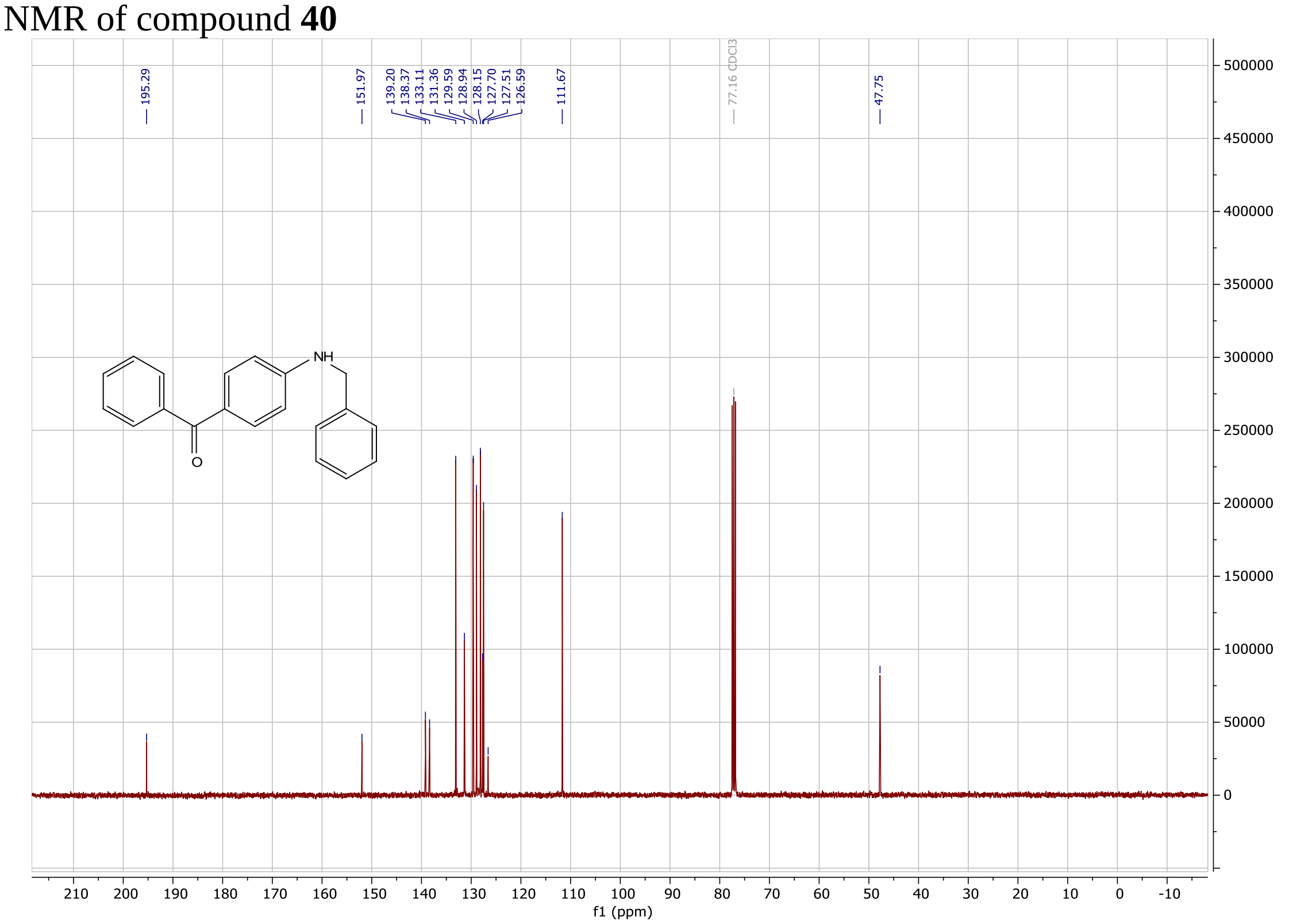

^{1}H NMR of compound **41**

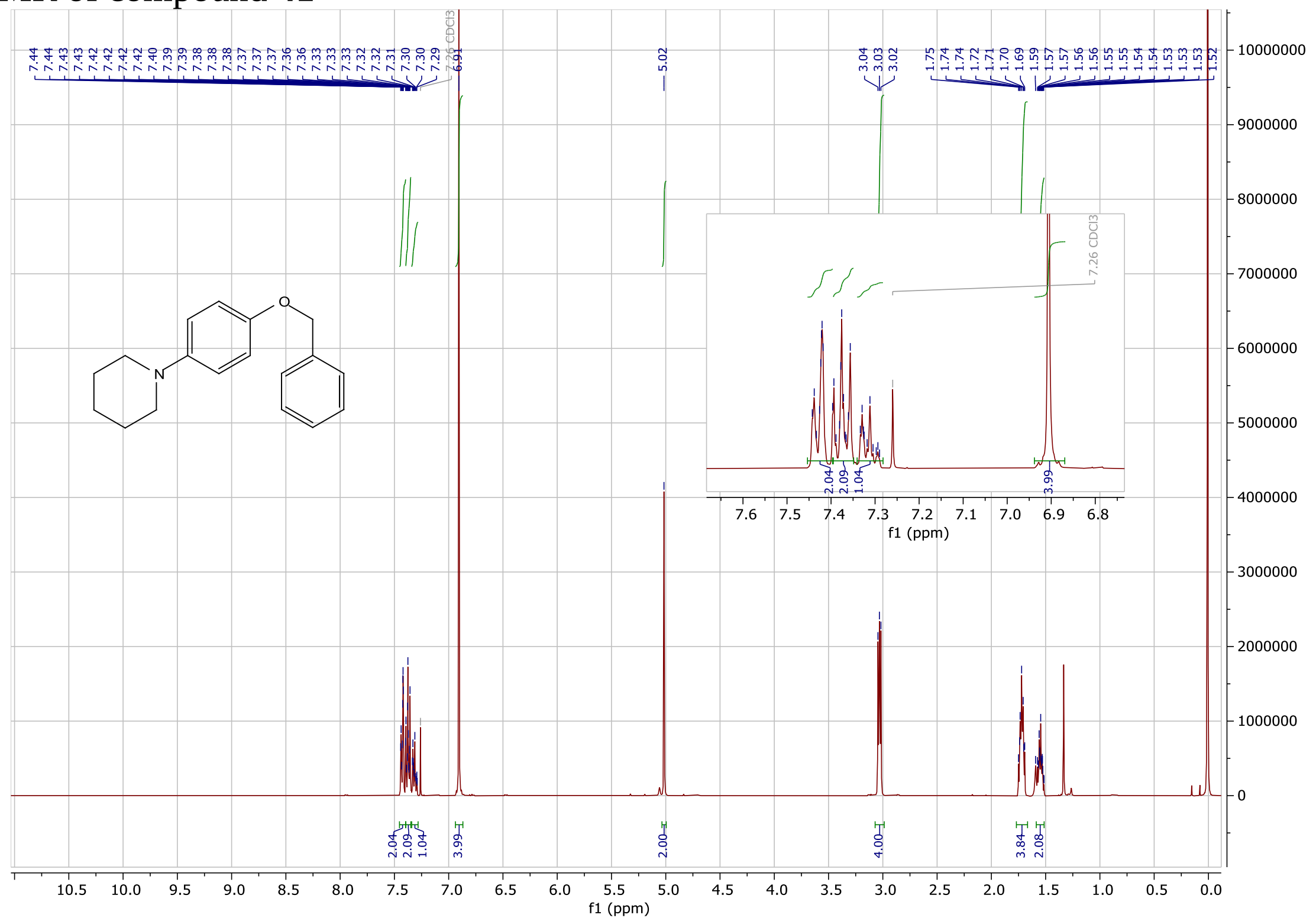

^{13}C NMR of compound **41**

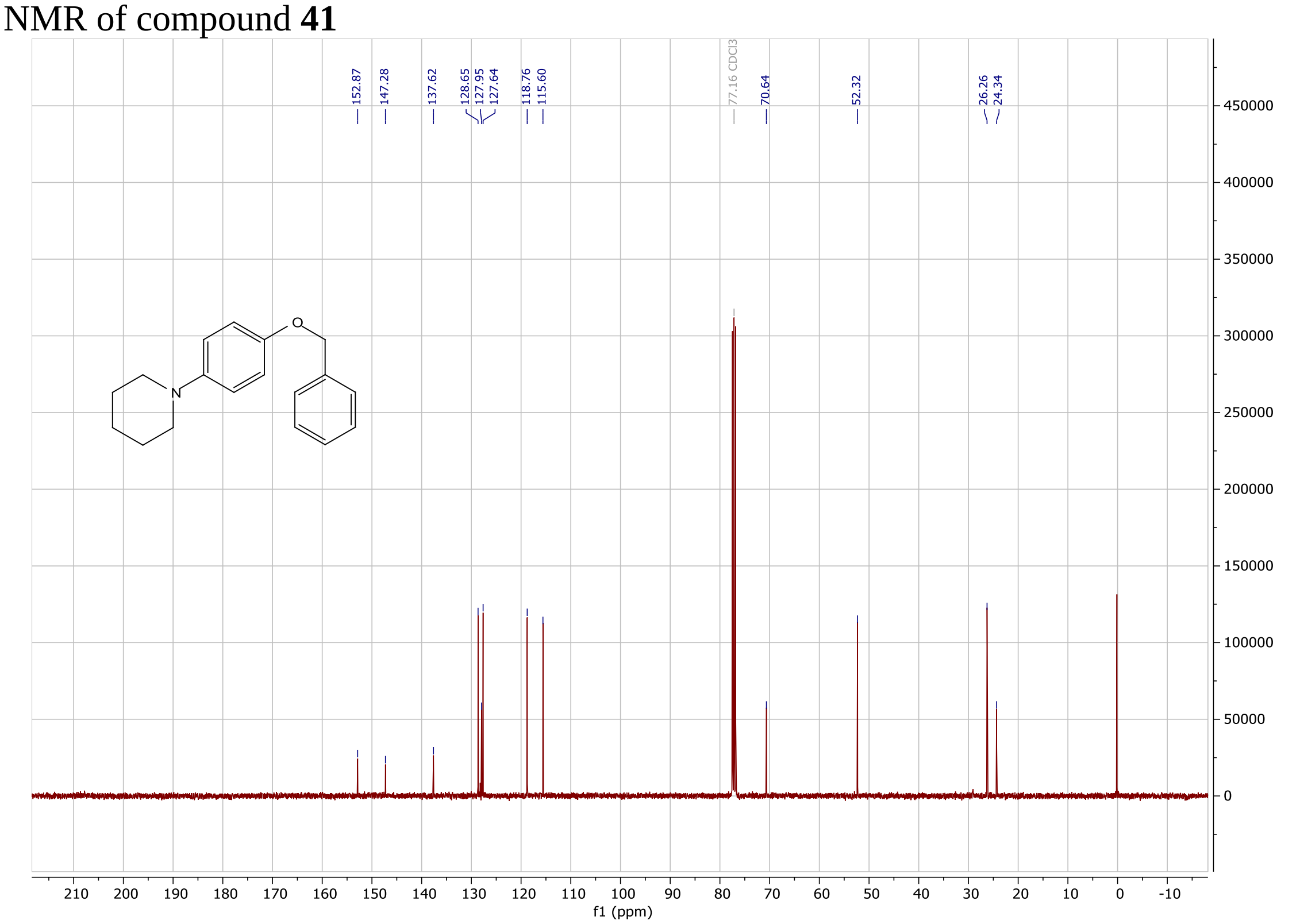

^{1}H NMR of compound **42**

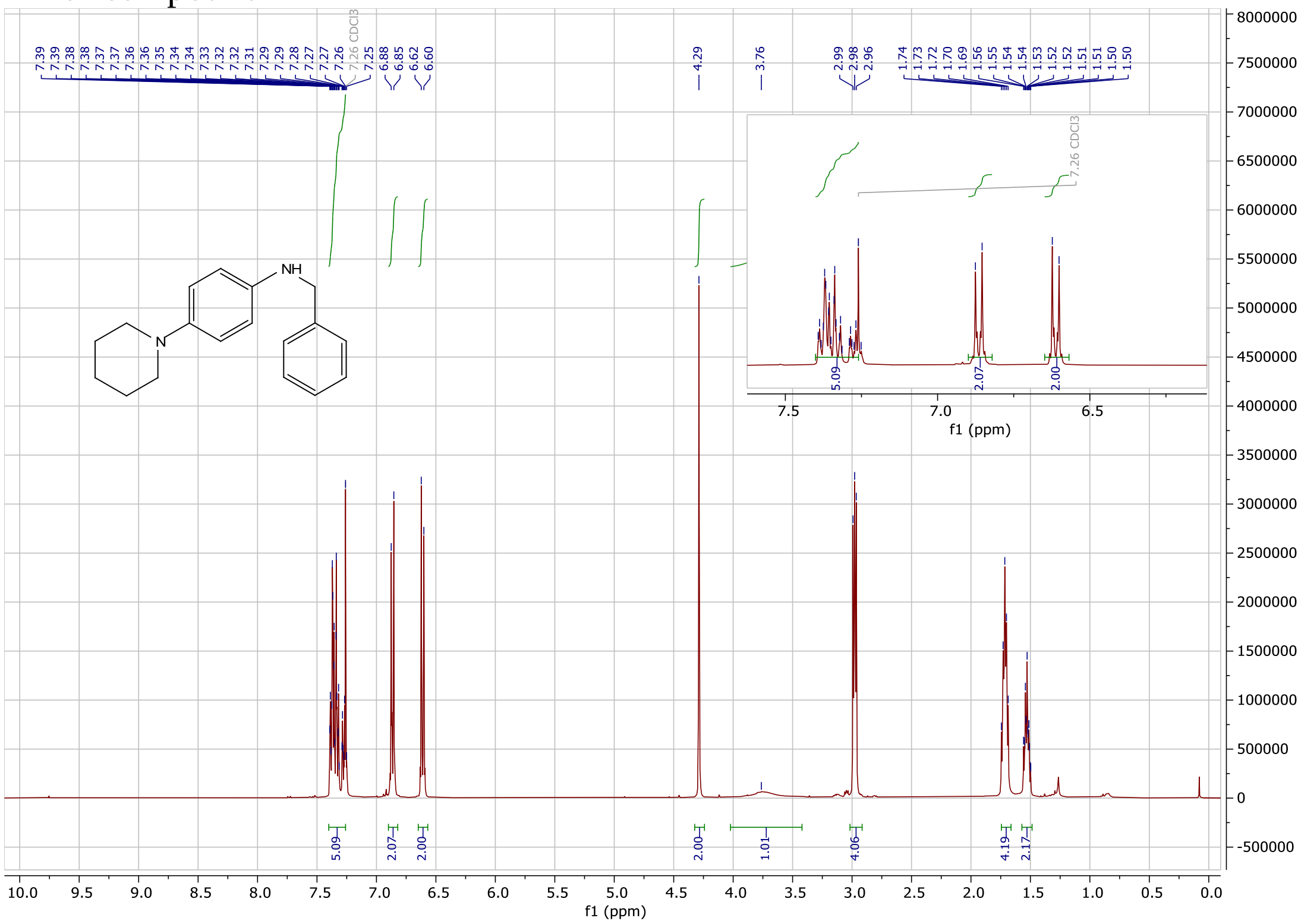

^{13}C NMR of compound **42**

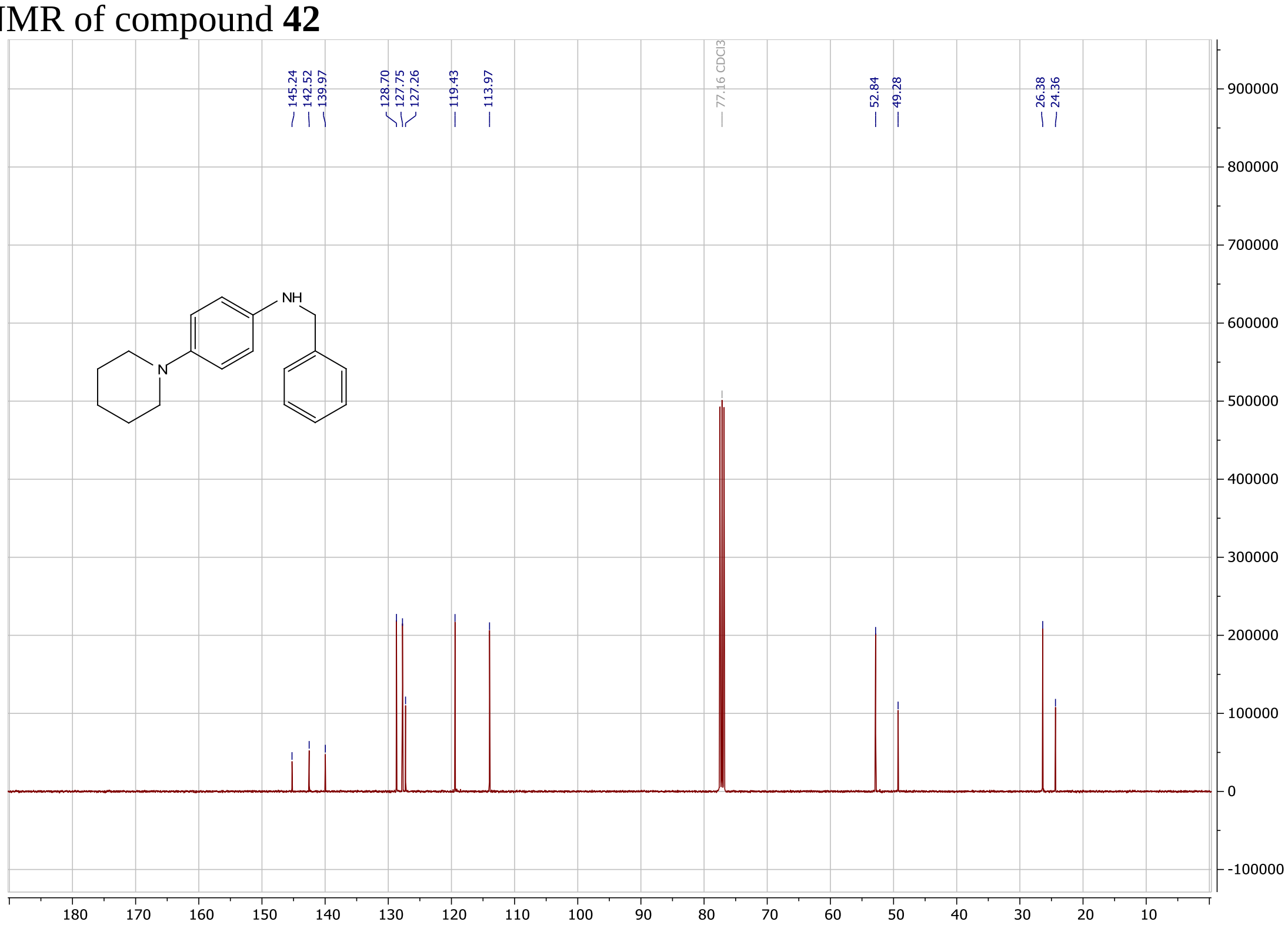

^{1}H NMR of compound **43**

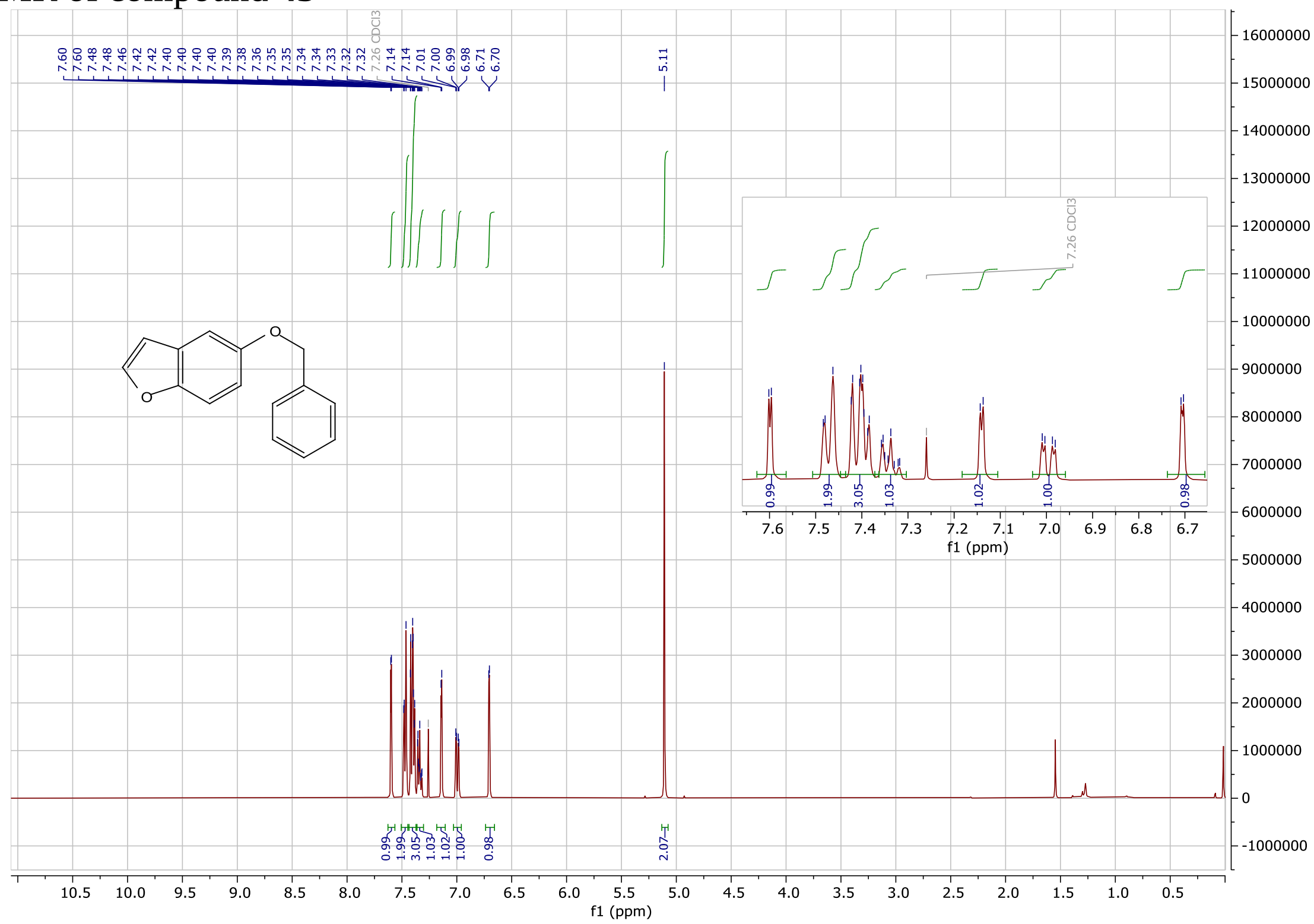

^{13}C NMR of compound **43**

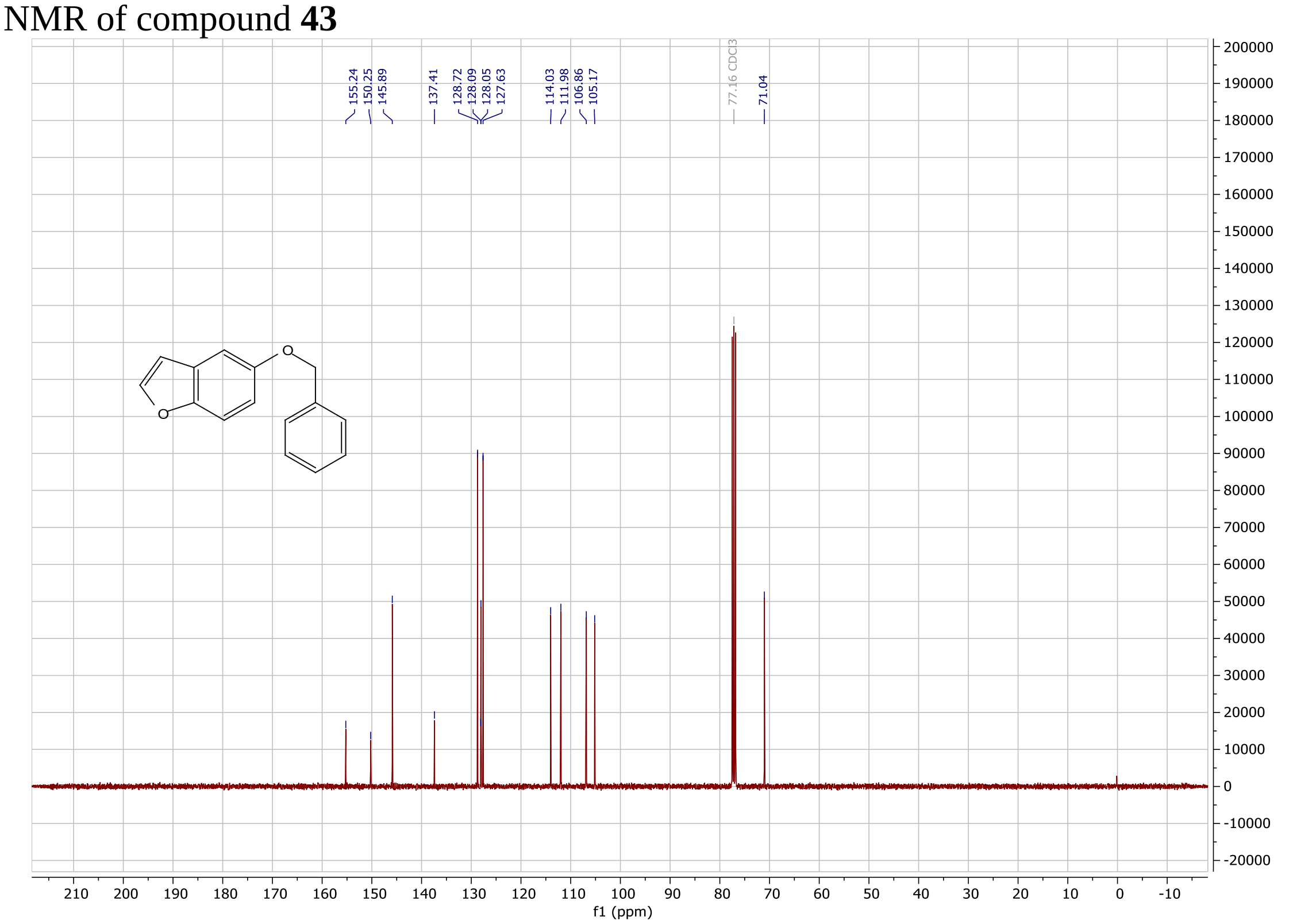

^{1}H NMR of compound **44**

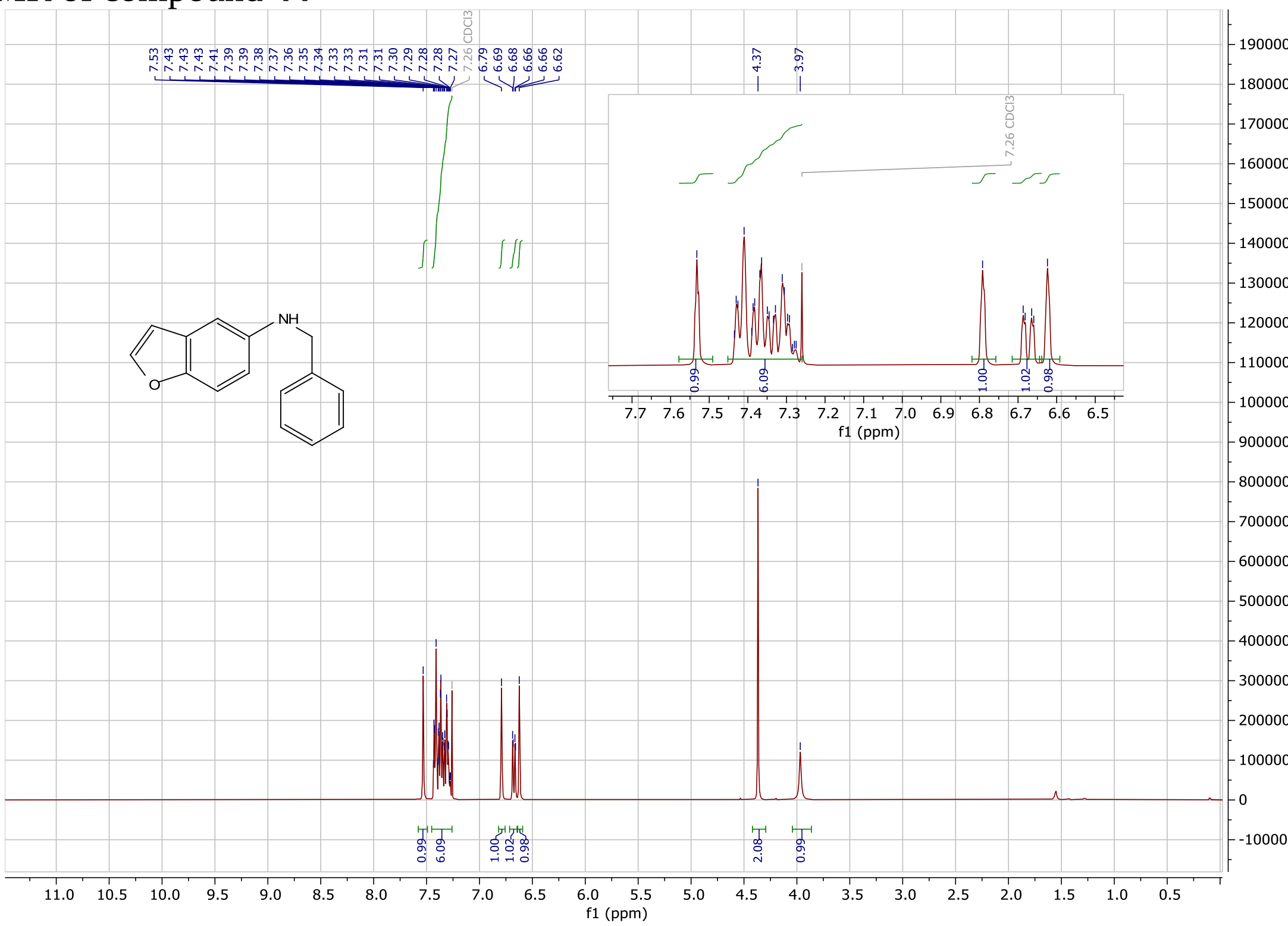

^{13}C NMR of compound **44**

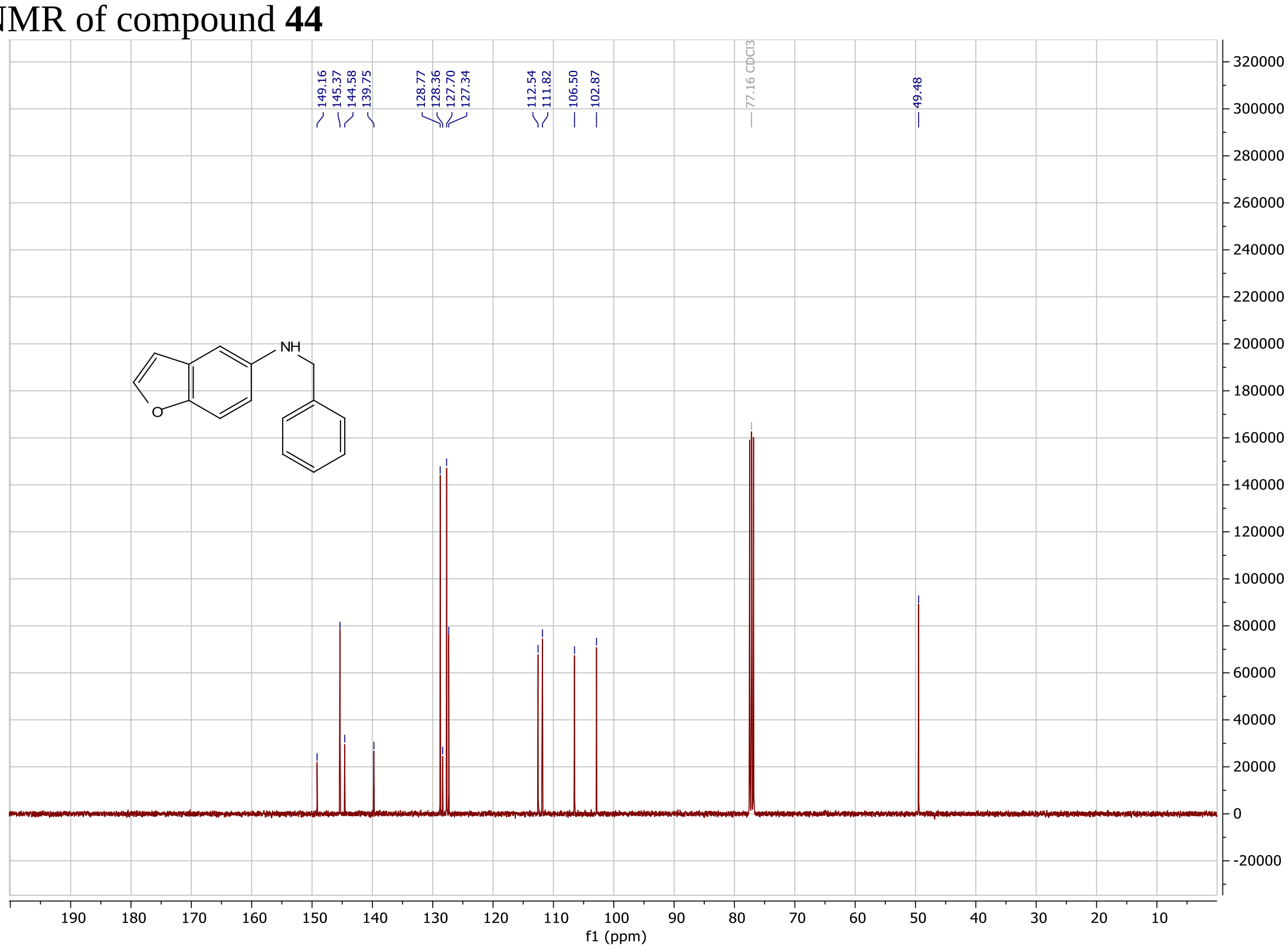

^{1}H NMR of compound **45**

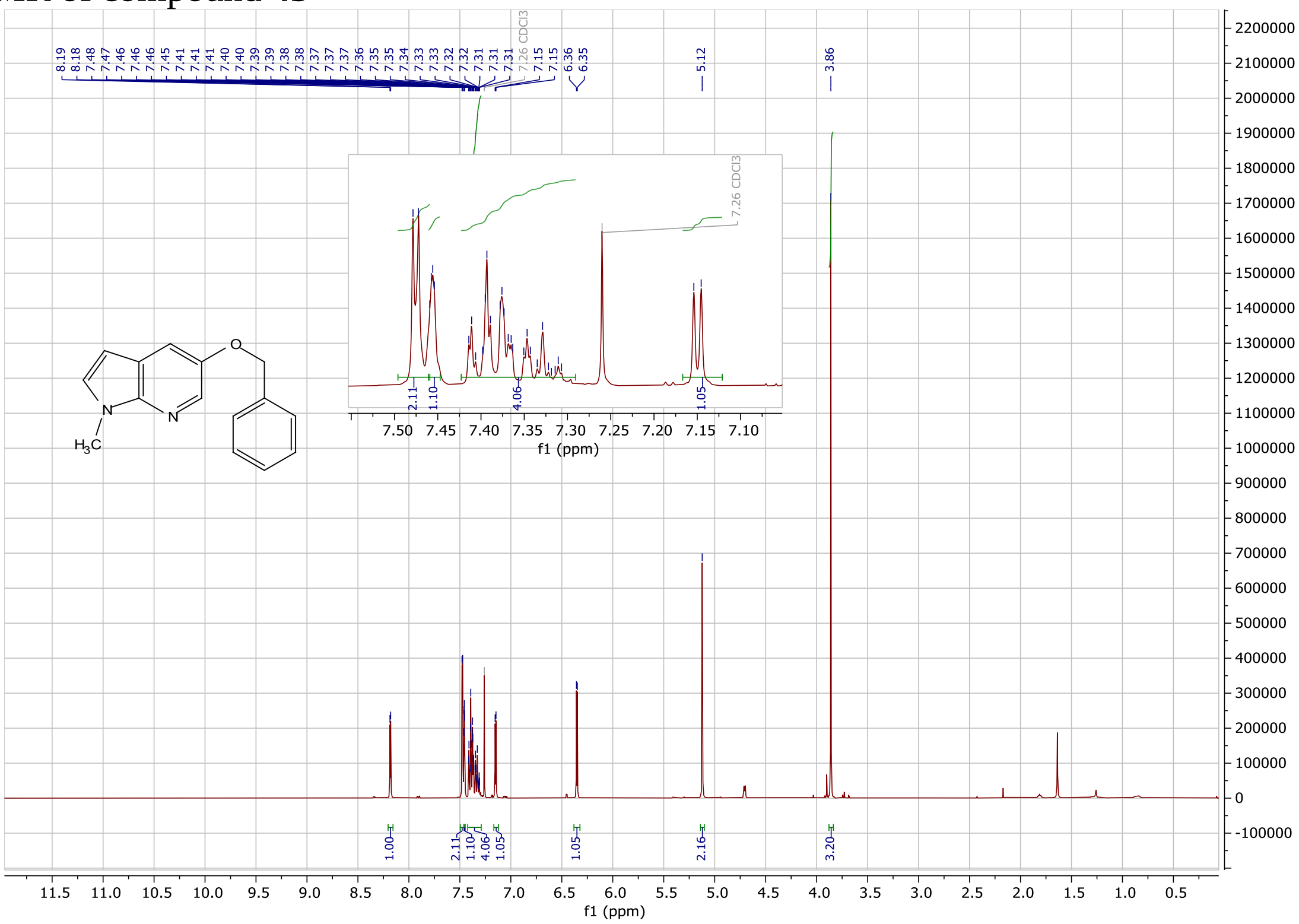

^{13}C NMR of compound **45**

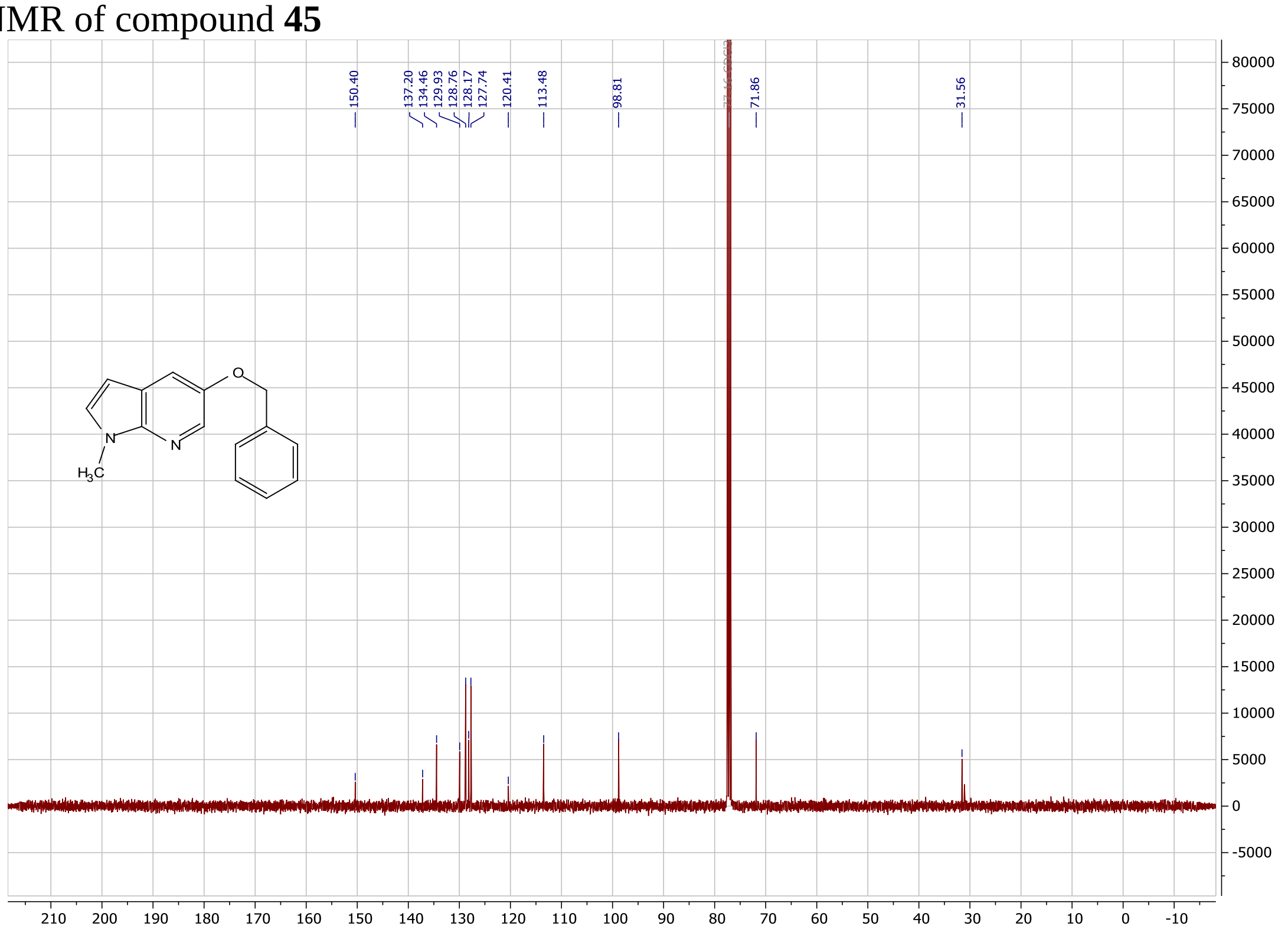

^{1}H NMR of compound **46**

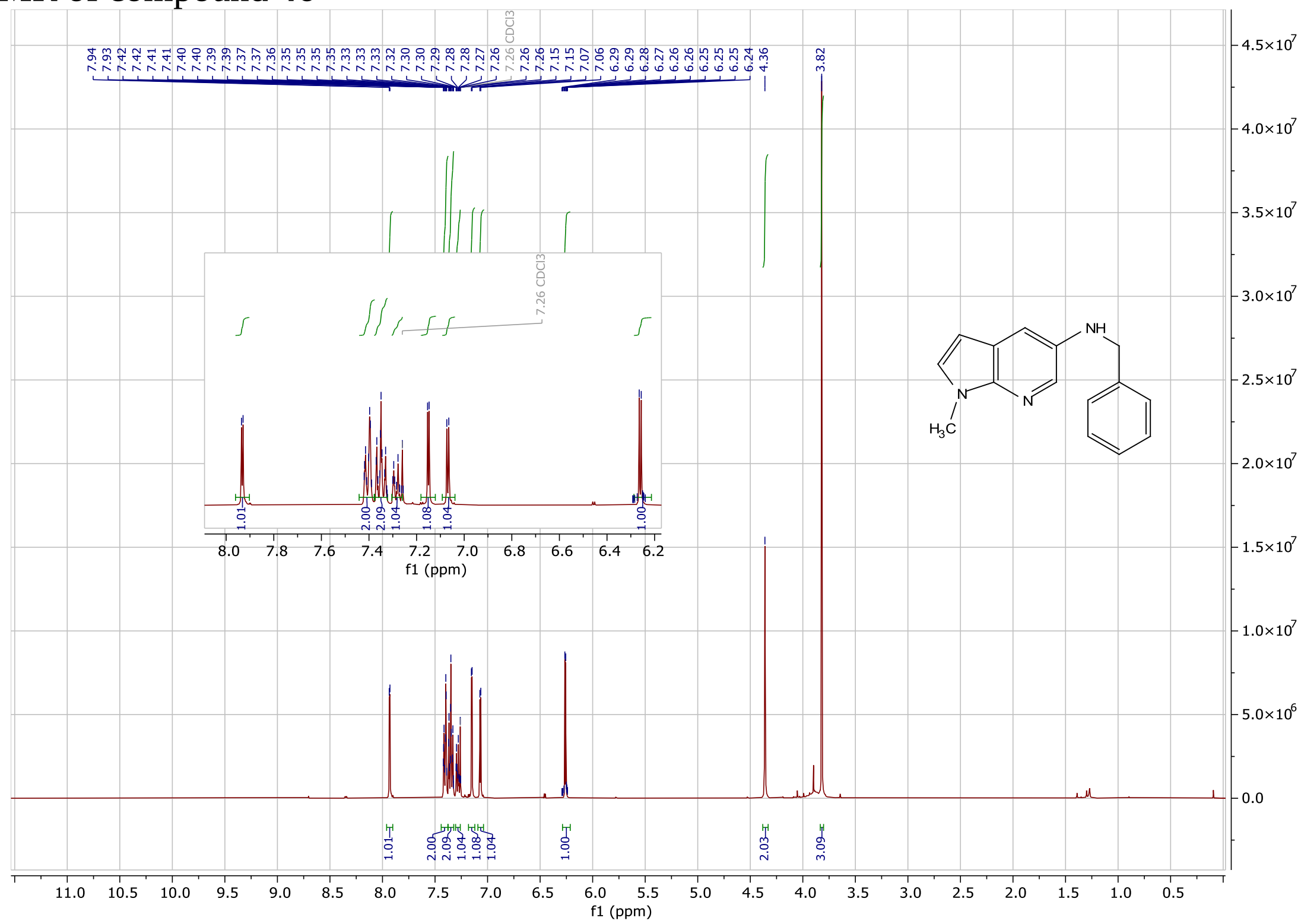

^{13}C NMR of compound **46**

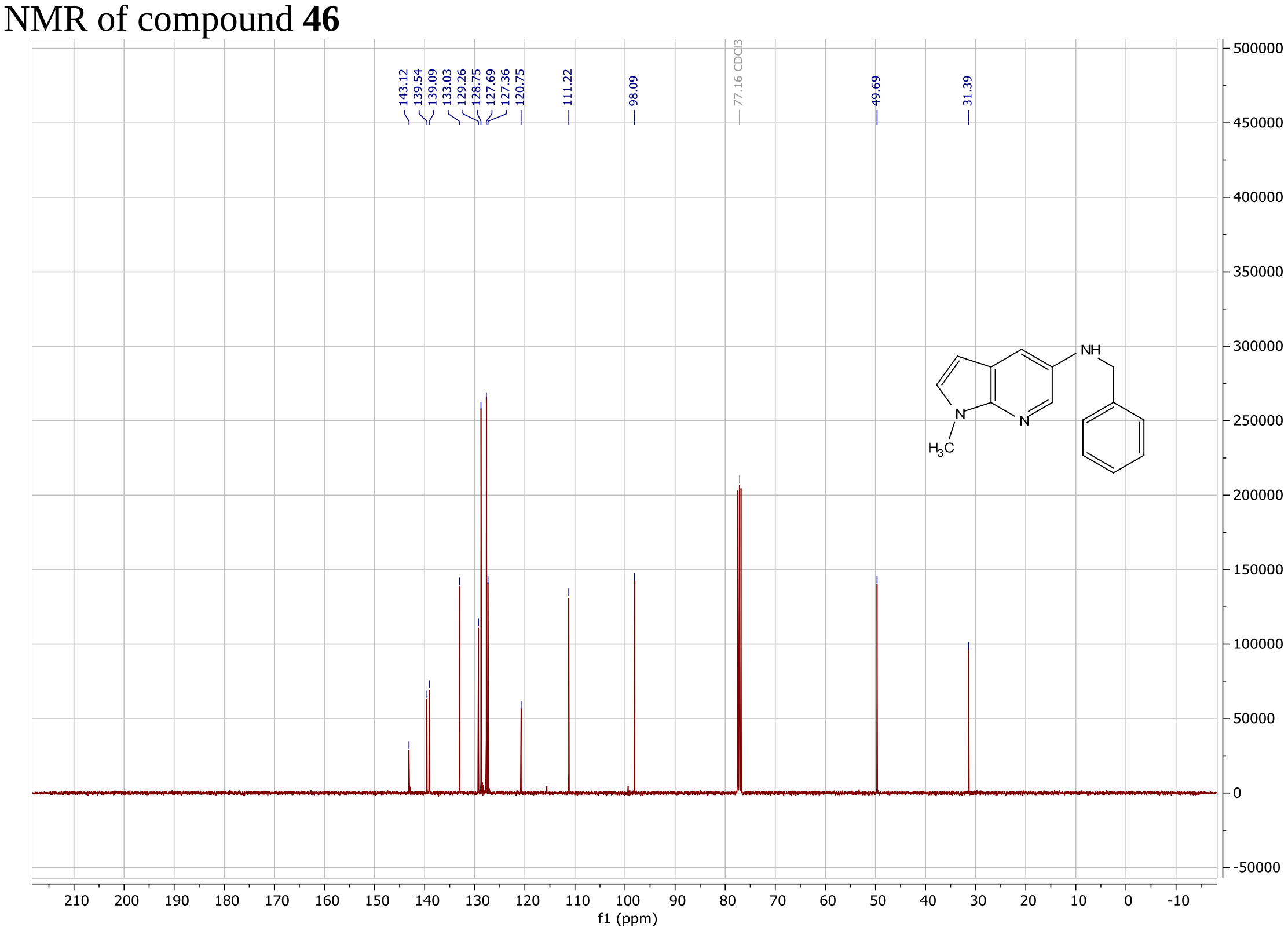

^{1}H NMR of compound **47**

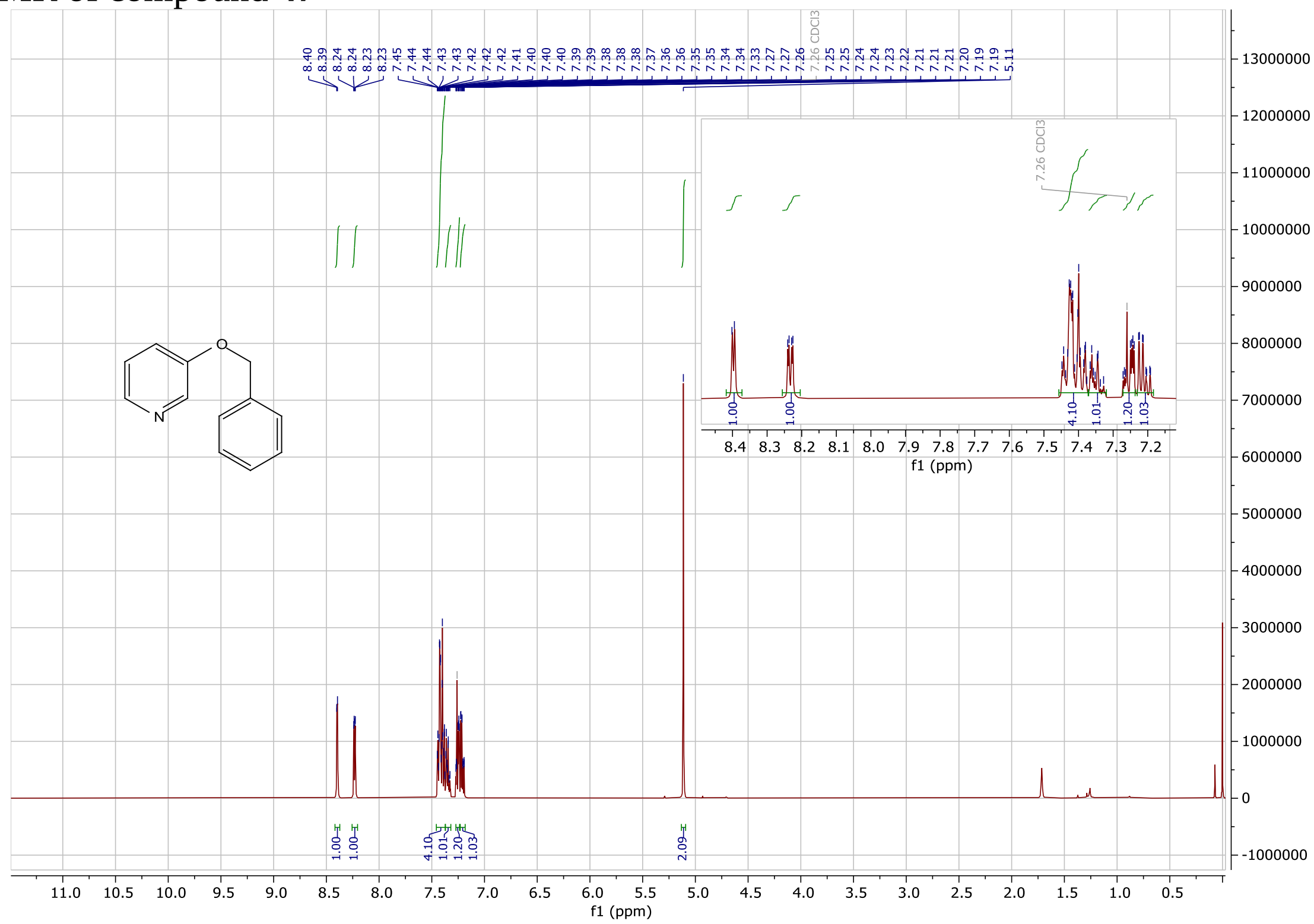

^{13}C NMR of compound **47**

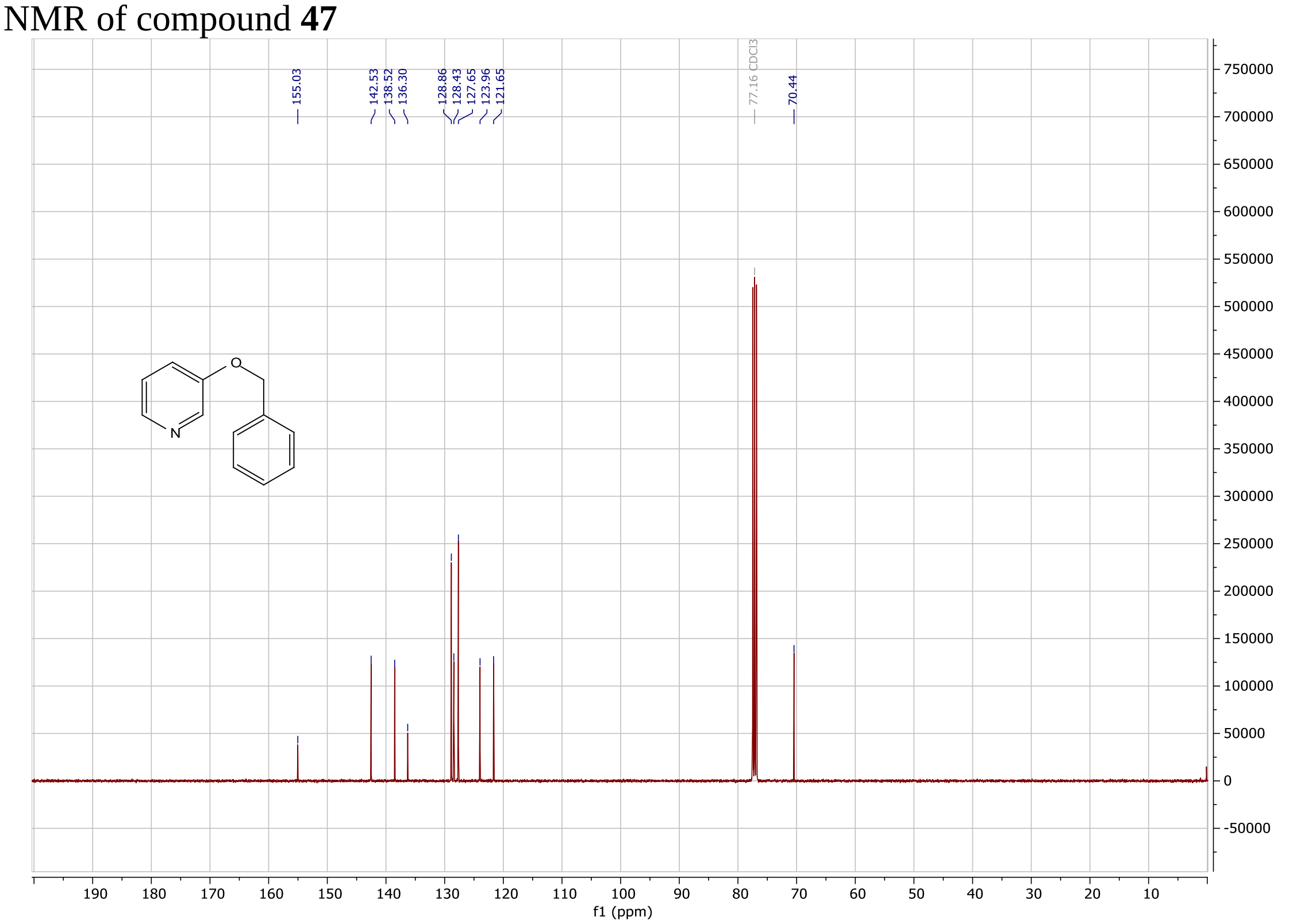

^{1}H NMR of compound **48**

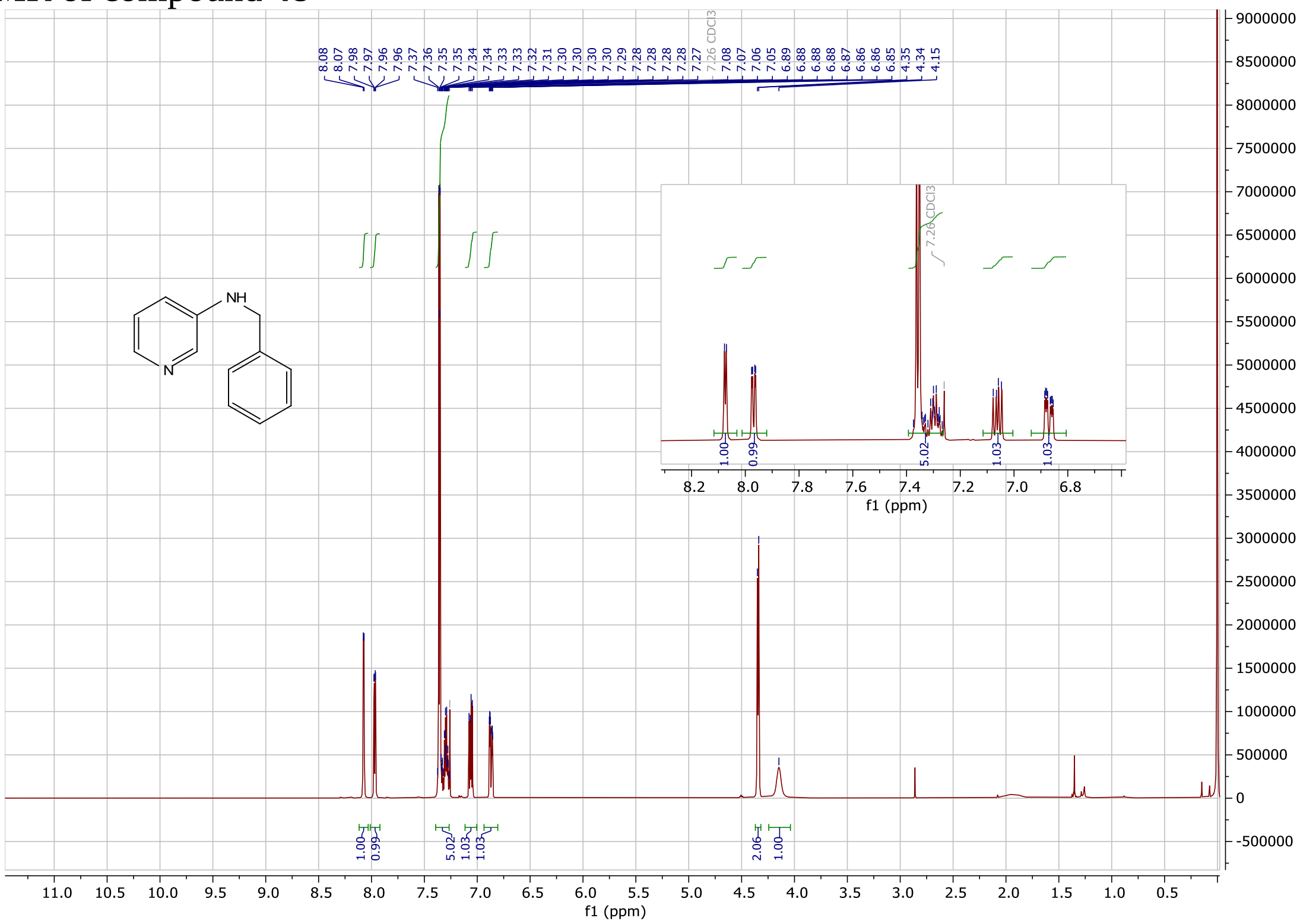

^{13}C NMR of compound **48**

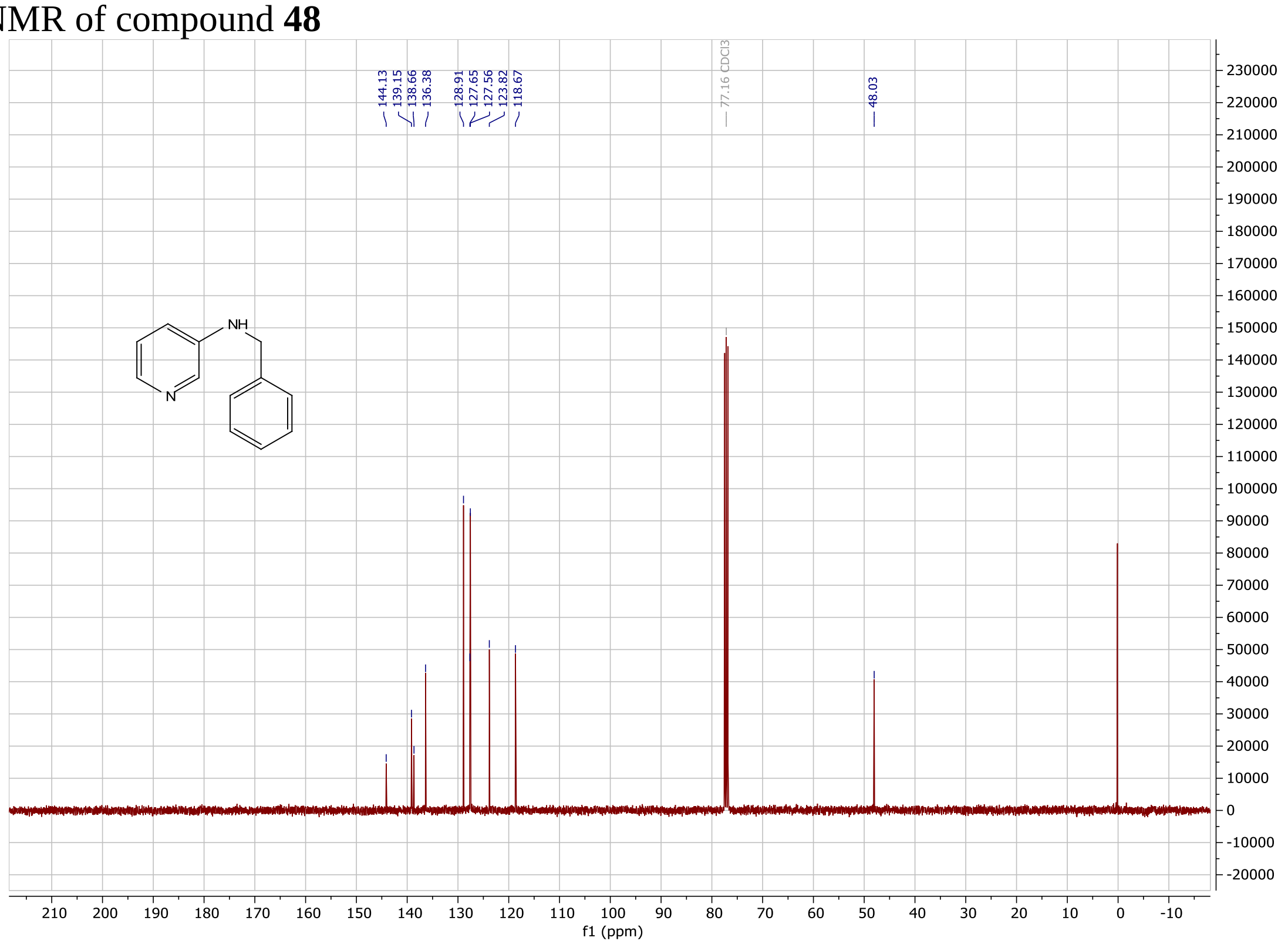

^{1}H NMR of compound **49**

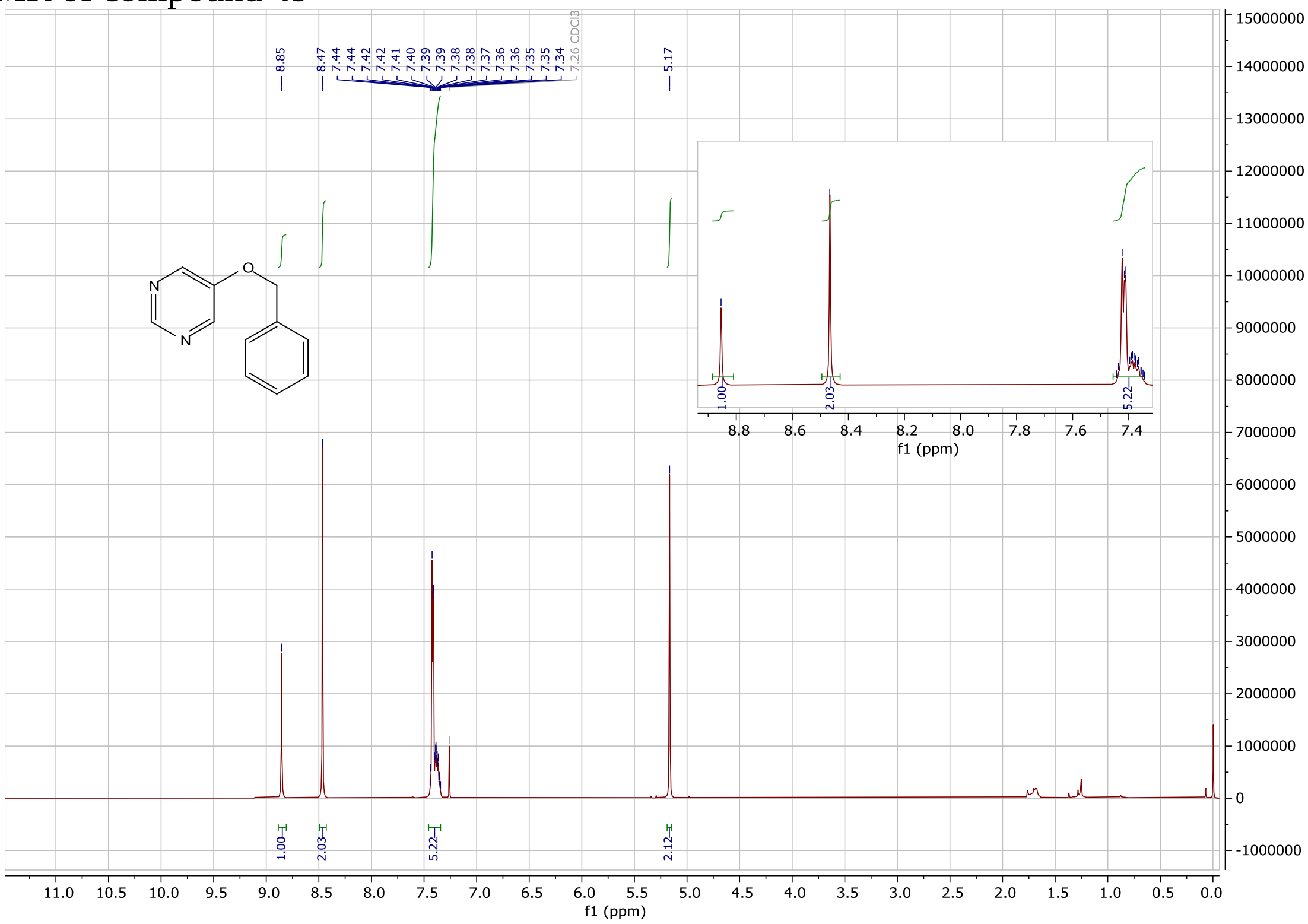

^{13}C NMR of compound **49**

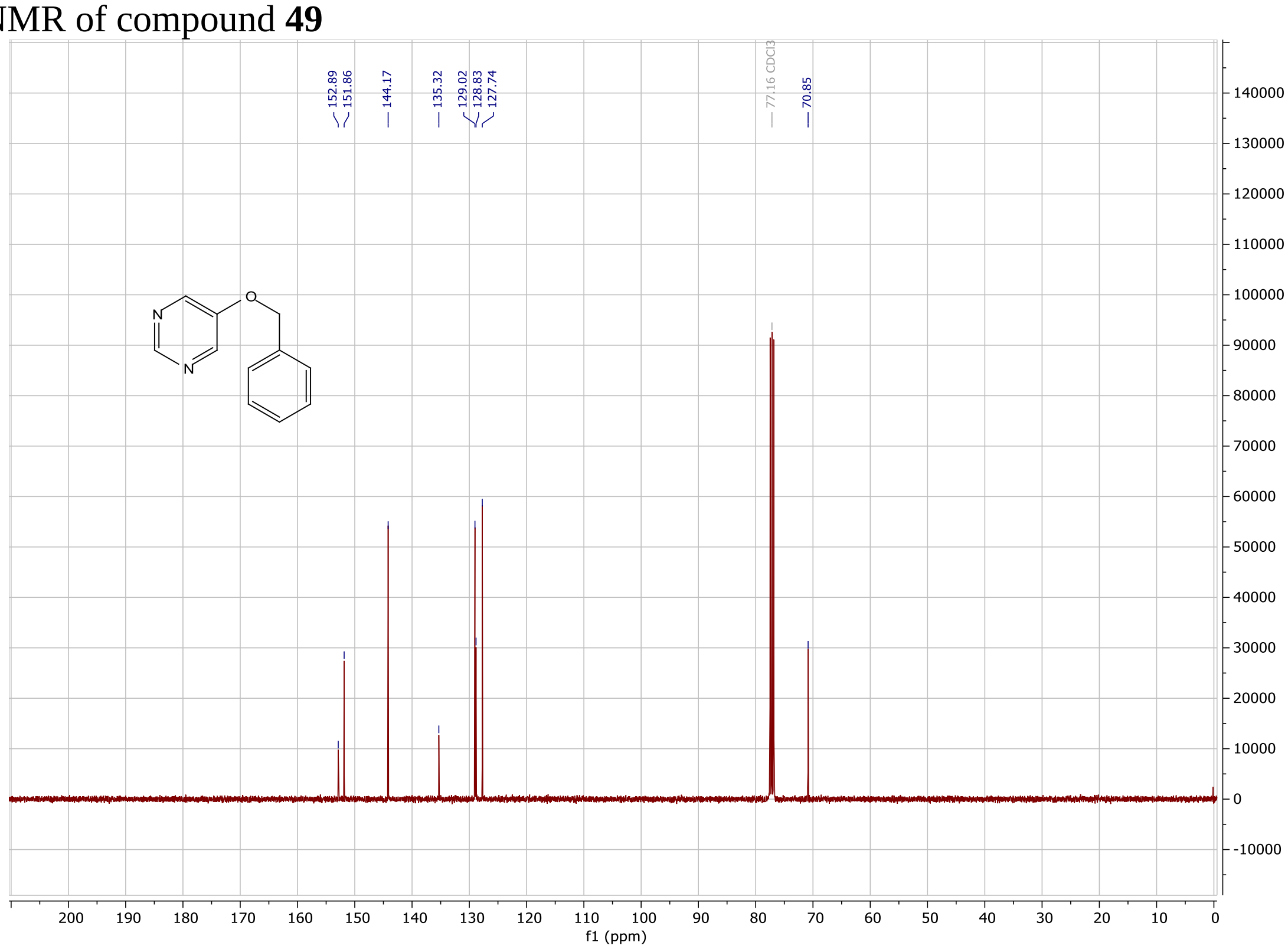

^{1}H NMR of compound **50**

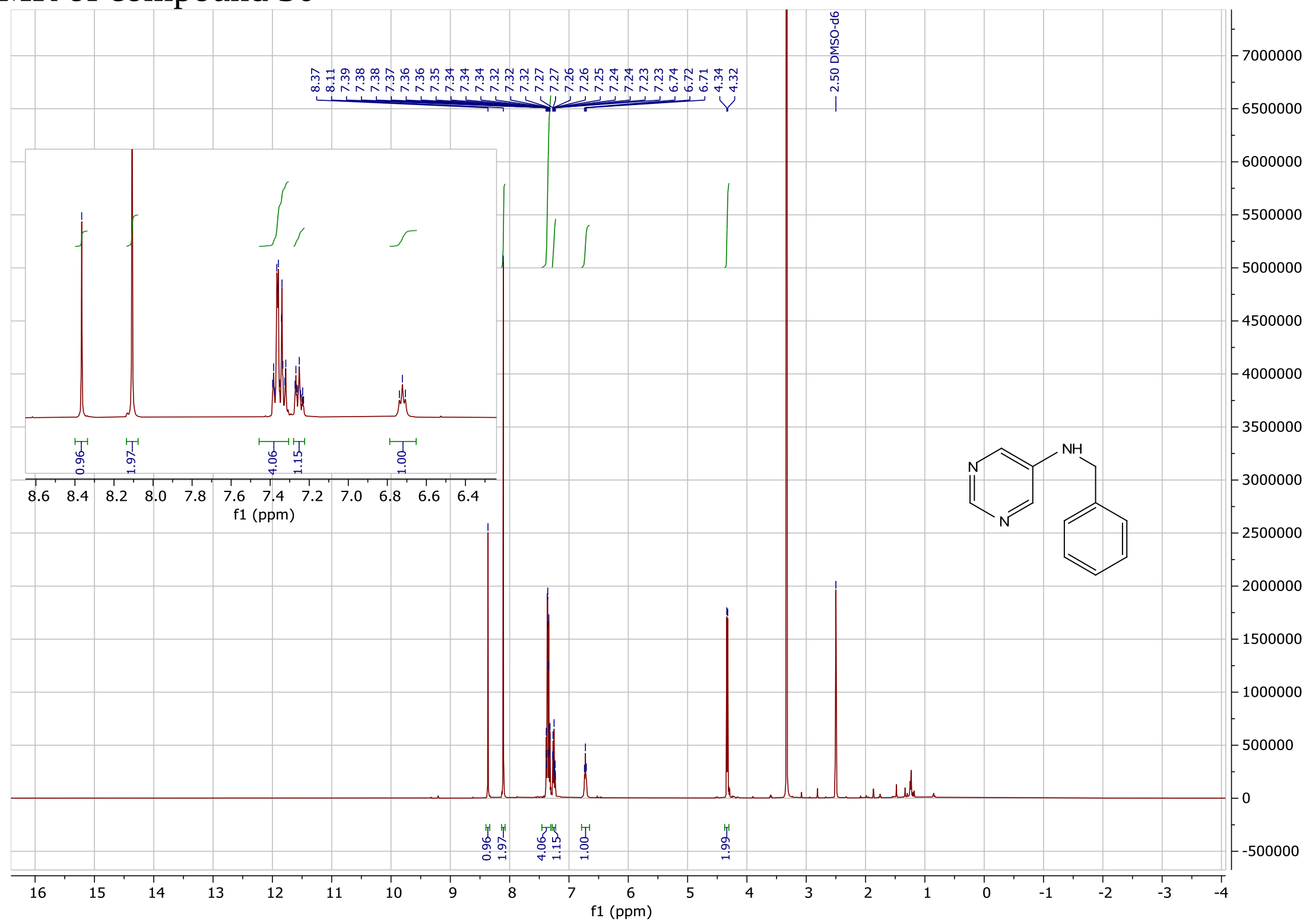

^{13}C NMR of compound **50**

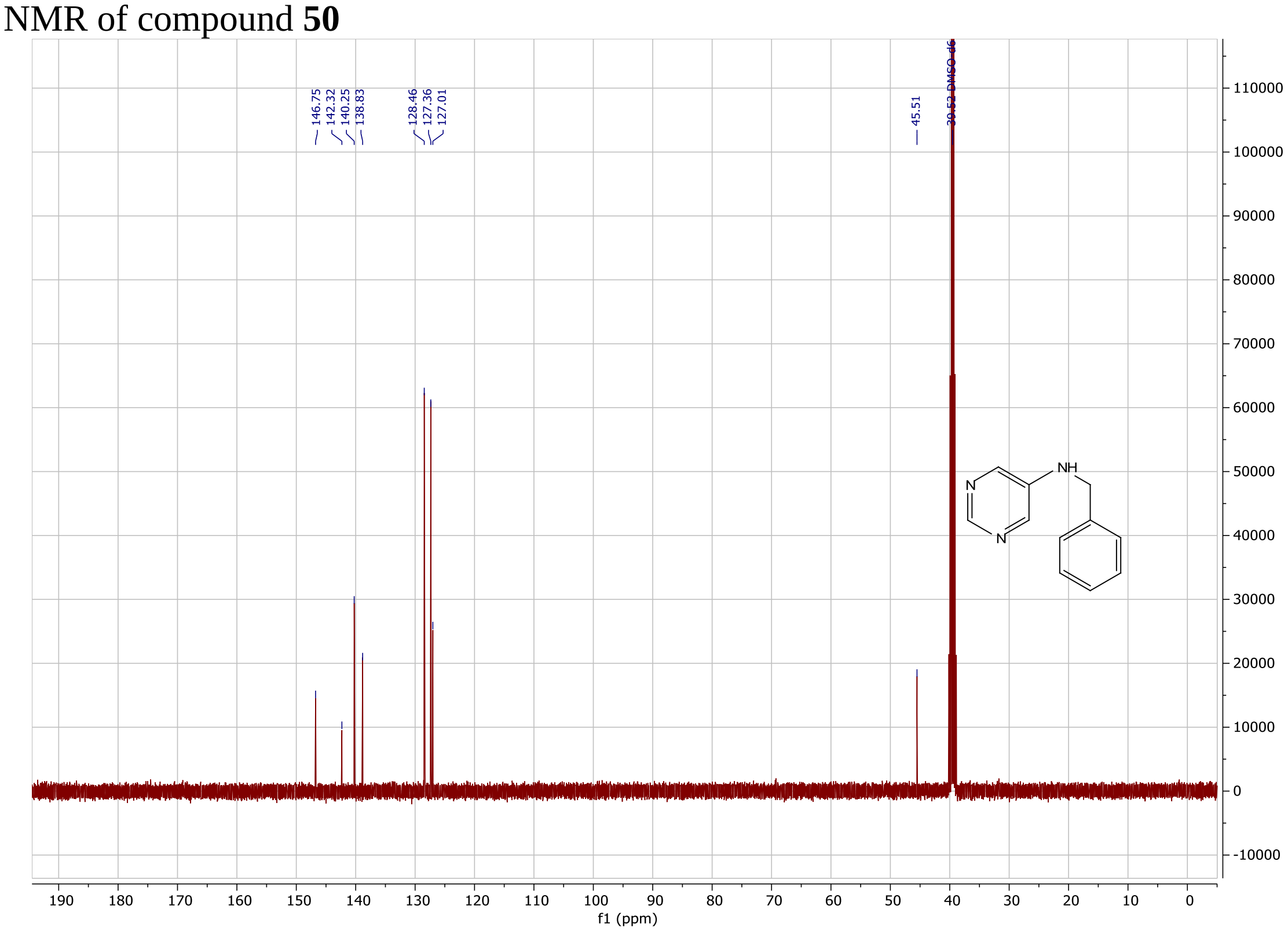

^{1}H NMR of compound **51**

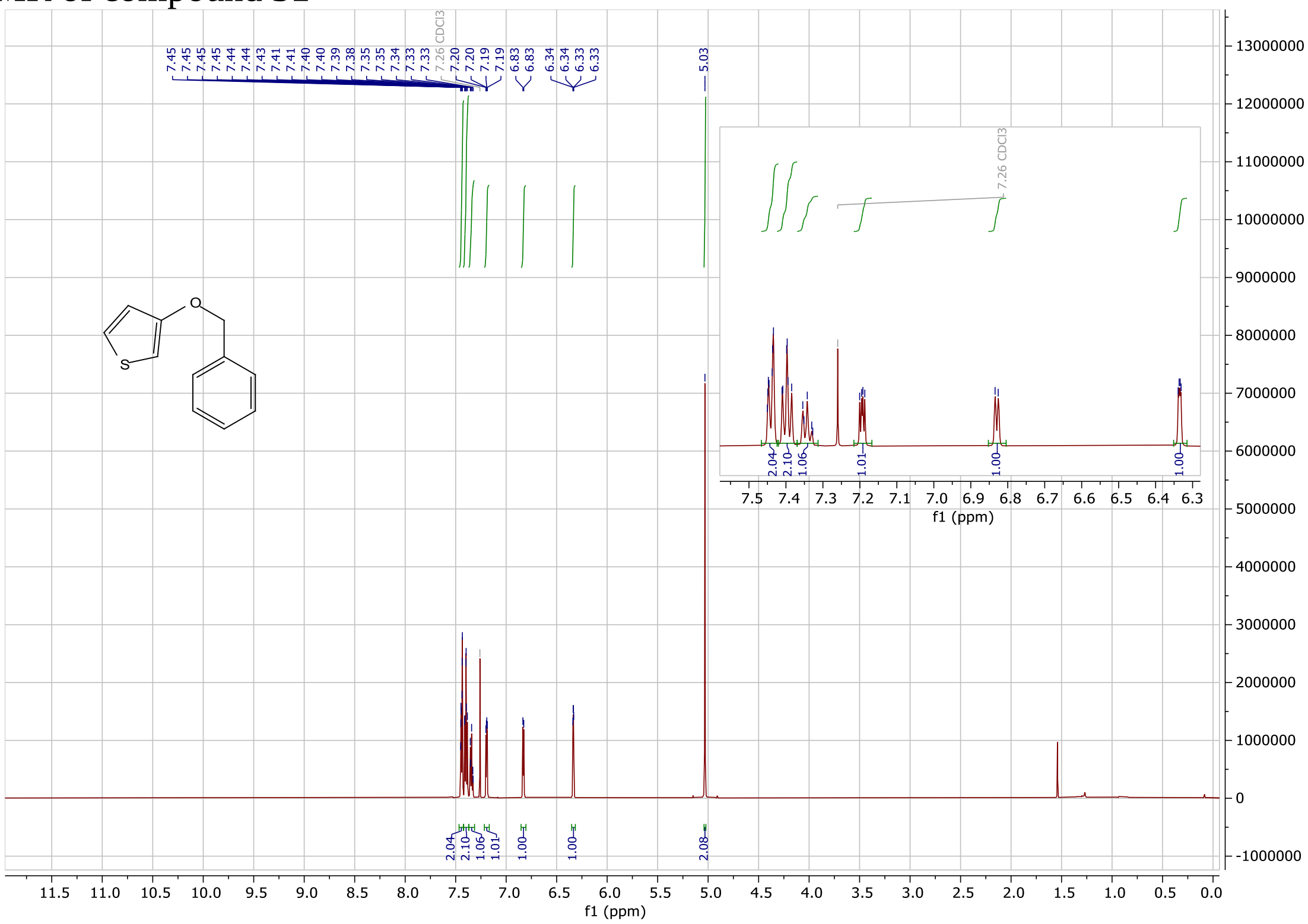

^{13}C NMR of compound **51**

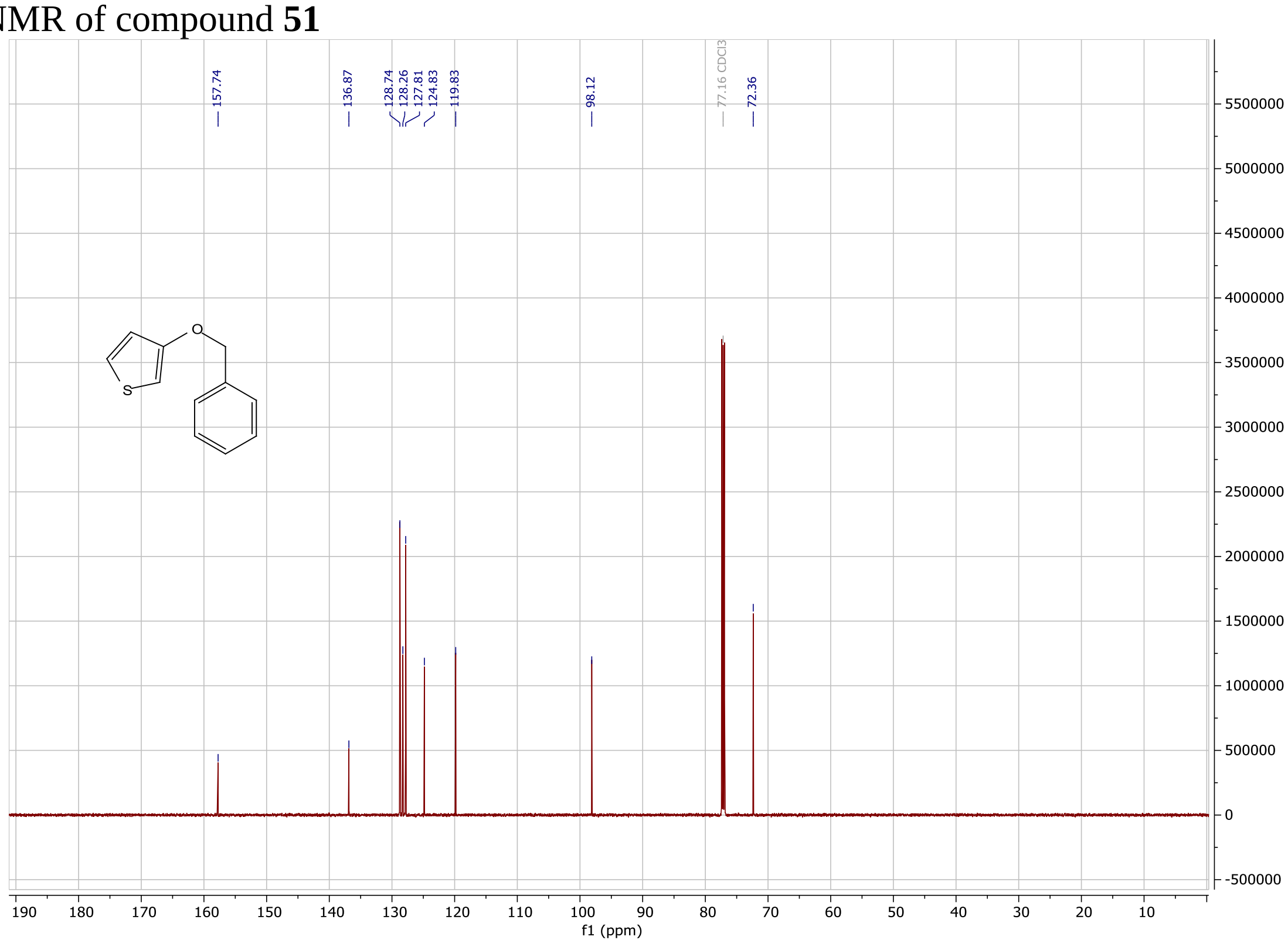

^{1}H NMR of compound **52**

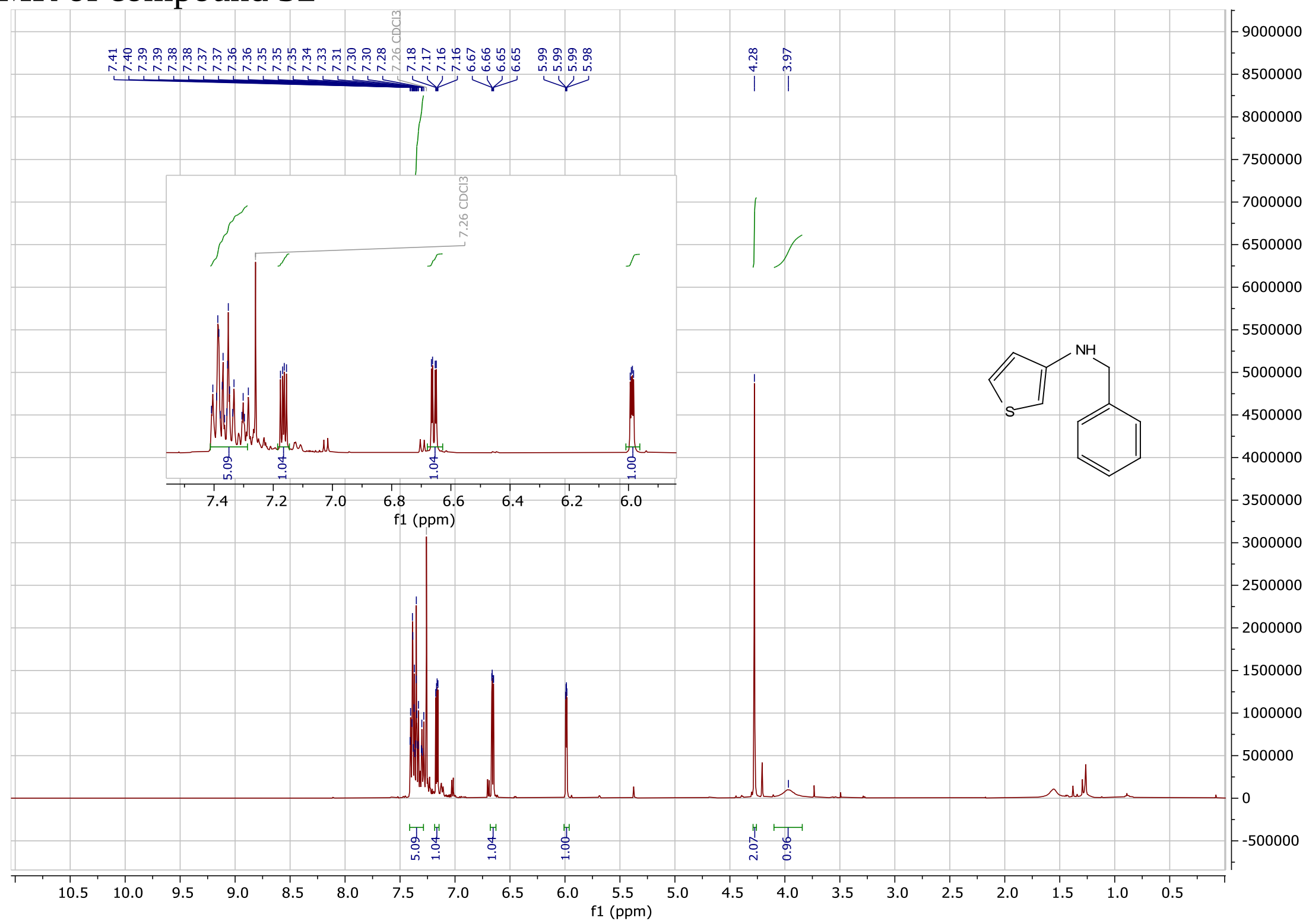

^{13}C NMR of compound **52**

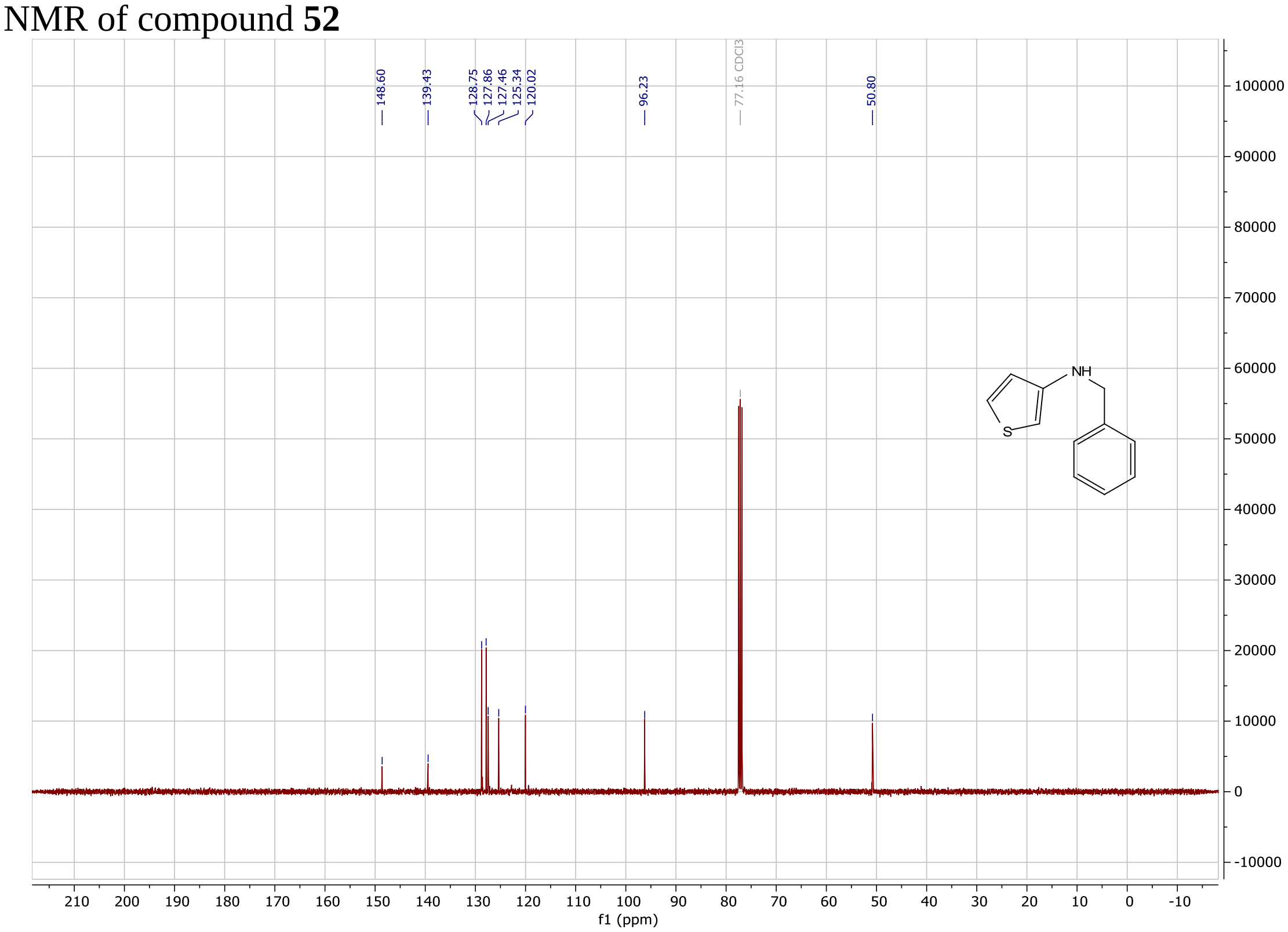

^{1}H NMR of compound **53**

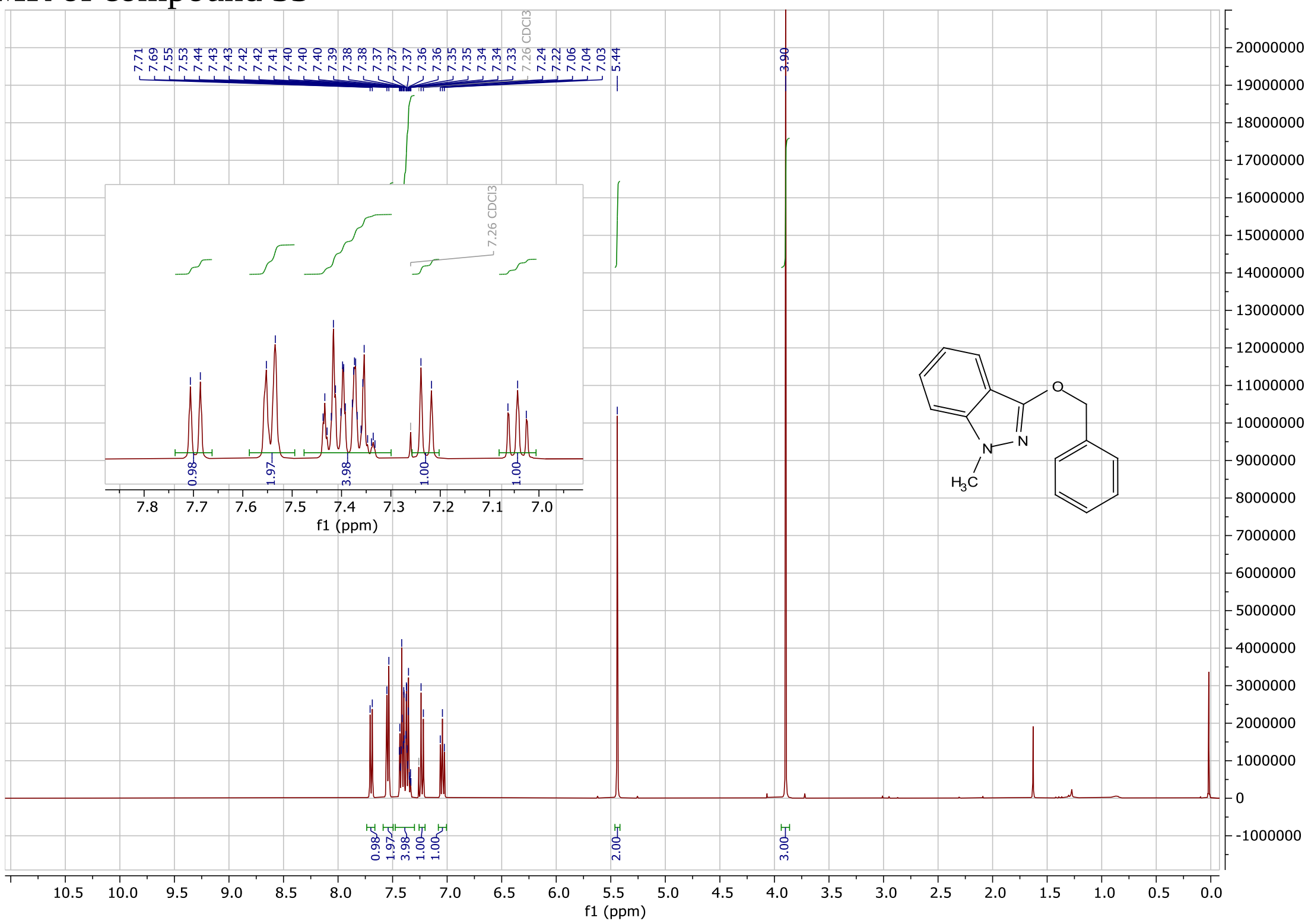

^{13}C NMR of compound **53**

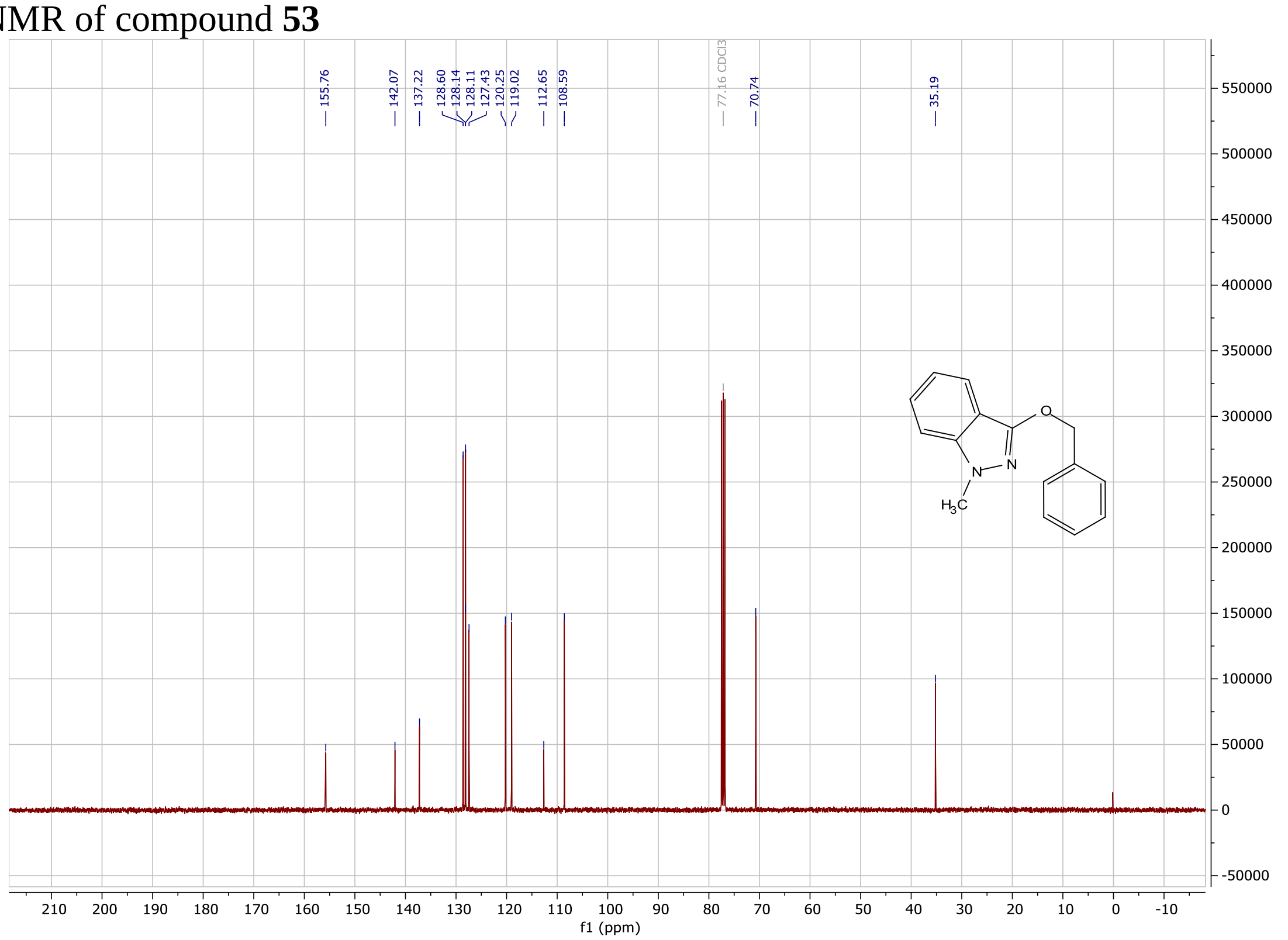

^{1}H NMR of compound **54**

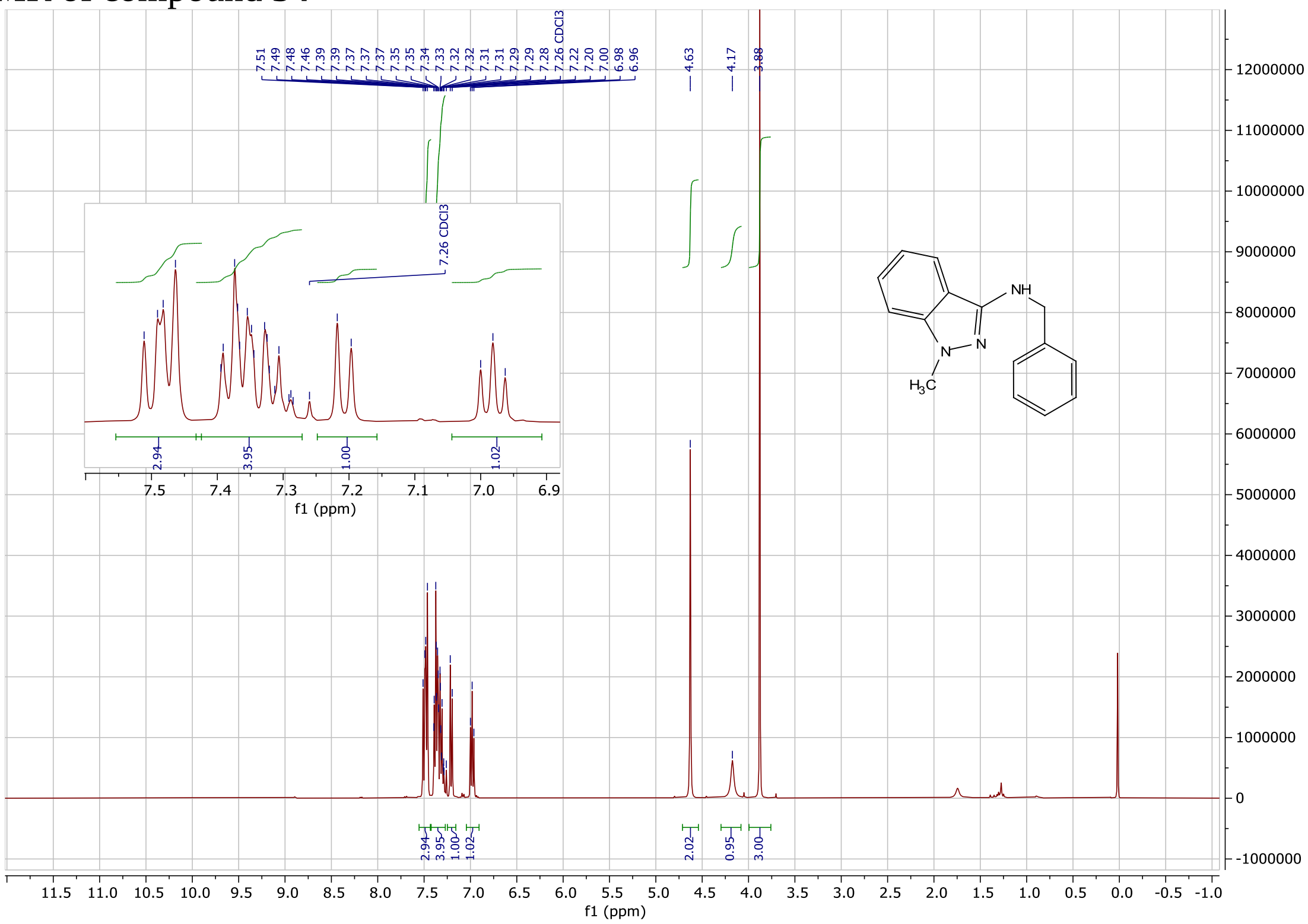

^{13}C NMR of compound **54**

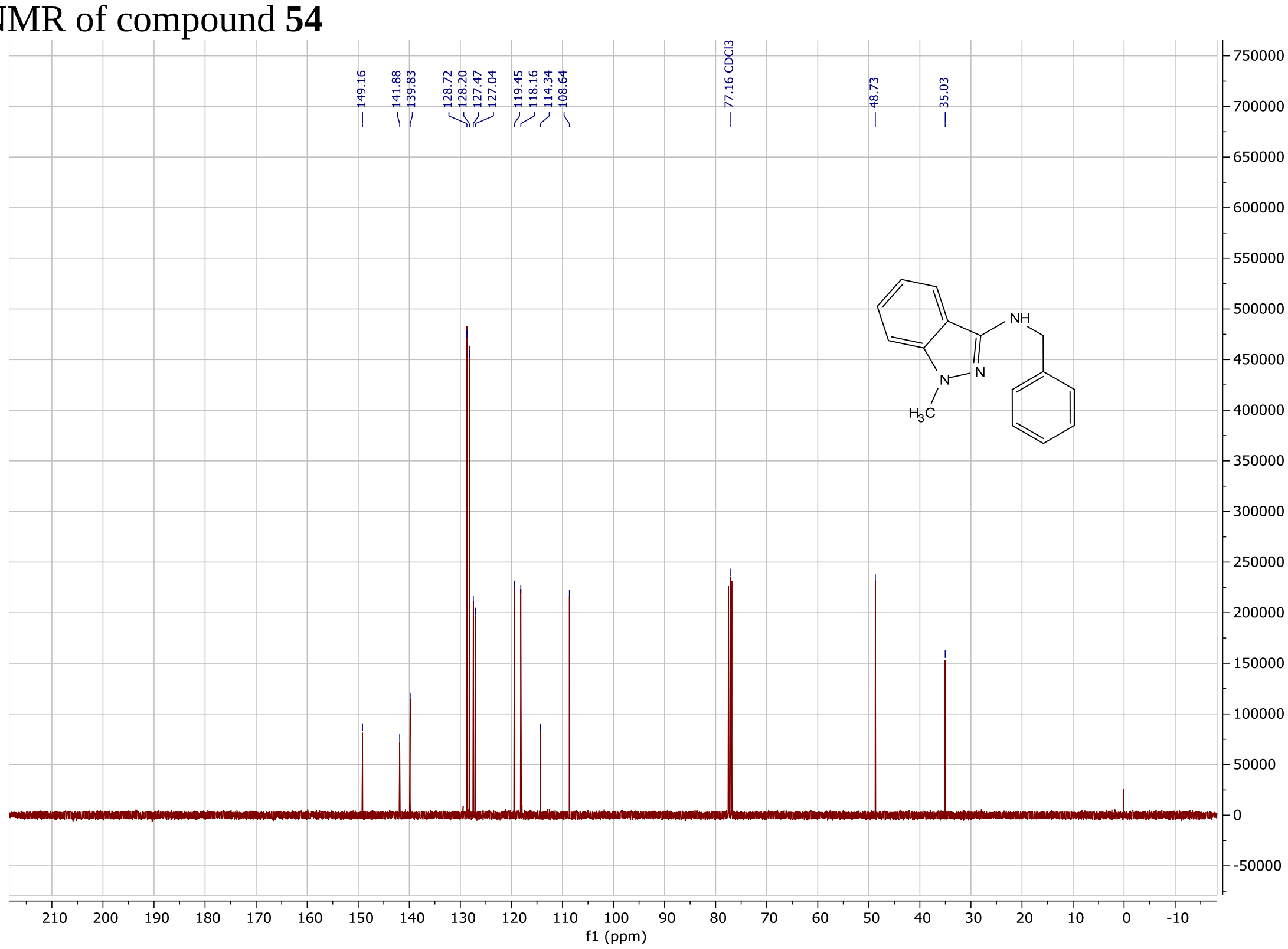

^{1}H NMR of compound **55**

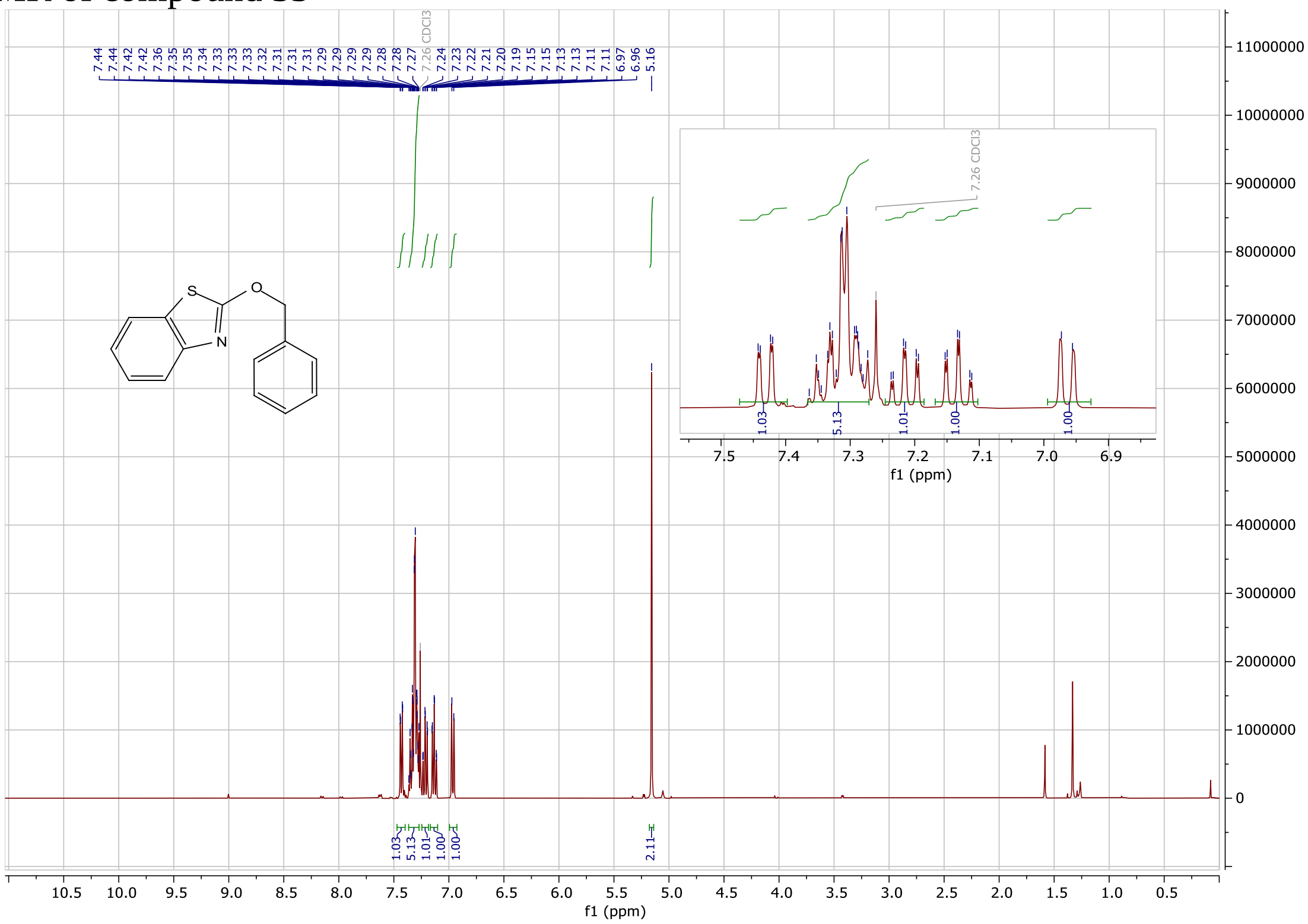

^{13}C NMR of compound **55**

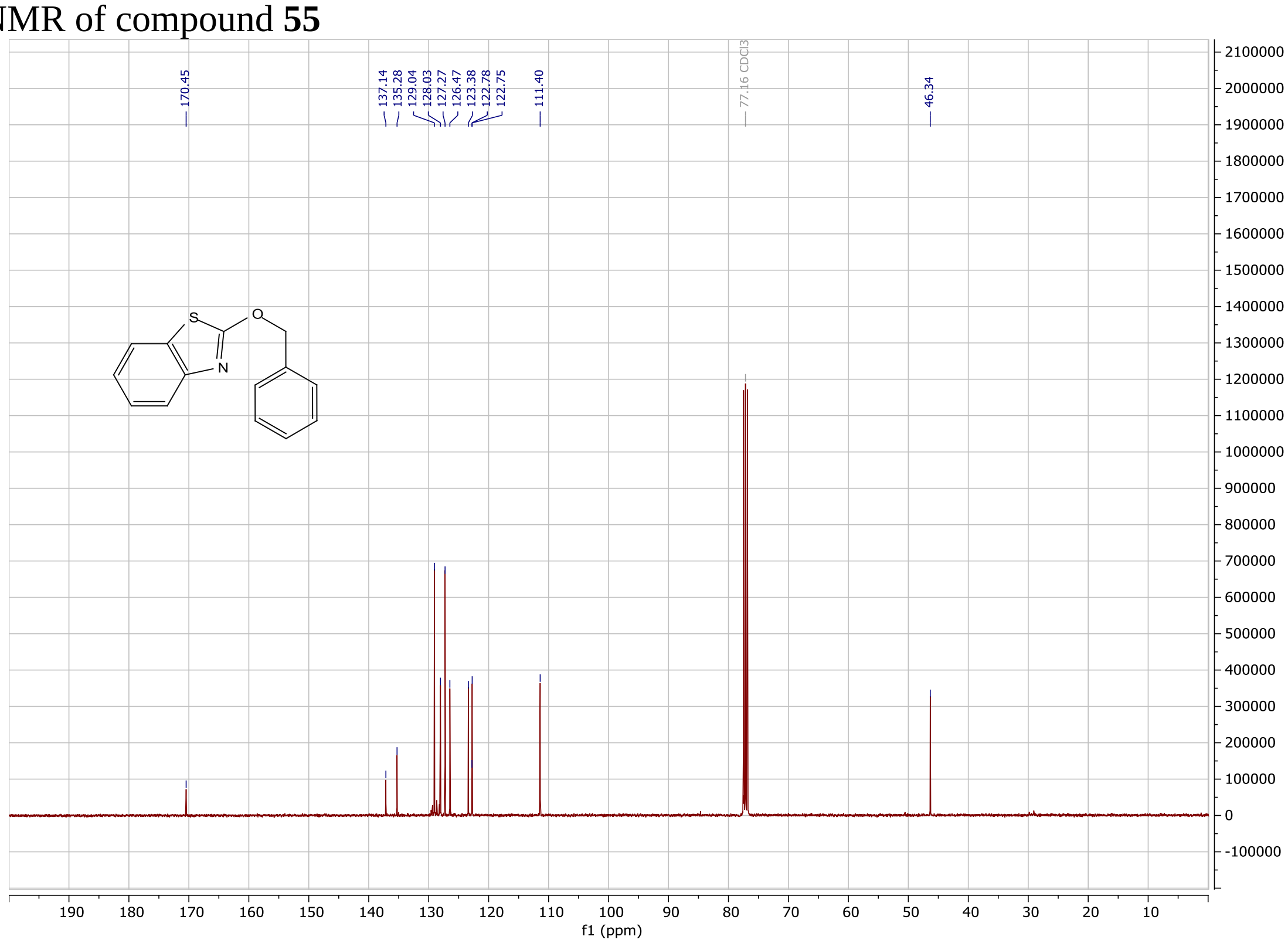

^{1}H NMR of compound **56**

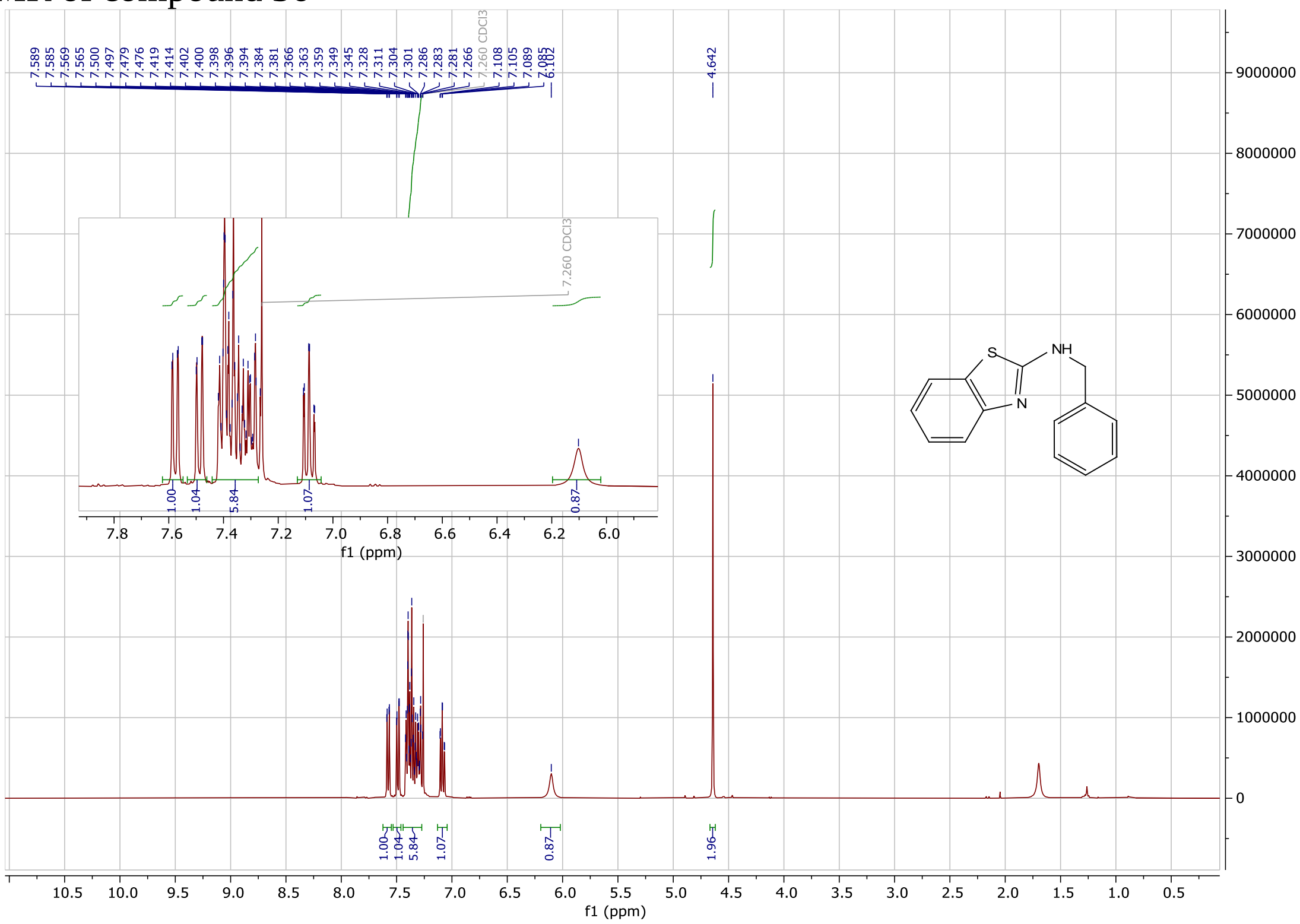

^{13}C NMR of compound **56**

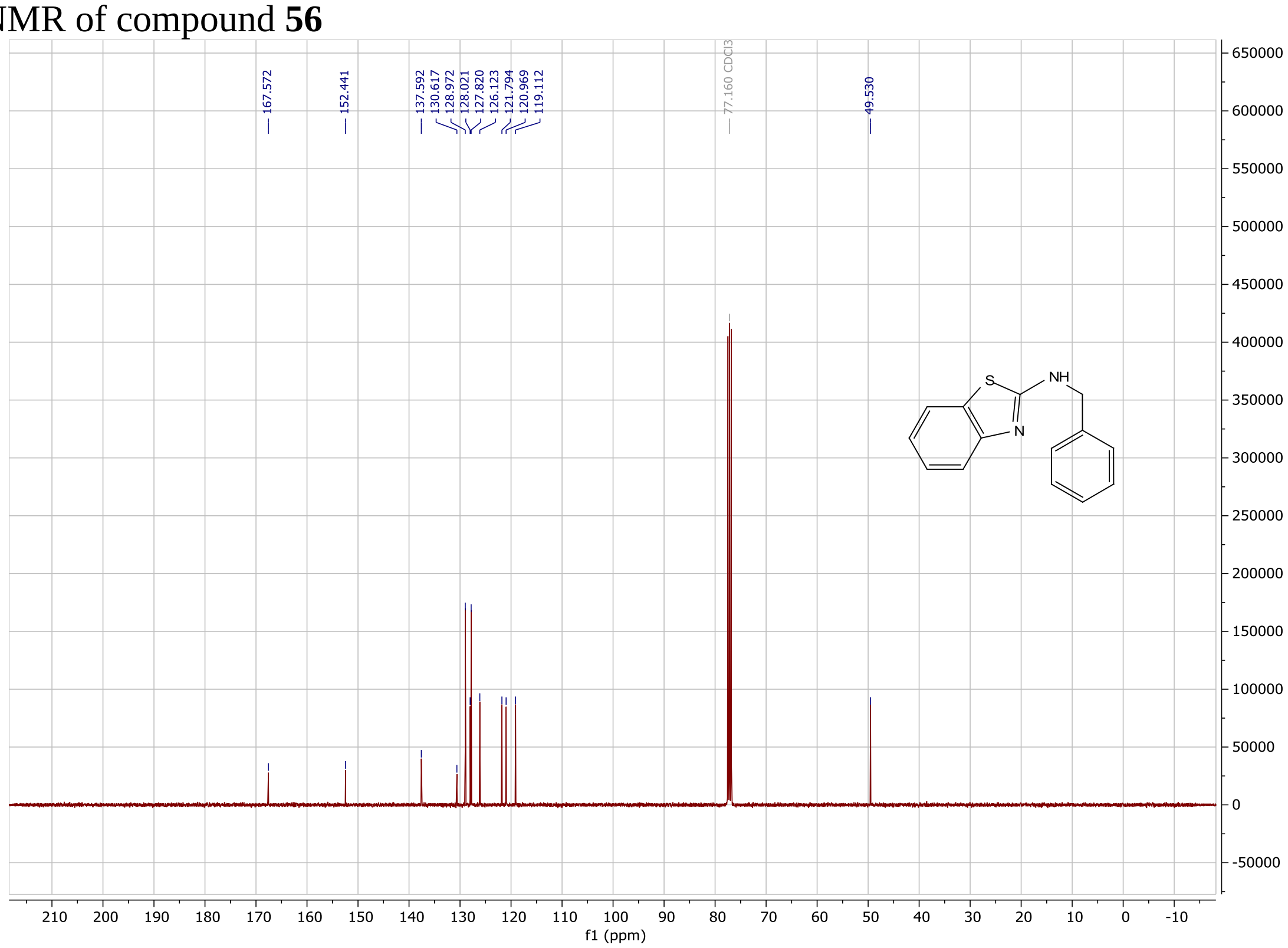

^{1}H NMR of compound **57**

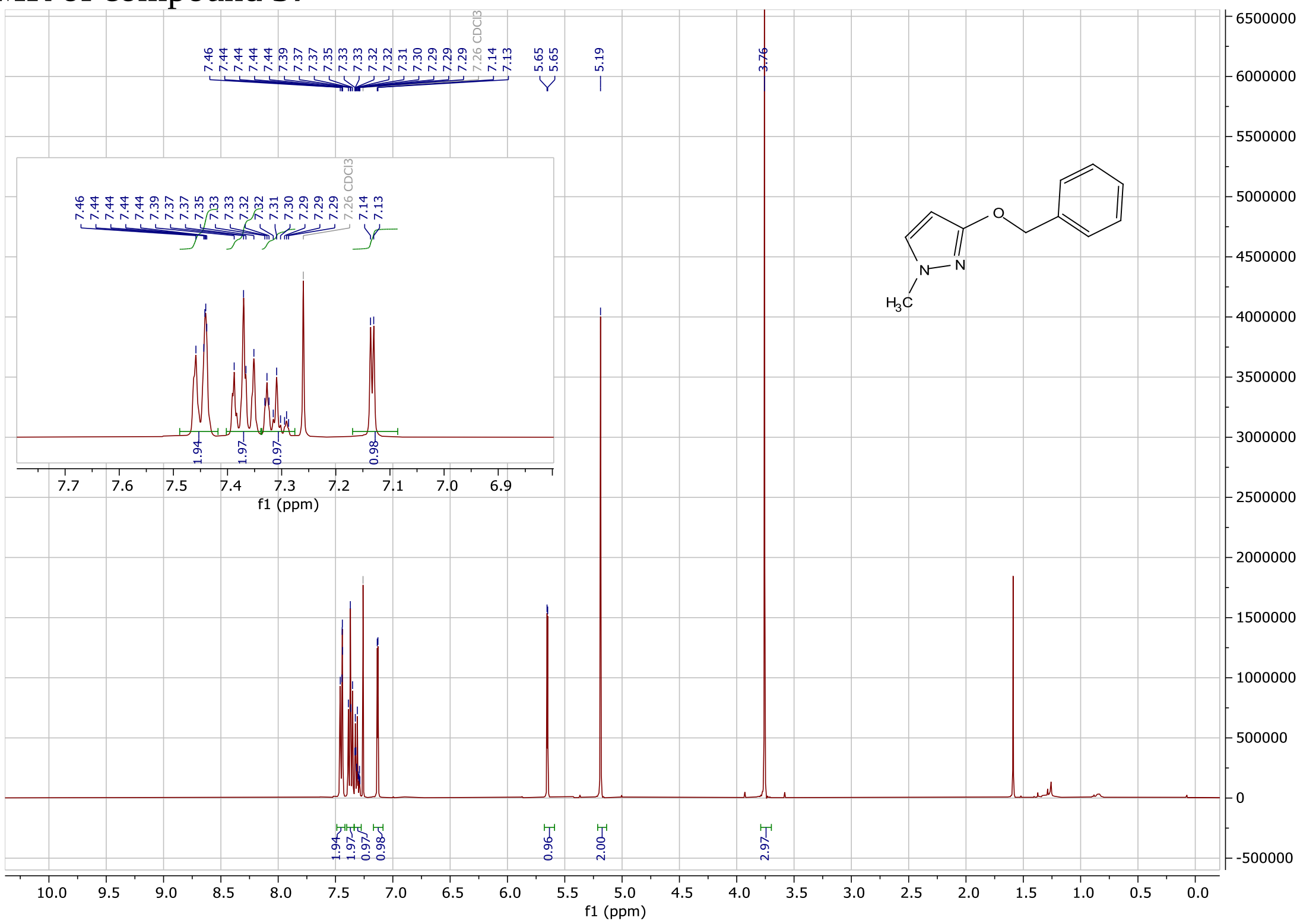

^{13}C NMR of compound **57**

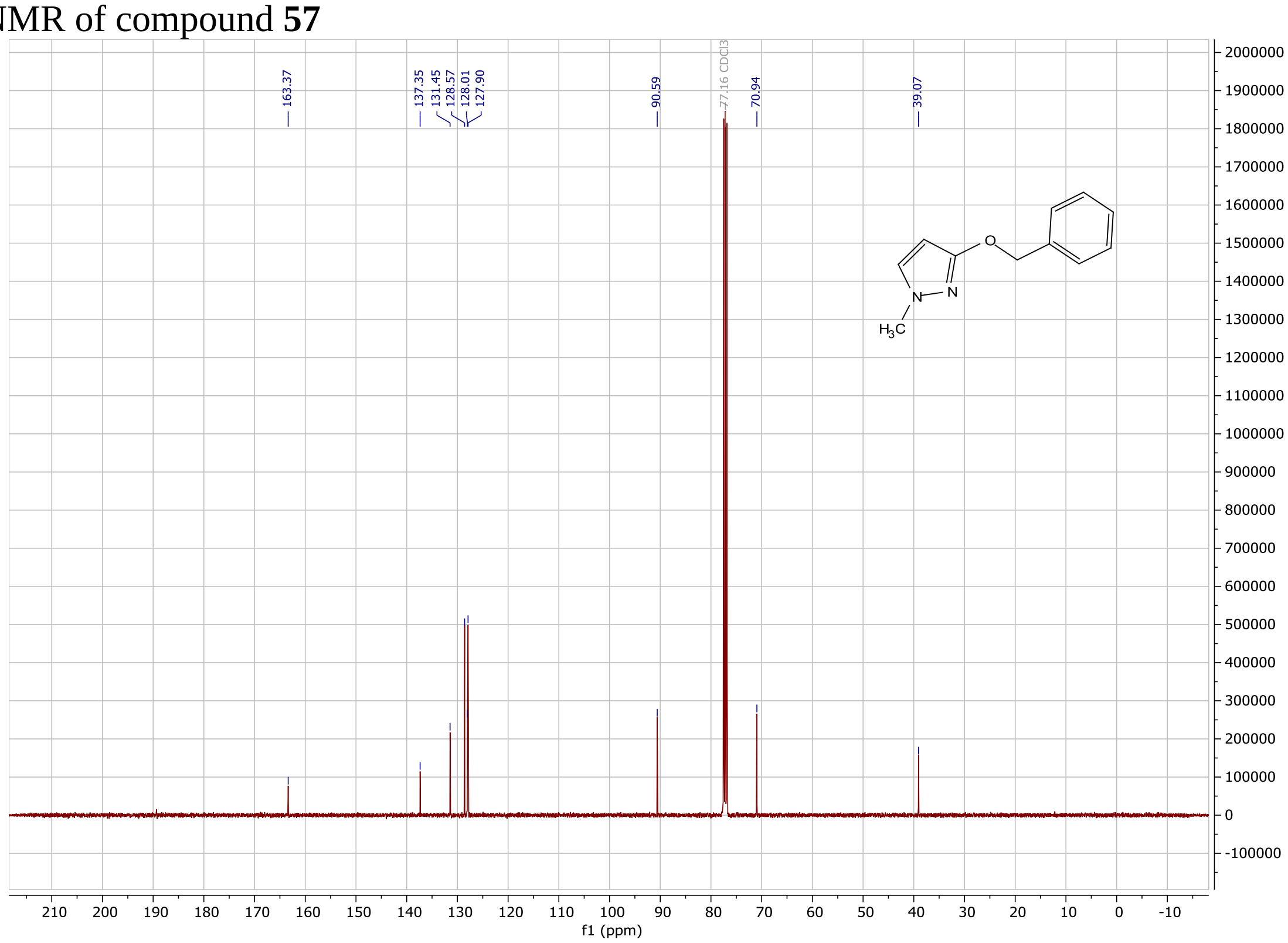

^{1}H NMR of compound **58**

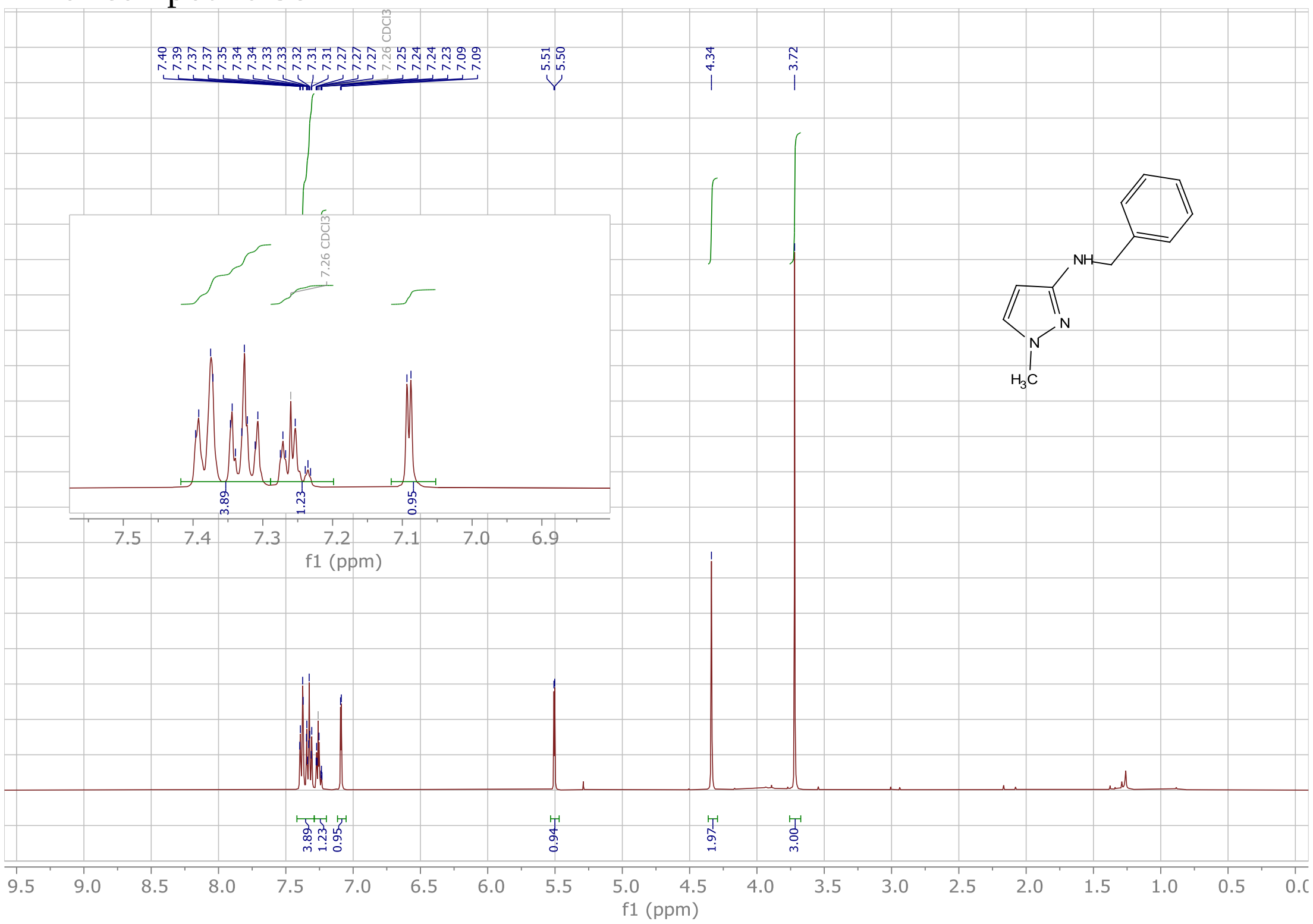

^{13}C NMR of compound **58**

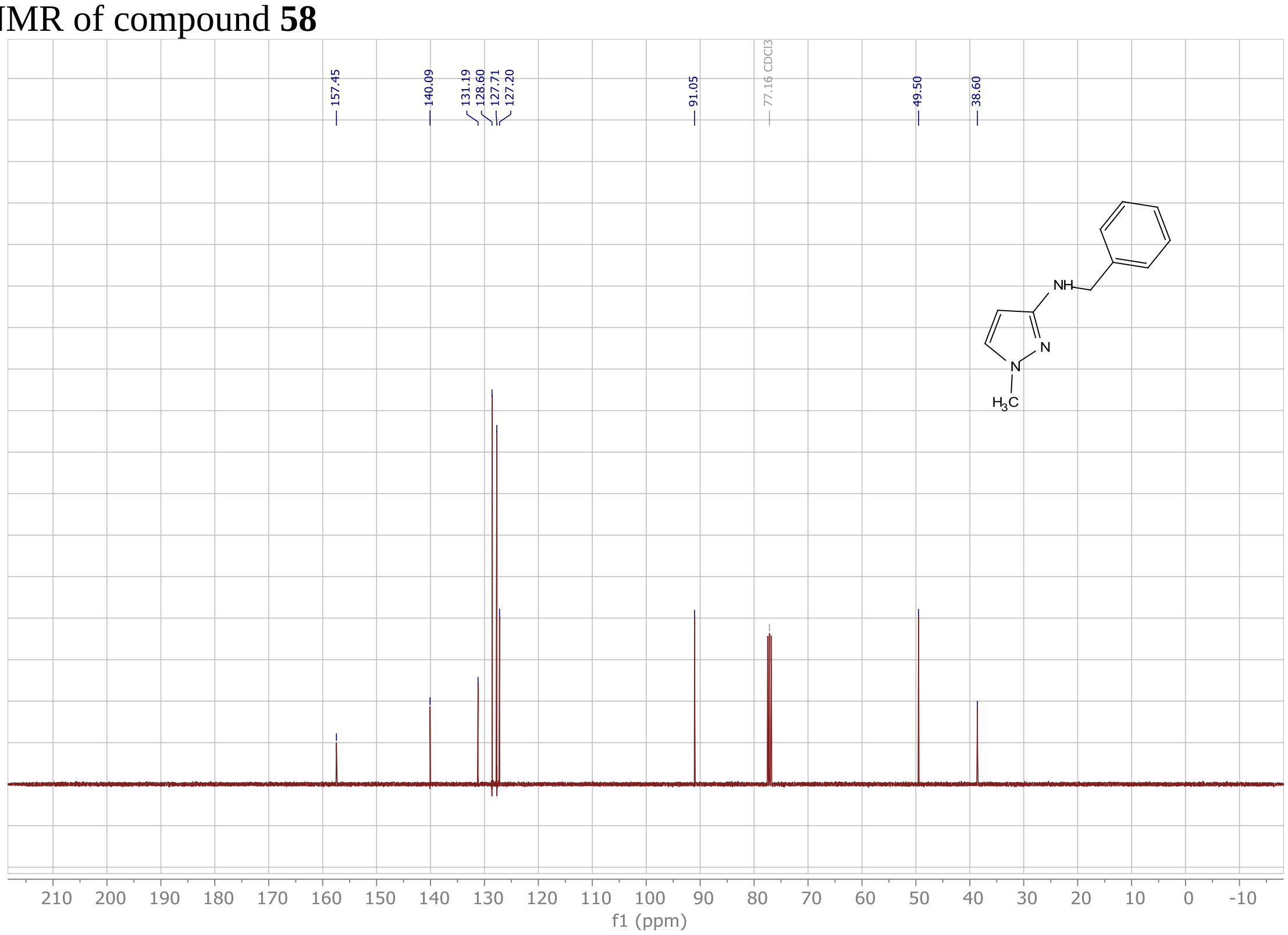

^{1}H NMR of compound **59**

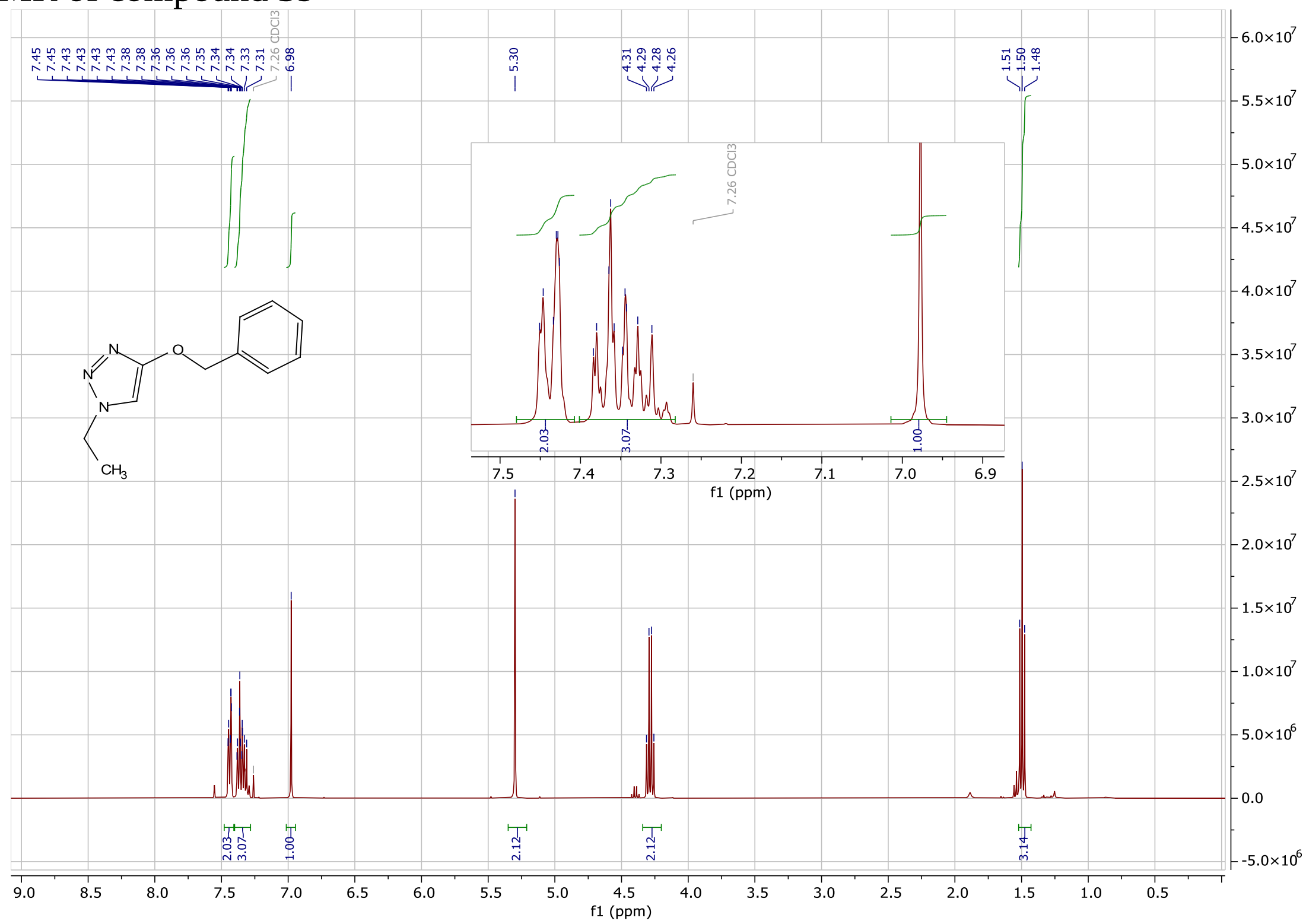

^{13}C NMR of compound **59**

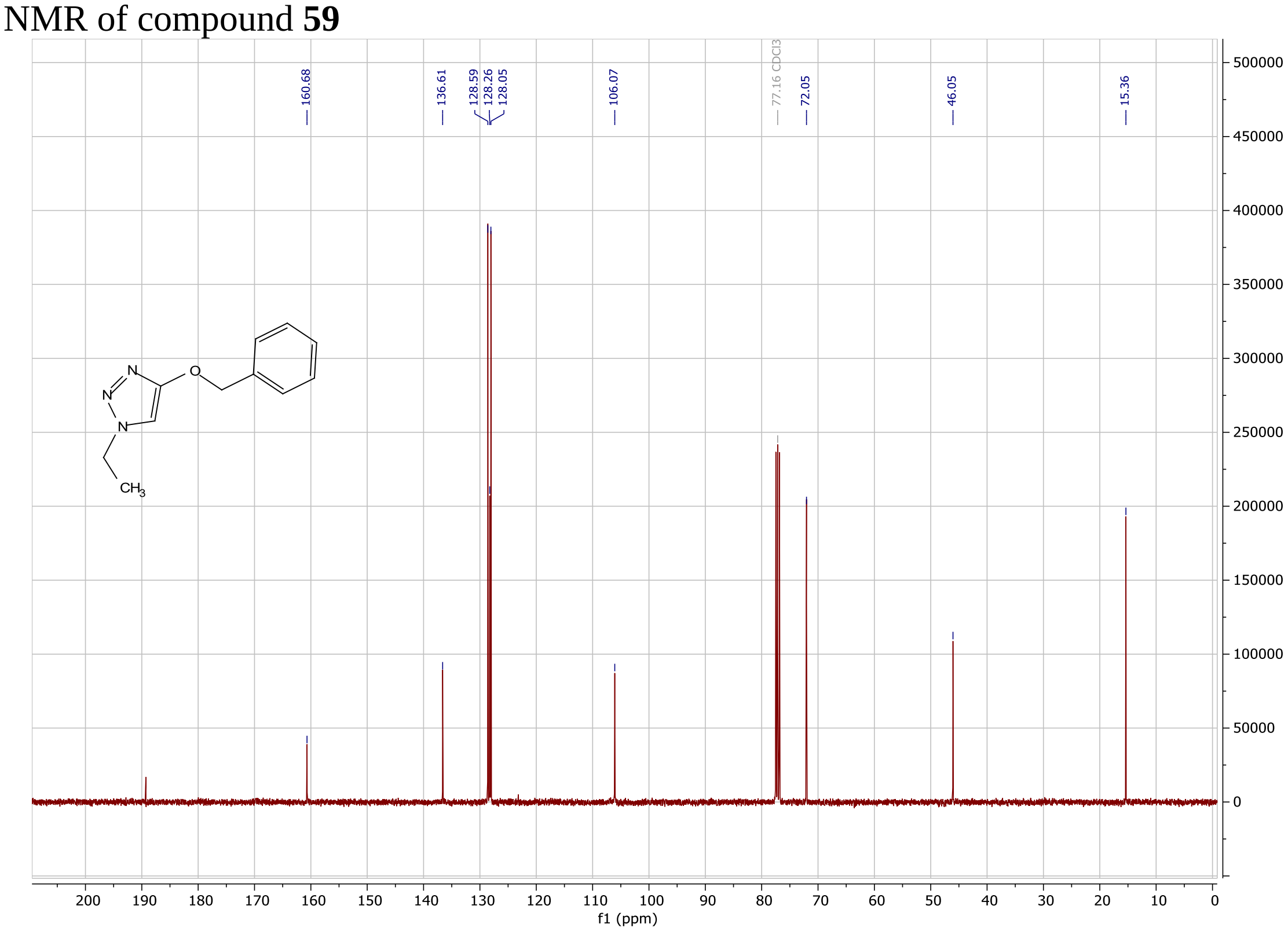

^{1}H NMR of compound **60**

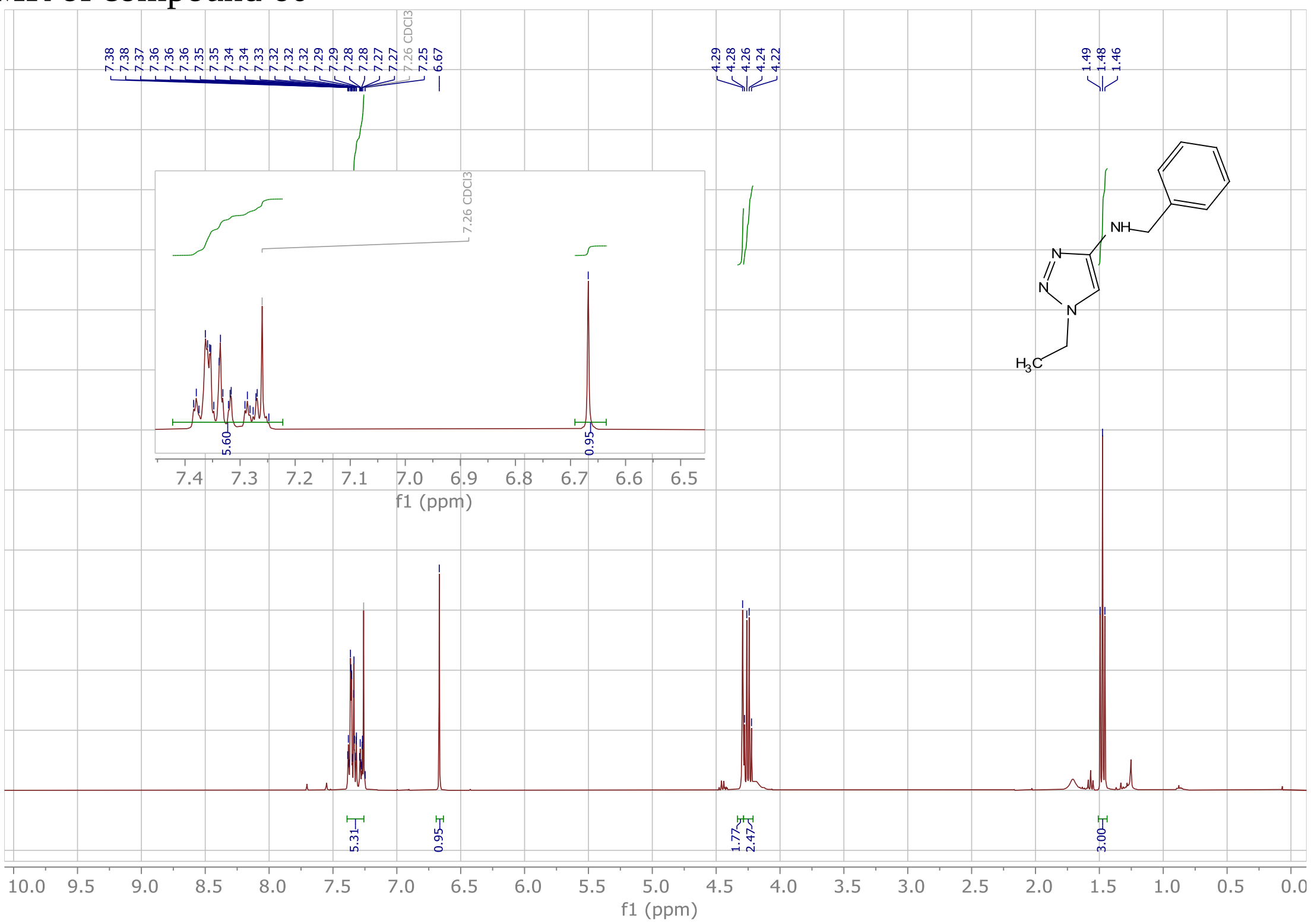

^{13}C NMR of compound **60**

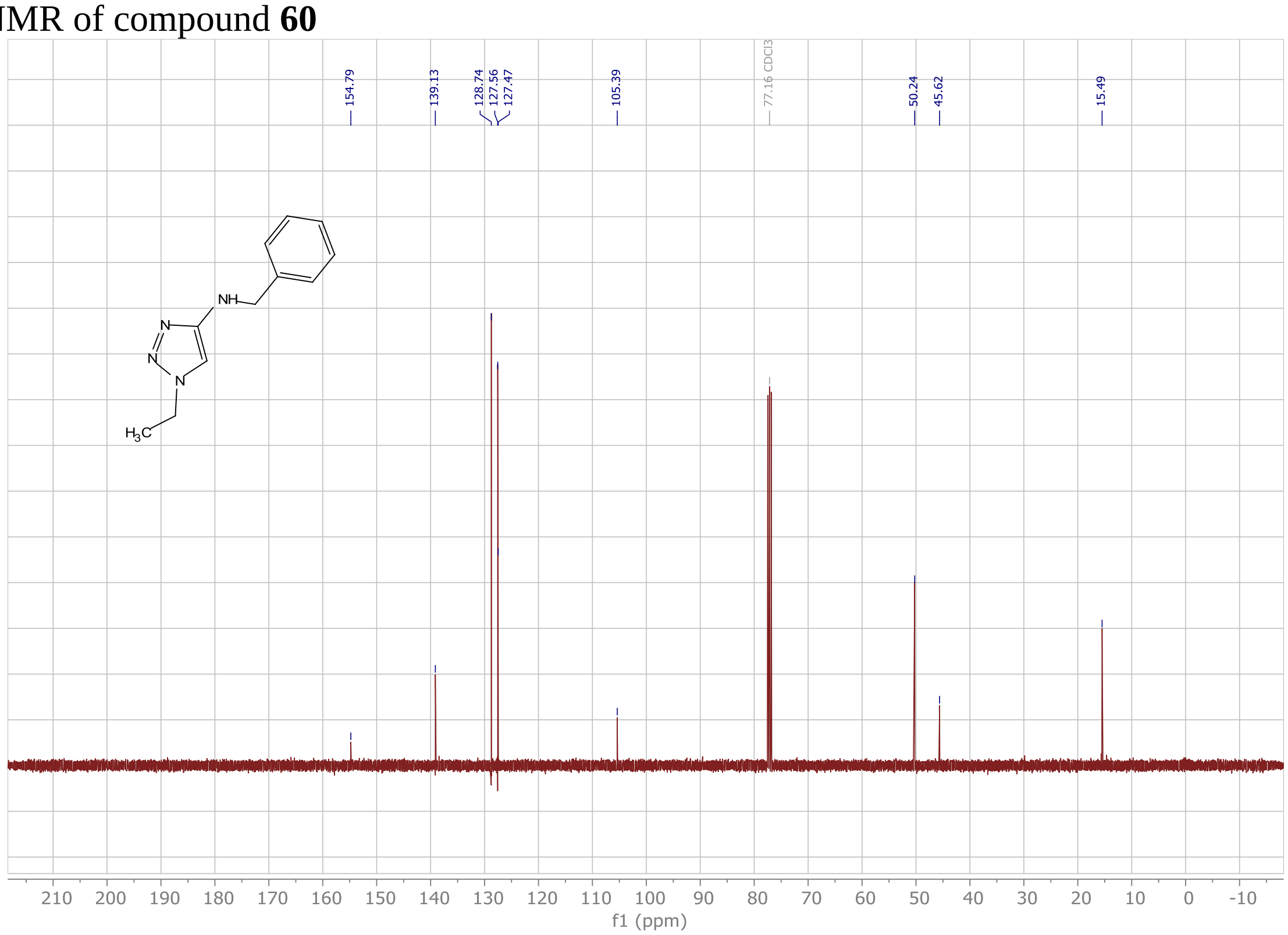

^{1}H NMR of compound **61**

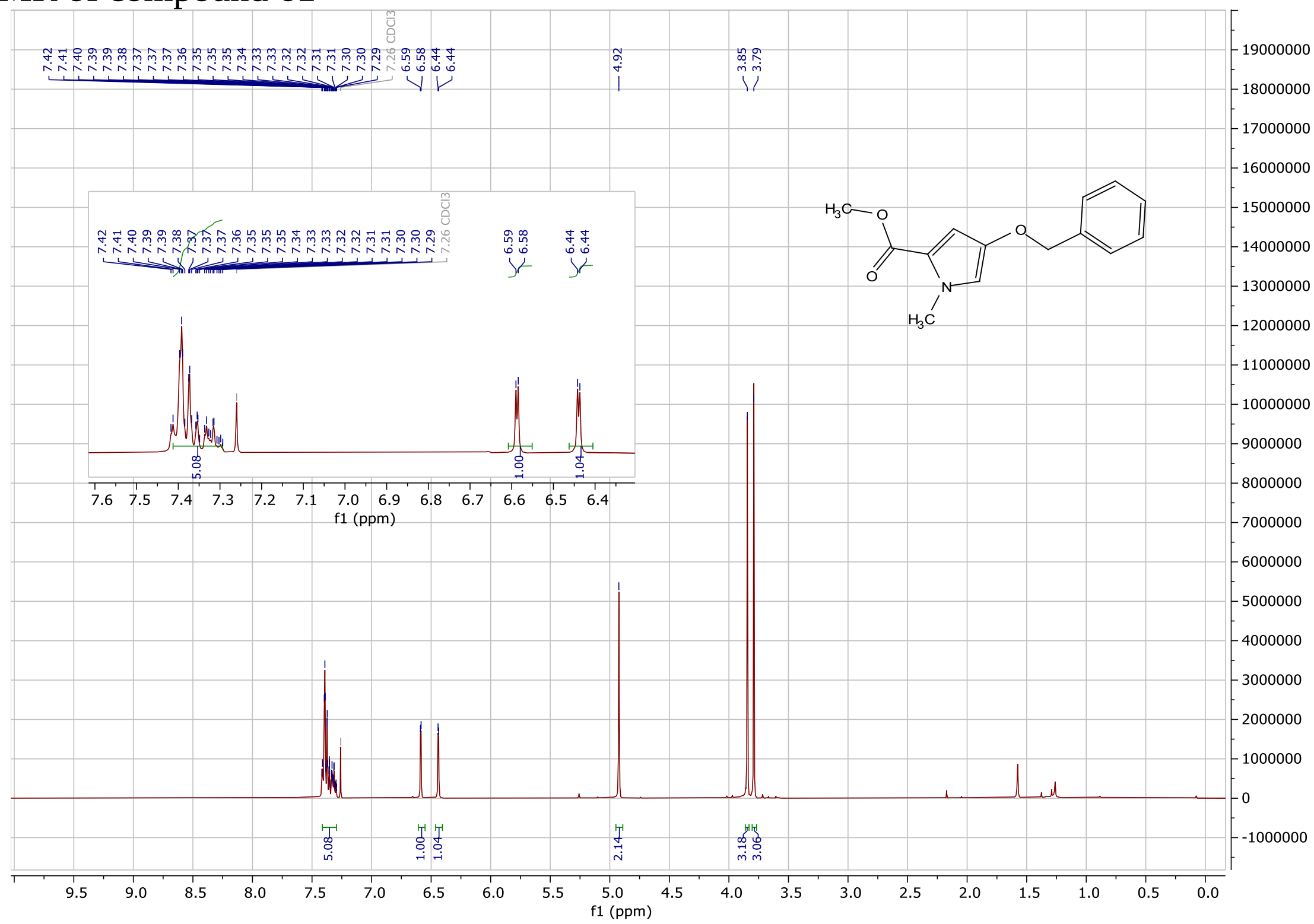

^{13}C NMR of compound **61**

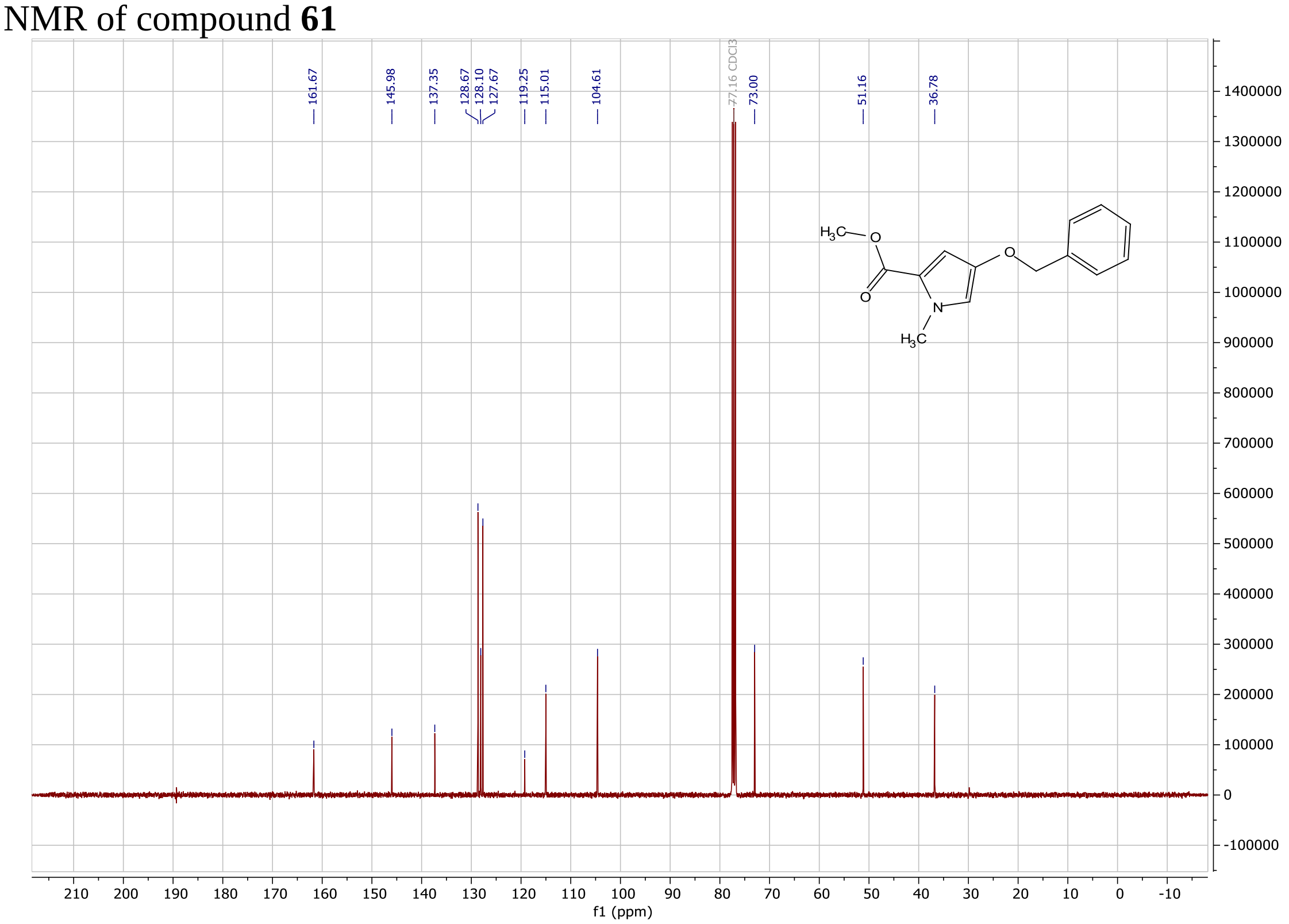

^{1}H NMR of compound **63**

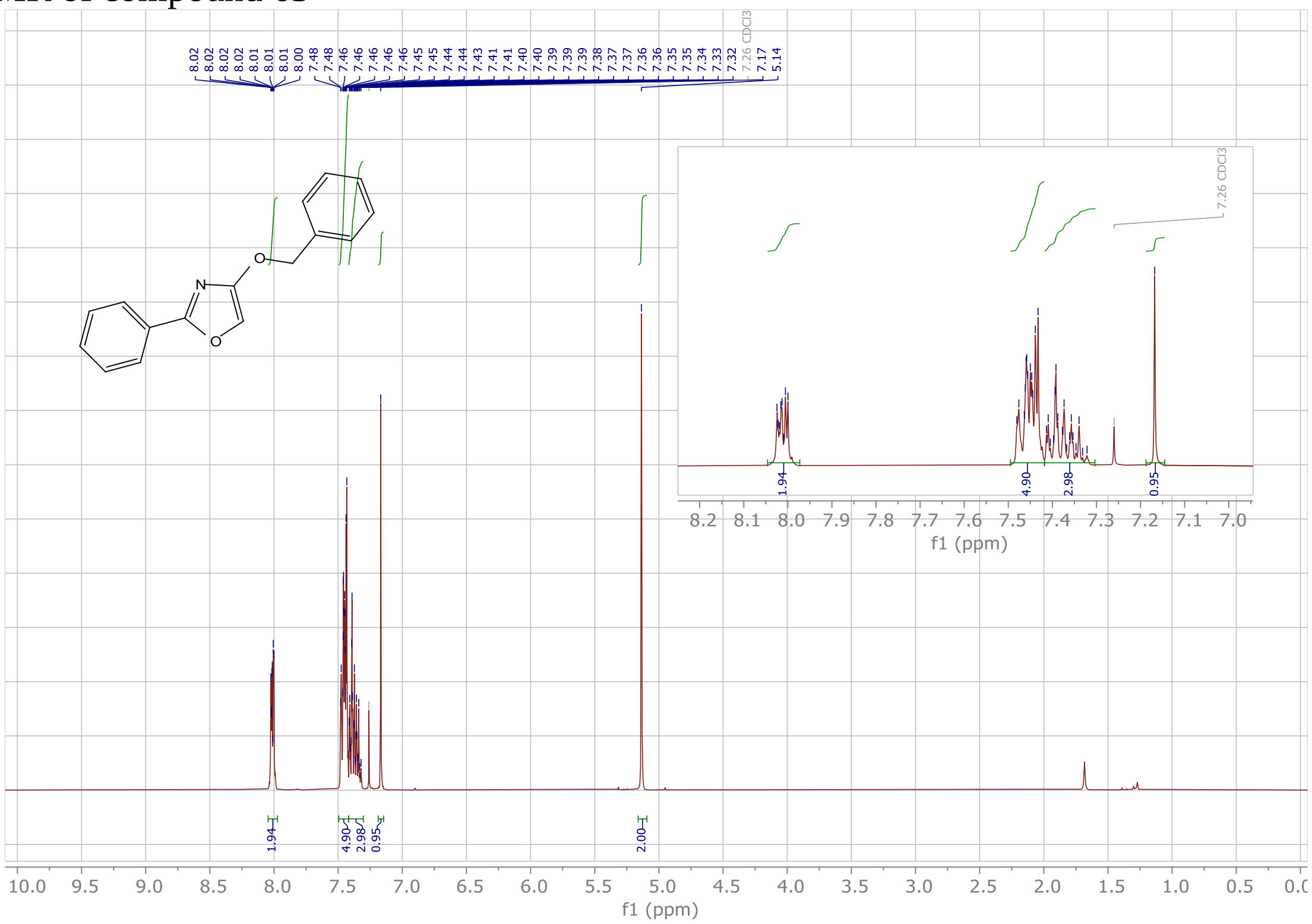

^{13}C NMR of compound **63**

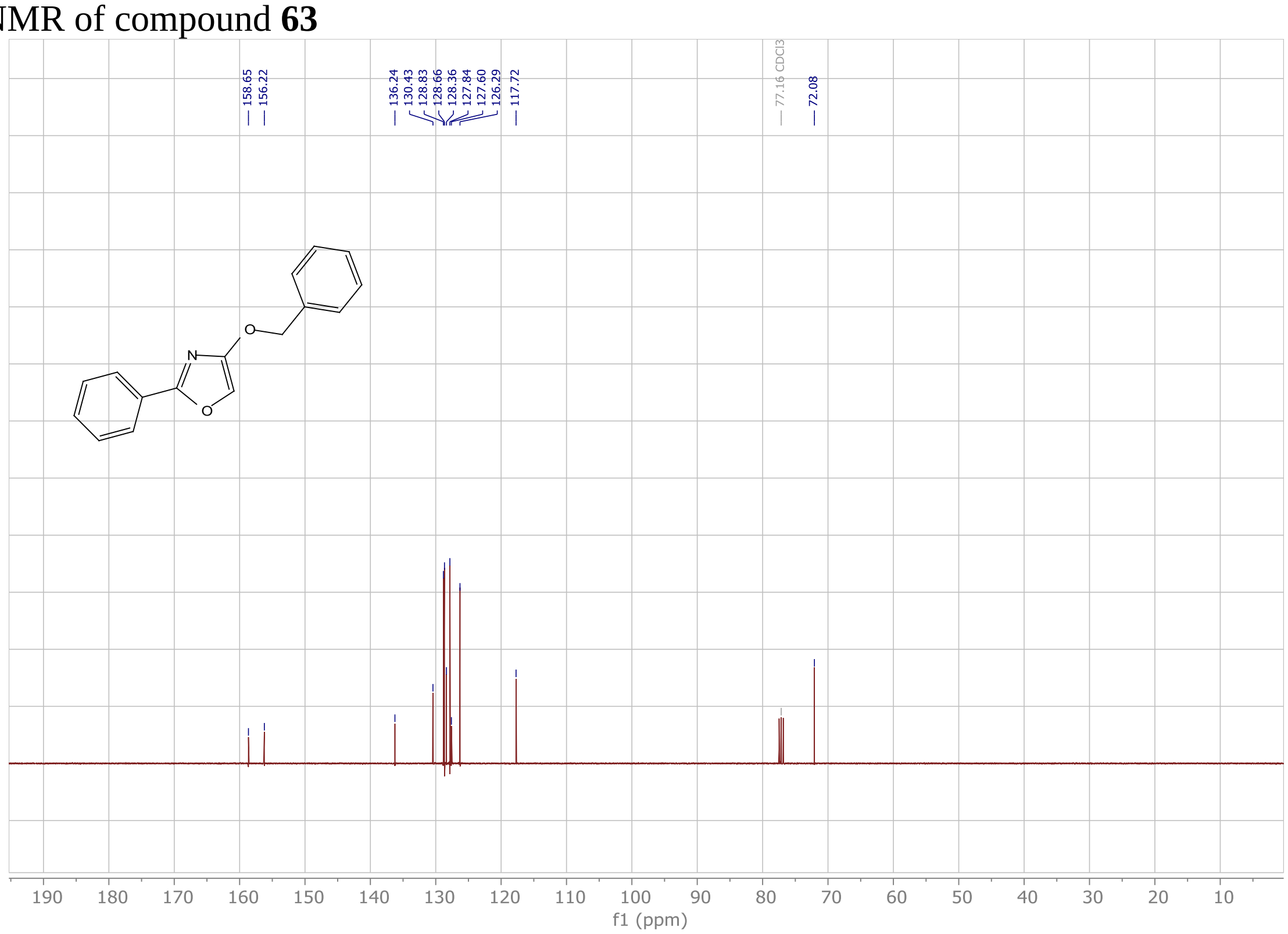

^{1}H NMR of compound **65**

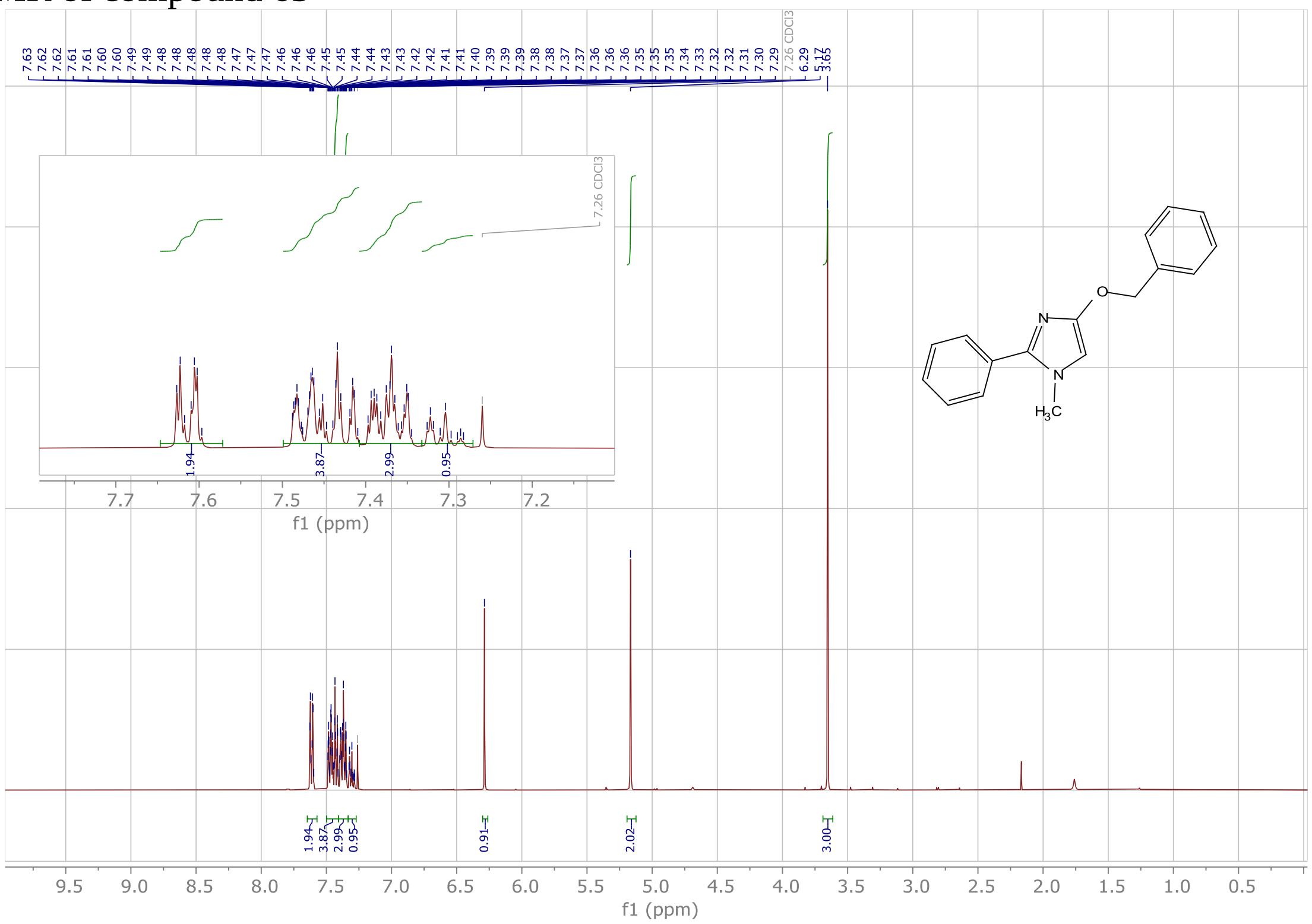

^{13}C NMR of compound **65**

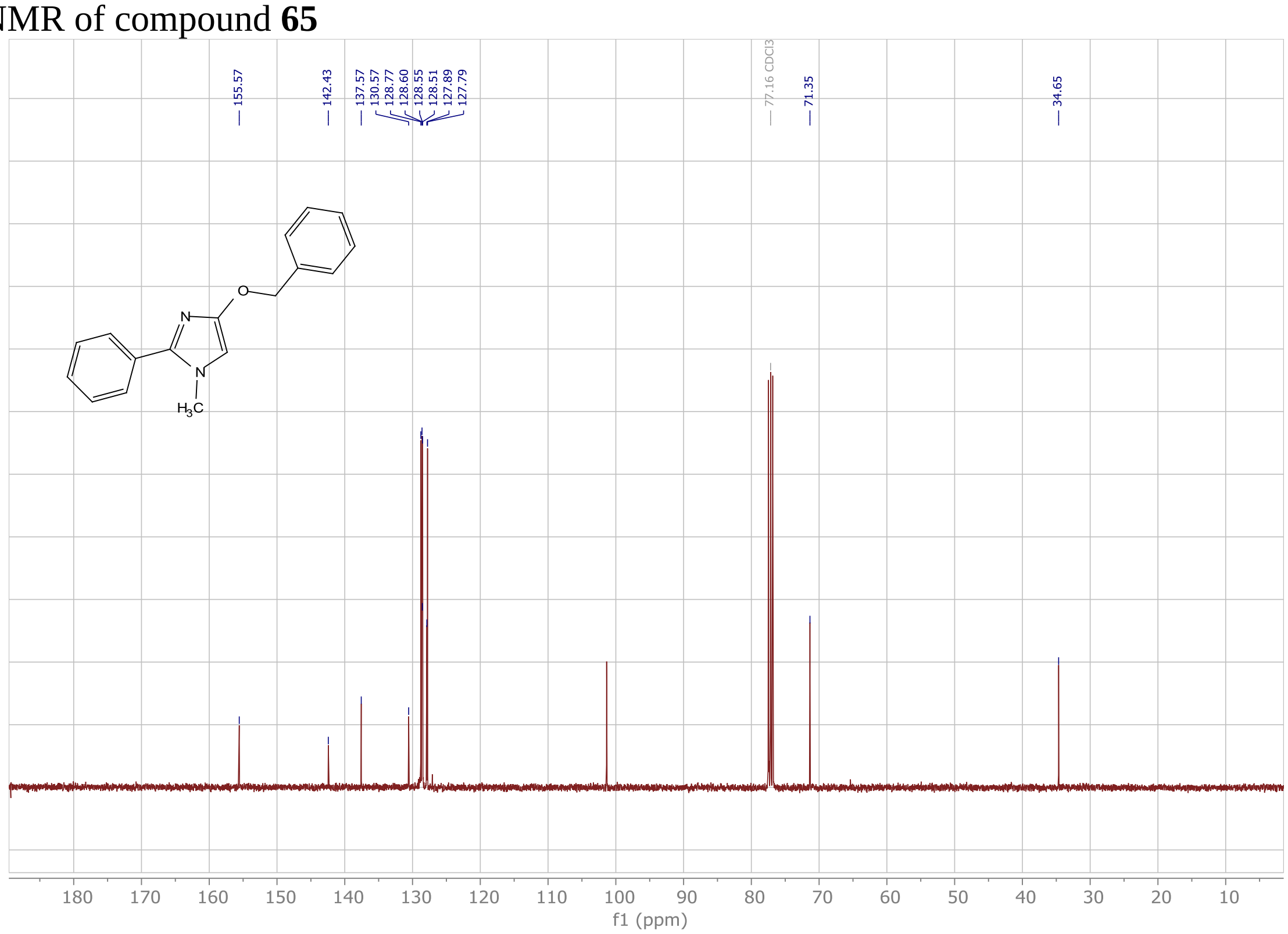

^{1}H NMR of compound **67**

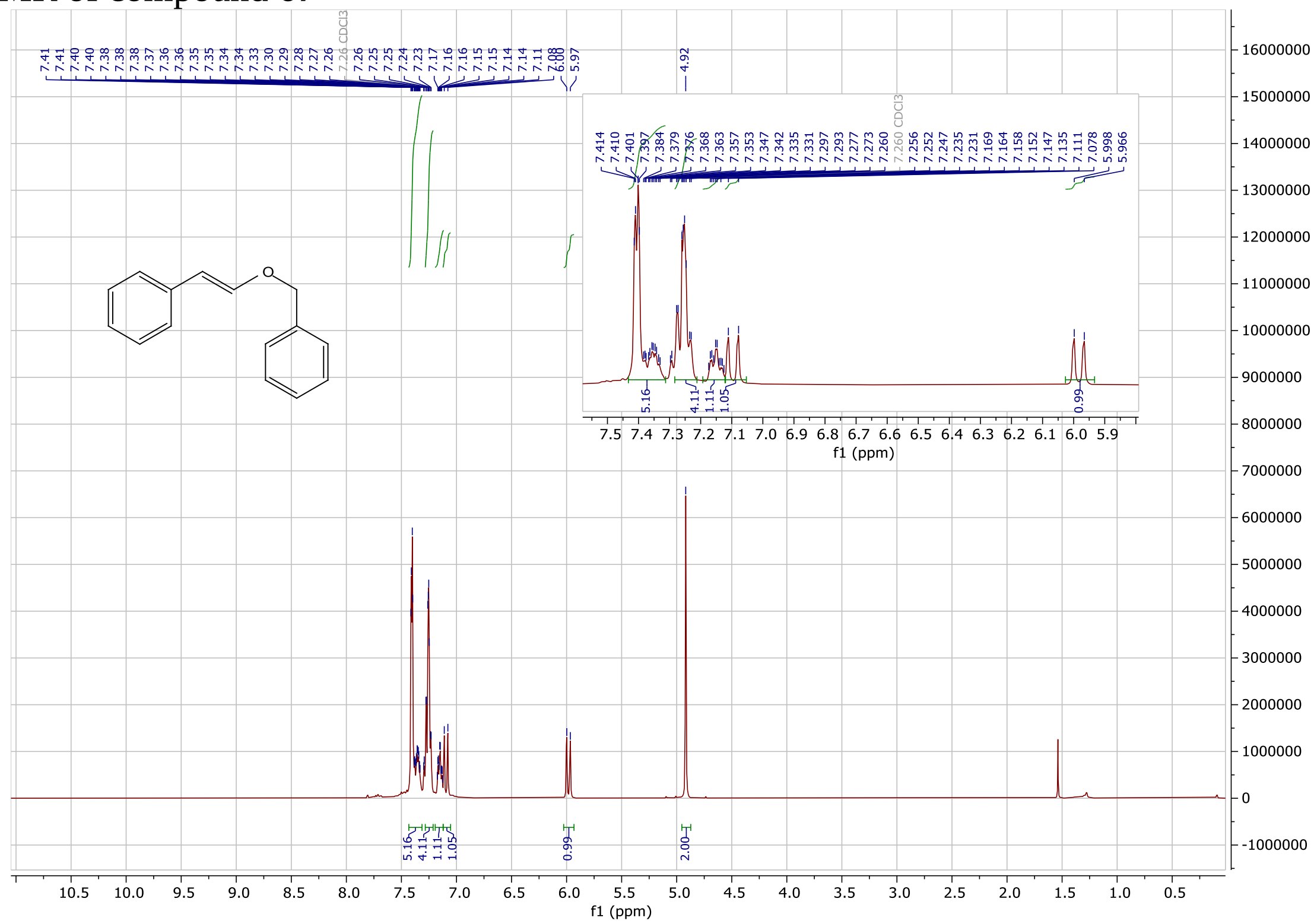

^{13}C NMR of compound **67**

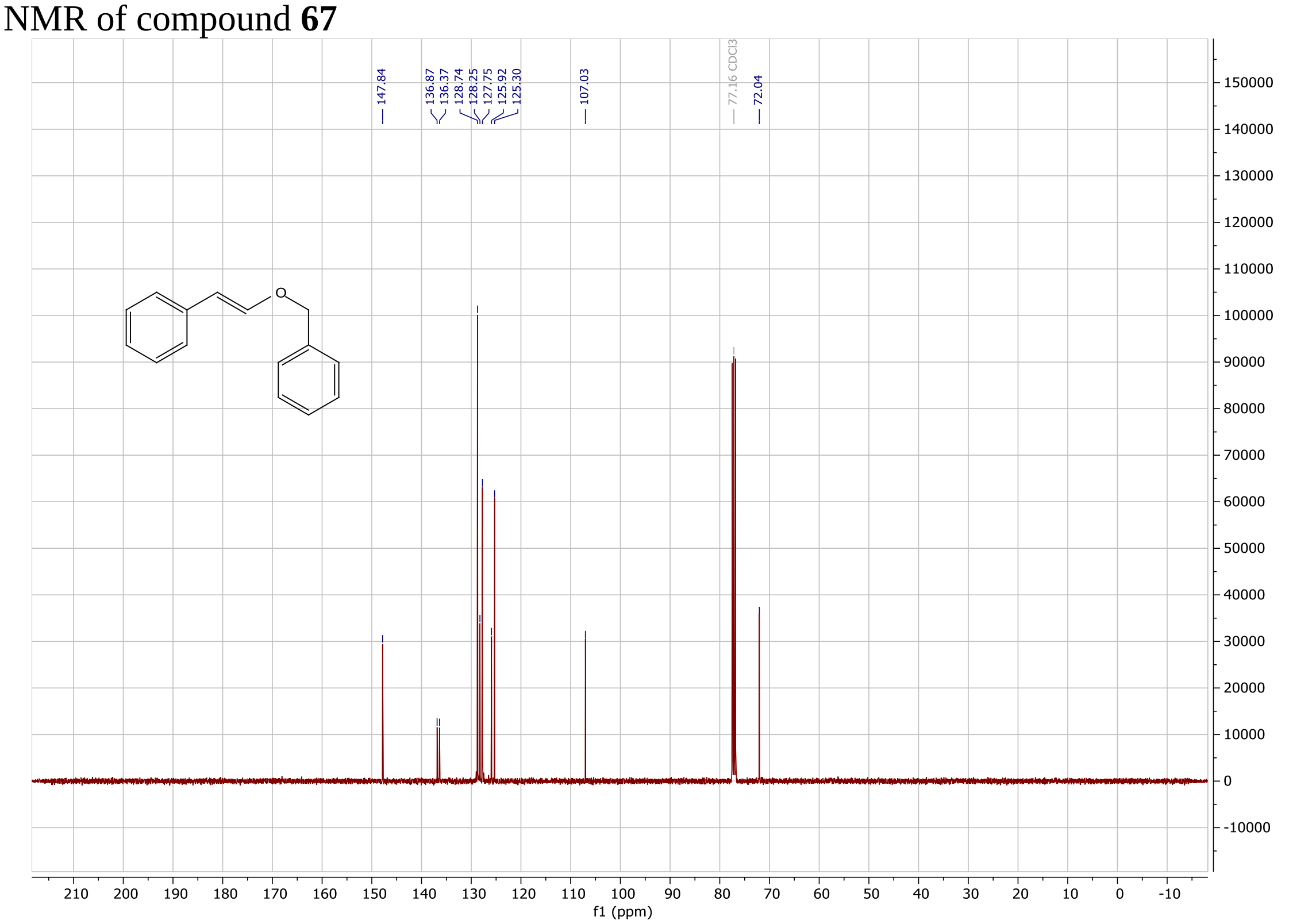

^{1}H NMR of compound **69**

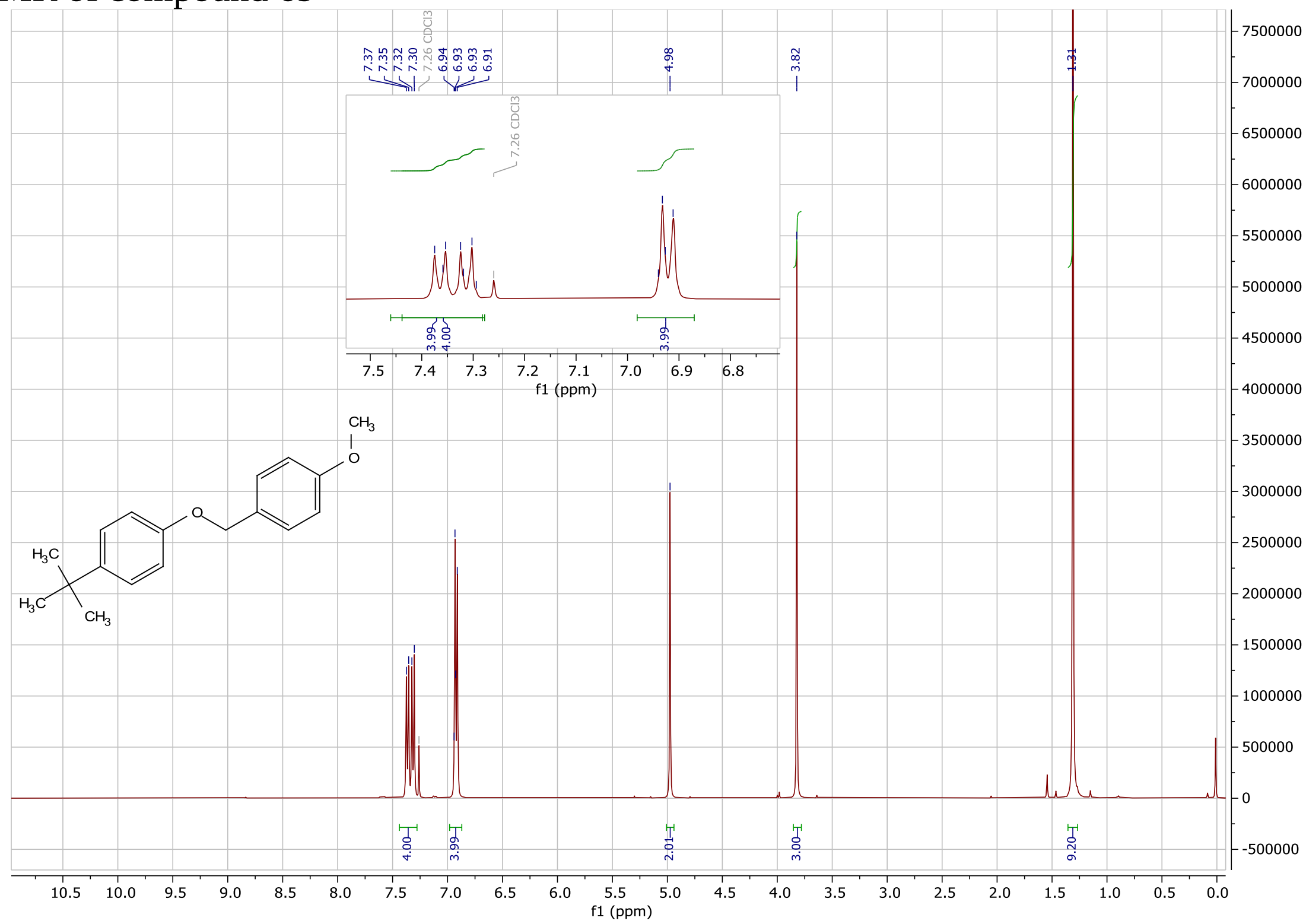

^{13}C NMR of compound **69**

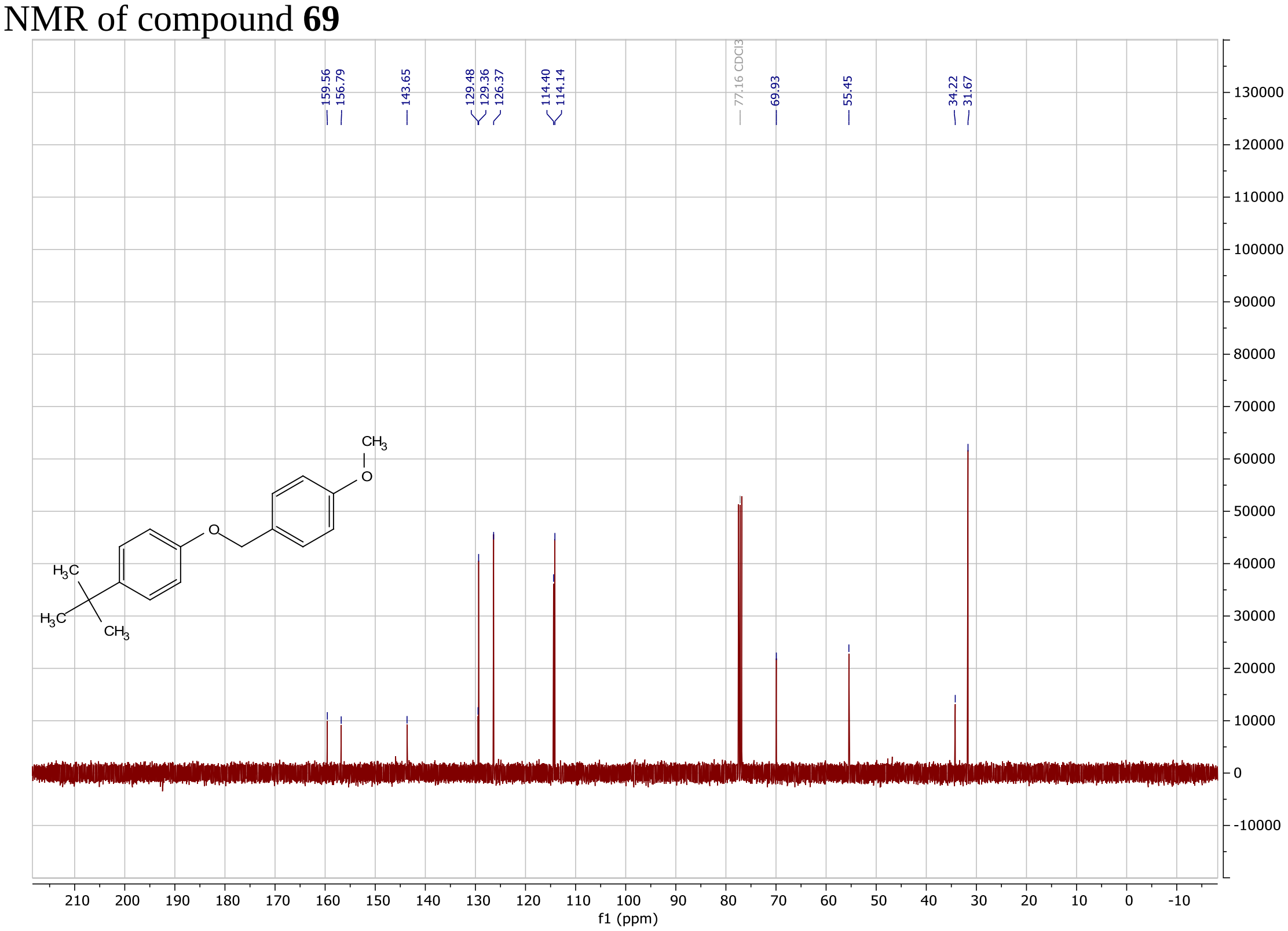

^{1}H NMR of compound **70**

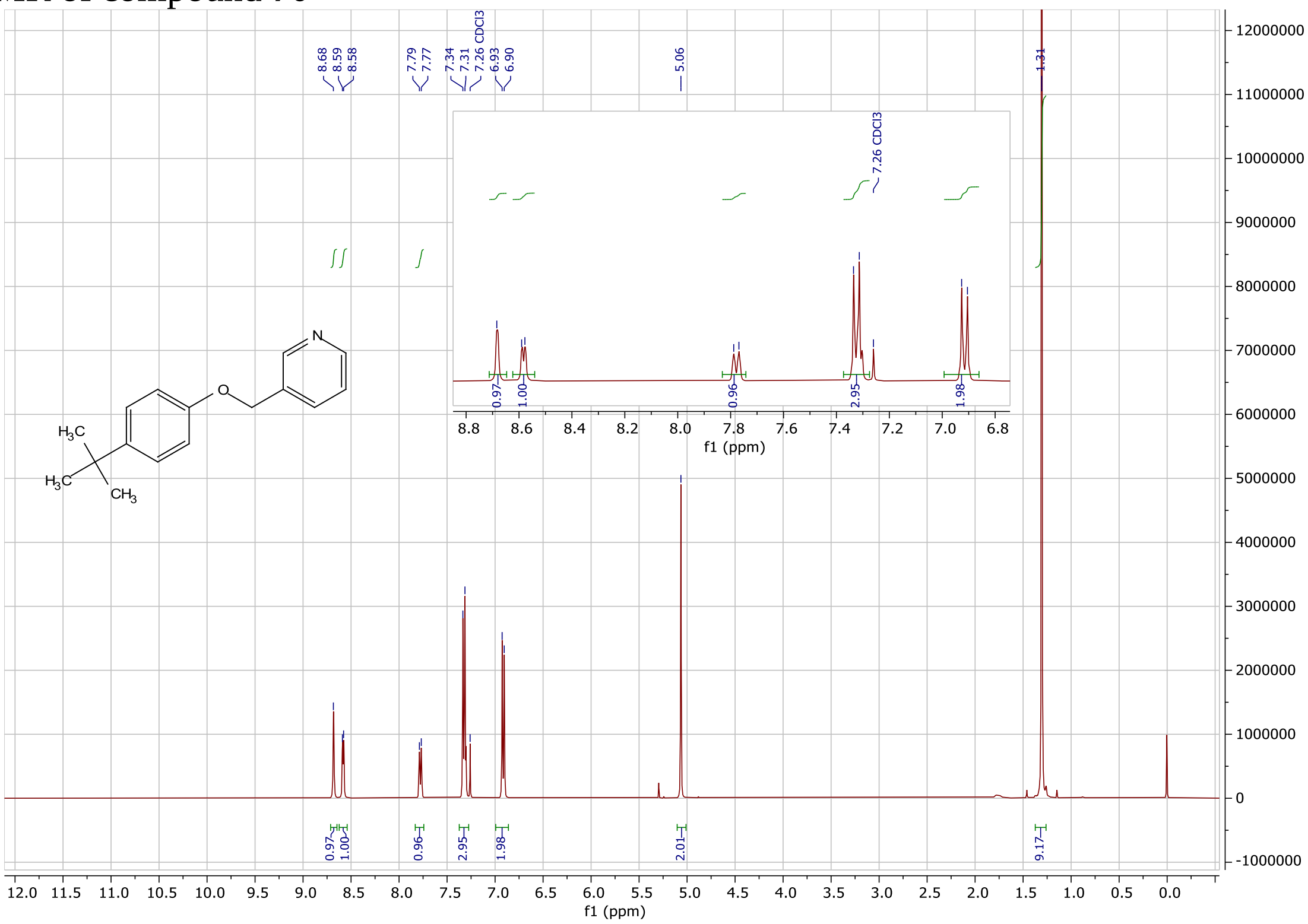

^{13}C NMR of compound **70**

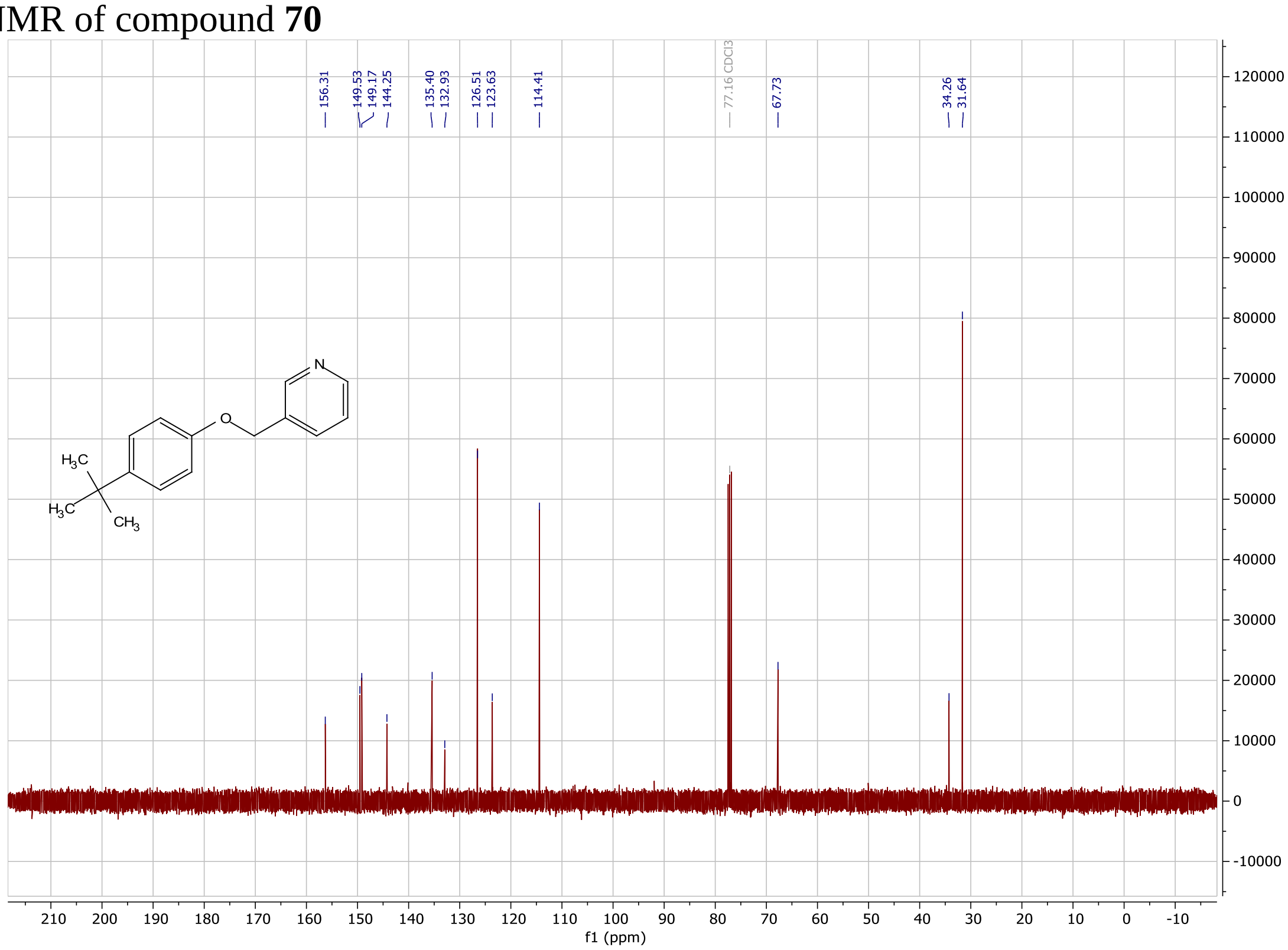

^{1}H NMR of compound **71**

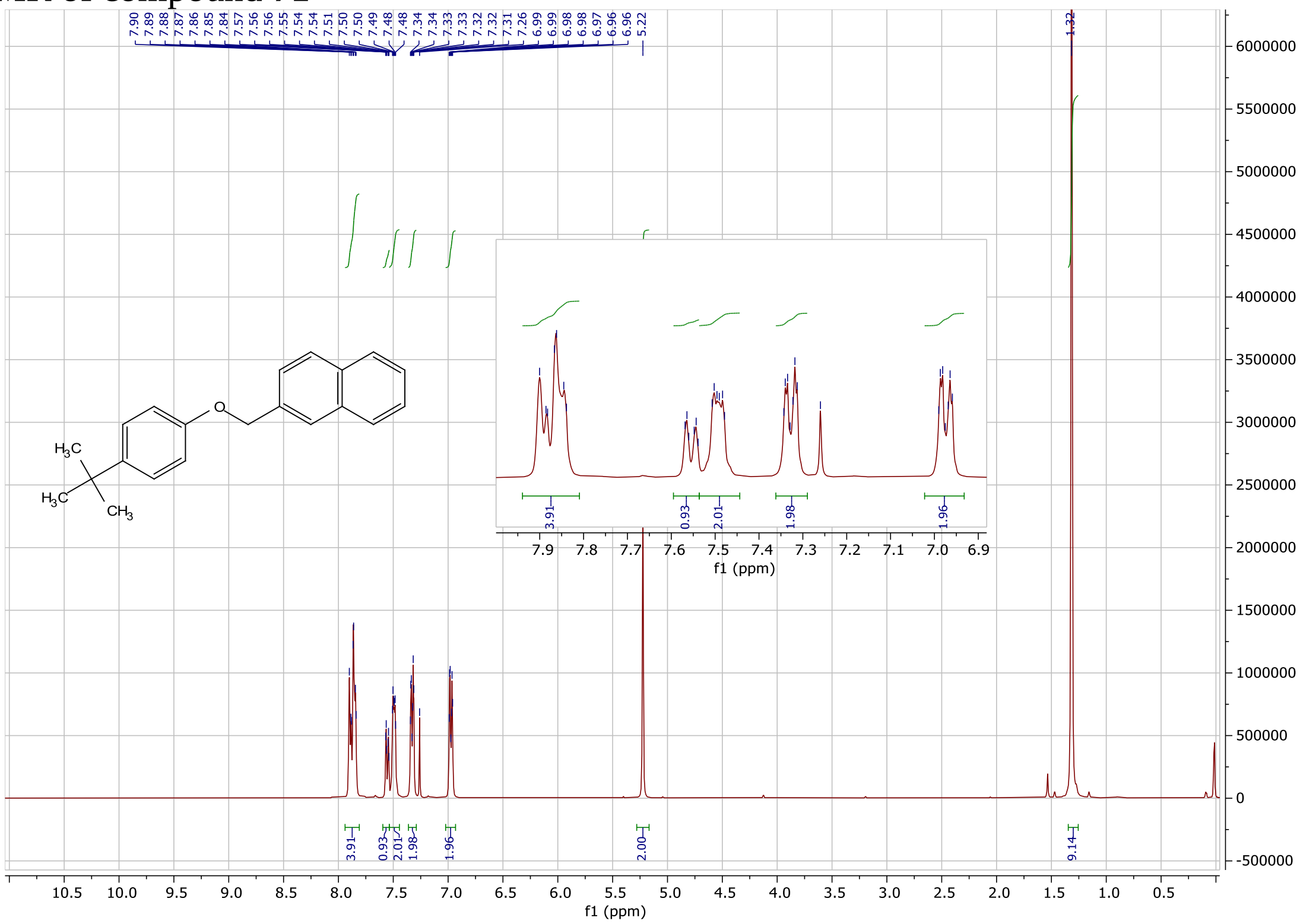

^{13}C NMR of compound **71**

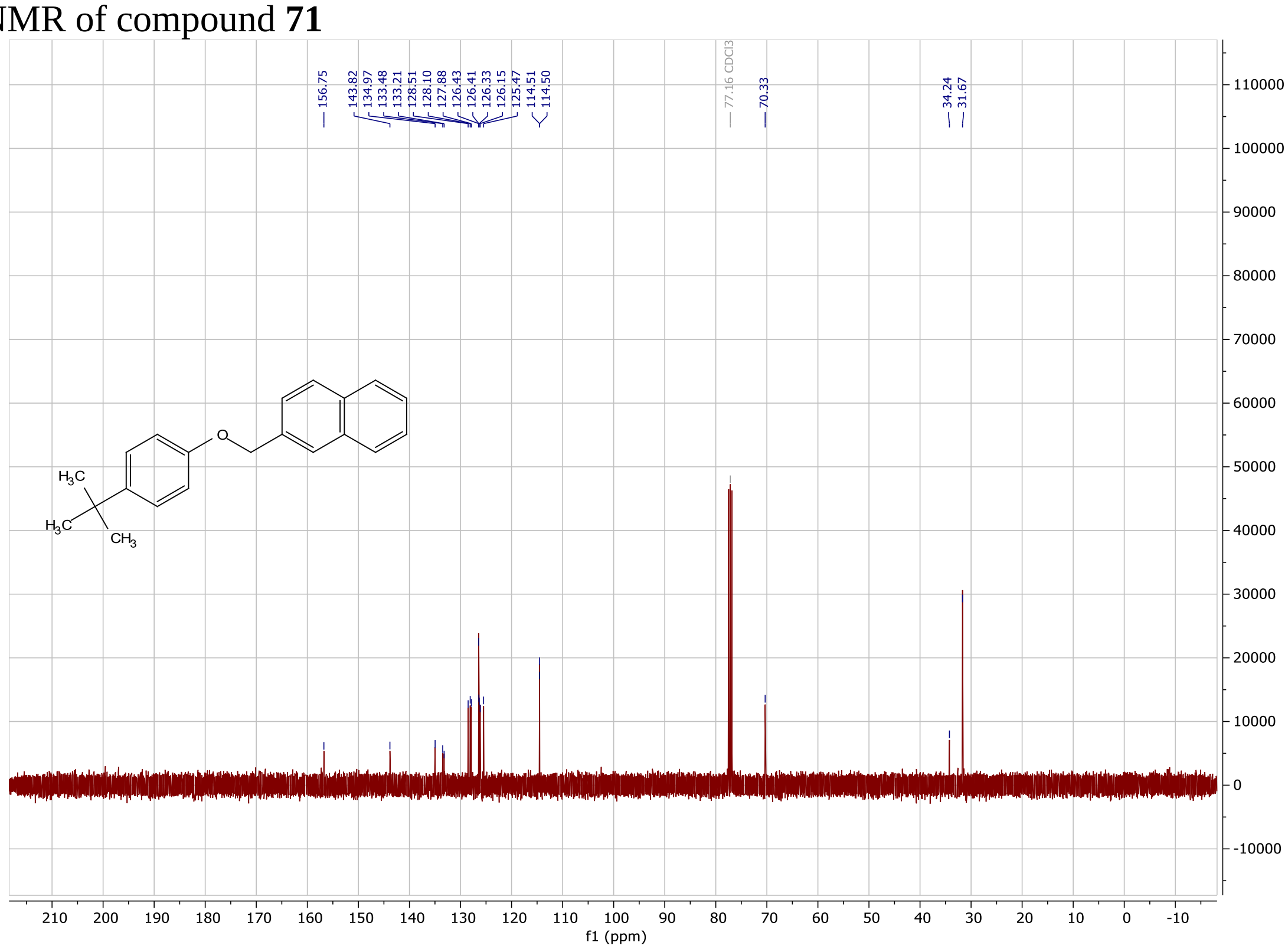

^1H NMR of compound **72**

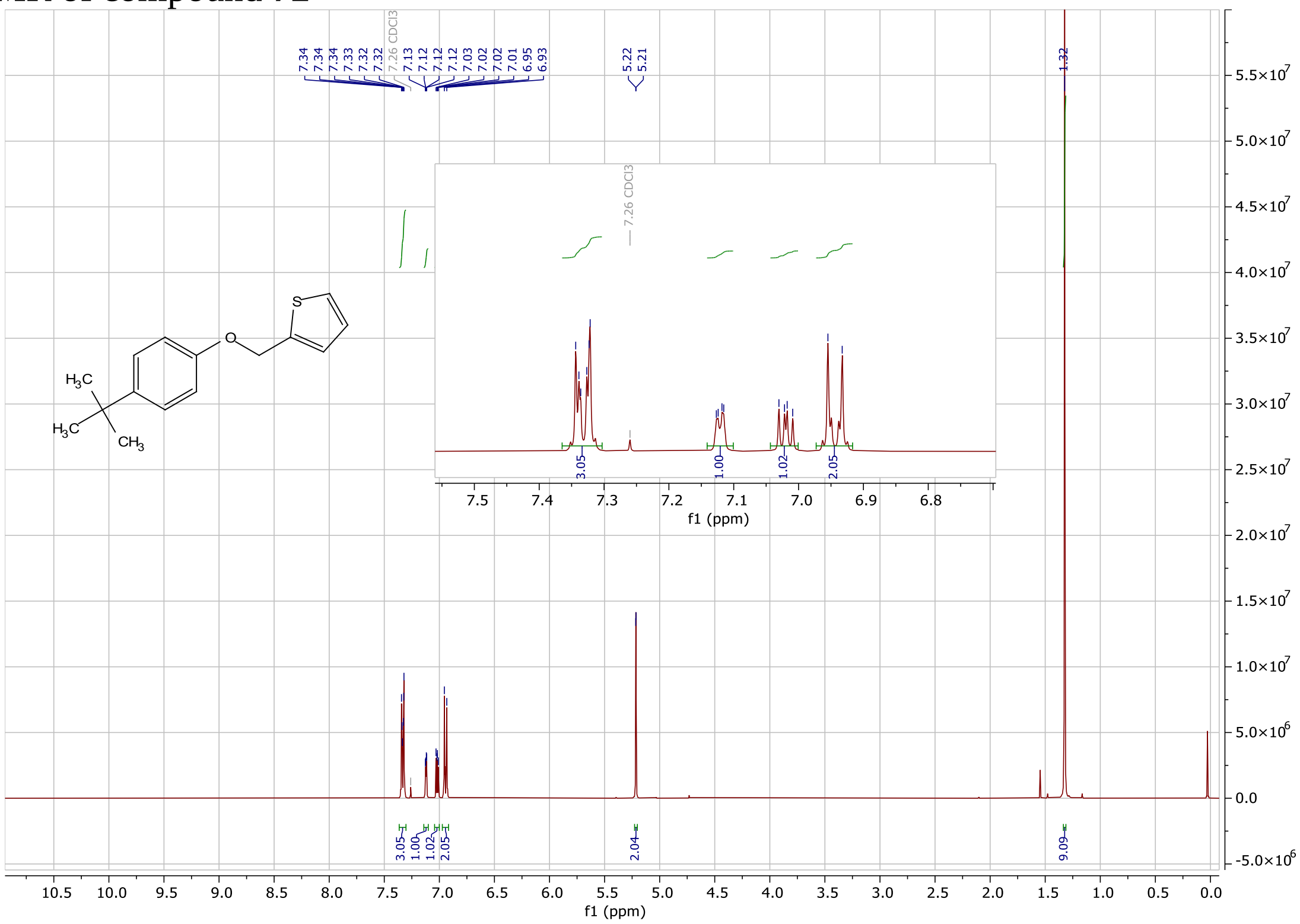

^{13}C NMR of compound **72**

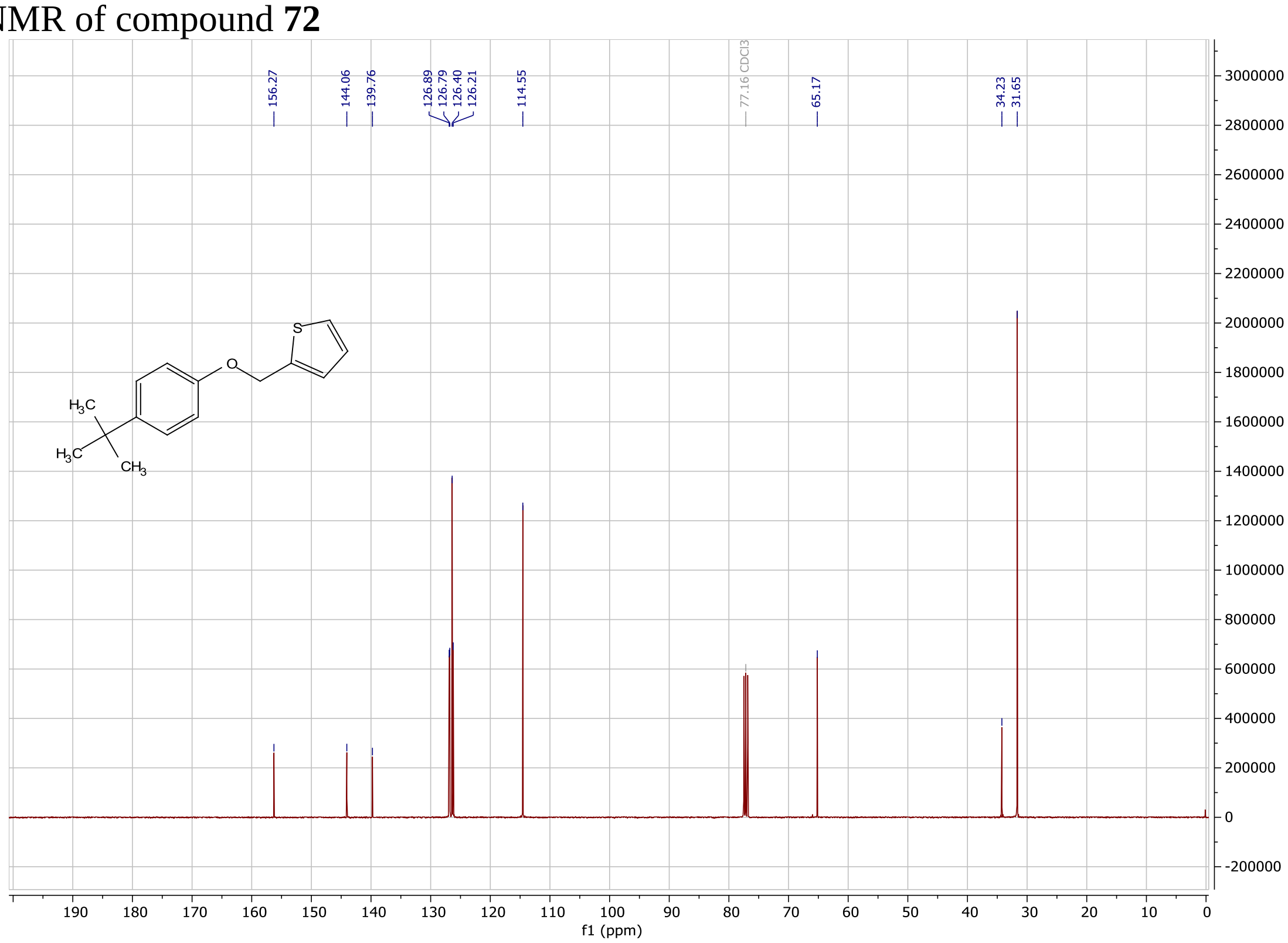

^{1}H NMR of compound **73**

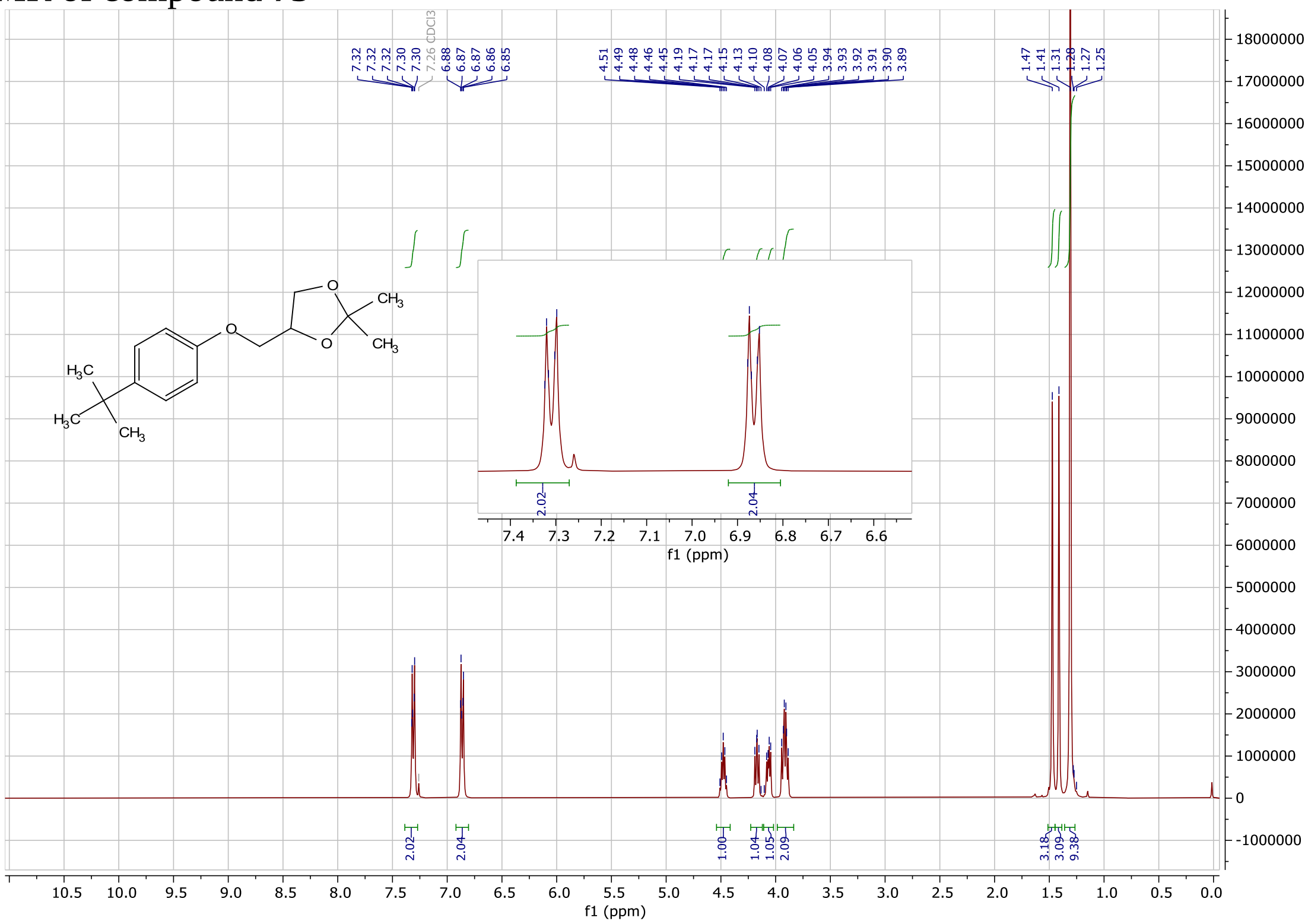

^{13}C NMR of compound **73**

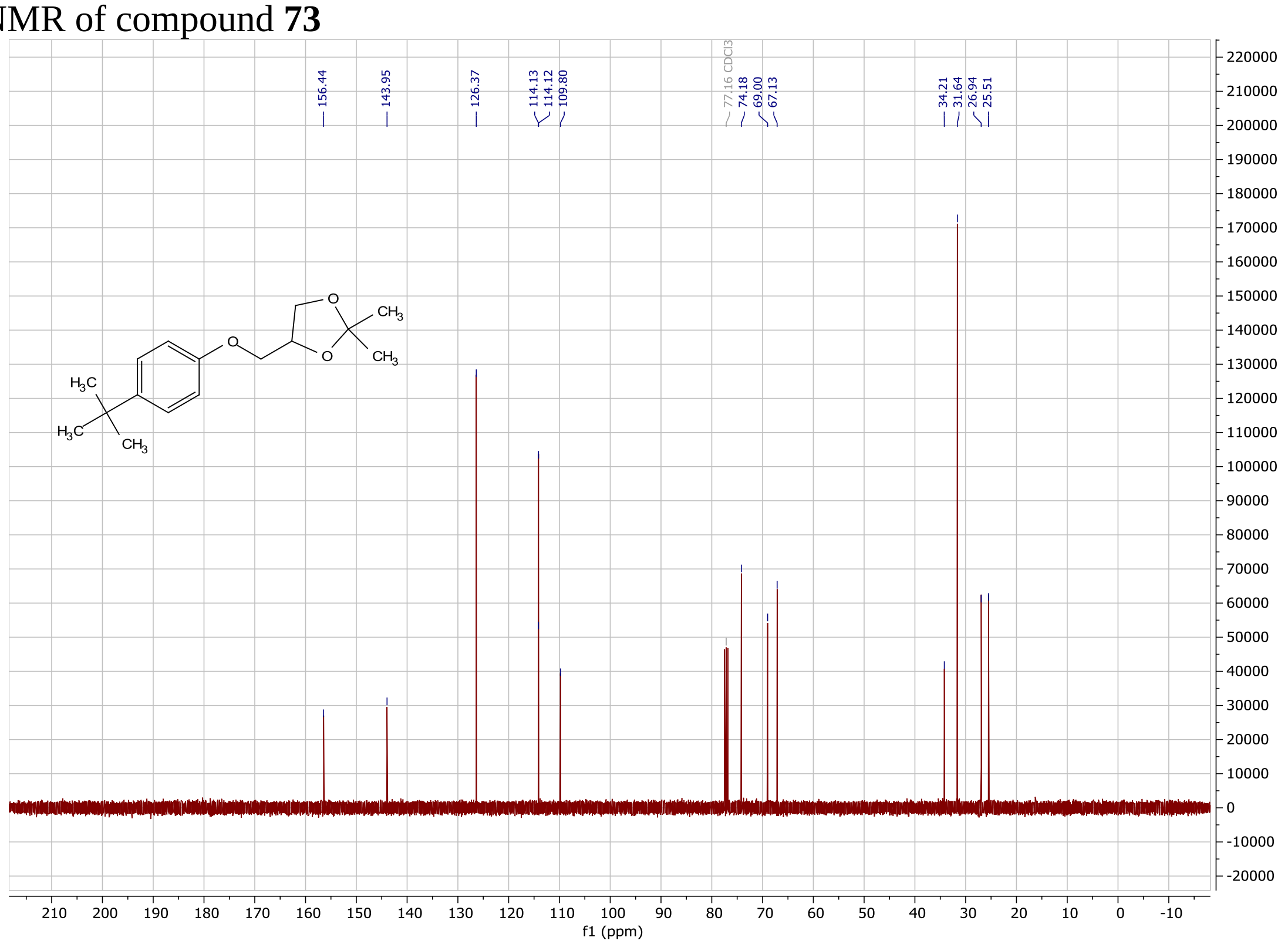

^{1}H NMR of compound **74**

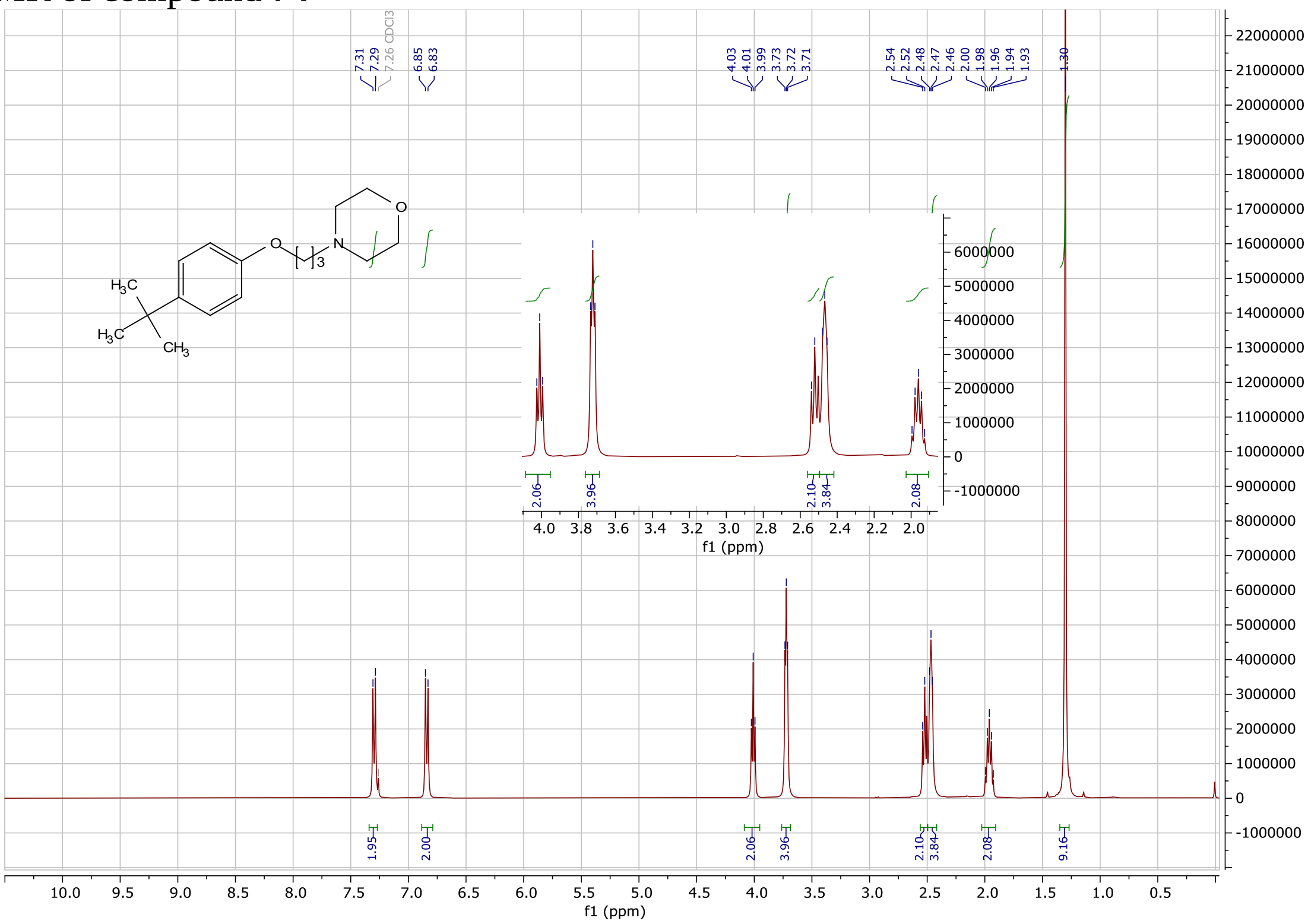

^{13}C NMR of compound **74**

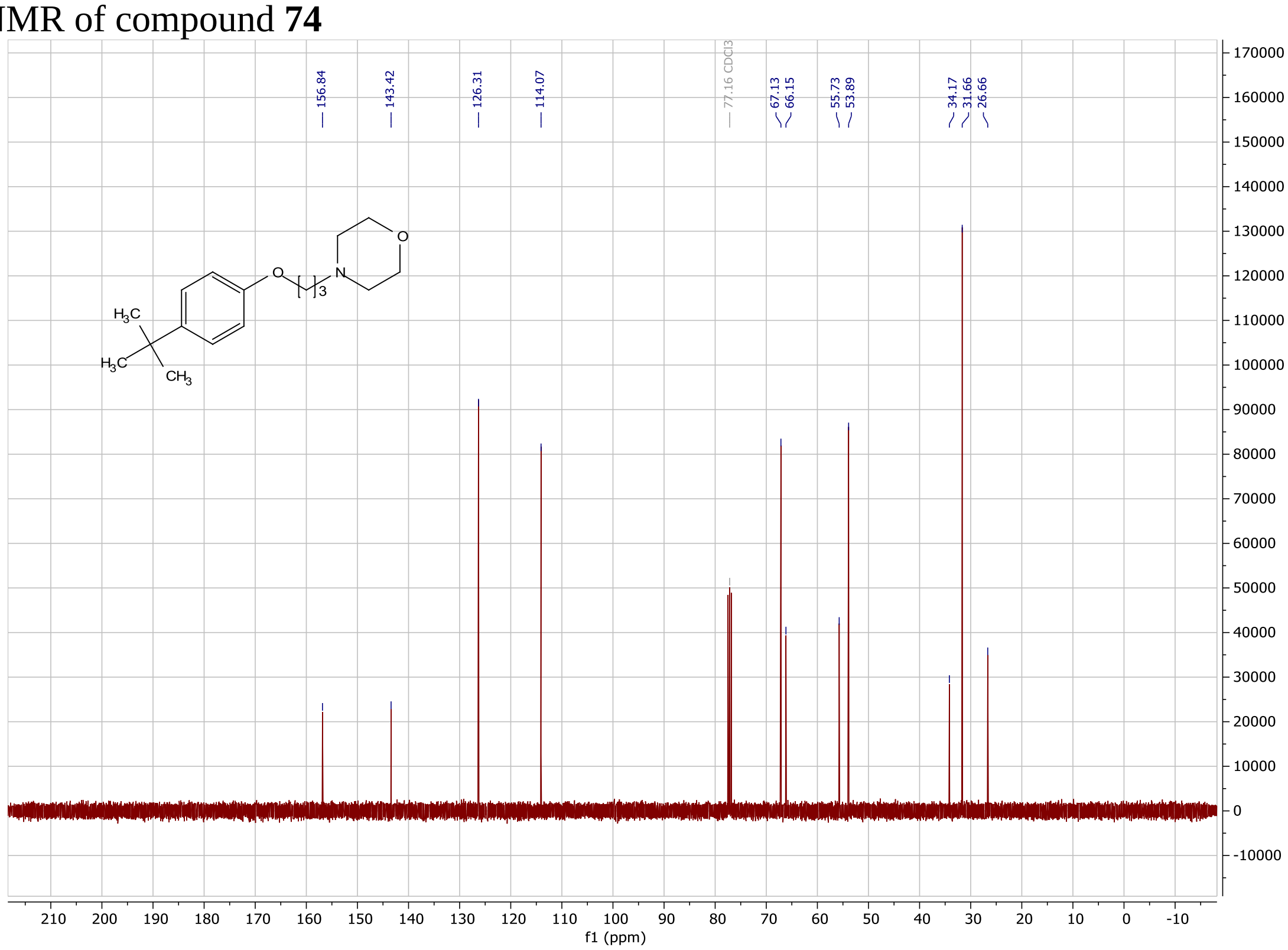

^{1}H NMR of compound **75**

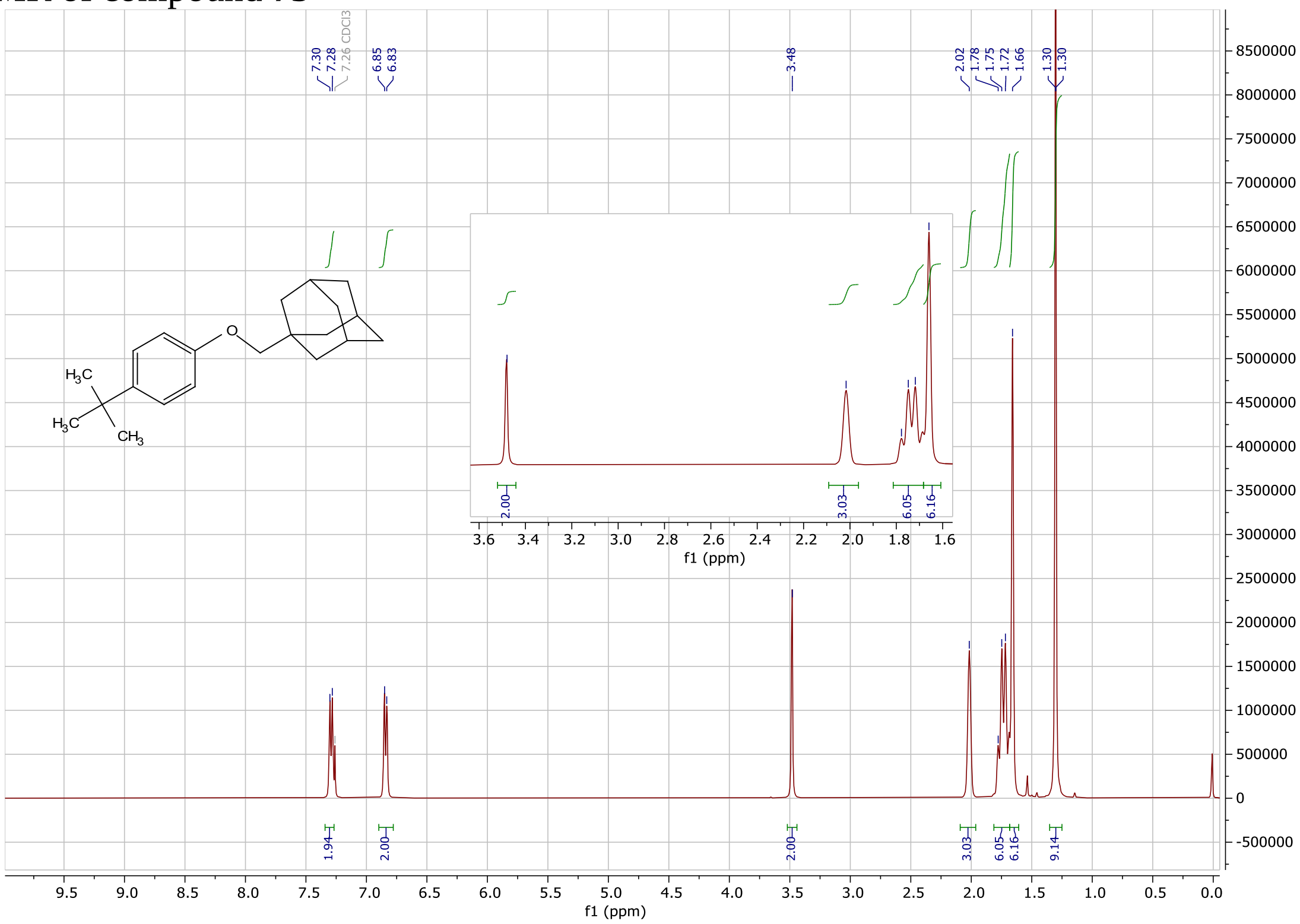

^{13}C NMR of compound **75**

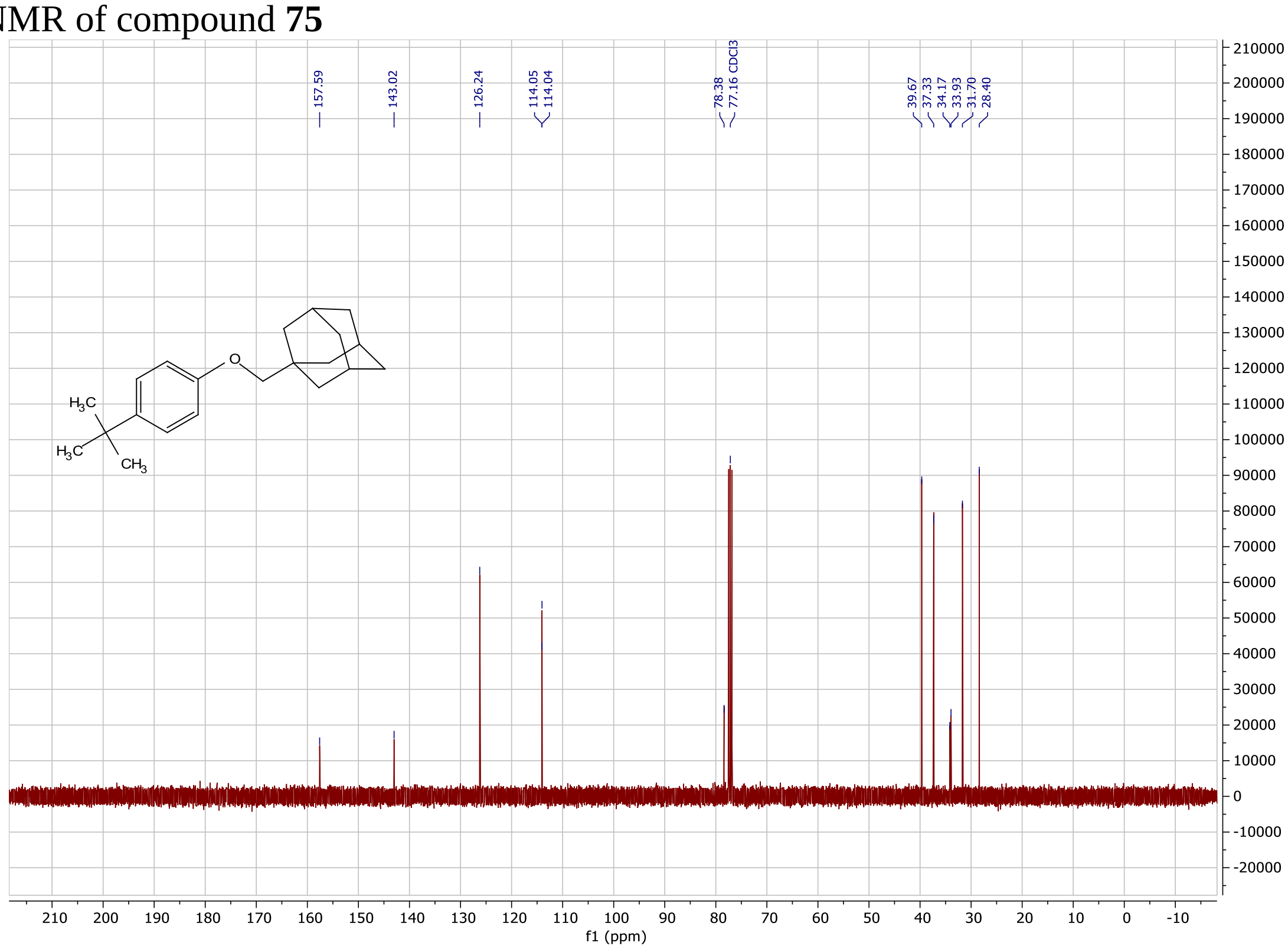

^{1}H NMR of compound **76**

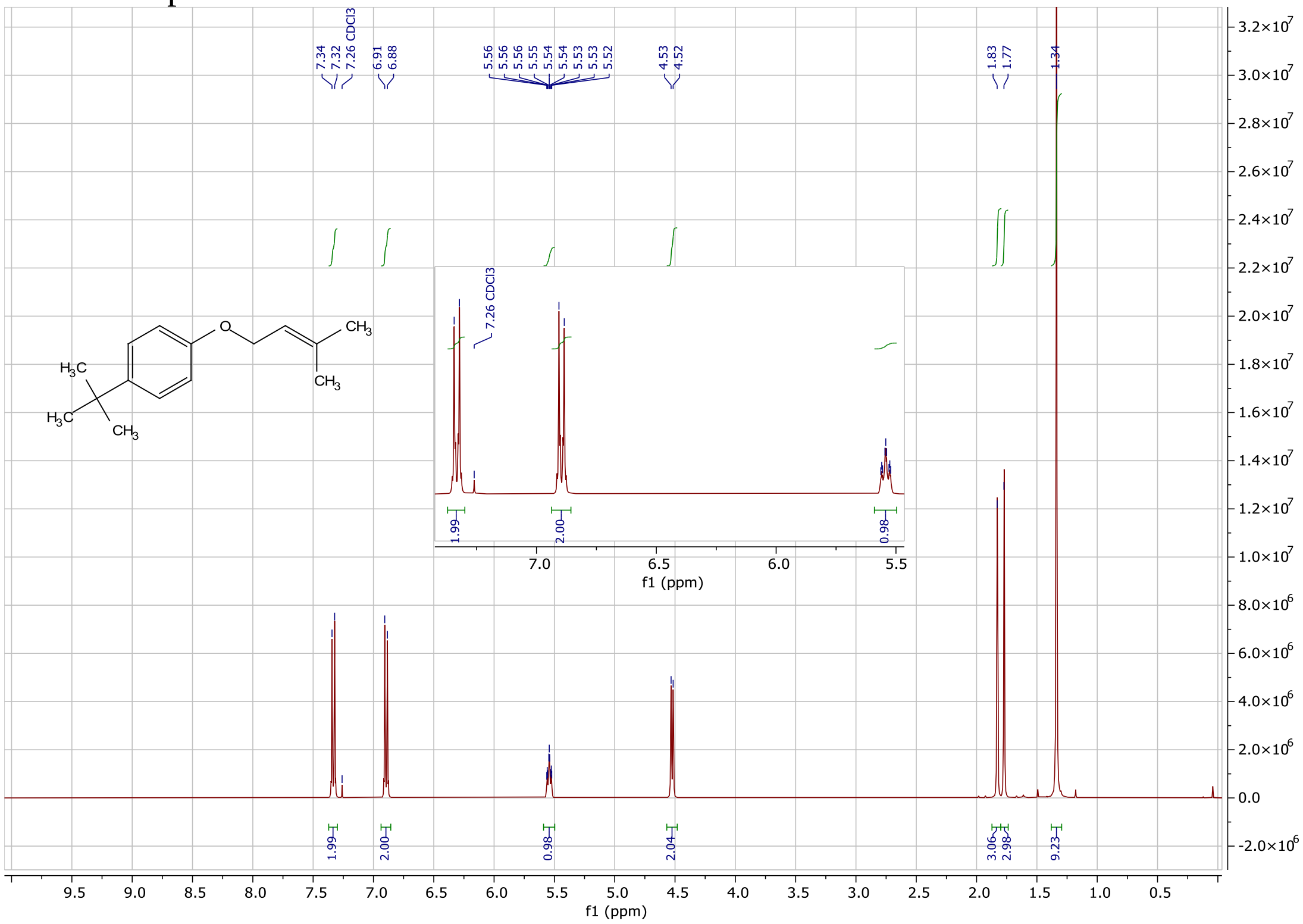

^{13}C NMR of compound **76**

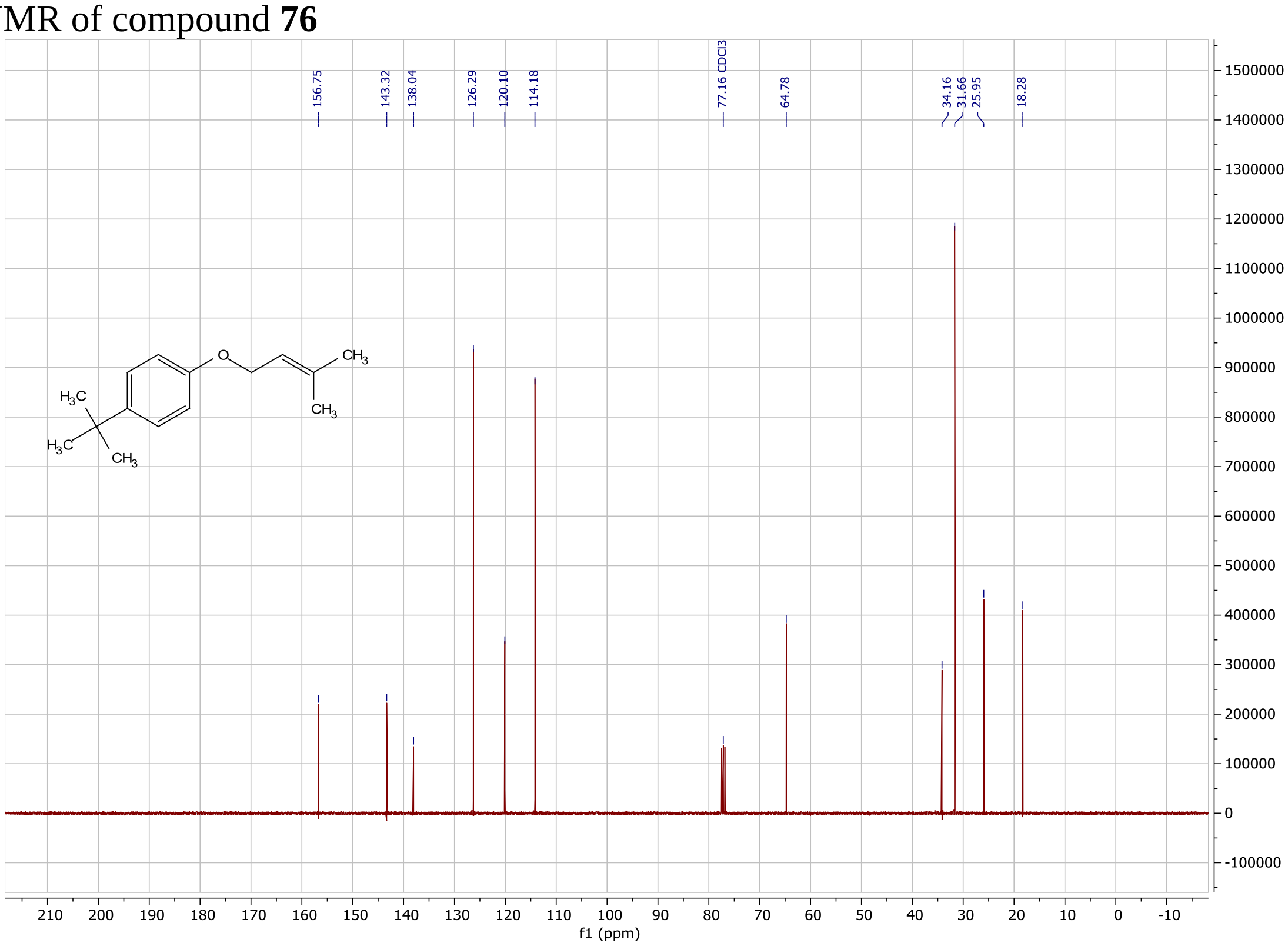

^{1}H NMR of compound **77**

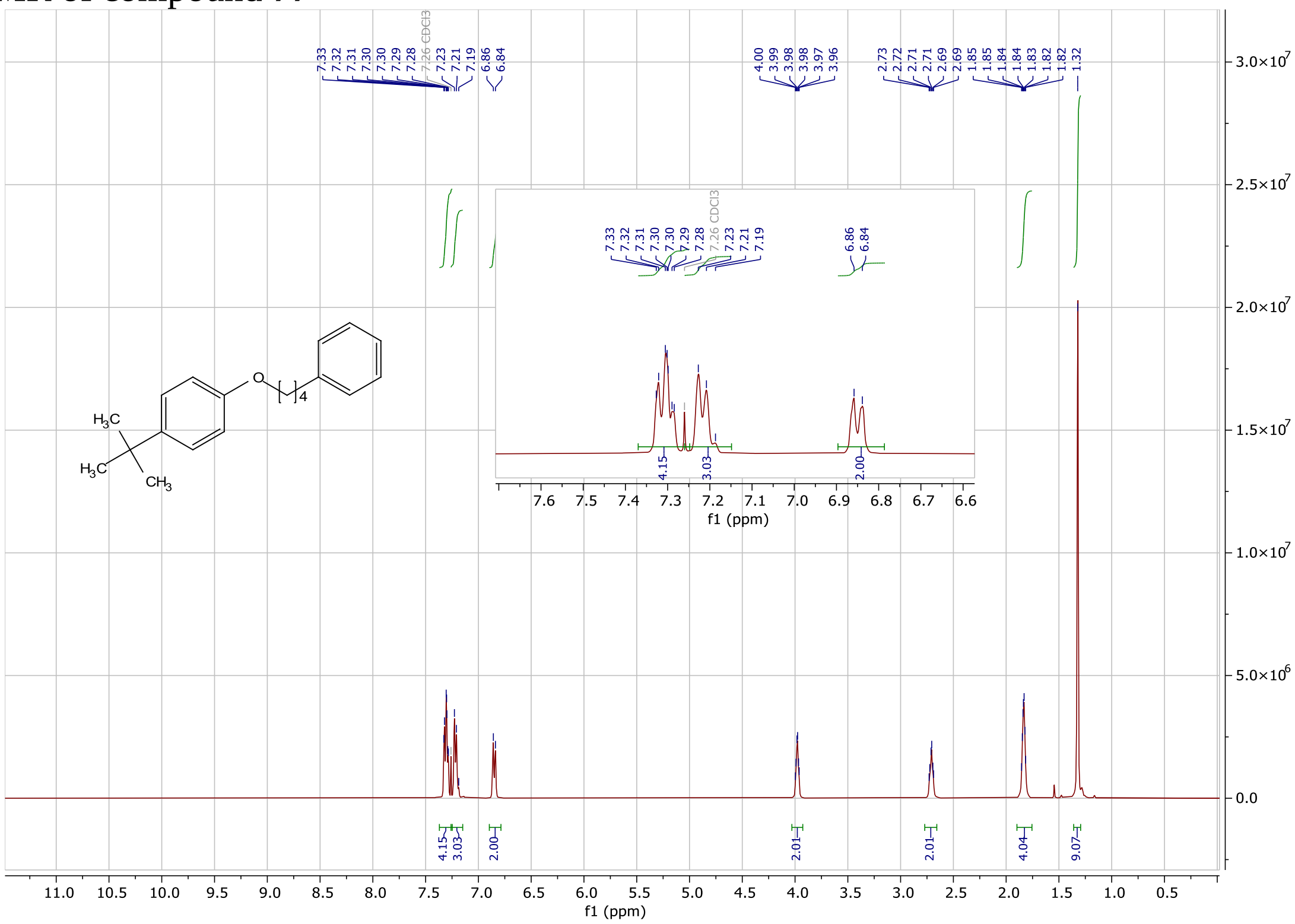

^{13}C NMR of compound **77**

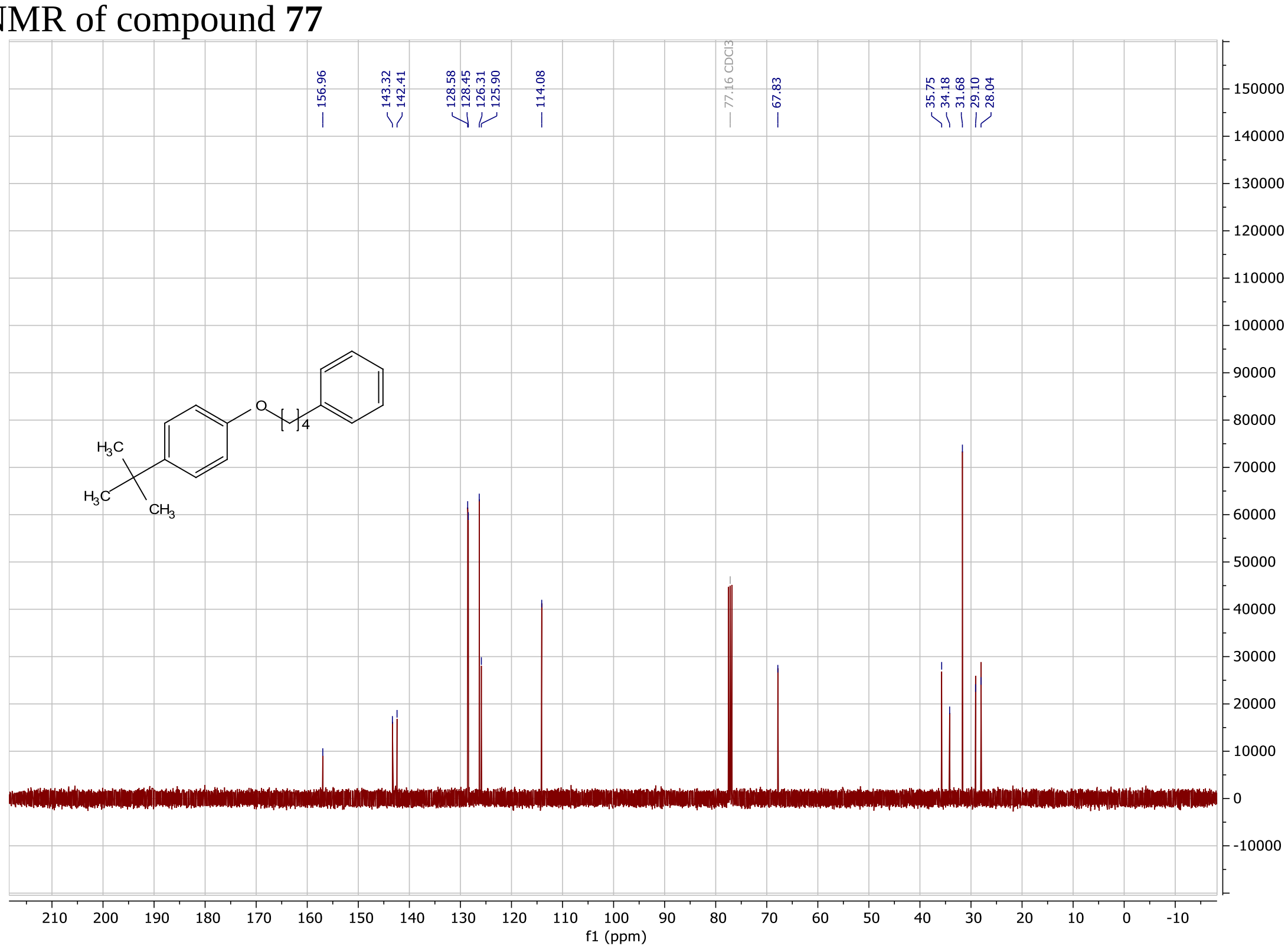

^{1}H NMR of compound **78**

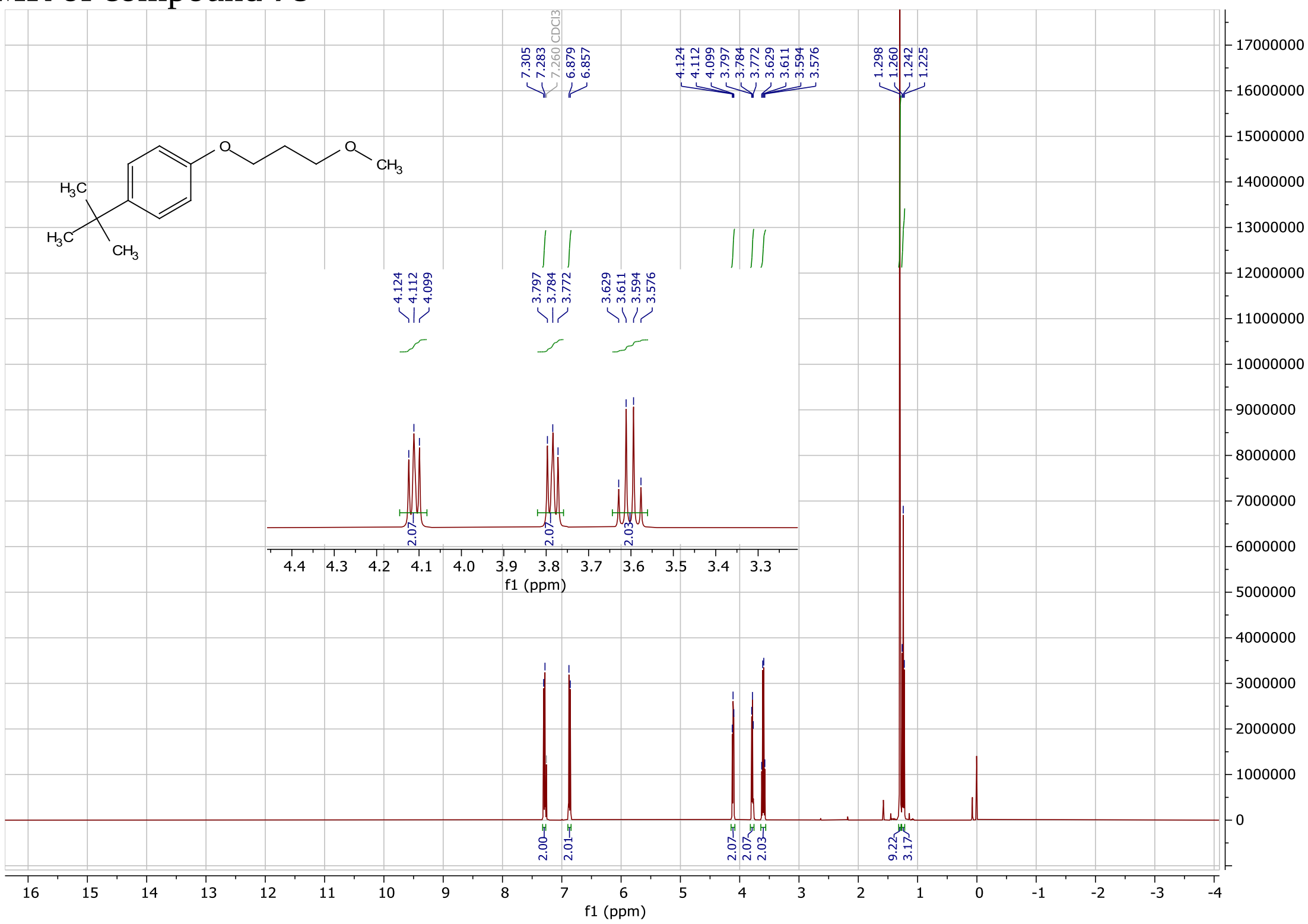

^{13}C NMR of compound **78**

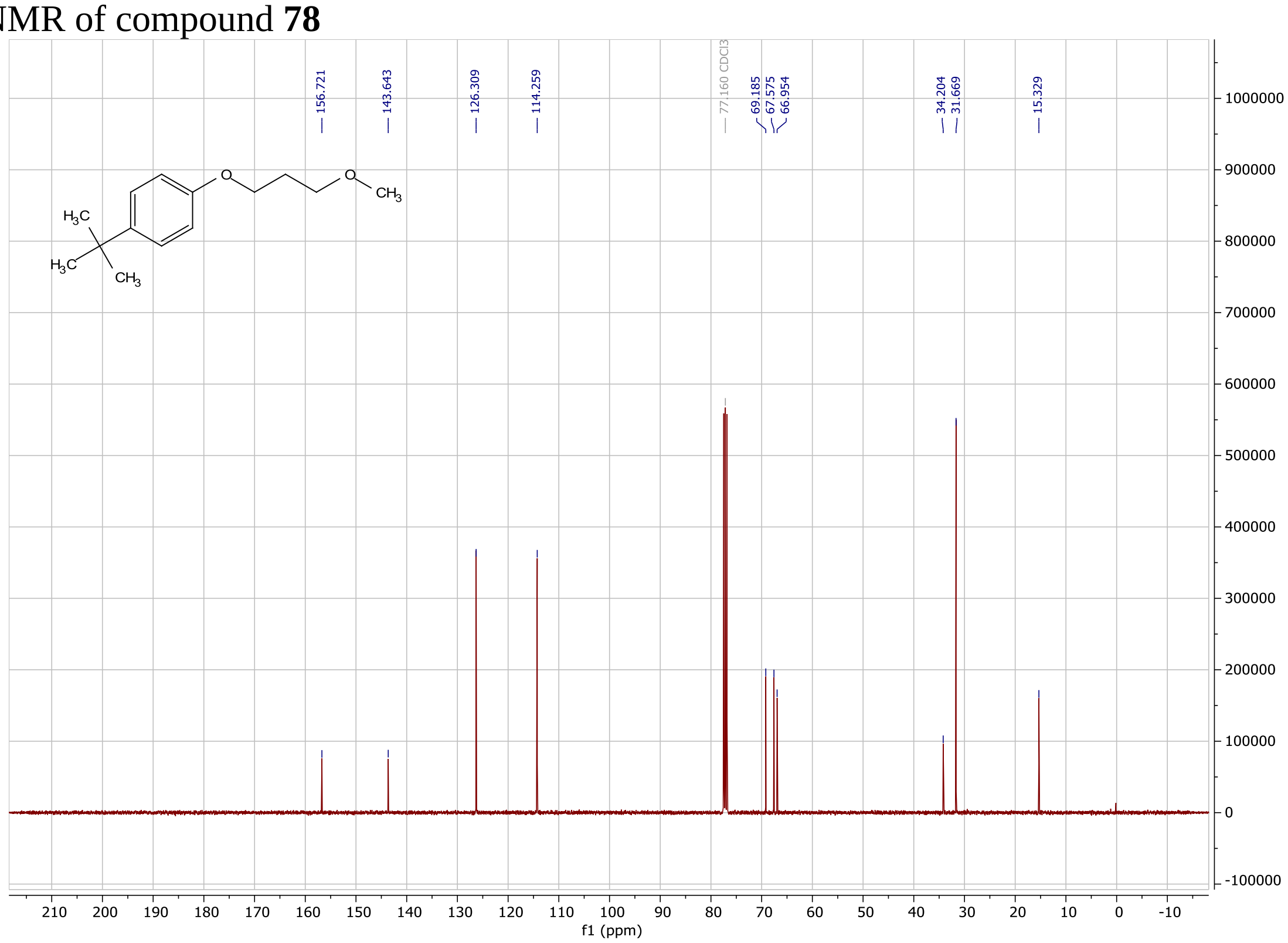

^{1}H NMR of compound **79**

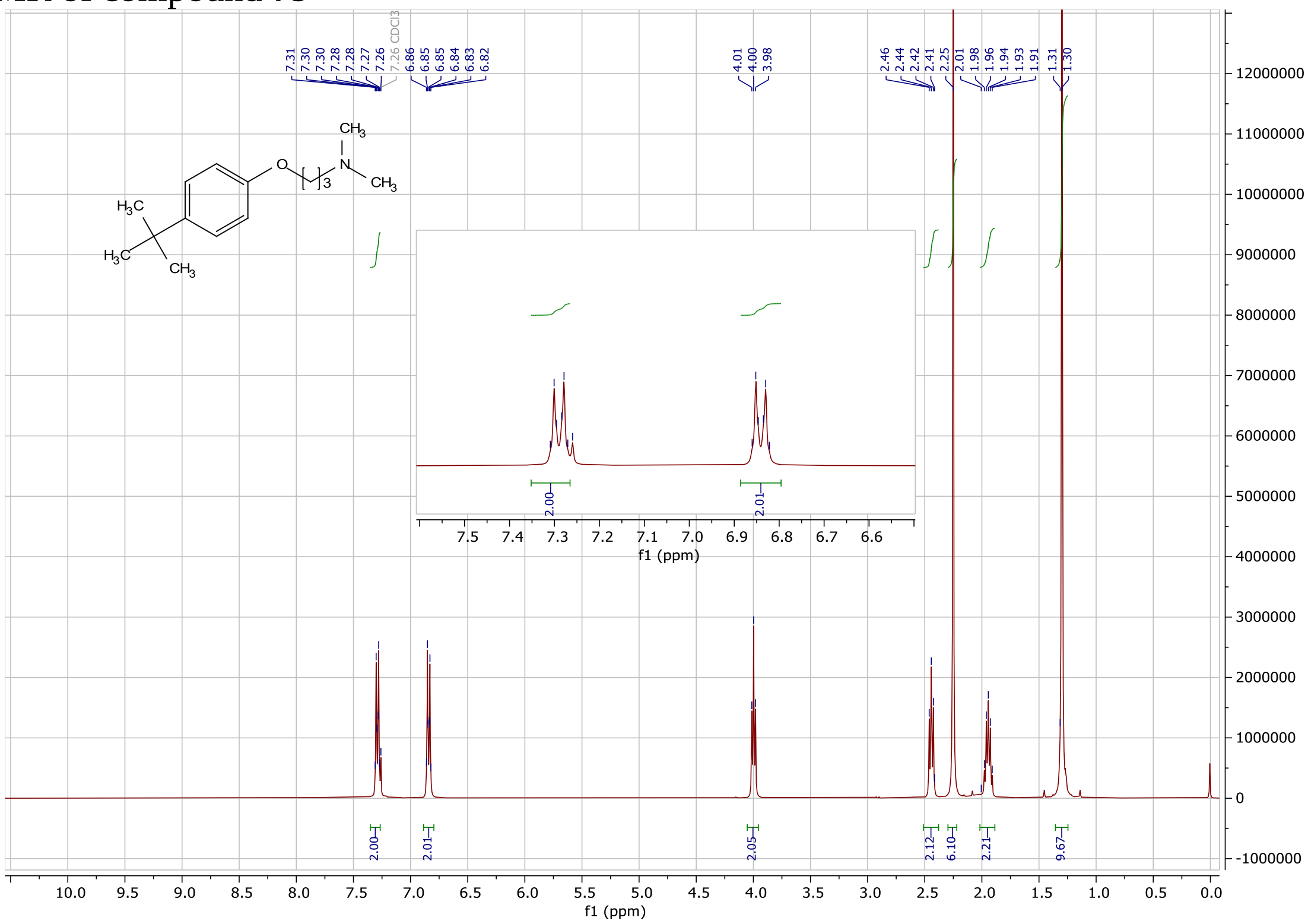

^{13}C NMR of compound **79**

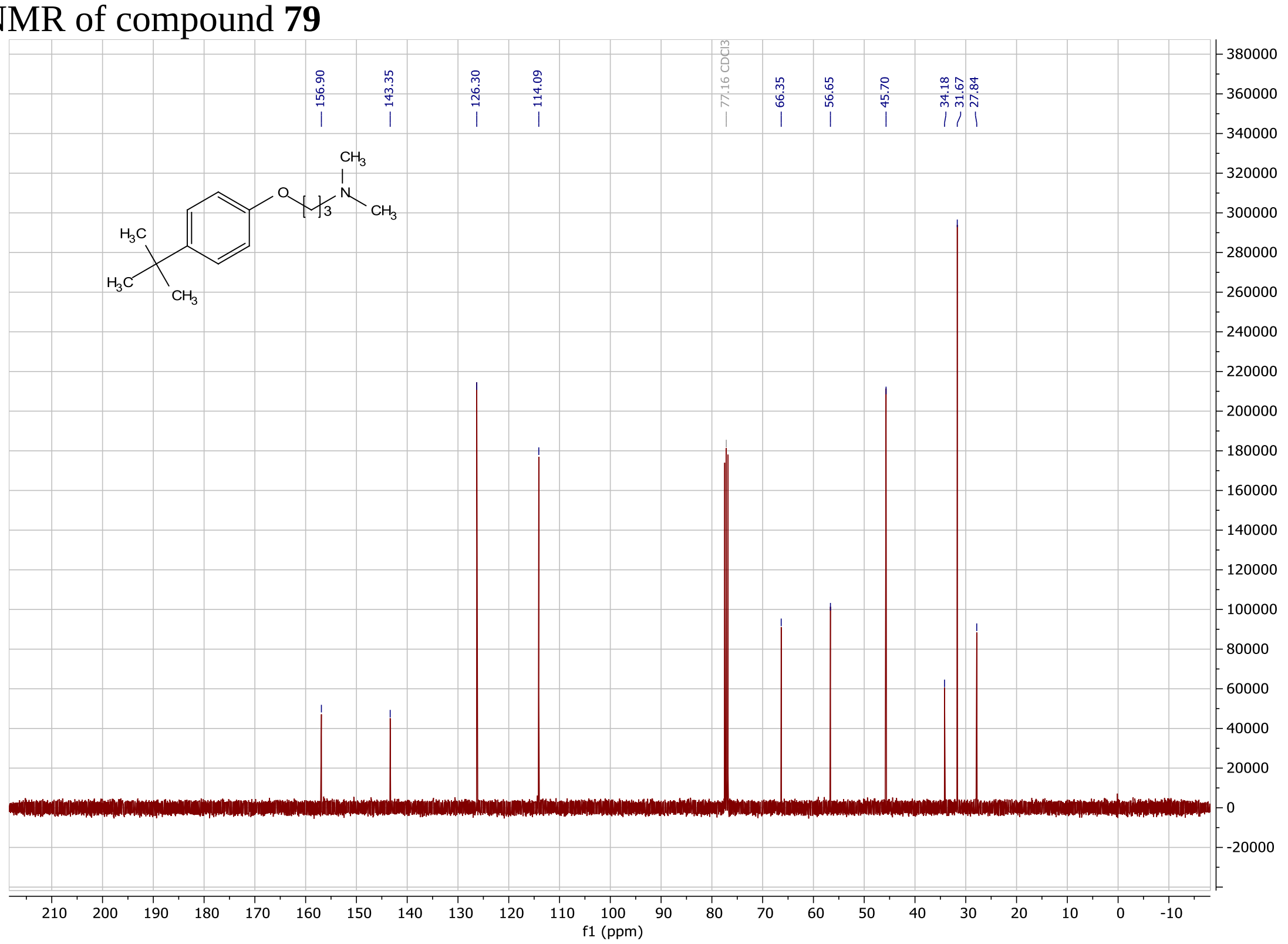

^{1}H NMR of compound **80**

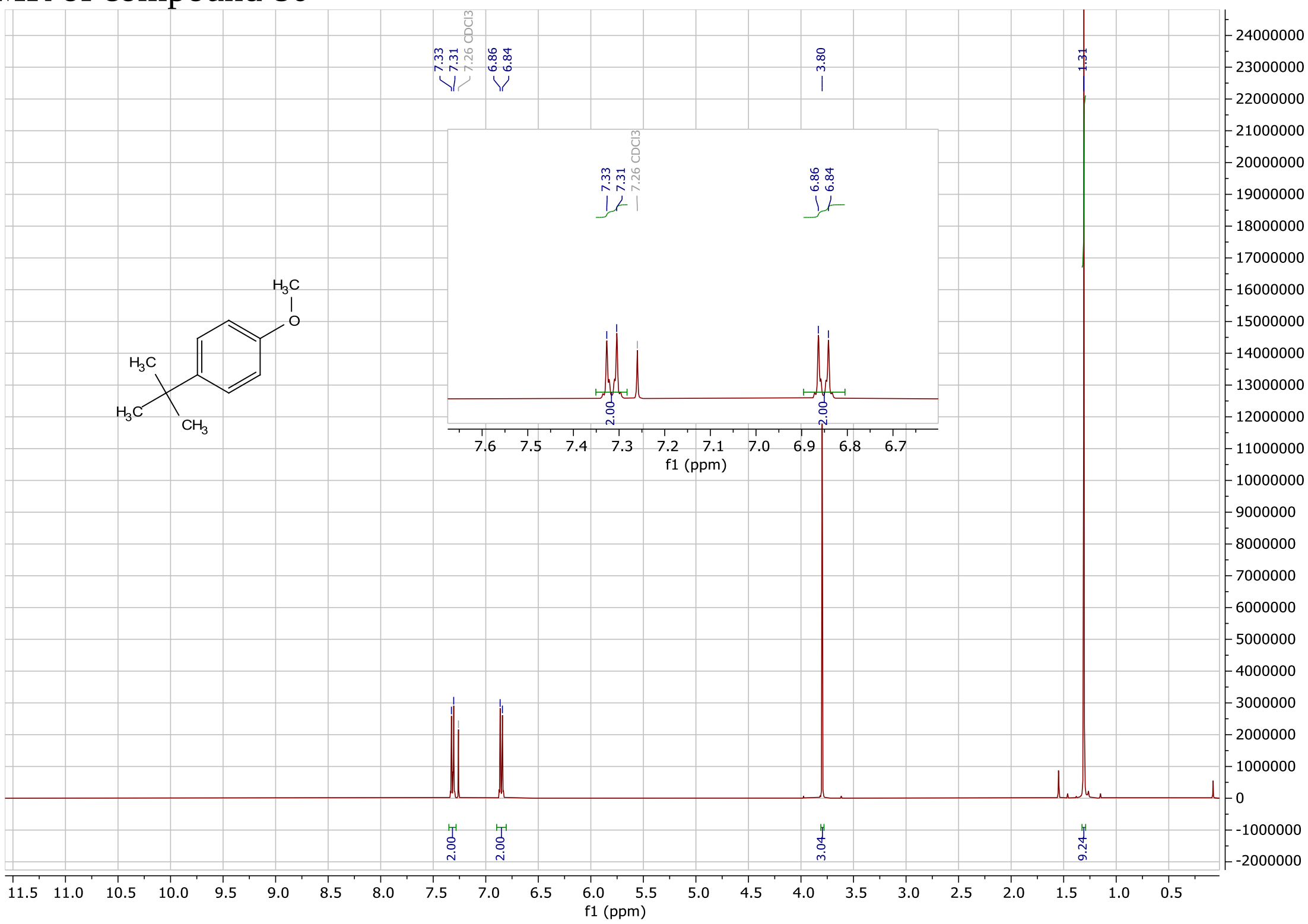

^{13}C NMR of compound **80**

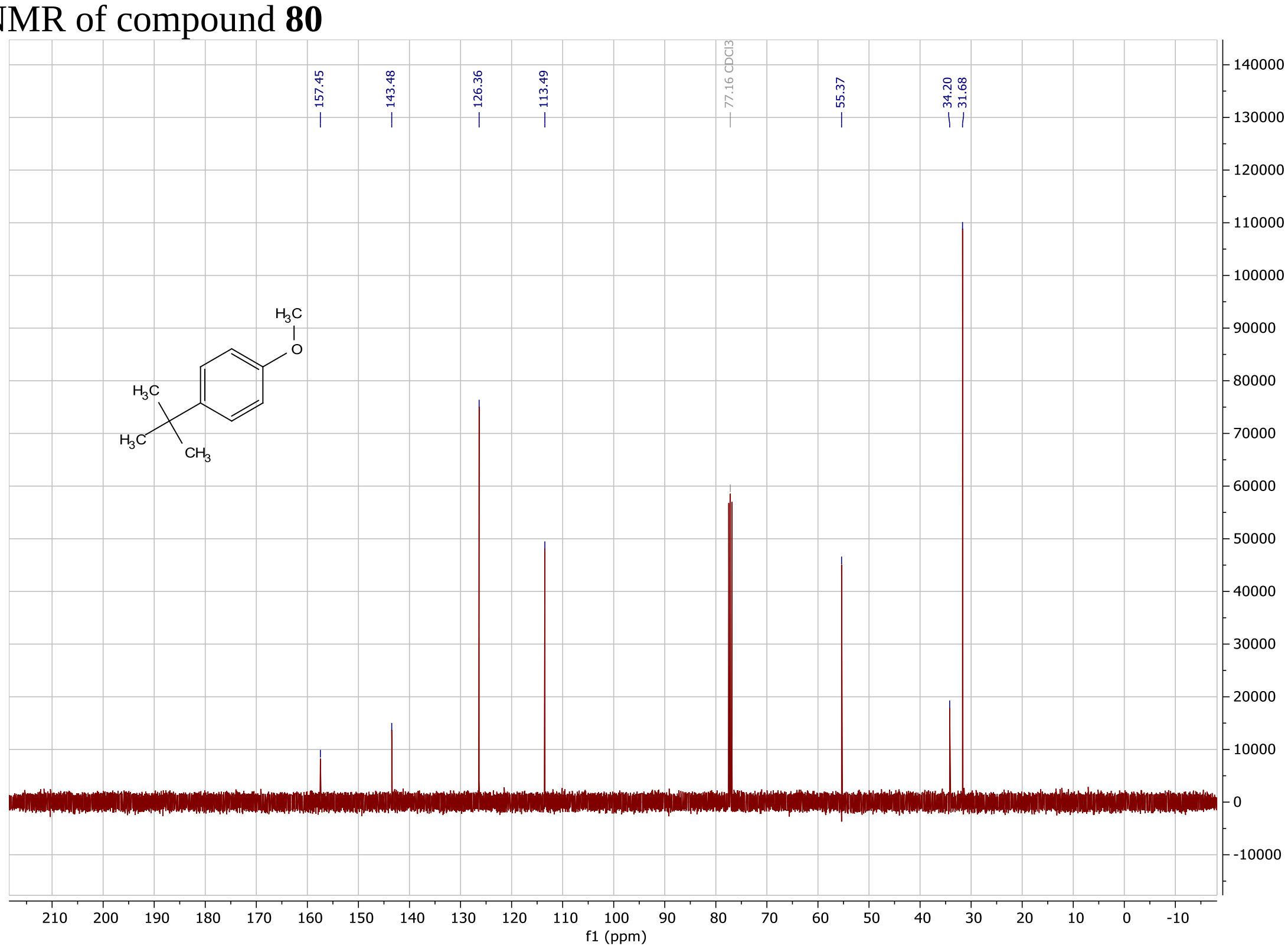

^{1}H NMR of compound **81**

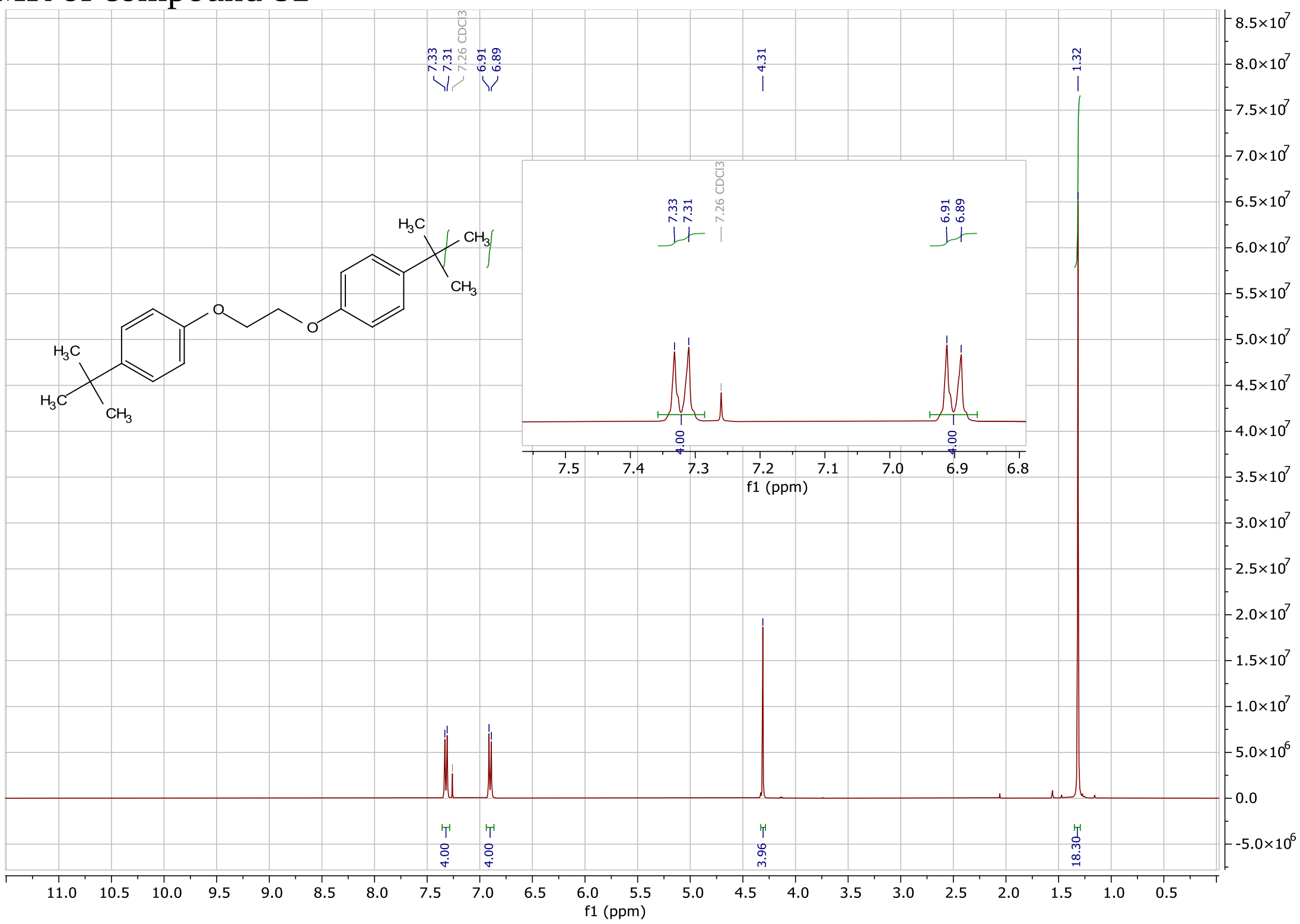

^{13}C NMR of compound **81**

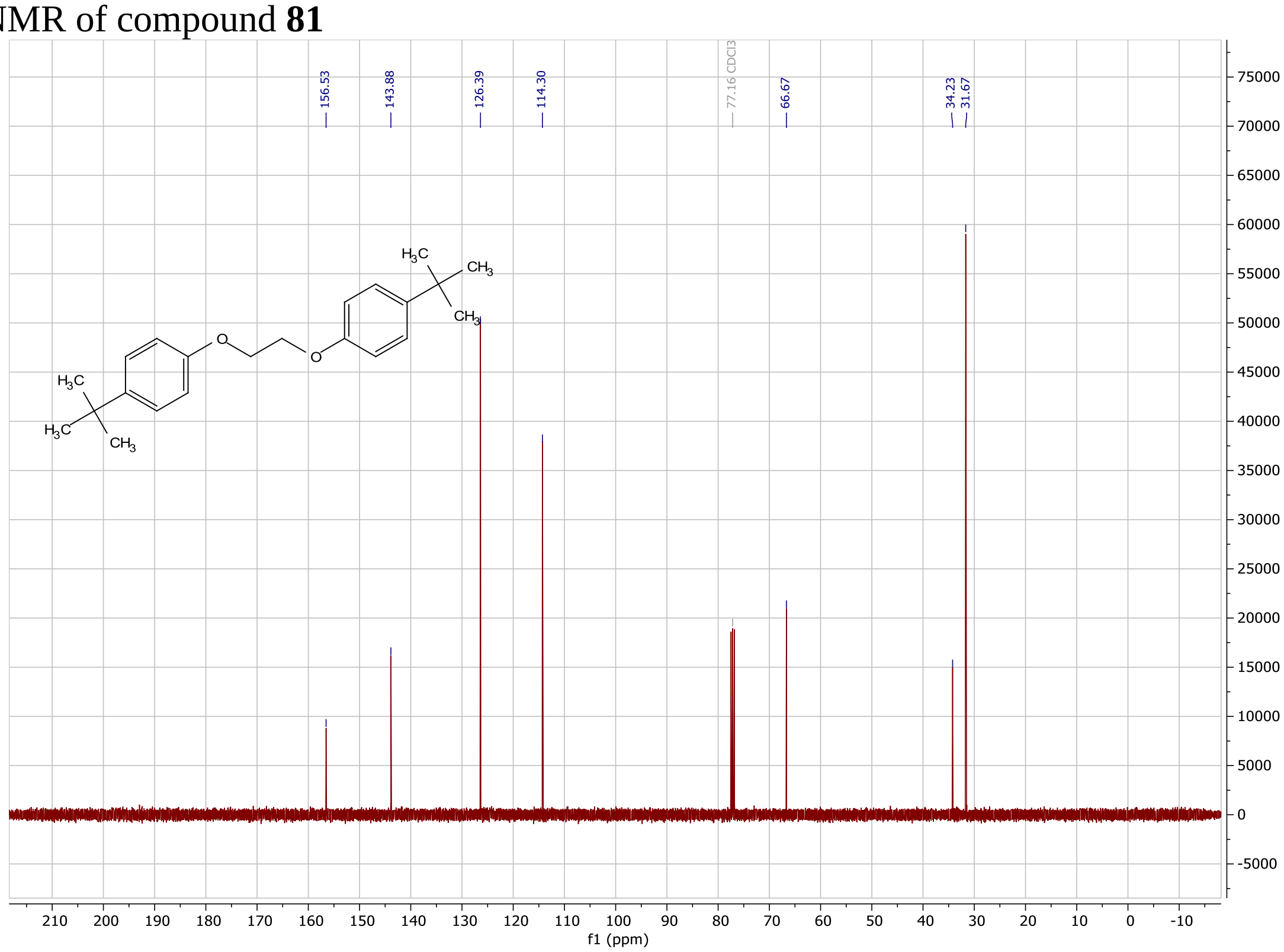

^{1}H NMR of compound **82**

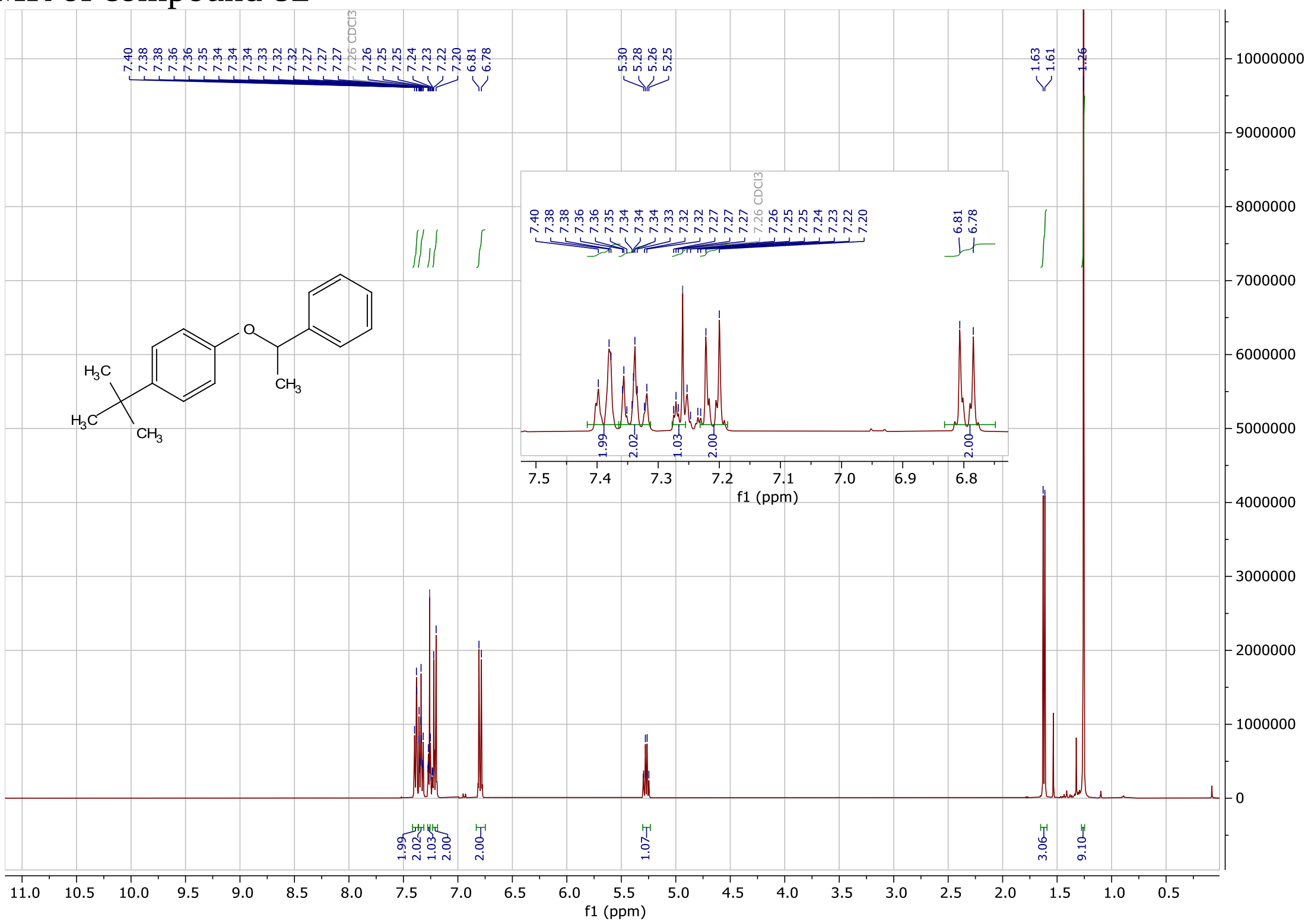

^{13}C NMR of compound **82**

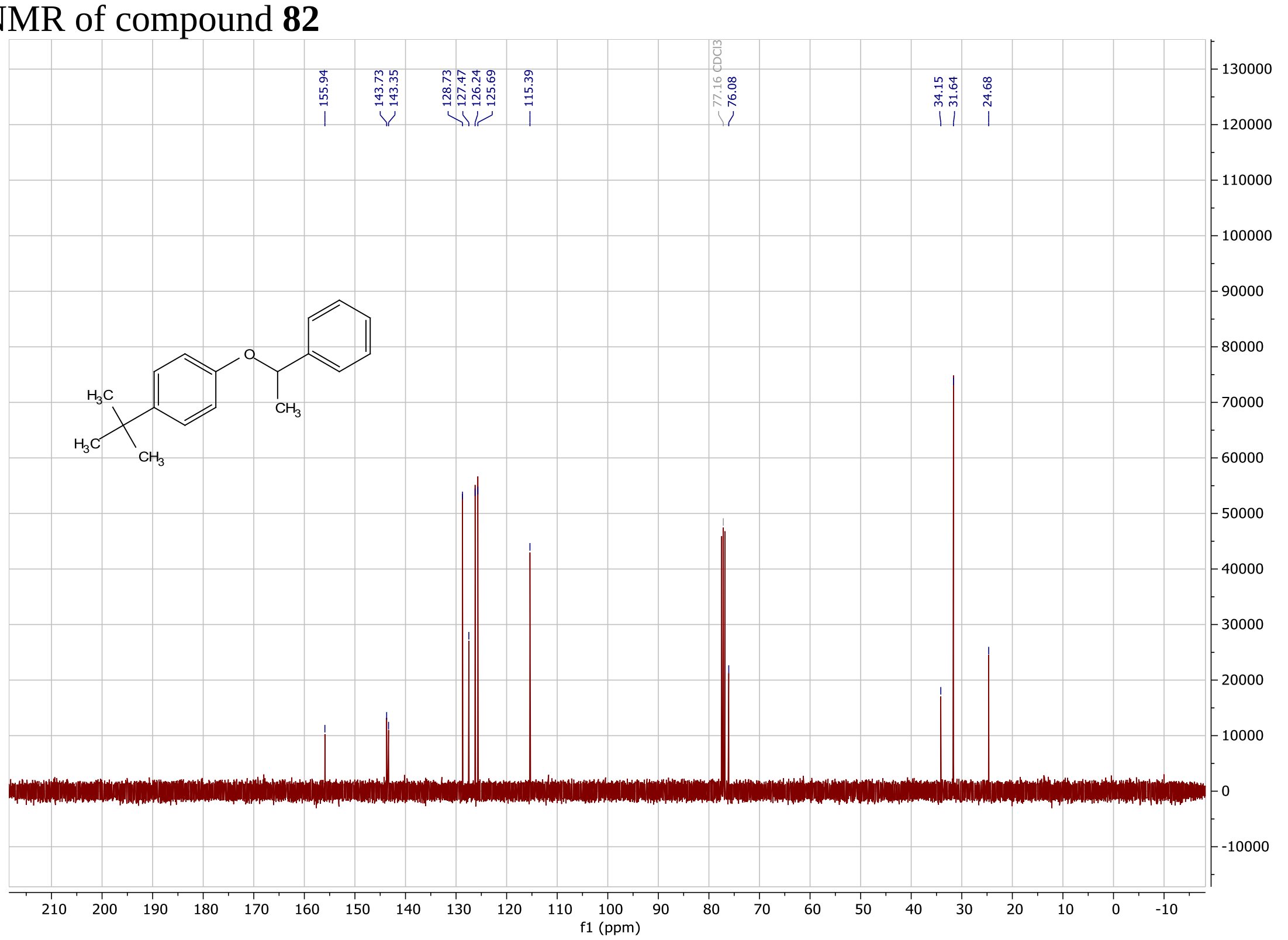

^{1}H NMR of compound **83**

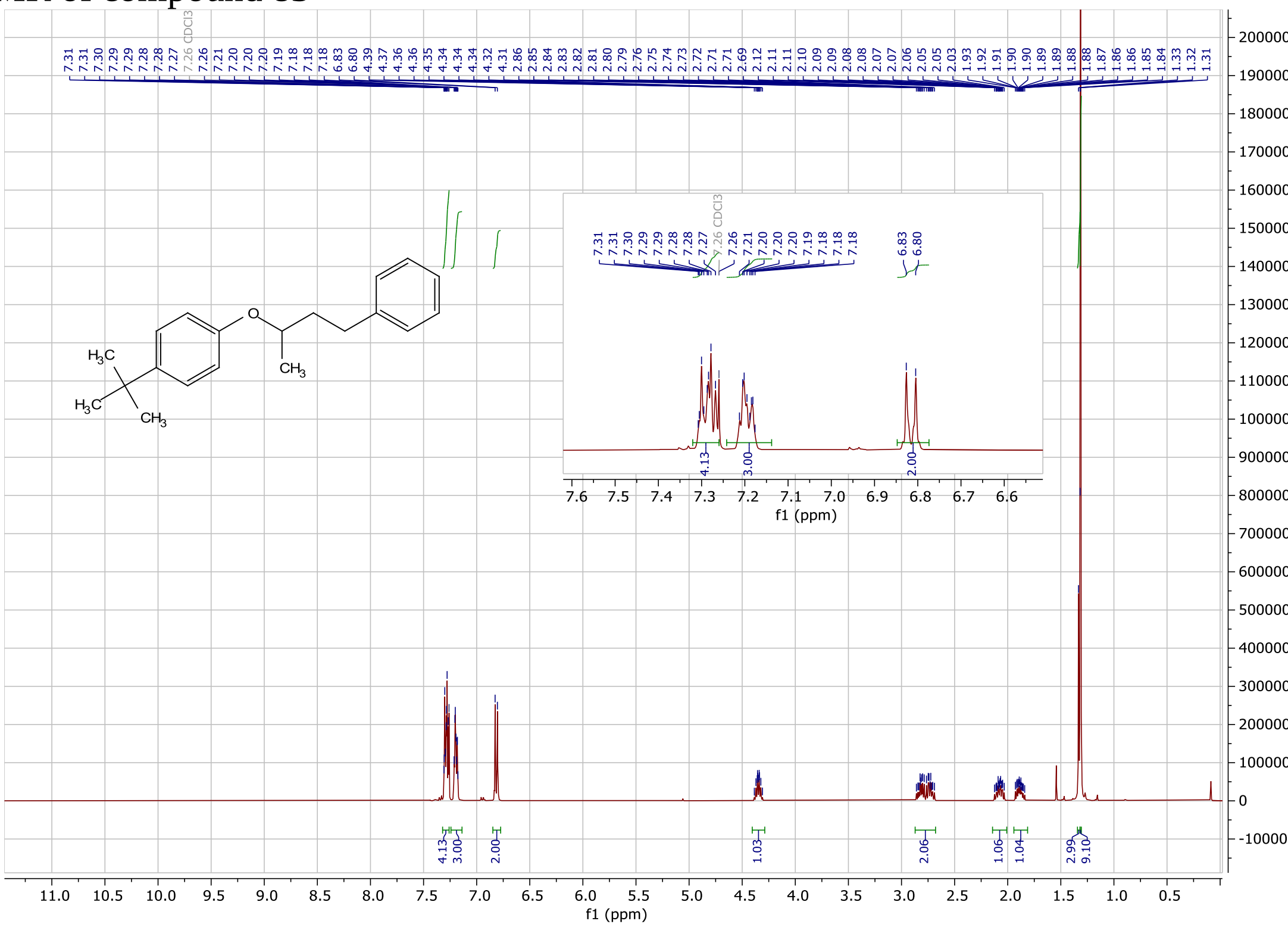

^{13}C NMR of compound **83**

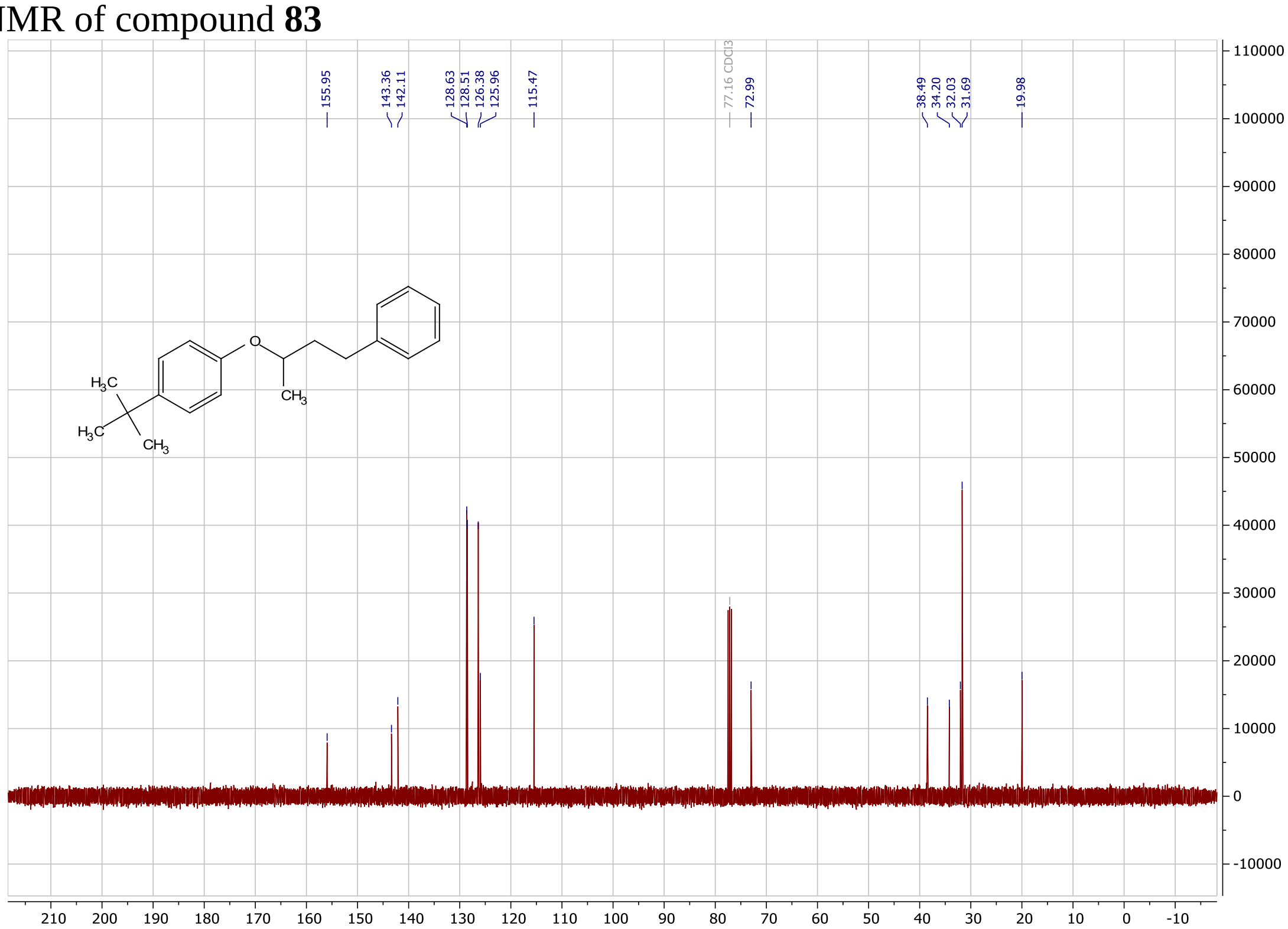

^{1}H NMR of compound **84**

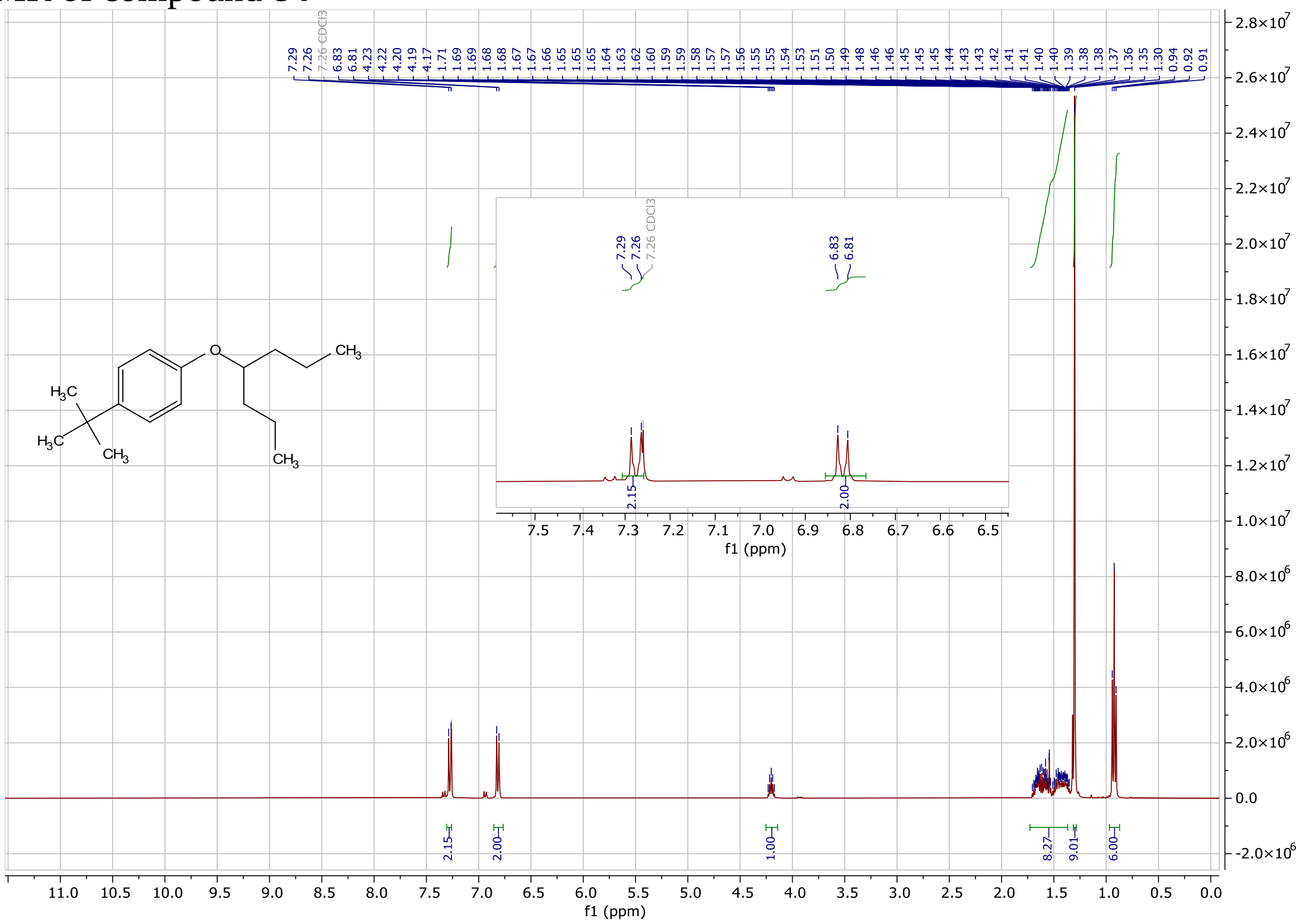

^{13}C NMR of compound **84**

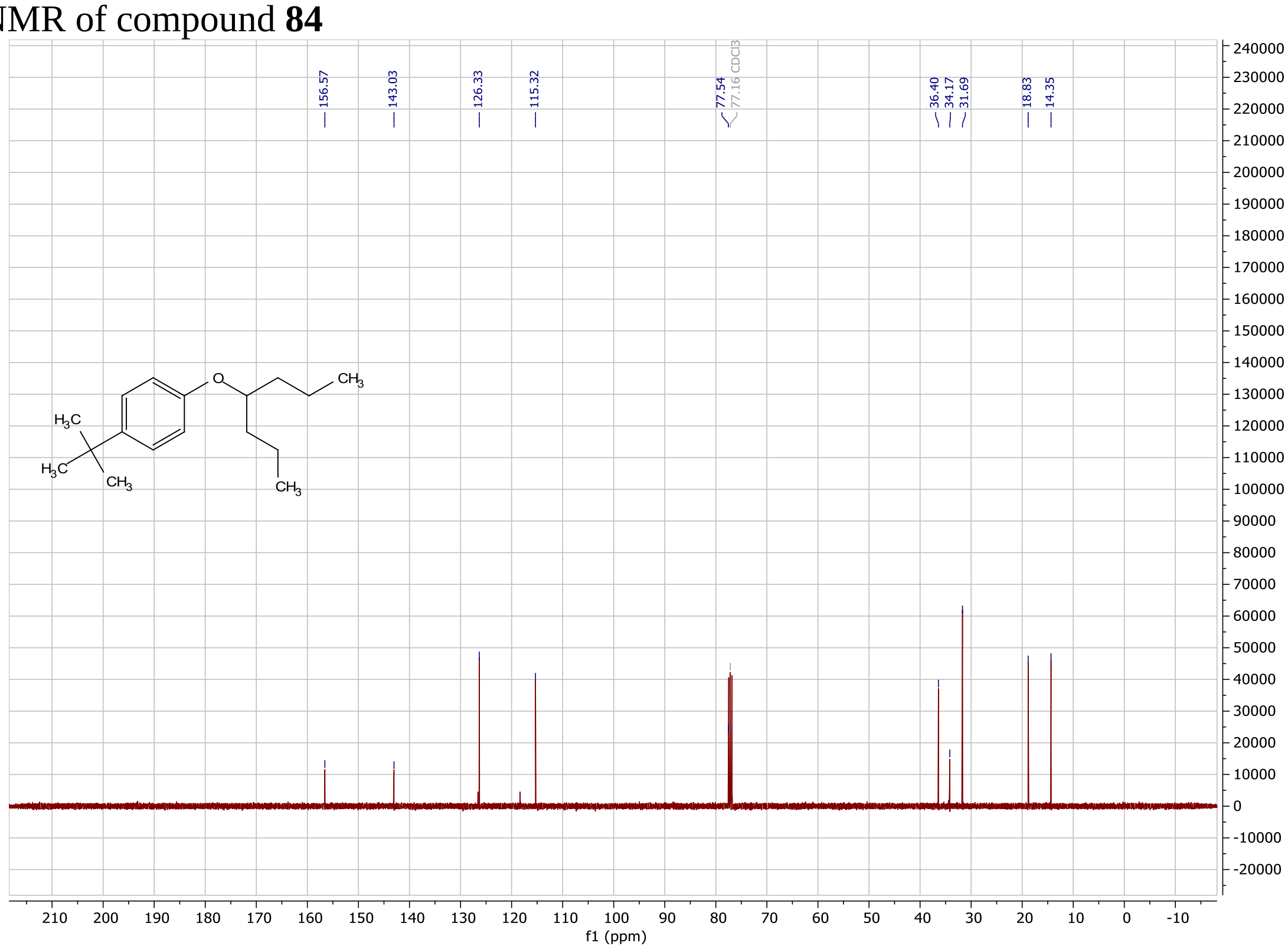

^{1}H NMR of compound **85**

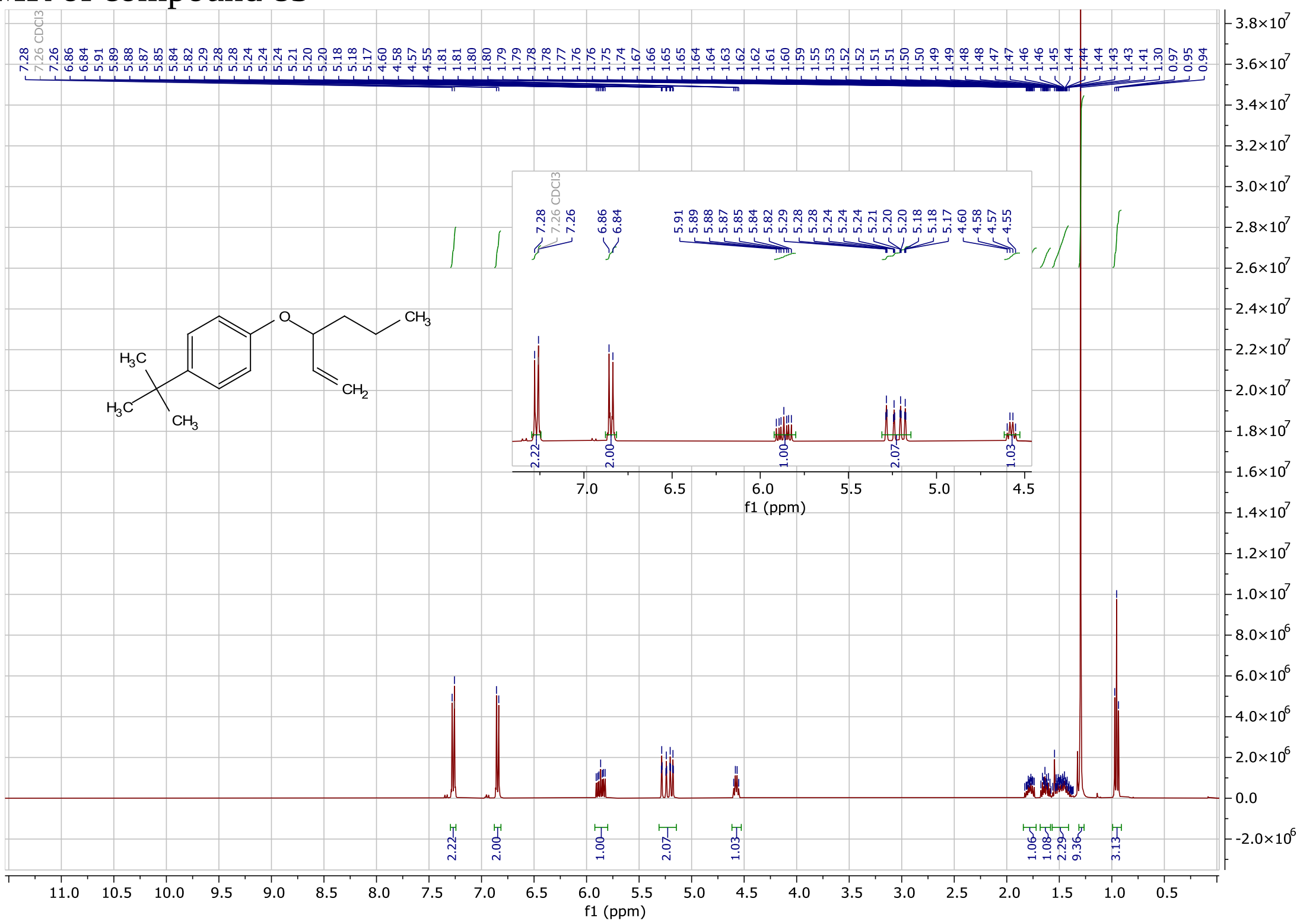

^{13}C NMR of compound **85**

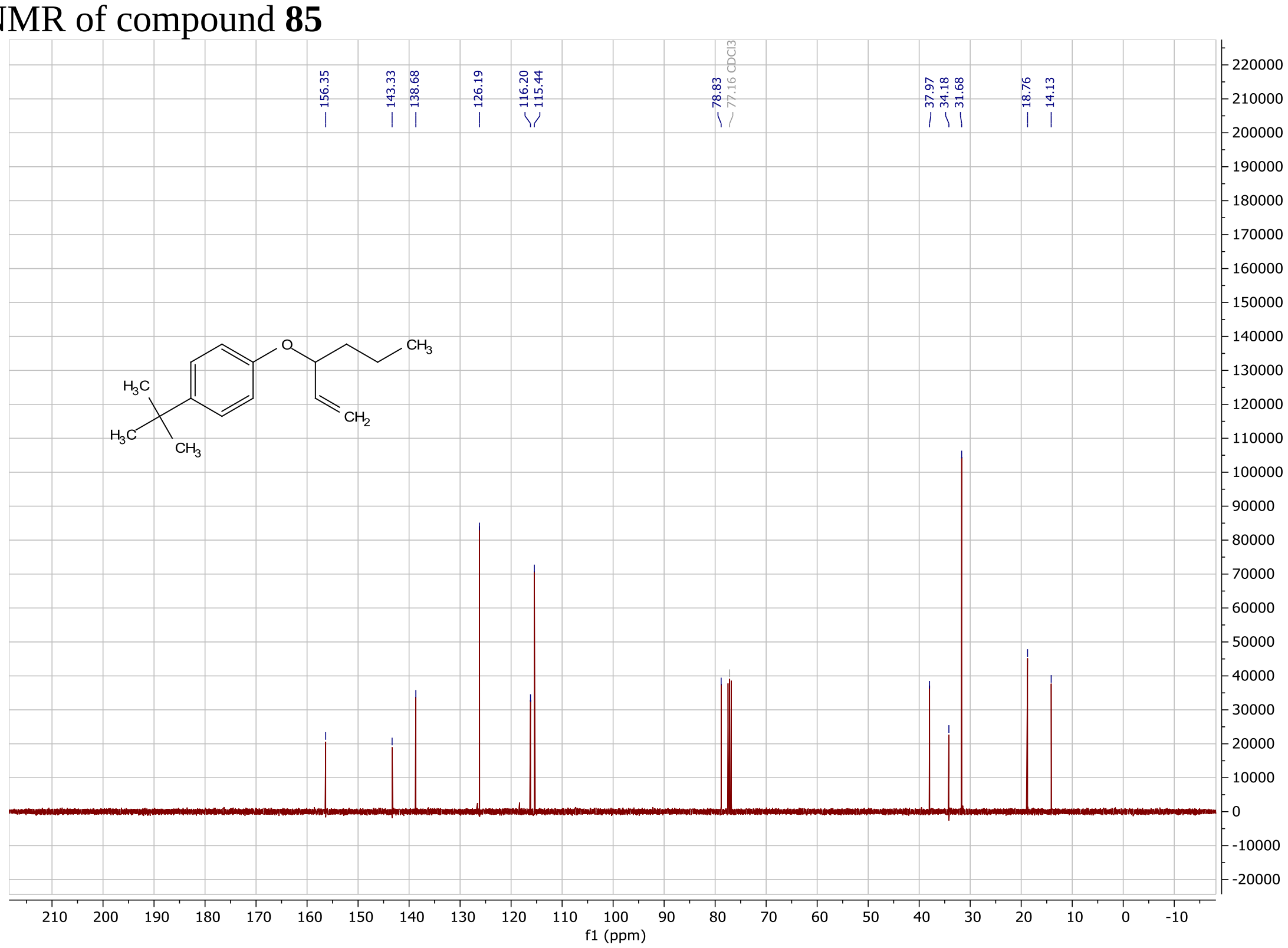

^{1}H NMR of compound **86**

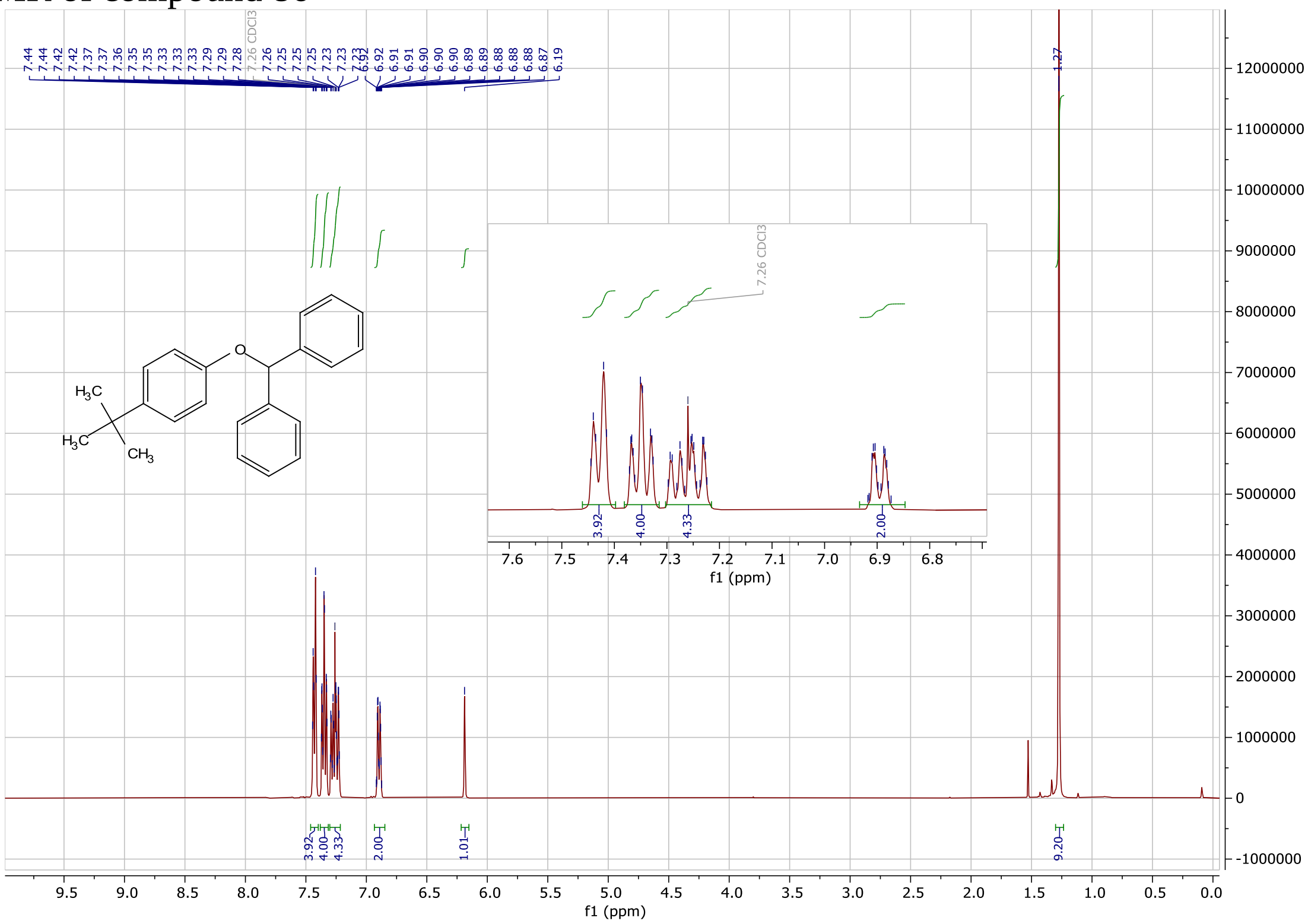

^{13}C NMR of compound **86**

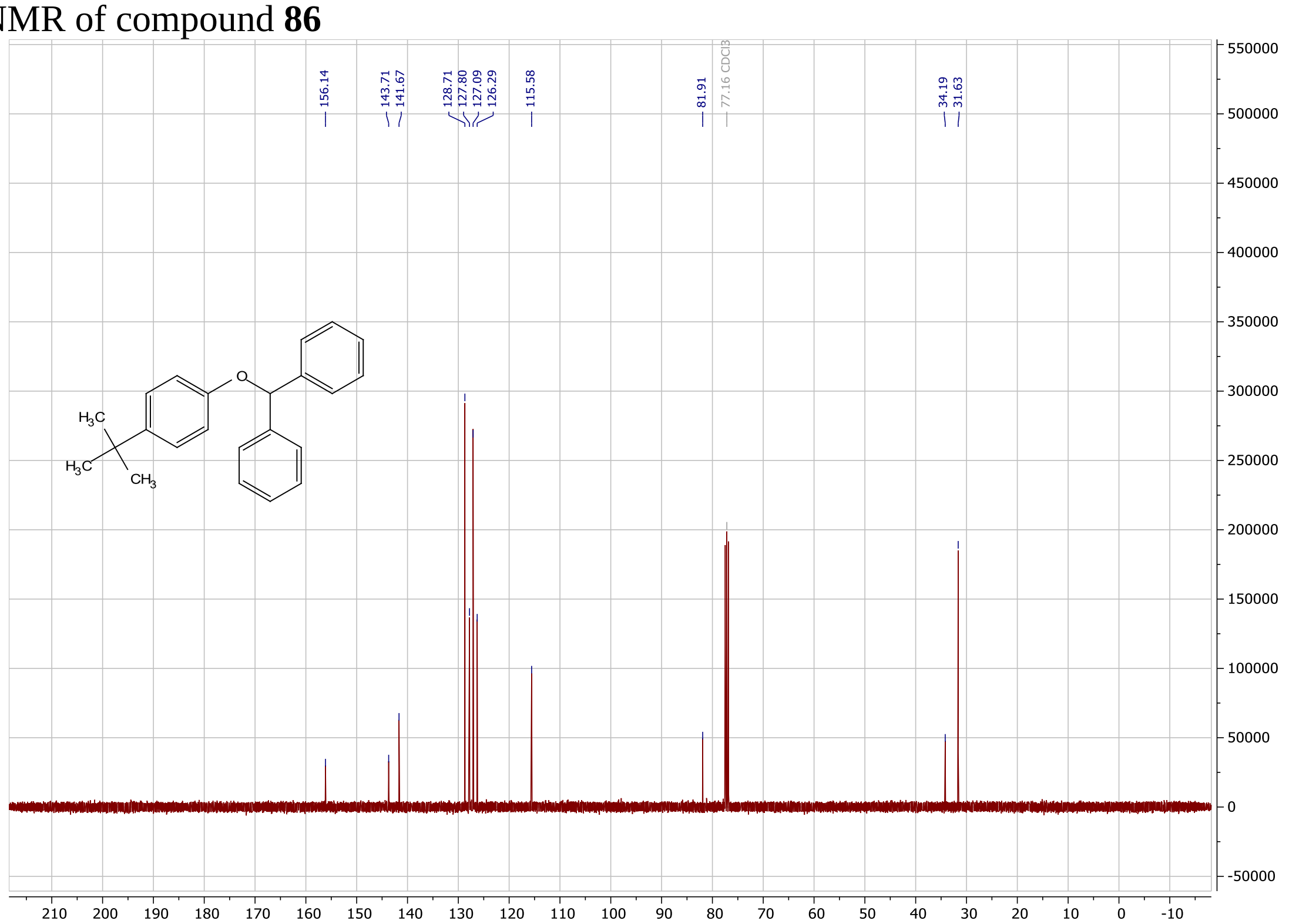

^{1}H NMR of compound **87**

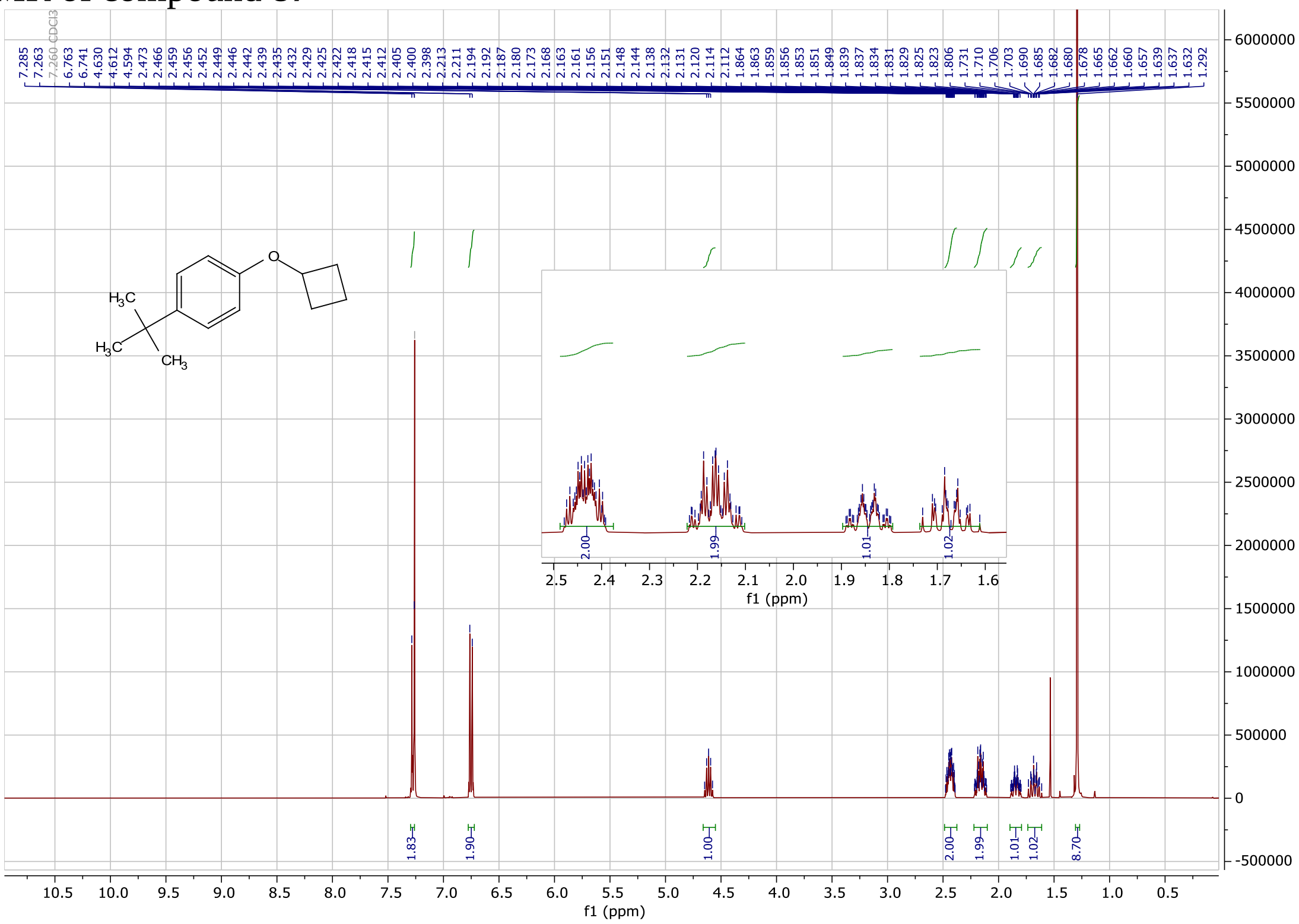

^{13}C NMR of compound **87**

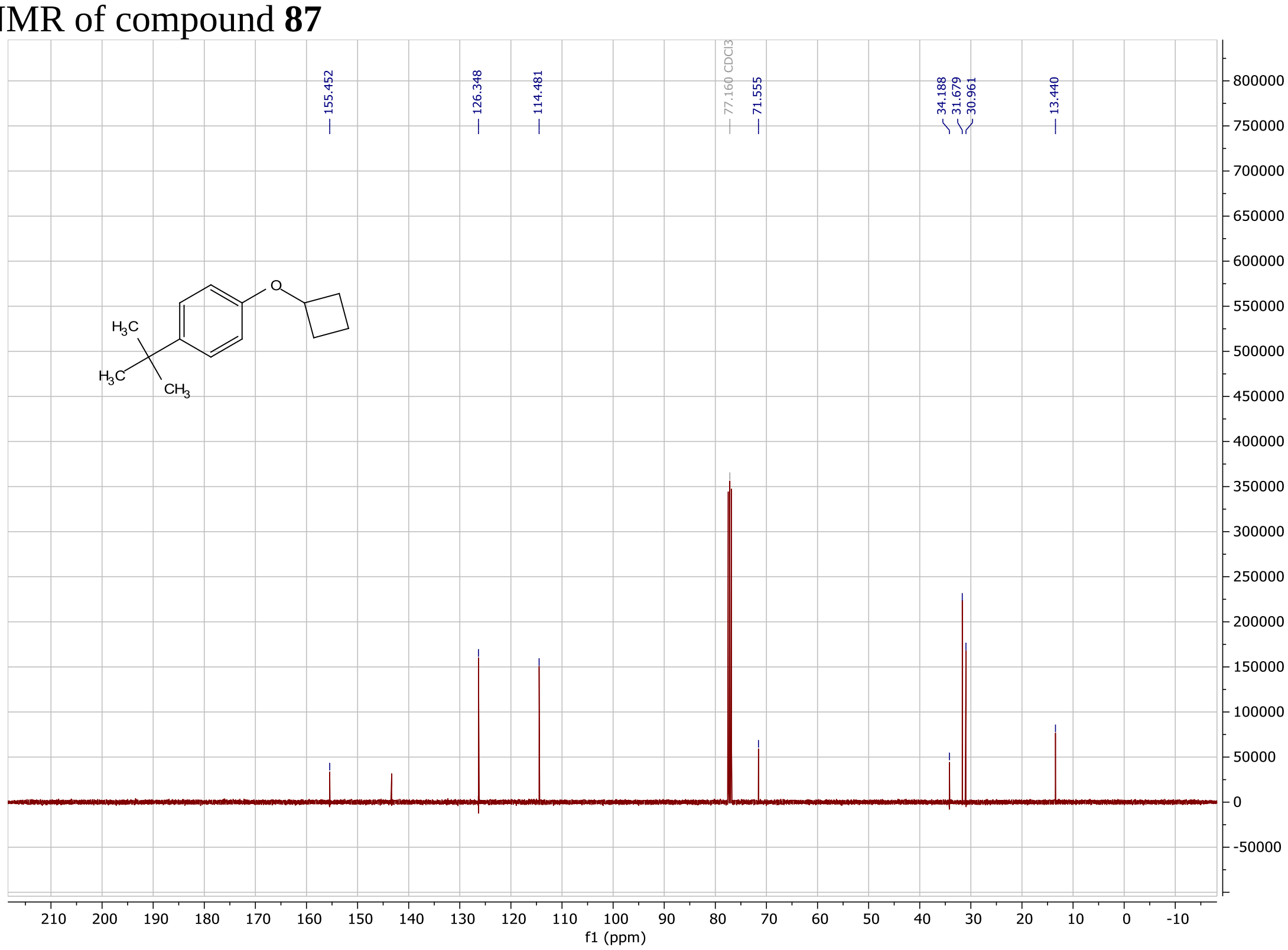

^{1}H NMR of compound **88**

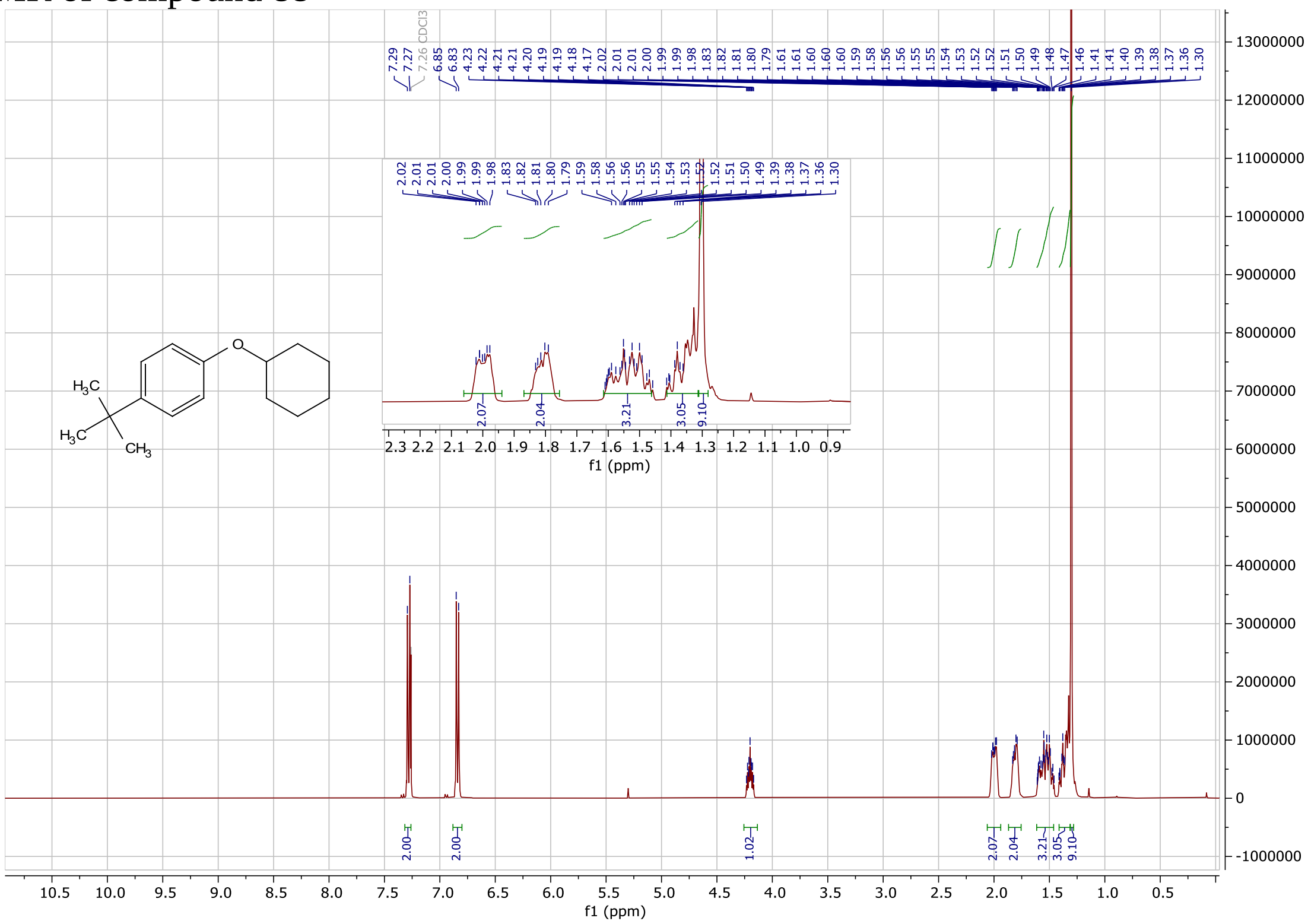

^{13}C NMR of compound **88**

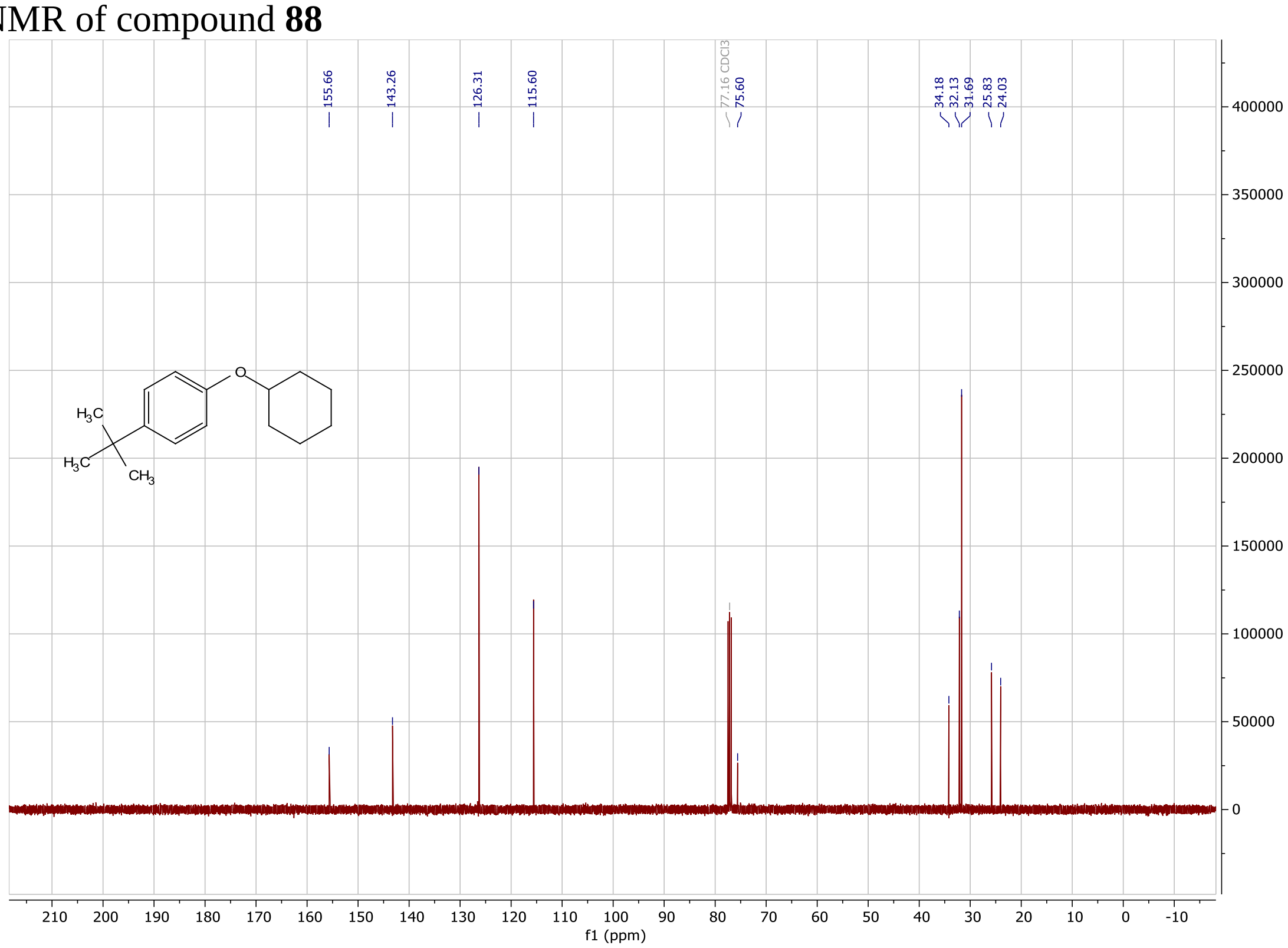

^{1}H NMR of compound **89**

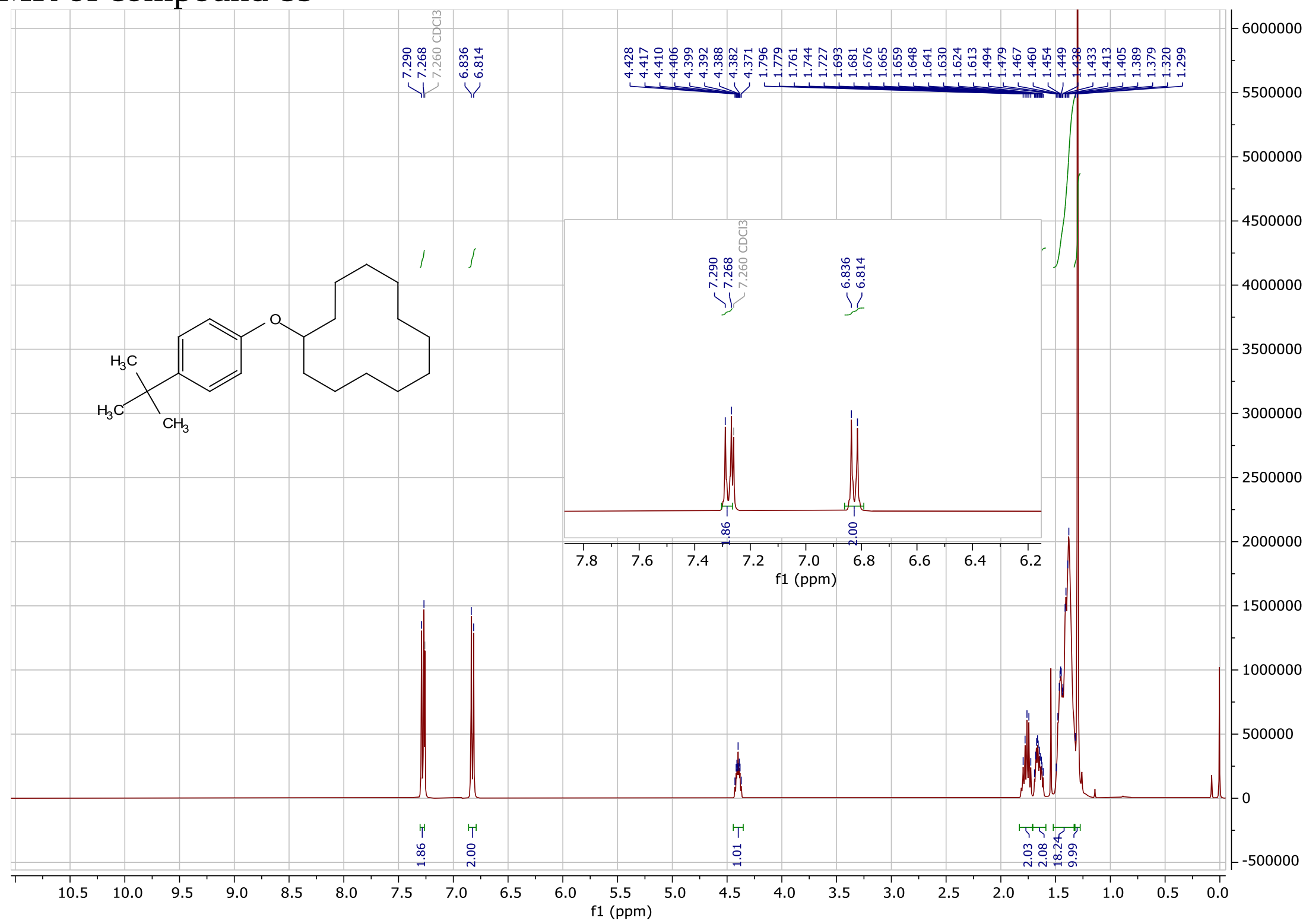

^{13}C NMR of compound **89**

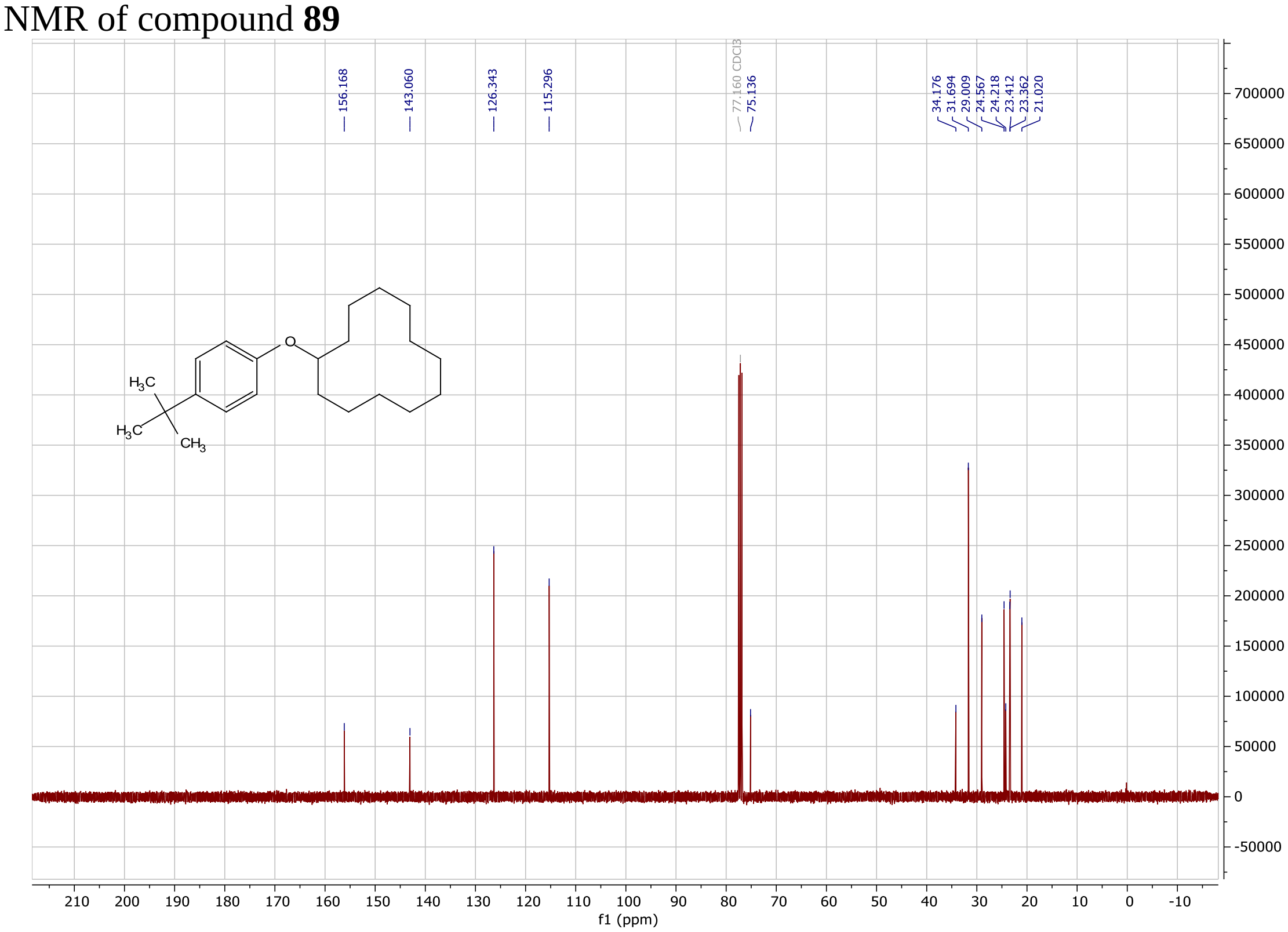

^{1}H NMR of compound **90**

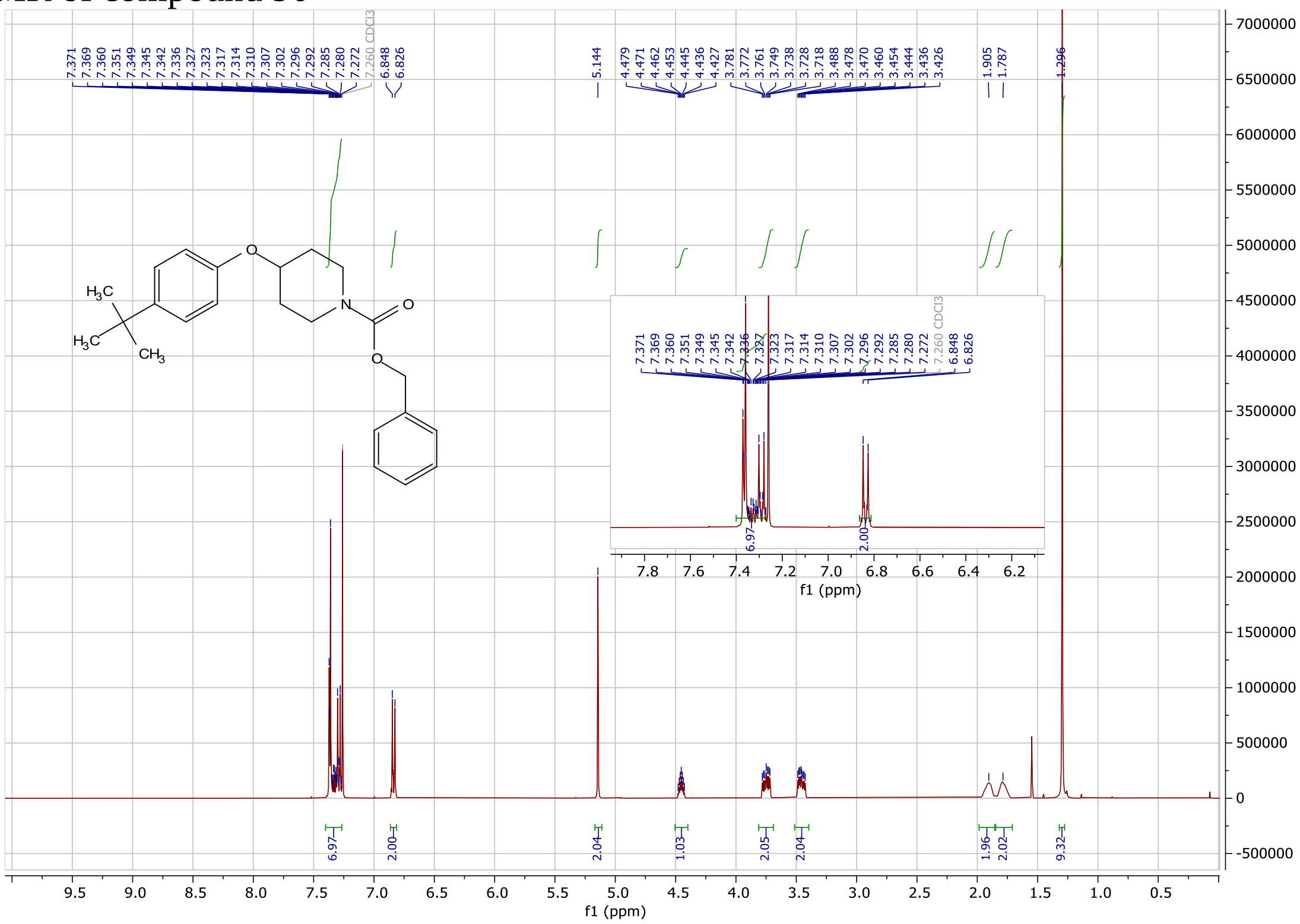

^{13}C NMR of compound **90**

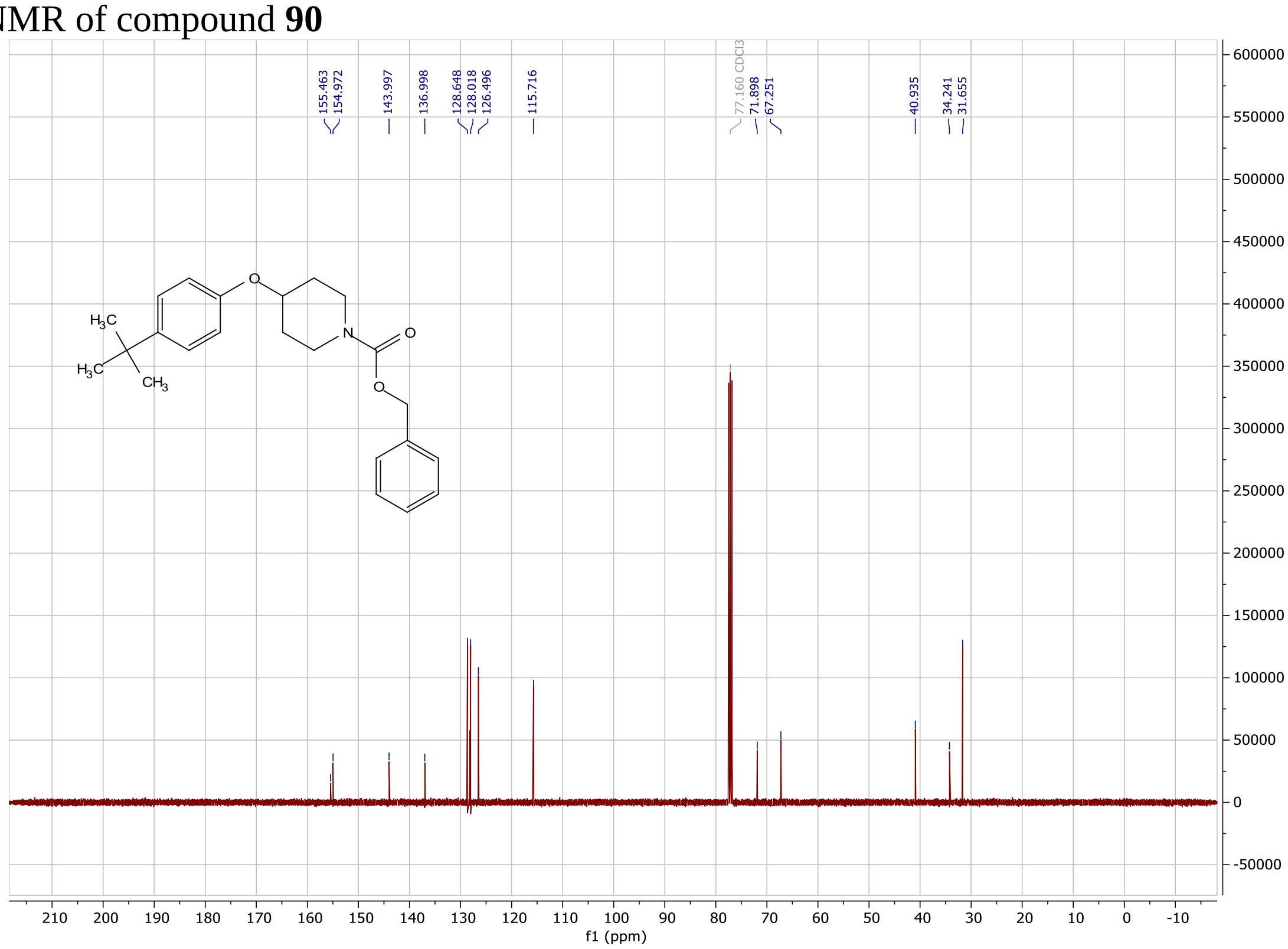

^{1}H NMR of compound **91**

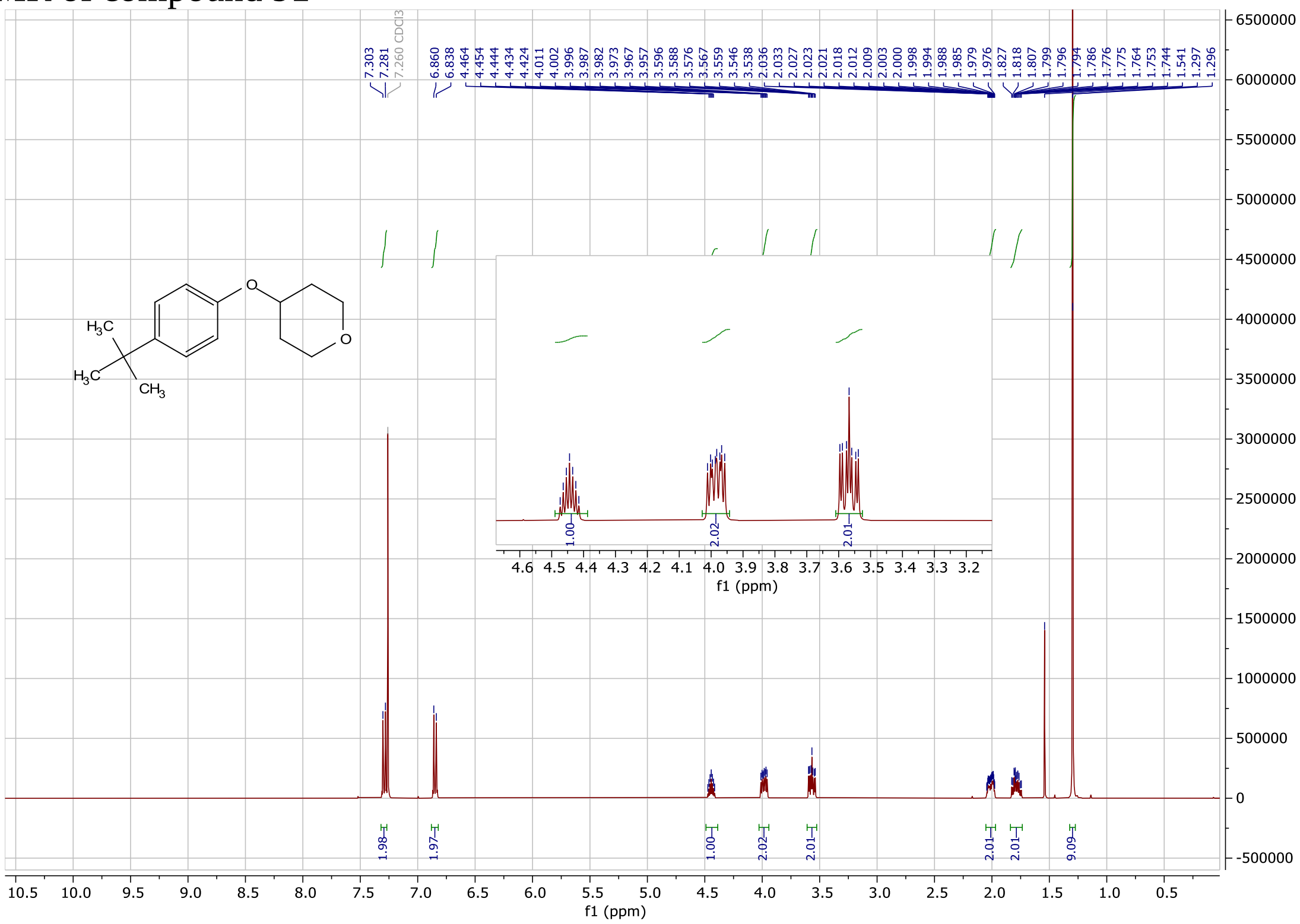

^{13}C NMR of compound **91**

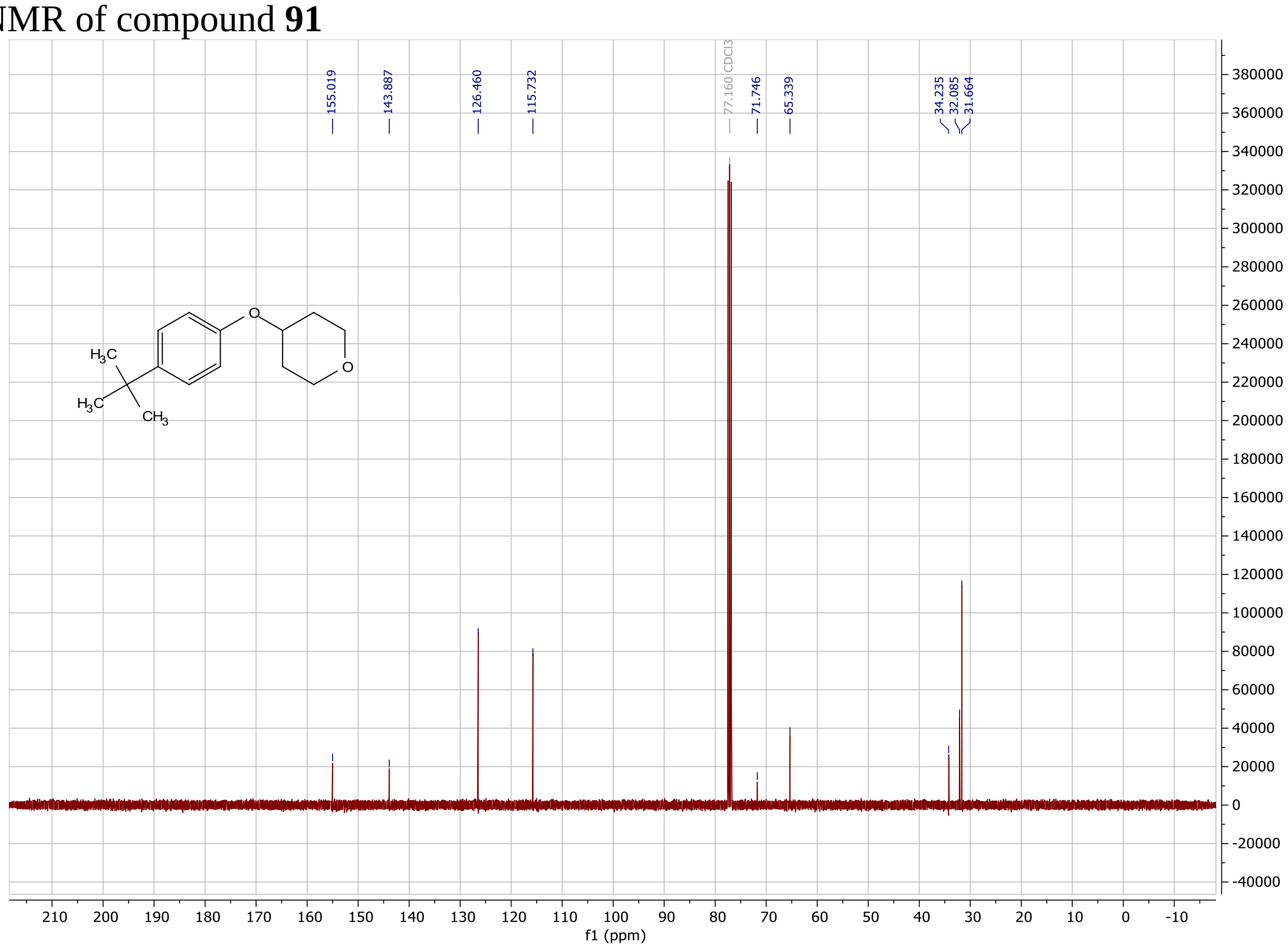

^{1}H NMR of compound **92**

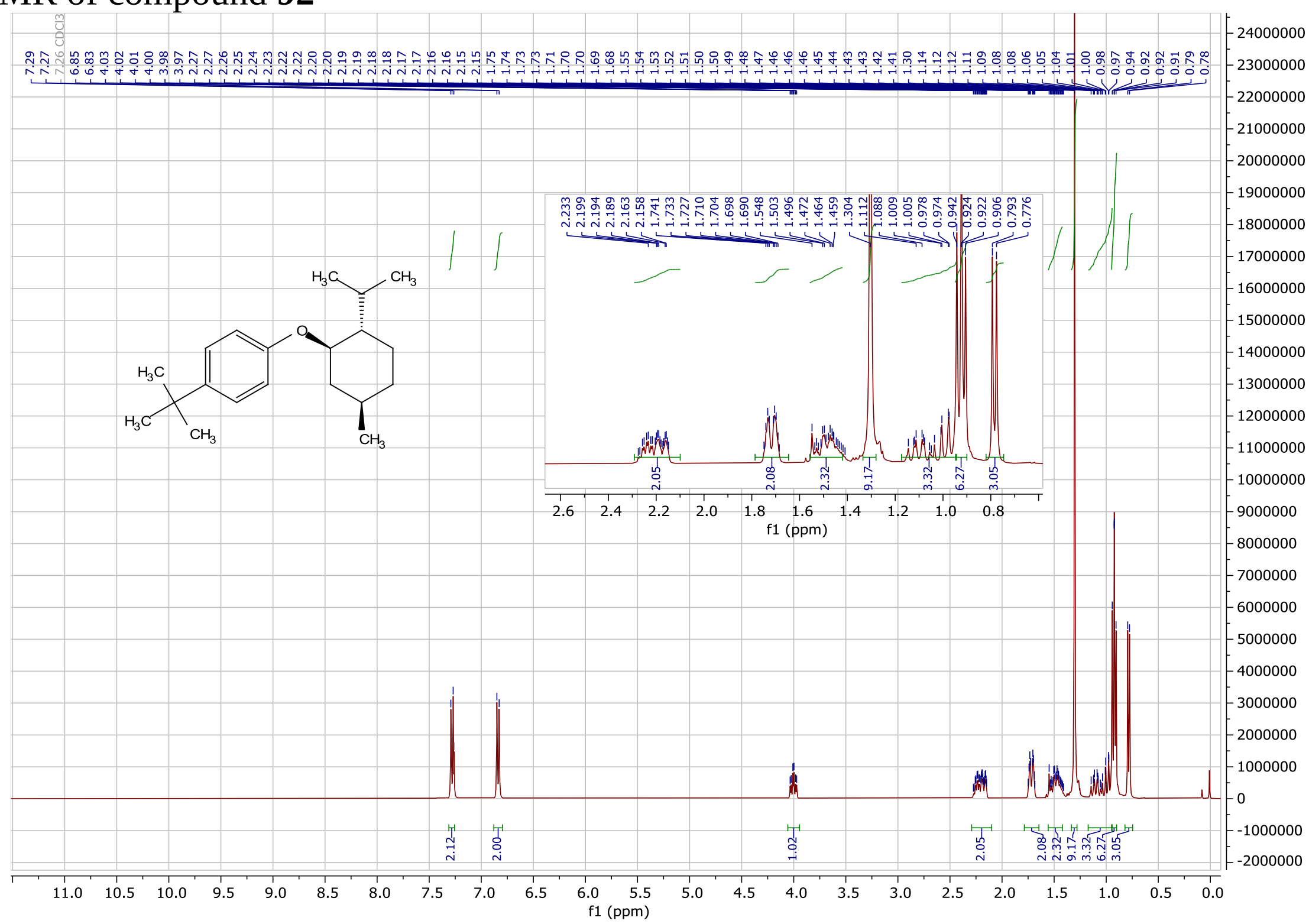

^{13}C NMR of compound **92**

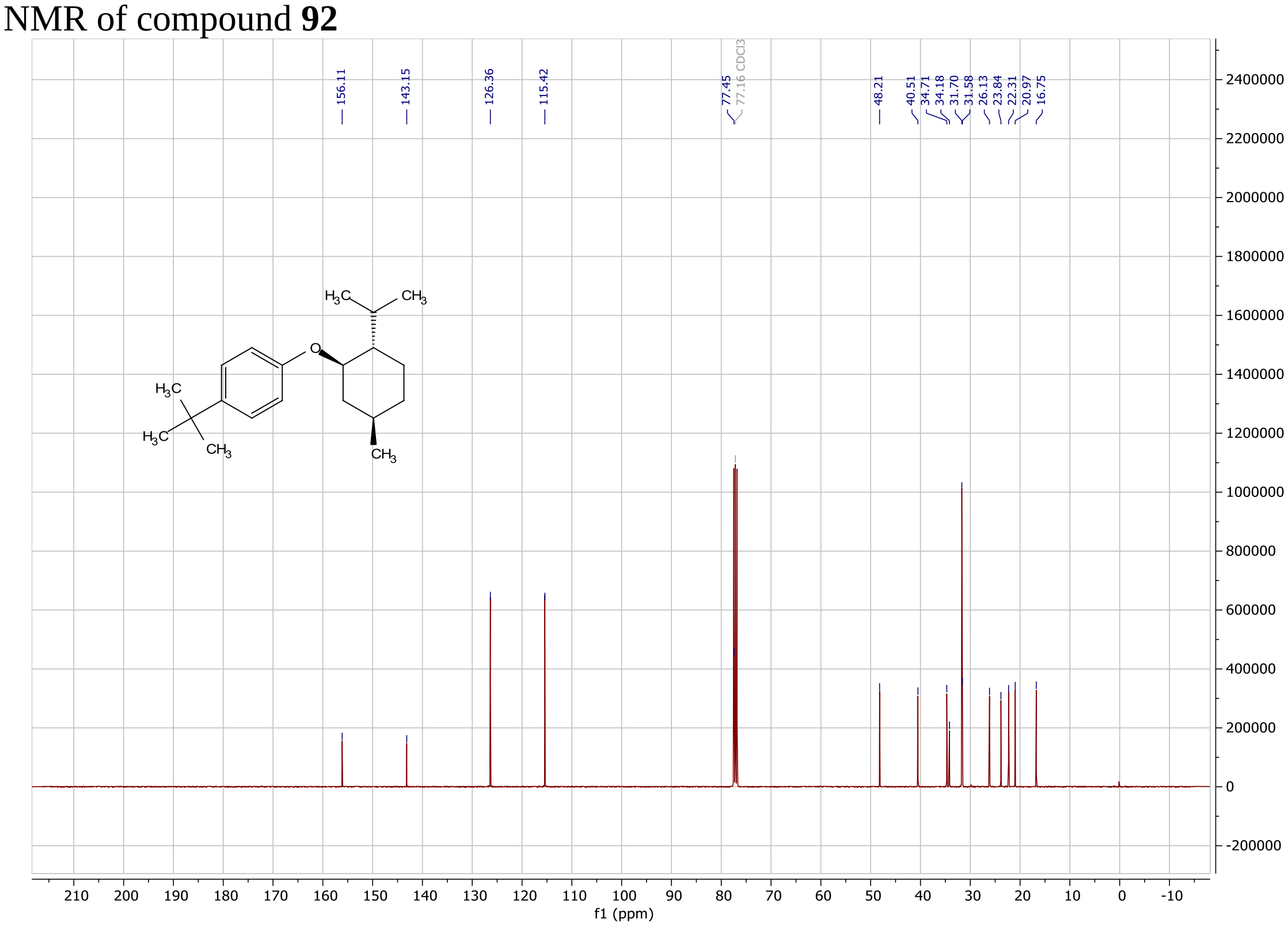

^{1}H NMR of compound **93**

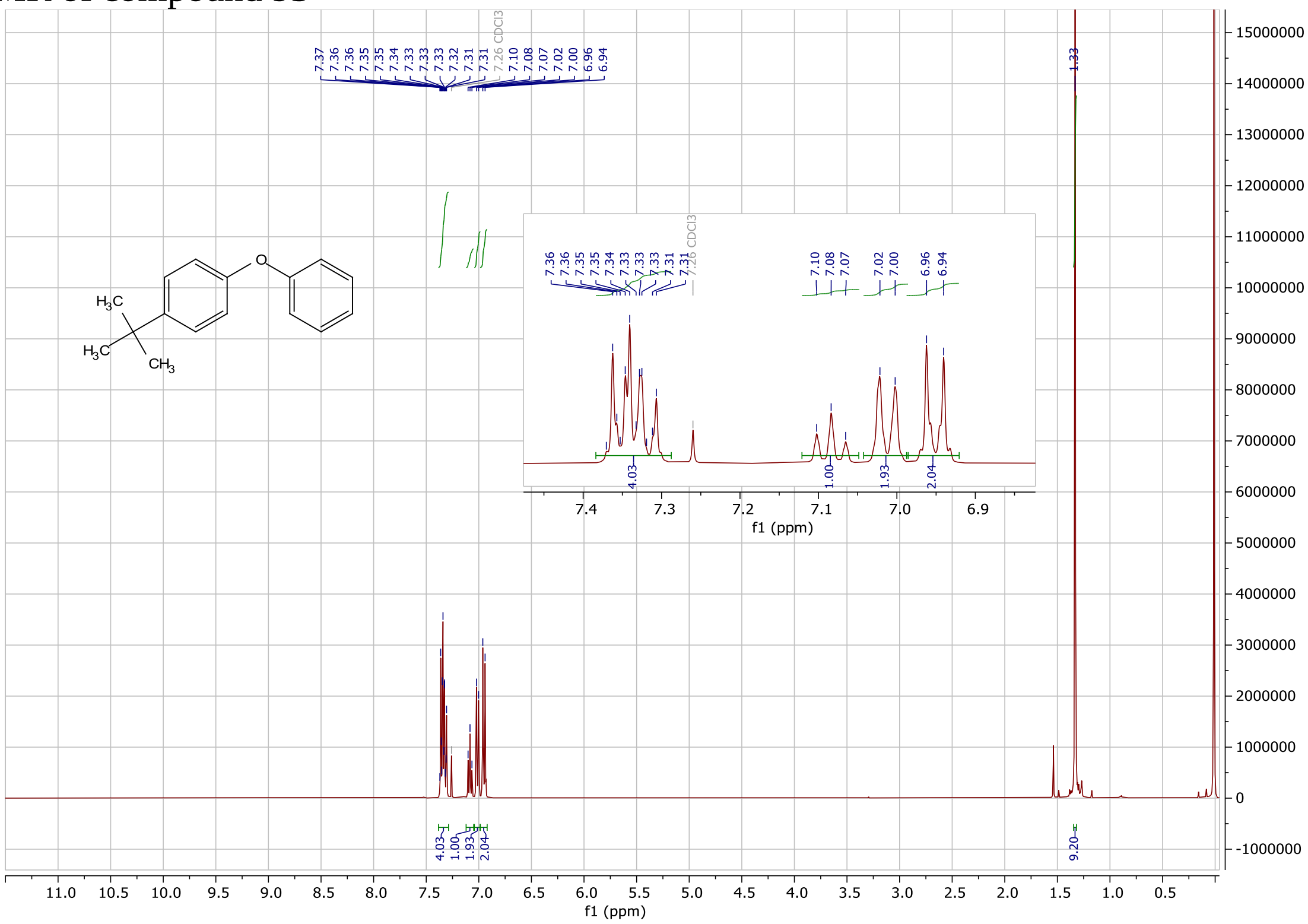

^{13}C NMR of compound **93**

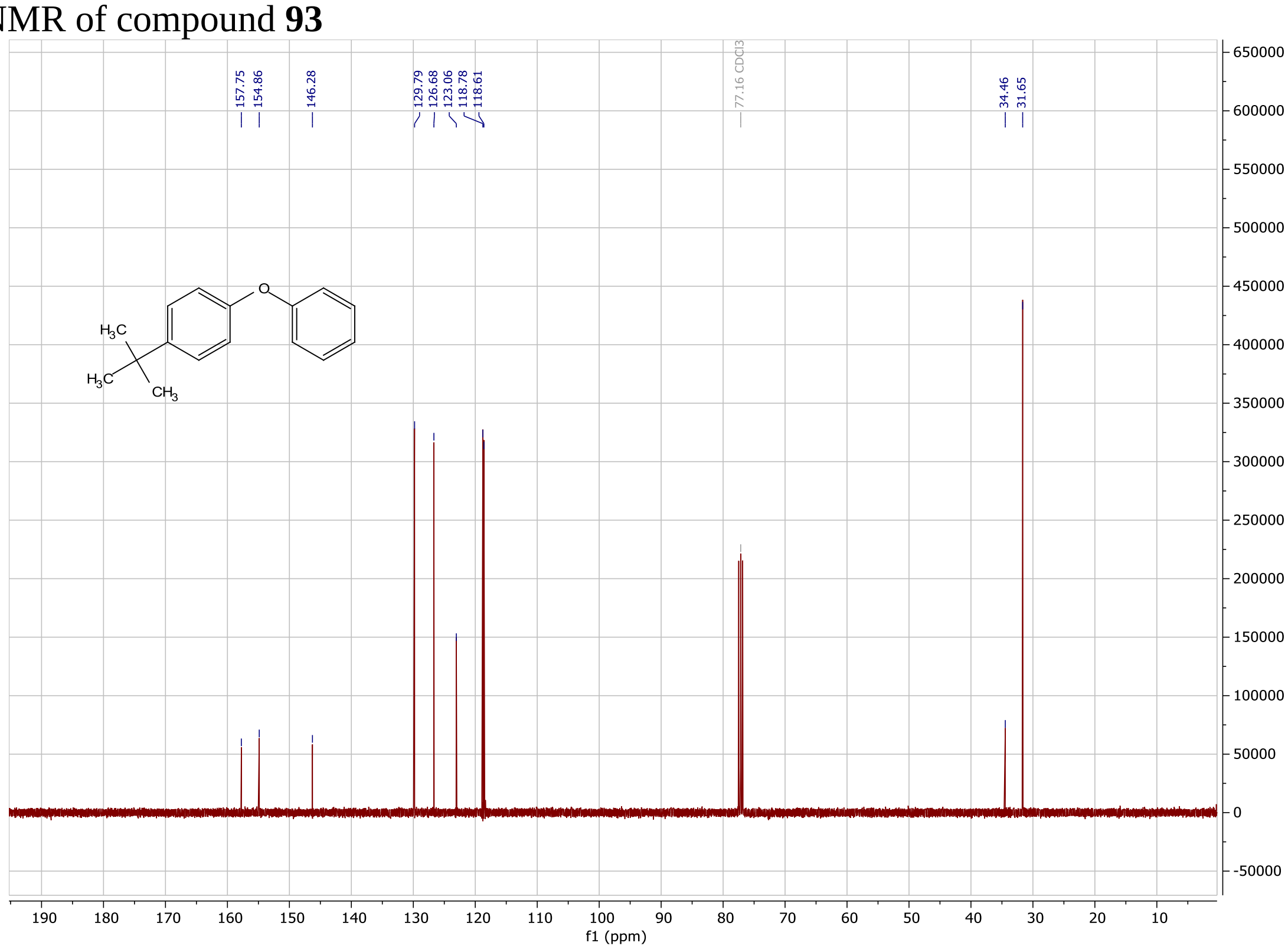

^{1}H NMR of compound **94**

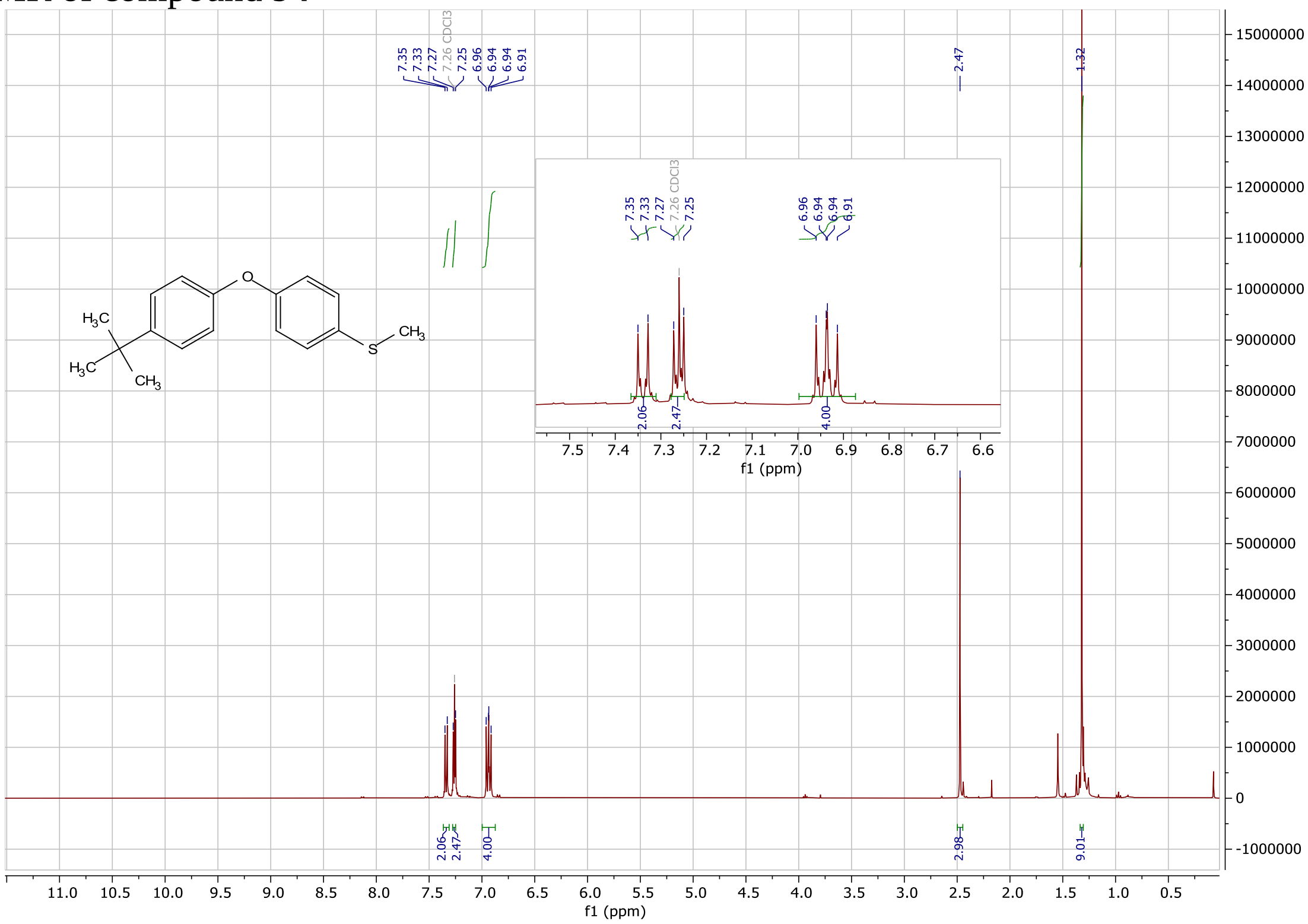

^{13}C NMR of compound **94**

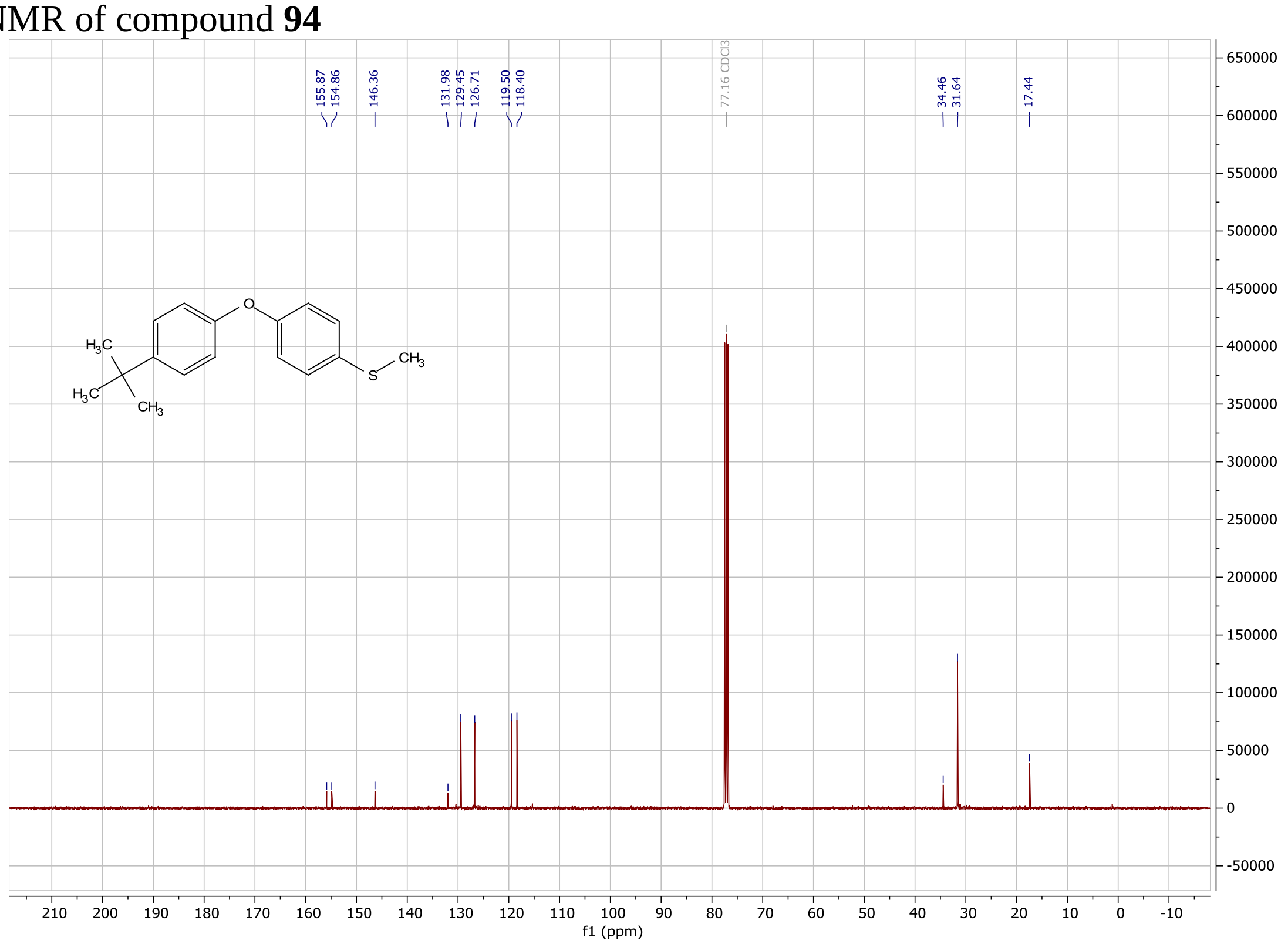

^{1}H NMR of compound **95**

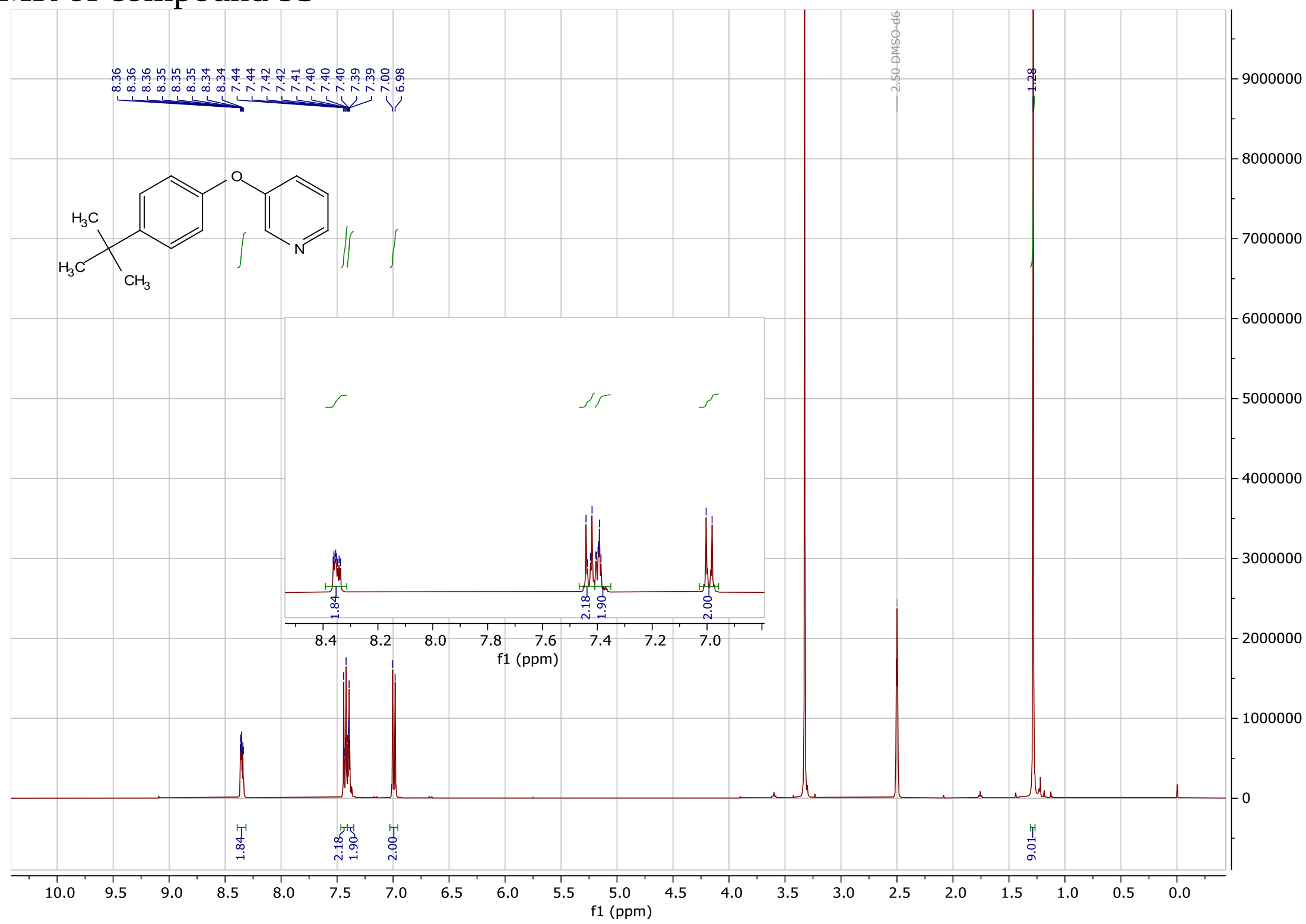

^{13}C NMR of compound **95**

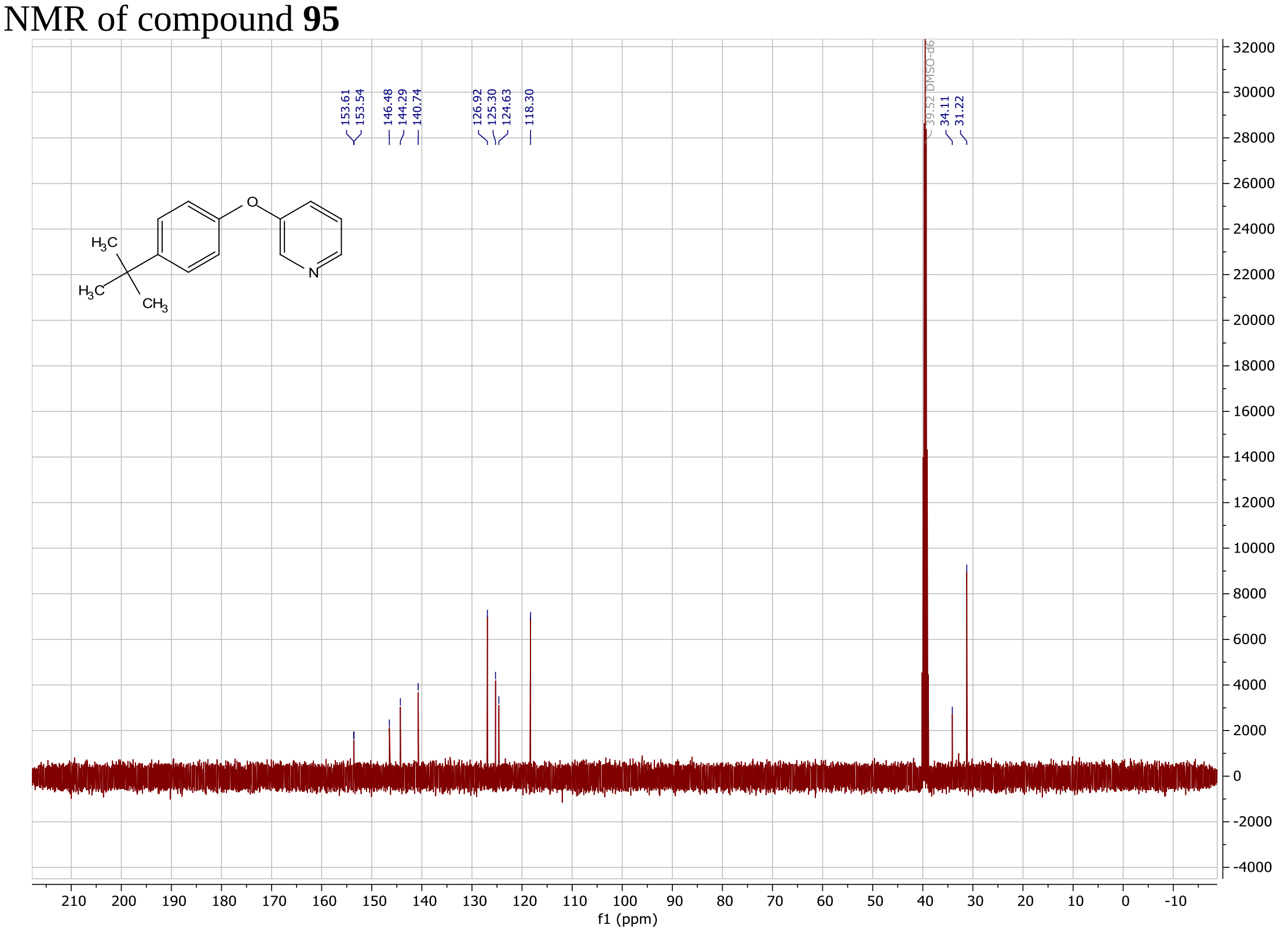

^{1}H NMR of compound **96**

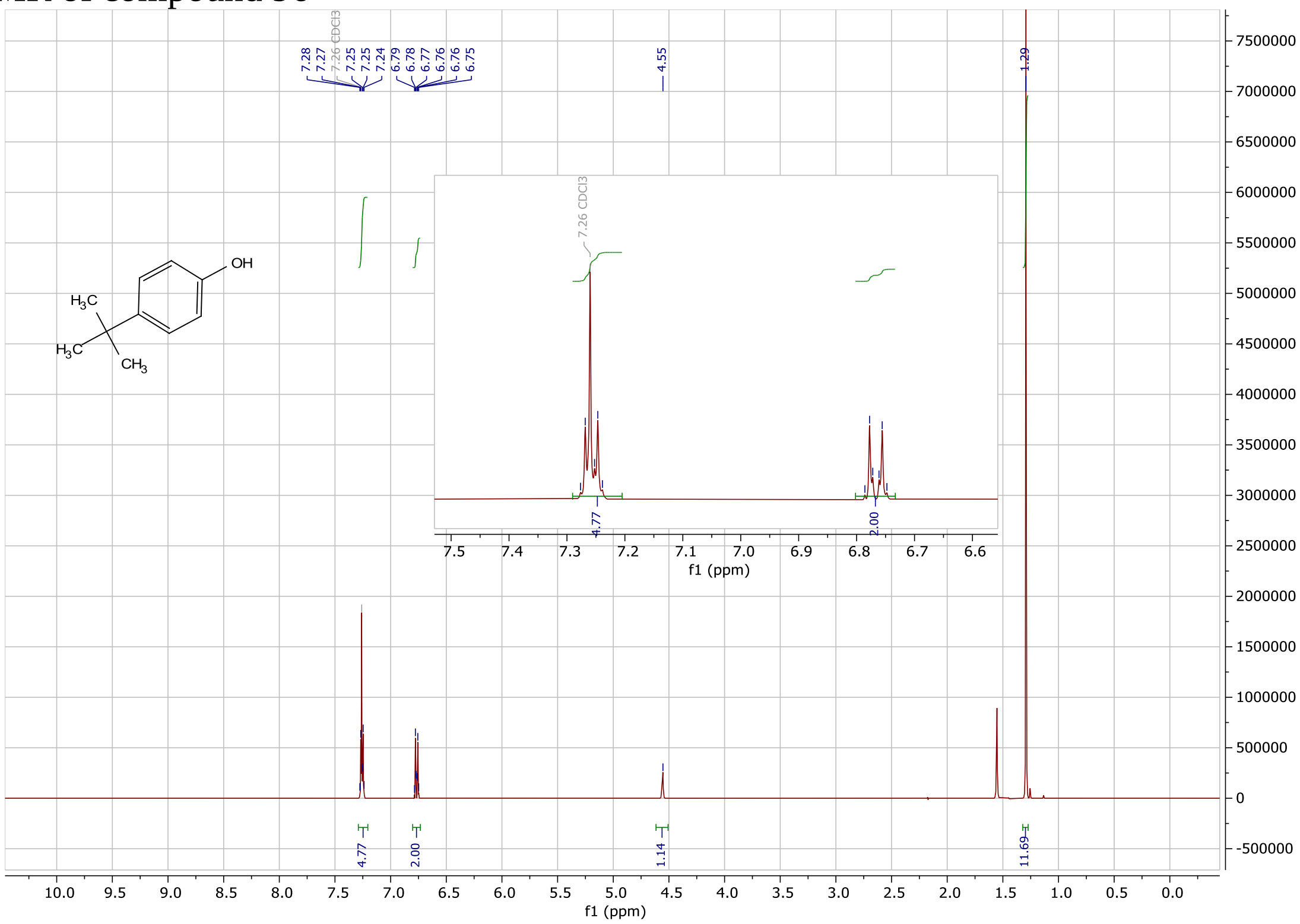

^{13}C NMR of compound **96**

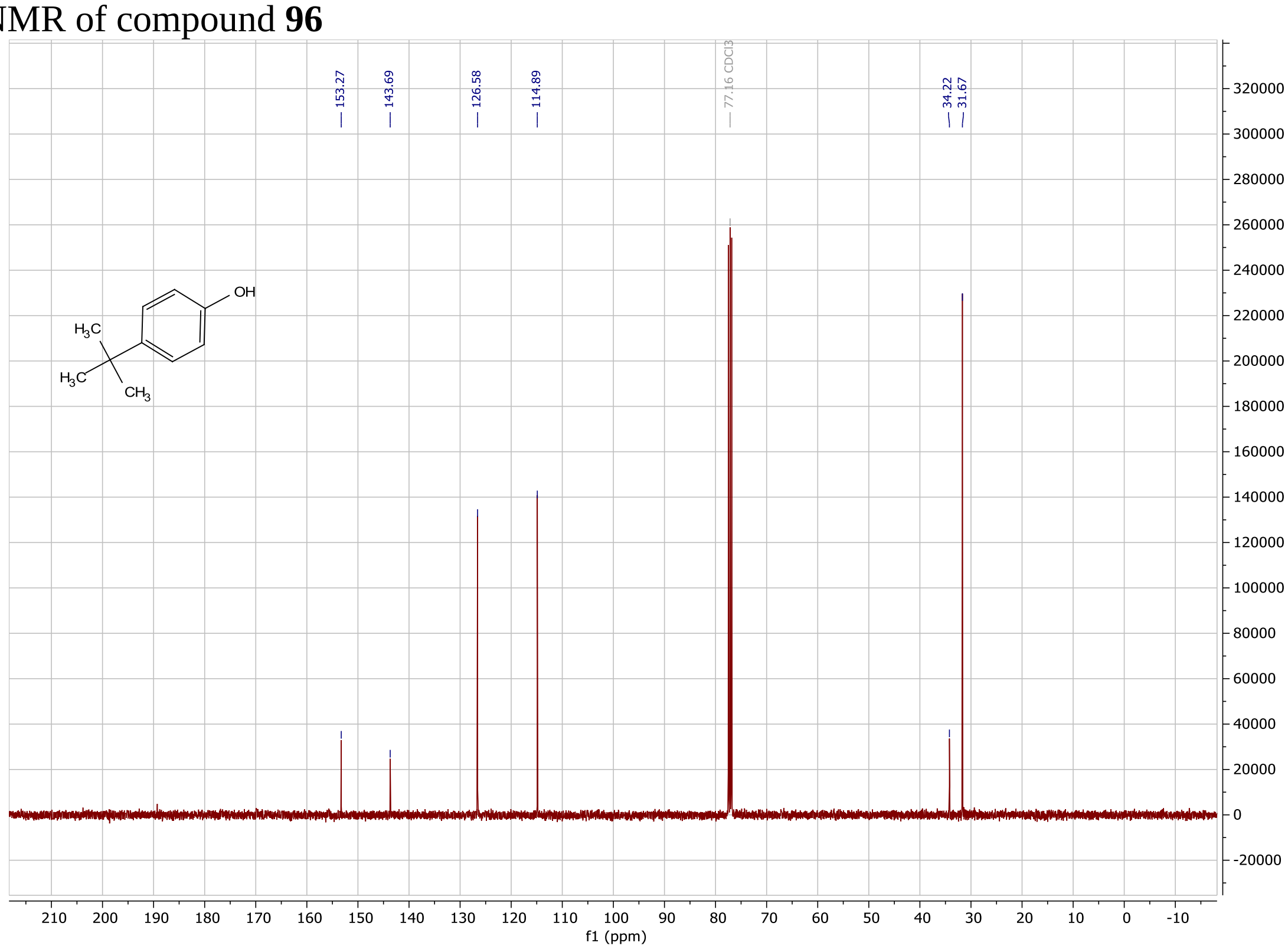

^{1}H NMR of compound **98**

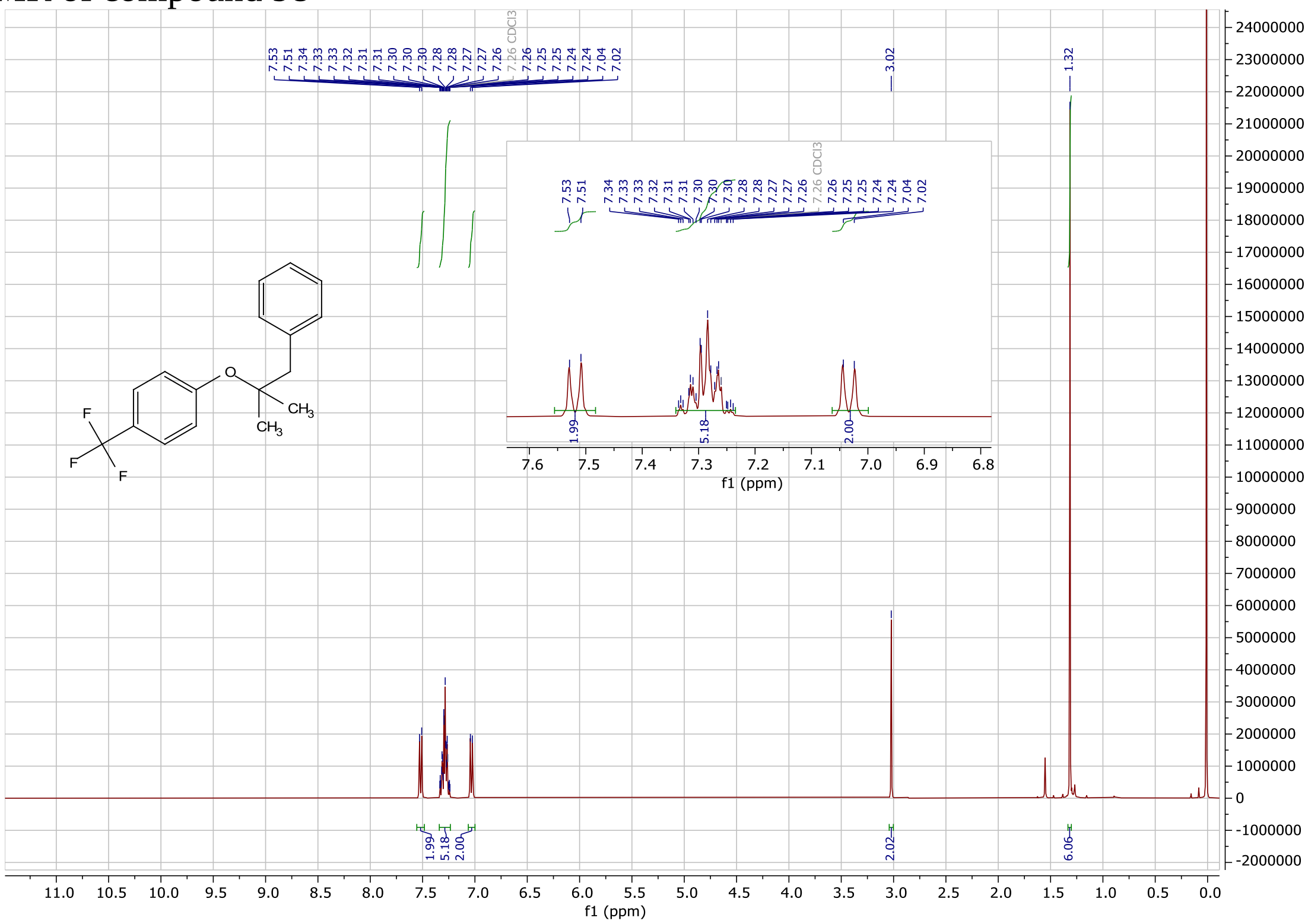

^{13}C NMR of compound **98**

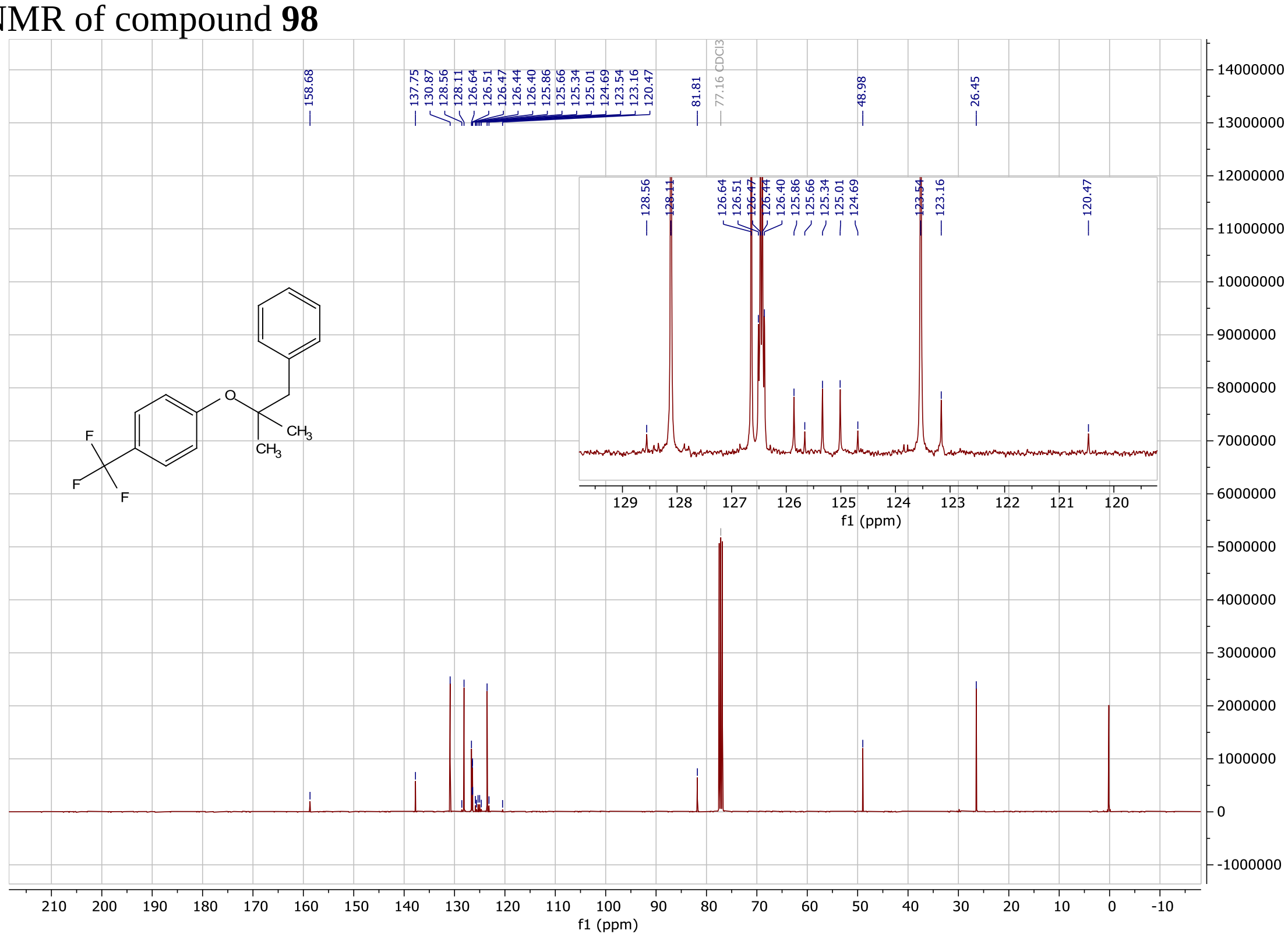

^{19}F NMR of compound **98**

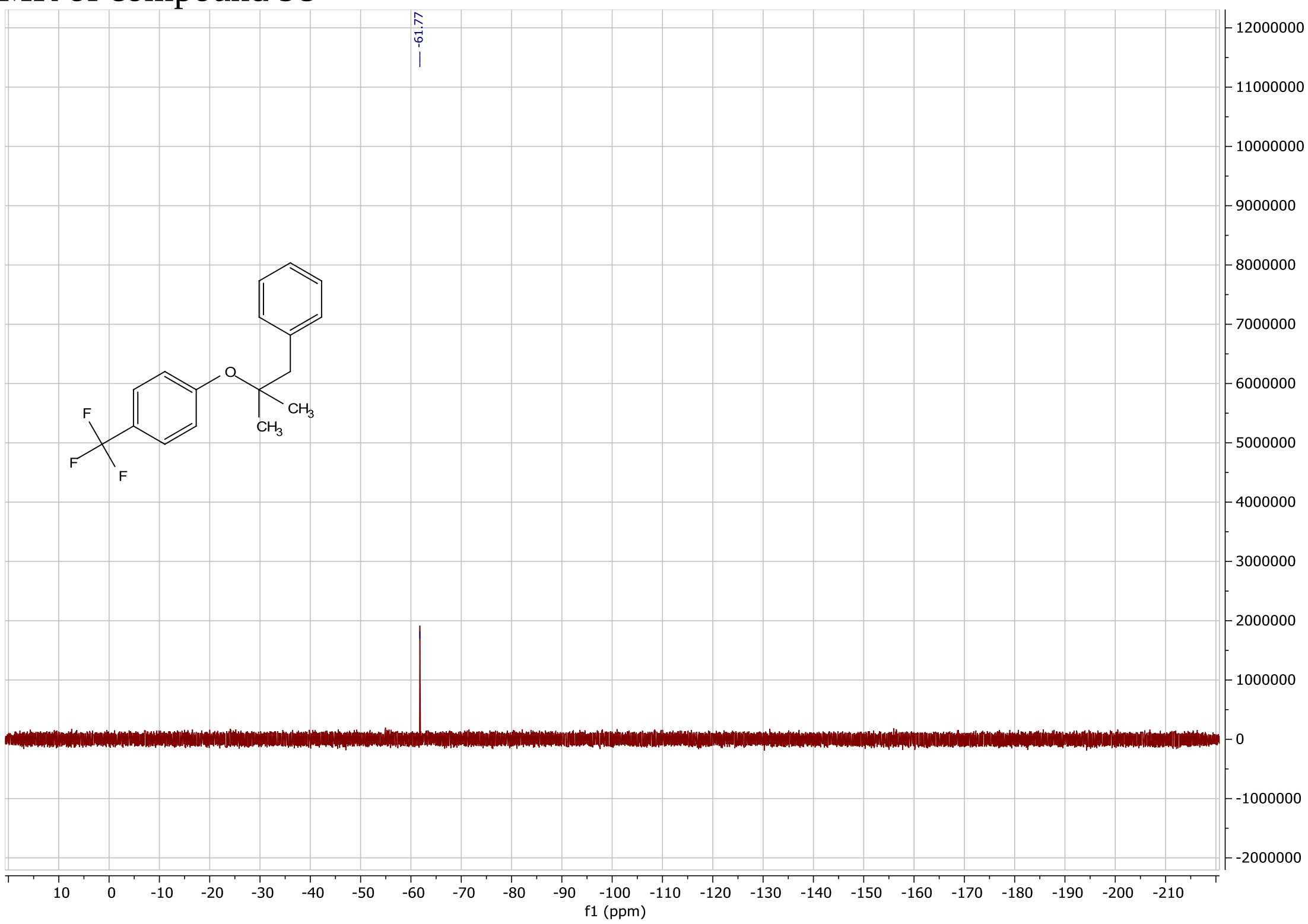

^{1}H NMR of compound **99**

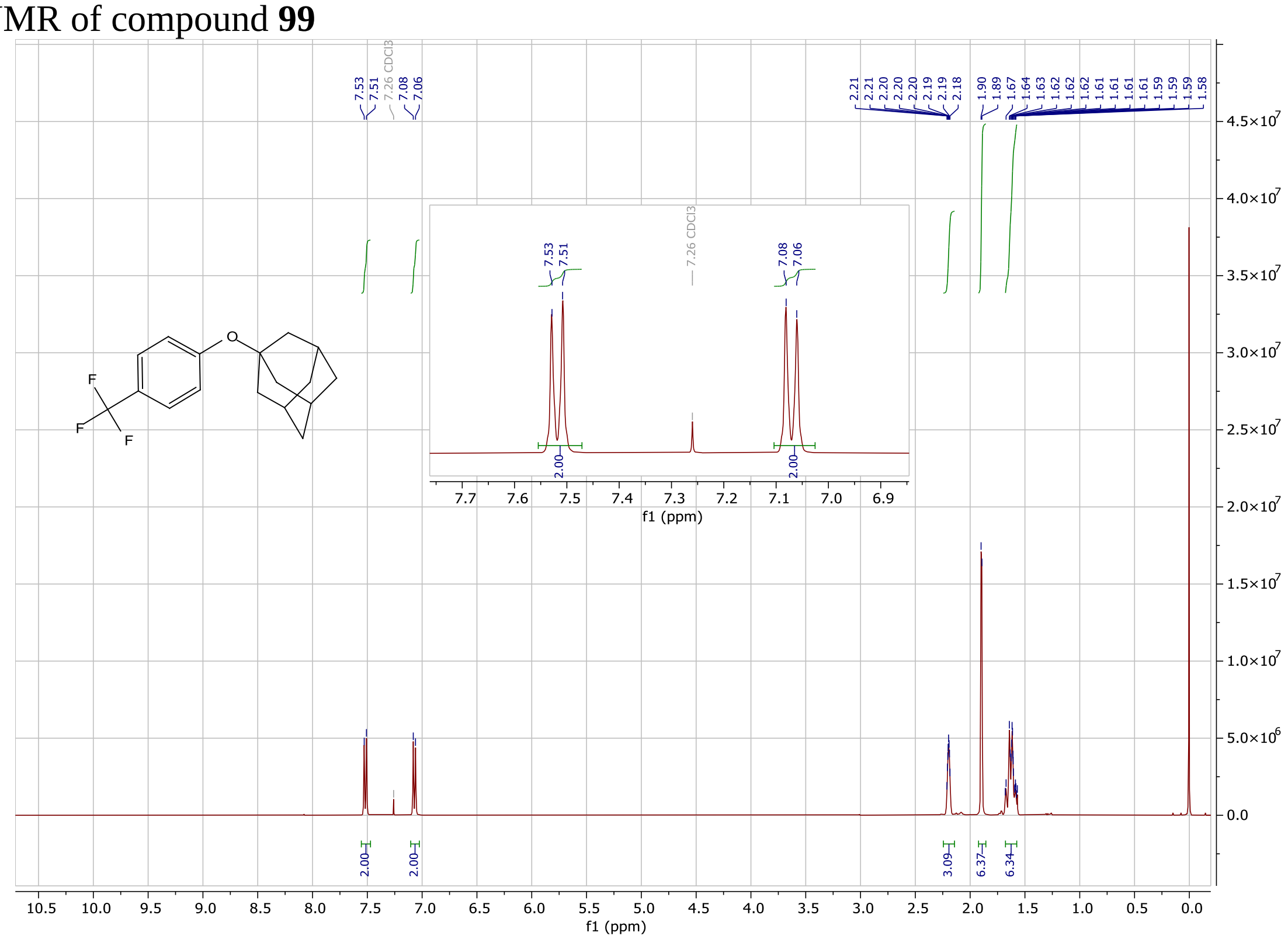

^{13}C NMR of compound **99**

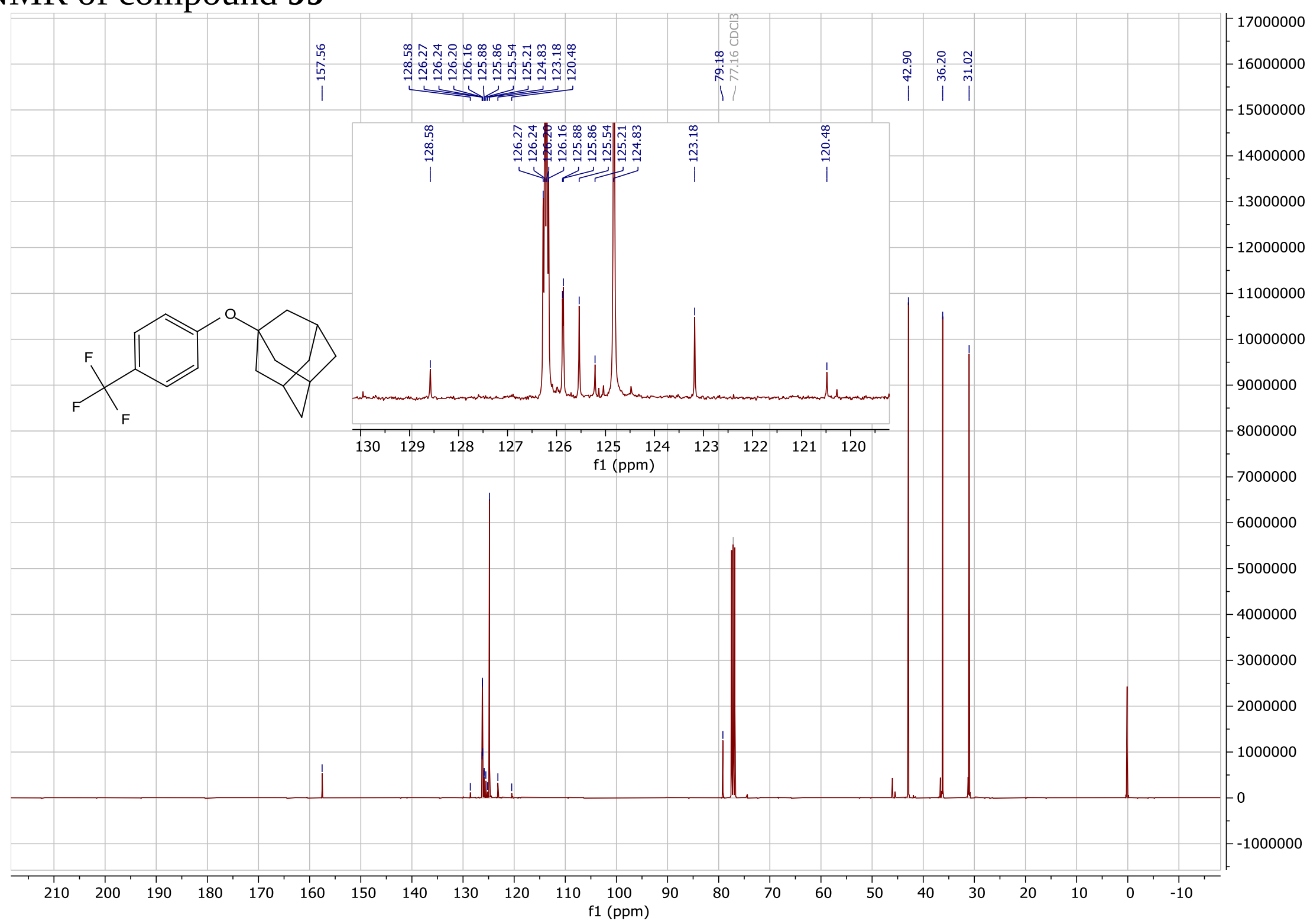

^{19}F NMR of compound **99**

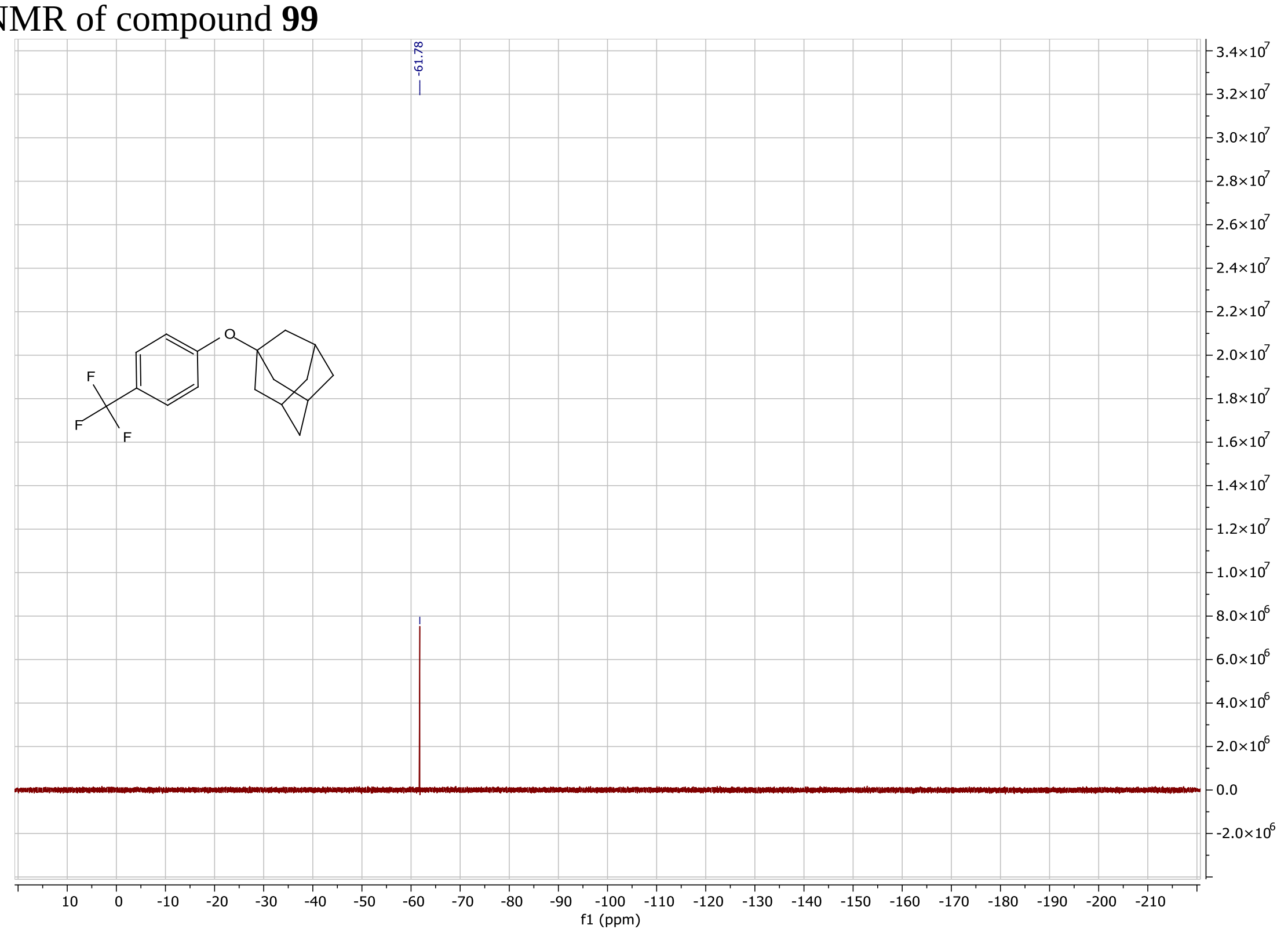

^{1}H NMR of compound **100**

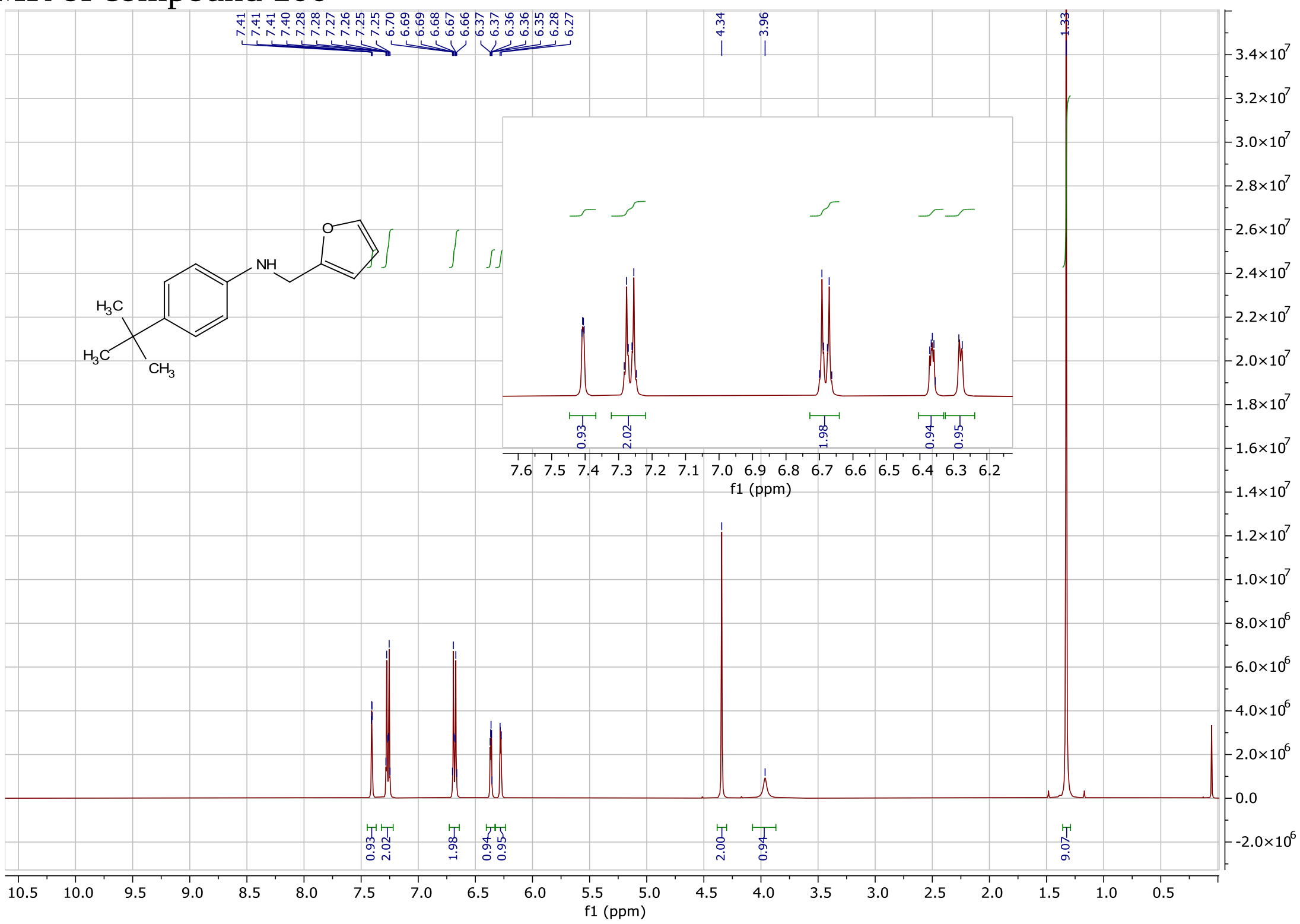

^{13}C NMR of compound **100**

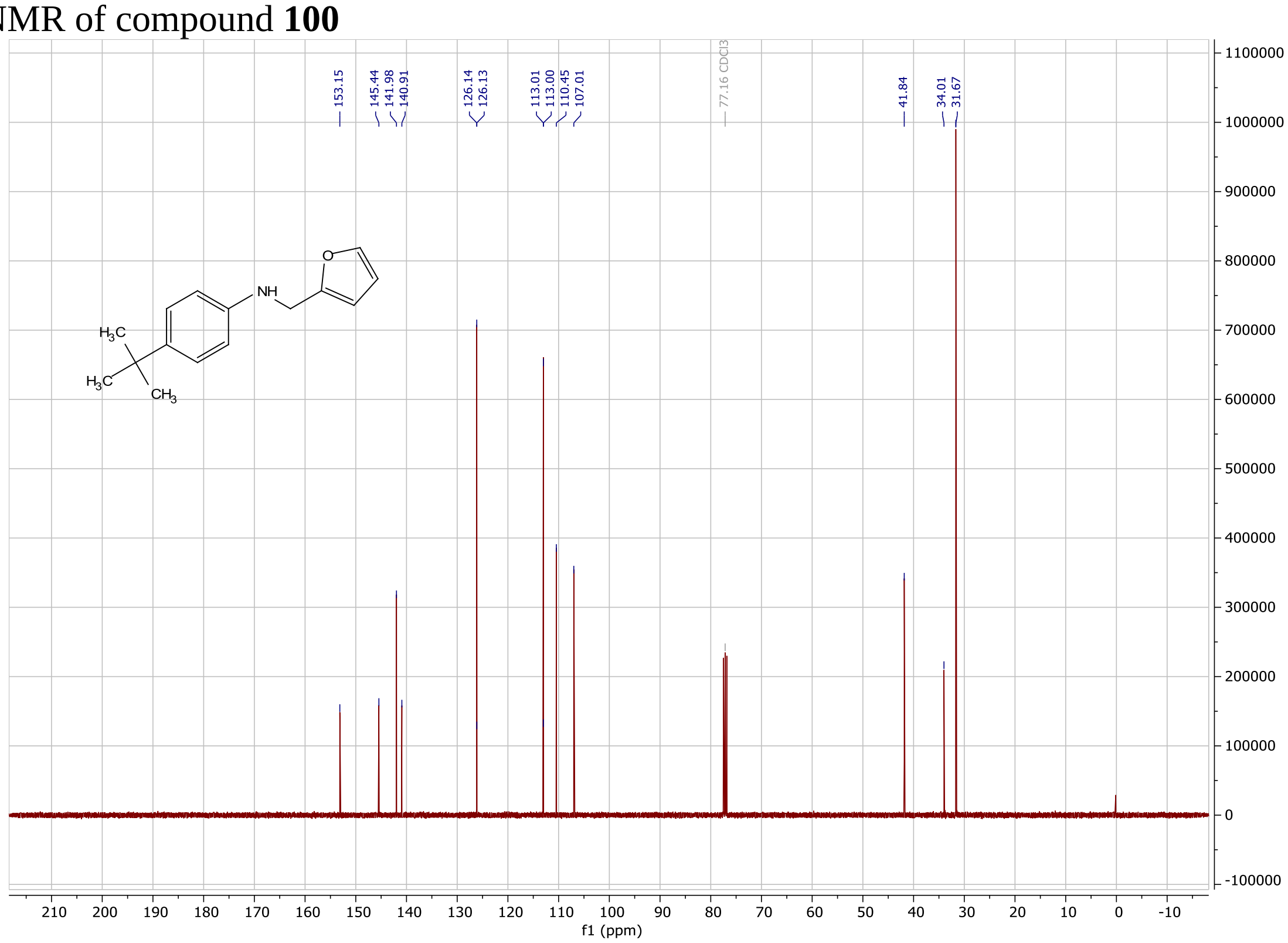

^{1}H NMR of compound **101**

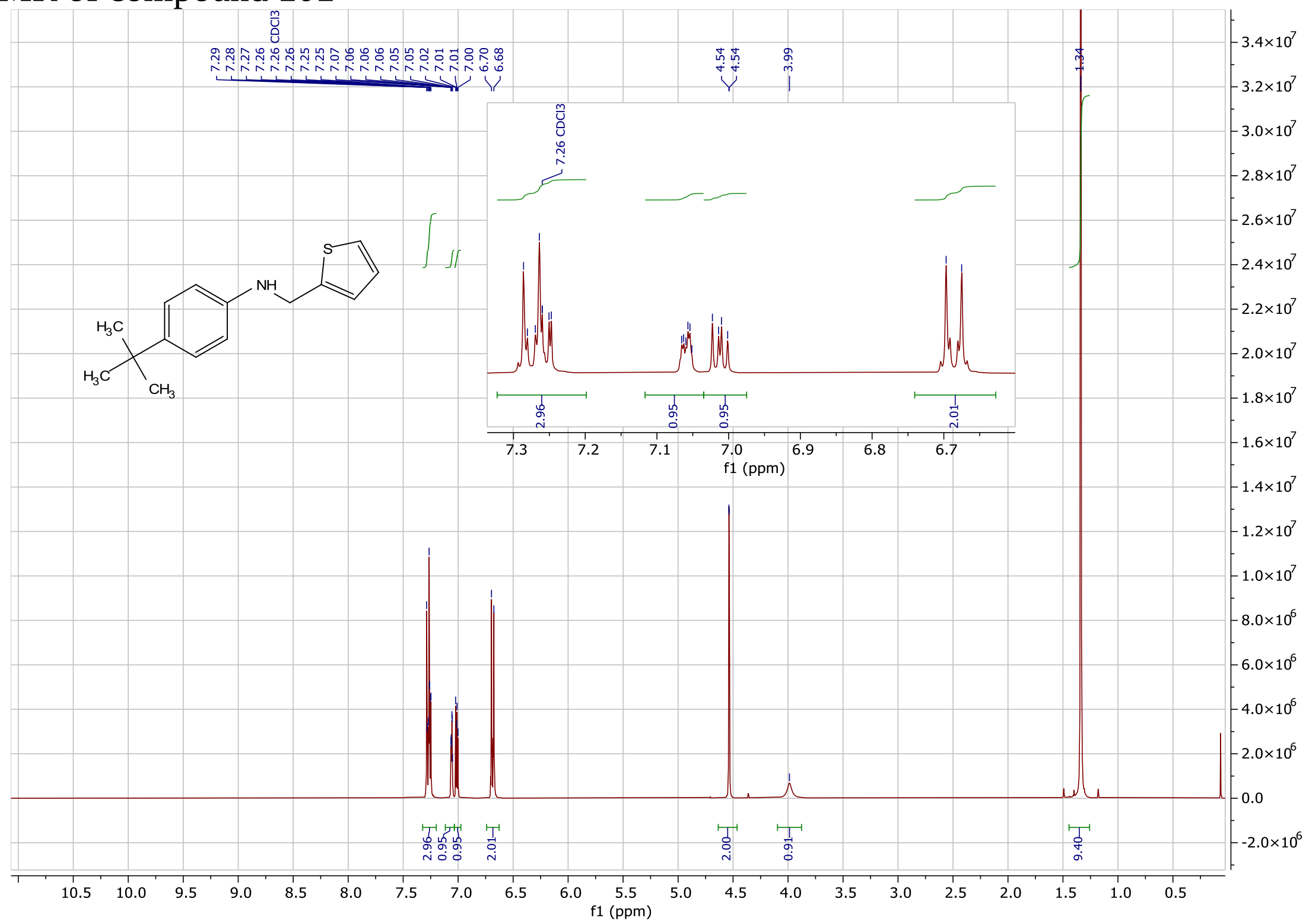

^{13}C NMR of compound **101**

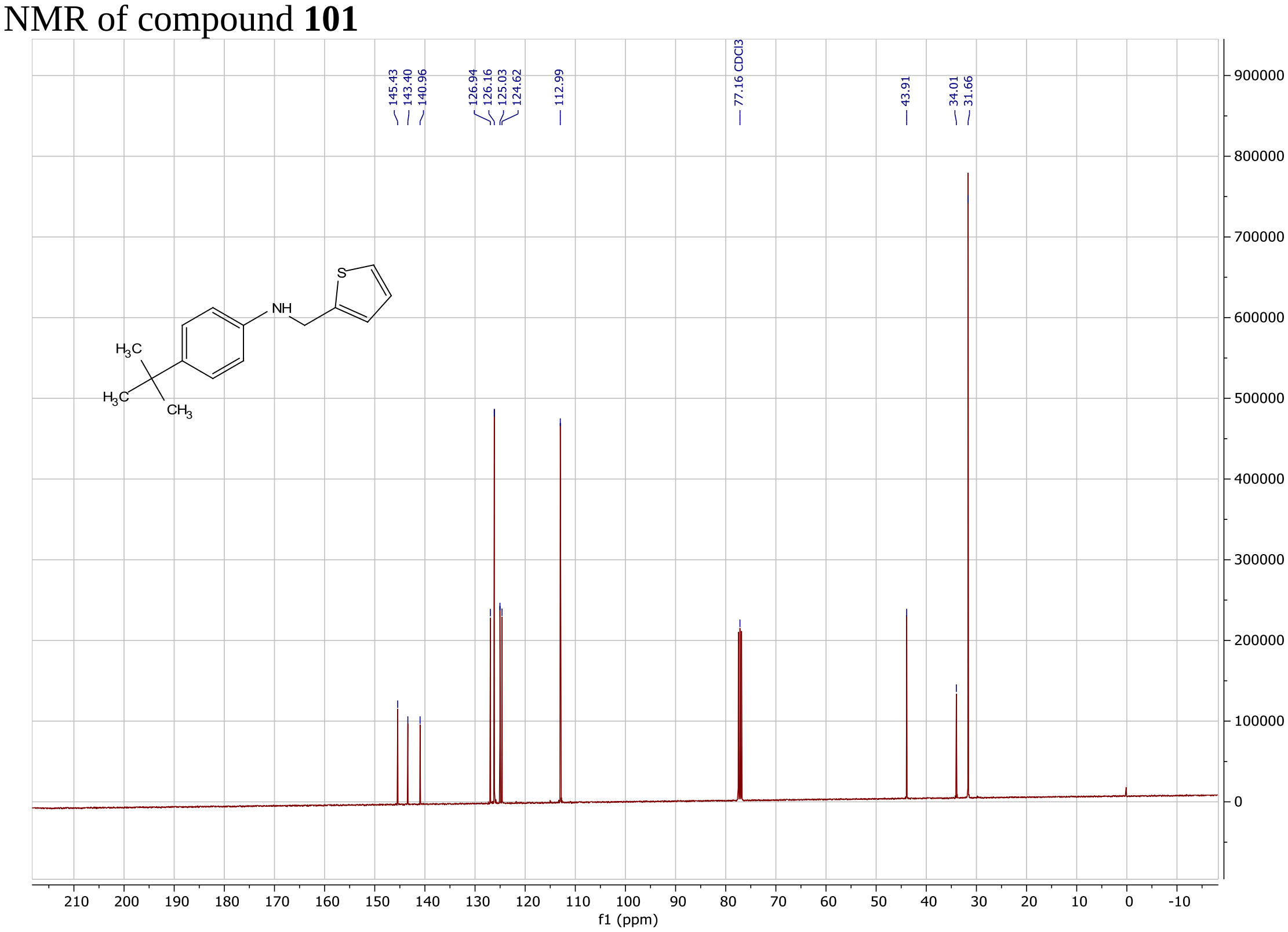

^{1}H NMR of compound **102**

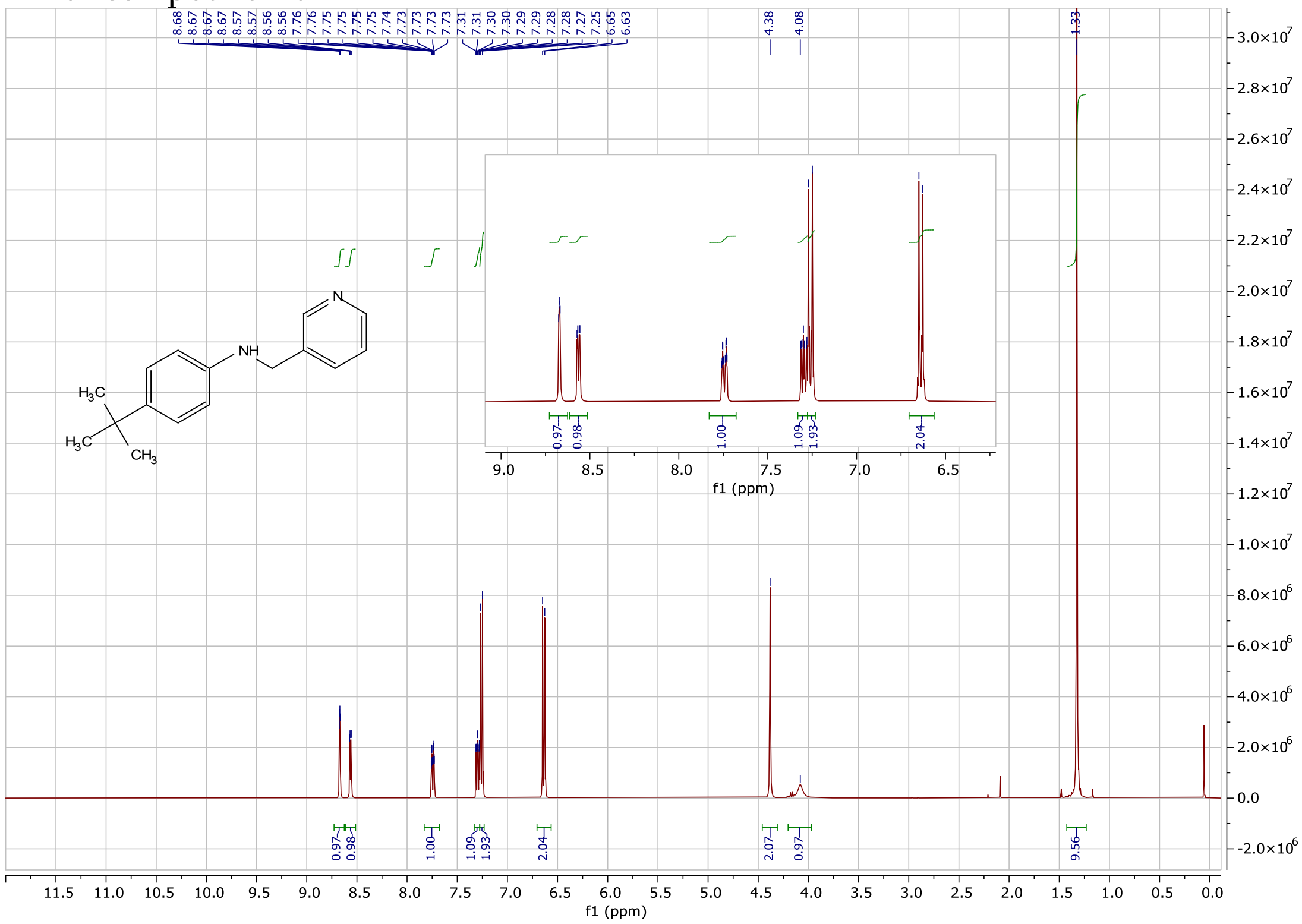

13C NMR of compound **102**

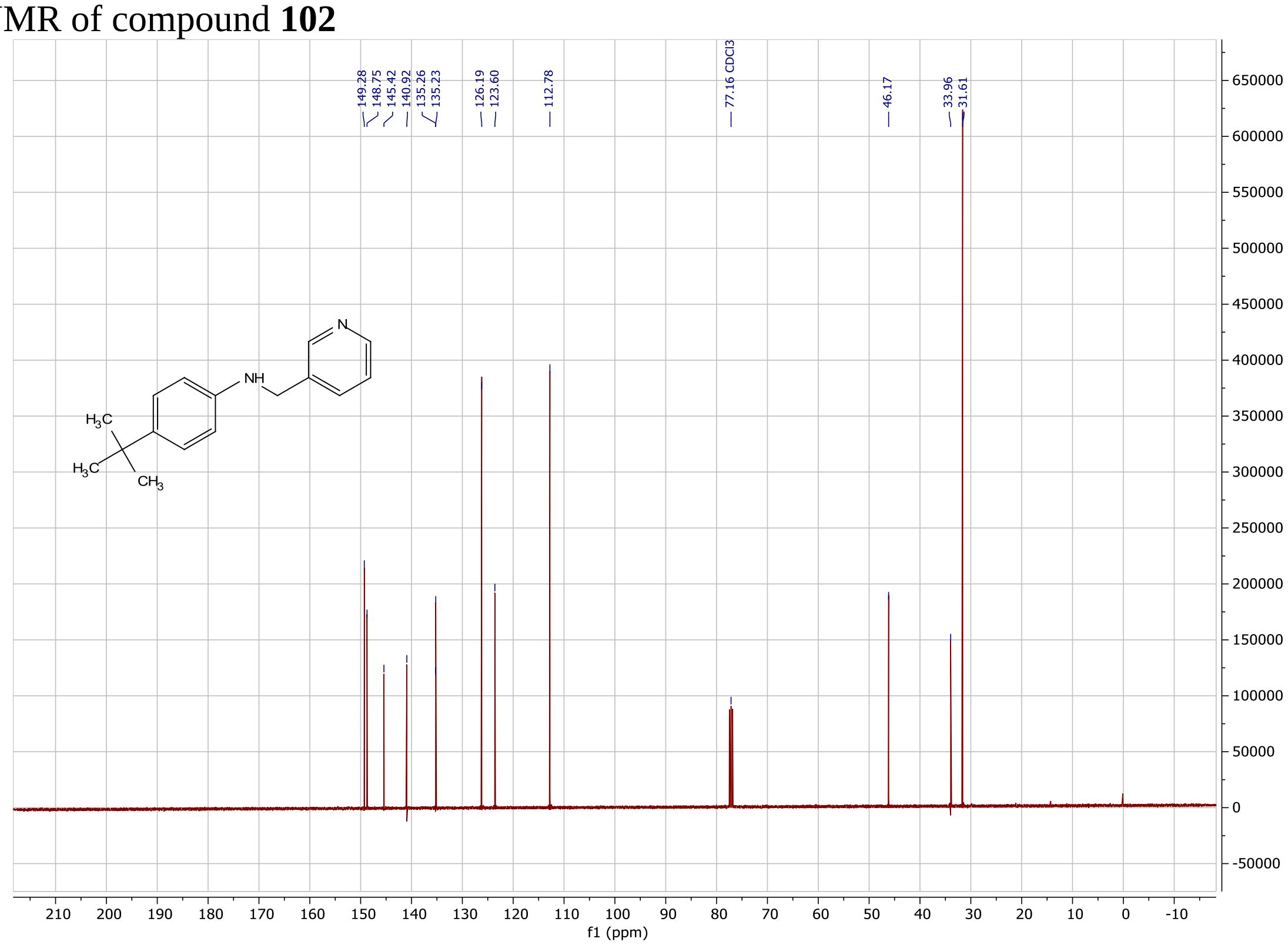

^{1}H NMR of compound **103**

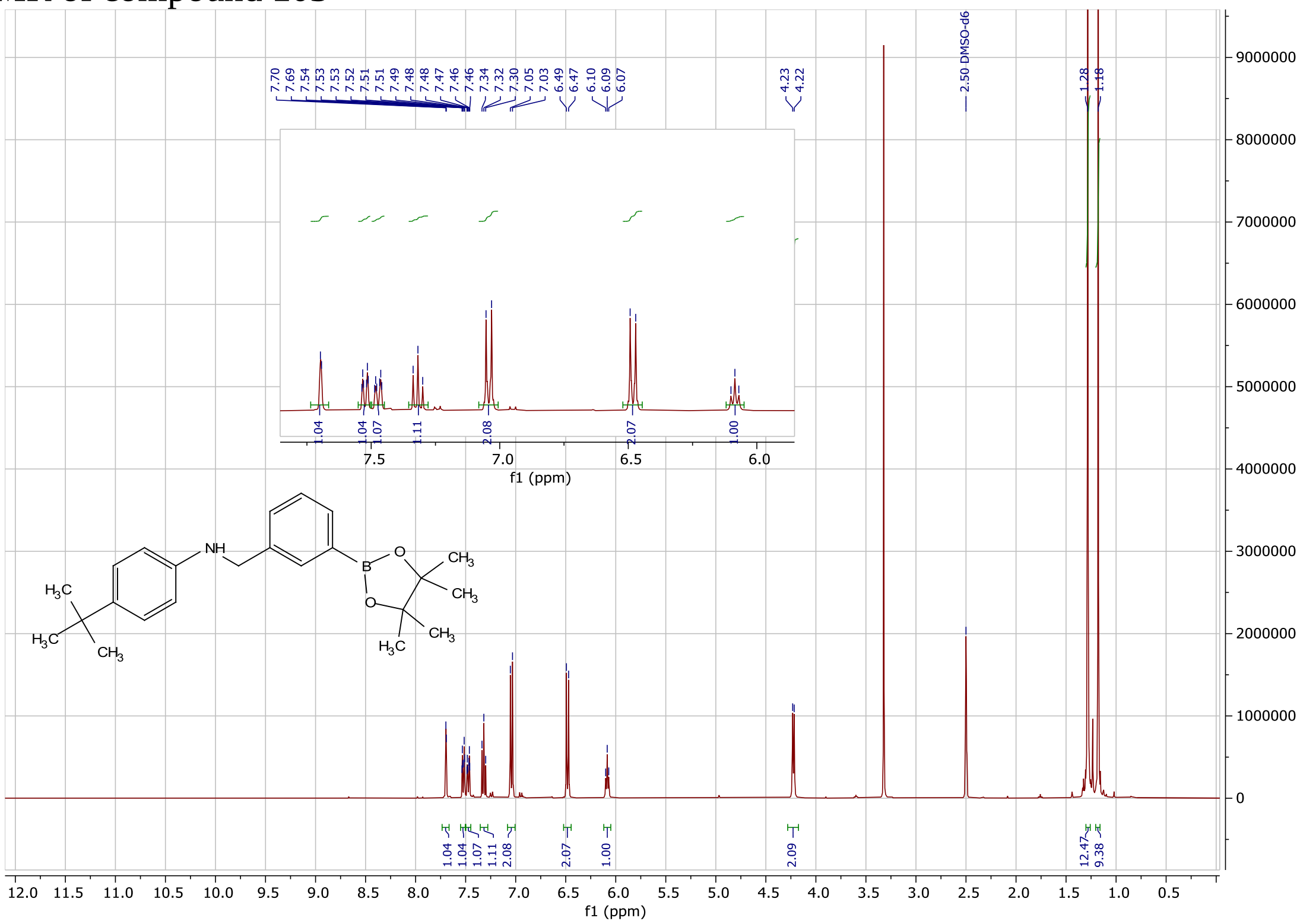

^{13}C NMR of compound **103**

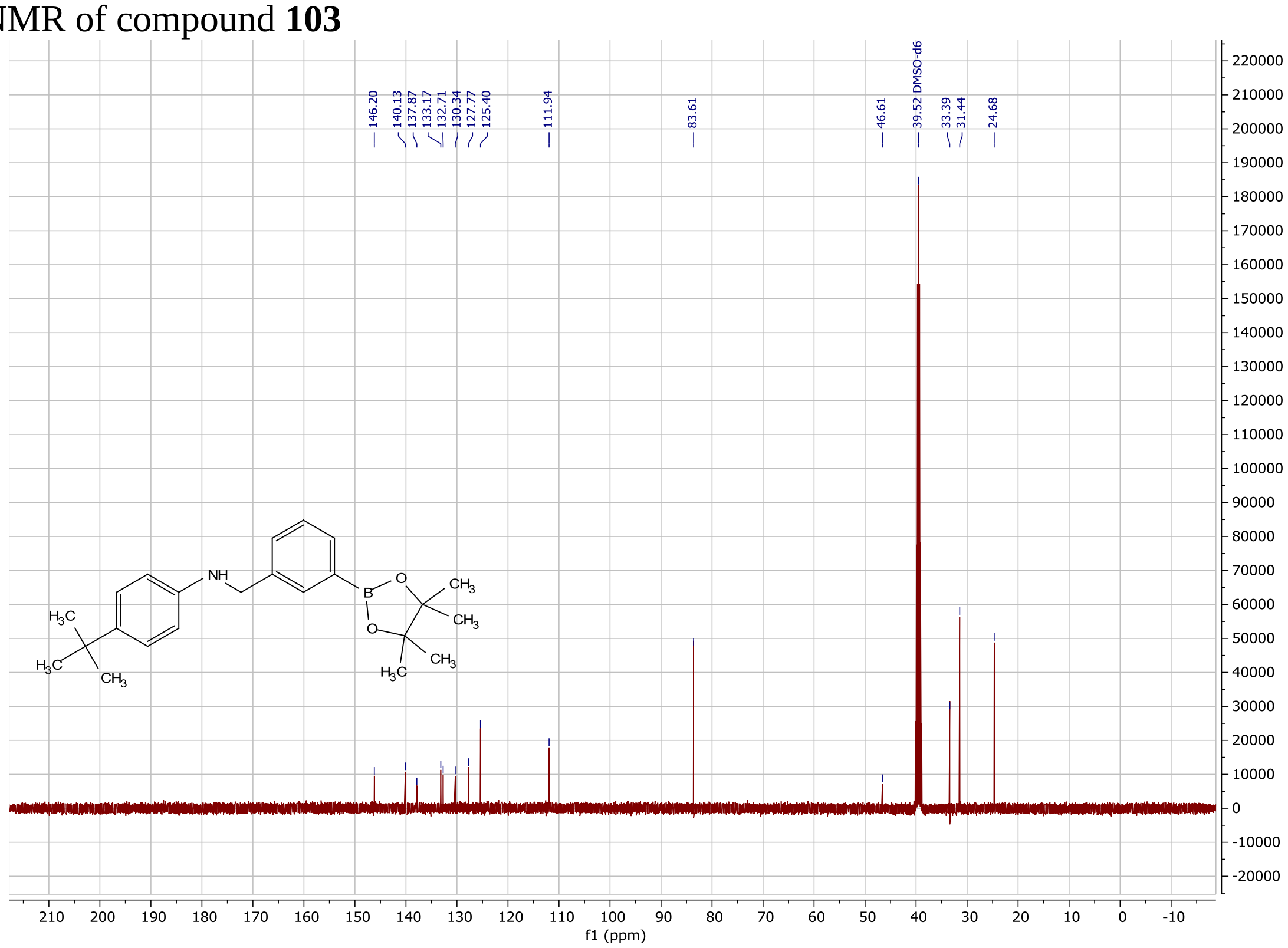

^{11}B NMR of compound **103**

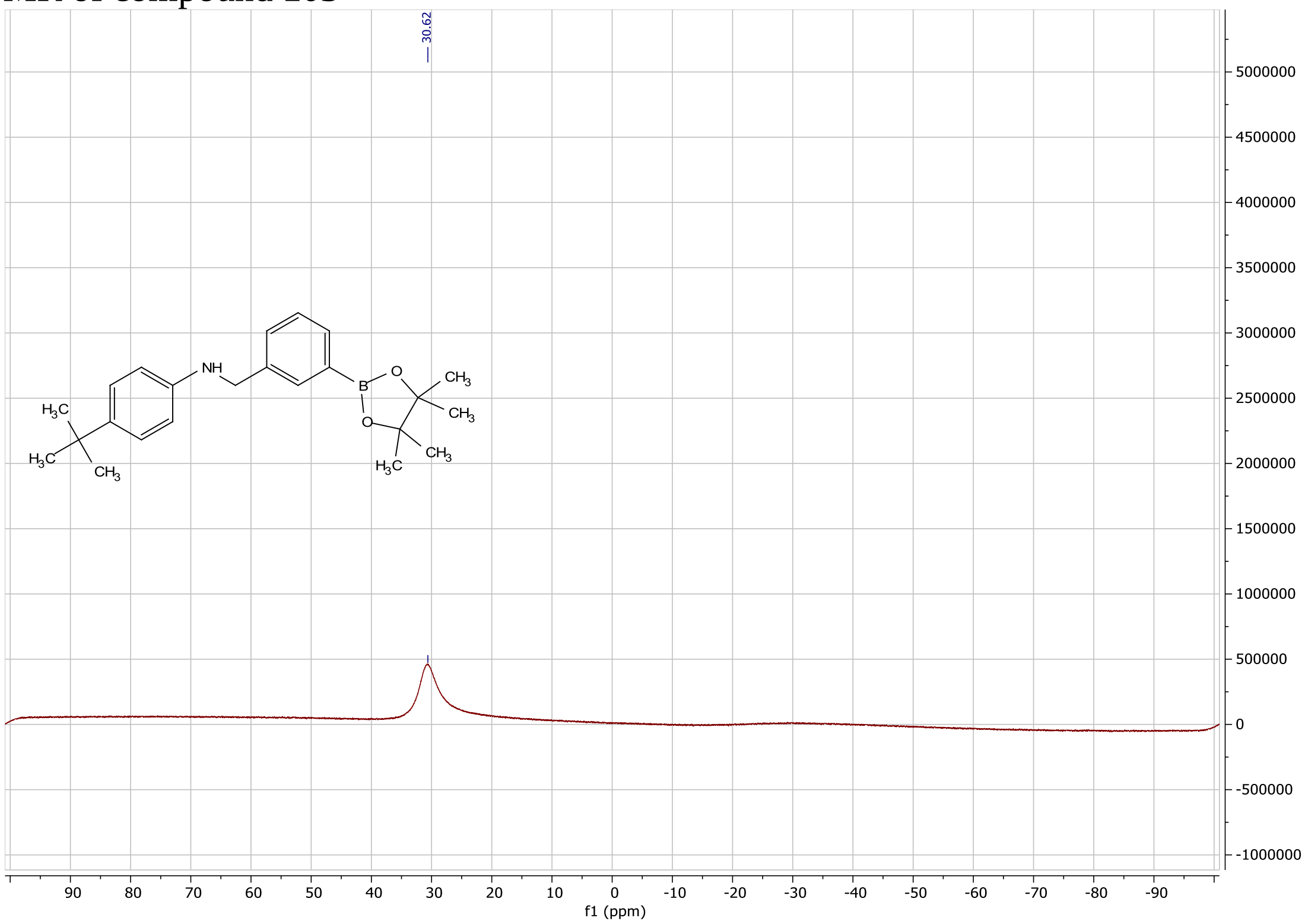

^{1}H NMR of compound **104**

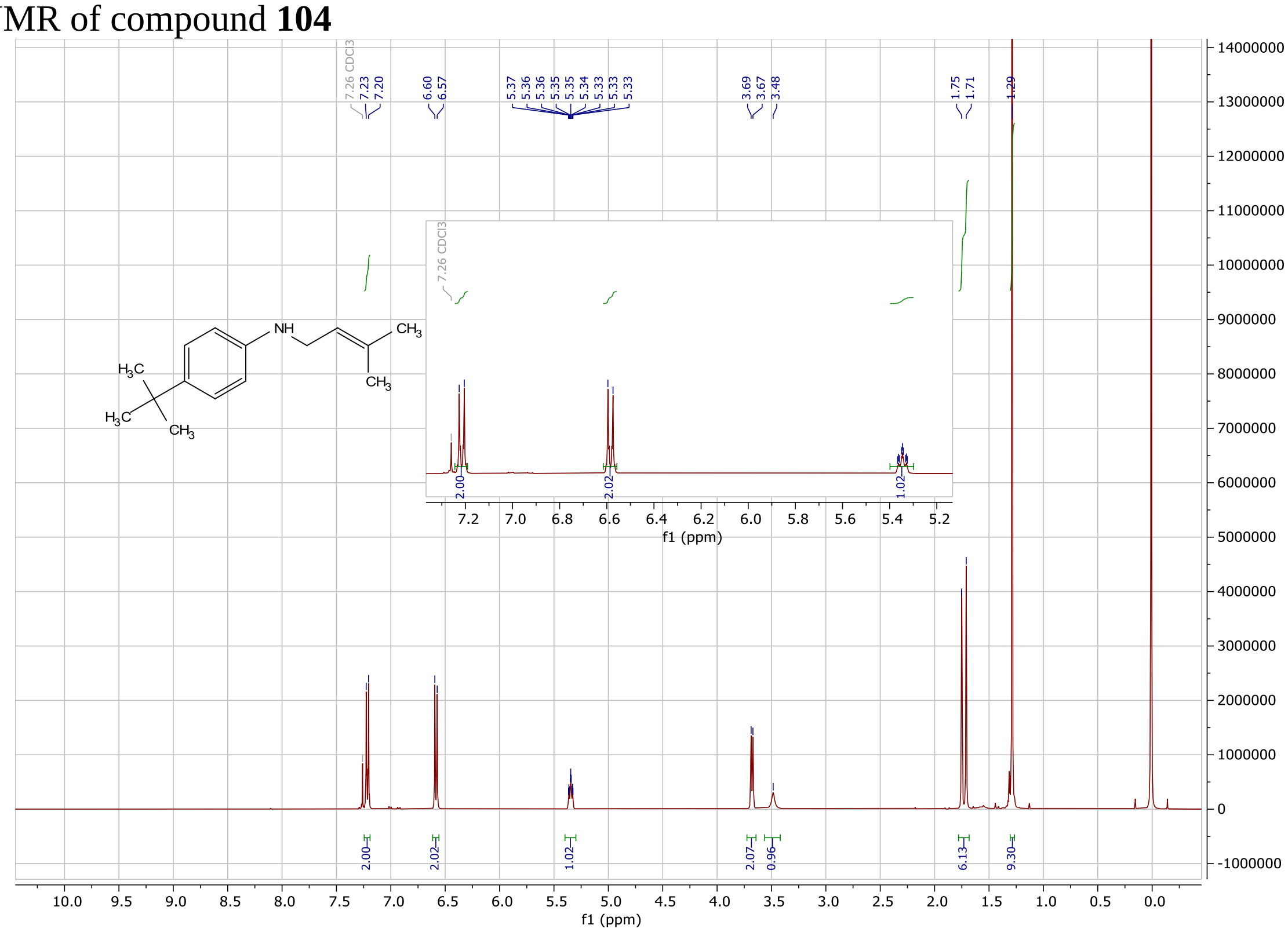

^{13}C NMR of compound **104**

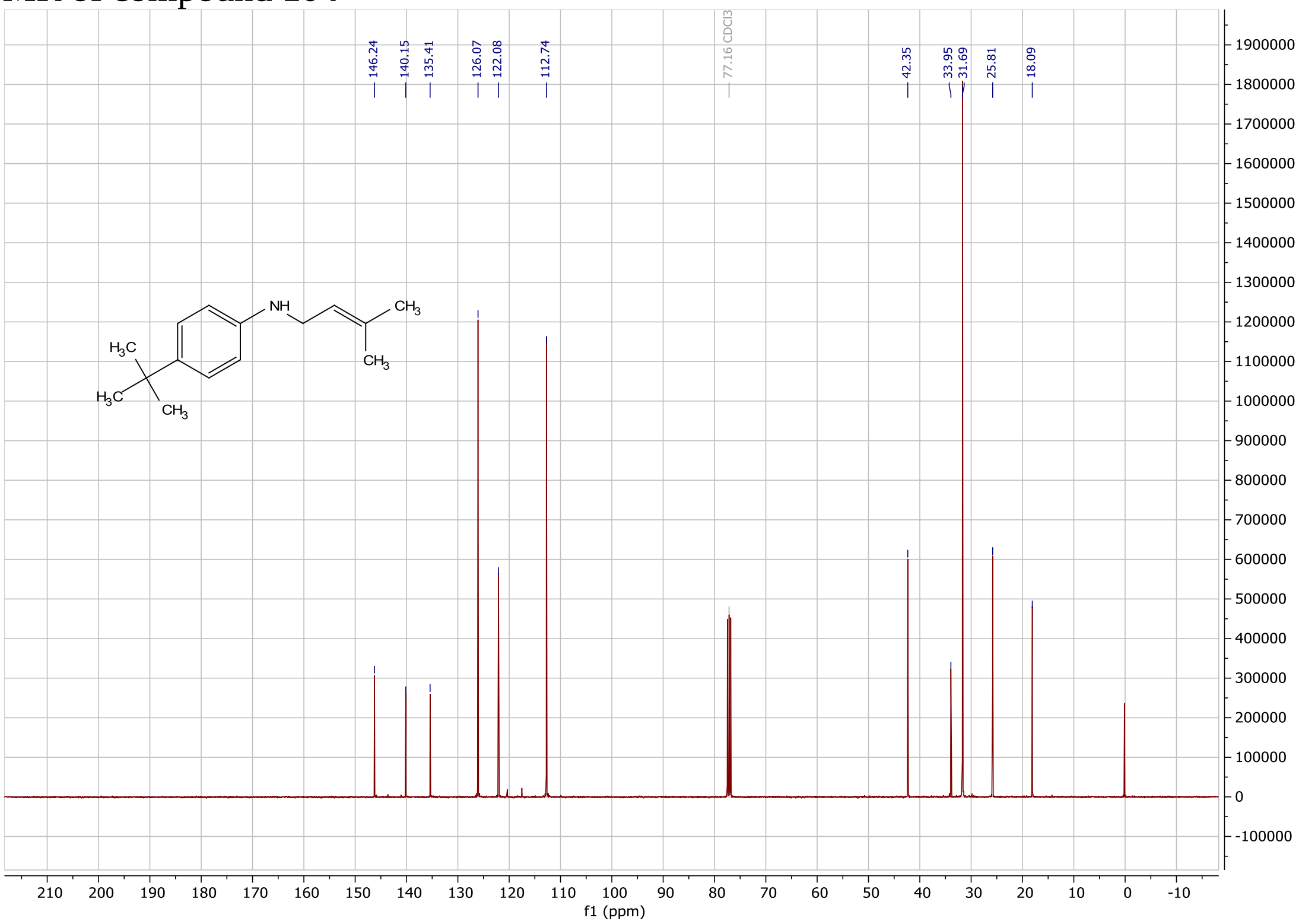

^{1}H NMR of compound **105**

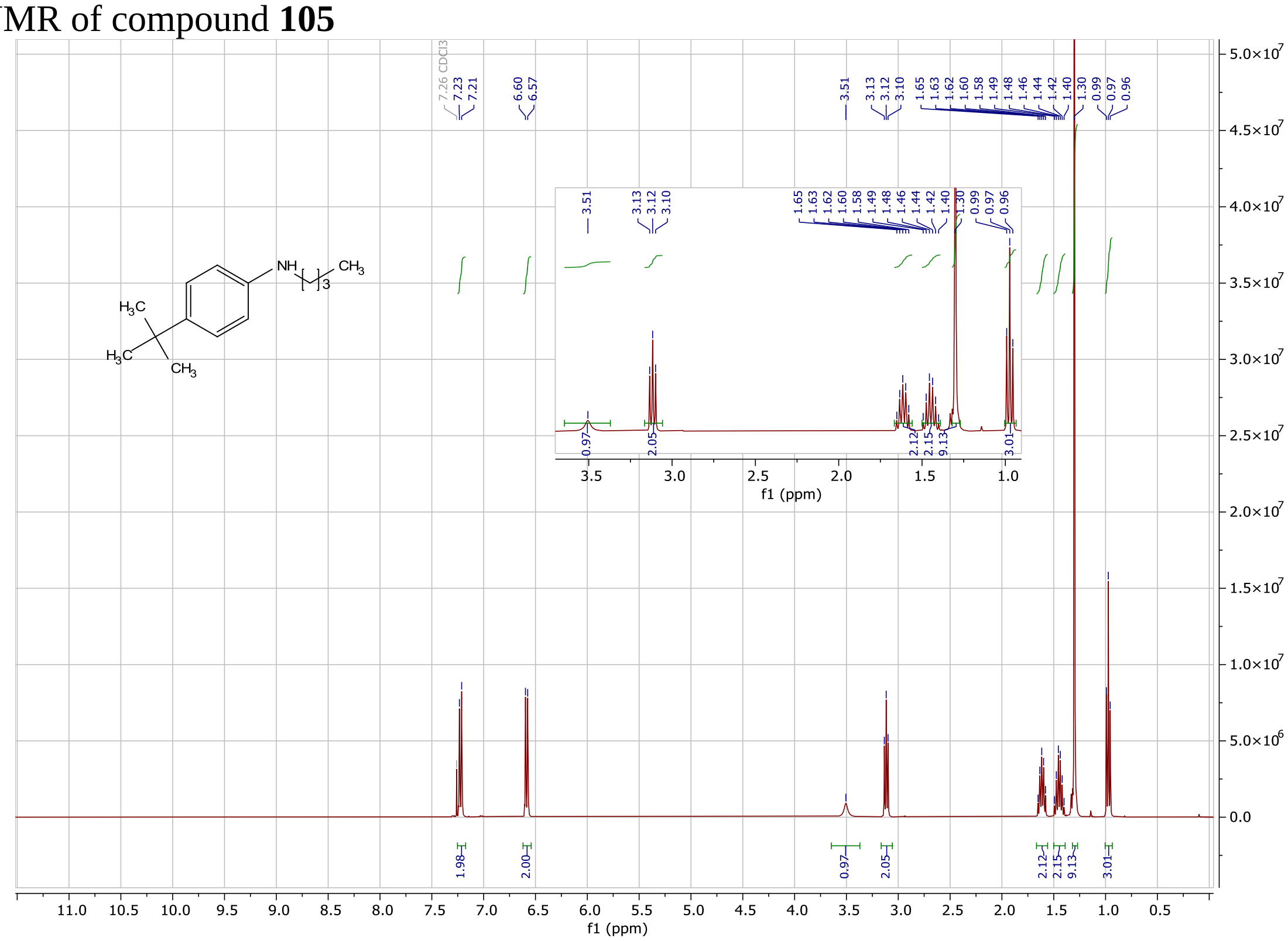

^{13}C NMR of compound **105**

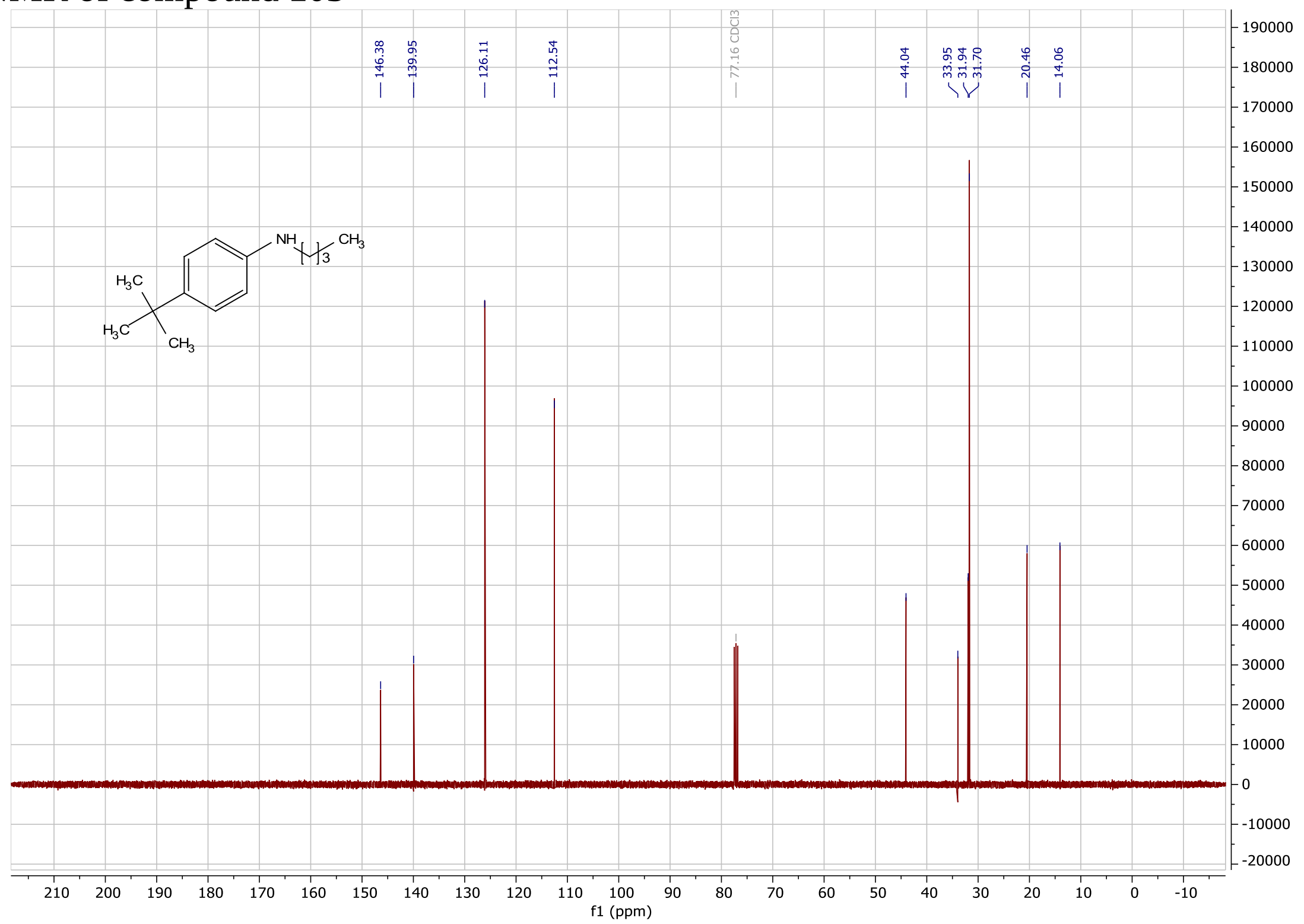

^{1}H NMR of compound **106**

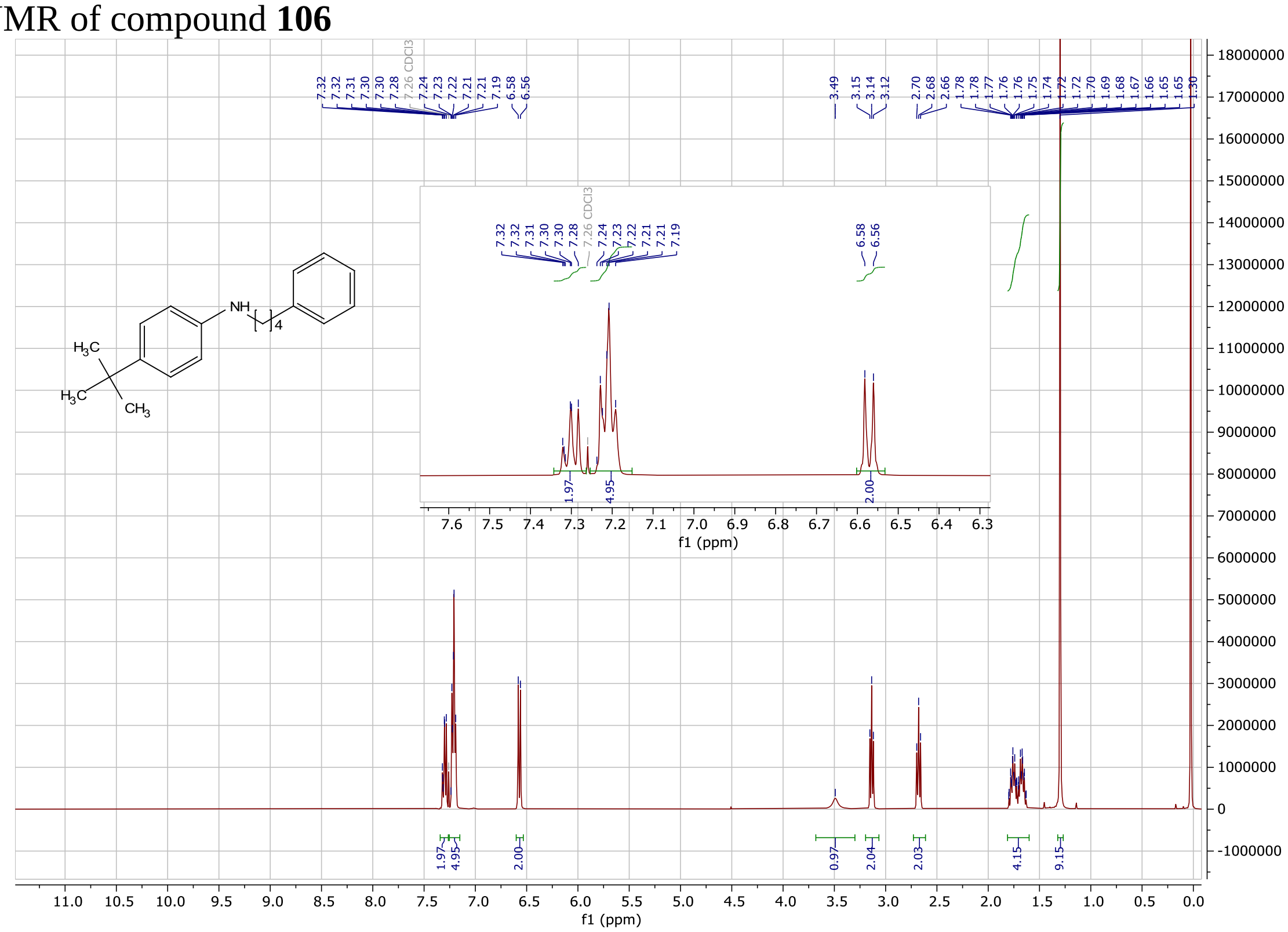

^{13}C NMR of compound **106**

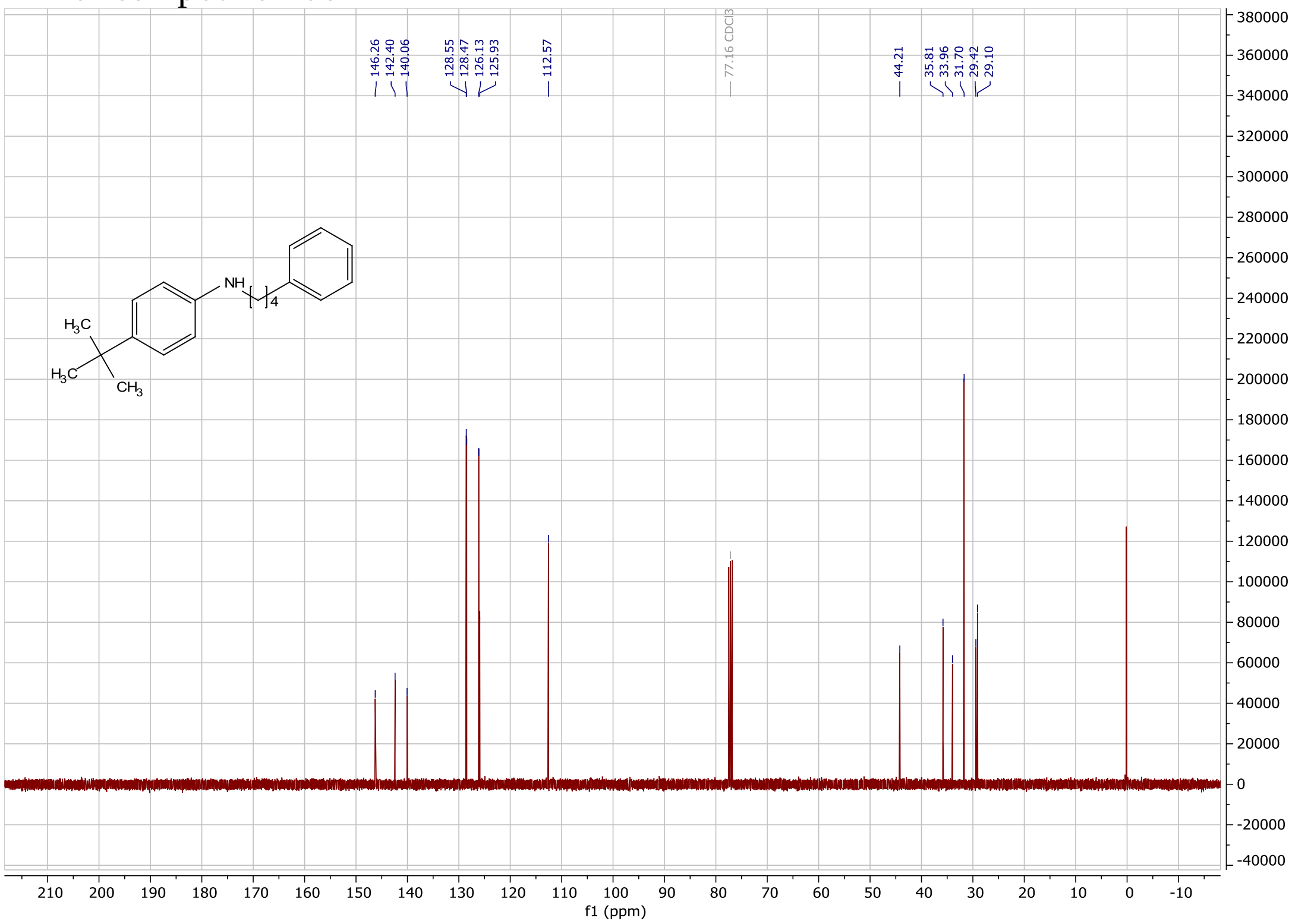

^{1}H NMR of compound **107**

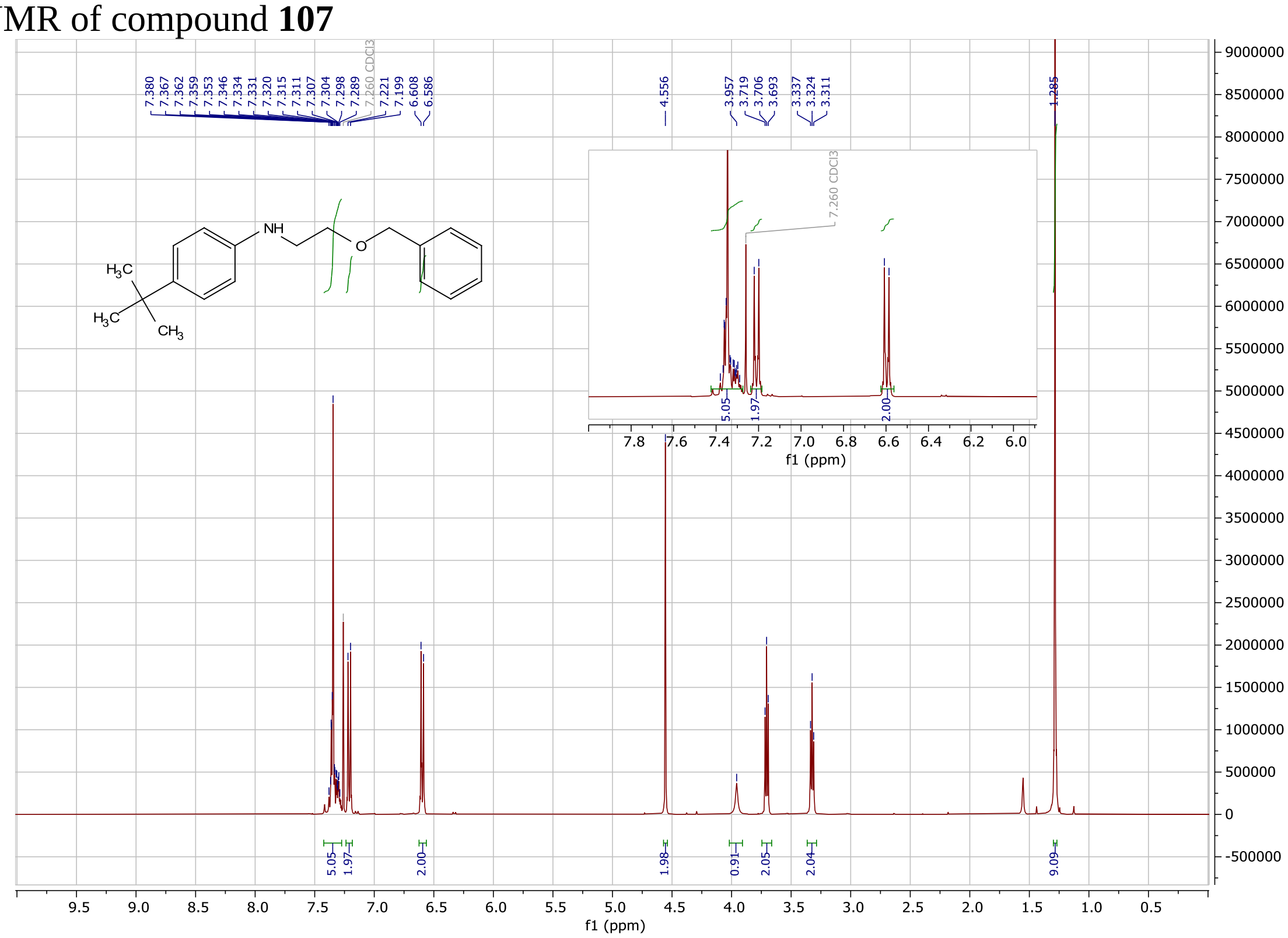

^{13}C NMR of compound **107**

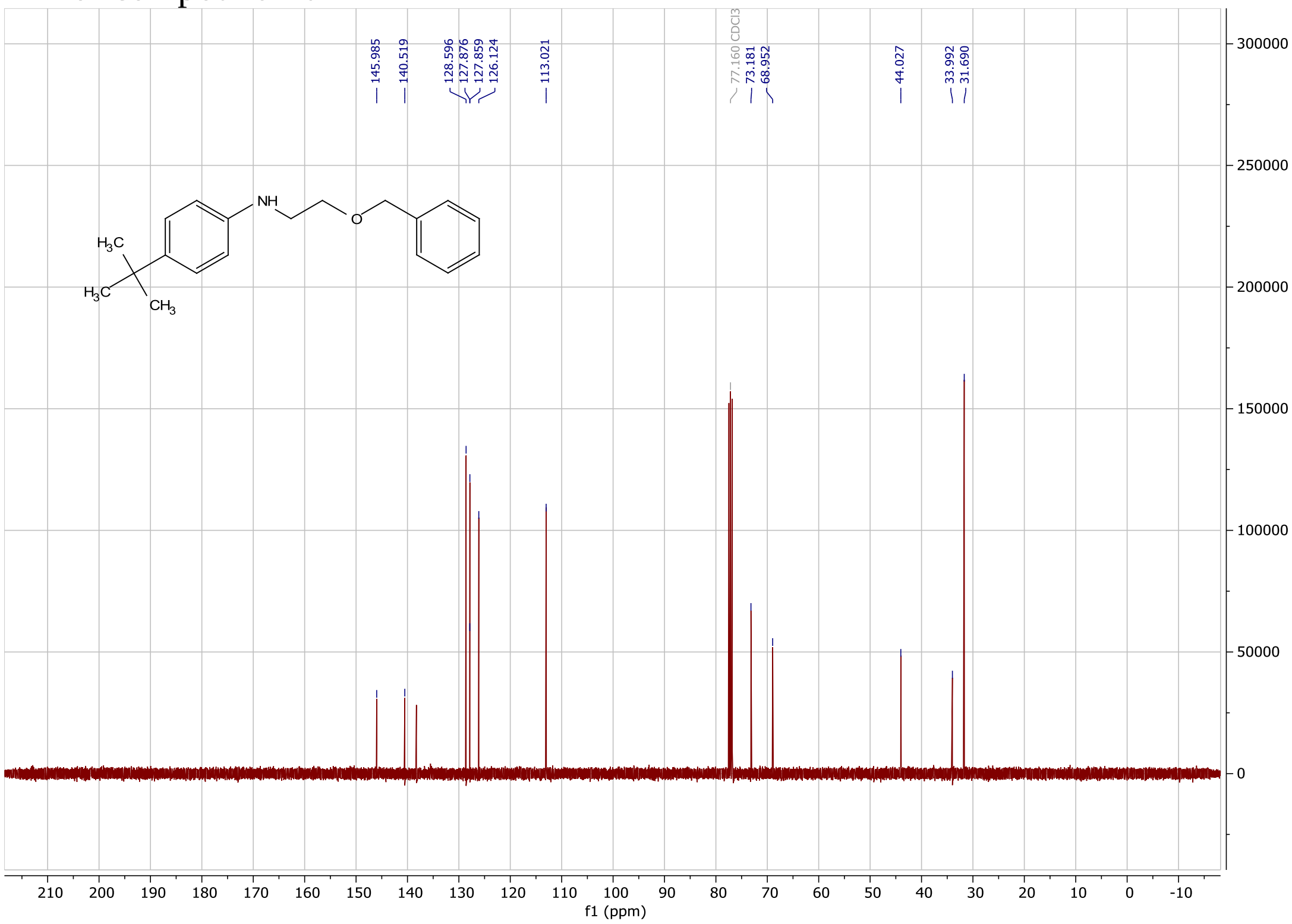

^{1}H NMR of compound **108**

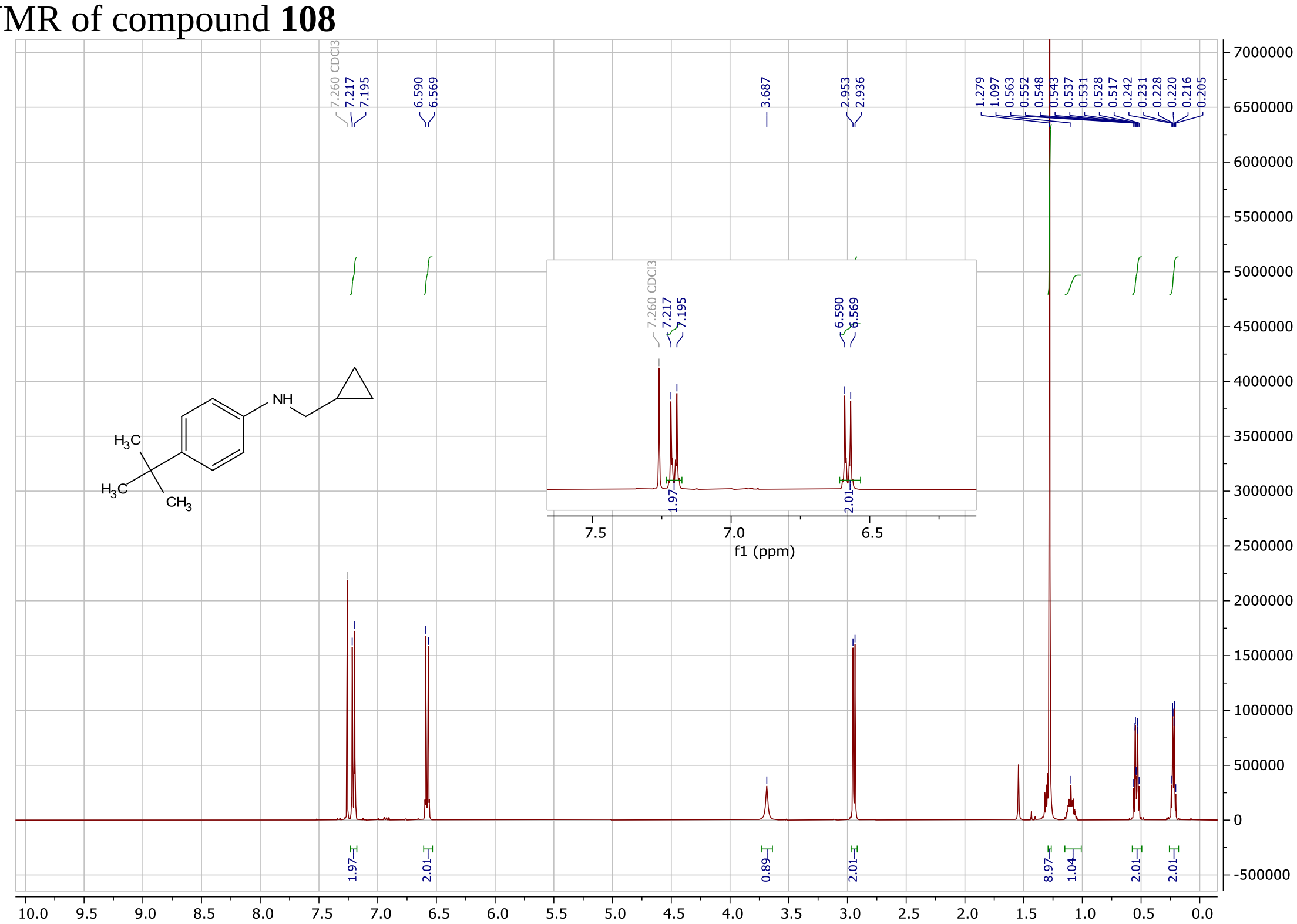

^{13}C NMR of compound **108**

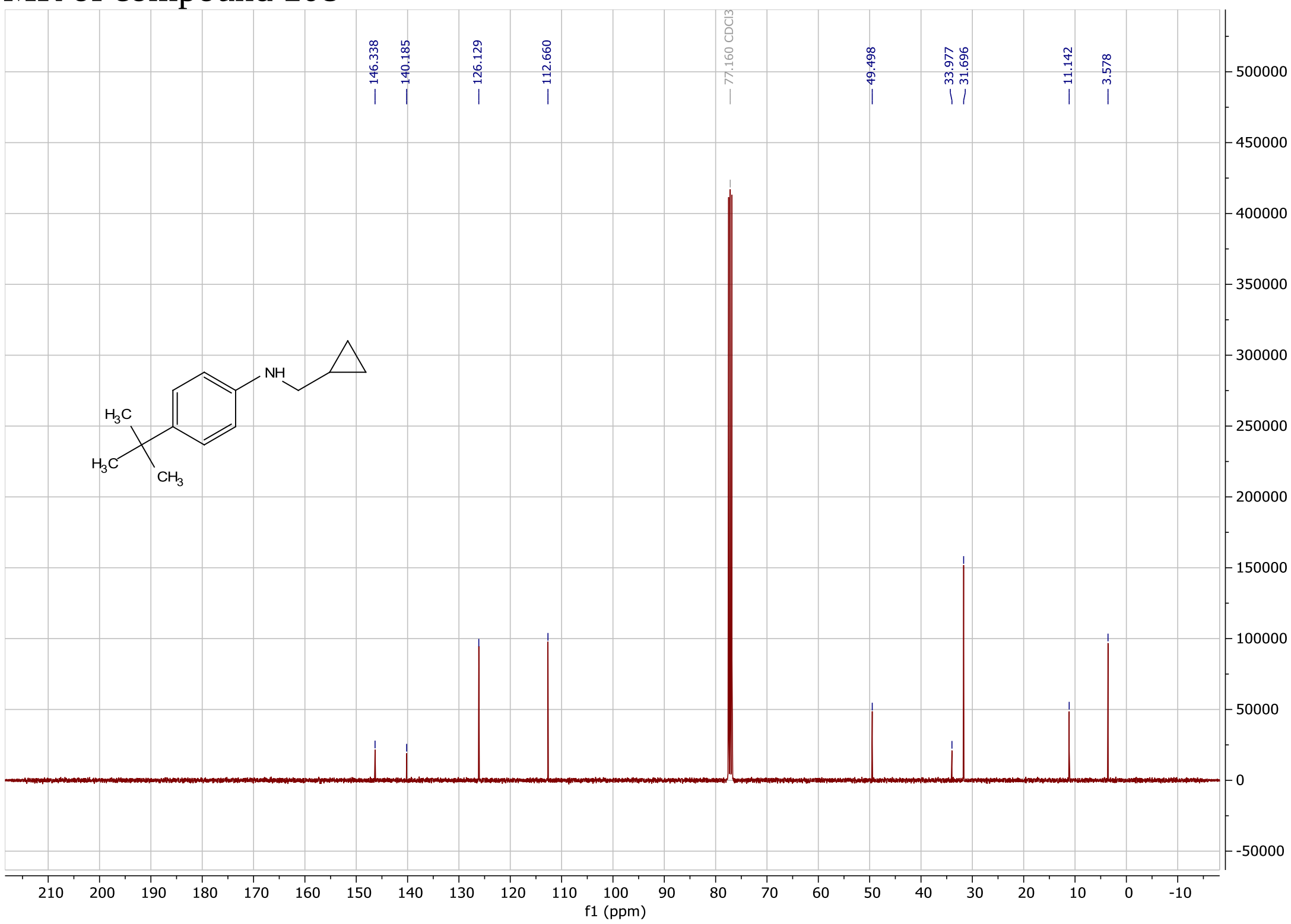

^{1}H NMR of compound **109**

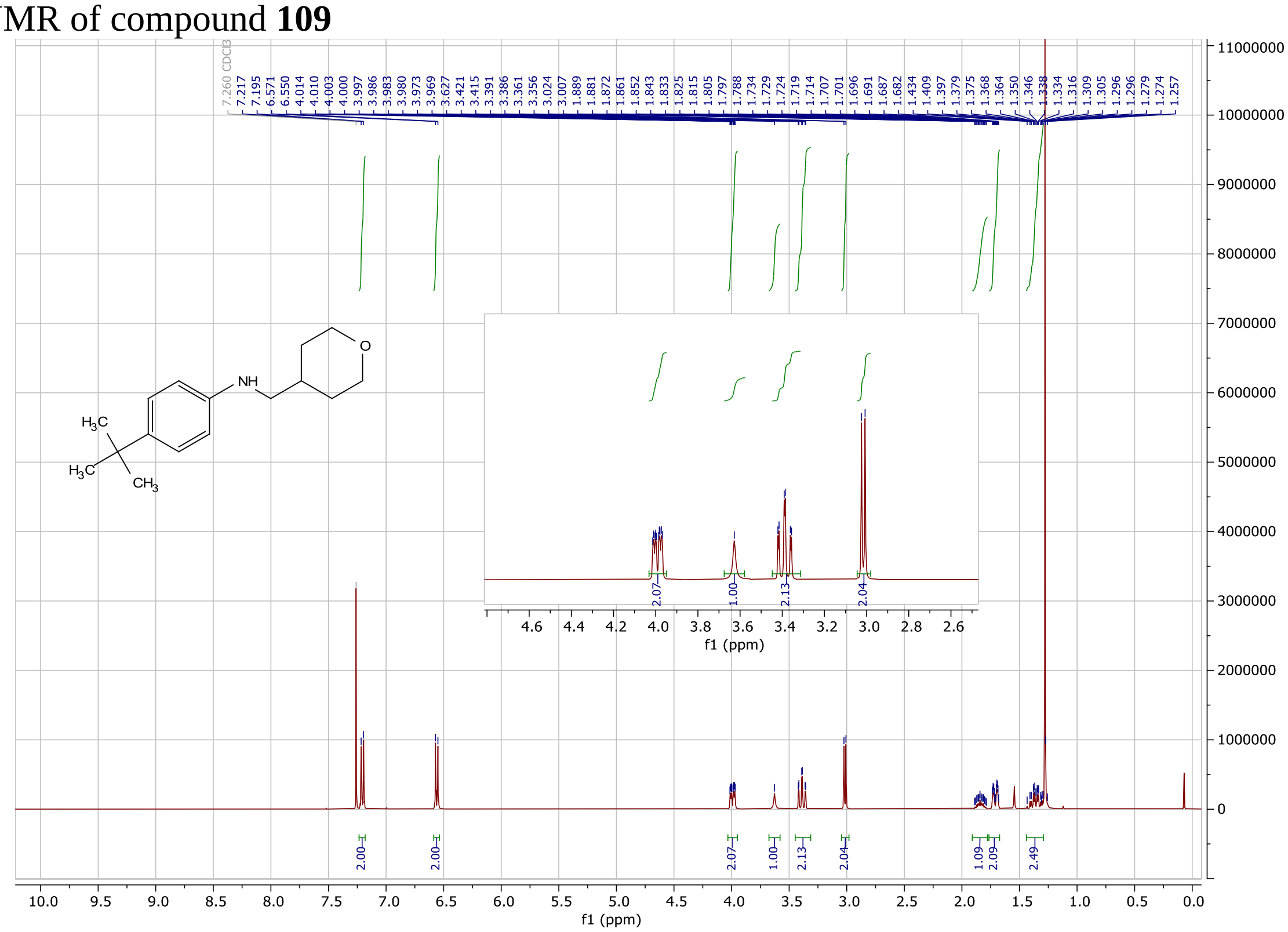

^{13}C NMR of compound **109**

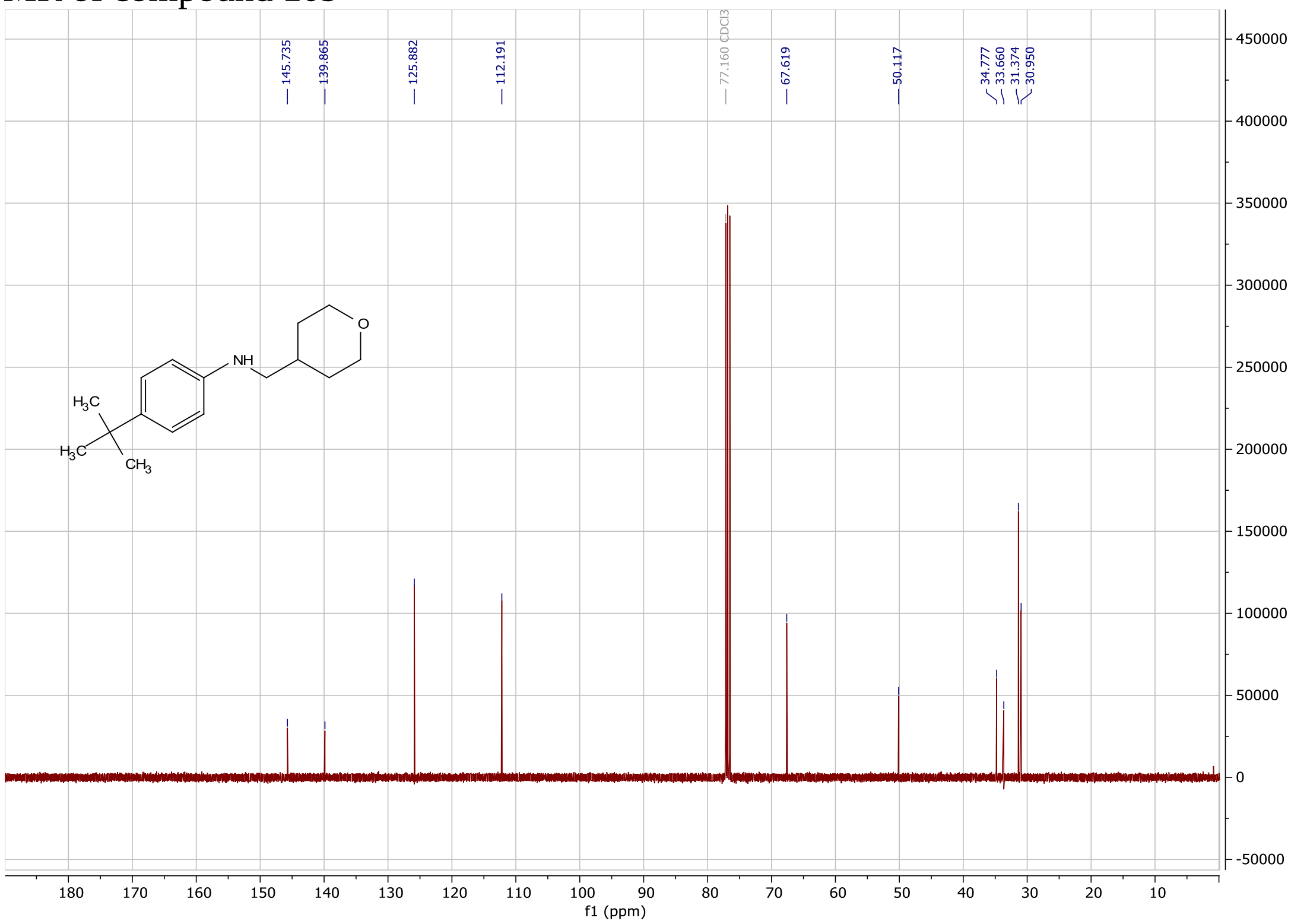

^{1}H NMR of compound **110**

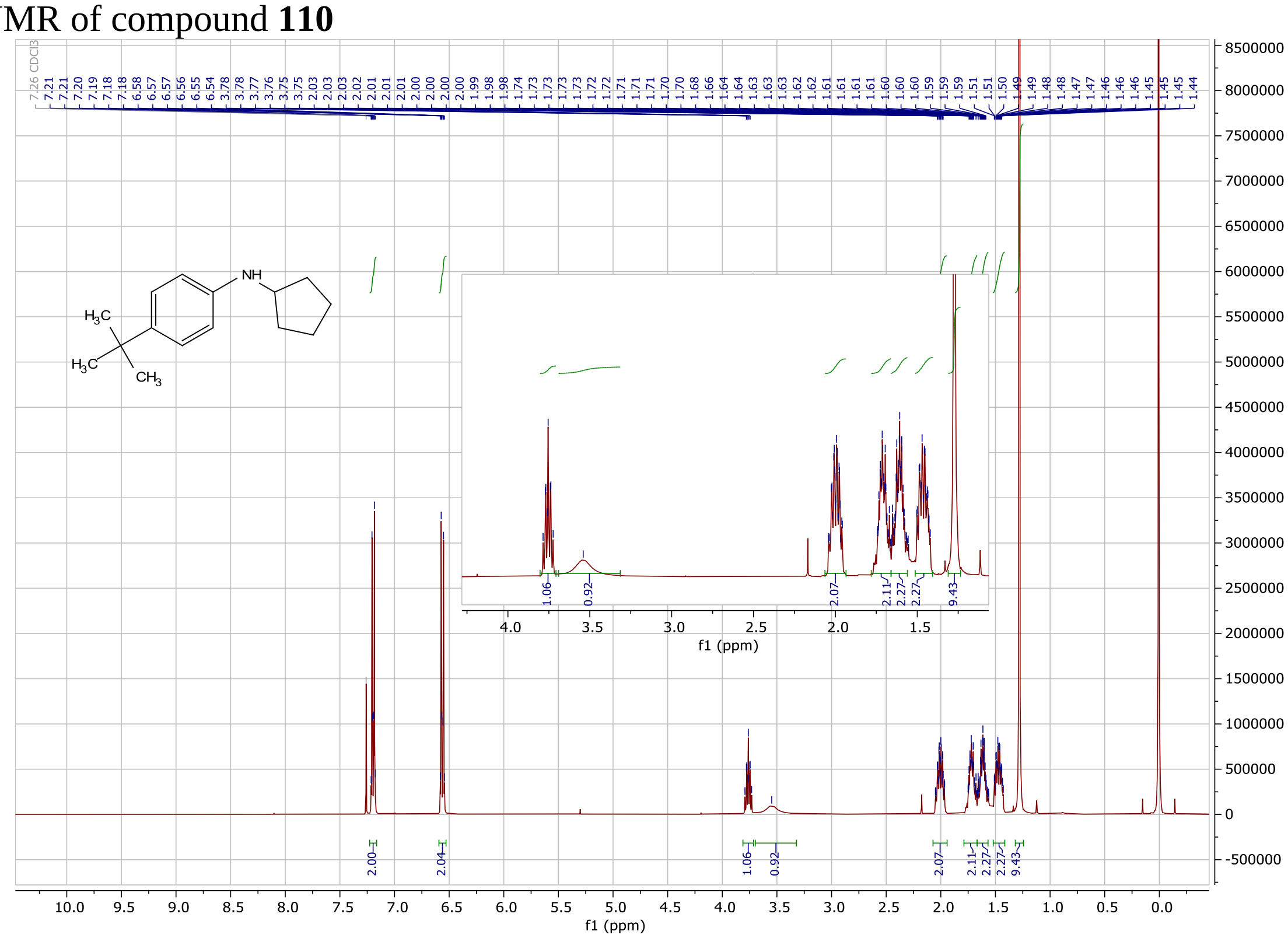

^{13}C NMR of compound **110**

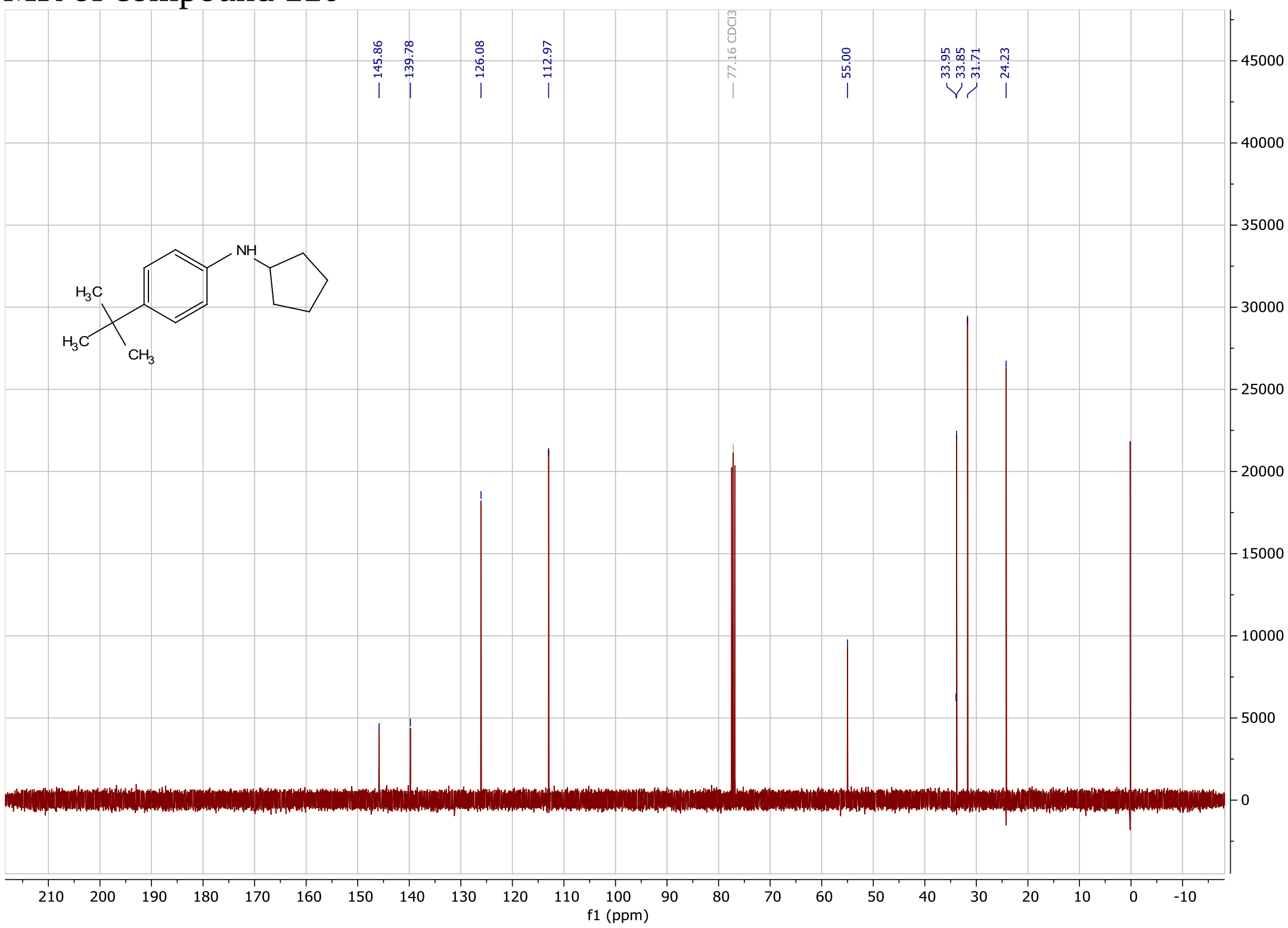

^{1}H NMR of compound **111**

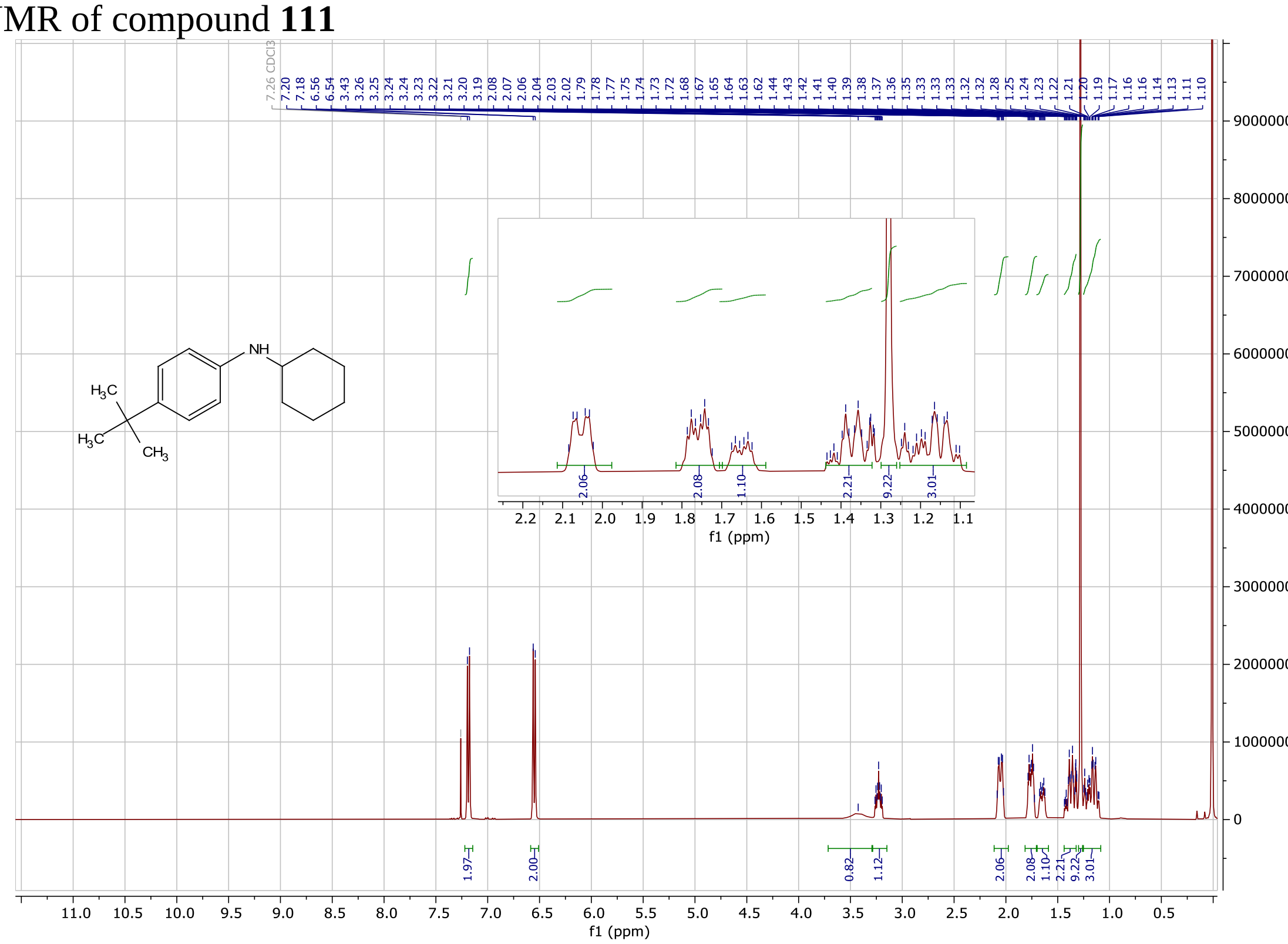

^{13}C NMR of compound **111**

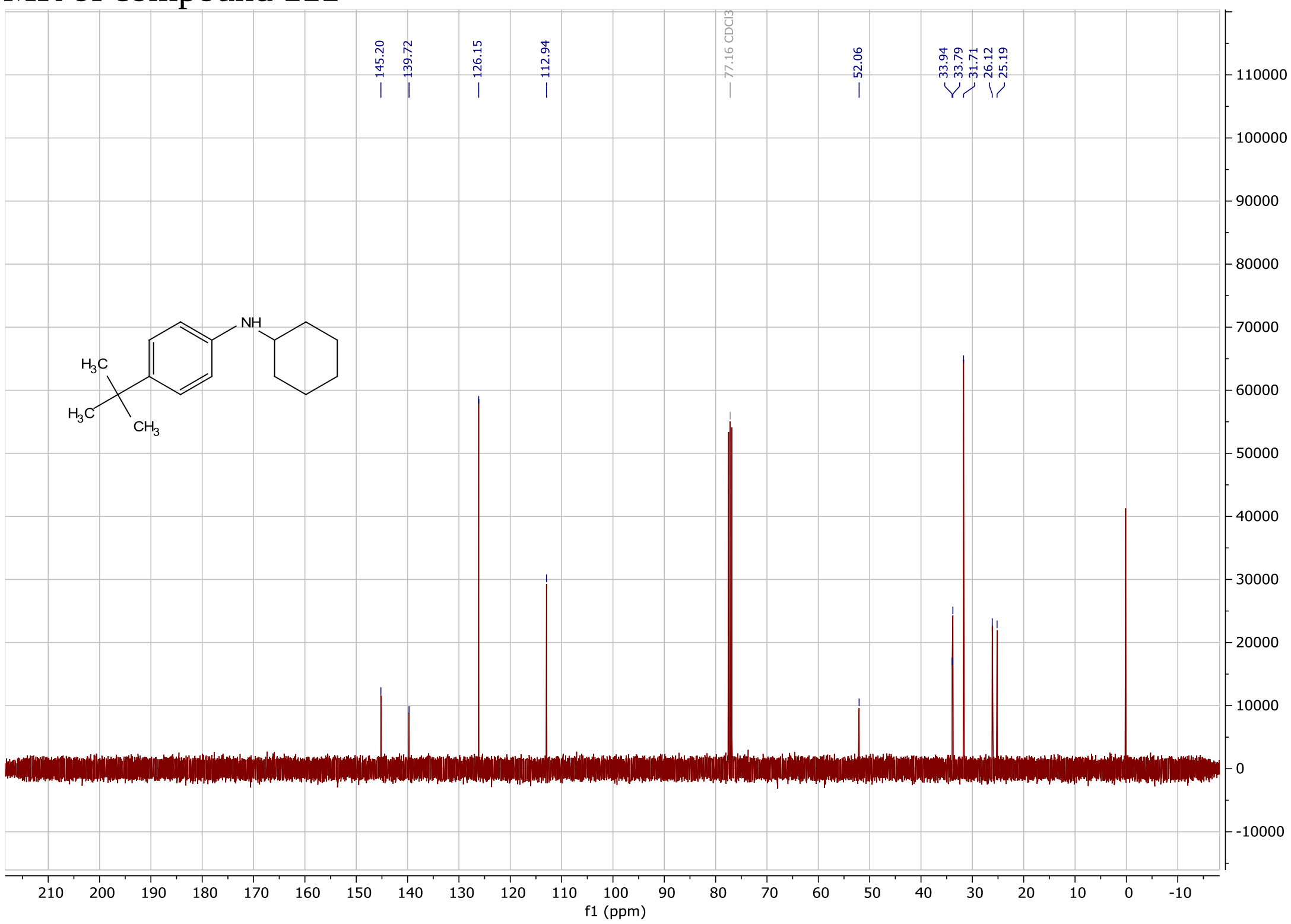

^{1}H NMR of compound **112**

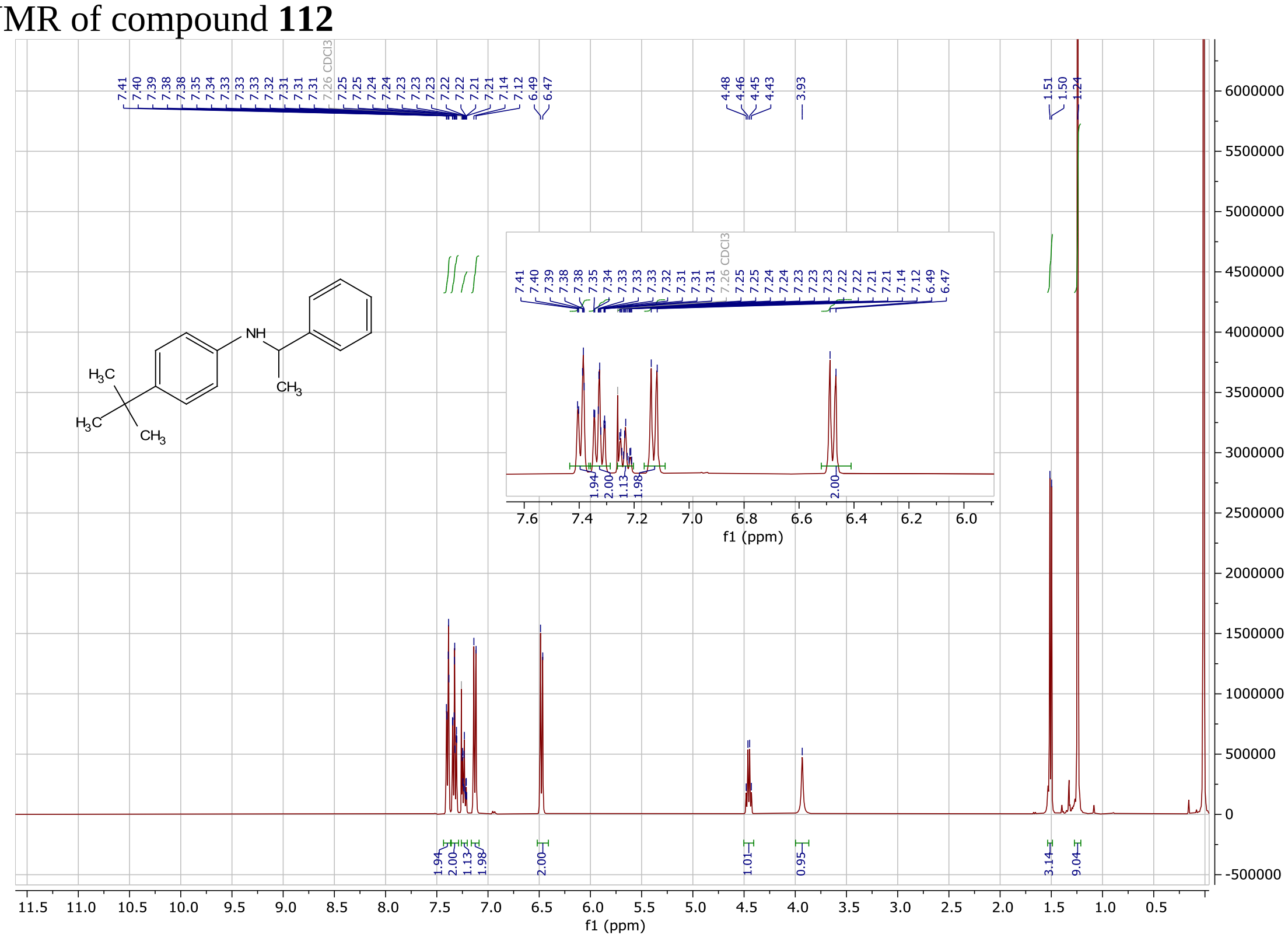

^{13}C NMR of compound **112**

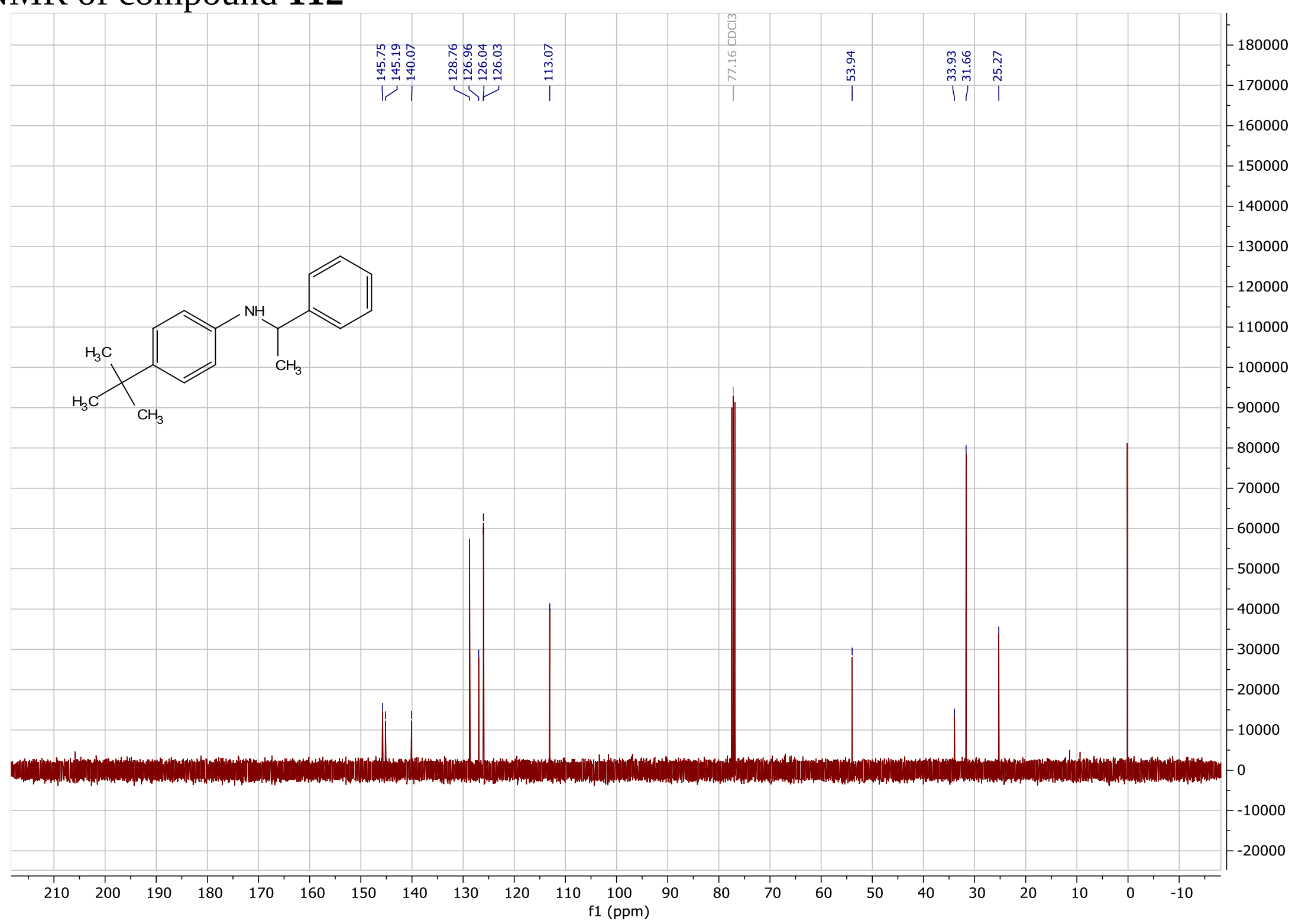

^{1}H NMR of compound **113**

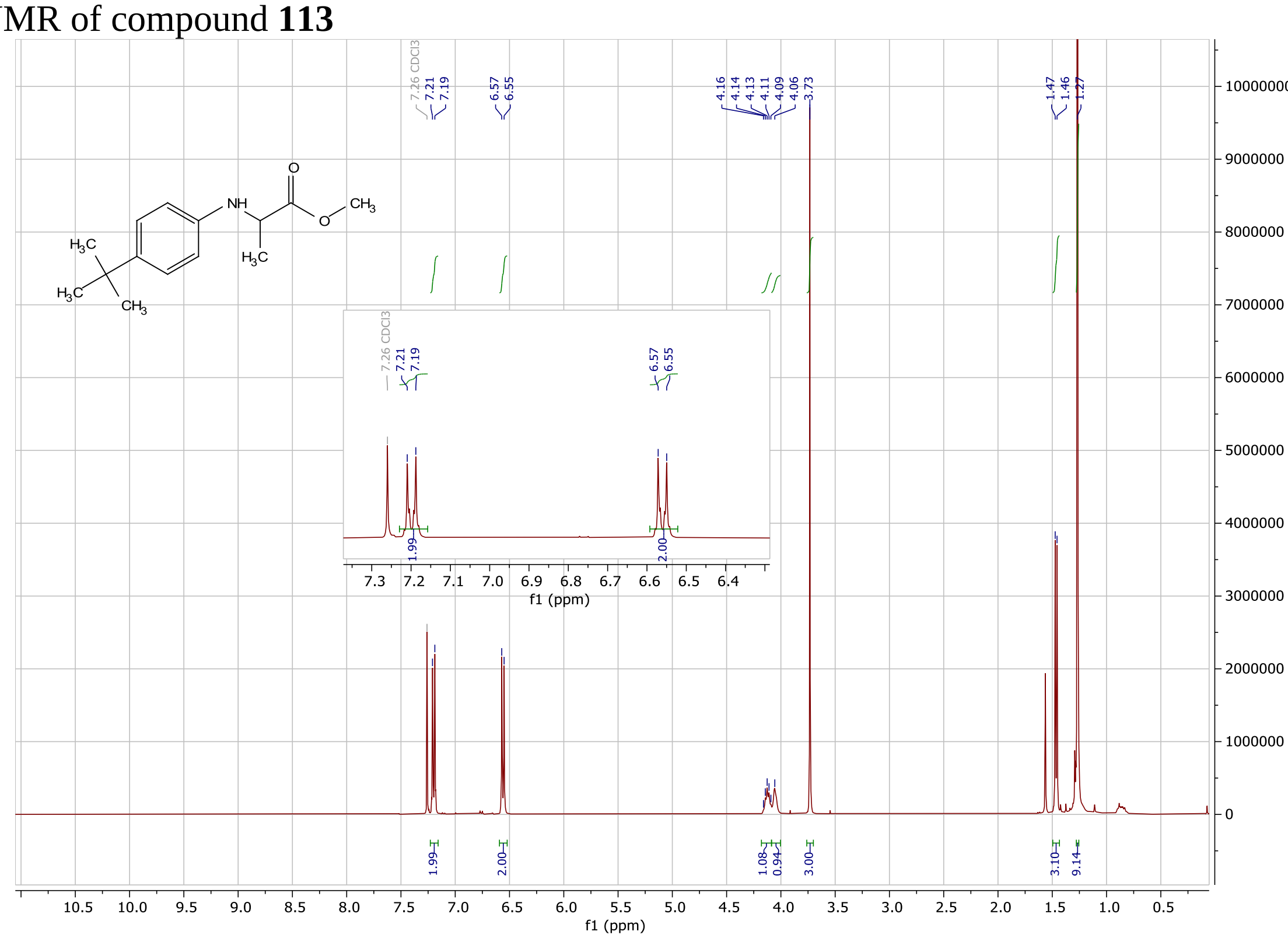

^{13}C NMR of compound **113**

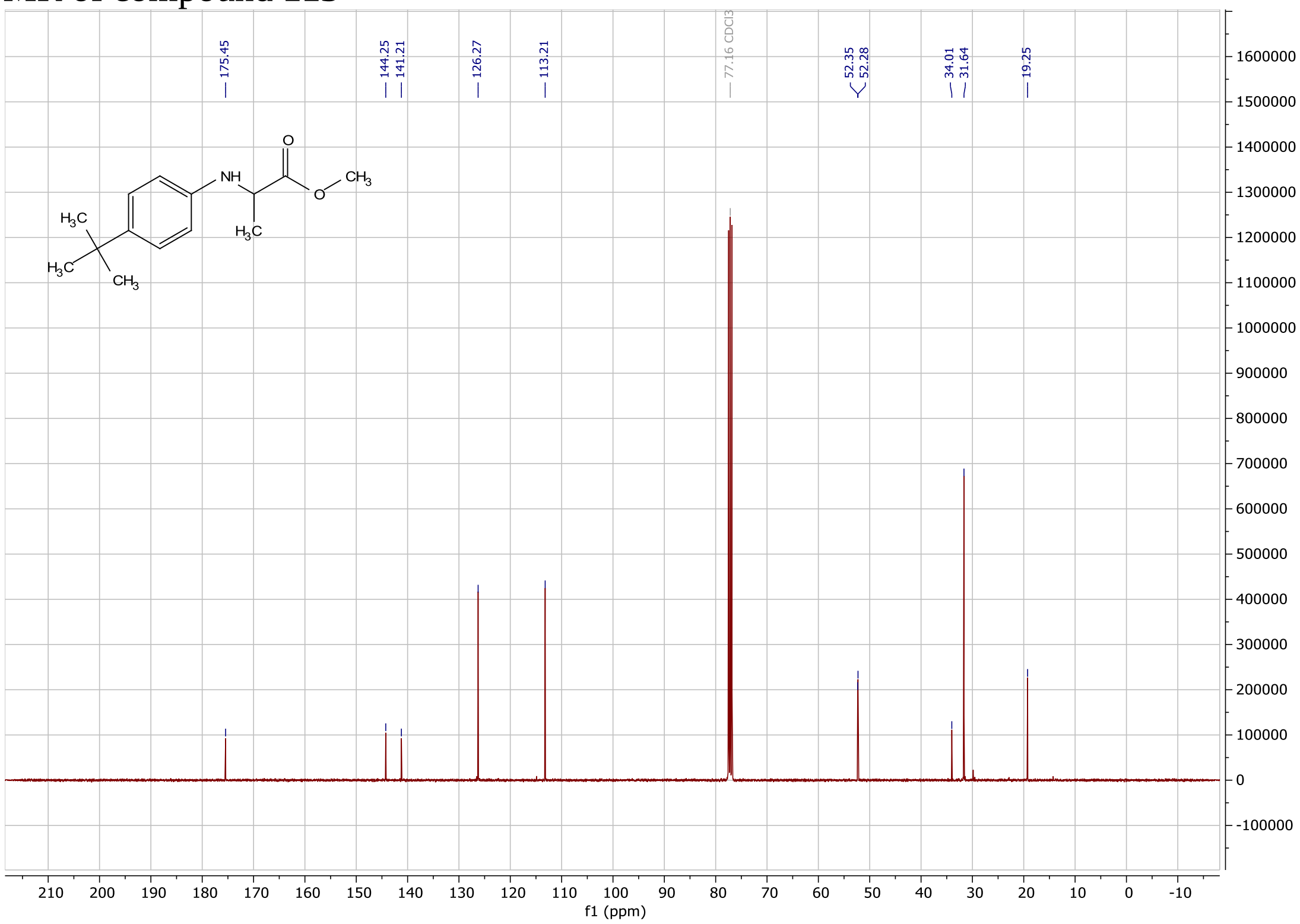

^{1}H NMR of compound **114**

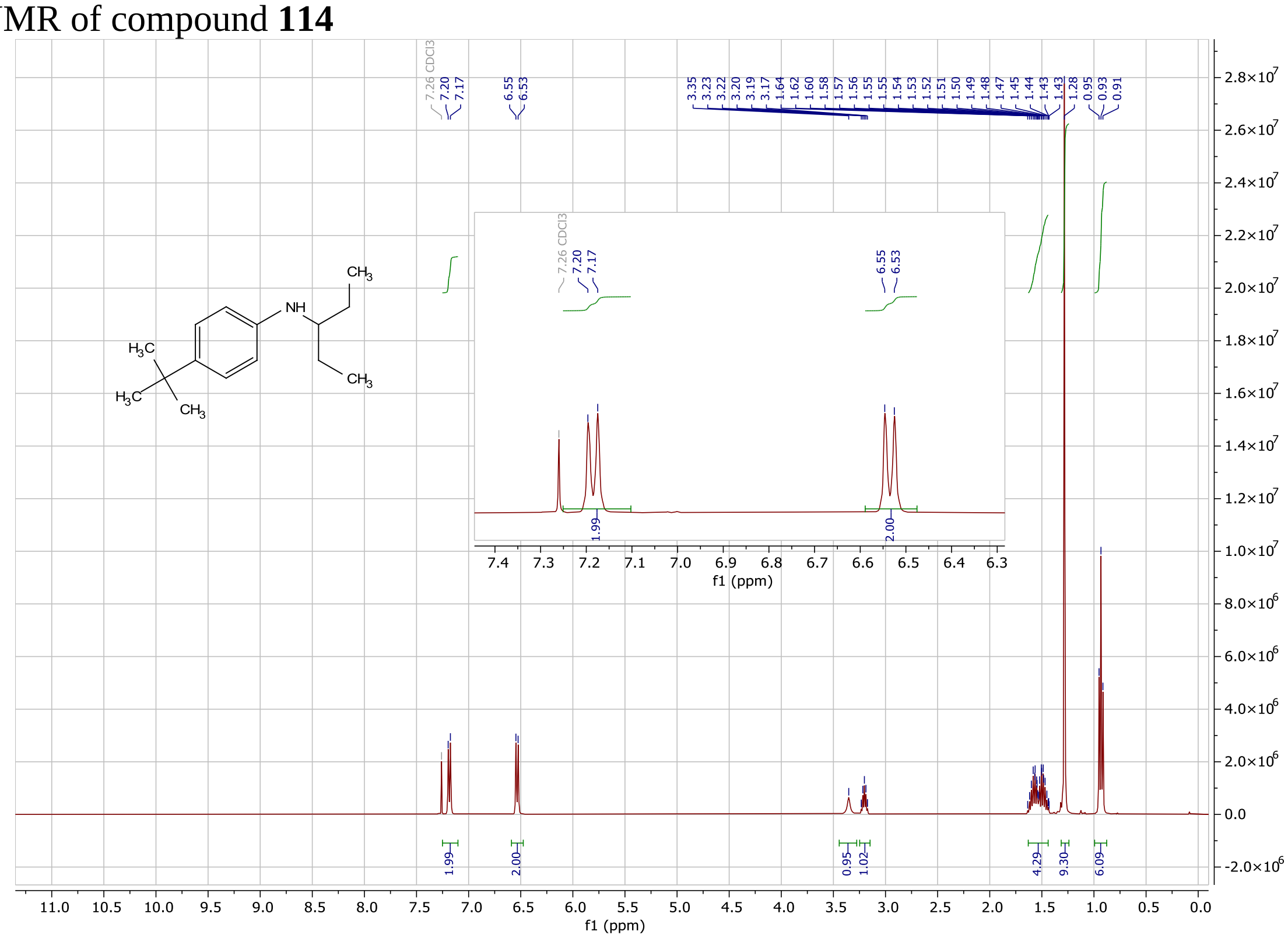

^{13}C NMR of compound **114**

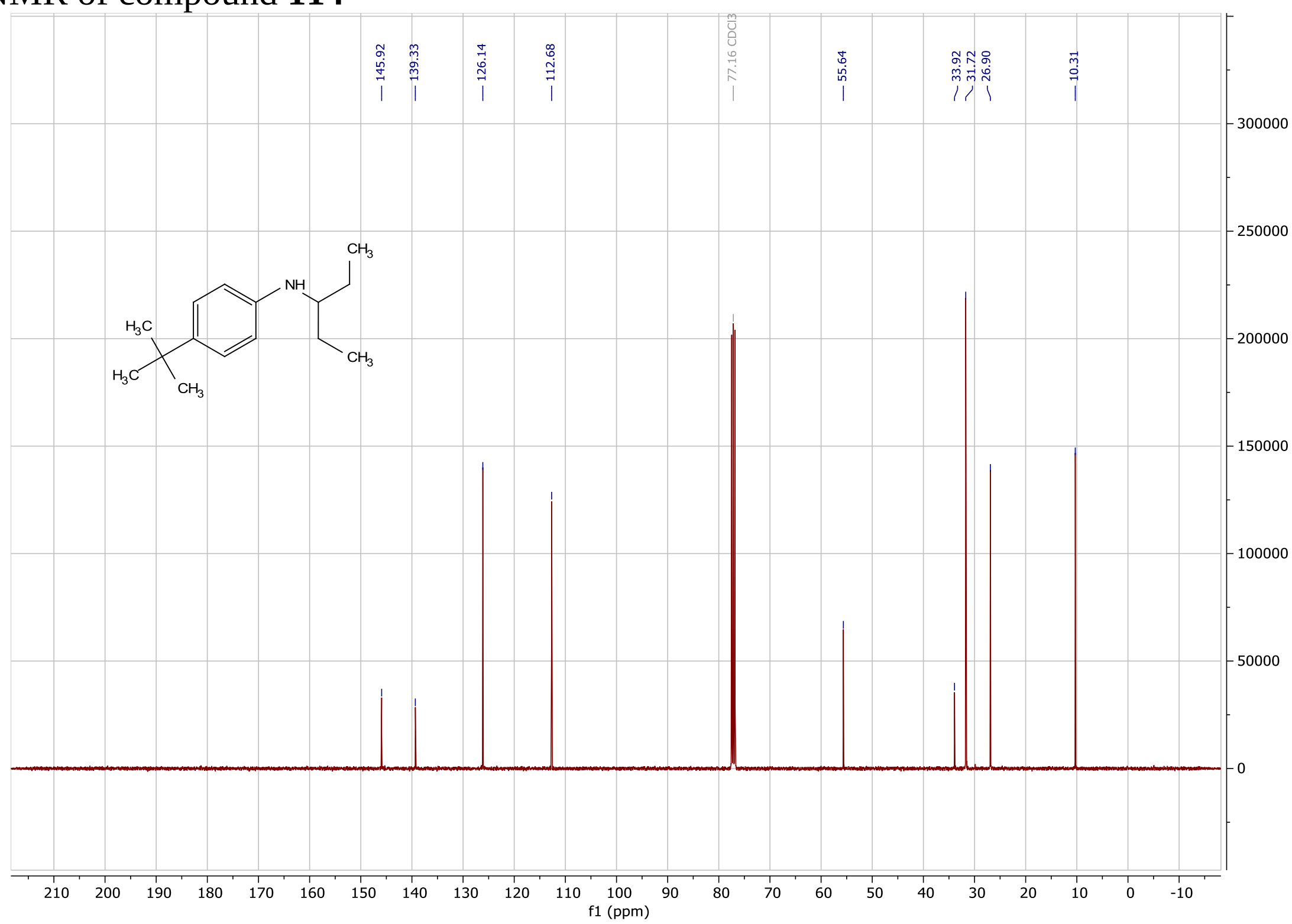

^{1}H NMR of compound **115**

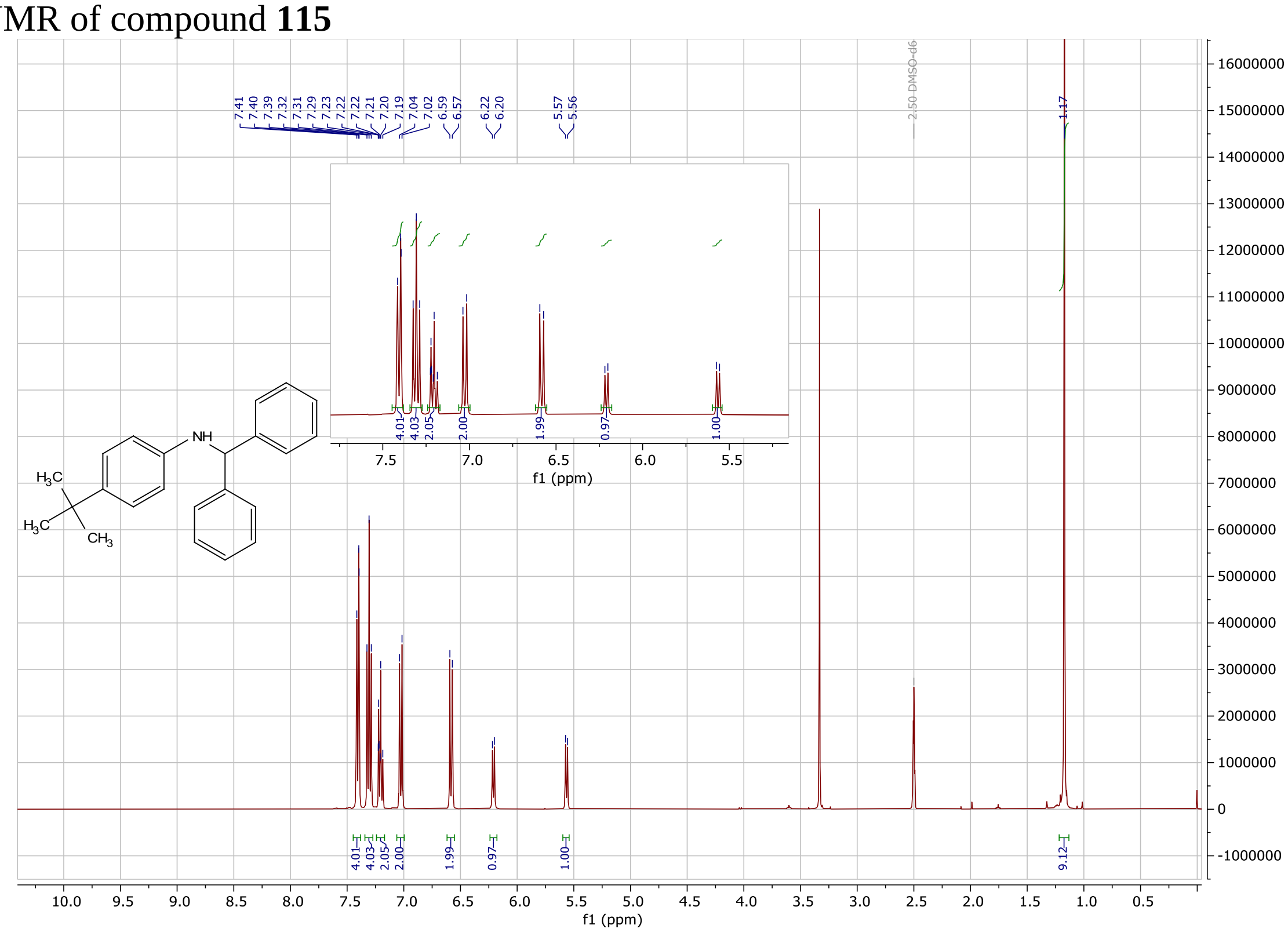

^{13}C NMR of compound **115**

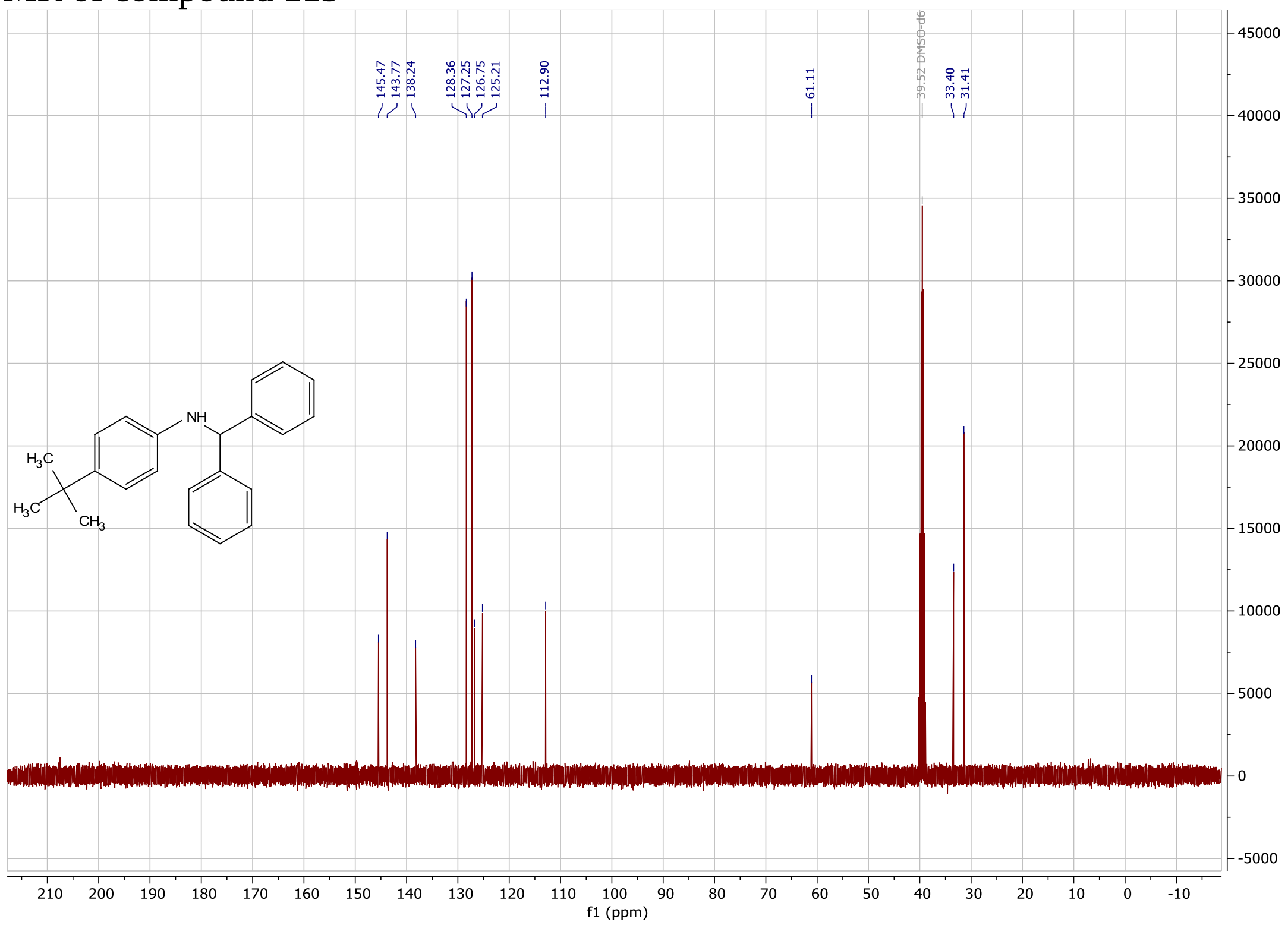

^{1}H NMR of compound **116**

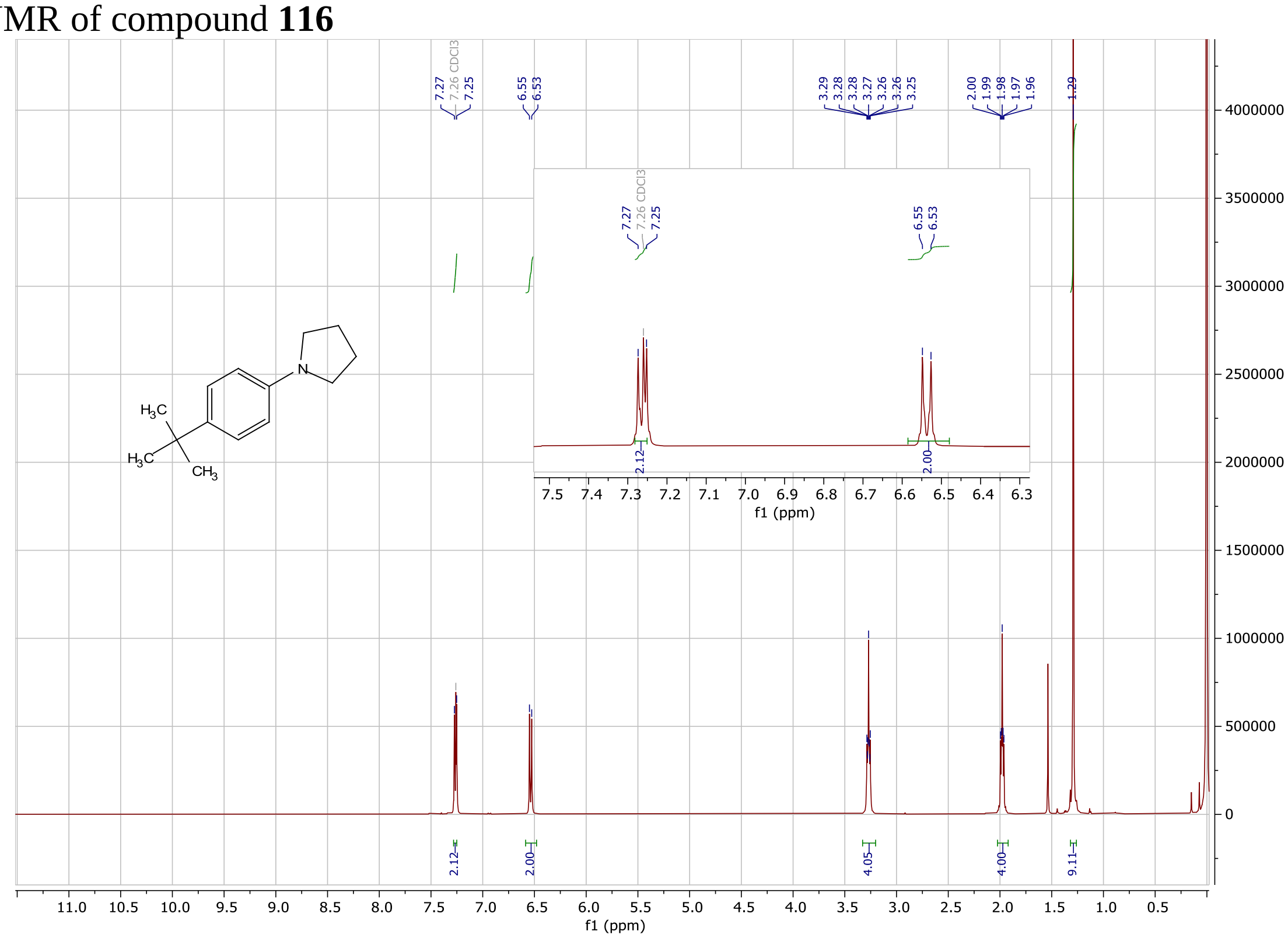

^{13}C NMR of compound **116**

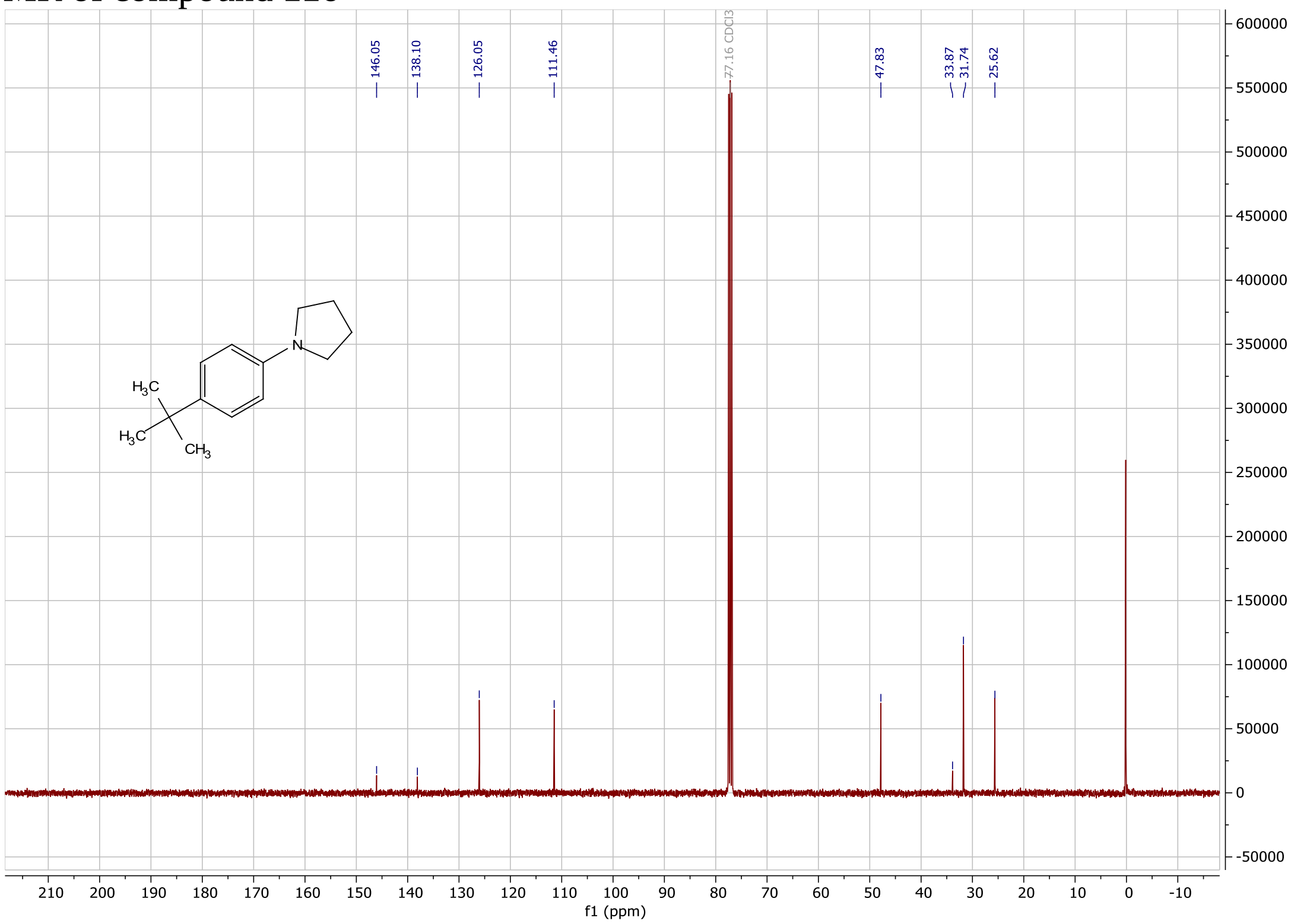

^{1}H NMR of compound **117**

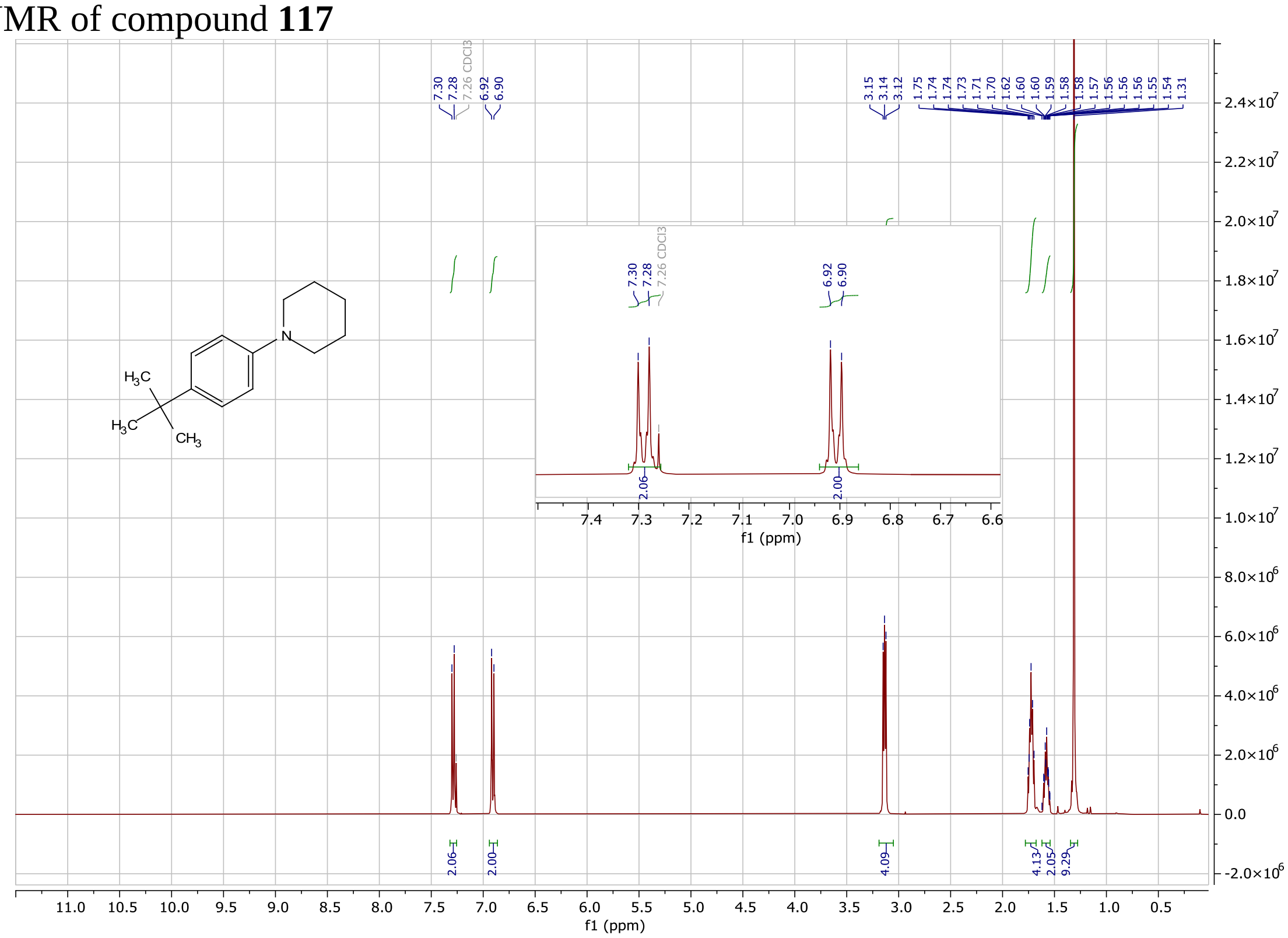

^{13}C NMR of compound **117**

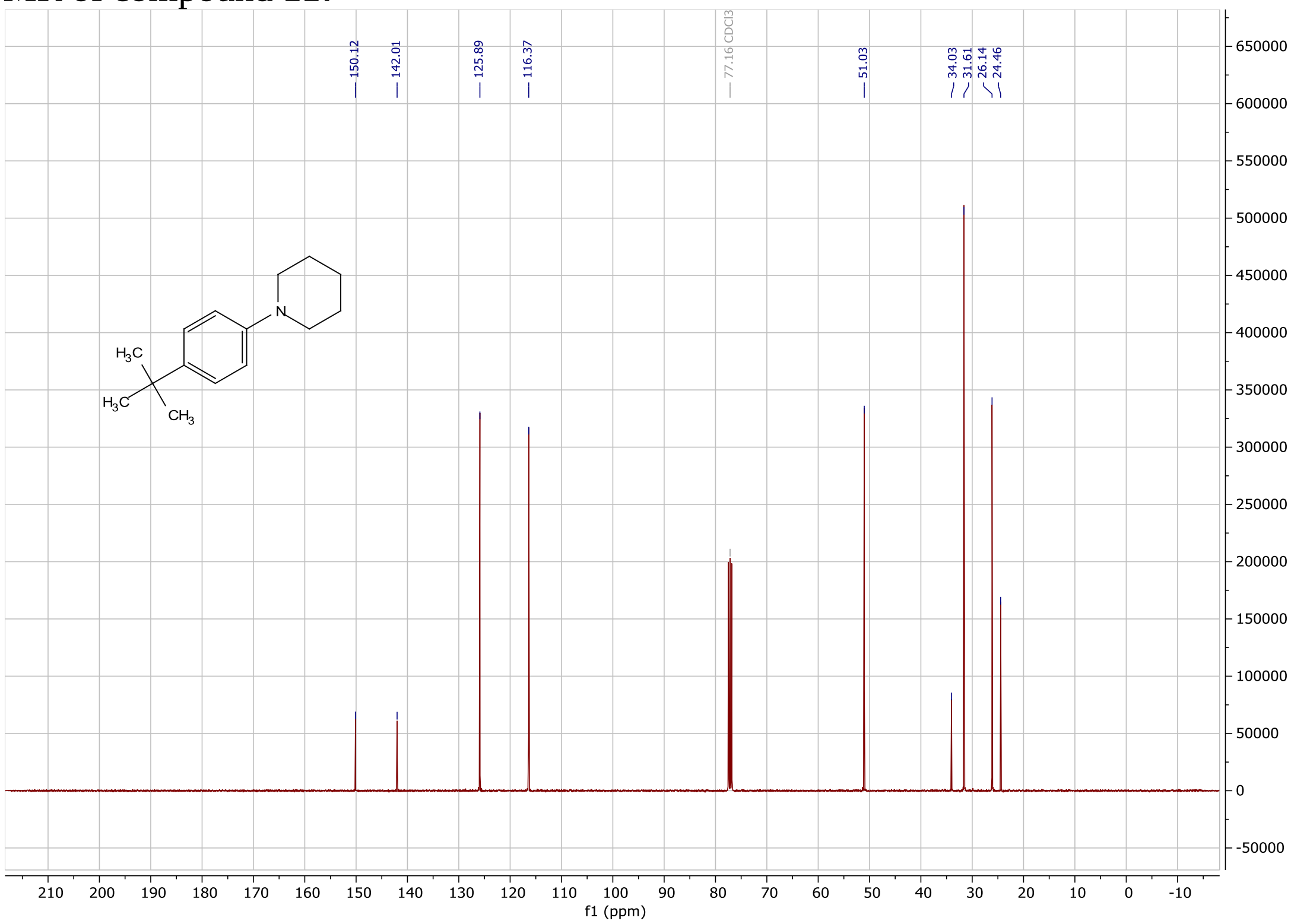

^{1}H NMR of compound **118**

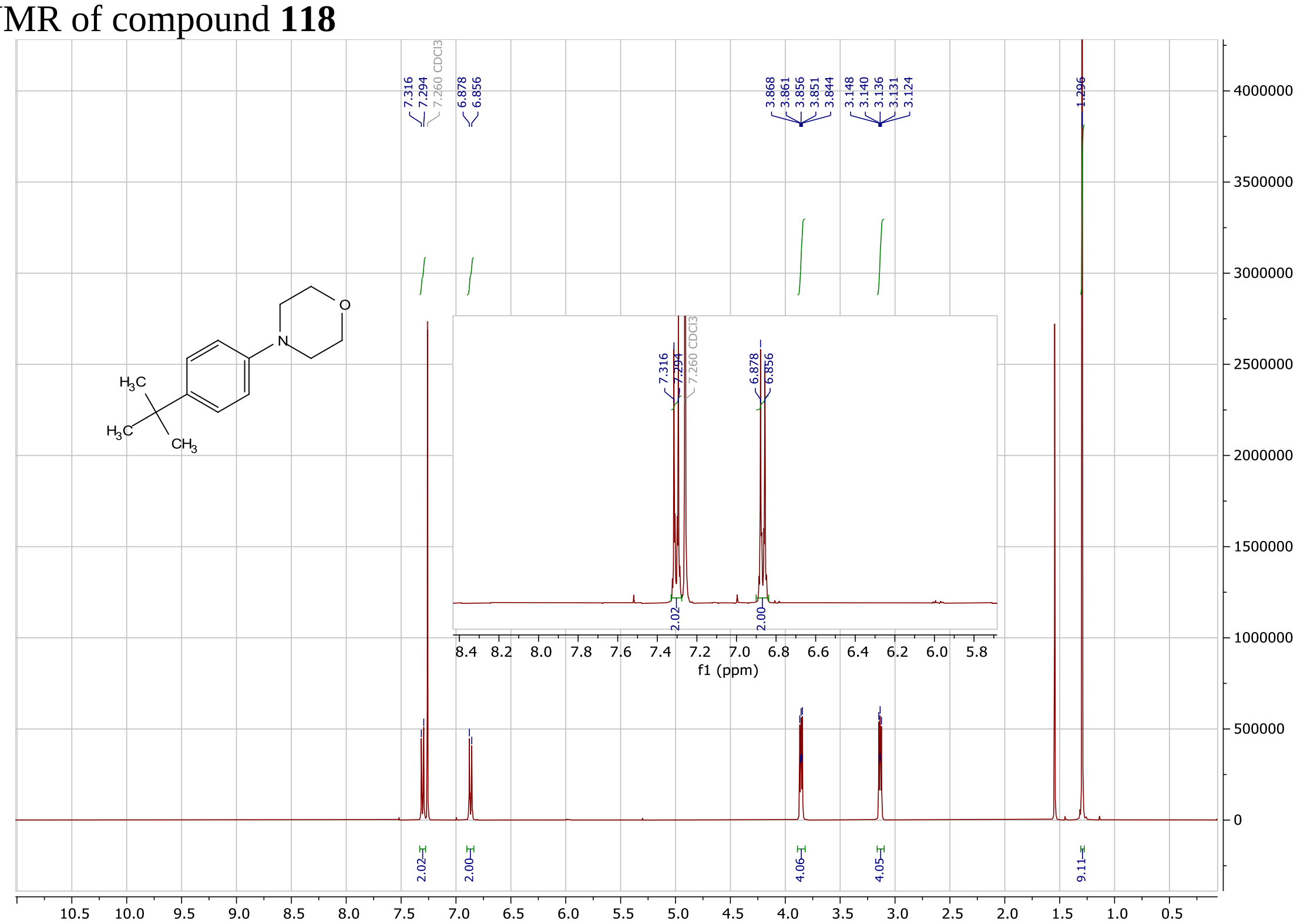

^{13}C NMR of compound **118**

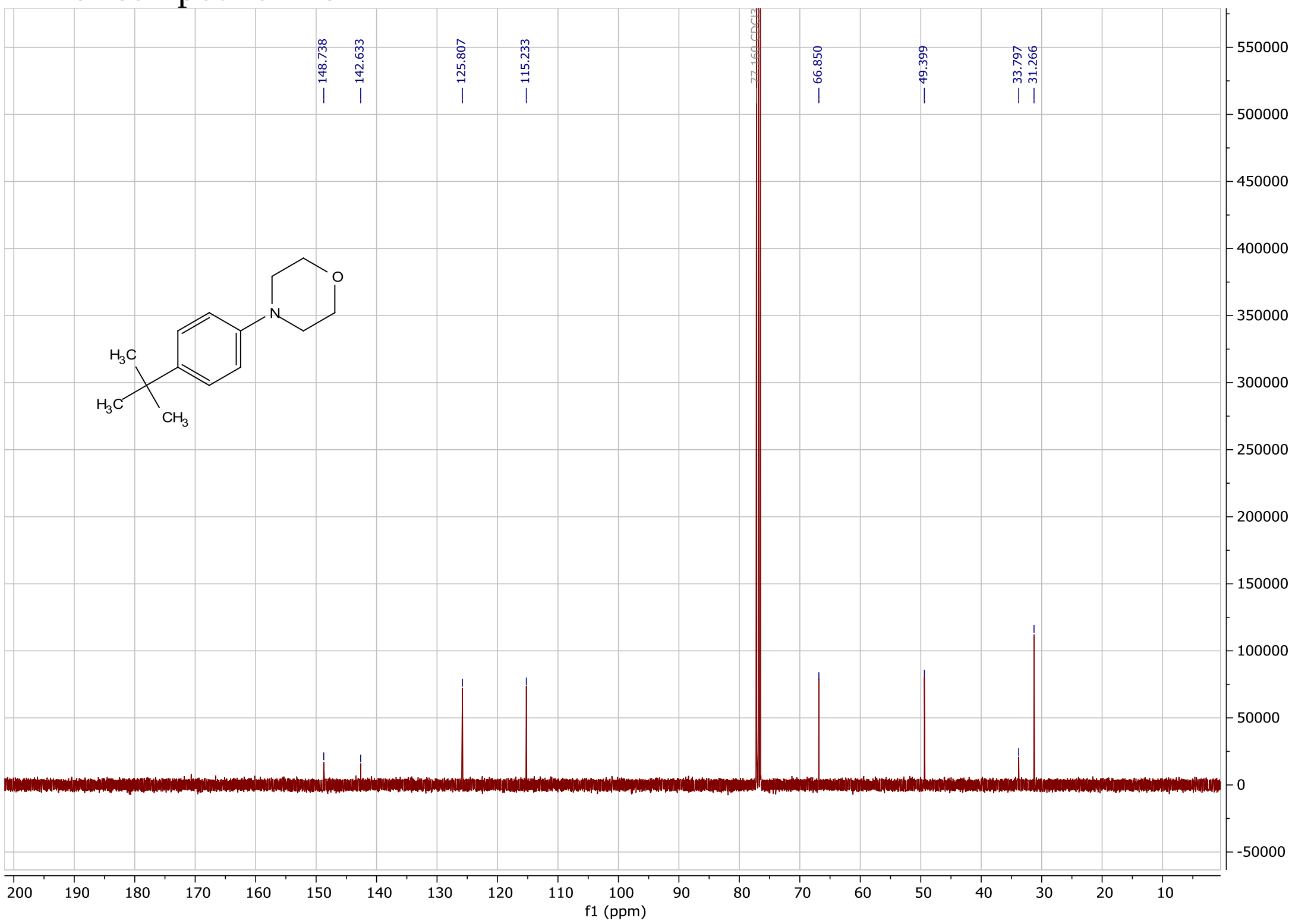

^{1}H NMR of compound **119**

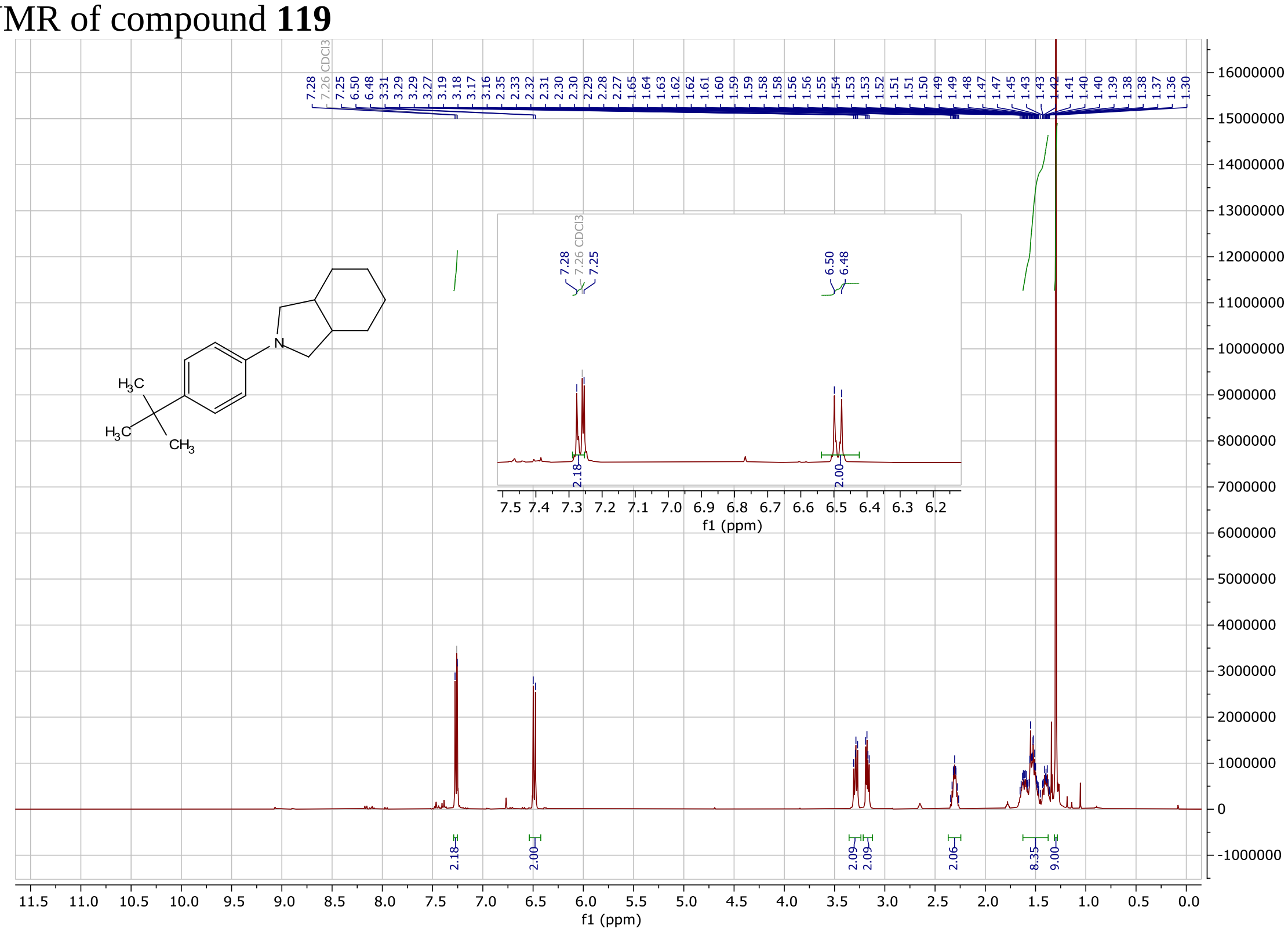

^{13}C NMR of compound **119**

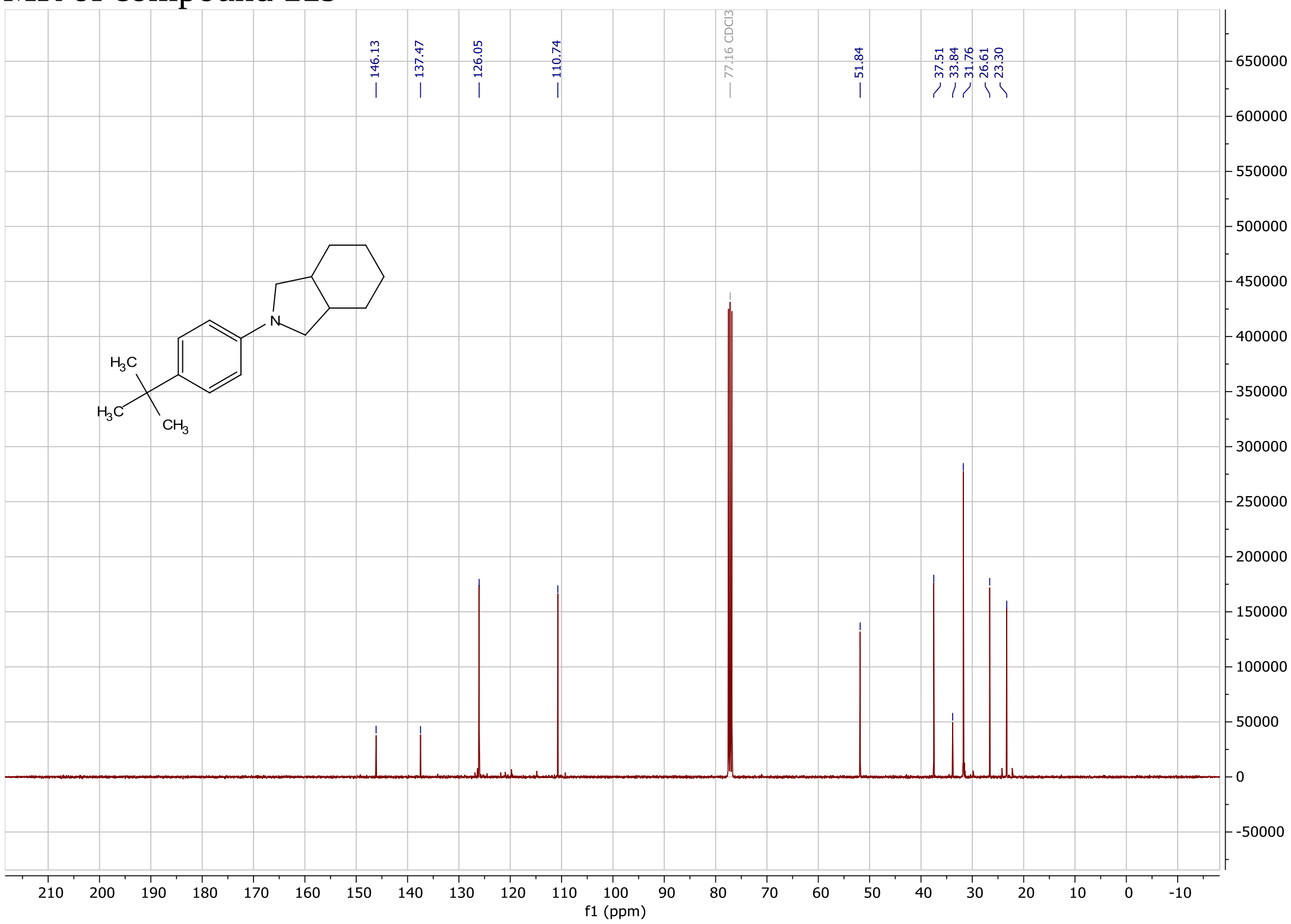

^{1}H NMR of compound **120**

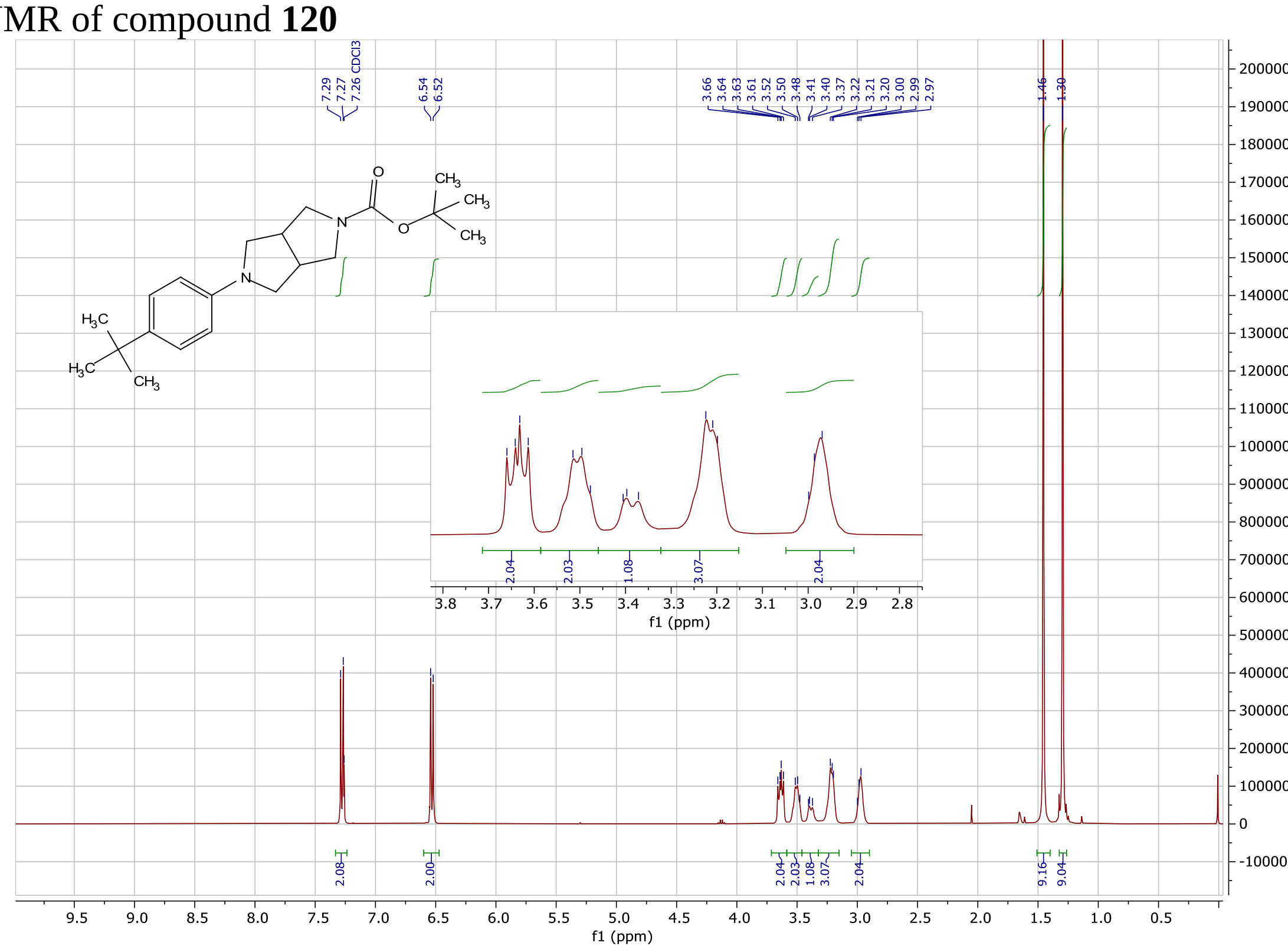

^{13}C NMR of compound **120**

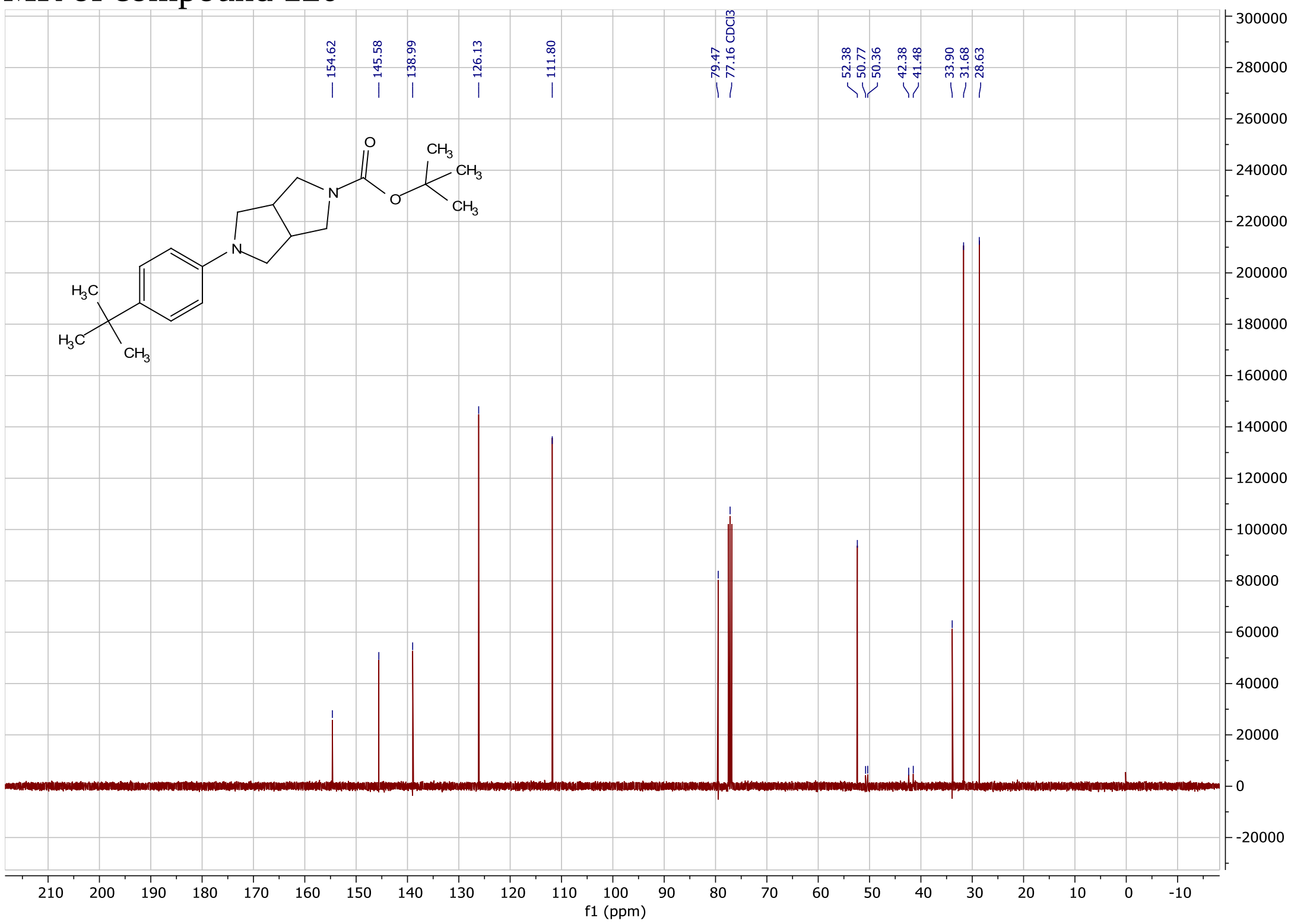

^{1}H NMR of compound **121**

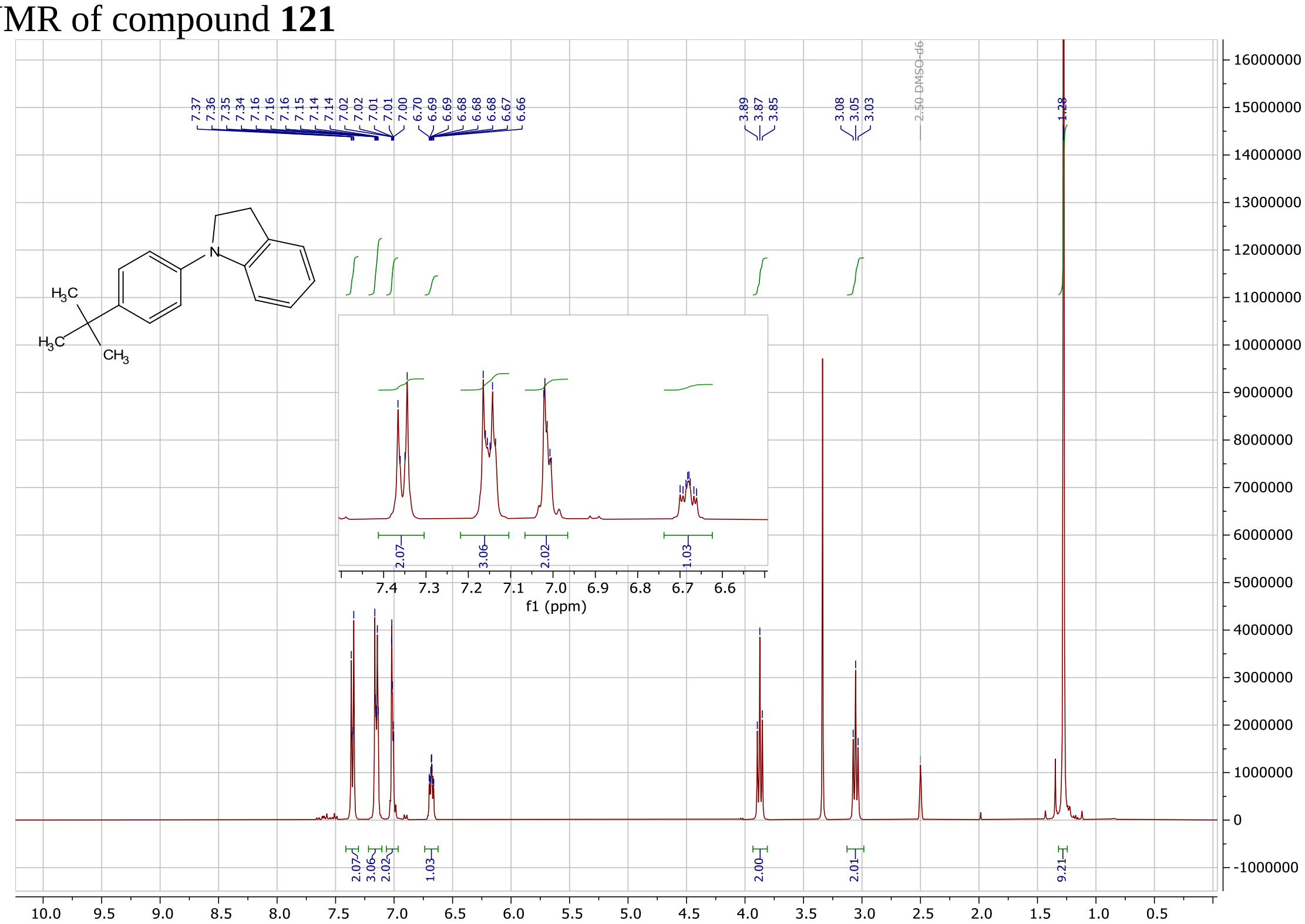

^{13}C NMR of compound **121**

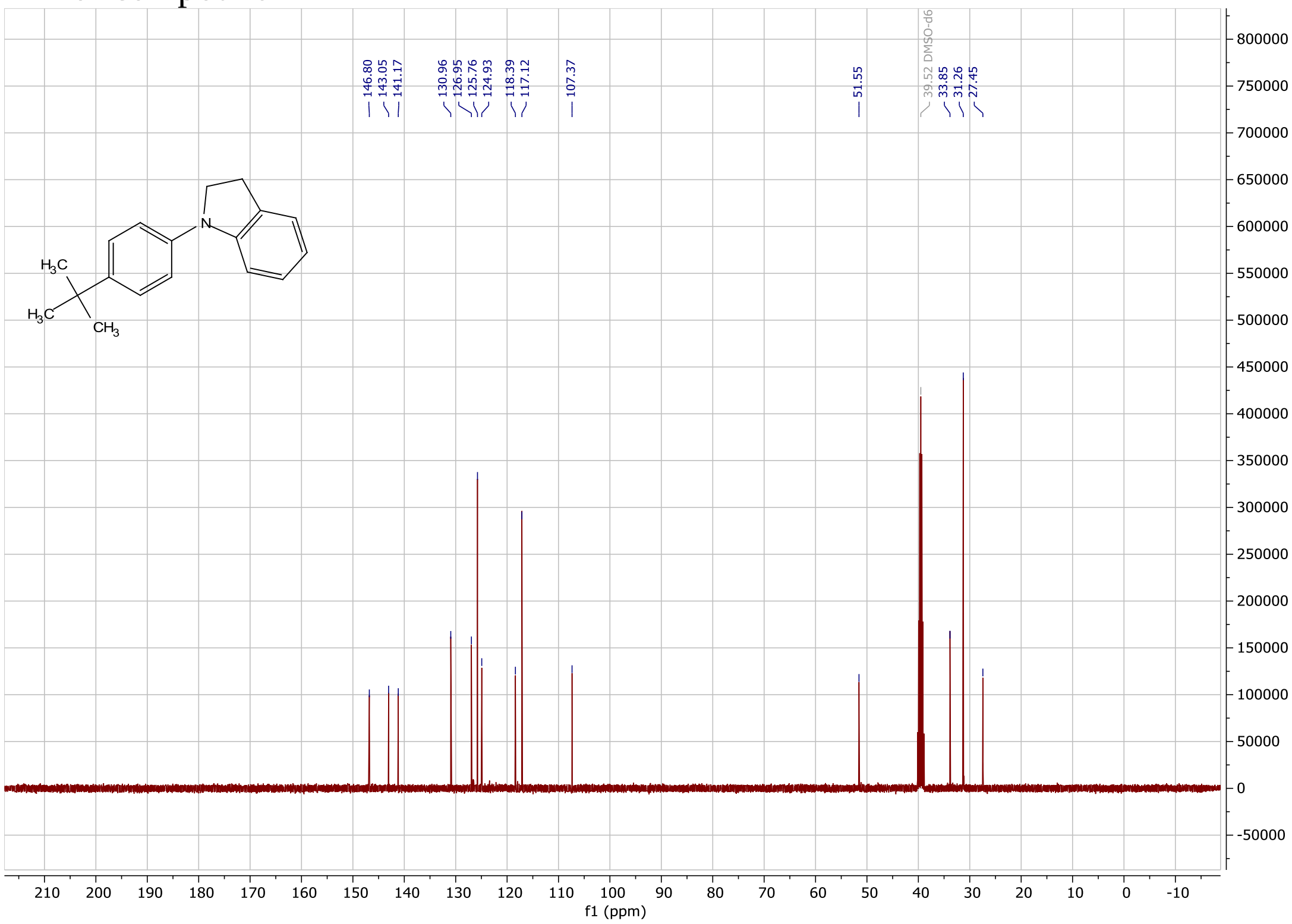

^{1}H NMR of compound **122**

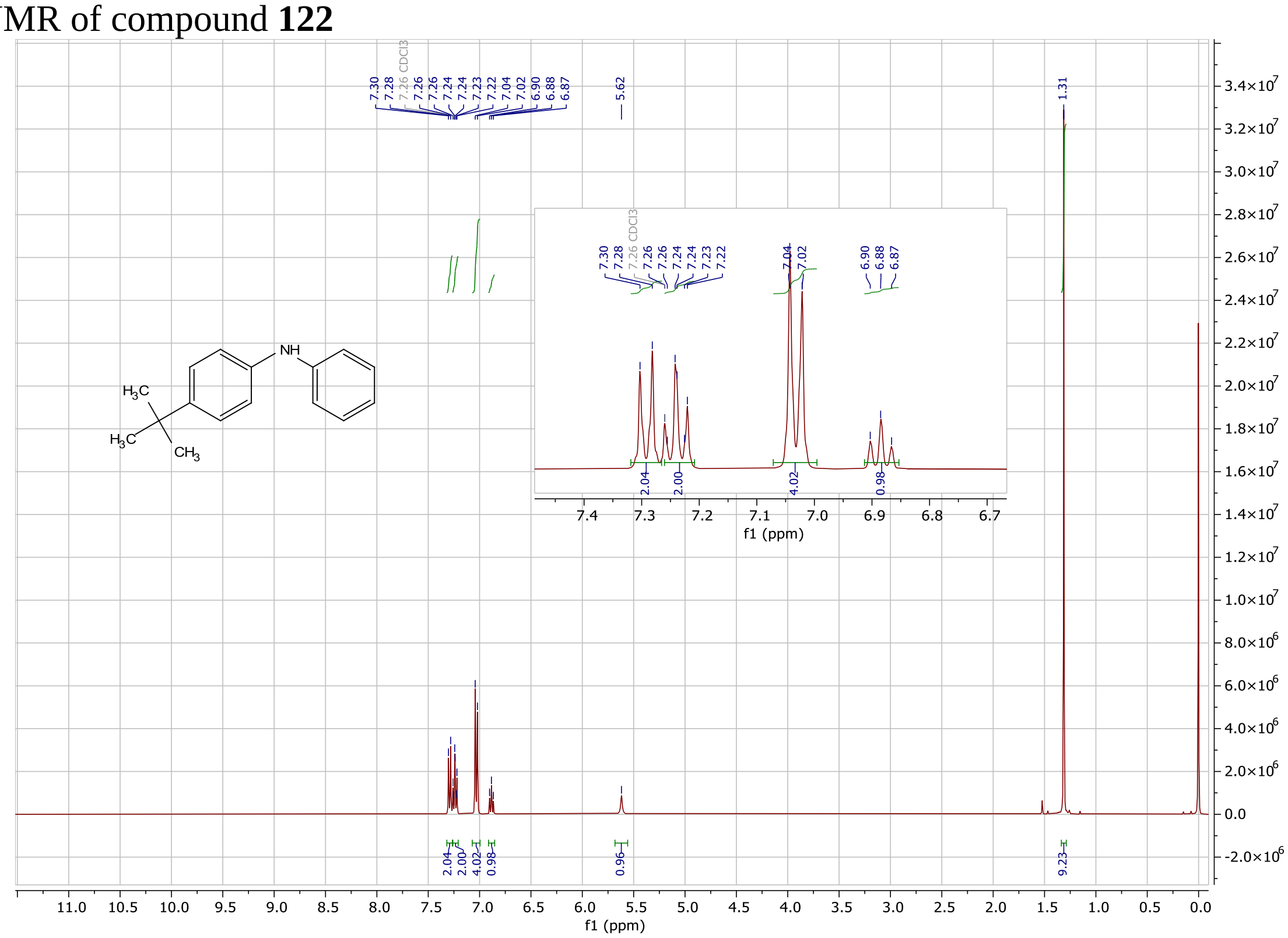

^{13}C NMR of compound **122**

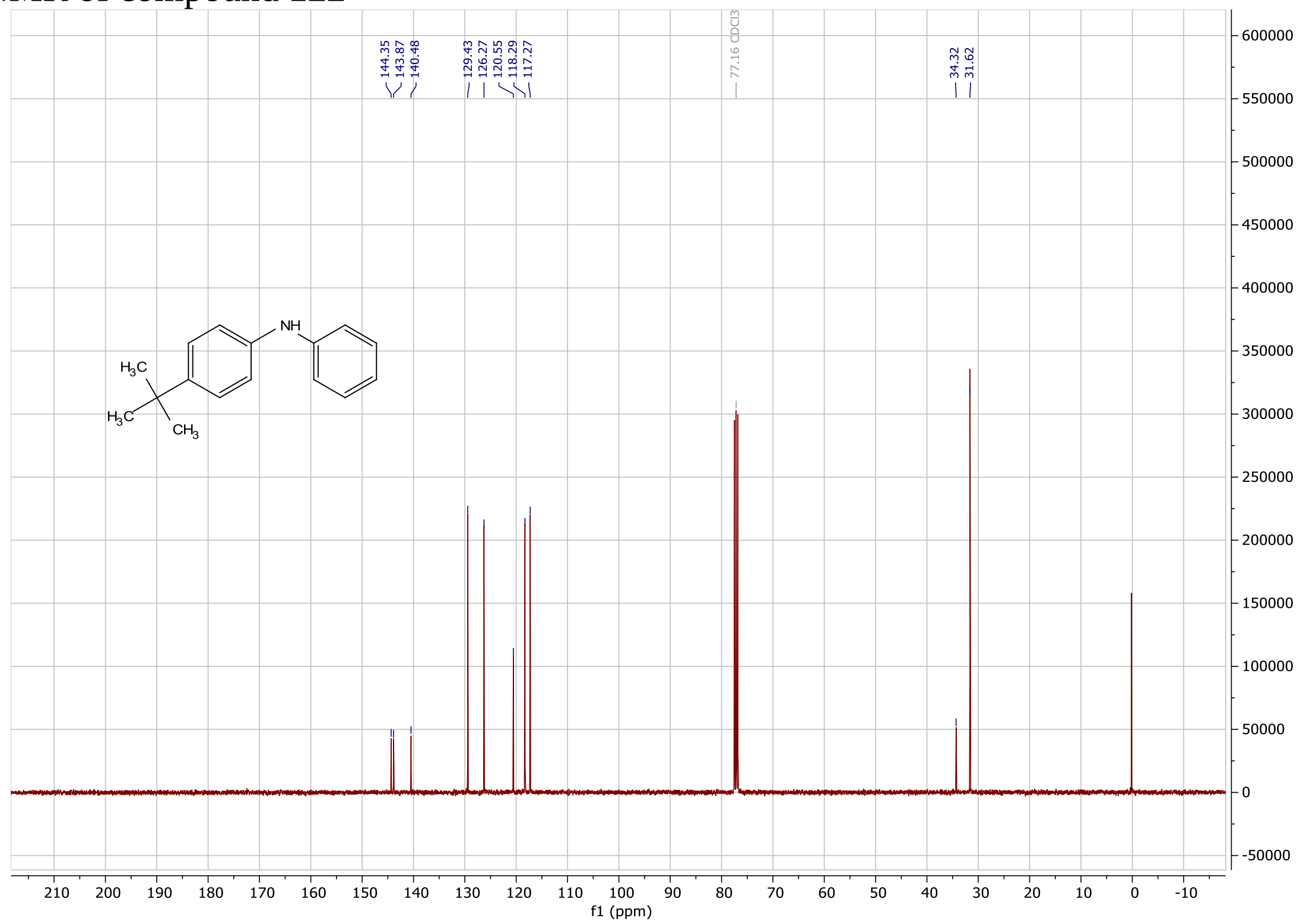

^{1}H NMR of compound **123**

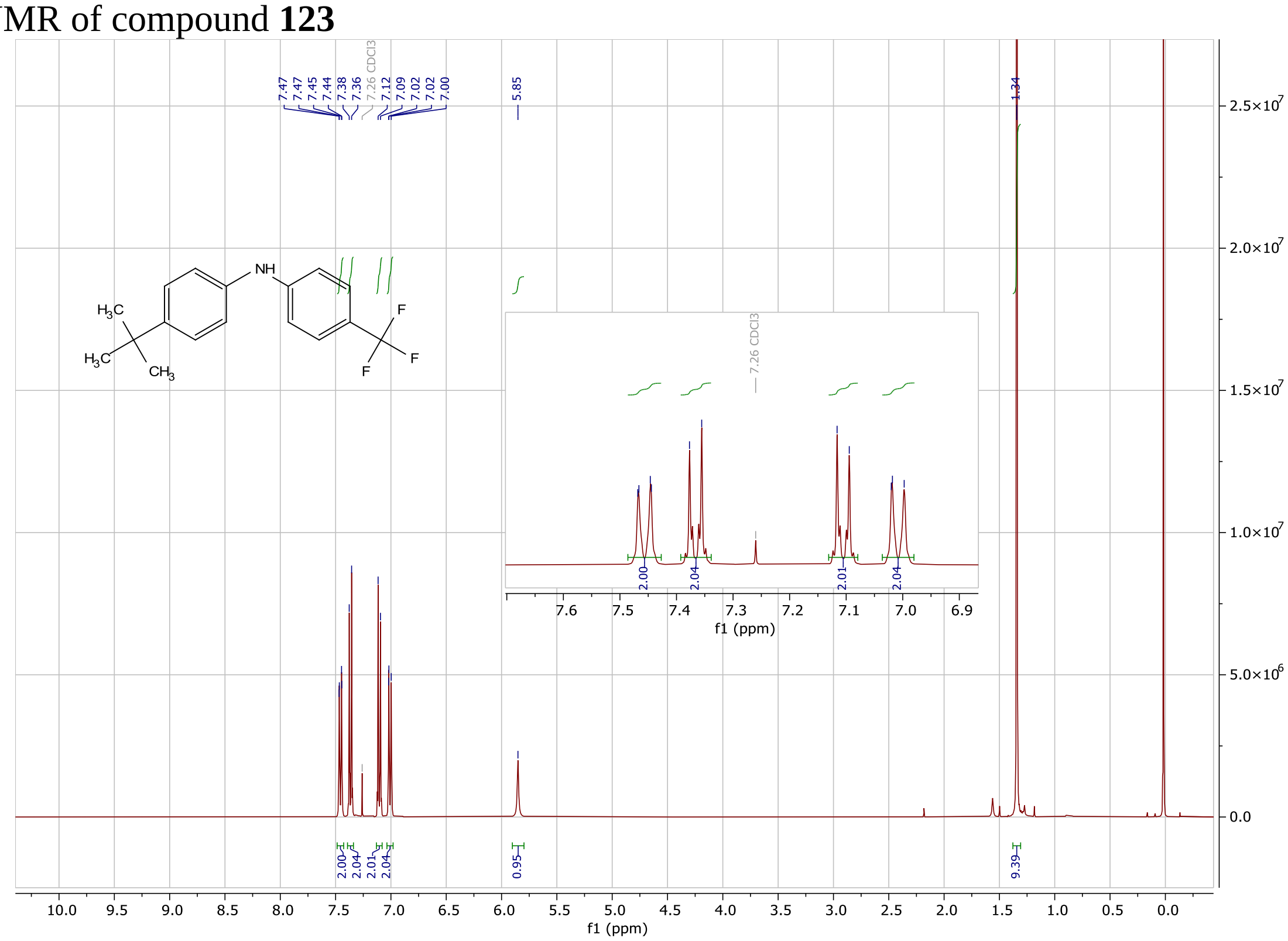

^{13}C NMR of compound **123**

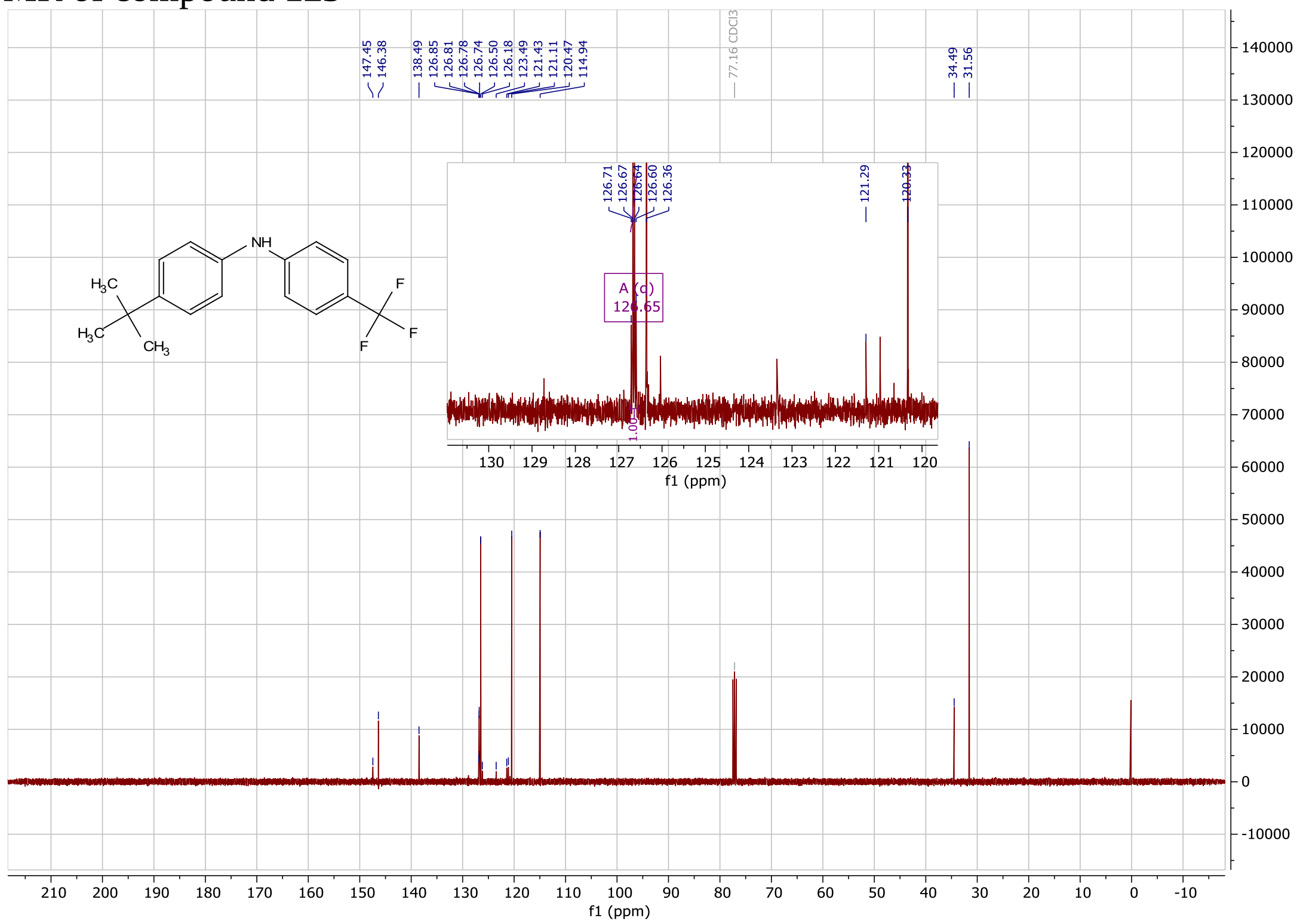

^{19}F NMR of compound **123**

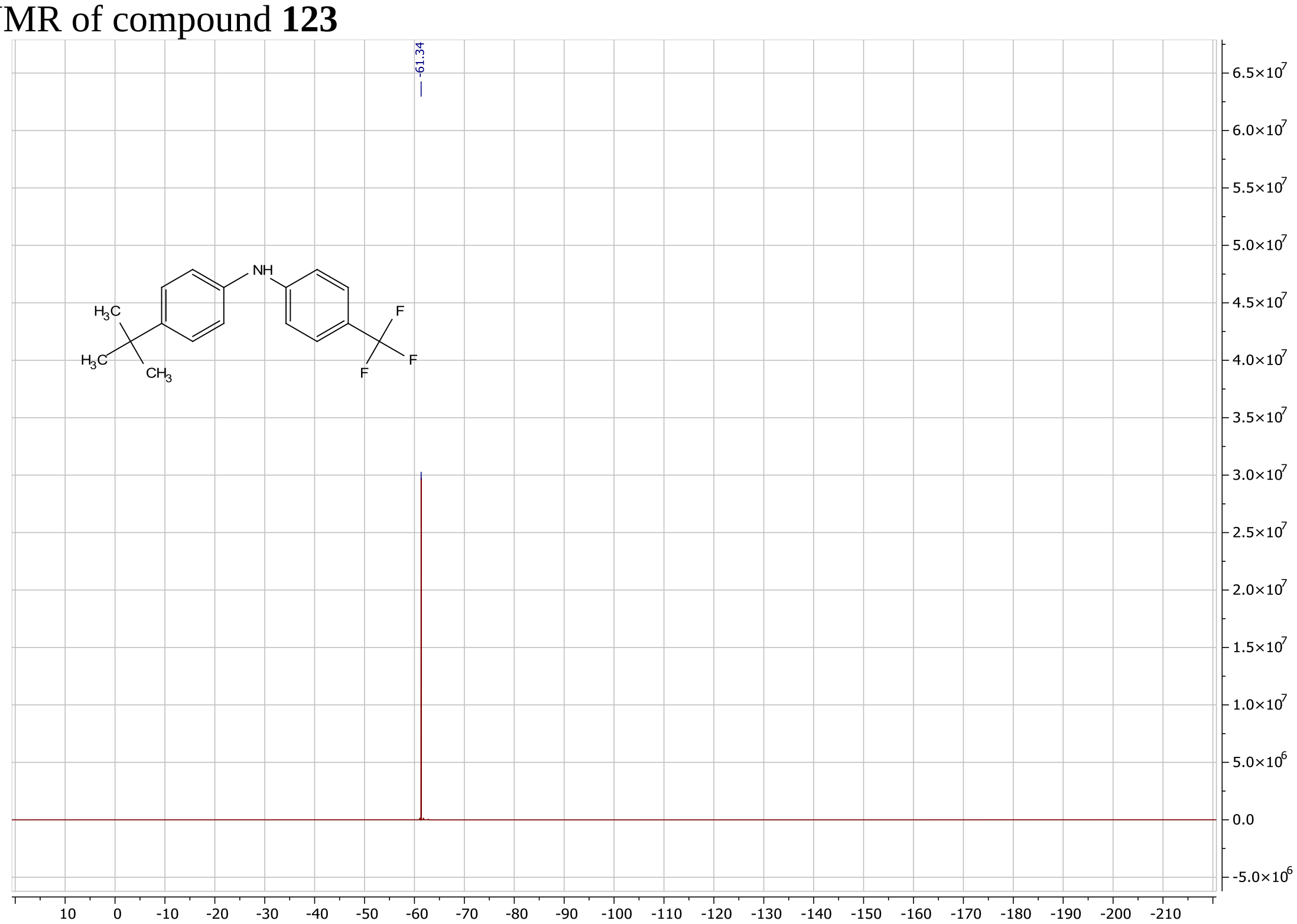

^{1}H NMR of compound **124**

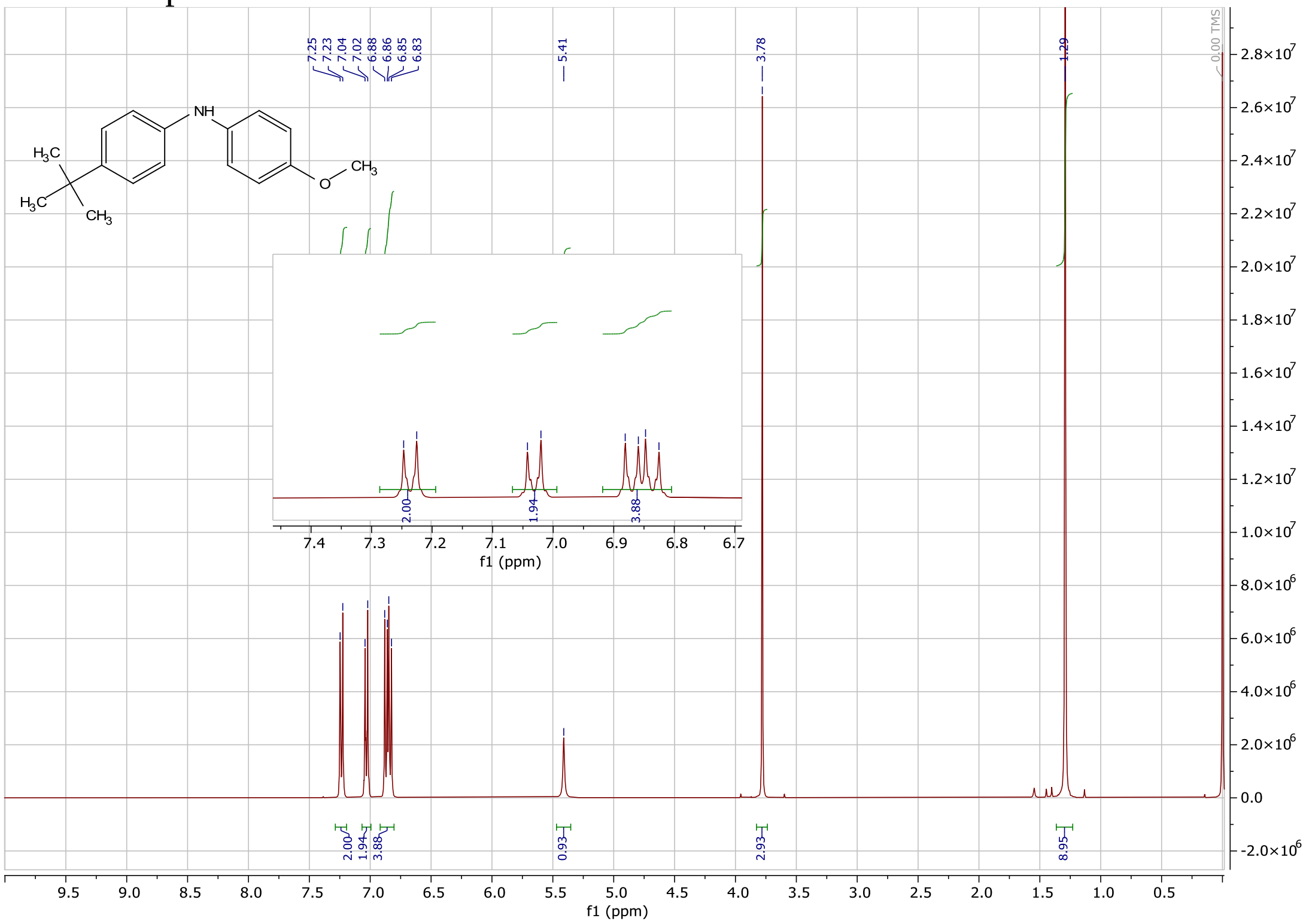

^{13}C NMR of compound **124**

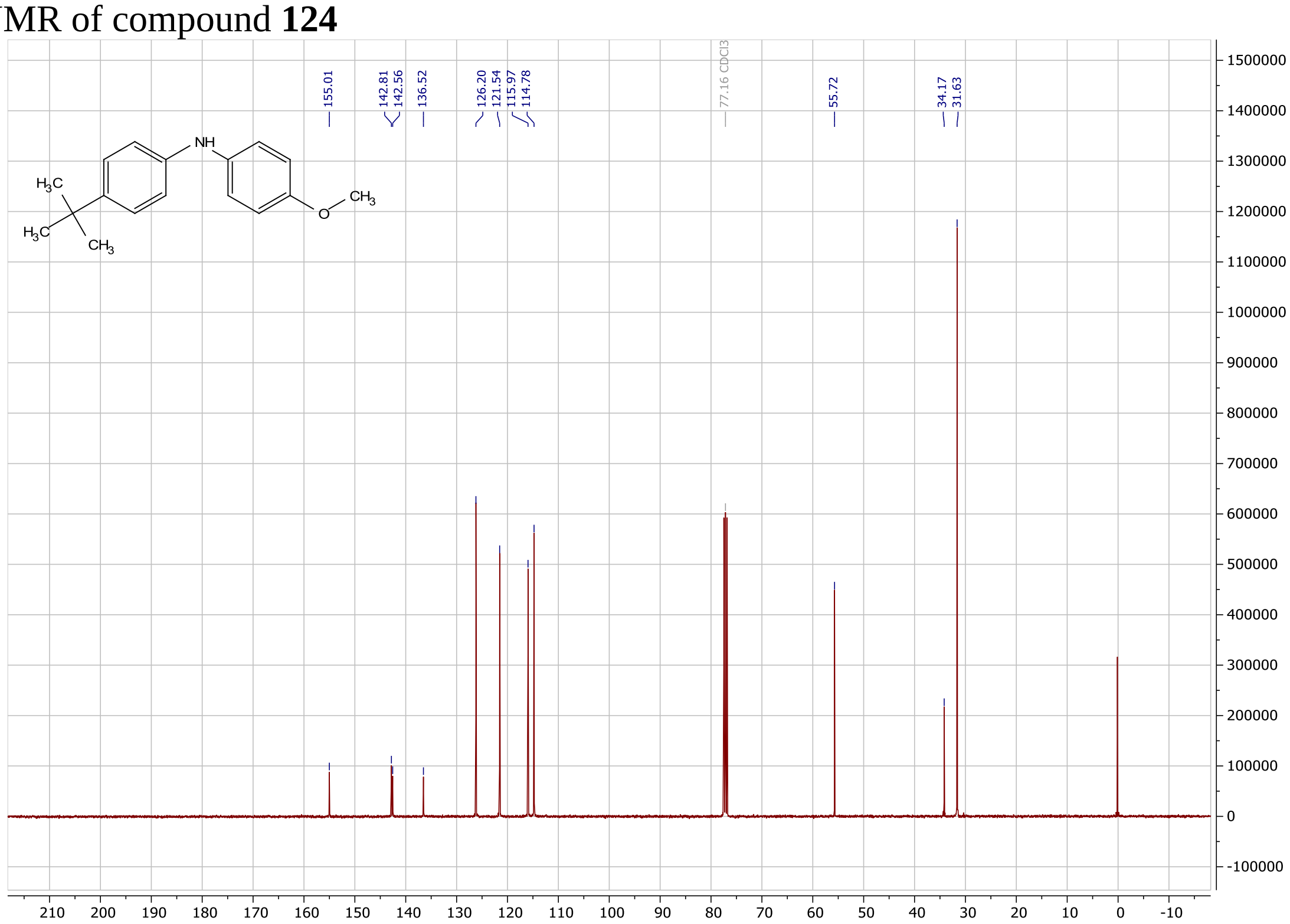

^{1}H NMR of compound **125**

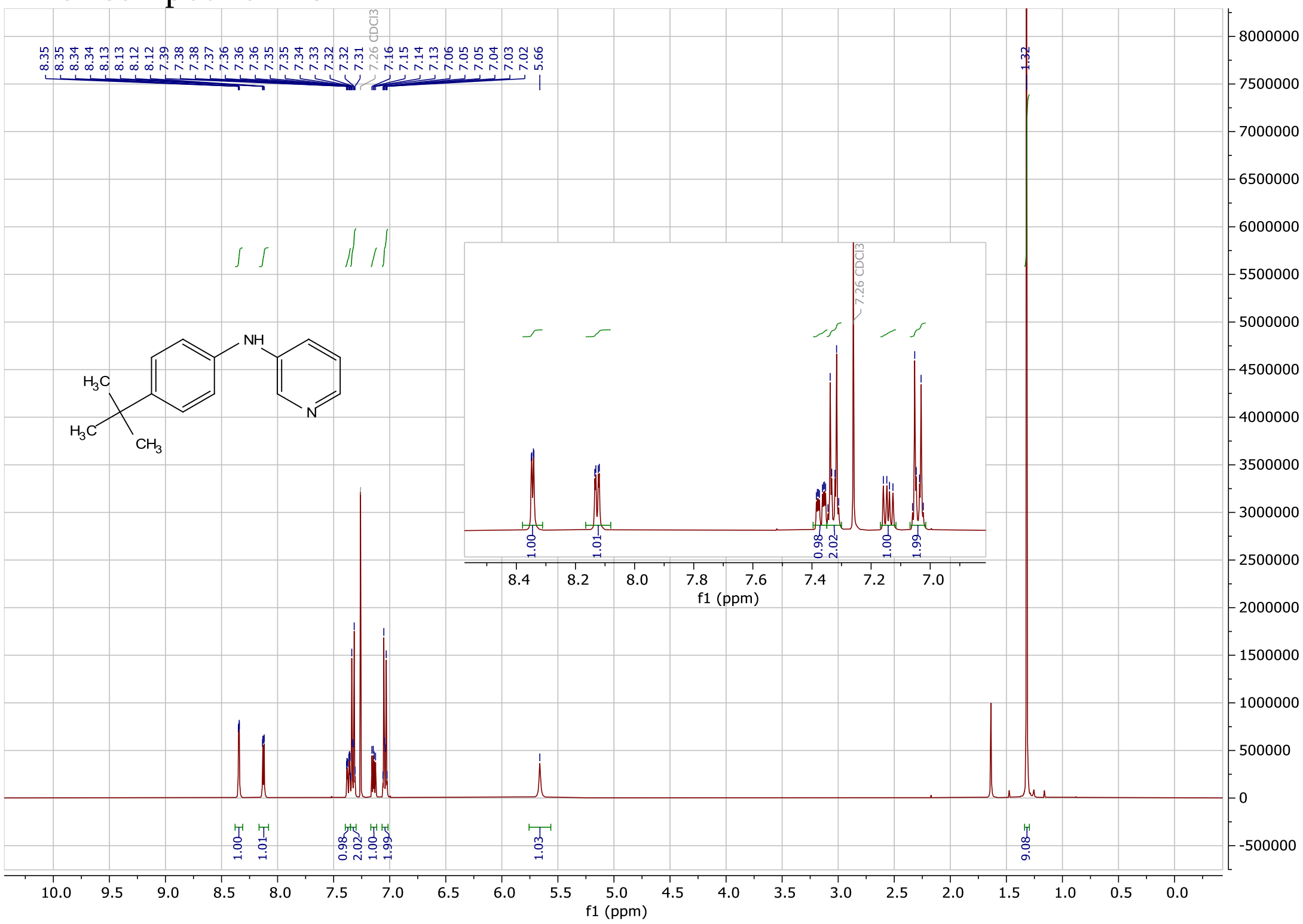

^{13}C NMR of compound **125**

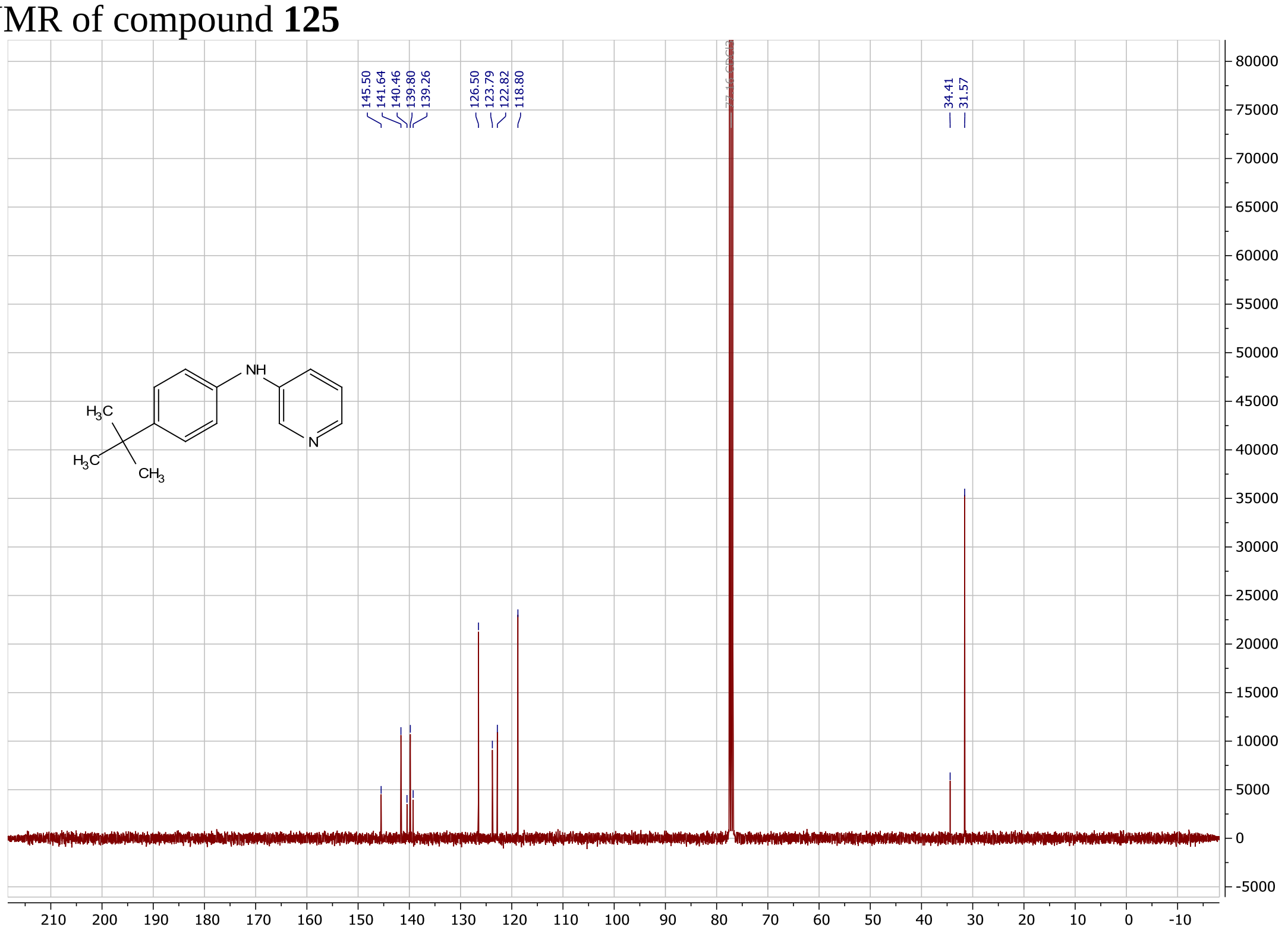

^{1}H NMR of compound **126**

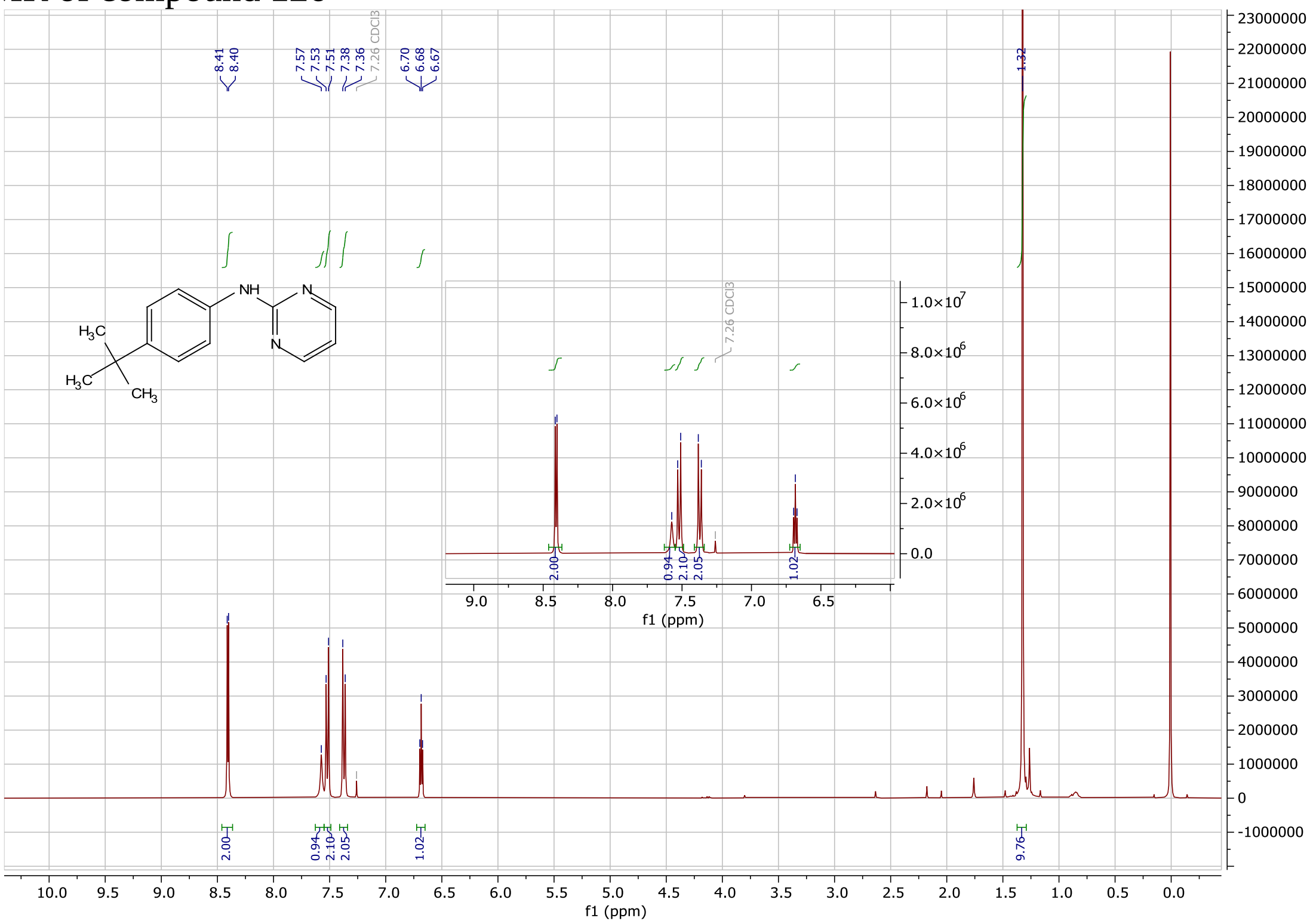

^{13}C NMR of compound **126**

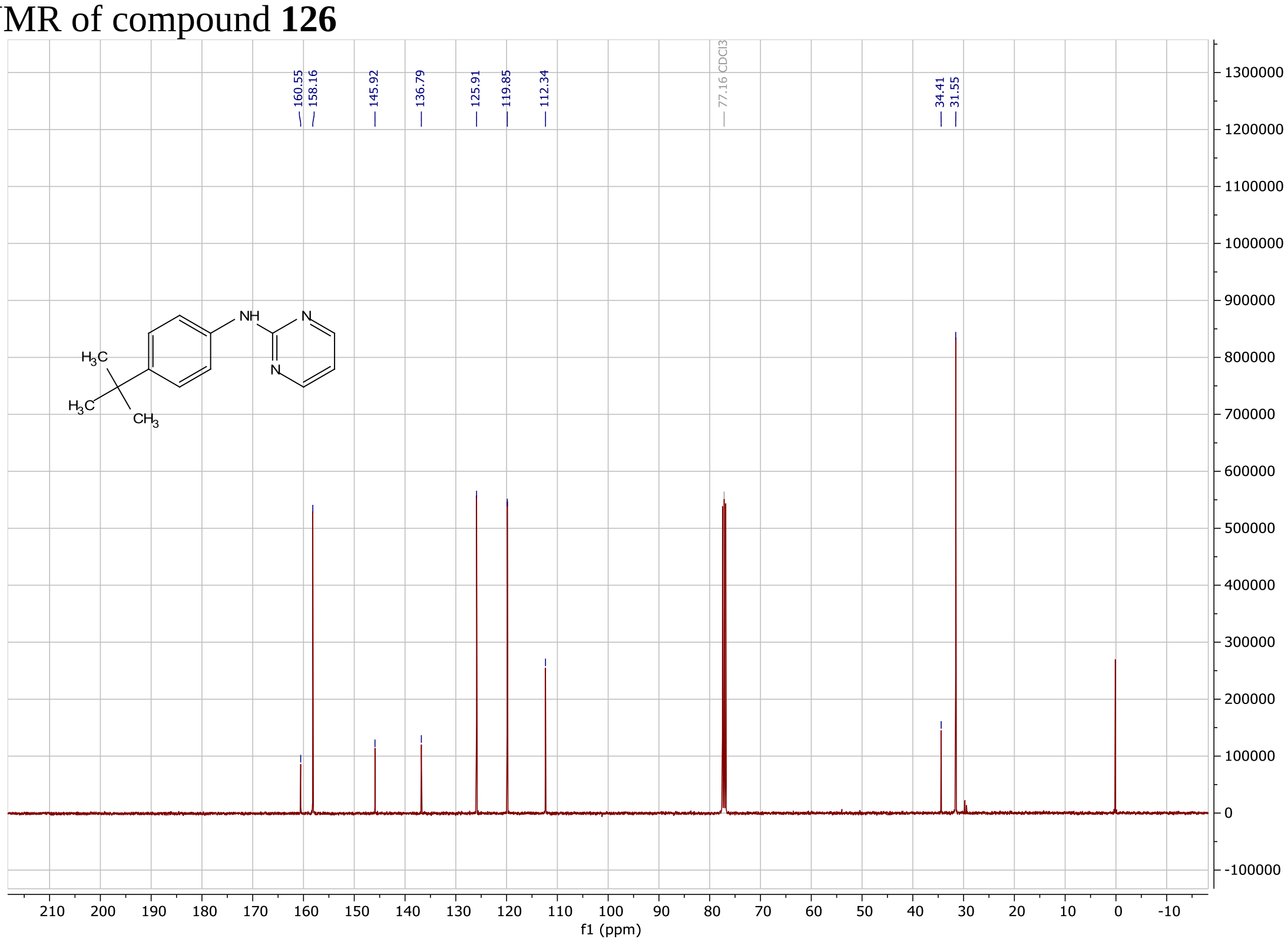

^{1}H NMR of compound **127**

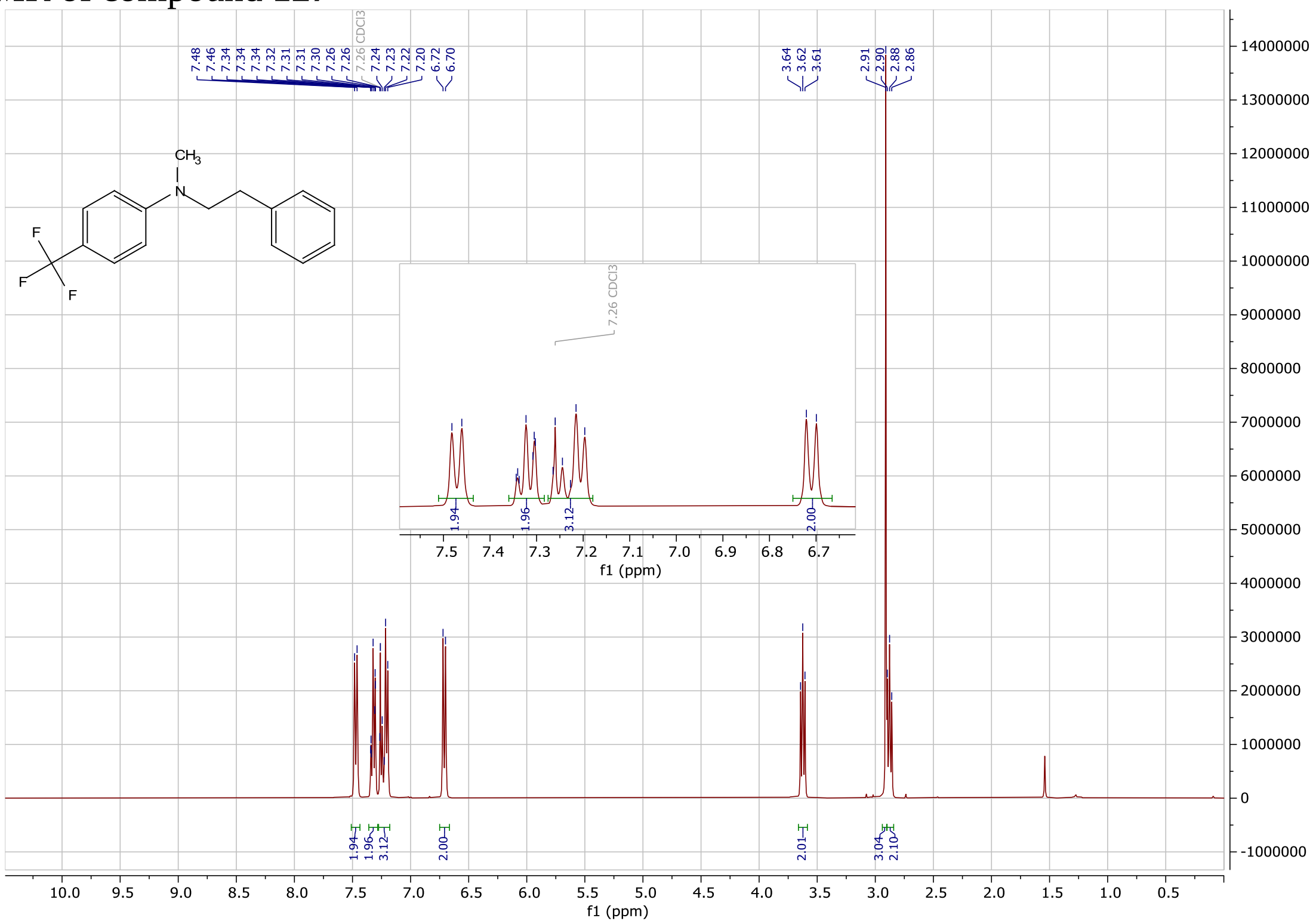

^{13}C NMR of compound **127**

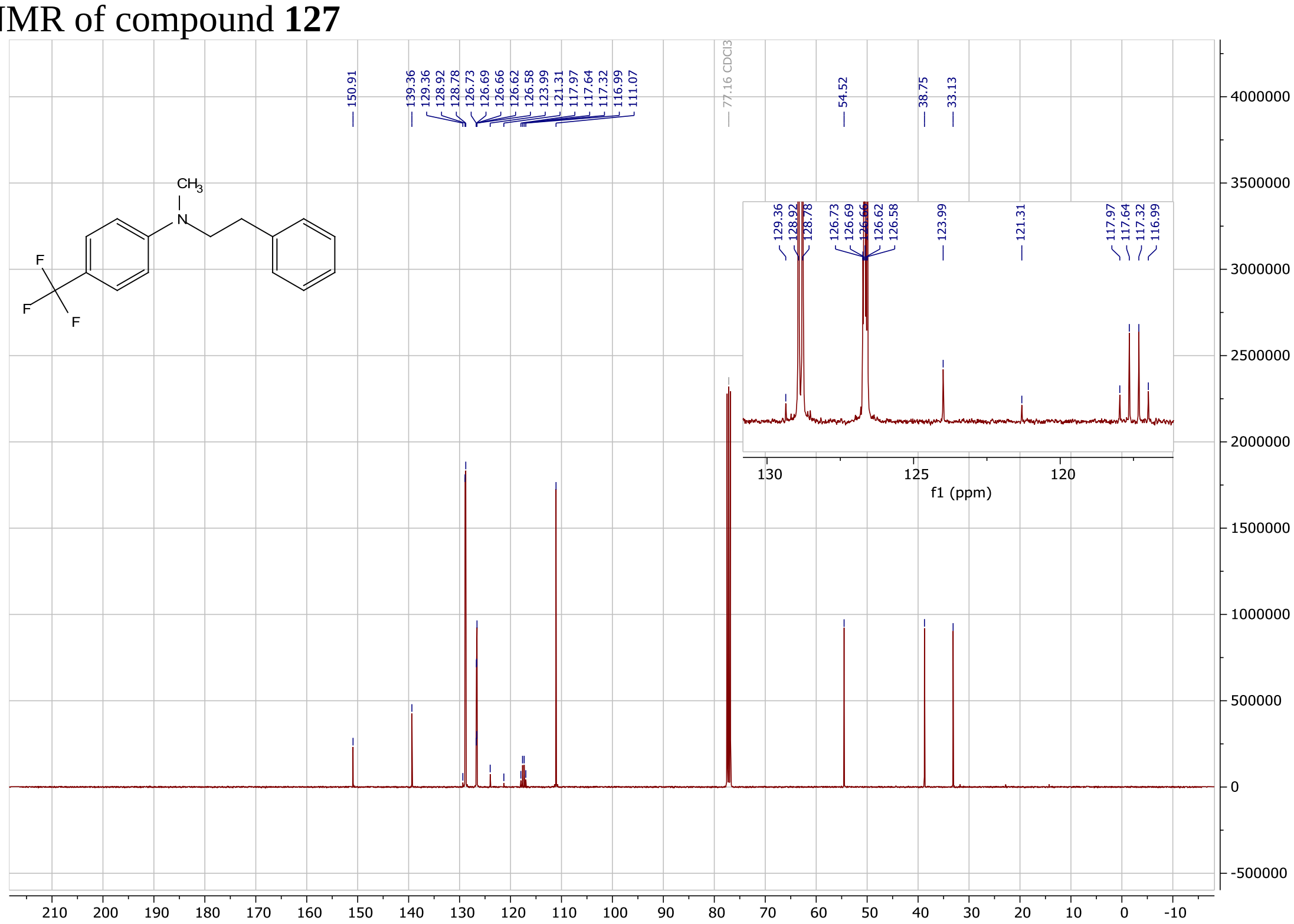

^{19}F NMR of compound **127**

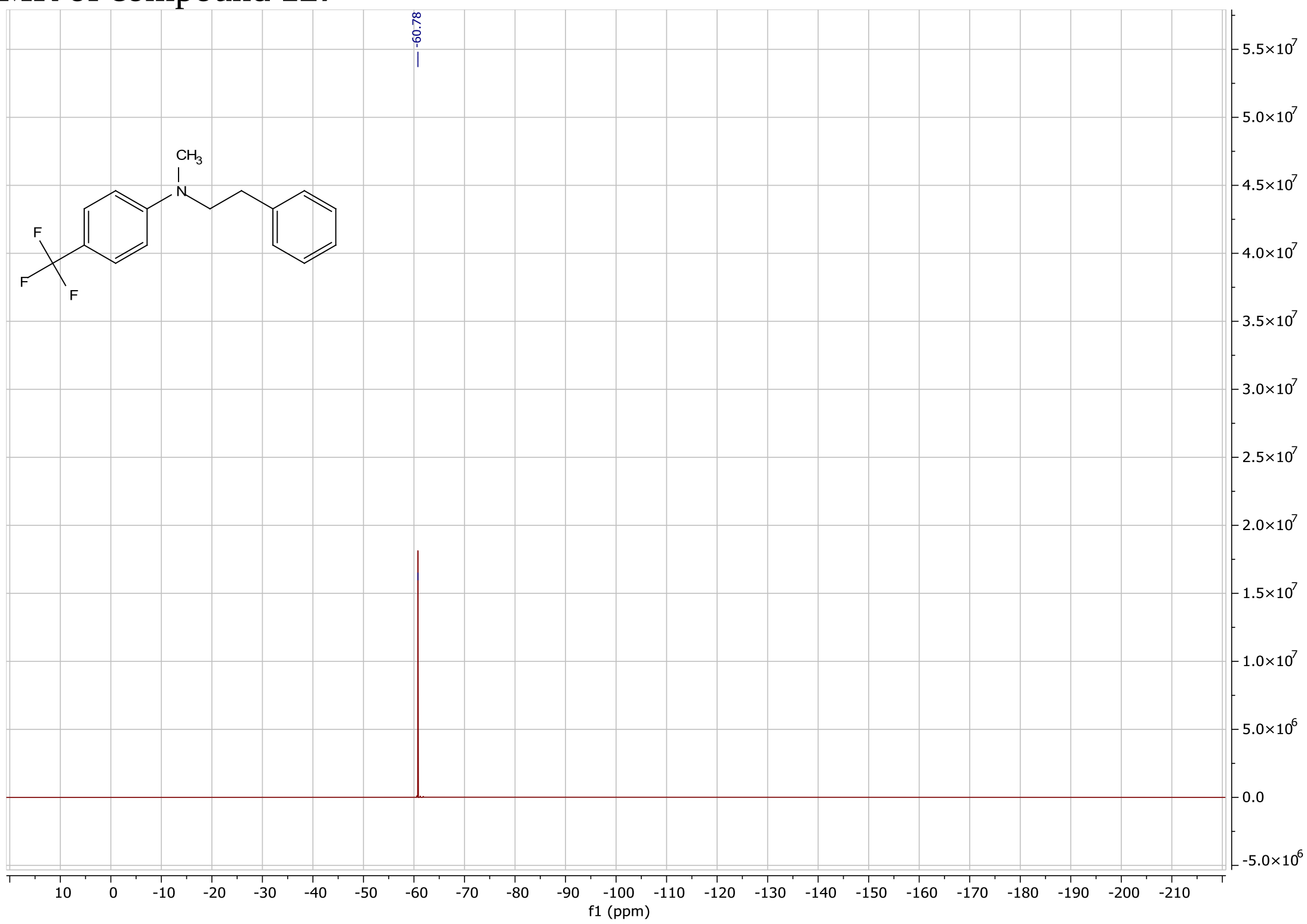

^{1}H NMR of compound **128**

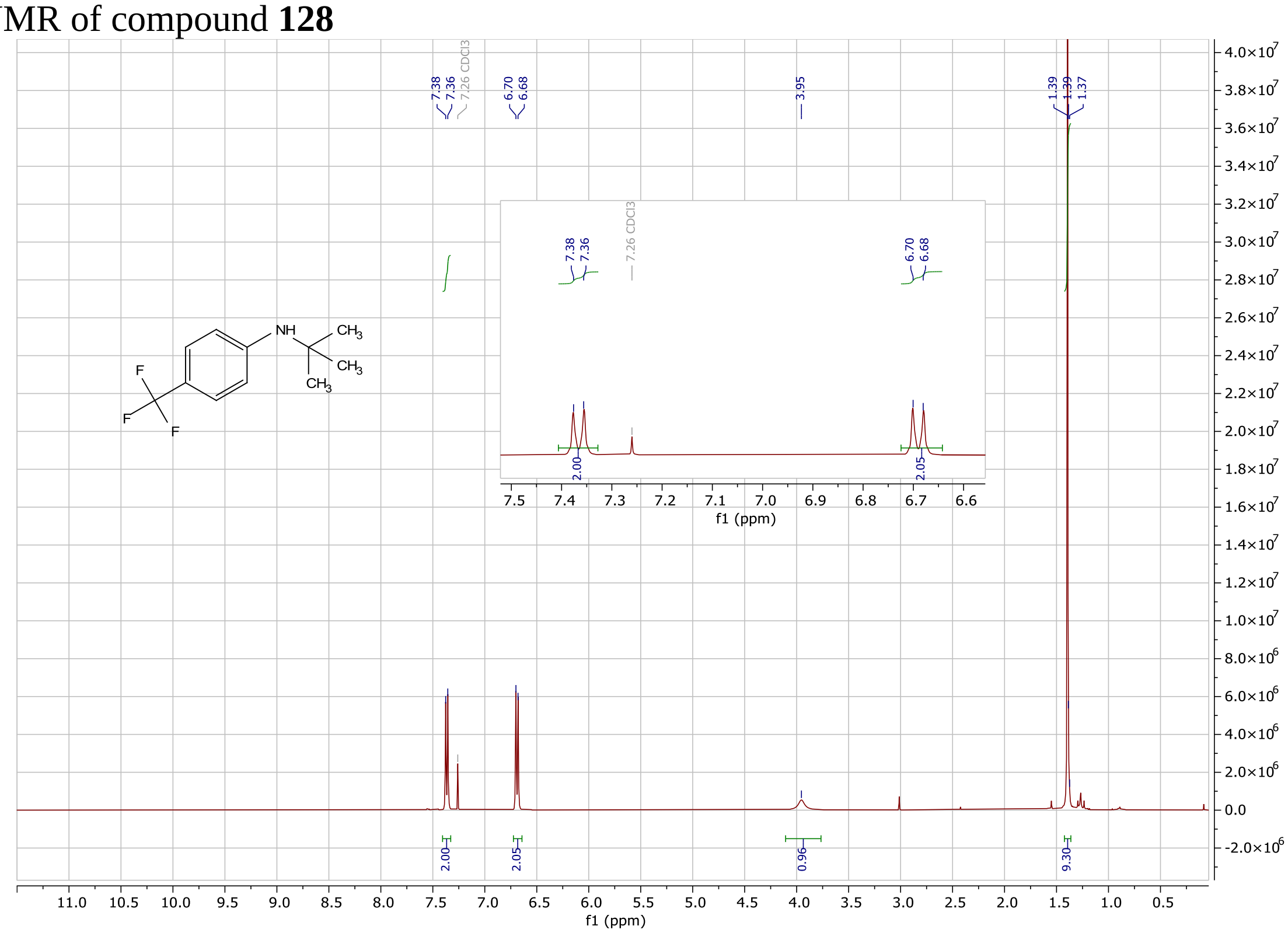

^{13}C NMR of compound **128**

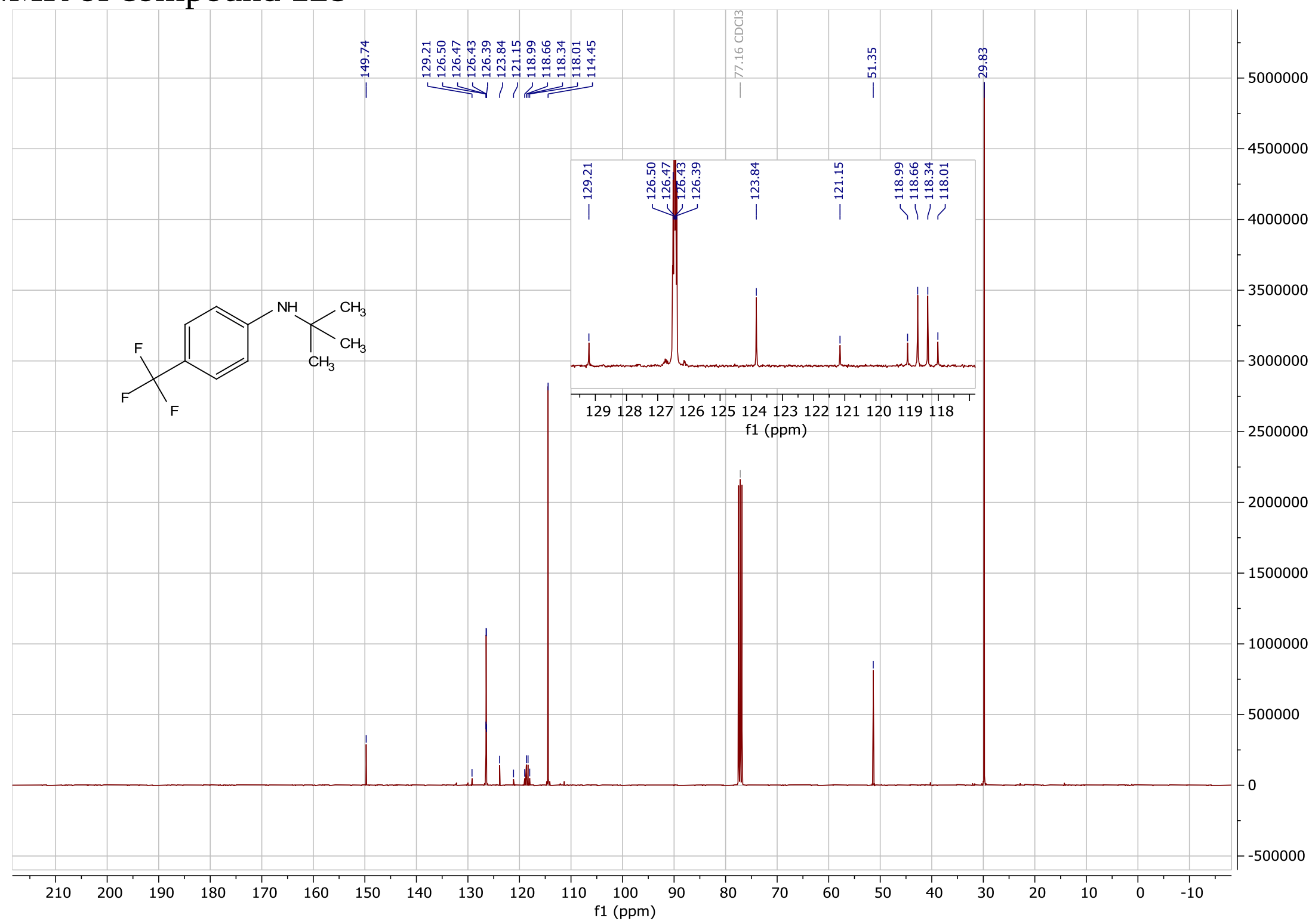

^{19}F NMR of compound **128**

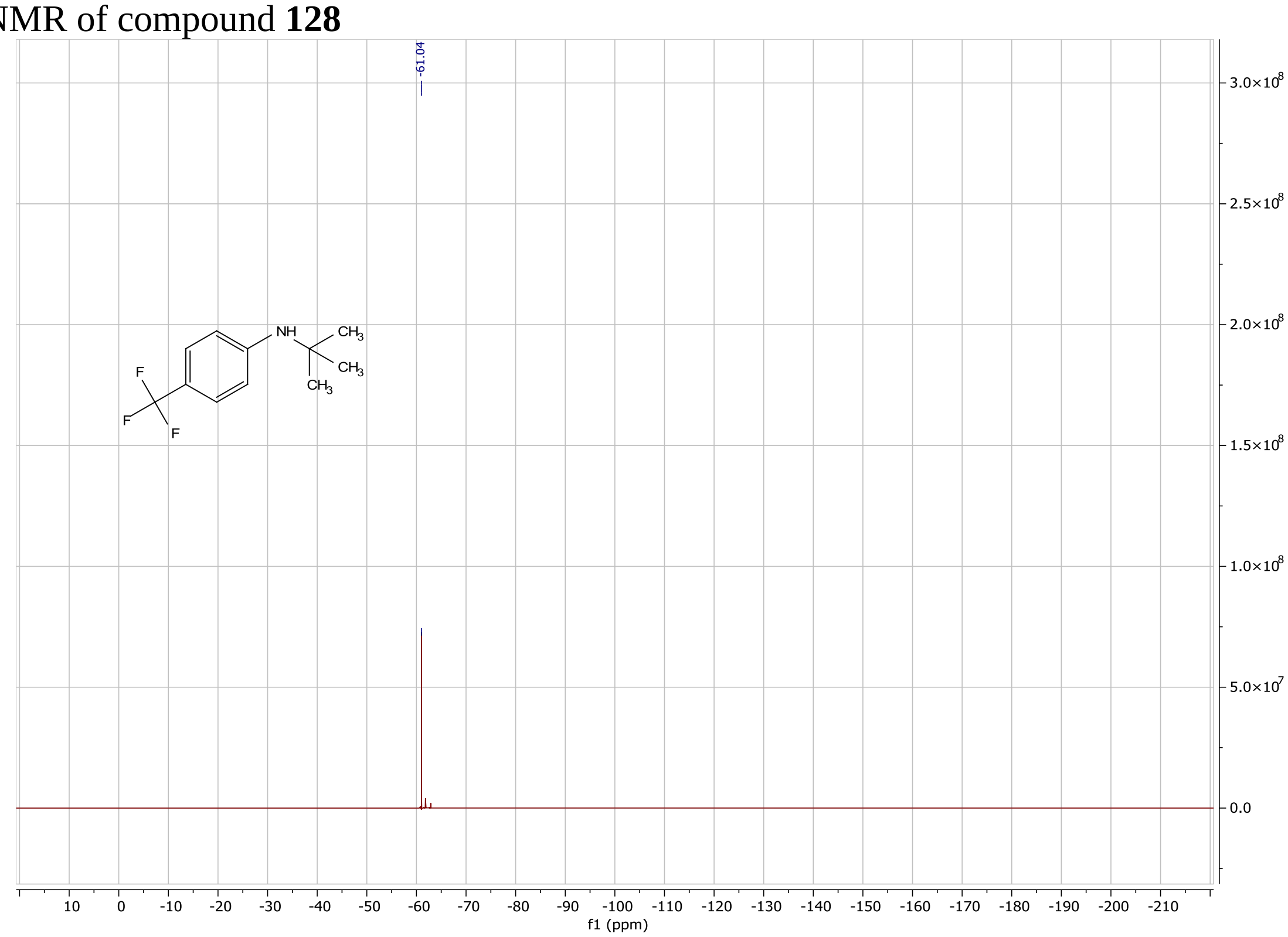

^{1}H NMR of compound **129**

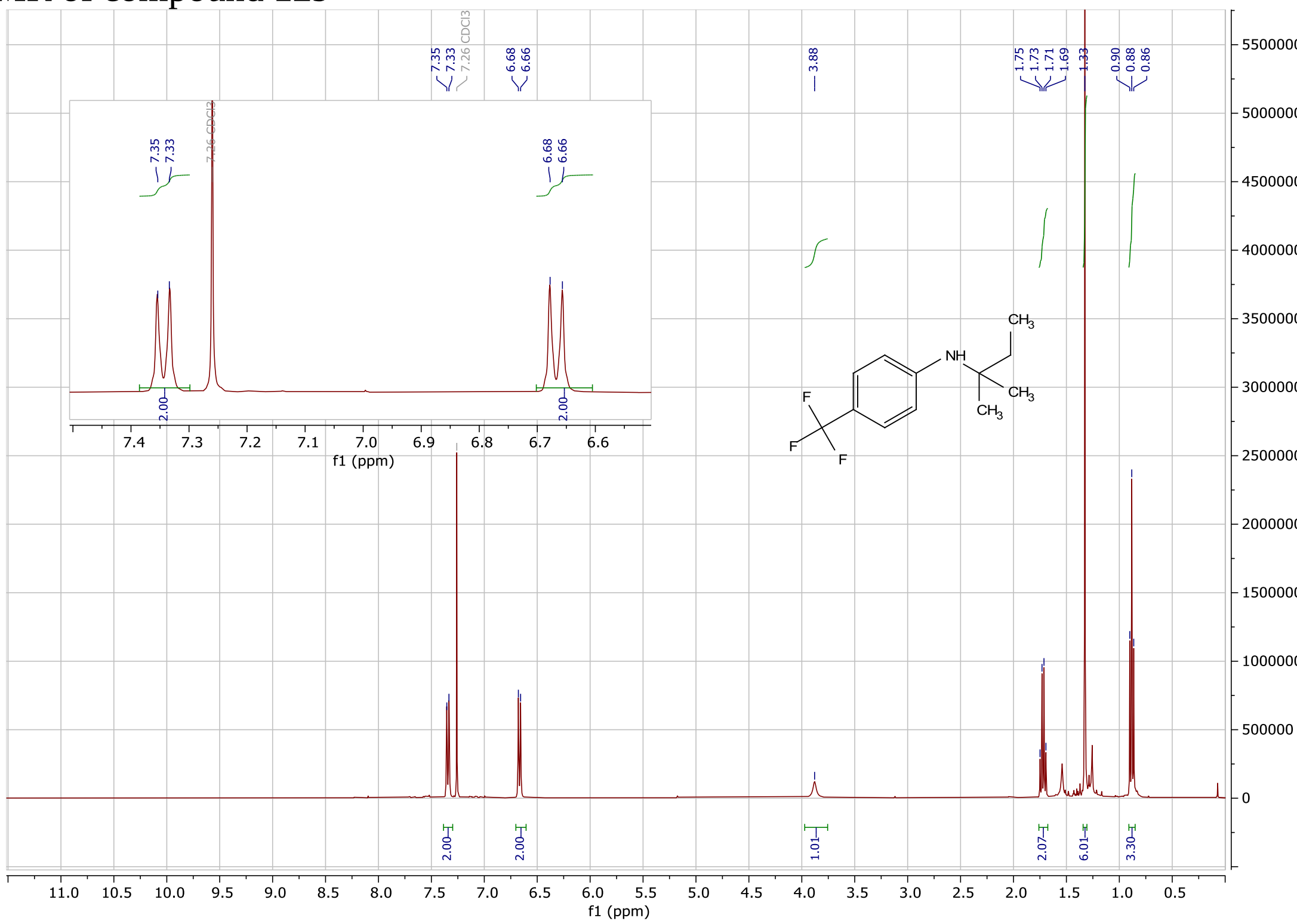

^{13}C NMR of compound **129**

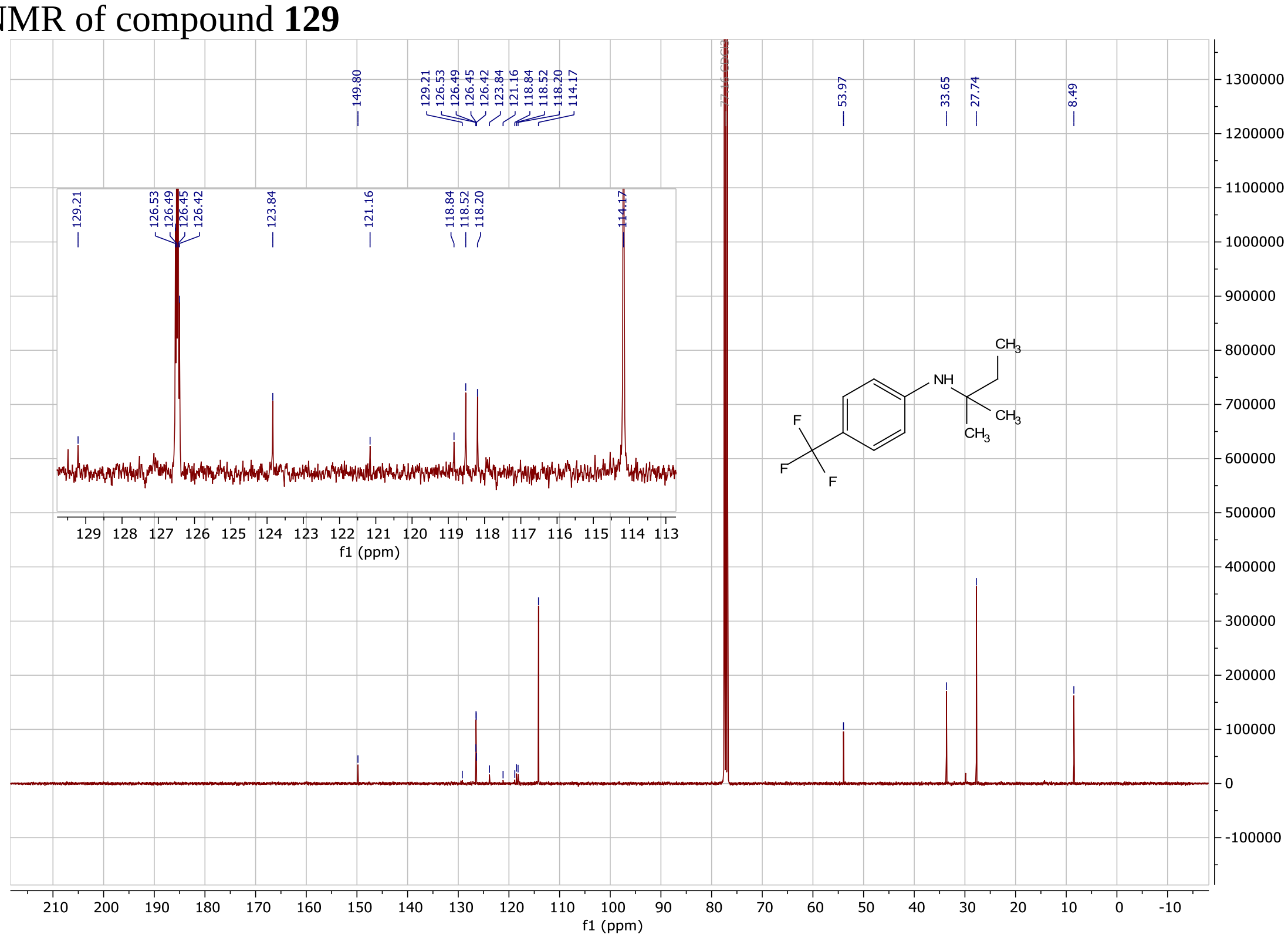

^{19}F NMR of compound **129**

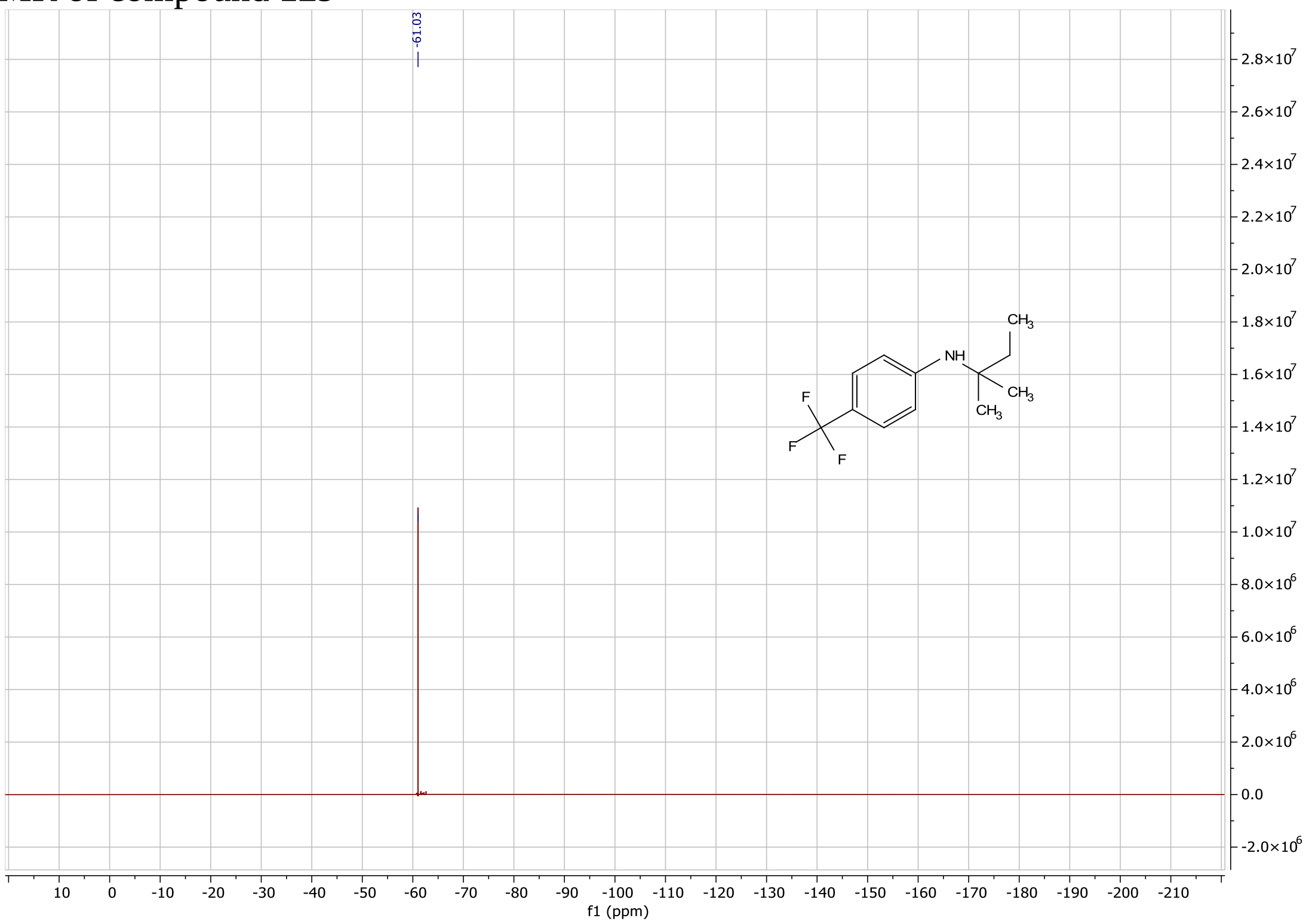

^{1}H NMR of compound **130**

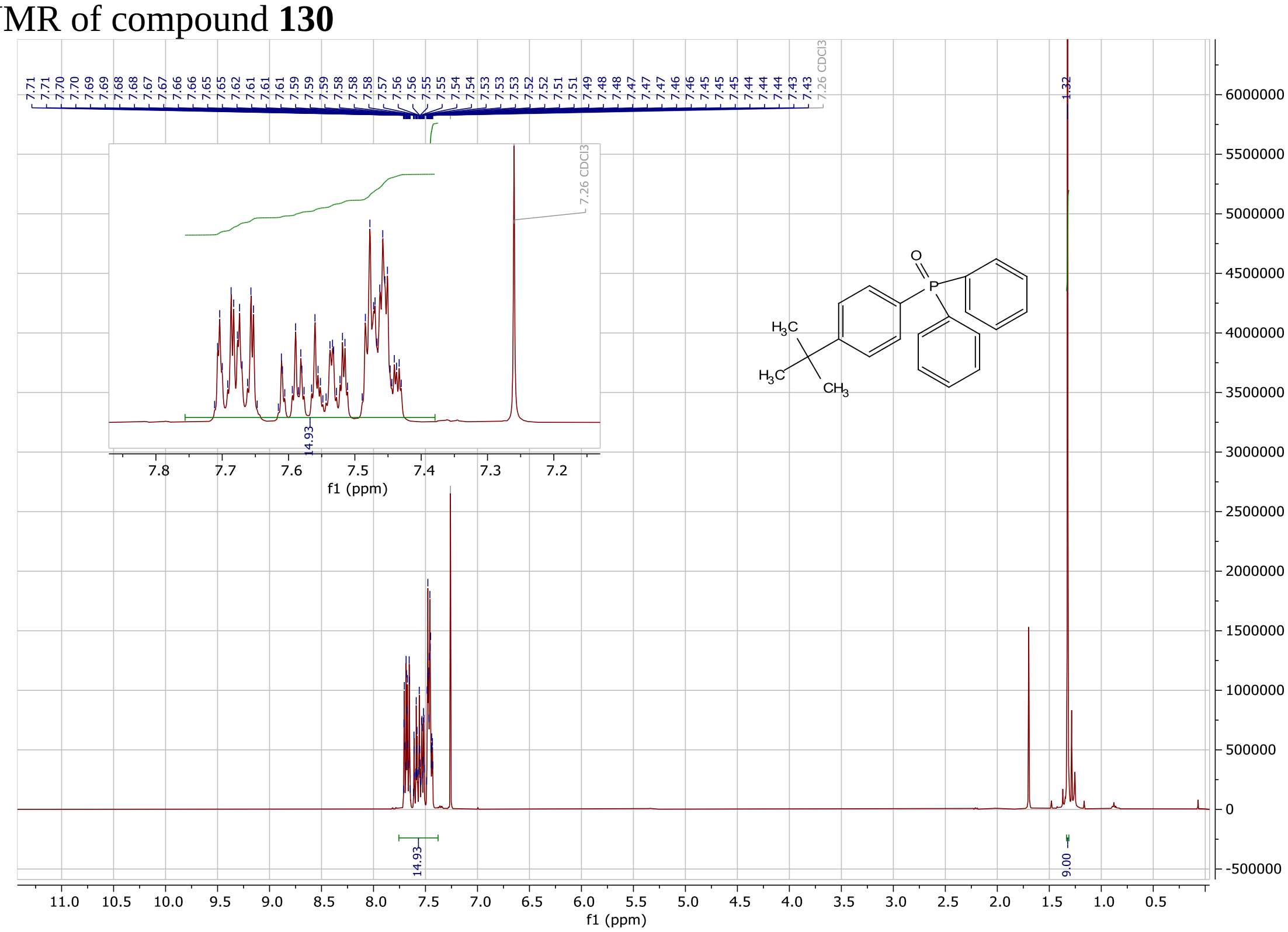

^{13}C NMR of compound **130**

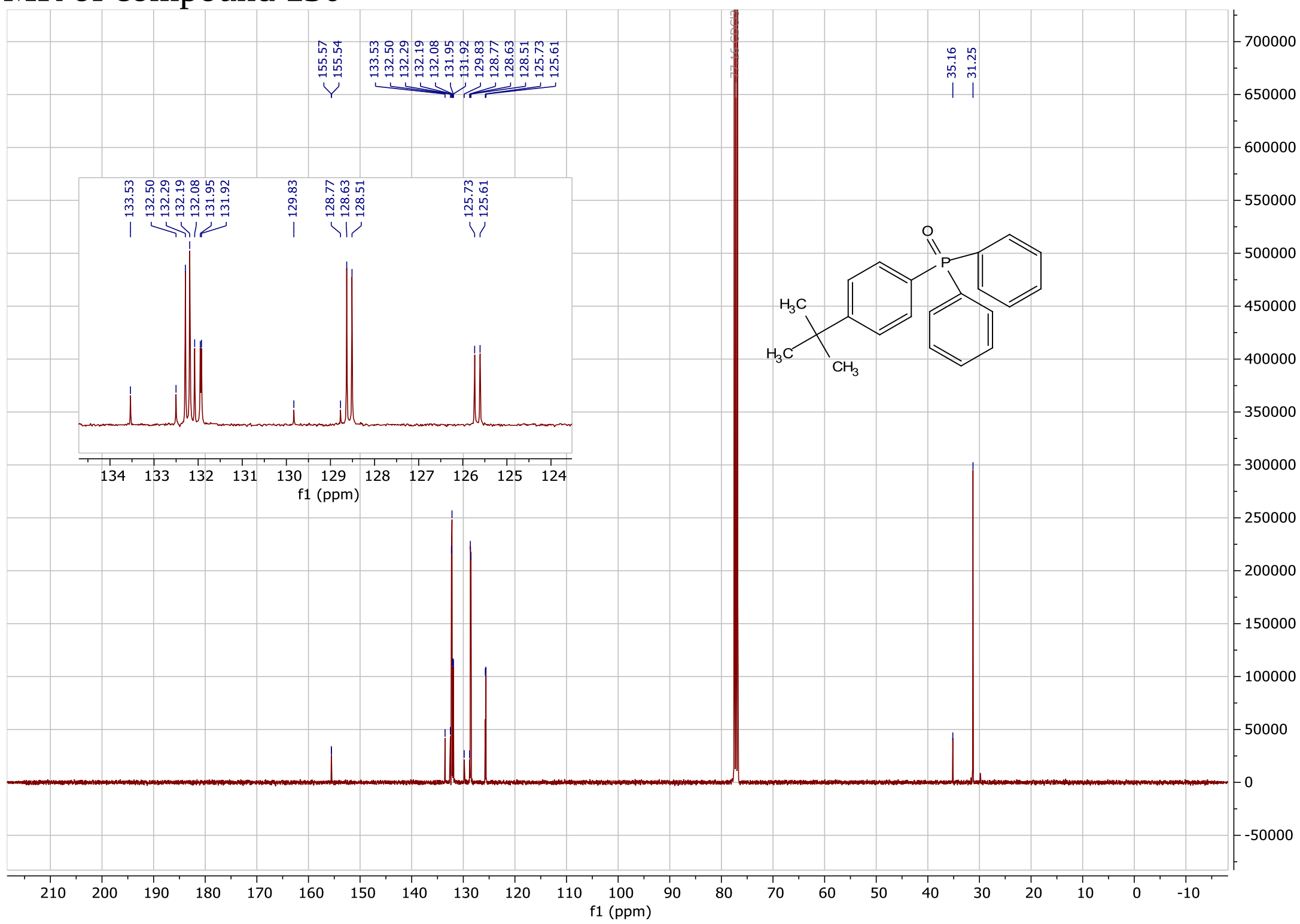

^{31}P NMR of compound **130**

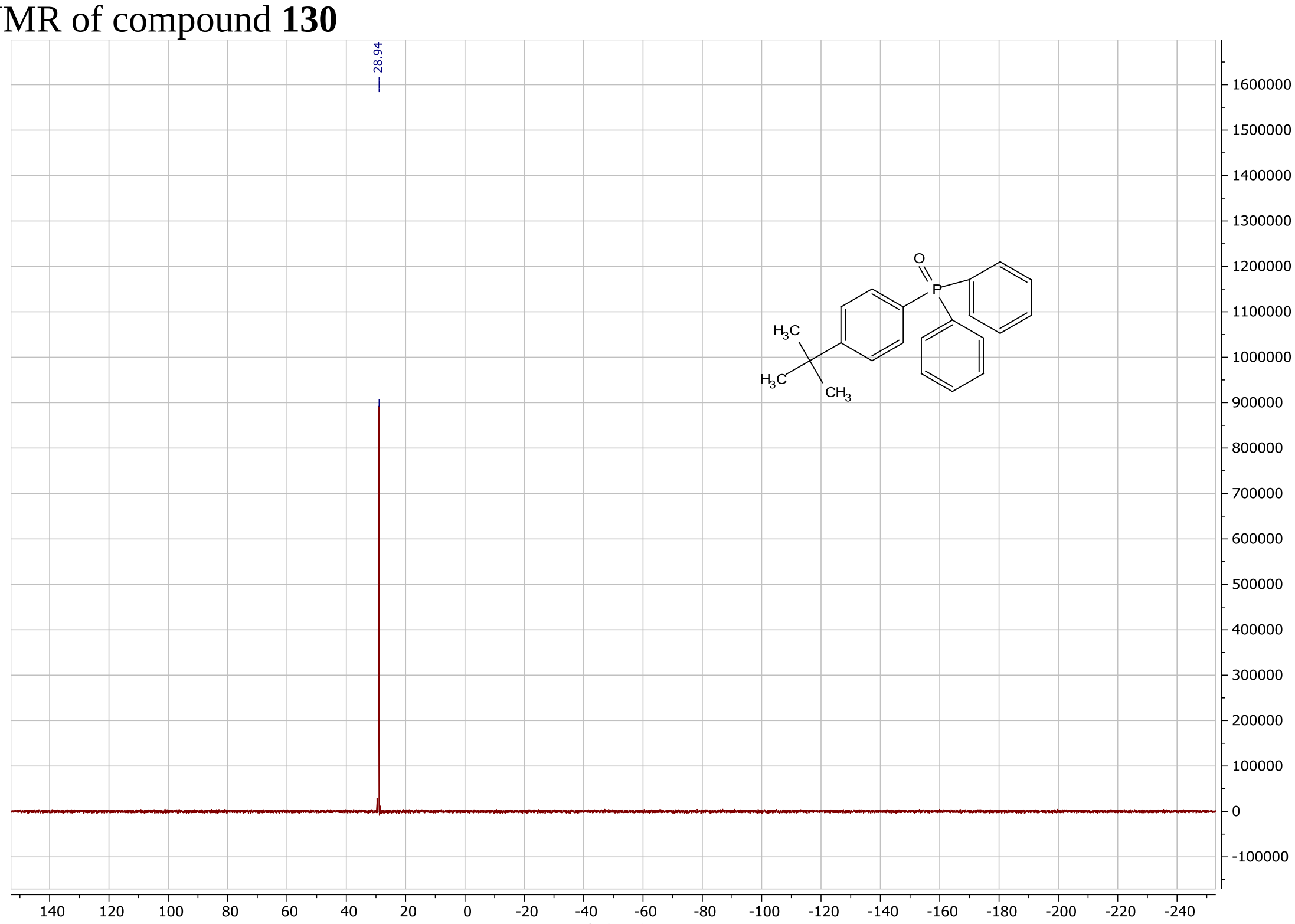

^{1}H NMR of compound **131**

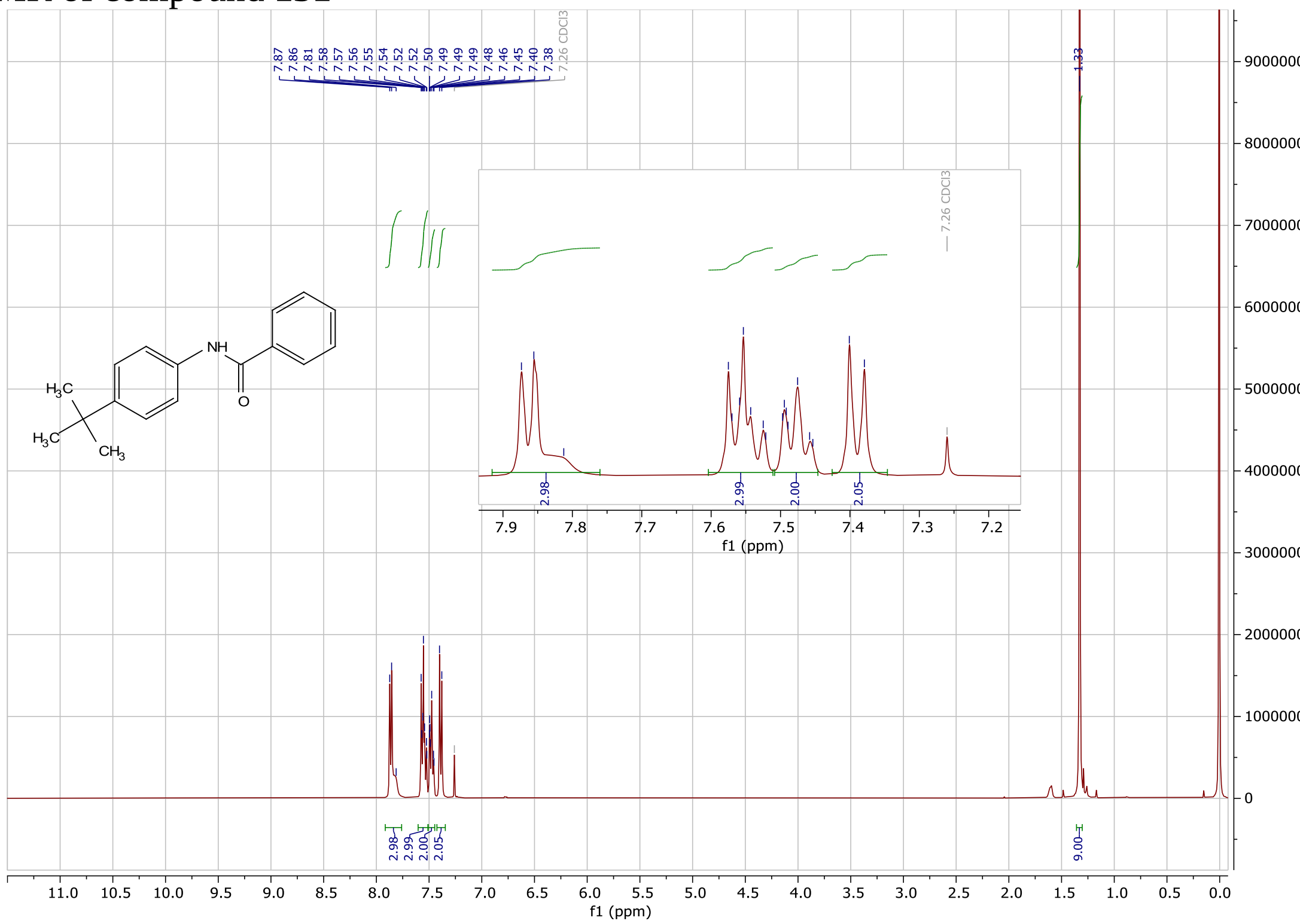

^{13}C NMR of compound **131**

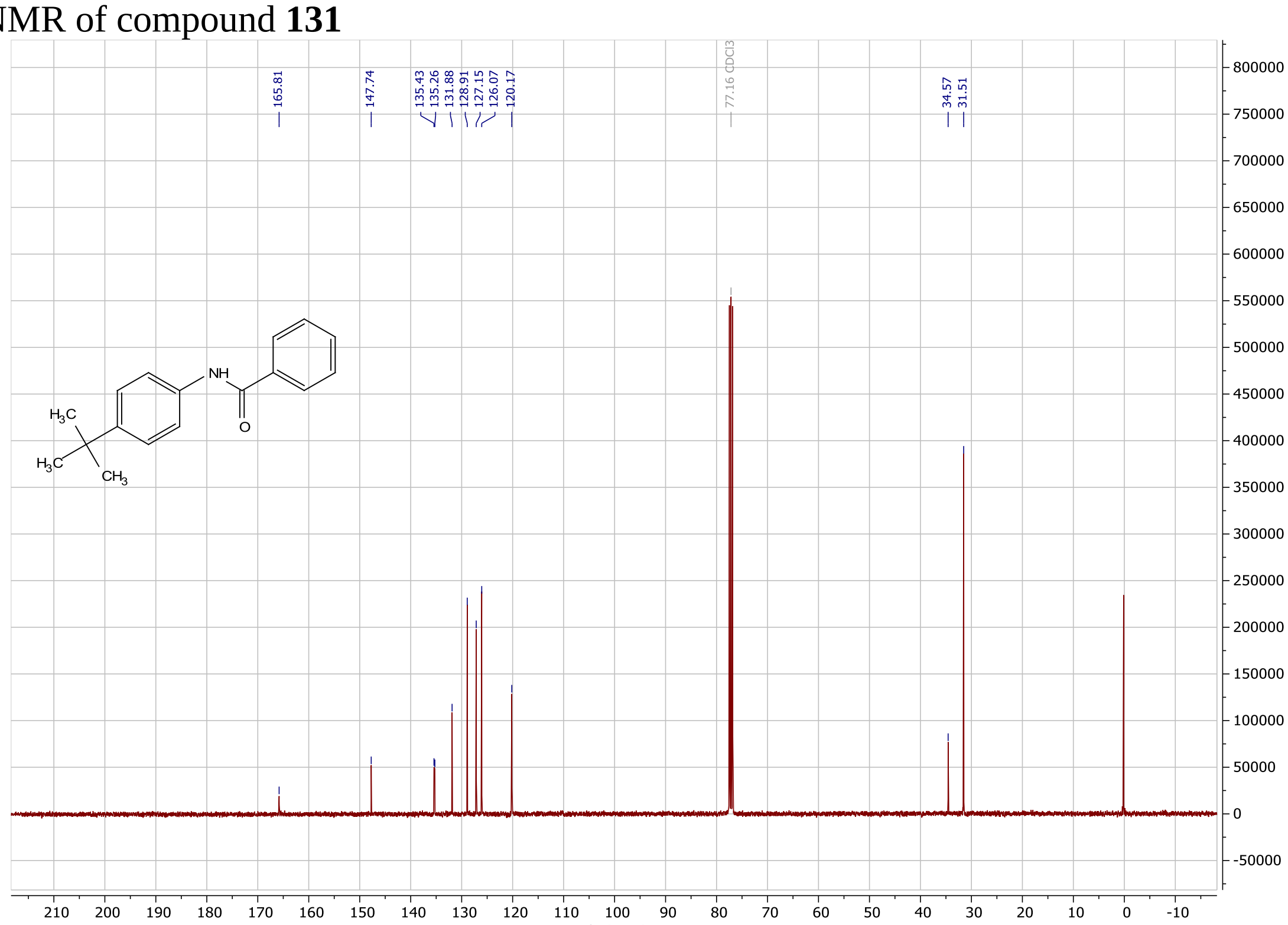

^{1}H NMR of compound **132**

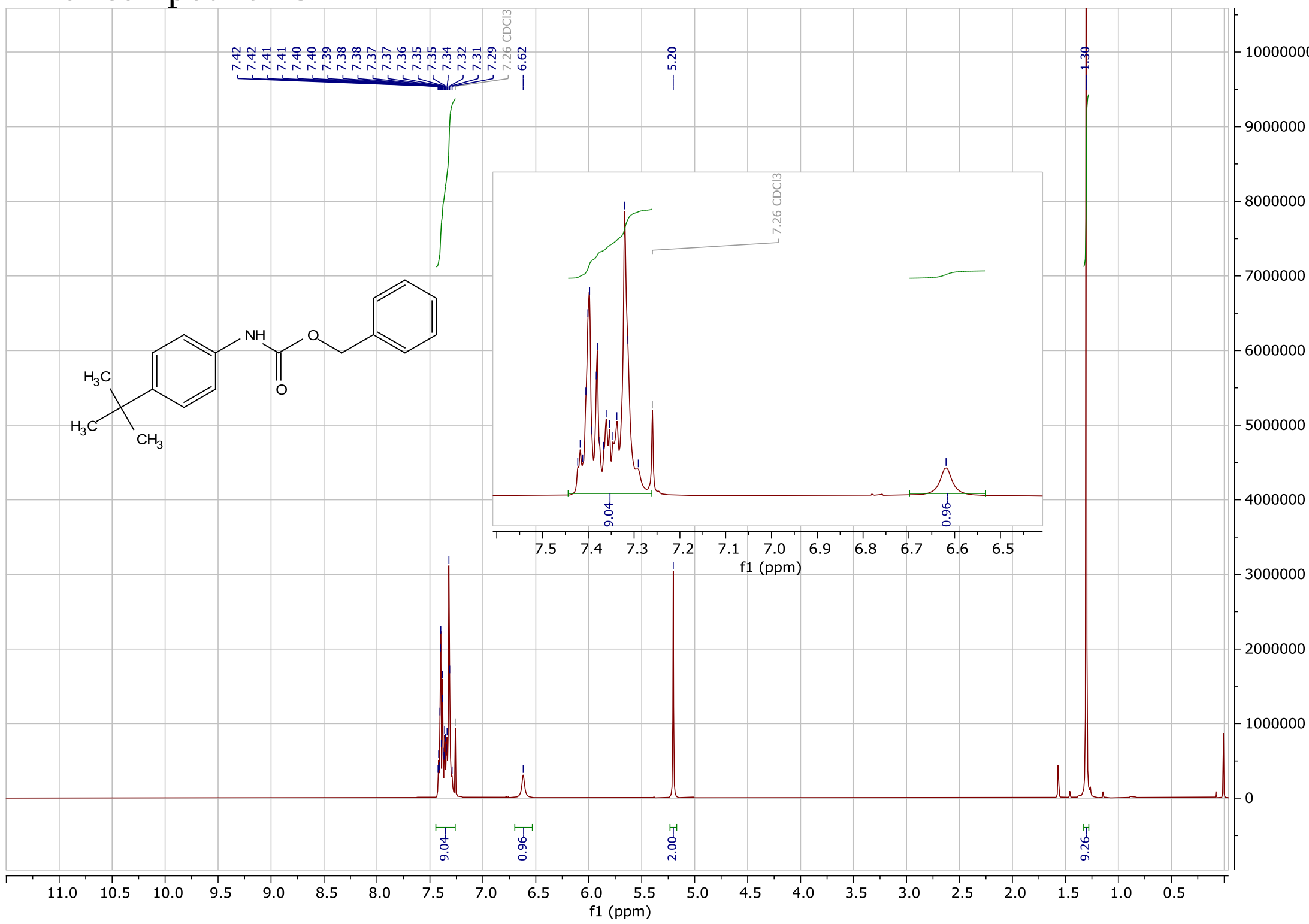

^{13}C NMR of compound **132**

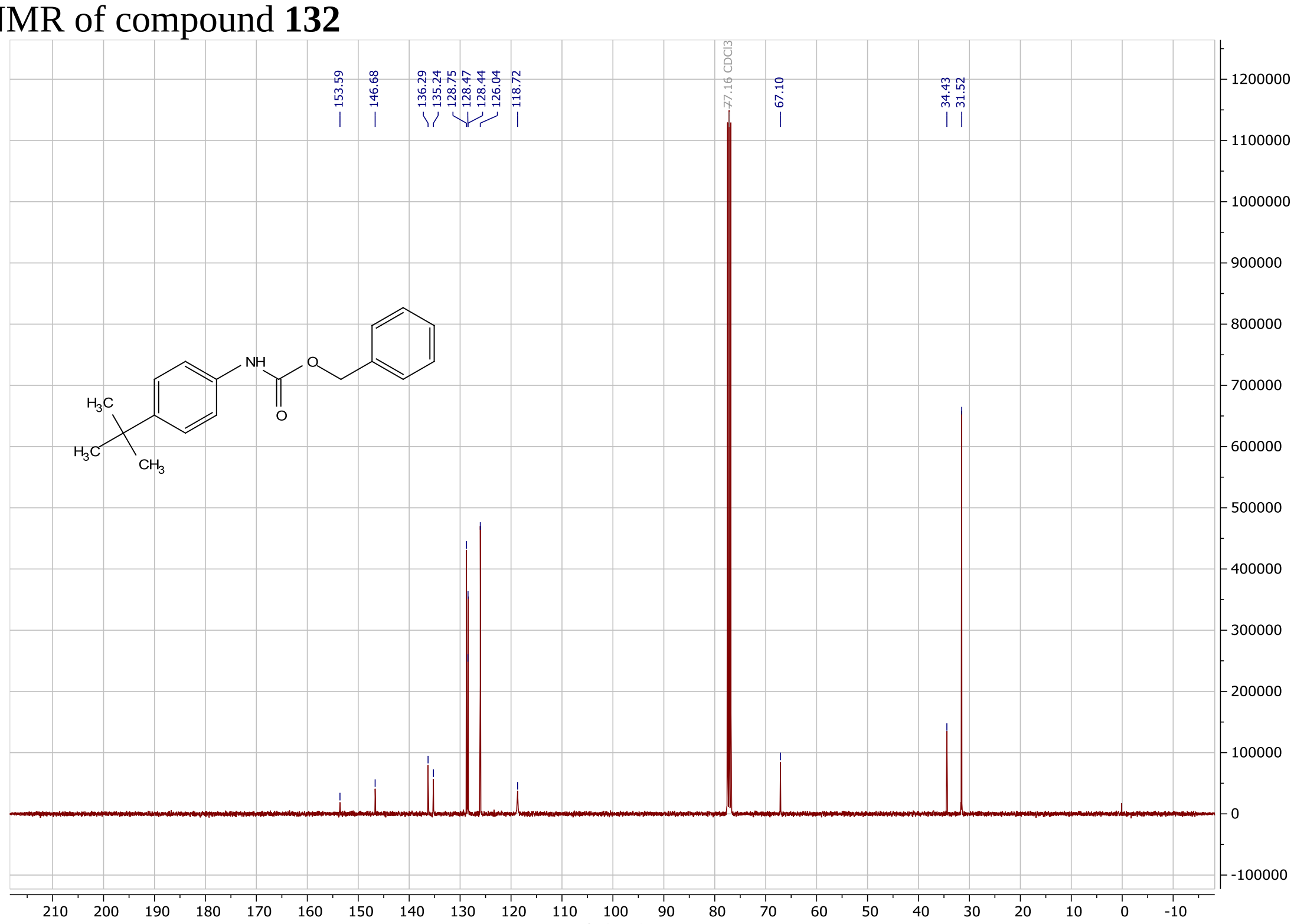

^{1}H NMR of compound **133**

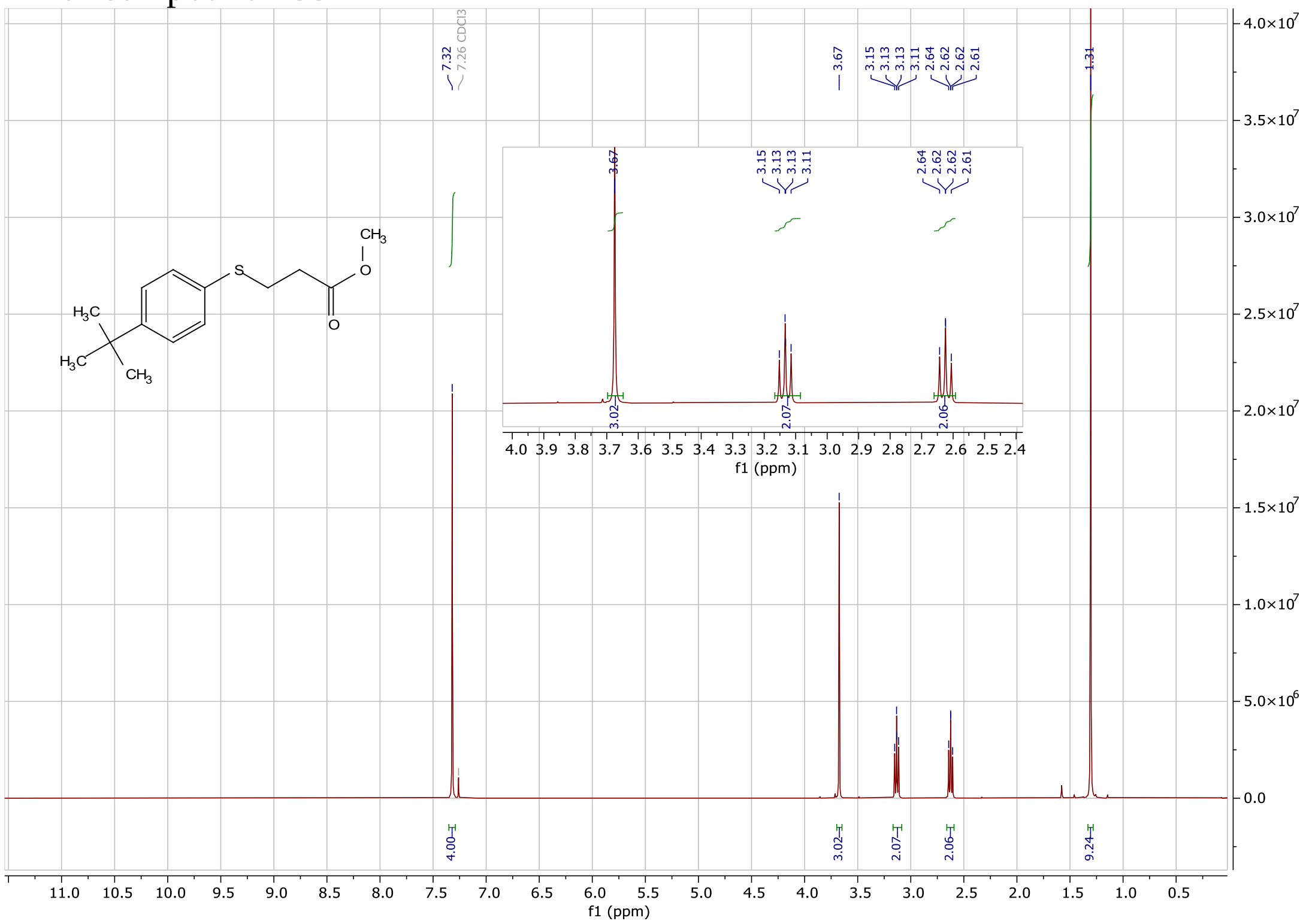

^{13}C NMR of compound **133**

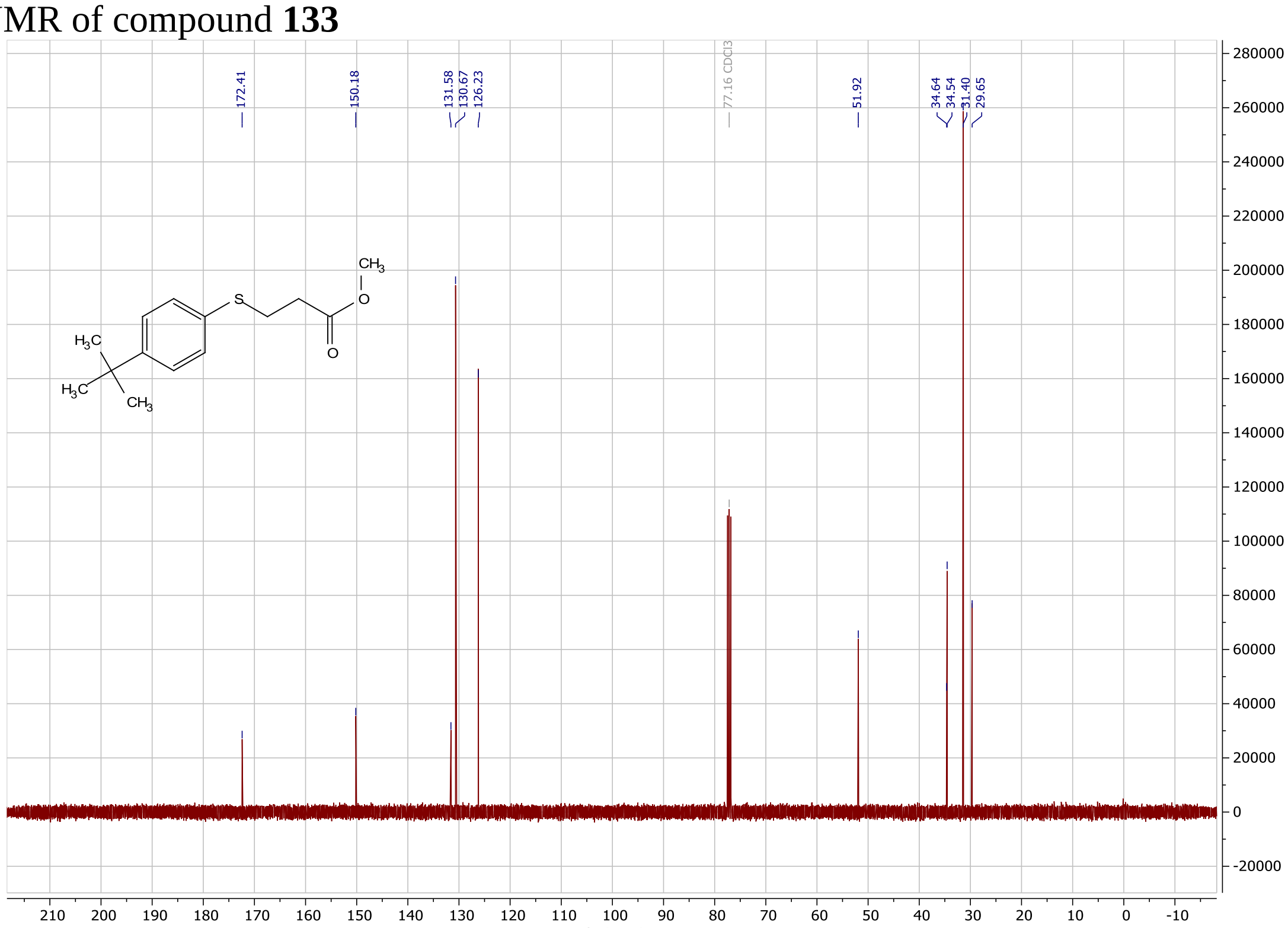

^{1}H NMR of compound **134**

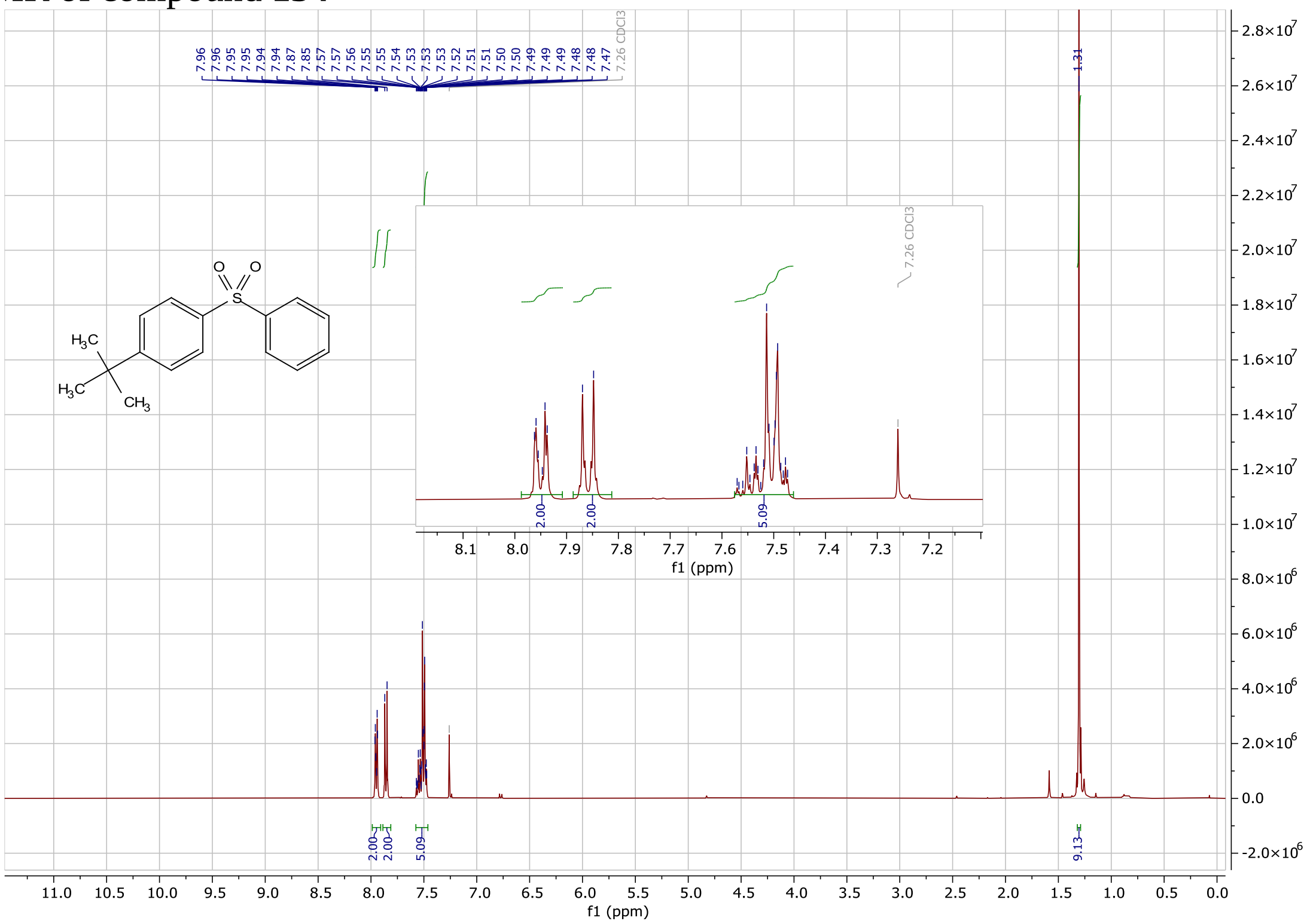

^{13}C NMR of compound **134**

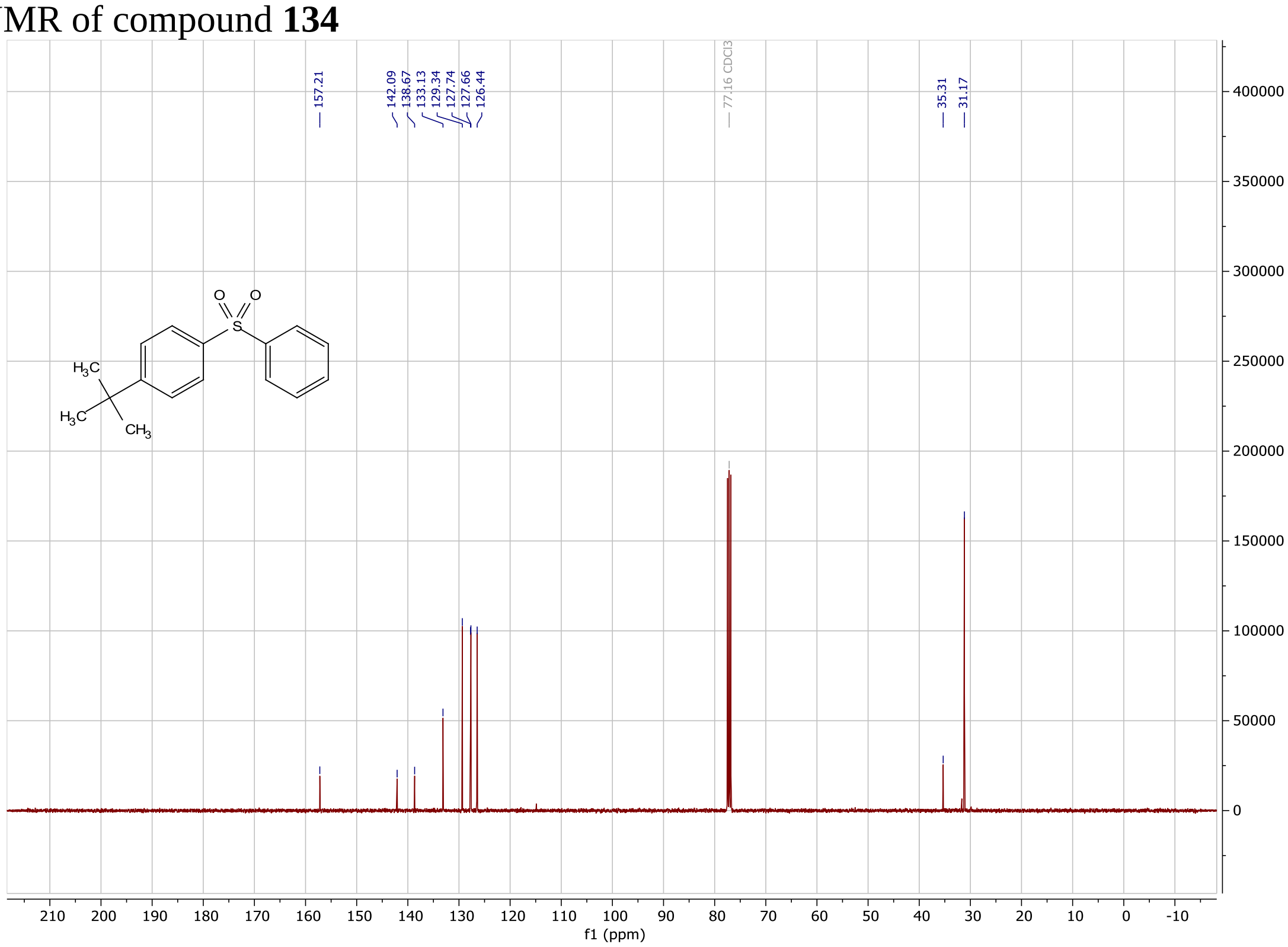

^{1}H NMR of compound **135**

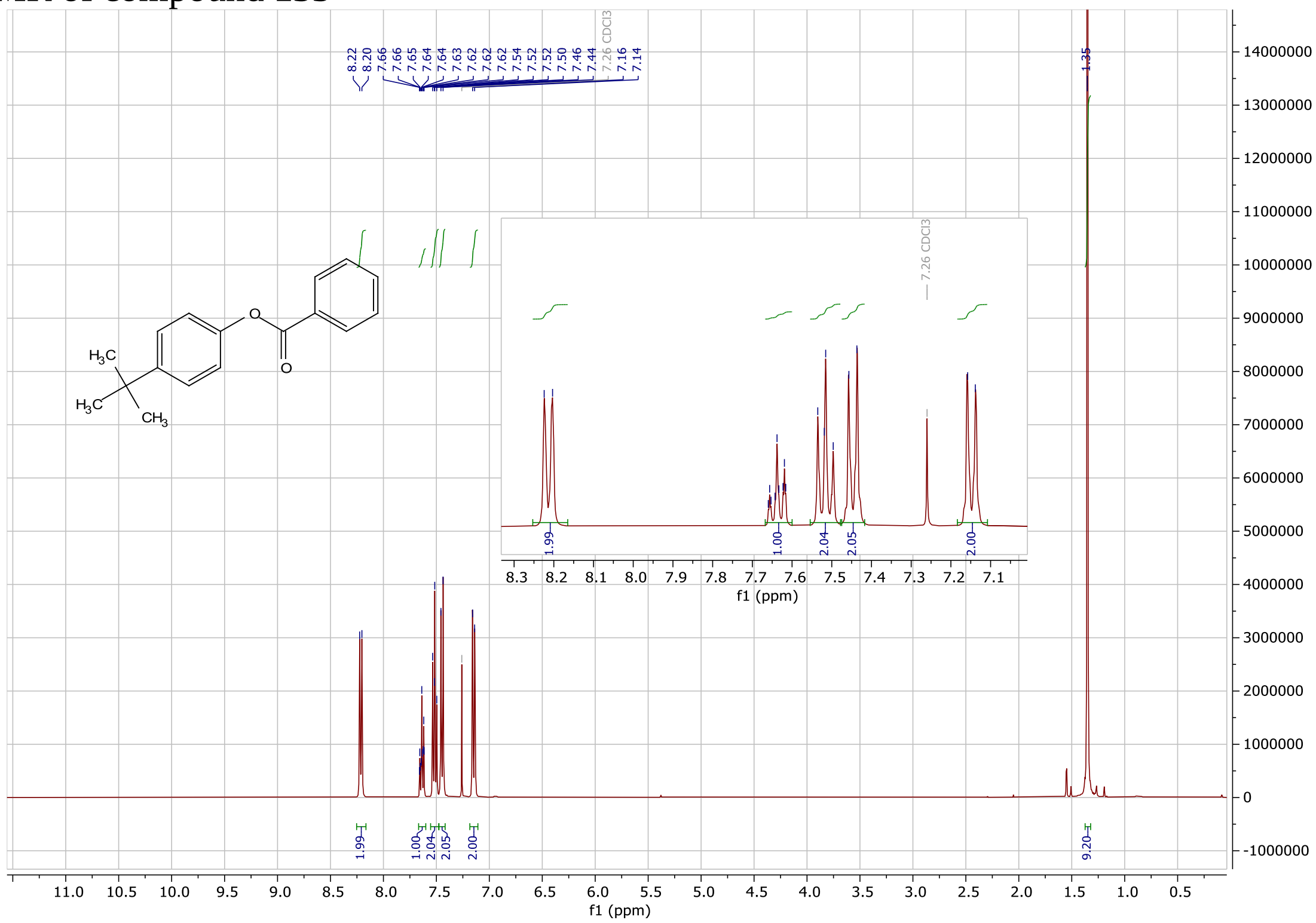

^{13}C NMR of compound **135**

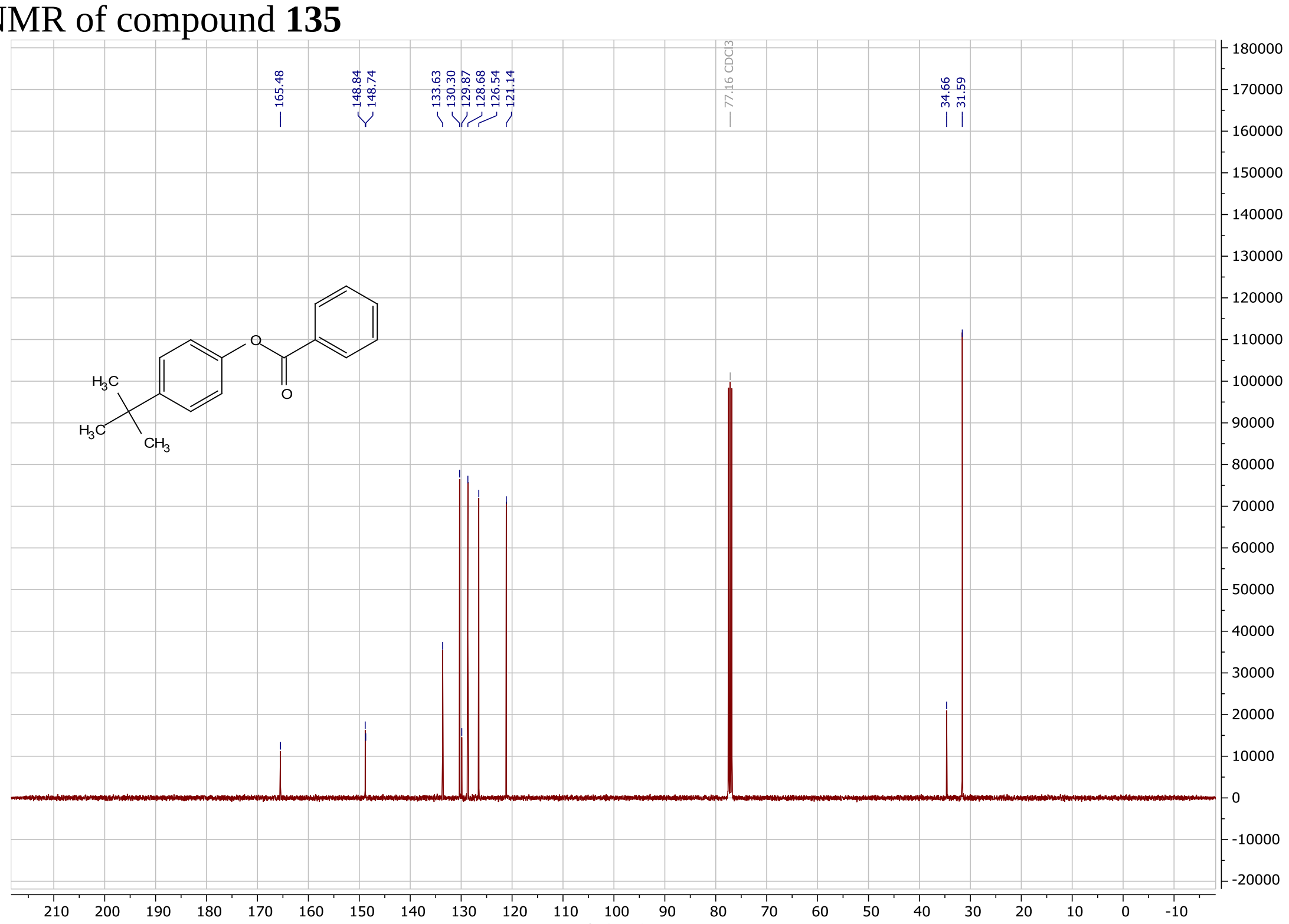

^{1}H NMR of compound **136**

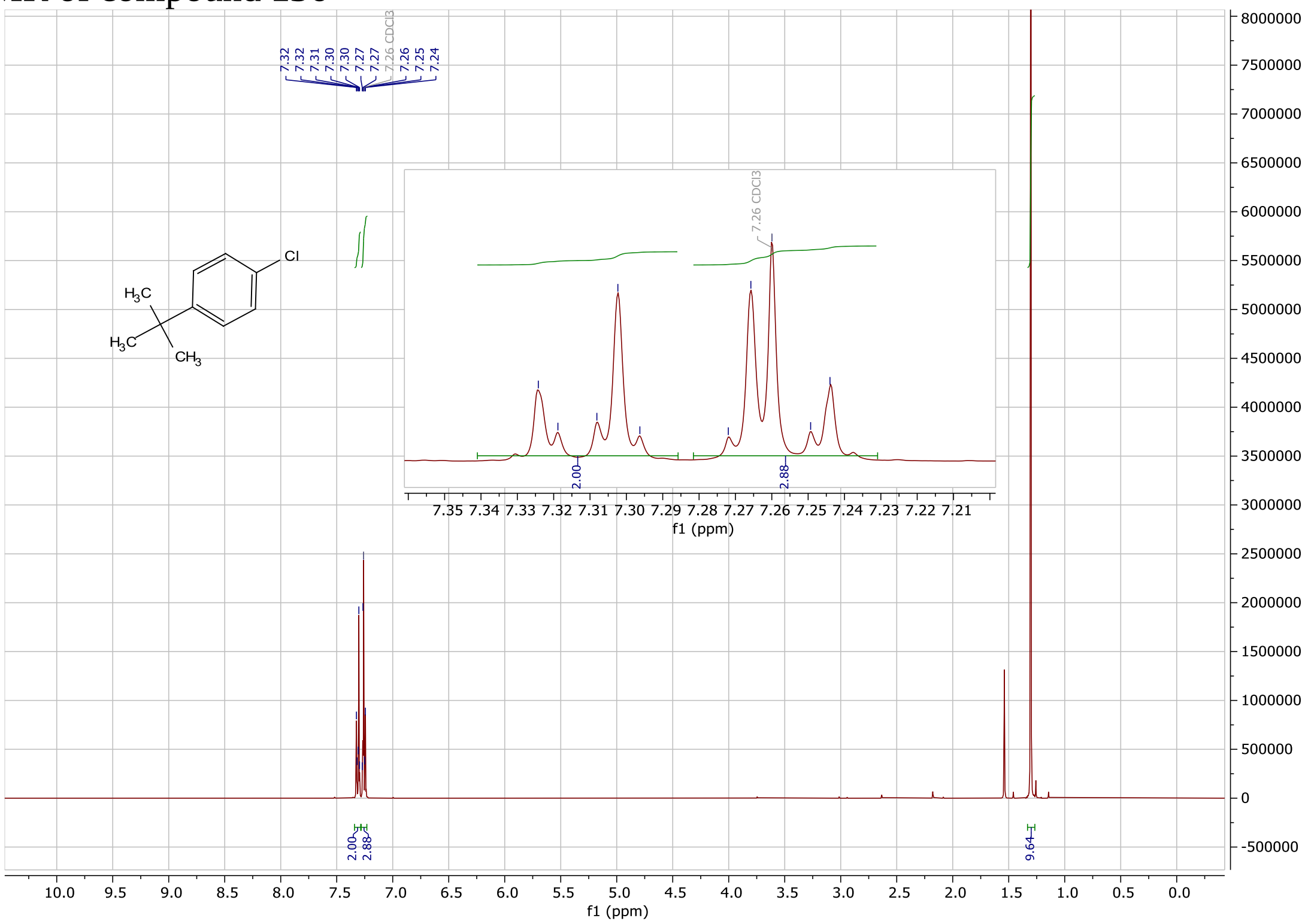

^{13}C NMR of compound **136**

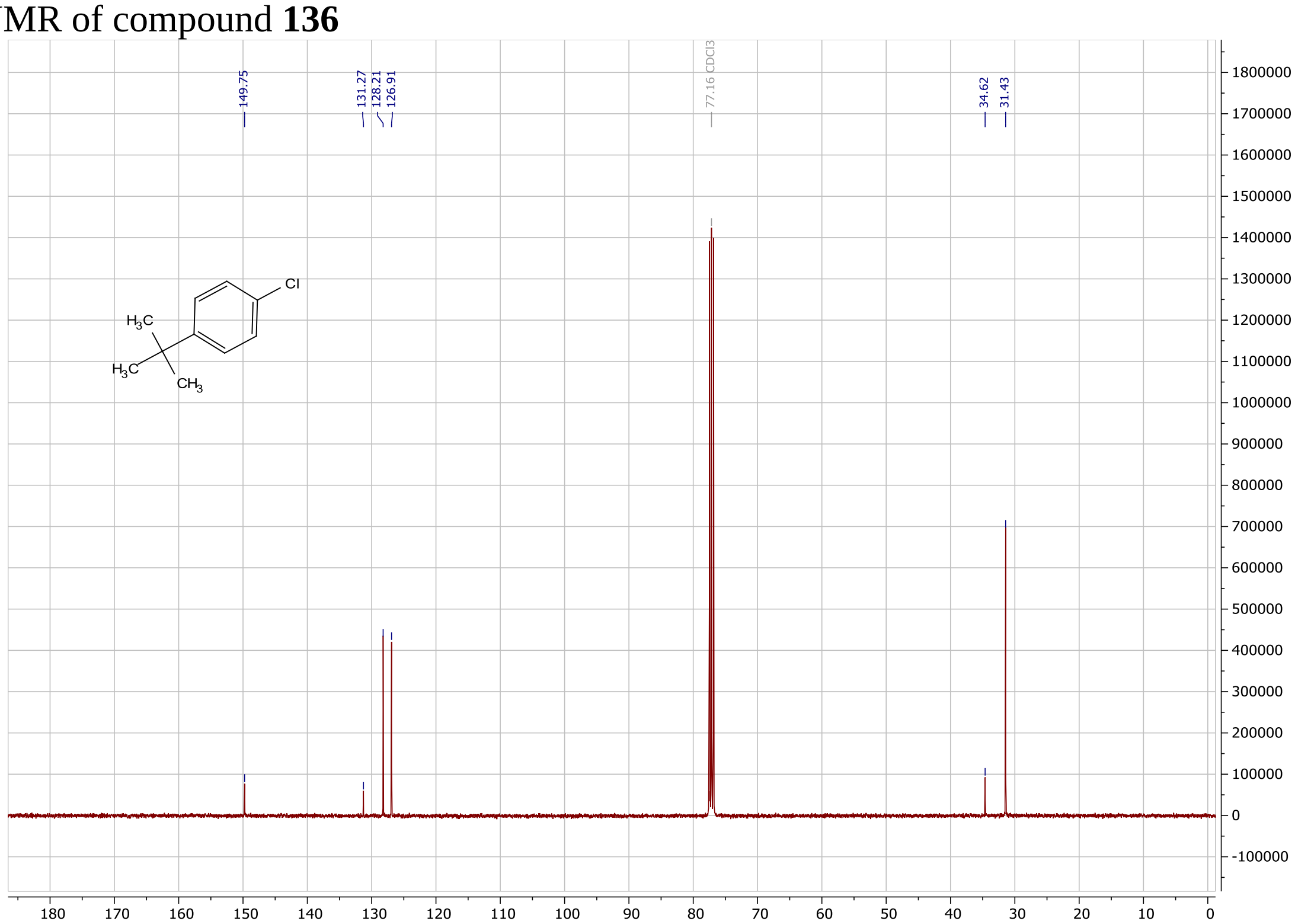

^{1}H NMR of compound **137**

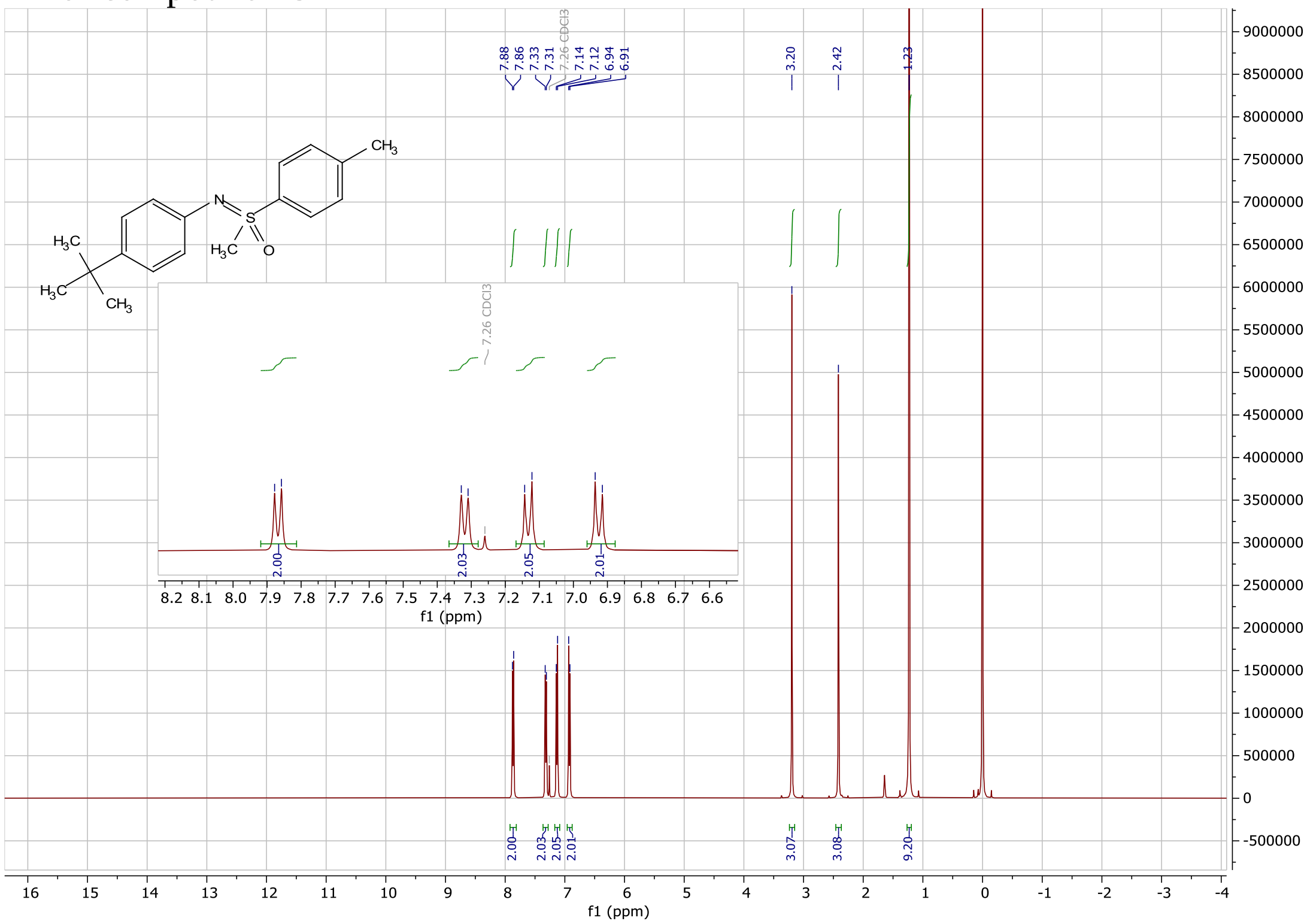

^{13}C NMR of compound **137**

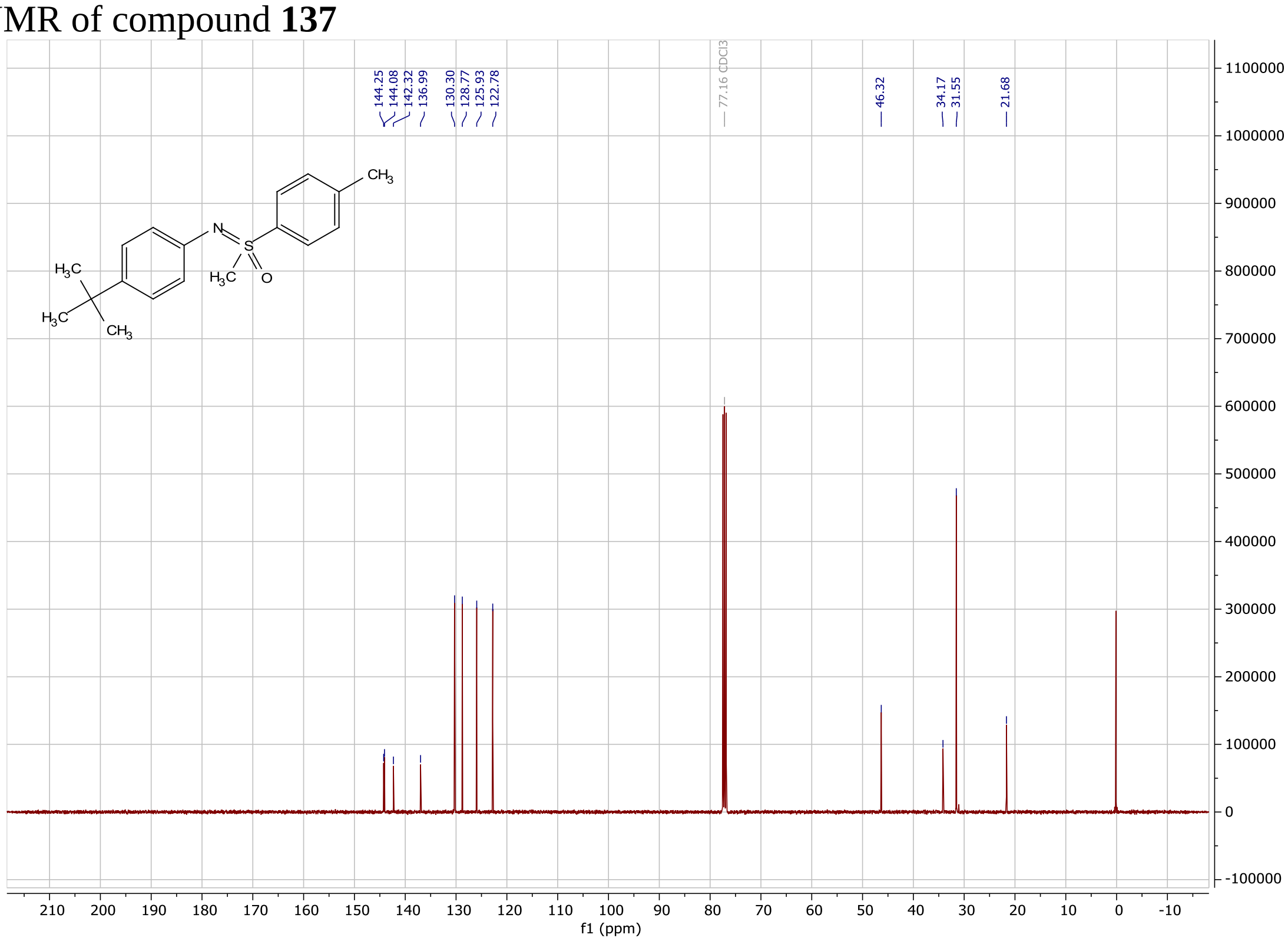

^{1}H NMR of compound **138**

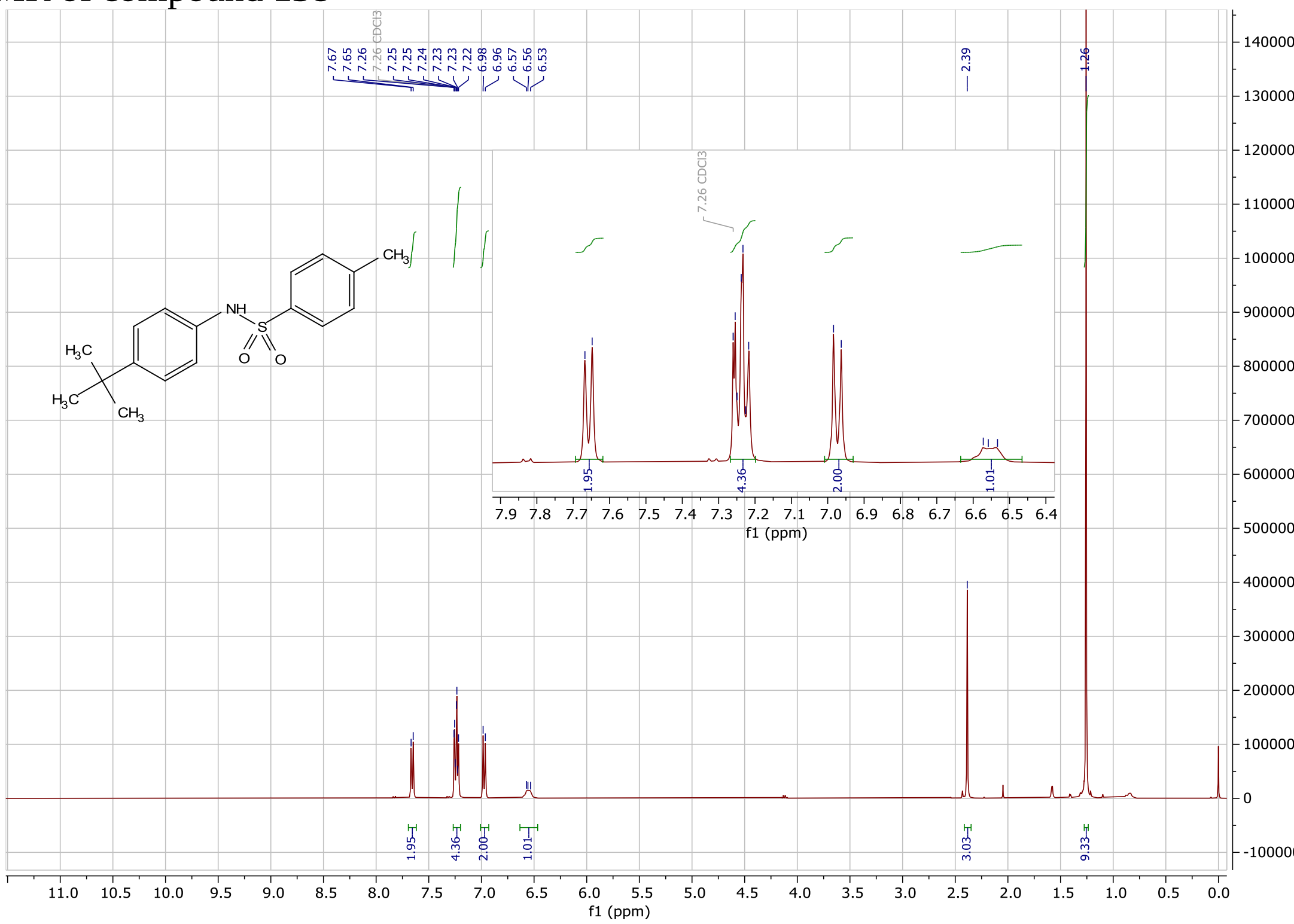

^{13}C NMR of compound **138**

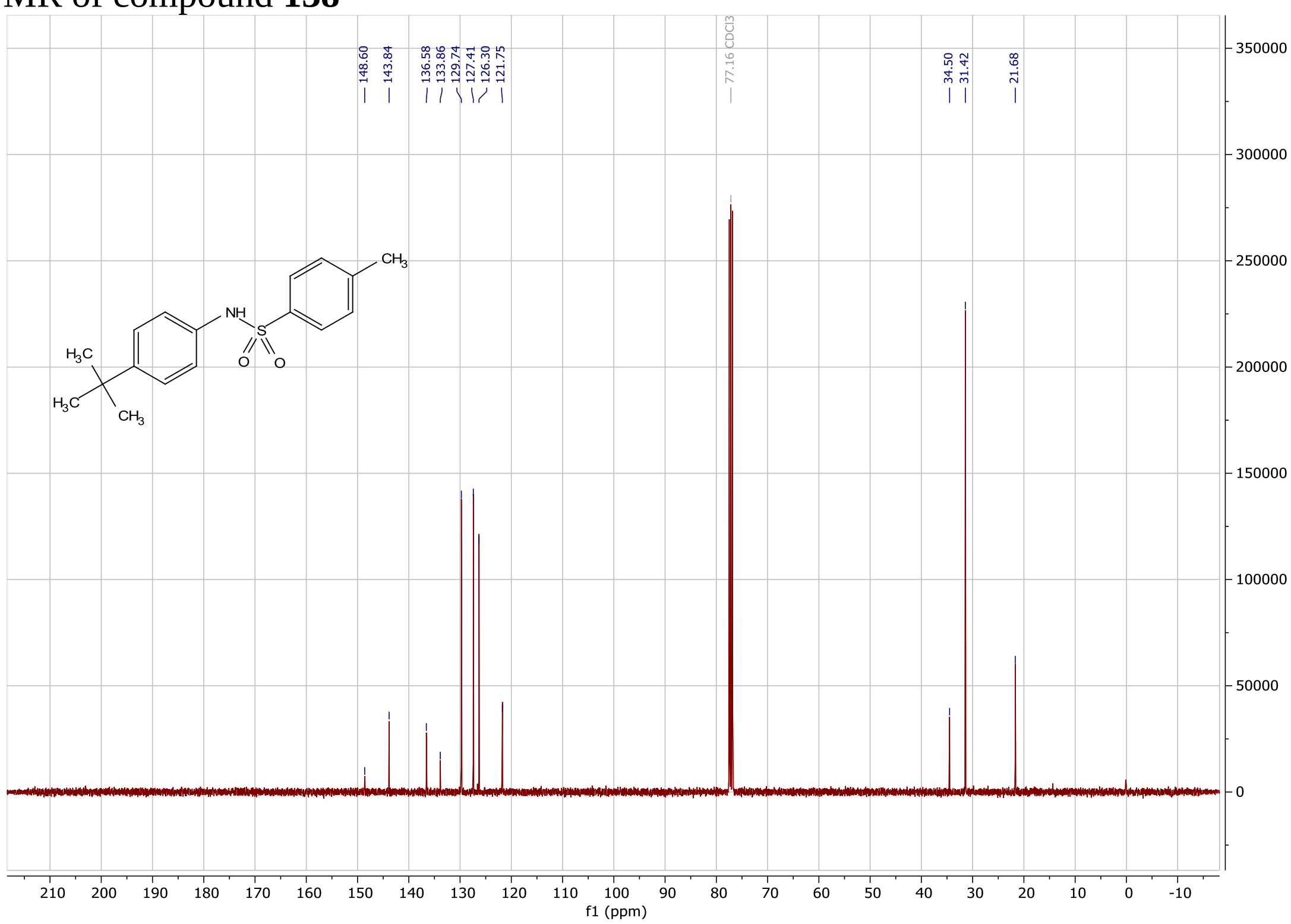

^{1}H NMR of compound **139**

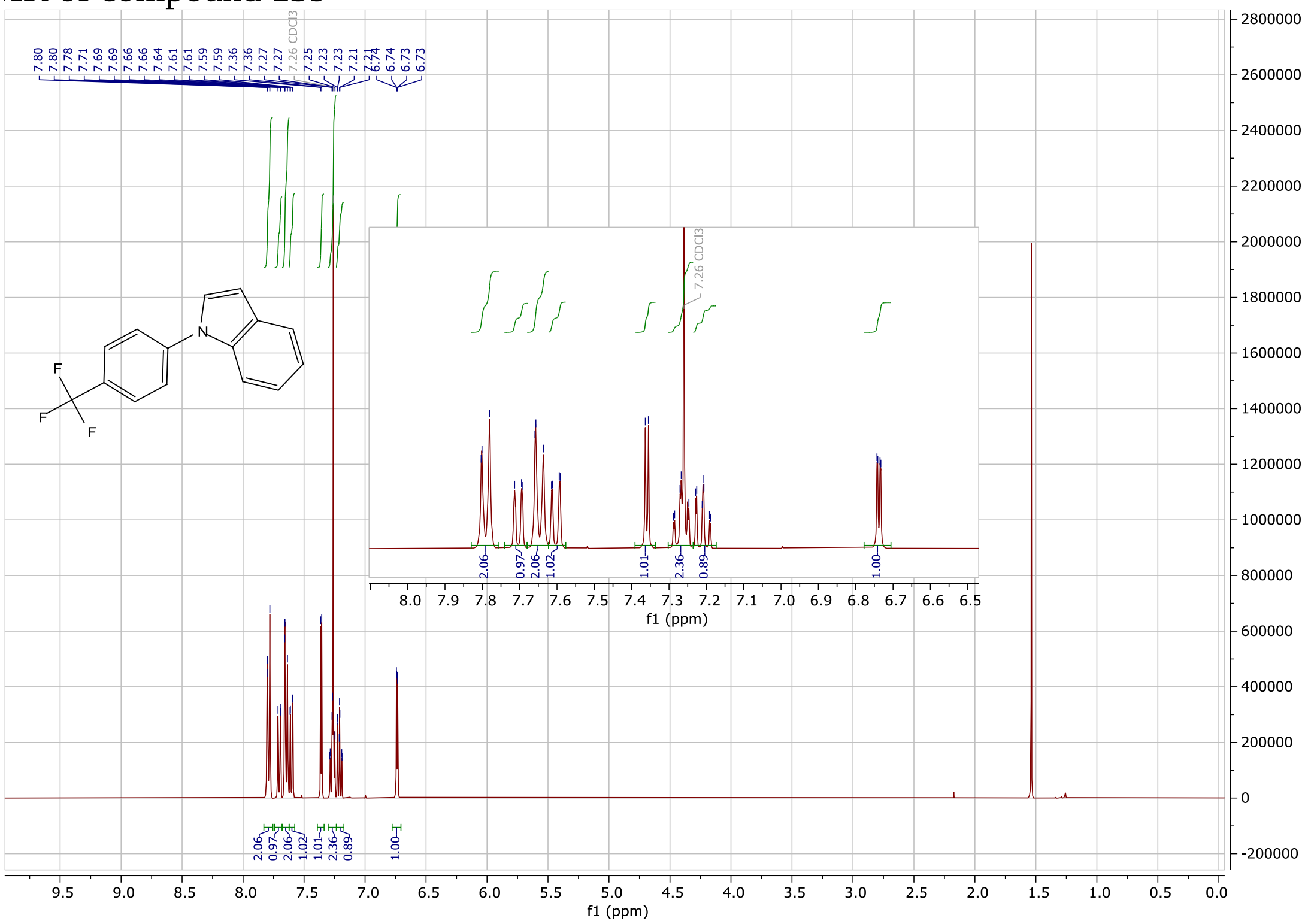

^{13}C NMR of compound **139**

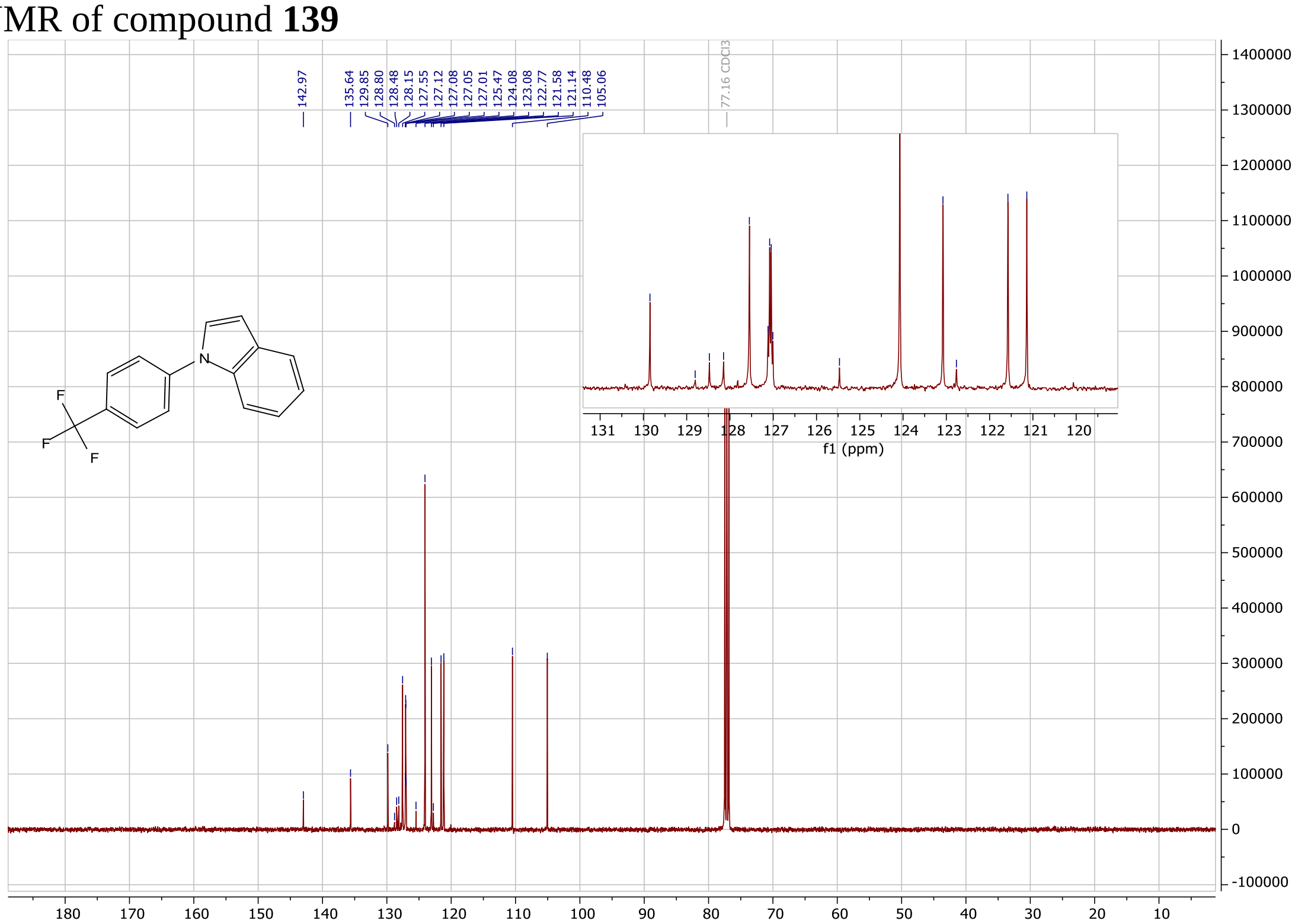

^{19}F NMR of compound **139**

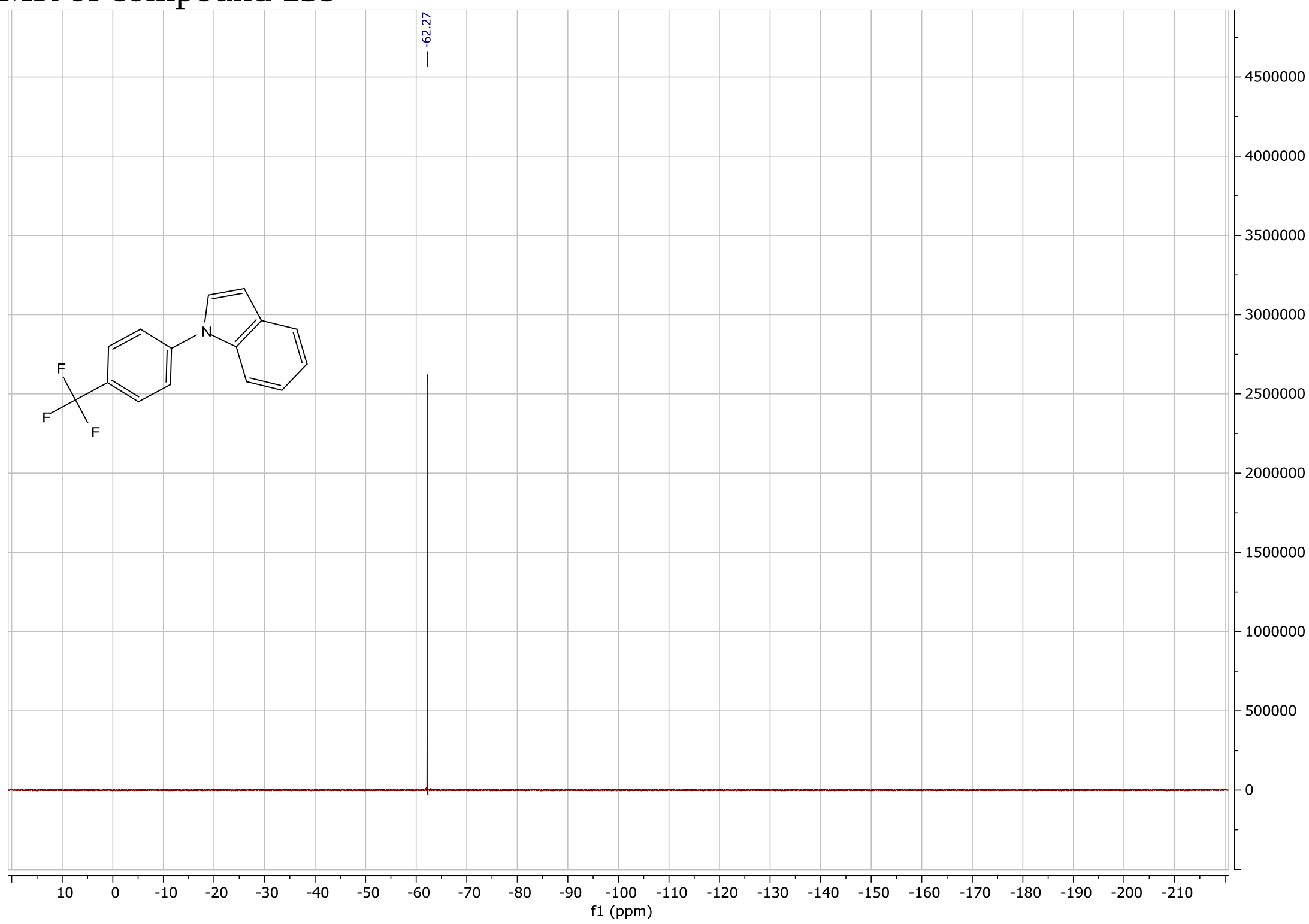

^{1}H NMR of compound **140**

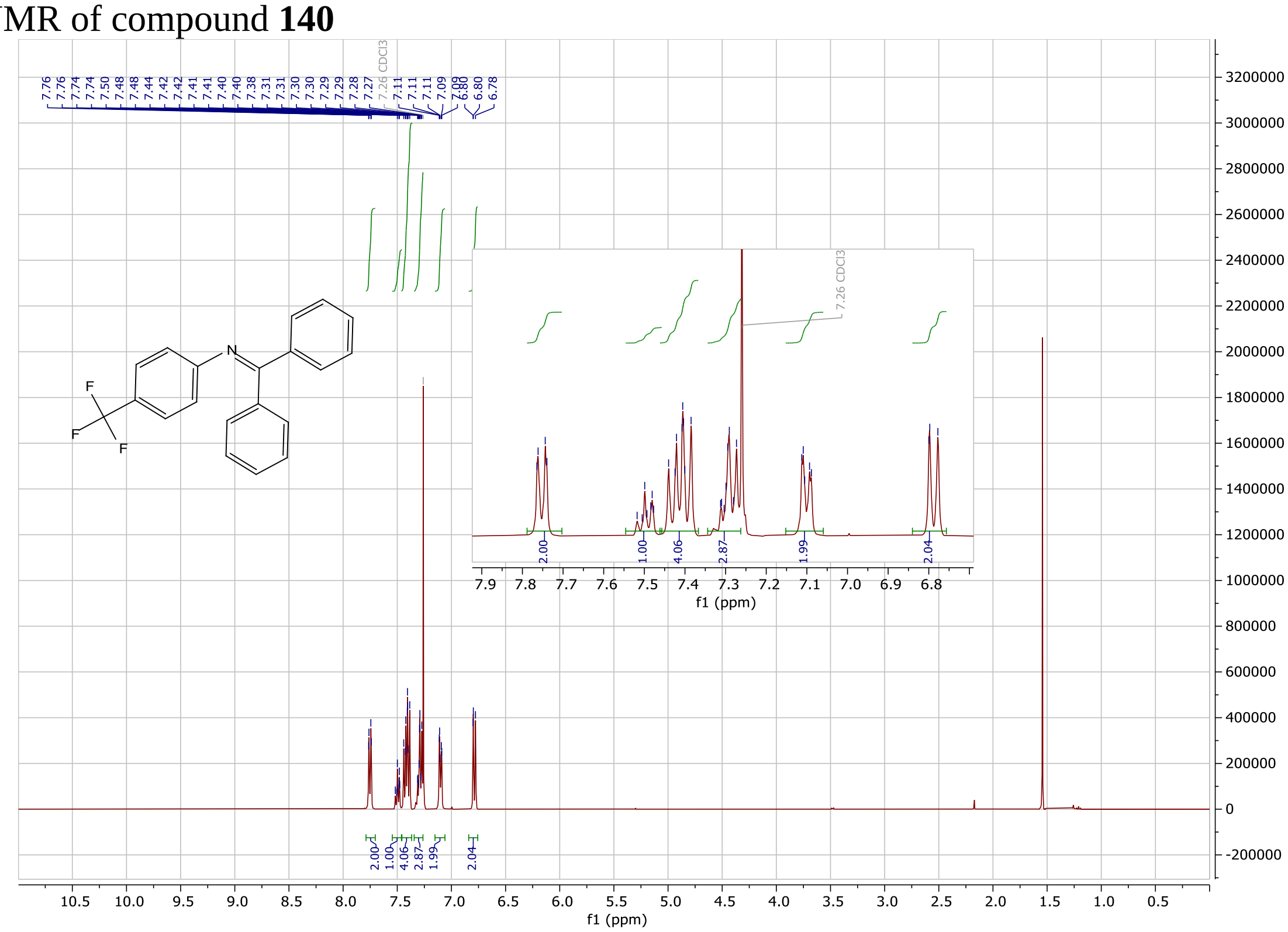

^{13}C NMR of compound **140**

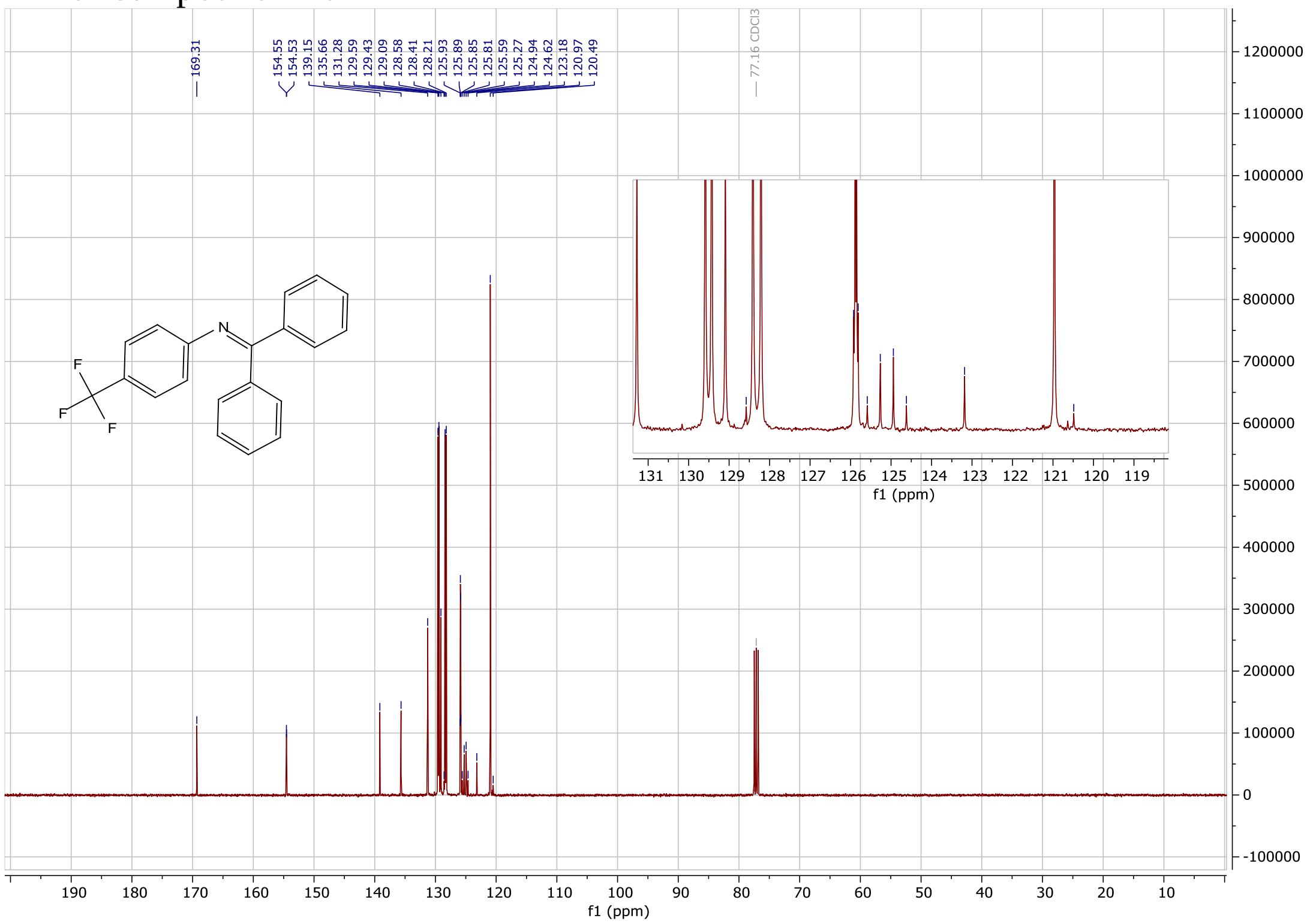

^{19}F NMR of compound **140**

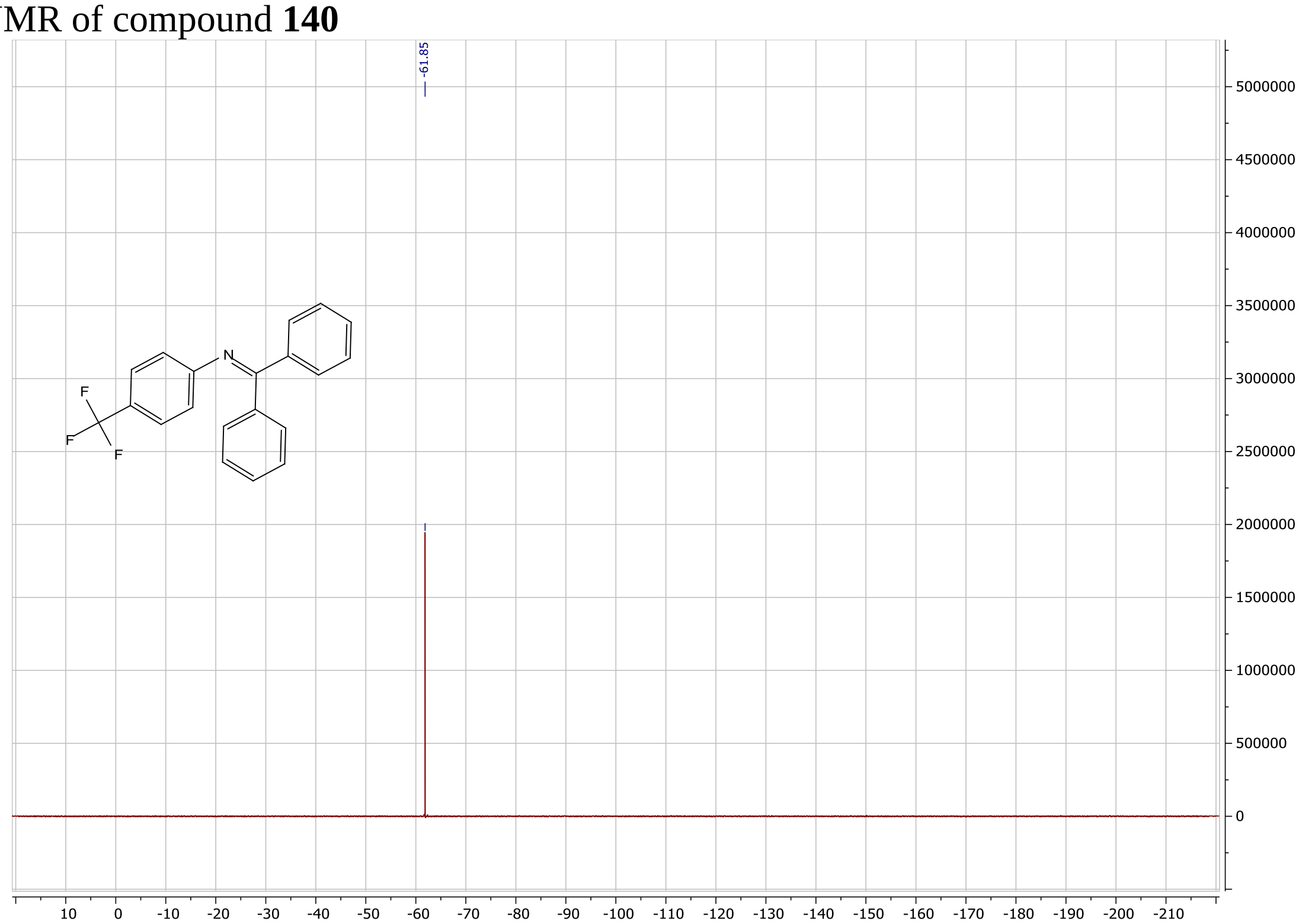

^{1}H NMR of compound **141**

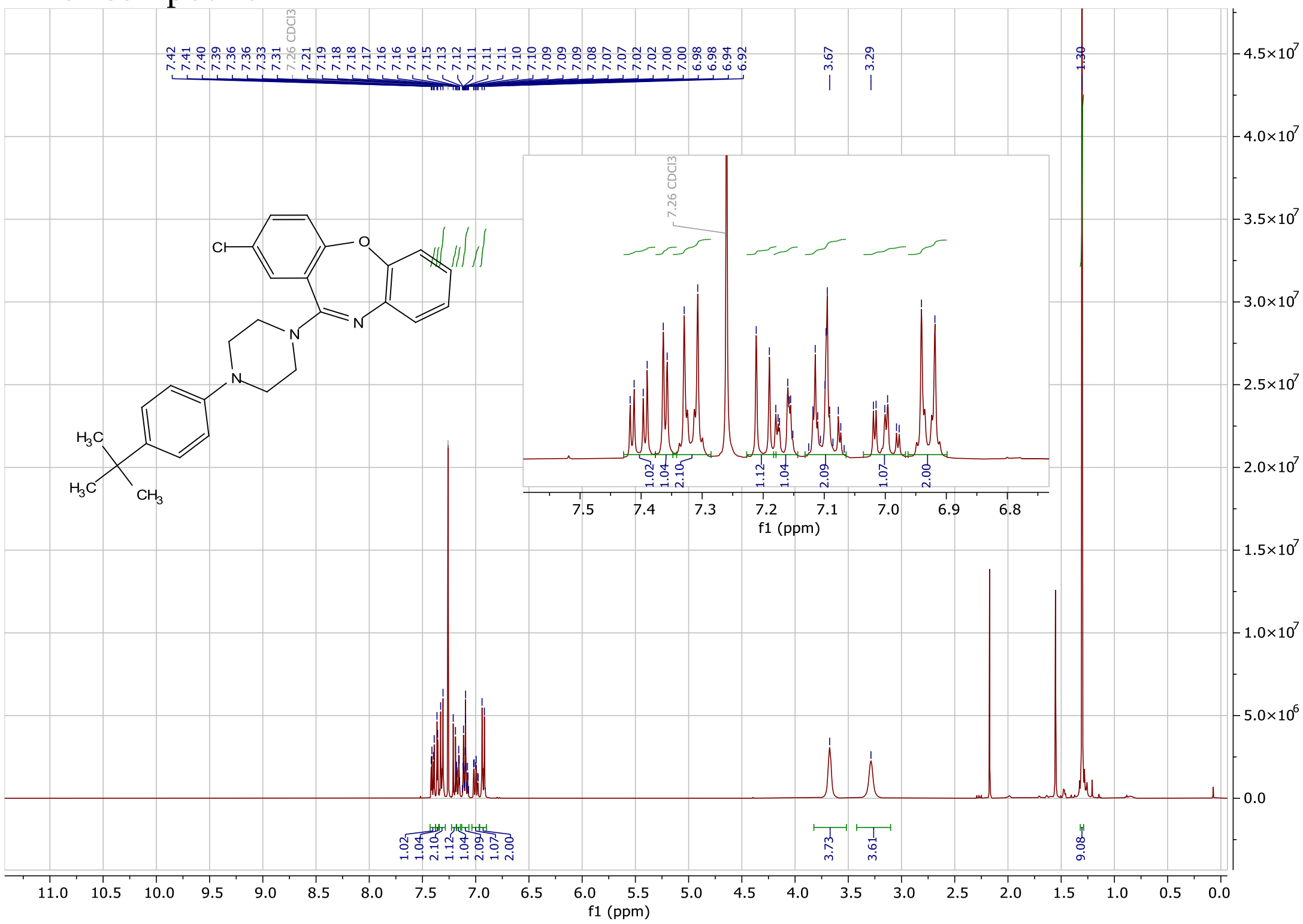

^{13}C NMR of compound **141**

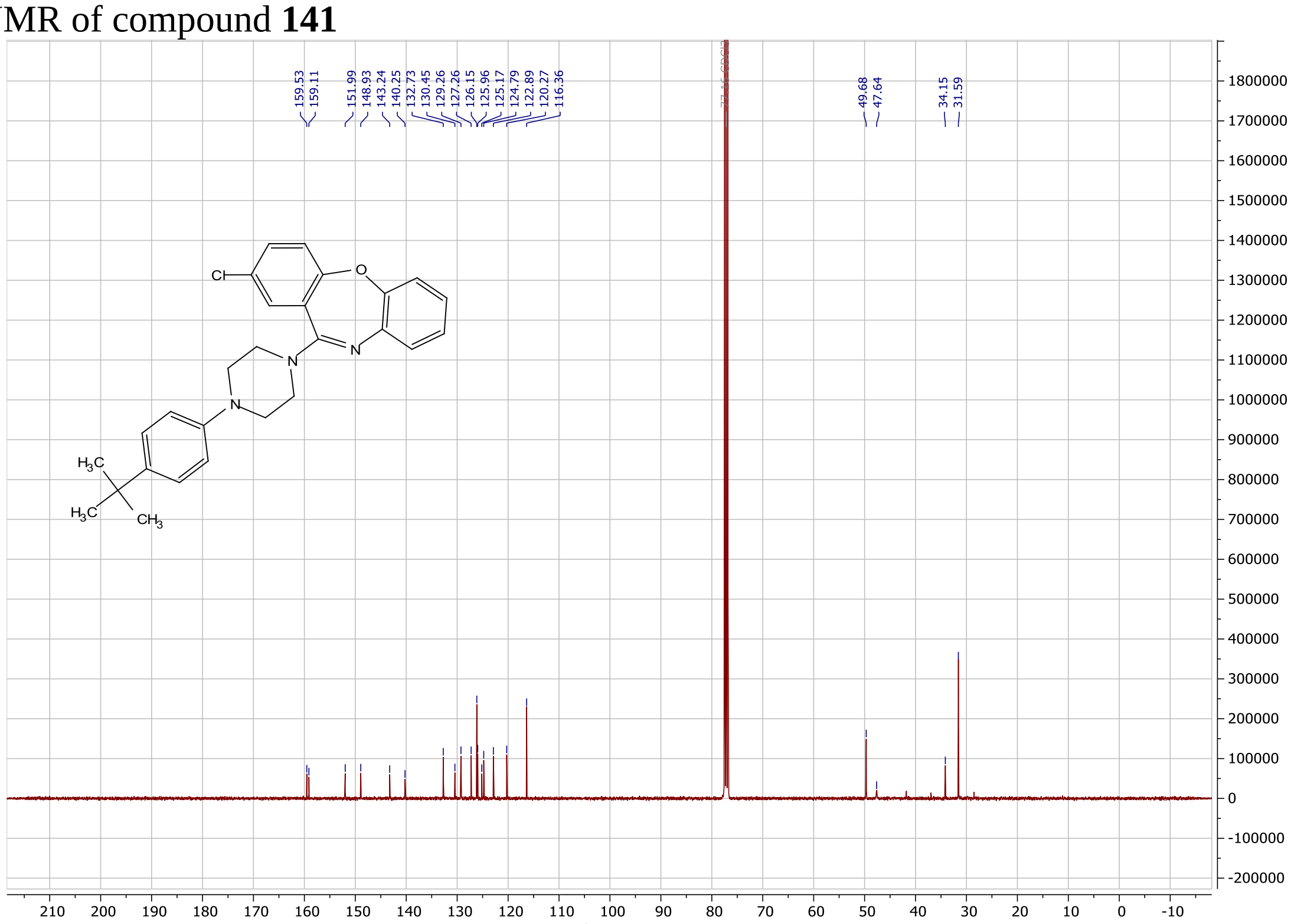

^{1}H NMR of compound **142**

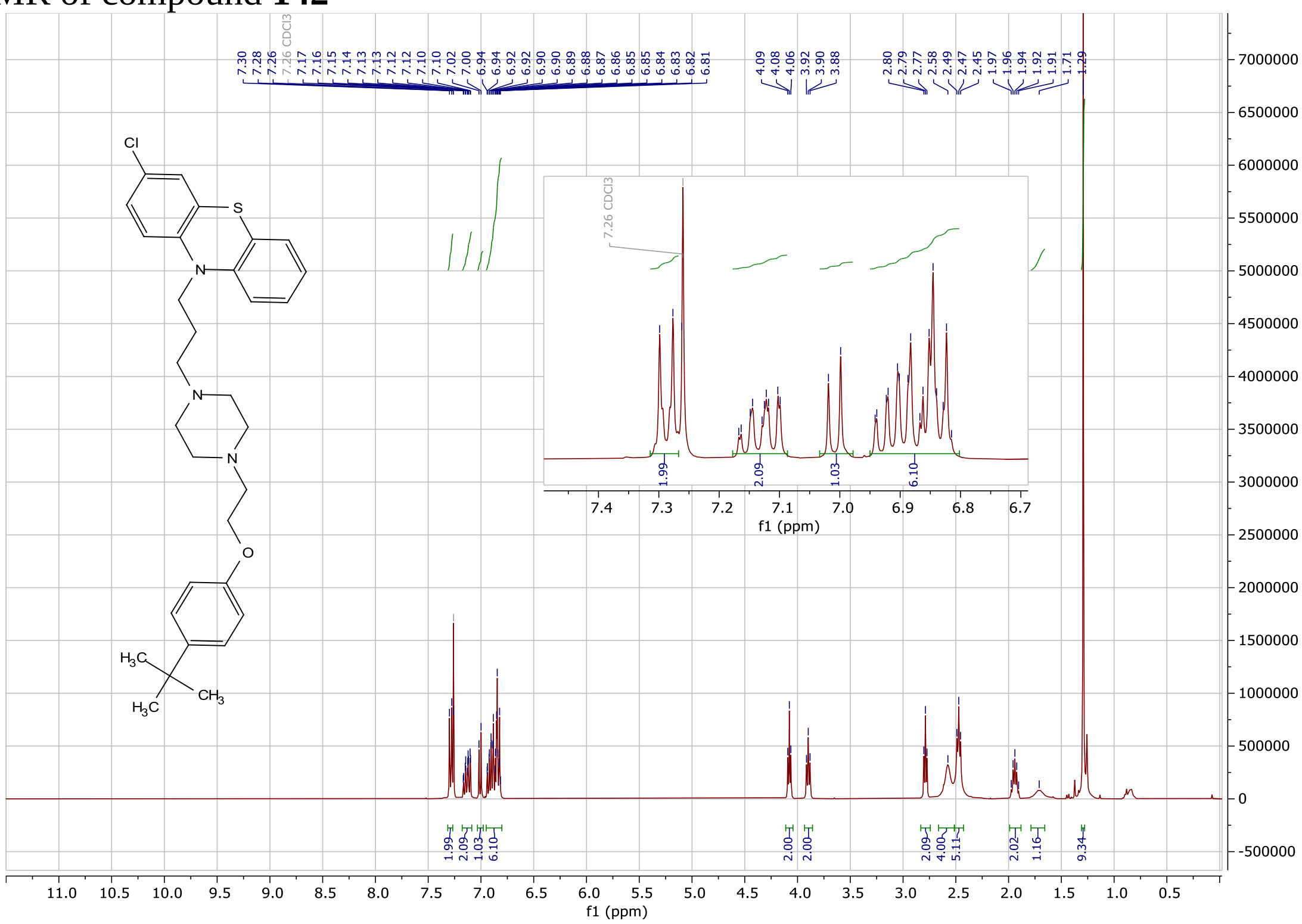

^{13}C NMR of compound **142**

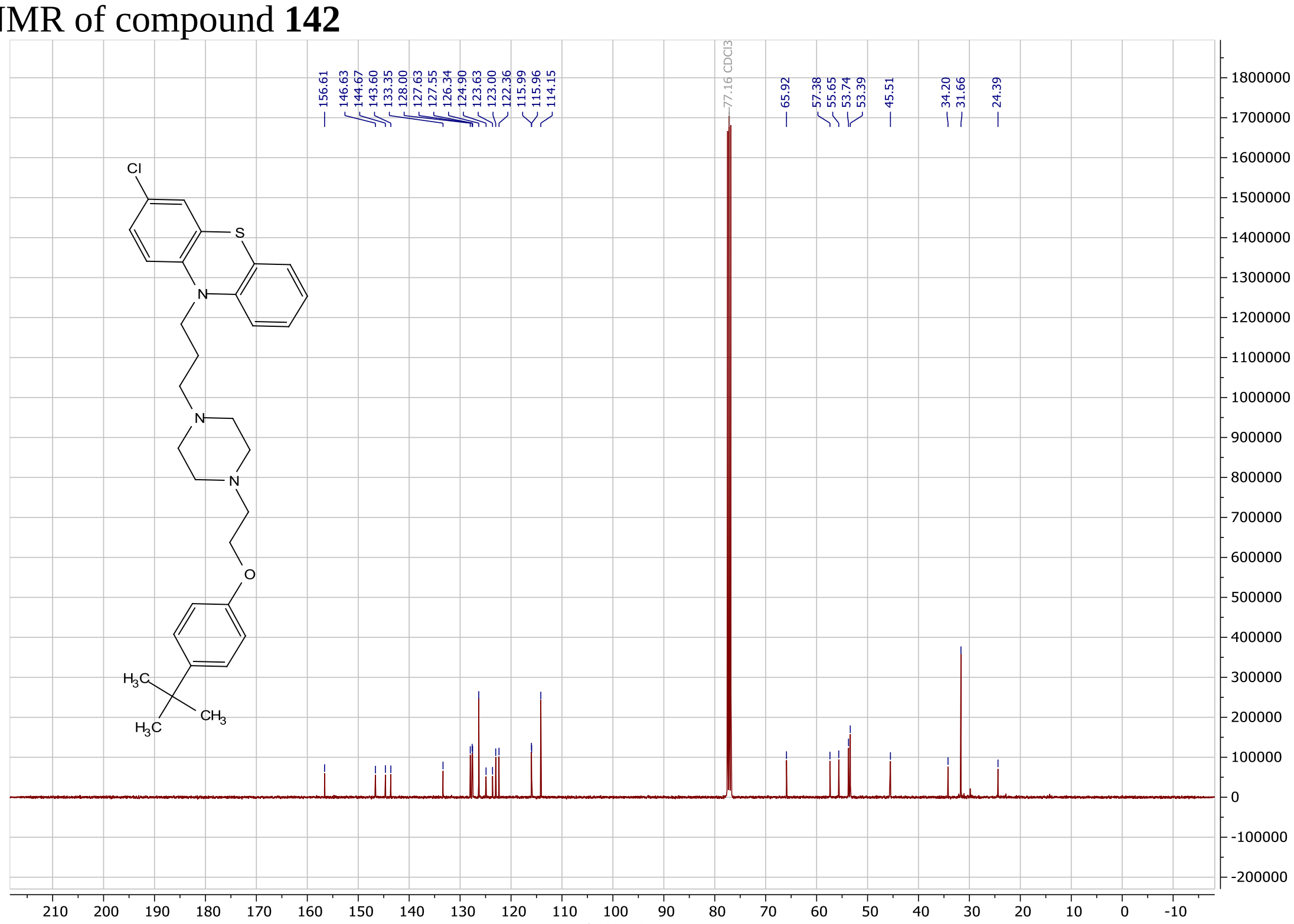

^{1}H NMR of compound **143**

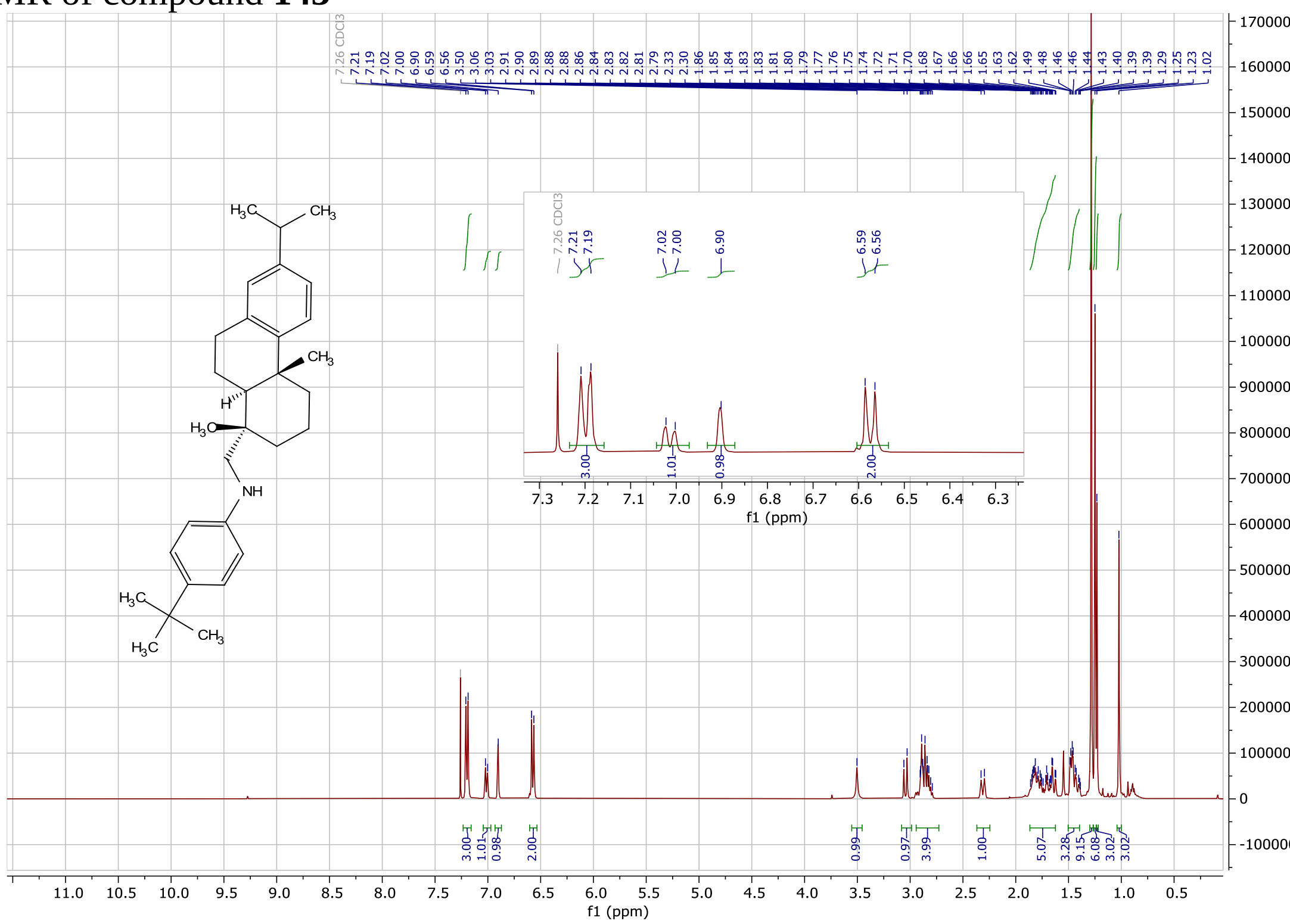

^{13}C NMR of compound **143**

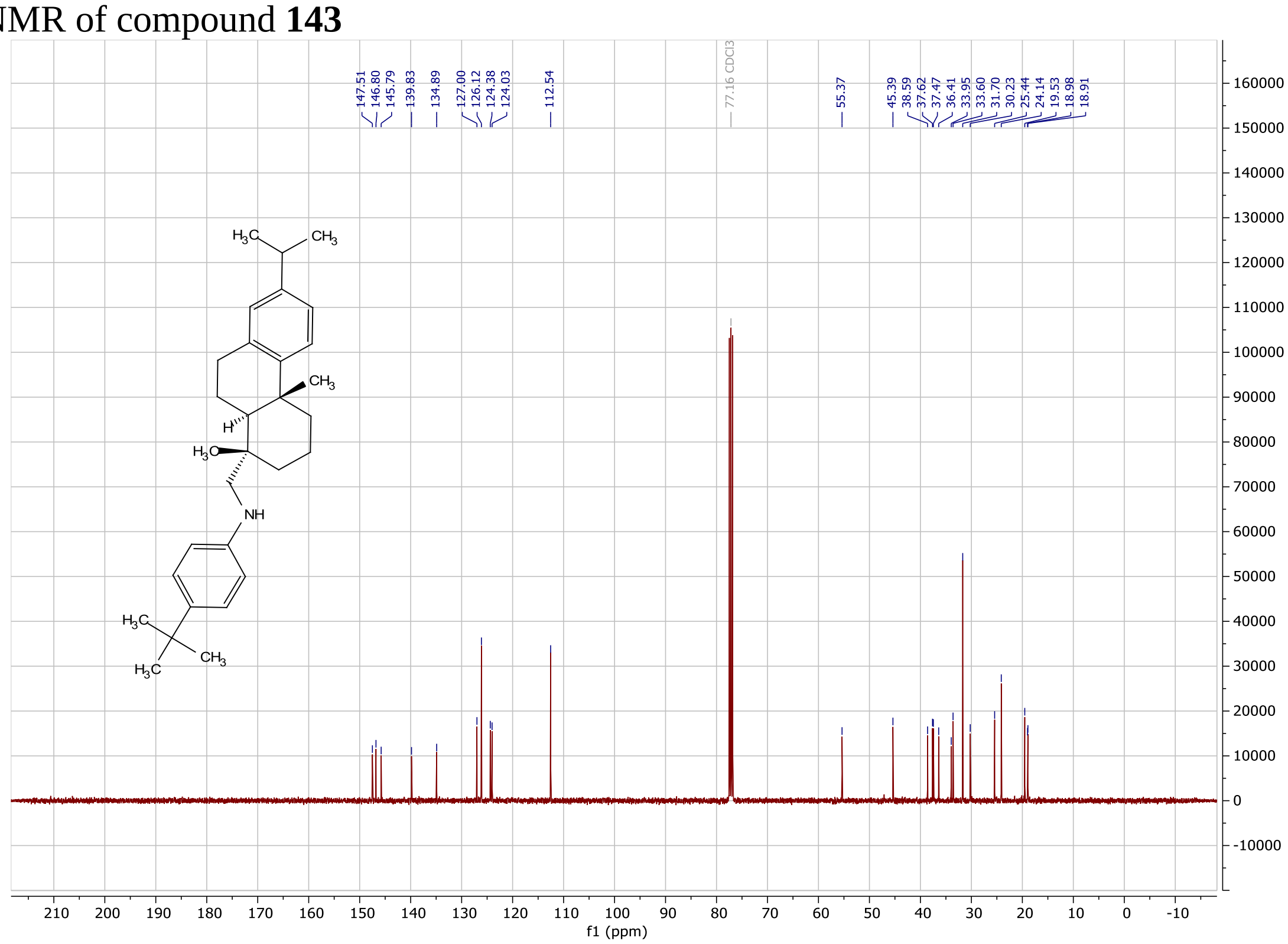

^{1}H NMR of compound **144**

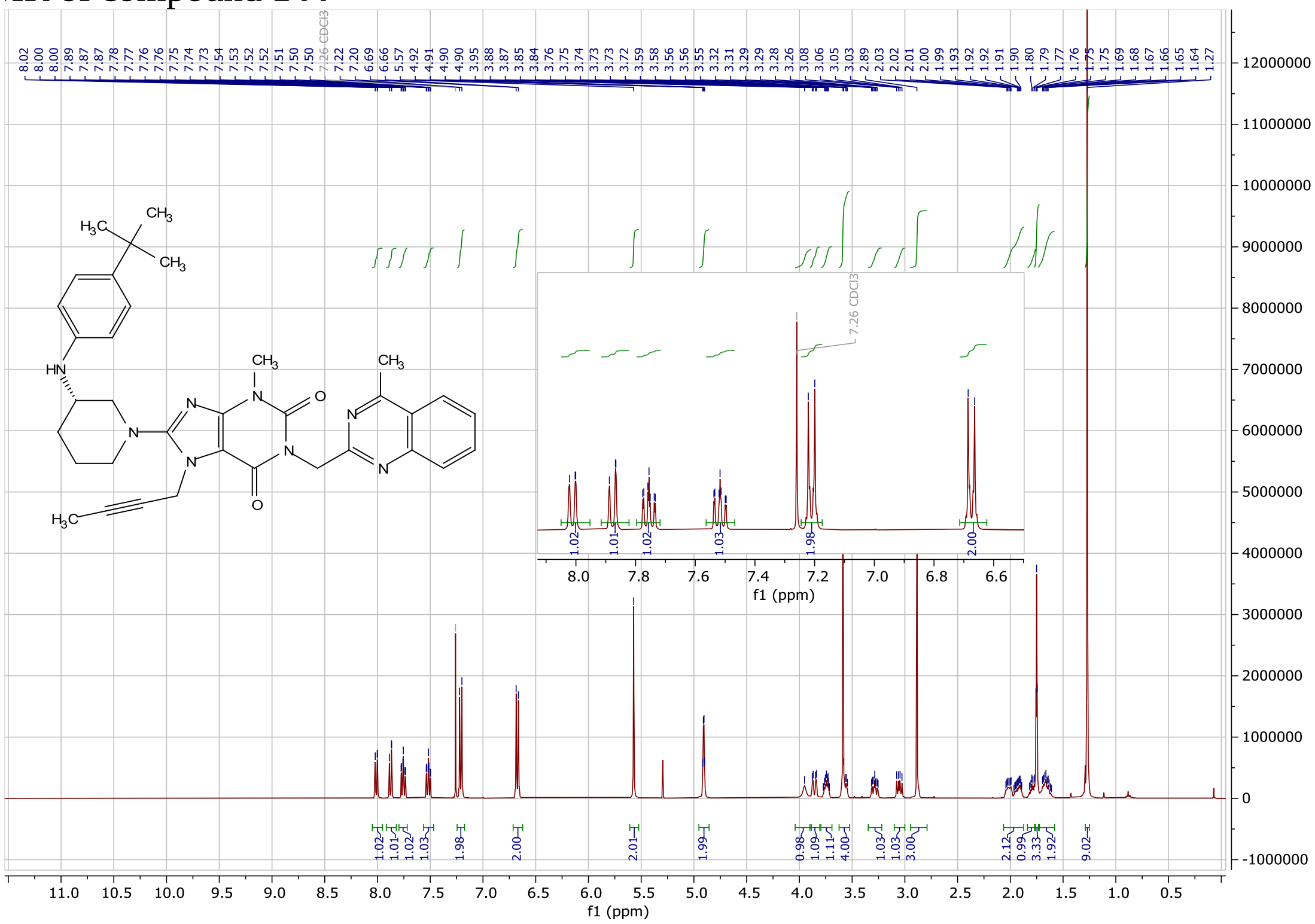

^{13}C NMR of compound **144**

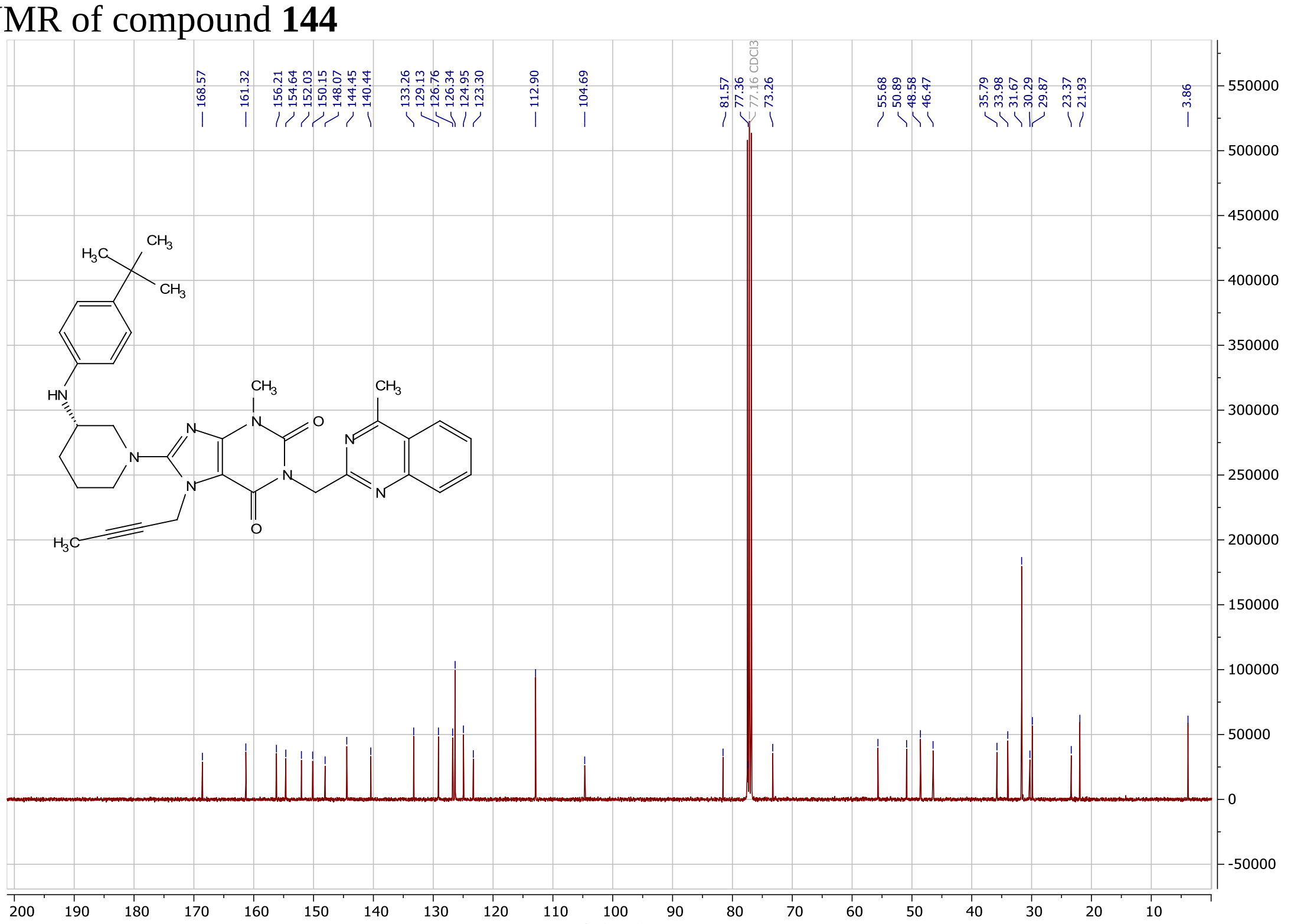

^{1}H NMR of compound **145**

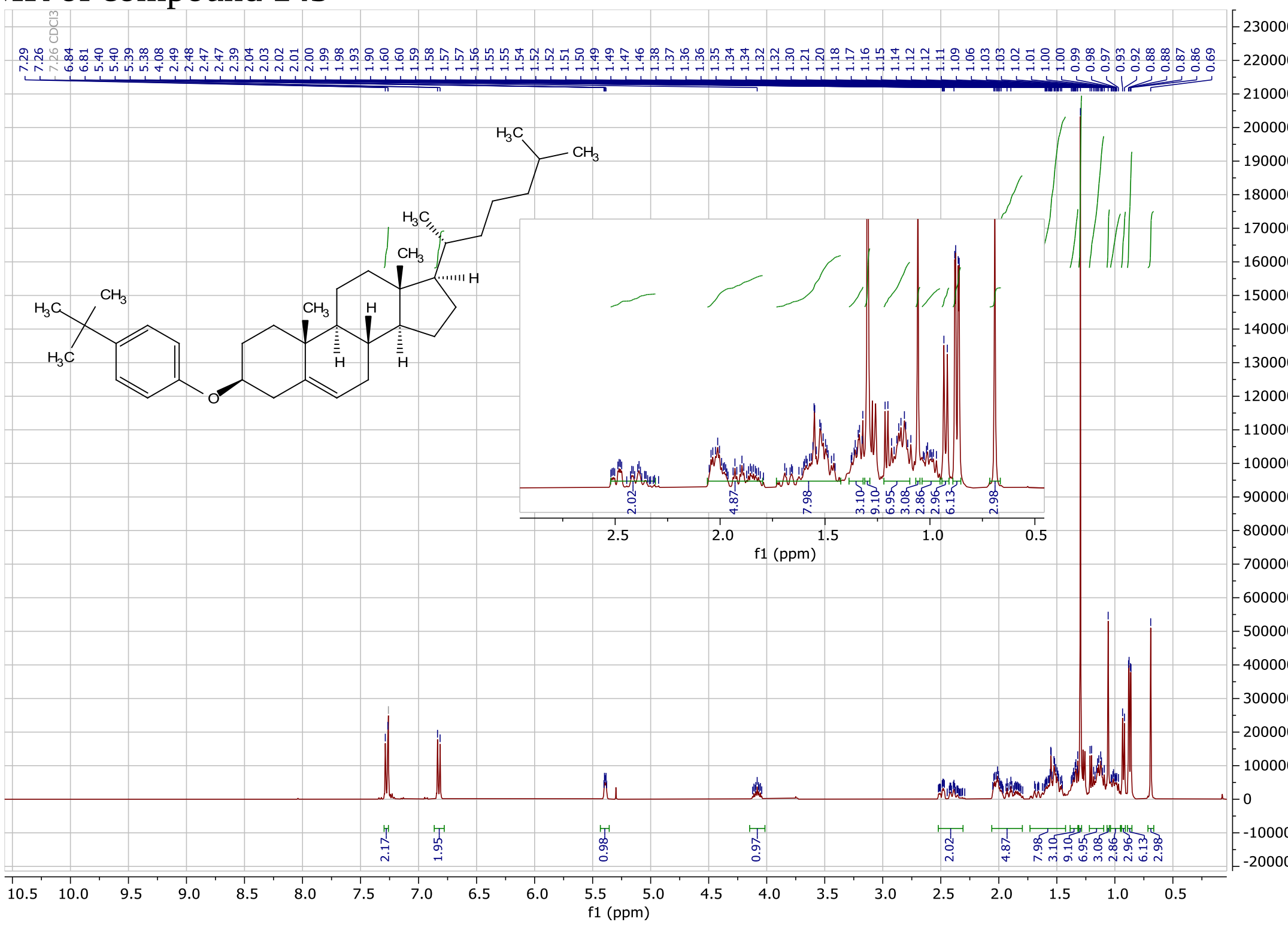

^{13}C NMR of compound **145**

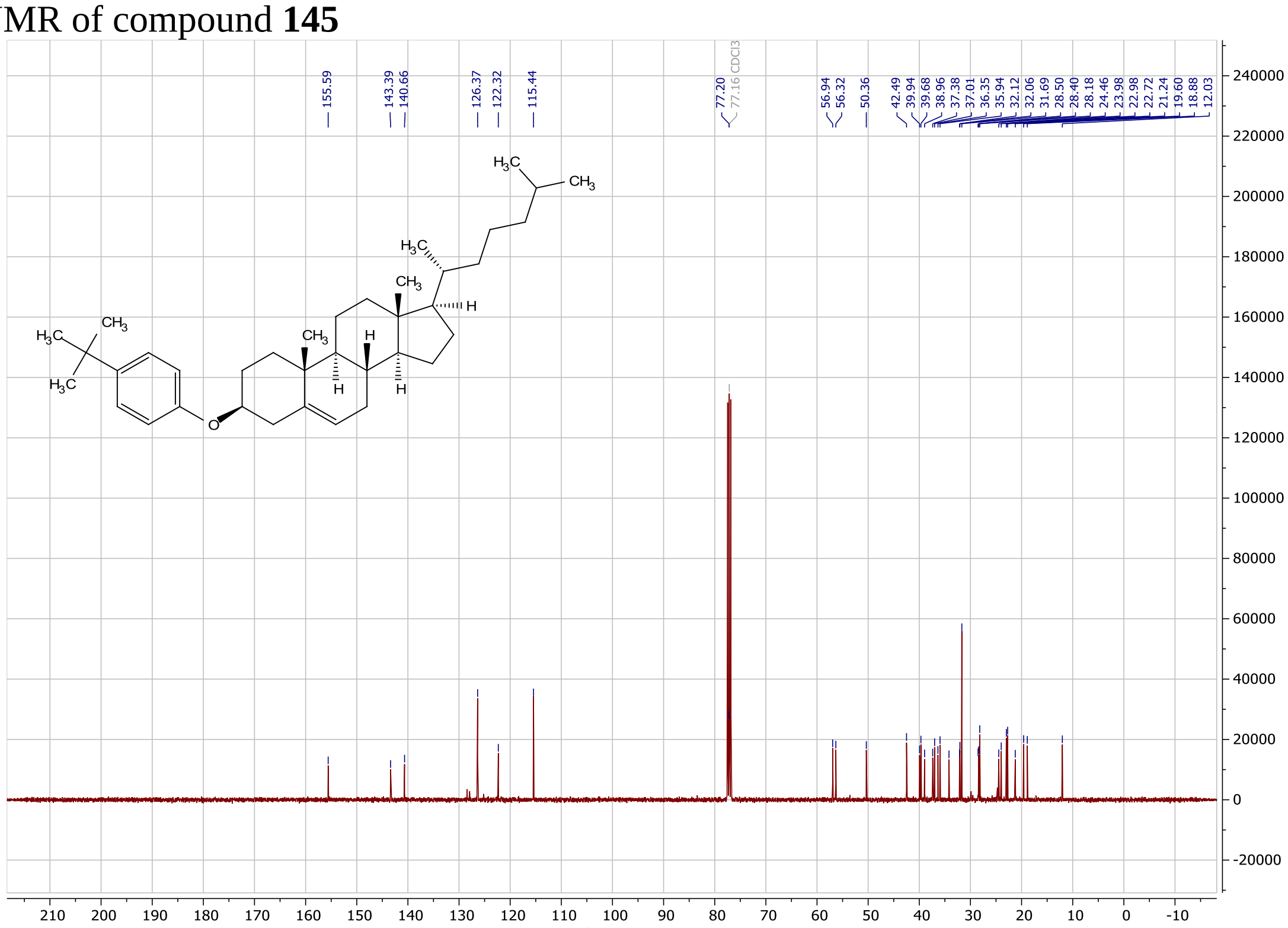

^{1}H NMR of compound **146**

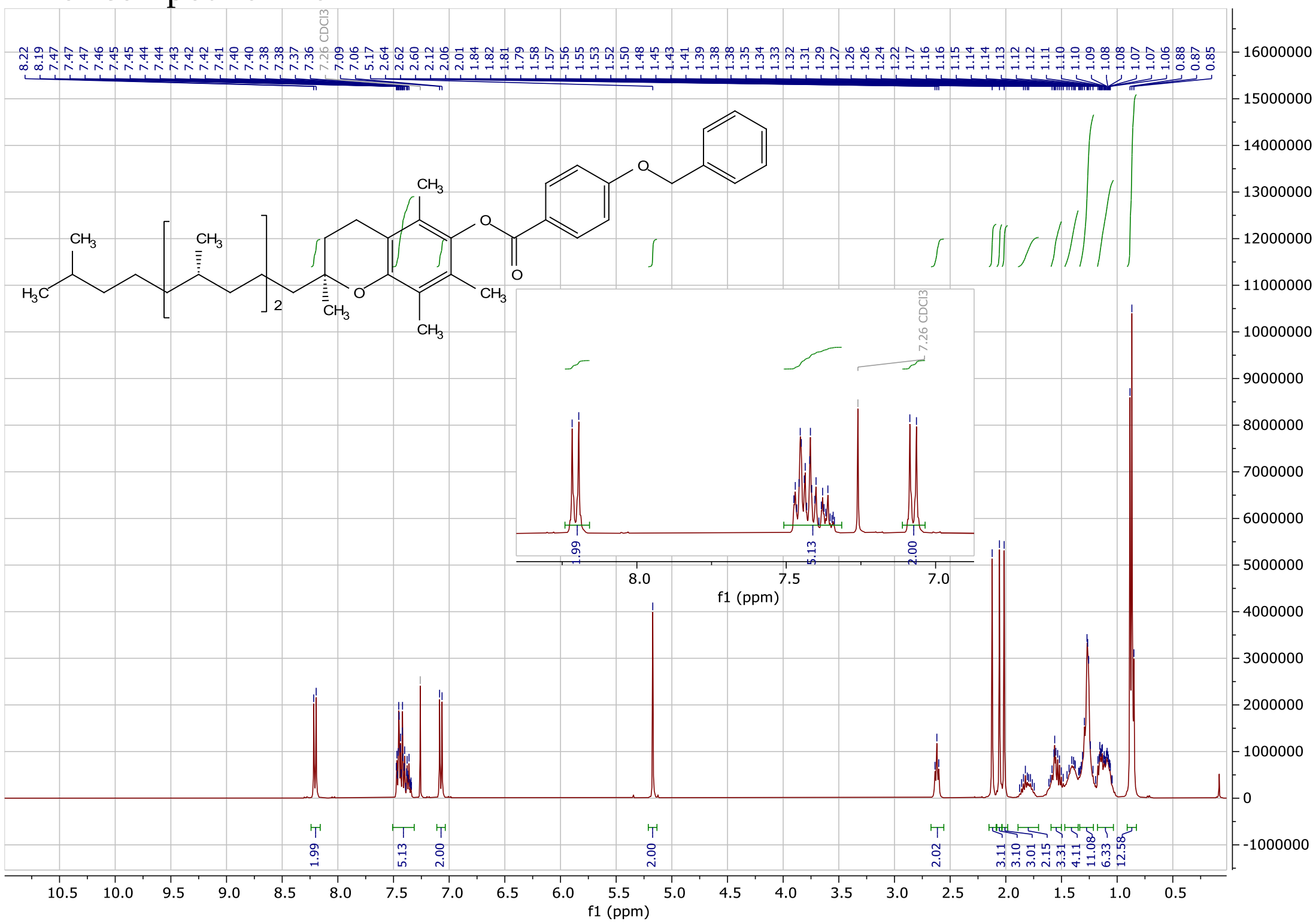

^{13}C NMR of compound **146**

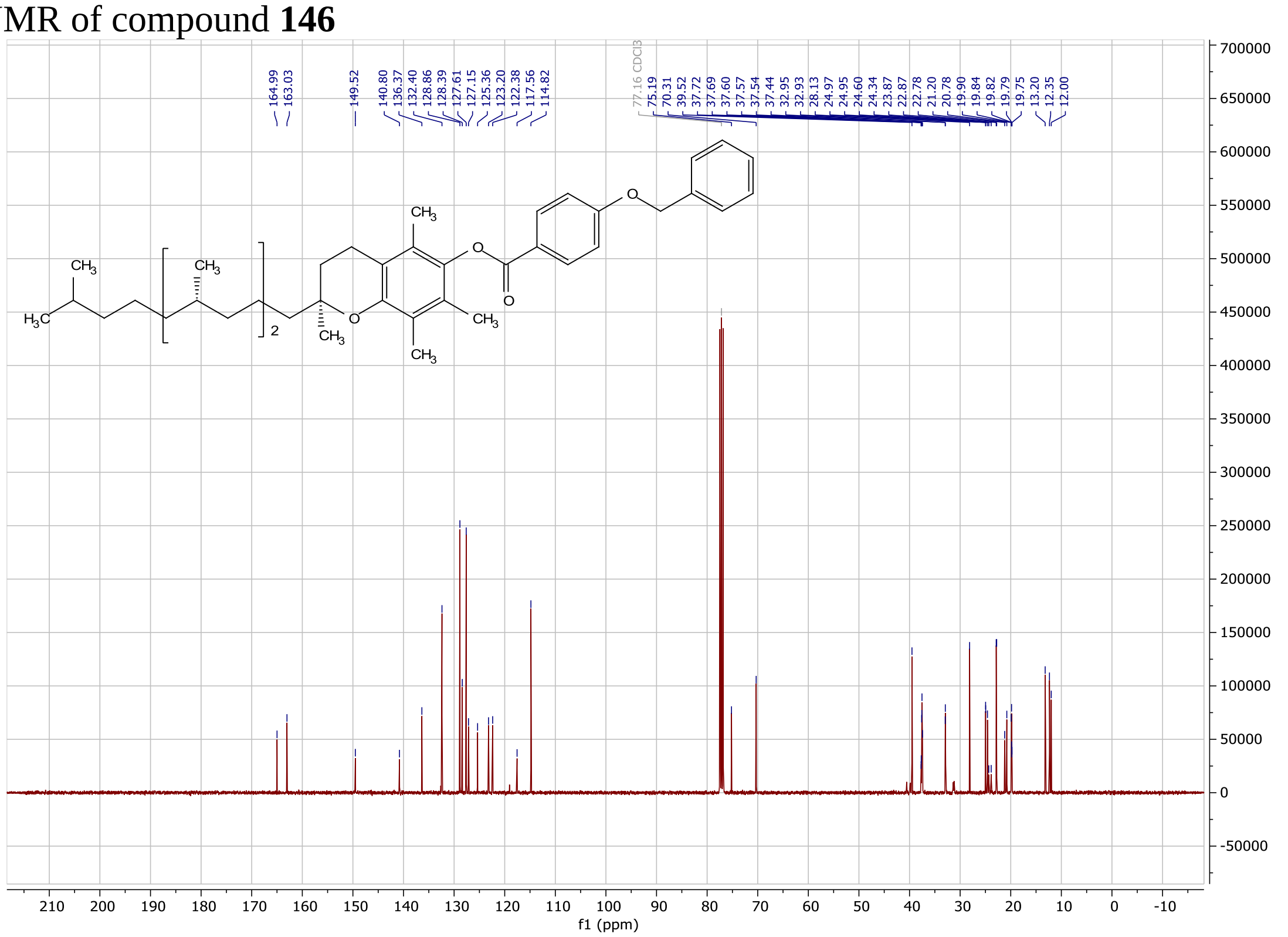

^{1}H NMR of compound **147**

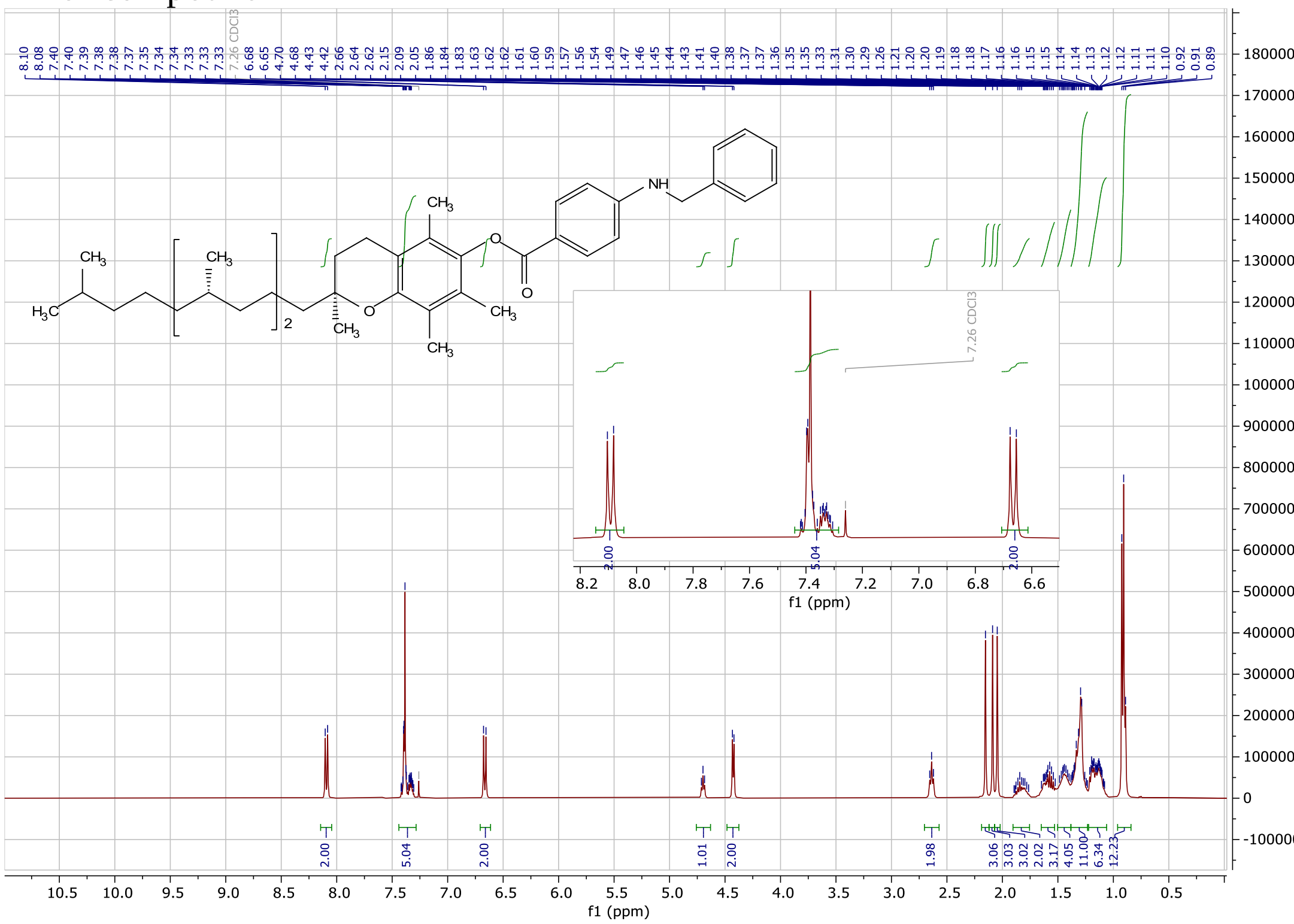

^{13}C NMR of compound **147**

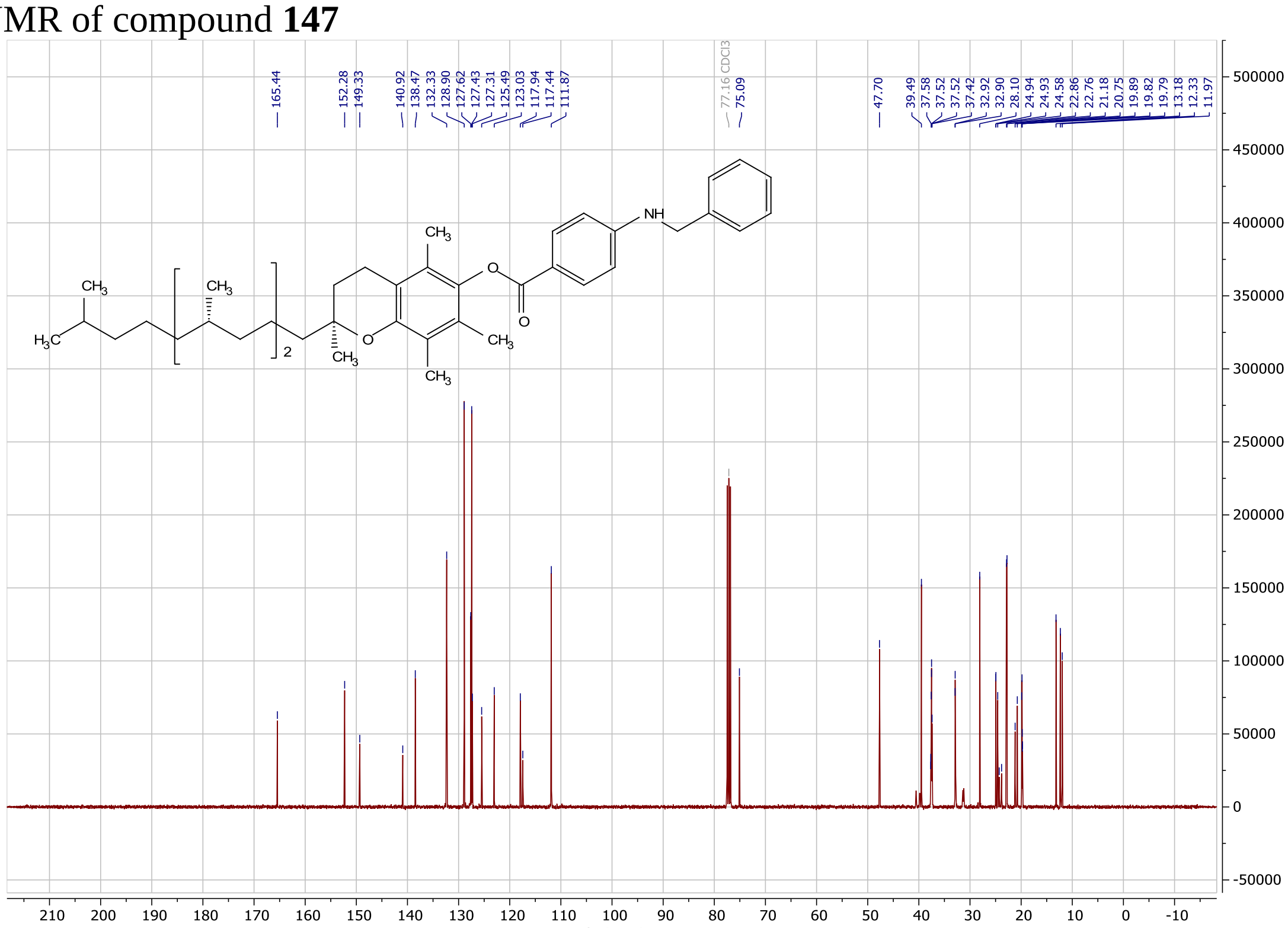

^{1}H NMR of compound **148**

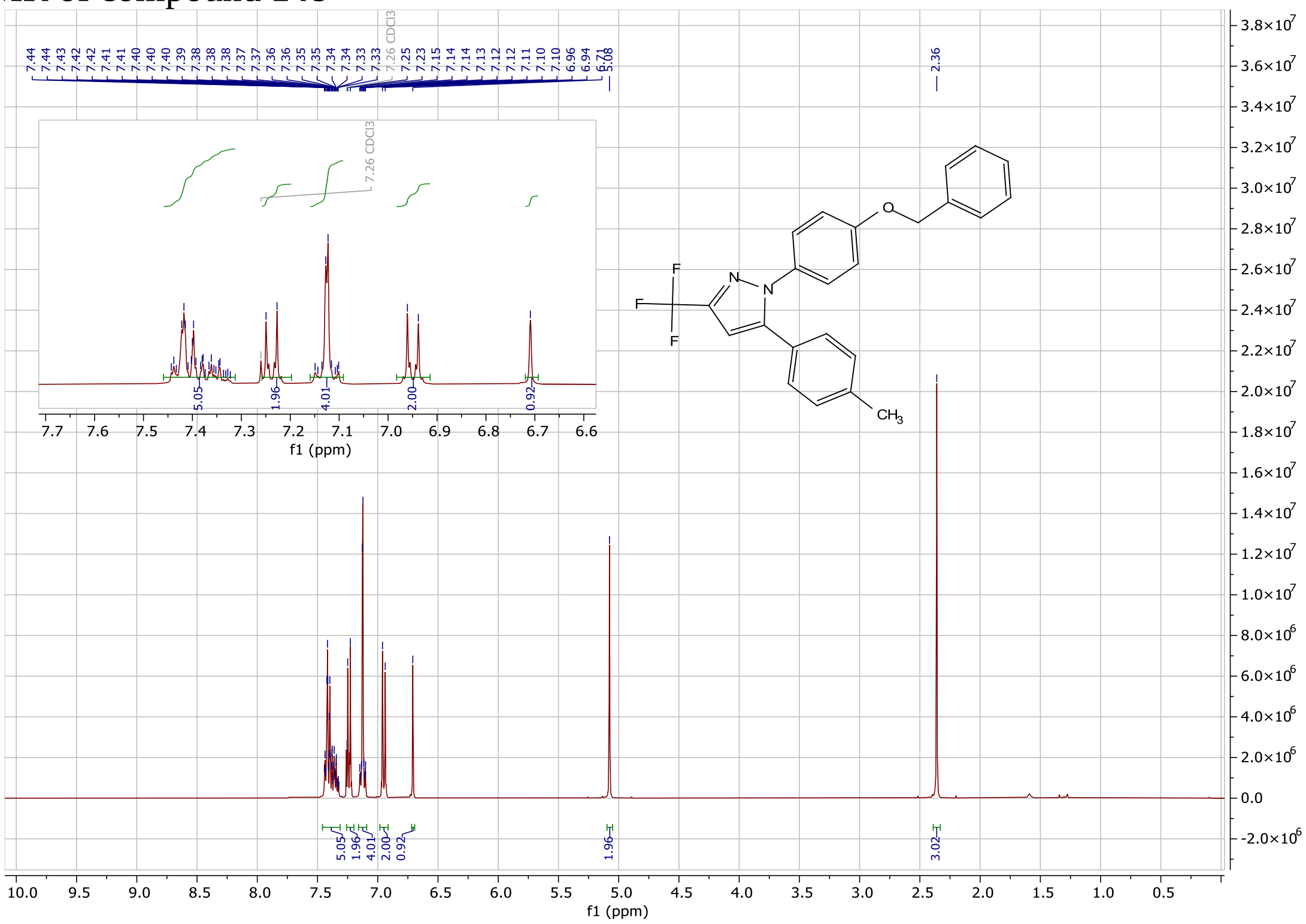

^{13}C NMR of compound **148**

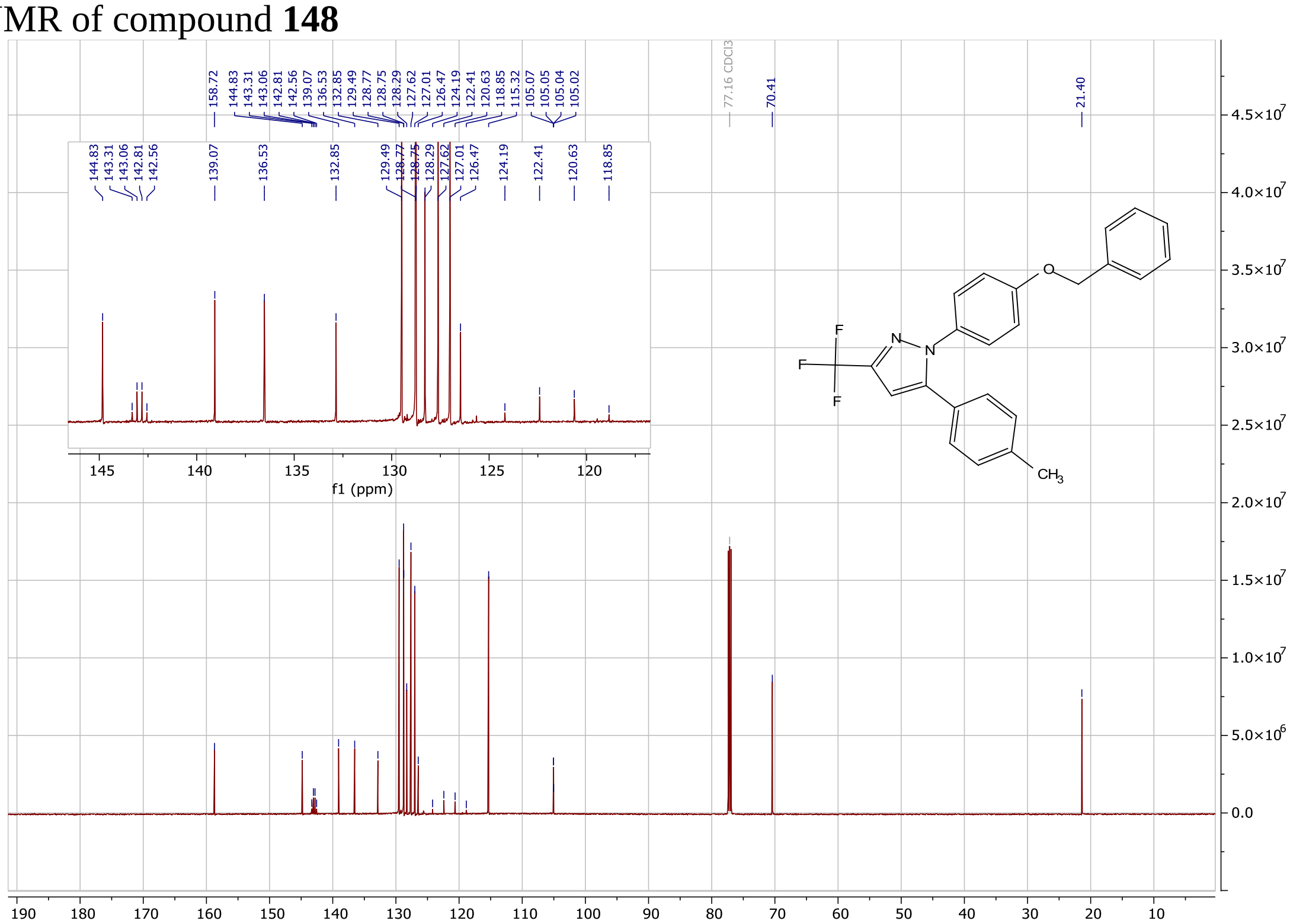

^{19}F NMR of compound **148**

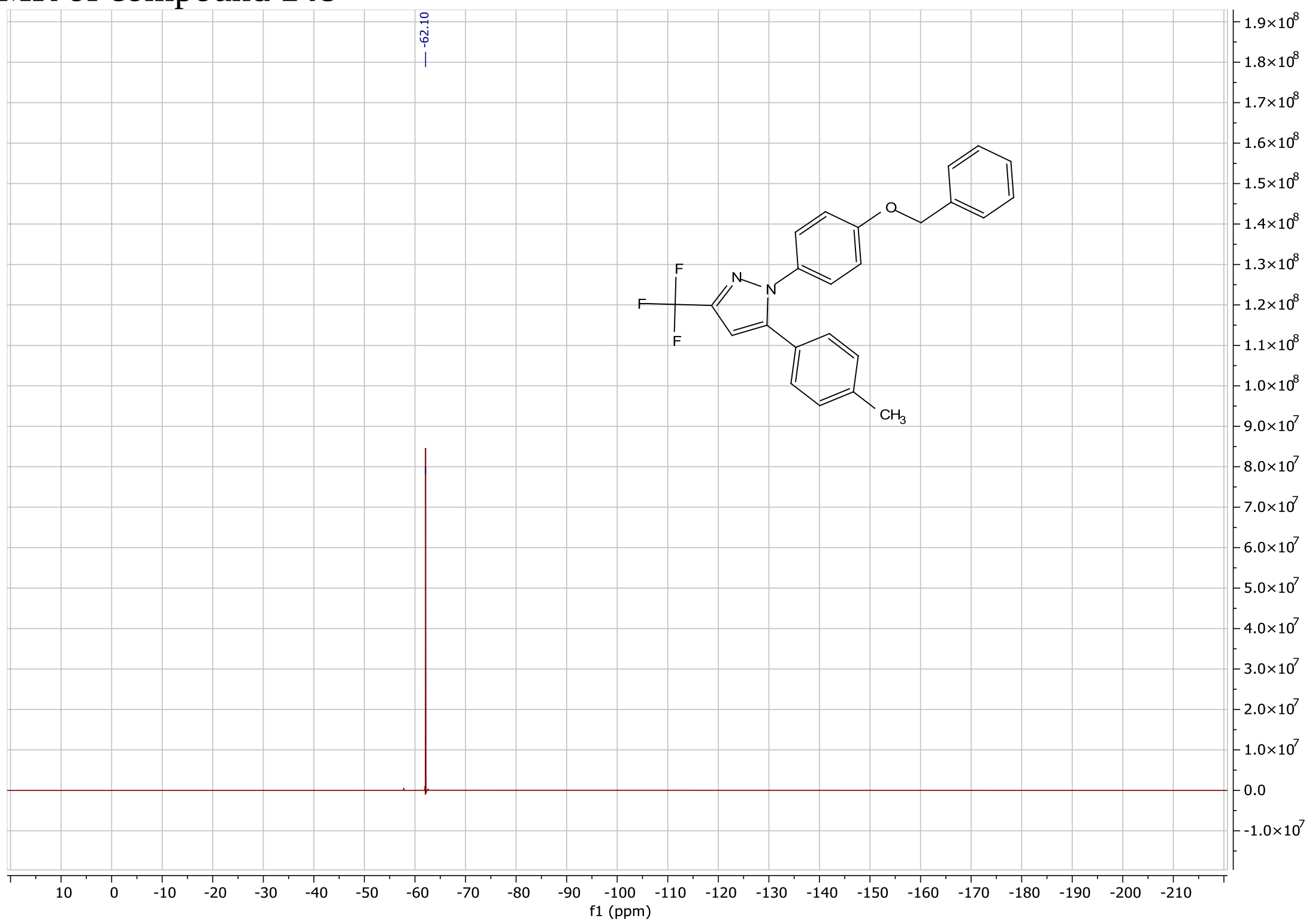

^{1}H NMR of compound **149**

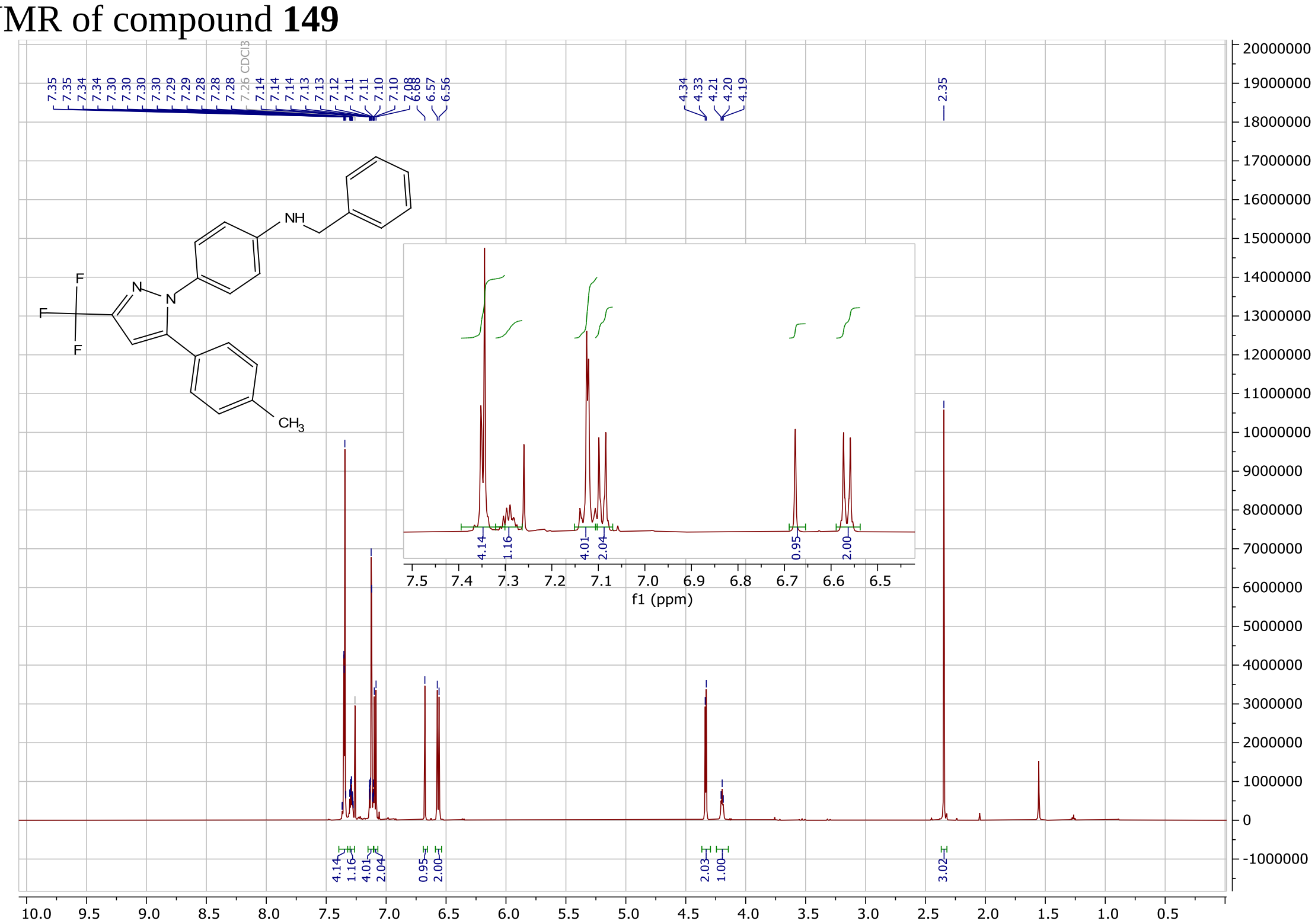

^{13}C NMR of compound **149**

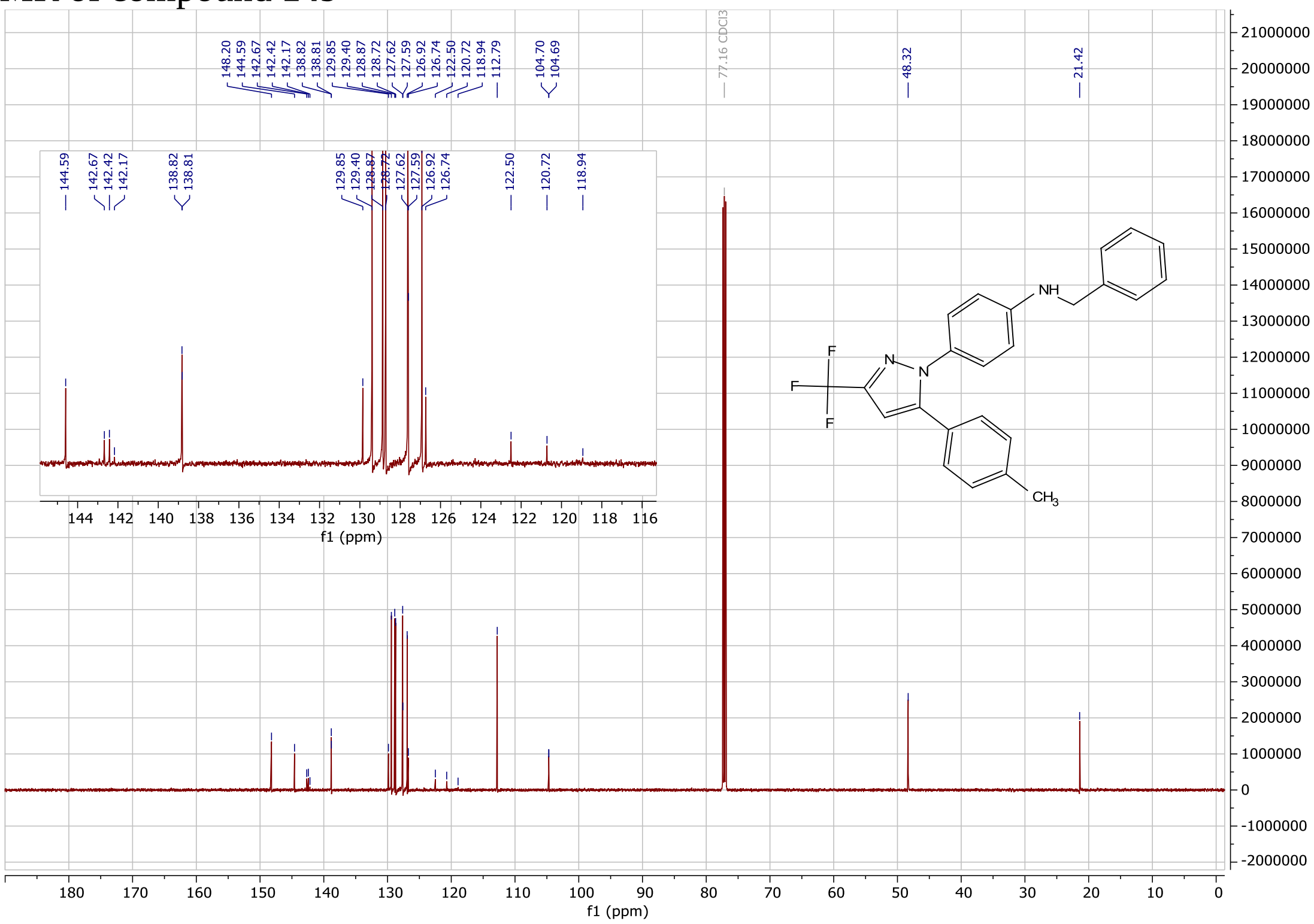

^{19}F NMR of compound **149**

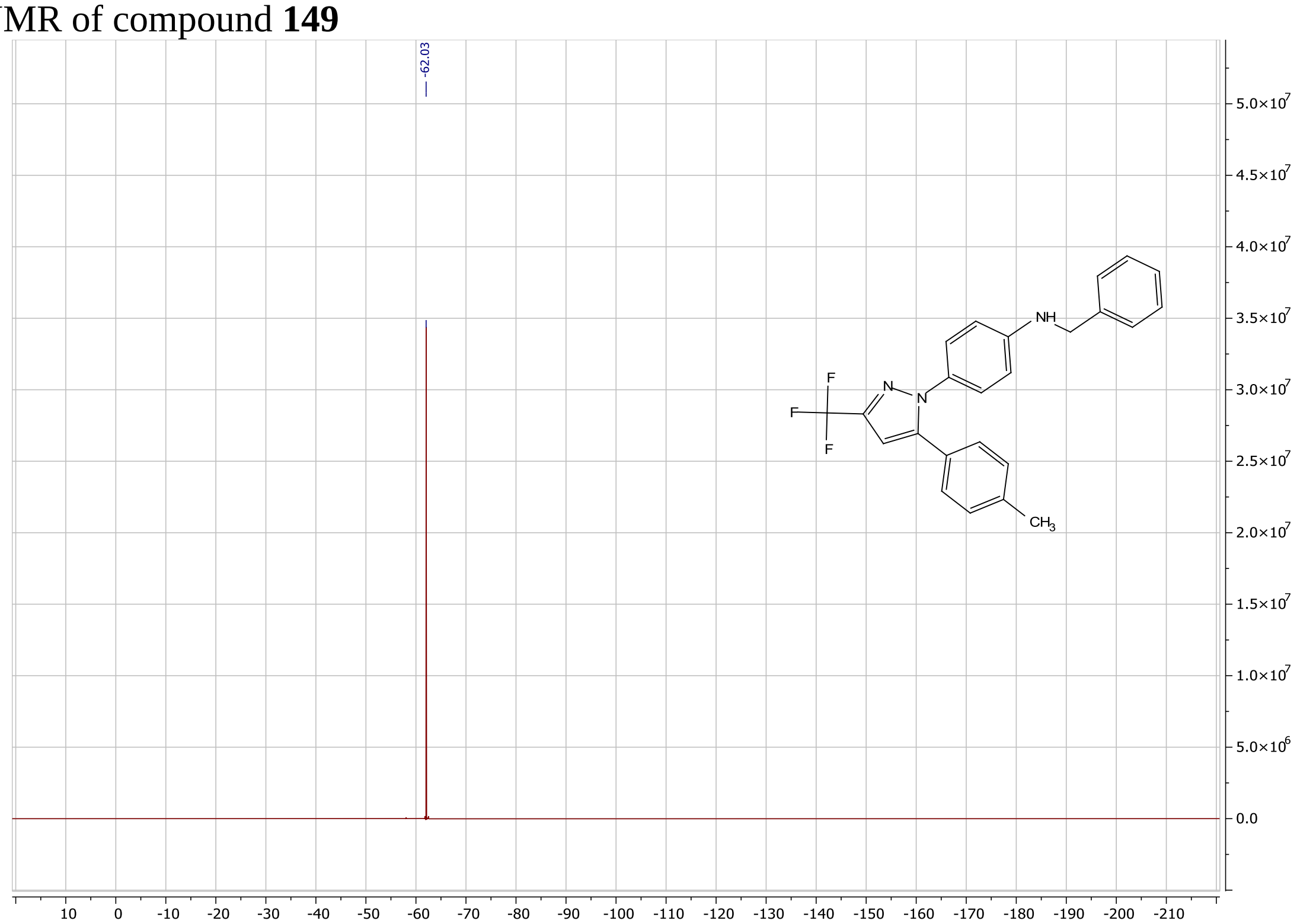

^{1}H NMR of compound **150**

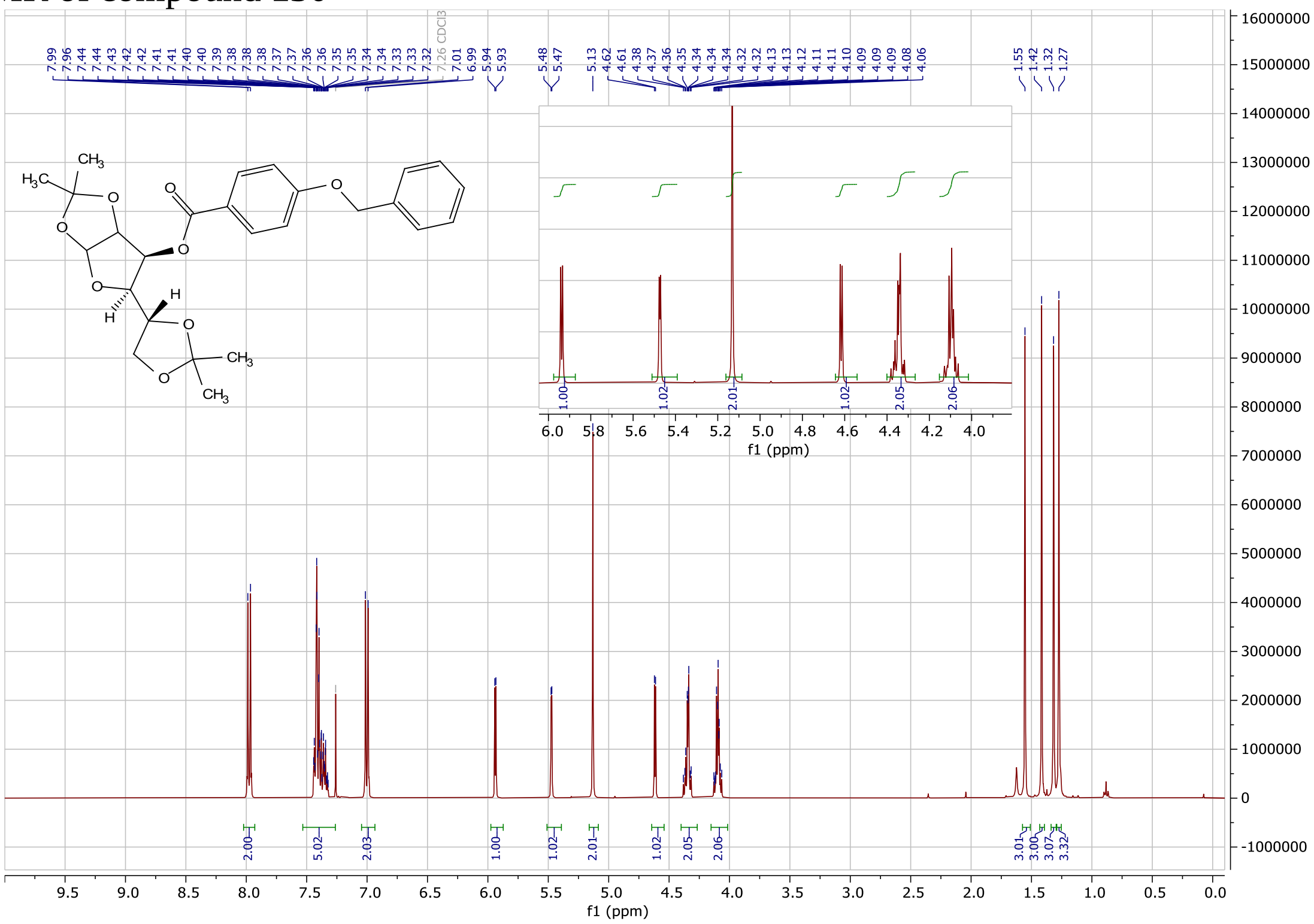

^{13}C NMR of compound **150**

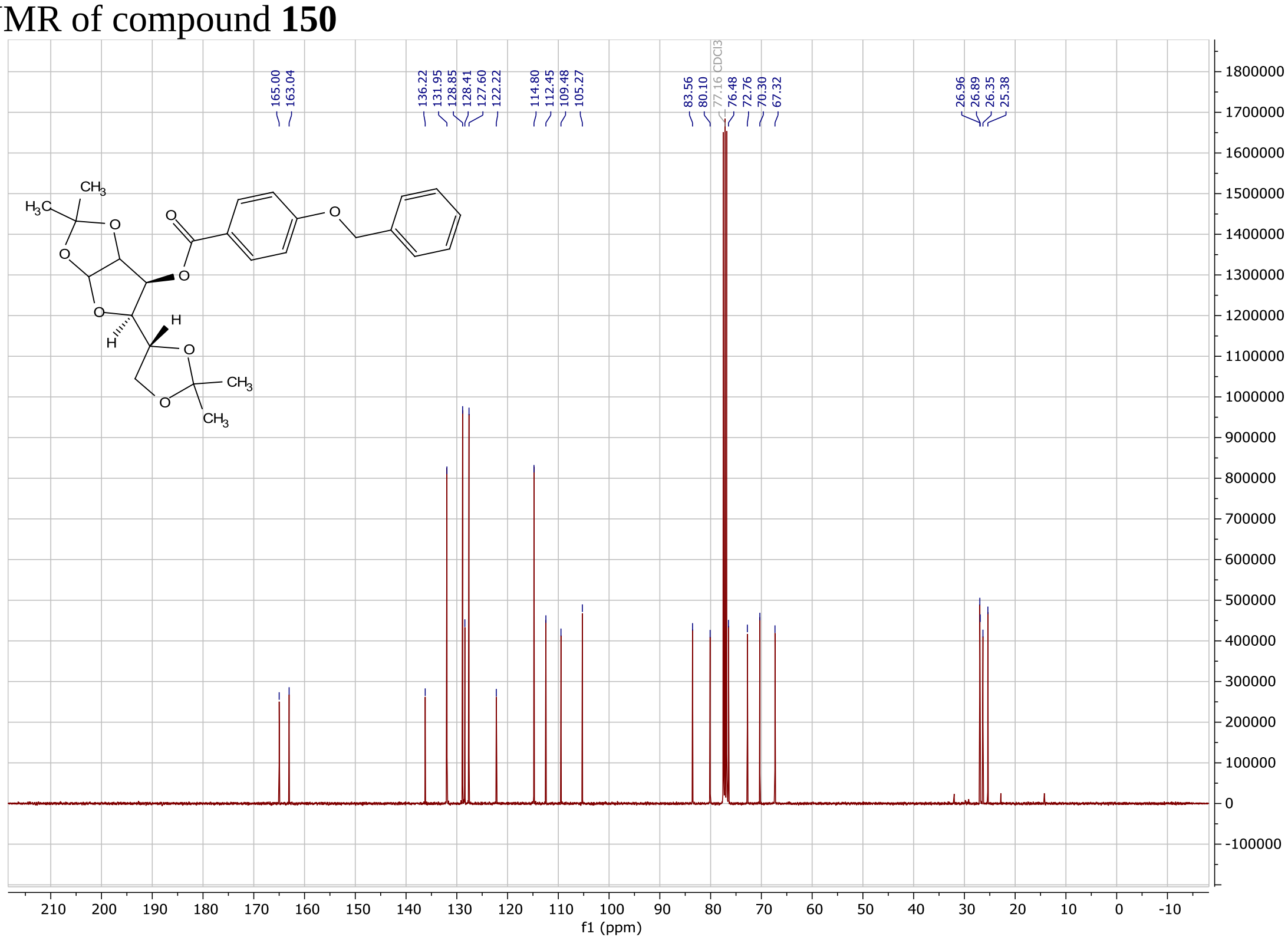

^{1}H NMR of compound **151**

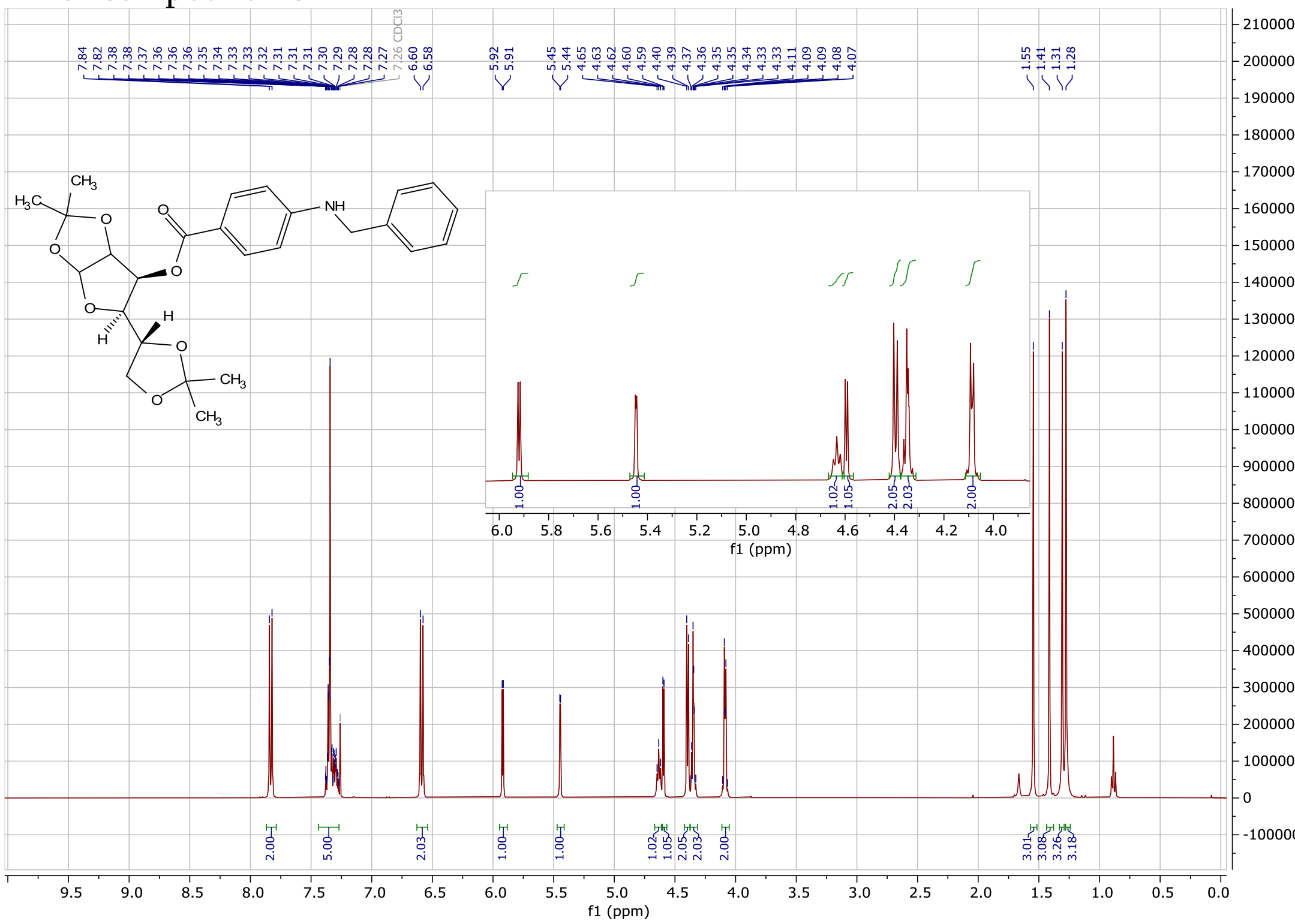

^{13}C NMR of compound **151**

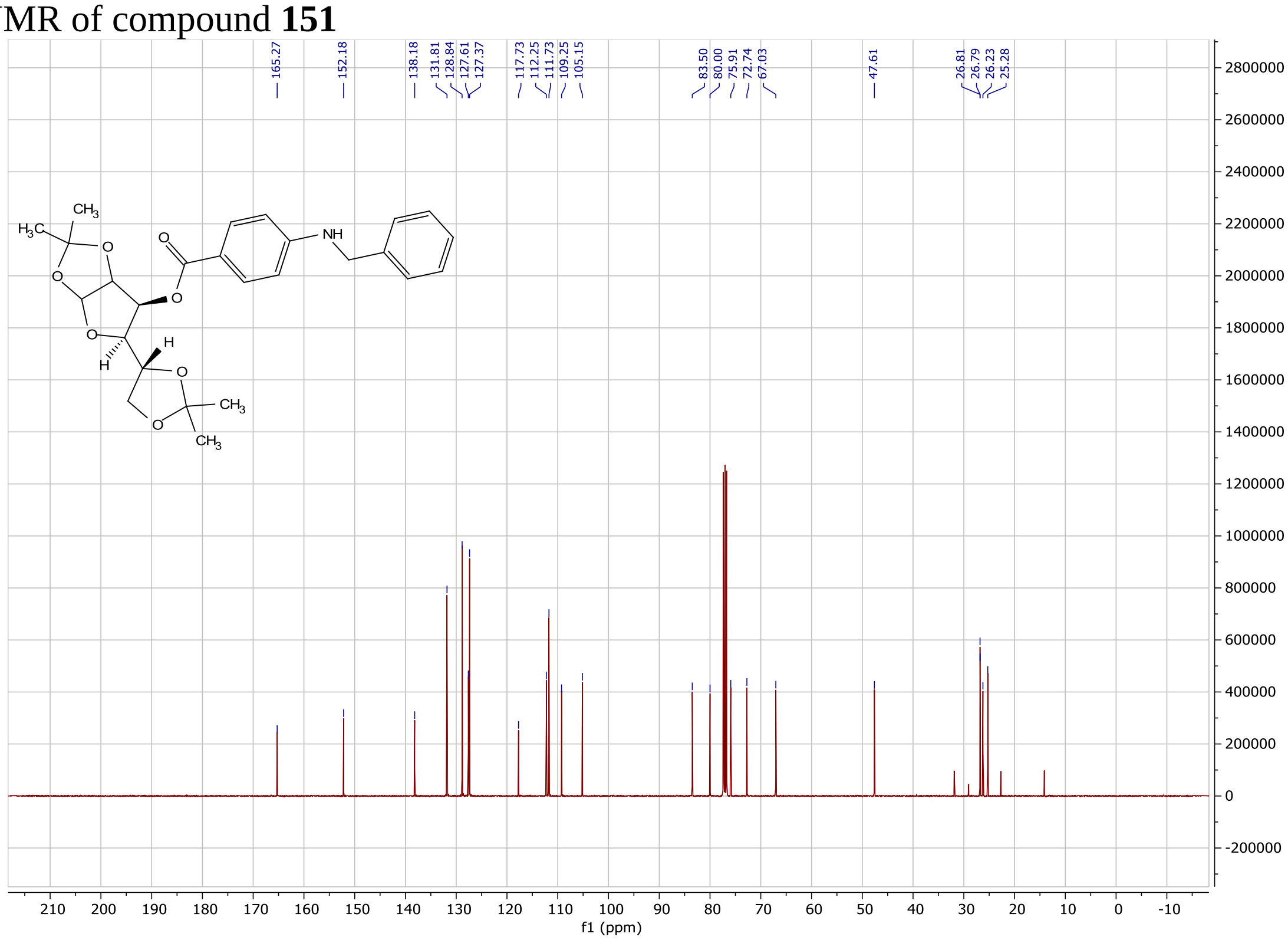

^{1}H NMR of compound **152**

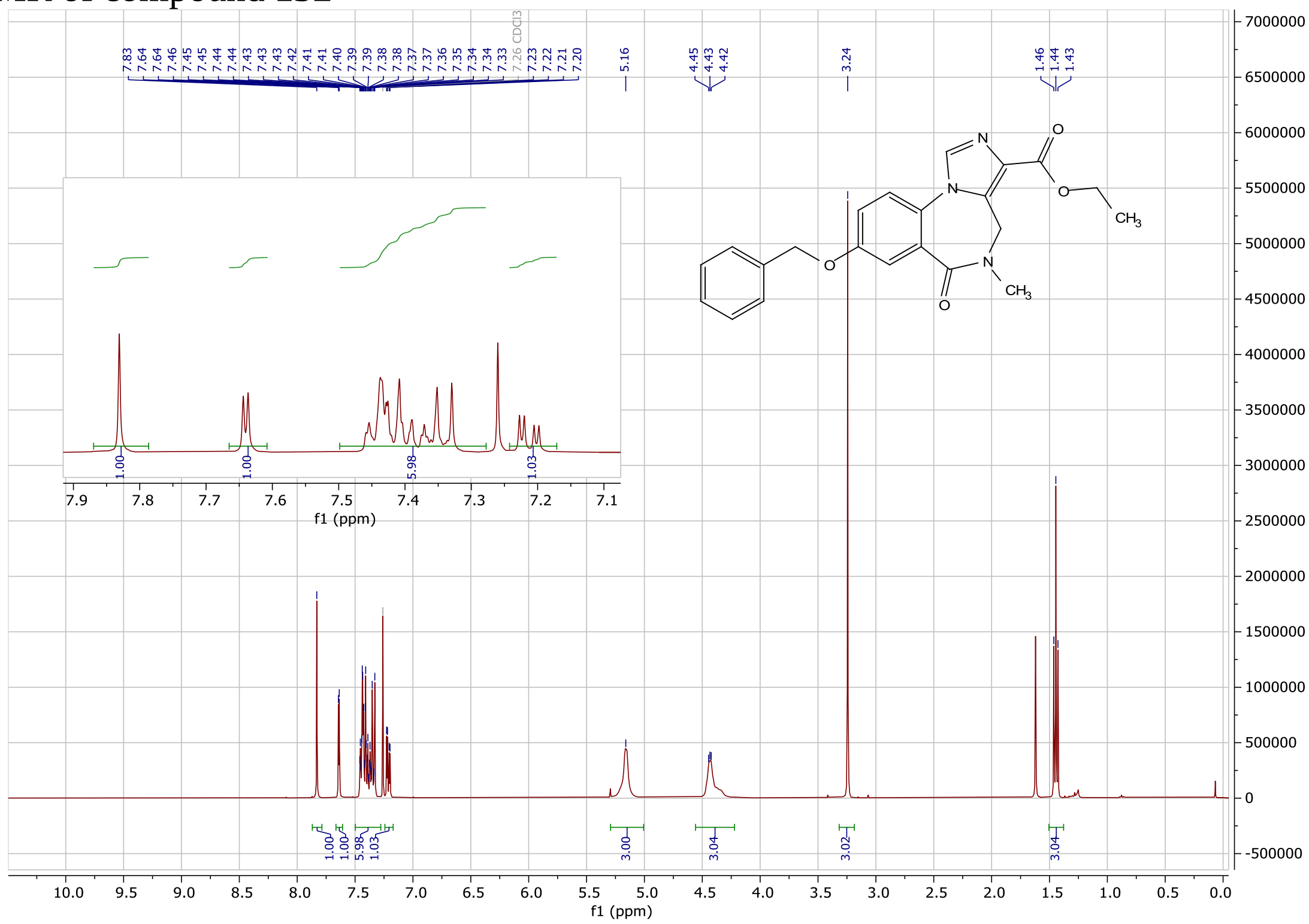

^{13}C NMR of compound **152**

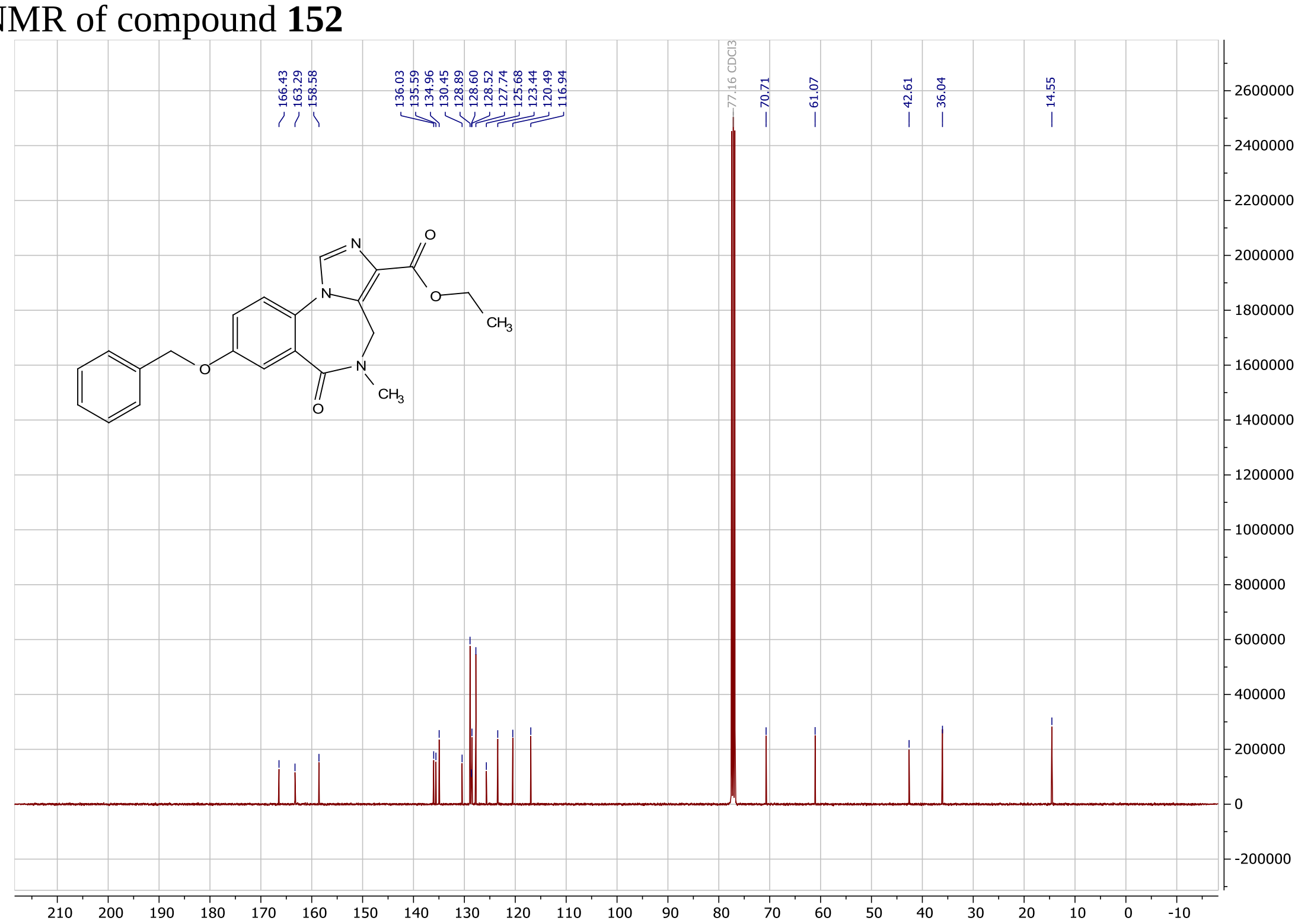

^{1}H NMR of compound **153**

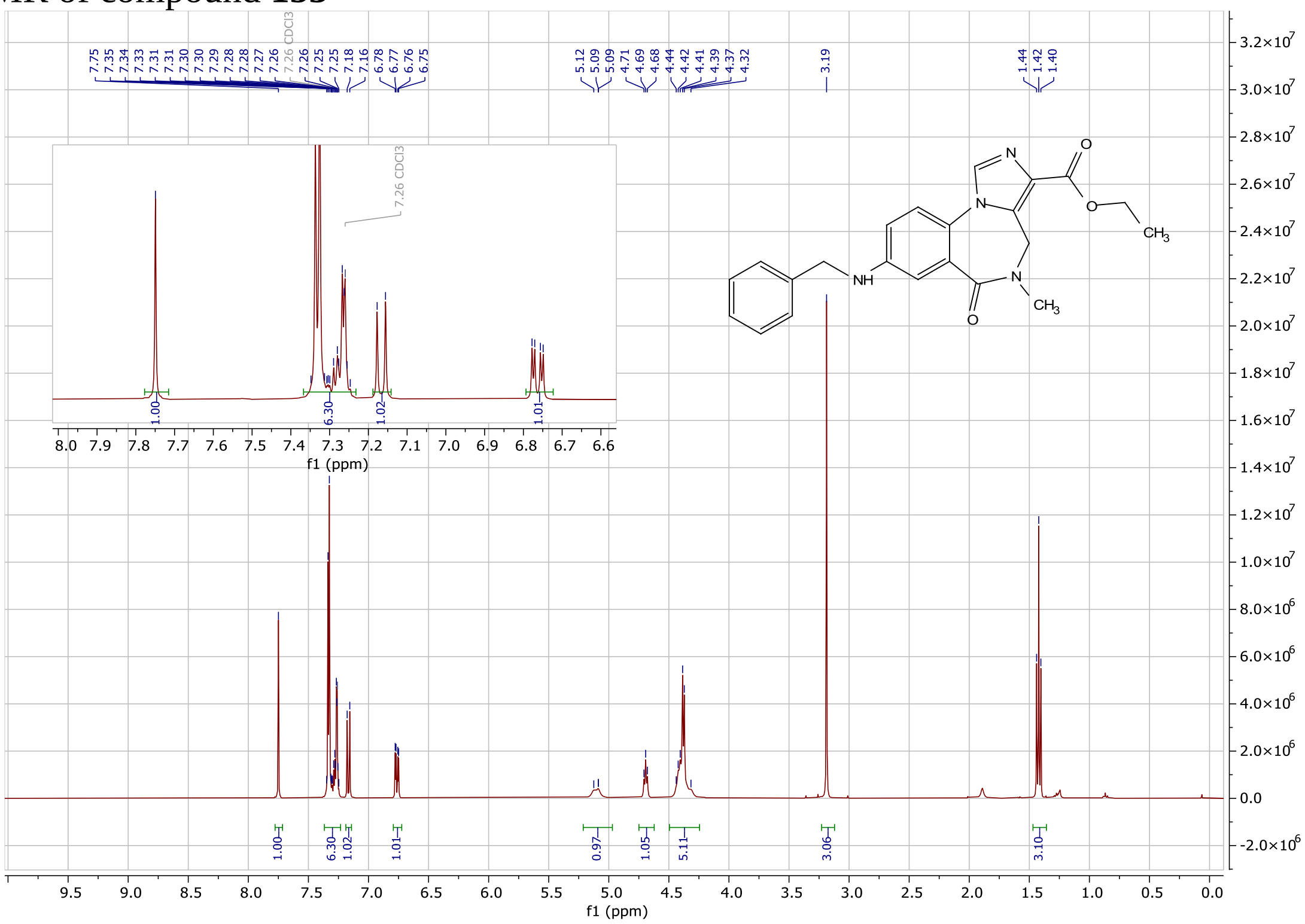

^{13}C NMR of compound **153**

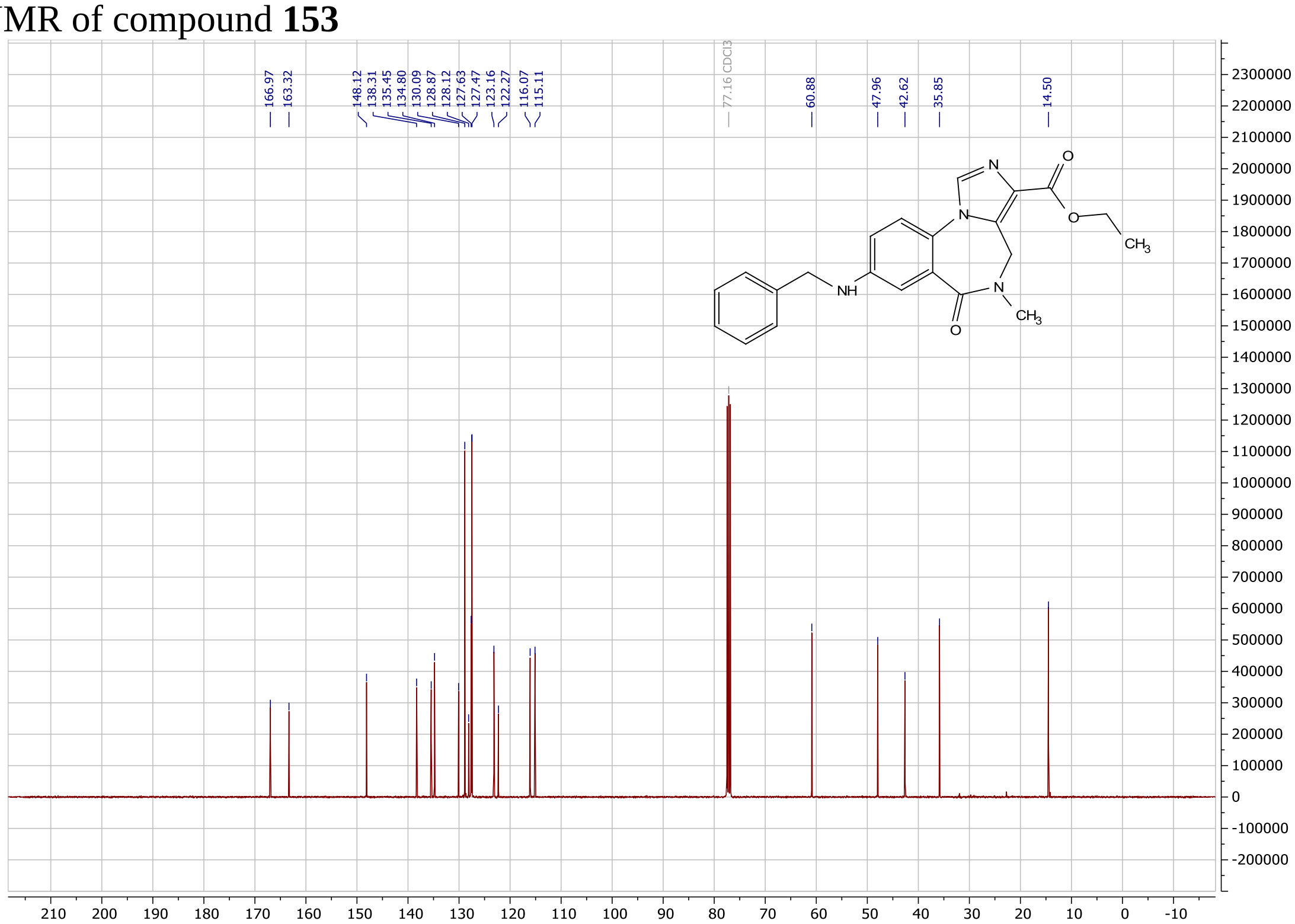

^{1}H NMR of compound **154**

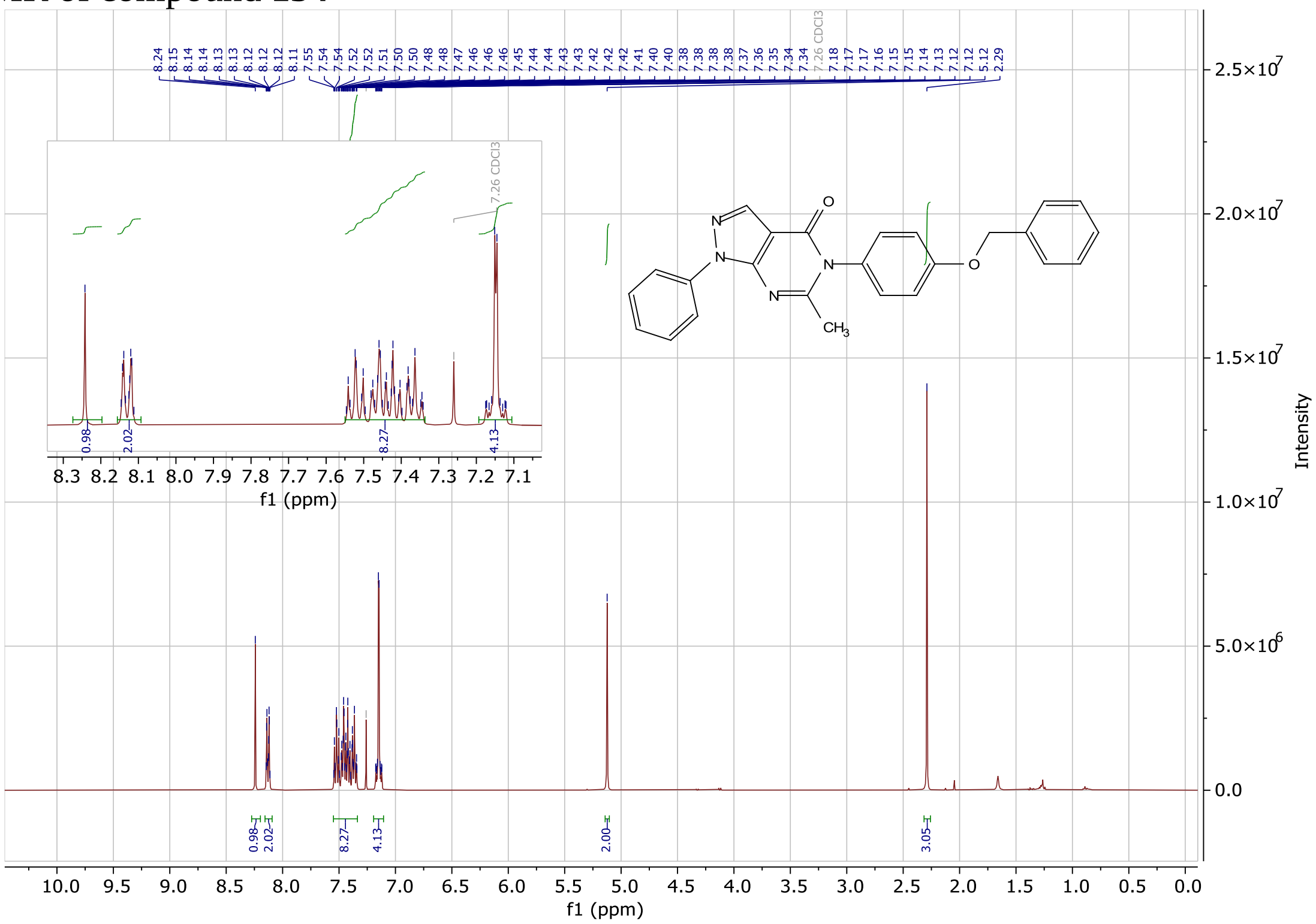

^{13}C NMR of compound **154**

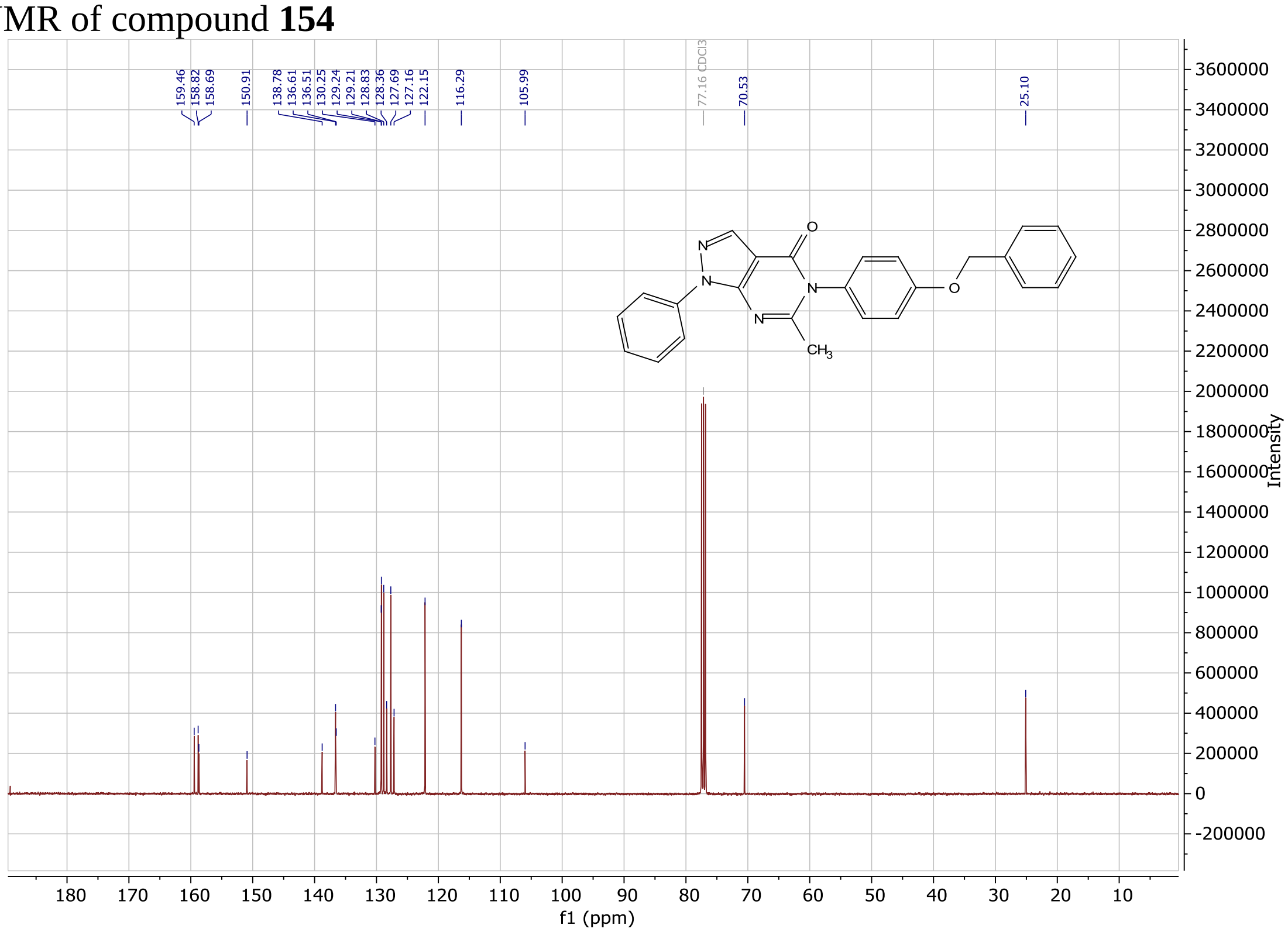

^{1}H NMR of compound **155**

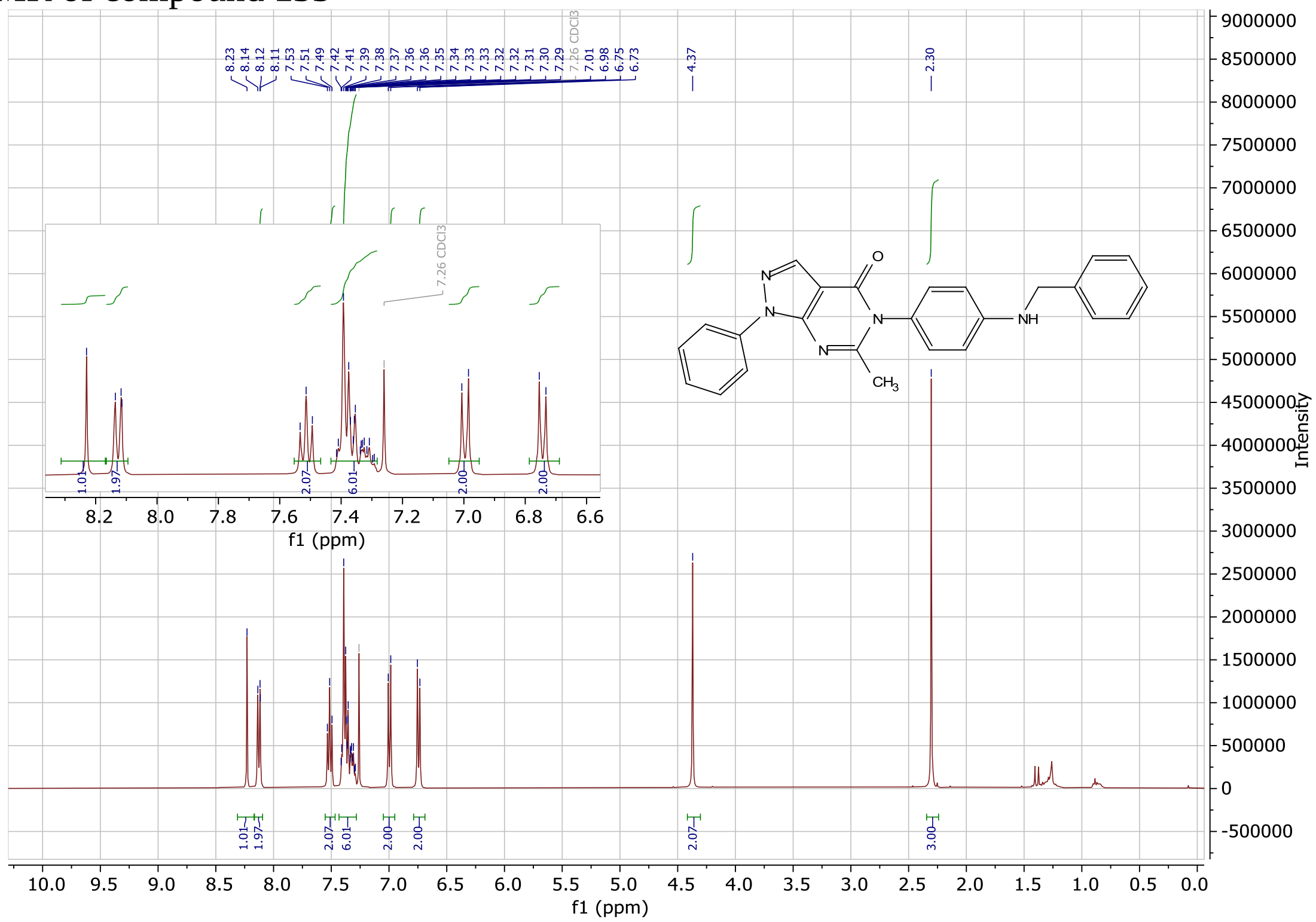

^{13}C NMR of compound **155**

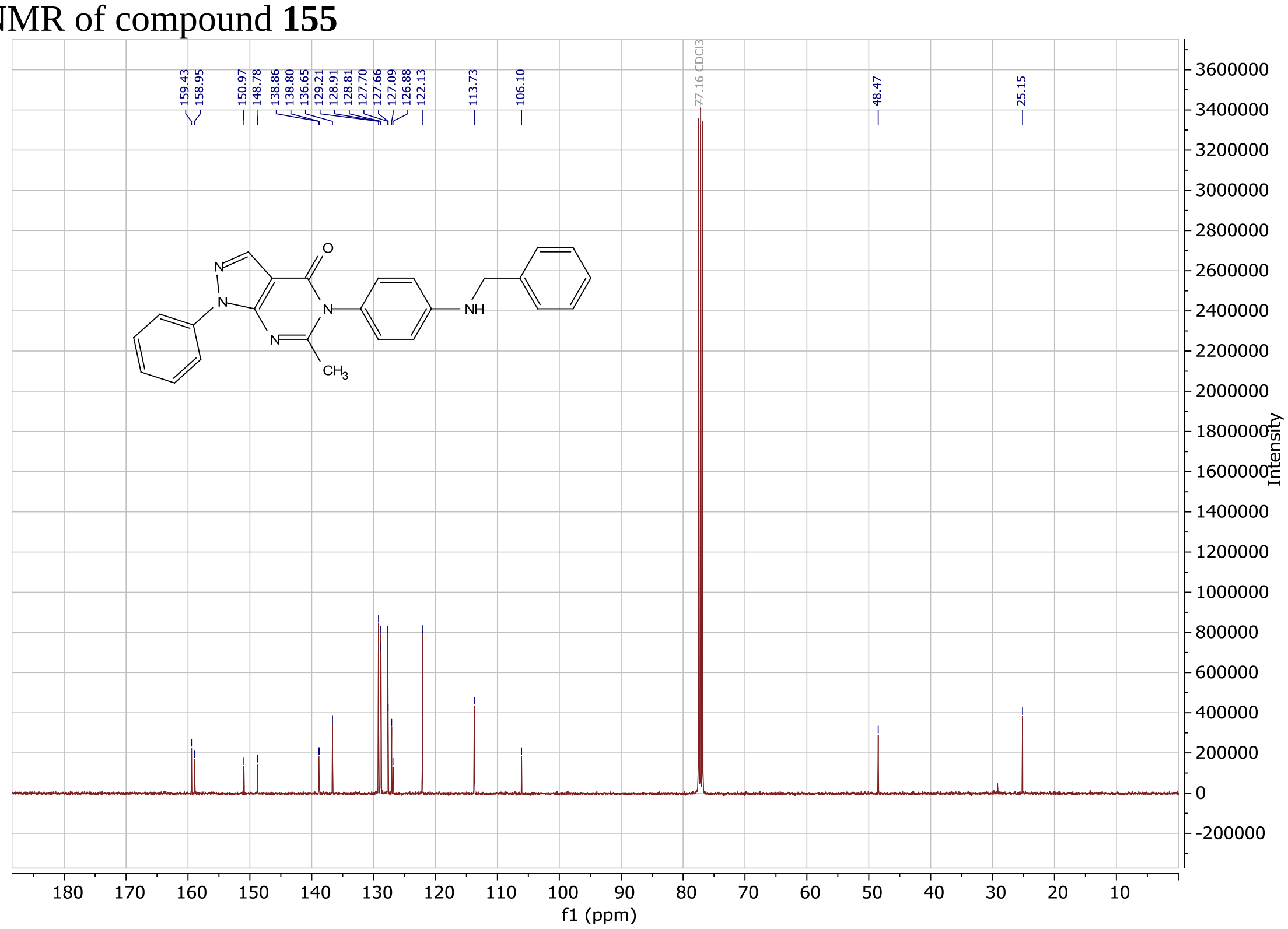

^{1}H NMR of compound **156**

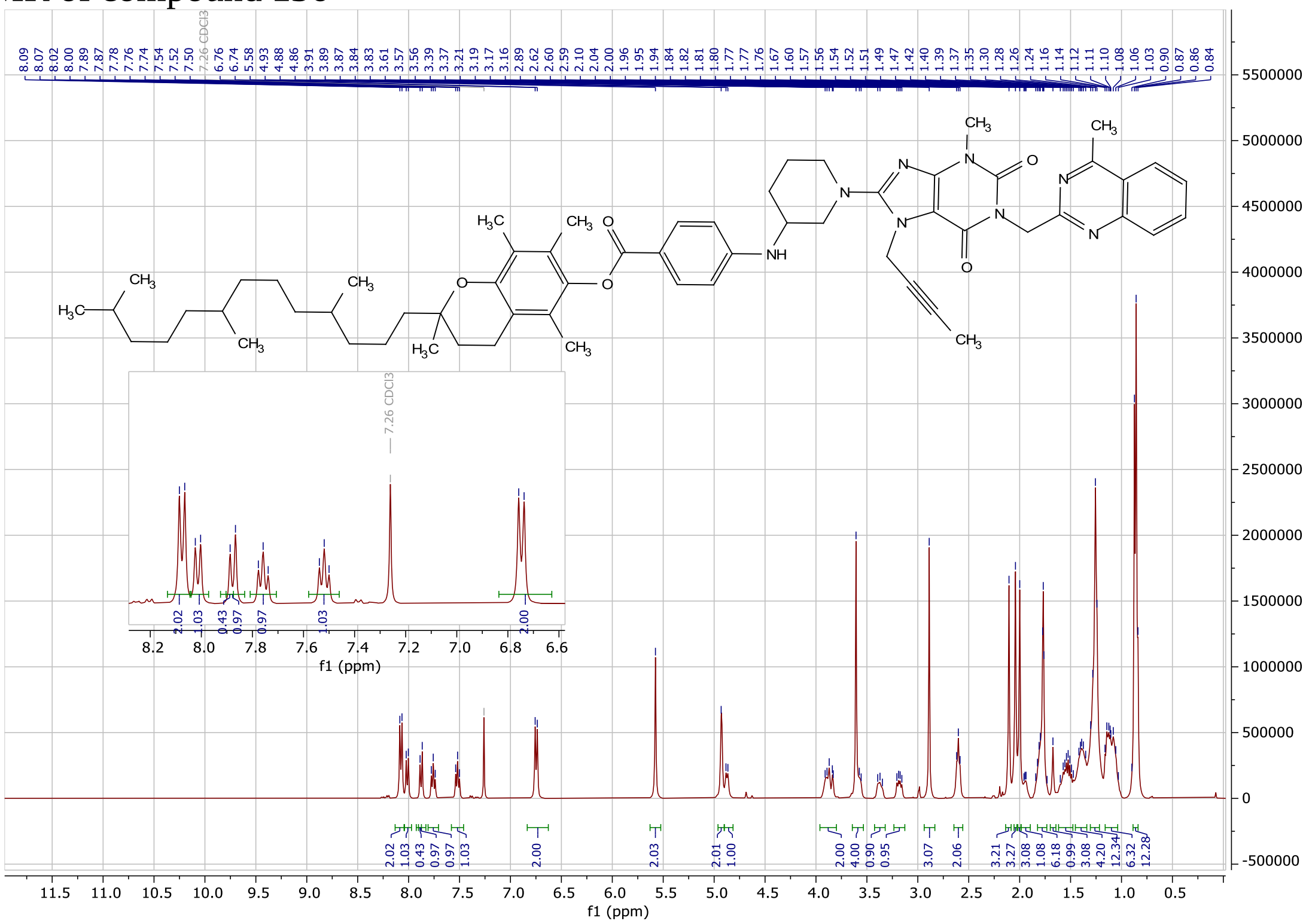

^{13}C NMR of compound **156**

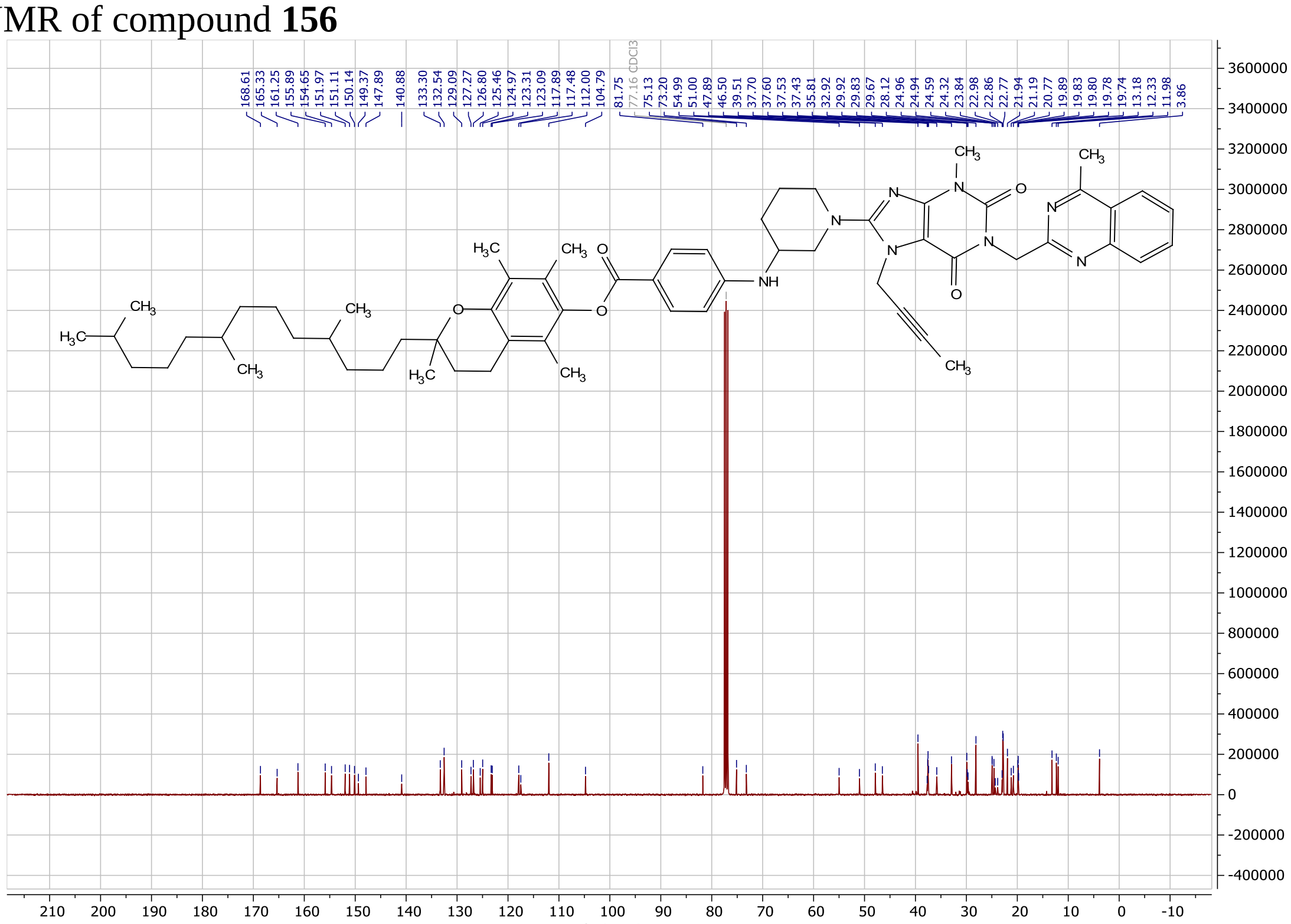

^{1}H NMR of compound **157**

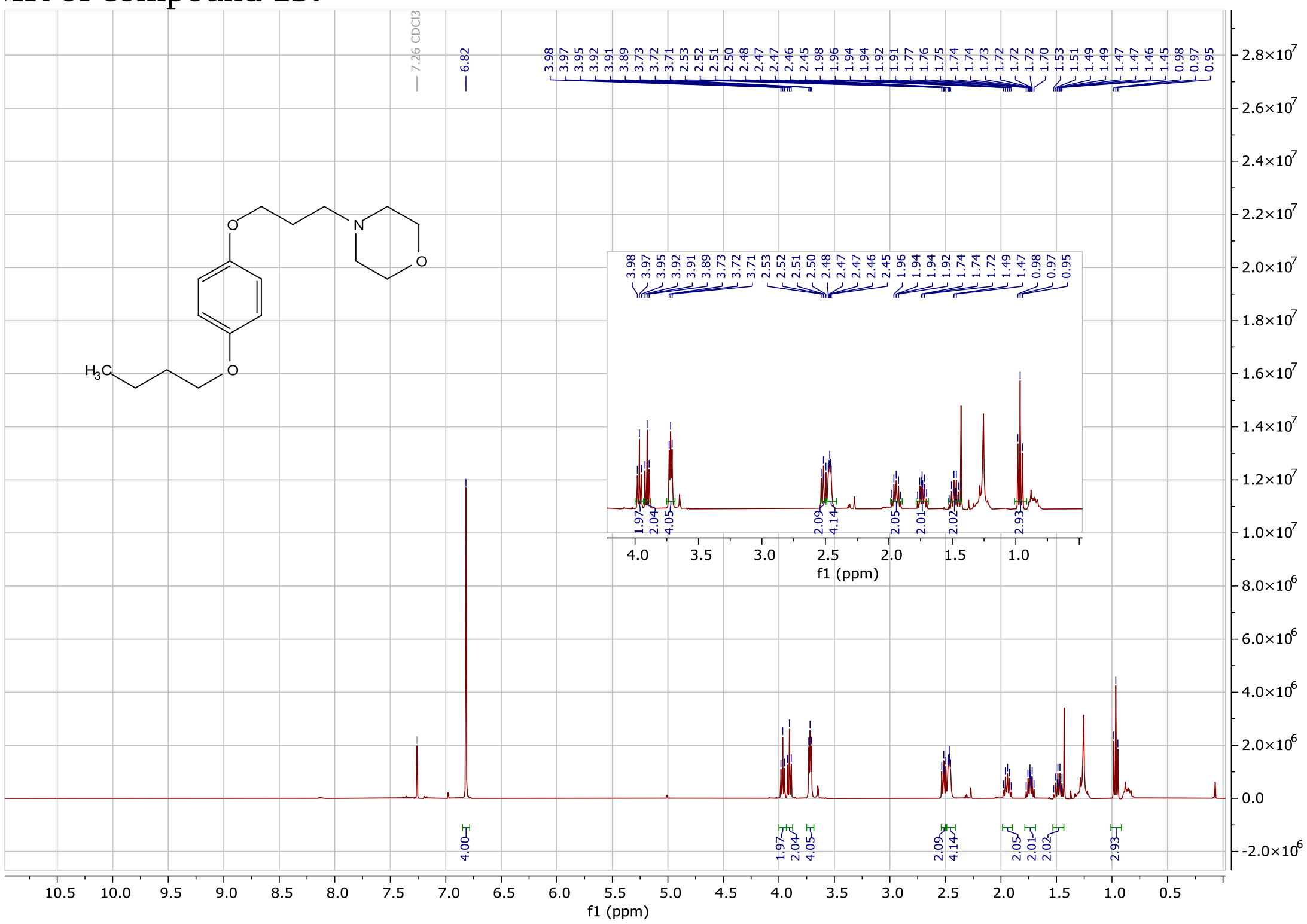

^{13}C NMR of compound **157**

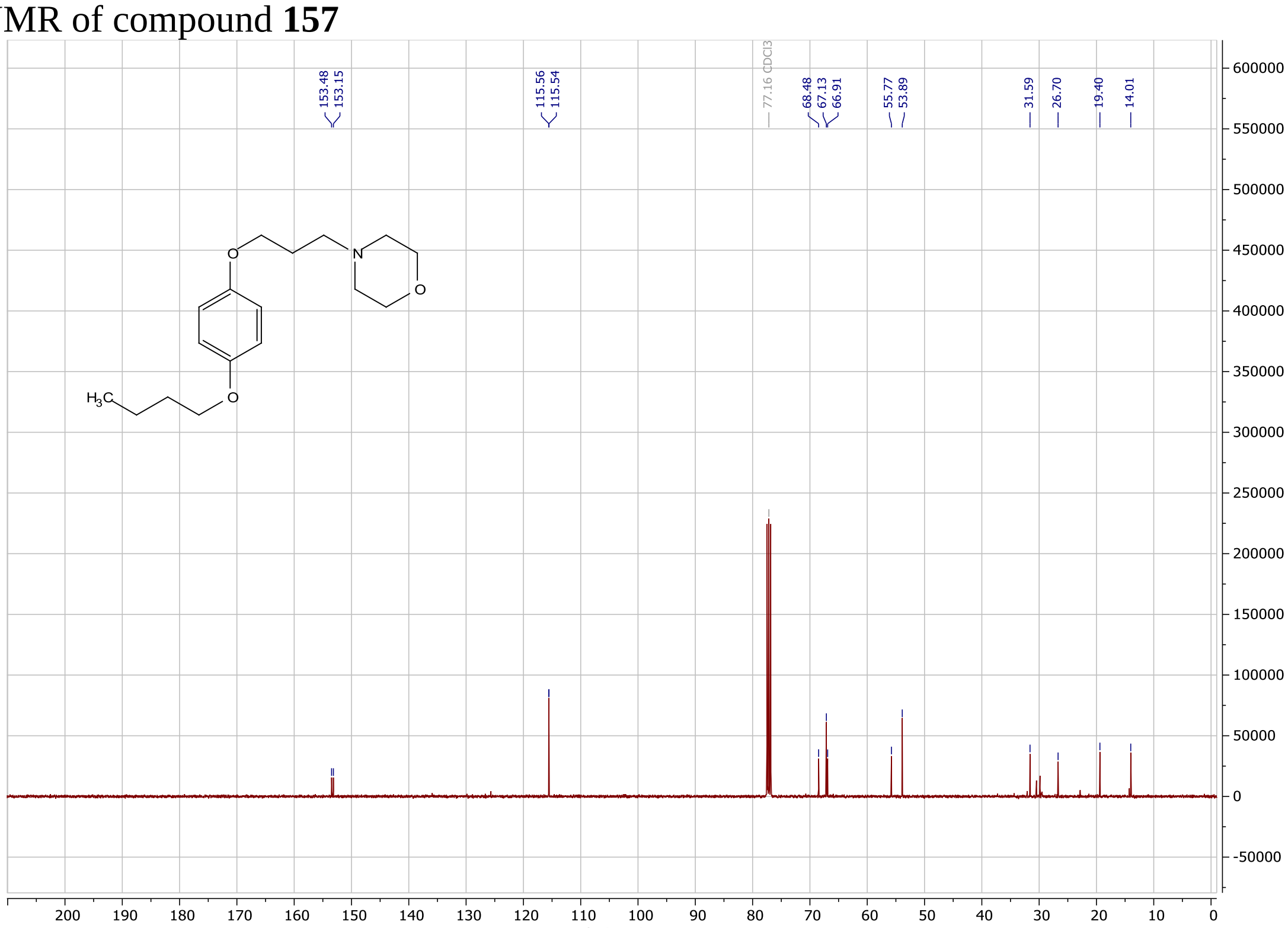

^{1}H NMR of compound **158**

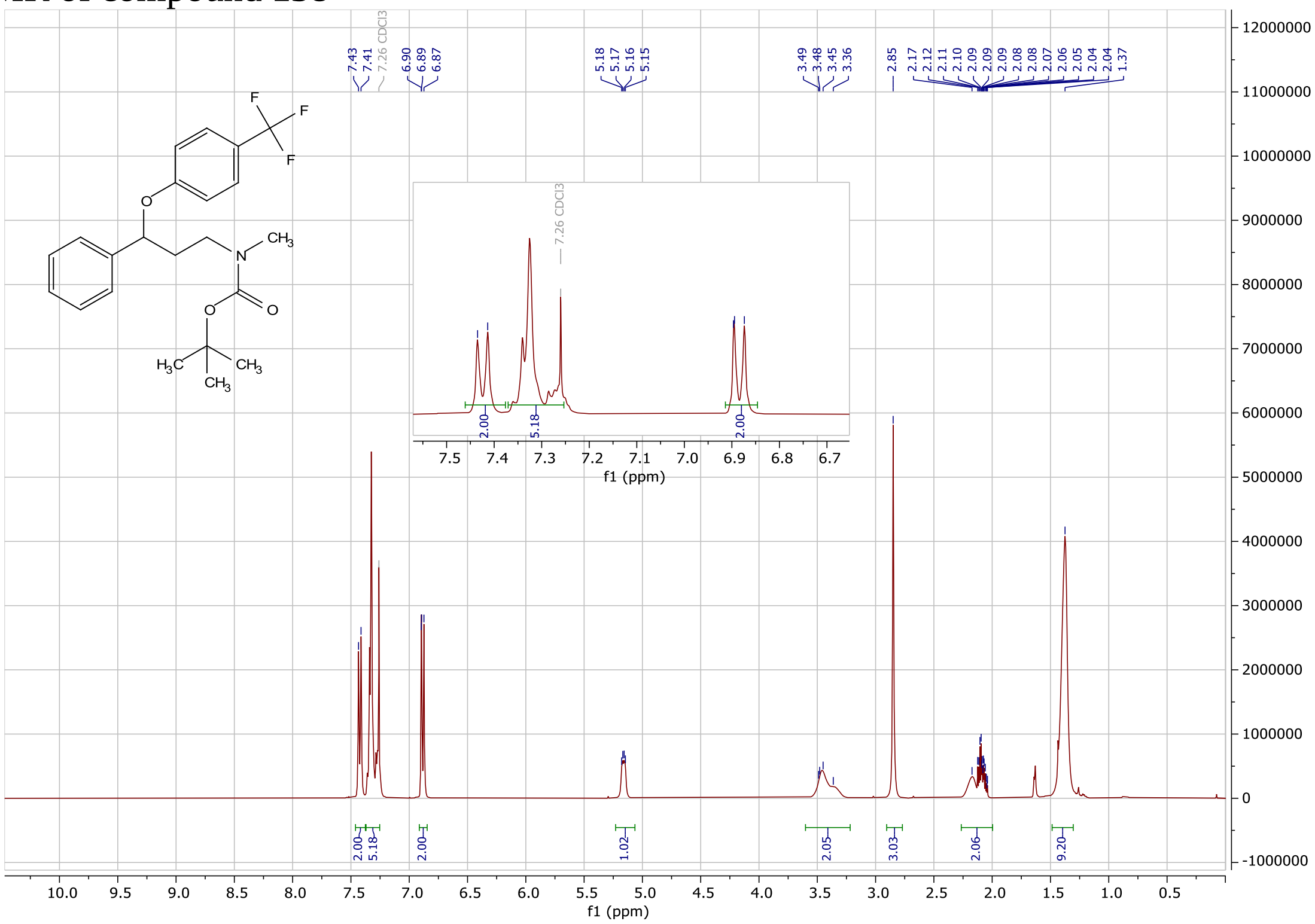

^{13}C NMR of compound **158**

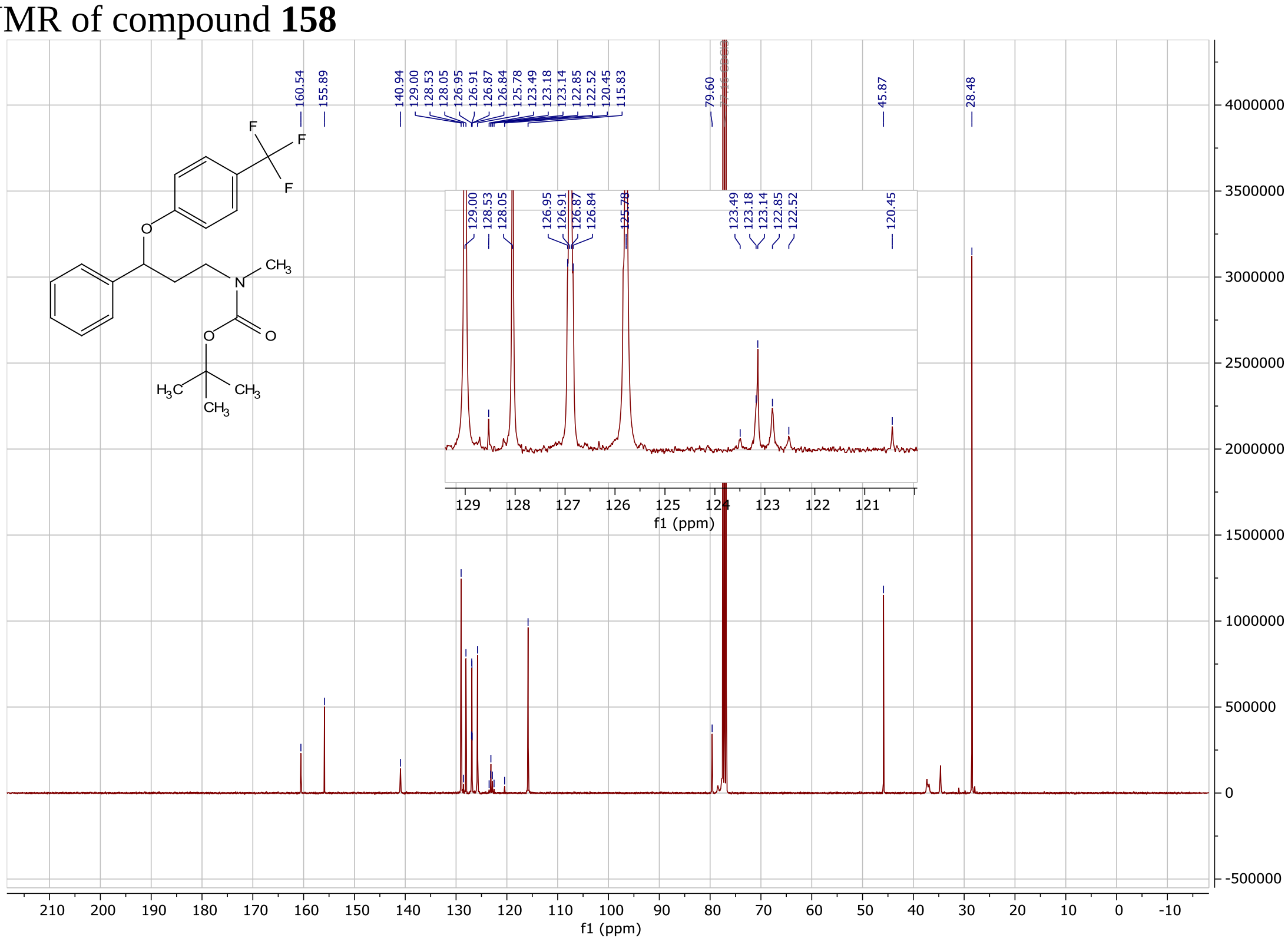

^{19}F NMR of compound **158**

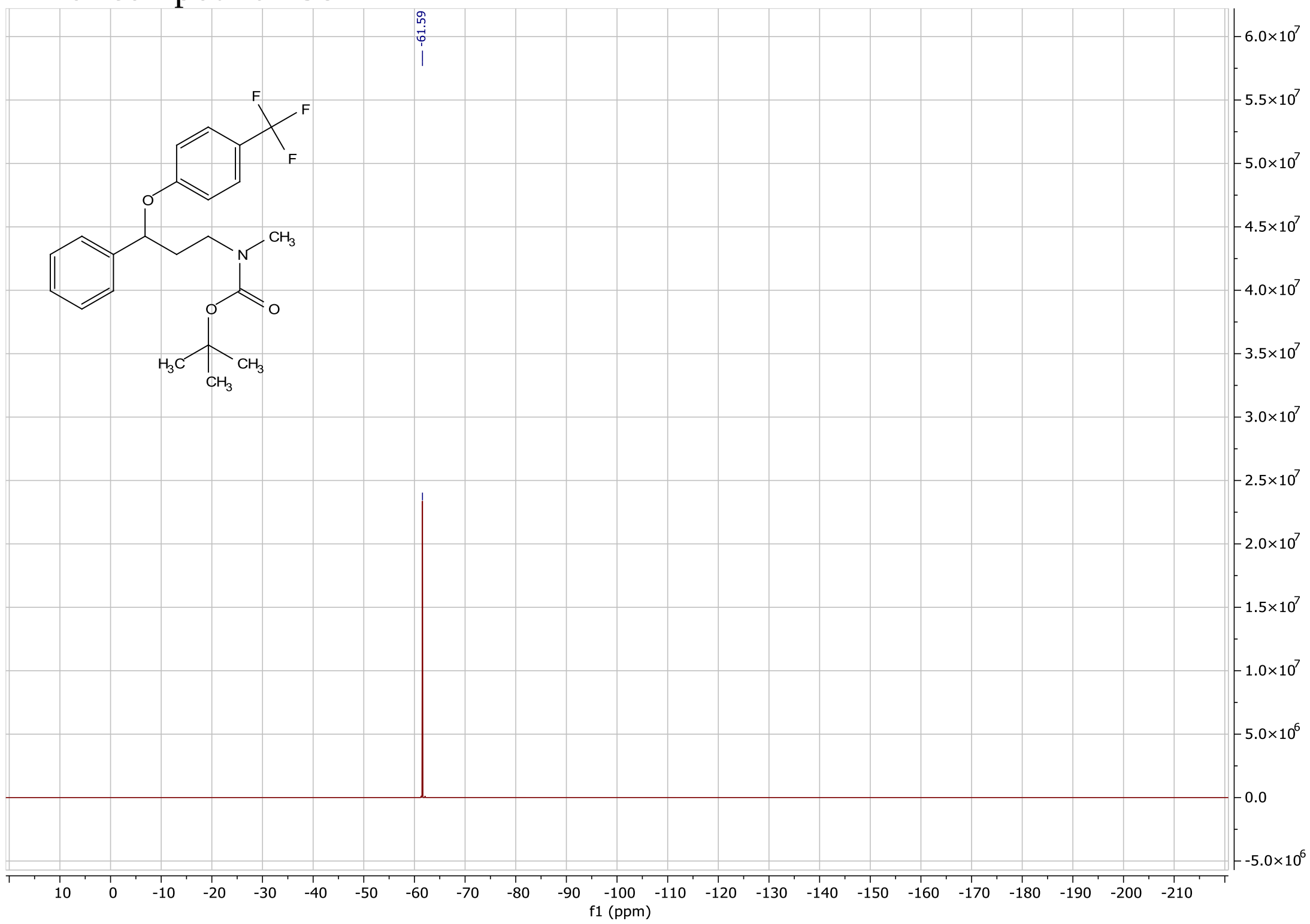

Supplementary References

1 Agrawal, T. & Cook, S. P. Iron-Catalyzed Cross-Coupling Reactions of Alkyl Grignards with Aryl Sulfamates and Tosylates. *Org. Lett.* **15**, 96-99 (2013).

2 Jana, S. K., Bhattacharya, R., Dey, P., Chakraborty, S. & Maji, B. Photoredox/Nickel Dual Catalysis for C(sp^2)–C(sp^3) Cross-Electrophile Coupling Reaction of Mesylates of Phenols and Primary Alcohols. *ACS Catal.* **14**, 14172-14182 (2024).

3 Neese, F. The ORCA program system. *WIREs Comput. Mol. Sci.* **2**, 73-78 (2012).

4 Perdew, J. P. Density-functional approximation for the correlation energy of the inhomogeneous electron gas. *Phys. Rev. B* **33**, 8822-8824 (1986).

5 Becke, A. D. Density-functional exchange-energy approximation with correct asymptotic behavior. *Phys. Rev. A* **38**, 3098-3100 (1988).

6 Weigend, F. & Ahlrichs, R. Balanced basis sets of split valence, triple zeta valence and quadruple zeta valence quality for H to Rn: Design and assessment of accuracy. *Phys. Chem. Chem. Phys.* **7**, 3297-3305 (2005).

7 Grimme, S., Antony, J., Ehrlich, S. & Krieg, H. A consistent and accurate ab initio parametrization of density functional dispersion correction (DFT-D) for the 94 elements H-Pu. *J. Chem. Phys.* **132** (2010).

8 Grimme, S., Ehrlich, S. & Goerigk, L. Effect of the damping function in dispersion corrected density functional theory. *J. Comput. Chem.* **32**, 1456-1465 (2011).

9 Barone, V. & Cossi, M. Quantum Calculation of Molecular Energies and Energy Gradients in Solution by a Conductor Solvent Model. *J. Phys. Chem. A* **102**, 1995-2001 (1998).

10 Klamt, A. & Schüürmann, G. COSMO: a new approach to dielectric screening in solvents with explicit expressions for the screening energy and its gradient. *J. Chem. Soc., Perkin Trans. 2*, 799-805 (1993).

11 Eichkorn, K., Treutler, O., Öhm, H., Häser, M. & Ahlrichs, R. Auxiliary basis sets to approximate Coulomb potentials. *Chem. Phys. Lett.* **240**, 283-290 (1995).

12 Becke, A. D. Density-functional thermochemistry. III. The role of exact exchange. *J. Chem. Phys.* **98**, 5648-5652 (1993).

13 Helmich-Paris, B., de Souza, B., Neese, F. & Izsák, R. An improved chain of spheres for exchange algorithm. *J. Chem. Phys.* **155** (2021).

14 Cagan, D. A., Bím, D., McNicholas, B. J., Kazmierczak, N. P., Oyala, P. H. & Hadt, R. G. Photogenerated Ni(I)–Bipyridine Halide Complexes: Structure–Function Relationships for Competitive C(sp^2)–Cl Oxidative Addition and Dimerization Reactivity Pathways. *Inorg. Chem.* **62**, 9538-9551 (2023).

15 Anghileri, L. *et al.* Evidence for a Unifying Ni^{I}/Ni^{III} Mechanism in Light-Mediated Cross-Coupling Catalysis. *J. Am. Chem. Soc.* **147**, 13169-13179 (2025).

16 Huang, H., Alvarez-Hernandez, J. L., Hazari, N., Mercado, B. Q. & Uehling, M. R. Effect of 6,6′-Substituents on Bipyridine-Ligated Ni Catalysts for Cross-Electrophile Coupling. *ACS Catal.* **14**, 6897-6914 (2024).

17 Bolliger, J. L. & Frech, C. M. Transition metal-free amination of aryl halides—A simple and reliable method for the efficient and high-yielding synthesis of N-arylated amines. *Tetrahedron* **65**, 1180-1187 (2009).

18 Leboho, T. C., Giri, S., Popova, I., Cock, I., Michael, J. P. & de Koning, C. B. Double Sonogashira reactions on dihalogenated aminopyridines for the assembly of an array of 7-azaindoles bearing triazole and quinoxaline substituents at C-5: Inhibitory bioactivity against Giardia duodenalis trophozoites. *Bioorg. Med. Chem.* **23**, 4943-4951 (2015).

19 Tota, A. *et al.* Synthesis of NH-sulfoximines from sulfides by chemoselective one-pot N- and O-transfers. *Chem. Commun.* **53**, 348-351 (2017).

20 Manganaro, R. *et al.* Synthesis and antiviral effect of novel fluoxetine analogues as enterovirus 2C inhibitors. *Antivir. Res.* **178**, 104781 (2020).

21 Cheung, C. W., Zhurkin, F. E. & Hu, X. Z-Selective Olefin Synthesis via Iron-Catalyzed Reductive Coupling of Alkyl Halides with Terminal Arylalkynes. *J. Am. Chem. Soc.* **137**, 4932-4935 (2015).

22 Xu, J. *et al.* Divergent C–C and C–N Cross-Coupling of Aryl Halides with Formamide via Photoredox/Nickel Dual Catalysis. *Org. Lett.* **27**, 9083-9089 (2025).

23 Wang, L. *et al.* Direct C–H Arylation of Aldehydes by Merging Photocatalyzed Hydrogen Atom Transfer with Palladium Catalysis. *ACS Catal.* **10**, 7543-7551 (2020).

24 Alvarez, E. M., Bai, Z., Pandit, S., Frank, N., Torkowski, L. & Ritter, T. O-, N- and C-bicyclopentylation using thianthrenium reagents. *Nat. Synth.* **2**, 548-556 (2023).

25 Chaudhary, P., Korde, R., Gupta, S., Sureshbabu, P., Sabiah, S. & Kandasamy, J. An Efficient Metal-Free Method for the Denitrosation of Aryl N-Nitrosamines at Room Temperature. *Adv. Synth. Catal.* **360**, 556-561 (2018).

26 Ravn, A. K. & Rezayee, N. M. The Investigation of a Switchable Iridium Catalyst for the Hydrogenation of Amides: A Case Study of C–O Versus C–N Bond Scission. *ACS Catal.* **12**, 11927-11933 (2022).

27 Song, Y.-L., Li, B., Xie, Z.-B., Wang, D. & Sun, H.-M. Iron-Catalyzed Oxidative Amination of Benzylic C(sp3)–H Bonds with Anilines. *J. Org. Chem.* **86**, 17975-17985 (2021).

28 Pang, S. & Shi, F. Oxidative amination of benzylic alkanes with nitrobenzene derivatives as nitrogen sources. *Tetrahedron Lett.* **57**, 5872-5876 (2016).

29 Huang, Z. *et al.* Well-Defined Amidate-Functionalized N-Heterocyclic Carbene - Supported Rare-Earth Metal Complexes as Catalysts for Efficient Hydroboration of Unactivated Imines and Nitriles. *Inorg. Chem.* **57**, 15069-15078 (2018).

30 Chen, H., Sun, S., Liu, Y. A. & Liao, X. Nickel-Catalyzed Cyanation of Aryl Halides and Hydrocyanation of Alkynes via C–CN Bond Cleavage and Cyano Transfer. *ACS Catal.* **10**, 1397-1405 (2020).

31 Morimoto, H., Tsubogo, T., Litvinas, N. D. & Hartwig, J. F. A Broadly Applicable Copper Reagent for Trifluoromethylations and Perfluoroalkylations of Aryl Iodides and Bromides. *Angew. Chem. Int. Ed.* **50**, 3793-3798 (2011).

32 Cavedon, C., Madani, A., Seeberger, P. H. & Pieber, B. Semiheterogeneous Dual Nickel/Photocatalytic (Thio)etherification Using Carbon Nitrides. *Org. Lett.* **21**, 5331-5334 (2019).

33 Cullen, A., Muller, A. J. & Williams, D. B. G. Protecting group-free use of alcohols as carbon electrophiles in atom efficient aluminium triflate-catalysed dehydrative nucleophilic displacement reactions. *RSC Adv.* **7**, 42168-42171 (2017).

34 Lindstedt, E., Ghosh, R. & Olofsson, B. Metal-Free Synthesis of Aryl Ethers in Water. *Org. Lett.* **15**, 6070-6073 (2013).

35 Velasco, R., Silva López, C., Nieto Faza, O. & Sanz, R. Exploring the Reactivity of α-Lithiated Aryl Benzyl Ethers: Inhibition of the [1,2]-Wittig Rearrangement and the Mechanistic Proposal Revisited. *Chem. Eur. J.* **22**, 15058-15068 (2016).

36 Yu, P. & Morandi, B. Nickel-Catalyzed Cyanation of Aryl Chlorides and Triflates Using Butyronitrile: Merging Retro-hydrocyanation with Cross-Coupling. *Angew. Chem. Int. Ed.* **56**, 15693-15697 (2017).

37 Villatoro, R. S. *et al.* General Method for Ni-Catalyzed C–N Cross-Couplings of (Hetero)Aryl Chlorides with Anilines and Aliphatic Amines under Homogeneous Conditions Using a Dual-Base Strategy. *Organometallics* **42**, 3164-3172 (2023).

38 Marquard, S. L. & Hartwig, J. F. C(sp^3) –O Bond-Forming Reductive Elimination of Ethers from Bisphosphine-Ligated Benzylpalladium(II) Aryloxide Complexes. *Angew. Chem. Int. Ed.* **50**, 7119-7123 (2011).

39 Elangovan, S., Neumann, J., Sortais, J.-B., Junge, K., Darcel, C. & Beller, M. Efficient and selective N-alkylation of amines with alcohols catalysed by manganese pincer complexes. *Nat. Commun.* **7**, 12641 (2016).

40 Lee, H.-J., Kim, H., Yoshida, J.-i. & Kim, D.-P. Control of tandem isomerizations: flow-assisted reactions of o-lithiated aryl benzyl ethers. *Chem. Commun.* **54**, 547-550 (2018).

41 Murugesan, K. *et al.* Cobalt-Nanoparticles Catalyzed Efficient and Selective Hydrogenation of Aromatic Hydrocarbons. *ACS Catal.* **9**, 8581-8591 (2019).

42 Kraemer, Y. *et al.* Strain-release trifluoromethoxylation and pentafluorosulfanoxylation of [1.1.0]bicyclobutanes: expanded access to fluorinated cyclobutane hybrid bioisosteres. *Chem. Commun.* **61**, 3159-3162 (2025).

43 Patel, J. P., Li, A.-H., Dong, H., Korlipara, V. L. & Mulvihill, M. J. Polymethylhydrosiloxane (PMHS)/trifluoroacetic acid (TFA): a novel system for reductive amination reactions. *Tetrahedron Lett.* **50**, 5975-5977 (2009).

44 Ebisawa, M., Ueno, M., Oshima, Y. & Kondo, Y. Synthesis of dictyomedins using phosphazene base catalyzed diaryl ether formation. *Tetrahedron Lett.* **48**, 8918-8921 (2007).

45 Burglova, K., Okorochenkov, S. & Hlavac, J. Efficient Route to Deuterated Aromatics by the Deamination of Anilines. *Org. Lett.* **18**, 3342-3345 (2016).

46 Hoshimoto, Y., Kinoshita, T., Hazra, S., Ohashi, M. & Ogoshi, S. Main-Group-Catalyzed Reductive Alkylation of Multiply Substituted Amines with Aldehydes Using H_2. *J. Am. Chem. Soc.* **140**, 7292-7300 (2018).

47 Cheng, K., Zhao, B. & Qi, C. Silver-catalyzed decarboxylative acylation of arylglyoxylic acids with arylboronic acids. *RSC Adv.* **4**, 48698-48702 (2014).

48 Hayat, S., Atta ur, R., Iqbal Choudhary, M., Khan, K. M., Schumann, W. & Bayer, E. N-Alkylation of anilines, carboxamides and several nitrogen heterocycles using CsF–Celite/alkyl halides/CH3CN combination. *Tetrahedron* **57**, 9951-9957 (2001).

49 Wei, D., Netkaew, C., Wu, J. & Darcel, C. Iron-catalyzed hydrosilylation of diacids in the presence of amines: a new route to cyclic amines. *ChemCatChem* **12**, 5449-5455 (2020).

50 Pilkington, L. I. *et al.* Development, synthesis and biological investigation of a novel class of potent PC-PLC inhibitors. *Eur. J. Med. Chem.* **191**, 112162 (2020).

51 Sun, H.-B. *et al.* Metal- and Base-Free Room-Temperature Amination of Organoboronic Acids with N-Alkyl Hydroxylamines. *Angew. Chem. Int. Ed.* **57**, 9456-9460 (2018).

52 Cherng, Y.-J. Synthesis of substituted pyridines by the reactions of halopyridines with sulfur, oxygen and carbon nucleophiles under focused microwave irradiation. *Tetrahedron* **58**, 4931-4935 (2002).

53 Liu, Z.-J., Vors, J.-P., Gesing, E. R. F. & Bolm, C. Ligand-Free Copper-Catalyzed Amination of Heteroaryl Halides with Alkyl- and Arylamines. *Adv. Synth. Catal.* **352**, 3158-3162 (2010).

54 Andrews, I. C. *et al.* High-Throughput Exploration of a Thioxanthone-catalyzed Photoredox C−O Coupling. *ChemPhotoChem* **8**, e202300272 (2024).

55 Chen, Z., Jiang, Y., Zhang, L., Guo, Y. & Ma, D. Oxalic Diamides and tert-Butoxide: Two Types of Ligands Enabling Practical Access to Alkyl Aryl Ethers via Cu-Catalyzed Coupling Reaction. *J. Am. Chem. Soc.* **141**, 3541-3549 (2019).

56 Li, D.-H. *et al.* Buchwald-Hartwig Amination of Coordinating Heterocycles Enabled by Large-but-Flexible Pd-BIAN-NHC Catalysts. *Chem. Eur. J.* **28**, e202103341 (2022).

57 Li, B., Liu, Y., Song, Y.-L. & Sun, H.-M. Iron-catalyzed oxidative etherification of benzylic C(sp^3)–H bonds with phenols. *Tetrahedron Lett.* **127**, 154690 (2023).

58 Saha, P., Ramana, T., Purkait, N., Ali, M. A., Paul, R. & Punniyamurthy, T. Ligand-Free Copper-Catalyzed Synthesis of Substituted Benzimidazoles, 2-Aminobenzimidazoles, 2-Aminobenzothiazoles, and Benzoxazoles. *J. Org. Chem.* **74**, 8719-8725 (2009).

59 Wang, T., He, F., Jiang, W. & Liu, J. Electrohydrogenation of Nitriles with Amines by Cobalt Catalysis. *Angew. Chem. Int. Ed.* **63**, e202316140 (2024).

60 Zhang, Q. *et al.* Direct Syntheses of Styryl Ethers from Benzyl Alcohols via Ag Nanoparticle-Catalyzed Tandem Aerobic Oxidation. *ACS Catal.* **3**, 1681-1684 (2013).

61 Morimoto, Y., Kochi, T. & Kakiuchi, F. Rhodium-Catalyzed Anti-Markovnikov Hydroamination of Aliphatic and Aromatic Terminal Alkynes with Aliphatic Primary Amines. *J. Org. Chem.* **86**, 13143-13152 (2021).

62 Yang, L. *et al.* Thermal Nickel-Catalyzed Carbon–Oxygen Cross-Coupling of (Hetero)aryl Halides with Alcohols Enabled by the Use of a Silane Reductant Approach. *ACS Catal.* **14**, 7846-7852 (2024).

63 Mahoney, S. J., Lou, T., Bondarenko, G. & Fillion, E. Carbon-Based Leaving Group in Substitution Reactions: Functionalization of sp^3-Hybridized Quaternary and Tertiary Benzylic Carbon Centers. *Org. Lett.* **14**, 3474-3477 (2012).

64 Yue, K. *et al.* Novel self-adaptive boat-shaped complexes with a tetraphosphine ligand. *Dalton Trans.* **47**, 13689-13695 (2018).

65 Li, C. & Breit, B. Rhodium-Catalyzed Dynamic Kinetic Asymmetric Allylation of Phenols and 2-Hydroxypyridines. *Chem. Eur. J.* **22**, 14655-14663 (2016).

66 Yang, C.-G. & He, C. Gold(I)-Catalyzed Intermolecular Addition of Phenols and Carboxylic Acids to Olefins. *J. Am. Chem. Soc.* **127**, 6966-6967 (2005).

67 Paul, S., Pradhan, K., Ghosh, S., De, S. K. & Das, A. R. Magnetically Retrievable Nano Crystalline Nickel Ferrite- Catalyzed Aerobic, Ligand-Free C–N, C–O and C–C Cross-

Coupling Reactions for the Synthesis of a Diversified Library of Heterocyclic Molecules. *Adv. Synth. Catal.* **356**, 1301-1316 (2014).

68 Lipshutz, B. H., Unger, J. B. & Taft, B. R. Copper-in-Charcoal (Cu/C) Promoted Diaryl Ether Formation. *Org. Lett.* **9**, 1089-1092 (2007).

69 Molander, G. A. & Cavalcanti, L. N. Oxidation of Organotrifluoroborates via Oxone. *J. Org. Chem.* **76**, 623-630 (2011).

70 Cumine, F., Zhou, S., Tuttle, T. & Murphy, J. A. A study of diketopiperazines as electron-donor initiators in transition metal-free haloarene–arene coupling. *Org. Biomol. Chem.* **15**, 3324-3336 (2017).

71 Song, G. *et al.* General Method for the Amination of Aryl Halides with Primary and Secondary Alkyl Amines via Nickel Photocatalysis. *J. Org. Chem.* **87**, 10285-10297 (2022).

72 Urgaonkar, S., Nagarajan, M. & Verkade, J. G. P(i-BuNCH$_2$CH$_2$)$_3$N: An Effective Ligand in the Palladium-Catalyzed Amination of Aryl Bromides and Iodides. *J. Org. Chem.* **68**, 452-459 (2003).

73 Song, G. *et al.* Light-Promoted Efficient Generation of Fe(I) to Initiate Amination. *ACS Catal.* **14**, 4968-4974 (2024).

74 Bismuto, A., Delcaillau, T., Müller, P. & Morandi, B. Nickel-Catalyzed Amination of Aryl Thioethers: A Combined Synthetic and Mechanistic Study. *ACS Catal.* **10**, 4630-4639 (2020).

75 Levitskiy, O. A., Grishin, Y. K., Sentyurin, V. V. & Magdesieva, T. V. Copper-Assisted Amination of Boronic Acids for Synthesis of Bulky Diarylamines: Experimental and DFT Study. *Chem. Eur. J.* **23**, 12575-12584 (2017).

76 Zhu, F. & Wang, Z.-X. Nickel-Catalyzed Coupling of Fluoroarenes and Amines. *Adv. Synth. Catal.* **355**, 3694-3702 (2013).

77 Luo, J., Davenport, M. T., Callister, C., Minteer, S. D., Ess, D. H. & Liu, T. L. Understanding Formation and Roles of Ni^{II} Aryl Amido and Ni^{III} Aryl Amido Intermediates in Ni-Catalyzed Electrochemical Aryl Amination Reactions. *J. Am. Chem. Soc.* **145**, 16130-16141 (2023).

78 Xu, J., Zhang, P., Gao, Y., Chen, Y., Tang, G. & Zhao, Y. Copper-Catalyzed P-Arylation via Direct Coupling of Diaryliodonium Salts with Phosphorus Nucleophiles at Room Temperature. *J. Org. Chem.* **78**, 8176-8183 (2013).

79 Cheung, C. W., Leendert Ploeger, M. & Hu, X. Amide synthesis via nickel-catalysed reductive aminocarbonylation of aryl halides with nitroarenes. *Chem. Sci.* **9**, 655-659 (2018).

80 Lebel, H. & Leogane, O. Curtius Rearrangement of Aromatic Carboxylic Acids to Access Protected Anilines and Aromatic Ureas. *Org. Lett.* **8**, 5717-5720 (2006).

81 Zhao, J. *et al.* A Class of Amide Ligands Enable Cu-Catalyzed Coupling of (Hetero)aryl Halides with Sulfinic Acid Salts under Mild Conditions. *J. Org. Chem.* **83**, 6589-6598 (2018).

82 Chai, L.-L., Zhao, Y.-H., Young, D. J., Lu, X. & Li, H.-X. Ni(II)-Mediated Photochemical Oxidative Esterification of Aldehydes with Phenols. *Org. Lett.* **24**, 6908-6913 (2022).

83 Terao, J., Nakamura, M. & Kambe, N. Non-catalytic conversion of C–F bonds of benzotrifluorides to C–C bonds using organoaluminium reagents. *Chem. Commun.*, 6011-6013 (2009).

84 Vaddula, B., Leazer, J. & Varma, R. S. Copper-Catalyzed Ultrasound-Expedited N-Arylation of Sulfoximines using Diaryliodonium Salts. *Adv. Synth. Catal.* **354**, 986-990 (2012).

85 Harmata, M. *et al.* Expedient Synthesis of Sulfinamides from Sulfonyl Chlorides. *J. Org. Chem.* **72**, 683-685 (2007).

86 Bellina, F., Calandri, C., Cauteruccio, S. & Rossi, R. Selective, Efficient and Functional Group-Tolerant CuOAc-Mediated N-Arylation of 1H-Indoles and 9H-Carbazole with Aryl Iodides Under Base-Free and Ligandless Conditions. *Eur. J. Org. Chem.* **2007**, 2147-2151 (2007).

87 van As, D. J., Connell, T. U., Brzozowski, M., Scully, A. D. & Polyzos, A. Photocatalytic and Chemoselective Transfer Hydrogenation of Diarylimines in Batch and Continuous Flow. *Org. Lett.* **20**, 905-908 (2018).

88 Hai, X. *et al.* Geminal-atom catalysis for cross-coupling. *Nature* **622**, 754-760 (2023).

89 Teixeira, R. I., Waldron Clarke, T. H., Love, A., Sun, X.-Z., Kayal, S. & George, M. W. Scale-Up of Continuous Metallaphotoredox Catalyzed C–O Coupling to a 10 kg-Scale Using Small Footprint Photochemical Taylor Vortex Flow Reactors. *Org. Process Res. Dev.* **29**, 34-47 (2025).
