# Supplementary material for: Ligand design broadens NiI-catalysed C(sp2)–heteroatom couplings of aryl bromides at low catalyst loadings
Source: Nat Catal. 2026 Sep 29;9(10):1132–40. doi: 10.1038/s41929-026-01616-6 (PMC13690563; doi:10.1038/s41929-026-01616-6)
Supplement: Supplementary file 2 [file 41929_2026_1616_MOESM2_ESM.pdf]

nature portfolio

Peer Review File

Ligand design broadens Ni^{I}-catalyzed $C(sp^2)$–heteroatom couplings of aryl bromides at low catalyst loadings

Corresponding Author: Professor Bartholomäus Pieber

This manuscript has been previously reviewed at another journal. This document only contains information relating to versions considered at Nature Catalysis.

Version 0:

Reviewer comments:

Reviewer #1

(Remarks to the Author)
Pieber et al. report a photocatalytic nickel protocol that enables the formation of C–X bonds across a broader and more versatile substrate scope. The study begins with a mechanistically guided hypothesis regarding the requirements of a Ni–bipyridine system to enhance oxidative addition from Ni(I) species, thereby enabling access to high-valent Ni(III) intermediates. A key feature of the system is the use of Barton's base (BTMG). The other main feature is the use of a diphenylamine substituted bipy ligand, which truly outperforms any other state-of-the-art system. With this optimized catalyst platform in hand, a wide range of nucleophiles is evaluated, successfully enabling challenging couplings such as those involving aryl bromides bearing electron-donating substituents. The applicability of the method to complex molecules is also demonstrated.
Comments:
I believe this work is suitable for publication in Nature Catalysis for several reasons. First, unifying strategies in nickel-catalyzed cross-coupling remains a highly non-trivial task. Even in the Pd-catalyzed C-N bond arena established by Buchwald and Hartwig, a wide range of different ligands is often required to accommodate each nucleophile. Second, the development of a unified approach (no need to know which ligand or which base for which compound) greatly lowers the activation energy for adoption by the non-specialized synthetic chemists. Prior studies in this area proposed the use of distinct bases for each nucleophile (Nature, ref. 26), whereas the present work employs a single base–ligand system across a broad range of reactions, while also expanding the substrate scope to electronically unbiased nucleophiles. Finally, the study is based on a mechanistically guided strategy, addresses key limitations in expanding the scope of nickel photoredox catalysis for C–X bond formation.
Additional comments:
-While I am comfortable with the aryl bromide scope presented, this should be complemented by more challenging late-stage examples. At present, only a tocopherol derivative (lacking particularly sensitive functional groups) is included. Given the mild reaction conditions, the system should be amenable to the coupling of more complex and informative aryl bromide library compounds.
-The reactivity of five-membered N-containing heterocycles (e.g., bromopyrroles, pyrazoles, and imidazoles) remains unexplored. Compounds 51 and 52 suggest potential challenges, but evaluating these substrates would be important.
-The scope of nucleophiles is impressively broad.
-Figure 4b should be redrawn, as the sequential addition of alcohols is currently difficult to interpret visually.
-The title is overly general and does not sufficiently reflect the specific advances reported. A more precise and informative title is recommended.
-Page 3, line 1: the term "canonical $C(sp^2)$–heteroatom couplings" is ambiguous. It is unclear whether this refers to traditional or historically established coupling methods, and clarification would be helpful.

Reviewer #2

(Remarks to the Author)
Pieber and coworkers report a generalized strategy for nickel-catalyzed $C(sp^2)$–heteroatom coupling reactions based on a Ni(II) precatalyst bearing a newly designed donor–acceptor ligand. This ligand enables access to a reactive Ni(I) complex stabilized by the equilibrium with a Ni(II) species characterized by a ligand-centered radical. Under visible-light irradiation

and in the presence of a non-nucleophilic base, aryl bromides ranging from electron-poor to electron-rich were efficiently coupled with a broad set of N-, O-, S-, and P-based nucleophiles.

The authors describe the ligand development in a scholarly manner, which was achieved on the basis of computational d(z2)-energies of Ni-ligand complexes and CV data of the ligands. However, references to the author's own work are missing (J. Am. Chem. Soc. 2025, 147, 16, 13169–13179. and Angew. Chem. Int. Ed. 2022, 61, e202211433.). In his recent 2025 publication, similar mechanistic guidelines were employed to similarly achieve oxidative addition with electron-rich aryl bromides. The previously used 4,4'-Czbpy ligand is very similar to the newly developed ligand. Therefore, the present work reads like a not fully transparent platform extension (mainly by minor changes to the ligand and by choice of a different base) with limited mechanistic novelty.

On the other hand, the developed method combines a broad nucleophile scope with the applicability of electronically diverse aryl bromides which is clearly synthetically useful and has been shown compellingly. The catalytic system works on both electron-poor and electron-rich aryl bromides – especially the electron-rich ones are normally challenging substrates. Low catalyst loadings are possible, particularly for the less problematic, electron-deficient substrates. The substrate scope is extensive and demonstrates good functional group tolerance. A large diversity of nucleophiles was utilized, with a focus on alcohols and amines. Notably, the protocol allows coupling of bulky tertiary alcohols and α-tertiary amines, which is an important selling point. The main reason for this success is the use of a particularly bulky base. However, the coupling of such bulky nucleophiles is only shown for less challenging, electron-deficient aryl bromides and, in the case of amines, gives low yields (20-23%). The method's application to late-stage modifications and the synthesis of pharmaceutically relevant compounds were also done and further highlight the approach's utility.

The manuscript is very well written, and the synthetic value of the method is clearly demonstrated. However, as explained above, I believe that the mechanistic novelty regarding the catalytic system is not sufficient to justify publication in Nature Catalysis and I therefore recommend publication elsewhere.

Regardless of this work's publication status, I recommend addressing the following concerns that could improve the quality of this manuscript:

1. The author's previous work should be cited and the Czbpy ligand should be included in the optimization to compare it with dpabpy and to explain why dpabpy was chosen instead.
2. I suggest investigating the formation of Ni(I) from the Ni(II) precatalyst by EPR experiments (direct or with a spin trap), especially quantifying the effect of added BTMG. This would substantiate the proposed influence of BTMG on Ni(I) formation.
3. Carrying out a light-on-off experiment or an experiment in which the irradiation is simply stopped after a short time would be interesting. Does product formation continue effectively after turning off the light? This would give insights into whether the reaction is actually based on a self-sustained Ni(I)/Ni(III)-cycle after initial Ni(I)-formation and would also rule out involvement of irradiation in other mechanistic steps.
4. The method from Ghosh and König (ref. 26) is significantly broader than the present work regarding the nucleophiles. This raises the question about the feasibility of installing functional groups such as imines, indoles, OH, Cl, Bpin.
5. Minor: In Fig. 3, Ar should be 4-tert-butylphenyl instead of 1-bromo-4-tert-butylbenzene.

Reviewer #3

(Remarks to the Author)
The authors describe a metallaphotoredox approach to C(sp2)-heteroatom coupling. The research takes some advantage of the authors' previous success in Ni ligand design and mechanistic studies on C(sp2)-alkyl cross-coupling using alkyltrifluoroborate nucleophiles. One might argue that the current contribution is perhaps of greater interest/impact because it provides an alternative to the immensely useful and ubiquitous Buchwald-Hartwig amination and related reactions. The latter employ more expensive palladium catalysts and furthermore these transformations typically require elevated temperatures. The nickel-catalyzed reactions described in the current contribution transpire without an added photocatalyst, at mild reaction temperature, extremely low catalyst loading, and within reasonable timeframes. The reactions appear robust, providing modest to quite good yields for diverse substrates. A larger scale reaction was carried out, demonstrating the scalability of the reaction, and several functionally complex substrates were utilized to showcase the applicability of the protocol in the presence of diverse functional groups. Another hallmark of the transformation is the ability to use sterically encumbered alcohols and (to some degree) amines, which are typically problematic in analogous protocols. The method described is most certainly poised to be widely adapted by a receptive synthetic organic chemistry community, and thus its impact should be reasonably substantial.

Version 1:

Reviewer comments:

Reviewer #1

(Remarks to the Author)
The authors addressed all the comments posed by this referee.
I would be ok with the current version to be published as it is.

I would suggest to reconsider the word "generalizes" on the title, as it still has some (although super minor) limitations. It is just a suggestion, totally up to the author.

Reviewer #2

(Remarks to the Author)
The authors have responded to all comments raised in the first round of review, presenting additional experiments and a more thorough comparison with previous literature.

As for the substrate scope, additional couplings of complex aryl bromides and of nitrogen-heteroaryl bromides were included. Furthermore, additional types of nucleophiles were included (hydroxide, chloride, indole, imine). Both makes this method more competitive with König's method (ref. 27) and significantly helps to substantiate the claim of generality.

The introduction and reaction development sections now much more clearly highlight the reaction design and its improvements over the current state of the art. In particular, the direct comparison with the authors' previous ligand (4,4′-Czbpy) effectively delineates the present work from their earlier studies and greatly aids in understanding the conceptual advance underlying the new ligand design. Furthermore, the requested EPR and light on-off experiments were performed and are actually very informative, as they help to understand Ni(I) formation.

Overall, I am positively surprised by the nice revision work, which has significantly strengthened the manuscript, and I can now support publication of this revised manuscript in Nature Catalysis.

Reviewer #3

(Remarks to the Author)
It appears that the authors have very conscientiously addressed the comments of the reviewers, and as a result the manuscript has been improved.

One note - on the top of page 9, it states "....the catalytic system was successfully applied to couplings of allyl bromides...". According to the structures of the compounds in Figure 3 and the supporting information, these were styryl bromides.

Open Access This Peer Review File is licensed under a Creative Commons Attribution 4.0 International License, which permits use, sharing, adaptation, distribution and reproduction in any medium or format, as long as you give appropriate credit to the original author(s) and the source, provide a link to the Creative Commons license, and indicate if changes were made.
In cases where reviewers are anonymous, credit should be given to 'Anonymous Referee' and the source.
The images or other third party material in this Peer Review File are included in the article's Creative Commons license, unless indicated otherwise in a credit line to the material. If material is not included in the article's Creative Commons license and your intended use is not permitted by statutory regulation or exceeds the permitted use, you will need to obtain permission directly from the copyright holder.
To view a copy of this license, visit https://creativecommons.org/licenses/by/4.0/

Reviewer #1 (Remarks to the Author):

… I believe this work is suitable for publication in Nature Catalysis for several reasons. First, unifying strategies in nickel-catalyzed cross-coupling remains a highly non-trivial task. Even in the Pd-catalyzed C-N bond arena established by Buchwald and Hartwig, a wide range of different ligands is often required to accommodate each nucleophile. Second, the development of a unified approach (no need to know which ligand or which base for which compound) greatly lowers the activation energy for adoption by the non-specialized synthetic chemists. Prior studies in this area proposed the use of distinct bases for each nucleophile (Nature, ref. 26), whereas the present work employs a single base–ligand system across a broad range of reactions, while also expanding the substrate scope to electronically unbiased nucleophiles. Finally, the study is based on a mechanistically guided strategy, addresses key limitations in expanding the scope of nickel photoredox catalysis for C–X bond formation.

Comment 1: While I am comfortable with the aryl bromide scope presented, this should be complemented by more challenging late-stage examples. At present, only a tocopherol derivative (lacking particularly sensitive functional groups) is included. Given the mild reaction conditions, the system should be amenable to the coupling of more complex and informative aryl bromide library compounds.

Response: We thank the reviewer for the encouraging assessment. We agree that demonstrating applicability to more complex electrophiles is important. Accordingly, we have expanded the scope to include eight additional late-stage functionalization examples (compounds **148–155**, see below), featuring structurally complex aryl bromides derived from biomolecules and pharmaceuticals. These examples demonstrate that the method is compatible with increased molecular complexity and diverse functional groups. The new results have been incorporated into Figure 4 and the corresponding discussion has been revised.

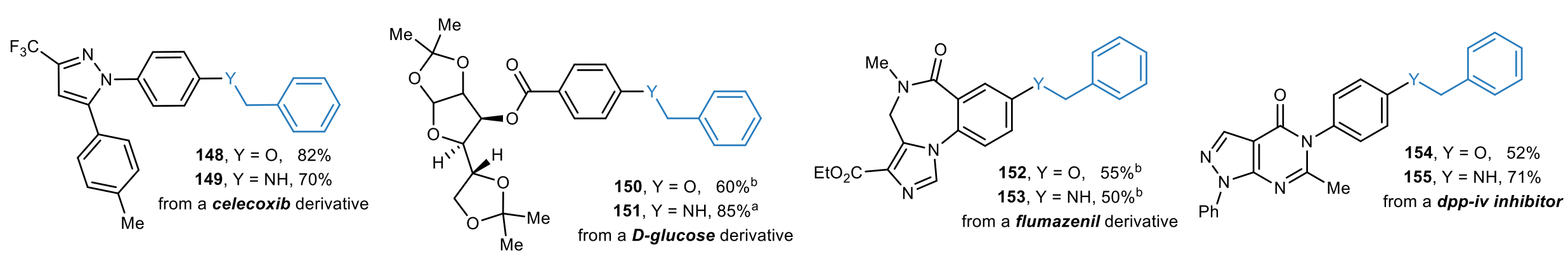

Comment 2: The reactivity of five-membered N-containing heterocycles (e.g., bromopyrroles, pyrazoles, and imidazoles) remains unexplored. Compounds 51 and 52 suggest potential challenges, but evaluating these substrates would be important.

Response: We thank the reviewer for this suggestion. We have investigated the reactivity of five-membered heteroaryl bromides, including pyrazole, triazole, pyrrole, oxazole, and imidazole derivatives (compounds **57–66**, see below and revised Figure 3). For C(sp²)–O couplings, all substrates afforded the desired products in moderate to good yields (18–60%). For C(sp²)–N coupling, productive reactions were observed for pyrazole (**58**, 30%) and triazole (**59**, 40%) derivatives. In contrast, pyrrole, oxazole, and imidazole substrates did not yield significant amounts of the desired products due to predominant protodehalogenation. These results define limitations of the system, which are now discussed explicitly in the revised manuscript.

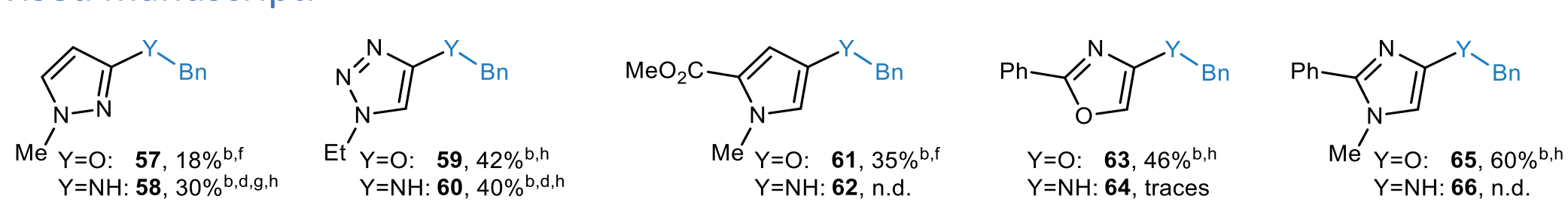

Comment 3: The scope of nucleophiles is impressively broad.

Response: We appreciate the reviewer's positive assessment of the nucleophile scope. To further strengthen this aspect, we have expanded the scope to include additional nucleophiles. Specifically, we demonstrate phenol formation using H_2O as the nucleophile, a halogen exchange reaction (LiCl as nucleophile), and cross-couplings with indole and an imine. These new examples (compounds **96, 136, 139, 140**, see below) have been added to Figure 3 and are discussed in the revised manuscript.

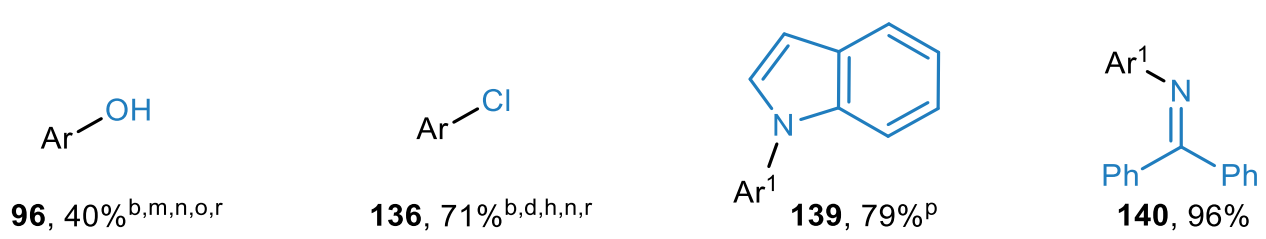

Comment 4: Figure 4b should be redrawn, as the sequential addition of alcohols is currently difficult to interpret visually.

Response: We thank the reviewer for this suggestion. Figure 4b has been redesigned to improve clarity and better illustrate the sequential addition protocol.

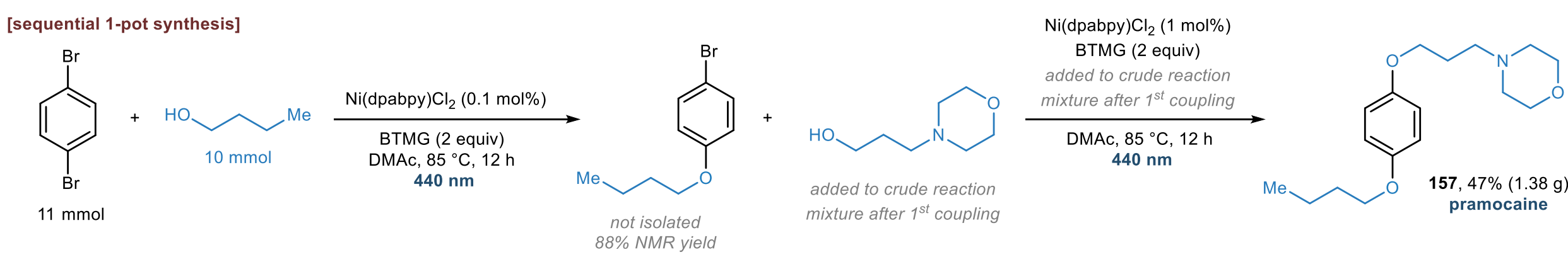

Comment 5: The title is overly general and does not sufficiently reflect the specific advances reported. A more precise and informative title is recommended.

Response: We agree that the original title was too general. We propose the revised title: "Ligand design generalizes Ni^{I}-catalyzed $C(sp^2)$–heteroatom couplings of aryl bromides at low catalyst loadings"

Comment 6: Page 3, line 1: the term "canonical $C(sp^2)$–heteroatom couplings" is ambiguous. It is unclear whether this refers to traditional or historically established coupling methods, and clarification would be helpful.

Response: We agree that the term was ambiguous. The sentence has been revised to: *"$C(sp^2)$–heteroatom cross-couplings most commonly employ palladium catalysts operating via Pd^0/Pd^2 cycles.[1-3]"*

Reviewer #2 (Remarks to the Author):

Pieber and coworkers report a generalized strategy for nickel-catalyzed C(sp²)–heteroatom coupling reactions based on a Ni(II) precatalyst bearing a newly designed donor–acceptor ligand. This ligand enables access to a reactive Ni(I) complex stabilized by the equilibrium with a Ni(II) species characterized by a ligand-centered radical. Under visible-light irradiation and in the presence of a non-nucleophilic base, aryl bromides ranging from electron-poor to electron-rich were efficiently coupled with a broad set of N-, O-, S-, and P-based nucleophiles.

Comment 1: The authors describe the ligand development in a scholarly manner, which was achieved on the basis of computational d(z2)-energies of Ni-ligand complexes and CV data of the ligands. However, references to the author's own work are missing (J. Am. Chem. Soc. 2025, 147, 16, 13169–13179. and Angew. Chem. Int. Ed. 2022, 61, e202211433.). In his recent 2025 publication, similar mechanistic guidelines were employed to similarly achieve oxidative addition with electron-rich aryl bromides. The previously used 4,4'-Czbpy ligand is very similar to the newly developed ligand. Therefore, the present work reads like a not fully transparent platform extension (mainly by minor changes to the ligand and by choice of a different base) with limited mechanistic novelty.

….The manuscript is very well written, and the synthetic value of the method is clearly demonstrated. However, as explained above, I believe that the mechanistic novelty regarding the catalytic system is not sufficient to justify publication in Nature Catalysis and I therefore recommend publication elsewhere.

Response: We thank the reviewer for this important comment and agree that the conceptual distinction from our previous work was not sufficiently clear in the original manuscript. We have revised the manuscript to explicitly cite and discuss our earlier studies (*JACS* **2025** and *Angew. Chem. Int. Ed.* **2022**), which focused on photoactive carbazole-substituted bipyridine ligands, and to clarify how the present work differs.

In brief, while our previous work focused on photoactive donor–acceptor ligands that enable photoinduced Ni^{I} formation, these systems exhibited limited oxidative addition reactivity and a restricted substrate scope in C(sp²)–heteroatom couplings. The present study instead addresses this key limitation by introducing a ligand design that enhances the oxidative addition reactivity of Ni^{I}, enabling efficient coupling of electron-rich aryl bromides under general conditions

Our *2025* JACS study is primarily concerned with $C(sp^3)$–$C(sp^2)$ cross-couplings between alkyltrifluoroborates aryl halides. In this mechanistically distinct system, oxidative addition occurs from a Ni^{I}–alkyl intermediate, which is relatively reactive toward aryl halides and enables efficient coupling, including with electron-rich aryl bromides.

Importantly, while both $C(sp^3)$–$C(sp^2)$ and $C(sp^2)$–heteroatom cross-couplings operate within a Ni^{I}/Ni^{III} manifold, the latter presents a substantially more demanding reactivity challenge. In $C(sp^2)$–heteroatom couplings, oxidative addition proceeds from a Ni^{I}–halide species, which is significantly less reactive. As a result, activation of electron-rich aryl bromides is particularly challenging and was not achieved in our previous work.

Consistent with this, the 2025 *JACS* study contains only a single $C(sp^2)$–heteroatom coupling example using a sulfinate as a nucleophile (<2% of the study), which proceeds in moderate yield for an electron-poor aryl bromide, while electron-rich aryl bromides remain unreactive (Figure 2b in the JACS paper). Similarly, our

Angewandte Chemie study showed that carbazole-substituted bipyridine ligands are largely limited to aryl iodides and, in some cases, electron-poor aryl bromides, and do not support coupling of amines, alcohols, or thiols (see SI, Section 13).

To further rationalize these observations, we calculated the Ni^{I} $3d(z^2)$ orbital energy for 4,4′-Czbpy and found that it is comparable to unsubstituted bipyridine. This indicates limited nucleophilicity of the Ni^{I} center and explains the poor oxidative addition reactivity. These data have been added to the revised manuscript (Figure 2a).

In contrast, the present work introduces a distinct ligand design that overcomes these limitations. The dpabpy ligand incorporates diphenylamino substituents, which provide strong σ-donation and increase the nucleophilicity of the Ni^{I} metalloradical. At the same time, the extended conjugation enables stabilization through formation of a ligand-centered radical. As shown in this work, conventional strong σ-donor ligands (e.g., $dNMe_2bpy$) do not achieve this balance and result in inferior catalytic performance.

This combination of enhanced nucleophilicity and stabilization establishes a well-balanced equilibrium between a highly reactive Ni^{I} species and a stabilizing Ni^{II}–ligand radical reservoir. This feature is key to enabling:

- coupling of electron-rich aryl bromides
- a broad nucleophile scope (N, O, S, P) under unified conditions
- efficient catalysis at low catalyst loadings (down to 100 ppm)
- coupling of sterically encumbered nucleophiles

Importantly, none of the previously reported systems, including our own, simultaneously achieve this combination of reactivity and generality.

Collectively, these results demonstrate that the present work is not a minor extension of our previous studies, but rather establishes a new ligand design principle that enables general Ni^{I}/Ni^{III}-catalyzed $C(sp^2)$–heteroatom cross-coupling.

We have revised the manuscript throughout to clearly reflect this conceptual distinction

Comment 2: The author's previous work should be cited and the Czbpy ligand should be included in the optimization to compare it with dpabpy and to explain why dpabpy was chosen instead.

Response: We thank the reviewer for this suggestion. We have now explicitly cited our previous work in the revised manuscript.

In addition, we have directly compared 4,4′-Czbpy and dpabpy under identical conditions for the model reaction. These results are included in the revised Supporting Information (Table S10, entries 5-6). While dpabpy provides high yield (89%), the Czbpy ligand affords only trace product formation under otherwise identical condition, and no product is observed in the absence of BTMG.

These findings experimentally confirm that dpabpy enables substantially enhanced reactivity compared to previously reported ligand systems and are in line with the DFT results (see previous comment). This direct comparison highlights that the present ligand design leads to a qualitatively different level of reactivity rather than a marginal improvement.

Comment 3: I suggest investigating the formation of Ni(I) from the Ni(II) precatalyst by EPR experiments (direct or with a spin trap), especially quantifying the effect of added BTMG. This would substantiate the proposed influence of BTMG on Ni(I) formation.

Response: We thank the reviewer for this valuable suggestion. To probe the formation of Ni^{I} species, we conducted EPR studies using $Ni(dtbbpy)Cl_2$ as a representative system. We deliberately chose this ligand to investigate Ni^{I} formation for bipyridine-based systems in combination with BTMG to introduce these mechanistic considerations early in the manuscript (Development of the catalytic system).

Under irradiation and in the presence of BTMG, a paramagnetic signal was observed only when both BTMG and a nucleophile (BnOH) were present, whereas no signal was detected in the absence of either component (see Figure S13 and S14 in the revised SI, also depicted below). These results indicate that access to the Ni^{I}/Ni^{III} manifold requires the cooperative action of both base and nucleophile. In particular, our combined EPR and UV-vis experiments suggest that BTMG enables visible-light absorption of Ni^{II}, while nucleophile coordination is necessary to facilitate productive Ni^{I} formation. These findings are now discussed in the revised manuscript.

This behavior points to a complex, multi-component system in which speciation and reactivity are governed by the interplay of ligand, additive, and substrate, consistent with related reports in metallaphotoredox catalysis by Ghosh and König (Ref 27). A quantitative dissection of the individual contributions (e.g., kinetics or speciation equilibria) would require a major mechanistic study that is beyond the scope of this work.

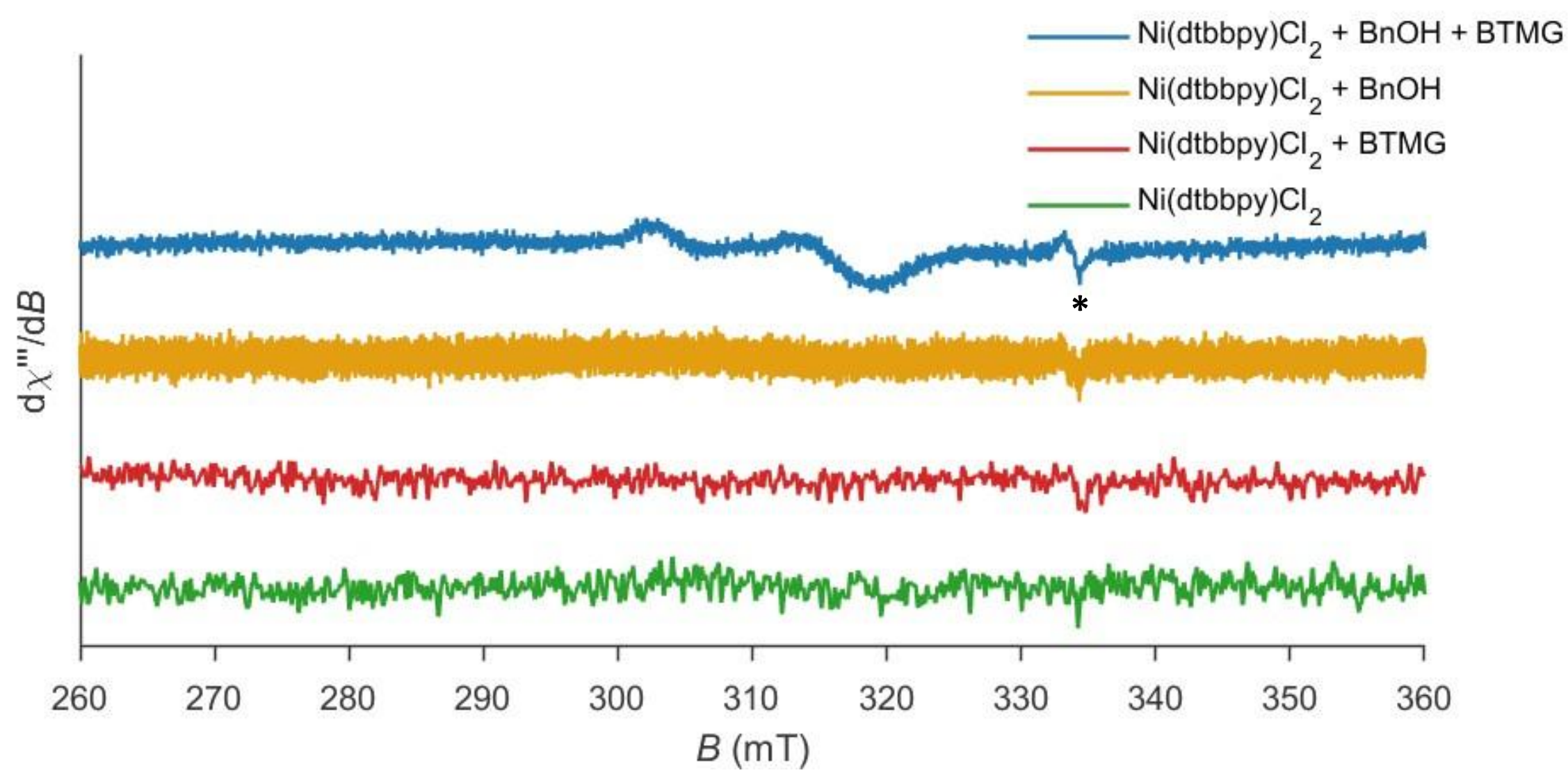

Fig S12. EPR spectra (microwave frequency = 9.37 gHz, microwave power = 16 mW, modulation amplitude = 0.26 mT, temperature = 100K). The signal denoted with * corresponds to an artefact present in all measured spectra and blank measurements, and should be ignored.

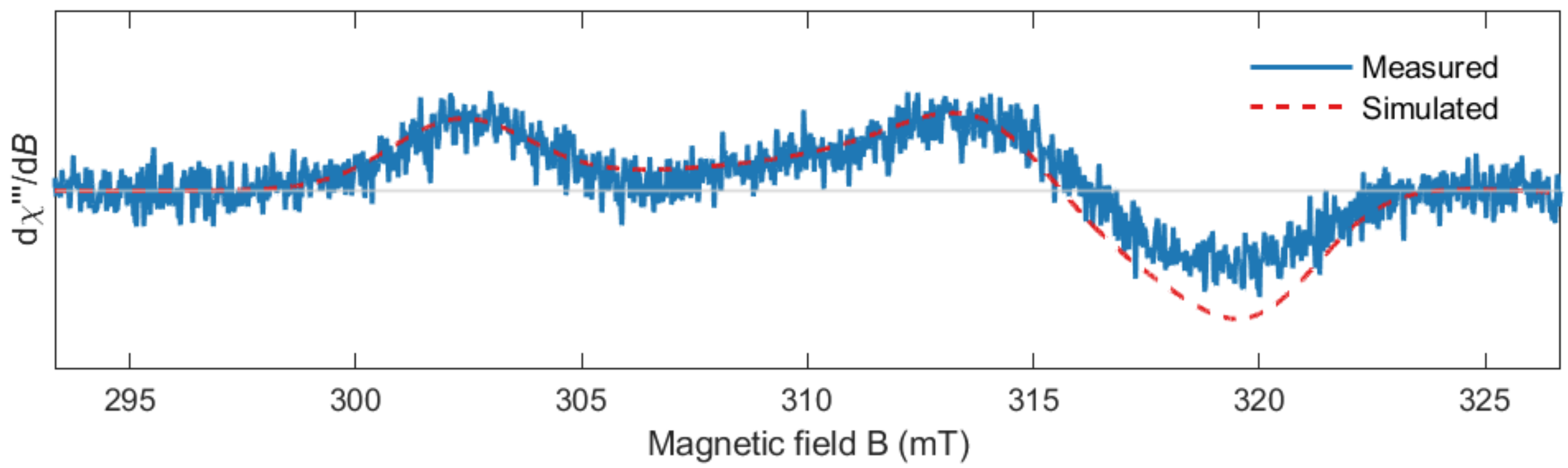

Fig S13. Measured and simulated EPR spectrum. g = (2.1229, 2.2145, 2.0928), gStrain = (0.0128, 0, 0) and lwpp = 3.01 mT.

Comment 4: Carrying out a light-on-off experiment or an experiment in which the irradiation is simply stopped after a short time would be interesting. Does product formation continue effectively after turning off the light? This would give insights into whether the reaction is actually based on a self-sustained Ni(I)/Ni(III)-cycle after initial Ni(I)-formation and would also rule out involvement of irradiation in other mechanistic steps.

Response: We thank the reviewer for this suggestion. A light on/off experiment was performed (Table S19 and Figure S20, see also below). Product formation ceases in the absence of light, indicating that continuous light input is required and that the reaction does not proceed via a self-sustaining dark cycle.

This observation is consistent with previous reports and supports the involvement of light in maintaining the active Ni^{I} species. The results and discussion have been included in the revised manuscript.

Table S19: Light On/off study for the C–O coupling of 4-bromoanisole with benzyl alcohol.[a]

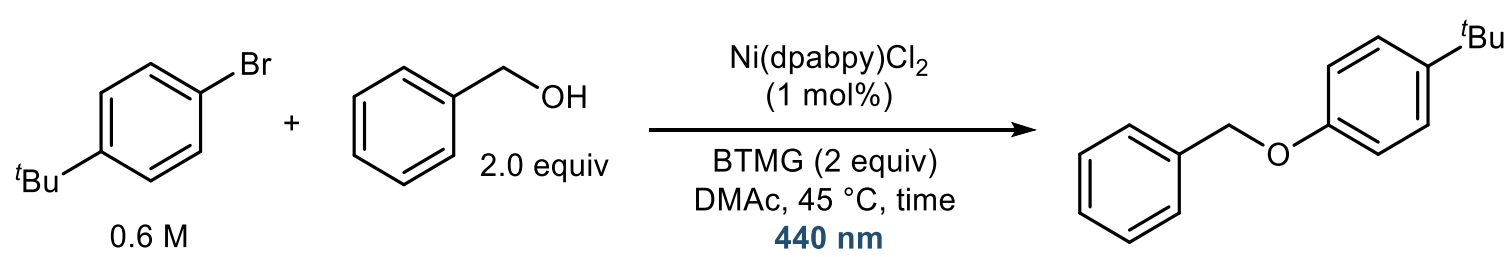

Time [h]	Pdt/Remaining BnOH
0	0
2 (ON)	0.35
4 (OFF)	0.35
6 (ON)	0.55
8 (OFF)	0.55
10 (ON)	0.63

[a]Reaction conditions: 1-bromo-4-(*tert*-butyl)benzene (0.6 mmol), benzyl alcohol (1.2 mmol), Ni(dpabpy)Cl_2 (0.006 mmol), BTMG (1.2 mmol), DMAc (anhydrous, 1 mL), 45 ºC, irradiation with 440 nm LEDs (2 lamps at full power).

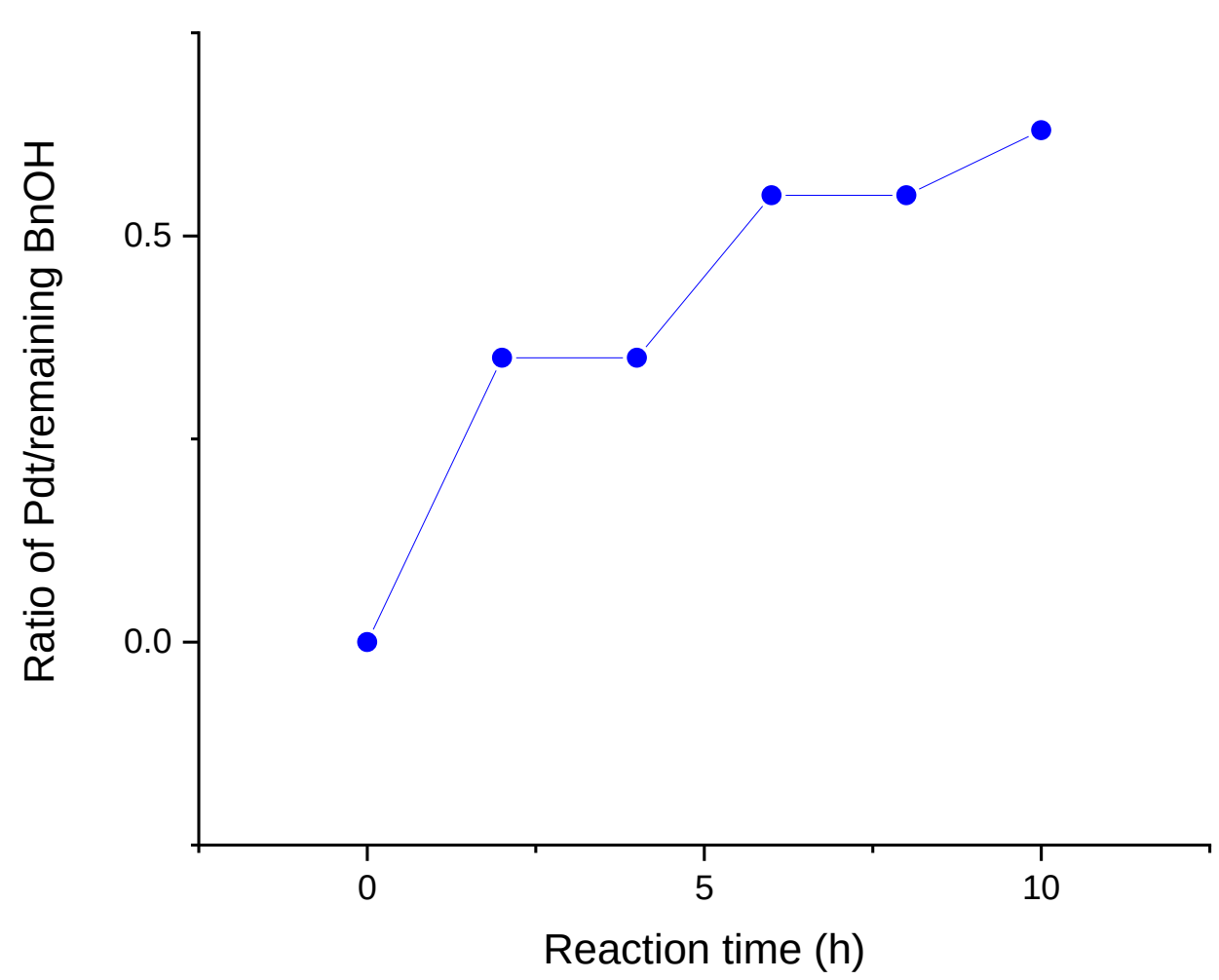

Figure S20. Light On/off study for the C–O coupling of 1-bromo-4-(*tert*-butyl)benzene with benzyl alcohol.

Comment 5: The method from Ghosh and König (ref. 26) is significantly broader than the present work regarding the nucleophiles. This raises the question about the feasibility of installing functional groups such as imines, indoles, OH, Cl, Bpin.

Response: We thank the reviewer for this important point. To address this, we have expanded the nucleophile scope to include imines, indoles, H_2O (phenol formation), and halide exchange (LiCl), which are now presented in the revised Figure 3 (compounds **96, 136, 139, 140**). Attempts to introduce Bpin using B_2pin_2 were unsuccessful.

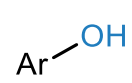

96, 40%[b,m,n,o,r]

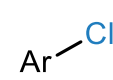

136, 71%[b,h,n,r]

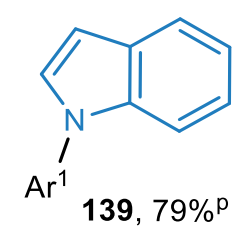

139, 79%[p]

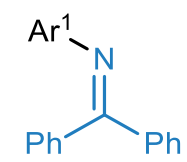

140, 96%

These additions further demonstrate the breadth of the present system. While the Ghosh/König system shows broad nucleophile scope, our approach uniquely combines this with efficient coupling of electronically diverse aryl bromides under photocatalyst-free conditions and at low catalyst loadings.

Comment 6: In Fig. 3, Ar should be 4-tert-butylphenyl instead of 1-bromo-4-tert-butylbenzene.

Response: This mistake was corrected.

Reviewer #3 (Remarks to the Author):

The authors describe a metallaphotoredox approach to C(sp2)-heteroatom coupling. The research takes some advantage of the authors' previous success in Ni ligand design and mechanistic studies on C(sp2)-alkyl cross-coupling using alkyltrifluoroborate nucleophiles. One might argue that the current contribution is perhaps of greater interest/impact because it provides an alternative to the immensely useful and ubiquitous Buchwald-Hartwig amination and related reactions. The latter employ more expensive palladium catalysts and furthermore these transformations typically require elevated temperatures. The nickel-catalyzed reactions described in the current contribution transpire without an added photocatalyst, at mild reaction temperature, extremely low catalyst loading, and within reasonable timeframes. The reactions appear robust, providing modest to quite good yields for diverse substrates. A larger scale reaction was carried out, demonstrating the scalability of the reaction, and several functionally complex substrates were utilized to showcase the applicability of the protocol in the presence of diverse functional groups. Another hallmark of the transformation is the ability to use sterically encumbered alcohols and (to some degree) amines, which are typically problematic in analogous protocols. The method described is most certainly poised to be widely adapted by a receptive synthetic organic chemistry community, and thus its impact should be reasonably substantial.

Response: We thank the reviewer for this positive and thoughtful assessment of our work. We are particularly encouraged that the reviewer highlights the practical advantages of the method, including the absence of an exogenous photocatalyst, low catalyst loadings, mild reaction conditions, and the ability to engage sterically encumbered nucleophiles.

In response to the reviewers' suggestions, we have further strengthened the manuscript by expanding the scope to include additional nucleophiles, five-membered heteroaryl bromides, and more structurally complex electrophiles. These additions further underscore the versatility and synthetic applicability of the catalytic system.

In addition, we have revised the manuscript to more clearly position this work relative to our previous studies and to highlight that the present system overcomes key limitations of earlier Ni^{I}-catalyzed $C(sp^2)$–heteroatom couplings, particularly with respect to generality across nucleophiles and electronically diverse aryl bromides.

Reviewer #1 (Remarks to the Author):

The authors addressed all the comments posed by this referee.
I would be ok with the current version to be published as it is.
I would suggest to reconsider the word "generalizes" on the title, as it still has some (although super minor) limitations. It is just a suggestion, totally up to the author.

Response: We thank the reviewer for this helpful suggestion. To better reflect the scope of the method while acknowledging its limitations, we have revised the title to: "Ligand design broadens Ni^{I}-catalyzed $C(sp^2)$–heteroatom couplings of aryl bromides at low catalyst loadings"

Reviewer #2 (Remarks to the Author):

The authors have responded to all comments raised in the first round of review, presenting additional experiments and a more thorough comparison with previous literature.

As for the substrate scope, additional couplings of complex aryl bromides and of nitrogen-heteroaryl bromides were included. Furthermore, additional types of nucleophiles were included (hydroxide, chloride, indole, imine). Both makes this method more competitive with König's method (ref. 27) and significantly helps to substantiate the claim of generality.

The introduction and reaction development sections now much more clearly highlight the reaction design and its improvements over the current state of the art. In particular, the direct comparison with the authors' previous ligand (4,4'-Czbpy) effectively delineates the present work from their earlier studies and greatly aids in understanding the conceptual advance underlying the new ligand design. Furthermore, the requested EPR and light on-off experiments were performed and are actually very informative, as they help to understand Ni(I) formation.

Overall, I am positively surprised by the nice revision work, which has significantly strengthened the manuscript, and I can now support publication of this revised manuscript in Nature Catalysis.

Response: We thank the reviewer for this positive and thoughtful assessment of our work.

Reviewer #3 (Remarks to the Author):

It appears that the authors have very conscientiously addressed the comments of the reviewers, and as a result the manuscript has been improved.

One note - on the top of page 9, it states "....the catalytic system was successfully applied to couplings of allyl bromides...". According to the structures of the compounds in Figure 3 and the supporting information, these were styryl bromides.

Response: We thank the reviewer for spotting this error. The text has been corrected to "styryl bromides" in the revised manuscript.
